# Impact of Pregnancy-Related Deaths on Female Life Expectancy in Zambia: Application of Life Table Techniques to Census Data

**DOI:** 10.1371/journal.pone.0141689

**Published:** 2015-10-29

**Authors:** Richard Banda, Ingvild Fossgard Sandøy, Knut Fylkesnes, Fanny Janssen

**Affiliations:** 1 Central Statistical Office, Lusaka, Zambia; 2 Centre for International Health, University of Bergen, Bergen, Norway; 3 Population Research Centre, University of Groningen, Groningen, The Netherlands; Institute of Psychiatry, UNITED KINGDOM

## Abstract

**Introduction:**

Since 2000, the world has been coalesced around efforts to reduce maternal mortality. However, few studies have estimated the significance of eliminating maternal deaths on female life expectancy. We estimated, based on census data, the potential gains in female life expectancy assuming complete elimination of pregnancy-related mortality in Zambia.

**Methods:**

We used data on all-cause and pregnancy-related deaths of females aged 15–49 reported in the Zambia 2010 census, and evaluated, adjusted and smoothed them using existing and verified techniques. We used associated single decrement life tables, assuming complete elimination of pregnancy-related deaths to estimate the potential gains in female life expectancy at birth, at age 15, and over the ages 15–49. We compared these gains with the gains from eliminating deaths from accidents, injury, violence and suicide.

**Results:**

Complete elimination of pregnancy-related deaths would extend life expectancy at birth among Zambian women by 1.35 years and life expectancy at age 15 by 1.65 years. In rural areas, this would be 1.69 years and 2.19 years, respectively, and in urban areas, 0.78 years and 0.85 years. An additional 0.72 years would be spent in the reproductive age group 15–49; 1.00 years in rural areas and 0.35 years in urban areas. Eliminating deaths from accidents, injury, suicide and violence among women aged 15–49 would cumulatively contribute 0.55 years to female life expectancy at birth.

**Conclusion:**

Eliminating pregnancy-related mortality would extend female life expectancy in Zambia substantially, with more gains among adolescents and females in rural areas. The application of life table techniques to census data proved very valuable, although rigorous evaluation and adjustment of reported deaths and age was necessary to attain plausible estimates. The collection of detailed high quality cause-specific mortality data in future censuses is indispensable.

## Introduction

Since 2000, global efforts to improve maternal and child health have been intensified, driven by the targets set in the millennium development goals (MDGs). Global estimates of maternal mortality indicate declining trends over the last decade or two in many parts of the world [1–3]. However, very little progress has been recorded in sub-Saharan Africa, the region where maternal mortality is highest [2, 4–9]. In 2010, sub-Saharan Africa accounted for 56% of the estimated deaths of women from pregnancy and childbirth related causes, and on average 500 maternal deaths occurred per 100,000 live births [2]. Ten percent of maternal deaths within the region were attributed to HIV, while globally, the region accounted for 91% of maternal deaths due to HIV/AIDS [2]. Other factors driving the high maternal mortality in the region include the high level of fertility and poor maternal health services [8]. Despite making significant progress in the last decade in improving availability and access to maternal health services, the sub-Saharan African region is unlikely to meet the targets under MDG 5 at the expiry of the MDGs in 2015 [10].

Whereas, global and national policy makers tend to focus on the reduction in the maternal mortality ratio (MMRatio), a key indicator of MDG 5, obtaining insight into the effect of reducing or eliminating maternal mortality on the overall health of female populations, measured using life expectancy, could offer an additional perspective to policy makers on the benefits of reducing maternal mortality. Till now, however, very few studies have quantified the effects of full reduction of maternal mortality on female life expectancy.

While the impact of maternal mortality on overall life expectancy can be assessed using life-table techniques [6, 11, 12], these very powerful techniques require detailed high quality data on deaths by cause. Such data are not readily available in many developing countries with high maternal mortality [13–16]. Civil registration systems are often incomplete, and surveys commonly do not have the appropriate sample size, especially when attempting to analyse the data at sub-national levels. As a result the use of census data for the estimation of maternal mortality has been advocated [7, 16–18]. Life table techniques have not yet been applied to census data in order to study the impact of maternal or pregnancy-related mortality on life expectancy, although this might be a powerful tool to do so when cause-of-death data and, in particular, data on pregnancy-related deaths is available.

The only previous study that assessed the impact of maternal mortality on female life expectancy in high-mortality countries, used survey data and focused on the gains in life expectancy during the reproductive period [11]. The study–covering 28 sub-Sahara African countries—showed striking disparities in the estimated potential gain in reproductive age life expectancy among the countries studied [11]. The disparities observed among countries are likely to manifest themselves also within countries, such as between rural and urban areas. Since pregnancy-related deaths tend to have a strong association with the age of the woman and her place of residence (rural or urban) [8, 19], it is imperative that studies assessing the effects of pregnancy-related mortality on female life expectancy take into account these key variables.

In this paper we study the impact of full elimination of pregnancy-related mortality on overall female life expectancy in Zambia for different age groups, by urban/rural residence, and further compare with elimination of four other causes of female deaths (accidents, injury, suicide and violence) among women aged 15–49. In doing so, we apply life table techniques to census data, a novel approach in this particular field.

For Zambia, knowing the effects of complete elimination of maternal mortality on life expectancy would be particularly relevant. Although trend data indicates that the MMRatio in Zambia has declined recently [3, 20], the country still has one of the highest levels of maternal mortality in the region [2].

## Methods

### Data sources

Zambia has a civil registration system established by law but the completeness of coverage is very low, even for urban areas [21]. In October 2010, Zambia conducted their 5^th^ national population census in which all households were asked about deaths in the household during the period 12 months prior to the census [22]. Information on age at death, sex and cause of death was collected for all deaths reported by households. Causes of death were established based on the following categorisation specified in the census questionnaire: ‘sickness/disease’, ‘accident’, ‘injury’, ‘spousal violence’, ‘other violence’, ‘suicide’, ‘witchcraft’ and ‘other’ (not specified). For all deaths of females aged 12–49, time-of-death relative to the pregnancy state was queried using the following approach: “Did the death occur while pregnant?” If the answer was “no” to this question, the respondent was asked: “Did the death occur during childbirth?”, and if the answer was “no” to this too, a third question was posed: “Did the death occur during the 6 weeks period following the end of pregnancy, irrespective of the way the pregnancy ended?” [22]. A positive response to any of the three questions was used to identify a pregnancy-related death. According to the World Health Organisation (WHO), a pregnancy-related death is a “death of a woman while pregnant or within 42 days of termination of pregnancy, irrespective of the cause of death” [23]. Pregnancy-related deaths are often used as proxy of maternal deaths, although the former obviously also includes deaths that are not causally related to pregnancy or childbirth [24].

### Data evaluation and adjustment

#### Controlling for age misreporting

We evaluated the reported age for all females enumerated in the census and the *age-at-death* for all recorded deaths using the age-sex accuracy index (ASAI) [12]. The ASAI is a summary of the *age and sex ratio scores* computed using data for 5-year age groups from 10–14 years through to 65–69 years. An index of <20 means age-sex data is *accurate;* an index of 20–40 means age-sex data is *inaccurate*, and an index of >40 means age-sex data is *highly inaccurate* [12]. To compute the ASAI, we used the spreadsheets *AGEMSTH* developed by the U.S. Census Bureau [25]. The ASAI results indicated inaccurate age reporting, and highly inaccurate age-at-death data for both rural and urban areas. Heaping was evident at ages ending in 0, 5, 8 and 2. We therefore adjusted for the inconsistencies in the reported age and age-at-death by smoothing the distributions using the *Arriaga technique* [25]. The smoothing method was chosen because it performs relatively mild smoothing compared to four other options tested (*Carrier-Farrag*, *K*.*King-Newton*, *United Nations* and *Strong*) and therefore minimises the effect on the age-specific mortality rates. Moreover the method maintained the distribution totals. It must be noted that all four methods employ mathematical graduation of recorded age-sex distributions in 5-year or 10-year age groups, by fitting these age distributions to different curves in order to correct for net reporting errors. In each case, the process modifies the original age group totals, while in the case of the *United Nations* method, the distribution totals are also modified.

#### Female deaths

To evaluate the completeness in the total number of female deaths by age reported by households in the census, we used a combination of the General Growth Balance method [26] with the Synthetic Extinct Generation (SEG) method [27, 28]. This combined method that adjusts for population coverage completeness in the estimation of deaths coverage completeness is considered a better alternative over the original Brass Growth Balance (BGB) method [29]. For a full appraisal of the methods please see WHO (2013) [29].

We used population age distributions from the Zambia 2000 and 2010 population censuses, and the age distribution of deaths from the 2010 census. To apply the methods, we used spreadsheets developed by the WHO for use in estimating maternal mortality from census data [29]. We applied these methods separately for rural and urban areas and obtained completeness estimates and adjustment factors for each respectively (see: S1 Text for a detailed discussion of the results of the evaluation and adjustments, and S1 and S2 Worksheets, for the application of the methods). The combined GGB-SEG estimates showed that reporting of deaths was 71% complete in rural areas and 139% complete in urban areas, giving adjustment factors (i.e. 1/completeness) of 1.41 and 0.72, respectively. We applied the adjustments to the age-specific deaths.

#### Pregnancy-related deaths

Since standard methods for evaluating the completeness of pregnancy-related deaths reported in a census are currently lacking, we used proxy methods suggested by earlier studies [18, 24] to determine plausibility of the reported levels and age patterns in rural and urban areas. The proxy methods used are based on the assessment of (i) the reported number of pregnancy-related deaths as a proportion of the total deaths of females in the reproductive age group (PMDF), (ii) the ratio of such deaths to total births by age among women in the reproductive age group during the given reference period (PRMRatios), and (iii) the proportion of pregnancy-related deaths by age [30]. Our evaluation showed high crude PRMRatios in both rural and urban areas. However, the age-specific PRMRatios formed the characteristic j-shape described by *Hill et al*. [30], indicating a plausible age pattern of high mortality risk in the two age extremes (<20 and >35) within the reproductive age group 15–49 for both rural and urban areas. Further, the proportions of pregnancy-related deaths by age showed correlation with the age pattern of fertility; rising rapidly with age, reaching a peak between age 25 and 29, for both rural and urban areas. Because of the plausibility of the pregnancy-related death age patterns, we only adjusted the level of completeness of pregnancy-related deaths assuming correlation with completeness of death reporting of total deaths for rural and urban areas.

See **Table 1**for the recorded and adjusted female populations, total female deaths and pregnancy-related deaths by age for both rural and urban areas.

**Table 1:** Total female population, total female deaths 12-months prior to the census, and total pregnancy-related deaths of females aged 12–49 12-months prior to the census, by age group and rural-urban residence, Zambia 2010.

	Rural						Urban					
Age	Female Population (reported)	Female Population (smoothed^*^)	Female deaths (smoothed^*^)	Female deaths (adjusted^+^)	Pregnancy-related deaths (reported)	Pregnancy-related deaths (adjusted^+^)	Female Population (reported)	Female Population (smoothed^*^)	Female deaths (smoothed^*^)	Female deaths (adjusted^+^)	Pregnancy-related deaths (reported)	Pregnancy-related deaths (adjusted^+^)
<1	144,369	142,995	8,200	11,524	-	-	74,045	68,922	3,583	2,570	-	-
1–4	585,858	580,283	7,237	10,172	-	-	308,343	287,011	2,725	1,955	-	-
5–9	604,006	610,954	8,434	11,854	-	-	327,935	354,390	3,503	2,512	-	-
10–14	515,999	503,171	2,360	3,317	62	87	342,209	348,825	1,128	809	24	17
15–19	405,259	418,087	927	1,303	404	568	329,766	323,150	906	650	156	112
20–24	328,902	339,832	2,431	3,417	478	672	283,698	291,998	2,180	1,564	237	170
25–29	288,345	277,415	2,553	3,588	547	769	253,406	245,106	2,416	1,733	318	228
30–34	216,425	219,798	2,603	3,658	474	666	186,651	184,220	2,541	1,822	279	200
35–39	178,320	174,947	2,260	3,176	307	431	136,532	138,963	2,189	1,570	159	114
40–44	127,920	132,609	1,596	2,243	160	225	87,411	91,831	1,442	1,034	67	48
45–49	108,965	104,276	1,270	1,785	75	105	71,191	66,771	1,112	798	36	26
50–54	86,527	79,867	987	1,387	-	-	55,031	51,855	890	638	-	-
55–59	59,286	65,946	919	1,291	-	-	35,505	38,681	777	558	-	-
60–64	61,277	59,636	968	1,361	-	-	27,179	26,637	742	532	-	-
65–69	46,546	48,187	1,009	1,418	-	-	18,341	18,883	695	498	-	-
70–74	35,835	35,762	1,061	1,492	-	-	13,038	12,881	653	469	-	-
75–79	22,287	22,360	1,126	1,582	-	-	8,471	8,628	617	442	-	-
80+	24,817	24,817	1,613	2,267	-	-	9,366	9,366	1,039	745	-	-
**Sub-total (15–49)**	1,654,136	1,666,964	13,640	19,171	2,445	3,436	1,348,655	1,342,039	12,786	9,171	1,252	898
**Total **	3,840,943	3,840,942	47,554	66,835	2,507	3,523	2,568,118	2,568,118	29,138	20,899	1,276	915

Note

^*^Arriaga technique

^+^Combined GGB-SEG.

#### Definition of rural versus urban

An extensive national cartographic mapping exercise was conducted prior to the census and the created census enumeration areas (EAs) were coded as either rural or urban. An urban area was defined as a locality of 5,000 inhabitants or more, having public services such as schools, hospitals, piped water, electricity, and 50% or more of households engaged in non-agricultural activities for their sustenance [31]. A rural area was defined as a locality in which agriculture was the main source of sustenance for the majority of households.

### The analysis

#### Application of life table techniques

Estimates of life expectancy, defined as the average number of additional years that a survivor to a particular age is expected to live beyond that age [32], are generated from life tables. A life table is an important demographic tool used in the analysis of mortality and other demographic processes [32]. Whereas the cohort life table depicts the mortality and survival experience of a real cohort, it can only be calculated once a cohort is extinct and therefore would only provide historical information. Instead the period life table is commonly used. A period life table depicts the impact of all-cause mortality at each age in a certain period on the survival and life expectancy of the population, assuming that the observed age-specific mortality rates in the period would prevail throughout the life course of the study population [32]. In doing so, the resulting life expectancy provides an estimate of the current health situation using the data that is available. In life table construction, the term decrement refers to the mode of exiting the life table [33], or–stated differently—of exiting the state “alive”. A standard life table having ‘death’ as the only mode of exiting is sometimes referred to as a single decrement life table. In multiple-decrement life tables more than one decrement operate [12, 32, 33], and such tables can be used to assess the contribution of, for example, different causes of death to the survival of the population [12]. However, it cannot be used to assess the effect on life expectancy. To estimate the significance of a single cause of death, “cause-deleted” life tables need to be constructed [34], also referred to as associated single decrement life tables (ASDT). The contribution of pregnancy-related mortality to female life expectancy can also be estimated using this approach, thereby examining the effect if all pregnancy-related deaths in the study population would no longer occur. In doing so it is assumed that mortality from all other causes of death would remain constant when pregnancy-related deaths were eliminated.

By comparing the life expectancy values from the ASDT with the life expectancy values of a standard (single decrement) life table, the potential gain in life expectancy (PGLE) can be assessed. For the construction of the standard life tables [12, 32] for Zambian women in 2010, we computed age-specific mortality rates by dividing the age-specific adjusted total female deaths by the age-specific adjusted average population size. To estimate the average number of person-years lived by those dying within the age interval (_n_ *a*_x_), we applied the graduation method with two iterations [32] to the adjusted deaths for age groups 5–9 through 75–79, and applied the method proposed by *Coale & Demeny* [32] for the age groups less than 1 and 1–4.

The starting point for the calculation of the ASDT was the calculation of the number of non-pregnancy related deaths among women aged 15–49 and the assumption of proportionality between the non-pregnancy-related death rate and the hypothetical death rate if all pregnancy-related deaths would be eliminated [32]. We used the same _n_ *a*_x_ values as in the standard life table. All the life tables were constructed in *Microsoft Excel* [35]. We did the calculations separately for Zambia as a whole, rural Zambia and urban Zambia.

#### Impact on reproductive age life expectancy (RALE)

A comprehensive understanding of female survival in the absence of pregnancy-related deaths can be achieved by analysing the PGLE at different ages together with changes in reproductive age life expectancy (RALE). RALE is defined as the average number of years lived between age 15 and 49 by female survivors to age 15, taking into account all-cause mortality in the reproductive age group [11]. Changes in RALE could provide a summary measure of female survival in the reproductive age group if pregnancy-related deaths were eliminated, while holding mortality from other causes constant. In the absence of death, a female would spend a total of (50–15 =) 35 years in the reproductive age group. We used standard life tables to estimate all-cause mortality RALE (i.e. before eliminating pregnancy-related deaths) using the formula _35_L_15_/*l*_15_, and estimated RALE after eliminating pregnancy-related deaths (RALE^*-i*^) from the ASDTs using the formula _35_^*^L^*-i*^_15_/^*^ *l* ^*-i*^_15_. We computed separate estimates for rural and urban areas.

#### Comparison with eliminating other causes of death

We further constructed separate ASDTs based on complete elimination of female deaths from accidents, injury, suicide and violence among women aged 15–49, in order to compare the gains this would give with PGLE from eliminating pregnancy-related deaths. Although deaths from ‘sickness/disease’, ‘witchcraft’ and ‘other’ (not specified) were also collected, we opted not to include them in the comparison due to a very high likelihood of overlap with pregnancy-related deaths.

For accidents, injury, suicide and violence, the values of each respective RALE^*-i*^ were computed using the same formula. We show the estimates for total Zambia, because we could not generate rural and urban estimates for each of these causes of death due to small numbers of deaths.

### Ethical approval

Our study used information accessible from a national descriptive tabulations report [36]. Use of such aggregate data does not require ethical approval [37].

## Results

Pregnancy-related deaths made up 15.3% of all deaths of women aged 15–49 in Zambia during the 12 months prior to the census in October 2010; 17.9% in rural areas and 9.8% in urban areas. The proportion of deaths that were pregnancy-related increased rapidly with age to a peak in the age group 25–29, and after that falling with increasing age (see: Figure C in S1 Text). This pattern was similar in both rural and urban areas and to a great extent reflects the age pattern of fertility. Half of all recorded pregnancy-related deaths were reported to have occurred in the antepartum period (during pregnancy). The other half was made up of 27% intrapartum deaths and 23% postpartum deaths. This distribution of pregnancy-related deaths was reflected in both rural and urban areas, but with a slightly higher proportion of postpartum deaths than intrapartum deaths in urban areas.

The abridged ASDTs are presented in **Table 2**for rural and urban areas, including as well some columns referring to all-cause mortality. See S2 Text for full versions of the ASDTs for Zambia Total, Rural and Urban. The PGLE at birth and at exact ages of 15, 25 and 35 are summarised in **Table 3**.

**Table 2:** Abridged associated single decrement life tables (ASDTs), assuming full elimination of pregnancy-related mortality (i) females only, Zambia 2010, by rural-urban residence

**Rural**												
Age _x_	_n_ *D*_x_	_n_ *D* ^i^_x_	_*n*_ *R* ^*-i*^_*x*_	_n_ *p*_x_	_n_ *a*_x_ = _n_ *a ^-i^_x_	*e*_x_	_n_*p ^-i^_x_	_n_ * *q* ^-i^_x_	* *l* ^-i^_x_	*T ^-i^_x_	* *e* ^-i^_x_	*PGLE* ^-i^ _x_
0	11,524	0	1	0.9238	0.28	46.58	0.9238	0.0762	100,000	4,827,306	48.27	1.69
1	10,172	0	1	0.9324	1.85	49.40	0.9324	0.0676	92,384	4,732,800	51.23	1.83
5	11,854	0	1	0.9083	1.99	48.85	0.9083	0.0917	86,142	4,376,688	50.81	1.96
10	3,317	87	0.9737	0.9676	2.46	48.58	0.9684	0.0316	78,246	3,969,728	50.73	2.16
15	1,303	568	0.5643	0.9845	2.66	45.12	0.9912	0.0088	75,775	3,584,772	47.31	2.19
20	3,417	672	0.8034	0.9509	2.66	40.79	0.9603	0.0397	75,111	3,207,448	42.70	1.91
25	3,588	769	0.7857	0.9373	2.60	37.76	0.9504	0.0496	72,132	2,838,867	39.36	1.60
30	3,658	666	0.8179	0.9201	2.52	35.11	0.9341	0.0659	68,554	2,486,808	36.28	1.16
35	3,176	431	0.8642	0.9132	2.47	32.94	0.9245	0.0755	64,039	2,155,243	33.66	0.71
40	2,243	225	0.8998	0.9189	2.46	30.84	0.9267	0.0733	59,207	1,847,274	31.20	0.36
45	1,785	105	0.9409	0.9179	2.50	28.34	0.9226	0.0774	54,868	1,562,243	28.47	0.13
50	1,387	0	1	0.9168	2.47	25.65	0.9168	0.0832	50,621	1,298,532	25.65	-
55	1,291	0	1	0.9066	2.52	22.76	0.9066	0.0934	46,409	1,056,064	22.76	-
60	1,361	0	1	0.8920	2.56	19.84	0.8920	0.1080	42,075	834,777	19.84	-
65	1,418	0	1	0.8625	2.61	16.93	0.8625	0.1375	37,529	635,514	16.93	-
70	1,492	0	1	0.8106	2.57	14.22	0.8106	0.1894	32,370	460,191	14.22	-
75	1,582	0	1	0.6895	3.03	11.94	0.6895	0.3105	26,239	313,214	11.94	-
80+	2,267	0	1	0.0000	10.95	10.95	0.0000	0.0762	18,091	198,049	10.95	-
**Urban**												
Age _x_	_n_ *D*_x_	_n_ *D* ^i^_x_	_*n*_ *R* ^*-i*^_*x*_	_n_ *p*_x_	_n_ *a*_x_	*e*_x_	_n_*p ^-i^_x_	_n_ * *q* ^-i^_x_	* *l* ^-i^_x_	*T ^-i^_x_	* *e* ^-i^_x_	*PGLE* ^-i^ _x_
0	2,570	0	1	0.9638	0.16	61.02	0.9638	0.0362	100,000	6,179,892	61.80	0.78
1	1,955	0	1	0.9732	1.80	62.31	0.9732	0.0268	96,385	6,082,938	63.11	0.81
5	2,512	0	1	0.9653	1.87	59.97	0.9653	0.0347	93,798	5,703,083	60.80	0.83
10	809	17	0.9787	0.9885	2.58	57.06	0.9887	0.0113	90,545	5,244,265	57.92	0.86
15	650	112	0.8279	0.9900	2.73	52.70	0.9917	0.0083	89,524	4,794,015	53.55	0.85
20	1,564	170	0.8913	0.9736	2.69	48.20	0.9764	0.0236	88,781	4,348,082	48.98	0.77
25	1,733	228	0.8684	0.9652	2.62	44.44	0.9697	0.0303	86,685	3,909,017	45.09	0.66
30	1,822	200	0.8902	0.9517	2.54	40.94	0.9569	0.0431	84,062	3,481,832	41.42	0.47
35	1,570	114	0.9274	0.9451	2.48	37.89	0.9490	0.0510	80,439	3,070,451	38.17	0.28
40	1,034	48	0.9535	0.9452	2.49	34.95	0.9477	0.0523	76,333	2,678,611	35.09	0.14
45	798	26	0.9676	0.9420	2.50	31.83	0.9438	0.0562	72,343	2,306,953	31.89	0.06
50	638	0	1	0.9403	2.46	28.64	0.9403	0.0597	68,278	1,955,382	28.64	-
55	558	0	1	0.9304	2.55	25.30	0.9304	0.0696	64,204	1,624,321	25.30	-
60	532	0	1	0.9047	2.57	22.00	0.9047	0.0953	59,735	1,314,263	22.00	-
65	498	0	1	0.8760	2.55	19.05	0.8760	0.1240	54,043	1,029,402	19.05	-
70	469	0	1	0.8333	2.50	16.38	0.8333	0.1667	47,343	775,588	16.38	-
75	442	0	1	0.7706	2.71	14.16	0.7706	0.2294	39,450	558,608	14.16	-
80+	745	0	1	0.0000	12.57	12.57	0.0000	1.0000	30,399	382,064	12.57	-

Notes

* denotes life table functions of an associated single decrement life table; *-i* denotes the elimination of pregnancy-related mortality.

_*n*_ *R* ^-i^_*x*_ refers to proportion of deaths in the age interval x to x+n due to other causes of death when pregnancy-related mortality is eliminated **(**_**n**_ ***R*** ^-i^_***x***_ ***= (***_***n***_ ***D***_***x***_ ***-***_***n***_ ***D*** ^i^_***x***_ ***)/***_***n***_ ***D***_***x***_ ***)***.

_n_*q ^-i^_*x*_ refers to the probability of dying between exact ages x and x+n assuming that pregnancy-related deaths had been eliminated (_n_*q ^-i^_***x***_ **= 1-**_n_*p ^-i^_**x**_).

*l ^-i^ refers to the life table cohort survivors to exact age x assuming that pregnancy-related deaths had been eliminated (*l ^-i^_***x+n***_ **=** *l ^-i^_**x**_ **.**_n_*p ^-i^_***x***_).

*e ^-i^_*x*_ refers to the average number of years of life remaining for survivors to exact age x assuming that pregnancy-related deaths had been eliminated (*e ^-i^_**x**_ **= ∑** ^*^ **T** ^-i^_**x+n**_ **/** *l ^-i^_***x***_).

*PGLE* ^-i^_*X*_ refers to the potential gain in life expectancy at exact age x by eliminating pregnancy-related deaths (***PGLE*** ^-i^_***x***_ **=** *e ^-i^_x_*- *e* ^i^_***x***_).

**Table 3:** Life expectancy and probability of dying before age 50, at different ages, for all-cause mortality and when pregnancy-related deaths are eliminated, and the potential gain in life expectancy (PGLE), females only, Zambia 2010, by rural-urban residence and combined.

	Zambia	Rural	Urban
Life expectancy at birth (all deaths)	51.43	46.58	61.02
Life expectancy at birth (pregnancy-related deaths eliminated)	52.78	48.27	61.80
Probability of a new born female dying before age 50 (all deaths)	0.4593	0.5292	0.3332
Probability of a new born female dying before age 50 (pregnancy-related deaths eliminated)	0.4311	0.4938	0.3172
**PGLE at birth (years)**	**1.35**	**1.69**	**0.78**
Life expectancy at age 15 (all deaths)	47.99	45.12	52.70
Life expectancy at age 15 (pregnancy-related deaths eliminated)	49.63	47.31	53.55
Probability of a 15 year old female dying between age 15 and 50 (all deaths)	0.3268	0.3781	0.2550
Probability of a 15 year old female dying between age 15 and 50 (pregnancy-related deaths eliminated)	0.2922	0.3320	0.2373
**PGLE at exact age 15 (years)**	**1.65**	**2.19**	**0.85**
Life expectancy at age 25 (all deaths)	40.23	37.76	44.44
Life expectancy at age 25 (pregnancy-related deaths eliminated)	41.45	39.36	45.09
Probability of a 25 year female old dying between age 25 and 50 (all deaths)	0.2904	0.3357	0.2270
Probability of a 25 year old female dying between age 25 and 50 (pregnancy-related deaths eliminated)	0.2622	0.2982	0.2123
**PGLE at exact age 25 (years)**	**1.22**	**1.60**	**0.66**
Life expectancy at age 35 (all deaths)	34.64	32.94	37.89
Life expectancy at age 35 (pregnancy-related deaths eliminated)	35.18	33.66	38.17
Probability of a 35 year old female dying between age 35 and 50 (all deaths)	0.2008	0.2297	0.1585
Probability of a 35 year old female dying between age 35 and 50 (pregnancy-related deaths eliminated)	0.1858	0.2095	0.1512
**PGLE at exact age 35 (years)**	**0.55**	**0.71**	**0.28**

For all-cause mortality, female life expectancy at birth (*e*_0_) was 51.43 years; 46.58 years in rural areas and 61.02 years in urban areas, respectively (**Table 2**). Eliminating pregnancy-related mortality would result in a PGLE at birth of 1.35 years overall, 1.69 years in rural areas and 0.78 years in urban areas (**Tables 2 & 3**). The PGLE would be highest at exact age 15, 1.65 years overall. The PGLE at birth if only 25% of pregnancy-related deaths were eliminated would be 0.34 years compared to 1.01 years if 75% of deaths were eliminated overall, while PGLE at age 15 would be 0.41 years and 1.23 respectively (data not shown).

If pregnancy-related mortality was eliminated, a new-born’s risk of dying between age 15 and 50 would be reduced by 6.1 percent overall; 6.7 percent in rural areas compared to 4.8 percent in urban areas. In the case of a female aged 15, the risk of dying between age 15 and 50 would be reduced by 10.6 percent overall; 12.2 percent in rural areas compared to 6.9 percent in urban areas (**Table 3**).

**Fig 1:**
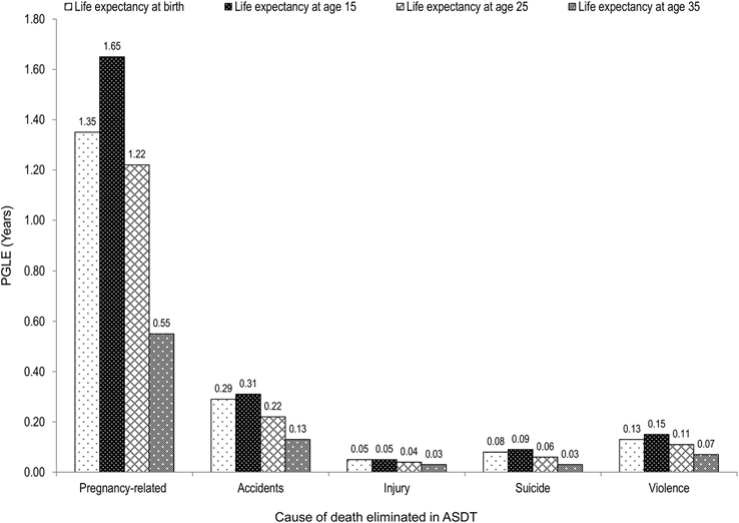
Potential gain in life expectancy (PGLE) at birth and at exact ages 15, 25 and 35 after eliminating specific causes of death among females aged 15–49, Zambia 2010.

As can be viewed from **Fig 1**, eliminating pregnancy-related mortality would result in a higher gain in female life expectancy compared to the elimination of deaths from accidents, injuries, violence and suicide among reproductive age women. Of all deaths of women aged 15–49, accidents accounted for 2.8% of the deaths, violence (1.3%), suicide (0.8%) and injury (0.4%). Eliminating deaths from accidents, injury, suicide and violence would cumulatively contribute half a year to life expectancy at birth (0.55 years), as well as to life expectancy at age 15 (0.60 years).

At prevailing mortality levels, women in Zambia spent an average of 30.1 years, out of a possible total of 35 years (50–15), in the reproductive age group 15–49 (**Table 4)**. Eliminating pregnancy-related mortality would extend this by 0.72 years overall; 1.00 years in rural areas and 0.35 years in urban areas.

**Table 4:** Reproductive age life expectancy (RALE) from all deaths and after complete elimination of specific causes of death (^*^RALE^*-i*^), females aged 15–49, Zambia, 2010

	Pregnancy-related			Suicide	Violence	Injury	Accidents
	Total	Rural	Urban				
RALE	30.13	29.17	31.36	30.13	30.13	30.13	30.13
^*^RALE^*-i*^	30.85	30.17	31.71	30.17	30.19	30.15	30.26
Gain in RALE (RALE-^*^RALE^*-i*^)	0.72	1.00	0.35	0.04	0.06	0.02	0.13

Note: RALE *=*_35_L_15_ */l*_15_

^*^RALE^*-i*^ *=*_35_^*^L_15_^*-i*^ */* ^*^ *l*_15_^*-i*^.

## Discussion

### Summary of results

Whereas the overall life expectancy at birth for Zambian women was 51.43 years in 2010, a complete elimination of pregnancy-related deaths would extend the life expectancy at birth by 1.35 years and life expectancy at age 15 by 1.65 years. Striking geographical and age disparities in this impact were revealed. In rural areas the gain in life expectancy was substantially higher than in urban areas. Moreover the impact of eliminating pregnancy-related deaths was strongest at age 15. In comparison, eliminating deaths from accidents, injury, suicide and violence among women aged 15–49 would contribute 0.55 years to female life expectancy at birth.

### Explanations of the observed results

#### Substantial overall impact

The impact of pregnancy-related mortality on female life expectancy in Zambia is substantial: (i) it is much higher than the marginal gains in life expectancy if deaths from accidents, injuries, suicide and violence among reproductive age women were eliminated; (ii) it is high despite pregnancy-related deaths mostly affecting those aged 15–49; (iii) it is high considering the fairly low overall life expectancy value for women in Zambia of 51.43 years; and (iv) it is high considering that the adjusted number of pregnancy-related deaths (4,439) only represents 5.1% of the adjusted total female deaths for the 12-months prior to the census. A study in China using health facility based data and covering the period 1950–2010 estimated PGLE at birth from eliminating maternal diseases (complications of pregnancy, childbirth and puerperium) of between 0.56 to 0.002 years over the same period [38]. The highest PGLE of 0.56 years was estimated for the period 1950, when the MMRatio for China was about 1,500 deaths per 100,000 live births [38].

#### Differentials in the impact by age

Maternal and pregnancy-related mortality varies significantly by age within the at-risk population of women aged 15–49, and therefore the effects from eliminating such deaths need to be disaggregated by age. Despite the lower rate of pregnancy-related death (per woman) among those aged 15–19 compared to those aged 25–29 or 30–34, we observed that eliminating pregnancy-related mortality would have greater impact at age 15 than at any other age. This is partly due to the high proportion of adolescent deaths that were pregnancy-related, 34.7% overall; 43.6% in rural areas and 17.2% in urban areas, which is also observed in other countries in Africa [39]. Another likely contributing factor is that other causes of death, such as HIV/AIDS, Tuberculosis and some forms of malignant neoplasms occur much less at these young ages compared to women at older ages [40].

In addition, because life expectancy at a particular age is calculated based on the total number of person-years lived above that age; the full benefit of eliminating pregnancy-related deaths accrues more at younger ages.

#### Differentials in the impact by rural-urban residence

The observed difference in PGLE from eliminating pregnancy-related deaths between rural areas and urban areas is likely due to the differences in the level of risks. The census estimate of pregnancy-related mortality ratio (PRMRatio) for the period was 789 deaths/100,000 live births overall; 960/100,000 live births in rural areas and 470/100,000 live births in urban areas (see: Table C in S1 Text). Although both rural and urban areas exhibited very high pregnancy-related mortality (>350/100,000 live births), mortality in rural areas was twice as high compared to urban areas. Thus eliminating pregnancy-related deaths would result in higher PGLE in rural compared to urban areas.

An indirect contributor to the high proportion of pregnancy-related deaths in rural areas is the level of fertility. On average, women in rural areas have 6.6 children during the course of their reproductive life compared to an average of 3.7 children for women in urban areas [20]. The high fertility in Zambia and in particular in rural areas is attributed to factors such as early childbearing, low socio-economic status of women, lack of a good social security system for old people (which makes parents depend on having many children to secure themselves) and low contraceptive use [41, 42]. One in every three women aged 15–19 in rural areas is either already a mother or pregnant compared to one in every five in urban areas [42]. Further, women in rural areas are far less likely to seek professional assistance for childbirth due to distance, fewer skilled health care workers, and fewer and poorly equipped health facilities compared to women in urban areas [43–47]. Giving birth without skilled attendance is associated with greater risk of mortality [48].

HIV/AIDS is also likely to have affected the size of the urban-rural differential in PGLE. Studies in South Africa and elsewhere [2, 49, 50] show that a significant proportion of maternal and pregnancy-related deaths in high HIV prevalence settings are due to the indirect effect of HIV/AIDS. Since the 1990s, HIV prevalence among adult females in urban areas in Zambia has been double that in rural areas [42, 51], and the mortality has been found to be 1.7 times higher in urban than in rural areas [52]. The United Nations (UN) estimates the proportion of maternal deaths due to HIV/AIDS in Zambia during the period 2010–2013 at 15–30% [2, 3]. HIV/AIDS is therefore likely to contribute more to the high pregnancy-related mortality in urban vs. rural areas. Therefore, in the absence of HIV/AIDS, it is likely that the PGLE would be much higher in rural areas as compared to urban areas, since other determinants of pregnancy-related deaths are more prominent in rural areas compared to urban areas.

#### PGLE from eliminating other causes of death

By comparing PGLE from eliminating pregnancy-related deaths against PGLE from eliminating deaths from accidents, injury, suicide and violence among women aged 15–49, our study gave an impression of the relative importance of pregnancy-related mortality on female life expectancy in Zambia. However, caution must be exercised in making this comparison due to overlap between the deaths. Since pregnancy-related deaths were identified using time-of-death relative to pregnancy, overlap between reported non-maternal deaths (deaths from accidents, injuries, suicide or violence) and reported pregnancy-related deaths was found. For example, among accident deaths of women aged 12–49, 11% were also pregnancy-related i.e. the accident deaths occurred when the woman was pregnant, in labour or in the six weeks postpartum period. Similarly, 18% of deaths from injury, 24% of deaths from violence and 28% of deaths from suicide in the same age group of women were also identified as pregnancy-related i.e. the deaths occurred when the woman was pregnant, in labour or in the six weeks postpartum period. Although such overlap could result in higher PGLE from eliminating pregnancy-related deaths relative to PGLE from eliminating deaths from other causes of death, the estimates of PGLE from eliminating pregnancy-related deaths remain valid due to the definition used to identify such deaths, which is based on time-of-death rather than cause. Incorporating a verbal autopsy tool or a more detailed coding scheme for causes of death in the census would be necessary to improve the identification of actual maternal deaths and minimise the observed overlap. The use of verbal autopsy in a census, however, requires adequate planning and piloting due to the size and scope of a census.

#### Levels and differentials in RALE

The gains in RALE after eliminating pregnancy-related deaths were–just as the PGLE—also substantial. Despite the increase in RALE from eliminating pregnancy-related deaths, women in both rural and urban areas lived, on average, about four years less than the maximum possible 35 years due to high all-cause mortality in the reproductive age group.

Our estimated gain in RALE for the 12-months period prior to the census of 0.72 years overall was marginally higher than the national estimate of 0.5 years by *Canudas-Romo et al*. (2014) estimated using DHS data for the year 2007 [22]. A difference in the reference period (the DHS data referred to the period 2001–2007) and the level of all-cause mortality (the earlier study used model life tables from the Human Mortality Database) could partly explain the differences. In contrast to *Canudas-Romo et al*. (2014), we were able to provide separate estimates for rural and urban areas. Due to sample size limitations, such disaggregated analyses are rare with survey data, and as such use of census data for such estimation becomes advantageous.

#### PGLE and healthy life expectancy gains

It should be noted that mortality reduction efforts do not only work to prevent death but usually work indirectly by improving health and wellbeing. For example, in Zambia, illegal abortions, a major contributor to maternal mortality, lead to post-abortion ill health from excessive haemorrhage, infections, perforated uterus and other complications [53]. Elimination of pregnancy-related mortality would not only lead to life expectancy gains but also healthy life expectancy gains; the average number of years that a person at a given age would live in functional and good health taking account of prevailing mortality and morbidity conditions [54]. Poor health outcomes can be averted if comprehensive reproductive health services, including access to effective contraceptives, and quality antenatal and postnatal care, are provided as part of maternal mortality reduction strategies.

#### Feasibility of eliminating pregnancy-related or maternal mortality

The high levels of maternal mortality in low income countries have caught the attention of global health policy makers because the deaths are readily preventable [8,50]. While complete elimination of both maternal and pregnancy-related mortality is not feasible, their reduction to very minimal levels is possible even for low income countries, as shown by Sri Lanka [3]. When one considers the WHO definitions of a maternal and a pregnancy-related death, it is easy for one to conclude that it is more feasible to reduce the former (maternal deaths). This is so because targeted interventions for significantly reducing maternal deaths have been well established over many decades, as shown in high income countries. Pregnancy-related deaths, by definition would be more challenging to prevent because the category includes all deaths occurring in the pregnancy-related period. Thus our estimate of PGLE from completely eliminating pregnancy-related deaths is likely to be very optimistic.

### Evaluation of data & methods

Our study demonstrates the relevance of life table techniques, particularly ASDTs in the study of maternal mortality in a LMIC setting with challenging public health issues and limited cause-of-death data. However, the life table techniques have limitations which also accrue to our study. While “cause deleted” life tables provide valuable insight into the contribution of a particular cause of death to the overall life expectancy, they remain a theoretical construct that might not provide real gains accrued from eliminating a particular cause-of-death. An individual is exposed to the risk of dying from multiple causes at any given time. Therefore causes of death do not operate independently, but as competing risks with great amounts of interaction [12]. For example, a woman could be at risk of dying from maternal causes, and at the same time she could be at risk of accidental causes such as traffic accidents. When estimating the gain from eliminating a single cause of death, competing risks need to be considered as they could affect the size of PGLE from eliminating that single cause of death. This is more so when the key ASDT assumption, that other causes of death remain constant, is not sustained. However, pregnancy-related deaths include all deaths in the ante-, peri- and postpartum periods, independent of their cause (i.e. there are no competing risks in these periods).

Evaluation of population age, age-at-death and reported deaths confirmed some concerns with regards to the accuracy of reporting in the Zambia censuses. Final estimates could only be obtained after adjustments of recorded data. Some significant adjustments were made to both total deaths and pregnancy-related deaths, and it is possible for such adjustments to potentially introduce bias in the estimates. Further, in the absence of formal methods to evaluate and adjust pregnancy-related deaths, we adjusted the reported pregnancy-related deaths for reporting completeness using the result of the evaluation of total deaths. By making this adjustment the estimated pregnancy-related mortality differential between rural and urban areas proved to be more plausible. Prior to the adjustment, the crude PRMRatios were 831 deaths/100,000 live births in rural areas and 846 deaths/100,000 live births in urban areas (see: Table C in S1 Text). However, the decision to adjust pregnancy-related deaths in this manner could in itself bias our results of PGLE if the completeness of reported pregnancy-related deaths differed markedly from that for total deaths. Several studies have stressed the urgent need for validated methods for evaluating and adjusting pregnancy-related death data from censuses [18, 24, 55].We support the call for such methods to support robust estimation of pregnancy-related mortality from census data.

## Conclusion and Recommendations

Eliminating pregnancy-related mortality would extend female life expectancy in Zambia in 2010 substantially, with more gains among adolescents and females in rural areas. The application of life table techniques to census data proved very valuable, although rigorous evaluation and adjustment of reported deaths and age was necessary to attain plausible estimates. Because of the importance of estimating the impact of pregnancy-related and maternal mortality on the overall health of women, by means of life expectancy or other measures, it is essential in future censuses to ensure the collection of high quality cause-specific mortality data.

## Supporting Information

S1 Table National fertility tables. (XLSX)

S2 Table National general and maternal mortality tables. (XLSX)

S3 Table National general population tables. (XLSX)

S1 Text Peer reviewed manuscript awaiting final journal decision. (PDF)

S2 Text Associated single decrement life tables, Zambia Total. (PDF)

S3 Text Estimates of _n_a_x_ values using graduation method. (PDF)

S4 Text Zambia 2010 Census of Population and Housing, National Descriptive Tables Volume 11. (PDF)

S1 Worksheet Application of GGB-SEG to deaths data for Zambia Rural. (XLSB)

S2 Worksheet Application of GGB-SEG to deaths data for Zambia Urban. (XLSB)
