# Supplementary material for: Impact of Pregnancy-Related Deaths on Female Life Expectancy in Zambia: Application of Life Table Techniques to Census Data
Source: PLoS One. 2015 Oct 29;10(10):e0141689. doi: 10.1371/journal.pone.0141689 (PMC4626102; doi:10.1371/journal.pone.0141689)
Supplement: S1 Text — (PDF) [file pone.0141689.s004.pdf]

Rural-urban differentials in pregnancy-related mortality in Zambia: estimates using data collected in a census

Richard Banda[1,2*], Knut Fylkesnes[2], Ingvild Fossgard Sandøy[2]

[1]Central Statistical Office, Lusaka, Zambia
[2]Centre for International Health, University of Bergen, Bergen, Norway

*Corresponding Author, email: richard.banda@student.uib.no; richardbanda@gmail.com

Co-authors: Knut.Fylkesnes@uib.no; Ingvild.Sandoy@uib.no

Abstract

Background: The use of census data to measure maternal mortality is a recent phenomenon, implemented in settings with non-functional vital registration systems and driven by needs for trend data. The 2010 round of population and housing censuses recorded a significant increase in the number of countries collecting maternal mortality data. The objective of this study was to estimate rural-urban differentials in pregnancy-related mortality in Zambia using census data.

Methods: We used data from the Zambia 2000 and 2010 censuses. Both censuses recorded, by age, the female population and live births and children ever born. The 2010 census further recorded, by age, household and pregnancy-related deaths 12-months prior to the census. We evaluated and adjusted recorded live births using the cohort PF ratio method, and household deaths using deaths distribution methods (General Growth Balance and Synthetic Extinct Generation). Adult female mortality and pregnancy-related mortality for rural and urban areas were estimated for the period October 2009-October 2010.

Results: Data evaluation showed errors in recorded population age, age-at-death, live births and deaths, and appropriate adjustments were made. Adjusted adult female mortality was high; an adolescent aged 15 had one-in-three chance of dying before her fiftieth birthday in rural areas and one-in-four in urban areas. Pregnancy-related deaths were 15.3% of all deaths of reproductive age women overall; 17.9% in rural areas and 9.8% in urban areas. The

pregnancy-related mortality ratio for the period was 789 deaths/100,000 live births overall: 960/100,000 live births in rural areas and 470/100,000 live births in urban areas.

Conclusions: Census-based estimates show very high adult female mortality and particularly high pregnancy-related mortality in both rural and urban areas of Zambia 12-months prior to the 2010 census. Future censuses should pay greater attention to strategies for improving data quality.

Key words: maternal mortality, pregnancy-related mortality, census, rural-urban, Zambia

Background

The millennium development goals (MDGs) related to health have pushed systems of data collection in many low and middle income countries (LMICs) into overdrive as global and national policy makers demand regular updates on progress towards achieving them. The fifth MDG focuses on maternal health, and one of the targets is to reduce maternal mortality ratio (MMRatio) by three quarters between 1990 and 2015 [1]. Continuous civil registration systems provide an ideal source of information on deaths and cause of death, including maternal mortality [2-4]. However, less than half of countries globally have complete civil registration systems with adequate cause of death information [4]. As a result, many countries rely on population based surveys to monitor MDG indicators. Unless verbal autopsy is included to enable ascertainment of actual maternal deaths, these surveys only provide measures of pregnancy-related mortality. The use of a census to measure pregnancy-related mortality is a recent phenomenon, realising the need to establish long term trend data. An increase in the number of countries including questions on pregnancy-related mortality has been noted following the United Nations (UN) recommendation for this to be a core topic in the 2010 round of censuses [5].

Due to the lack of a functional civil registration system, Zambia has relied on survey data generated using the sisterhood method in the Zambia Demographic and Health Surveys (ZDHS). Recent survey estimates of the pregnancy-related mortality ratio (PRMRatio) indicate declining trends; 729 (95% CI: 586-872) in the 2001/2 survey, 591 (95% CI: 450-732) in the 2007 survey and 389 (95% CI: 323-474) in the 2013/14 survey [6]. These estimates refer to a period 0-7 years prior to each survey and are only available at national level due to sample size limitations.

The advantage of the census approach lies in the ability to decompose estimates to subnational levels such as rural and urban areas [4, 7]. Subnational estimates are important in understanding levels of maternal mortality and its drivers within individual countries. The national requirement for subnational estimates was one of the key factors leading to the inclusion of pregnancy-related mortality in the Zambia 2010 census. The scope and primary focus of a census have given rise to concerns on whether it can succeed as a source of quality pregnancy-related mortality data [8]. The strengths and weaknesses of census based approaches for estimating pregnancy-related mortality have been highlighted in several studies [7-14]. When household deaths also are included in the census, the level of mortality during the reproductive age period can be used as a plausibility check on the estimated level of pregnancy-related mortality. The main objective of this study was to estimate pregnancy-related mortality differentials between rural and urban areas in Zambia using census data and to capture this against prevailing adult female mortality differentials.

Methods

Data sources

We used data collected in the Zambia 2000 and 2010 censuses of population and housing. The two censuses were conducted exactly ten years apart, both having 16th October as census

reference night. Each census was preceded by an extensive cartographic mapping exercise aimed at subdividing the country into standard enumeration areas (SEAs) having between 80 and 150 households. An enumerator was assigned to each SEA for the purpose of census enumeration. For the 2010 census, about 25, 000 enumerators took part in the data collection exercise [15]. The census estimated a total population (*de jure*) of 13,092,666, of which 39.5% resided in urban areas [15]. Females made up 50.7% of the total population, and 47.2% of the female population was in the reproductive age group 15-49 [15].

Data evaluation

Age

Age data in the two censuses was collected using the question on *age-at-last-birthday*. Errors in age data are common in censuses, but tend to be more problematic in populations where literacy levels are low [16]. Dynamic "hot deck" imputation was used to generate values for missing ages during data processing of both the 2000 and 2010 censuses. We still evaluated the quality of age data using the Age-Sex Accuracy Index [16]. The ASAI is a summary of the *age and sex ratio scores* computed using data for 5-year age groups from 10-14 through 65-69. The index only provides a measure of the quality of age data as follows: An index of <20 means age-sex data is *accurate;* an index of 20-40 means age-sex data is *inaccurate,* and an index of >40 means age-sex data is *highly inaccurate* [16]. To compute the ASAI, we used the spreadsheets *AGEMSTH* developed by the US Census Bureau [17].

Live Births and children ever born

Data on children ever born (CEB) was collected from all women aged 12 years and older, while data on live births in the 12-months prior to the census was collected from women aged 12-49 [18, 19]. Such data is often affected by reporting errors due to omission of children that die in infancy or that live elsewhere at the time of census [16, 20]. Detailed questions were

used, including asking about children that have since died or live elsewhere, in order to reduce such errors [18, 19]. We applied the cohort PF Ratio method to assess the quality of data and generate adjustment factors for live births [20].

Household deaths

Zambia collected household deaths in the census for the third time during the 2010 census, after two unsuccessful attempts in the 1969 and 1990 censuses [21, 22]. Households reported on deaths of household members 12 months prior to the census. Information on age-at-death, sex of the deceased and cause of death was collected for all reported deaths. Misreporting (omissions and duplications) are common for such data, including erroneous recording of age-at-death and sex. Similar to population age, missing values for age at death were generated using dynamic "hot deck" imputations during data processing. We evaluated the completeness of deaths reported in the 2010 census using three methods; the General Growth Balance (GGB) [23, 24], the Synthetic Extinct Generation (SEG) and the combined GGB-SEG [23]. Both the SEG and GGB only require a population closed to migration (or with negligible migration) as well as accurate recording of age for both population and deaths [23]. The SEG further requires that population coverage is constant across age and in each of the two censuses [23]. We first used the GGB to estimate completeness of death reporting and census coverage. We then applied the SEG to estimate completeness of deaths reporting and checked the estimates with those obtained from the GGB. We finally applied the combined GGB-SEG in order to adjust for population coverage between the two censuses. Final estimates of mortality were adjusted based on the combined GGB-SEG using age-trims 5+ to 65+. Given the high level of mortality in early adulthood in Zambia, we opted against fitting the age-trims 30+ to 65+ as that would exclude a huge number of female deaths before age 30. Hill *et al.* [25] recommended the use of the combined GGB-SEG approach fitted to the age-trims 5+ to 65+ for optimal results of the conditional probability of dying between exact age 15 and 60

($_{45}q_{15}$). To apply the data evaluation methods, we used the spreadsheets developed by the World Health Organisation (WHO) for the estimation of pregnancy-related mortality in a census [23].

Pregnancy-related deaths

The WHO defines a maternal death as one that occurs while the woman is pregnant, during childbirth or within 42 days of termination of pregnancy from a cause directly related to or aggravated by the pregnancy or its management and not an accidental or incidental cause [26]. In the 2010 census, all deaths of females aged 12-49 years reported to have taken place in the 12-months prior to the census attracted further probing to determine the time-of-death relative to the pregnancy state. The first probing question was: "Did the death occur while pregnant?" If the answer was "no" to this question, the respondent was asked: "Did the death occur during childbirth?", and if the answer was "no" to this too, a third question was posed: "Did the death occur during the 6 weeks period following the end of pregnancy, irrespective of the way the pregnancy ended?" [15]. A "yes" to any of the three questions was used to estimate the number of pregnancy-related deaths. No attempt was made to ascertain whether the cause of death was actually related to the pregnancy, and thus "pregnancy-related deaths" is a more appropriate term than maternal deaths.

Formal methods for assessing the accuracy of pregnancy-related deaths (PR deaths) recorded in a census are unavailable [23]. We used proxy methods to evaluate the plausibility of pregnancy-related deaths recorded;

i. We first reviewed the numbers of deaths recorded overall and within each 5-year age group.
ii. We computed and reviewed the proportions of pregnancy-related deaths based on reported time-of-death

iii. We computed and reviewed the proportions of total female deaths that were pregnancy-related (PMDF) overall and within each 5-year age group

iv. We computed and reviewed the percent share of pregnancy-related deaths within each 5-year age group.

v. We finally computed and reviewed the crude pregnancy-related mortality ratio (PRMRatio) overall and for each of the 5-year age group.

We applied three options in estimating the PRMRatios; *no adjustment*, *partial adjustment* (deaths only) and *full adjustment* (both deaths and live births). In the first option, deaths and live births as recorded in the 2010 census were used to estimate the PRMRatios. In the second option, adjustment was made to deaths using adjustment factors from the combined GGB-SEG. Pregnancy-related deaths were adjusted on the assumption that the direction and magnitude of reporting completeness was similar to that estimated for total deaths. In the third option, adjustment was also made to live births using an adjusted factor obtained by averaging the PF ratios for age groups 20-24, 25-29 and 30-34.

Ethical approval

Both censuses were conducted under the Census and Statistics ACT 127 of the laws of Zambia [27]. The Central Statistical Office (CSO) is mandated to conduct censuses and surveys as prescribed by the ACT and guided by national and international requirements for data on Zambia. The data used in this study is publicly available in a series of census tabulation reports [28]. Use of such data does not require ethical approval [29].

Results

The evaluation results indicated errors in the data on age; ASAI values of 34.2 and 38.5 were estimated for population age distributions recorded for rural and urban areas, respectively, in the 2000 census. Marginal improvements in age reporting occurred between the two censuses

as ASAI values of 31.2 and 36.6 were estimated for the population age distributions in rural and urban areas respectively in the 2010 census. Therefore age data from both censuses could be classified as inaccurate. Further evidence of this was found in age heaping, common in both censuses at ages ending in 0, 5, 8 and 2. The ASAI values for age-at-death were much higher, 48.7 for rural areas and 65.7 for urban areas respectively, indicating highly inaccurate age-at-death data. We adjusted both the age distributions of the population and deaths using smoothing with the *Arriaga technique,* a method that applies mild smoothing and maintains the original distribution totals [17].

The numbers of reproductive age women, CEB and live births 12-months prior to the census are presented in **Table 1**, together with the results of the cohort PF ratio method. Results indicated higher cohort parities (completed fertility) compared to cumulated “current” fertility at each age. The age-specific PF ratios were greater than unity in both rural and urban areas, indicating underreporting of live births and a need for upward adjustment. The adjusted number of live births 12-months prior to the 2010 census was 357,784 in rural areas and 191,216 in urban areas.

A total of 26,427 deaths of women aged 15-49 were recorded for the 12-months prior to the 2010 census; 13,640 deaths in rural areas and 12,786 deaths in urban areas. For rural areas, all three methods of evaluation (GGB, SEG & combined GGB-SEG) indicated underreporting of female deaths, while in urban areas the three methods indicated over-reporting of deaths (**Table 2**). In rural areas, the percentage point difference between the GGB and SEG estimates of deaths coverage was 18%, twice the difference in urban areas. For both rural and urban areas, deaths coverage was differential by age as observed from the plots of the SEG and combined GGB-SEG (**Figure 1** & **Figure 2**) showing that the estimates were not aligned along a straight line. (The same was also the case for the GGB plots; data not shown.) However, deaths coverage by age was more stable in rural areas compared to urban areas

(where the line was highly curvilinear; see **Figure 2**). The coverage estimates from the combined GGB-SEG were used to adjust recorded deaths. Summary measures of adult mortality are also provided in **Table 2**. The number of deaths in early adulthood (before age 40) was higher than in late adulthood (age group 40-60) in both rural and urban areas. However, the probability of dying was high in late adulthood relative to the probability of dying in early adulthood. The probability of dying between age 15 and 50 was 39% in rural areas and 25% in urban areas, while that of dying between age 15 and 60 was 50% in rural and 34% in urban areas..

About half of all recorded pregnancy-related deaths in the 2010 census were reported to have occurred during the antepartum period (while the woman was pregnant); 48% in rural areas and 50% in urban areas, while postpartum deaths constituted 28% in rural areas and 17% in urban areas. A total of 2,445 pregnancy-related deaths were recorded in rural areas, representing a PMDF of 17.9%, while in urban areas, 1,252 pregnancy-related deaths were recorded, representing a PMDF of 9.8% (**Table 3**). Pregnancy-related deaths as a proportion of total deaths of women was highest among young women aged 15-19, particularly in rural areas where the PMDF in this age group was 44% compared to 17% in urban areas. However, women in the age group 25-29 had the highest proportion of total pregnancy-related deaths recorded in both rural and urban areas (**Figure 3**). The crude PRMRatio was marginally higher in urban areas; 846/100,000 live births compared to 831/100,000 live births in rural areas (**Table 3**). The age-specific crude PRMRatios were higher among urban women aged 15-19 and older than 35 years compared to their rural peers (**Figure 4**). Adjustment of pregnancy-related deaths and live births resulted in the adjusted PRMRatio of 789/100,000 live births overall; 960/100,000 live births in rural areas and 470/100,000 in urban areas.

Discussion

Census-based estimates show very high adult female mortality and particularly high pregnancy-related mortality in both rural and urban areas of Zambia. Adjusted mortality was particularly high in rural areas. A woman aged 15 in rural areas had a chance of dying before her fiftieth birthday of *one-in-three* compared to *one-in-four* for her peer in urban areas. The chance of dying before her sixtieth birthday increased to *one-in-two* in rural areas and *one-in-three* in urban areas. In rural areas, *one-in-six* deaths of women aged 15-49 was pregnancy-related and in urban areas, the proportion was *one-in-ten*. The adjusted PRMRatios were 960/100,000 live births and 470/100,000 live births in rural and urban areas respectively.

Direct measurement of adult mortality in Zambia using household deaths collected in a census has not been successful prior to the 2010 census due to poor field implementation and questionnaire designs. In the 1969 census, enumerators confused questions on household deaths with questions on dead children among those ever born to reproductive age women [21]. In the 1990 census, household deaths were collected and disaggregated by sex, but age at death was never included [22]. The 2010 census was the first attempt to measure pregnancy-related mortality in the census [15]. This study shows both the potential and challenges of measuring mortality in a census. For both rural and urban areas, adult female mortality overall and pregnancy-related mortality were high, before and after adjustment for reporting completeness. When compared with other estimates for Zambia from the same time period, our estimates are much higher. For example, the census PMDF of 15.3% is much higher than the UN estimate of 9.1% for the year 2010, and the census PRMRatio is high compared to the UN estimated MMRatio of 440/100,000 live births (95% Uncertainty Range: 220-790) [30]. These differences partly arise from the UN estimates being based on modelling of data from a number of different sources, and from the fact that the UN modelling includes downward adjustment of reported pregnancy-related deaths to arrive at an estimate of actual maternal deaths. The census estimates are also higher than the estimated

PMDF of 9.5% from the 2013/14 ZDHS for the period 2007-2013 [6]. This discrepancy was somewhat surprising because a comparison by *Hill et al.* [31] of the “household deaths” and “sibling history” approaches to measuring pregnancy-related mortality applied to surveys found close agreement in the estimates of the PRMRatios. When age distributions of the PMDF are compared, possible sources for the difference in the overall estimate emerge. The census PMDF was 35% for women aged 15-19 and 19% for women aged 25-29 compared with 4% and 7% respectively for the 2013/14 ZDHS. Since data on pregnancy-related mortality both from the census and the DHS have substantial limitations and neither data source can be regarded as a gold standard, we are unable conclude which of the estimates are most likely to be valid.

One potential reporting problem affecting both a census and surveys pertains to misclassification of deaths. Maternal deaths are known to be concentrated around childbirth or soon after and the majority are usually due to haemorrhage [32-34]. In the 2010 Census the majority of pregnancy-related deaths were, however, concentrated in the antepartum period, in both rural and urban areas. It is possible that the sequenced order of asking about the timing of death could have biased the final tally. The use of three questions in a sequence was meant to improve recall and hence improve the quality of data collected. However, no formal validation has been done to determine how well the questions worked, including assessing whether the order of the questions affects the level of reporting bias [35]. A maternal mortality study in Bangladesh found misclassification of the timing of deaths when responses given to direct household questions were checked against information collected using verbal autopsy; 20% of pregnancy-related deaths were found to have been misclassified by households as having occurred during pregnancy [36]. This indicates the need to further validate these questions before use in future censuses to reduce misclassifications of deaths.

The adjusted PRMRatio was higher in rural areas than in urban areas. This was as expected given the challenging reproductive health situation in rural areas: Early childbearing is more

common in rural areas, where 36% of young women aged 15-19 have initiated childbearing compared to 20% in urban areas [6], and pregnant adolescents have poor access and utilisation of antenatal care services [37], increasing the risk of maternal death if they develop complications. Only 56% of women deliver in health facilities in rural areas and 52% have skilled attendants during delivery compared to 89% of women delivering in health facilities, and 88% having assistance of skilled attendants in urban areas [6]. Long distances to health facilities and lack of health workers prevent women in rural areas from accessing maternal health services [38-42]. Endemic malaria, anaemia and malnutrition, which are more prevalent in rural areas, also contribute to the high pregnancy-related mortality in Zambia.

In a high prevalence country like Zambia, HIV/AIDS has been found to be the major cause of young adult female mortality [43] and thus likely to substantially affect pregnancy-related mortality. The HIV prevalence was 21% in urban areas and 10% in rural areas of Zambia in 2013-14 among women aged 15-49 [6], and the UN estimates between 15% and 30% of maternal deaths in Zambia to be a result of HIV infection [4, 30]. Due to elevated mortality, mainly from the HIV/AIDS epidemic, the UN projected life expectancy at birth of 45.6 years for the period 2010-2015 is 10 years lower than it would be in the absence of HIV/AIDS [44]. The census-based estimates of the adult conditional probabilities of dying before age 50 (${}_{35}q_{15}$) and before age 60 (${}_{45}q_{15}$) were somewhat high, but plausible in depicting the prevailing level of mortality due to HIV/AIDS, and the observed high mortality in early adulthood relative to mortality in late adulthood could be a reflection of the impact of the epidemic [43, 45, 46]. The scaling up of free anti-retroviral therapy in public health facilities started around 2004-05 and is likely to some extent have reduce mortality. However, by 2010, coverage was well below 50% and with still substantial HIV-related mortality [47].

Evaluation studies of census-based measurement of pregnancy-related mortality emphasise the need for rigorous data evaluation before making any estimates [8]. We applied standard

data evaluation methods for use with census data. Results indicated errors in recorded population age, age-at-death, live births and deaths; hence adjustments to correct for the observed deficiencies in the data were made before final estimates could be generated. Where data quality is high, adjustments can be avoided as they could introduce their own biases [16]. Both the GGB and GGB-SEG showed too high coverage of urban female deaths; 1.79 and 1.39 respectively. However, coverage of rural female deaths was low; 0.72 and 0.71 for the GGB and GGB-SEG, respectively. Although under-reporting of deaths is usually more likely, over-reporting is equally possible. Hill *et al.* [11] found as much as 20-30% over-reporting of deaths in the Nicaragua and Paraguay censuses using the GGB. Over-reporting was attributed to possible confusion with the reference periods [11].

Confusion with the reference period is also possible in the 2010 census since deaths were required for the period October 2009-October 2010, but this would have affected all household deaths and not only urban female deaths. Another possible source of over-reporting of deaths would arise from the conduct of funerals. In Zambia, a death attracts funeral gatherings for days and it is not uncommon for such gatherings to be hosted away from the original home where the death occurred. Funeral hosting may be determined by several factors, including the capacity to meet funeral expenses. In such a case, the census might record the same death twice at two different homes. If there was no systematic over-reporting of urban female deaths in the 2010 census, coverage would be similar for male and female deaths. The GGB estimate for urban males was 0.97 and the GGB-SEG was 0.86, which indicates a relatively lower coverage of male than female deaths. It is possible that the high coverage of urban female deaths to be a data quality issue due to over-reporting. We made further assessments of the data by computing age standardised crude death rates (ASCDRs) and age-specific mortality rates (ASMR) for females in rural and urban areas. Urban female mortality was marginally high than in rural area; Urban ASCDR was 12.65 compared with

12.38 for rural. The age standardised ASMR for women over the age 25 was much higher among urban females compared to rural females in the same age group (results not shown). Higher mortality in urban areas compared to rural areas is a bit odd given relatively better living conditions and access to health services in urban areas. The results could therefore reinforce the findings that urban female deaths were actually overstated.

The result (over-reported urban female deaths) could equally have been a case of possible violation of basic assumptions of the DDMs. All the three methods, GGB, SEG and the combined GGB-SEG used have certain assumptions, and if these are violated, they could perform poorly [48]. Although age data was inaccurate in both censuses, surprisingly, age reporting accuracy was relatively better in rural areas than in urban areas for both population age and age-at-death. Smoothing may have corrected some, but not all the errors. A sensitivity analysis by *Hill et al.* [25] found the methods to be more robust to age misreporting than variations in deaths coverage. The latter could have affected urban female deaths as shown by the "curvy" shape of both the SEG and GGB-SEG instead of the expected straight line with constant deaths coverage by age.

Live births 12-months prior to the 2010 census were significantly adjusted upwards for both rural and urban areas, following the outcome of the cohort PF ratio method assessment. Although the method is unlikely to be affected by changing fertility over time, the method can be affected by changing mortality levels among reproductive age women during the period [20, 49]. The assumption that there are no differences in fertility of women interviewed and those that died during the intercensal period maybe hard to sustain given the high mortality among reproductive age women in Zambia. High fertility women may also face greater risk of dying from pregnancy-related causes since they are more often pregnant.

A major limitation of our study stems from the inability to adjust for rural-urban migration due to lack of data. Census-based migration data is affected by under-recording of actual migration volumes and directions during a lengthy intercensal period like 10 years [16]. Although the 2010 census indicated a positive rural-urban net migration of females aged 10-19 during the 2000-2010 intercensal period, the indicated volume was very small [50]. We therefore assumed zero migration effect in our application of the DDMs as required. However, the likelihood that this assumption was sustained for the 10-year intercensal period is low, and therefore violation could to some extent have affected the operations of the methods. Further, our study could have been affected by information bias, which could not be addressed even with the methods used to evaluate the data. Although training was provided, we cannot rule out that data collectors could have also contributed to information bias by making wrong entries or failure to correctly translate the questions into local languages where needed. However, the effect of this is likely to be negligible, since office editing was conducted before data entry and field practices during training included questionnaire translations into local languages.

Conclusion

Census-based estimates showed very high adult female mortality and particularly high pregnancy-related mortality in both rural and urban areas 12-months prior to the 2010 census. However, significant adjustments were necessary due to evidence of errors in the data on population age, age-at-death, live births and deaths. The adjustments resulted in more plausible mortality differentials between rural and urban areas, albeit with still very high mortality in both. The adjusted PRMRatio was twice higher in rural than in urban areas. Future censuses should incorporate strategies for improving the completeness of recording and quality of data collected.

List of Abbreviations

ASAI	Age-sex accuracy index
ASCDR	Age standardised crude death rate
ASFR	Age-specific fertility rate
ASMR	Age-specific mortality rate
CEB	Children ever born
CI	Confidence interval
CSO	Central Statistical Office
DDM	Death Distribution Method
DHS	Demographic and Health Survey
GGB	General Growth Balance
HIV	Human Immuno-deficiency Virus
LMIC	Low and Middle Income Country
MDG	Millennium Development Goal
MMRatio	Maternal mortality ratio
PMDF	Proportion of female deaths that are pregnancy-related
PRMRatio	Pregnancy-related mortality ratio
SEG	Synthetic Extinct Generation
UN	United Nations
WHO	World Health Organisation
${}_{35}q_{15}$	Conditional probability of dying between exact age 15 and 50
${}_{45}q_{15}$	Conditional probability of dying between exact age 15 and 60
${}_{20}q_{20}/{}_{20}q_{40}$	Probability of dying between exact age 20 and 40 relative to dying between exact age 40 and 60
${}_{30}d_{10}/{}_{20}d_{40}$	Number of life table deaths between exact age 10 and 40 relative to deaths between exact age 40 and 60
${}_{20}d_{20}/{}_{20}d_{40}$	Number of life table deaths between exact age 20 and 40 relative to deaths between exact age 40 and 60.

Competing interest

The authors declare that they have no competing interest

Author's contribution

RB participated in the planning and execution of the 2010 census, the conceptualisation of the study, data analysis, interpretation and drafting of the manuscript. **KF** participated in data analysis, interpretation, drafting and review of the manuscript. **IFS** participated in data analysis, interpretation, drafting and review of the manuscript.

Acknowledgement

This study is part of a PhD training programme in organised research and is fully supported by the Norwegian State Educational Loan Fund, Lånekassen. The authors also acknowledge the contribution of Therese Marianne Istad to the preparation of the graphics.

References

1. United Nations General Assembly. **United Nations Millennium Declaration.** New York: United Nations Secretariat; 2000.
2. Abouzahr C. **New estimates of maternal mortality and how to interpret them: choice or confusion?** Reprod Health Matters. 2011; **19:**117-128.
3. Boerma JT, Stansfield SK. **Health statistics now: are we making the right investments?** The Lancet. 2007; **369:**779-786.
4. WHO, UNFPA, UNICEF, The World Bank. The United Nations Population Division. **Trends in Maternal Mortality: 1990 to 2013.** Geneva: World Health Organisation; 2014.
5. United Nations Statistical Division. *Principles and recommendations for population and housing censuses* Rev. 2 edn. New York: United Nations 2008.
6. Central Statistical Office. Ministry of Health, ICF Macro International. **Zambia Demographic and Health Survey, 2013-14.** Rockville, Maryland, USA; 2014.
7. Stanton C, Hobcraft J, Hill K, Kodjogbe N, Mapeta WT, Munene F, Naghavi M, Rabeza V, Sisouphanthong B, Campbell O. **Every death counts: measurement of maternal mortality via a census.** Bull World Health Organ. 2001; **79:**657-664.
8. Hill K, Stanton C. **Measuring maternal mortality through the census: rapier or bludgeon?** J Pop Research. 2011; **28:**31-47.
9. Moultrie TA, Dorrington RE, Hill AG, Hill K, Timaeus IM, Zaba B, Editors. **Tools for Demographic Estimation**. Paris: International Union for the Scientific Study of Population (IUSSP); 2013.
10. Graham WJ, Ahmed S, Stanton C, Abou-Zahr CL, Campbell OMR. **Measuring maternal mortality: An overview of opportunities and options for developing countries.** BMC Medicine. 2008; **6**.
11. Hill K, Queiroz BL, Wong L, Plata J, Del Popolo F, Rosales J, Stanton C. **Estimating pregnancy-related mortality from census data: experience in Latin America.** Bull World Health Organ. 2009; **87:**288-295.
12. Leone T. **Measuring Differential Maternal Mortality Using Census Data in Developing Countries.** Population, Space and Place. 2013.

13. Garenne M, McCaa R, Nacro K. **Maternal mortality in South Africa in 2001: From demographic census to epidemiological investigation.** Popul Health Metr. 2008; **6:**4.
14. Hill K, Thomas K, AbouZahr C, Walker N, Say L, Inoue M, Suzuki E, Maternal Mortality Working Group. **Estimates of maternal mortality worldwide between 1990 and 2005: an assessment of available data.** The Lancet. 2007; **370:**1311-1319.
15. Central Statistical Office. **Zambia 2010 Census of Population and Housing: Population Summary Report.** Lusaka: Central Statistical Office; 2012.
16. Shryock HS, Siegel JS. *The Methods and Materials of Demography, 2nd Edition.* London: US Bureau of the Census; 2004.
17. US Census Bureau. **Population Analysis with Microcomputers.** Washington DC; 1994.
18. Central Statistical Office. **2000 Census of Population and Housing: National Analytical Report, vol. 10.** Lusaka: Central Statistical Office; 2003.
19. Central Statistical Office. **2010 Census of Population and Housing: National Analytical Report.** Lusaka: Central Statistical Office; 2012.
20. United Nations. **Manual X: Indirect Techniques for Demographic Estimation.** New York: United Nations; 1983.
21. Central Statistical Office. **Census of Population and Housing 1969: Final Report, Volume 1-Total Zambia.** Lusaka: Central Statistical Office; 1973.
22. Central Statistical Office. **Zambia Census of Population, Housing and Agriculture, 1990: Analytical Report Volume 10.** Lusaka: Central Statistical Office; 1995.
23. World Health Organization. **WHO guidance for measuring maternal mortality from a census.** Geneva: World Health Organisation; 2013.
24. United Nations. **Methods for estimating adult mortality.** New York:United Nations; 2002.
25. Hill K, You D, Choi Y. **Death distribution methods for estimating adult mortality: sensitivity analysis with simulated data error.** Demographic Research. 2009; **21:**235-254.
26. World Health Organization. **International statistical classification of diseases and related heath problems.-10th revision, 2008 edition.** Geneva: World Health Organisation; 2009.
27. **The Census and Statistics Act.** Republic of Zambia: Constitution of the Republic of Zambia; 1994.
28. Central Statistical Office. **2010 Zambia Census of Population and Housing: National Desscriptive Tables, Vol. 11.** Lusaka: Central Statistical Office; 2012:396.
29. ICO. **Anonymisation: managing data protection risk code of practice.** Cheshire; 2012.
30. WHO, UNFPA, UNICEF, and The World Bank. **Trends in Maternal Mortality: 1990 to 2010.** Geneva: World Health Organisation; 2012.
31. Hill K, El Arifeen S, Koenig M, Al-Sabir A, Jamil K, Raggers H. **How should we measure maternal mortality in the developing world? A comparison of household deaths and sibling history approaches.** Bull World Health Organ. 2006; **84:**173-180.
32. Campbell O, Graham WJ. **Strategies for reducing maternal mortality: getting on with what works.** The Lancet. 2006; **368:**1284–1299.
33. Say L, Chou D, Gemmill A, Tunçalp Ö, Moller AB, Daniel J, Gülmezoglu AM, Temmenerman M, Alkema L. **Global causes of maternal death: a WHO systematic analysis.** The Lancet. 2014; **2**.
34. Ronsmans C, Graham WJ, on behalf of The Lancet Maternal Survival Series steering group. **Maternal mortality: who, when, where, and why.** The Lancet. 2006; **368:**1189-1200.
35. Hill K, Stanton C, Levin M, Queiroz BL, Choi Y, Mahanani AWR, AbouZahr C. **Measuring Maternal Mortality from a Census: Guidelines for Potential Users, 2nd Revision, 2009.** Cambridge.World Health Organisation; 2010.
36. National Institute of Population Research and Training (NIPORT), Measure Evaluation, and icddr,b. **Bangladesh Maternal Mortality and Health Care Survey 2010.** Dhaka.NIPORT; 2012.
37. Magadia MA, Agwandab AO, Obare FO. **A comparative analysis of the use of maternal health services between teenagers and older mothers in sub-Saharan Africa: Evidence from Demographic and Health Surveys (DHS).** Soc Sci Med. 2006; **64:**15.
38. Kyei NA, Chansa C, Gabrysch S. **Quality of antenatal care in Zambia: A National Assessment.** BMC Pregnancy and Childbirth. 2012.

39. Kyei NNA, Campbell OMR, Gabrysch S. **The Influence of Distance and Level of Service Provision on Antenatal Care Use in Rural Zambia.** PLoS One. 2012.
40. Gabrysch S, Simushi V, Campbell OMR. **Availability and distribution of, and geographic access to emergency obstetric care in Zambia.** International Journal of Gynecology and Obstetrics. 2011.
41. Levine AC, Marsh RH, Nelson SW, Viola-Tyler L, Burke TF. **Measuring access to emergency obstetric care in rural Zambia.** Int J Emerg Med. 2008;7.
42. Ng'anjo-Phiri S, Fylkesnes K, Lorena-Ruano A, Marie-Moland K. **'Born before arrival': user and provider perspectives on health facility childbirths in Kapiri Mposhi district, Zambia.** BMC Pregnancy and Childbirth. 2014;**14**.
43. Dzekedzeke K, Siziya S, Fylkesnes K. **The impact of HIV infection on adult mortality in some communities in Zambia: a cohort study.** Trop Med Int Health. 2008; **13:**152-161.
44. United Nations. **World Mortality Report 2011.** New York: United Nations; 2012.
45. GBD 2013 Mortality and Causes of Death Collaborators. **Global, regional, and national age–sex specific all-cause and cause-specific mortality for 240 causes of death, 1990–2013: a systematic analysis for the Global Burden of Disease Study 2013.** The Lancet. 2015; **385:**117–171.
46. Mudenda SS, Kamocha S, Mswia R, Conkling M, Sikanyiti P, Potter D, Mayaka WC, Marx MA. **Feasibility of using a World Health Organization-standard methodology for Sample Vital Registration with Verbal Autopsy (SAVVY) to report leading causes of death in Zambia: results of a pilot in four provinces, 2010.** Popul Health Metr. 2011; **9:**40.
47. UNAIDS. **The Gap Report.** Geneva. UNAIDS; 2014.
48. Murray CJ, Rajaratnam JK, Marcus J, Laakso T, Lopez AD. **What can we conclude from death registration? Improved methods for evaluating completeness.** *PLoS Med.* 2010.
49. Newell C. *Methods and Models in Demography.* New York. Guilford Press; 1988.
50. Central Statistical Office. **Migration and Urbanisation Analytical Report.** Lusaka. Central Statistical Office; 2013.

List of Tables

Table A: Results of the application of cohort PF ratio method to fertility data for rural and urban areas for the intercensal period 2000-2010

	Rural															
	2000 census					2010 census					Application of cohort PF ratio method					
Age Group	Number of Women	Children Ever Born Alive	Children Born in last year	Age-Specific Fertility Rates	Average Parity P	Number of Women	Children Ever Born Alive	Children Born in last year	Age-Specific Fertility Rates	Average Parity P	Parity Change	Synthetic Cohort Parity	Age-Specific Fertility Rates	Cumulated Fertility to Age x	Parity Equivalent F	Ratio P/F
15-19	328,766	113,801	37,364	0.114	0.35	418,087	121,237	41,899	0.100	0.29	0.290	0.29	0.107	0.00	0.26	1.10
20-24	283,827	456,590	72,720	0.256	1.61	339,832	539,787	84,736	0.249	1.59	1.588	1.59	0.253	0.53	1.29	1.23
25-29	232,022	685,824	56,057	0.242	2.96	277,415	897,227	72,639	0.262	3.23	2.888	3.18	0.252	1.80	2.57	1.24
30-34	171,981	749,596	36,379	0.212	4.36	219,798	970,606	47,489	0.216	4.42	2.807	4.40	0.214	3.06	3.71	1.18
35-39	134,471	778,153	23,385	0.174	5.79	174,947	977,140	31,641	0.181	5.59	2.630	5.81	0.177	4.13	4.69	1.24
40-44	102,396	697,947	9,268	0.091	6.82	132,609	790,430	12,286	0.093	5.96	1.602	6.00	0.092	5.01	5.31	1.13
45-49	83,693	570,474	2,882	0.034	6.82	104,276	695,798	3,441	0.033	6.67	0.886	6.69	0.034	5.47	5.42	1.24
**Total**	**1,337,156**	**4,052,385**	**238,055**	**TFR= 5.61**		**1,666,964**	**4,992,225**	**294,131**	**TFR= 5.67**				**TFR= 5.64**			
	**Urban**															
15-19	210,914	46,597	14,270	0.068	0.22	323,150	54,574	17,100	0.053	0.17	0.169	0.17	0.060	0.00	0.14	1.18
20-24	195,623	224,804	31,597	0.162	1.15	291,998	282,823	43,534	0.149	0.97	0.969	0.97	0.155	0.30	0.77	1.27
25-29	160,364	347,923	24,210	0.151	2.17	245,106	522,434	42,062	0.172	2.13	1.911	2.08	0.161	1.08	1.57	1.32
30-34	108,011	382,196	14,464	0.134	3.54	184,220	582,924	26,577	0.144	3.16	2.015	2.98	0.139	1.88	2.32	1.29
35-39	79,602	397,839	7,671	0.096	5.00	138,963	548,664	13,811	0.099	3.95	1.779	3.86	0.098	2.58	2.90	1.33
40-44	58,991	362,437	2,414	0.041	6.14	91,831	420,391	3,864	0.042	4.58	1.039	4.02	0.041	3.07	3.20	1.26
45-49	42,351	269,331	678	0.016	6.36	66,771	384,966	998	0.015	5.77	0.768	4.63	0.015	3.28	3.25	1.42
**Total**	**855,856**	**2,031,127**	**95,304**	**TFR= 3.34**		**1,342,039**	**2,796,776**	**147,946**	**TFR= 3.37**				**TFR= 3.35**			

Table B: Summary results of data evaluation and estimated adult female mortality for the period October 2009-2010, Zambia

**Summary result of mortality data evaluation**							
Statistic	Age Range	Rural			Urban		
		GGB	SEG	GGB-SEG	GGB	SEG	GGB-SEG
Slope	5+ to 65+	1.382			0.56		
Intersection		0.008			-0.007		
K1:k2		1.078			0.934		
Coverage		0.724	0.548	0.712	1.785	1.876	1.394
Final Coverage		0.712					1.785

**Summary measures of adult female mortality**								
Indicator	Rural				Urban			
	Crude	GGB	SEG	GGB-SEG	Crude	GGB	SEG	GGB-SEG
${}_{35}q_{15}$	0.287	0.373	0.460	0.389	0.337	0.205	0.196	0.248
${}_{45}q_{15}$	0.375	0.477	0.576	0.496	0.449	0.284	0.272	0.339
${}_{20}q_{20}/{}_{20}q_{40}$	0.833	0.839	0.847	0.841	0.711	0.694	0.693	0.699
${}_{30}d_{10}/{}_{20}d_{40}$			1.63	1.38			0.98	1.13
${}_{20}d_{20}/{}_{20}d_{40}$			1.32	1.15			0.88	1.00

Table C: Pregnancy-related mortality using different adjustment options; option I (No adjustment), option II (Partial adjustment-deaths only) and option III (Full adjustment-both deaths and births)

Option I (No adjustment)						Option II (Partial adjustment)					Option III (Full adjustment)				
**Zambia Total**															
Age group	Recorded Deaths	Recorded PR Deaths	Recorded Live Births	Crude PRMRatio	Crude PMDF	Adj. Deaths	Adj. PR Deaths	Recorded Live Births	Partially Adj. PRMRatio	Adj. PMDF	Adj. Deaths	Adj. PR Deaths	Adj. Live Births	Adj. PRMRatio	Adj. PMDF
15-19	1,834	560	58,999	949	0.31	1,953	680	58,999	1,152	0.35	1,953	680	73,068	930	0.35
20-24	4,611	715	128,270	557	0.16	4,981	842	128,270	656	0.17	4,981	842	159,340	528	0.17
25-29	4,969	865	114,701	754	0.17	5,320	997	114,701	869	0.19	5,320	997	142,723	698	0.19
30-34	5,144	753	74,066	1,017	0.15	5,481	866	74,066	1,170	0.16	5,481	866	92,116	940	0.16
35-39	4,449	466	45,452	1,025	0.10	4,747	546	45,452	1,200	0.11	4,747	546	56,339	968	0.11
40-44	3,038	227	16,150	1,406	0.07	3,277	273	16,150	1,690	0.08	3,277	273	19,939	1,369	0.08
45-49	2,382	111	4,439	2,501	0.05	2,582	131	4,439	2,956	0.05	2,582	131	5,476	2,397	0.05
**Total**	**26,427**	**3,697**	**442,077**	**836**	**14.0%**	**28,342**	**4,334**	**442,077**	**980**	**15.3%**	**28,342**	**4,334**	**549,000**	**789**	**15.3%**
**Zambia Rural (Adj. factors: deaths=1.42; births=1.22)**															
15-19	927	404	41,899	964	0.44	1,303	568	41,899	1,355	0.44	1,303	568	50,966	1,114	0.44
20-24	2,431	478	84,736	564	0.20	3,417	672	84,736	793	0.20	3,417	672	103,074	652	0.20
25-29	2,553	547	72,639	753	0.21	3,588	769	72,639	1,058	0.21	3,588	769	88,359	870	0.21
30-34	2,603	474	47,489	998	0.18	3,658	666	47,489	1,403	0.18	3,658	666	57,766	1,153	0.18
35-39	2,260	307	31,641	970	0.14	3,176	431	31,641	1,364	0.14	3,176	431	38,488	1,121	0.14
40-44	1,596	160	12,286	1,302	0.10	2,243	225	12,286	1,830	0.10	2,243	225	14,945	1,505	0.10
45-49	1,270	75	3,441	2,180	0.06	1,785	105	3,441	3,063	0.06	1,785	105	4,186	2,518	0.06
**Total**	**13,640**	**2,445**	**294,131**	**831**	**17.9%**	**19,171**	**3,436**	**294,131**	**1,168**	**17.9%**	**19,171**	**3,436**	**357,784**	**960**	**17.9%**
**Zambia Urban (Adj. factors: deaths=0.72; births=1.29)**															
15-19	906	156	17,100	912	0.17	650	112	17,100	654	0.17	650	112	22,101	506	0.17
20-24	2,180	237	43,534	544	0.11	1,564	170	43,534	390	0.11	1,564	170	56,266	302	0.11
25-29	2,416	318	42,062	756	0.13	1,733	228	42,062	542	0.13	1,733	228	54,364	420	0.13
30-34	2,541	279	26,577	1,050	0.11	1,822	200	26,577	753	0.11	1,822	200	34,350	583	0.11
35-39	2,189	159	13,811	1,151	0.07	1,570	114	13,811	826	0.07	1,570	114	17,850	639	0.07
40-44	1,442	67	3,864	1,734	0.05	1,034	48	3,864	1,244	0.05	1,034	48	4,994	962	0.05
45-49	1,112	36	998	3,607	0.03	798	26	998	2,587	0.03	798	26	1,290	2,002	0.03
**Total**	**12,786**	**1,252**	**147,946**	**846**	**9.8%**	**9,171**	**898**	**147,946**	**607**	**9.8%**	**9,171**	**898**	**191,216**	**470**	**9.8%**

List of figures

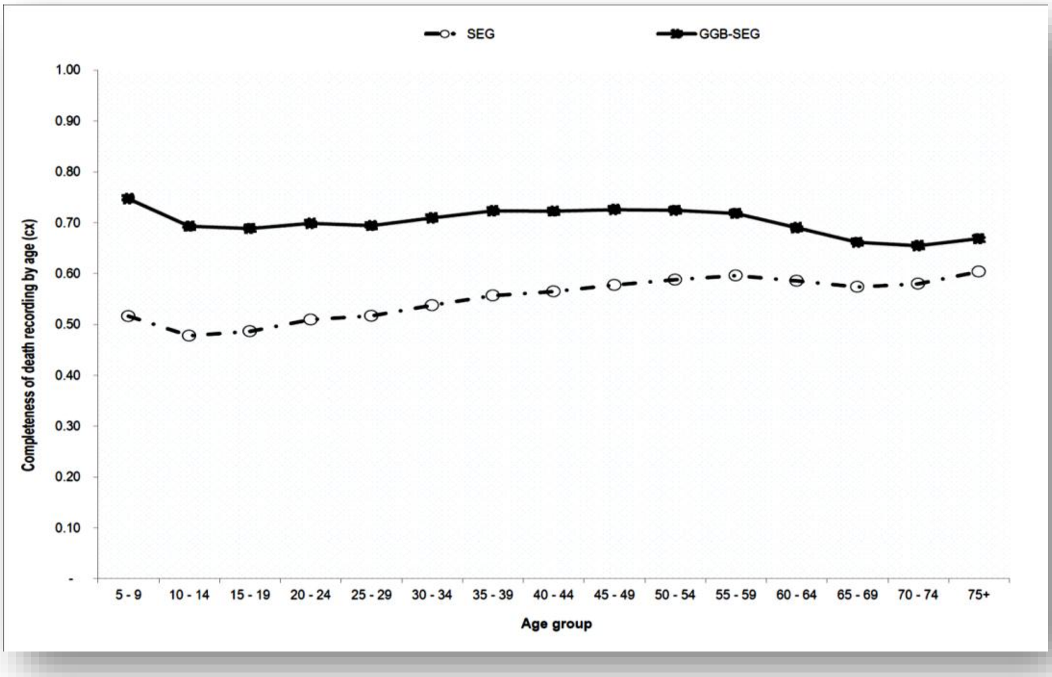

Figure A: Completeness of deaths recording by age: results of the SEG & SEG-adjusted for Zambia rural

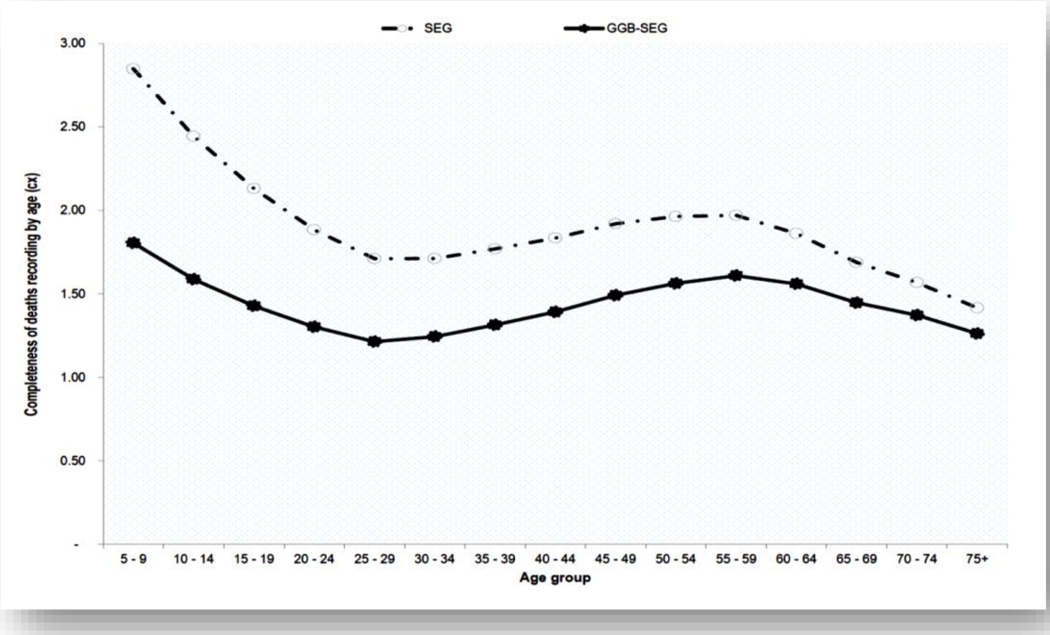

Figure B: Completeness of deaths recording by age: results of the SEG & SEG-adjusted for Zambia urban

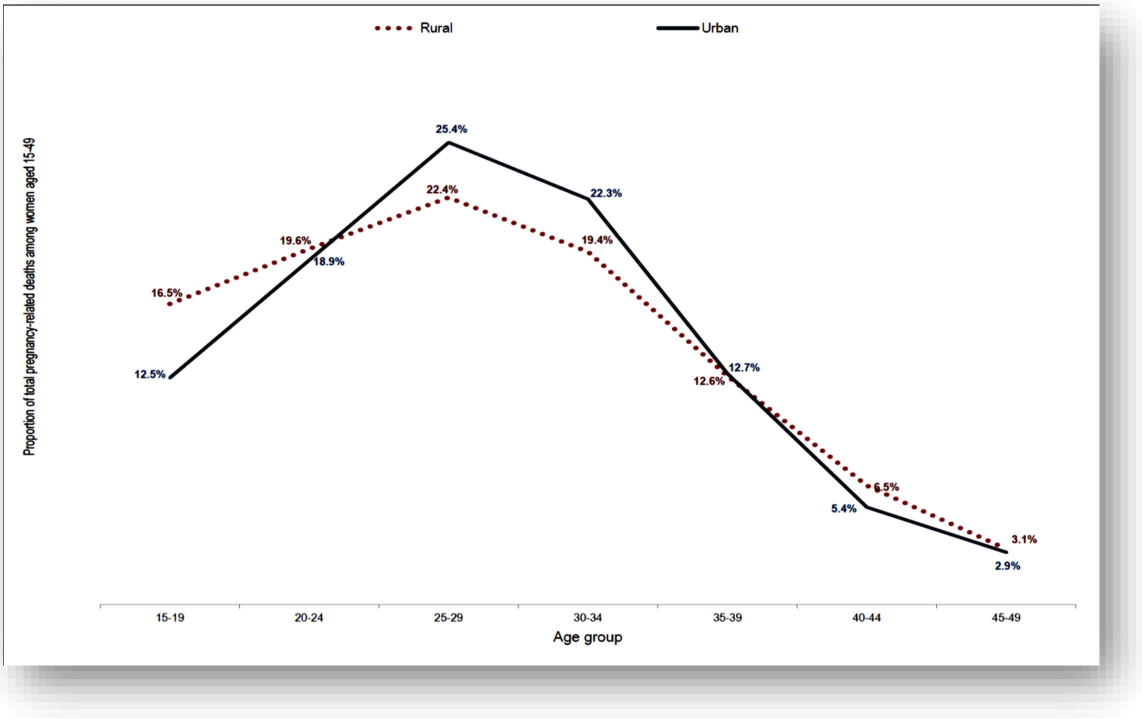

Figure C: Percent of total pregnancy-related deaths for each 5-year age group within rural and urban areas

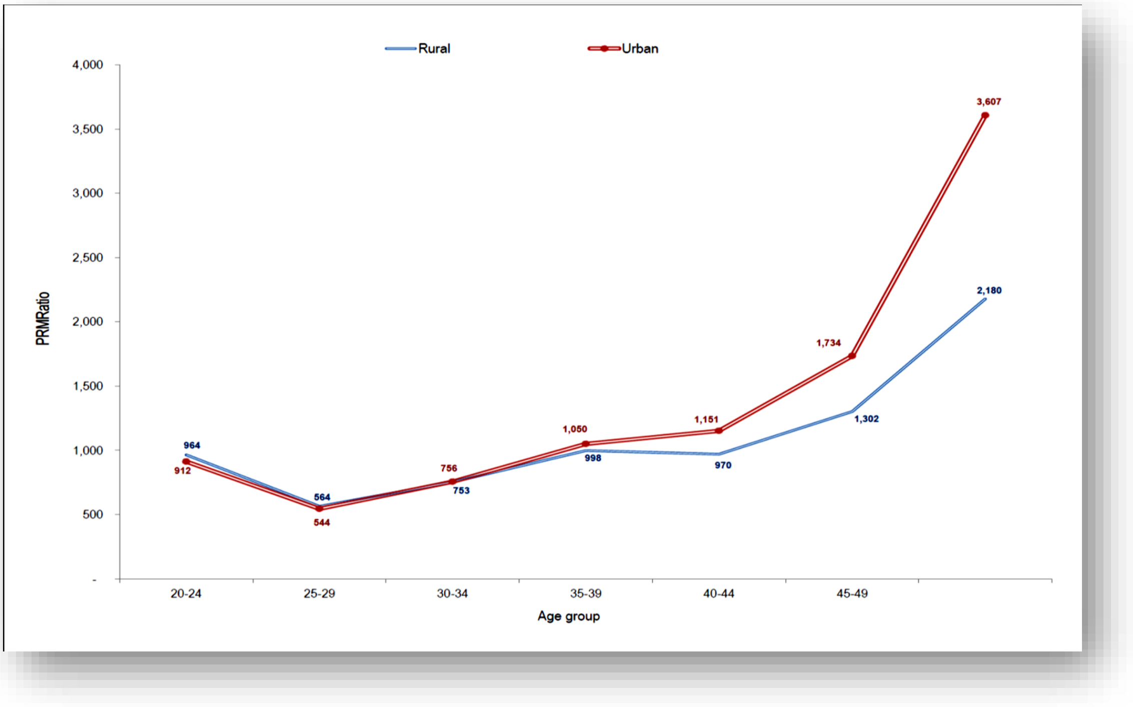

Figure D: Age-specific crude PRMRatios by rural and urban residence
