# Supplementary material for: Impact of Pregnancy-Related Deaths on Female Life Expectancy in Zambia: Application of Life Table Techniques to Census Data
Source: PLoS One. 2015 Oct 29;10(10):e0141689. doi: 10.1371/journal.pone.0141689 (PMC4626102; doi:10.1371/journal.pone.0141689)
Supplement: S2 Text — (PDF) [file pone.0141689.s005.pdf]

Table 1: Associated single decrement life tables (ASDTs), assuming full elimination of pregnancy-related deaths (i); Zambia 2010, Total (Full version).

Age x	${}_nD_x$	${}_nD^i_x$	R^{-i}	l_x	${}_np_x$	${}_na_x$	e_x	${}_n^*p^{-i}_x$	${}^*l^{-i}_x$	${}_n^*a^{-i}_x$	${}_nd_x$	${}_nq_x$	${}_n^*q^{-i}_x$	${}_nd^{-i}_x$	${}_n^*L^{-i}_x$	${}^*T^{-i}_x$	${}^*e^{-i}_x$	${}_nM_x$	*PGLE*$^{-i}_x$
	All-cause deaths	*Pregnancy-related deaths*	$R^{-i}=({}_nD_x-{}_nD^i_x)/{}_nD_x$	l_x	${}_np_x=l_{x+1}/l_x$		All-cause life expectancy	${}_n^*p^{-i}_x=[{}_np_x]^{R-i}$	${}^*l^{-i}_{x+n}={}_*l_x^{-l}\ .\ {}_n^*p^{-i}x$		${}_nd_x=l_x-l_{x+n}$	${}_nq_x=1-{}_np_x$	${}_n^*q^i_x=1-{}_n{}^*p^{-i}_x$	${}_nd^{-i}_x={}_nq^{-i}_x.l^{-i}_x$	${}_n^*L^{-i}_x=n.{}^*l^{-i}_{x+n}+{}_n{}^*a^{-i}_x\ .\ {}_nd^i_x$	${}^*T^{-i}_x=\sum_n{}^*L^{-i}_{x+n}$	${}^*e^{-i}_x={}^*T^{-i}_{x+n}/\ {}^*l^{-i}_x$	ASMR	*PGLE*$^{-i}_x={}^*e^{-i}_x-e_x$
0	14,094	0	1.0000	100,000	0.9368	0.22	51.43	0.93678	100,000	0.22	6,322	0.0632	0.0632	6,322	95,056	5,277,963	52.78	0.0665	1.35
1	12,126	0	1.0000	93,678	0.9457	1.83	53.89	0.94572	93,678	1.83	5,085	0.0543	0.0543	5,085	363,660	5,182,907	55.33	0.0140	1.44
5	14,366	0	1.0000	88,593	0.9288	1.95	52.88	0.92884	88,593	1.95	6,304	0.0712	0.0712	6,304	423,764	4,819,247	54.40	0.0149	1.52
10	4,125	104	0.9747	82,289	0.9761	2.49	51.78	0.97668	82,289	2.49	1,969	0.0239	0.0233	1,919	406,637	4,395,484	53.42	0.0048	1.64
15	1,953	680	0.6520	80,321	0.9869	2.69	47.99	0.99144	80,370	2.69	1,052	0.0131	0.0086	688	400,261	3,988,847	49.63	0.0026	1.65
20	4,981	842	0.8310	79,269	0.9613	2.67	43.59	0.96773	79,682	2.67	3,068	0.0387	0.0323	2,571	392,427	3,588,586	45.04	0.0079	1.45
25	5,320	997	0.8126	76,201	0.9503	2.61	40.23	0.95942	77,111	2.61	3,787	0.0497	0.0406	3,129	378,067	3,196,159	41.45	0.0102	1.22
30	5,481	866	0.8419	72,413	0.9344	2.53	37.20	0.94445	73,981	2.53	4,752	0.0656	0.0555	4,109	359,741	2,818,092	38.09	0.0136	0.89
35	4,747	546	0.8851	67,661	0.9272	2.47	34.64	0.93527	69,872	2.47	4,927	0.0728	0.0647	4,523	337,927	2,458,351	35.18	0.0151	0.55
40	3,277	273	0.9167	62,734	0.9296	2.47	32.16	0.93525	65,349	2.47	4,418	0.0704	0.0647	4,231	316,062	2,120,424	32.45	0.0146	0.28
45	2,582	131	0.9492	58,316	0.9273	2.50	29.41	0.93082	61,118	2.50	4,242	0.0727	0.0692	4,228	295,024	1,804,362	29.52	0.0151	0.11
50	2,025	0	1.0000	54,074	0.9260	2.47	26.52	0.92600	56,890	2.47	4,002	0.0740	0.0740	4,210	273,796	1,509,339	26.53	0.0154	0.01
55	1,849	0	1.0000	50,072	0.9153	2.52	23.45	0.91533	52,680	2.52	4,240	0.0847	0.0847	4,460	252,349	1,235,543	23.45	0.0177	0.01
60	1,893	0	1.0000	45,833	0.8959	2.56	20.38	0.89587	48,219	2.56	4,773	0.1041	0.1041	5,021	228,835	983,193	20.39	0.0219	0.01
65	1,917	0	1.0000	41,060	0.8664	2.59	17.45	0.86636	43,198	2.59	5,487	0.1336	0.1336	5,773	202,055	754,358	17.46	0.0286	0.01
70	1,960	0	1.0000	35,573	0.8167	2.55	14.74	0.81669	37,425	2.55	6,521	0.1833	0.1833	6,860	170,319	552,304	14.76	0.0403	0.01
75	2,024	0	1.0000	29,052	0.7132	2.92	12.48	0.71324	30,565	2.92	8,331	0.2868	0.2868	8,765	134,592	381,984	12.50	0.0653	0.01
80	3,012	0	1.0000	20,721	0.0000	11.76	11.35	0.00000	21,800	11.76	20,721	1.0000	1.0000	21,800	247,392	247,392	11.35	0.0881	-
Total deaths	87,734	4,439																	

Table 2: Associated single decrement life tables (ASDTs), assuming full elimination of pregnancy-related deaths (i); Zambia 2010, Rural (Full version).

Age x	${}_nD_x$	${}_nD^i_x$	*R^{-i}*	l_x	${}_np_x$	${}_na_x$	e_x	${}_n{}^*p^{-i}_x$	${}^*l^{-i}_x$	${}_n{}^*a^{-i}_x$	${}_nd_x$	${}_nq_x$	${}_n{}^*q^{-i}_x$	${}_nd^{-i}_x$	${}_n{}^*L^{-i}_x$	${}^*T^{-i}_x$	${}^*e^{-i}_x$	${}_nM_x$	*$PGLE^{-i}_x$*
	All-cause deaths	*Pregnancy-related deaths*	*$R^{-i}=({}_nD_x-{}_nD^i_x)/{}_nD_x$*	l_x	${}_np_x=l_{x+1}/l_x$		All-cause life expectancy	${}_n{}^*p^{-i}_x=[{}_np_x]^{R-i}$	${}^*l^{-i}_{x+n}={}_*l_x^{-l}\ .\ {}_n{}^*p^{-i}x$		${}_nd_x=l_x-l_{x+n}$	${}_nq_x=1-{}_np_x$	${}_n{}^*q^i_x=1-{}_n{}^*p^{-i}_x$	${}_nd^{-i}_x={}_nq^{-i}_x.l^{-i}_x$	${}_n{}^*L^{-i}_x=n.{}^*l^{-i}_{x+n}+{}_n{}^*a^{-i}_x\ .\ {}_nd^i_x$	${}^*T^{-i}_x=\sum_n{}^*L^{-i}_{x+n}$	${}^*e^{-i}_x={}^*T^{-i}_{x+n}/\ {}^*l^{-i}_x$	ASMR	*$PGLE^{-i}_x={}^*e^{-i}_x-e_x$*
0	11,524	0	1.0000	100,000	0.9238	0.28	46.58	0.9238	100,000	0.28	7,616	0.0762	0.0762	7,616	94,506	4,827,306	48.27	0.0806	1.69
1	10,172	0	1.0000	92,384	0.9324	1.85	49.40	0.9324	92,384	1.85	6,242	0.0676	0.0676	6,242	356,112	4,732,800	51.23	0.0175	1.83
5	11,854	0	1.0000	86,142	0.9083	1.99	48.85	0.9083	86,142	1.99	7,896	0.0917	0.0917	7,896	406,961	4,376,688	50.81	0.0194	1.96
10	3,317	87	0.9737	78,246	0.9676	2.46	48.58	0.9684	78,246	2.46	2,536	0.0324	0.0316	2,471	384,956	3,969,728	50.73	0.0066	2.16
15	1,303	568	0.5643	75,709	0.9845	2.66	45.12	0.9912	75,775	2.66	1,171	0.0155	0.0088	664	377,324	3,584,772	47.31	0.0031	2.19
20	3,417	672	0.8034	74,538	0.9509	2.66	40.79	0.9603	75,111	2.66	3,661	0.0491	0.0397	2,979	368,581	3,207,448	42.70	0.0101	1.91
25	3,588	769	0.7857	70,877	0.9373	2.60	37.76	0.9504	72,132	2.60	4,445	0.0627	0.0496	3,579	352,059	2,838,867	39.36	0.0129	1.60
30	3,658	666	0.8179	66,432	0.9201	2.52	35.11	0.9341	68,554	2.52	5,309	0.0799	0.0659	4,515	331,565	2,486,808	36.28	0.0166	1.16
35	3,176	431	0.8642	61,123	0.9132	2.47	32.94	0.9245	64,039	2.47	5,305	0.0868	0.0755	4,832	307,969	2,155,243	33.66	0.0182	0.71
40	2,243	225	0.8998	55,818	0.9189	2.46	30.84	0.9267	59,207	2.46	4,527	0.0811	0.0733	4,339	285,031	1,847,274	31.20	0.0169	0.36
45	1,785	105	0.9409	51,290	0.9179	2.50	28.34	0.9226	54,868	2.50	4,209	0.0821	0.0774	4,247	263,711	1,562,243	28.47	0.0171	0.13
50	1,387	0	1.0000	47,082	0.9168	2.47	25.65	0.9168	50,621	2.47	3,917	0.0832	0.0832	4,212	242,468	1,298,532	25.65	0.0174	-
55	1,291	0	1.0000	43,164	0.9066	2.52	22.76	0.9066	46,409	2.52	4,031	0.0934	0.0934	4,334	221,287	1,056,064	22.76	0.0196	-
60	1,361	0	1.0000	39,134	0.8920	2.56	19.84	0.8920	42,075	2.56	4,228	0.1080	0.1080	4,546	199,262	834,777	19.84	0.0228	-
65	1,418	0	1.0000	34,906	0.8625	2.61	16.93	0.8625	37,529	2.61	4,799	0.1375	0.1375	5,159	175,323	635,514	16.93	0.0294	-
70	1,492	0	1.0000	30,107	0.8106	2.57	14.22	0.8106	32,370	2.57	5,703	0.1894	0.1894	6,131	146,977	460,191	14.22	0.0417	-
75	1,582	0	1.0000	24,404	0.6895	3.03	11.94	0.6895	26,239	3.03	7,578	0.3105	0.3105	8,147	115,165	313,214	11.94	0.0707	-
80	2,267	0	1.0000	16,827	0.0000	10.95	10.95	0.0000	18,091	10.95	16,827	1.0000	1.0000	18,091	198,049	198,049	10.95	0.0913	-
Total deaths	66,835	3,523																	

Table 3: Associated single decrement life tables (ASDTs), assuming full elimination of pregnancy-related deaths (i); Zambia 2010, Urban (Full version).

Age x	$_nD_x$	$_nD^i_x$	R^{-i}	l_x	$_np_x$	$_na_x$	e_x	$_n{}^*p^{-i}_x$	$^*l^{-i}_x$	$_n{}^*a^{-i}_x$	$_nd_x$	$_nq_x$	$_n{}^*q^{-i}_x$	$_nd^{-i}_x$	$_n{}^*L^{-i}_x$	$^*T^{-i}_x$	$^*e^{-i}_x$	$_nM_x$	$PGLE^{-i}_x$
	All-cause deaths	*Pregnancy-related deaths*	$R^{-i}=({}_nD_x-{}_nD^i_x)/{}_nD_x$	l_x	$_np_x=l_{x+1}/l_x$		All-cause life expectancy	$_n{}^*p^{-i}_x=[{}_np_x]^{R-i}$	$^*l^{-i}_{x+n}={}_*l_x^{-l}\ .\ {}_n{}^*p^{-i}x$		$_nd_x=l_x-l_{x+n}$	$_nq_x=1-{}_np_x$	$_n{}^*q^i_x=1-{}_n{}^*p^{-i}_x$	$_nd^{-i}_x={}_nq^{-i}_x.l^{-i}_x$	$_n{}^*L^{-i}_x=n.{}^*l^{-i}_{x+n}+{}_n{}^*a^{-i}_x\ .\ {}_nd^i_x$	$^*T^{-i}_x=\sum_n{}^*L^{-i}_{x+n}$	$^*e^{-i}_x={}^*T^{-i}_{x+n}/\ {}^*l^{-i}_x$	ASMR	$PGLE^{-i}_x={}^*e^{-i}_x-e_x$
0	2,570	0	1.0000	100,000	0.9638	0.16	61.02	0.9638	100,000	0.16	3,615	0.0362	0.0362	3,615	96,954	6,179,892	61.80	0.0373	0.78
1	1,955	0	1.0000	96,385	0.9732	1.80	62.31	0.9732	96,385	1.80	2,587	0.0268	0.0268	2,587	379,855	6,082,938	63.11	0.0068	0.81
5	2,512	0	1.0000	93,798	0.9653	1.87	59.97	0.9653	93,798	1.87	3,252	0.0347	0.0347	3,252	458,818	5,703,083	60.80	0.0071	0.83
10	809	17	0.9787	90,545	0.9885	2.58	57.06	0.9887	90,545	2.58	1,044	0.0115	0.0113	1,022	450,250	5,244,265	57.92	0.0023	0.86
15	650	112	0.8279	89,502	0.9900	2.73	52.70	0.9917	89,524	2.73	896	0.0100	0.0083	743	445,934	4,794,015	53.55	0.0020	0.85
20	1,564	170	0.8913	88,605	0.9736	2.69	48.20	0.9764	88,781	2.69	2,343	0.0264	0.0236	2,096	439,065	4,348,082	48.98	0.0054	0.77
25	1,733	228	0.8684	86,262	0.9652	2.62	44.44	0.9697	86,685	2.62	2,999	0.0348	0.0303	2,623	427,185	3,909,017	45.09	0.0071	0.66
30	1,822	200	0.8902	83,263	0.9517	2.54	40.94	0.9569	84,062	2.54	4,020	0.0483	0.0431	3,623	411,381	3,481,832	41.42	0.0099	0.47
35	1,570	114	0.9274	79,243	0.9451	2.48	37.89	0.9490	80,439	2.48	4,353	0.0549	0.0510	4,106	391,840	3,070,451	38.17	0.0113	0.28
40	1,034	48	0.9535	74,890	0.9452	2.49	34.95	0.9477	76,333	2.49	4,100	0.0548	0.0523	3,990	371,659	2,678,611	35.09	0.0113	0.14
45	798	26	0.9676	70,790	0.9420	2.50	31.83	0.9438	72,343	2.50	4,107	0.0580	0.0562	4,065	351,571	2,306,953	31.89	0.0119	0.06
50	638	0	1.0000	66,683	0.9403	2.46	28.64	0.9403	68,278	2.46	3,979	0.0597	0.0597	4,074	331,060	1,955,382	28.64	0.0123	-
55	558	0	1.0000	62,704	0.9304	2.55	25.30	0.9304	64,204	2.55	4,365	0.0696	0.0696	4,469	310,058	1,624,321	25.30	0.0144	-
60	532	0	1.0000	58,339	0.9047	2.57	22.00	0.9047	59,735	2.57	5,559	0.0953	0.0953	5,691	284,861	1,314,263	22.00	0.0200	-
65	498	0	1.0000	52,781	0.8760	2.55	19.05	0.8760	54,043	2.55	6,544	0.1240	0.1240	6,700	253,814	1,029,402	19.05	0.0264	-
70	469	0	1.0000	46,237	0.8333	2.50	16.38	0.8333	47,343	2.50	7,708	0.1667	0.1667	7,893	216,980	775,588	16.38	0.0364	-
75	442	0	1.0000	38,529	0.7706	2.71	14.16	0.7706	39,450	2.71	8,840	0.2294	0.2294	9,051	176,544	558,608	14.16	0.0513	-
80	745	0	1.0000	29,689	0.0000	12.57	12.57	0.0000	30,399	12.57	29,689	1.0000	1.0000	30,399	382,064	382,064	12.57	0.0796	-
Total deaths	20,899	915																	
