# Supplementary material for: Impact of Pregnancy-Related Deaths on Female Life Expectancy in Zambia: Application of Life Table Techniques to Census Data
Source: PLoS One. 2015 Oct 29;10(10):e0141689. doi: 10.1371/journal.pone.0141689 (PMC4626102; doi:10.1371/journal.pone.0141689)
Supplement: S3 Text — (PDF) [file pone.0141689.s006.pdf]

# ZAMBIA

# 2010 CENSUS OF POPULATION AND HOUSING

# VOLUME 11
# NATIONAL DESCRIPTIVE TABLES

Published by

Central Statistical Office
Nationalist Road
P.O. Box 31908
Lusaka

www.zamstats.gov.zm
email: info@zamstats.gov.zm

November, 2012

# Table of Contents


| Table of Contents | Page |
|---|---|
| **Preface** | **ix** |
| **Population Size Tables** | **1** |
| Table A1: Population (De facto) by Province, District and Sex, Rural/Urban, Zambia 2010 | 2 |
| Table A1: Population (De Jure) by Province, District and Sex, Rural/Urban, Zambia 2010 | 4 |
| Table A2: Population Distribution by Urban Centres (15,000 and above Population) by Province, District and Sex, Zambia 2010 | 6 |
| Table A3: Population (De Jure), Households, Average Household Size, Size of Household and Sex of Household Head by Province, Zambia 2010 | 7 |
| Table A4: Population (De Jure) by Single Year Age and Sex by Province, Rural/Urban, Zambia 2010 | 8 |
| Table A4: Population (De Jure) by Single Years Age and Sex by Province, Rural/Urban Zambia 2010 | 12 |
| Table A4: Population (De Jure) by Single Years Age and Sex by Province, Rural/Urban Zambia 2010 | 16 |
| Table A5: Population, Land Area and Population Density by Province and District, Zambia, 2010 | 20 |
| Table A6: Population and Average Annual Growth Rate by Province and District, Rural/Urban, Zambia, 2000 and 2010 | 22 |
| Table A7: Population (De facto) by 5 Year Age Group, Household Membership Status and Sex, Rural/Urban, Zambia 2010 | 24 |
| Table A8: Population (De facto) by 5 Year Age Group, Sex and Citizenship, Rural/Urban, Zambia 2010 | 26 |
| Table A9: Total Population (De Facto) by 5 Year Age Group, Citizenship and Place of Birth, Rural/Urban, Zambia 2010 | 28 |
| Table A10: Households and Population by (De jure) Sex, Province, District, Constituency and Ward, Zambia 2010 | 30 |
| Table A11: Population (De Jure) by Age 0-14 Years (Children), 15-34 Years (Youths) and Total Population 0-34 Years by Sex, Province, District, Constituency and Ward, Zambia 2010 | 61 |
| **Social Tables** | **93** |
| Table B1: Population 15 Years and Older by 5 Year Age Group, Sex and Marital Status, Rural/Urban, Zambia 2010 | 94 |
| Table B2: Percent Distribution of Population 15 Years and Above by 5 Year Age Group, Sex, Selected Exact Ages at First Marriage, Never Married and Median Age at First Marriage, Rural/Urban, Zambia 2010 | 97 |
| Table B3: Population Aged 0-17 Years With and Without Birth Certificates by Sex and Age (Single and Grouped), Rural/Urban, Zambia 2010 | 100 |
| Table B4i. Population (De facto) 3 Years and Above (Excluding the Deaf and Dumb and Persons with a Speech Impairment) by Sex and Predominant Language of Communication, Rural/Urban, Zambia 2010 | 102 |
| Table B4 ii. Population (De facto) by Sex and Ethnicity, Rural/Urban, Zambia 2010 | 104 |
| Table B5: Population (De facto) by Religion, 5 Year Age Group and Sex, Rural/Urban, Zambia 2010 | 106 |
| Table B5: Population (De facto) by Religion, 5 Year Age Group and Sex, Rural/Urban, Zambia 2010 | 107 |
| Table B5: Population (De facto) by Religion, 5 Year Age Group and Sex, Rural/Urban, Zambia 2010 | 108 |
| Table B6: Population (De Facto) Aged 0-17 Years by 5 Year Age Group; Survival Status of Parents; Province and Rural/Urban, Zambia 2010 | 109 |
| Table B6: Population (De Facto) Aged 0-17 Years by 5 Year Age Group; Survival Status of Parents; Province and Rural/Urban, Zambia 2010 | 111 |
| Table B6: Population (De Facto) Aged 0-17 Years by 5 Year Age Group; Survival Status of Parents; Province and Rural/Urban, Zambia 2010 | 113 |
| Table B7: Population (De Facto) Aged 0-17 Years by 5 Year Age Group; Survival Status of Parents; Province and Rural/Urban, Zambia 2010 | 115 |
| Table B7: Population (De Facto) Aged 0-17 Years by 5 Year Age Group; Survival Status of Parents; Province and Rural/Urban, Zambia 2010 | 117 |
| Table B7: Population (De Facto) Aged 0-17 Years by 5 Year Age Group; Survival Status of Parents; Province and Rural/Urban, Zambia 2010 | 119 |

Table B8: Population (De Facto) Aged 0-17 Years by 5 Years Age Groups; Survival and Household Living Status of Parents; Province and Rural/Urban, Zambia 2010 121

Table B8: Population (De Facto) Aged 0-17 Years by 5 Years Age Groups and Survival; Household Living Status of Parents; Province and Rural, Zambia 2010 123

Table B8: Population (De Facto) Aged 0-17 Years by 5 Years Age Groups and Survival; Household Living Status of Parents; Province and Urban, Zambia 2010 125

Table B9: Type of Orphan as a Percent of Population Aged 0-17 Years by Province and Rural/Urban, Zambia 2010 127

Table B10: Type of Orphan as a Percent of Population Aged 0-17 Years by 5 Year Age Group and Sex, Rural/Urban, Zambia 2010 128

Table B11: Population of Orphans Aged 5-17 Years by Age (Single and Grouped), Sex and School Attendance, Rural/Urban, Zambia 2010 129

Table B12: Population of Orphans Aged 12-17 Years by Age, Sex and Economic Activity Status, Rural/Urban, Zambia 2010 130

Table B13: Population 16 Years and Older with National Registration Cards (NRCs) by Sex, Province and Constituency, Zambia 2010 132

Table B14: Population 18 Years and Older (Eligible Voters) and Registered Voters at the time of the Census by Sex, Province and Constituency, Zambia 2010 136

**Fertility Tables 139**

Table C1: Population of Women 12 Years and Older by Average Number of Children Ever Born, Children Ever Born Alive, Children Still Living, Children Dead, Sex of Child, Province and Rural/Urban, Zambia 2010 140

Table C2: Population of Women aged 12 Years and Older by Average Number of Children Ever Born Alive, Children Ever Born Alive, Children Still Living, Children Dead, Sex of Child, 5 Year Age Group and Rural/Urban, Zambia 2010 141

Table C2i. Population of Women 12-49 Years by Age Group, Number of Children Born Alive 12 Months Prior to the Census, Children Still Living, Children Dead, Sex of Child and Rural/Urban, Zambia 2010 142

Table C3: Population of Women 12 Years and Older by Number of Children Ever Born Alive, Children Still Living, Children Dead, Sex of Child, Age Group and Marital Status, Rural/Urban, Zambia 2010 143

Table C3: Population of Women 12 Years and Older by Number of Children Ever Born Alive, Children Still Living, Children Dead, Sex of Child, Age Group and Marital Status, Rural, Zambia 2010 145

Table C3i: Population of Women 12-49 Years by Number of Children Born Alive 12 Months Prior to the Census, Children Still Living, Children Dead, Sex of Child, Age Group and Marital Status, Rural/Urban, Zambia 2010 149

Table C3i: Population of Women 12-49 Years by Number of Children Born Alive 12 Months Prior to the Census, Children Still Living, Children Dead, Sex of Child, Age Group and Marital Status, Rural, Zambia 2010 151

Table C3i: Population of Women 12-49 Years by Number of Children Born Alive 12 Months Prior to the Census, Children Still Living, Children Dead, Sex of Child, Age Group and Marital Status, Urban, Zambia 2010 153

Table C4: Population of Women 12 Years and Older by Age Group, Average Number of Children Ever Born, Number of Children Ever Born Alive, Children Still Living, Children Dead, Sex of Child and Highest Educational Attainment, Rural/Urban, Zambia 2010 155

Table C4: Population of Women 12 Years and Older by Age Group, Average Number of Children Ever Born, Number of Children Ever Born Alive, Children Still Living, Children Dead, Sex of Child and Highest Educational Attainment, Rural, Zambia 2010 157

Table C4: Population of Women 12 Years and Older by Age Group, Average Number of Children Ever Born, Number of Children Ever Born Alive, Children Still Living, Children Dead, Sex of Child and Highest Educational Attainment, Urban, Zambia 2010 159

Table C4i: Population of Women 12-49 Years by Age Group, Number of Children Born Alive 12 Months Prior to the Census, Children Still Living, Children Dead, Sex of Child and Educational Attainment, Rural/Urban, Zambia 2010 161

Table C4i: Population of Women 12-49 Years by Age Group, Number of Children Born Alive 12 Months Prior to the Census, Children Still Living, Children Dead, Sex of Child and Educational Attainment, Rural, Zambia 2010 162

Table C4i: Population of Women 12-49 Years by Age Group, Number of Children Born Alive 12 Months Prior to the Census, Children Still Living, Children Dead, Sex of Child and Educational Attainment, Urban, Zambia 2010 163

Table C5: Population of Women 12 years and Older by Age Group, Number of Children Ever Born Alive, Children Still Living, Children Dead, Sex of Child and Economic Activity Status, Rural/Urban, Zambia 2010 164
Table C5: Population of Women 12 years and Older by Age Group, Number of Children Ever Born Alive, Children Still Living, Children Dead, Sex of Child and Economic Activity Status, Rural, Zambia 2010 165
Table C5: Population of Women 12 years and Older by Age Group, Number of Children Ever Born Alive, Children Still Living, Children Dead, Sex of Child and Economic Activity Status, Urban, Zambia 2010 166
Table C5i: Population of Women 12-49 Years by Age Group, Number of Children Born Alive 12 Months Prior to the Census, Children Still Living, Children Dead, Sex of Child and Economic Activity Status, Rural/Urban, Zambia 2010 167
Table C5i: Population of Women 12-49 Years by Age Group, Number of Children Born Alive 12 Months Prior to the Census, Children Still Living, Children Dead, Sex of Child and Economic Activity Status, Rural, Zambia 2010 168
Table C5i: Population of Women 12-49 Years by Age Group, Number of Children Born Alive 12 Months Prior to the Census, Children Still Living, Children Dead, Sex of Child and Economic Activity Status, Urban, Zambia 2010 169
Table C6: Population of Women 12 Years and Older by Age Group, Number of Children Ever Born, Total Children Ever Born and Averag Number of Children Ever Born and Rural/Urban, Zambia 2010 170
Table C7: Population of Women 12 Years and Older by Age Group, Average Number of Children Ever Born Alive, Children Ever Born Alive, Children Still Living, Children Dead, Sex of Child and Religion, Rural/Urban, Zambia 2010 171
Table C7: Population of Women 12 Years and Older by Age Group, Average Number of Children Ever Born Alive, Children Ever Born Alive, Children Still Living, Children Dead, Sex of Child and Religion, Rural, Zambia 2010 174
Table C7: Population of Women 12 Years and Older by Age Group, Average Number of Children Ever Born Alive, Children Ever Born Alive, Children Still Living, Children Dead, Sex of Child and Religion, Urban, Zambia 2010 177
Table C7i: Popuplation of Women Aged 12-49 Years By Age Group, Number of Children Born Alive 12 Months Prior to the Census, Children still Living, Children Dead and Religion, Rural/Urban, Zambia 2010 180
**Education Characteristics Tables** **183**
Table D1: Population (De Facto) 5 Years and Older by 5 Year Age Group, School Attendance and Province, Zambia 2010 184
Table D2: Population (De facto) 5 Years and Older by 5 Year Age Group, Sex and School Attendance, Rural/Urban, Zambia 2010 187
Table D2: Population (De facto) 5 Years and Older by 5 Year Age Group, Sex and School Attendance, Rural, Zambia 2010 188
Table D2: Population (De facto) 5 Years and Older by 5 Year Age Group, Sex and School Attendance, Urban, Zambia 2010 189
Table D2i: Population (De Facto) 5 Years and Older by Age (Single and Grouped) School Attendance and Rural/Urban, Zambia 2010 190
Table D2 i: Population (De Facto) 5 Years and Older by Age (Single and Grouped), School Attendance and Rural, Zambia 2010 193
Table D2i: Population (De Facto) 5 Years and Older by Age (Single and Grouped), School Attendance and Urban/Rural, Zambia 2010 196
Table D3: Population (De Facto) 5 Years and Older by Age (Single and Grouped), Sex, Literacy Rate and Rural/Urban, Zambia 2010 199
Table D3: Population (De Facto) 5 Years and Older by Age (Single and Grouped), Sex, Literacy Rate and Rural, Zambia 2010 200
Table D3: Population (De Facto) 5 Years and Older by Age (Single and Grouped), Sex, Literacy Rate and Urban, Zambia 2010 201
Table D4: Population (De Facto) 5 Years and Older by Age (Single and Grouped), Sex, Highest Level of Education Completed and Rural/Urban, Zambia 2010 202
Table D4: Population (De Facto) 5 Years and Older by Age (Single and Grouped), Sex, Highest Level of Education Completed and Rural, Zambia 2010 205
Table D4: Population (De Facto) 5 Years and Older by Age (Single and Grouped), Sex, Highest Level of Education Completed and Urban, Zambia 2010 208
Table D5: Population (De Facto) 5 Years and Older by Field of Study, Highest Professional/Vocational Qualification Completed and Sex, Zambia 2010 211
Table D6: Population (De Facto) by Sex, Current Primary School Attendance, Net and Gross Attendance Ratios and Rural/Urban, Zambia 2010 214

Table D7: Population (De Facto) by Sex, Current Secondary School Attendance, Net and Gross Attendance Ratios and Rural/Urban, Zambia 2010 214
**Economic Activity Tables** **215**
Table E1: Usually Working Population (De Facto) 12 Years and Older by Employment Status, Sex and Province, Zambia 2010 216
Table E2: Usually Working Population (De Facto) 12 Years and Older by Employment Status, Age Group, Sex and Rural/Urban, Zambia 2010 217
Table E2: Usually Working Population (De Facto) 12 Years and Older by Employment Status, Age Group, Sex and Rural, Zambia 2010 218
Table E2: Usually Working Population (De Facto) 12 Years and Older by Employment Status, Age Group, Sex and Urban, Zambia 2010 219
Table E3: Population (De Facto) 12 Years and Older by Economic Activity Status, Age Group, Sex and Rural/Urban, Zambia 2010 220
Table E3: Population (De Facto) 12 Years and Older by Economic Activity Status, Age Group, Sex and Urban, Zambia 2010 222
Table E4: Usually Working Population (De Facto) 12 Years and Older by Industry of Employment, Age Group, Sex and Rural/Urban, Zambia 2010 223
Table E4: Usually Working Population (De Facto) 12 Years and Older by Industry of Employment, Age Group, Sex and Rural, Zambia 2010 224
Table E4: Usually Working Population (De Facto) 12 Years and Older by Industry of Employment, Age Group, Sex and Urban, Zambia 2010 225
Table E5: Population (De Facto) 12 Years and Older by Age Group, Sex and Occupation Category, Rural/Urban, Zambia 2010 226
Table E5: Population (De Facto) 12 Years and Older by Age Group, Sex and Occupation Category, Rural, Zambia 2010 227
Table E5: Population (De Facto) 12 Years and Older by Age Group, Sex and Occupation Category, Urban, Zambia 2010 228
Table E6: Usually Working Population (De Facto) 12 Years and Older by Industry, Sex and Highest Level of Education Completed, Rural/Urban, Zambia 2010 229
Table E6: Usually Working Population (De Facto) 12 Years and Older by Industry, Sex and Highest Level of Education Completed, Rural, Zambia 2010 230
Table E6: Usually Working Population (De Facto) 12 Years and Older by Industry, Sex and Highest Level of Education Completed, Urban, Zambia 2010 231
Table E7: Usually Working Population (De Facto) 12 Years and Older by Occupation, Sex and Highest Level of Education Completed, Rural/Urban, Zambia 2010 232
Table E7: Usually Working Population (De Facto) 12 Years and Older by Occupation, Sex and Highest Level of Education Completed, Rural, Zambia 2010 233
Table E7: Usually Working Population (De Facto) 12 Years and Older by Occupation, Sex and Highest Level of Education Completed, Urban, Zambia 2010 234
Table E8: Usually Working Population (De Facto) 12 Years and Older by Age Group, Sex and Highest Level of Education Completed, Rural/Urban, Zambia 2010 235
Table E8: Usually Working Population (De Facto) 12 Years and Older by Age Group, Sex and Highest Level of Education Completed, Rural, Zambia 2010 236
Table E8: Usually Working Population (De Facto) 12 Years and Older by Age Group, Sex and Highest Level of Education Completed, Urban, Zambia 2010 237
Table E9: Usually Unemployed Population (De Facto) 12 Years and Older by Age Group, Sex and Highest Level of Education Completed, Rural/Urban, Zambia 2010 238
Table E9: Usually Unemployed Population (De Facto) 12 Years and Older by Age Group, Sex and Highest Level of Education Completed, Rural, Zambia 2010 239
Table E9: Usually Unemployed Population (De Facto) 12 Years and Older by Age Group, Sex and Highest Level of Education Completed, Urban, Zambia 2010 240
Table E10: Usually Working Population (De Facto) 12 Years and Older by Employment Status, Sex and Highest Professional/Vocational Qualification Completed, Rural/Urban, Zambia 2010 241
Table E11: Usually Working Population (De Facto) 12 Years and Older by Field of Study, Sex and Highest Professional/Vocational Qualification Completed, Rural/Urban, Zambia 2010 243
Table E11: Usually Working Population (De Facto) 12 Years and Older by Field of Study, Sex and Highest Professional/Vocational Qualification Completed, Rural, Zambia 2010 246
Table E11: Usually Working Population (De Facto) 12 Years and Older by Field of Study, Sex and Highest Professional/Vocational Qualification Completed, Urban, Zambia 2010 249
Table E12: Usually Working Population (De Facto) 12 Years and Older by Industry, Sex and Employment Status, Rural/Urban, Zambia 2010 252
Table E12: Usually Working Population (De Facto) 12 Years and Older by Industry, Sex and Employment Status, Rural, Zambia 2010 253

Table E12: Usually Working Population (De Facto) 12 Years and Older by Industry, Sex and Employment Status, Urban, Zambia 2010 254
Table E13: Usually Working Population (De Facto) 12 Years and Older by Occupation, Sex and Employment Status, Rural/Urban, Zambia 2010 255
Table E13: Usually Working Population (De Facto) 12 Years and Older by Occupation, Sex and Employment Status, Rural, Zambia 2010 256
Table E13: Usually Working Population (De Facto) 12 Years and Older by Occupation, Sex and Employment Status, Urban, Zambia 2010 257
**Disability Tables** **259**
Table F1: Population (De Facto) and Total Disabled Population by Sex, Province and Rural/Urban, Zambia 2010 260
Table F2: Population (De facto) and Total Disabled Population by 5 Year Age Group, Sex and Rural/Urban, Zambia, 2010 261
Table F3: Disabled Household Heads by Sex of Household Head, Number of Household Members and Employment Status, Rural/Urban, Zambia 2010 263
Table F3: Disabled Household Heads by Sex of Household Head, Number of Household Members and Employment Status, Rural, Zambia 2010 264
Table F3: Disabled Household Heads by Sex of Household Head, Number of Household Members and Employment Status, Urban, Zambia 2010 265
Table F4: Disabled Population (De Facto) 5 Years and Older by 5 Year Age Group, Sex and School Attendance, Rural/Urban, Zambia 2010 266
Table F4: Disabled Population (De Facto) 5 Years and Older by 5 Year Age Group, Sex and School Attendance, Rural, Zambia 2010 267
Table F4: Disabled Population (De Facto) 5 Years and Older by 5 Year Age Group, Sex and School Attendance, Urban, Zambia 2010 268
Table F5: Disabled Population (De Facto) 5 Years and Older by 5 Year Age Group and Highest Level of Education Completed, Rural/Urban, Zambia 2010 269
Table F5i: Disabled Population (De Facto) 5 Years and Older by 5 Year Age Group, Sex and Highest Level of Education Completed, Rural/Urban, Zambia 2010 271
Table F5ii: Disabled Population (De Facto) 5 Years and Older by 5 Year Age Group, Sex and Highest Level of Education Completed, Rural/Urban, Zambia 2010 273
Table F6: Disabled Population (De Facto) 12 Years and Older by 5 Year Age Group, Sex and Economic Activity, Rural/Urban, Zambia 2010 275
Table F6: Disabled Population (De Facto) 12 Years and Older by 5 Year Age Group, Sex and Economic Activity, Rural, Zambia 2010 276
Table F6: Disabled Population (De Facto) 12 Years and Older by 5 Year Age Group, Sex and Economic Activity, Urban, Zambia 2010 277
Table F7: Population (De Facto) and Albino Population by 5 Year Age Group and Sex, Rural/Urban, Zambia 2010 278
**Migration Tables** **281**
Table G1: Population Born Outside Zambia by Country of Birth, Age Group and Sex, Zambia 2010 282
Table G2: Population (De facto) by Rural/Urban Part of District of Birth, Age, Sex and District of Residence, Zambia 2010 285
Table G2: Population (De facto) by Rural Part of District of Birth, Age, Sex and District of Residence, Zambia 2010 288
Table G2: Population (De facto) by Urban Part of District of Birth, Age, Sex and District of Residence, Zambia 2010 291
Table G3: Population Born in Zambia by Sex, Province of Birth and Province of Residence, Zambia 2010 294
Table G4: Population (De Facto) and Immigrants by 5 Year Age Group, Duration of Stay in Zambia and Sex, Zambia 2010 295
Table G5: Migrant Population by Sex and Country of Citizenship, Rural/Urban, Zambian 2010 296
Table G6: Migrant Population by Purpose of Stay in Zambia, 5 Year Age Group, Sex and Province, Zambia 2010 300
Table G7: Migrant Population (5 Years and Older) by 5 Year Age Group , Sex and Educational Attainment, Rural/Urban, Zambia 2010 312
Table G7: Migrant Population (5 Years and Older) by 5 Year Age Group , Sex and Educational Attainment, Rural, Zambia 2010 313
Table G7: Migrant Population (5 Years and Older) by 5 Year Age Group , Sex and Educational Attainment, Urban, Zambia 2010 314
Table G8: Migrant Population 12 Years and Older by Employment Status, 5 Year Age Groups and Sex, Rural/Urban, Zambia 2010 315
Table G8: Migrant Population 12 Years and Older by Employment Status, 5 Year Age Groups and Sex, Rural, Zambia 2010 316
Table G8: Migrant Population 12 Years and Older by Employment Status, 5 Year Age Groups and Sex, Urban, Zambia 2010 317

| | |
|---|---|
| **Housing Tables** | **319** |
| Table H1: Households by Sex of Household Head and Province, Rural/Urban, Zambia 2010 | 320 |
| Table H2: Percent Distribution of Housing Units by Occupancy Status, Rural/Urban and Province, Zambia 2010 | 320 |
| Table H3:Number Residential Housing Units by Type of Housing Unit, Province and Rural/Urban, Zambia 2010 | 321 |
| Table H4: Number of Residential Housing Units, Households and Population (De Jure) by Construction Material of Walls and Roof, Rural/Urban, Zambia 2010 | 322 |
| Table H4: Number of Residential Housing Units, Households and Population (De Jure) by Construction Material of Walls and Roof, Rural, Zambia 2010 | 323 |
| Table H4: Number of Residential Housing Units, Households and Population (De Jure) by Construction Material of Walls and Roof, Urban, Zambia 2010 | 324 |
| Table H5: Number of Residential Housing Units, Households and Population (De Jure) by Construction Material of Walls and Floors, Rural/Urban, Zambia 2010 | 325 |
| Table H5: Number of Residential Housing Units, Households and Population (De Jure) by Construction Material of Walls and Floors, Rural, Zambia 2010 | 326 |
| Table H5: Number of Residential Housing Units, Households and Population (De Jure) by Construction Material of Walls and Floors, Urban, Zambia 2010 | 327 |
| Table H6i: Number of Households by Number of Living Rooms, Type of Occupancy and Rural/Urban, Zambia 2010 | 328 |
| Table H6ii: Number of Household by Number of Bedrooms Rooms, Type of Occupancy and Rural/Urban, Zambia 2010 | 328 |
| Table H7: Number of Households by Household Size, Number of Living Rooms and Rural/Urban, Zambia 2010 | 329 |
| Table H8: Number of Households by Household Size, Number of Bedrooms and Rural/Urban, Zambia 2010 | 330 |
| Table H9 : Number of Housing Units, Households and Population (DeJure) by Type of Housing Unit and Source of Energy for Lighting, Rural/Urban, Zambia 2010 | 331 |
| Table H9 : Number of Housing Units, Households and Population (DeJure) by Type of Housing Unit and Source of Energy for Lighting, Rural/Urban, Zambia 2010 | 332 |
| Table H9 : Number of Housing Units, Households and Population (DeJure) by Type of Housing Unit and Source of Energy for Lighting, Rural/Urban, Zambia 2010 | 333 |
| Table H10: Number of Housing Units, Households and Population (DeJure) by Type of Housing Unit and Source of Energy for Heating, Rural/Urban, Zambia 2010 | 334 |
| Table H10: Number of Housing Units, Households and Population (DeJure) by Type of Housing Unit and Source of Energy for Heating, Rural, Zambia 2010 | 335 |
| Table H10: Number of Housing Units, Households and Population (DeJure) by Type of Housing Unit and Source of Energy for Heating, Urban, Zambia 2010 | 336 |
| Table H11 : Number of Housing Units with/without Kitchen by Type of Housing Unit and Source of Energy for Cooking, Rural/Urban, Zambia 2010 | 337 |
| Table H11 : Number of Housing Units with/without Kitchen by Type of Housing Unit and Source of Energy for Cooking, Rural, Zambia 2010 | 338 |
| Table H11 : Number of Housing Units with/without Kitchen by Type of Housing Unit and Source of Energy for Cooking, Urban, Zambia 2010 | 339 |
| Table H12: Number of Households and Population (Dejure) by Type of Housing Unit and Source of Water Supply, Rural/Urban, Zambia 2010 | 340 |
| Table H12: Number of Households and Population (Dejure) by Type of Housing Unit and Source of Water Supply, Rural, Zambia 2010 | 341 |
| Table H12: Number of Households and Population (Dejure) by Type of Housing Unit and Source of Water Supply, Urban, Zambia 2010 | 342 |
| Table H13: Number of Households and Population by Type of Housing Unit and Source of Drinking Water, Rural/Urban, Zambia 2010 | 343 |
| Table H13: Number of Households and Population by Type of Housing Unit and Source of Drinking Water, Rural, Zambia 2010 | 344 |
| Table H13: Total Number of Households and Population by Type of Housing Unit and Source of Drinking Water, Urban, Zambia 2010 | 345 |
| Table H14: Number of Households by Ownership of Household Assets and Type of Housing Unit, Rural/Urban, Zambia 2010 | 346 |
| Table H15: Occupied Housing Units by Type of Housing Unit and How the Housing Units were Acquired, Rural/Urban, Zambia 2010 | 348 |
| Table H16: Occupied Housing Units and Housing Units rented from Employer by Type of Housing Unit and Type of Employer, Rural/Urban, Zambia 2010 | 349 |
| Table H17: Occupied Housing Units and Total Rented Housing Units by Type of Housing Unit and Source of Housing Unit Rented by Households, Rural/Urban, Zambia 2010 | 350 |
| Table H18: Number of Households with Access to a Toilet Facility by Type of Toilet Facility and Location of Toilet, Rural/Urban, Zambia 2010 | 351 |
| Table H19: Number of Households by Type of Housing Unit and Refuse Disposal, Rural/Urban, Zambia 2010 | 352 |

**Agriculture Tables** **353**
Table I1: Total Number of Households and Number of Households engaged in Agricultural Activity 12 Months prior to the Census by Sex of Household Head, Province and Rural/Urban, Zambia 2010 354
Table I2: Total Number of Households and Number of Households engaged in raising of Poultry/Livestock 12 Months prior to the Census by Sex of Household Head, Province and Rural/Urban, Zambia 2010 354
Table I3: Total Number of Households and Number of Households engaged in Fish Farming 12 Months prior to the Census by Sex of Household Head and, Province and Rural/Urban, Zambia 2010 355
Table I4: Total Number of Households and Number of Households engaged in Game Ranching 12 Months prior to the Census by Sex of Household Head, Province and Rural/Urban, Zambia 2010 355
Table I5: Total Number of Households and Number of Households Enganged in Raising of Poultry/Livestock 12 Months prior to the Census by Type of Poultry/Livestock and Province, Zambia 2010 356
Table I6: Total Number of Households and Number of Households Engaged in Crop Growing 12 Months Prior to the Census by Type of Crop and Province, Zambia 2010 356
General and Maternal Mortality Tables 357
Table J1: Reported Number of Deaths 12 Months Prior to the Census by Province, District, Sex and Rural/Urban, Zambia 2010 358
Table J2: Population (De facto) of Women 12-49 Years and Total Live Births 12 Months Prior to the Census by 5 Year Age Group, Maternal Mortality and Province, Rural/Urban, Zambia 2010 360
Table J3: Population (De Facto), Deaths 12 Months Prior to the Census and Age Specific Death Rates by Age (Single and Grouped) and Sex, Rural/Urban, Zambia 2010 371
Table J4: Population (De Facto) and Number of Household Deaths 12 months prior to the Census by Cause of Death, 5 Year Age Group, Sex and Province, Zambia 2010 373

2010 Census of Population and Housing Questionnaire 391

Persons involved in the Production of the Descriptive Tabulation Reports 396

# Preface

The 2010 Zambia Census of Population and Housing is the fifth national census of the Zambian population since independence in 1964. The 2010 Census covered both standard census topics as well as new ones. The inclusion of standard topics ensured the analysis of trends over time and facilitated comparison of past censuses with the 2010 Census in those areas. The inclusion of standard topics also allows for international comparability. The inclusion of new topics showed that the Zambian census is responsive to new data user needs and policy environment.

Standard topics covered included Fertility (Children Ever Born and Births in the period 12 months prior to the census), Mortality (Children Ever Born and Children Dead, Births in the period 12 months prior to the census and Children Dead), Migration (Internal and International) and Urbanization, Household and Housing characteristics, Education characteristics, Economic activity and Disability. However, within the standard topics like Household and Housing characteristics, new questions were added such as household access to an internet facility and household ownership of a mobile phone.

Some new topics and questions covered issues on orphanhood and fosterhood for children below the age of 18 years (i.e. those aged 0-17 years). A new question was also included for children below the age of 18 years on whether the child had a birth certificate or not.

The 2010 Census as with the last two censuses in 2000 and 1990 respectively also included a section on Agriculture with a new question on households engaged in game ranching in the period 12 months prior to the census included.

In order to effectively assess and monitor mortality patterns and trends, several questions on general and maternal mortality were included in the 2010 Census of Population and Housing. Information on the occurrence of deaths in the household during the period 12 months prior to the census, the sex of the deceased person, the age and cause of death was collected from all households interviewed. For any reported deaths of females aged 12-49 years, information on maternal related deaths (i.e. whether the death of the woman occurred while pregnant, during childbirth or within six weeks following the end of the pregnancy, irrespective of the way the pregnancy ended) was also collected.

Analysis of census data can only be achieved if conducted in a systematic manner. A comprehensive tabulation plan helps guide the analysis and output from a census. In the development of the series of tabulation reports, the Central Statistical Office (CSO) conducted a stakeholder consultative meeting in October 2011 to gain input from stakeholders on the tabulation and analysis plans.

Following these consultations, the 2010 Census Tabulation Plan was finalised and used as a guide in the development of this series of Descriptive Tabulation Reports. It must be noted that what is contained in this series of Descriptive Tabulation Reports are key tabulations based on national and international consideration for tabulation and analysis of topics covered in the 2010 Census. The tabulations have also taken into account submissions and input from stakeholders.

The Provincial Tabulation Reports run from Volume 1; Central Province to Volume 10; Western Province. The Volume 11 is the National Level Descriptive Tabulation Report. Within the Tabulation Reports, Tables have been organized by topic and run from Series A; General Population Tables to Series J; Disability Tables.

The 2010 Census of Population and Housing forms a rich resource of information on several topics covered during the census. This resource will be made available to users and researchers through the different series of analytical, thematic and descriptive tabulation reports. A huge attempt has been made by the CSO to produce key tabulations on different topics covered in the 2010 Census of Population and Housing, however individual users and researcher are encouraged to make special requests for tabulations not covered in this series of tabulation reports.

I would like to thank all CSO staff involved in the development of this important output from the 2010 Census of Population and Housing. I also thank and appreciate the support of the technical staff of the US Census Bureau during the development of the Tabulation Plan.

I extend my sincere gratitude to the Government of the Republic of Zambia (GRZ), its cooperating partners UNFPA, DfID, USAID and AfDB for supporting the 2010 Census of Population and Housing and ensuring that the Descriptive Tabulation Reports from the 2010 Census are made available to users and stakeholders timely.

John Kalumbi
**Director**
**CENSUS AND STATISTICS**

November, 2012

# Population Size Tables

## Table A1: Population (De facto) by Province, District and Sex, Rural/Urban, Zambia 2010

| Province and District | Total | | | Rural | | | Urban | | |
|---|---|---|---|---|---|---|---|---|---|
| | Total | Male | Female | Total | Male | Female | Total | Male | Female |
| **Zambia Total** | **12,526,314** | **6,117,253** | **6,409,061** | **7,505,292** | **3,664,349** | **3,840,943** | **5,021,022** | **2,452,904** | **2,568,118** |
| | | | | | | | | | |
| **Central Province** | **1,245,089** | **613,372** | **631,717** | **929,042** | **460,146** | **468,896** | **316,047** | **153,226** | **162,821** |
| Chibombo | 290,556 | 144,038 | 146,518 | 277,781 | 137,906 | 139,875 | 12,775 | 6,132 | 6,643 |
| Kabwe | 195,979 | 94,835 | 101,144 | - | - | - | 195,979 | 94,835 | 101,144 |
| Kapiri Mposhi | 240,638 | 118,811 | 121,827 | 197,992 | 98,234 | 99,758 | 42,646 | 20,577 | 22,069 |
| Mkushi | 148,814 | 74,292 | 74,522 | 130,066 | 65,073 | 64,993 | 18,748 | 9,219 | 9,529 |
| Mumbwa | 210,847 | 103,900 | 106,947 | 181,489 | 89,466 | 92,023 | 29,358 | 14,434 | 14,924 |
| Serenje | 158,255 | 77,496 | 80,759 | 141,714 | 69,467 | 72,247 | 16,541 | 8,029 | 8,512 |
| | | | | | | | | | |
| **Copperbelt Province** | **1,920,611** | **950,775** | **969,836** | **361,652** | **181,734** | **179,918** | **1,558,959** | **769,041** | **789,918** |
| Chililabombwe | 89,120 | 45,232 | 43,888 | 13,469 | 6,864 | 6,605 | 75,651 | 38,368 | 37,283 |
| Chingola | 209,897 | 104,302 | 105,595 | 29,850 | 15,005 | 14,845 | 180,047 | 89,297 | 90,750 |
| Kalulushi | 97,225 | 48,332 | 48,893 | 23,331 | 11,989 | 11,342 | 73,894 | 36,343 | 37,551 |
| Kitwe | 506,045 | 249,582 | 256,463 | 15,298 | 7,855 | 7,443 | 490,747 | 241,727 | 249,020 |
| Luanshya | 151,076 | 74,366 | 76,710 | 21,766 | 11,202 | 10,564 | 129,310 | 63,164 | 66,146 |
| Lufwanyama | 75,030 | 37,203 | 37,827 | 73,221 | 36,321 | 36,900 | 1,809 | 882 | 927 |
| Masaiti | 100,551 | 50,163 | 50,388 | 98,557 | 49,175 | 49,382 | 1,994 | 988 | 1,006 |
| Mpongwe | 89,950 | 44,891 | 45,059 | 75,407 | 37,840 | 37,567 | 14,543 | 7,051 | 7,492 |
| Mufulira | 158,371 | 78,753 | 79,618 | 10,753 | 5,483 | 5,270 | 147,618 | 73,270 | 74,348 |
| Ndola | 443,346 | 217,951 | 225,395 | - | - | - | 443,346 | 217,951 | 225,395 |
| | | | | | | | | | |
| **Eastern Province** | **1,525,123** | **744,354** | **780,769** | **1,333,443** | **651,737** | **681,706** | **191,680** | **92,617** | **99,063** |
| Chadiza | 102,341 | 50,310 | 52,031 | 99,638 | 48,967 | 50,671 | 2,703 | 1,343 | 1,360 |
| Chipata | 436,894 | 213,873 | 223,021 | 324,509 | 159,500 | 165,009 | 112,385 | 54,373 | 58,012 |
| Katete | 234,585 | 114,416 | 120,169 | 214,218 | 104,613 | 109,605 | 20,367 | 9,803 | 10,564 |
| Lundazi | 308,420 | 149,376 | 159,044 | 293,406 | 142,227 | 151,179 | 15,014 | 7,149 | 7,865 |
| Mambwe | 64,672 | 31,643 | 33,029 | 59,076 | 28,903 | 30,173 | 5,596 | 2,740 | 2,856 |
| Nyimba | 81,025 | 39,632 | 41,393 | 73,895 | 36,206 | 37,689 | 7,130 | 3,426 | 3,704 |
| Petauke | 297,186 | 145,104 | 152,082 | 268,701 | 131,321 | 137,380 | 28,485 | 13,783 | 14,702 |
| | | | | | | | | | |
| **Luapula Province** | **938,391** | **455,046** | **483,345** | **756,351** | **367,462** | **388,889** | **182,040** | **87,584** | **94,456** |
| Chienge | 107,997 | 52,226 | 55,771 | 104,541 | 50,599 | 53,942 | 3,456 | 1,627 | 1,829 |
| Kawambwa | 128,814 | 62,791 | 66,023 | 98,920 | 48,406 | 50,514 | 29,894 | 14,385 | 15,509 |
| Mansa | 212,028 | 102,839 | 109,189 | 139,940 | 68,134 | 71,806 | 72,088 | 34,705 | 37,383 |
| Milenge | 41,097 | 19,908 | 21,189 | 38,094 | 18,395 | 19,699 | 3,003 | 1,513 | 1,490 |
| Mwense | 115,642 | 56,225 | 59,417 | 94,885 | 46,264 | 48,621 | 20,757 | 9,961 | 10,796 |
| Nchelenge | 144,987 | 71,140 | 73,847 | 110,341 | 54,499 | 55,842 | 34,646 | 16,641 | 18,005 |
| Samfya | 187,826 | 89,917 | 97,909 | 169,630 | 81,165 | 88,465 | 18,196 | 8,752 | 9,444 |
| | | | | | | | | | |
| **Lusaka Province** | **2,138,907** | **1,049,215** | **1,089,692** | **319,732** | **160,216** | **159,516** | **1,819,175** | **888,999** | **930,176** |
| Chongwe | 181,816 | 90,829 | 90,987 | 169,973 | 85,091 | 84,882 | 11,843 | 5,738 | 6,105 |
| Kafue | 219,000 | 108,939 | 110,061 | 131,293 | 66,115 | 65,178 | 87,707 | 42,824 | 44,883 |
| Luangwa | 23,059 | 11,237 | 11,822 | 18,466 | 9,010 | 9,456 | 4,593 | 2,227 | 2,366 |
| Lusaka | 1,715,032 | 838,210 | 876,822 | - | - | - | 1,715,032 | 838,210 | 876,822 |

## Table A1: Population (De facto) by Province, District and Sex, Rural/Urban, Zambia 2010

| Province and District | Total | | | Rural | | | Urban | | |
|---|---|---|---|---|---|---|---|---|---|
| | Total | Male | Female | Total | Male | Female | Total | Male | Female |
| **Muchinga Province** | **677,507** | **328,980** | **348,527** | **562,025** | **272,884** | **289,141** | **115,482** | **56,096** | **59,386** |
| Chama | 99,434 | 47,951 | 51,483 | 92,620 | 44,546 | 48,074 | 6,814 | 3,405 | 3,409 |
| Chinsali | 138,659 | 67,671 | 70,988 | 124,244 | 60,828 | 63,416 | 14,415 | 6,843 | 7,572 |
| Isoka | 68,238 | 33,013 | 35,225 | 52,068 | 25,288 | 26,780 | 16,170 | 7,725 | 8,445 |
| Mafinga | 62,748 | 30,117 | 32,631 | 62,748 | 30,117 | 32,631 | - | - | - |
| Mpika | 191,329 | 93,064 | 98,265 | 154,199 | 75,055 | 79,144 | 37,130 | 18,009 | 19,121 |
| Nakonde | 117,099 | 57,164 | 59,935 | 76,146 | 37,050 | 39,096 | 40,953 | 20,114 | 20,839 |
| | | | | | | | | | |
| **Northern Province** | **1,044,955** | **508,226** | **536,729** | **852,963** | **415,508** | **437,455** | **191,992** | **92,718** | **99,274** |
| Chilubi | 74,884 | 35,342 | 39,542 | 71,149 | 33,654 | 37,495 | 3,735 | 1,688 | 2,047 |
| Kaputa | 111,613 | 53,812 | 57,801 | 102,688 | 49,508 | 53,180 | 8,925 | 4,304 | 4,621 |
| Kasama | 222,383 | 108,880 | 113,503 | 125,492 | 62,001 | 63,491 | 96,891 | 46,879 | 50,012 |
| Luwingu | 114,508 | 55,674 | 58,834 | 104,373 | 50,727 | 53,646 | 10,135 | 4,947 | 5,188 |
| Mbala | 192,636 | 94,101 | 98,535 | 169,811 | 83,053 | 86,758 | 22,825 | 11,048 | 11,777 |
| Mporokoso | 96,061 | 47,441 | 48,620 | 81,098 | 40,179 | 40,919 | 14,963 | 7,262 | 7,701 |
| Mpulungu | 91,316 | 44,189 | 47,127 | 63,961 | 31,034 | 32,927 | 27,355 | 13,155 | 14,200 |
| Mungwi | 141,554 | 68,787 | 72,767 | 134,391 | 65,352 | 69,039 | 7,163 | 3,435 | 3,728 |
| | | | | | | | | | |
| **North-Western Province** | **681,698** | **332,376** | **349,322** | **525,156** | **256,167** | **268,989** | **156,542** | **76,209** | **80,333** |
| Chavuma | 33,837 | 16,245 | 17,592 | 29,205 | 14,010 | 15,195 | 4,632 | 2,235 | 2,397 |
| Ikelenge | 31,995 | 15,605 | 16,390 | 31,995 | 15,605 | 16,390 | - | - | - |
| Kabompo | 86,497 | 42,032 | 44,465 | 65,211 | 31,837 | 33,374 | 21,286 | 10,195 | 11,091 |
| Kasempa | 63,520 | 30,802 | 32,718 | 59,644 | 28,957 | 30,687 | 3,876 | 1,845 | 2,031 |
| Mufumbwe | 53,683 | 26,185 | 27,498 | 44,745 | 21,886 | 22,859 | 8,938 | 4,299 | 4,639 |
| Mwinilunga | 98,784 | 48,004 | 50,780 | 83,403 | 40,638 | 42,765 | 15,381 | 7,366 | 8,015 |
| Solwezi | 237,233 | 116,535 | 120,698 | 144,527 | 70,898 | 73,629 | 92,706 | 45,637 | 47,069 |
| Zambezi | 76,149 | 36,968 | 39,181 | 66,426 | 32,336 | 34,090 | 9,723 | 4,632 | 5,091 |
| | | | | | | | | | |
| **Southern Province** | **1,517,088** | **738,996** | **778,092** | **1,141,540** | **556,592** | **584,948** | **375,548** | **182,404** | **193,144** |
| Choma | 238,348 | 115,528 | 122,820 | 181,630 | 88,293 | 93,337 | 56,718 | 27,235 | 29,483 |
| Gwembe | 50,136 | 24,238 | 25,898 | 47,577 | 23,050 | 24,527 | 2,559 | 1,188 | 1,371 |
| Itezhi-tezhi | 63,050 | 30,936 | 32,114 | 50,221 | 24,794 | 25,427 | 12,829 | 6,142 | 6,687 |
| Kalomo | 246,207 | 118,959 | 127,248 | 229,432 | 110,982 | 118,450 | 16,775 | 7,977 | 8,798 |
| Kazungula | 100,881 | 49,830 | 51,051 | 98,390 | 48,669 | 49,721 | 2,491 | 1,161 | 1,330 |
| Livingstone | 133,881 | 65,489 | 68,392 | 4,805 | 2,490 | 2,315 | 129,076 | 62,999 | 66,077 |
| Mazabuka | 221,893 | 109,876 | 112,017 | 149,588 | 74,059 | 75,529 | 72,305 | 35,817 | 36,488 |
| Monze | 183,817 | 89,502 | 94,315 | 143,832 | 70,122 | 73,710 | 39,985 | 19,380 | 20,605 |
| Namwala | 94,818 | 45,726 | 49,092 | 89,996 | 43,469 | 46,527 | 4,822 | 2,257 | 2,565 |
| Siavonga | 85,811 | 41,659 | 44,152 | 61,452 | 29,928 | 31,524 | 24,359 | 11,731 | 12,628 |
| Sinazongwe | 98,246 | 47,253 | 50,993 | 84,617 | 40,736 | 43,881 | 13,629 | 6,517 | 7,112 |
| | | | | | | | | | |
| **Western Province** | **836,945** | **395,913** | **441,032** | **723,388** | **341,903** | **381,485** | **113,557** | **54,010** | **59,547** |
| Kalabo | 120,308 | 55,017 | 65,291 | 117,131 | 53,523 | 63,608 | 3,177 | 1,494 | 1,683 |
| Kaoma | 172,927 | 82,629 | 90,298 | 154,208 | 73,704 | 80,504 | 18,719 | 8,925 | 9,794 |
| Lukulu | 81,568 | 38,900 | 42,668 | 71,098 | 33,908 | 37,190 | 10,470 | 4,992 | 5,478 |
| Mongu | 168,743 | 79,807 | 88,936 | 119,289 | 56,341 | 62,948 | 49,454 | 23,466 | 25,988 |
| Senanga | 117,359 | 55,372 | 61,987 | 103,939 | 48,982 | 54,957 | 13,420 | 6,390 | 7,030 |
| Sesheke | 91,970 | 44,657 | 47,313 | 75,694 | 36,907 | 38,787 | 16,276 | 7,750 | 8,526 |
| Shang'ombo | 84,070 | 39,531 | 44,539 | 82,029 | 38,538 | 43,491 | 2,041 | 993 | 1,048 |

## Table A1: Population (De Jure) by Province, District and Sex, Rural/Urban, Zambia 2010

| Province and District | Total | | | Rural | | | Urban | | |
|---|---|---|---|---|---|---|---|---|---|
| | Total | Male | Female | Total | Male | Female | Total | Male | Female |
| **Zambia Total** | **13,092,666** | **6,454,647** | **6,638,019** | **7,919,216** | **3,906,636** | **4,012,580** | **5,173,450** | **2,548,011** | **2,625,439** |
| | | | | | | | | | |
| **Central Province** | **1,307,111** | **648,465** | **658,646** | **978,574** | **487,713** | **490,861** | **328,537** | **160,752** | **167,785** |
| Chibombo | 303,519 | 151,155 | 152,364 | 290,115 | 144,652 | 145,463 | 13,404 | 6,503 | 6,901 |
| Kabwe | 202,360 | 98,781 | 103,579 | - | - | - | 202,360 | 98,781 | 103,579 |
| Kapiri Mposhi | 253,786 | 126,154 | 127,632 | 209,003 | 104,349 | 104,654 | 44,783 | 21,805 | 22,978 |
| Mkushi | 154,534 | 77,536 | 76,998 | 135,338 | 68,053 | 67,285 | 19,196 | 9,483 | 9,713 |
| Mumbwa | 226,171 | 112,469 | 113,702 | 195,131 | 97,007 | 98,124 | 31,040 | 15,462 | 15,578 |
| Serenje | 166,741 | 82,370 | 84,371 | 148,987 | 73,652 | 75,335 | 17,754 | 8,718 | 9,036 |
| | | | | | | | | | |
| **Copperbelt Province** | **1,972,317** | **981,887** | **990,430** | **376,861** | **190,178** | **186,683** | **1,595,456** | **791,709** | **803,747** |
| Chililabombwe | 91,833 | 46,792 | 45,041 | 14,015 | 7,154 | 6,861 | 77,818 | 39,638 | 38,180 |
| Chingola | 216,626 | 108,464 | 108,162 | 31,380 | 15,840 | 15,540 | 185,246 | 92,624 | 92,622 |
| Kalulushi | 100,381 | 50,164 | 50,217 | 24,366 | 12,552 | 11,814 | 76,015 | 37,612 | 38,403 |
| Kitwe | 517,543 | 256,740 | 260,803 | 16,183 | 8,331 | 7,852 | 501,360 | 248,409 | 252,951 |
| Luanshya | 156,059 | 77,368 | 78,691 | 22,691 | 11,711 | 10,980 | 133,368 | 65,657 | 67,711 |
| Lufwanyama | 78,503 | 39,182 | 39,321 | 76,482 | 38,198 | 38,284 | 2,021 | 984 | 1,037 |
| Masaiti | 103,857 | 52,017 | 51,840 | 101,763 | 50,977 | 50,786 | 2,094 | 1,040 | 1,054 |
| Mpongwe | 93,380 | 46,785 | 46,595 | 78,401 | 39,482 | 38,919 | 14,979 | 7,303 | 7,676 |
| Mufulira | 162,889 | 81,355 | 81,534 | 11,580 | 5,933 | 5,647 | 151,309 | 75,422 | 75,887 |
| Ndola | 451,246 | 223,020 | 228,226 | - | - | - | 451,246 | 223,020 | 228,226 |
| | | | | | | | | | |
| **Eastern Province** | **1,592,661** | **784,680** | **807,981** | **1,392,338** | **686,577** | **705,761** | **200,323** | **98,103** | **102,220** |
| Chadiza | 107,327 | 53,404 | 53,923 | 104,445 | 51,950 | 52,495 | 2,882 | 1,454 | 1,428 |
| Chipata | 455,783 | 224,934 | 230,849 | 339,156 | 167,781 | 171,375 | 116,627 | 57,153 | 59,474 |
| Katete | 243,849 | 119,995 | 123,854 | 222,390 | 109,546 | 112,844 | 21,459 | 10,449 | 11,010 |
| Lundazi | 323,870 | 158,379 | 165,491 | 307,968 | 150,691 | 157,277 | 15,902 | 7,688 | 8,214 |
| Mambwe | 68,918 | 34,253 | 34,665 | 62,859 | 31,227 | 31,632 | 6,059 | 3,026 | 3,033 |
| Nyimba | 85,025 | 41,992 | 43,033 | 77,359 | 38,254 | 39,105 | 7,666 | 3,738 | 3,928 |
| Petauke | 307,889 | 151,723 | 156,166 | 278,161 | 137,128 | 141,033 | 29,728 | 14,595 | 15,133 |
| | | | | | | | | | |
| **Luapula Province** | **991,927** | **488,589** | **503,338** | **797,407** | **393,615** | **403,792** | **194,520** | **94,974** | **99,546** |
| Chienge | 114,225 | 56,542 | 57,683 | 110,602 | 54,782 | 55,820 | 3,623 | 1,760 | 1,863 |
| Kawambwa | 134,414 | 66,091 | 68,323 | 102,727 | 50,682 | 52,045 | 31,687 | 15,409 | 16,278 |
| Mansa | 228,392 | 112,336 | 116,056 | 150,239 | 74,188 | 76,051 | 78,153 | 38,148 | 40,005 |
| Milenge | 43,337 | 21,338 | 21,999 | 40,218 | 19,742 | 20,476 | 3,119 | 1,596 | 1,523 |
| Mwense | 119,841 | 58,744 | 61,097 | 98,704 | 48,552 | 50,152 | 21,137 | 10,192 | 10,945 |
| Nchelenge | 152,807 | 76,124 | 76,683 | 115,913 | 58,060 | 57,853 | 36,894 | 18,064 | 18,830 |
| Samfya | 198,911 | 97,414 | 101,497 | 179,004 | 87,609 | 91,395 | 19,907 | 9,805 | 10,102 |
| | | | | | | | | | |
| **Lusaka Province** | **2,191,225** | **1,082,998** | **1,108,227** | **336,318** | **169,604** | **166,714** | **1,854,907** | **913,394** | **941,513** |
| Chongwe | 192,303 | 96,685 | 95,618 | 180,143 | 90,744 | 89,399 | 12,160 | 5,941 | 6,219 |
| Kafue | 227,466 | 113,910 | 113,556 | 136,668 | 69,233 | 67,435 | 90,798 | 44,677 | 46,121 |
| Luangwa | 24,304 | 11,979 | 12,325 | 19,507 | 9,627 | 9,880 | 4,797 | 2,352 | 2,445 |
| Lusaka | 1,747,152 | 860,424 | 886,728 | - | - | - | 1,747,152 | 860,424 | 886,728 |

## Table A1: Population (De Jure) by Province, District and Sex, Rural/Urban, Zambia 2010

| Province and District | Total | | | Rural | | | Urban | | |
|---|---|---|---|---|---|---|---|---|---|
| | Total | Male | Female | Total | Male | Female | Total | Male | Female |
| **Muchinga Province** | **711,657** | **349,872** | **361,785** | **590,575** | **290,490** | **300,085** | **121,082** | **59,382** | **61,700** |
| Chama | 103,894 | 50,856 | 53,038 | 96,865 | 47,302 | 49,563 | 7,029 | 3,554 | 3,475 |
| Chinsali | 146,518 | 72,526 | 73,992 | 131,320 | 65,180 | 66,140 | 15,198 | 7,346 | 7,852 |
| Isoka | 72,189 | 35,314 | 36,875 | 54,894 | 26,920 | 27,974 | 17,295 | 8,394 | 8,901 |
| Mafinga | 65,969 | 32,035 | 33,934 | 65,969 | 32,035 | 33,934 | - | - | - |
| Mpika | 203,379 | 100,267 | 103,112 | 163,655 | 80,853 | 82,802 | 39,724 | 19,414 | 20,310 |
| Nakonde | 119,708 | 58,874 | 60,834 | 77,872 | 38,200 | 39,672 | 41,836 | 20,674 | 21,162 |
| | | | | | | | | | |
| **Northern Province** | **1,105,824** | **546,851** | **558,973** | **903,208** | **447,755** | **455,453** | **202,616** | **99,096** | **103,520** |
| Chilubi | 81,248 | 39,792 | 41,456 | 76,953 | 37,696 | 39,257 | 4,295 | 2,096 | 2,199 |
| Kaputa | 119,514 | 59,312 | 60,202 | 110,304 | 54,804 | 55,500 | 9,210 | 4,508 | 4,702 |
| Kasama | 231,824 | 114,208 | 117,616 | 129,979 | 64,526 | 65,453 | 101,845 | 49,682 | 52,163 |
| Luwingu | 122,136 | 60,294 | 61,842 | 111,370 | 54,989 | 56,381 | 10,766 | 5,305 | 5,461 |
| Mbala | 203,129 | 100,703 | 102,426 | 179,119 | 88,935 | 90,184 | 24,010 | 11,768 | 12,242 |
| Mporokoso | 98,842 | 49,161 | 49,681 | 83,286 | 41,566 | 41,720 | 15,556 | 7,595 | 7,961 |
| Mpulungu | 98,073 | 48,651 | 49,422 | 68,970 | 34,362 | 34,608 | 29,103 | 14,289 | 14,814 |
| Mungwi | 151,058 | 74,730 | 76,328 | 143,227 | 70,877 | 72,350 | 7,831 | 3,853 | 3,978 |
| | | | | | | | | | |
| **North-Western Province** | **727,044** | **358,141** | **368,903** | **563,061** | **277,503** | **285,558** | **163,983** | **80,638** | **83,345** |
| Chavuma | 35,041 | 17,003 | 18,038 | 30,389 | 14,749 | 15,640 | 4,652 | 2,254 | 2,398 |
| Ikelenge | 32,919 | 16,151 | 16,768 | 32,919 | 16,151 | 16,768 | - | - | - |
| Kabompo | 92,321 | 45,224 | 47,097 | 69,696 | 34,246 | 35,450 | 22,625 | 10,978 | 11,647 |
| Kasempa | 69,608 | 34,357 | 35,251 | 65,206 | 32,233 | 32,973 | 4,402 | 2,124 | 2,278 |
| Mufumbwe | 58,062 | 28,612 | 29,450 | 48,486 | 23,945 | 24,541 | 9,576 | 4,667 | 4,909 |
| Mwinilunga | 104,317 | 51,267 | 53,050 | 88,613 | 43,712 | 44,901 | 15,704 | 7,555 | 8,149 |
| Solwezi | 254,470 | 126,249 | 128,221 | 157,745 | 78,161 | 79,584 | 96,725 | 48,088 | 48,637 |
| Zambezi | 80,306 | 39,278 | 41,028 | 70,007 | 34,306 | 35,701 | 10,299 | 4,972 | 5,327 |
| | | | | | | | | | |
| **Southern Province** | **1,589,926** | **779,659** | **810,267** | **1,197,751** | **587,448** | **610,303** | **392,175** | **192,211** | **199,964** |
| Choma | 247,860 | 120,689 | 127,171 | 189,035 | 92,145 | 96,890 | 58,825 | 28,544 | 30,281 |
| Gwembe | 53,117 | 25,908 | 27,209 | 50,355 | 24,606 | 25,749 | 2,762 | 1,302 | 1,460 |
| Itezhi-tezhi | 68,599 | 34,017 | 34,582 | 54,741 | 27,257 | 27,484 | 13,858 | 6,760 | 7,098 |
| Kalomo | 258,570 | 125,767 | 132,803 | 240,791 | 117,177 | 123,614 | 17,779 | 8,590 | 9,189 |
| Kazungula | 104,731 | 51,994 | 52,737 | 101,638 | 50,490 | 51,148 | 3,093 | 1,504 | 1,589 |
| Livingstone | 139,509 | 68,763 | 70,746 | 5,160 | 2,679 | 2,481 | 134,349 | 66,084 | 68,265 |
| Mazabuka | 230,972 | 114,783 | 116,189 | 156,491 | 77,685 | 78,806 | 74,481 | 37,098 | 37,383 |
| Monze | 191,872 | 93,958 | 97,914 | 149,982 | 73,485 | 76,497 | 41,890 | 20,473 | 21,417 |
| Namwala | 102,866 | 50,127 | 52,739 | 97,467 | 47,566 | 49,901 | 5,399 | 2,561 | 2,838 |
| Siavonga | 90,213 | 44,444 | 45,769 | 64,796 | 32,042 | 32,754 | 25,417 | 12,402 | 13,015 |
| Sinazongwe | 101,617 | 49,209 | 52,408 | 87,295 | 42,316 | 44,979 | 14,322 | 6,893 | 7,429 |
| | | | | | | | | | |
| **Western Province** | **902,974** | **433,505** | **469,469** | **783,123** | **375,753** | **407,370** | **119,851** | **57,752** | **62,099** |
| Kalabo | 128,904 | 60,114 | 68,790 | 125,602 | 58,543 | 67,059 | 3,302 | 1,571 | 1,731 |
| Kaoma | 189,290 | 91,872 | 97,418 | 169,439 | 82,214 | 87,225 | 19,851 | 9,658 | 10,193 |
| Lukulu | 86,002 | 41,390 | 44,612 | 75,427 | 36,319 | 39,108 | 10,575 | 5,071 | 5,504 |
| Mongu | 179,585 | 85,964 | 93,621 | 127,261 | 60,860 | 66,401 | 52,324 | 25,104 | 27,220 |
| Senanga | 126,506 | 60,428 | 66,078 | 112,404 | 53,652 | 58,752 | 14,102 | 6,776 | 7,326 |
| Sesheke | 99,384 | 48,913 | 50,471 | 81,946 | 40,448 | 41,498 | 17,438 | 8,465 | 8,973 |
| Shang'ombo | 93,303 | 44,824 | 48,479 | 91,044 | 43,717 | 47,327 | 2,259 | 1,107 | 1,152 |

## Table A2: Population Distribution by Urban Centres (15,000 and above Population) by Province, District and Sex, Zambia 2010

| Province/District | Urban Centre | Total | Male | Female |
|---|---|---|---|---|
| **Central Province** | | | | |
| Kabwe | Kabwe | 202,360 | 98,781 | 103,579 |
| Kapiri Mposhi | Kapiri Mposhi | 44,783 | 21,805 | 22,978 |
| Mkushi | Mkushi | 19,196 | 9,483 | 9,713 |
| Mumbwa | Mumbwa | 20,390 | 9,953 | 10,437 |
| Serenje | Serenje | 17,754 | 8,718 | 9,036 |
| **Copperbelt Province** | | | | |
| Chililabombwe District | Chililabombwe | 77,818 | 39,638 | 38,180 |
| Chingola District | Chingola | 185,246 | 92,624 | 92,622 |
| Kalulushi District | Kalulushi | 51,863 | 25,509 | 26,354 |
| | Chambishi | 24,152 | 12,103 | 12,049 |
| Kitwe District | Kitwe | 501,360 | 248,409 | 252,951 |
| Luanshya District | Luanshya | 130,076 | 64,036 | 66,040 |
| Mufulira District | Mufulira | 151,309 | 75,422 | 75,887 |
| Ndola District | Ndola | 451,246 | 223,020 | 228,226 |
| **Eastern Province** | | | | |
| Chipata District | Chipata | 116,627 | 57,153 | 59,474 |
| Katete District | Katete | 21,459 | 10,449 | 11,010 |
| Lundazi District | Lundazi | 15,902 | 7,688 | 8,214 |
| Petauke District | Petauke | 29,728 | 14,595 | 15,133 |
| **Luapula Province** | | | | |
| Kawambwa District | Kawambwa | 15,953 | 7,871 | 8,082 |
| | Mwansabombwe | 15,734 | 7,538 | 8,196 |
| Mansa District | Mansa | 78,153 | 38,148 | 40,005 |
| Mwense District | Mwense | 21,137 | 10,192 | 10,945 |
| Nchelenge District | Nchelenge/Kashikishi | 36,894 | 18,064 | 18,830 |
| Samfya District | Samfya | 19,907 | 9,805 | 10,102 |
| **Lusaka Province** | | | | |
| Kafue District | Kafue | 72,166 | 35,411 | 36,755 |
| | Chilanga | 18,632 | 9,266 | 9,366 |
| Lusaka District | Lusaka | 1,747,152 | 860,424 | 886,728 |
| **Muchinga Province** | | | | |
| Chinsali District | Chinsali | 15,198 | 7,346 | 7,852 |
| Isoka District | Isoka | 17,295 | 8,394 | 8,901 |
| Mpika District | Mpika | 39,724 | 19,414 | 20,310 |
| Nakonde District | Nakonde | 41,836 | 20,674 | 21,162 |
| **North Western Province** | | | | |
| Solwezi District | Solwezi | 90,856 | 45,126 | 45,730 |
| **Northern Province** | | | | |
| Kasama District | Kasama | 101,845 | 49,682 | 52,163 |
| Mbala District | Mbala | 24,010 | 11,768 | 12,242 |
| Mporokoso District | Mporokoso | 15,556 | 7,595 | 7,961 |
| Mpulungu District | Mpulungu | 29,103 | 14,289 | 14,814 |
| **Southern Province** | | | | |
| Choma District | Choma | 51,842 | 25,254 | 26,588 |
| Livingstone District | Livingstone | 134,349 | 66,084 | 68,265 |
| Mazabuka District | Mazabuka | 71,700 | 35,764 | 35,936 |
| Kalomo District | Kalomo | 15,394 | 7,438 | 7,956 |
| Monze District | Monze | 39,752 | 19,445 | 20,307 |
| **Western Province** | | | | |
| Mongu District | Mongu | 52,324 | 25,104 | 27,220 |
| Kaoma District | Kaoma | 19,851 | 9,658 | 10,193 |

## Table A3: Population (De Jure), Households, Average Household Size, Size of Household and Sex of Household Head by Province, Zambia 2010

| Province and Sex of Household Head | Population | Number of Households | Average Household Size | Size of Household | | | | | | | | | |
|---|---|---|---|---|---|---|---|---|---|---|---|---|---|
| | | | | 1 | 2 | 3 | 4 | 5 | 6 | 7 | 8 | 9 | 10+ |
| **Zambia Total** | **13,092,666** | **2,513,768** | **5.2** | **163,630** | **225,661** | **320,669** | **371,193** | **368,344** | **324,706** | **260,447** | **280,640** | **62,281** | **136,197** |
| Male | 10,581,257 | 1,947,501 | 5.4 | 99,105 | 149,814 | 231,063 | 281,679 | 290,934 | 265,259 | 219,022 | 240,651 | 53,698 | 116,276 |
| Female | 2,511,409 | 566,267 | 4.4 | 64,525 | 75,847 | 89,606 | 89,514 | 77,410 | 59,447 | 41,425 | 39,989 | 8,583 | 19,921 |
| | | | | | | | | | | | | | |
| **Central** | **1,307,111** | **235,560** | **5.5** | **14,492** | **18,628** | **26,280** | **31,481** | **33,046** | **30,512** | **25,517** | **31,430** | **6,773** | **17,401** |
| Male | 1,067,003 | 185,348 | 5.8 | 9,865 | 12,536 | 18,920 | 23,999 | 26,087 | 24,929 | 21,286 | 26,800 | 5,839 | 15,087 |
| Female | 240,108 | 50,212 | 4.8 | 4,627 | 6,092 | 7,360 | 7,482 | 6,959 | 5,583 | 4,231 | 4,630 | 934 | 2,314 |
| | | | | | | | | | | | | | |
| **Copperbelt** | **1,972,317** | **371,125** | **5.3** | **25,339** | **32,792** | **44,542** | **52,419** | **52,700** | **47,755** | **39,074** | **41,096** | **11,075** | **24,333** |
| Male | 1,609,476 | 295,018 | 5.5 | 18,045 | 23,365 | 33,360 | 40,889 | 42,418 | 39,310 | 32,912 | 34,849 | 9,511 | 20,359 |
| Female | 362,841 | 76,107 | 4.8 | 7,294 | 9,427 | 11,182 | 11,530 | 10,282 | 8,445 | 6,162 | 6,247 | 1,564 | 3,974 |
| | | | | | | | | | | | | | |
| **Eastern** | **1,592,661** | **305,198** | **5.2** | **16,564** | **25,079** | **39,233** | **46,776** | **47,031** | **41,779** | **33,238** | **34,464** | **6,913** | **14,121** |
| Male | 1,294,019 | 236,449 | 5.5 | 8,048 | 16,025 | 28,556 | 35,999 | 37,298 | 34,364 | 28,150 | 29,837 | 5,985 | 12,187 |
| Female | 298,642 | 68,749 | 4.3 | 8,516 | 9,054 | 10,677 | 10,777 | 9,733 | 7,415 | 5,088 | 4,627 | 928 | 1,934 |
| | | | | | | | | | | | | | |
| **Luapula** | **991,927** | **194,962** | **5.1** | **12,334** | **18,648** | **25,560** | **28,543** | **28,638** | **25,420** | **20,952** | **23,176** | **4,078** | **7,613** |
| Male | 818,297 | 151,913 | 5.4 | 6,020 | 12,248 | 18,398 | 21,692 | 22,888 | 21,273 | 18,186 | 20,749 | 3,659 | 6,800 |
| Female | 173,630 | 43,049 | 4.0 | 6,314 | 6,400 | 7,162 | 6,851 | 5,750 | 4,147 | 2,766 | 2,427 | 419 | 813 |
| | | | | | | | | | | | | | |
| **Lusaka** | **2,191,225** | **444,418** | **4.9** | **35,038** | **44,631** | **61,695** | **70,251** | **67,001** | **54,959** | **41,145** | **37,998** | **10,316** | **21,384** |
| Male | 1,779,768 | 353,797 | 5.0 | 25,359 | 32,740 | 47,179 | 55,810 | 54,678 | 45,843 | 34,543 | 31,723 | 8,607 | 17,315 |
| Female | 411,457 | 90,621 | 4.5 | 9,679 | 11,891 | 14,516 | 14,441 | 12,323 | 9,116 | 6,602 | 6,275 | 1,709 | 4,069 |
| | | | | | | | | | | | | | |
| **Muchinga** | **711,657** | **138,783** | **5.1** | **7,055** | **12,706** | **18,682** | **21,083** | **20,769** | **18,740** | **15,420** | **16,678** | **2,687** | **4,963** |
| Male | 588,000 | 108,853 | 5.4 | 3,064 | 8,440 | 13,783 | 16,269 | 16,678 | 15,531 | 13,367 | 14,874 | 2,440 | 4,407 |
| Female | 123,657 | 29,930 | 4.1 | 3,991 | 4,266 | 4,899 | 4,814 | 4,091 | 3,209 | 2,053 | 1,804 | 247 | 556 |
| | | | | | | | | | | | | | |
| **Northern** | **1,105,824** | **220,561** | **5.0** | **12,509** | **21,423** | **30,563** | **33,961** | **32,796** | **29,089** | **23,618** | **25,877** | **4,058** | **6,667** |
| Male | 927,914 | 175,037 | 5.3 | 5,625 | 14,319 | 22,916 | 26,449 | 26,759 | 24,683 | 20,888 | 23,593 | 3,745 | 6,060 |
| Female | 177,910 | 45,524 | 3.9 | 6,884 | 7,104 | 7,647 | 7,512 | 6,037 | 4,406 | 2,730 | 2,284 | 313 | 607 |
| | | | | | | | | | | | | | |
| **North-Western** | **727,044** | **130,803** | **5.6** | **8,528** | **10,375** | **13,947** | **17,253** | **17,962** | **16,736** | **14,436** | **17,151** | **4,272** | **10,143** |
| Male | 587,873 | 99,754 | 5.9 | 4,439 | 6,399 | 9,416 | 12,613 | 13,896 | 13,434 | 12,163 | 14,765 | 3,759 | 8,870 |
| Female | 139,171 | 31,049 | 4.5 | 4,089 | 3,976 | 4,531 | 4,640 | 4,066 | 3,302 | 2,273 | 2,386 | 513 | 1,273 |
| | | | | | | | | | | | | | |
| **Southern** | **1,589,926** | **292,179** | **5.4** | **18,414** | **23,343** | **35,770** | **42,063** | **42,090** | **37,458** | **29,927** | **33,253** | **8,478** | **21,383** |
| Male | 1,259,332 | 220,606 | 5.7 | 11,901 | 14,261 | 24,449 | 30,476 | 32,044 | 29,521 | 24,351 | 27,836 | 7,220 | 18,547 |
| Female | 330,594 | 71,573 | 4.6 | 6,513 | 9,082 | 11,321 | 11,587 | 10,046 | 7,937 | 5,576 | 5,417 | 1,258 | 2,836 |
| | | | | | | | | | | | | | |
| **Western** | **902,974** | **180,179** | **5.0** | **13,357** | **18,036** | **24,397** | **27,363** | **26,311** | **22,258** | **17,120** | **19,517** | **3,631** | **8,189** |
| Male | 649,575 | 120,726 | 5.4 | 6,739 | 9,481 | 14,086 | 17,483 | 18,188 | 16,371 | 13,176 | 15,625 | 2,933 | 6,644 |
| Female | 253,399 | 59,453 | 4.3 | 6,618 | 8,555 | 10,311 | 9,880 | 8,123 | 5,887 | 3,944 | 3,892 | 698 | 1,545 |

## Table A4: Population (De Jure) by Single Year Age and Sex by Province, Rural/Urban, Zambia 2010

| Single Year Ages | Zambia Total | | | Central | | | Copperbelt | | | Eastern | | | Luapula | | | Lusaka | | |
|---|---|---|---|---|---|---|---|---|---|---|---|---|---|---|---|---|---|---|
| | Total | Male | Female | Total | Male | Female | Total | Male | Female | Total | Male | Female | Total | Male | Female | Total | Male | Female |
| **Total** | **13,092,666** | **6,454,647** | **6,638,019** | **1,307,111** | **648,465** | **658,646** | **1,972,317** | **981,887** | **990,430** | **1,592,661** | **784,680** | **807,981** | **991,927** | **488,589** | **503,338** | **2,191,225** | **1,082,998** | **1,108,227** |
| 0 | 440,712 | 220,221 | 220,491 | 44,911 | 22,369 | 22,542 | 54,120 | 27,038 | 27,082 | 55,845 | 27,773 | 28,072 | 35,778 | 17,884 | 17,894 | 65,616 | 32,718 | 32,898 |
| 1 | 443,560 | 221,119 | 222,441 | 44,581 | 22,183 | 22,398 | 60,017 | 29,923 | 30,094 | 56,786 | 28,273 | 28,513 | 34,783 | 17,383 | 17,400 | 68,592 | 34,017 | 34,575 |
| 2 | 460,659 | 229,460 | 231,199 | 46,968 | 23,374 | 23,594 | 58,529 | 29,360 | 29,169 | 59,103 | 29,209 | 29,894 | 38,110 | 18,884 | 19,226 | 66,304 | 33,327 | 32,977 |
| 3 | 451,273 | 223,214 | 228,059 | 46,237 | 22,942 | 23,295 | 58,094 | 28,460 | 29,634 | 56,654 | 27,771 | 28,883 | 36,035 | 17,866 | 18,169 | 66,198 | 32,608 | 33,590 |
| 4 | 456,544 | 227,454 | 229,090 | 45,860 | 22,769 | 23,091 | 58,871 | 29,515 | 29,356 | 56,944 | 28,448 | 28,496 | 37,782 | 18,764 | 19,018 | 64,703 | 32,131 | 32,572 |
| 5 | 418,173 | 209,889 | 208,284 | 42,924 | 21,710 | 21,214 | 53,914 | 26,970 | 26,944 | 54,244 | 27,206 | 27,038 | 34,072 | 17,153 | 16,919 | 60,365 | 30,167 | 30,198 |
| 6 | 408,665 | 203,360 | 205,305 | 42,400 | 21,277 | 21,123 | 52,671 | 26,102 | 26,569 | 52,007 | 25,897 | 26,110 | 33,340 | 16,736 | 16,604 | 58,130 | 28,585 | 29,545 |
| 7 | 400,962 | 201,017 | 199,945 | 40,957 | 20,617 | 20,340 | 52,511 | 26,287 | 26,224 | 53,435 | 26,743 | 26,692 | 32,028 | 16,088 | 15,940 | 58,340 | 29,172 | 29,168 |
| 8 | 363,988 | 179,457 | 184,531 | 37,091 | 18,324 | 18,767 | 48,294 | 23,555 | 24,739 | 46,881 | 23,284 | 23,597 | 30,897 | 15,394 | 15,503 | 52,931 | 25,606 | 27,325 |
| 9 | 324,499 | 160,609 | 163,890 | 33,320 | 16,427 | 16,893 | 45,065 | 21,981 | 23,084 | 40,173 | 20,280 | 19,893 | 24,764 | 12,352 | 12,412 | 48,921 | 23,677 | 25,244 |
| 10 | 467,573 | 233,415 | 234,158 | 47,774 | 23,876 | 23,898 | 63,270 | 31,276 | 31,994 | 60,732 | 30,765 | 29,967 | 40,186 | 20,330 | 19,856 | 65,838 | 32,039 | 33,799 |
| 11 | 301,313 | 148,932 | 152,381 | 30,946 | 15,405 | 15,541 | 44,124 | 21,403 | 22,721 | 38,249 | 19,122 | 19,127 | 22,957 | 11,489 | 11,468 | 47,322 | 22,635 | 24,687 |
| 12 | 366,952 | 182,563 | 184,389 | 38,369 | 19,178 | 19,191 | 53,952 | 26,435 | 27,517 | 46,230 | 23,507 | 22,723 | 28,652 | 14,410 | 14,242 | 56,751 | 26,954 | 29,797 |
| 13 | 319,019 | 156,719 | 162,300 | 33,262 | 16,420 | 16,842 | 50,900 | 24,535 | 26,365 | 38,158 | 19,215 | 18,943 | 22,125 | 11,241 | 10,884 | 52,785 | 24,501 | 28,284 |
| 14 | 319,277 | 156,943 | 162,334 | 33,544 | 16,650 | 16,894 | 52,276 | 25,240 | 27,036 | 38,126 | 19,256 | 18,870 | 23,347 | 11,745 | 11,602 | 52,766 | 24,484 | 28,282 |
| 15 | 331,625 | 164,979 | 166,646 | 33,815 | 17,018 | 16,797 | 52,531 | 25,447 | 27,084 | 40,357 | 20,907 | 19,450 | 23,936 | 12,136 | 11,800 | 53,254 | 25,140 | 28,114 |
| 16 | 316,166 | 154,124 | 162,042 | 33,196 | 16,341 | 16,855 | 52,530 | 24,911 | 27,619 | 37,029 | 18,718 | 18,311 | 22,337 | 11,091 | 11,246 | 52,428 | 24,305 | 28,123 |
| 17 | 279,393 | 136,547 | 142,846 | 28,304 | 14,021 | 14,283 | 48,698 | 23,644 | 25,054 | 31,166 | 15,718 | 15,448 | 19,683 | 9,660 | 10,023 | 48,387 | 22,909 | 25,478 |
| 18 | 333,797 | 163,074 | 170,723 | 34,329 | 17,064 | 17,265 | 55,716 | 27,360 | 28,356 | 38,775 | 19,354 | 19,421 | 24,395 | 11,880 | 12,515 | 55,109 | 26,125 | 28,984 |
| 19 | 270,134 | 129,892 | 140,242 | 27,305 | 13,345 | 13,960 | 44,807 | 21,983 | 22,824 | 32,208 | 15,507 | 16,701 | 18,615 | 8,891 | 9,724 | 49,004 | 22,878 | 26,126 |
| 20 | 297,206 | 138,864 | 158,342 | 29,462 | 14,188 | 15,274 | 46,881 | 22,340 | 24,541 | 34,768 | 16,601 | 18,167 | 22,304 | 10,243 | 12,061 | 52,899 | 23,994 | 28,905 |
| 21 | 214,845 | 100,677 | 114,168 | 21,728 | 10,481 | 11,247 | 35,136 | 16,811 | 18,325 | 23,225 | 11,186 | 12,039 | 13,362 | 6,110 | 7,252 | 42,324 | 19,184 | 23,140 |
| 22 | 241,224 | 110,127 | 131,097 | 23,326 | 10,815 | 12,511 | 38,631 | 18,045 | 20,586 | 28,328 | 13,041 | 15,287 | 16,292 | 7,169 | 9,123 | 46,704 | 20,925 | 25,779 |
| 23 | 224,594 | 104,451 | 120,143 | 21,511 | 10,256 | 11,255 | 36,355 | 17,293 | 19,062 | 26,049 | 12,272 | 13,777 | 13,861 | 6,149 | 7,712 | 46,030 | 21,018 | 25,012 |
| 24 | 216,773 | 99,148 | 117,625 | 20,511 | 9,552 | 10,959 | 35,433 | 16,689 | 18,744 | 24,318 | 11,247 | 13,071 | 14,077 | 6,044 | 8,033 | 44,924 | 20,686 | 24,238 |
| 25 | 248,544 | 117,637 | 130,907 | 23,031 | 11,026 | 12,005 | 39,363 | 18,879 | 20,484 | 28,492 | 13,644 | 14,848 | 17,807 | 8,231 | 9,576 | 49,314 | 23,464 | 25,850 |
| 26 | 213,339 | 98,499 | 114,840 | 19,426 | 9,105 | 10,321 | 35,157 | 16,672 | 18,485 | 24,000 | 11,062 | 12,938 | 14,229 | 6,229 | 8,000 | 44,621 | 20,947 | 23,674 |
| 27 | 194,540 | 92,257 | 102,283 | 17,611 | 8,321 | 9,290 | 32,335 | 15,869 | 16,466 | 21,531 | 10,282 | 11,249 | 13,024 | 5,794 | 7,230 | 40,955 | 20,016 | 20,939 |
| 28 | 234,707 | 110,168 | 124,539 | 21,135 | 9,829 | 11,306 | 38,230 | 18,609 | 19,621 | 25,902 | 12,110 | 13,792 | 16,607 | 7,389 | 9,218 | 49,135 | 23,896 | 25,239 |
| 29 | 165,947 | 79,213 | 86,734 | 15,539 | 7,376 | 8,163 | 27,436 | 13,464 | 13,972 | 18,505 | 8,754 | 9,751 | 10,820 | 5,126 | 5,694 | 35,740 | 17,216 | 18,524 |
| 30 | 249,921 | 125,731 | 124,190 | 22,545 | 11,088 | 11,457 | 39,584 | 20,216 | 19,368 | 28,312 | 14,410 | 13,902 | 19,305 | 9,814 | 9,491 | 51,112 | 26,058 | 25,054 |
| 31 | 131,295 | 66,688 | 64,607 | 12,619 | 6,400 | 6,219 | 21,126 | 11,124 | 10,002 | 14,028 | 7,147 | 6,881 | 8,396 | 4,094 | 4,302 | 28,485 | 14,714 | 13,771 |
| 32 | 187,162 | 94,514 | 92,648 | 17,411 | 8,703 | 8,708 | 31,473 | 16,430 | 15,043 | 20,523 | 10,163 | 10,360 | 12,976 | 6,412 | 6,564 | 41,119 | 21,568 | 19,551 |
| 33 | 129,877 | 65,422 | 64,455 | 12,137 | 6,055 | 6,082 | 21,196 | 11,107 | 10,089 | 15,014 | 7,557 | 7,457 | 8,710 | 4,284 | 4,426 | 27,739 | 14,202 | 13,537 |
| 34 | 142,053 | 72,872 | 69,181 | 13,560 | 6,917 | 6,643 | 23,646 | 12,414 | 11,232 | 15,049 | 7,574 | 7,475 | 9,413 | 4,652 | 4,761 | 30,851 | 16,621 | 14,230 |
| 35 | 183,055 | 98,632 | 84,423 | 16,919 | 9,064 | 7,855 | 29,374 | 16,225 | 13,149 | 20,234 | 10,670 | 9,564 | 13,763 | 7,291 | 6,472 | 37,611 | 21,400 | 16,211 |
| 36 | 141,677 | 72,674 | 69,003 | 13,647 | 7,018 | 6,629 | 22,647 | 11,903 | 10,744 | 16,220 | 8,153 | 8,067 | 10,085 | 4,929 | 5,156 | 29,447 | 16,074 | 13,373 |
| 37 | 114,806 | 59,351 | 55,455 | 11,046 | 5,699 | 5,347 | 18,639 | 9,814 | 8,825 | 13,261 | 6,742 | 6,519 | 7,962 | 4,027 | 3,935 | 23,797 | 13,313 | 10,484 |
| 38 | 147,688 | 77,038 | 70,650 | 14,082 | 7,284 | 6,798 | 23,649 | 12,576 | 11,073 | 17,580 | 8,894 | 8,686 | 10,736 | 5,541 | 5,195 | 29,422 | 16,525 | 12,897 |
| 39 | 95,695 | 49,402 | 46,293 | 9,276 | 4,814 | 4,462 | 15,784 | 8,138 | 7,646 | 11,074 | 5,697 | 5,377 | 6,870 | 3,474 | 3,396 | 19,481 | 10,748 | 8,733 |
| 40 | 149,363 | 81,698 | 67,665 | 13,876 | 7,671 | 6,205 | 24,034 | 13,323 | 10,711 | 17,370 | 9,284 | 8,086 | 12,252 | 6,723 | 5,529 | 28,270 | 16,594 | 11,676 |
| 41 | 77,491 | 40,726 | 36,765 | 7,594 | 4,029 | 3,565 | 13,140 | 7,181 | 5,959 | 8,667 | 4,355 | 4,312 | 5,658 | 2,937 | 2,721 | 14,335 | 8,245 | 6,090 |
| 42 | 116,976 | 62,565 | 54,411 | 11,402 | 6,128 | 5,274 | 19,015 | 10,391 | 8,624 | 13,732 | 7,178 | 6,554 | 9,175 | 4,828 | 4,347 | 21,075 | 12,181 | 8,894 |
| 43 | 66,624 | 34,522 | 32,102 | 6,515 | 3,420 | 3,095 | 11,661 | 6,071 | 5,590 | 7,438 | 3,717 | 3,721 | 5,059 | 2,669 | 2,390 | 12,409 | 6,837 | 5,572 |
| 44 | 62,784 | 30,904 | 31,880 | 6,648 | 3,244 | 3,404 | 9,999 | 5,163 | 4,836 | 7,296 | 3,504 | 3,792 | 4,911 | 2,392 | 2,519 | 10,565 | 5,649 | 4,916 |
| 45 | 93,919 | 48,713 | 45,206 | 8,959 | 4,581 | 4,378 | 15,652 | 8,192 | 7,460 | 11,002 | 5,684 | 5,318 | 8,062 | 4,235 | 3,827 | 15,885 | 8,798 | 7,087 |
| 46 | 94,567 | 47,588 | 46,979 | 9,260 | 4,721 | 4,539 | 16,389 | 8,426 | 7,963 | 11,092 | 5,382 | 5,710 | 7,067 | 3,515 | 3,552 | 16,277 | 8,764 | 7,513 |
| 47 | 59,148 | 29,727 | 29,421 | 5,829 | 2,912 | 2,917 | 10,154 | 5,206 | 4,948 | 7,107 | 3,556 | 3,551 | 4,359 | 2,203 | 2,156 | 9,479 | 5,044 | 4,435 |
| 48 | 76,478 | 37,613 | 38,865 | 7,649 | 3,682 | 3,967 | 12,275 | 5,872 | 6,403 | 9,365 | 4,747 | 4,618 | 6,544 | 3,359 | 3,185 | 11,668 | 6,060 | 5,608 |
| 49 | 52,052 | 25,406 | 26,646 | 5,125 | 2,553 | 2,572 | 8,668 | 4,330 | 4,338 | 6,708 | 3,134 | 3,574 | 3,875 | 1,893 | 1,982 | 8,057 | 4,096 | 3,961 |
| 50 | 83,330 | 41,111 | 42,219 | 7,824 | 3,811 | 4,013 | 13,975 | 6,945 | 7,030 | 10,641 | 5,265 | 5,376 | 7,257 | 3,684 | 3,573 | 12,408 | 6,409 | 5,999 |
| 51 | 43,946 | 21,049 | 22,897 | 4,666 | 2,238 | 2,428 | 7,510 | 3,661 | 3,849 | 5,006 | 2,409 | 2,597 | 3,476 | 1,664 | 1,812 | 6,657 | 3,333 | 3,324 |
| 52 | 64,582 | 31,300 | 33,282 | 6,562 | 3,176 | 3,386 | 11,303 | 5,510 | 5,793 | 7,310 | 3,476 | 3,834 | 5,490 | 2,785 | 2,705 | 9,696 | 4,845 | 4,851 |
| 53 | 45,890 | 22,565 | 23,325 | 4,730 | 2,306 | 2,424 | 8,192 | 4,197 | 3,995 | 5,454 | 2,536 | 2,918 | 3,482 | 1,731 | 1,751 | 7,211 | 3,691 | 3,520 |

## Table A4: Population (De Jure) by Single Year Age and Sex by Province, Rural/Urban, Zambia 2010

| Single Year Ages | Zambia Total | | | Central | | | Copperbelt | | | Eastern | | | Luapula | | | Lusaka | | |
|---|---|---|---|---|---|---|---|---|---|---|---|---|---|---|---|---|---|---|
| | Total | Male | Female | Total | Male | Female | Total | Male | Female | Total | Male | Female | Total | Male | Female | Total | Male | Female |
| 54 | 47,116 | 22,739 | 24,377 | 4,802 | 2,372 | 2,430 | 8,672 | 4,334 | 4,338 | 5,429 | 2,443 | 2,986 | 3,841 | 1,893 | 1,948 | 7,182 | 3,601 | 3,581 |
| 55 | 47,431 | 24,029 | 23,402 | 4,622 | 2,366 | 2,256 | 8,770 | 4,641 | 4,129 | 5,810 | 2,847 | 2,963 | 3,761 | 1,902 | 1,859 | 7,373 | 3,901 | 3,472 |
| 56 | 45,806 | 23,067 | 22,739 | 4,612 | 2,321 | 2,291 | 8,532 | 4,609 | 3,923 | 5,267 | 2,577 | 2,690 | 3,737 | 1,824 | 1,913 | 6,839 | 3,649 | 3,190 |
| 57 | 32,839 | 16,459 | 16,380 | 3,495 | 1,808 | 1,687 | 5,991 | 3,241 | 2,750 | 4,071 | 1,961 | 2,110 | 2,616 | 1,234 | 1,382 | 4,786 | 2,497 | 2,289 |
| 58 | 42,137 | 20,568 | 21,569 | 4,254 | 2,085 | 2,169 | 7,454 | 3,863 | 3,591 | 5,145 | 2,440 | 2,705 | 3,746 | 1,805 | 1,941 | 5,774 | 2,938 | 2,836 |
| 59 | 25,949 | 12,595 | 13,354 | 2,531 | 1,277 | 1,254 | 4,454 | 2,404 | 2,050 | 3,535 | 1,593 | 1,942 | 2,150 | 998 | 1,152 | 3,587 | 1,837 | 1,750 |
| 60 | 49,200 | 23,402 | 25,798 | 4,626 | 2,286 | 2,340 | 7,815 | 4,117 | 3,698 | 7,321 | 3,217 | 4,104 | 4,437 | 2,129 | 2,308 | 6,309 | 3,281 | 3,028 |
| 61 | 27,410 | 12,339 | 15,071 | 3,177 | 1,434 | 1,743 | 4,505 | 2,354 | 2,151 | 3,702 | 1,525 | 2,177 | 2,365 | 1,002 | 1,363 | 3,326 | 1,676 | 1,650 |
| 62 | 36,793 | 16,945 | 19,848 | 3,743 | 1,787 | 1,956 | 6,231 | 3,348 | 2,883 | 4,746 | 1,925 | 2,821 | 3,197 | 1,464 | 1,733 | 4,628 | 2,349 | 2,279 |
| 63 | 29,111 | 13,947 | 15,164 | 3,208 | 1,547 | 1,661 | 4,781 | 2,608 | 2,173 | 3,743 | 1,565 | 2,178 | 2,391 | 1,170 | 1,221 | 3,627 | 1,904 | 1,723 |
| 64 | 26,049 | 11,668 | 14,381 | 2,691 | 1,294 | 1,397 | 3,982 | 2,037 | 1,945 | 3,632 | 1,465 | 2,167 | 2,089 | 932 | 1,157 | 3,193 | 1,599 | 1,594 |
| 65 | 34,715 | 16,019 | 18,696 | 3,476 | 1,661 | 1,815 | 5,673 | 2,925 | 2,748 | 4,936 | 2,046 | 2,890 | 2,881 | 1,375 | 1,506 | 4,179 | 2,126 | 2,053 |
| 66 | 20,232 | 8,585 | 11,647 | 2,102 | 938 | 1,164 | 2,890 | 1,400 | 1,490 | 3,041 | 1,175 | 1,866 | 1,608 | 714 | 894 | 2,176 | 1,028 | 1,148 |
| 67 | 19,906 | 9,418 | 10,488 | 2,059 | 1,044 | 1,015 | 3,020 | 1,614 | 1,406 | 2,906 | 1,250 | 1,656 | 1,567 | 713 | 854 | 2,250 | 1,182 | 1,068 |
| 68 | 31,467 | 14,745 | 16,722 | 3,353 | 1,604 | 1,749 | 4,447 | 2,298 | 2,149 | 4,146 | 1,785 | 2,361 | 2,818 | 1,366 | 1,452 | 3,127 | 1,520 | 1,607 |
| 69 | 16,611 | 8,047 | 8,564 | 1,765 | 898 | 867 | 2,102 | 1,150 | 952 | 2,557 | 1,124 | 1,433 | 1,363 | 660 | 703 | 1,759 | 919 | 840 |
| 70 | 31,036 | 14,462 | 16,574 | 2,925 | 1,482 | 1,443 | 4,033 | 2,033 | 2,000 | 5,074 | 2,114 | 2,960 | 2,661 | 1,247 | 1,414 | 3,027 | 1,436 | 1,591 |
| 71 | 14,789 | 6,880 | 7,909 | 1,580 | 765 | 815 | 2,115 | 1,068 | 1,047 | 2,294 | 971 | 1,323 | 1,124 | 543 | 581 | 1,418 | 684 | 734 |
| 72 | 19,534 | 9,158 | 10,376 | 2,085 | 1,042 | 1,043 | 2,791 | 1,454 | 1,337 | 2,952 | 1,237 | 1,715 | 1,541 | 758 | 783 | 2,009 | 936 | 1,073 |
| 73 | 13,433 | 6,555 | 6,878 | 1,464 | 781 | 683 | 1,790 | 966 | 824 | 1,896 | 784 | 1,112 | 1,045 | 542 | 503 | 1,309 | 642 | 667 |
| 74 | 14,556 | 6,890 | 7,666 | 1,606 | 773 | 833 | 1,893 | 947 | 946 | 2,044 | 877 | 1,167 | 1,150 | 604 | 546 | 1,404 | 695 | 709 |
| 75 | 18,706 | 8,952 | 9,754 | 1,968 | 956 | 1,012 | 2,529 | 1,346 | 1,183 | 2,869 | 1,246 | 1,623 | 1,447 | 782 | 665 | 1,809 | 784 | 1,025 |
| 76 | 11,748 | 5,515 | 6,233 | 1,263 | 614 | 649 | 1,555 | 782 | 773 | 1,751 | 762 | 989 | 908 | 447 | 461 | 1,094 | 496 | 598 |
| 77 | 8,368 | 4,208 | 4,160 | 955 | 500 | 455 | 1,063 | 547 | 516 | 1,363 | 652 | 711 | 554 | 284 | 270 | 768 | 349 | 419 |
| 78 | 16,128 | 8,737 | 7,391 | 1,810 | 989 | 821 | 1,956 | 1,092 | 864 | 2,419 | 1,233 | 1,186 | 1,245 | 746 | 499 | 1,515 | 727 | 788 |
| 79 | 8,113 | 4,517 | 3,596 | 871 | 496 | 375 | 916 | 533 | 383 | 1,243 | 632 | 611 | 548 | 341 | 207 | 717 | 353 | 364 |
| 80 | 13,204 | 6,001 | 7,203 | 1,323 | 641 | 682 | 1,644 | 765 | 879 | 2,161 | 894 | 1,267 | 868 | 407 | 461 | 1,192 | 498 | 694 |
| 81 | 5,150 | 2,679 | 2,471 | 513 | 292 | 221 | 616 | 345 | 271 | 811 | 366 | 445 | 349 | 207 | 142 | 465 | 237 | 228 |
| 82 | 6,942 | 3,522 | 3,420 | 724 | 399 | 325 | 765 | 395 | 370 | 1,153 | 510 | 643 | 467 | 275 | 192 | 677 | 297 | 380 |
| 83 | 4,210 | 2,318 | 1,892 | 443 | 266 | 177 | 452 | 248 | 204 | 652 | 304 | 348 | 279 | 176 | 103 | 411 | 205 | 206 |
| 84 | 4,092 | 2,049 | 2,043 | 394 | 221 | 173 | 522 | 269 | 253 | 681 | 311 | 370 | 257 | 136 | 121 | 443 | 188 | 255 |
| 85 | 5,713 | 2,616 | 3,097 | 559 | 309 | 250 | 692 | 322 | 370 | 1,006 | 406 | 600 | 372 | 178 | 194 | 587 | 202 | 385 |
| 86 | 4,255 | 2,110 | 2,145 | 489 | 240 | 249 | 515 | 260 | 255 | 651 | 302 | 349 | 237 | 137 | 100 | 468 | 202 | 266 |
| 87 | 2,474 | 1,201 | 1,273 | 254 | 134 | 120 | 271 | 125 | 146 | 407 | 179 | 228 | 150 | 74 | 76 | 264 | 120 | 144 |
| 88 | 4,036 | 2,262 | 1,774 | 458 | 281 | 177 | 382 | 204 | 178 | 660 | 357 | 303 | 293 | 174 | 119 | 447 | 230 | 217 |
| 89 | 2,640 | 1,462 | 1,178 | 282 | 165 | 117 | 267 | 156 | 111 | 447 | 249 | 198 | 131 | 71 | 60 | 236 | 105 | 131 |
| 90 | 2,998 | 1,488 | 1,510 | 288 | 172 | 116 | 309 | 168 | 141 | 522 | 228 | 294 | 166 | 80 | 86 | 292 | 127 | 165 |
| 91 | 977 | 552 | 425 | 101 | 60 | 41 | 104 | 63 | 41 | 172 | 89 | 83 | 41 | 27 | 14 | 95 | 53 | 42 |
| 92 | 1,192 | 609 | 583 | 118 | 62 | 56 | 124 | 58 | 66 | 195 | 92 | 103 | 63 | 39 | 24 | 121 | 57 | 64 |
| 93 | 569 | 268 | 301 | 44 | 22 | 22 | 70 | 28 | 42 | 88 | 37 | 51 | 28 | 14 | 14 | 63 | 24 | 39 |
| 94 | 714 | 328 | 386 | 69 | 36 | 33 | 50 | 25 | 25 | 95 | 26 | 69 | 27 | 14 | 13 | 63 | 25 | 38 |
| 95+ | 7,935 | 2,896 | 5,039 | 755 | 272 | 483 | 642 | 234 | 408 | 1,365 | 498 | 867 | 321 | 108 | 213 | 770 | 226 | 544 |
| Median Age | 16.9 | 16.7 | 17.0 | 16.3 | 16.2 | 16.4 | 18.5 | 18.7 | 18.2 | 16.1 | 15.7 | 16.4 | 15.9 | 15.5 | 16.2 | 19.0 | 19.5 | 18.7 |
| **Age groups** | | | | | | | | | | | | | | | | | | |
| **Total** | **13,092,666** | **6,454,647** | **6,638,019** | **1,307,111** | **648,465** | **658,646** | **1,972,317** | **981,887** | **990,430** | **1,592,661** | **784,680** | **807,981** | **991,927** | **488,589** | **503,338** | **2,191,225** | **1,082,998** | **1,108,227** |
| 0 - 14 | 5,943,169 | 2,954,372 | 2,988,797 | 609,144 | 303,521 | 305,623 | 806,608 | 398,080 | 408,528 | 753,567 | 376,749 | 376,818 | 474,856 | 237,719 | 237,137 | 885,562 | 432,621 | 452,941 |
| 15 - 64 | 6,803,054 | 3,335,226 | 3,467,828 | 661,871 | 326,829 | 335,042 | 1,119,511 | 559,987 | 559,524 | 786,537 | 385,195 | 401,342 | 490,562 | 237,631 | 252,931 | 1,270,508 | 633,704 | 636,804 |
| 65+ | 346,443 | 165,049 | 181,394 | 36,096 | 18,115 | 17,981 | 46,198 | 23,820 | 22,378 | 52,557 | 22,736 | 29,821 | 26,509 | 13,239 | 13,270 | 35,155 | 16,673 | 18,482 |
| | | | | | | | | | | | | | | | | | | |
| Overall Dependency Ratio | 92.5 | 93.5 | 91.4 | 97.5 | 98.4 | 96.6 | 76.2 | 75.3 | 77.0 | 102.5 | 103.7 | 101.3 | 102.2 | 105.6 | 99.0 | 72.5 | 70.9 | 74.0 |
| Child Dependency Ratio | 87.4 | 88.6 | 86.2 | 92.0 | 92.9 | 91.2 | 72.1 | 71.1 | 73.0 | 95.8 | 97.8 | 93.9 | 96.8 | 100.0 | 93.8 | 69.7 | 68.3 | 71.1 |
| Aged Dependency Ratio | 5.1 | 4.9 | 5.2 | 5.5 | 5.5 | 5.4 | 4.1 | 4.3 | 4.0 | 6.7 | 5.9 | 7.4 | 5.4 | 5.6 | 5.2 | 2.8 | 2.6 | 2.9 |

## Table A4: Population (De Jure) by Single Year Age and Sex by Province, Rural/Urban, Zambia 2010

| Single Year Ages | Muchinga | | | Northern | | | North Western | | | Southern | | | Western | | |
|---|---|---|---|---|---|---|---|---|---|---|---|---|---|---|---|
| | Total | Male | Female | Total | Male | Female | Total | Male | Female | Total | Male | Female | Total | Male | Female |
| **Total** | **711,657** | **349,872** | **361,785** | **1,105,824** | **546,851** | **558,973** | **727,044** | **358,141** | **368,903** | **1,589,926** | **779,659** | **810,267** | **902,974** | **433,505** | **469,469** |
| 0 | 26,539 | 13,154 | 13,385 | 42,516 | 21,299 | 21,217 | 26,411 | 13,311 | 13,100 | 57,793 | 28,935 | 28,858 | 31,183 | 15,740 | 15,443 |
| 1 | 25,290 | 12,737 | 12,553 | 38,868 | 19,369 | 19,499 | 26,814 | 13,277 | 13,537 | 57,567 | 28,769 | 28,798 | 30,262 | 15,188 | 15,074 |
| 2 | 26,502 | 13,142 | 13,360 | 42,942 | 21,573 | 21,369 | 28,757 | 14,288 | 14,469 | 58,826 | 29,126 | 29,700 | 34,618 | 17,177 | 17,441 |
| 3 | 26,335 | 13,063 | 13,272 | 41,769 | 20,917 | 20,852 | 27,695 | 13,763 | 13,932 | 58,429 | 29,003 | 29,426 | 33,827 | 16,821 | 17,006 |
| 4 | 27,725 | 13,683 | 14,042 | 44,090 | 21,887 | 22,203 | 27,628 | 13,772 | 13,856 | 58,320 | 29,081 | 29,239 | 34,621 | 17,404 | 17,217 |
| 5 | 25,141 | 12,830 | 12,311 | 38,954 | 19,469 | 19,485 | 25,500 | 12,785 | 12,715 | 52,580 | 26,239 | 26,341 | 30,479 | 15,360 | 15,119 |
| 6 | 24,451 | 12,122 | 12,329 | 37,978 | 18,923 | 19,055 | 24,851 | 12,352 | 12,499 | 53,434 | 26,543 | 26,891 | 29,403 | 14,823 | 14,580 |
| 7 | 24,295 | 12,191 | 12,104 | 37,017 | 18,670 | 18,347 | 24,424 | 12,278 | 12,146 | 49,694 | 24,945 | 24,749 | 28,261 | 14,026 | 14,235 |
| 8 | 21,921 | 10,763 | 11,158 | 34,328 | 16,806 | 17,522 | 21,321 | 10,591 | 10,730 | 45,840 | 22,815 | 23,025 | 24,484 | 12,319 | 12,165 |
| 9 | 19,279 | 9,673 | 9,606 | 27,816 | 13,768 | 14,048 | 18,845 | 9,400 | 9,445 | 42,694 | 21,186 | 21,508 | 23,622 | 11,865 | 11,757 |
| 10 | 27,703 | 13,899 | 13,804 | 44,445 | 22,333 | 22,112 | 27,999 | 14,106 | 13,893 | 57,181 | 28,508 | 28,673 | 32,445 | 16,283 | 16,162 |
| 11 | 16,023 | 8,028 | 7,995 | 24,965 | 12,527 | 12,438 | 17,990 | 8,958 | 9,032 | 37,976 | 18,943 | 19,033 | 20,761 | 10,422 | 10,339 |
| 12 | 21,917 | 11,191 | 10,726 | 32,606 | 16,765 | 15,841 | 19,937 | 10,011 | 9,926 | 45,058 | 22,361 | 22,697 | 23,480 | 11,751 | 11,729 |
| 13 | 16,019 | 8,013 | 8,006 | 24,414 | 12,195 | 12,219 | 17,819 | 8,957 | 8,862 | 42,183 | 20,956 | 21,227 | 21,354 | 10,686 | 10,668 |
| 14 | 16,874 | 8,544 | 8,330 | 25,418 | 12,858 | 12,560 | 18,503 | 9,170 | 9,333 | 36,994 | 18,318 | 18,676 | 21,429 | 10,678 | 10,751 |
| 15 | 18,436 | 9,435 | 9,001 | 27,222 | 13,904 | 13,318 | 18,223 | 9,016 | 9,207 | 42,661 | 21,439 | 21,222 | 21,190 | 10,537 | 10,653 |
| 16 | 15,916 | 8,002 | 7,914 | 24,236 | 11,963 | 12,273 | 17,426 | 8,708 | 8,718 | 40,165 | 19,755 | 20,410 | 20,903 | 10,330 | 10,573 |
| 17 | 13,824 | 6,717 | 7,107 | 21,705 | 10,603 | 11,102 | 15,657 | 7,730 | 7,927 | 33,746 | 16,688 | 17,058 | 18,223 | 8,857 | 9,366 |
| 18 | 17,464 | 8,364 | 9,100 | 27,099 | 13,103 | 13,996 | 17,922 | 8,841 | 9,081 | 41,589 | 20,646 | 20,943 | 21,399 | 10,337 | 11,062 |
| 19 | 13,194 | 6,088 | 7,106 | 20,394 | 9,483 | 10,911 | 14,081 | 6,917 | 7,164 | 32,929 | 16,439 | 16,490 | 17,597 | 8,361 | 9,236 |
| 20 | 16,451 | 7,299 | 9,152 | 25,689 | 11,463 | 14,226 | 14,328 | 6,916 | 7,412 | 35,480 | 17,063 | 18,417 | 18,944 | 8,757 | 10,187 |
| 21 | 10,180 | 4,665 | 5,515 | 14,855 | 6,963 | 7,892 | 11,180 | 5,243 | 5,937 | 28,280 | 13,475 | 14,805 | 14,575 | 6,559 | 8,016 |
| 22 | 12,241 | 5,455 | 6,786 | 18,996 | 8,603 | 10,393 | 12,514 | 5,724 | 6,790 | 28,238 | 13,344 | 14,894 | 15,954 | 7,006 | 8,948 |
| 23 | 10,679 | 4,937 | 5,742 | 16,126 | 7,441 | 8,685 | 11,607 | 5,517 | 6,090 | 28,046 | 13,322 | 14,724 | 14,330 | 6,246 | 8,084 |
| 24 | 10,545 | 4,795 | 5,750 | 15,897 | 6,982 | 8,915 | 11,095 | 5,039 | 6,056 | 25,906 | 11,908 | 13,998 | 14,067 | 6,206 | 7,861 |
| 25 | 13,613 | 6,429 | 7,184 | 21,681 | 10,207 | 11,474 | 12,265 | 5,788 | 6,477 | 27,565 | 13,070 | 14,495 | 15,413 | 6,899 | 8,514 |
| 26 | 10,147 | 4,557 | 5,590 | 16,118 | 7,324 | 8,794 | 10,892 | 5,017 | 5,875 | 24,996 | 11,476 | 13,520 | 13,753 | 6,110 | 7,643 |
| 27 | 9,289 | 4,288 | 5,001 | 14,297 | 6,662 | 7,635 | 10,505 | 4,733 | 5,772 | 22,420 | 10,620 | 11,800 | 12,573 | 5,672 | 6,901 |
| 28 | 11,948 | 5,471 | 6,477 | 18,378 | 8,471 | 9,907 | 11,997 | 5,555 | 6,442 | 26,623 | 12,318 | 14,305 | 14,752 | 6,520 | 8,232 |
| 29 | 7,693 | 3,596 | 4,097 | 11,655 | 5,549 | 6,106 | 8,152 | 3,907 | 4,245 | 19,590 | 9,205 | 10,385 | 10,817 | 5,020 | 5,797 |
| 30 | 13,375 | 6,835 | 6,540 | 20,937 | 10,859 | 10,078 | 12,476 | 6,115 | 6,361 | 26,931 | 13,151 | 13,780 | 15,344 | 7,185 | 8,159 |
| 31 | 5,586 | 2,817 | 2,769 | 8,650 | 4,289 | 4,361 | 6,908 | 3,449 | 3,459 | 16,715 | 8,489 | 8,226 | 8,782 | 4,165 | 4,617 |
| 32 | 8,935 | 4,429 | 4,506 | 13,622 | 6,869 | 6,753 | 8,704 | 4,289 | 4,415 | 21,360 | 10,487 | 10,873 | 11,039 | 5,164 | 5,875 |
| 33 | 5,538 | 2,749 | 2,789 | 8,572 | 4,287 | 4,285 | 6,638 | 3,392 | 3,246 | 15,738 | 7,732 | 8,006 | 8,595 | 4,057 | 4,538 |
| 34 | 6,394 | 3,205 | 3,189 | 9,739 | 4,862 | 4,877 | 6,952 | 3,576 | 3,376 | 17,298 | 8,592 | 8,706 | 9,151 | 4,459 | 4,692 |
| 35 | 9,692 | 5,073 | 4,619 | 15,293 | 8,189 | 7,104 | 8,726 | 4,542 | 4,184 | 20,308 | 10,647 | 9,661 | 11,135 | 5,531 | 5,604 |
| 36 | 6,776 | 3,408 | 3,368 | 10,774 | 5,285 | 5,489 | 6,700 | 3,354 | 3,346 | 16,562 | 8,324 | 8,238 | 8,819 | 4,226 | 4,593 |
| 37 | 5,188 | 2,612 | 2,576 | 8,396 | 4,197 | 4,199 | 5,959 | 2,905 | 3,054 | 13,260 | 6,661 | 6,599 | 7,298 | 3,381 | 3,917 |
| 38 | 7,675 | 3,806 | 3,869 | 12,074 | 6,205 | 5,869 | 7,356 | 3,698 | 3,658 | 16,183 | 8,201 | 7,982 | 8,931 | 4,308 | 4,623 |
| 39 | 4,491 | 2,286 | 2,205 | 7,168 | 3,693 | 3,475 | 4,942 | 2,394 | 2,548 | 10,583 | 5,352 | 5,231 | 6,026 | 2,806 | 3,220 |
| 40 | 8,194 | 4,410 | 3,784 | 12,734 | 6,958 | 5,776 | 7,811 | 4,071 | 3,740 | 15,377 | 7,934 | 7,443 | 9,445 | 4,730 | 4,715 |
| 41 | 3,819 | 1,909 | 1,910 | 5,765 | 2,930 | 2,835 | 4,467 | 2,239 | 2,228 | 9,189 | 4,573 | 4,616 | 4,857 | 2,328 | 2,529 |
| 42 | 5,961 | 3,138 | 2,823 | 9,517 | 5,153 | 4,364 | 6,275 | 3,218 | 3,057 | 13,054 | 6,698 | 6,356 | 7,770 | 3,652 | 4,118 |
| 43 | 3,022 | 1,550 | 1,472 | 5,354 | 2,750 | 2,604 | 3,067 | 1,574 | 1,493 | 7,563 | 3,765 | 3,798 | 4,536 | 2,169 | 2,367 |
| 44 | 3,108 | 1,477 | 1,631 | 4,917 | 2,401 | 2,516 | 3,193 | 1,525 | 1,668 | 7,913 | 3,664 | 4,249 | 4,234 | 1,885 | 2,349 |
| 45 | 5,404 | 2,811 | 2,593 | 8,594 | 4,524 | 4,070 | 4,550 | 2,301 | 2,249 | 9,765 | 4,795 | 4,970 | 6,046 | 2,792 | 3,254 |
| 46 | 4,961 | 2,445 | 2,516 | 7,789 | 3,850 | 3,939 | 5,110 | 2,517 | 2,593 | 10,752 | 5,287 | 5,465 | 5,870 | 2,681 | 3,189 |
| 47 | 2,892 | 1,433 | 1,459 | 4,860 | 2,452 | 2,408 | 3,367 | 1,709 | 1,658 | 6,815 | 3,297 | 3,518 | 4,286 | 1,915 | 2,371 |
| 48 | 4,020 | 1,967 | 2,053 | 6,780 | 3,429 | 3,351 | 3,994 | 1,973 | 2,021 | 8,588 | 4,067 | 4,521 | 5,595 | 2,457 | 3,138 |
| 49 | 2,691 | 1,288 | 1,403 | 4,535 | 2,276 | 2,259 | 2,726 | 1,333 | 1,393 | 5,712 | 2,739 | 2,973 | 3,955 | 1,764 | 2,191 |
| 50 | 4,742 | 2,361 | 2,381 | 7,768 | 3,964 | 3,804 | 4,152 | 1,971 | 2,181 | 8,311 | 3,943 | 4,368 | 6,252 | 2,758 | 3,494 |
| 51 | 2,232 | 1,047 | 1,185 | 3,595 | 1,777 | 1,818 | 2,357 | 1,133 | 1,224 | 5,098 | 2,389 | 2,709 | 3,349 | 1,398 | 1,951 |
| 52 | 3,339 | 1,638 | 1,701 | 5,273 | 2,677 | 2,596 | 3,308 | 1,646 | 1,662 | 7,430 | 3,474 | 3,956 | 4,871 | 2,073 | 2,798 |
| 53 | 2,194 | 1,099 | 1,095 | 3,640 | 1,879 | 1,761 | 2,299 | 1,151 | 1,148 | 4,973 | 2,379 | 2,594 | 3,715 | 1,596 | 2,119 |

## Table A4: Population (De Jure) by Single Year Age and Sex by Province, Rural/Urban, Zambia 2010

| Single Year Ages | Muchinga | | | Northern | | | North Western | | | Southern | | | Western | | |
|---|---|---|---|---|---|---|---|---|---|---|---|---|---|---|---|
| | Total | Male | Female | Total | Male | Female | Total | Male | Female | Total | Male | Female | Total | Male | Female |
| 54 | 2,303 | 1,118 | 1,185 | 3,825 | 1,920 | 1,905 | 2,305 | 1,054 | 1,251 | 5,362 | 2,511 | 2,851 | 3,395 | 1,493 | 1,902 |
| 55 | 2,524 | 1,292 | 1,232 | 4,149 | 2,150 | 1,999 | 2,362 | 1,168 | 1,194 | 4,785 | 2,330 | 2,455 | 3,275 | 1,432 | 1,843 |
| 56 | 2,376 | 1,192 | 1,184 | 3,694 | 1,862 | 1,832 | 2,375 | 1,176 | 1,199 | 4,960 | 2,347 | 2,613 | 3,414 | 1,510 | 1,904 |
| 57 | 1,592 | 774 | 818 | 2,591 | 1,301 | 1,290 | 1,660 | 820 | 840 | 3,317 | 1,640 | 1,677 | 2,720 | 1,183 | 1,537 |
| 58 | 2,153 | 1,011 | 1,142 | 3,763 | 1,876 | 1,887 | 2,238 | 1,091 | 1,147 | 4,203 | 1,974 | 2,229 | 3,407 | 1,485 | 1,922 |
| 59 | 1,360 | 641 | 719 | 2,374 | 1,088 | 1,286 | 1,455 | 717 | 738 | 2,380 | 1,155 | 1,225 | 2,123 | 885 | 1,238 |
| 60 | 3,013 | 1,329 | 1,684 | 4,603 | 2,159 | 2,444 | 2,566 | 1,169 | 1,397 | 4,489 | 2,019 | 2,470 | 4,021 | 1,696 | 2,325 |
| 61 | 1,469 | 603 | 866 | 2,280 | 965 | 1,315 | 1,513 | 697 | 816 | 2,863 | 1,228 | 1,635 | 2,210 | 855 | 1,355 |
| 62 | 2,008 | 866 | 1,142 | 3,149 | 1,405 | 1,744 | 1,963 | 890 | 1,073 | 3,794 | 1,607 | 2,187 | 3,334 | 1,304 | 2,030 |
| 63 | 1,730 | 808 | 922 | 2,548 | 1,228 | 1,320 | 1,494 | 703 | 791 | 3,114 | 1,393 | 1,721 | 2,475 | 1,021 | 1,454 |
| 64 | 1,564 | 688 | 876 | 2,239 | 986 | 1,253 | 1,417 | 582 | 835 | 2,890 | 1,206 | 1,684 | 2,352 | 879 | 1,473 |
| 65 | 2,182 | 969 | 1,213 | 3,346 | 1,536 | 1,810 | 1,858 | 813 | 1,045 | 3,460 | 1,462 | 1,998 | 2,724 | 1,106 | 1,618 |
| 66 | 1,210 | 485 | 725 | 1,748 | 770 | 978 | 1,150 | 445 | 705 | 2,379 | 898 | 1,481 | 1,928 | 732 | 1,196 |
| 67 | 1,202 | 579 | 623 | 1,858 | 847 | 1,011 | 1,020 | 446 | 574 | 2,296 | 997 | 1,299 | 1,728 | 746 | 982 |
| 68 | 2,038 | 912 | 1,126 | 3,042 | 1,432 | 1,610 | 1,915 | 925 | 990 | 3,628 | 1,582 | 2,046 | 2,953 | 1,321 | 1,632 |
| 69 | 1,005 | 511 | 494 | 1,667 | 824 | 843 | 1,040 | 473 | 567 | 1,769 | 769 | 1,000 | 1,584 | 719 | 865 |
| 70 | 2,050 | 970 | 1,080 | 3,066 | 1,505 | 1,561 | 2,027 | 935 | 1,092 | 3,108 | 1,346 | 1,762 | 3,065 | 1,394 | 1,671 |
| 71 | 895 | 413 | 482 | 1,258 | 608 | 650 | 797 | 394 | 403 | 1,859 | 785 | 1,074 | 1,449 | 649 | 800 |
| 72 | 1,233 | 591 | 642 | 1,732 | 869 | 863 | 1,121 | 525 | 596 | 2,187 | 919 | 1,268 | 1,883 | 827 | 1,056 |
| 73 | 891 | 459 | 432 | 1,250 | 669 | 581 | 769 | 378 | 391 | 1,543 | 622 | 921 | 1,476 | 712 | 764 |
| 74 | 957 | 489 | 468 | 1,312 | 681 | 631 | 874 | 440 | 434 | 1,913 | 758 | 1,155 | 1,403 | 626 | 777 |
| 75 | 1,262 | 626 | 636 | 1,770 | 924 | 846 | 1,262 | 590 | 672 | 2,048 | 884 | 1,164 | 1,742 | 814 | 928 |
| 76 | 806 | 411 | 395 | 1,056 | 556 | 500 | 709 | 335 | 374 | 1,392 | 592 | 800 | 1,214 | 520 | 694 |
| 77 | 506 | 280 | 226 | 783 | 449 | 334 | 542 | 269 | 273 | 1,012 | 471 | 541 | 822 | 407 | 415 |
| 78 | 1,051 | 577 | 474 | 1,444 | 870 | 574 | 1,018 | 578 | 440 | 1,956 | 1,005 | 951 | 1,714 | 920 | 794 |
| 79 | 486 | 277 | 209 | 813 | 516 | 297 | 593 | 364 | 229 | 883 | 432 | 451 | 1,043 | 573 | 470 |
| 80 | 849 | 424 | 425 | 1,229 | 631 | 598 | 1,003 | 469 | 534 | 1,426 | 593 | 833 | 1,509 | 679 | 830 |
| 81 | 313 | 171 | 142 | 438 | 245 | 193 | 357 | 195 | 162 | 687 | 330 | 357 | 601 | 291 | 310 |
| 82 | 412 | 231 | 181 | 613 | 349 | 264 | 405 | 213 | 192 | 788 | 371 | 417 | 938 | 482 | 456 |
| 83 | 291 | 179 | 112 | 390 | 261 | 129 | 272 | 157 | 115 | 520 | 250 | 270 | 500 | 272 | 228 |
| 84 | 257 | 159 | 98 | 340 | 196 | 144 | 232 | 126 | 106 | 506 | 224 | 282 | 460 | 219 | 241 |
| 85 | 322 | 176 | 146 | 469 | 238 | 231 | 365 | 188 | 177 | 651 | 278 | 373 | 690 | 319 | 371 |
| 86 | 228 | 119 | 109 | 303 | 154 | 149 | 226 | 125 | 101 | 540 | 257 | 283 | 598 | 314 | 284 |
| 87 | 130 | 66 | 64 | 213 | 110 | 103 | 144 | 75 | 69 | 293 | 138 | 155 | 348 | 180 | 168 |
| 88 | 260 | 149 | 111 | 388 | 236 | 152 | 246 | 161 | 85 | 459 | 233 | 226 | 443 | 237 | 206 |
| 89 | 146 | 75 | 71 | 216 | 140 | 76 | 203 | 125 | 78 | 323 | 181 | 142 | 389 | 195 | 194 |
| 90 | 167 | 88 | 79 | 243 | 139 | 104 | 252 | 151 | 101 | 357 | 145 | 212 | 402 | 190 | 212 |
| 91 | 38 | 26 | 12 | 67 | 41 | 26 | 76 | 47 | 29 | 122 | 63 | 59 | 161 | 83 | 78 |
| 92 | 62 | 28 | 34 | 82 | 42 | 40 | 91 | 54 | 37 | 160 | 85 | 75 | 176 | 92 | 84 |
| 93 | 39 | 19 | 20 | 35 | 19 | 16 | 30 | 12 | 18 | 78 | 38 | 40 | 94 | 55 | 39 |
| 94 | 32 | 13 | 19 | 40 | 15 | 25 | 57 | 28 | 29 | 104 | 47 | 57 | 177 | 99 | 78 |
| 95+ | 382 | 154 | 228 | 578 | 204 | 374 | 667 | 293 | 374 | 1,041 | 358 | 683 | 1,414 | 549 | 865 |
| Median Age | 15.5 | 15.2 | 15.9 | 15.5 | 15.3 | 15.8 | 15.5 | 15.2 | 15.8 | 15.9 | 15.7 | 16.2 | 16.5 | 15.6 | 17.4 |
| **Age groups** | | | | | | | | | | | | | | | |
| **Total** | **711,657** | **349,872** | **361,785** | **1,105,824** | **546,851** | **558,973** | **727,044** | **358,141** | **368,903** | **1,589,926** | **779,659** | **810,267** | **902,974** | **433,505** | **469,469** |
| 0 - 14 | 346,014 | 173,033 | 172,981 | 538,126 | 269,359 | 268,767 | 354,494 | 177,019 | 177,475 | 754,569 | 375,728 | 378,841 | 420,229 | 210,543 | 209,686 |
| 15 - 64 | 343,941 | 166,213 | 177,728 | 535,909 | 261,416 | 274,493 | 351,229 | 170,793 | 180,436 | 795,869 | 386,818 | 409,051 | 447,117 | 206,640 | 240,477 |
| 65+ | 21,702 | 10,626 | 11,076 | 31,789 | 16,076 | 15,713 | 21,321 | 10,329 | 10,992 | 39,488 | 17,113 | 22,375 | 35,628 | 16,322 | 19,306 |
| | | | | | | | | | | | | | | | |
| Overall Dependency Ratio | 106.9 | 110.5 | 103.6 | 106.3 | 109.2 | 103.6 | 107.0 | 109.7 | 104.5 | 99.8 | 101.6 | 98.1 | 102.0 | 109.8 | 95.2 |
| Child Dependency Ratio | 100.6 | 104.1 | 97.3 | 100.4 | 103.0 | 97.9 | 100.9 | 103.6 | 98.4 | 94.8 | 97.1 | 92.6 | 94.0 | 101.9 | 87.2 |
| Aged Dependency Ratio | 6.3 | 6.4 | 6.2 | 5.9 | 6.1 | 5.7 | 6.1 | 6.0 | 6.1 | 5.0 | 4.4 | 5.5 | 8.0 | 7.9 | 8.0 |

## Table A4: Population (De Jure) by Single Years Age and Sex by Province, Rural/Urban Zambia 2010

| Single Year Ages | Zambia Rural | | | Central | | | Copperbelt | | | Eastern | | | Luapula | | | Lusaka | | |
|---|---|---|---|---|---|---|---|---|---|---|---|---|---|---|---|---|---|---|
| | Total | Male | Female | Total | Male | Female | Total | Male | Female | Total | Male | Female | Total | Male | Female | Total | Male | Female |
| **Total Rural** | **7,919,216** | **3,906,636** | **4,012,580** | **978,574** | **487,713** | **490,861** | **376,861** | **190,178** | **186,683** | **1,392,338** | **686,577** | **705,761** | **797,407** | **393,615** | **403,792** | **336,318** | **169,604** | **166,714** |
| 0 | 292,656 | 146,131 | 146,525 | 35,783 | 17,859 | 17,924 | 11,944 | 5,962 | 5,982 | 50,274 | 24,995 | 25,279 | 29,927 | 14,948 | 14,979 | 10,885 | 5,333 | 5,552 |
| 1 | 285,812 | 142,580 | 143,232 | 35,029 | 17,423 | 17,606 | 12,986 | 6,527 | 6,459 | 50,426 | 25,147 | 25,279 | 28,722 | 14,364 | 14,358 | 11,640 | 5,738 | 5,902 |
| 2 | 308,024 | 152,959 | 155,065 | 37,511 | 18,599 | 18,912 | 13,620 | 6,804 | 6,816 | 52,877 | 26,087 | 26,790 | 31,676 | 15,707 | 15,969 | 11,608 | 5,872 | 5,736 |
| 3 | 298,292 | 148,004 | 150,288 | 36,637 | 18,168 | 18,469 | 13,117 | 6,411 | 6,706 | 50,488 | 24,736 | 25,752 | 29,682 | 14,720 | 14,962 | 11,562 | 5,767 | 5,795 |
| 4 | 303,372 | 151,330 | 152,042 | 36,186 | 17,964 | 18,222 | 13,128 | 6,606 | 6,522 | 50,720 | 25,331 | 25,389 | 31,361 | 15,594 | 15,767 | 11,086 | 5,490 | 5,596 |
| 5 | 277,239 | 139,762 | 137,477 | 34,075 | 17,313 | 16,762 | 12,430 | 6,227 | 6,203 | 48,327 | 24,268 | 24,059 | 28,213 | 14,220 | 13,993 | 10,592 | 5,347 | 5,245 |
| 6 | 272,443 | 136,270 | 136,173 | 33,814 | 17,010 | 16,804 | 12,131 | 6,093 | 6,038 | 46,313 | 23,060 | 23,253 | 27,564 | 13,918 | 13,646 | 10,344 | 5,086 | 5,258 |
| 7 | 263,208 | 132,536 | 130,672 | 32,071 | 16,234 | 15,837 | 11,967 | 6,065 | 5,902 | 47,620 | 23,908 | 23,712 | 26,111 | 13,184 | 12,927 | 10,309 | 5,275 | 5,034 |
| 8 | 239,430 | 119,224 | 120,206 | 28,983 | 14,416 | 14,567 | 11,035 | 5,540 | 5,495 | 41,767 | 20,778 | 20,989 | 25,525 | 12,762 | 12,763 | 9,223 | 4,568 | 4,655 |
| 9 | 207,797 | 104,272 | 103,525 | 25,626 | 12,690 | 12,936 | 9,468 | 4,730 | 4,738 | 35,642 | 18,110 | 17,532 | 19,962 | 10,001 | 9,961 | 8,334 | 4,168 | 4,166 |
| 10 | 306,022 | 154,656 | 151,366 | 37,106 | 18,716 | 18,390 | 13,918 | 7,059 | 6,859 | 53,927 | 27,497 | 26,430 | 32,632 | 16,583 | 16,049 | 11,840 | 5,865 | 5,975 |
| 11 | 188,184 | 94,843 | 93,341 | 23,616 | 11,892 | 11,724 | 8,504 | 4,211 | 4,293 | 33,664 | 16,947 | 16,717 | 18,426 | 9,272 | 9,154 | 8,011 | 3,975 | 4,036 |
| 12 | 228,795 | 116,589 | 112,206 | 28,920 | 14,633 | 14,287 | 11,051 | 5,615 | 5,436 | 40,681 | 20,879 | 19,802 | 22,838 | 11,611 | 11,227 | 9,595 | 4,832 | 4,763 |
| 13 | 190,813 | 96,561 | 94,252 | 24,822 | 12,473 | 12,349 | 9,189 | 4,627 | 4,562 | 33,143 | 16,932 | 16,211 | 17,449 | 8,919 | 8,530 | 8,673 | 4,217 | 4,456 |
| 14 | 188,231 | 95,500 | 92,731 | 24,668 | 12,488 | 12,180 | 9,623 | 4,916 | 4,707 | 33,129 | 16,894 | 16,235 | 18,367 | 9,276 | 9,091 | 8,235 | 4,066 | 4,169 |
| 15 | 197,550 | 101,506 | 96,044 | 24,646 | 12,641 | 12,005 | 9,337 | 4,786 | 4,551 | 35,049 | 18,355 | 16,694 | 18,788 | 9,598 | 9,190 | 8,698 | 4,415 | 4,283 |
| 16 | 183,685 | 92,471 | 91,214 | 24,215 | 12,199 | 12,016 | 9,042 | 4,550 | 4,492 | 31,863 | 16,275 | 15,588 | 17,406 | 8,660 | 8,746 | 8,413 | 4,167 | 4,246 |
| 17 | 155,396 | 77,518 | 77,878 | 19,661 | 9,950 | 9,711 | 7,703 | 3,909 | 3,794 | 26,593 | 13,502 | 13,091 | 15,177 | 7,459 | 7,718 | 6,985 | 3,543 | 3,442 |
| 18 | 189,819 | 93,664 | 96,155 | 24,203 | 12,152 | 12,051 | 9,318 | 4,699 | 4,619 | 33,112 | 16,520 | 16,592 | 18,794 | 9,128 | 9,666 | 8,438 | 4,307 | 4,131 |
| 19 | 148,604 | 71,535 | 77,069 | 18,838 | 9,181 | 9,657 | 6,940 | 3,397 | 3,543 | 27,463 | 13,196 | 14,267 | 14,365 | 6,809 | 7,556 | 6,935 | 3,424 | 3,511 |
| 20 | 166,465 | 77,602 | 88,863 | 20,673 | 9,882 | 10,791 | 7,493 | 3,565 | 3,928 | 29,577 | 14,114 | 15,463 | 17,178 | 7,761 | 9,417 | 7,195 | 3,357 | 3,838 |
| 21 | 115,395 | 54,570 | 60,825 | 15,419 | 7,521 | 7,898 | 5,130 | 2,483 | 2,647 | 19,534 | 9,457 | 10,077 | 10,242 | 4,654 | 5,588 | 5,451 | 2,575 | 2,876 |
| 22 | 132,107 | 60,321 | 71,786 | 16,499 | 7,662 | 8,837 | 6,055 | 2,774 | 3,281 | 24,119 | 11,170 | 12,949 | 12,643 | 5,421 | 7,222 | 6,112 | 2,888 | 3,224 |
| 23 | 120,269 | 56,292 | 63,977 | 15,379 | 7,361 | 8,018 | 5,347 | 2,565 | 2,782 | 22,137 | 10,542 | 11,595 | 10,695 | 4,682 | 6,013 | 5,773 | 2,798 | 2,975 |
| 24 | 115,766 | 52,939 | 62,827 | 14,791 | 6,913 | 7,878 | 5,143 | 2,420 | 2,723 | 20,451 | 9,564 | 10,887 | 11,104 | 4,752 | 6,352 | 5,529 | 2,702 | 2,827 |
| 25 | 136,077 | 64,745 | 71,332 | 16,406 | 7,999 | 8,407 | 6,307 | 3,045 | 3,262 | 24,063 | 11,610 | 12,453 | 14,078 | 6,516 | 7,562 | 6,189 | 3,043 | 3,146 |
| 26 | 113,714 | 52,230 | 61,484 | 13,904 | 6,612 | 7,292 | 5,262 | 2,477 | 2,785 | 20,215 | 9,372 | 10,843 | 11,375 | 5,020 | 6,355 | 5,405 | 2,630 | 2,775 |
| 27 | 102,340 | 47,893 | 54,447 | 12,519 | 5,959 | 6,560 | 4,720 | 2,300 | 2,420 | 18,106 | 8,640 | 9,466 | 10,224 | 4,586 | 5,638 | 4,797 | 2,381 | 2,416 |
| 28 | 124,698 | 57,579 | 67,119 | 14,940 | 7,002 | 7,938 | 5,857 | 2,764 | 3,093 | 21,791 | 10,260 | 11,531 | 13,227 | 5,925 | 7,302 | 5,654 | 2,812 | 2,842 |
| 29 | 87,535 | 41,501 | 46,034 | 11,121 | 5,373 | 5,748 | 4,164 | 2,039 | 2,125 | 15,594 | 7,391 | 8,203 | 8,569 | 4,059 | 4,510 | 4,293 | 2,136 | 2,157 |
| 30 | 134,918 | 67,329 | 67,589 | 15,874 | 7,835 | 8,039 | 6,514 | 3,358 | 3,156 | 23,759 | 12,081 | 11,678 | 15,232 | 7,810 | 7,422 | 6,037 | 3,155 | 2,882 |
| 31 | 69,936 | 35,023 | 34,913 | 9,098 | 4,637 | 4,461 | 3,357 | 1,767 | 1,590 | 11,786 | 5,999 | 5,787 | 6,711 | 3,300 | 3,411 | 3,495 | 1,861 | 1,634 |
| 32 | 96,804 | 47,794 | 49,010 | 12,129 | 6,062 | 6,067 | 4,992 | 2,565 | 2,427 | 17,099 | 8,422 | 8,677 | 10,113 | 5,041 | 5,072 | 5,103 | 2,683 | 2,420 |
| 33 | 69,855 | 34,638 | 35,217 | 8,806 | 4,411 | 4,395 | 3,461 | 1,836 | 1,625 | 12,658 | 6,338 | 6,320 | 6,933 | 3,411 | 3,522 | 3,512 | 1,833 | 1,679 |
| 34 | 75,421 | 37,513 | 37,908 | 9,794 | 4,945 | 4,849 | 3,843 | 1,977 | 1,866 | 12,728 | 6,324 | 6,404 | 7,413 | 3,590 | 3,823 | 3,752 | 2,089 | 1,663 |
| 35 | 99,631 | 52,187 | 47,444 | 11,920 | 6,334 | 5,586 | 5,137 | 2,920 | 2,217 | 17,177 | 8,940 | 8,237 | 10,916 | 5,782 | 5,134 | 4,685 | 2,654 | 2,031 |
| 36 | 77,895 | 38,586 | 39,309 | 9,909 | 5,043 | 4,866 | 3,932 | 2,052 | 1,880 | 13,842 | 6,879 | 6,963 | 8,075 | 3,937 | 4,138 | 3,783 | 2,062 | 1,721 |
| 37 | 62,949 | 31,267 | 31,682 | 7,973 | 4,045 | 3,928 | 3,166 | 1,675 | 1,491 | 11,330 | 5,685 | 5,645 | 6,264 | 3,169 | 3,095 | 3,070 | 1,690 | 1,380 |
| 38 | 82,468 | 41,472 | 40,996 | 10,052 | 5,115 | 4,937 | 4,226 | 2,315 | 1,911 | 15,080 | 7,523 | 7,557 | 8,639 | 4,425 | 4,214 | 3,841 | 2,086 | 1,755 |
| 39 | 52,796 | 26,472 | 26,324 | 6,609 | 3,394 | 3,215 | 2,766 | 1,479 | 1,287 | 9,557 | 4,845 | 4,712 | 5,502 | 2,762 | 2,740 | 2,499 | 1,360 | 1,139 |
| 40 | 84,527 | 44,692 | 39,835 | 9,818 | 5,363 | 4,455 | 4,252 | 2,418 | 1,834 | 14,998 | 7,901 | 7,097 | 9,847 | 5,393 | 4,454 | 3,719 | 2,093 | 1,626 |
| 41 | 44,657 | 22,469 | 22,188 | 5,582 | 2,933 | 2,649 | 2,343 | 1,282 | 1,061 | 7,620 | 3,793 | 3,827 | 4,652 | 2,387 | 2,265 | 2,022 | 1,180 | 842 |
| 42 | 68,007 | 35,186 | 32,821 | 8,286 | 4,416 | 3,870 | 3,394 | 1,877 | 1,517 | 11,947 | 6,206 | 5,741 | 7,454 | 3,922 | 3,532 | 3,117 | 1,772 | 1,345 |
| 43 | 37,495 | 19,052 | 18,443 | 4,682 | 2,448 | 2,234 | 2,001 | 1,061 | 940 | 6,512 | 3,225 | 3,287 | 4,087 | 2,173 | 1,914 | 1,714 | 966 | 748 |
| 44 | 37,835 | 17,954 | 19,881 | 5,034 | 2,444 | 2,590 | 1,859 | 968 | 891 | 6,378 | 3,041 | 3,337 | 3,992 | 1,918 | 2,074 | 1,673 | 882 | 791 |
| 45 | 55,016 | 27,860 | 27,156 | 6,437 | 3,261 | 3,176 | 2,839 | 1,485 | 1,354 | 9,586 | 4,917 | 4,669 | 6,475 | 3,434 | 3,041 | 2,341 | 1,279 | 1,062 |
| 46 | 55,290 | 27,119 | 28,171 | 6,783 | 3,424 | 3,359 | 2,838 | 1,488 | 1,350 | 9,717 | 4,696 | 5,021 | 5,703 | 2,826 | 2,877 | 2,503 | 1,348 | 1,155 |
| 47 | 35,287 | 17,306 | 17,981 | 4,256 | 2,142 | 2,114 | 1,897 | 951 | 946 | 6,314 | 3,130 | 3,184 | 3,453 | 1,754 | 1,699 | 1,522 | 823 | 699 |
| 48 | 47,259 | 23,178 | 24,081 | 5,740 | 2,782 | 2,958 | 2,417 | 1,190 | 1,227 | 8,262 | 4,198 | 4,064 | 5,302 | 2,772 | 2,530 | 1,946 | 987 | 959 |
| 49 | 31,948 | 15,306 | 16,642 | 3,792 | 1,911 | 1,881 | 1,648 | 799 | 849 | 5,985 | 2,791 | 3,194 | 3,160 | 1,564 | 1,596 | 1,400 | 694 | 706 |
| 50 | 50,863 | 24,740 | 26,123 | 5,521 | 2,694 | 2,827 | 2,686 | 1,323 | 1,363 | 9,472 | 4,648 | 4,824 | 5,852 | 3,008 | 2,844 | 2,044 | 1,053 | 991 |
| 51 | 27,007 | 12,685 | 14,322 | 3,549 | 1,713 | 1,836 | 1,452 | 689 | 763 | 4,436 | 2,123 | 2,313 | 2,829 | 1,367 | 1,462 | 1,157 | 577 | 580 |
| 52 | 39,312 | 18,914 | 20,398 | 4,832 | 2,342 | 2,490 | 2,194 | 1,071 | 1,123 | 6,412 | 3,037 | 3,375 | 4,369 | 2,258 | 2,111 | 1,749 | 883 | 866 |
| 53 | 27,783 | 13,358 | 14,425 | 3,504 | 1,705 | 1,799 | 1,541 | 773 | 768 | 4,827 | 2,216 | 2,611 | 2,783 | 1,379 | 1,404 | 1,376 | 719 | 657 |

## Table A4: Population (De Jure) by Single Year Age and Sex by Province, Rural/Urban Zambia 2010

| Single Year Ages | Zambia Rural | | | Central | | | Copperbelt | | | Eastern | | | Luapula | | | Lusaka | | |
|---|---|---|---|---|---|---|---|---|---|---|---|---|---|---|---|---|---|---|
| | Total | Male | Female | Total | Male | Female | Total | Male | Female | Total | Male | Female | Total | Male | Female | Total | Male | Female |
| 54 | 28,108 | 13,217 | 14,891 | 3,438 | 1,700 | 1,738 | 1,650 | 777 | 873 | 4,786 | 2,144 | 2,642 | 3,056 | 1,532 | 1,524 | 1,283 | 628 | 655 |
| 55 | 27,969 | 13,611 | 14,358 | 3,281 | 1,616 | 1,665 | 1,681 | 807 | 874 | 5,097 | 2,464 | 2,633 | 2,997 | 1,524 | 1,473 | 1,170 | 610 | 560 |
| 56 | 27,589 | 13,423 | 14,166 | 3,388 | 1,701 | 1,687 | 1,773 | 920 | 853 | 4,667 | 2,269 | 2,398 | 2,991 | 1,444 | 1,547 | 1,157 | 620 | 537 |
| 57 | 19,981 | 9,625 | 10,356 | 2,548 | 1,261 | 1,287 | 1,220 | 619 | 601 | 3,648 | 1,756 | 1,892 | 2,103 | 985 | 1,118 | 793 | 415 | 378 |
| 58 | 26,622 | 12,609 | 14,013 | 3,163 | 1,535 | 1,628 | 1,693 | 818 | 875 | 4,635 | 2,179 | 2,456 | 3,052 | 1,496 | 1,556 | 1,080 | 545 | 535 |
| 59 | 16,545 | 7,660 | 8,885 | 1,858 | 916 | 942 | 1,025 | 528 | 497 | 3,227 | 1,442 | 1,785 | 1,755 | 823 | 932 | 651 | 312 | 339 |
| 60 | 32,397 | 14,620 | 17,777 | 3,481 | 1,693 | 1,788 | 1,857 | 914 | 943 | 6,642 | 2,872 | 3,770 | 3,584 | 1,732 | 1,852 | 1,191 | 585 | 606 |
| 61 | 18,518 | 7,804 | 10,714 | 2,545 | 1,127 | 1,418 | 1,137 | 551 | 586 | 3,396 | 1,374 | 2,022 | 1,960 | 826 | 1,134 | 735 | 359 | 376 |
| 62 | 24,456 | 10,484 | 13,972 | 2,879 | 1,338 | 1,541 | 1,583 | 757 | 826 | 4,336 | 1,740 | 2,596 | 2,563 | 1,157 | 1,406 | 995 | 491 | 504 |
| 63 | 19,666 | 8,954 | 10,712 | 2,545 | 1,189 | 1,356 | 1,263 | 638 | 625 | 3,427 | 1,402 | 2,025 | 1,942 | 958 | 984 | 809 | 413 | 396 |
| 64 | 17,862 | 7,573 | 10,289 | 2,096 | 985 | 1,111 | 1,041 | 507 | 534 | 3,315 | 1,317 | 1,998 | 1,679 | 741 | 938 | 734 | 341 | 393 |
| 65 | 23,522 | 10,325 | 13,197 | 2,716 | 1,275 | 1,441 | 1,546 | 770 | 776 | 4,520 | 1,841 | 2,679 | 2,323 | 1,103 | 1,220 | 931 | 434 | 497 |
| 66 | 14,530 | 5,955 | 8,575 | 1,686 | 745 | 941 | 822 | 403 | 419 | 2,806 | 1,069 | 1,737 | 1,349 | 606 | 743 | 561 | 252 | 309 |
| 67 | 13,988 | 6,389 | 7,599 | 1,648 | 829 | 819 | 846 | 445 | 401 | 2,686 | 1,144 | 1,542 | 1,257 | 564 | 693 | 582 | 295 | 287 |
| 68 | 22,968 | 10,612 | 12,356 | 2,766 | 1,317 | 1,449 | 1,354 | 707 | 647 | 3,817 | 1,637 | 2,180 | 2,318 | 1,111 | 1,207 | 881 | 440 | 441 |
| 69 | 12,371 | 5,896 | 6,475 | 1,437 | 742 | 695 | 620 | 357 | 263 | 2,416 | 1,067 | 1,349 | 1,139 | 545 | 594 | 516 | 289 | 227 |
| 70 | 23,059 | 10,646 | 12,413 | 2,307 | 1,200 | 1,107 | 1,304 | 705 | 599 | 4,747 | 1,957 | 2,790 | 2,171 | 1,009 | 1,162 | 834 | 388 | 446 |
| 71 | 11,047 | 5,089 | 5,958 | 1,298 | 652 | 646 | 706 | 377 | 329 | 2,148 | 896 | 1,252 | 935 | 452 | 483 | 421 | 197 | 224 |
| 72 | 14,220 | 6,606 | 7,614 | 1,689 | 850 | 839 | 878 | 502 | 376 | 2,768 | 1,153 | 1,615 | 1,251 | 628 | 623 | 577 | 245 | 332 |
| 73 | 9,996 | 4,891 | 5,105 | 1,191 | 635 | 556 | 601 | 358 | 243 | 1,768 | 725 | 1,043 | 857 | 457 | 400 | 396 | 208 | 188 |
| 74 | 10,819 | 5,114 | 5,705 | 1,339 | 653 | 686 | 552 | 303 | 249 | 1,911 | 802 | 1,109 | 945 | 507 | 438 | 409 | 209 | 200 |
| 75 | 13,827 | 6,700 | 7,127 | 1,589 | 796 | 793 | 874 | 508 | 366 | 2,642 | 1,141 | 1,501 | 1,168 | 638 | 530 | 519 | 240 | 279 |
| 76 | 8,784 | 4,148 | 4,636 | 1,050 | 533 | 517 | 465 | 250 | 215 | 1,642 | 716 | 926 | 737 | 363 | 374 | 349 | 161 | 188 |
| 77 | 6,386 | 3,298 | 3,088 | 801 | 430 | 371 | 353 | 214 | 139 | 1,284 | 616 | 668 | 439 | 226 | 213 | 255 | 127 | 128 |
| 78 | 12,199 | 6,859 | 5,340 | 1,531 | 871 | 660 | 672 | 435 | 237 | 2,267 | 1,160 | 1,107 | 1,014 | 643 | 371 | 435 | 240 | 195 |
| 79 | 6,276 | 3,601 | 2,675 | 735 | 434 | 301 | 331 | 214 | 117 | 1,172 | 604 | 568 | 449 | 289 | 160 | 235 | 123 | 112 |
| 80 | 9,949 | 4,644 | 5,305 | 1,062 | 542 | 520 | 531 | 284 | 247 | 2,032 | 851 | 1,181 | 702 | 329 | 373 | 353 | 162 | 191 |
| 81 | 3,941 | 2,070 | 1,871 | 401 | 243 | 158 | 216 | 125 | 91 | 780 | 353 | 427 | 290 | 171 | 119 | 145 | 82 | 63 |
| 82 | 5,215 | 2,731 | 2,484 | 587 | 324 | 263 | 229 | 142 | 87 | 1,073 | 476 | 597 | 374 | 226 | 148 | 204 | 106 | 98 |
| 83 | 3,176 | 1,804 | 1,372 | 376 | 237 | 139 | 119 | 81 | 38 | 615 | 283 | 332 | 221 | 142 | 79 | 118 | 65 | 53 |
| 84 | 2,968 | 1,532 | 1,436 | 301 | 169 | 132 | 138 | 79 | 59 | 633 | 289 | 344 | 221 | 119 | 102 | 127 | 67 | 60 |
| 85 | 4,292 | 2,048 | 2,244 | 462 | 258 | 204 | 223 | 126 | 97 | 935 | 376 | 559 | 295 | 148 | 147 | 168 | 61 | 107 |
| 86 | 3,084 | 1,594 | 1,490 | 405 | 207 | 198 | 135 | 80 | 55 | 599 | 284 | 315 | 189 | 115 | 74 | 125 | 62 | 63 |
| 87 | 1,849 | 946 | 903 | 191 | 109 | 82 | 77 | 42 | 35 | 385 | 172 | 213 | 116 | 61 | 55 | 76 | 47 | 29 |
| 88 | 3,049 | 1,764 | 1,285 | 382 | 244 | 138 | 127 | 80 | 47 | 625 | 341 | 284 | 252 | 155 | 97 | 88 | 50 | 38 |
| 89 | 2,082 | 1,181 | 901 | 235 | 141 | 94 | 91 | 54 | 37 | 430 | 243 | 187 | 110 | 60 | 50 | 80 | 38 | 42 |
| 90 | 2,319 | 1,199 | 1,120 | 230 | 143 | 87 | 115 | 73 | 42 | 494 | 221 | 273 | 137 | 69 | 68 | 87 | 49 | 38 |
| 91 | 768 | 436 | 332 | 79 | 49 | 30 | 38 | 23 | 15 | 167 | 85 | 82 | 37 | 25 | 12 | 34 | 18 | 16 |
| 92 | 899 | 476 | 423 | 97 | 55 | 42 | 27 | 13 | 14 | 190 | 90 | 100 | 56 | 35 | 21 | 39 | 17 | 22 |
| 93 | 408 | 211 | 197 | 38 | 21 | 17 | 17 | 8 | 9 | 83 | 33 | 50 | 21 | 11 | 10 | 19 | 14 | 5 |
| 94 | 579 | 271 | 308 | 54 | 28 | 26 | 15 | 9 | 6 | 90 | 23 | 67 | 23 | 12 | 11 | 21 | 11 | 10 |
| 95+ | 6,236 | 2,337 | 3,899 | 628 | 236 | 392 | 232 | 90 | 142 | 1,290 | 475 | 815 | 253 | 87 | 166 | 271 | 82 | 189 |
| Median Age | 15.6 | 15.2 | 15.9 | 15.6 | 15.5 | 15.7 | 16.6 | 16.6 | 16.5 | 15.8 | 15.4 | 16.2 | 15.5 | 15.2 | 15.9 | 16.9 | 17.2 | 16.6 |
| **Age groups** | | | | | | | | | | | | | | | | | | |
| **Total Rural** | **7,919,216** | **3,906,636** | **4,012,580** | **978,574** | **487,713** | **490,861** | **376,861** | **190,178** | **186,683** | **1,392,338** | **686,577** | **705,761** | **797,407** | **393,615** | **403,792** | **336,318** | **169,604** | **166,714** |
| 0 - 14 | 3,850,318 | 1,931,217 | 1,919,101 | 474,847 | 237,878 | 236,969 | 174,111 | 87,393 | 86,718 | 668,998 | 335,569 | 333,429 | 388,455 | 195,079 | 193,376 | 151,937 | 75,599 | 76,338 |
| 15 - 64 | 3,814,092 | 1,854,046 | 1,960,046 | 474,416 | 234,867 | 239,549 | 188,496 | 94,928 | 93,568 | 674,325 | 329,885 | 344,440 | 387,303 | 187,630 | 199,673 | 174,525 | 89,226 | 85,299 |
| 65+ | 254,806 | 121,373 | 133,433 | 29,311 | 14,968 | 14,343 | 14,254 | 7,857 | 6,397 | 49,015 | 21,123 | 27,892 | 21,649 | 10,906 | 10,743 | 9,856 | 4,779 | 5,077 |
| | | | | | | | | | | | | | | | | | | |
| Overall Dependency Ratio | 107.6 | 110.7 | 104.7 | 106.3 | 107.7 | 104.9 | 99.9 | 100.3 | 99.5 | 106.5 | 108.1 | 104.9 | 105.9 | 109.8 | 102.2 | 92.7 | 90.1 | 95.4 |
| Child Dependency Ratio | 100.9 | 104.2 | 97.9 | 100.1 | 101.3 | 98.9 | 92.4 | 92.1 | 92.7 | 99.2 | 101.7 | 96.8 | 100.3 | 104.0 | 96.8 | 87.1 | 84.7 | 89.5 |
| Aged Dependency Ratio | 6.7 | 6.5 | 6.8 | 6.2 | 6.4 | 6.0 | 7.6 | 8.3 | 6.8 | 7.3 | 6.4 | 8.1 | 5.6 | 5.8 | 5.4 | 5.6 | 5.4 | 6.0 |

## Table A4: Population (De Jure) by Single Year Age and Sex by Province, Rural/Urban Zambia 2010

| Single Year Ages | Muchinga | | | Northern | | | North Western | | | Southern | | | Western | | |
|---|---|---|---|---|---|---|---|---|---|---|---|---|---|---|---|
| | Total | Male | Female | Total | Male | Female | Total | Male | Female | Total | Male | Female | Total | Male | Female |
| **Total Rural** | **590,575** | **290,490** | **300,085** | **903,208** | **447,755** | **455,453** | **563,061** | **277,503** | **285,558** | **1,197,751** | **587,448** | **610,303** | **783,123** | **375,753** | **407,370** |
| 0 | 22,687 | 11,206 | 11,481 | 36,335 | 18,247 | 18,088 | 20,960 | 10,558 | 10,402 | 46,141 | 23,060 | 23,081 | 27,720 | 13,963 | 13,757 |
| 1 | 21,355 | 10,754 | 10,601 | 32,684 | 16,262 | 16,422 | 21,069 | 10,437 | 10,632 | 45,265 | 22,566 | 22,699 | 26,636 | 13,362 | 13,274 |
| 2 | 22,627 | 11,158 | 11,469 | 36,251 | 18,198 | 18,053 | 23,184 | 11,505 | 11,679 | 47,587 | 23,603 | 23,984 | 31,083 | 15,426 | 15,657 |
| 3 | 22,219 | 11,032 | 11,187 | 35,029 | 17,585 | 17,444 | 22,210 | 11,136 | 11,074 | 47,085 | 23,417 | 23,668 | 30,263 | 15,032 | 15,231 |
| 4 | 23,516 | 11,694 | 11,822 | 37,278 | 18,534 | 18,744 | 22,247 | 11,080 | 11,167 | 46,845 | 23,427 | 23,418 | 31,005 | 15,610 | 15,395 |
| 5 | 21,414 | 10,948 | 10,466 | 32,630 | 16,396 | 16,234 | 20,463 | 10,341 | 10,122 | 41,841 | 20,982 | 20,859 | 27,254 | 13,720 | 13,534 |
| 6 | 20,766 | 10,319 | 10,447 | 32,040 | 16,034 | 16,006 | 20,010 | 9,974 | 10,036 | 43,115 | 21,480 | 21,635 | 26,346 | 13,296 | 13,050 |
| 7 | 20,564 | 10,295 | 10,269 | 30,592 | 15,510 | 15,082 | 19,507 | 9,792 | 9,715 | 39,405 | 19,847 | 19,558 | 25,062 | 12,426 | 12,636 |
| 8 | 18,706 | 9,178 | 9,528 | 28,817 | 14,213 | 14,604 | 17,136 | 8,562 | 8,574 | 36,533 | 18,275 | 18,258 | 21,705 | 10,932 | 10,773 |
| 9 | 16,272 | 8,214 | 8,058 | 22,803 | 11,364 | 11,439 | 14,967 | 7,531 | 7,436 | 33,853 | 16,953 | 16,900 | 20,870 | 10,511 | 10,359 |
| 10 | 23,351 | 11,736 | 11,615 | 36,900 | 18,641 | 18,259 | 22,312 | 11,331 | 10,981 | 45,462 | 22,819 | 22,643 | 28,574 | 14,409 | 14,165 |
| 11 | 13,385 | 6,803 | 6,582 | 20,394 | 10,316 | 10,078 | 14,325 | 7,198 | 7,127 | 29,717 | 15,076 | 14,641 | 18,142 | 9,153 | 8,989 |
| 12 | 18,428 | 9,527 | 8,901 | 26,600 | 13,791 | 12,809 | 15,485 | 7,871 | 7,614 | 34,938 | 17,673 | 17,265 | 20,259 | 10,157 | 10,102 |
| 13 | 13,194 | 6,626 | 6,568 | 19,512 | 9,878 | 9,634 | 13,826 | 7,044 | 6,782 | 32,539 | 16,532 | 16,007 | 18,466 | 9,313 | 9,153 |
| 14 | 13,878 | 7,131 | 6,747 | 20,142 | 10,319 | 9,823 | 14,262 | 7,189 | 7,073 | 27,622 | 14,013 | 13,609 | 18,305 | 9,208 | 9,097 |
| 15 | 15,165 | 7,843 | 7,322 | 21,618 | 11,127 | 10,491 | 13,973 | 7,017 | 6,956 | 32,296 | 16,673 | 15,623 | 17,980 | 9,051 | 8,929 |
| 16 | 12,931 | 6,547 | 6,384 | 19,033 | 9,491 | 9,542 | 13,284 | 6,746 | 6,538 | 29,895 | 15,016 | 14,879 | 17,603 | 8,820 | 8,783 |
| 17 | 11,045 | 5,387 | 5,658 | 17,034 | 8,318 | 8,716 | 11,794 | 5,840 | 5,954 | 24,170 | 12,223 | 11,947 | 15,234 | 7,387 | 7,847 |
| 18 | 13,999 | 6,654 | 7,345 | 21,118 | 10,110 | 11,008 | 13,247 | 6,580 | 6,667 | 29,924 | 15,014 | 14,910 | 17,666 | 8,500 | 9,166 |
| 19 | 10,582 | 4,863 | 5,719 | 15,758 | 7,194 | 8,564 | 10,249 | 5,109 | 5,140 | 22,896 | 11,500 | 11,396 | 14,578 | 6,862 | 7,716 |
| 20 | 13,134 | 5,765 | 7,369 | 20,235 | 8,865 | 11,370 | 10,370 | 5,076 | 5,294 | 24,942 | 12,021 | 12,921 | 15,668 | 7,196 | 8,472 |
| 21 | 8,045 | 3,705 | 4,340 | 11,501 | 5,336 | 6,165 | 8,089 | 3,828 | 4,261 | 19,808 | 9,555 | 10,253 | 12,176 | 5,456 | 6,720 |
| 22 | 9,704 | 4,358 | 5,346 | 14,948 | 6,788 | 8,160 | 9,130 | 4,158 | 4,972 | 19,569 | 9,321 | 10,248 | 13,328 | 5,781 | 7,547 |
| 23 | 8,472 | 3,955 | 4,517 | 12,692 | 5,864 | 6,828 | 8,390 | 3,965 | 4,425 | 19,388 | 9,323 | 10,065 | 11,996 | 5,237 | 6,759 |
| 24 | 8,362 | 3,807 | 4,555 | 12,660 | 5,546 | 7,114 | 8,027 | 3,625 | 4,402 | 17,896 | 8,381 | 9,515 | 11,803 | 5,229 | 6,574 |
| 25 | 11,022 | 5,267 | 5,755 | 17,431 | 8,286 | 9,145 | 8,879 | 4,237 | 4,642 | 18,790 | 8,958 | 9,832 | 12,912 | 5,784 | 7,128 |
| 26 | 8,048 | 3,637 | 4,411 | 12,904 | 5,865 | 7,039 | 7,845 | 3,524 | 4,321 | 17,177 | 7,917 | 9,260 | 11,579 | 5,176 | 6,403 |
| 27 | 7,341 | 3,431 | 3,910 | 11,329 | 5,341 | 5,988 | 7,672 | 3,397 | 4,275 | 15,093 | 7,106 | 7,987 | 10,539 | 4,752 | 5,787 |
| 28 | 9,403 | 4,232 | 5,171 | 14,720 | 6,857 | 7,863 | 8,747 | 4,011 | 4,736 | 17,960 | 8,220 | 9,740 | 12,399 | 5,496 | 6,903 |
| 29 | 6,178 | 2,866 | 3,312 | 9,191 | 4,447 | 4,744 | 5,909 | 2,793 | 3,116 | 13,440 | 6,210 | 7,230 | 9,076 | 4,187 | 4,889 |
| 30 | 10,656 | 5,470 | 5,186 | 16,721 | 8,677 | 8,044 | 9,034 | 4,346 | 4,688 | 18,220 | 8,615 | 9,605 | 12,871 | 5,982 | 6,889 |
| 31 | 4,461 | 2,240 | 2,221 | 6,778 | 3,366 | 3,412 | 5,133 | 2,514 | 2,619 | 11,699 | 5,824 | 5,875 | 7,418 | 3,515 | 3,903 |
| 32 | 7,058 | 3,494 | 3,564 | 10,560 | 5,377 | 5,183 | 6,175 | 2,980 | 3,195 | 14,350 | 6,908 | 7,442 | 9,225 | 4,262 | 4,963 |
| 33 | 4,423 | 2,157 | 2,266 | 6,747 | 3,359 | 3,388 | 5,010 | 2,510 | 2,500 | 11,003 | 5,346 | 5,657 | 7,302 | 3,437 | 3,865 |
| 34 | 5,102 | 2,520 | 2,582 | 7,743 | 3,849 | 3,894 | 5,181 | 2,606 | 2,575 | 12,143 | 5,868 | 6,275 | 7,722 | 3,745 | 3,977 |
| 35 | 7,738 | 3,997 | 3,741 | 12,152 | 6,491 | 5,661 | 6,510 | 3,322 | 3,188 | 14,032 | 7,152 | 6,880 | 9,364 | 4,595 | 4,769 |
| 36 | 5,422 | 2,697 | 2,725 | 8,636 | 4,180 | 4,456 | 5,077 | 2,463 | 2,614 | 11,708 | 5,740 | 5,968 | 7,511 | 3,533 | 3,978 |
| 37 | 4,154 | 2,039 | 2,115 | 6,742 | 3,317 | 3,425 | 4,530 | 2,157 | 2,373 | 9,469 | 4,627 | 4,842 | 6,251 | 2,863 | 3,388 |
| 38 | 6,239 | 3,031 | 3,208 | 9,645 | 4,931 | 4,714 | 5,673 | 2,806 | 2,867 | 11,492 | 5,629 | 5,863 | 7,581 | 3,611 | 3,970 |
| 39 | 3,711 | 1,855 | 1,856 | 5,800 | 2,969 | 2,831 | 3,789 | 1,799 | 1,990 | 7,402 | 3,622 | 3,780 | 5,161 | 2,387 | 2,774 |
| 40 | 6,764 | 3,575 | 3,189 | 10,356 | 5,571 | 4,785 | 5,923 | 3,069 | 2,854 | 10,844 | 5,350 | 5,494 | 8,006 | 3,959 | 4,047 |
| 41 | 3,226 | 1,595 | 1,631 | 4,717 | 2,364 | 2,353 | 3,541 | 1,728 | 1,813 | 6,780 | 3,247 | 3,533 | 4,174 | 1,960 | 2,214 |
| 42 | 4,922 | 2,545 | 2,377 | 7,739 | 4,143 | 3,596 | 4,891 | 2,472 | 2,419 | 9,510 | 4,714 | 4,796 | 6,747 | 3,119 | 3,628 |
| 43 | 2,479 | 1,263 | 1,216 | 4,370 | 2,247 | 2,123 | 2,288 | 1,161 | 1,127 | 5,441 | 2,647 | 2,794 | 3,921 | 1,861 | 2,060 |
| 44 | 2,638 | 1,237 | 1,401 | 4,054 | 1,978 | 2,076 | 2,494 | 1,157 | 1,337 | 6,025 | 2,707 | 3,318 | 3,688 | 1,622 | 2,066 |
| 45 | 4,547 | 2,345 | 2,202 | 6,998 | 3,679 | 3,319 | 3,492 | 1,740 | 1,752 | 7,091 | 3,361 | 3,730 | 5,210 | 2,359 | 2,851 |
| 46 | 4,241 | 2,085 | 2,156 | 6,405 | 3,154 | 3,251 | 4,074 | 1,947 | 2,127 | 7,964 | 3,862 | 4,102 | 5,062 | 2,289 | 2,773 |
| 47 | 2,459 | 1,211 | 1,248 | 3,963 | 1,976 | 1,987 | 2,648 | 1,335 | 1,313 | 5,029 | 2,341 | 2,688 | 3,746 | 1,643 | 2,103 |
| 48 | 3,402 | 1,667 | 1,735 | 5,578 | 2,843 | 2,735 | 3,154 | 1,568 | 1,586 | 6,543 | 3,029 | 3,514 | 4,915 | 2,142 | 2,773 |
| 49 | 2,275 | 1,081 | 1,194 | 3,821 | 1,904 | 1,917 | 2,129 | 1,024 | 1,105 | 4,260 | 2,003 | 2,257 | 3,478 | 1,535 | 1,943 |
| 50 | 4,036 | 2,003 | 2,033 | 6,462 | 3,297 | 3,165 | 3,171 | 1,502 | 1,669 | 6,120 | 2,809 | 3,311 | 5,499 | 2,403 | 3,096 |
| 51 | 1,920 | 902 | 1,018 | 2,987 | 1,484 | 1,503 | 1,909 | 914 | 995 | 3,816 | 1,704 | 2,112 | 2,952 | 1,212 | 1,740 |
| 52 | 2,857 | 1,394 | 1,463 | 4,333 | 2,214 | 2,119 | 2,585 | 1,273 | 1,312 | 5,696 | 2,607 | 3,089 | 4,285 | 1,835 | 2,450 |
| 53 | 1,877 | 939 | 938 | 3,041 | 1,583 | 1,458 | 1,819 | 895 | 924 | 3,707 | 1,736 | 1,971 | 3,308 | 1,413 | 1,895 |

## Table A4: Population (De Jure) by Single Years Age and Sex by Province, Rural/Urban Zambia 2010

| Single Year Ages | Muchinga | | | Northern | | | North Western | | | Southern | | | Western | | |
|---|---|---|---|---|---|---|---|---|---|---|---|---|---|---|---|
| | Total | Male | Female | Total | Male | Female | Total | Male | Female | Total | Male | Female | Total | Male | Female |
| 54 | 1,913 | 921 | 992 | 3,132 | 1,578 | 1,554 | 1,802 | 809 | 993 | 4,091 | 1,848 | 2,243 | 2,957 | 1,280 | 1,677 |
| 55 | 2,077 | 1,051 | 1,026 | 3,399 | 1,750 | 1,649 | 1,871 | 890 | 981 | 3,567 | 1,690 | 1,877 | 2,829 | 1,209 | 1,620 |
| 56 | 1,987 | 991 | 996 | 3,044 | 1,540 | 1,504 | 1,847 | 893 | 954 | 3,748 | 1,733 | 2,015 | 2,987 | 1,312 | 1,675 |
| 57 | 1,341 | 645 | 696 | 2,140 | 1,078 | 1,062 | 1,326 | 646 | 680 | 2,499 | 1,200 | 1,299 | 2,363 | 1,020 | 1,343 |
| 58 | 1,803 | 840 | 963 | 3,172 | 1,563 | 1,609 | 1,813 | 868 | 945 | 3,232 | 1,477 | 1,755 | 2,979 | 1,288 | 1,691 |
| 59 | 1,178 | 560 | 618 | 1,952 | 888 | 1,064 | 1,192 | 558 | 634 | 1,824 | 863 | 961 | 1,883 | 770 | 1,113 |
| 60 | 2,623 | 1,144 | 1,479 | 3,819 | 1,778 | 2,041 | 2,078 | 902 | 1,176 | 3,513 | 1,508 | 2,005 | 3,609 | 1,492 | 2,117 |
| 61 | 1,274 | 507 | 767 | 1,911 | 808 | 1,103 | 1,247 | 547 | 700 | 2,336 | 957 | 1,379 | 1,977 | 748 | 1,229 |
| 62 | 1,739 | 731 | 1,008 | 2,663 | 1,169 | 1,494 | 1,649 | 741 | 908 | 3,025 | 1,203 | 1,822 | 3,024 | 1,157 | 1,867 |
| 63 | 1,531 | 706 | 825 | 2,157 | 1,039 | 1,118 | 1,245 | 597 | 648 | 2,499 | 1,078 | 1,421 | 2,248 | 934 | 1,314 |
| 64 | 1,369 | 599 | 770 | 1,916 | 850 | 1,066 | 1,191 | 483 | 708 | 2,381 | 958 | 1,423 | 2,140 | 792 | 1,348 |
| 65 | 1,895 | 830 | 1,065 | 2,820 | 1,284 | 1,536 | 1,542 | 667 | 875 | 2,804 | 1,139 | 1,665 | 2,425 | 982 | 1,443 |
| 66 | 1,075 | 423 | 652 | 1,506 | 665 | 841 | 972 | 383 | 589 | 1,989 | 746 | 1,243 | 1,764 | 663 | 1,101 |
| 67 | 1,058 | 507 | 551 | 1,572 | 730 | 842 | 854 | 375 | 479 | 1,915 | 814 | 1,101 | 1,570 | 686 | 884 |
| 68 | 1,816 | 820 | 996 | 2,604 | 1,253 | 1,351 | 1,604 | 795 | 809 | 3,109 | 1,326 | 1,783 | 2,699 | 1,206 | 1,493 |
| 69 | 903 | 453 | 450 | 1,463 | 715 | 748 | 891 | 412 | 479 | 1,517 | 644 | 873 | 1,469 | 672 | 797 |
| 70 | 1,830 | 861 | 969 | 2,656 | 1,326 | 1,330 | 1,723 | 791 | 932 | 2,650 | 1,119 | 1,531 | 2,837 | 1,290 | 1,547 |
| 71 | 810 | 372 | 438 | 1,104 | 529 | 575 | 681 | 346 | 335 | 1,599 | 665 | 934 | 1,345 | 603 | 742 |
| 72 | 1,097 | 522 | 575 | 1,459 | 734 | 725 | 949 | 455 | 494 | 1,836 | 757 | 1,079 | 1,716 | 760 | 956 |
| 73 | 792 | 415 | 377 | 1,084 | 587 | 497 | 648 | 319 | 329 | 1,301 | 523 | 778 | 1,358 | 664 | 694 |
| 74 | 860 | 444 | 416 | 1,117 | 597 | 520 | 754 | 376 | 378 | 1,654 | 646 | 1,008 | 1,278 | 577 | 701 |
| 75 | 1,131 | 570 | 561 | 1,519 | 807 | 712 | 1,050 | 493 | 557 | 1,734 | 747 | 987 | 1,601 | 760 | 841 |
| 76 | 734 | 371 | 363 | 920 | 497 | 423 | 616 | 282 | 334 | 1,183 | 500 | 683 | 1,088 | 475 | 613 |
| 77 | 458 | 259 | 199 | 688 | 397 | 291 | 472 | 232 | 240 | 873 | 410 | 463 | 763 | 387 | 376 |
| 78 | 954 | 535 | 419 | 1,248 | 771 | 477 | 855 | 493 | 362 | 1,684 | 866 | 818 | 1,539 | 845 | 694 |
| 79 | 438 | 254 | 184 | 721 | 466 | 255 | 498 | 316 | 182 | 748 | 365 | 383 | 949 | 536 | 413 |
| 80 | 766 | 387 | 379 | 1,070 | 558 | 512 | 834 | 403 | 431 | 1,213 | 494 | 719 | 1,386 | 634 | 752 |
| 81 | 278 | 155 | 123 | 390 | 222 | 168 | 313 | 170 | 143 | 579 | 279 | 300 | 549 | 270 | 279 |
| 82 | 362 | 202 | 160 | 526 | 307 | 219 | 339 | 184 | 155 | 668 | 313 | 355 | 853 | 451 | 402 |
| 83 | 261 | 165 | 96 | 336 | 228 | 108 | 233 | 139 | 94 | 439 | 214 | 225 | 458 | 250 | 208 |
| 84 | 231 | 145 | 86 | 289 | 175 | 114 | 187 | 107 | 80 | 436 | 191 | 245 | 405 | 191 | 214 |
| 85 | 290 | 160 | 130 | 408 | 216 | 192 | 311 | 163 | 148 | 567 | 243 | 324 | 633 | 297 | 336 |
| 86 | 197 | 103 | 94 | 256 | 134 | 122 | 189 | 108 | 81 | 451 | 213 | 238 | 538 | 288 | 250 |
| 87 | 114 | 61 | 53 | 186 | 100 | 86 | 124 | 64 | 60 | 255 | 123 | 132 | 325 | 167 | 158 |
| 88 | 231 | 135 | 96 | 344 | 208 | 136 | 204 | 136 | 68 | 394 | 197 | 197 | 402 | 218 | 184 |
| 89 | 132 | 72 | 60 | 184 | 123 | 61 | 175 | 111 | 64 | 289 | 158 | 131 | 356 | 181 | 175 |
| 90 | 151 | 79 | 72 | 218 | 127 | 91 | 215 | 134 | 81 | 305 | 124 | 181 | 367 | 180 | 187 |
| 91 | 35 | 23 | 12 | 61 | 39 | 22 | 61 | 39 | 22 | 108 | 56 | 52 | 148 | 79 | 69 |
| 92 | 51 | 23 | 28 | 63 | 36 | 27 | 75 | 45 | 30 | 140 | 74 | 66 | 161 | 88 | 73 |
| 93 | 31 | 17 | 14 | 28 | 15 | 13 | 26 | 11 | 15 | 64 | 32 | 32 | 81 | 49 | 32 |
| 94 | 29 | 12 | 17 | 36 | 13 | 23 | 52 | 26 | 26 | 93 | 43 | 50 | 166 | 94 | 72 |
| 95+ | 330 | 140 | 190 | 500 | 179 | 321 | 555 | 251 | 304 | 903 | 303 | 600 | 1,274 | 494 | 780 |
| Median Age | 15.2 | 14.8 | 15.6 | 15.2 | 14.9 | 15.5 | 15.0 | 14.6 | 15.3 | 15 | 14.6 | 15.4 | 16.1 | 15.2 | 17.1 |
| **Age groups** | | | | | | | | | | | | | | | |
| **Total Rural** | **590,575** | **290,490** | **300,085** | **903,208** | **447,755** | **455,453** | **563,061** | **277,503** | **285,558** | **1,197,751** | **587,448** | **610,303** | **783,123** | **375,753** | **407,370** |
| 0 - 14 | 292,362 | 146,621 | 145,741 | 448,007 | 225,288 | 222,719 | 281,963 | 141,549 | 140,414 | 597,948 | 299,723 | 298,225 | 371,690 | 186,518 | 185,172 |
| 15 - 64 | 278,873 | 134,354 | 144,519 | 427,825 | 208,429 | 219,396 | 263,096 | 127,128 | 135,968 | 566,303 | 273,401 | 292,902 | 378,930 | 174,198 | 204,732 |
| 65+ | 19,340 | 9,515 | 9,825 | 27,376 | 14,038 | 13,338 | 18,002 | 8,826 | 9,176 | 33,500 | 14,324 | 19,176 | 32,503 | 15,037 | 17,466 |
| | | | | | | | | | | | | | | | |
| Overall Dependency Ratio | 111.8 | 116.2 | 107.6 | 111.1 | 114.8 | 107.6 | 114.0 | 118.3 | 110.0 | 111.5 | 114.9 | 108.4 | 106.7 | 115.7 | 99.0 |
| Child Dependency Ratio | 104.8 | 109.1 | 100.8 | 104.7 | 108.1 | 101.5 | 107.2 | 111.3 | 103.3 | 105.6 | 109.6 | 101.8 | 98.1 | 107.1 | 90.4 |
| Aged Dependency Ratio | 6.9 | 7.1 | 6.8 | 6.4 | 6.7 | 6.1 | 6.8 | 6.9 | 6.7 | 5.9 | 5.2 | 6.5 | 8.6 | 8.6 | 8.5 |

## Table A4: Population (De Jure) by Single Years Age and Sex by Province, Rural/Urban Zambia 2010

| Single Year Ages | Zambia Urban | | | Central | | | Copperbelt | | | Eastern | | | Luapula | | | Lusaka | | |
|---|---|---|---|---|---|---|---|---|---|---|---|---|---|---|---|---|---|---|
| | Total | Male | Female | Total | Male | Female | Total | Male | Female | Total | Male | Female | Total | Male | Female | Total | Male | Female |
| **Total Urban** | **5,173,450** | **2,548,011** | **2,625,439** | **328,537** | **160,752** | **167,785** | **1,595,456** | **791,709** | **803,747** | **200,323** | **98,103** | **102,220** | **194,520** | **94,974** | **99,546** | **1,854,907** | **913,394** | **941,513** |
| 0 | 148,056 | 74,090 | 73,966 | 9,128 | 4,510 | 4,618 | 42,176 | 21,076 | 21,100 | 5,571 | 2,778 | 2,793 | 5,851 | 2,936 | 2,915 | 54,731 | 27,385 | 27,346 |
| 1 | 157,748 | 78,539 | 79,209 | 9,552 | 4,760 | 4,792 | 47,031 | 23,396 | 23,635 | 6,360 | 3,126 | 3,234 | 6,061 | 3,019 | 3,042 | 56,952 | 28,279 | 28,673 |
| 2 | 152,635 | 76,501 | 76,134 | 9,457 | 4,775 | 4,682 | 44,909 | 22,556 | 22,353 | 6,226 | 3,122 | 3,104 | 6,434 | 3,177 | 3,257 | 54,696 | 27,455 | 27,241 |
| 3 | 152,981 | 75,210 | 77,771 | 9,600 | 4,774 | 4,826 | 44,977 | 22,049 | 22,928 | 6,166 | 3,035 | 3,131 | 6,353 | 3,146 | 3,207 | 54,636 | 26,841 | 27,795 |
| 4 | 153,172 | 76,124 | 77,048 | 9,674 | 4,805 | 4,869 | 45,743 | 22,909 | 22,834 | 6,224 | 3,117 | 3,107 | 6,421 | 3,170 | 3,251 | 53,617 | 26,641 | 26,976 |
| 5 | 140,934 | 70,127 | 70,807 | 8,849 | 4,397 | 4,452 | 41,484 | 20,743 | 20,741 | 5,917 | 2,938 | 2,979 | 5,859 | 2,933 | 2,926 | 49,773 | 24,820 | 24,953 |
| 6 | 136,222 | 67,090 | 69,132 | 8,586 | 4,267 | 4,319 | 40,540 | 20,009 | 20,531 | 5,694 | 2,837 | 2,857 | 5,776 | 2,818 | 2,958 | 47,786 | 23,499 | 24,287 |
| 7 | 137,754 | 68,481 | 69,273 | 8,886 | 4,383 | 4,503 | 40,544 | 20,222 | 20,322 | 5,815 | 2,835 | 2,980 | 5,917 | 2,904 | 3,013 | 48,031 | 23,897 | 24,134 |
| 8 | 124,558 | 60,233 | 64,325 | 8,108 | 3,908 | 4,200 | 37,259 | 18,015 | 19,244 | 5,114 | 2,506 | 2,608 | 5,372 | 2,632 | 2,740 | 43,708 | 21,038 | 22,670 |
| 9 | 116,702 | 56,337 | 60,365 | 7,694 | 3,737 | 3,957 | 35,597 | 17,251 | 18,346 | 4,531 | 2,170 | 2,361 | 4,802 | 2,351 | 2,451 | 40,587 | 19,509 | 21,078 |
| 10 | 161,551 | 78,759 | 82,792 | 10,668 | 5,160 | 5,508 | 49,352 | 24,217 | 25,135 | 6,805 | 3,268 | 3,537 | 7,554 | 3,747 | 3,807 | 53,998 | 26,174 | 27,824 |
| 11 | 113,129 | 54,089 | 59,040 | 7,330 | 3,513 | 3,817 | 35,620 | 17,192 | 18,428 | 4,585 | 2,175 | 2,410 | 4,531 | 2,217 | 2,314 | 39,311 | 18,660 | 20,651 |
| 12 | 138,157 | 65,974 | 72,183 | 9,449 | 4,545 | 4,904 | 42,901 | 20,820 | 22,081 | 5,549 | 2,628 | 2,921 | 5,814 | 2,799 | 3,015 | 47,156 | 22,122 | 25,034 |
| 13 | 128,206 | 60,158 | 68,048 | 8,440 | 3,947 | 4,493 | 41,711 | 19,908 | 21,803 | 5,015 | 2,283 | 2,732 | 4,676 | 2,322 | 2,354 | 44,112 | 20,284 | 23,828 |
| 14 | 131,046 | 61,443 | 69,603 | 8,876 | 4,162 | 4,714 | 42,653 | 20,324 | 22,329 | 4,997 | 2,362 | 2,635 | 4,980 | 2,469 | 2,511 | 44,531 | 20,418 | 24,113 |
| 15 | 134,075 | 63,473 | 70,602 | 9,169 | 4,377 | 4,792 | 43,194 | 20,661 | 22,533 | 5,308 | 2,552 | 2,756 | 5,148 | 2,538 | 2,610 | 44,556 | 20,725 | 23,831 |
| 16 | 132,481 | 61,653 | 70,828 | 8,981 | 4,142 | 4,839 | 43,488 | 20,361 | 23,127 | 5,166 | 2,443 | 2,723 | 4,931 | 2,431 | 2,500 | 44,015 | 20,138 | 23,877 |
| 17 | 123,997 | 59,029 | 64,968 | 8,643 | 4,071 | 4,572 | 40,995 | 19,735 | 21,260 | 4,573 | 2,216 | 2,357 | 4,506 | 2,201 | 2,305 | 41,402 | 19,366 | 22,036 |
| 18 | 143,978 | 69,410 | 74,568 | 10,126 | 4,912 | 5,214 | 46,398 | 22,661 | 23,737 | 5,663 | 2,834 | 2,829 | 5,601 | 2,752 | 2,849 | 46,671 | 21,818 | 24,853 |
| 19 | 121,530 | 58,357 | 63,173 | 8,467 | 4,164 | 4,303 | 37,867 | 18,586 | 19,281 | 4,745 | 2,311 | 2,434 | 4,250 | 2,082 | 2,168 | 42,069 | 19,454 | 22,615 |
| 20 | 130,741 | 61,262 | 69,479 | 8,789 | 4,306 | 4,483 | 39,388 | 18,775 | 20,613 | 5,191 | 2,487 | 2,704 | 5,126 | 2,482 | 2,644 | 45,704 | 20,637 | 25,067 |
| 21 | 99,450 | 46,107 | 53,343 | 6,309 | 2,960 | 3,349 | 30,006 | 14,328 | 15,678 | 3,691 | 1,729 | 1,962 | 3,120 | 1,456 | 1,664 | 36,873 | 16,609 | 20,264 |
| 22 | 109,117 | 49,806 | 59,311 | 6,827 | 3,153 | 3,674 | 32,576 | 15,271 | 17,305 | 4,209 | 1,871 | 2,338 | 3,649 | 1,748 | 1,901 | 40,592 | 18,037 | 22,555 |
| 23 | 104,325 | 48,159 | 56,166 | 6,132 | 2,895 | 3,237 | 31,008 | 14,728 | 16,280 | 3,912 | 1,730 | 2,182 | 3,166 | 1,467 | 1,699 | 40,257 | 18,220 | 22,037 |
| 24 | 101,007 | 46,209 | 54,798 | 5,720 | 2,639 | 3,081 | 30,290 | 14,269 | 16,021 | 3,867 | 1,683 | 2,184 | 2,973 | 1,292 | 1,681 | 39,395 | 17,984 | 21,411 |
| 25 | 112,467 | 52,892 | 59,575 | 6,625 | 3,027 | 3,598 | 33,056 | 15,834 | 17,222 | 4,429 | 2,034 | 2,395 | 3,729 | 1,715 | 2,014 | 43,125 | 20,421 | 22,704 |
| 26 | 99,625 | 46,269 | 53,356 | 5,522 | 2,493 | 3,029 | 29,895 | 14,195 | 15,700 | 3,785 | 1,690 | 2,095 | 2,854 | 1,209 | 1,645 | 39,216 | 18,317 | 20,899 |
| 27 | 92,200 | 44,364 | 47,836 | 5,092 | 2,362 | 2,730 | 27,615 | 13,569 | 14,046 | 3,425 | 1,642 | 1,783 | 2,800 | 1,208 | 1,592 | 36,158 | 17,635 | 18,523 |
| 28 | 110,009 | 52,589 | 57,420 | 6,195 | 2,827 | 3,368 | 32,373 | 15,845 | 16,528 | 4,111 | 1,850 | 2,261 | 3,380 | 1,464 | 1,916 | 43,481 | 21,084 | 22,397 |
| 29 | 78,412 | 37,712 | 40,700 | 4,418 | 2,003 | 2,415 | 23,272 | 11,425 | 11,847 | 2,911 | 1,363 | 1,548 | 2,251 | 1,067 | 1,184 | 31,447 | 15,080 | 16,367 |
| 30 | 115,003 | 58,402 | 56,601 | 6,671 | 3,253 | 3,418 | 33,070 | 16,858 | 16,212 | 4,553 | 2,329 | 2,224 | 4,073 | 2,004 | 2,069 | 45,075 | 22,903 | 22,172 |
| 31 | 61,359 | 31,665 | 29,694 | 3,521 | 1,763 | 1,758 | 17,769 | 9,357 | 8,412 | 2,242 | 1,148 | 1,094 | 1,685 | 794 | 891 | 24,990 | 12,853 | 12,137 |
| 32 | 90,358 | 46,720 | 43,638 | 5,282 | 2,641 | 2,641 | 26,481 | 13,865 | 12,616 | 3,424 | 1,741 | 1,683 | 2,863 | 1,371 | 1,492 | 36,016 | 18,885 | 17,131 |
| 33 | 60,022 | 30,784 | 29,238 | 3,331 | 1,644 | 1,687 | 17,735 | 9,271 | 8,464 | 2,356 | 1,219 | 1,137 | 1,777 | 873 | 904 | 24,227 | 12,369 | 11,858 |
| 34 | 66,632 | 35,359 | 31,273 | 3,766 | 1,972 | 1,794 | 19,803 | 10,437 | 9,366 | 2,321 | 1,250 | 1,071 | 2,000 | 1,062 | 938 | 27,099 | 14,532 | 12,567 |
| 35 | 83,424 | 46,445 | 36,979 | 4,999 | 2,730 | 2,269 | 24,237 | 13,305 | 10,932 | 3,057 | 1,730 | 1,327 | 2,847 | 1,509 | 1,338 | 32,926 | 18,746 | 14,180 |
| 36 | 63,782 | 34,088 | 29,694 | 3,738 | 1,975 | 1,763 | 18,715 | 9,851 | 8,864 | 2,378 | 1,274 | 1,104 | 2,010 | 992 | 1,018 | 25,664 | 14,012 | 11,652 |
| 37 | 51,857 | 28,084 | 23,773 | 3,073 | 1,654 | 1,419 | 15,473 | 8,139 | 7,334 | 1,931 | 1,057 | 874 | 1,698 | 858 | 840 | 20,727 | 11,623 | 9,104 |
| 38 | 65,220 | 35,566 | 29,654 | 4,030 | 2,169 | 1,861 | 19,423 | 10,261 | 9,162 | 2,500 | 1,371 | 1,129 | 2,097 | 1,116 | 981 | 25,581 | 14,439 | 11,142 |
| 39 | 42,899 | 22,930 | 19,969 | 2,667 | 1,420 | 1,247 | 13,018 | 6,659 | 6,359 | 1,517 | 852 | 665 | 1,368 | 712 | 656 | 16,982 | 9,388 | 7,594 |
| 40 | 64,836 | 37,006 | 27,830 | 4,058 | 2,308 | 1,750 | 19,782 | 10,905 | 8,877 | 2,372 | 1,383 | 989 | 2,405 | 1,330 | 1,075 | 24,551 | 14,501 | 10,050 |
| 41 | 32,834 | 18,257 | 14,577 | 2,012 | 1,096 | 916 | 10,797 | 5,899 | 4,898 | 1,047 | 562 | 485 | 1,006 | 550 | 456 | 12,313 | 7,065 | 5,248 |
| 42 | 48,969 | 27,379 | 21,590 | 3,116 | 1,712 | 1,404 | 15,621 | 8,514 | 7,107 | 1,785 | 972 | 813 | 1,721 | 906 | 815 | 17,958 | 10,409 | 7,549 |
| 43 | 29,129 | 15,470 | 13,659 | 1,833 | 972 | 861 | 9,660 | 5,010 | 4,650 | 926 | 492 | 434 | 972 | 496 | 476 | 10,695 | 5,871 | 4,824 |
| 44 | 24,949 | 12,950 | 11,999 | 1,614 | 800 | 814 | 8,140 | 4,195 | 3,945 | 918 | 463 | 455 | 919 | 474 | 445 | 8,892 | 4,767 | 4,125 |
| 45 | 38,903 | 20,853 | 18,050 | 2,522 | 1,320 | 1,202 | 12,813 | 6,707 | 6,106 | 1,416 | 767 | 649 | 1,587 | 801 | 786 | 13,544 | 7,519 | 6,025 |
| 46 | 39,277 | 20,469 | 18,808 | 2,477 | 1,297 | 1,180 | 13,551 | 6,938 | 6,613 | 1,375 | 686 | 689 | 1,364 | 689 | 675 | 13,774 | 7,416 | 6,358 |
| 47 | 23,861 | 12,421 | 11,440 | 1,573 | 770 | 803 | 8,257 | 4,255 | 4,002 | 793 | 426 | 367 | 906 | 449 | 457 | 7,957 | 4,221 | 3,736 |
| 48 | 29,219 | 14,435 | 14,784 | 1,909 | 900 | 1,009 | 9,858 | 4,682 | 5,176 | 1,103 | 549 | 554 | 1,242 | 587 | 655 | 9,722 | 5,073 | 4,649 |
| 49 | 20,104 | 10,100 | 10,004 | 1,333 | 642 | 691 | 7,020 | 3,531 | 3,489 | 723 | 343 | 380 | 715 | 329 | 386 | 6,657 | 3,402 | 3,255 |
| 50 | 32,467 | 16,371 | 16,096 | 2,303 | 1,117 | 1,186 | 11,289 | 5,622 | 5,667 | 1,169 | 617 | 552 | 1,405 | 676 | 729 | 10,364 | 5,356 | 5,008 |
| 51 | 16,939 | 8,364 | 8,575 | 1,117 | 525 | 592 | 6,058 | 2,972 | 3,086 | 570 | 286 | 284 | 647 | 297 | 350 | 5,500 | 2,756 | 2,744 |
| 52 | 25,270 | 12,386 | 12,884 | 1,730 | 834 | 896 | 9,109 | 4,439 | 4,670 | 898 | 439 | 459 | 1,121 | 527 | 594 | 7,947 | 3,962 | 3,985 |
| 53 | 18,107 | 9,207 | 8,900 | 1,226 | 601 | 625 | 6,651 | 3,424 | 3,227 | 627 | 320 | 307 | 699 | 352 | 347 | 5,835 | 2,972 | 2,863 |

## Table A4: Population (De Jure) by Single Years Age and Sex by Province, Rural/Urban Zambia 2010

| Single Year Ages | Zambia Urban | | | Central | | | Copperbelt | | | Eastern | | | Luapula | | | Lusaka | | |
|---|---|---|---|---|---|---|---|---|---|---|---|---|---|---|---|---|---|---|
| | Total | Male | Female | Total | Male | Female | Total | Male | Female | Total | Male | Female | Total | Male | Female | Total | Male | Female |
| 54 | 19,008 | 9,522 | 9,486 | 1,364 | 672 | 692 | 7,022 | 3,557 | 3,465 | 643 | 299 | 344 | 785 | 361 | 424 | 5,899 | 2,973 | 2,926 |
| 55 | 19,462 | 10,418 | 9,044 | 1,341 | 750 | 591 | 7,089 | 3,834 | 3,255 | 713 | 383 | 330 | 764 | 378 | 386 | 6,203 | 3,291 | 2,912 |
| 56 | 18,217 | 9,644 | 8,573 | 1,224 | 620 | 604 | 6,759 | 3,689 | 3,070 | 600 | 308 | 292 | 746 | 380 | 366 | 5,682 | 3,029 | 2,653 |
| 57 | 12,858 | 6,834 | 6,024 | 947 | 547 | 400 | 4,771 | 2,622 | 2,149 | 423 | 205 | 218 | 513 | 249 | 264 | 3,993 | 2,082 | 1,911 |
| 58 | 15,515 | 7,959 | 7,556 | 1,091 | 550 | 541 | 5,761 | 3,045 | 2,716 | 510 | 261 | 249 | 694 | 309 | 385 | 4,694 | 2,393 | 2,301 |
| 59 | 9,404 | 4,935 | 4,469 | 673 | 361 | 312 | 3,429 | 1,876 | 1,553 | 308 | 151 | 157 | 395 | 175 | 220 | 2,936 | 1,525 | 1,411 |
| 60 | 16,803 | 8,782 | 8,021 | 1,145 | 593 | 552 | 5,958 | 3,203 | 2,755 | 679 | 345 | 334 | 853 | 397 | 456 | 5,118 | 2,696 | 2,422 |
| 61 | 8,892 | 4,535 | 4,357 | 632 | 307 | 325 | 3,368 | 1,803 | 1,565 | 306 | 151 | 155 | 405 | 176 | 229 | 2,591 | 1,317 | 1,274 |
| 62 | 12,337 | 6,461 | 5,876 | 864 | 449 | 415 | 4,648 | 2,591 | 2,057 | 410 | 185 | 225 | 634 | 307 | 327 | 3,633 | 1,858 | 1,775 |
| 63 | 9,445 | 4,993 | 4,452 | 663 | 358 | 305 | 3,518 | 1,970 | 1,548 | 316 | 163 | 153 | 449 | 212 | 237 | 2,818 | 1,491 | 1,327 |
| 64 | 8,187 | 4,095 | 4,092 | 595 | 309 | 286 | 2,941 | 1,530 | 1,411 | 317 | 148 | 169 | 410 | 191 | 219 | 2,459 | 1,258 | 1,201 |
| 65 | 11,193 | 5,694 | 5,499 | 760 | 386 | 374 | 4,127 | 2,155 | 1,972 | 416 | 205 | 211 | 558 | 272 | 286 | 3,248 | 1,692 | 1,556 |
| 66 | 5,702 | 2,630 | 3,072 | 416 | 193 | 223 | 2,068 | 997 | 1,071 | 235 | 106 | 129 | 259 | 108 | 151 | 1,615 | 776 | 839 |
| 67 | 5,918 | 3,029 | 2,889 | 411 | 215 | 196 | 2,174 | 1,169 | 1,005 | 220 | 106 | 114 | 310 | 149 | 161 | 1,668 | 887 | 781 |
| 68 | 8,499 | 4,133 | 4,366 | 587 | 287 | 300 | 3,093 | 1,591 | 1,502 | 329 | 148 | 181 | 500 | 255 | 245 | 2,246 | 1,080 | 1,166 |
| 69 | 4,240 | 2,151 | 2,089 | 328 | 156 | 172 | 1,482 | 793 | 689 | 141 | 57 | 84 | 224 | 115 | 109 | 1,243 | 630 | 613 |
| 70 | 7,977 | 3,816 | 4,161 | 618 | 282 | 336 | 2,729 | 1,328 | 1,401 | 327 | 157 | 170 | 490 | 238 | 252 | 2,193 | 1,048 | 1,145 |
| 71 | 3,742 | 1,791 | 1,951 | 282 | 113 | 169 | 1,409 | 691 | 718 | 146 | 75 | 71 | 189 | 91 | 98 | 997 | 487 | 510 |
| 72 | 5,314 | 2,552 | 2,762 | 396 | 192 | 204 | 1,913 | 952 | 961 | 184 | 84 | 100 | 290 | 130 | 160 | 1,432 | 691 | 741 |
| 73 | 3,437 | 1,664 | 1,773 | 273 | 146 | 127 | 1,189 | 608 | 581 | 128 | 59 | 69 | 188 | 85 | 103 | 913 | 434 | 479 |
| 74 | 3,737 | 1,776 | 1,961 | 267 | 120 | 147 | 1,341 | 644 | 697 | 133 | 75 | 58 | 205 | 97 | 108 | 995 | 486 | 509 |
| 75 | 4,879 | 2,252 | 2,627 | 379 | 160 | 219 | 1,655 | 838 | 817 | 227 | 105 | 122 | 279 | 144 | 135 | 1,290 | 544 | 746 |
| 76 | 2,964 | 1,367 | 1,597 | 213 | 81 | 132 | 1,090 | 532 | 558 | 109 | 46 | 63 | 171 | 84 | 87 | 745 | 335 | 410 |
| 77 | 1,982 | 910 | 1,072 | 154 | 70 | 84 | 710 | 333 | 377 | 79 | 36 | 43 | 115 | 58 | 57 | 513 | 222 | 291 |
| 78 | 3,929 | 1,878 | 2,051 | 279 | 118 | 161 | 1,284 | 657 | 627 | 152 | 73 | 79 | 231 | 103 | 128 | 1,080 | 487 | 593 |
| 79 | 1,837 | 916 | 921 | 136 | 62 | 74 | 585 | 319 | 266 | 71 | 28 | 43 | 99 | 52 | 47 | 482 | 230 | 252 |
| 80 | 3,255 | 1,357 | 1,898 | 261 | 99 | 162 | 1,113 | 481 | 632 | 129 | 43 | 86 | 166 | 78 | 88 | 839 | 336 | 503 |
| 81 | 1,209 | 609 | 600 | 112 | 49 | 63 | 400 | 220 | 180 | 31 | 13 | 18 | 59 | 36 | 23 | 320 | 155 | 165 |
| 82 | 1,727 | 791 | 936 | 137 | 75 | 62 | 536 | 253 | 283 | 80 | 34 | 46 | 93 | 49 | 44 | 473 | 191 | 282 |
| 83 | 1,034 | 514 | 520 | 67 | 29 | 38 | 333 | 167 | 166 | 37 | 21 | 16 | 58 | 34 | 24 | 293 | 140 | 153 |
| 84 | 1,124 | 517 | 607 | 93 | 52 | 41 | 384 | 190 | 194 | 48 | 22 | 26 | 36 | 17 | 19 | 316 | 121 | 195 |
| 85 | 1,421 | 568 | 853 | 97 | 51 | 46 | 469 | 196 | 273 | 71 | 30 | 41 | 77 | 30 | 47 | 419 | 141 | 278 |
| 86 | 1,171 | 516 | 655 | 84 | 33 | 51 | 380 | 180 | 200 | 52 | 18 | 34 | 48 | 22 | 26 | 343 | 140 | 203 |
| 87 | 625 | 255 | 370 | 63 | 25 | 38 | 194 | 83 | 111 | 22 | 7 | 15 | 34 | 13 | 21 | 188 | 73 | 115 |
| 88 | 987 | 498 | 489 | 76 | 37 | 39 | 255 | 124 | 131 | 35 | 16 | 19 | 41 | 19 | 22 | 359 | 180 | 179 |
| 89 | 558 | 281 | 277 | 47 | 24 | 23 | 176 | 102 | 74 | 17 | 6 | 11 | 21 | 11 | 10 | 156 | 67 | 89 |
| 90 | 679 | 289 | 390 | 58 | 29 | 29 | 194 | 95 | 99 | 28 | 7 | 21 | 29 | 11 | 18 | 205 | 78 | 127 |
| 91 | 209 | 116 | 93 | 22 | 11 | 11 | 66 | 40 | 26 | 5 | 4 | 1 | 4 | 2 | 2 | 61 | 35 | 26 |
| 92 | 293 | 133 | 160 | 21 | 7 | 14 | 97 | 45 | 52 | 5 | 2 | 3 | 7 | 4 | 3 | 82 | 40 | 42 |
| 93 | 161 | 57 | 104 | 6 | 1 | 5 | 53 | 20 | 33 | 5 | 4 | 1 | 7 | 3 | 4 | 44 | 10 | 34 |
| 94 | 135 | 57 | 78 | 15 | 8 | 7 | 35 | 16 | 19 | 5 | 3 | 2 | 4 | 2 | 2 | 42 | 14 | 28 |
| 95+ | 1,699 | 559 | 1,140 | 127 | 36 | 91 | 410 | 144 | 266 | 75 | 23 | 52 | 68 | 21 | 47 | 499 | 144 | 355 |
| Median | 18.7 | 19 | 18.5 | 18.3 | 18.4 | 18.2 | 18.8 | 19.1 | 18.6 | 18.1 | 18.2 | 18 | 17.2 | 16.9 | 17.4 | 19.4 | 19.9 | 19 |
| **Age group** | | | | | | | | | | | | | | | | | | |
| **Total Urban** | **5,173,450** | **2,548,011** | **2,625,439** | **328,537** | **160,752** | **167,785** | **1,595,456** | **791,709** | **803,747** | **200,323** | **98,103** | **102,220** | **194,520** | **94,974** | **99,546** | **1,854,907** | **913,394** | **941,513** |
| 0 - 14 | 2,092,851 | 1,023,155 | 1,069,696 | 134,297 | 65,643 | 68,654 | 632,497 | 310,687 | 321,810 | 84,569 | 41,180 | 43,389 | 86,401 | 42,640 | 43,761 | 733,625 | 357,022 | 376,603 |
| 15 - 64 | 2,988,962 | 1,481,180 | 1,507,782 | 187,455 | 91,962 | 95,493 | 931,015 | 465,059 | 465,956 | 112,212 | 55,310 | 56,902 | 103,259 | 50,001 | 53,258 | 1,095,983 | 544,478 | 551,505 |
| 65+ | 91,637 | 43,676 | 47,961 | 6,785 | 3,147 | 3,638 | 31,944 | 15,963 | 15,981 | 3,542 | 1,613 | 1,929 | 4,860 | 2,333 | 2,527 | 25,299 | 11,894 | 13,405 |
| | | | | | | | | | | | | | | | | | | |
| Overall Dependency Ratio | 73.1 | 72.0 | 74.1 | 75.3 | 74.8 | 75.7 | 71.4 | 70.2 | 72.5 | 78.5 | 77.4 | 79.6 | 88.4 | 89.9 | 86.9 | 69.2 | 67.8 | 70.7 |
| Child Dependency Ratio | 70.0 | 69.1 | 70.9 | 71.6 | 71.4 | 71.9 | 67.9 | 66.8 | 69.1 | 75.4 | 74.5 | 76.3 | 83.7 | 85.3 | 82.2 | 66.9 | 65.6 | 68.3 |
| Aged Dependency Ratio | 3.1 | 2.9 | 3.2 | 3.6 | 3.4 | 3.8 | 3.4 | 3.4 | 3.4 | 3.2 | 2.9 | 3.4 | 4.7 | 4.7 | 4.7 | 2.3 | 2.2 | 2.4 |

## Table A4: Population (De Jure) by Single Years Age and Sex by Province, Rural/Urban Zambia 2010

| Single Year Ages | Muchinga | | | Northern | | | North Western | | | Southern | | | Western | | |
|---|---|---|---|---|---|---|---|---|---|---|---|---|---|---|---|
| | Total | Male | Female | Total | Male | Female | Total | Male | Female | Total | Male | Female | Total | Male | Female |
| **Total Urban** | **121,082** | **59,382** | **61,700** | **202,616** | **99,096** | **103,520** | **163,983** | **80,638** | **83,345** | **392,175** | **192,211** | **199,964** | **119,851** | **57,752** | **62,099** |
| 0 | 3,852 | 1,948 | 1,904 | 6,181 | 3,052 | 3,129 | 5,451 | 2,753 | 2,698 | 11,652 | 5,875 | 5,777 | 3,463 | 1,777 | 1,686 |
| 1 | 3,935 | 1,983 | 1,952 | 6,184 | 3,107 | 3,077 | 5,745 | 2,840 | 2,905 | 12,302 | 6,203 | 6,099 | 3,626 | 1,826 | 1,800 |
| 2 | 3,875 | 1,984 | 1,891 | 6,691 | 3,375 | 3,316 | 5,573 | 2,783 | 2,790 | 11,239 | 5,523 | 5,716 | 3,535 | 1,751 | 1,784 |
| 3 | 4,116 | 2,031 | 2,085 | 6,740 | 3,332 | 3,408 | 5,485 | 2,627 | 2,858 | 11,344 | 5,586 | 5,758 | 3,564 | 1,789 | 1,775 |
| 4 | 4,209 | 1,989 | 2,220 | 6,812 | 3,353 | 3,459 | 5,381 | 2,692 | 2,689 | 11,475 | 5,654 | 5,821 | 3,616 | 1,794 | 1,822 |
| 5 | 3,727 | 1,882 | 1,845 | 6,324 | 3,073 | 3,251 | 5,037 | 2,444 | 2,593 | 10,739 | 5,257 | 5,482 | 3,225 | 1,640 | 1,585 |
| 6 | 3,685 | 1,803 | 1,882 | 5,938 | 2,889 | 3,049 | 4,841 | 2,378 | 2,463 | 10,319 | 5,063 | 5,256 | 3,057 | 1,527 | 1,530 |
| 7 | 3,731 | 1,896 | 1,835 | 6,425 | 3,160 | 3,265 | 4,917 | 2,486 | 2,431 | 10,289 | 5,098 | 5,191 | 3,199 | 1,600 | 1,599 |
| 8 | 3,215 | 1,585 | 1,630 | 5,511 | 2,593 | 2,918 | 4,185 | 2,029 | 2,156 | 9,307 | 4,540 | 4,767 | 2,779 | 1,387 | 1,392 |
| 9 | 3,007 | 1,459 | 1,548 | 5,013 | 2,404 | 2,609 | 3,878 | 1,869 | 2,009 | 8,841 | 4,233 | 4,608 | 2,752 | 1,354 | 1,398 |
| 10 | 4,352 | 2,163 | 2,189 | 7,545 | 3,692 | 3,853 | 5,687 | 2,775 | 2,912 | 11,719 | 5,689 | 6,030 | 3,871 | 1,874 | 1,997 |
| 11 | 2,638 | 1,225 | 1,413 | 4,571 | 2,211 | 2,360 | 3,665 | 1,760 | 1,905 | 8,259 | 3,867 | 4,392 | 2,619 | 1,269 | 1,350 |
| 12 | 3,489 | 1,664 | 1,825 | 6,006 | 2,974 | 3,032 | 4,452 | 2,140 | 2,312 | 10,120 | 4,688 | 5,432 | 3,221 | 1,594 | 1,627 |
| 13 | 2,825 | 1,387 | 1,438 | 4,902 | 2,317 | 2,585 | 3,993 | 1,913 | 2,080 | 9,644 | 4,424 | 5,220 | 2,888 | 1,373 | 1,515 |
| 14 | 2,996 | 1,413 | 1,583 | 5,276 | 2,539 | 2,737 | 4,241 | 1,981 | 2,260 | 9,372 | 4,305 | 5,067 | 3,124 | 1,470 | 1,654 |
| 15 | 3,271 | 1,592 | 1,679 | 5,604 | 2,777 | 2,827 | 4,250 | 1,999 | 2,251 | 10,365 | 4,766 | 5,599 | 3,210 | 1,486 | 1,724 |
| 16 | 2,985 | 1,455 | 1,530 | 5,203 | 2,472 | 2,731 | 4,142 | 1,962 | 2,180 | 10,270 | 4,739 | 5,531 | 3,300 | 1,510 | 1,790 |
| 17 | 2,779 | 1,330 | 1,449 | 4,671 | 2,285 | 2,386 | 3,863 | 1,890 | 1,973 | 9,576 | 4,465 | 5,111 | 2,989 | 1,470 | 1,519 |
| 18 | 3,465 | 1,710 | 1,755 | 5,981 | 2,993 | 2,988 | 4,675 | 2,261 | 2,414 | 11,665 | 5,632 | 6,033 | 3,733 | 1,837 | 1,896 |
| 19 | 2,612 | 1,225 | 1,387 | 4,636 | 2,289 | 2,347 | 3,832 | 1,808 | 2,024 | 10,033 | 4,939 | 5,094 | 3,019 | 1,499 | 1,520 |
| 20 | 3,317 | 1,534 | 1,783 | 5,454 | 2,598 | 2,856 | 3,958 | 1,840 | 2,118 | 10,538 | 5,042 | 5,496 | 3,276 | 1,561 | 1,715 |
| 21 | 2,135 | 960 | 1,175 | 3,354 | 1,627 | 1,727 | 3,091 | 1,415 | 1,676 | 8,472 | 3,920 | 4,552 | 2,399 | 1,103 | 1,296 |
| 22 | 2,537 | 1,097 | 1,440 | 4,048 | 1,815 | 2,233 | 3,384 | 1,566 | 1,818 | 8,669 | 4,023 | 4,646 | 2,626 | 1,225 | 1,401 |
| 23 | 2,207 | 982 | 1,225 | 3,434 | 1,577 | 1,857 | 3,217 | 1,552 | 1,665 | 8,658 | 3,999 | 4,659 | 2,334 | 1,009 | 1,325 |
| 24 | 2,183 | 988 | 1,195 | 3,237 | 1,436 | 1,801 | 3,068 | 1,414 | 1,654 | 8,010 | 3,527 | 4,483 | 2,264 | 977 | 1,287 |
| 25 | 2,591 | 1,162 | 1,429 | 4,250 | 1,921 | 2,329 | 3,386 | 1,551 | 1,835 | 8,775 | 4,112 | 4,663 | 2,501 | 1,115 | 1,386 |
| 26 | 2,099 | 920 | 1,179 | 3,214 | 1,459 | 1,755 | 3,047 | 1,493 | 1,554 | 7,819 | 3,559 | 4,260 | 2,174 | 934 | 1,240 |
| 27 | 1,948 | 857 | 1,091 | 2,968 | 1,321 | 1,647 | 2,833 | 1,336 | 1,497 | 7,327 | 3,514 | 3,813 | 2,034 | 920 | 1,114 |
| 28 | 2,545 | 1,239 | 1,306 | 3,658 | 1,614 | 2,044 | 3,250 | 1,544 | 1,706 | 8,663 | 4,098 | 4,565 | 2,353 | 1,024 | 1,329 |
| 29 | 1,515 | 730 | 785 | 2,464 | 1,102 | 1,362 | 2,243 | 1,114 | 1,129 | 6,150 | 2,995 | 3,155 | 1,741 | 833 | 908 |
| 30 | 2,719 | 1,365 | 1,354 | 4,216 | 2,182 | 2,034 | 3,442 | 1,769 | 1,673 | 8,711 | 4,536 | 4,175 | 2,473 | 1,203 | 1,270 |
| 31 | 1,125 | 577 | 548 | 1,872 | 923 | 949 | 1,775 | 935 | 840 | 5,016 | 2,665 | 2,351 | 1,364 | 650 | 714 |
| 32 | 1,877 | 935 | 942 | 3,062 | 1,492 | 1,570 | 2,529 | 1,309 | 1,220 | 7,010 | 3,579 | 3,431 | 1,814 | 902 | 912 |
| 33 | 1,115 | 592 | 523 | 1,825 | 928 | 897 | 1,628 | 882 | 746 | 4,735 | 2,386 | 2,349 | 1,293 | 620 | 673 |
| 34 | 1,292 | 685 | 607 | 1,996 | 1,013 | 983 | 1,771 | 970 | 801 | 5,155 | 2,724 | 2,431 | 1,429 | 714 | 715 |
| 35 | 1,954 | 1,076 | 878 | 3,141 | 1,698 | 1,443 | 2,216 | 1,220 | 996 | 6,276 | 3,495 | 2,781 | 1,771 | 936 | 835 |
| 36 | 1,354 | 711 | 643 | 2,138 | 1,105 | 1,033 | 1,623 | 891 | 732 | 4,854 | 2,584 | 2,270 | 1,308 | 693 | 615 |
| 37 | 1,034 | 573 | 461 | 1,654 | 880 | 774 | 1,429 | 748 | 681 | 3,791 | 2,034 | 1,757 | 1,047 | 518 | 529 |
| 38 | 1,436 | 775 | 661 | 2,429 | 1,274 | 1,155 | 1,683 | 892 | 791 | 4,691 | 2,572 | 2,119 | 1,350 | 697 | 653 |
| 39 | 780 | 431 | 349 | 1,368 | 724 | 644 | 1,153 | 595 | 558 | 3,181 | 1,730 | 1,451 | 865 | 419 | 446 |
| 40 | 1,430 | 835 | 595 | 2,378 | 1,387 | 991 | 1,888 | 1,002 | 886 | 4,533 | 2,584 | 1,949 | 1,439 | 771 | 668 |
| 41 | 593 | 314 | 279 | 1,048 | 566 | 482 | 926 | 511 | 415 | 2,409 | 1,326 | 1,083 | 683 | 368 | 315 |
| 42 | 1,039 | 593 | 446 | 1,778 | 1,010 | 768 | 1,384 | 746 | 638 | 3,544 | 1,984 | 1,560 | 1,023 | 533 | 490 |
| 43 | 543 | 287 | 256 | 984 | 503 | 481 | 779 | 413 | 366 | 2,122 | 1,118 | 1,004 | 615 | 308 | 307 |
| 44 | 470 | 240 | 230 | 863 | 423 | 440 | 699 | 368 | 331 | 1,888 | 957 | 931 | 546 | 263 | 283 |
| 45 | 857 | 466 | 391 | 1,596 | 845 | 751 | 1,058 | 561 | 497 | 2,674 | 1,434 | 1,240 | 836 | 433 | 403 |
| 46 | 720 | 360 | 360 | 1,384 | 696 | 688 | 1,036 | 570 | 466 | 2,788 | 1,425 | 1,363 | 808 | 392 | 416 |
| 47 | 433 | 222 | 211 | 897 | 476 | 421 | 719 | 374 | 345 | 1,786 | 956 | 830 | 540 | 272 | 268 |
| 48 | 618 | 300 | 318 | 1,202 | 586 | 616 | 840 | 405 | 435 | 2,045 | 1,038 | 1,007 | 680 | 315 | 365 |
| 49 | 416 | 207 | 209 | 714 | 372 | 342 | 597 | 309 | 288 | 1,452 | 736 | 716 | 477 | 229 | 248 |
| 50 | 706 | 358 | 348 | 1,306 | 667 | 639 | 981 | 469 | 512 | 2,191 | 1,134 | 1,057 | 753 | 355 | 398 |
| 51 | 312 | 145 | 167 | 608 | 293 | 315 | 448 | 219 | 229 | 1,282 | 685 | 597 | 397 | 186 | 211 |
| 52 | 482 | 244 | 238 | 940 | 463 | 477 | 723 | 373 | 350 | 1,734 | 867 | 867 | 586 | 238 | 348 |
| 53 | 317 | 160 | 157 | 599 | 296 | 303 | 480 | 256 | 224 | 1,266 | 643 | 623 | 407 | 183 | 224 |

## Table A4: Population (De Jure) by Single Years Age and Sex by Province, Rural/Urban Zambia 2010 - Continued

| Single Year Ages | Muchinga | | | Northern | | | North Western | | | Southern | | | Western | | |
|---|---|---|---|---|---|---|---|---|---|---|---|---|---|---|---|
| | Total | Male | Female | Total | Male | Female | Total | Male | Female | Total | Male | Female | Total | Male | Female |
| 54 | 390 | 197 | 193 | 693 | 342 | 351 | 503 | 245 | 258 | 1,271 | 663 | 608 | 438 | 213 | 225 |
| 55 | 447 | 241 | 206 | 750 | 400 | 350 | 491 | 278 | 213 | 1,218 | 640 | 578 | 446 | 223 | 223 |
| 56 | 389 | 201 | 188 | 650 | 322 | 328 | 528 | 283 | 245 | 1,212 | 614 | 598 | 427 | 198 | 229 |
| 57 | 251 | 129 | 122 | 451 | 223 | 228 | 334 | 174 | 160 | 818 | 440 | 378 | 357 | 163 | 194 |
| 58 | 350 | 171 | 179 | 591 | 313 | 278 | 425 | 223 | 202 | 971 | 497 | 474 | 428 | 197 | 231 |
| 59 | 182 | 81 | 101 | 422 | 200 | 222 | 263 | 159 | 104 | 556 | 292 | 264 | 240 | 115 | 125 |
| 60 | 390 | 185 | 205 | 784 | 381 | 403 | 488 | 267 | 221 | 976 | 511 | 465 | 412 | 204 | 208 |
| 61 | 195 | 96 | 99 | 369 | 157 | 212 | 266 | 150 | 116 | 527 | 271 | 256 | 233 | 107 | 126 |
| 62 | 269 | 135 | 134 | 486 | 236 | 250 | 314 | 149 | 165 | 769 | 404 | 365 | 310 | 147 | 163 |
| 63 | 199 | 102 | 97 | 391 | 189 | 202 | 249 | 106 | 143 | 615 | 315 | 300 | 227 | 87 | 140 |
| 64 | 195 | 89 | 106 | 323 | 136 | 187 | 226 | 99 | 127 | 509 | 248 | 261 | 212 | 87 | 125 |
| 65 | 287 | 139 | 148 | 526 | 252 | 274 | 316 | 146 | 170 | 656 | 323 | 333 | 299 | 124 | 175 |
| 66 | 135 | 62 | 73 | 242 | 105 | 137 | 178 | 62 | 116 | 390 | 152 | 238 | 164 | 69 | 95 |
| 67 | 144 | 72 | 72 | 286 | 117 | 169 | 166 | 71 | 95 | 381 | 183 | 198 | 158 | 60 | 98 |
| 68 | 222 | 92 | 130 | 438 | 179 | 259 | 311 | 130 | 181 | 519 | 256 | 263 | 254 | 115 | 139 |
| 69 | 102 | 58 | 44 | 204 | 109 | 95 | 149 | 61 | 88 | 252 | 125 | 127 | 115 | 47 | 68 |
| 70 | 220 | 109 | 111 | 410 | 179 | 231 | 304 | 144 | 160 | 458 | 227 | 231 | 228 | 104 | 124 |
| 71 | 85 | 41 | 44 | 154 | 79 | 75 | 116 | 48 | 68 | 260 | 120 | 140 | 104 | 46 | 58 |
| 72 | 136 | 69 | 67 | 273 | 135 | 138 | 172 | 70 | 102 | 351 | 162 | 189 | 167 | 67 | 100 |
| 73 | 99 | 44 | 55 | 166 | 82 | 84 | 121 | 59 | 62 | 242 | 99 | 143 | 118 | 48 | 70 |
| 74 | 97 | 45 | 52 | 195 | 84 | 111 | 120 | 64 | 56 | 259 | 112 | 147 | 125 | 49 | 76 |
| 75 | 131 | 56 | 75 | 251 | 117 | 134 | 212 | 97 | 115 | 314 | 137 | 177 | 141 | 54 | 87 |
| 76 | 72 | 40 | 32 | 136 | 59 | 77 | 93 | 53 | 40 | 209 | 92 | 117 | 126 | 45 | 81 |
| 77 | 48 | 21 | 27 | 95 | 52 | 43 | 70 | 37 | 33 | 139 | 61 | 78 | 59 | 20 | 39 |
| 78 | 97 | 42 | 55 | 196 | 99 | 97 | 163 | 85 | 78 | 272 | 139 | 133 | 175 | 75 | 100 |
| 79 | 48 | 23 | 25 | 92 | 50 | 42 | 95 | 48 | 47 | 135 | 67 | 68 | 94 | 37 | 57 |
| 80 | 83 | 37 | 46 | 159 | 73 | 86 | 169 | 66 | 103 | 213 | 99 | 114 | 123 | 45 | 78 |
| 81 | 35 | 16 | 19 | 48 | 23 | 25 | 44 | 25 | 19 | 108 | 51 | 57 | 52 | 21 | 31 |
| 82 | 50 | 29 | 21 | 87 | 42 | 45 | 66 | 29 | 37 | 120 | 58 | 62 | 85 | 31 | 54 |
| 83 | 30 | 14 | 16 | 54 | 33 | 21 | 39 | 18 | 21 | 81 | 36 | 45 | 42 | 22 | 20 |
| 84 | 26 | 14 | 12 | 51 | 21 | 30 | 45 | 19 | 26 | 70 | 33 | 37 | 55 | 28 | 27 |
| 85 | 32 | 16 | 16 | 61 | 22 | 39 | 54 | 25 | 29 | 84 | 35 | 49 | 57 | 22 | 35 |
| 86 | 31 | 16 | 15 | 47 | 20 | 27 | 37 | 17 | 20 | 89 | 44 | 45 | 60 | 26 | 34 |
| 87 | 16 | 5 | 11 | 27 | 10 | 17 | 20 | 11 | 9 | 38 | 15 | 23 | 23 | 13 | 10 |
| 88 | 29 | 14 | 15 | 44 | 28 | 16 | 42 | 25 | 17 | 65 | 36 | 29 | 41 | 19 | 22 |
| 89 | 14 | 3 | 11 | 32 | 17 | 15 | 28 | 14 | 14 | 34 | 23 | 11 | 33 | 14 | 19 |
| 90 | 16 | 9 | 7 | 25 | 12 | 13 | 37 | 17 | 20 | 52 | 21 | 31 | 35 | 10 | 25 |
| 91 | 3 | 3 | - | 6 | 2 | 4 | 15 | 8 | 7 | 14 | 7 | 7 | 13 | 4 | 9 |
| 92 | 11 | 5 | 6 | 19 | 6 | 13 | 16 | 9 | 7 | 20 | 11 | 9 | 15 | 4 | 11 |
| 93 | 8 | 2 | 6 | 7 | 4 | 3 | 4 | 1 | 3 | 14 | 6 | 8 | 13 | 6 | 7 |
| 94 | 3 | 1 | 2 | 4 | 2 | 2 | 5 | 2 | 3 | 11 | 4 | 7 | 11 | 5 | 6 |
| 95+ | 52 | 14 | 38 | 78 | 25 | 53 | 112 | 42 | 70 | 138 | 55 | 83 | 140 | 55 | 85 |
| Median | 17.2 | 17.2 | 17.3 | 17.1 | 17.1 | 17.1 | 17.3 | 17.5 | 17.1 | 18.8 | 19.1 | 18.5 | 18.5 | 18.2 | 18.8 |
| **Age group** | | | | | | | | | | | | | | | |
| **Total Urban** | **121,082** | **59,382** | **61,700** | **202,616** | **99,096** | **103,520** | **163,983** | **80,638** | **83,345** | **392,175** | **192,211** | **199,964** | **119,851** | **57,752** | **62,099** |
| 0 - 14 | 53,652 | 26,412 | 27,240 | 90,119 | 44,071 | 46,048 | 72,531 | 35,470 | 37,061 | 156,621 | 76,005 | 80,616 | 48,539 | 24,025 | 24,514 |
| 15 - 64 | 65,068 | 31,859 | 33,209 | 108,084 | 52,987 | 55,097 | 88,133 | 43,665 | 44,468 | 229,566 | 113,417 | 116,149 | 68,187 | 32,442 | 35,745 |
| 65+ | 2,362 | 1,111 | 1,251 | 4,413 | 2,038 | 2,375 | 3,319 | 1,503 | 1,816 | 5,988 | 2,789 | 3,199 | 3,125 | 1,285 | 1,840 |
| | | | | | | | | | | | | | | | |
| Overall Dependency Ratio | 86.1 | 86.4 | 85.8 | 87.5 | 87.0 | 87.9 | 86.1 | 84.7 | 87.4 | 70.8 | 69.5 | 72.2 | 75.8 | 78.0 | 73.7 |
| Child Dependency Ratio | 82.5 | 82.9 | 82.0 | 83.4 | 83.2 | 83.6 | 82.3 | 81.2 | 83.3 | 68.2 | 67.0 | 69.4 | 71.2 | 74.1 | 68.6 |
| Aged Dependency Ratio | 3.6 | 3.5 | 3.8 | 4.1 | 3.8 | 4.3 | 3.8 | 3.4 | 4.1 | 2.6 | 2.5 | 2.8 | 4.6 | 4.0 | 5.1 |

## Table A5: Population, Land Area and Population Density by Province and District, Zambia, 2010

| Province and District | 2010 Population | Land Area (km$^2$) | Population Density (Persons per Square Km) |
|---|---|---|---|
| **Zambia** | **13,092,666** | **752,612** | **17.4** |
| Central | 1,307,111 | 94,394 | 13.8 |
| Copperbelt | 1,972,317 | 31,328 | 63.0 |
| Eastern | 1,592,661 | 51,476 | 30.9 |
| Luapula | 991,927 | 50,567 | 19.6 |
| Lusaka | 2,191,225 | 21,896 | 100.1 |
| Muchinga | 711,657 | 87,806 | 8.1 |
| Northern | 1,105,824 | 77,650 | 14.2 |
| North-Western | 727,044 | 125,826 | 5.8 |
| Southern | 1,589,926 | 85,283 | 18.6 |
| Western | 902,974 | 126,386 | 7.1 |
| | | | |
| **Central Province** | **1,307,111** | **94,394** | **13.8** |
| Chibombo | 303,519 | 13,423 | 22.6 |
| Kabwe | 202,360 | 1,572 | 128.7 |
| Kapiri Mposhi | 253,786 | 17,219 | 14.7 |
| Mkushi | 154,534 | 17,726 | 8.7 |
| Mumbwa | 226,171 | 21,103 | 10.7 |
| Serenje | 166,741 | 23,351 | 7.1 |
| | | | |
| **Copperbelt Province** | **1,972,317** | **31,328** | **63.0** |
| Chililabombwe | 91,833 | 1,026 | 89.5 |
| Chingola | 216,626 | 1,678 | 129.1 |
| Kalulushi | 100,381 | 725 | 138.5 |
| Kitwe | 517,543 | 777 | 666.1 |
| Luanshya | 156,059 | 811 | 192.4 |
| Lufwanyama | 78,503 | 9,849 | 8.0 |
| Masaiti | 103,857 | 5,383 | 19.3 |
| Mpongwe | 93,380 | 8,339 | 11.2 |
| Mufulira | 162,889 | 1,637 | 99.5 |
| Ndola | 451,246 | 1,103 | 409.1 |
| | | | |
| **Eastern Province** | **1,592,661** | **51,476** | **30.9** |
| Chadiza | 107,327 | 2,574 | 41.7 |
| Chipata | 455,783 | 6,693 | 68.1 |
| Katete | 243,849 | 3,989 | 61.1 |
| Lundazi | 323,870 | 14,058 | 23.0 |
| Mambwe | 68,918 | 5,294 | 13.0 |
| Nyimba | 85,025 | 10,509 | 8.1 |
| Petauke | 307,889 | 8,359 | 36.8 |
| | | | |
| **Luapula Province** | **991,927** | **50,567** | **19.6** |
| Chienge | 114,225 | 3,965 | 28.8 |
| Kawambwa | 134,414 | 9,303 | 14.4 |
| Mansa | 228,392 | 9,901 | 23.1 |
| Milenge | 43,337 | 6,261 | 6.9 |
| Mwense | 119,841 | 6,718 | 17.8 |
| Nchelenge | 152,807 | 4,090 | 37.4 |
| Samfya | 198,911 | 10,329 | 19.3 |

## Table A5: Population, Land Area and Population Density by Province and District, Zambia, 2010

| Province and District | 2010 Population | Land Area (km$^2$) | Population Density (Persons per Square Km) |
|---|---|---|---|
| **Lusaka Province** | **2,191,225** | **21,896** | **100.1** |
| Chongwe | 192,303 | 8,669 | 22.2 |
| Kafue | 227,466 | 9,396 | 24.2 |
| Luangwa | 24,304 | 3,471 | 7.0 |
| Lusaka | 1,747,152 | 360 | 4,853.2 |
| | | | |
| **Muchinga Province** | **711,657** | **87,806** | **8.1** |
| Chama | 103,894 | 17,630 | 5.9 |
| Chinsali | 146,518 | 15,395 | 9.5 |
| Isoka | 72,189 | 5,091 | 14.2 |
| Mafinga | 65,969 | 4,134 | 16.0 |
| Mpika | 203,379 | 40,935 | 5.0 |
| Nakonde | 119,708 | 4,621 | 25.9 |
| | | | |
| **Northern Province** | **1,105,824** | **77,650** | **14.2** |
| Chilubi | 81,248 | 4,648 | 17.5 |
| Kaputa | 119,514 | 13,004 | 9.2 |
| Kasama | 231,824 | 10,788 | 21.5 |
| Luwingu | 122,136 | 8,892 | 13.7 |
| Mbala | 203,129 | 8,339 | 24.3 |
| Mporokoso | 98,842 | 12,043 | 8.2 |
| Mpulungu | 98,073 | 10,170 | 9.6 |
| Mungwi | 151,058 | 9,766 | 15.5 |
| | | | |
| **North Western Province** | **727,044** | **125,826** | **5.8** |
| Chavuma | 35,041 | 4,280 | 8.2 |
| Ikelenge | 32,919 | 2,353 | 14.0 |
| Kabompo | 92,321 | 14,532 | 6.4 |
| Kasempa | 69,608 | 20,821 | 3.3 |
| Mufumbwe | 58,062 | 20,756 | 2.8 |
| Mwinilunga | 104,317 | 18,763 | 5.6 |
| Solwezi | 254,470 | 30,261 | 8.4 |
| Zambezi | 80,306 | 14,060 | 5.7 |
| | | | |
| **Southern Province** | **1,589,926** | **85,283** | **18.6** |
| Choma | 247,860 | 7,296 | 34.0 |
| Gwembe | 53,117 | 3,879 | 13.7 |
| Itezhi-tezhi | 68,599 | 16,064 | 4.3 |
| Kalomo | 262,344 | 15,000 | 17.2 |
| Kazungula | 100,957 | 16,835 | 6.0 |
| Livingstone | 139,509 | 695 | 200.7 |
| Mazabuka | 230,972 | 6,242 | 37.0 |
| Monze | 191,872 | 4,854 | 39.5 |
| Namwala | 102,866 | 5,687 | 18.1 |
| Siavonga | 90,213 | 3,871 | 23.3 |
| Sinazongwe | 101,617 | 4,860 | 20.9 |
| | | | |
| **Western Province** | **902,974** | **126,386** | **7.1** |
| Kalabo | 128,904 | 17,526 | 7.4 |
| Kaoma | 189,290 | 23,315 | 8.1 |
| Lukulu | 86,002 | 16,292 | 5.3 |
| Mongu | 179,585 | 10,075 | 17.8 |
| Senanga | 126,506 | 15,537 | 8.1 |
| Sesheke | 99,384 | 29,272 | 3.4 |
| Shang'ombo | 93,303 | 14,369 | 6.5 |

## Table A6: Population and Average Annual Growth Rate by Province and District, Rural/Urban, Zambia, 2000 and 2010

| Province, District and Rural/Urban | 2000 Census | 2010 Census | Average Annual Growth Rate |
|---|---|---|---|
| **Zambia** | **9,885,591** | **13,092,666** | **2.8** |
| Rural | 6,458,729 | 7,919,216 | 2.1 |
| Urban | 3,426,862 | 5,173,450 | 4.2 |
| | | | |
| **Central Province** | **1,012,257** | **1,307,111** | **2.6** |
| Chibombo | 241,612 | 303,519 | 2.3 |
| Kabwe | 176,758 | 202,360 | 1.4 |
| Kapiri-Mposhi | 194,752 | 253,786 | 2.7 |
| Mkushi | 107,438 | 154,534 | 3.7 |
| Mumbwa | 158,861 | 226,171 | 3.6 |
| Serenje | 132,836 | 166,741 | 2.3 |
| | | | |
| **Copperbelt Province** | **1,581,221** | **1,972,317** | **2.2** |
| Chililabombwe | 67,533 | 91,833 | 3.1 |
| Chingola | 172,026 | 216,626 | 2.3 |
| Kalulushi | 75,806 | 100,381 | 2.8 |
| Kitwe | 376,124 | 517,543 | 3.2 |
| Luanshya | 147,908 | 156,059 | 0.5 |
| Lufwanyama | 63,185 | 78,503 | 2.2 |
| Masaiti | 95,581 | 103,857 | 0.8 |
| Mpongwe | 64,371 | 93,380 | 3.8 |
| Mufulira | 143,930 | 162,889 | 1.2 |
| Ndola | 374,757 | 451,246 | 1.9 |
| | | | |
| **Eastern Province** | **1,231,283** | **1,592,661** | **2.6** |
| Chadiza | 83,981 | 107,327 | 2.5 |
| Chipata | 367,539 | 455,783 | 2.2 |
| Katete | 189,250 | 243,849 | 2.6 |
| Lundazi | 236,833 | 323,870 | 3.2 |
| Mambwe | 47,376 | 68,918 | 3.8 |
| Nyimba | 70,425 | 85,025 | 1.9 |
| Petauke | 235,879 | 307,889 | 2.7 |
| | | | |
| **Luapula Province** | **775,353** | **991,927** | **2.5** |
| Chienge | 83,824 | 114,225 | 3.1 |
| Kawambwa | 102,503 | 134,414 | 2.7 |
| Mansa | 179,749 | 228,392 | 2.4 |
| Milenge | 28,790 | 43,337 | 4.2 |
| Mwense | 105,759 | 119,841 | 1.3 |
| Nchelenge | 111,119 | 152,807 | 3.2 |
| Samfya | 163,609 | 198,911 | 2.0 |
| | | | |
| **Lusaka Province** | **1,391,329** | **2,191,225** | **4.6** |
| Chongwe | 137,461 | 192,303 | 3.4 |
| Kafue | 150,217 | 227,466 | 4.2 |
| Luangwa | 18,948 | 24,304 | 2.5 |
| Lusaka | 1,084,703 | 1,747,152 | 4.9 |

## Table A6: Population and Average Annual Growth Rate by Province and District, Rural/Urban, Zambia, 2000 and 2010

| Province, District and Rural/Urban | 2000 Census | 2010 Census | Average Annual Growth Rate |
|---|---|---|---|
| **Muchinga Province** | **524,186** | **711,657** | **3.1** |
| Chama | 74,890 | 103,894 | 3.3 |
| Chinsali | 128,646 | 146,518 | 1.3 |
| Isoka | 57,239 | 72,189 | 2.3 |
| Mafinga | 42,080 | 65,969 | 4.6 |
| Mpika | 146,196 | 203,379 | 3.4 |
| Nakonde | 75,135 | 119,708 | 4.8 |
| | | | |
| **Northern Province** | **809,400** | **1,105,824** | **3.2** |
| Chilubi | 66,338 | 81,248 | 2.0 |
| Kaputa | 87,233 | 119,514 | 3.2 |
| Kasama | 170,929 | 231,824 | 3.1 |
| Luwingu | 80,758 | 122,136 | 4.2 |
| Mbala | 149,634 | 203,129 | 3.1 |
| Mporokoso | 73,929 | 98,842 | 2.9 |
| Mpulungu | 67,602 | 98,073 | 3.8 |
| Mungwi | 112,977 | 151,058 | 2.9 |
| | | | |
| **North Western Province** | **583,350** | **727,044** | **2.2** |
| Chavuma | 29,941 | 35,041 | 1.6 |
| Ikelenge | 31,487 | 32,919 | 0.4 |
| Kabompo | 71,238 | 92,321 | 2.6 |
| Kasempa | 51,904 | 69,608 | 3.0 |
| Mufumbwe | 44,002 | 58,062 | 2.8 |
| Mwinilunga | 86,018 | 104,317 | 1.9 |
| Solwezi | 203,797 | 254,470 | 2.2 |
| Zambezi | 64,963 | 80,306 | 2.1 |
| | | | |
| **Southern Province** | **1,212,124** | **1,589,926** | **2.8** |
| Choma | 204,898 | 247,860 | 1.9 |
| Gwembe | 34,133 | 53,117 | 4.5 |
| Itezhi-tezhi | 43,111 | 68,599 | 4.8 |
| Kalomo | 169,503 | 258,570 | 4.4 |
| Kazungula | 68,265 | 104,731 | 4.3 |
| Livingstone | 103,288 | 139,509 | 3.1 |
| Mazabuka | 203,219 | 230,972 | 1.3 |
| Monze | 163,578 | 191,872 | 1.6 |
| Namwala | 82,810 | 102,866 | 2.2 |
| Siavonga | 58,864 | 90,213 | 4.4 |
| Sinazongwe | 80,455 | 101,617 | 2.4 |
| | | | |
| **Western Province** | **765,088** | **902,974** | **1.7** |
| Kalabo | 114,806 | 128,904 | 1.2 |
| Kaoma | 162,568 | 189,290 | 1.5 |
| Lukulu | 68,375 | 86,002 | 2.3 |
| Mongu | 162,002 | 179,585 | 1.0 |
| Senanga | 109,119 | 126,506 | 1.5 |
| Sesheke | 78,169 | 99,384 | 2.4 |
| Shang'ombo | 70,049 | 93,303 | 2.9 |

## Table A7: Population (De facto) by 5 Year Age Group, Household Membership Status and Sex, Rural/Urban, Zambia 2010

| Age Group and Rural/Urban | Household Membership Status | | | | | | | | |
|---|---|---|---|---|---|---|---|---|---|
| | Usual Residents Present | | | Usual Residents Absent | | | Visitors | | |
| | Total | Male | Female | Total | Male | Female | Total | Male | Female |
| **Zambia Total** | **12,362,720** | **6,051,211** | **6,311,509** | **729,946** | **403,436** | **326,510** | **163,594** | **66,042** | **97,552** |
| 0 - 4 | 2,186,052 | 1,088,514 | 1,097,538 | 66,696 | 32,954 | 33,742 | 28,835 | 13,758 | 15,077 |
| 5 - 9 | 1,841,526 | 917,655 | 923,871 | 74,761 | 36,677 | 38,084 | 14,810 | 6,740 | 8,070 |
| 10 - 14 | 1,685,987 | 835,314 | 850,673 | 88,147 | 43,258 | 44,889 | 13,055 | 5,520 | 7,535 |
| 15 - 19 | 1,407,600 | 684,868 | 722,732 | 123,515 | 63,748 | 59,767 | 20,284 | 7,991 | 12,293 |
| 20 - 24 | 1,095,127 | 495,897 | 599,230 | 99,515 | 57,370 | 42,145 | 22,349 | 8,979 | 13,370 |
| 25 - 29 | 989,894 | 457,775 | 532,119 | 67,183 | 39,999 | 27,184 | 16,350 | 6,718 | 9,632 |
| 30 - 34 | 789,984 | 393,240 | 396,744 | 50,324 | 31,987 | 18,337 | 10,786 | 4,454 | 6,332 |
| 35 - 39 | 640,246 | 329,753 | 310,493 | 42,675 | 27,344 | 15,331 | 7,567 | 3,208 | 4,359 |
| 40 - 44 | 442,153 | 230,130 | 212,023 | 31,085 | 20,285 | 10,800 | 5,431 | 2,123 | 3,308 |
| 45 - 49 | 350,436 | 173,371 | 177,065 | 25,728 | 15,676 | 10,052 | 4,553 | 1,462 | 3,091 |
| 50 - 54 | 265,953 | 127,588 | 138,365 | 18,911 | 11,176 | 7,735 | 4,301 | 1,108 | 3,193 |
| 55 - 59 | 181,362 | 89,188 | 92,174 | 12,800 | 7,530 | 5,270 | 3,466 | 849 | 2,617 |
| 60 - 64 | 158,417 | 72,618 | 85,799 | 10,146 | 5,683 | 4,463 | 3,458 | 801 | 2,657 |
| 65 - 69 | 115,797 | 53,035 | 62,762 | 7,134 | 3,779 | 3,355 | 2,785 | 660 | 2,125 |
| 70 - 74 | 88,443 | 41,288 | 47,155 | 4,905 | 2,657 | 2,248 | 2,359 | 641 | 1,718 |
| 75 - 79 | 59,833 | 30,164 | 29,669 | 3,230 | 1,765 | 1,465 | 1,587 | 498 | 1,089 |
| 80 - 84 | 31,983 | 15,777 | 16,206 | 1,615 | 792 | 823 | 838 | 268 | 570 |
| 85 - 89 | 18,219 | 9,197 | 9,022 | 899 | 454 | 445 | 436 | 150 | 286 |
| 90 - 94 | 6,180 | 3,099 | 3,081 | 270 | 146 | 124 | 128 | 53 | 75 |
| 95+ | 7,528 | 2,740 | 4,788 | 407 | 156 | 251 | 216 | 61 | 155 |
| | | | | | | | | | |
| **Zambia Rural** | **7,420,667** | **3,628,531** | **3,792,136** | **498,549** | **278,105** | **220,444** | **84,625** | **35,818** | **48,807** |
| 0 - 4 | 1,438,404 | 716,436 | 721,968 | 49,752 | 24,568 | 25,184 | 15,981 | 7,722 | 8,259 |
| 5 - 9 | 1,203,637 | 604,310 | 599,327 | 56,480 | 27,754 | 28,726 | 8,610 | 3,931 | 4,679 |
| 10 - 14 | 1,038,514 | 526,648 | 511,866 | 63,531 | 31,501 | 32,030 | 7,463 | 3,330 | 4,133 |
| 15 - 19 | 792,486 | 393,411 | 399,075 | 82,568 | 43,283 | 39,285 | 10,511 | 4,327 | 6,184 |
| 20 - 24 | 587,230 | 264,739 | 322,491 | 62,772 | 36,985 | 25,787 | 11,040 | 4,629 | 6,411 |
| 25 - 29 | 521,347 | 237,779 | 283,568 | 43,017 | 26,169 | 16,848 | 8,134 | 3,357 | 4,777 |
| 30 - 34 | 414,456 | 201,211 | 213,245 | 32,478 | 21,086 | 11,392 | 5,476 | 2,296 | 3,180 |
| 35 - 39 | 348,098 | 171,943 | 176,155 | 27,641 | 18,041 | 9,600 | 3,872 | 1,707 | 2,165 |
| 40 - 44 | 252,173 | 125,853 | 126,320 | 20,348 | 13,500 | 6,848 | 2,791 | 1,191 | 1,600 |
| 45 - 49 | 207,581 | 100,103 | 107,478 | 17,219 | 10,666 | 6,553 | 2,287 | 800 | 1,487 |
| 50 - 54 | 160,370 | 75,229 | 85,141 | 12,703 | 7,685 | 5,018 | 2,025 | 639 | 1,386 |
| 55 - 59 | 109,995 | 51,769 | 58,226 | 8,711 | 5,159 | 3,552 | 1,516 | 456 | 1,060 |
| 60 - 64 | 105,633 | 45,362 | 60,271 | 7,266 | 4,073 | 3,193 | 1,405 | 399 | 1,006 |
| 65 - 69 | 82,043 | 36,295 | 45,748 | 5,336 | 2,882 | 2,454 | 1,114 | 316 | 798 |
| 70 - 74 | 65,399 | 30,271 | 35,128 | 3,742 | 2,075 | 1,667 | 989 | 282 | 707 |
| 75 - 79 | 44,988 | 23,188 | 21,800 | 2,484 | 1,418 | 1,066 | 693 | 206 | 487 |
| 80 - 84 | 23,986 | 12,144 | 11,842 | 1,263 | 637 | 626 | 332 | 103 | 229 |
| 85 - 89 | 13,669 | 7,165 | 6,504 | 687 | 368 | 319 | 214 | 73 | 141 |
| 90 - 94 | 4,749 | 2,467 | 2,282 | 224 | 126 | 98 | 69 | 29 | 40 |
| 95+ | 5,909 | 2,208 | 3,701 | 327 | 129 | 198 | 103 | 25 | 78 |

## Table A7: Population by 5 Year Age Group, Household Membership Status and Sex, Rural/Urban, Zambia 2010

| Age Group and Rural/Urban | Household Membership Status | | | | | | | | |
|---|---|---|---|---|---|---|---|---|---|
| | Usual Residents Present | | | Usual Residents Absent | | | Visitors | | |
| | Total | Male | Female | Total | Male | Female | Total | Male | Female |
| **Zambia Urban** | **4,942,053** | **2,422,680** | **2,519,373** | **231,397** | **125,331** | **106,066** | **78,969** | **30,224** | **48,745** |
| 0 - 4 | 747,648 | 372,078 | 375,570 | 16,944 | 8,386 | 8,558 | 12,854 | 6,036 | 6,818 |
| 5 - 9 | 637,889 | 313,345 | 324,544 | 18,281 | 8,923 | 9,358 | 6,200 | 2,809 | 3,391 |
| 10 - 14 | 647,473 | 308,666 | 338,807 | 24,616 | 11,757 | 12,859 | 5,592 | 2,190 | 3,402 |
| 15 - 19 | 615,114 | 291,457 | 323,657 | 40,947 | 20,465 | 20,482 | 9,773 | 3,664 | 6,109 |
| 20 - 24 | 507,897 | 231,158 | 276,739 | 36,743 | 20,385 | 16,358 | 11,309 | 4,350 | 6,959 |
| 25 - 29 | 468,547 | 219,996 | 248,551 | 24,166 | 13,830 | 10,336 | 8,216 | 3,361 | 4,855 |
| 30 - 34 | 375,528 | 192,029 | 183,499 | 17,846 | 10,901 | 6,945 | 5,310 | 2,158 | 3,152 |
| 35 - 39 | 292,148 | 157,810 | 134,338 | 15,034 | 9,303 | 5,731 | 3,695 | 1,501 | 2,194 |
| 40 - 44 | 189,980 | 104,277 | 85,703 | 10,737 | 6,785 | 3,952 | 2,640 | 932 | 1,708 |
| 45 - 49 | 142,855 | 73,268 | 69,587 | 8,509 | 5,010 | 3,499 | 2,266 | 662 | 1,604 |
| 50 - 54 | 105,583 | 52,359 | 53,224 | 6,208 | 3,491 | 2,717 | 2,276 | 469 | 1,807 |
| 55 - 59 | 71,367 | 37,419 | 33,948 | 4,089 | 2,371 | 1,718 | 1,950 | 393 | 1,557 |
| 60 - 64 | 52,784 | 27,256 | 25,528 | 2,880 | 1,610 | 1,270 | 2,053 | 402 | 1,651 |
| 65 - 69 | 33,754 | 16,740 | 17,014 | 1,798 | 897 | 901 | 1,671 | 344 | 1,327 |
| 70 - 74 | 23,044 | 11,017 | 12,027 | 1,163 | 582 | 581 | 1,370 | 359 | 1,011 |
| 75 - 79 | 14,845 | 6,976 | 7,869 | 746 | 347 | 399 | 894 | 292 | 602 |
| 80 - 84 | 7,997 | 3,633 | 4,364 | 352 | 155 | 197 | 506 | 165 | 341 |
| 85 - 89 | 4,550 | 2,032 | 2,518 | 212 | 86 | 126 | 222 | 77 | 145 |
| 90 - 94 | 1,431 | 632 | 799 | 46 | 20 | 26 | 59 | 24 | 35 |
| 95+ | 1,619 | 532 | 1,087 | 80 | 27 | 53 | 113 | 36 | 77 |

## Table A8: Population (De facto) by 5 Year Age Group, Sex and Citizenship, Rural/Urban, Zambia 2010

| Age Group and Rural/Urban | Total Population | | | Citizenship | | | | | |
|---|---|---|---|---|---|---|---|---|---|
| | | | | Zambian | | | Non-Zambian | | |
| | Total | Male | Female | Total | Male | Female | Total | Male | Female |
| **Zambia Total** | **12,526,314** | **6,117,253** | **6,409,061** | **12,482,447** | **6,093,943** | **6,388,504** | **43,867** | **23,310** | **20,557** |
| 0 - 4 | 2,214,887 | 1,102,272 | 1,112,615 | 2,210,726 | 1,100,171 | 1,110,555 | 4,161 | 2,101 | 2,060 |
| 5 - 9 | 1,856,336 | 924,395 | 931,941 | 1,852,513 | 922,476 | 930,037 | 3,823 | 1,919 | 1,904 |
| 10 - 14 | 1,699,042 | 840,834 | 858,208 | 1,695,282 | 838,998 | 856,284 | 3,760 | 1,836 | 1,924 |
| 15 - 19 | 1,427,884 | 692,859 | 735,025 | 1,424,765 | 691,407 | 733,358 | 3,119 | 1,452 | 1,667 |
| 20 - 24 | 1,117,476 | 504,876 | 612,600 | 1,114,277 | 503,445 | 610,832 | 3,199 | 1,431 | 1,768 |
| 25 - 29 | 1,006,244 | 464,493 | 541,751 | 1,002,539 | 462,671 | 539,868 | 3,705 | 1,822 | 1,883 |
| 30 - 34 | 800,770 | 397,694 | 403,076 | 796,835 | 395,615 | 401,220 | 3,935 | 2,079 | 1,856 |
| 35 - 39 | 647,813 | 332,961 | 314,852 | 643,975 | 330,746 | 313,229 | 3,838 | 2,215 | 1,623 |
| 40 - 44 | 447,584 | 232,253 | 215,331 | 444,360 | 230,255 | 214,105 | 3,224 | 1,998 | 1,226 |
| 45 - 49 | 354,989 | 174,833 | 180,156 | 352,415 | 173,216 | 179,199 | 2,574 | 1,617 | 957 |
| 50 - 54 | 270,254 | 128,696 | 141,558 | 267,970 | 127,363 | 140,607 | 2,284 | 1,333 | 951 |
| 55 - 59 | 184,828 | 90,037 | 94,791 | 183,097 | 88,992 | 94,105 | 1,731 | 1,045 | 686 |
| 60 - 64 | 161,875 | 73,419 | 88,456 | 160,442 | 72,642 | 87,800 | 1,433 | 777 | 656 |
| 65 - 69 | 118,582 | 53,695 | 64,887 | 117,474 | 53,078 | 64,396 | 1,108 | 617 | 491 |
| 70 - 74 | 90,802 | 41,929 | 48,873 | 89,970 | 41,470 | 48,500 | 832 | 459 | 373 |
| 75 - 79 | 61,420 | 30,662 | 30,758 | 60,863 | 30,354 | 30,509 | 557 | 308 | 249 |
| 80 - 84 | 32,821 | 16,045 | 16,776 | 32,524 | 15,880 | 16,644 | 297 | 165 | 132 |
| 85 - 89 | 18,655 | 9,347 | 9,308 | 18,490 | 9,261 | 9,229 | 165 | 86 | 79 |
| 90 - 94 | 6,308 | 3,152 | 3,156 | 6,246 | 3,127 | 3,119 | 62 | 25 | 37 |
| 95+ | 7,744 | 2,801 | 4,943 | 7,684 | 2,776 | 4,908 | 60 | 25 | 35 |
| | | | | | | | | | |
| **Zambia Rural** | **7,505,292** | **3,664,349** | **3,840,943** | **7,487,478** | **3,655,198** | **3,832,280** | **17,814** | **9,151** | **8,663** |
| 0 - 4 | 1,454,385 | 724,158 | 730,227 | 1,452,192 | 723,028 | 729,164 | 2,193 | 1,130 | 1,063 |
| 5 - 9 | 1,212,247 | 608,241 | 604,006 | 1,210,255 | 607,256 | 602,999 | 1,992 | 985 | 1,007 |
| 10 - 14 | 1,045,977 | 529,978 | 515,999 | 1,043,932 | 528,987 | 514,945 | 2,045 | 991 | 1,054 |
| 15 - 19 | 802,997 | 397,738 | 405,259 | 801,382 | 396,977 | 404,405 | 1,615 | 761 | 854 |
| 20 - 24 | 598,270 | 269,368 | 328,902 | 596,991 | 268,798 | 328,193 | 1,279 | 570 | 709 |
| 25 - 29 | 529,481 | 241,136 | 288,345 | 528,537 | 240,718 | 287,819 | 944 | 418 | 526 |
| 30 - 34 | 419,932 | 203,507 | 216,425 | 418,930 | 203,027 | 215,903 | 1,002 | 480 | 522 |
| 35 - 39 | 351,970 | 173,650 | 178,320 | 350,836 | 173,071 | 177,765 | 1,134 | 579 | 555 |
| 40 - 44 | 254,964 | 127,044 | 127,920 | 253,880 | 126,424 | 127,456 | 1,084 | 620 | 464 |
| 45 - 49 | 209,868 | 100,903 | 108,965 | 208,931 | 100,323 | 108,608 | 937 | 580 | 357 |
| 50 - 54 | 162,395 | 75,868 | 86,527 | 161,546 | 75,386 | 86,160 | 849 | 482 | 367 |
| 55 - 59 | 111,511 | 52,225 | 59,286 | 110,849 | 51,826 | 59,023 | 662 | 399 | 263 |
| 60 - 64 | 107,038 | 45,761 | 61,277 | 106,471 | 45,439 | 61,032 | 567 | 322 | 245 |
| 65 - 69 | 83,157 | 36,611 | 46,546 | 82,653 | 36,334 | 46,319 | 504 | 277 | 227 |
| 70 - 74 | 66,388 | 30,553 | 35,835 | 65,989 | 30,322 | 35,667 | 399 | 231 | 168 |
| 75 - 79 | 45,681 | 23,394 | 22,287 | 45,395 | 23,241 | 22,154 | 286 | 153 | 133 |
| 80 - 84 | 24,318 | 12,247 | 12,071 | 24,161 | 12,157 | 12,004 | 157 | 90 | 67 |
| 85 - 89 | 13,883 | 7,238 | 6,645 | 13,786 | 7,188 | 6,598 | 97 | 50 | 47 |
| 90 - 94 | 4,818 | 2,496 | 2,322 | 4,789 | 2,480 | 2,309 | 29 | 16 | 13 |
| 95+ | 6,012 | 2,233 | 3,779 | 5,973 | 2,216 | 3,757 | 39 | 17 | 22 |

## Table A8: Population (De facto) by 5 Year Age Group, Sex and Citizenship, Rural/Urban, Zambia 2010

| Age Group and Rural/Urban | Total Population | | | Citizenship | | | | | |
|---|---|---|---|---|---|---|---|---|---|
| | | | | Zambian | | | Non-Zambian | | |
| | Total | Male | Female | Total | Male | Female | Total | Male | Female |
| **Zambia Urban** | **5,021,022** | **2,452,904** | **2,568,118** | **4,994,969** | **2,438,745** | **2,556,224** | **26,053** | **14,159** | **11,894** |
| 0 - 4 | 760,502 | 378,114 | 382,388 | 758,534 | 377,143 | 381,391 | 1,968 | 971 | 997 |
| 5 - 9 | 644,089 | 316,154 | 327,935 | 642,258 | 315,220 | 327,038 | 1,831 | 934 | 897 |
| 10 - 14 | 653,065 | 310,856 | 342,209 | 651,350 | 310,011 | 341,339 | 1,715 | 845 | 870 |
| 15 - 19 | 624,887 | 295,121 | 329,766 | 623,383 | 294,430 | 328,953 | 1,504 | 691 | 813 |
| 20 - 24 | 519,206 | 235,508 | 283,698 | 517,286 | 234,647 | 282,639 | 1,920 | 861 | 1,059 |
| 25 - 29 | 476,763 | 223,357 | 253,406 | 474,002 | 221,953 | 252,049 | 2,761 | 1,404 | 1,357 |
| 30 - 34 | 380,838 | 194,187 | 186,651 | 377,905 | 192,588 | 185,317 | 2,933 | 1,599 | 1,334 |
| 35 - 39 | 295,843 | 159,311 | 136,532 | 293,139 | 157,675 | 135,464 | 2,704 | 1,636 | 1,068 |
| 40 - 44 | 192,620 | 105,209 | 87,411 | 190,480 | 103,831 | 86,649 | 2,140 | 1,378 | 762 |
| 45 - 49 | 145,121 | 73,930 | 71,191 | 143,484 | 72,893 | 70,591 | 1,637 | 1,037 | 600 |
| 50 - 54 | 107,859 | 52,828 | 55,031 | 106,424 | 51,977 | 54,447 | 1,435 | 851 | 584 |
| 55 - 59 | 73,317 | 37,812 | 35,505 | 72,248 | 37,166 | 35,082 | 1,069 | 646 | 423 |
| 60 - 64 | 54,837 | 27,658 | 27,179 | 53,971 | 27,203 | 26,768 | 866 | 455 | 411 |
| 65 - 69 | 35,425 | 17,084 | 18,341 | 34,821 | 16,744 | 18,077 | 604 | 340 | 264 |
| 70 - 74 | 24,414 | 11,376 | 13,038 | 23,981 | 11,148 | 12,833 | 433 | 228 | 205 |
| 75 - 79 | 15,739 | 7,268 | 8,471 | 15,468 | 7,113 | 8,355 | 271 | 155 | 116 |
| 80 - 84 | 8,503 | 3,798 | 4,705 | 8,363 | 3,723 | 4,640 | 140 | 75 | 65 |
| 85 - 89 | 4,772 | 2,109 | 2,663 | 4,704 | 2,073 | 2,631 | 68 | 36 | 32 |
| 90 - 94 | 1,490 | 656 | 834 | 1,457 | 647 | 810 | 33 | 9 | 24 |
| 95+ | 1,732 | 568 | 1,164 | 1,711 | 560 | 1,151 | 21 | 8 | 13 |

## Table A9: Total Population (De Facto) by 5 Year Age Group, Citizenship and Place of Birth, Rural/Urban, Zambia 2010

| Age Group and Sex | Total | Zambian Citizens | | | | | | Non Zambian Citizens | | | | | |
|---|---|---|---|---|---|---|---|---|---|---|---|---|---|
| | | Born In Zambia | | | Born Outside Zambia | | | Born In Zambia | | | Born Outside Zambia | | |
| | | Total | Rural | Urban | Total | Africa | Outside Africa | Total | Rural | Urban | Total | Africa | Outside Africa |
| **Both Sexes** | **12,526,314** | **12,415,676** | **7,874,161** | **4,541,515** | **66,771** | **61,115** | **5,656** | **8,858** | **5,311** | **3,547** | **35,009** | **25,674** | **9,335** |
| 0 - 4 | 2,214,887 | 2,207,059 | 1,434,310 | 772,749 | 3,667 | 2,937 | 730 | 2,775 | 1,839 | 936 | 1,386 | 905 | 481 |
| 5 - 9 | 1,856,336 | 1,848,452 | 1,211,383 | 637,069 | 4,061 | 3,382 | 679 | 2,036 | 1,382 | 654 | 1,787 | 1,322 | 465 |
| 10 - 14 | 1,699,042 | 1,691,018 | 1,055,357 | 635,661 | 4,264 | 3,683 | 581 | 1,124 | 670 | 454 | 2,636 | 2,203 | 433 |
| 15 - 19 | 1,427,884 | 1,420,474 | 827,040 | 593,434 | 4,291 | 3,760 | 531 | 590 | 293 | 297 | 2,529 | 2,223 | 306 |
| 20 - 24 | 1,117,476 | 1,109,733 | 639,843 | 469,890 | 4,544 | 4,062 | 482 | 372 | 209 | 163 | 2,827 | 2,283 | 544 |
| 25 - 29 | 1,006,244 | 997,480 | 572,777 | 424,703 | 5,059 | 4,587 | 472 | 361 | 161 | 200 | 3,344 | 2,336 | 1,008 |
| 30 - 34 | 800,770 | 792,055 | 458,653 | 333,402 | 4,780 | 4,343 | 437 | 338 | 158 | 180 | 3,597 | 2,464 | 1,133 |
| 35 - 39 | 647,813 | 639,273 | 388,956 | 250,317 | 4,702 | 4,350 | 352 | 305 | 140 | 165 | 3,533 | 2,454 | 1,079 |
| 40 - 44 | 447,584 | 439,769 | 291,898 | 147,871 | 4,591 | 4,300 | 291 | 241 | 108 | 133 | 2,983 | 2,111 | 872 |
| 45 - 49 | 354,989 | 347,689 | 248,183 | 99,506 | 4,726 | 4,510 | 216 | 188 | 100 | 88 | 2,386 | 1,633 | 753 |
| 50 - 54 | 270,254 | 263,490 | 197,159 | 66,331 | 4,480 | 4,315 | 165 | 171 | 78 | 93 | 2,113 | 1,500 | 613 |
| 55 - 59 | 184,828 | 179,445 | 138,263 | 41,182 | 3,652 | 3,483 | 169 | 106 | 52 | 54 | 1,625 | 1,126 | 499 |
| 60 - 64 | 161,875 | 156,879 | 129,257 | 27,622 | 3,563 | 3,389 | 174 | 87 | 42 | 45 | 1,346 | 883 | 463 |
| 65+ | 336,332 | 322,860 | 281,082 | 41,778 | 10,391 | 10,014 | 377 | 164 | 79 | 85 | 2,917 | 2,231 | 686 |
| **Male** | | | | | | | | | | | | | |
| **Total** | **6,117,253** | **6,061,367** | **3,854,548** | **2,206,819** | **32,576** | **29,750** | **2,826** | **4,519** | **2,699** | **1,820** | **18,791** | **13,486** | **5,305** |
| 0 - 4 | 1,102,272 | 1,098,366 | 713,934 | 384,432 | 1,805 | 1,452 | 353 | 1,398 | 933 | 465 | 703 | 460 | 243 |
| 5 - 9 | 924,395 | 920,569 | 605,981 | 314,588 | 1,907 | 1,604 | 303 | 1,015 | 686 | 329 | 904 | 658 | 246 |
| 10 - 14 | 840,834 | 836,936 | 529,618 | 307,318 | 2,062 | 1,785 | 277 | 553 | 335 | 218 | 1,283 | 1,034 | 249 |
| 15 - 19 | 692,859 | 689,505 | 405,783 | 283,722 | 1,902 | 1,623 | 279 | 292 | 155 | 137 | 1,160 | 1,006 | 154 |
| 20 - 24 | 504,876 | 501,589 | 289,654 | 211,935 | 1,856 | 1,626 | 230 | 170 | 92 | 78 | 1,261 | 997 | 264 |
| 25 - 29 | 464,493 | 460,287 | 265,849 | 194,438 | 2,384 | 2,157 | 227 | 184 | 79 | 105 | 1,638 | 1,086 | 552 |
| 30 - 34 | 397,694 | 393,102 | 228,406 | 164,696 | 2,513 | 2,293 | 220 | 186 | 84 | 102 | 1,893 | 1,275 | 618 |
| 35 - 39 | 332,961 | 328,138 | 196,701 | 131,437 | 2,608 | 2,417 | 191 | 185 | 86 | 99 | 2,030 | 1,389 | 641 |
| 40 - 44 | 232,253 | 227,723 | 148,550 | 79,173 | 2,532 | 2,374 | 158 | 148 | 60 | 88 | 1,850 | 1,313 | 537 |
| 45 - 49 | 174,833 | 170,808 | 121,128 | 49,680 | 2,408 | 2,298 | 110 | 103 | 57 | 46 | 1,514 | 1,026 | 488 |
| 50 - 54 | 128,696 | 125,176 | 93,741 | 31,435 | 2,187 | 2,107 | 80 | 97 | 44 | 53 | 1,236 | 874 | 362 |
| 55 - 59 | 90,037 | 87,273 | 66,806 | 20,467 | 1,719 | 1,630 | 89 | 56 | 28 | 28 | 989 | 694 | 295 |
| 60 - 64 | 73,419 | 71,011 | 57,521 | 13,490 | 1,631 | 1,544 | 87 | 51 | 24 | 27 | 726 | 466 | 260 |
| 65+ | 157,631 | 150,884 | 130,876 | 20,008 | 5,062 | 4,840 | 222 | 81 | 36 | 45 | 1,604 | 1,208 | 396 |

## Table A9: Total Population (De Facto) by 5 Year Age Group, Citizenship and Place of Birth, Rural/Urban, Zambia 2010

| Age Group and Sex | Total | Zambian Citizens | | | | | | Non Zambian Citizens | | | | | |
|---|---|---|---|---|---|---|---|---|---|---|---|---|---|
| | | Born In Zambia | | | Born Outside Zambia | | | Born In Zambia | | | Born Outside Zambia | | |
| | | Total | Rural | Urban | Total | Africa | Outside Africa | Total | Rural | Urban | Total | Africa | Outside Africa |
| **Female** | | | | | | | | | | | | | |
| **Total** | **6,409,061** | **6,354,309** | **4,019,613** | **2,334,696** | **34,195** | **31,365** | **2,830** | **4,339** | **2,612** | **1,727** | **16,218** | **12,188** | **4,030** |
| 0 - 4 | 1,112,615 | 1,108,693 | 720,376 | 388,317 | 1,862 | 1,485 | 377 | 1,377 | 906 | 471 | 683 | 445 | 238 |
| 5 - 9 | 931,941 | 927,883 | 605,402 | 322,481 | 2,154 | 1,778 | 376 | 1,021 | 696 | 325 | 883 | 664 | 219 |
| 10 - 14 | 858,208 | 854,082 | 525,739 | 328,343 | 2,202 | 1,898 | 304 | 571 | 335 | 236 | 1,353 | 1,169 | 184 |
| 15 - 19 | 735,025 | 730,969 | 421,257 | 309,712 | 2,389 | 2,137 | 252 | 298 | 138 | 160 | 1,369 | 1,217 | 152 |
| 20 - 24 | 612,600 | 608,144 | 350,189 | 257,955 | 2,688 | 2,436 | 252 | 202 | 117 | 85 | 1,566 | 1,286 | 280 |
| 25 - 29 | 541,751 | 537,193 | 306,928 | 230,265 | 2,675 | 2,430 | 245 | 177 | 82 | 95 | 1,706 | 1,250 | 456 |
| 30 - 34 | 403,076 | 398,953 | 230,247 | 168,706 | 2,267 | 2,050 | 217 | 152 | 74 | 78 | 1,704 | 1,189 | 515 |
| 35 - 39 | 314,852 | 311,135 | 192,255 | 118,880 | 2,094 | 1,933 | 161 | 120 | 54 | 66 | 1,503 | 1,065 | 438 |
| 40 - 44 | 215,331 | 212,046 | 143,348 | 68,698 | 2,059 | 1,926 | 133 | 93 | 48 | 45 | 1,133 | 798 | 335 |
| 45 - 49 | 180,156 | 176,881 | 127,055 | 49,826 | 2,318 | 2,212 | 106 | 85 | 43 | 42 | 872 | 607 | 265 |
| 50 - 54 | 141,558 | 138,314 | 103,418 | 34,896 | 2,293 | 2,208 | 85 | 74 | 34 | 40 | 877 | 626 | 251 |
| 55 - 59 | 94,791 | 92,172 | 71,457 | 20,715 | 1,933 | 1,853 | 80 | 50 | 24 | 26 | 636 | 432 | 204 |
| 60 - 64 | 88,456 | 85,868 | 71,736 | 14,132 | 1,932 | 1,845 | 87 | 36 | 18 | 18 | 620 | 417 | 203 |
| 65+ | 178,701 | 171,976 | 150,206 | 21,770 | 5,329 | 5,174 | 155 | 83 | 43 | 40 | 1,313 | 1,023 | 290 |

## Table A10: Households and Population by (De jure) Sex, Province, District, Constituency and Ward, Zambia 2010

| Province,District,Constituency and Ward | Households | Population | | |
|---|---|---|---|---|
| | | Total | Male | Female |
| **Zambia** | **2,513,768** | **13,092,666** | **6,454,647** | **6,638,019** |
| **Central Province** | **235,560** | **1,307,111** | **648,465** | **658,646** |
| **Chibombo District** | **53,179** | **303,519** | **151,155** | **152,364** |
| **Chisamba Constituency** | **18,092** | **103,983** | **52,028** | **51,955** |
| Muswishi | 2,813 | 16,483 | 8,244 | 8,239 |
| Mulungushi | 1,112 | 6,814 | 3,461 | 3,353 |
| Chikonkomene | 2,503 | 15,802 | 7,940 | 7,862 |
| Chamuka | 3,833 | 21,210 | 10,685 | 10,525 |
| Chisamba | 4,435 | 23,635 | 11,894 | 11,741 |
| Liteta | 3,396 | 20,039 | 9,804 | 10,235 |
| **Katuba Constituency** | **14,490** | **79,306** | **39,613** | **39,693** |
| Katuba | 3,087 | 16,634 | 8,283 | 8,351 |
| Chunga | 1,617 | 8,083 | 4,015 | 4,068 |
| Mungule | 4,840 | 25,613 | 12,812 | 12,801 |
| Muchenje | 1,962 | 11,105 | 5,542 | 5,563 |
| Chilochabalenge | 1,323 | 7,651 | 3,773 | 3,878 |
| Kabile | 1,661 | 10,220 | 5,188 | 5,032 |
| **Keembe Constituency** | **20,597** | **120,230** | **59,514** | **60,716** |
| Chaloshi | 1,809 | 9,206 | 4,659 | 4,547 |
| Kalola | 2,559 | 14,058 | 7,078 | 6,980 |
| Kakoma | 3,252 | 18,613 | 9,158 | 9,455 |
| Chikobo | 1,060 | 5,778 | 2,873 | 2,905 |
| Chibombo | 1,729 | 9,541 | 4,649 | 4,892 |
| Chitanda | 2,100 | 12,554 | 6,309 | 6,245 |
| Mashikili | 3,744 | 23,000 | 11,312 | 11,688 |
| Keembe | 1,460 | 9,103 | 4,484 | 4,619 |
| Lunjofwa | 1,242 | 8,478 | 4,139 | 4,339 |
| Ipongo | 1,642 | 9,899 | 4,853 | 5,046 |
| **Kabwe District** | **39,862** | **202,360** | **98,781** | **103,579** |
| **Bwacha Constituency** | **16,468** | **83,721** | **41,357** | **42,364** |
| Muwowo | 342 | 1,792 | 897 | 895 |
| Muwowo East | 801 | 4,223 | 2,082 | 2,141 |
| Ngungu | 1,120 | 5,775 | 2,781 | 2,994 |
| Chimanimani | 867 | 4,787 | 2,272 | 2,515 |
| Bwacha | 1,773 | 9,565 | 4,566 | 4,999 |
| Kawama | 2,085 | 10,234 | 5,062 | 5,172 |
| Ben Kapufi | 1,082 | 4,998 | 2,550 | 2,448 |
| Makululu | 773 | 3,828 | 1,854 | 1,974 |
| Moomba | 2,219 | 11,038 | 5,509 | 5,529 |
| Zambezi | 1,561 | 8,230 | 4,011 | 4,219 |
| Kang'omba | 969 | 4,856 | 2,486 | 2,370 |
| Chinyanja | 594 | 3,261 | 1,620 | 1,641 |
| Munyama | 453 | 2,248 | 1,189 | 1,059 |
| Munga | 705 | 3,397 | 1,744 | 1,653 |
| Chililalila | 1,124 | 5,489 | 2,734 | 2,755 |
| **Kabwe Central** | **23,394** | **118,639** | **57,424** | **61,215** |
| Mpima | 1,228 | 6,588 | 3,332 | 3,256 |
| Luansase | 539 | 3,004 | 1,546 | 1,458 |
| Waya | 1,064 | 5,601 | 2,745 | 2,856 |
| Chirwa | 5,770 | 26,987 | 13,341 | 13,646 |
| Njanji | 1,131 | 6,076 | 2,758 | 3,318 |
| Justine Kabwe | 976 | 5,376 | 2,543 | 2,833 |
| David Ramushu | 1,516 | 8,148 | 3,970 | 4,178 |
| Highridge | 1,497 | 7,195 | 3,354 | 3,841 |

## Table A10: Households and Population by (De jure) Sex, Province, District, Constituency and Ward, Zambia 2010

| Province,District,Constituency and Ward | Households | Population | | |
|---|---|---|---|---|
| | | Total | Male | Female |
| Kalonga | 1,991 | 10,680 | 5,108 | 5,572 |
| Luangwa | 3,188 | 16,005 | 7,522 | 8,483 |
| Nakoli | 2,089 | 9,895 | 4,805 | 5,090 |
| Kaputula | 2,405 | 13,084 | 6,400 | 6,684 |
| **Kapiri Mposhi District** | **45,977** | **253,786** | **126,154** | **127,632** |
| **Kapiri Mposhi Constituency** | **45,977** | **253,786** | **126,154** | **127,632** |
| Ngabwe | 1,102 | 5,692 | 2,863 | 2,829 |
| Mukumbwe | 2,957 | 15,546 | 7,778 | 7,768 |
| Lwanchele | 2,622 | 15,117 | 7,443 | 7,674 |
| Chipepo | 2,563 | 14,323 | 7,176 | 7,147 |
| Kapandwe | 697 | 3,852 | 1,986 | 1,866 |
| Mpunde | 4,865 | 25,832 | 12,775 | 13,057 |
| Chibwelo | 8,742 | 44,783 | 21,805 | 22,978 |
| Kapiri Mposhi | 2,788 | 14,792 | 7,440 | 7,352 |
| Kashitu | 1,330 | 7,506 | 3,784 | 3,722 |
| Mushimbili | 3,315 | 18,634 | 9,270 | 9,364 |
| Lunchu | 5,649 | 32,138 | 15,992 | 16,146 |
| Chango'ndo | 4,073 | 23,935 | 12,037 | 11,898 |
| Kakwelesa | 4,254 | 25,556 | 12,779 | 12,777 |
| Kampumba | 1,020 | 6,080 | 3,026 | 3,054 |
| **Mkushi District** | **28,389** | **154,534** | **77,536** | **76,998** |
| **Mkushi North Constituency** | **23,980** | **128,986** | **64,607** | **64,379** |
| Masofu | 849 | 4,237 | 2,110 | 2,127 |
| Upper Lunsenfwa | 2,276 | 12,590 | 6,431 | 6,159 |
| Chalata | 2,570 | 14,422 | 7,049 | 7,373 |
| Chibefwe | 3,831 | 19,196 | 9,483 | 9,713 |
| Nkumbi | 2,293 | 12,415 | 6,183 | 6,232 |
| Mushibemba | 2,340 | 11,589 | 5,932 | 5,657 |
| Nshinso | 1,602 | 9,287 | 4,728 | 4,559 |
| Matuku | 2,808 | 16,131 | 7,963 | 8,168 |
| Tembwe | 2,180 | 9,855 | 5,016 | 4,839 |
| Munda | 2,652 | 15,730 | 7,935 | 7,795 |
| Chikanda | 579 | 3,534 | 1,777 | 1,757 |
| **Mkushi South Constituency** | **4,409** | **25,548** | **12,929** | **12,619** |
| Kalwa | 711 | 4,074 | 2,048 | 2,026 |
| Ching'ombe | 364 | 2,225 | 1,134 | 1,091 |
| Nkomashi | 1,877 | 10,453 | 5,311 | 5,142 |
| Kamimbya | 815 | 5,015 | 2,519 | 2,496 |
| Chapaba | 288 | 1,710 | 862 | 848 |
| Mwalala | 354 | 2,071 | 1,055 | 1,016 |
| **Mumbwa District** | **39,142** | **226,171** | **112,469** | **113,702** |
| **Mwembeshi Constituency** | **9,579** | **52,860** | **26,527** | **26,333** |
| Kapyanga | 877 | 5,466 | 2,686 | 2,780 |
| Chabota | 951 | 6,169 | 3,016 | 3,153 |
| Kalundu | 1,284 | 7,772 | 3,858 | 3,914 |
| Milandu | 2,169 | 11,398 | 5,702 | 5,696 |
| Makombwe | 1,008 | 5,332 | 2,622 | 2,710 |
| Nampundwe | 3,290 | 16,723 | 8,643 | 8,080 |
| **Mumbwa Constituency** | **15,911** | **88,698** | **44,065** | **44,633** |
| Nalusanga | 1,687 | 10,418 | 5,184 | 5,234 |
| Lutale | 1,862 | 10,319 | 5,073 | 5,246 |
| Kalwanyembe | 1,367 | 8,401 | 4,261 | 4,140 |
| Mpusu | 1,168 | 7,076 | 3,573 | 3,503 |
| Mumba | 2,533 | 14,624 | 7,337 | 7,287 |
| Mupona | 4,222 | 20,390 | 9,953 | 10,437 |
| Chibolyo | 999 | 5,677 | 2,780 | 2,897 |

## Table A10: Households and Population by (De jure) Sex, Province, District, Constituency and Ward, Zambia 2010

| Province,District,Constituency and Ward | Households | Population | | |
|---|---|---|---|---|
| | | Total | Male | Female |
| Nambala | 1,019 | 5,833 | 2,964 | 2,869 |
| Shimbizhi | 1,054 | 5,960 | 2,940 | 3,020 |
| **Nangoma Constituency** | **13,652** | **84,613** | **41,877** | **42,736** |
| Nakasaka | 2,114 | 12,855 | 6,510 | 6,345 |
| Nangoma | 2,486 | 15,502 | 7,560 | 7,942 |
| Myooye | 806 | 4,609 | 2,341 | 2,268 |
| Shichanzu | 2,013 | 12,038 | 5,876 | 6,162 |
| Nalubanda | 1,783 | 11,999 | 6,032 | 5,967 |
| Choma | 1,438 | 8,272 | 4,135 | 4,137 |
| Chisalu | 3,012 | 19,338 | 9,423 | 9,915 |
| **Serenje District** | **29,011** | **166,741** | **82,370** | **84,371** |
| **Chitambo Constituency** | **8,398** | **48,861** | **24,051** | **24,810** |
| Lulimala | 1,561 | 9,238 | 4,517 | 4,721 |
| Mpelembe | 337 | 2,203 | 1,067 | 1,136 |
| Chipundu | 1,238 | 7,555 | 3,768 | 3,787 |
| Luombwa | 371 | 1,945 | 923 | 1,022 |
| Chalilo | 1,322 | 7,669 | 3,810 | 3,859 |
| Chitambo | 1,737 | 9,843 | 4,836 | 5,007 |
| Muchinka | 1,832 | 10,408 | 5,130 | 5,278 |
| **Muchinga Constituency** | **9,136** | **53,448** | **26,458** | **26,990** |
| Mailo | 1,286 | 7,533 | 3,635 | 3,898 |
| Kanona | 1,108 | 6,557 | 3,245 | 3,312 |
| Serenje | 920 | 5,224 | 2,588 | 2,636 |
| Kabansa | 140 | 796 | 386 | 410 |
| Chisomo | 447 | 2,520 | 1,257 | 1,263 |
| Lukusashi | 623 | 3,781 | 1,904 | 1,877 |
| Sancha | 927 | 5,539 | 2,803 | 2,736 |
| Chibale | 1,338 | 7,982 | 3,963 | 4,019 |
| Masaninga | 2,347 | 13,516 | 6,677 | 6,839 |
| **Serenje Constituency** | **11,477** | **64,432** | **31,861** | **32,571** |
| Kabamba | 1,388 | 7,538 | 3,685 | 3,853 |
| Ibolelo | 4,204 | 22,642 | 11,135 | 11,507 |
| Muchinda | 2,884 | 16,443 | 8,181 | 8,262 |
| Ng'answa | 1,238 | 7,550 | 3,731 | 3,819 |
| Lupiya | 1,327 | 7,615 | 3,812 | 3,803 |
| Musangashi | 436 | 2,644 | 1,317 | 1,327 |
| **Copperbelt Province** | **371,125** | **1,972,317** | **981,887** | **990,430** |
| **Chililabombwe District** | **17,326** | **91,833** | **46,792** | **45,041** |
| **Chililabombwe Constituency** | **17,326** | **91,833** | **46,792** | **45,041** |
| Chilimina | 557 | 2,787 | 1,407 | 1,380 |
| Miyanda | 490 | 2,367 | 1,233 | 1,134 |
| Joseph Mwilwa | 660 | 3,807 | 1,941 | 1,866 |
| Anoya Zulu | 188 | 1,384 | 709 | 675 |
| Kawama | 262 | 1,329 | 689 | 640 |
| Miteta | 1,308 | 6,094 | 3,063 | 3,031 |
| Chitambi | 156 | 629 | 341 | 288 |
| Kakoso | 1,148 | 6,158 | 3,098 | 3,060 |
| Kafue | 819 | 3,796 | 2,001 | 1,795 |
| Mvula | 434 | 2,789 | 1,435 | 1,354 |
| Mathew Nkoloma | 958 | 5,471 | 2,850 | 2,621 |
| James Phiri | 1,356 | 7,496 | 3,908 | 3,588 |
| Silwizya | 163 | 809 | 421 | 388 |
| Helen Kaunda | 460 | 3,121 | 1,570 | 1,551 |
| Chitimukulu | 517 | 2,823 | 1,414 | 1,409 |
| Yeta | 770 | 4,502 | 2,291 | 2,211 |
| Ngebe | 828 | 4,697 | 2,447 | 2,250 |

| Table A10: Households and Population by (De jure) Sex, Province, District, Constituency and Ward, Zambia 2010 | | | | |
|---|---|---|---|---|
| Province,District,Constituency and Ward | Households | Population | | |
| | | Total | Male | Female |
| Kamima | 711 | 4,014 | 2,142 | 1,872 |
| Mukuka | 1,198 | 5,965 | 3,020 | 2,945 |
| Yotam Muleya | 2,509 | 12,209 | 6,059 | 6,150 |
| Nakatindi | 1,412 | 7,316 | 3,649 | 3,667 |
| Mumba | 422 | 2,270 | 1,104 | 1,166 |
| **Chingola District** | **39,657** | **216,626** | **108,464** | **108,162** |
| **Chingola Constituency** | **23,748** | **127,362** | **63,315** | **64,047** |
| Musenga | 540 | 2,772 | 1,392 | 1,380 |
| Kasompe | 1,051 | 5,191 | 2,583 | 2,608 |
| Mimbula | 1,274 | 6,652 | 3,342 | 3,310 |
| Lulamba | 2,478 | 15,080 | 7,621 | 7,459 |
| Twatasha | 1,484 | 8,235 | 4,158 | 4,077 |
| Gibson Chimfwembe | 672 | 3,964 | 2,002 | 1,962 |
| Chabanyama | 1,325 | 7,512 | 3,682 | 3,830 |
| Maiteneke | 2,145 | 11,705 | 5,814 | 5,891 |
| Chitimukulu | 1,514 | 7,904 | 3,856 | 4,048 |
| Chikola | 1,481 | 8,737 | 4,232 | 4,505 |
| Chiwempala | 2,384 | 12,454 | 6,145 | 6,309 |
| Kabungo | 1,381 | 6,966 | 3,492 | 3,474 |
| Kalilo | 1,328 | 6,219 | 3,215 | 3,004 |
| Ipafu | 1,127 | 5,819 | 2,906 | 2,913 |
| Muchinshi | 837 | 4,355 | 2,130 | 2,225 |
| Mutenda | 1,077 | 5,798 | 2,816 | 2,982 |
| Chingola | 1,650 | 7,999 | 3,929 | 4,070 |
| **Nchanga Constituency** | **15,909** | **89,264** | **45,149** | **44,115** |
| Kwacha | 1,153 | 5,027 | 2,618 | 2,409 |
| Nchanga | 984 | 5,266 | 2,711 | 2,555 |
| Sekela | 953 | 5,864 | 2,941 | 2,923 |
| Nsansa | 1,676 | 10,201 | 5,187 | 5,014 |
| Buntungwa | 1,810 | 12,193 | 6,271 | 5,922 |
| Kabundi | 1,254 | 7,102 | 3,567 | 3,535 |
| Kasala | 1,040 | 7,031 | 3,517 | 3,514 |
| Kapisha | 6,370 | 33,377 | 16,607 | 16,770 |
| Luano | 202 | 814 | 454 | 360 |
| Bupalo | 467 | 2,389 | 1,276 | 1,113 |
| **Kalulushi District** | **19,203** | **100,381** | **50,164** | **50,217** |
| **Kalulushi Constituency** | **19,203** | **100,381** | **50,164** | **50,217** |
| Musakashi | 177 | 994 | 485 | 509 |
| Chambishi | 20 | 90 | 49 | 41 |
| Twaiteka | 4,541 | 24,062 | 12,054 | 12,008 |
| Lukoshi | 312 | 1,518 | 754 | 764 |
| Lulamba | 933 | 4,486 | 2,309 | 2,177 |
| Mwambashi | 772 | 3,415 | 1,802 | 1,613 |
| Ichimpe | 737 | 3,457 | 1,804 | 1,653 |
| Kalanga | 1,492 | 7,529 | 3,678 | 3,851 |
| Lubuto | 636 | 4,071 | 1,949 | 2,122 |
| Kalungwishi | 631 | 3,501 | 1,723 | 1,778 |
| Luapula | 544 | 3,112 | 1,524 | 1,588 |
| Ngweshi | 609 | 3,217 | 1,576 | 1,641 |
| Kalengwa | 356 | 1,921 | 915 | 1,006 |
| Chibuluma | 681 | 3,358 | 1,658 | 1,700 |
| Kankonshi | 284 | 1,418 | 702 | 716 |
| Remmy Chisupa | 829 | 4,178 | 2,120 | 2,058 |
| Buseko | 729 | 3,601 | 1,753 | 1,848 |
| Dongwe | 261 | 1,557 | 782 | 775 |
| Kafue | 2,045 | 11,187 | 5,463 | 5,724 |

## Table A10: Households and Population by (De jure) Sex, Province, District, Constituency and Ward, Zambia 2010

| Province,District,Constituency and Ward | Households | Population | | |
|---|---|---|---|---|
| | | Total | Male | Female |
| Chankalamo | 621 | 3,100 | 1,602 | 1,498 |
| Chati | 1,567 | 8,589 | 4,414 | 4,175 |
| Chembe | 426 | 2,020 | 1,048 | 972 |
| **Kitwe District** | **96,666** | **517,543** | **256,740** | **260,803** |
| **Chimwemwe Constituency** | **21,742** | **117,341** | **57,740** | **59,601** |
| Itimpi | 2,306 | 11,777 | 5,931 | 5,846 |
| Twatasha | 4,614 | 23,719 | 11,640 | 12,079 |
| Kawama | 7,273 | 37,966 | 18,861 | 19,105 |
| Buntungwa | 3,063 | 17,483 | 8,469 | 9,014 |
| Lubuto | 2,044 | 12,203 | 5,931 | 6,272 |
| Chimwemwe | 2,442 | 14,193 | 6,908 | 7,285 |
| **Kamfinsa Constituency** | **16,473** | **86,834** | **42,921** | **43,913** |
| Bupe | 2,974 | 17,442 | 8,513 | 8,929 |
| Ndeke | 9,581 | 50,300 | 24,696 | 25,604 |
| Kafue | 1,476 | 7,051 | 3,544 | 3,507 |
| Kamfinsa | 2,442 | 12,041 | 6,168 | 5,873 |
| **Kwacha Constituency** | **25,539** | **133,155** | **66,276** | **66,879** |
| Kwacha | 3,227 | 18,362 | 9,142 | 9,220 |
| Bulangililo | 5,066 | 28,038 | 13,650 | 14,388 |
| Ipusukilo | 8,430 | 42,784 | 21,625 | 21,159 |
| Chantete | 684 | 3,091 | 1,576 | 1,515 |
| Riverside | 5,601 | 28,359 | 13,977 | 14,382 |
| Lubwa | 2,531 | 12,521 | 6,306 | 6,215 |
| **Nkana Constituency** | **15,757** | **83,067** | **41,322** | **41,745** |
| Rokana | 2,568 | 11,547 | 5,721 | 5,826 |
| Parklands | 1,487 | 6,086 | 3,013 | 3,073 |
| Buchi | 4,272 | 24,193 | 12,032 | 12,161 |
| Mukuba | 1,191 | 5,753 | 2,771 | 2,982 |
| Miseshi | 2,913 | 17,535 | 8,773 | 8,762 |
| Mindolo | 2,493 | 14,053 | 7,052 | 7,001 |
| Kamakonde | 833 | 3,900 | 1,960 | 1,940 |
| **Wusakile Constituency** | **17,155** | **97,146** | **48,481** | **48,665** |
| Limaposa | 438 | 2,192 | 1,084 | 1,108 |
| Luangwa | 5,704 | 30,626 | 15,256 | 15,370 |
| Chamboli | 3,598 | 22,054 | 11,163 | 10,891 |
| Chibote | 2,752 | 16,748 | 8,332 | 8,416 |
| Wusakile | 4,663 | 25,526 | 12,646 | 12,880 |
| **Luanshya District** | **29,043** | **156,059** | **77,368** | **78,691** |
| **Luanshya Constituency** | **19,037** | **98,498** | **48,744** | **49,754** |
| Misaka | 2,237 | 10,871 | 5,558 | 5,313 |
| Fisenge | 1,511 | 7,057 | 3,584 | 3,473 |
| Twashuka | 190 | 778 | 418 | 360 |
| Chitwi | 482 | 2,249 | 1,118 | 1,131 |
| Chifulube | 194 | 794 | 425 | 369 |
| Buntungwa | 3,002 | 15,323 | 7,614 | 7,709 |
| Mpelembe | 1,194 | 6,263 | 3,073 | 3,190 |
| Buteko | 1,940 | 10,281 | 5,008 | 5,273 |
| James Phiri | 1,660 | 8,478 | 4,154 | 4,324 |
| Levi Chito | 1,329 | 7,254 | 3,546 | 3,708 |
| Mikomfwa | 2,140 | 11,573 | 5,709 | 5,864 |
| Zambezi | 1,260 | 6,668 | 3,227 | 3,441 |
| Mulungushi | 933 | 5,428 | 2,646 | 2,782 |
| Mipundu | 965 | 5,481 | 2,664 | 2,817 |
| **Roan Constituency** | **10,006** | **57,561** | **28,624** | **28,937** |
| Kafubu | 544 | 3,120 | 1,547 | 1,573 |

## Table A10: Households and Population by (De jure) Sex, Province, District, Constituency and Ward, Zambia 2010

| Province,District,Constituency and Ward | Households | Population | | |
|---|---|---|---|---|
| | | Total | Male | Female |
| Nkoloma | 470 | 2,779 | 1,395 | 1,384 |
| Lumumba | 1,249 | 7,235 | 3,524 | 3,711 |
| Kafue | 1,461 | 8,206 | 4,059 | 4,147 |
| Chilabula | 374 | 1,772 | 909 | 863 |
| Mpatamatu | 934 | 5,289 | 2,563 | 2,726 |
| Justine Kabwe | 751 | 4,347 | 2,143 | 2,204 |
| Nkulumashiba | 490 | 2,871 | 1,423 | 1,448 |
| Baluba | 540 | 3,505 | 1,763 | 1,742 |
| Milyashi | 1,193 | 6,502 | 3,245 | 3,257 |
| Ngebe | 624 | 4,280 | 2,129 | 2,151 |
| Kansengu | 785 | 5,193 | 2,604 | 2,589 |
| Kawama | 493 | 1,976 | 1,062 | 914 |
| Muva Hill | 98 | 486 | 258 | 228 |
| **Lufwanyama District** | **15,597** | **78,503** | **39,182** | **39,321** |
| **Lufwanyama Constituency** | **15,597** | **78,503** | **39,182** | **39,321** |
| Kansanta | 1,237 | 6,541 | 3,209 | 3,332 |
| Kabundia | 1,297 | 6,786 | 3,325 | 3,461 |
| Boso | 1,362 | 6,180 | 3,087 | 3,093 |
| Kansonka | 1,267 | 6,360 | 3,151 | 3,209 |
| Kafubu | 1,375 | 6,804 | 3,446 | 3,358 |
| Chibanga | 2,496 | 12,126 | 5,993 | 6,133 |
| Sokotwe | 972 | 4,805 | 2,423 | 2,382 |
| Mibenge | 718 | 3,414 | 1,849 | 1,565 |
| Chantete | 172 | 860 | 456 | 404 |
| Bulaya | 116 | 563 | 294 | 269 |
| Mukumbo | 870 | 4,491 | 2,296 | 2,195 |
| Mwelushi | 817 | 4,352 | 2,211 | 2,141 |
| Mpindi | 704 | 3,766 | 1,830 | 1,936 |
| Lufwanyama | 641 | 3,240 | 1,589 | 1,651 |
| Luswishi | 692 | 3,628 | 1,788 | 1,840 |
| Mushingashi | 861 | 4,587 | 2,235 | 2,352 |
| **Masaiti District** | **20,511** | **103,857** | **52,017** | **51,840** |
| **Kafulafuta Constituency** | **8,402** | **43,069** | **21,646** | **21,423** |
| Mwatishi | 1,190 | 5,748 | 2,902 | 2,846 |
| Majaliwa | 1,352 | 6,892 | 3,465 | 3,427 |
| Chondwe | 1,884 | 9,346 | 4,637 | 4,709 |
| Mutaba | 1,261 | 6,797 | 3,404 | 3,393 |
| Miengwe | 1,661 | 8,730 | 4,370 | 4,360 |
| Katonte | 119 | 823 | 434 | 389 |
| Ishitwe | 935 | 4,733 | 2,434 | 2,299 |
| **Masaiti Constituency** | **12,109** | **60,788** | **30,371** | **30,417** |
| Kashitu | 1,482 | 7,520 | 3,789 | 3,731 |
| Luansobe | 989 | 5,543 | 2,834 | 2,709 |
| Chinondo | 610 | 3,373 | 1,713 | 1,660 |
| Mishikishi | 1,184 | 6,099 | 2,928 | 3,171 |
| Masangano | 1,856 | 9,073 | 4,535 | 4,538 |
| Lumano | 1,789 | 8,580 | 4,352 | 4,228 |
| Katuba | 1,111 | 5,674 | 2,850 | 2,824 |
| Shimibanga | 1,408 | 7,103 | 3,474 | 3,629 |
| Chilulu | 694 | 3,318 | 1,620 | 1,698 |
| Miputu | 986 | 4,505 | 2,276 | 2,229 |
| **Mpongwe District** | **17,350** | **93,380** | **46,785** | **46,595** |
| **Mpongwe Constituency** | **17,350** | **93,380** | **46,785** | **46,595** |
| Luswishi | 287 | 1,718 | 820 | 898 |
| Kasonga | 434 | 2,266 | 1,148 | 1,118 |
| Munkumpu | 1,629 | 8,509 | 4,319 | 4,190 |

## Table A10: Households and Population by (De jure) Sex, Province, District, Constituency and Ward, Zambia 2010

| Province,District,Constituency and Ward | Households | Population | | |
|---|---|---|---|---|
| | | Total | Male | Female |
| Kashiba | 1,731 | 9,536 | 4,767 | 4,769 |
| Mpongwe | 2,091 | 11,130 | 5,494 | 5,636 |
| Kanyenda | 1,668 | 8,635 | 4,373 | 4,262 |
| Kasamba | 370 | 1,895 | 981 | 914 |
| Ibenga | 2,831 | 15,747 | 7,710 | 8,037 |
| Kalweo | 1,344 | 7,878 | 3,990 | 3,888 |
| Mikata | 1,830 | 10,313 | 5,189 | 5,124 |
| Nampamba | 2,568 | 12,650 | 6,350 | 6,300 |
| Musofu | 567 | 3,103 | 1,644 | 1,459 |
| **Mufulira District** | **30,065** | **162,889** | **81,355** | **81,534** |
| **Kankoyo Constituency** | **8,193** | **45,258** | **22,754** | **22,504** |
| John Kampengele | 1,126 | 5,789 | 2,847 | 2,942 |
| Buntungwa | 787 | 4,178 | 2,038 | 2,140 |
| Kangwa Nsuluka | 249 | 1,079 | 595 | 484 |
| Luansobe | 1,333 | 6,654 | 3,342 | 3,312 |
| Butondo | 1,324 | 8,400 | 4,238 | 4,162 |
| Kwacha | 860 | 5,354 | 2,691 | 2,663 |
| Fibusa | 1,029 | 6,078 | 3,050 | 3,028 |
| Mpelembe | 1,485 | 7,726 | 3,953 | 3,773 |
| **Kantanshi Constituency** | **10,117** | **56,378** | **28,315** | **28,063** |
| Minambe | 712 | 3,695 | 1,784 | 1,911 |
| Murundu | 1,521 | 7,635 | 3,832 | 3,803 |
| Francis Mukuka | 1,471 | 7,829 | 3,982 | 3,847 |
| Maina Soko | 979 | 4,812 | 2,360 | 2,452 |
| Bwafwano | 761 | 4,087 | 2,013 | 2,074 |
| Mulungushi | 1,434 | 8,806 | 4,472 | 4,334 |
| Shinde | 1,733 | 10,259 | 5,219 | 5,040 |
| Bwembya Silwizya | 553 | 3,591 | 1,809 | 1,782 |
| Leya Mukutu | 627 | 4,134 | 2,082 | 2,052 |
| David Lunda | 326 | 1,530 | 762 | 768 |
| **Mufurila Constituency** | **11,755** | **61,253** | **30,286** | **30,967** |
| Mutundu | 1,038 | 4,812 | 2,518 | 2,294 |
| Bwananyina | 2,321 | 11,432 | 5,762 | 5,670 |
| David Kaunda | 803 | 4,461 | 2,180 | 2,281 |
| Chachacha | 1,860 | 10,414 | 5,014 | 5,400 |
| Kamuchanga | 753 | 4,207 | 1,981 | 2,226 |
| Kasempa | 792 | 4,549 | 2,243 | 2,306 |
| Kansuswa | 1,133 | 5,884 | 2,900 | 2,984 |
| Kafue | 198 | 998 | 519 | 479 |
| Kawama | 1,441 | 6,953 | 3,464 | 3,489 |
| Hanky Kalanga | 1,416 | 7,543 | 3,705 | 3,838 |
| **Ndola District** | **85,707** | **451,246** | **223,020** | **228,226** |
| **Bwana Mkumbwa Constituency** | **21,912** | **118,325** | **58,808** | **59,517** |
| Itawa | 3,908 | 20,813 | 10,229 | 10,584 |
| Munkulungwe | 3,312 | 16,725 | 8,498 | 8,227 |
| Twashuka | 2,725 | 14,055 | 7,111 | 6,944 |
| Kavu | 1,011 | 4,995 | 2,531 | 2,464 |
| Mushili | 8,151 | 46,509 | 22,952 | 23,557 |
| Chichele | 1,449 | 7,987 | 3,976 | 4,011 |
| Kantolomba | 1,356 | 7,241 | 3,511 | 3,730 |
| **Chifubu Constituency** | **18,073** | **98,677** | **48,652** | **50,025** |
| Pamodzi | 7,306 | 40,186 | 19,707 | 20,479 |
| Kawama | 3,402 | 18,425 | 9,207 | 9,218 |
| Fibobe | 2,687 | 15,011 | 7,355 | 7,656 |
| Chifubu | 2,464 | 13,307 | 6,543 | 6,764 |
| Kamba | 2,214 | 11,748 | 5,840 | 5,908 |

## Table A10: Households and Population by (De jure) Sex, Province, District, Constituency and Ward, Zambia 2010

| Province,District,Constituency and Ward | Households | Population | | |
|---|---|---|---|---|
| | | Total | Male | Female |
| **Kabushi Constituency** | **17,048** | **93,918** | **46,035** | **47,883** |
| Lubuto | 4,057 | 22,915 | 11,220 | 11,695 |
| Mukuba | 1,843 | 9,686 | 4,764 | 4,922 |
| Toka | 1,501 | 8,186 | 3,966 | 4,220 |
| Kaloko | 1,686 | 9,032 | 4,375 | 4,657 |
| Kabushi | 2,251 | 12,363 | 6,126 | 6,237 |
| Kafubu | 1,353 | 6,892 | 3,430 | 3,462 |
| Skyways | 2,798 | 15,785 | 7,663 | 8,122 |
| Masala | 1,559 | 9,059 | 4,491 | 4,568 |
| **Ndola Central Constituency** | **28,674** | **140,326** | **69,525** | **70,801** |
| Kansenshi | 2,397 | 11,693 | 5,567 | 6,126 |
| Nkwazi | 4,558 | 22,218 | 11,164 | 11,054 |
| Yengwe | 3,406 | 16,669 | 8,001 | 8,668 |
| Chipulukusu | 7,424 | 37,703 | 18,859 | 18,844 |
| Kanini | 2,511 | 11,299 | 5,380 | 5,919 |
| Twapia | 5,899 | 29,683 | 14,759 | 14,924 |
| Dag Hammerskjoeld | 2,182 | 9,729 | 5,109 | 4,620 |
| Kaniki | 297 | 1,332 | 686 | 646 |
| **Eastern Province** | **305,198** | **1,592,661** | **784,680** | **807,981** |
| **Chadiza District** | **19,822** | **107,327** | **53,404** | **53,923** |
| **Chadiza Constituency** | **11,218** | **62,742** | **31,064** | **31,678** |
| Mangwe | 1,695 | 10,318 | 5,187 | 5,131 |
| Nsadzu | 992 | 5,773 | 2,860 | 2,913 |
| Manje | 1,241 | 6,846 | 3,378 | 3,468 |
| Chanjowe | 447 | 2,633 | 1,369 | 1,264 |
| Kapachi | 737 | 4,029 | 1,992 | 2,037 |
| Chadiza | 1,020 | 5,670 | 2,797 | 2,873 |
| Chilenga | 816 | 4,491 | 2,195 | 2,296 |
| Naviluri | 1,133 | 6,350 | 3,125 | 3,225 |
| Chamandala | 806 | 4,129 | 1,998 | 2,131 |
| Kandabwako | 700 | 4,050 | 1,963 | 2,087 |
| Kampini | 851 | 4,405 | 2,186 | 2,219 |
| Tafelansoni | 780 | 4,048 | 2,014 | 2,034 |
| **Vubwi Constituency** | **8,604** | **44,585** | **22,340** | **22,245** |
| Ambidzi | 600 | 2,924 | 1,472 | 1,452 |
| Kabvumo | 487 | 2,638 | 1,320 | 1,318 |
| Khumba | 658 | 3,555 | 1,786 | 1,769 |
| Mwangazi | 966 | 4,978 | 2,531 | 2,447 |
| Mbozi | 766 | 4,154 | 2,050 | 2,104 |
| Vubwi | 1,612 | 8,561 | 4,254 | 4,307 |
| Chisiya | 468 | 2,450 | 1,235 | 1,215 |
| Chimphanje | 457 | 2,332 | 1,179 | 1,153 |
| Mlawe | 906 | 4,688 | 2,350 | 2,338 |
| Zozwe | 1,684 | 8,305 | 4,163 | 4,142 |
| **Chipata District** | **88,065** | **455,783** | **224,934** | **230,849** |
| **Chipangali Constituency** | **23,636** | **122,916** | **61,360** | **61,556** |
| Sisinje | 3,400 | 18,656 | 9,263 | 9,393 |
| Nthope | 6,421 | 32,993 | 16,612 | 16,381 |
| Chipangali | 3,787 | 20,558 | 10,276 | 10,282 |
| Kasenga | 2,738 | 14,350 | 7,089 | 7,261 |
| Rukuzye | 3,898 | 19,936 | 9,917 | 10,019 |
| Msandile | 3,392 | 16,423 | 8,203 | 8,220 |
| **Chipata Central Constituency** | **31,179** | **159,325** | **78,547** | **80,778** |
| Msanga | 12,948 | 66,455 | 32,696 | 33,759 |
| Kanjala | 6,990 | 35,577 | 17,640 | 17,937 |
| Dilika | 4,634 | 23,324 | 11,762 | 11,562 |
| Kapata | 6,607 | 33,969 | 16,449 | 17,520 |

## Table A10: Households and Population by (De jure) Sex, Province, District, Constituency and Ward, Zambia 2010

| Province,District,Constituency and Ward | Households | Population | | |
|---|---|---|---|---|
| | | Total | Male | Female |
| **Kasenengwa Constituency** | **18,401** | **98,117** | **47,911** | **50,206** |
| Makungwa | 2,609 | 12,901 | 6,229 | 6,672 |
| Chingazi | 2,807 | 15,149 | 7,309 | 7,840 |
| Kwenje | 5,288 | 29,330 | 14,389 | 14,941 |
| Ng'ongwe | 2,856 | 15,425 | 7,527 | 7,898 |
| Mkowe | 375 | 2,009 | 1,011 | 998 |
| Mboza | 2,195 | 11,792 | 5,827 | 5,965 |
| Chiparamba | 2,271 | 11,511 | 5,619 | 5,892 |
| **Luangeni Constituency** | **14,849** | **75,425** | **37,116** | **38,309** |
| Nsingo | 4,170 | 20,376 | 10,090 | 10,286 |
| Khova | 1,988 | 10,590 | 5,274 | 5,316 |
| Makangila | 1,562 | 7,559 | 3,754 | 3,805 |
| Chikando | 4,927 | 25,424 | 12,389 | 13,035 |
| Kazimule | 2,202 | 11,476 | 5,609 | 5,867 |
| **Katete District** | **46,852** | **243,849** | **119,995** | **123,854** |
| **Milanzi Constituency** | **12,255** | **65,079** | **32,053** | **33,026** |
| Kafumbwe | 1,409 | 7,434 | 3,672 | 3,762 |
| Kazala | 1,632 | 8,597 | 4,273 | 4,324 |
| Milanzi | 1,031 | 5,532 | 2,724 | 2,808 |
| Kapoche | 459 | 2,402 | 1,221 | 1,181 |
| Chindwale | 917 | 4,576 | 2,205 | 2,371 |
| Kapangulula | 1,846 | 9,113 | 4,468 | 4,645 |
| Dole | 1,752 | 8,963 | 4,363 | 4,600 |
| Chimwa | 332 | 1,813 | 874 | 939 |
| Mwandafisi | 1,015 | 5,913 | 2,910 | 3,003 |
| Katiula | 1,862 | 10,736 | 5,343 | 5,393 |
| **Mkaika Constituency** | **18,431** | **95,906** | **47,344** | **48,562** |
| Mphangwe | 3,785 | 19,090 | 9,379 | 9,711 |
| Chavuka | 1,977 | 10,208 | 5,013 | 5,195 |
| Kadula | 1,748 | 9,329 | 4,602 | 4,727 |
| Vulamkoko | 2,063 | 10,762 | 5,337 | 5,425 |
| Chimtende | 1,777 | 9,786 | 4,815 | 4,971 |
| Lukweta | 1,142 | 6,131 | 3,078 | 3,053 |
| Mkaika | 4,733 | 24,171 | 11,961 | 12,210 |
| Matunga | 1,206 | 6,429 | 3,159 | 3,270 |
| **Sinda Constituency** | **16,166** | **82,864** | **40,598** | **42,266** |
| Kamwaza | 1,434 | 7,144 | 3,441 | 3,703 |
| Nchingilizya | 996 | 5,133 | 2,556 | 2,577 |
| Luandazi | 1,593 | 7,833 | 3,812 | 4,021 |
| Chiwuyu | 1,741 | 8,482 | 4,155 | 4,327 |
| Sinda | 2,699 | 13,457 | 6,550 | 6,907 |
| Mnyamanzi | 1,060 | 5,459 | 2,673 | 2,786 |
| Nyamasonkho | 1,961 | 10,274 | 5,121 | 5,153 |
| Mng'omba | 2,041 | 10,572 | 5,166 | 5,406 |
| Kasangazi | 1,868 | 10,184 | 5,014 | 5,170 |
| Chitawe | 773 | 4,326 | 2,110 | 2,216 |
| **Lundazi District** | **62,069** | **323,870** | **158,379** | **165,491** |
| **Chasefu Constituency** | **19,289** | **99,828** | **48,810** | **51,018** |
| Manda Hill | 805 | 4,371 | 2,159 | 2,212 |
| Magodi | 3,375 | 17,762 | 8,682 | 9,080 |
| Susa | 1,936 | 10,576 | 5,177 | 5,399 |
| Luwerezi | 929 | 4,961 | 2,453 | 2,508 |
| Kajilime | 3,526 | 18,363 | 9,035 | 9,328 |
| Kapilisanga | 2,085 | 10,169 | 4,890 | 5,279 |
| Nkhanga | 3,947 | 20,060 | 9,749 | 10,311 |
| Membe | 986 | 5,044 | 2,464 | 2,580 |
| Chaboli | 1,700 | 8,522 | 4,201 | 4,321 |

## Table A10: Households and Population by (De jure) Sex, Province, District, Constituency and Ward, Zambia 2010

| Province,District,Constituency and Ward | Households | Population | | |
|---|---|---|---|---|
| | | Total | Male | Female |
| **Lumezi Constituency** | **17,805** | **93,717** | **45,987** | **47,730** |
| Chamtowa | 2,546 | 12,923 | 6,390 | 6,533 |
| Kachama | 1,709 | 9,119 | 4,547 | 4,572 |
| Wachitangachi | 1,535 | 7,960 | 3,852 | 4,108 |
| Kamimba | 2,153 | 11,306 | 5,460 | 5,846 |
| Kazembe | 1,724 | 8,780 | 4,209 | 4,571 |
| Lumimba | 1,654 | 8,679 | 4,197 | 4,482 |
| Lukusuzi | 438 | 2,155 | 1,063 | 1,092 |
| Diwa | 4,143 | 23,410 | 11,675 | 11,735 |
| Chibande | 1,903 | 9,385 | 4,594 | 4,791 |
| **Lundazi Constituency** | **24,975** | **130,325** | **63,582** | **66,743** |
| Vuu | 3,815 | 19,976 | 9,854 | 10,122 |
| Mnyamazi | 6,653 | 34,442 | 16,806 | 17,636 |
| Ndonda | 1,815 | 9,606 | 4,667 | 4,939 |
| Msuzi | 3,184 | 16,622 | 8,082 | 8,540 |
| Mkomba | 988 | 5,097 | 2,520 | 2,577 |
| Chilola | 2,045 | 10,195 | 4,972 | 5,223 |
| Chimaliro | 2,017 | 10,180 | 4,969 | 5,211 |
| Nthintimila | 1,482 | 8,241 | 4,029 | 4,212 |
| Lunevwa | 2,976 | 15,966 | 7,683 | 8,283 |
| **Mambwe District** | **13,196** | **68,918** | **34,253** | **34,665** |
| **Mambwe Constituency** | **13,196** | **68,918** | **34,253** | **34,665** |
| Nsefu | 1,562 | 8,019 | 3,973 | 4,046 |
| Jumbe | 1,001 | 5,113 | 2,518 | 2,595 |
| Chipapa | 965 | 5,014 | 2,514 | 2,500 |
| Mphomwa | 1,509 | 8,413 | 4,249 | 4,164 |
| Chikowa | 898 | 4,907 | 2,413 | 2,494 |
| Mnkhanya | 1,349 | 7,094 | 3,532 | 3,562 |
| Kakumbi | 2,872 | 14,714 | 7,284 | 7,430 |
| Ncheka | 97 | 561 | 296 | 265 |
| Malama | 99 | 429 | 219 | 210 |
| Mdima | 1,936 | 9,772 | 4,819 | 4,953 |
| Msoro | 147 | 848 | 439 | 409 |
| Kasamanda | 558 | 2,846 | 1,411 | 1,435 |
| Nyakatokoli | 203 | 1,188 | 586 | 602 |
| **Nyimba District** | **16,040** | **85,025** | **41,992** | **43,033** |
| **Nyimba Constituency** | **16,040** | **85,025** | **41,992** | **43,033** |
| Chinsimbwe | 179 | 857 | 429 | 428 |
| Katipa | 213 | 980 | 494 | 486 |
| Vizimumba | 2,489 | 13,604 | 6,769 | 6,835 |
| Ngozi | 1,756 | 9,146 | 4,524 | 4,622 |
| Luezi | 1,023 | 5,290 | 2,581 | 2,709 |
| Mtilizi | 1,110 | 5,899 | 2,935 | 2,964 |
| Nyimba | 1,721 | 9,148 | 4,475 | 4,673 |
| Kaliwe | 1,567 | 8,651 | 4,269 | 4,382 |
| Chiweza | 2,143 | 11,086 | 5,509 | 5,577 |
| Mombe | 959 | 4,889 | 2,349 | 2,540 |
| Chamilala | 771 | 3,970 | 1,999 | 1,971 |
| Chinambi | 1,683 | 9,213 | 4,560 | 4,653 |
| Luangwa | 426 | 2,292 | 1,099 | 1,193 |

## Table A10: Households and Population by (De jure) Sex, Province, District, Constituency and Ward, Zambia 2010

| Province,District,Constituency and Ward | Households | Population | | |
|---|---|---|---|---|
| | | Total | Male | Female |
| **Petauke District** | **59,154** | **307,889** | **151,723** | **156,166** |
| **Kapoche Constituency** | **22,918** | **120,179** | **58,997** | **61,182** |
| Kapoche | 2,153 | 10,640 | 5,206 | 5,434 |
| Ching'ombe | 3,124 | 15,939 | 7,842 | 8,097 |
| Mwangaila | 3,709 | 18,858 | 9,277 | 9,581 |
| Matambazi | 3,873 | 21,396 | 10,594 | 10,802 |
| Kaumbwe | 3,234 | 17,326 | 8,442 | 8,884 |
| Lusinde | 2,155 | 11,243 | 5,499 | 5,744 |
| Manjazi | 1,690 | 9,131 | 4,439 | 4,692 |
| Manyane | 2,980 | 15,646 | 7,698 | 7,948 |
| **Msanzala Constituency** | **23,655** | **121,429** | **59,819** | **61,610** |
| Msumbazi | 3,282 | 16,964 | 8,394 | 8,570 |
| Ongolwe | 3,090 | 16,133 | 7,973 | 8,160 |
| Kovyane | 2,394 | 12,307 | 5,968 | 6,339 |
| Mbala | 2,232 | 11,533 | 5,619 | 5,914 |
| Nsimbo | 1,612 | 8,468 | 4,213 | 4,255 |
| Nyika | 7,502 | 38,001 | 18,708 | 19,293 |
| Chimanyama | 3,543 | 18,023 | 8,944 | 9,079 |
| **Petauke Constituency** | **12,581** | **66,281** | **32,907** | **33,374** |
| Nyakawise | 2,038 | 10,788 | 5,247 | 5,541 |
| Singozi | 2,412 | 12,802 | 6,322 | 6,480 |
| Mateyo Mzeka | 1,960 | 10,111 | 5,003 | 5,108 |
| Mawanda | 2,367 | 11,809 | 5,879 | 5,930 |
| Lusangazi | 438 | 2,216 | 1,112 | 1,104 |
| Ukwimi | 1,800 | 9,945 | 5,000 | 4,945 |
| Chisangu | 1,566 | 8,610 | 4,344 | 4,266 |
| **Luapula Province** | **194,962** | **991,927** | **488,589** | **503,338** |
| **Chienge District** | **24,415** | **114,225** | **56,542** | **57,683** |
| **Chienge Constituency** | **24,415** | **114,225** | **56,542** | **57,683** |
| Lunchinda | 1,773 | 7,486 | 3,767 | 3,719 |
| Chipungu | 3,458 | 15,241 | 7,443 | 7,798 |
| Luau | 1,587 | 6,632 | 3,271 | 3,361 |
| Lambwe Chomba | 963 | 3,882 | 1,943 | 1,939 |
| Chienge | 2,100 | 9,161 | 4,564 | 4,597 |
| Katete | 2,222 | 11,092 | 5,484 | 5,608 |
| Ifuna | 1,625 | 7,895 | 3,981 | 3,914 |
| Chipamba | 3,251 | 15,938 | 7,879 | 8,059 |
| Kalobwa | 1,023 | 4,801 | 2,381 | 2,420 |
| Chitutu | 1,321 | 6,491 | 3,214 | 3,277 |
| Munwa | 2,108 | 10,529 | 5,186 | 5,343 |
| Kulungwishi | 1,185 | 5,942 | 2,890 | 3,052 |
| Mununga | 1,799 | 9,135 | 4,539 | 4,596 |
| **Kawambwa District** | **25,196** | **134,414** | **66,091** | **68,323** |
| **Kawambwa Constituency** | **8,639** | **47,511** | **23,466** | **24,045** |
| Senga | 2,004 | 11,172 | 5,470 | 5,702 |
| Luena | 490 | 2,891 | 1,414 | 1,477 |
| Ntumbachushi | 392 | 2,187 | 1,076 | 1,111 |
| Kawambwa | 1,734 | 9,415 | 4,619 | 4,796 |
| Ng'ona | 2,197 | 12,085 | 5,994 | 6,091 |
| Fisaka | 957 | 5,252 | 2,613 | 2,639 |
| Iyanga | 865 | 4,509 | 2,280 | 2,229 |
| **Mwansabombwe Constituency** | **8,803** | **45,294** | **22,008** | **23,286** |
| Mununshi | 953 | 4,820 | 2,293 | 2,527 |
| Mulele | 2,116 | 10,854 | 5,261 | 5,593 |
| Lufubu | 643 | 3,304 | 1,650 | 1,654 |
| Mwansabombwe | 1,048 | 5,495 | 2,599 | 2,896 |

## Table A10: Households and Population by (De jure) Sex, Province, District, Constituency and Ward, Zambia 2010

| Province,District,Constituency and Ward | Households | Population | | |
|---|---|---|---|---|
| | | Total | Male | Female |
| Kakose | 1,053 | 5,306 | 2,543 | 2,763 |
| Chipita | 148 | 752 | 378 | 374 |
| Kayo | 1,592 | 8,406 | 4,155 | 4,251 |
| Mbereshi | 1,250 | 6,357 | 3,129 | 3,228 |
| **Pambashe Constituency** | **7,754** | **41,609** | **20,617** | **20,992** |
| Kabanse | 1,259 | 7,050 | 3,494 | 3,556 |
| Ilombe | 2,290 | 12,103 | 5,898 | 6,205 |
| Mulunda | 2,016 | 10,859 | 5,350 | 5,509 |
| Chibote | 545 | 2,773 | 1,402 | 1,371 |
| Chimpili | 463 | 2,506 | 1,237 | 1,269 |
| Luongo | 727 | 3,948 | 2,040 | 1,908 |
| Pambashe | 454 | 2,370 | 1,196 | 1,174 |
| **Mansa District** | **43,631** | **228,392** | **112,336** | **116,056** |
| **Bahati Constituency** | **17,823** | **92,040** | **45,676** | **46,364** |
| Mutuna | 2,660 | 13,998 | 6,969 | 7,029 |
| Misakalala | 1,993 | 10,473 | 5,271 | 5,202 |
| Kaole | 639 | 3,079 | 1,550 | 1,529 |
| Mushipashi | 1,630 | 8,392 | 4,119 | 4,273 |
| Mansa | 5,661 | 29,039 | 14,300 | 14,739 |
| Myulu | 3,764 | 19,532 | 9,728 | 9,804 |
| Muleshi | 1,476 | 7,527 | 3,739 | 3,788 |
| **Mansa Central Constituency** | **25,808** | **136,352** | **66,660** | **69,692** |
| Muchinka | 6,235 | 32,985 | 16,101 | 16,884 |
| Mulelenshi | 3,951 | 21,533 | 10,427 | 11,106 |
| Lukangaba | 3,409 | 17,988 | 8,906 | 9,082 |
| Chilyapa | 1,158 | 6,034 | 2,911 | 3,123 |
| Chansusu | 1,886 | 9,695 | 4,697 | 4,998 |
| Lwingishi | 3,082 | 16,532 | 8,173 | 8,359 |
| Chibeleka | 1,584 | 8,191 | 3,982 | 4,209 |
| Lukola | 1,333 | 7,231 | 3,594 | 3,637 |
| Luapula | 3,170 | 16,163 | 7,869 | 8,294 |
| **Milenge District** | **7,594** | **43,337** | **21,338** | **21,999** |
| **Chembe Constituency** | **7,594** | **43,337** | **21,338** | **21,999** |
| Chiswishi | 408 | 2,112 | 1,071 | 1,041 |
| Mulumbi | 412 | 2,307 | 1,111 | 1,196 |
| Itemba | 880 | 4,770 | 2,302 | 2,468 |
| Lusumbwe | 433 | 2,420 | 1,186 | 1,234 |
| Milambo | 689 | 3,877 | 1,867 | 2,010 |
| Nsaka | 56 | 298 | 146 | 152 |
| Fibalala | 1,001 | 5,590 | 2,775 | 2,815 |
| Nsunga | 377 | 2,123 | 1,057 | 1,066 |
| Chipundu | 129 | 792 | 379 | 413 |
| Sokontwe | 839 | 5,126 | 2,519 | 2,607 |
| Mumbotuta | 509 | 2,930 | 1,425 | 1,505 |
| Kapalala | 215 | 1,297 | 660 | 637 |
| Mikula | 1,646 | 9,695 | 4,840 | 4,855 |
| **Mwense District** | **23,990** | **119,841** | **58,744** | **61,097** |
| **Chipili Constituency** | **6,281** | **32,565** | **16,241** | **16,324** |
| Nsenga | 1,039 | 5,385 | 2,695 | 2,690 |
| Mweshi | 700 | 3,626 | 1,803 | 1,823 |
| Mumbwe | 884 | 4,511 | 2,210 | 2,301 |
| Chibalashi | 1,075 | 5,443 | 2,719 | 2,724 |
| Nalupembe | 1,486 | 7,854 | 3,927 | 3,927 |
| Nkonge | 1,097 | 5,746 | 2,887 | 2,859 |

## Table A10: Households and Population by (De jure) Sex, Province, District, Constituency and Ward, Zambia 2010

| Province,District,Constituency and Ward | Households | Population | | |
|---|---|---|---|---|
| | | Total | Male | Female |
| **Mambilima Constituency** | **5,281** | **25,830** | **12,526** | **13,304** |
| Mpasa | 871 | 4,736 | 2,351 | 2,385 |
| Chibembe | 433 | 2,220 | 1,109 | 1,111 |
| Musonda | 757 | 3,960 | 1,928 | 2,032 |
| Lundashi | 1,352 | 6,411 | 3,047 | 3,364 |
| Mambilima | 375 | 1,753 | 869 | 884 |
| Munwa | 284 | 1,231 | 580 | 651 |
| Nsomfi | 1,209 | 5,519 | 2,642 | 2,877 |
| **Mwense Constituency** | **12,428** | **61,446** | **29,977** | **31,469** |
| Kalanga | 1,347 | 6,309 | 3,052 | 3,257 |
| Kasengu | 1,465 | 7,446 | 3,639 | 3,807 |
| Katiti | 1,219 | 6,460 | 3,135 | 3,325 |
| Chachacha | 852 | 4,503 | 2,255 | 2,248 |
| Kapela | 752 | 3,678 | 1,772 | 1,906 |
| Pabe Kabesa | 1,886 | 8,949 | 4,391 | 4,558 |
| Luche | 2,590 | 12,541 | 6,138 | 6,403 |
| Nkanga | 1,263 | 6,287 | 3,051 | 3,236 |
| Kaombe | 1,054 | 5,273 | 2,544 | 2,729 |
| **Nchelenge District** | **30,157** | **152,807** | **76,124** | **76,683** |
| **Nchelenge Constituency** | **30,157** | **152,807** | **76,124** | **76,683** |
| Kabuta | 1,655 | 8,419 | 4,292 | 4,127 |
| Mwatishi | 3,103 | 16,364 | 8,096 | 8,268 |
| Munkombwe | 317 | 1,658 | 828 | 830 |
| Kilwa | 1,786 | 8,668 | 4,387 | 4,281 |
| Kashikishi | 4,847 | 24,989 | 12,448 | 12,541 |
| Nchelenge | 233 | 1,272 | 634 | 638 |
| Chilongo | 7,489 | 37,810 | 18,738 | 19,072 |
| Chisenga | 2,096 | 10,192 | 5,170 | 5,022 |
| Kasamba | 2,610 | 12,912 | 6,391 | 6,521 |
| Mulwe | 2,569 | 12,937 | 6,439 | 6,498 |
| Shabo | 821 | 4,049 | 1,992 | 2,057 |
| Mofwe | 1,298 | 6,663 | 3,301 | 3,362 |
| Katofyo | 1,333 | 6,874 | 3,408 | 3,466 |
| **Samfya District** | **39,979** | **198,911** | **97,414** | **101,497** |
| **Bangweulu Constituency** | **18,070** | **91,569** | **44,841** | **46,728** |
| Chimana | 3,875 | 19,907 | 9,805 | 10,102 |
| Mano | 3,354 | 17,781 | 8,799 | 8,982 |
| Katanshya | 2,148 | 10,119 | 4,874 | 5,245 |
| Isamba | 1,742 | 8,723 | 4,265 | 4,458 |
| Kapata | 3,389 | 16,294 | 7,917 | 8,377 |
| Musaba | 1,757 | 9,415 | 4,626 | 4,789 |
| Kapilibila | 723 | 3,661 | 1,787 | 1,874 |
| Lumamya | 1,082 | 5,669 | 2,768 | 2,901 |
| **Chifunabuli Constituency** | **16,663** | **83,337** | **40,856** | **42,481** |
| Masonde | 1,659 | 8,921 | 4,358 | 4,563 |
| Kasansa | 1,394 | 6,884 | 3,346 | 3,538 |
| Kasaba | 2,316 | 11,397 | 5,547 | 5,850 |
| Kapamba | 1,042 | 5,217 | 2,539 | 2,678 |
| Kasongele | 1,367 | 6,585 | 3,275 | 3,310 |
| Kafumbo | 1,505 | 7,651 | 3,717 | 3,934 |
| Chinkutila | 2,125 | 10,067 | 4,932 | 5,135 |
| Chishi | 999 | 5,354 | 2,628 | 2,726 |
| Chifunabuli | 3,223 | 16,082 | 7,965 | 8,117 |
| Mbambala | 1,033 | 5,179 | 2,549 | 2,630 |

## Table A10: Households and Population by (De jure) Sex, Province, District, Constituency and Ward, Zambia 2010

| Province,District,Constituency and Ward | Households | Population | | |
|---|---|---|---|---|
| | | Total | Male | Female |
| **Luapula Constituency** | **5,246** | **24,005** | **11,717** | **12,288** |
| Nkutila | 2,115 | 9,597 | 4,665 | 4,932 |
| Lunga | 898 | 3,567 | 1,711 | 1,856 |
| Ncheta | 1,004 | 5,041 | 2,582 | 2,459 |
| Nsalushi | 1,229 | 5,800 | 2,759 | 3,041 |
| **Lusaka Province** | **444,418** | **2,191,225** | **1,082,998** | **1,108,227** |
| **Chongwe District** | **36,319** | **192,303** | **96,685** | **95,618** |
| **Chongwe Constituency** | **27,022** | **141,301** | **70,900** | **70,401** |
| Kapwayambale | 3,652 | 17,465 | 8,797 | 8,668 |
| Chinkuli | 4,397 | 22,662 | 11,440 | 11,222 |
| Ntandabale | 3,962 | 19,510 | 9,913 | 9,597 |
| Chongwe | 5,238 | 26,167 | 13,007 | 13,160 |
| Kanakantapa | 2,619 | 14,017 | 7,179 | 6,838 |
| Chalimbana | 1,792 | 10,418 | 5,141 | 5,277 |
| Nakatindi | 1,197 | 6,578 | 3,236 | 3,342 |
| Lukoshi | 1,511 | 8,738 | 4,357 | 4,381 |
| Manyika | 1,842 | 10,617 | 5,235 | 5,382 |
| Lwimba | 812 | 5,129 | 2,595 | 2,534 |
| **Rufunsa Constituency** | **9,297** | **51,002** | **25,785** | **25,217** |
| Mwachilele | 794 | 4,363 | 2,209 | 2,154 |
| Nyangwena | 1,327 | 7,631 | 3,871 | 3,760 |
| Bunda Bunda | 2,621 | 14,305 | 7,233 | 7,072 |
| Nyamanongo | 499 | 2,983 | 1,538 | 1,445 |
| Rufunsa | 2,881 | 15,724 | 7,955 | 7,769 |
| Mankanda | 813 | 4,042 | 1,986 | 2,056 |
| Shikabeta | 362 | 1,954 | 993 | 961 |
| **Kafue District** | **44,556** | **227,466** | **113,910** | **113,556** |
| **Kafue Constituency** | **23,296** | **120,415** | **60,047** | **60,368** |
| Chiyaba | 1,132 | 5,882 | 3,107 | 2,775 |
| Kambale | 991 | 5,047 | 2,563 | 2,484 |
| Malundu | 1,838 | 9,716 | 4,930 | 4,786 |
| Chisankane | 1,692 | 8,547 | 4,348 | 4,199 |
| Lukolongo | 629 | 3,140 | 1,590 | 1,550 |
| Kafue | 1,584 | 8,285 | 4,162 | 4,123 |
| Matanda | 2,124 | 10,923 | 5,323 | 5,600 |
| Shabusele | 5,993 | 30,527 | 14,982 | 15,545 |
| Kasenje | 4,283 | 21,838 | 10,651 | 11,187 |
| Mungu | 2,823 | 15,474 | 7,829 | 7,645 |
| Chikupi | 207 | 1,036 | 562 | 474 |
| **Chilanga Constituency** | **21,260** | **107,051** | **53,863** | **53,188** |
| Chilanga | 6,177 | 31,953 | 15,965 | 15,988 |
| Chilongolo | 4,034 | 19,191 | 9,725 | 9,466 |
| Namalombwe | 6,777 | 32,230 | 16,231 | 15,999 |
| Nyemba | 1,806 | 10,007 | 5,095 | 4,912 |
| Nakachenje | 394 | 2,152 | 1,081 | 1,071 |
| Chinyanja | 2,072 | 11,518 | 5,766 | 5,752 |
| **Luangwa District** | **4,672** | **24,304** | **11,979** | **12,325** |
| **Feira Constituency** | **4,672** | **24,304** | **11,979** | **12,325** |
| Dzalo | 1,005 | 5,100 | 2,520 | 2,580 |
| M'kaliva | 172 | 868 | 443 | 425 |
| Mandombe | 142 | 730 | 363 | 367 |
| Phwazi | 194 | 920 | 447 | 473 |
| Mphuka | 246 | 1,165 | 553 | 612 |
| Kabowo | 38 | 204 | 105 | 99 |
| Kapoche | 290 | 1,468 | 736 | 732 |
| Chiriwe | 51 | 301 | 154 | 147 |

## Table A10: Households and Population by (De jure) Sex, Province, District, Constituency and Ward, Zambia 2010

| Province,District,Constituency and Ward | Households | Population | | |
|---|---|---|---|---|
| | | Total | Male | Female |
| Lunya | 116 | 626 | 305 | 321 |
| Katondwe | 452 | 2,514 | 1,232 | 1,282 |
| Chikoma | 392 | 2,245 | 1,096 | 1,149 |
| Mburuma | 532 | 2,878 | 1,420 | 1,458 |
| Mwalilia | 384 | 1,820 | 888 | 932 |
| Kaunga | 534 | 2,795 | 1,384 | 1,411 |
| Mankhokwe | 124 | 670 | 333 | 337 |
| **Lusaka District** | **358,871** | **1,747,152** | **860,424** | **886,728** |
| **Chawama Constituency** | **40,055** | **187,565** | **93,490** | **94,075** |
| Nkoloma | 16,366 | 75,082 | 37,596 | 37,486 |
| Chawama | 14,799 | 70,181 | 34,898 | 35,283 |
| John Howard | 5,906 | 28,900 | 14,362 | 14,538 |
| Lilayi | 2,984 | 13,402 | 6,634 | 6,768 |
| **Kabwata Constituency** | **34,202** | **174,338** | **83,109** | **91,229** |
| Kamwala | 9,777 | 51,299 | 25,011 | 26,288 |
| Kabwata | 4,629 | 22,537 | 10,390 | 12,147 |
| Libala | 4,396 | 22,347 | 10,411 | 11,936 |
| Chilenje | 10,330 | 52,220 | 24,367 | 27,853 |
| Kamulanga | 5,070 | 25,935 | 12,930 | 13,005 |
| **Kanyama Constituency** | **76,662** | **364,655** | **181,742** | **182,913** |
| Kanyama | 35,682 | 169,253 | 84,714 | 84,539 |
| Harry Mwaanga Nkumbula | 35,068 | 167,542 | 83,212 | 84,330 |
| Munkolo | 5,912 | 27,860 | 13,816 | 14,044 |
| **Lusaka Central Constituency** | **24,484** | **117,097** | **56,016** | **61,081** |
| Silwizya | 1,521 | 6,614 | 3,100 | 3,514 |
| Independence | 3,390 | 16,134 | 7,543 | 8,591 |
| Lubwa | 7,195 | 36,445 | 16,981 | 19,464 |
| Kabulonga | 12,378 | 57,904 | 28,392 | 29,512 |
| **Mandevu Constituency** | **73,585** | **358,788** | **178,602** | **180,186** |
| Roma | 13,804 | 66,491 | 33,044 | 33,447 |
| Mulungushi | 2,883 | 13,224 | 6,332 | 6,892 |
| Ngwerere | 14,120 | 66,205 | 32,911 | 33,294 |
| Chaisa | 4,445 | 19,858 | 9,981 | 9,877 |
| Justine Kabwe | 8,398 | 40,373 | 19,863 | 20,510 |
| Raphael Chota | 18,667 | 95,041 | 47,780 | 47,261 |
| Mpulungu | 11,268 | 57,596 | 28,691 | 28,905 |
| **Matero Constituency** | **55,956** | **282,734** | **140,242** | **142,492** |
| Muchinga | 7,975 | 40,985 | 20,032 | 20,953 |
| Kapwepwe | 10,745 | 54,650 | 26,938 | 27,712 |
| Lima | 12,884 | 62,592 | 31,513 | 31,079 |
| Mwembeshi | 12,827 | 68,878 | 34,259 | 34,619 |
| Matero | 11,525 | 55,629 | 27,500 | 28,129 |
| **Munali Contituency** | **53,927** | **261,975** | **127,223** | **134,752** |
| Chainda | 8,076 | 38,393 | 18,467 | 19,926 |
| Mtendere | 22,074 | 106,128 | 52,310 | 53,818 |
| Kalingalinga | 8,356 | 39,139 | 18,945 | 20,194 |
| Chakunkula | 6,395 | 32,382 | 15,481 | 16,901 |
| Munali | 9,026 | 45,933 | 22,020 | 23,913 |
| **Muchinga Province** | **138,783** | **711,657** | **349,872** | **361,785** |
| **Chama District** | **19,420** | **103,894** | **50,856** | **53,038** |
| **Chama North Constituency** | **9,974** | **53,313** | **26,285** | **27,028** |
| Mazonde | 296 | 1,587 | 761 | 826 |
| Nkhankha | 1,039 | 5,339 | 2,642 | 2,697 |
| Luangwa | 760 | 3,868 | 1,906 | 1,962 |
| Chisunga | 729 | 3,812 | 1,874 | 1,938 |
| Ndunda | 391 | 1,919 | 946 | 973 |

## Table A10: Households and Population by (De jure) Sex, Province, District, Constituency and Ward, Zambia 2010

| Province,District,Constituency and Ward | Households | Population | | |
|---|---|---|---|---|
| | | Total | Male | Female |
| Mbazi | 566 | 2,969 | 1,478 | 1,491 |
| Manthepa | 408 | 2,183 | 1,088 | 1,095 |
| Mphalausenga | 1,159 | 6,421 | 3,177 | 3,244 |
| Kalinkhu | 394 | 2,193 | 1,045 | 1,148 |
| Kamphemba | 2,468 | 13,542 | 6,711 | 6,831 |
| Mwalala | 1,124 | 6,377 | 3,129 | 3,248 |
| Muchinga | 640 | 3,103 | 1,528 | 1,575 |
| **Chama South Constituency** | **9,446** | **50,581** | **24,571** | **26,010** |
| Chipala | 483 | 2,555 | 1,220 | 1,335 |
| Bazimu | 1,112 | 5,936 | 2,896 | 3,040 |
| Mabinga | 1,070 | 5,543 | 2,711 | 2,832 |
| Lupamazi | 437 | 2,392 | 1,177 | 1,215 |
| Lumezi | 918 | 5,284 | 2,520 | 2,764 |
| Chibungwe | 954 | 5,352 | 2,611 | 2,741 |
| Lunzi | 1,458 | 7,772 | 3,781 | 3,991 |
| Vilimukulu | 1,054 | 5,513 | 2,710 | 2,803 |
| Chilenje | 1,124 | 6,052 | 2,940 | 3,112 |
| Mapamba | 836 | 4,182 | 2,005 | 2,177 |
| **Chinsali District** | **28,668** | **146,518** | **72,526** | **73,992** |
| **Chinsali Constituency** | **17,172** | **86,723** | **42,915** | **43,808** |
| Itapa | 1,880 | 9,892 | 4,870 | 5,022 |
| Chilunda | 238 | 1,287 | 636 | 651 |
| Chilinda | 1,826 | 9,102 | 4,543 | 4,559 |
| Kaunga | 1,785 | 9,005 | 4,486 | 4,519 |
| Malalo | 882 | 4,410 | 2,186 | 2,224 |
| Chipanga | 2,037 | 10,348 | 5,099 | 5,249 |
| Chambeshi | 346 | 1,669 | 833 | 836 |
| Lubwa | 2,175 | 10,363 | 5,228 | 5,135 |
| Ichinga | 3,179 | 16,191 | 7,869 | 8,322 |
| Nkakula | 919 | 4,858 | 2,387 | 2,471 |
| Munwa Kubili | 1,905 | 9,598 | 4,778 | 4,820 |
| **Shiwang'andu Constituency** | **11,496** | **59,795** | **29,611** | **30,184** |
| Chamusenga | 1,494 | 7,823 | 3,854 | 3,969 |
| Muchinga | 1,219 | 6,354 | 3,111 | 3,243 |
| Chandaula | 789 | 4,164 | 2,016 | 2,148 |
| Mukumbi | 1,324 | 7,008 | 3,506 | 3,502 |
| Mwila Kabuswe | 1,372 | 7,070 | 3,510 | 3,560 |
| Chibinda | 212 | 1,113 | 550 | 563 |
| Mayembe | 517 | 2,935 | 1,448 | 1,487 |
| Mwiche | 664 | 3,551 | 1,802 | 1,749 |
| Ichingo | 867 | 4,488 | 2,249 | 2,239 |
| Chimpunda | 1,694 | 8,617 | 4,294 | 4,323 |
| Nkulungwe | 1,344 | 6,672 | 3,271 | 3,401 |
| **Isoka District** | **14,136** | **72,189** | **35,314** | **36,875** |
| **Isoka Constituency** | **14,136** | **72,189** | **35,314** | **36,875** |
| Kasoka | 3,458 | 17,295 | 8,394 | 8,901 |
| Kantenshya | 1,359 | 7,449 | 3,638 | 3,811 |
| Sasamwenje | 2,452 | 12,898 | 6,291 | 6,607 |
| Kapililonga | 1,729 | 8,623 | 4,250 | 4,373 |
| Itukuta | 1,130 | 5,656 | 2,792 | 2,864 |
| Milongo | 419 | 2,089 | 1,020 | 1,069 |
| Nkombwa | 1,291 | 6,503 | 3,210 | 3,293 |
| Luangwa | 684 | 3,702 | 1,824 | 1,878 |
| Mpungu | 1,614 | 7,974 | 3,895 | 4,079 |
| **Mafinga District** | **12,648** | **65,969** | **32,035** | **33,934** |
| **Mafinga Constituency** | **12,648** | **65,969** | **32,035** | **33,934** |

## Table A10: Households and Population by (De jure) Sex, Province, District, Constituency and Ward, Zambia 2010

| Province,District,Constituency and Ward | Households | Population | | |
|---|---|---|---|---|
| | | Total | Male | Female |
| Mafinga | 1,165 | 5,966 | 2,899 | 3,067 |
| Ntonga | 2,154 | 11,581 | 5,671 | 5,910 |
| Mukutu | 1,399 | 7,105 | 3,480 | 3,625 |
| Thendere | 1,878 | 10,030 | 4,794 | 5,236 |
| Bemba | 1,898 | 10,046 | 4,749 | 5,297 |
| Kakoma | 834 | 4,174 | 2,034 | 2,140 |
| Luhoka | 808 | 4,249 | 2,066 | 2,183 |
| Kalanga | 2,512 | 12,818 | 6,342 | 6,476 |
| **Mpika District** | **39,956** | **203,379** | **100,267** | **103,112** |
| **Kanchibiya Constituency** | **16,123** | **82,151** | **40,538** | **41,613** |
| Chambeshi | 1,238 | 6,237 | 3,077 | 3,160 |
| Mansha | 1,209 | 6,410 | 3,193 | 3,217 |
| Mumbubu | 1,640 | 8,424 | 4,134 | 4,290 |
| Lubaleshi | 2,378 | 12,344 | 6,056 | 6,288 |
| Lulingila | 2,118 | 10,223 | 4,967 | 5,256 |
| Munikashi | 1,607 | 7,789 | 3,886 | 3,903 |
| Chinama | 1,129 | 5,800 | 2,848 | 2,952 |
| Lukulu | 1,685 | 8,420 | 4,166 | 4,254 |
| Lulimala | 1,928 | 10,447 | 5,180 | 5,267 |
| Chibwa | 1,191 | 6,057 | 3,031 | 3,026 |
| **Mfuwe Constituency** | **5,552** | **26,811** | **13,153** | **13,658** |
| Chifungwe | 1,266 | 5,860 | 2,816 | 3,044 |
| Muchinga | 970 | 4,445 | 2,139 | 2,306 |
| Chikanda | 1,630 | 8,221 | 4,059 | 4,162 |
| Mupamadzi | 1,686 | 8,285 | 4,139 | 4,146 |
| **Mpika Central Constituency** | **18,281** | **94,417** | **46,576** | **47,841** |
| Mukungwa | 1,553 | 7,884 | 3,940 | 3,944 |
| Lwitikila | 2,104 | 10,400 | 5,091 | 5,309 |
| Musakanya | 4,380 | 22,631 | 11,137 | 11,494 |
| Lubambala | 3,023 | 16,191 | 7,857 | 8,334 |
| Chishibe Isonde | 3,153 | 15,430 | 7,591 | 7,839 |
| Nachikufu | 1,533 | 8,015 | 4,062 | 3,953 |
| Mutekwe | 912 | 5,097 | 2,574 | 2,523 |
| Chipembele | 1,623 | 8,769 | 4,324 | 4,445 |
| **Nakonde District** | **23,955** | **119,708** | **58,874** | **60,834** |
| **Nakonde Constituency** | **23,955** | **119,708** | **58,874** | **60,834** |
| Mulalo | 1,872 | 9,675 | 4,673 | 5,002 |
| Luchinde | 1,110 | 5,754 | 2,769 | 2,985 |
| Ng'umba | 1,322 | 6,728 | 3,324 | 3,404 |
| Musyani | 1,430 | 7,194 | 3,562 | 3,632 |
| Popomozi | 1,262 | 6,449 | 3,248 | 3,201 |
| Chiwaza | 1,416 | 7,797 | 3,728 | 4,069 |
| Ilonda | 965 | 5,016 | 2,450 | 2,566 |
| Isunda | 1,314 | 6,582 | 3,275 | 3,307 |
| Nakonde | 8,181 | 38,634 | 19,101 | 19,533 |
| Old Fife | 2,369 | 11,968 | 5,879 | 6,089 |
| Musele | 791 | 4,079 | 2,007 | 2,072 |
| Mpande | 1,423 | 7,273 | 3,585 | 3,688 |
| Mukulika | 500 | 2,559 | 1,273 | 1,286 |
| **Northern Province** | **220,561** | **1,105,824** | **546,851** | **558,973** |
| **Chilubi District** | **16,716** | **81,248** | **39,792** | **41,456** |
| **Chilubi Constituency** | **16,716** | **81,248** | **39,792** | **41,456** |
| Mulanda | 1,120 | 5,497 | 2,710 | 2,787 |
| Chifwenge | 1,618 | 8,130 | 4,061 | 4,069 |
| Bulilo | 1,250 | 6,489 | 3,193 | 3,296 |
| Chisupa | 756 | 3,664 | 1,804 | 1,860 |

## Table A10: Households and Population by (De jure) Sex, Province, District, Constituency and Ward, Zambia 2010

| Province,District,Constituency and Ward | Households | Population | | |
|---|---|---|---|---|
| | | Total | Male | Female |
| Ndela | 678 | 3,303 | 1,606 | 1,697 |
| Katamba | 678 | 3,227 | 1,554 | 1,673 |
| Muteka | 633 | 2,998 | 1,521 | 1,477 |
| Lwenda | 782 | 3,729 | 1,823 | 1,906 |
| Mofu | 622 | 2,994 | 1,457 | 1,537 |
| Mpanshya | 333 | 1,636 | 807 | 829 |
| Kashitu | 570 | 2,659 | 1,289 | 1,370 |
| Kambashi | 624 | 3,081 | 1,448 | 1,633 |
| Nguni | 273 | 1,208 | 600 | 608 |
| Chinkundu | 345 | 1,612 | 758 | 854 |
| Chiloba | 885 | 4,355 | 2,110 | 2,245 |
| Kanchindi | 1,455 | 7,192 | 3,502 | 3,690 |
| Kapoka | 492 | 2,304 | 1,145 | 1,159 |
| Kawena | 1,209 | 5,699 | 2,762 | 2,937 |
| Kanama | 300 | 1,458 | 695 | 763 |
| Bumba | 1,190 | 5,923 | 2,888 | 3,035 |
| Mubemba | 412 | 1,972 | 952 | 1,020 |
| Luangwa | 491 | 2,118 | 1,107 | 1,011 |
| **Kaputa District** | **23,740** | **119,514** | **59,312** | **60,202** |
| **Chimbamilonga Constituency** | **9,541** | **48,633** | **24,102** | **24,531** |
| Kapisha | 1,951 | 9,204 | 4,594 | 4,610 |
| Nsumbu | 841 | 4,404 | 2,173 | 2,231 |
| Chishela | 376 | 2,033 | 991 | 1,042 |
| Munwa | 1,502 | 7,789 | 3,921 | 3,868 |
| Kampinda | 1,306 | 6,740 | 3,361 | 3,379 |
| Kakusu | 225 | 1,196 | 581 | 615 |
| Kashikishi | 800 | 4,070 | 2,024 | 2,046 |
| Mwambeshi | 319 | 1,745 | 859 | 886 |
| Chubo | 582 | 3,077 | 1,518 | 1,559 |
| Fungwa | 852 | 4,458 | 2,171 | 2,287 |
| Mukubwe | 787 | 3,917 | 1,909 | 2,008 |
| **Kaputa Constituency** | **14,199** | **70,881** | **35,210** | **35,671** |
| Chiyilunda | 633 | 3,045 | 1,559 | 1,486 |
| Mofwe | 268 | 1,422 | 715 | 707 |
| Kalungwishi | 961 | 5,084 | 2,492 | 2,592 |
| Nkota | 1,143 | 5,769 | 2,880 | 2,889 |
| Chipili | 1,917 | 9,932 | 5,047 | 4,885 |
| Choma | 1,693 | 9,210 | 4,508 | 4,702 |
| Mowa | 2,814 | 12,825 | 6,316 | 6,509 |
| Kaleulu | 1,942 | 9,467 | 4,707 | 4,760 |
| Mwawe | 493 | 2,424 | 1,200 | 1,224 |
| Kapulwa | 1,061 | 5,527 | 2,727 | 2,800 |
| Munkonge | 1,274 | 6,176 | 3,059 | 3,117 |
| **Kasama District** | **45,862** | **231,824** | **114,208** | **117,616** |
| **Kasama Central Constituency** | **31,433** | **160,088** | **78,542** | **81,546** |
| Kasenga | 2,412 | 12,501 | 6,213 | 6,288 |
| Bululu | 3,668 | 18,968 | 9,512 | 9,456 |
| Chilunga | 2,402 | 12,147 | 5,856 | 6,291 |
| Lukulu | 744 | 3,874 | 1,930 | 1,944 |
| Julia Chikamoneka | 562 | 2,780 | 1,341 | 1,439 |
| Lukupa | 1,925 | 9,437 | 4,652 | 4,785 |
| Mulilansolo | 7,083 | 36,470 | 17,865 | 18,605 |
| Buseko | 12,637 | 63,911 | 31,173 | 32,738 |
| **Lukashya Constituency** | **14,429** | **71,736** | **35,666** | **36,070** |
| Lusenga | 2,174 | 11,029 | 5,595 | 5,434 |
| Mukanga | 1,583 | 7,881 | 3,874 | 4,007 |

## Table A10: Households and Population by (De jure) Sex, Province, District, Constituency and Ward, Zambia 2010

| Province,District,Constituency and Ward | Households | Population | | |
|---|---|---|---|---|
| | | Total | Male | Female |
| Lualuo | 1,667 | 8,569 | 4,320 | 4,249 |
| Chiba | 1,648 | 7,818 | 3,794 | 4,024 |
| Kapumaula | 1,965 | 9,812 | 4,961 | 4,851 |
| Chibundu | 1,812 | 9,095 | 4,410 | 4,685 |
| Kapongolo | 1,374 | 6,726 | 3,411 | 3,315 |
| Musowa | 1,167 | 5,765 | 2,806 | 2,959 |
| Chumba | 1,039 | 5,041 | 2,495 | 2,546 |
| **Luwingu District** | **24,307** | **122,136** | **60,294** | **61,842** |
| **Lubansenshi Costituency** | **10,422** | **52,779** | **26,095** | **26,684** |
| Ipusukilo | 834 | 4,015 | 1,923 | 2,092 |
| Katopola | 2,105 | 10,766 | 5,305 | 5,461 |
| Namukolo | 208 | 1,158 | 584 | 574 |
| Chulungoma | 1,341 | 6,720 | 3,294 | 3,426 |
| Masonde | 1,343 | 6,594 | 3,367 | 3,227 |
| Chifwile | 908 | 4,642 | 2,318 | 2,324 |
| Mushitu-Wamboo | 1,243 | 6,377 | 3,196 | 3,181 |
| Lwata | 981 | 5,170 | 2,515 | 2,655 |
| Isangano | 1,459 | 7,337 | 3,593 | 3,744 |
| **Lupososhi Constituency** | **13,885** | **69,357** | **34,199** | **35,158** |
| Ntandashi | 969 | 5,101 | 2,501 | 2,600 |
| Kaela | 1,151 | 5,836 | 2,897 | 2,939 |
| Munshishinga | 1,101 | 5,650 | 2,773 | 2,877 |
| Katilye | 783 | 4,026 | 1,930 | 2,096 |
| Kafinsa | 692 | 3,480 | 1,700 | 1,780 |
| Kampemba | 1,260 | 6,755 | 3,340 | 3,415 |
| Mulalashi | 576 | 2,652 | 1,263 | 1,389 |
| Mufili | 929 | 4,859 | 2,426 | 2,433 |
| Ilambo | 854 | 4,212 | 2,017 | 2,195 |
| Ibale | 563 | 2,643 | 1,325 | 1,318 |
| Bwalinde | 2,135 | 9,987 | 4,914 | 5,073 |
| Mwelawamangu | 1,805 | 8,828 | 4,442 | 4,386 |
| Isansa | 1,067 | 5,328 | 2,671 | 2,657 |
| **Mbala District** | **40,096** | **203,129** | **100,703** | **102,426** |
| **Mbala Constituency** | **21,761** | **110,738** | **54,625** | **56,113** |
| Intala | 4,603 | 23,064 | 11,408 | 11,656 |
| Motomoto | 579 | 3,057 | 1,521 | 1,536 |
| Kazimolwa | 4,699 | 24,077 | 11,833 | 12,244 |
| Mwambezi | 1,589 | 8,118 | 4,039 | 4,079 |
| Nsunzu | 2,973 | 15,124 | 7,444 | 7,680 |
| Kawimbe | 1,740 | 8,768 | 4,256 | 4,512 |
| Mwamba | 1,198 | 6,098 | 3,036 | 3,062 |
| Luandi | 4,380 | 22,432 | 11,088 | 11,344 |
| **Nsenga Hill Constituency** | **18,335** | **92,391** | **46,078** | **46,313** |
| Mukololo | 2,658 | 13,129 | 6,552 | 6,577 |
| Lapisha | 2,465 | 12,379 | 6,234 | 6,145 |
| Malamba | 1,563 | 7,918 | 3,990 | 3,928 |
| Chimbili | 2,470 | 12,476 | 6,198 | 6,278 |
| Chela | 3,223 | 16,338 | 8,199 | 8,139 |
| Mwiluzi | 1,430 | 6,965 | 3,488 | 3,477 |
| Chinyika | 1,854 | 9,478 | 4,689 | 4,789 |
| Ipembe | 1,338 | 6,577 | 3,225 | 3,352 |
| Chozi | 1,334 | 7,131 | 3,503 | 3,628 |
| **Mporokoso District** | **19,347** | **98,842** | **49,161** | **49,681** |
| **Lunte Constituency** | **11,269** | **57,839** | **28,849** | **28,990** |
| Kasanshi | 704 | 3,679 | 1,861 | 1,818 |
| Isenga | 974 | 5,018 | 2,491 | 2,527 |

## Table A10: Households and Population by (De jure) Sex, Province, District, Constituency and Ward, Zambia 2010

| Province,District,Constituency and Ward | Households | Population | | |
|---|---|---|---|---|
| | | Total | Male | Female |
| Nchelenge | 1,129 | 5,943 | 2,944 | 2,999 |
| Malambwa | 703 | 3,549 | 1,747 | 1,802 |
| Malaila | 842 | 4,333 | 2,204 | 2,129 |
| Bwandela | 922 | 4,871 | 2,429 | 2,442 |
| Masonde | 390 | 1,722 | 869 | 853 |
| Luangwa | 1,280 | 6,564 | 3,310 | 3,254 |
| Kalungwishi | 1,511 | 7,531 | 3,764 | 3,767 |
| Lunte | 1,332 | 7,049 | 3,508 | 3,541 |
| Kanyanta | 876 | 4,439 | 2,172 | 2,267 |
| Lubushi | 606 | 3,141 | 1,550 | 1,591 |
| **Mporokoso Constituency** | **8,078** | **41,003** | **20,312** | **20,691** |
| Lumangwe | 841 | 4,531 | 2,299 | 2,232 |
| Chikulu | 414 | 2,261 | 1,146 | 1,115 |
| Mumbuluma | 485 | 2,492 | 1,250 | 1,242 |
| Chisha Mwamba | 1,662 | 8,352 | 4,132 | 4,220 |
| Mikomba | 1,053 | 5,424 | 2,678 | 2,746 |
| Kapumo | 777 | 3,726 | 1,803 | 1,923 |
| Muchinga | 911 | 4,420 | 2,181 | 2,239 |
| Chimpolonge | 738 | 3,656 | 1,762 | 1,894 |
| Mutotoshi | 595 | 3,028 | 1,494 | 1,534 |
| Mabale | 602 | 3,113 | 1,567 | 1,546 |
| **Mpulungu District** | **19,650** | **98,073** | **48,651** | **49,422** |
| **Mpulungu District** | **19,650** | **98,073** | **48,651** | **49,422** |
| Kapembwa | 428 | 2,109 | 1,061 | 1,048 |
| Katwe | 625 | 3,213 | 1,611 | 1,602 |
| Chibulula | 5,060 | 24,254 | 12,042 | 12,212 |
| Mpulungu Central | 4,395 | 21,465 | 10,483 | 10,982 |
| Tanganyika | 576 | 2,813 | 1,398 | 1,415 |
| Chilumba | 155 | 768 | 394 | 374 |
| Isoko | 1,872 | 9,111 | 4,490 | 4,621 |
| Iyendwe | 779 | 4,060 | 1,982 | 2,078 |
| Mumila | 747 | 4,249 | 2,137 | 2,112 |
| Itimbwe | 466 | 2,375 | 1,179 | 1,196 |
| Vyamba | 2,054 | 10,431 | 5,236 | 5,195 |
| Chisha | 1,903 | 10,120 | 5,051 | 5,069 |
| Isunga | 590 | 3,105 | 1,587 | 1,518 |
| **Mungwi District** | **30,843** | **151,058** | **74,730** | **76,328** |
| **Malole Constituency** | **30,843** | **151,058** | **74,730** | **76,328** |
| Lubala | 2,765 | 13,650 | 6,747 | 6,903 |
| Mpanda | 2,615 | 12,742 | 6,304 | 6,438 |
| Kabisha | 1,870 | 8,825 | 4,346 | 4,479 |
| Fibwe | 2,176 | 10,791 | 5,425 | 5,366 |
| Chibamba | 3,575 | 17,739 | 8,779 | 8,960 |
| Iyaya | 2,356 | 11,613 | 5,759 | 5,854 |
| Kalungu | 3,054 | 14,407 | 7,086 | 7,321 |
| Fube | 2,975 | 14,296 | 6,984 | 7,312 |
| Ngulula | 2,075 | 9,870 | 4,820 | 5,050 |
| Mungwi | 2,612 | 12,978 | 6,425 | 6,553 |
| Chambeshi | 2,729 | 13,792 | 6,866 | 6,926 |
| Mabula | 1,057 | 5,342 | 2,669 | 2,673 |
| Musensenshe | 984 | 5,013 | 2,520 | 2,493 |
| **North-Western Province** | **130,803** | **727,044** | **358,141** | **368,903** |
| **Chavuma District** | **6,670** | **35,041** | **17,003** | **18,038** |
| **Chavuma Constituency** | **6,670** | **35,041** | **17,003** | **18,038** |
| Chambi Mandalo | 476 | 2,494 | 1,204 | 1,290 |
| Sewe | 471 | 2,472 | 1,203 | 1,269 |

# Table A10: Households and Population by (De jure) Sex, Province, District, Constituency and Ward, Zambia 2010

| Province,District,Constituency and Ward | Households | Population | | |
|---|---|---|---|---|
| | | Total | Male | Female |
| Lingelengenda | 318 | 1,672 | 803 | 869 |
| Chiyeke | 1,126 | 5,875 | 2,856 | 3,019 |
| Kalombo Kamisamba | 523 | 2,300 | 1,098 | 1,202 |
| Chivombo Mbalango | 242 | 1,362 | 669 | 693 |
| Chavuma Central | 1,365 | 7,263 | 3,495 | 3,768 |
| Sanjongo | 266 | 1,388 | 690 | 698 |
| Lingundu | 202 | 1,046 | 519 | 527 |
| Lukolwe Musumba | 215 | 1,152 | 571 | 581 |
| Kambuya Mukelangombe | 733 | 3,884 | 1,905 | 1,979 |
| Nyatanda Nyambingila | 452 | 2,647 | 1,281 | 1,366 |
| Nguvu | 281 | 1,486 | 709 | 777 |
| **Ikelenge District** | **5,830** | **32,919** | **16,151** | **16,768** |
| **Ikelenge Constituency** | **5,830** | **32,919** | **16,151** | **16,768** |
| Chana Chamuhinga | 553 | 3,214 | 1,576 | 1,638 |
| Jimbe | 683 | 3,772 | 1,866 | 1,906 |
| Nyakaseya | 1,481 | 8,255 | 4,017 | 4,238 |
| Ikeleng'i | 1,403 | 8,075 | 4,015 | 4,060 |
| Mwininyilamba | 921 | 5,038 | 2,464 | 2,574 |
| Mukangala | 789 | 4,565 | 2,213 | 2,352 |
| **Kabompo District** | **16,536** | **92,321** | **45,224** | **47,097** |
| **Kabompo East Constituency** | **8,440** | **48,141** | **23,625** | **24,516** |
| Dihamba | 420 | 2,491 | 1,219 | 1,272 |
| Lunsona | 856 | 5,220 | 2,608 | 2,612 |
| Kashinakaji | 71 | 505 | 251 | 254 |
| Chiteve | 237 | 1,367 | 664 | 703 |
| Manyinga | 281 | 1,474 | 741 | 733 |
| Loloma | 2,227 | 12,760 | 6,147 | 6,613 |
| Kawanda | 1,187 | 6,566 | 3,218 | 3,348 |
| Kaula | 2,803 | 15,811 | 7,818 | 7,993 |
| Chongo | 358 | 1,947 | 959 | 988 |
| **Kabompo West Constituency** | **8,096** | **44,180** | **21,599** | **22,581** |
| Kamafwafwa | 656 | 3,762 | 1,820 | 1,942 |
| Kabompo | 1,857 | 9,865 | 4,831 | 5,034 |
| Litoya | 305 | 1,569 | 773 | 796 |
| Kamisombo | 750 | 4,118 | 2,053 | 2,065 |
| Kabulamema | 753 | 4,177 | 1,961 | 2,216 |
| Mumbeji | 732 | 3,965 | 1,938 | 2,027 |
| Luli | 490 | 2,660 | 1,275 | 1,385 |
| Katuva | 438 | 2,402 | 1,200 | 1,202 |
| Maveve | 302 | 1,769 | 878 | 891 |
| Chikenge | 518 | 2,796 | 1,365 | 1,431 |
| Lunyiwe | 261 | 1,503 | 756 | 747 |
| Kayombo | 325 | 1,884 | 905 | 979 |
| Chikonkwelo | 709 | 3,710 | 1,844 | 1,866 |
| **Kasempa District** | **11,970** | **69,608** | **34,357** | **35,251** |
| **Kasempa Constituency** | **11,970** | **69,608** | **34,357** | **35,251** |
| Kamakuku | 184 | 1,046 | 519 | 527 |
| Nselauke | 590 | 3,644 | 1,748 | 1,896 |
| Ingwe | 90 | 579 | 273 | 306 |
| Kaimbwe | 585 | 3,215 | 1,609 | 1,606 |
| Mukema | 738 | 4,412 | 2,238 | 2,174 |
| Kamatete | 763 | 4,316 | 2,134 | 2,182 |
| Dengwe | 711 | 3,709 | 1,831 | 1,878 |
| Njenga | 1,043 | 6,193 | 3,085 | 3,108 |
| Kalombe | 614 | 3,686 | 1,799 | 1,887 |
| Kamusongolwa | 196 | 1,177 | 594 | 583 |

## Table A10: Households and Population by (De jure) Sex, Province, District, Constituency and Ward, Zambia 2010

| Province,District,Constituency and Ward | Households | Population | | |
|---|---|---|---|---|
| | | Total | Male | Female |
| Kikonkomene | 770 | 4,489 | 2,124 | 2,365 |
| Nkenyauna | 1,190 | 6,700 | 3,235 | 3,465 |
| Mukinge | 250 | 1,394 | 660 | 734 |
| Mutenda | 851 | 4,939 | 2,473 | 2,466 |
| Lubofu | 381 | 2,222 | 1,096 | 1,126 |
| Mpungu | 210 | 1,382 | 681 | 701 |
| Nyoka | 590 | 3,723 | 1,820 | 1,903 |
| Kelongwa | 445 | 2,774 | 1,429 | 1,345 |
| Mukunanshi | 648 | 3,653 | 1,858 | 1,795 |
| Kanongo | 284 | 1,689 | 825 | 864 |
| Kamankechi | 621 | 3,547 | 1,726 | 1,821 |
| Jifumpa | 216 | 1,119 | 600 | 519 |
| **Mufumbwe District** | **10,119** | **58,062** | **28,612** | **29,450** |
| **Mufumbwe Constituency** | **10,119** | **58,062** | **28,612** | **29,450** |
| Kashima West | 1,185 | 6,851 | 3,265 | 3,586 |
| Kashima East | 338 | 2,019 | 1,026 | 993 |
| Matushi | 1,531 | 8,791 | 4,341 | 4,450 |
| Kamabuta | 550 | 3,292 | 1,638 | 1,654 |
| Kalambu | 1,908 | 10,864 | 5,345 | 5,519 |
| Chizela | 590 | 3,082 | 1,451 | 1,631 |
| Shukwe | 376 | 2,372 | 1,237 | 1,135 |
| Kikonge | 341 | 1,833 | 932 | 901 |
| Munyambala | 256 | 1,585 | 802 | 783 |
| Kalengwa | 339 | 2,075 | 1,025 | 1,050 |
| Kabipupu | 237 | 1,426 | 695 | 731 |
| Mushima | 751 | 4,417 | 2,164 | 2,253 |
| Musonweji | 357 | 2,076 | 1,055 | 1,021 |
| Kaminzekenzeke | 354 | 2,111 | 1,032 | 1,079 |
| Lalafuta | 176 | 991 | 493 | 498 |
| Miluji | 830 | 4,277 | 2,111 | 2,166 |
| **Mwinilunga District** | **18,103** | **104,317** | **51,267** | **53,050** |
| **Mwinilunga Constituency** | **18,103** | **104,317** | **51,267** | **53,050** |
| Kanongesha | 2,475 | 13,317 | 6,525 | 6,792 |
| Kawiku | 600 | 3,162 | 1,522 | 1,640 |
| Mulumbi | 2,702 | 15,704 | 7,555 | 8,149 |
| Mundwinji | 1,448 | 8,241 | 4,026 | 4,215 |
| Kapundu | 866 | 5,057 | 2,467 | 2,590 |
| Kanyama | 757 | 4,643 | 2,324 | 2,319 |
| Kakoma | 1,252 | 7,127 | 3,532 | 3,595 |
| Kasampula | 192 | 1,085 | 521 | 564 |
| Lumwana | 850 | 5,021 | 2,517 | 2,504 |
| Sailunga | 502 | 3,035 | 1,503 | 1,532 |
| Chisasa | 631 | 3,932 | 1,966 | 1,966 |
| Ntambu | 1,479 | 9,319 | 4,559 | 4,760 |
| Samuteba | 1,176 | 6,875 | 3,384 | 3,491 |
| Mudyanyama | 555 | 3,120 | 1,550 | 1,570 |
| Chibwika | 1,761 | 9,944 | 4,939 | 5,005 |
| Kamapanda | 857 | 4,735 | 2,377 | 2,358 |
| **Solwezi District** | **46,574** | **254,470** | **126,249** | **128,221** |
| **Solwezi Central Constituency** | **24,466** | **132,532** | **65,682** | **66,850** |
| Kapijimpanga | 3,315 | 18,307 | 9,089 | 9,218 |
| Sandangombe | 3,342 | 18,361 | 8,979 | 9,382 |
| Kamalamba | 3,578 | 19,068 | 9,458 | 9,610 |
| Tumvwanganai | 7,433 | 40,388 | 20,094 | 20,294 |
| Kimasala | 6,798 | 36,408 | 18,062 | 18,346 |

## Table A10: Households and Population by (De jure) Sex, Province, District, Constituency and Ward, Zambia 2010

| Province,District,Constituency and Ward | Households | Population | | |
|---|---|---|---|---|
| | | Total | Male | Female |
| **Solwezi East Constituency** | **6,643** | **36,433** | **18,019** | **18,414** |
| Musaka | 1,662 | 8,811 | 4,379 | 4,432 |
| Chikola | 686 | 3,965 | 2,035 | 1,930 |
| Kangwena | 765 | 4,097 | 1,929 | 2,168 |
| Kalilele | 549 | 3,045 | 1,472 | 1,573 |
| Mulonga | 1,810 | 9,888 | 4,904 | 4,984 |
| Mapunga | 702 | 3,857 | 1,958 | 1,899 |
| Mujimanzovu | 469 | 2,770 | 1,342 | 1,428 |
| **Solwezi West Constituency** | **15,465** | **85,505** | **42,548** | **42,957** |
| Mumena | 2,043 | 11,810 | 5,893 | 5,917 |
| Mwajimambwe | 2,404 | 12,654 | 6,349 | 6,305 |
| Kibanza | 994 | 5,388 | 2,722 | 2,666 |
| Mukumbi | 1,729 | 8,997 | 4,535 | 4,462 |
| Matebo | 438 | 2,391 | 1,232 | 1,159 |
| Shilenda | 3,285 | 16,493 | 8,181 | 8,312 |
| Lumwana | 1,351 | 7,501 | 3,657 | 3,844 |
| Mumbezhi | 301 | 1,626 | 817 | 809 |
| Musele | 1,905 | 11,960 | 5,888 | 6,072 |
| Chovwe | 1,015 | 6,685 | 3,274 | 3,411 |
| **Zambezi District** | **15,001** | **80,306** | **39,278** | **41,028** |
| **Zambezi East Constituency** | **10,809** | **58,334** | **28,478** | **29,856** |
| Lunkunyi | 812 | 4,409 | 2,153 | 2,256 |
| Nyakuleng'a | 692 | 3,920 | 1,936 | 1,984 |
| Dipalata | 1,001 | 5,786 | 2,805 | 2,981 |
| Mukanda Nkunda | 1,399 | 7,567 | 3,660 | 3,907 |
| Chileng'a Chizenzi | 964 | 5,096 | 2,497 | 2,599 |
| Lwitadi Lwatembo | 576 | 3,156 | 1,598 | 1,558 |
| Mpidi Kakong'a | 1,418 | 7,598 | 3,772 | 3,826 |
| Chitokoloki | 805 | 4,199 | 2,051 | 2,148 |
| Chivweji Kasesi | 1,258 | 6,304 | 3,034 | 3,270 |
| Zambezi | 1,884 | 10,299 | 4,972 | 5,327 |
| **Zambezi West Constituency** | **4,192** | **21,972** | **10,800** | **11,172** |
| Mapachi Chinyingi | 740 | 3,750 | 1,865 | 1,885 |
| Likungu | 898 | 4,765 | 2,339 | 2,426 |
| Liyovu | 1,000 | 5,036 | 2,485 | 2,551 |
| Muyembe | 376 | 1,984 | 944 | 1,040 |
| Mwange Nyawanda | 624 | 3,698 | 1,830 | 1,868 |
| Matondo Nyachikayi | 554 | 2,739 | 1,337 | 1,402 |
| **Southern Province** | **292,179** | **1,589,926** | **779,659** | **810,267** |
| **Choma District** | **44,483** | **247,860** | **120,689** | **127,171** |
| **Choma Central Constituency** | **23,398** | **122,736** | **60,181** | **62,555** |
| Batoka | 1,183 | 6,974 | 3,414 | 3,560 |
| Sikalongo | 1,446 | 8,111 | 3,940 | 4,171 |
| Simamvwa | 2,127 | 11,505 | 5,789 | 5,716 |
| Stateland | 1,289 | 6,544 | 3,257 | 3,287 |
| Nakeempa | 931 | 5,595 | 2,741 | 2,854 |
| Moomba | 703 | 3,300 | 1,642 | 1,658 |
| Kalundana | 2,703 | 13,142 | 6,374 | 6,768 |
| Simacheche | 1,888 | 8,431 | 4,206 | 4,225 |
| Sikalundu | 1,844 | 9,431 | 4,541 | 4,890 |
| Mubula | 4,269 | 20,838 | 10,133 | 10,705 |
| Singani | 1,528 | 8,927 | 4,470 | 4,457 |
| Siasikabole | 1,413 | 8,421 | 4,105 | 4,316 |
| Namuswa | 2,074 | 11,517 | 5,569 | 5,948 |

## Table A10: Households and Population by (De jure) Sex, Province, District, Constituency and Ward, Zambia 2010

| Province,District,Constituency and Ward | Households | Population | | |
|---|---|---|---|---|
| | | Total | Male | Female |
| **Mbabala Constituency** | **9,671** | **57,937** | **28,159** | **29,778** |
| Simaubi | 1,973 | 11,991 | 5,871 | 6,120 |
| Mapanza | 960 | 5,998 | 2,910 | 3,088 |
| Mang'unza | 982 | 6,132 | 2,957 | 3,175 |
| Chilalantambo | 1,578 | 9,521 | 4,639 | 4,882 |
| Macha | 1,283 | 7,501 | 3,631 | 3,870 |
| Kabimba | 344 | 2,035 | 984 | 1,051 |
| Mbabala | 2,551 | 14,759 | 7,167 | 7,592 |
| **Pemba Constituency** | **11,414** | **67,187** | **32,349** | **34,838** |
| Kasiya | 2,110 | 12,861 | 6,332 | 6,529 |
| Pemba | 492 | 2,328 | 1,101 | 1,227 |
| Hamaundu | 3,165 | 18,408 | 8,875 | 9,533 |
| Maambo | 2,117 | 12,716 | 6,142 | 6,574 |
| Kauba | 1,225 | 7,232 | 3,443 | 3,789 |
| Habunkululu | 771 | 4,388 | 2,063 | 2,325 |
| Nachibanga | 1,534 | 9,254 | 4,393 | 4,861 |
| **Gwembe District** | **9,846** | **53,117** | **25,908** | **27,209** |
| **Gwembe Constituency** | **9,846** | **53,117** | **25,908** | **27,209** |
| Chisanga | 485 | 2,862 | 1,384 | 1,478 |
| Sinafala | 373 | 2,060 | 1,015 | 1,045 |
| Jumbo | 409 | 2,425 | 1,183 | 1,242 |
| Kkoma | 1,049 | 5,880 | 2,892 | 2,988 |
| Chibuwe | 1,079 | 5,420 | 2,642 | 2,778 |
| Siampande | 263 | 1,450 | 751 | 699 |
| Kota Kota | 200 | 1,113 | 557 | 556 |
| Luumbo | 722 | 3,668 | 1,803 | 1,865 |
| Kkole | 511 | 2,743 | 1,317 | 1,426 |
| Bbondo | 1,625 | 8,682 | 4,193 | 4,489 |
| Chaamwe | 536 | 2,894 | 1,415 | 1,479 |
| Fumbo | 1,700 | 9,029 | 4,407 | 4,622 |
| Jongola | 111 | 660 | 312 | 348 |
| Lukonde | 783 | 4,231 | 2,037 | 2,194 |
| **Itezhi Tezhi District** | **12,237** | **68,599** | **34,017** | **34,582** |
| **Itezhi Tezhi Constituency** | **12,237** | **68,599** | **34,017** | **34,582** |
| Itezhi Tezhi | 1,559 | 8,290 | 4,030 | 4,260 |
| Itumbi | 1,095 | 5,803 | 2,887 | 2,916 |
| Kaanzwa | 965 | 6,290 | 3,087 | 3,203 |
| Banamwaze | 460 | 2,830 | 1,416 | 1,414 |
| Makunku | 632 | 3,680 | 1,831 | 1,849 |
| Nyambo | 418 | 2,518 | 1,288 | 1,230 |
| Kabulungwe | 371 | 1,702 | 886 | 816 |
| Lubanda | 1,226 | 6,691 | 3,394 | 3,297 |
| Masemu | 1,928 | 9,864 | 4,886 | 4,978 |
| Luubwe | 614 | 3,348 | 1,704 | 1,644 |
| Basanga | 1,340 | 7,309 | 3,545 | 3,764 |
| Luchena | 397 | 2,189 | 1,092 | 1,097 |
| Mbila | 1,232 | 8,085 | 3,971 | 4,114 |
| **Kalomo District** | **44,728** | **258,570** | **125,767** | **132,803** |
| **Dundumwezi Constituency** | **12,684** | **80,415** | **39,133** | **41,282** |
| Chikanta | 3,337 | 20,836 | 10,201 | 10,635 |
| Chamuka | 1,601 | 10,065 | 4,825 | 5,240 |
| Kasukwe | 2,336 | 15,141 | 7,333 | 7,808 |
| Omba | 1,677 | 10,676 | 5,162 | 5,514 |
| Bbilili | 1,614 | 9,990 | 4,911 | 5,079 |
| Naluja | 2,119 | 13,707 | 6,701 | 7,006 |

## Table A10: Households and Population by (De jure) Sex, Province, District, Constituency and Ward, Zambia 2010

| Province,District,Constituency and Ward | Households | Population | | |
|---|---|---|---|---|
| | | Total | Male | Female |
| **Kalomo Central Constituency** | **19,154** | **108,278** | **52,796** | **55,482** |
| Siachitema | 4,451 | 26,725 | 13,033 | 13,692 |
| Kalonda | 2,219 | 13,562 | 6,531 | 7,031 |
| Choonga | 4,229 | 21,559 | 10,454 | 11,105 |
| Mayoba | 2,059 | 11,337 | 5,626 | 5,711 |
| Namwianga | 1,826 | 9,785 | 4,850 | 4,935 |
| Simayakwe | 824 | 5,034 | 2,454 | 2,580 |
| Chawila | 1,391 | 8,063 | 3,916 | 4,147 |
| Sipatunyana | 698 | 3,814 | 1,899 | 1,915 |
| Nachikungu | 1,457 | 8,399 | 4,033 | 4,366 |
| **Mapatizya Constituency** | **12,890** | **69,877** | **33,838** | **36,039** |
| Chidi | 1,897 | 10,347 | 4,915 | 5,432 |
| Mulamfu | 1,227 | 6,549 | 3,113 | 3,436 |
| Simwatachela | 930 | 5,057 | 2,486 | 2,571 |
| Luyaba | 2,677 | 15,165 | 7,356 | 7,809 |
| Zimba | 2,466 | 13,185 | 6,400 | 6,785 |
| Siamafumba | 1,936 | 10,413 | 5,113 | 5,300 |
| Mbwiko | 1,757 | 9,161 | 4,455 | 4,706 |
| **Kazungula District** | **20,024** | **104,731** | **51,994** | **52,737** |
| **Katombola Constituency** | **20,024** | **104,731** | **51,994** | **52,737** |
| Moomba | 494 | 2,425 | 1,228 | 1,197 |
| Chooma | 1,178 | 7,264 | 3,584 | 3,680 |
| Nguba | 1,800 | 11,393 | 5,551 | 5,842 |
| Kauwe | 1,123 | 6,539 | 3,324 | 3,215 |
| Nyawa | 1,962 | 11,075 | 5,407 | 5,668 |
| Ngwezi | 1,975 | 10,182 | 5,133 | 5,049 |
| Sikaunzwe | 1,536 | 7,542 | 3,817 | 3,725 |
| Mandia | 2,074 | 9,246 | 4,558 | 4,688 |
| Sekute | 656 | 3,228 | 1,652 | 1,576 |
| Kanchele | 2,154 | 10,860 | 5,319 | 5,541 |
| Simango | 861 | 4,745 | 2,377 | 2,368 |
| Musokotwane | 990 | 4,960 | 2,458 | 2,502 |
| Katapazi | 1,271 | 6,410 | 3,167 | 3,243 |
| Mukuni | 1,950 | 8,862 | 4,419 | 4,443 |
| **Livingstone District** | **30,461** | **139,509** | **68,763** | **70,746** |
| **Livingstone Constituency** | **30,461** | **139,509** | **68,763** | **70,746** |
| Freedom | 2,360 | 11,974 | 5,901 | 6,073 |
| Musi-oa-tunya | 1,882 | 8,213 | 3,930 | 4,283 |
| Dr. Mubitana | 1,391 | 6,543 | 3,259 | 3,284 |
| Namatama | 2,717 | 11,606 | 5,875 | 5,731 |
| Kasiya | 1,839 | 9,165 | 4,598 | 4,567 |
| Libuyu | 1,481 | 7,817 | 3,967 | 3,850 |
| Mwalibonena | 2,315 | 9,886 | 4,840 | 5,046 |
| Mulungushi | 2,048 | 8,263 | 4,007 | 4,256 |
| Maramba | 1,797 | 9,590 | 4,553 | 5,037 |
| Akapelwa | 742 | 3,058 | 1,412 | 1,646 |
| Lizuma | 553 | 2,761 | 1,313 | 1,448 |
| Simonga | 2,984 | 12,926 | 6,483 | 6,443 |
| Dambwa Central | 1,384 | 6,329 | 3,043 | 3,286 |
| Zambezi | 3,811 | 16,098 | 7,902 | 8,196 |
| Kariba | 939 | 4,521 | 2,231 | 2,290 |
| Nansanzu | 1,418 | 7,194 | 3,611 | 3,583 |
| Shungu | 800 | 3,565 | 1,838 | 1,727 |

## Table A10: Households and Population by (De jure) Sex, Province, District, Constituency and Ward, Zambia 2010

| Province,District,Constituency and Ward | Households | Population | | |
|---|---|---|---|---|
| | | Total | Male | Female |
| **Mazabuka District** | **43,411** | **230,972** | **114,783** | **116,189** |
| **Chikankanta Constituency** | **11,070** | **59,909** | **29,696** | **30,213** |
| Kasengo | 1,223 | 6,787 | 3,391 | 3,396 |
| Namalundu | 1,506 | 7,989 | 3,999 | 3,990 |
| Musaya | 224 | 1,133 | 559 | 574 |
| Nansenga | 532 | 2,701 | 1,363 | 1,338 |
| Chitete | 1,963 | 10,531 | 5,188 | 5,343 |
| Malala | 1,171 | 6,454 | 3,226 | 3,228 |
| Mabwe Atuba | 2,369 | 12,974 | 6,358 | 6,616 |
| Upper Kaleya | 2,082 | 11,340 | 5,612 | 5,728 |
| **Magoye Constituency** | **12,298** | **71,231** | **35,223** | **36,008** |
| Konkola | 1,126 | 6,462 | 3,195 | 3,267 |
| Chivuna | 3,355 | 19,508 | 9,453 | 10,055 |
| Musuma | 874 | 5,534 | 2,714 | 2,820 |
| Munjile | 435 | 2,508 | 1,265 | 1,243 |
| Ngwezi | 2,785 | 16,557 | 8,160 | 8,397 |
| Kalama | 581 | 3,351 | 1,684 | 1,667 |
| Munenga | 507 | 2,713 | 1,374 | 1,339 |
| Mwanachingwala | 2,125 | 11,742 | 5,898 | 5,844 |
| Itebe | 510 | 2,856 | 1,480 | 1,376 |
| **Mazabuka Central Constituency** | **20,043** | **99,832** | **49,864** | **49,968** |
| Mazabuka Central | 12,067 | 58,944 | 29,407 | 29,537 |
| Nakambala | 1,553 | 8,565 | 4,266 | 4,299 |
| Chizobo | 1,213 | 6,040 | 3,035 | 3,005 |
| Lubombo | 4,300 | 21,496 | 10,729 | 10,767 |
| Nega Nega | 910 | 4,787 | 2,427 | 2,360 |
| **Monze District** | **32,849** | **191,872** | **93,958** | **97,914** |
| **Bweengwa Constituency** | **9,525** | **60,417** | **29,507** | **30,910** |
| Malundu | 1,351 | 8,291 | 4,111 | 4,180 |
| Kaila | 1,156 | 7,405 | 3,557 | 3,848 |
| Keemba | 2,063 | 12,513 | 6,141 | 6,372 |
| Choongo West | 680 | 3,668 | 1,735 | 1,933 |
| Bweengwa | 1,064 | 7,281 | 3,538 | 3,743 |
| Hamangaba | 1,108 | 8,461 | 4,102 | 4,359 |
| Choongo East | 2,103 | 12,798 | 6,323 | 6,475 |
| **Monze Central Constituency** | **18,162** | **101,402** | **49,687** | **51,715** |
| Chipembele | 1,291 | 7,141 | 3,525 | 3,616 |
| Ufwenuka | 1,689 | 9,601 | 4,690 | 4,911 |
| Manungu | 7,605 | 38,590 | 18,824 | 19,766 |
| Chisekesi | 1,358 | 7,206 | 3,527 | 3,679 |
| Mayaba | 798 | 4,685 | 2,238 | 2,447 |
| Hufwa/Hamapande | 607 | 3,589 | 1,751 | 1,838 |
| Hamamvwa | 718 | 4,290 | 2,122 | 2,168 |
| Katimba | 1,047 | 7,051 | 3,417 | 3,634 |
| Hatontola | 1,883 | 11,960 | 5,933 | 6,027 |
| Bbombo | 914 | 5,787 | 2,867 | 2,920 |
| Monze Urban | 252 | 1,502 | 793 | 709 |
| **Moomba Constituency** | **5,162** | **30,053** | **14,764** | **15,289** |
| Mwanza West | 2,167 | 13,041 | 6,391 | 6,650 |
| Chona | 1,749 | 9,716 | 4,784 | 4,932 |
| Mwanza East | 1,075 | 6,286 | 3,088 | 3,198 |
| Moomba | 171 | 1,010 | 501 | 509 |

## Table A10: Households and Population by (De jure) Sex, Province, District, Constituency and Ward, Zambia 2010

| Province,District,Constituency and Ward | Households | Population | | |
|---|---|---|---|---|
| | | Total | Male | Female |
| **Namwala District** | **16,662** | **102,866** | **50,127** | **52,739** |
| **Namwala Constituency** | **16,662** | **102,866** | **50,127** | **52,739** |
| Namwala Central | 2,147 | 10,300 | 5,014 | 5,286 |
| Ngabo | 455 | 2,729 | 1,406 | 1,323 |
| Baambwe | 771 | 4,069 | 2,076 | 1,993 |
| Maala | 1,036 | 5,776 | 2,781 | 2,995 |
| Kantengwa | 780 | 4,506 | 2,281 | 2,225 |
| Kabulamwanda | 1,162 | 7,221 | 3,565 | 3,656 |
| Chitongo | 773 | 4,939 | 2,361 | 2,578 |
| Mandondo | 660 | 4,474 | 2,188 | 2,286 |
| Nakamboma | 2,071 | 13,614 | 6,565 | 7,049 |
| Mbeza | 824 | 5,122 | 2,506 | 2,616 |
| Ndema | 1,390 | 9,308 | 4,443 | 4,865 |
| Namakube | 1,588 | 10,943 | 5,323 | 5,620 |
| Itapa | 994 | 7,117 | 3,442 | 3,675 |
| Moobola | 2,011 | 12,748 | 6,176 | 6,572 |
| **Siavonga District** | **17,757** | **90,213** | **44,444** | **45,769** |
| **Siavonga Constituency** | **17,757** | **90,213** | **44,444** | **45,769** |
| Ibwemunyama | 570 | 3,376 | 1,656 | 1,720 |
| Musaya | 1,050 | 5,342 | 2,659 | 2,683 |
| Chirundu | 3,025 | 14,864 | 7,132 | 7,732 |
| Ng'ombe Ilede | 2,326 | 11,782 | 5,741 | 6,041 |
| Sikoongo | 684 | 3,905 | 1,898 | 2,007 |
| Lusitu | 1,508 | 8,075 | 4,010 | 4,065 |
| Nanyanga | 505 | 2,588 | 1,306 | 1,282 |
| Kariba | 3,496 | 16,415 | 8,196 | 8,219 |
| Simamba | 1,310 | 6,304 | 3,125 | 3,179 |
| Mulimya | 1,021 | 5,439 | 2,732 | 2,707 |
| Manchamvwa | 930 | 4,723 | 2,349 | 2,374 |
| Sinadambwe | 775 | 4,325 | 2,150 | 2,175 |
| Lusangazi | 557 | 3,075 | 1,490 | 1,585 |
| **Sinazongwe District** | **19,721** | **101,617** | **49,209** | **52,408** |
| **Sinazongwe Constituency** | **19,721** | **101,617** | **49,209** | **52,408** |
| Mabinga | 156 | 778 | 385 | 393 |
| Namazambwe | 1,030 | 5,120 | 2,507 | 2,613 |
| Mweenda | 1,026 | 5,398 | 2,559 | 2,839 |
| Muuka | 910 | 4,819 | 2,374 | 2,445 |
| Tekelo | 313 | 1,923 | 917 | 1,006 |
| Mweemba | 2,245 | 11,331 | 5,551 | 5,780 |
| Muchekwa | 1,280 | 6,791 | 3,274 | 3,517 |
| Maamba | 1,898 | 10,249 | 4,967 | 5,282 |
| Mweezya | 3,421 | 17,200 | 8,302 | 8,898 |
| Nkamdabwe | 1,334 | 6,490 | 3,085 | 3,405 |
| Sinazongwe | 2,135 | 10,698 | 5,224 | 5,474 |
| Nang'ombe | 1,185 | 6,384 | 3,035 | 3,349 |
| Sinenge | 1,560 | 8,274 | 3,998 | 4,276 |
| Malima | 1,228 | 6,162 | 3,031 | 3,131 |

## Table A10: Households and Population by (De jure) Sex, Province, District, Constituency and Ward, Zambia 2010

| Province,District,Constituency and Ward | Households | Population | | |
|---|---|---|---|---|
| | | Total | Male | Female |
| **Western Province** | **180,179** | **902,974** | **433,505** | **469,469** |
| **Kalabo District** | **26,480** | **128,904** | **60,114** | **68,790** |
| **Kalabo Central Constituency** | **11,639** | **56,963** | **26,742** | **30,221** |
| Mapungu | 758 | 3,741 | 1,753 | 1,988 |
| Luanginga | 1,716 | 8,745 | 4,271 | 4,474 |
| Liumba | 700 | 3,661 | 1,732 | 1,929 |
| Yuka | 1,560 | 7,097 | 3,273 | 3,824 |
| Buleya | 892 | 4,183 | 1,920 | 2,263 |
| Lutwi | 1,211 | 5,719 | 2,654 | 3,065 |
| Ndoka | 1,673 | 7,774 | 3,581 | 4,193 |
| Namulilo | 1,547 | 7,933 | 3,724 | 4,209 |
| Nguma | 852 | 4,284 | 2,045 | 2,239 |
| Kandambo | 730 | 3,826 | 1,789 | 2,037 |
| **Liuwa Constituency** | **5,303** | **26,479** | **12,620** | **13,859** |
| Siluwe | 463 | 2,161 | 1,012 | 1,149 |
| Likulundundu | 438 | 2,254 | 1,066 | 1,188 |
| Luola | 365 | 1,773 | 821 | 952 |
| Salunda | 646 | 3,002 | 1,392 | 1,610 |
| Sishekanu | 1,146 | 5,570 | 2,588 | 2,982 |
| Kuuli | 750 | 3,878 | 1,897 | 1,981 |
| Libonda | 1,495 | 7,841 | 3,844 | 3,997 |
| **Sikongo Constituency** | **9,538** | **45,462** | **20,752** | **24,710** |
| Lueti | 1,811 | 8,817 | 4,083 | 4,734 |
| Lulan'gunyi | 519 | 2,386 | 1,103 | 1,283 |
| Tuuwa | 1,031 | 4,659 | 2,088 | 2,571 |
| Maala | 1,190 | 5,706 | 2,643 | 3,063 |
| Liumena | 1,377 | 6,596 | 2,999 | 3,597 |
| Licha | 1,704 | 8,262 | 3,703 | 4,559 |
| Lwambi | 332 | 1,711 | 790 | 921 |
| Nengu | 491 | 2,367 | 1,062 | 1,305 |
| Mutala | 333 | 1,516 | 715 | 801 |
| Mwenyi | 750 | 3,442 | 1,566 | 1,876 |
| **Kaoma District** | **36,068** | **189,290** | **91,872** | **97,418** |
| **Kaoma Central Constituency** | **16,276** | **87,027** | **43,163** | **43,864** |
| Lalafuta | 1,307 | 7,462 | 3,741 | 3,721 |
| Shitwa | 1,547 | 8,409 | 4,170 | 4,239 |
| Namilangi | 3,829 | 21,066 | 10,310 | 10,756 |
| Nkeyema | 2,131 | 12,031 | 6,022 | 6,009 |
| Litoya | 1,618 | 9,073 | 4,699 | 4,374 |
| Mulamatila | 4,358 | 21,434 | 10,429 | 11,005 |
| Longe | 1,486 | 7,552 | 3,792 | 3,760 |
| **Luampa Constituency** | **8,422** | **43,840** | **20,788** | **23,052** |
| Naliele | 1,365 | 7,123 | 3,403 | 3,720 |
| Namando | 228 | 1,205 | 587 | 618 |
| Nyambi | 1,156 | 6,081 | 2,997 | 3,084 |
| Mulwa | 806 | 4,187 | 2,021 | 2,166 |
| Lui | 1,476 | 7,793 | 3,603 | 4,190 |
| Nkenga | 1,740 | 8,844 | 4,120 | 4,724 |
| Mbanyutu | 370 | 2,069 | 945 | 1,124 |
| Luampa | 1,281 | 6,538 | 3,112 | 3,426 |
| **Mangango Constituency** | **11,370** | **58,423** | **27,921** | **30,502** |
| Namafulo | 3,267 | 16,161 | 7,560 | 8,601 |
| Luambuwa | 1,435 | 7,665 | 3,619 | 4,046 |
| Mushwala | 3,301 | 16,903 | 8,156 | 8,747 |
| Kapili | 442 | 2,261 | 1,112 | 1,149 |
| Mangango | 1,006 | 5,279 | 2,542 | 2,737 |

## Table A10: Households and Population by (De jure) Sex, Province, District, Constituency and Ward, Zambia 2010

| Province,District,Constituency and Ward | Households | Population | | |
|---|---|---|---|---|
| | | Total | Male | Female |
| Kanabilumbu | 617 | 3,144 | 1,526 | 1,618 |
| Shikombwe | 1,302 | 7,010 | 3,406 | 3,604 |
| **Lukulu District** | **16,676** | **86,002** | **41,390** | **44,612** |
| **Lukulu East Constituency** | **11,230** | **58,534** | **28,290** | **30,244** |
| Simakumba | 880 | 4,752 | 2,303 | 2,449 |
| Kamilende | 475 | 2,727 | 1,360 | 1,367 |
| Dongwe | 432 | 2,422 | 1,207 | 1,215 |
| Kashamba | 475 | 2,602 | 1,270 | 1,332 |
| Mwito | 729 | 3,977 | 1,867 | 2,110 |
| Kang'oti | 1,251 | 6,583 | 3,145 | 3,438 |
| Lukau | 700 | 3,578 | 1,718 | 1,860 |
| Likapai | 300 | 1,637 | 812 | 825 |
| Mbanga | 964 | 5,021 | 2,430 | 2,591 |
| Kawaya | 845 | 4,096 | 1,986 | 2,110 |
| Mwandi | 2,125 | 10,575 | 5,071 | 5,504 |
| Namayula | 777 | 3,861 | 1,875 | 1,986 |
| Luanchuma | 1,277 | 6,703 | 3,246 | 3,457 |
| **Lukulu West Constituency** | **5,446** | **27,468** | **13,100** | **14,368** |
| Muyondoti | 620 | 2,979 | 1,448 | 1,531 |
| Nyaala | 628 | 3,299 | 1,602 | 1,697 |
| Mataba | 687 | 3,604 | 1,696 | 1,908 |
| Lupui | 549 | 2,753 | 1,288 | 1,465 |
| Kakwacha | 345 | 1,649 | 763 | 886 |
| Lutembwe | 187 | 988 | 490 | 498 |
| Mitete | 667 | 3,281 | 1,529 | 1,752 |
| Kashizhi | 1,303 | 6,550 | 3,156 | 3,394 |
| Chin'onwe | 460 | 2,365 | 1,128 | 1,237 |
| **Mongu District** | **36,605** | **179,585** | **85,964** | **93,621** |
| **Luena Constituency** | **10,180** | **50,741** | **24,219** | **26,522** |
| Limulunga | 2,921 | 14,199 | 6,682 | 7,517 |
| Mabili | 789 | 4,006 | 1,995 | 2,011 |
| Ikwichi | 734 | 3,433 | 1,615 | 1,818 |
| Namboma | 1,097 | 5,176 | 2,501 | 2,675 |
| Nangula | 2,442 | 12,236 | 5,895 | 6,341 |
| Ushaa | 990 | 5,248 | 2,504 | 2,744 |
| Simaa | 920 | 4,908 | 2,310 | 2,598 |
| Ndanda | 287 | 1,535 | 717 | 818 |
| **Mongu Central Constituency** | **18,410** | **88,827** | **42,616** | **46,211** |
| Namushakende | 1,120 | 5,428 | 2,512 | 2,916 |
| Yeta | 1,455 | 6,612 | 3,234 | 3,378 |
| Kama | 608 | 2,911 | 1,452 | 1,459 |
| Lumbo | 954 | 4,558 | 2,145 | 2,413 |
| Katongo | 2,164 | 9,777 | 4,558 | 5,219 |
| Kanyonyo | 2,425 | 11,440 | 5,521 | 5,919 |
| Kambule | 1,921 | 9,552 | 4,543 | 5,009 |
| Lewanika | 659 | 3,004 | 1,478 | 1,526 |
| Mulambwa | 1,908 | 9,813 | 4,684 | 5,129 |
| Imwiko | 3,300 | 16,967 | 8,113 | 8,854 |
| Lealui | 1,038 | 4,810 | 2,422 | 2,388 |
| Mabumbu | 496 | 2,207 | 1,083 | 1,124 |
| Kaande | 362 | 1,748 | 871 | 877 |
| **Nalikwanda Constituency** | **8,015** | **40,017** | **19,129** | **20,888** |
| Lui | 2,017 | 10,291 | 4,832 | 5,459 |
| Imalyo | 1,234 | 6,247 | 2,985 | 3,262 |
| Mutondo | 661 | 3,402 | 1,702 | 1,700 |
| Namengo | 734 | 3,285 | 1,576 | 1,709 |

## Table A10: Households and Population by (De jure) Sex, Province, District, Constituency and Ward, Zambia 2010

| Province,District,Constituency and Ward | Households | Population | | |
|---|---|---|---|---|
| | | Total | Male | Female |
| Nakanyaa | 1,378 | 6,882 | 3,321 | 3,561 |
| Mbekise | 837 | 4,133 | 1,991 | 2,142 |
| Nakato | 1,154 | 5,777 | 2,722 | 3,055 |
| **Senanga District** | **25,162** | **126,506** | **60,428** | **66,078** |
| **Nalolo Constituency** | **10,841** | **55,569** | **26,558** | **29,011** |
| Lyamakumba | 2,261 | 11,977 | 5,719 | 6,258 |
| Silowana | 1,326 | 6,890 | 3,300 | 3,590 |
| Shekela | 1,417 | 7,281 | 3,581 | 3,700 |
| Makoka | 1,254 | 6,500 | 3,036 | 3,464 |
| Kambai | 1,292 | 6,650 | 3,171 | 3,479 |
| Kataba | 906 | 4,586 | 2,220 | 2,366 |
| Muoyo | 1,032 | 4,911 | 2,317 | 2,594 |
| Nanjucha | 1,353 | 6,774 | 3,214 | 3,560 |
| **Senanga Central Constituency** | **14,321** | **70,937** | **33,870** | **37,067** |
| Mwanambuyu | 2,255 | 11,581 | 5,445 | 6,136 |
| Imatongo | 1,557 | 7,498 | 3,585 | 3,913 |
| Imatanda | 3,575 | 17,325 | 8,302 | 9,023 |
| Wanyau | 990 | 4,811 | 2,267 | 2,544 |
| Lipuwe | 1,824 | 9,127 | 4,286 | 4,841 |
| Naluywa | 1,214 | 6,161 | 3,073 | 3,088 |
| Lumbe | 821 | 4,141 | 1,992 | 2,149 |
| Sibukali | 990 | 4,974 | 2,370 | 2,604 |
| Mata | 1,095 | 5,319 | 2,550 | 2,769 |
| **Sesheke District** | **20,159** | **99,384** | **48,913** | **50,471** |
| **Mulobezi Constituency** | **6,075** | **30,482** | **14,851** | **15,631** |
| Nawinda | 1,010 | 5,197 | 2,605 | 2,592 |
| Kamanga | 662 | 3,499 | 1,636 | 1,863 |
| Luamuloba | 783 | 4,020 | 1,990 | 2,030 |
| Sichili | 2,026 | 9,834 | 4,741 | 5,093 |
| Mulobezi | 880 | 4,378 | 2,172 | 2,206 |
| Machile | 714 | 3,554 | 1,707 | 1,847 |
| **Mwandi Constituency** | **5,065** | **25,054** | **12,343** | **12,711** |
| Magumwi | 590 | 3,138 | 1,562 | 1,576 |
| Sankolonga | 316 | 1,579 | 802 | 777 |
| Mabumbu | 818 | 3,928 | 1,975 | 1,953 |
| Mwandi | 808 | 4,271 | 2,050 | 2,221 |
| Simungoma | 213 | 1,038 | 503 | 535 |
| Loanja | 659 | 3,123 | 1,484 | 1,639 |
| Lwazamba | 942 | 4,619 | 2,273 | 2,346 |
| Mushukula | 719 | 3,358 | 1,694 | 1,664 |
| **Sesheke Constituency** | **9,019** | **43,848** | **21,719** | **22,129** |
| Maondo | 1,738 | 8,491 | 4,236 | 4,255 |
| Mulimambango | 4,125 | 19,850 | 9,777 | 10,073 |
| Lusu | 646 | 2,987 | 1,490 | 1,497 |
| Luampungu | 994 | 5,273 | 2,628 | 2,645 |
| Kalobolelwa | 1,045 | 5,139 | 2,574 | 2,565 |
| Imusho | 471 | 2,108 | 1,014 | 1,094 |

## Table A10: Households and Population by (De jure) Sex, Province, District, Constituency and Ward, Zambia 2010

| Province,District,Constituency and Ward | Households | Population | | |
|---|---|---|---|---|
| | | Total | Male | Female |
| **Shang'ombo District** | **19,029** | **93,303** | **44,824** | **48,479** |
| **Sinjembela Constituency** | **19,029** | **93,303** | **44,824** | **48,479** |
| Mambolomoka | 2,333 | 10,994 | 5,185 | 5,809 |
| Keyana | 1,299 | 6,178 | 2,939 | 3,239 |
| Nalwashi | 633 | 3,336 | 1,626 | 1,710 |
| Kalongola | 571 | 2,867 | 1,419 | 1,448 |
| Mbeta | 2,558 | 12,614 | 6,056 | 6,558 |
| Sioma | 1,040 | 5,730 | 2,803 | 2,927 |
| Sikabenga | 1,798 | 9,225 | 4,507 | 4,718 |
| Mutomena | 2,124 | 10,831 | 5,134 | 5,697 |
| Mulamba | 263 | 1,217 | 595 | 622 |
| Kaunga Mashi | 973 | 4,509 | 2,194 | 2,315 |
| Beshe | 530 | 2,722 | 1,304 | 1,418 |
| Sipuma | 1,596 | 7,393 | 3,485 | 3,908 |
| Mulonga | 1,870 | 8,660 | 4,160 | 4,500 |
| Simu | 1,441 | 7,027 | 3,417 | 3,610 |

## Table A11: Population (De Jure) by Age 0-14 Years (Children), 15-34 Years (Youths) and Total Population 0-34 Years by Sex, Province, District, Constituency and Ward, Zambia 2010

| Province,District,Constituency and Ward | 0 -14 Years | | | 15 - 34 Years | | | 0 - 34 Years | | |
|---|---|---|---|---|---|---|---|---|---|
| | Total | Male | Female | Total | Male | Female | Total | Male | Female |
| **Zambia** | **5,943,169** | **2,954,372** | **2,988,797** | **4,623,142** | **2,224,884** | **2,398,258** | **10,566,311** | **5,179,256** | **5,387,055** |
| **Central Province** | **609,144** | **303,521** | **305,623** | **448,501** | **217,901** | **230,600** | **1,057,645** | **521,422** | **536,223** |
| **Chibombo District** | **145,700** | **72,900** | **72,800** | **101,270** | **49,874** | **51,396** | **246,970** | **122,774** | **124,196** |
| **Chisamba Constituency** | **50,206** | **25,233** | **24,973** | **34,462** | **17,045** | **17,417** | **84,668** | **42,278** | **42,390** |
| Muswishi | 8,025 | 4,067 | 3,958 | 5,220 | 2,589 | 2,631 | 13,245 | 6,656 | 6,589 |
| Mulungushi | 3,267 | 1,665 | 1,602 | 2,203 | 1,119 | 1,084 | 5,470 | 2,784 | 2,686 |
| Chikonkomene | 7,961 | 4,035 | 3,926 | 4,989 | 2,529 | 2,460 | 12,950 | 6,564 | 6,386 |
| Chamuka | 9,957 | 4,987 | 4,970 | 7,188 | 3,610 | 3,578 | 17,145 | 8,597 | 8,548 |
| Chisamba | 11,367 | 5,701 | 5,666 | 8,156 | 3,973 | 4,183 | 19,523 | 9,674 | 9,849 |
| Liteta | 9,629 | 4,778 | 4,851 | 6,706 | 3,225 | 3,481 | 16,335 | 8,003 | 8,332 |
| **Katuba Constituency** | **36,507** | **18,330** | **18,177** | **26,935** | **13,371** | **13,564** | **63,442** | **31,701** | **31,741** |
| Katuba | 7,461 | 3,714 | 3,747 | 5,628 | 2,766 | 2,862 | 13,089 | 6,480 | 6,609 |
| Chunga | 3,401 | 1,669 | 1,732 | 2,993 | 1,447 | 1,546 | 6,394 | 3,116 | 3,278 |
| Mungule | 11,552 | 5,794 | 5,758 | 8,743 | 4,401 | 4,342 | 20,295 | 10,195 | 10,100 |
| Muchenje | 5,311 | 2,709 | 2,602 | 3,703 | 1,850 | 1,853 | 9,014 | 4,559 | 4,455 |
| Chilochabalenge | 3,703 | 1,836 | 1,867 | 2,502 | 1,222 | 1,280 | 6,205 | 3,058 | 3,147 |
| Kabile | 5,079 | 2,608 | 2,471 | 3,366 | 1,685 | 1,681 | 8,445 | 4,293 | 4,152 |
| **Keembe Constituency** | **58,987** | **29,337** | **29,650** | **39,873** | **19,458** | **20,415** | **98,860** | **48,795** | **50,065** |
| Chaloshi | 4,312 | 2,125 | 2,187 | 3,310 | 1,607 | 1,703 | 7,622 | 3,732 | 3,890 |
| Kalola | 6,802 | 3,398 | 3,404 | 4,763 | 2,412 | 2,351 | 11,565 | 5,810 | 5,755 |
| Kakoma | 9,163 | 4,563 | 4,600 | 6,081 | 2,948 | 3,133 | 15,244 | 7,511 | 7,733 |
| Chikobo | 2,537 | 1,299 | 1,238 | 1,961 | 950 | 1,011 | 4,498 | 2,249 | 2,249 |
| Chibombo | 4,302 | 2,124 | 2,178 | 3,450 | 1,660 | 1,790 | 7,752 | 3,784 | 3,968 |
| Chitanda | 6,417 | 3,228 | 3,189 | 4,020 | 1,973 | 2,047 | 10,437 | 5,201 | 5,236 |
| Mashikili | 11,456 | 5,690 | 5,766 | 7,440 | 3,635 | 3,805 | 18,896 | 9,325 | 9,571 |
| Keembe | 4,501 | 2,202 | 2,299 | 3,029 | 1,515 | 1,514 | 7,530 | 3,717 | 3,813 |
| Lunjofwa | 4,404 | 2,194 | 2,210 | 2,744 | 1,315 | 1,429 | 7,148 | 3,509 | 3,639 |
| Ipongo | 5,093 | 2,514 | 2,579 | 3,075 | 1,443 | 1,632 | 8,168 | 3,957 | 4,211 |
| **Kabwe District** | **80,706** | **39,518** | **41,188** | **79,838** | **37,867** | **41,971** | **160,544** | **77,385** | **83,159** |
| **Bwacha Constituency** | **35,157** | **17,404** | **17,753** | **30,993** | **14,899** | **16,094** | **66,150** | **32,303** | **33,847** |
| Muwowo | 817 | 397 | 420 | 559 | 281 | 278 | 1,376 | 678 | 698 |
| Muwowo East | 1,809 | 873 | 936 | 1,601 | 744 | 857 | 3,410 | 1,617 | 1,793 |
| Ngungu | 2,122 | 1,037 | 1,085 | 2,426 | 1,149 | 1,277 | 4,548 | 2,186 | 2,362 |
| Chimanimani | 1,802 | 867 | 935 | 1,995 | 935 | 1,060 | 3,797 | 1,802 | 1,995 |
| Bwacha | 3,618 | 1,721 | 1,897 | 3,953 | 1,855 | 2,098 | 7,571 | 3,576 | 3,995 |
| Kawama | 4,375 | 2,189 | 2,186 | 3,830 | 1,809 | 2,021 | 8,205 | 3,998 | 4,207 |
| Ben Kapufi | 1,859 | 958 | 901 | 1,991 | 1,029 | 962 | 3,850 | 1,987 | 1,863 |
| Makululu | 1,612 | 778 | 834 | 1,436 | 680 | 756 | 3,048 | 1,458 | 1,590 |
| Moomba | 4,914 | 2,489 | 2,425 | 3,935 | 1,871 | 2,064 | 8,849 | 4,360 | 4,489 |
| Zambezi | 3,747 | 1,819 | 1,928 | 2,695 | 1,297 | 1,398 | 6,442 | 3,116 | 3,326 |
| Kang'omba | 2,078 | 1,071 | 1,007 | 1,522 | 756 | 766 | 3,600 | 1,827 | 1,773 |
| Chinyanja | 1,491 | 735 | 756 | 1,073 | 528 | 545 | 2,564 | 1,263 | 1,301 |
| Munyama | 1,027 | 541 | 486 | 788 | 399 | 389 | 1,815 | 940 | 875 |
| Munga | 1,504 | 733 | 771 | 1,242 | 618 | 624 | 2,746 | 1,351 | 1,395 |
| Chililalila | 2,382 | 1,196 | 1,186 | 1,947 | 948 | 999 | 4,329 | 2,144 | 2,185 |
| **Kabwe Central Constituency** | **45,549** | **22,114** | **23,435** | **48,845** | **22,968** | **25,877** | **94,394** | **45,082** | **49,312** |
| Mpima | 2,793 | 1,394 | 1,399 | 2,486 | 1,206 | 1,280 | 5,279 | 2,600 | 2,679 |
| Luansase | 1,330 | 655 | 675 | 1,022 | 527 | 495 | 2,352 | 1,182 | 1,170 |
| Waya | 2,265 | 1,091 | 1,174 | 2,085 | 1,052 | 1,033 | 4,350 | 2,143 | 2,207 |
| Chirwa | 10,512 | 5,209 | 5,303 | 11,214 | 5,429 | 5,785 | 21,726 | 10,638 | 11,088 |
| Njanji | 2,024 | 921 | 1,103 | 2,799 | 1,243 | 1,556 | 4,823 | 2,164 | 2,659 |
| Justine Kabwe | 1,982 | 957 | 1,025 | 2,339 | 1,053 | 1,286 | 4,321 | 2,010 | 2,311 |
| David Ramushu | 3,320 | 1,590 | 1,730 | 3,246 | 1,590 | 1,656 | 6,566 | 3,180 | 3,386 |
| Highridge | 2,142 | 1,009 | 1,133 | 3,430 | 1,546 | 1,884 | 5,572 | 2,555 | 3,017 |
| Kalonga | 4,011 | 1,935 | 2,076 | 4,339 | 2,014 | 2,325 | 8,350 | 3,949 | 4,401 |

## Table A11: Population (De Jure) by Age 0-14 Years (Children), 15-34 Years (Youths) and Total Population 0-34 Years by Sex, Province, District, Constituency and Ward, Zambia 2010

| Province,District,Constituency and Ward | 0 -14 Years | | | 15 - 34 Years | | | 0 - 34 Years | | |
|---|---|---|---|---|---|---|---|---|---|
| | Total | Male | Female | Total | Male | Female | Total | Male | Female |
| Luangwa | 5,609 | 2,663 | 2,946 | 6,992 | 3,182 | 3,810 | 12,601 | 5,845 | 6,756 |
| Nakoli | 4,235 | 2,056 | 2,179 | 3,678 | 1,767 | 1,911 | 7,913 | 3,823 | 4,090 |
| Kaputula | 5,326 | 2,634 | 2,692 | 5,215 | 2,359 | 2,856 | 10,541 | 4,993 | 5,548 |
| **Kapiri Mposhi District** | **118,835** | **59,357** | **59,478** | **85,058** | **41,321** | **43,737** | **203,893** | **100,678** | **103,215** |
| **Kapiri Mposhi Constituency** | **118,835** | **59,357** | **59,478** | **85,058** | **41,321** | **43,737** | **203,893** | **100,678** | **103,215** |
| Ngabwe | 2,826 | 1,414 | 1,412 | 1,728 | 857 | 871 | 4,554 | 2,271 | 2,283 |
| Mukumbwe | 7,446 | 3,747 | 3,699 | 4,862 | 2,338 | 2,524 | 12,308 | 6,085 | 6,223 |
| Lwanchele | 7,602 | 3,794 | 3,808 | 4,799 | 2,297 | 2,502 | 12,401 | 6,091 | 6,310 |
| Chipepo | 6,857 | 3,530 | 3,327 | 4,660 | 2,254 | 2,406 | 11,517 | 5,784 | 5,733 |
| Kapandwe | 1,858 | 944 | 914 | 1,238 | 640 | 598 | 3,096 | 1,584 | 1,512 |
| Mpunde | 12,204 | 5,963 | 6,241 | 8,543 | 4,148 | 4,395 | 20,747 | 10,111 | 10,636 |
| Chibwelo | 19,571 | 9,594 | 9,977 | 16,876 | 7,808 | 9,068 | 36,447 | 17,402 | 19,045 |
| Kapiri Mposhi | 6,724 | 3,426 | 3,298 | 4,959 | 2,440 | 2,519 | 11,683 | 5,866 | 5,817 |
| Kashitu | 3,517 | 1,788 | 1,729 | 2,404 | 1,182 | 1,222 | 5,921 | 2,970 | 2,951 |
| Mushimbili | 8,719 | 4,376 | 4,343 | 6,026 | 2,978 | 3,048 | 14,745 | 7,354 | 7,391 |
| Lunchu | 15,211 | 7,576 | 7,635 | 10,110 | 4,980 | 5,130 | 25,321 | 12,556 | 12,765 |
| Chango'ndo | 11,282 | 5,635 | 5,647 | 8,295 | 4,180 | 4,115 | 19,577 | 9,815 | 9,762 |
| Kakwelesa | 12,119 | 6,146 | 5,973 | 8,494 | 4,202 | 4,292 | 20,613 | 10,348 | 10,265 |
| Kampumba | 2,899 | 1,424 | 1,475 | 2,064 | 1,017 | 1,047 | 4,963 | 2,441 | 2,522 |
| **Mkushi District** | **74,037** | **36,678** | **37,359** | **53,008** | **25,885** | **27,123** | **127,045** | **62,563** | **64,482** |
| **Mkushi North Constituency** | **61,410** | **30,319** | **31,091** | **44,796** | **21,805** | **22,991** | **106,206** | **52,124** | **54,082** |
| Masofu | 1,926 | 938 | 988 | 1,357 | 657 | 700 | 3,283 | 1,595 | 1,688 |
| Upper Lunsenfwa | 5,957 | 3,066 | 2,891 | 3,982 | 1,956 | 2,026 | 9,939 | 5,022 | 4,917 |
| Chalata | 6,867 | 3,284 | 3,583 | 4,880 | 2,361 | 2,519 | 11,747 | 5,645 | 6,102 |
| Chibefwe | 8,146 | 3,958 | 4,188 | 7,850 | 3,757 | 4,093 | 15,996 | 7,715 | 8,281 |
| Nkumbi | 5,677 | 2,768 | 2,909 | 4,336 | 2,122 | 2,214 | 10,013 | 4,890 | 5,123 |
| Mushibemba | 5,689 | 2,797 | 2,892 | 4,181 | 2,081 | 2,100 | 9,870 | 4,878 | 4,992 |
| Nshinso | 4,525 | 2,293 | 2,232 | 2,939 | 1,461 | 1,478 | 7,464 | 3,754 | 3,710 |
| Matuku | 8,094 | 3,964 | 4,130 | 5,201 | 2,479 | 2,722 | 13,295 | 6,443 | 6,852 |
| Tembwe | 4,871 | 2,374 | 2,497 | 3,745 | 1,832 | 1,913 | 8,616 | 4,206 | 4,410 |
| Munda | 7,853 | 3,968 | 3,885 | 5,230 | 2,564 | 2,666 | 13,083 | 6,532 | 6,551 |
| Chikanda | 1,805 | 909 | 896 | 1,095 | 535 | 560 | 2,900 | 1,444 | 1,456 |
| **Mkushi South Constituency** | **12,627** | **6,359** | **6,268** | **8,212** | **4,080** | **4,132** | **20,839** | **10,439** | **10,400** |
| Kalwa | 2,070 | 1,041 | 1,029 | 1,253 | 612 | 641 | 3,323 | 1,653 | 1,670 |
| Ching'ombe | 1,114 | 586 | 528 | 692 | 338 | 354 | 1,806 | 924 | 882 |
| Nkomashi | 4,961 | 2,512 | 2,449 | 3,552 | 1,776 | 1,776 | 8,513 | 4,288 | 4,225 |
| Kamimbya | 2,556 | 1,255 | 1,301 | 1,558 | 788 | 770 | 4,114 | 2,043 | 2,071 |
| Chapaba | 864 | 433 | 431 | 529 | 248 | 281 | 1,393 | 681 | 712 |
| Mwalala | 1,062 | 532 | 530 | 628 | 318 | 310 | 1,690 | 850 | 840 |
| **Mumbwa District** | **108,835** | **54,502** | **54,333** | **75,752** | **37,402** | **38,350** | **184,587** | **91,904** | **92,683** |
| **Mwembezhi Constituency** | **24,534** | **12,359** | **12,175** | **18,498** | **9,308** | **9,190** | **43,032** | **21,667** | **21,365** |
| Kapyanga | 2,682 | 1,370 | 1,312 | 1,848 | 892 | 956 | 4,530 | 2,262 | 2,268 |
| Chabota | 3,113 | 1,550 | 1,563 | 1,917 | 938 | 979 | 5,030 | 2,488 | 2,542 |
| Kalundu | 3,758 | 1,908 | 1,850 | 2,532 | 1,250 | 1,282 | 6,290 | 3,158 | 3,132 |
| Milandu | 5,470 | 2,750 | 2,720 | 3,740 | 1,803 | 1,937 | 9,210 | 4,553 | 4,657 |
| Makombwe | 2,374 | 1,183 | 1,191 | 1,837 | 903 | 934 | 4,211 | 2,086 | 2,125 |
| Nampundwe | 7,137 | 3,598 | 3,539 | 6,624 | 3,522 | 3,102 | 13,761 | 7,120 | 6,641 |
| **Mumbwa Constituency** | **41,652** | **20,800** | **20,852** | **30,100** | **14,788** | **15,312** | **71,752** | **35,588** | **36,164** |
| Nalusanga | 5,143 | 2,590 | 2,553 | 3,457 | 1,670 | 1,787 | 8,600 | 4,260 | 4,340 |
| Lutale | 4,995 | 2,486 | 2,509 | 3,106 | 1,510 | 1,596 | 8,101 | 3,996 | 4,105 |
| Kalwanyembe | 4,193 | 2,107 | 2,086 | 2,783 | 1,394 | 1,389 | 6,976 | 3,501 | 3,475 |
| Mpusu | 3,561 | 1,794 | 1,767 | 2,137 | 1,089 | 1,048 | 5,698 | 2,883 | 2,815 |
| Mumba | 7,040 | 3,583 | 3,457 | 4,499 | 2,244 | 2,255 | 11,539 | 5,827 | 5,712 |
| Mupona | 8,298 | 4,011 | 4,287 | 8,455 | 4,068 | 4,387 | 16,753 | 8,079 | 8,674 |
| Chibolyo | 2,918 | 1,458 | 1,460 | 1,791 | 853 | 938 | 4,709 | 2,311 | 2,398 |

## Table A11: Population (De Jure) by Age 0-14 Years (Children), 15-34 Years (Youths) and Total Population 0-34 Years by Sex, Province, District, Constituency and Ward, Zambia 2010

| Province,District,Constituency and Ward | 0 -14 Years | | | 15 - 34 Years | | | 0 - 34 Years | | |
|---|---|---|---|---|---|---|---|---|---|
| | Total | Male | Female | Total | Male | Female | Total | Male | Female |
| Nambala | 2,706 | 1,374 | 1,332 | 1,970 | 1,019 | 951 | 4,676 | 2,393 | 2,283 |
| Shimbizhi | 2,798 | 1,397 | 1,401 | 1,902 | 941 | 961 | 4,700 | 2,338 | 2,362 |
| **Nangoma Constituency** | **42,649** | **21,343** | **21,306** | **27,154** | **13,306** | **13,848** | **69,803** | **34,649** | **35,154** |
| Nakasaka | 5,946 | 3,018 | 2,928 | 4,656 | 2,332 | 2,324 | 10,602 | 5,350 | 5,252 |
| Nangoma | 7,840 | 3,905 | 3,935 | 4,778 | 2,304 | 2,474 | 12,618 | 6,209 | 6,409 |
| Myooye | 2,146 | 1,109 | 1,037 | 1,498 | 740 | 758 | 3,644 | 1,849 | 1,795 |
| Shichanzu | 6,243 | 3,033 | 3,210 | 3,721 | 1,818 | 1,903 | 9,964 | 4,851 | 5,113 |
| Nalubanda | 6,318 | 3,256 | 3,062 | 3,810 | 1,844 | 1,966 | 10,128 | 5,100 | 5,028 |
| Choma | 4,180 | 2,091 | 2,089 | 2,623 | 1,297 | 1,326 | 6,803 | 3,388 | 3,415 |
| Chisalu | 9,976 | 4,931 | 5,045 | 6,068 | 2,971 | 3,097 | 16,044 | 7,902 | 8,142 |
| **Serenje District** | **81,031** | **40,566** | **40,465** | **53,575** | **25,552** | **28,023** | **134,606** | **66,118** | **68,488** |
| **Chitambo Constituency** | **24,112** | **12,088** | **12,024** | **15,166** | **7,143** | **8,023** | **39,278** | **19,231** | **20,047** |
| Lulimala | 4,683 | 2,301 | 2,382 | 2,825 | 1,338 | 1,487 | 7,508 | 3,639 | 3,869 |
| Mpelembe | 1,162 | 574 | 588 | 665 | 295 | 370 | 1,827 | 869 | 958 |
| Chipundu | 3,781 | 1,927 | 1,854 | 2,394 | 1,149 | 1,245 | 6,175 | 3,076 | 3,099 |
| Luombwa | 994 | 479 | 515 | 578 | 254 | 324 | 1,572 | 733 | 839 |
| Chalilo | 3,784 | 1,920 | 1,864 | 2,423 | 1,127 | 1,296 | 6,207 | 3,047 | 3,160 |
| Chitambo | 4,727 | 2,399 | 2,328 | 2,990 | 1,405 | 1,585 | 7,717 | 3,804 | 3,913 |
| Muchinka | 4,981 | 2,488 | 2,493 | 3,291 | 1,575 | 1,716 | 8,272 | 4,063 | 4,209 |
| **Muchinga Constituency** | **26,525** | **13,294** | **13,231** | **16,822** | **8,065** | **8,757** | **43,347** | **21,359** | **21,988** |
| Mailo | 3,757 | 1,878 | 1,879 | 2,259 | 1,050 | 1,209 | 6,016 | 2,928 | 3,088 |
| Kanona | 3,053 | 1,531 | 1,522 | 2,280 | 1,099 | 1,181 | 5,333 | 2,630 | 2,703 |
| Serenje | 2,626 | 1,329 | 1,297 | 1,644 | 763 | 881 | 4,270 | 2,092 | 2,178 |
| Kabansa | 393 | 191 | 202 | 239 | 115 | 124 | 632 | 306 | 326 |
| Chisomo | 1,258 | 627 | 631 | 851 | 413 | 438 | 2,109 | 1,040 | 1,069 |
| Lukusashi | 1,924 | 992 | 932 | 1,178 | 586 | 592 | 3,102 | 1,578 | 1,524 |
| Sancha | 2,848 | 1,472 | 1,376 | 1,677 | 811 | 866 | 4,525 | 2,283 | 2,242 |
| Chibale | 3,990 | 1,953 | 2,037 | 2,451 | 1,203 | 1,248 | 6,441 | 3,156 | 3,285 |
| Masaninga | 6,676 | 3,321 | 3,355 | 4,243 | 2,025 | 2,218 | 10,919 | 5,346 | 5,573 |
| **Serenje Constituency** | **30,394** | **15,184** | **15,210** | **21,587** | **10,344** | **11,243** | **51,981** | **25,528** | **26,453** |
| Kabamba | 3,588 | 1,816 | 1,772 | 2,377 | 1,092 | 1,285 | 5,965 | 2,908 | 3,057 |
| Ibolelo | 9,981 | 4,934 | 5,047 | 8,389 | 3,971 | 4,418 | 18,370 | 8,905 | 9,465 |
| Muchinda | 7,943 | 3,999 | 3,944 | 5,171 | 2,533 | 2,638 | 13,114 | 6,532 | 6,582 |
| Ng'answa | 3,744 | 1,862 | 1,882 | 2,382 | 1,149 | 1,233 | 6,126 | 3,011 | 3,115 |
| Lupiya | 3,833 | 1,906 | 1,927 | 2,397 | 1,199 | 1,198 | 6,230 | 3,105 | 3,125 |
| Musangashi | 1,305 | 667 | 638 | 871 | 400 | 471 | 2,176 | 1,067 | 1,109 |
| **Copperbelt Province** | **806,608** | **398,080** | **408,528** | **756,264** | **369,307** | **386,957** | **1,562,872** | **767,387** | **795,485** |
| **Chililabombwe District** | **37,124** | **18,362** | **18,762** | **37,379** | **19,001** | **18,378** | **74,503** | **37,363** | **37,140** |
| **Chililabombwe Constituency** | **37,124** | **18,362** | **18,762** | **37,379** | **19,001** | **18,378** | **74,503** | **37,363** | **37,140** |
| Chilimina | 1,367 | 700 | 667 | 824 | 398 | 426 | 2,191 | 1,098 | 1,093 |
| Miyanda | 1,139 | 585 | 554 | 672 | 342 | 330 | 1,811 | 927 | 884 |
| Joseph Mwilwa | 1,801 | 896 | 905 | 1,379 | 690 | 689 | 3,180 | 1,586 | 1,594 |
| Anoya Zulu | 563 | 274 | 289 | 549 | 286 | 263 | 1,112 | 560 | 552 |
| Kawama | 586 | 301 | 285 | 464 | 233 | 231 | 1,050 | 534 | 516 |
| Miteta | 2,738 | 1,349 | 1,389 | 1,981 | 984 | 997 | 4,719 | 2,333 | 2,386 |
| Chitambi | 249 | 136 | 113 | 163 | 82 | 81 | 412 | 218 | 194 |
| Kakoso | 2,540 | 1,251 | 1,289 | 2,364 | 1,213 | 1,151 | 4,904 | 2,464 | 2,440 |
| Kafue | 992 | 506 | 486 | 1,913 | 1,005 | 908 | 2,905 | 1,511 | 1,394 |
| Mvula | 940 | 477 | 463 | 1,319 | 680 | 639 | 2,259 | 1,157 | 1,102 |
| Mathew Nkoloma | 2,223 | 1,058 | 1,165 | 2,348 | 1,261 | 1,087 | 4,571 | 2,319 | 2,252 |
| James Phiri | 2,751 | 1,366 | 1,385 | 3,388 | 1,744 | 1,644 | 6,139 | 3,110 | 3,029 |
| Silwizya | 348 | 176 | 172 | 257 | 125 | 132 | 605 | 301 | 304 |
| Helen Kaunda | 1,008 | 456 | 552 | 1,513 | 803 | 710 | 2,521 | 1,259 | 1,262 |
| Chitimukulu | 1,033 | 505 | 528 | 1,276 | 636 | 640 | 2,309 | 1,141 | 1,168 |
| Yeta | 1,786 | 853 | 933 | 1,948 | 997 | 951 | 3,734 | 1,850 | 1,884 |

## Table A11: Population (De Jure) by Age 0-14 Years (Children), 15-34 Years (Youths) and Total Population 0-34 Years by Sex, Province, District, Constituency and Ward, Zambia 2010

| Province,District,Constituency and Ward | 0 -14 Years | | | 15 - 34 Years | | | 0 - 34 Years | | |
|---|---|---|---|---|---|---|---|---|---|
| | Total | Male | Female | Total | Male | Female | Total | Male | Female |
| Ngebe | 1,856 | 943 | 913 | 2,081 | 1,075 | 1,006 | 3,937 | 2,018 | 1,919 |
| Kamima | 1,651 | 846 | 805 | 1,756 | 936 | 820 | 3,407 | 1,782 | 1,625 |
| Mukuka | 2,487 | 1,258 | 1,229 | 2,452 | 1,242 | 1,210 | 4,939 | 2,500 | 2,439 |
| Yotam Muleya | 5,545 | 2,725 | 2,820 | 4,421 | 2,146 | 2,275 | 9,966 | 4,871 | 5,095 |
| Nakatindi | 2,786 | 1,347 | 1,439 | 3,220 | 1,602 | 1,618 | 6,006 | 2,949 | 3,057 |
| Mumba | 735 | 354 | 381 | 1,091 | 521 | 570 | 1,826 | 875 | 951 |
| **Chingola District** | **87,174** | **43,323** | **43,851** | **84,923** | **41,720** | **43,203** | **172,097** | **85,043** | **87,054** |
| **Chingola Constituency** | **52,269** | **25,981** | **26,288** | **48,424** | **23,494** | **24,930** | **100,693** | **49,475** | **51,218** |
| Musenga | 1,304 | 642 | 662 | 962 | 476 | 486 | 2,266 | 1,118 | 1,148 |
| Kasompe | 2,174 | 1,094 | 1,080 | 1,904 | 913 | 991 | 4,078 | 2,007 | 2,071 |
| Mimbula | 2,748 | 1,359 | 1,389 | 2,402 | 1,211 | 1,191 | 5,150 | 2,570 | 2,580 |
| Lulamba | 5,834 | 2,937 | 2,897 | 5,974 | 2,947 | 3,027 | 11,808 | 5,884 | 5,924 |
| Twatasha | 3,429 | 1,777 | 1,652 | 3,145 | 1,522 | 1,623 | 6,574 | 3,299 | 3,275 |
| Gibson Chimfwembe | 1,582 | 821 | 761 | 1,591 | 756 | 835 | 3,173 | 1,577 | 1,596 |
| Chabanyama | 2,998 | 1,483 | 1,515 | 2,961 | 1,438 | 1,523 | 5,959 | 2,921 | 3,038 |
| Maiteneke | 4,763 | 2,369 | 2,394 | 4,674 | 2,292 | 2,382 | 9,437 | 4,661 | 4,776 |
| Chitimukulu | 3,313 | 1,632 | 1,681 | 3,185 | 1,502 | 1,683 | 6,498 | 3,134 | 3,364 |
| Chikola | 3,110 | 1,509 | 1,601 | 3,759 | 1,798 | 1,961 | 6,869 | 3,307 | 3,562 |
| Chiwempala | 5,193 | 2,520 | 2,673 | 4,941 | 2,415 | 2,526 | 10,134 | 4,935 | 5,199 |
| Kabungo | 3,040 | 1,541 | 1,499 | 2,661 | 1,264 | 1,397 | 5,701 | 2,805 | 2,896 |
| Kalilo | 2,911 | 1,473 | 1,438 | 1,794 | 900 | 894 | 4,705 | 2,373 | 2,332 |
| Ipafu | 2,679 | 1,319 | 1,360 | 1,721 | 846 | 875 | 4,400 | 2,165 | 2,235 |
| Muchinshi | 2,012 | 1,015 | 997 | 1,338 | 606 | 732 | 3,350 | 1,621 | 1,729 |
| Mutenda | 2,684 | 1,324 | 1,360 | 1,720 | 819 | 901 | 4,404 | 2,143 | 2,261 |
| Chingola | 2,495 | 1,166 | 1,329 | 3,692 | 1,789 | 1,903 | 6,187 | 2,955 | 3,232 |
| **Nchanga Constituency** | **34,905** | **17,342** | **17,563** | **36,499** | **18,226** | **18,273** | **71,404** | **35,568** | **35,836** |
| Kwacha | 1,373 | 694 | 679 | 2,528 | 1,338 | 1,190 | 3,901 | 2,032 | 1,869 |
| Nchanga | 2,198 | 1,149 | 1,049 | 2,107 | 972 | 1,135 | 4,305 | 2,121 | 2,184 |
| Sekela | 1,977 | 949 | 1,028 | 2,719 | 1,370 | 1,349 | 4,696 | 2,319 | 2,377 |
| Nsansa | 3,920 | 1,945 | 1,975 | 4,426 | 2,237 | 2,189 | 8,346 | 4,182 | 4,164 |
| Buntungwa | 4,545 | 2,320 | 2,225 | 5,386 | 2,739 | 2,647 | 9,931 | 5,059 | 4,872 |
| Kabundi | 2,120 | 1,044 | 1,076 | 3,432 | 1,721 | 1,711 | 5,552 | 2,765 | 2,787 |
| Kasala | 2,475 | 1,210 | 1,265 | 3,052 | 1,501 | 1,551 | 5,527 | 2,711 | 2,816 |
| Kapisha | 14,963 | 7,344 | 7,619 | 11,922 | 5,843 | 6,079 | 26,885 | 13,187 | 13,698 |
| Luano | 297 | 157 | 140 | 202 | 113 | 89 | 499 | 270 | 229 |
| Bupalo | 1,037 | 530 | 507 | 725 | 392 | 333 | 1,762 | 922 | 840 |
| **Kalulushi District** | **41,542** | **20,420** | **21,122** | **37,809** | **18,553** | **19,256** | **79,351** | **38,973** | **40,378** |
| **Kalulushi Constituency** | **41,542** | **20,420** | **21,122** | **37,809** | **18,553** | **19,256** | **79,351** | **38,973** | **40,378** |
| Musakashi | 461 | 228 | 233 | 299 | 128 | 171 | 760 | 356 | 404 |
| Chambishi | 31 | 14 | 17 | 44 | 28 | 16 | 75 | 42 | 33 |
| Twaiteka | 10,013 | 4,845 | 5,168 | 9,579 | 4,781 | 4,798 | 19,592 | 9,626 | 9,966 |
| Lukoshi | 649 | 309 | 340 | 517 | 251 | 266 | 1,166 | 560 | 606 |
| Lulamba | 1,817 | 893 | 924 | 1,663 | 869 | 794 | 3,480 | 1,762 | 1,718 |
| Mwambashi | 1,440 | 748 | 692 | 980 | 515 | 465 | 2,420 | 1,263 | 1,157 |
| Ichimpe | 1,479 | 742 | 737 | 1,153 | 615 | 538 | 2,632 | 1,357 | 1,275 |
| Kalanga | 2,475 | 1,176 | 1,299 | 3,308 | 1,579 | 1,729 | 5,783 | 2,755 | 3,028 |
| Lubuto | 1,418 | 684 | 734 | 1,776 | 816 | 960 | 3,194 | 1,500 | 1,694 |
| Kalungwishi | 1,367 | 656 | 711 | 1,457 | 701 | 756 | 2,824 | 1,357 | 1,467 |
| Luapula | 1,242 | 624 | 618 | 1,298 | 601 | 697 | 2,540 | 1,225 | 1,315 |
| Ngweshi | 1,367 | 643 | 724 | 1,327 | 630 | 697 | 2,694 | 1,273 | 1,421 |
| Kalengwa | 840 | 407 | 433 | 718 | 331 | 387 | 1,558 | 738 | 820 |
| Chibuluma | 1,471 | 724 | 747 | 1,176 | 587 | 589 | 2,647 | 1,311 | 1,336 |
| Kankonshi | 659 | 331 | 328 | 420 | 209 | 211 | 1,079 | 540 | 539 |
| Remmy Chisupa | 1,936 | 980 | 956 | 1,327 | 659 | 668 | 3,263 | 1,639 | 1,624 |
| Buseko | 1,515 | 745 | 770 | 1,325 | 629 | 696 | 2,840 | 1,374 | 1,466 |

## Table A11: Population (De Jure) by Age 0-14 Years (Children), 15-34 Years (Youths) and Total Population 0-34 Years by Sex, Province, District, Constituency and Ward, Zambia 2010

| Province,District,Constituency and Ward | 0 -14 Years | | | 15 - 34 Years | | | 0 - 34 Years | | |
|---|---|---|---|---|---|---|---|---|---|
| | Total | Male | Female | Total | Male | Female | Total | Male | Female |
| Dongwe | 609 | 305 | 304 | 645 | 318 | 327 | 1,254 | 623 | 631 |
| Kafue | 4,729 | 2,329 | 2,400 | 4,192 | 1,966 | 2,226 | 8,921 | 4,295 | 4,626 |
| Chankalamo | 1,349 | 700 | 649 | 1,001 | 511 | 490 | 2,350 | 1,211 | 1,139 |
| Chati | 3,800 | 1,894 | 1,906 | 2,983 | 1,509 | 1,474 | 6,783 | 3,403 | 3,380 |
| Chembe | 875 | 443 | 432 | 621 | 320 | 301 | 1,496 | 763 | 733 |
| **Kitwe District** | **206,176** | **100,585** | **105,591** | **207,771** | **100,930** | **106,841** | **413,947** | **201,515** | **212,432** |
| **Chimwemwe Constituency** | **48,737** | **23,755** | **24,982** | **44,709** | **21,616** | **23,093** | **93,446** | **45,371** | **48,075** |
| Itimpi | 5,064 | 2,510 | 2,554 | 4,127 | 2,067 | 2,060 | 9,191 | 4,577 | 4,614 |
| Twatasha | 10,409 | 5,066 | 5,343 | 8,551 | 4,091 | 4,460 | 18,960 | 9,157 | 9,803 |
| Kawama | 17,411 | 8,484 | 8,927 | 13,202 | 6,340 | 6,862 | 30,613 | 14,824 | 15,789 |
| Buntungwa | 6,442 | 3,112 | 3,330 | 7,419 | 3,595 | 3,824 | 13,861 | 6,707 | 7,154 |
| Lubuto | 4,280 | 2,059 | 2,221 | 5,351 | 2,621 | 2,730 | 9,631 | 4,680 | 4,951 |
| Chimwemwe | 5,131 | 2,524 | 2,607 | 6,059 | 2,902 | 3,157 | 11,190 | 5,426 | 5,764 |
| **Kamfinsa Constituency** | **34,632** | **16,858** | **17,774** | **34,492** | **16,745** | **17,747** | **69,124** | **33,603** | **35,521** |
| Bupe | 6,043 | 2,932 | 3,111 | 7,686 | 3,658 | 4,028 | 13,729 | 6,590 | 7,139 |
| Ndeke | 20,591 | 9,984 | 10,607 | 19,797 | 9,563 | 10,234 | 40,388 | 19,547 | 20,841 |
| Kafue | 3,061 | 1,483 | 1,578 | 2,446 | 1,243 | 1,203 | 5,507 | 2,726 | 2,781 |
| Kamfinsa | 4,937 | 2,459 | 2,478 | 4,563 | 2,281 | 2,282 | 9,500 | 4,740 | 4,760 |
| **Kwacha Constituency** | **52,869** | **26,021** | **26,848** | **52,919** | **25,581** | **27,338** | **105,788** | **51,602** | **54,186** |
| Kwacha | 7,282 | 3,704 | 3,578 | 7,399 | 3,506 | 3,893 | 14,681 | 7,210 | 7,471 |
| Bulangililo | 11,192 | 5,391 | 5,801 | 11,342 | 5,386 | 5,956 | 22,534 | 10,777 | 11,757 |
| Ipusukilo | 18,941 | 9,372 | 9,569 | 15,543 | 7,635 | 7,908 | 34,484 | 17,007 | 17,477 |
| Chantete | 1,359 | 676 | 683 | 907 | 469 | 438 | 2,266 | 1,145 | 1,121 |
| Riverside | 10,194 | 4,931 | 5,263 | 11,891 | 5,667 | 6,224 | 22,085 | 10,598 | 11,487 |
| Lubwa | 3,901 | 1,947 | 1,954 | 5,837 | 2,918 | 2,919 | 9,738 | 4,865 | 4,873 |
| **Nkana Constituency** | **30,704** | **14,880** | **15,824** | **36,077** | **17,603** | **18,474** | **66,781** | **32,483** | **34,298** |
| Rokana | 4,100 | 1,975 | 2,125 | 5,286 | 2,551 | 2,735 | 9,386 | 4,526 | 4,860 |
| Parklands | 1,553 | 778 | 775 | 3,137 | 1,529 | 1,608 | 4,690 | 2,307 | 2,383 |
| Buchi | 9,491 | 4,620 | 4,871 | 10,038 | 4,918 | 5,120 | 19,529 | 9,538 | 9,991 |
| Mukuba | 1,834 | 867 | 967 | 2,716 | 1,267 | 1,449 | 4,550 | 2,134 | 2,416 |
| Miseshi | 6,502 | 3,130 | 3,372 | 7,648 | 3,772 | 3,876 | 14,150 | 6,902 | 7,248 |
| Mindolo | 5,432 | 2,640 | 2,792 | 5,966 | 2,931 | 3,035 | 11,398 | 5,571 | 5,827 |
| Kamakonde | 1,792 | 870 | 922 | 1,286 | 635 | 651 | 3,078 | 1,505 | 1,573 |
| **Wusakile Constituency** | **39,234** | **19,071** | **20,163** | **39,574** | **19,385** | **20,189** | **78,808** | **38,456** | **40,352** |
| Limaposa | 1,026 | 495 | 531 | 748 | 368 | 380 | 1,774 | 863 | 911 |
| Luangwa | 13,343 | 6,603 | 6,740 | 11,026 | 5,326 | 5,700 | 24,369 | 11,929 | 12,440 |
| Chamboli | 8,190 | 3,975 | 4,215 | 9,972 | 5,045 | 4,927 | 18,162 | 9,020 | 9,142 |
| Chibote | 6,022 | 2,855 | 3,167 | 7,524 | 3,724 | 3,800 | 13,546 | 6,579 | 6,967 |
| Wusakile | 10,653 | 5,143 | 5,510 | 10,304 | 4,922 | 5,382 | 20,957 | 10,065 | 10,892 |
| **Luanshya District** | **60,372** | **29,925** | **30,447** | **59,476** | **29,124** | **30,352** | **119,848** | **59,049** | **60,799** |
| **Luanshya Constituency** | **38,801** | **19,296** | **19,505** | **36,631** | **17,891** | **18,740** | **75,432** | **37,187** | **38,245** |
| Misaka | 4,894 | 2,459 | 2,435 | 3,354 | 1,670 | 1,684 | 8,248 | 4,129 | 4,119 |
| Fisenge | 3,027 | 1,556 | 1,471 | 2,193 | 1,108 | 1,085 | 5,220 | 2,664 | 2,556 |
| Twashuka | 313 | 169 | 144 | 240 | 126 | 114 | 553 | 295 | 258 |
| Chitwi | 970 | 469 | 501 | 627 | 327 | 300 | 1,597 | 796 | 801 |
| Chifulube | 319 | 161 | 158 | 232 | 129 | 103 | 551 | 290 | 261 |
| Buntungwa | 6,844 | 3,400 | 3,444 | 5,005 | 2,456 | 2,549 | 11,849 | 5,856 | 5,993 |
| Mpelembe | 1,975 | 956 | 1,019 | 2,810 | 1,365 | 1,445 | 4,785 | 2,321 | 2,464 |
| Buteko | 3,146 | 1,509 | 1,637 | 4,588 | 2,263 | 2,325 | 7,734 | 3,772 | 3,962 |
| James Phiri | 3,278 | 1,636 | 1,642 | 3,169 | 1,493 | 1,676 | 6,447 | 3,129 | 3,318 |
| Levi Chito | 2,754 | 1,354 | 1,400 | 3,016 | 1,469 | 1,547 | 5,770 | 2,823 | 2,947 |
| Mikomfwa | 4,893 | 2,434 | 2,459 | 4,311 | 2,083 | 2,228 | 9,204 | 4,517 | 4,687 |
| Zambezi | 2,559 | 1,263 | 1,296 | 2,609 | 1,265 | 1,344 | 5,168 | 2,528 | 2,640 |
| Mulungushi | 1,864 | 964 | 900 | 2,197 | 1,032 | 1,165 | 4,061 | 1,996 | 2,065 |
| Mipundu | 1,965 | 966 | 999 | 2,280 | 1,105 | 1,175 | 4,245 | 2,071 | 2,174 |

## Table A11: Population (De Jure) by Age 0-14 Years (Children), 15-34 Years (Youths) and Total Population 0-34 Years by Sex, Province, District, Constituency and Ward, Zambia 2010

| Province,District,Constituency and Ward | 0 -14 Years | | | 15 - 34 Years | | | 0 - 34 Years | | |
|---|---|---|---|---|---|---|---|---|---|
| | Total | Male | Female | Total | Male | Female | Total | Male | Female |
| **Roan Constituency** | **21,571** | **10,629** | **10,942** | **22,845** | **11,233** | **11,612** | **44,416** | **21,862** | **22,554** |
| Kafubu | 1,085 | 548 | 537 | 1,279 | 619 | 660 | 2,364 | 1,167 | 1,197 |
| Nkoloma | 1,079 | 535 | 544 | 1,113 | 546 | 567 | 2,192 | 1,081 | 1,111 |
| Lumumba | 2,540 | 1,238 | 1,302 | 3,064 | 1,464 | 1,600 | 5,604 | 2,702 | 2,902 |
| Kafue | 3,346 | 1,642 | 1,704 | 3,152 | 1,534 | 1,618 | 6,498 | 3,176 | 3,322 |
| Chilabula | 810 | 410 | 400 | 518 | 259 | 259 | 1,328 | 669 | 659 |
| Mpatamatu | 1,909 | 924 | 985 | 2,153 | 1,022 | 1,131 | 4,062 | 1,946 | 2,116 |
| Justine Kabwe | 1,702 | 831 | 871 | 1,704 | 811 | 893 | 3,406 | 1,642 | 1,764 |
| Nkulumashiba | 989 | 492 | 497 | 1,206 | 596 | 610 | 2,195 | 1,088 | 1,107 |
| Baluba | 1,284 | 640 | 644 | 1,431 | 726 | 705 | 2,715 | 1,366 | 1,349 |
| Milyashi | 2,489 | 1,222 | 1,267 | 2,528 | 1,269 | 1,259 | 5,017 | 2,491 | 2,526 |
| Ngebe | 1,365 | 676 | 689 | 1,906 | 961 | 945 | 3,271 | 1,637 | 1,634 |
| Kansengu | 1,942 | 949 | 993 | 2,063 | 1,050 | 1,013 | 4,005 | 1,999 | 2,006 |
| Kawama | 808 | 405 | 403 | 570 | 296 | 274 | 1,378 | 701 | 677 |
| Muva Hill | 223 | 117 | 106 | 158 | 80 | 78 | 381 | 197 | 184 |
| **Lufwanyama District** | **37,092** | **18,411** | **18,681** | **24,654** | **12,057** | **12,597** | **61,746** | **30,468** | **31,278** |
| **Lufwanyama Constituency** | **37,092** | **18,411** | **18,681** | **24,654** | **12,057** | **12,597** | **61,746** | **30,468** | **31,278** |
| Kansanta | 3,155 | 1,594 | 1,561 | 2,022 | 954 | 1,068 | 5,177 | 2,548 | 2,629 |
| Kabundia | 3,260 | 1,578 | 1,682 | 2,051 | 998 | 1,053 | 5,311 | 2,576 | 2,735 |
| Boso | 2,810 | 1,378 | 1,432 | 1,906 | 936 | 970 | 4,716 | 2,314 | 2,402 |
| Kansonka | 2,917 | 1,473 | 1,444 | 2,086 | 999 | 1,087 | 5,003 | 2,472 | 2,531 |
| Kafubu | 3,246 | 1,652 | 1,594 | 2,205 | 1,107 | 1,098 | 5,451 | 2,759 | 2,692 |
| Chibanga | 5,492 | 2,715 | 2,777 | 3,931 | 1,915 | 2,016 | 9,423 | 4,630 | 4,793 |
| Sokotwe | 2,252 | 1,135 | 1,117 | 1,459 | 706 | 753 | 3,711 | 1,841 | 1,870 |
| Mibenge | 1,548 | 807 | 741 | 1,087 | 571 | 516 | 2,635 | 1,378 | 1,257 |
| Chantete | 376 | 196 | 180 | 276 | 144 | 132 | 652 | 340 | 312 |
| Bulaya | 262 | 129 | 133 | 155 | 77 | 78 | 417 | 206 | 211 |
| Mukumbo | 2,151 | 1,046 | 1,105 | 1,430 | 738 | 692 | 3,581 | 1,784 | 1,797 |
| Mwelushi | 2,096 | 1,065 | 1,031 | 1,367 | 677 | 690 | 3,463 | 1,742 | 1,721 |
| Mpindi | 1,889 | 890 | 999 | 1,148 | 552 | 596 | 3,037 | 1,442 | 1,595 |
| Lufwanyama | 1,571 | 790 | 781 | 1,002 | 466 | 536 | 2,573 | 1,256 | 1,317 |
| Luswishi | 1,798 | 871 | 927 | 1,099 | 534 | 565 | 2,897 | 1,405 | 1,492 |
| Mushingashi | 2,269 | 1,092 | 1,177 | 1,430 | 683 | 747 | 3,699 | 1,775 | 1,924 |
| **Masaiti District** | **47,800** | **24,080** | **23,720** | **33,740** | **16,485** | **17,255** | **81,540** | **40,565** | **40,975** |
| **Kafulafuta Constituency** | **20,302** | **10,189** | **10,113** | **13,839** | **6,807** | **7,032** | **34,141** | **16,996** | **17,145** |
| Mwatishi | 2,672 | 1,366 | 1,306 | 1,848 | 919 | 929 | 4,520 | 2,285 | 2,235 |
| Majaliwa | 3,331 | 1,677 | 1,654 | 2,182 | 1,088 | 1,094 | 5,513 | 2,765 | 2,748 |
| Chondwe | 4,285 | 2,146 | 2,139 | 3,110 | 1,464 | 1,646 | 7,395 | 3,610 | 3,785 |
| Mutaba | 3,294 | 1,622 | 1,672 | 2,131 | 1,064 | 1,067 | 5,425 | 2,686 | 2,739 |
| Miengwe | 4,013 | 2,002 | 2,011 | 2,862 | 1,399 | 1,463 | 6,875 | 3,401 | 3,474 |
| Katonte | 419 | 213 | 206 | 250 | 129 | 121 | 669 | 342 | 327 |
| Ishitwe | 2,288 | 1,163 | 1,125 | 1,456 | 744 | 712 | 3,744 | 1,907 | 1,837 |
| **Masaiti Constituency** | **27,498** | **13,891** | **13,607** | **19,901** | **9,678** | **10,223** | **47,399** | **23,569** | **23,830** |
| Kashitu | 3,460 | 1,785 | 1,675 | 2,415 | 1,186 | 1,229 | 5,875 | 2,971 | 2,904 |
| Luansobe | 2,517 | 1,289 | 1,228 | 1,838 | 933 | 905 | 4,355 | 2,222 | 2,133 |
| Chinondo | 1,616 | 836 | 780 | 1,045 | 508 | 537 | 2,661 | 1,344 | 1,317 |
| Mishikishi | 2,733 | 1,326 | 1,407 | 2,057 | 957 | 1,100 | 4,790 | 2,283 | 2,507 |
| Masangano | 4,060 | 2,026 | 2,034 | 3,058 | 1,497 | 1,561 | 7,118 | 3,523 | 3,595 |
| Lumano | 3,864 | 2,000 | 1,864 | 2,679 | 1,314 | 1,365 | 6,543 | 3,314 | 3,229 |
| Katuba | 2,583 | 1,281 | 1,302 | 1,836 | 916 | 920 | 4,419 | 2,197 | 2,222 |
| Shimibanga | 3,267 | 1,622 | 1,645 | 2,294 | 1,099 | 1,195 | 5,561 | 2,721 | 2,840 |
| Chilulu | 1,409 | 713 | 696 | 1,193 | 564 | 629 | 2,602 | 1,277 | 1,325 |
| Miputu | 1,989 | 1,013 | 976 | 1,486 | 704 | 782 | 3,475 | 1,717 | 1,758 |

## Table A11: Population (De Jure) by Age 0-14 Years (Children), 15-34 Years (Youths) and Total Population 0-34 Years by Sex, Province, District, Constituency and Ward, Zambia 2010

| Province,District,Constituency and Ward | 0 -14 Years | | | 15 - 34 Years | | | 0 - 34 Years | | |
|---|---|---|---|---|---|---|---|---|---|
| | Total | Male | Female | Total | Male | Female | Total | Male | Female |
| **Mpongwe District** | **44,356** | **22,236** | **22,120** | **31,047** | **15,176** | **15,871** | **75,403** | **37,412** | **37,991** |
| **Mpongwe Constituency** | **44,356** | **22,236** | **22,120** | **31,047** | **15,176** | **15,871** | **75,403** | **37,412** | **37,991** |
| Luswishi | 830 | 387 | 443 | 534 | 242 | 292 | 1,364 | 629 | 735 |
| Kasonga | 1,109 | 546 | 563 | 724 | 383 | 341 | 1,833 | 929 | 904 |
| Munkumpu | 4,183 | 2,101 | 2,082 | 2,808 | 1,378 | 1,430 | 6,991 | 3,479 | 3,512 |
| Kashiba | 4,560 | 2,266 | 2,294 | 3,133 | 1,541 | 1,592 | 7,693 | 3,807 | 3,886 |
| Mpongwe | 4,928 | 2,443 | 2,485 | 4,020 | 1,966 | 2,054 | 8,948 | 4,409 | 4,539 |
| Kanyenda | 4,013 | 2,049 | 1,964 | 2,778 | 1,378 | 1,400 | 6,791 | 3,427 | 3,364 |
| Kasamba | 940 | 499 | 441 | 590 | 294 | 296 | 1,530 | 793 | 737 |
| Ibenga | 7,457 | 3,728 | 3,729 | 5,155 | 2,424 | 2,731 | 12,612 | 6,152 | 6,460 |
| Kalweo | 3,843 | 1,970 | 1,873 | 2,545 | 1,260 | 1,285 | 6,388 | 3,230 | 3,158 |
| Mikata | 4,977 | 2,482 | 2,495 | 3,451 | 1,706 | 1,745 | 8,428 | 4,188 | 4,240 |
| Nampamba | 6,025 | 2,975 | 3,050 | 4,253 | 2,052 | 2,201 | 10,278 | 5,027 | 5,251 |
| Musofu | 1,491 | 790 | 701 | 1,056 | 552 | 504 | 2,547 | 1,342 | 1,205 |
| **Mufulira District** | **63,955** | **31,948** | **32,007** | **63,736** | **31,338** | **32,398** | **127,691** | **63,286** | **64,405** |
| **Kankoyo Constituency** | **18,809** | **9,379** | **9,430** | **17,336** | **8,598** | **8,738** | **36,145** | **17,977** | **18,168** |
| John Kampengele | 2,403 | 1,193 | 1,210 | 2,267 | 1,111 | 1,156 | 4,670 | 2,304 | 2,366 |
| Buntungwa | 1,629 | 780 | 849 | 1,673 | 830 | 843 | 3,302 | 1,610 | 1,692 |
| Kangwa Nsuluka | 446 | 253 | 193 | 352 | 183 | 169 | 798 | 436 | 362 |
| Luansobe | 3,048 | 1,565 | 1,483 | 2,053 | 988 | 1,065 | 5,101 | 2,553 | 2,548 |
| Butondo | 3,337 | 1,614 | 1,723 | 3,403 | 1,734 | 1,669 | 6,740 | 3,348 | 3,392 |
| Kwacha | 2,143 | 1,052 | 1,091 | 2,182 | 1,090 | 1,092 | 4,325 | 2,142 | 2,183 |
| Fibusa | 2,562 | 1,300 | 1,262 | 2,355 | 1,127 | 1,228 | 4,917 | 2,427 | 2,490 |
| Mpelembe | 3,241 | 1,622 | 1,619 | 3,051 | 1,535 | 1,516 | 6,292 | 3,157 | 3,135 |
| **Kantanshi Constituency** | **20,618** | **10,227** | **10,391** | **23,791** | **11,882** | **11,909** | **44,409** | **22,109** | **22,300** |
| Minambe | 1,747 | 847 | 900 | 1,139 | 520 | 619 | 2,886 | 1,367 | 1,519 |
| Murundu | 3,475 | 1,749 | 1,726 | 2,380 | 1,185 | 1,195 | 5,855 | 2,934 | 2,921 |
| Francis Mukuka | 2,552 | 1,288 | 1,264 | 3,487 | 1,770 | 1,717 | 6,039 | 3,058 | 2,981 |
| Maina Soko | 1,474 | 734 | 740 | 2,272 | 1,100 | 1,172 | 3,746 | 1,834 | 1,912 |
| Bwafwano | 1,622 | 786 | 836 | 1,693 | 806 | 887 | 3,315 | 1,592 | 1,723 |
| Mulungushi | 2,834 | 1,427 | 1,407 | 4,090 | 2,086 | 2,004 | 6,924 | 3,513 | 3,411 |
| Shinde | 3,814 | 1,890 | 1,924 | 4,494 | 2,257 | 2,237 | 8,308 | 4,147 | 4,161 |
| Bwembya Silwizya | 1,281 | 631 | 650 | 1,585 | 799 | 786 | 2,866 | 1,430 | 1,436 |
| Leya Mukutu | 1,425 | 697 | 728 | 1,842 | 943 | 899 | 3,267 | 1,640 | 1,627 |
| David Lunda | 394 | 178 | 216 | 809 | 416 | 393 | 1,203 | 594 | 609 |
| **Mufurila Constituency** | **24,528** | **12,342** | **12,186** | **22,609** | **10,858** | **11,751** | **47,137** | **23,200** | **23,937** |
| Mutundu | 2,061 | 1,046 | 1,015 | 1,463 | 757 | 706 | 3,524 | 1,803 | 1,721 |
| Bwananyina | 5,100 | 2,590 | 2,510 | 3,759 | 1,840 | 1,919 | 8,859 | 4,430 | 4,429 |
| David Kaunda | 1,604 | 775 | 829 | 1,957 | 918 | 1,039 | 3,561 | 1,693 | 1,868 |
| Chachacha | 4,010 | 1,974 | 2,036 | 4,150 | 1,957 | 2,193 | 8,160 | 3,931 | 4,229 |
| Kamuchanga | 1,493 | 728 | 765 | 1,715 | 784 | 931 | 3,208 | 1,512 | 1,696 |
| Kasempa | 1,705 | 859 | 846 | 1,848 | 902 | 946 | 3,553 | 1,761 | 1,792 |
| Kansuswa | 2,331 | 1,188 | 1,143 | 2,074 | 1,002 | 1,072 | 4,405 | 2,190 | 2,215 |
| Kafue | 415 | 208 | 207 | 348 | 177 | 171 | 763 | 385 | 378 |
| Kawama | 3,061 | 1,569 | 1,492 | 2,208 | 1,045 | 1,163 | 5,269 | 2,614 | 2,655 |
| Hanky Kalanga | 2,748 | 1,405 | 1,343 | 3,087 | 1,476 | 1,611 | 5,835 | 2,881 | 2,954 |
| **Ndola District** | **181,017** | **88,790** | **92,227** | **175,729** | **84,923** | **90,806** | **356,746** | **173,713** | **183,033** |
| **Bwana Mkumbwa Constituency** | **48,498** | **23,792** | **24,706** | **45,665** | **22,024** | **23,641** | **94,163** | **45,816** | **48,347** |
| Itawa | 7,294 | 3,543 | 3,751 | 8,945 | 4,291 | 4,654 | 16,239 | 7,834 | 8,405 |
| Munkulungwe | 7,319 | 3,608 | 3,711 | 6,290 | 3,064 | 3,226 | 13,609 | 6,672 | 6,937 |
| Twashuka | 6,430 | 3,223 | 3,207 | 4,763 | 2,355 | 2,408 | 11,193 | 5,578 | 5,615 |
| Kavu | 2,101 | 1,050 | 1,051 | 1,687 | 854 | 833 | 3,788 | 1,904 | 1,884 |
| Mushili | 18,783 | 9,207 | 9,576 | 18,484 | 8,837 | 9,647 | 37,267 | 18,044 | 19,223 |
| Chichele | 3,540 | 1,706 | 1,834 | 2,892 | 1,370 | 1,522 | 6,432 | 3,076 | 3,356 |
| Kantolomba | 3,031 | 1,455 | 1,576 | 2,604 | 1,253 | 1,351 | 5,635 | 2,708 | 2,927 |

## Table A11: Population (De Jure) by Age 0-14 Years (Children), 15-34 Years (Youths) and Total Population 0-34 Years by Sex, Province, District, Constituency and Ward, Zambia 2010

| Province,District,Constituency and Ward | 0 -14 Years | | | 15 - 34 Years | | | 0 - 34 Years | | |
|---|---|---|---|---|---|---|---|---|---|
| | Total | Male | Female | Total | Male | Female | Total | Male | Female |
| **Chifubu Constituency** | **39,392** | **19,356** | **20,036** | **38,824** | **18,843** | **19,981** | **78,216** | **38,199** | **40,017** |
| Pamodzi | 16,375 | 7,923 | 8,452 | 15,698 | 7,565 | 8,133 | 32,073 | 15,488 | 16,585 |
| Kawama | 7,781 | 3,890 | 3,891 | 6,591 | 3,285 | 3,306 | 14,372 | 7,175 | 7,197 |
| Fibobe | 5,717 | 2,790 | 2,927 | 6,275 | 3,042 | 3,233 | 11,992 | 5,832 | 6,160 |
| Chifubu | 5,016 | 2,512 | 2,504 | 5,452 | 2,619 | 2,833 | 10,468 | 5,131 | 5,337 |
| Kamba | 4,503 | 2,241 | 2,262 | 4,808 | 2,332 | 2,476 | 9,311 | 4,573 | 4,738 |
| **Kabushi Constituency** | **36,640** | **17,841** | **18,799** | **38,051** | **18,326** | **19,725** | **74,691** | **36,167** | **38,524** |
| Lubuto | 8,546 | 4,197 | 4,349 | 9,555 | 4,610 | 4,945 | 18,101 | 8,807 | 9,294 |
| Mukuba | 3,891 | 1,890 | 2,001 | 3,777 | 1,830 | 1,947 | 7,668 | 3,720 | 3,948 |
| Toka | 3,164 | 1,544 | 1,620 | 3,294 | 1,571 | 1,723 | 6,458 | 3,115 | 3,343 |
| Kaloko | 3,590 | 1,711 | 1,879 | 3,704 | 1,761 | 1,943 | 7,294 | 3,472 | 3,822 |
| Kabushi | 5,205 | 2,551 | 2,654 | 4,794 | 2,329 | 2,465 | 9,999 | 4,880 | 5,119 |
| Kafubu | 2,698 | 1,304 | 1,394 | 2,789 | 1,359 | 1,430 | 5,487 | 2,663 | 2,824 |
| Skyways | 6,012 | 2,923 | 3,089 | 6,435 | 3,074 | 3,361 | 12,447 | 5,997 | 6,450 |
| Masala | 3,534 | 1,721 | 1,813 | 3,703 | 1,792 | 1,911 | 7,237 | 3,513 | 3,724 |
| **Ndola Central Constituency** | **56,487** | **27,801** | **28,686** | **53,189** | **25,730** | **27,459** | **109,676** | **53,531** | **56,145** |
| Kansenshi | 3,445 | 1,633 | 1,812 | 5,512 | 2,541 | 2,971 | 8,957 | 4,174 | 4,783 |
| Nkwazi | 9,659 | 4,835 | 4,824 | 7,906 | 3,923 | 3,983 | 17,565 | 8,758 | 8,807 |
| Yengwe | 5,176 | 2,451 | 2,725 | 7,491 | 3,509 | 3,982 | 12,667 | 5,960 | 6,707 |
| Chipulukusu | 16,865 | 8,349 | 8,516 | 13,338 | 6,527 | 6,811 | 30,203 | 14,876 | 15,327 |
| Kanini | 3,784 | 1,805 | 1,979 | 5,017 | 2,314 | 2,703 | 8,801 | 4,119 | 4,682 |
| Twapia | 12,921 | 6,373 | 6,548 | 10,606 | 5,165 | 5,441 | 23,527 | 11,538 | 11,989 |
| Dag Hammerskjoeld | 4,063 | 2,081 | 1,982 | 2,893 | 1,526 | 1,367 | 6,956 | 3,607 | 3,349 |
| Kaniki | 574 | 274 | 300 | 426 | 225 | 201 | 1,000 | 499 | 501 |
| **Eastern Province** | **753,567** | **376,749** | **376,818** | **527,579** | **257,254** | **270,325** | **1,281,146** | **634,003** | **647,143** |
| **Chadiza District** | **51,908** | **25,922** | **25,986** | **35,101** | **17,581** | **17,520** | **87,009** | **43,503** | **43,506** |
| **Chadiza Constituency** | **30,015** | **15,041** | **14,974** | **20,779** | **10,290** | **10,489** | **50,794** | **25,331** | **25,463** |
| Mangwe | 5,138 | 2,612 | 2,526 | 3,322 | 1,646 | 1,676 | 8,460 | 4,258 | 4,202 |
| Nsadzu | 2,815 | 1,432 | 1,383 | 1,863 | 897 | 966 | 4,678 | 2,329 | 2,349 |
| Manje | 3,283 | 1,656 | 1,627 | 2,218 | 1,109 | 1,109 | 5,501 | 2,765 | 2,736 |
| Chanjowe | 1,249 | 674 | 575 | 862 | 436 | 426 | 2,111 | 1,110 | 1,001 |
| Kapachi | 1,934 | 957 | 977 | 1,287 | 639 | 648 | 3,221 | 1,596 | 1,625 |
| Chadiza | 2,284 | 1,103 | 1,181 | 2,418 | 1,209 | 1,209 | 4,702 | 2,312 | 2,390 |
| Chilenga | 2,150 | 1,062 | 1,088 | 1,461 | 714 | 747 | 3,611 | 1,776 | 1,835 |
| Naviluri | 3,204 | 1,594 | 1,610 | 1,950 | 960 | 990 | 5,154 | 2,554 | 2,600 |
| Chamandala | 1,945 | 936 | 1,009 | 1,333 | 676 | 657 | 3,278 | 1,612 | 1,666 |
| Kandabwako | 2,003 | 982 | 1,021 | 1,277 | 624 | 653 | 3,280 | 1,606 | 1,674 |
| Kampini | 2,083 | 1,061 | 1,022 | 1,466 | 716 | 750 | 3,549 | 1,777 | 1,772 |
| Tafelansoni | 1,927 | 972 | 955 | 1,322 | 664 | 658 | 3,249 | 1,636 | 1,613 |
| **Vubwi Constituency** | **21,893** | **10,881** | **11,012** | **14,322** | **7,291** | **7,031** | **36,215** | **18,172** | **18,043** |
| Ambidzi | 1,380 | 688 | 692 | 926 | 502 | 424 | 2,306 | 1,190 | 1,116 |
| Kabvumo | 1,288 | 659 | 629 | 840 | 407 | 433 | 2,128 | 1,066 | 1,062 |
| Khumba | 1,813 | 908 | 905 | 1,054 | 532 | 522 | 2,867 | 1,440 | 1,427 |
| Mwangazi | 2,482 | 1,293 | 1,189 | 1,573 | 775 | 798 | 4,055 | 2,068 | 1,987 |
| Mbozi | 2,084 | 1,015 | 1,069 | 1,327 | 685 | 642 | 3,411 | 1,700 | 1,711 |
| Vubwi | 4,112 | 1,997 | 2,115 | 2,874 | 1,464 | 1,410 | 6,986 | 3,461 | 3,525 |
| Chisiya | 1,160 | 583 | 577 | 842 | 435 | 407 | 2,002 | 1,018 | 984 |
| Chimphanje | 1,116 | 557 | 559 | 744 | 391 | 353 | 1,860 | 948 | 912 |
| Mlawe | 2,359 | 1,157 | 1,202 | 1,473 | 761 | 712 | 3,832 | 1,918 | 1,914 |
| Zozwe | 4,099 | 2,024 | 2,075 | 2,669 | 1,339 | 1,330 | 6,768 | 3,363 | 3,405 |
| **Chipata District** | **209,305** | **104,156** | **105,149** | **157,103** | **76,832** | **80,271** | **366,408** | **180,988** | **185,420** |
| **Chipangali Constituency** | **59,123** | **29,564** | **29,559** | **39,535** | **19,727** | **19,808** | **98,658** | **49,291** | **49,367** |
| Sisinje | 8,996 | 4,424 | 4,572 | 6,092 | 3,066 | 3,026 | 15,088 | 7,490 | 7,598 |
| Nthope | 15,880 | 7,974 | 7,906 | 10,560 | 5,302 | 5,258 | 26,440 | 13,276 | 13,164 |
| Chipangali | 10,115 | 5,030 | 5,085 | 6,631 | 3,289 | 3,342 | 16,746 | 8,319 | 8,427 |

## Table A11: Population (De Jure) by Age 0-14 Years (Children), 15-34 Years (Youths) and Total Population 0-34 Years by Sex, Province, District, Constituency and Ward, Zambia 2010

| Province,District,Constituency and Ward | 0 -14 Years | | | 15 - 34 Years | | | 0 - 34 Years | | |
|---|---|---|---|---|---|---|---|---|---|
| | Total | Male | Female | Total | Male | Female | Total | Male | Female |
| Kasenga | 6,929 | 3,445 | 3,484 | 4,612 | 2,277 | 2,335 | 11,541 | 5,722 | 5,819 |
| Rukuzye | 9,549 | 4,764 | 4,785 | 6,521 | 3,238 | 3,283 | 16,070 | 8,002 | 8,068 |
| Msandile | 7,654 | 3,927 | 3,727 | 5,119 | 2,555 | 2,564 | 12,773 | 6,482 | 6,291 |
| **Chipata Central Constituency** | **68,392** | **33,454** | **34,938** | **61,075** | **29,553** | **31,522** | **129,467** | **63,007** | **66,460** |
| Msanga | 28,738 | 13,926 | 14,812 | 25,408 | 12,327 | 13,081 | 54,146 | 26,253 | 27,893 |
| Kanjala | 15,279 | 7,568 | 7,711 | 13,394 | 6,498 | 6,896 | 28,673 | 14,066 | 14,607 |
| Dilika | 10,663 | 5,353 | 5,310 | 8,126 | 4,032 | 4,094 | 18,789 | 9,385 | 9,404 |
| Kapata | 13,712 | 6,607 | 7,105 | 14,147 | 6,696 | 7,451 | 27,859 | 13,303 | 14,556 |
| **Kasenengwa Constituency** | **47,166** | **23,593** | **23,573** | **31,342** | **15,186** | **16,156** | **78,508** | **38,779** | **39,729** |
| Makungwa | 5,927 | 2,979 | 2,948 | 4,077 | 1,935 | 2,142 | 10,004 | 4,914 | 5,090 |
| Chingazi | 7,354 | 3,572 | 3,782 | 4,643 | 2,291 | 2,352 | 11,997 | 5,863 | 6,134 |
| Kwenje | 14,306 | 7,213 | 7,093 | 9,601 | 4,626 | 4,975 | 23,907 | 11,839 | 12,068 |
| Ng'ongwe | 7,584 | 3,815 | 3,769 | 4,839 | 2,283 | 2,556 | 12,423 | 6,098 | 6,325 |
| Mkowe | 990 | 495 | 495 | 651 | 335 | 316 | 1,641 | 830 | 811 |
| Mboza | 5,563 | 2,806 | 2,757 | 3,821 | 1,891 | 1,930 | 9,384 | 4,697 | 4,687 |
| Chiparamba | 5,442 | 2,713 | 2,729 | 3,710 | 1,825 | 1,885 | 9,152 | 4,538 | 4,614 |
| **Luangeni Constituency** | **34,624** | **17,545** | **17,079** | **25,151** | **12,366** | **12,785** | **59,775** | **29,911** | **29,864** |
| Nsingo | 9,000 | 4,539 | 4,461 | 7,337 | 3,624 | 3,713 | 16,337 | 8,163 | 8,174 |
| Khova | 5,030 | 2,570 | 2,460 | 3,504 | 1,764 | 1,740 | 8,534 | 4,334 | 4,200 |
| Makangila | 3,501 | 1,831 | 1,670 | 2,413 | 1,163 | 1,250 | 5,914 | 2,994 | 2,920 |
| Chikando | 11,604 | 5,835 | 5,769 | 8,328 | 4,091 | 4,237 | 19,932 | 9,926 | 10,006 |
| Kazimule | 5,489 | 2,770 | 2,719 | 3,569 | 1,724 | 1,845 | 9,058 | 4,494 | 4,564 |
| **Katete District** | **117,098** | **58,515** | **58,583** | **79,798** | **39,039** | **40,759** | **196,896** | **97,554** | **99,342** |
| **Milanzi Constituency** | **31,558** | **15,804** | **15,754** | **20,976** | **10,359** | **10,617** | **52,534** | **26,163** | **26,371** |
| Kafumbwe | 3,747 | 1,870 | 1,877 | 2,281 | 1,115 | 1,166 | 6,028 | 2,985 | 3,043 |
| Kazala | 4,225 | 2,118 | 2,107 | 2,782 | 1,403 | 1,379 | 7,007 | 3,521 | 3,486 |
| Milanzi | 2,704 | 1,346 | 1,358 | 1,736 | 850 | 886 | 4,440 | 2,196 | 2,244 |
| Kapoche | 1,205 | 629 | 576 | 767 | 379 | 388 | 1,972 | 1,008 | 964 |
| Chindwale | 2,188 | 1,054 | 1,134 | 1,476 | 721 | 755 | 3,664 | 1,775 | 1,889 |
| Kapangulula | 4,278 | 2,148 | 2,130 | 2,911 | 1,456 | 1,455 | 7,189 | 3,604 | 3,585 |
| Dole | 4,206 | 2,096 | 2,110 | 2,944 | 1,446 | 1,498 | 7,150 | 3,542 | 3,608 |
| Chimwa | 891 | 436 | 455 | 572 | 283 | 289 | 1,463 | 719 | 744 |
| Mwandafisi | 2,812 | 1,416 | 1,396 | 2,029 | 974 | 1,055 | 4,841 | 2,390 | 2,451 |
| Katiula | 5,302 | 2,691 | 2,611 | 3,478 | 1,732 | 1,746 | 8,780 | 4,423 | 4,357 |
| **Mkaika Constituency** | **45,301** | **22,678** | **22,623** | **32,333** | **15,757** | **16,576** | **77,634** | **38,435** | **39,199** |
| Mphangwe | 8,150 | 4,030 | 4,120 | 7,614 | 3,626 | 3,988 | 15,764 | 7,656 | 8,108 |
| Chavuka | 4,776 | 2,392 | 2,384 | 3,370 | 1,652 | 1,718 | 8,146 | 4,044 | 4,102 |
| Kadula | 4,607 | 2,306 | 2,301 | 2,943 | 1,440 | 1,503 | 7,550 | 3,746 | 3,804 |
| Vulamkoko | 5,302 | 2,665 | 2,637 | 3,379 | 1,691 | 1,688 | 8,681 | 4,356 | 4,325 |
| Chimtende | 4,820 | 2,338 | 2,482 | 3,107 | 1,544 | 1,563 | 7,927 | 3,882 | 4,045 |
| Lukweta | 3,063 | 1,556 | 1,507 | 1,897 | 947 | 950 | 4,960 | 2,503 | 2,457 |
| Mkaika | 11,471 | 5,820 | 5,651 | 7,973 | 3,887 | 4,086 | 19,444 | 9,707 | 9,737 |
| Matunga | 3,112 | 1,571 | 1,541 | 2,050 | 970 | 1,080 | 5,162 | 2,541 | 2,621 |
| **Sinda Constituency** | **40,239** | **20,033** | **20,206** | **26,489** | **12,923** | **13,566** | **66,728** | **32,956** | **33,772** |
| Kamwaza | 3,278 | 1,616 | 1,662 | 2,341 | 1,145 | 1,196 | 5,619 | 2,761 | 2,858 |
| Nchingilizya | 2,430 | 1,217 | 1,213 | 1,634 | 838 | 796 | 4,064 | 2,055 | 2,009 |
| Luandazi | 3,829 | 1,874 | 1,955 | 2,481 | 1,230 | 1,251 | 6,310 | 3,104 | 3,206 |
| Chiwuyu | 4,001 | 2,022 | 1,979 | 2,707 | 1,317 | 1,390 | 6,708 | 3,339 | 3,369 |
| Sinda | 6,347 | 3,141 | 3,206 | 4,452 | 2,136 | 2,316 | 10,799 | 5,277 | 5,522 |
| Mnyamanzi | 2,750 | 1,356 | 1,394 | 1,674 | 819 | 855 | 4,424 | 2,175 | 2,249 |
| Nyamasonkho | 4,908 | 2,491 | 2,417 | 3,368 | 1,705 | 1,663 | 8,276 | 4,196 | 4,080 |
| Mng'omba | 5,381 | 2,691 | 2,690 | 3,263 | 1,549 | 1,714 | 8,644 | 4,240 | 4,404 |
| Kasangazi | 5,145 | 2,549 | 2,596 | 3,209 | 1,547 | 1,662 | 8,354 | 4,096 | 4,258 |
| Chitawe | 2,170 | 1,076 | 1,094 | 1,360 | 637 | 723 | 3,530 | 1,713 | 1,817 |

## Table A11: Population (De Jure) by Age 0-14 Years (Children), 15-34 Years (Youths) and Total Population 0-34 Years by Sex, Province, District, Constituency and Ward, Zambia 2010

| Province,District,Constituency and Ward | 0 -14 Years | | | 15 - 34 Years | | | 0 - 34 Years | | |
|---|---|---|---|---|---|---|---|---|---|
| | Total | Male | Female | Total | Male | Female | Total | Male | Female |
| **Lundazi District** | **156,077** | **77,734** | **78,343** | **103,270** | **49,653** | **53,617** | **259,347** | **127,387** | **131,960** |
| **Chasefu Constituency** | **48,715** | **24,335** | **24,380** | **30,958** | **14,959** | **15,999** | **79,673** | **39,294** | **40,379** |
| Manda Hill | 2,140 | 1,083 | 1,057 | 1,343 | 645 | 698 | 3,483 | 1,728 | 1,755 |
| Magodi | 8,707 | 4,364 | 4,343 | 5,485 | 2,580 | 2,905 | 14,192 | 6,944 | 7,248 |
| Susa | 5,295 | 2,689 | 2,606 | 3,209 | 1,536 | 1,673 | 8,504 | 4,225 | 4,279 |
| Luwerezi | 2,476 | 1,259 | 1,217 | 1,539 | 763 | 776 | 4,015 | 2,022 | 1,993 |
| Kajilime | 9,061 | 4,526 | 4,535 | 5,801 | 2,810 | 2,991 | 14,862 | 7,336 | 7,526 |
| Kapilisanga | 4,869 | 2,421 | 2,448 | 3,157 | 1,498 | 1,659 | 8,026 | 3,919 | 4,107 |
| Nkhanga | 9,822 | 4,842 | 4,980 | 6,176 | 3,011 | 3,165 | 15,998 | 7,853 | 8,145 |
| Membe | 2,316 | 1,126 | 1,190 | 1,610 | 809 | 801 | 3,926 | 1,935 | 1,991 |
| Chaboli | 4,029 | 2,025 | 2,004 | 2,638 | 1,307 | 1,331 | 6,667 | 3,332 | 3,335 |
| **Lumezi Constituency** | **46,692** | **23,207** | **23,485** | **29,218** | **14,117** | **15,101** | **75,910** | **37,324** | **38,586** |
| Chamtowa | 6,262 | 3,128 | 3,134 | 4,206 | 2,057 | 2,149 | 10,468 | 5,185 | 5,283 |
| Kachama | 4,410 | 2,225 | 2,185 | 2,897 | 1,419 | 1,478 | 7,307 | 3,644 | 3,663 |
| Wachitangachi | 3,921 | 1,908 | 2,013 | 2,432 | 1,191 | 1,241 | 6,353 | 3,099 | 3,254 |
| Kamimba | 5,522 | 2,708 | 2,814 | 3,519 | 1,666 | 1,853 | 9,041 | 4,374 | 4,667 |
| Kazembe | 4,465 | 2,202 | 2,263 | 2,728 | 1,264 | 1,464 | 7,193 | 3,466 | 3,727 |
| Lumimba | 4,449 | 2,217 | 2,232 | 2,669 | 1,252 | 1,417 | 7,118 | 3,469 | 3,649 |
| Lukusuzi | 1,082 | 548 | 534 | 661 | 319 | 342 | 1,743 | 867 | 876 |
| Diwa | 11,762 | 5,861 | 5,901 | 7,239 | 3,571 | 3,668 | 19,001 | 9,432 | 9,569 |
| Chibande | 4,819 | 2,410 | 2,409 | 2,867 | 1,378 | 1,489 | 7,686 | 3,788 | 3,898 |
| **Lundazi Constituency** | **60,670** | **30,192** | **30,478** | **43,094** | **20,577** | **22,517** | **103,764** | **50,769** | **52,995** |
| Vuu | 9,193 | 4,624 | 4,569 | 6,443 | 3,140 | 3,303 | 15,636 | 7,764 | 7,872 |
| Mnyamazi | 15,244 | 7,522 | 7,722 | 12,515 | 5,928 | 6,587 | 27,759 | 13,450 | 14,309 |
| Ndonda | 4,449 | 2,212 | 2,237 | 3,160 | 1,493 | 1,667 | 7,609 | 3,705 | 3,904 |
| Msuzi | 7,881 | 3,871 | 4,010 | 5,229 | 2,524 | 2,705 | 13,110 | 6,395 | 6,715 |
| Mkomba | 2,512 | 1,260 | 1,252 | 1,594 | 775 | 819 | 4,106 | 2,035 | 2,071 |
| Chilola | 4,942 | 2,487 | 2,455 | 3,216 | 1,524 | 1,692 | 8,158 | 4,011 | 4,147 |
| Chimaliro | 4,706 | 2,372 | 2,334 | 3,256 | 1,561 | 1,695 | 7,962 | 3,933 | 4,029 |
| Nthintimila | 4,001 | 2,009 | 1,992 | 2,693 | 1,294 | 1,399 | 6,694 | 3,303 | 3,391 |
| Lunevwa | 7,742 | 3,835 | 3,907 | 4,988 | 2,338 | 2,650 | 12,730 | 6,173 | 6,557 |
| **Mambwe District** | **32,511** | **16,252** | **16,259** | **23,051** | **11,324** | **11,727** | **55,562** | **27,576** | **27,986** |
| **Mambwe Constituency** | **32,511** | **16,252** | **16,259** | **23,051** | **11,324** | **11,727** | **55,562** | **27,576** | **27,986** |
| Nsefu | 3,824 | 1,913 | 1,911 | 2,678 | 1,336 | 1,342 | 6,502 | 3,249 | 3,253 |
| Jumbe | 2,413 | 1,209 | 1,204 | 1,615 | 784 | 831 | 4,028 | 1,993 | 2,035 |
| Chipapa | 2,187 | 1,127 | 1,060 | 1,772 | 867 | 905 | 3,959 | 1,994 | 1,965 |
| Mphomwa | 4,128 | 2,060 | 2,068 | 2,636 | 1,340 | 1,296 | 6,764 | 3,400 | 3,364 |
| Chikowa | 2,483 | 1,238 | 1,245 | 1,526 | 722 | 804 | 4,009 | 1,960 | 2,049 |
| Mnkhanya | 3,328 | 1,650 | 1,678 | 2,384 | 1,166 | 1,218 | 5,712 | 2,816 | 2,896 |
| Kakumbi | 6,737 | 3,347 | 3,390 | 5,247 | 2,545 | 2,702 | 11,984 | 5,892 | 6,092 |
| Ncheka | 284 | 160 | 124 | 155 | 75 | 80 | 439 | 235 | 204 |
| Malama | 192 | 94 | 98 | 145 | 72 | 73 | 337 | 166 | 171 |
| Mdima | 4,663 | 2,297 | 2,366 | 3,213 | 1,576 | 1,637 | 7,876 | 3,873 | 4,003 |
| Msoro | 403 | 210 | 193 | 292 | 149 | 143 | 695 | 359 | 336 |
| Kasamanda | 1,308 | 659 | 649 | 973 | 488 | 485 | 2,281 | 1,147 | 1,134 |
| Nyakatokoli | 561 | 288 | 273 | 415 | 204 | 211 | 976 | 492 | 484 |
| **Nyimba District** | **40,366** | **20,454** | **19,912** | **27,608** | **13,458** | **14,150** | **67,974** | **33,912** | **34,062** |
| **Nyimba Constituency** | **40,366** | **20,454** | **19,912** | **27,608** | **13,458** | **14,150** | **67,974** | **33,912** | **34,062** |
| Chinsimbwe | 410 | 207 | 203 | 267 | 135 | 132 | 677 | 342 | 335 |
| Katipa | 454 | 244 | 210 | 307 | 154 | 153 | 761 | 398 | 363 |
| Vizimumba | 6,724 | 3,402 | 3,322 | 4,191 | 2,069 | 2,122 | 10,915 | 5,471 | 5,444 |
| Ngozi | 4,163 | 2,157 | 2,006 | 3,062 | 1,528 | 1,534 | 7,225 | 3,685 | 3,540 |
| Luezi | 2,391 | 1,221 | 1,170 | 1,730 | 853 | 877 | 4,121 | 2,074 | 2,047 |

## Table A11: Population (De Jure) by Age 0-14 Years (Children), 15-34 Years (Youths) and Total Population 0-34 Years by Sex, Province, District, Constituency and Ward, Zambia 2010

| Province,District,Constituency and Ward | 0 -14 Years | | | 15 - 34 Years | | | 0 - 34 Years | | |
|---|---|---|---|---|---|---|---|---|---|
| | Total | Male | Female | Total | Male | Female | Total | Male | Female |
| Mtilizi | 2,833 | 1,455 | 1,378 | 1,852 | 912 | 940 | 4,685 | 2,367 | 2,318 |
| Nyimba | 3,984 | 2,015 | 1,969 | 3,406 | 1,582 | 1,824 | 7,390 | 3,597 | 3,793 |
| Kaliwe | 4,161 | 2,124 | 2,037 | 2,731 | 1,341 | 1,390 | 6,892 | 3,465 | 3,427 |
| Chiweza | 5,218 | 2,612 | 2,606 | 3,638 | 1,838 | 1,800 | 8,856 | 4,450 | 4,406 |
| Mombe | 2,306 | 1,100 | 1,206 | 1,672 | 798 | 874 | 3,978 | 1,898 | 2,080 |
| Chamilala | 1,995 | 1,016 | 979 | 1,245 | 605 | 640 | 3,240 | 1,621 | 1,619 |
| Chinambi | 4,527 | 2,314 | 2,213 | 2,856 | 1,362 | 1,494 | 7,383 | 3,676 | 3,707 |
| Luangwa | 1,200 | 587 | 613 | 651 | 281 | 370 | 1,851 | 868 | 983 |
| **Petauke District** | **146,302** | **73,716** | **72,586** | **101,648** | **49,367** | **52,281** | **247,950** | **123,083** | **124,867** |
| **Kapoche Constituency** | **57,836** | **29,248** | **28,588** | **39,245** | **19,000** | **20,245** | **97,081** | **48,248** | **48,833** |
| Kapoche | 4,888 | 2,469 | 2,419 | 3,652 | 1,708 | 1,944 | 8,540 | 4,177 | 4,363 |
| Ching'ombe | 7,339 | 3,719 | 3,620 | 5,413 | 2,654 | 2,759 | 12,752 | 6,373 | 6,379 |
| Mwangaila | 8,888 | 4,495 | 4,393 | 6,342 | 3,098 | 3,244 | 15,230 | 7,593 | 7,637 |
| Matambazi | 10,640 | 5,429 | 5,211 | 6,795 | 3,300 | 3,495 | 17,435 | 8,729 | 8,706 |
| Kaumbwe | 8,652 | 4,335 | 4,317 | 5,366 | 2,550 | 2,816 | 14,018 | 6,885 | 7,133 |
| Lusinde | 5,448 | 2,758 | 2,690 | 3,618 | 1,784 | 1,834 | 9,066 | 4,542 | 4,524 |
| Manjazi | 4,537 | 2,274 | 2,263 | 2,884 | 1,397 | 1,487 | 7,421 | 3,671 | 3,750 |
| Manyane | 7,444 | 3,769 | 3,675 | 5,175 | 2,509 | 2,666 | 12,619 | 6,278 | 6,341 |
| **Msanzala Constituency** | **56,266** | **28,343** | **27,923** | **41,303** | **20,046** | **21,257** | **97,569** | **48,389** | **49,180** |
| Msumbazi | 8,067 | 4,062 | 4,005 | 5,438 | 2,675 | 2,763 | 13,505 | 6,737 | 6,768 |
| Ongolwe | 7,900 | 4,053 | 3,847 | 5,058 | 2,401 | 2,657 | 12,958 | 6,454 | 6,504 |
| Kovyane | 5,846 | 2,901 | 2,945 | 3,997 | 1,915 | 2,082 | 9,843 | 4,816 | 5,027 |
| Mbala | 5,287 | 2,660 | 2,627 | 3,862 | 1,900 | 1,962 | 9,149 | 4,560 | 4,589 |
| Nsimbo | 4,085 | 2,121 | 1,964 | 2,663 | 1,314 | 1,349 | 6,748 | 3,435 | 3,313 |
| Nyika | 16,385 | 8,125 | 8,260 | 14,485 | 7,029 | 7,456 | 30,870 | 15,154 | 15,716 |
| Chimanyama | 8,696 | 4,421 | 4,275 | 5,800 | 2,812 | 2,988 | 14,496 | 7,233 | 7,263 |
| **Petauke Central Constituency** | **32,200** | **16,125** | **16,075** | **21,100** | **10,321** | **10,779** | **53,300** | **26,446** | **26,854** |
| Nyakawise | 5,123 | 2,531 | 2,592 | 3,439 | 1,675 | 1,764 | 8,562 | 4,206 | 4,356 |
| Singozi | 6,316 | 3,147 | 3,169 | 4,026 | 1,958 | 2,068 | 10,342 | 5,105 | 5,237 |
| Mateyo Mzeka | 4,899 | 2,460 | 2,439 | 3,158 | 1,546 | 1,612 | 8,057 | 4,006 | 4,051 |
| Mawanda | 5,662 | 2,813 | 2,849 | 3,735 | 1,846 | 1,889 | 9,397 | 4,659 | 4,738 |
| Lusangazi | 1,076 | 552 | 524 | 721 | 335 | 386 | 1,797 | 887 | 910 |
| Ukwimi | 4,895 | 2,463 | 2,432 | 3,179 | 1,554 | 1,625 | 8,074 | 4,017 | 4,057 |
| Chisangu | 4,229 | 2,159 | 2,070 | 2,842 | 1,407 | 1,435 | 7,071 | 3,566 | 3,505 |
| **Luapula Province** | **474,856** | **237,719** | **237,137** | **320,149** | **151,398** | **168,751** | **795,005** | **389,117** | **405,888** |
| **Chienge District** | **53,879** | **26,785** | **27,094** | **37,832** | **18,048** | **19,784** | **91,711** | **44,833** | **46,878** |
| **Chienge Constituency** | **53,879** | **26,785** | **27,094** | **37,832** | **18,048** | **19,784** | **91,711** | **44,833** | **46,878** |
| Lunchinda | 3,324 | 1,693 | 1,631 | 2,674 | 1,272 | 1,402 | 5,998 | 2,965 | 3,033 |
| Chipungu | 7,124 | 3,532 | 3,592 | 5,078 | 2,352 | 2,726 | 12,202 | 5,884 | 6,318 |
| Luau | 3,001 | 1,461 | 1,540 | 2,173 | 1,052 | 1,121 | 5,174 | 2,513 | 2,661 |
| Lambwe Chomba | 1,803 | 965 | 838 | 1,249 | 562 | 687 | 3,052 | 1,527 | 1,525 |
| Chienge | 4,183 | 2,063 | 2,120 | 3,139 | 1,512 | 1,627 | 7,322 | 3,575 | 3,747 |
| Katete | 5,126 | 2,547 | 2,579 | 3,882 | 1,878 | 2,004 | 9,008 | 4,425 | 4,583 |
| Ifuna | 3,778 | 1,914 | 1,864 | 2,631 | 1,284 | 1,347 | 6,409 | 3,198 | 3,211 |
| Chipamba | 7,732 | 3,849 | 3,883 | 5,254 | 2,500 | 2,754 | 12,986 | 6,349 | 6,637 |
| Kalobwa | 2,336 | 1,163 | 1,173 | 1,524 | 733 | 791 | 3,860 | 1,896 | 1,964 |
| Chitutu | 3,079 | 1,534 | 1,545 | 2,117 | 1,011 | 1,106 | 5,196 | 2,545 | 2,651 |
| Munwa | 5,030 | 2,455 | 2,575 | 3,405 | 1,647 | 1,758 | 8,435 | 4,102 | 4,333 |
| Kulungwishi | 2,897 | 1,422 | 1,475 | 1,778 | 818 | 960 | 4,675 | 2,240 | 2,435 |
| Mununga | 4,466 | 2,187 | 2,279 | 2,928 | 1,427 | 1,501 | 7,394 | 3,614 | 3,780 |

## Table A11: Population (De Jure) by Age 0-14 Years (Children), 15-34 Years (Youths) and Total Population 0-34 Years by Sex, Province, District, Constituency and Ward, Zambia 2010

| Province,District,Constituency and Ward | 0 -14 Years | | | 15 - 34 Years | | | 0 - 34 Years | | |
|---|---|---|---|---|---|---|---|---|---|
| | Total | Male | Female | Total | Male | Female | Total | Male | Female |
| **Kawambwa District** | **65,183** | **32,617** | **32,566** | **42,921** | **20,292** | **22,629** | **108,104** | **52,909** | **55,195** |
| **Kawambwa Central Constituency** | **22,566** | **11,346** | **11,220** | **15,752** | **7,513** | **8,239** | **38,318** | **18,859** | **19,459** |
| Senga | 5,386 | 2,702 | 2,684 | 3,677 | 1,727 | 1,950 | 9,063 | 4,429 | 4,634 |
| Luena | 1,403 | 682 | 721 | 998 | 482 | 516 | 2,401 | 1,164 | 1,237 |
| Ntumbachushi | 1,056 | 533 | 523 | 661 | 309 | 352 | 1,717 | 842 | 875 |
| Kawambwa | 4,274 | 2,150 | 2,124 | 3,334 | 1,581 | 1,753 | 7,608 | 3,731 | 3,877 |
| Ng'ona | 5,595 | 2,781 | 2,814 | 4,174 | 2,033 | 2,141 | 9,769 | 4,814 | 4,955 |
| Fisaka | 2,596 | 1,338 | 1,258 | 1,581 | 740 | 841 | 4,177 | 2,078 | 2,099 |
| Iyanga | 2,256 | 1,160 | 1,096 | 1,327 | 641 | 686 | 3,583 | 1,801 | 1,782 |
| **Mwansabombwe Constituency** | **21,765** | **10,799** | **10,966** | **14,210** | **6,588** | **7,622** | **35,975** | **17,387** | **18,588** |
| Mununshi | 2,334 | 1,147 | 1,187 | 1,463 | 662 | 801 | 3,797 | 1,809 | 1,988 |
| Mulele | 5,322 | 2,559 | 2,763 | 3,440 | 1,640 | 1,800 | 8,762 | 4,199 | 4,563 |
| Lufubu | 1,604 | 805 | 799 | 1,049 | 492 | 557 | 2,653 | 1,297 | 1,356 |
| Mwansabombwe | 2,535 | 1,213 | 1,322 | 1,769 | 832 | 937 | 4,304 | 2,045 | 2,259 |
| Kakose | 2,485 | 1,222 | 1,263 | 1,700 | 780 | 920 | 4,185 | 2,002 | 2,183 |
| Chipita | 359 | 185 | 174 | 215 | 97 | 118 | 574 | 282 | 292 |
| Kayo | 4,109 | 2,106 | 2,003 | 2,522 | 1,156 | 1,366 | 6,631 | 3,262 | 3,369 |
| Mbereshi | 3,017 | 1,562 | 1,455 | 2,052 | 929 | 1,123 | 5,069 | 2,491 | 2,578 |
| **Pambashe Constituency** | **20,852** | **10,472** | **10,380** | **12,959** | **6,191** | **6,768** | **33,811** | **16,663** | **17,148** |
| Kabanse | 3,597 | 1,792 | 1,805 | 2,134 | 1,016 | 1,118 | 5,731 | 2,808 | 2,923 |
| Ilombe | 5,945 | 2,966 | 2,979 | 3,789 | 1,764 | 2,025 | 9,734 | 4,730 | 5,004 |
| Mulunda | 5,412 | 2,693 | 2,719 | 3,335 | 1,615 | 1,720 | 8,747 | 4,308 | 4,439 |
| Chibote | 1,383 | 703 | 680 | 895 | 437 | 458 | 2,278 | 1,140 | 1,138 |
| Chimpili | 1,314 | 643 | 671 | 800 | 387 | 413 | 2,114 | 1,030 | 1,084 |
| Luongo | 2,026 | 1,084 | 942 | 1,278 | 615 | 663 | 3,304 | 1,699 | 1,605 |
| Pambashe | 1,175 | 591 | 584 | 728 | 357 | 371 | 1,903 | 948 | 955 |
| **Mansa District** | **107,354** | **53,792** | **53,562** | **76,395** | **36,272** | **40,123** | **183,749** | **90,064** | **93,685** |
| **Bahati Constituency** | **44,038** | **22,381** | **21,657** | **29,889** | **14,251** | **15,638** | **73,927** | **36,632** | **37,295** |
| Mutuna | 6,974 | 3,572 | 3,402 | 4,334 | 2,020 | 2,314 | 11,308 | 5,592 | 5,716 |
| Misakalala | 5,014 | 2,596 | 2,418 | 3,290 | 1,633 | 1,657 | 8,304 | 4,229 | 4,075 |
| Kaole | 1,502 | 789 | 713 | 1,042 | 471 | 571 | 2,544 | 1,260 | 1,284 |
| Mushipashi | 4,106 | 2,065 | 2,041 | 2,558 | 1,197 | 1,361 | 6,664 | 3,262 | 3,402 |
| Mansa | 12,785 | 6,403 | 6,382 | 10,446 | 5,005 | 5,441 | 23,231 | 11,408 | 11,823 |
| Myulu | 9,748 | 4,941 | 4,807 | 6,044 | 2,914 | 3,130 | 15,792 | 7,855 | 7,937 |
| Muleshi | 3,909 | 2,015 | 1,894 | 2,175 | 1,011 | 1,164 | 6,084 | 3,026 | 3,058 |
| **Mansa Central Constituency** | **63,316** | **31,411** | **31,905** | **46,506** | **22,021** | **24,485** | **109,822** | **53,432** | **56,390** |
| Muchinka | 14,523 | 7,168 | 7,355 | 12,359 | 5,940 | 6,419 | 26,882 | 13,108 | 13,774 |
| Mulelenshi | 8,740 | 4,253 | 4,487 | 8,370 | 3,913 | 4,457 | 17,110 | 8,166 | 8,944 |
| Lukangaba | 8,846 | 4,434 | 4,412 | 5,465 | 2,646 | 2,819 | 14,311 | 7,080 | 7,231 |
| Chilyapa | 2,848 | 1,371 | 1,477 | 1,933 | 915 | 1,018 | 4,781 | 2,286 | 2,495 |
| Chansusu | 4,390 | 2,174 | 2,216 | 3,200 | 1,506 | 1,694 | 7,590 | 3,680 | 3,910 |
| Lwingishi | 8,204 | 4,127 | 4,077 | 5,186 | 2,456 | 2,730 | 13,390 | 6,583 | 6,807 |
| Chibeleka | 4,196 | 2,035 | 2,161 | 2,593 | 1,210 | 1,383 | 6,789 | 3,245 | 3,544 |
| Lukola | 3,677 | 1,882 | 1,795 | 2,288 | 1,064 | 1,224 | 5,965 | 2,946 | 3,019 |
| Luapula | 7,892 | 3,967 | 3,925 | 5,112 | 2,371 | 2,741 | 13,004 | 6,338 | 6,666 |
| **Milenge District** | **22,222** | **11,042** | **11,180** | **13,383** | **6,356** | **7,027** | **35,605** | **17,398** | **18,207** |
| **Chembe Constituency** | **22,222** | **11,042** | **11,180** | **13,383** | **6,356** | **7,027** | **35,605** | **17,398** | **18,207** |
| Chiswishi | 967 | 472 | 495 | 725 | 378 | 347 | 1,692 | 850 | 842 |
| Mulumbi | 1,184 | 564 | 620 | 718 | 342 | 376 | 1,902 | 906 | 996 |
| Itemba | 2,261 | 1,113 | 1,148 | 1,584 | 750 | 834 | 3,845 | 1,863 | 1,982 |
| Lusumbwe | 1,230 | 602 | 628 | 713 | 334 | 379 | 1,943 | 936 | 1,007 |
| Milambo | 2,033 | 998 | 1,035 | 1,140 | 501 | 639 | 3,173 | 1,499 | 1,674 |
| Nsaka | 155 | 76 | 79 | 88 | 39 | 49 | 243 | 115 | 128 |
| Fibalala | 2,900 | 1,466 | 1,434 | 1,664 | 783 | 881 | 4,564 | 2,249 | 2,315 |

## Table A11: Population (De Jure) by Age 0-14 Years (Children), 15-34 Years (Youths) and Total Population 0-34 Years by Sex, Province, District, Constituency and Ward, Zambia 2010

| Province,District,Constituency and Ward | 0 -14 Years | | | 15 - 34 Years | | | 0 - 34 Years | | |
|---|---|---|---|---|---|---|---|---|---|
| | Total | Male | Female | Total | Male | Female | Total | Male | Female |
| Nsunga | 1,187 | 591 | 596 | 618 | 282 | 336 | 1,805 | 873 | 932 |
| Chipundu | 415 | 193 | 222 | 248 | 119 | 129 | 663 | 312 | 351 |
| Sokontwe | 2,618 | 1,319 | 1,299 | 1,510 | 703 | 807 | 4,128 | 2,022 | 2,106 |
| Mumbotuta | 1,599 | 781 | 818 | 880 | 406 | 474 | 2,479 | 1,187 | 1,292 |
| Kapalala | 714 | 385 | 329 | 363 | 168 | 195 | 1,077 | 553 | 524 |
| Mikula | 4,959 | 2,482 | 2,477 | 3,132 | 1,551 | 1,581 | 8,091 | 4,033 | 4,058 |
| **Mwense District** | **56,998** | **28,647** | **28,351** | **37,930** | **17,759** | **20,171** | **94,928** | **46,406** | **48,522** |
| **Chipili Constituency** | **15,736** | **8,021** | **7,715** | **10,330** | **4,917** | **5,413** | **26,066** | **12,938** | **13,128** |
| Nsenga | 2,592 | 1,330 | 1,262 | 1,739 | 826 | 913 | 4,331 | 2,156 | 2,175 |
| Mweshi | 1,736 | 863 | 873 | 1,164 | 558 | 606 | 2,900 | 1,421 | 1,479 |
| Mumbwe | 2,192 | 1,098 | 1,094 | 1,437 | 684 | 753 | 3,629 | 1,782 | 1,847 |
| Chibalashi | 2,717 | 1,393 | 1,324 | 1,601 | 762 | 839 | 4,318 | 2,155 | 2,163 |
| Nalupembe | 3,691 | 1,872 | 1,819 | 2,625 | 1,262 | 1,363 | 6,316 | 3,134 | 3,182 |
| Nkonge | 2,808 | 1,465 | 1,343 | 1,764 | 825 | 939 | 4,572 | 2,290 | 2,282 |
| **Mambilima Constituency** | **12,386** | **6,135** | **6,251** | **7,867** | **3,671** | **4,196** | **20,253** | **9,806** | **10,447** |
| Mpasa | 2,451 | 1,220 | 1,231 | 1,368 | 662 | 706 | 3,819 | 1,882 | 1,937 |
| Chibembe | 1,112 | 576 | 536 | 665 | 317 | 348 | 1,777 | 893 | 884 |
| Musonda | 1,849 | 920 | 929 | 1,289 | 588 | 701 | 3,138 | 1,508 | 1,630 |
| Lundashi | 2,970 | 1,439 | 1,531 | 2,031 | 944 | 1,087 | 5,001 | 2,383 | 2,618 |
| Mambilima | 803 | 409 | 394 | 556 | 267 | 289 | 1,359 | 676 | 683 |
| Munwa | 587 | 278 | 309 | 373 | 172 | 201 | 960 | 450 | 510 |
| Nsomfi | 2,614 | 1,293 | 1,321 | 1,585 | 721 | 864 | 4,199 | 2,014 | 2,185 |
| **Mwense Constituency** | **28,876** | **14,491** | **14,385** | **19,733** | **9,171** | **10,562** | **48,609** | **23,662** | **24,947** |
| Kalanga | 2,934 | 1,445 | 1,489 | 1,979 | 919 | 1,060 | 4,913 | 2,364 | 2,549 |
| Kasengu | 3,233 | 1,595 | 1,638 | 2,718 | 1,270 | 1,448 | 5,951 | 2,865 | 3,086 |
| Katiti | 3,075 | 1,529 | 1,546 | 2,020 | 961 | 1,059 | 5,095 | 2,490 | 2,605 |
| Chachacha | 2,236 | 1,146 | 1,090 | 1,388 | 655 | 733 | 3,624 | 1,801 | 1,823 |
| Kapela | 1,767 | 905 | 862 | 1,104 | 493 | 611 | 2,871 | 1,398 | 1,473 |
| Pabe Kabesa | 4,231 | 2,169 | 2,062 | 2,899 | 1,350 | 1,549 | 7,130 | 3,519 | 3,611 |
| Luche | 5,802 | 2,892 | 2,910 | 4,085 | 1,924 | 2,161 | 9,887 | 4,816 | 5,071 |
| Nkanga | 3,061 | 1,558 | 1,503 | 1,895 | 836 | 1,059 | 4,956 | 2,394 | 2,562 |
| Kaombe | 2,537 | 1,252 | 1,285 | 1,645 | 763 | 882 | 4,182 | 2,015 | 2,167 |
| **Nchelenge District** | **72,705** | **36,313** | **36,392** | **50,425** | **24,221** | **26,204** | **123,130** | **60,534** | **62,596** |
| **Nchelenge Constituency** | **72,705** | **36,313** | **36,392** | **50,425** | **24,221** | **26,204** | **123,130** | **60,534** | **62,596** |
| Kabuta | 4,050 | 2,095 | 1,955 | 2,795 | 1,356 | 1,439 | 6,845 | 3,451 | 3,394 |
| Mwatishi | 8,078 | 3,987 | 4,091 | 5,267 | 2,525 | 2,742 | 13,345 | 6,512 | 6,833 |
| Munkombwe | 785 | 384 | 401 | 552 | 268 | 284 | 1,337 | 652 | 685 |
| Kilwa | 4,171 | 2,092 | 2,079 | 2,720 | 1,353 | 1,367 | 6,891 | 3,445 | 3,446 |
| Kashikishi | 11,786 | 5,804 | 5,982 | 8,591 | 4,210 | 4,381 | 20,377 | 10,014 | 10,363 |
| Nchelenge | 479 | 231 | 248 | 566 | 272 | 294 | 1,045 | 503 | 542 |
| Chilongo | 17,450 | 8,700 | 8,750 | 13,020 | 6,206 | 6,814 | 30,470 | 14,906 | 15,564 |
| Chisenga | 4,882 | 2,491 | 2,391 | 3,280 | 1,553 | 1,727 | 8,162 | 4,044 | 4,118 |
| Kasamba | 6,248 | 3,140 | 3,108 | 4,122 | 1,920 | 2,202 | 10,370 | 5,060 | 5,310 |
| Mulwe | 6,245 | 3,118 | 3,127 | 4,058 | 1,971 | 2,087 | 10,303 | 5,089 | 5,214 |
| Shabo | 1,935 | 967 | 968 | 1,281 | 604 | 677 | 3,216 | 1,571 | 1,645 |
| Mofwe | 3,194 | 1,603 | 1,591 | 2,081 | 990 | 1,091 | 5,275 | 2,593 | 2,682 |
| Katofyo | 3,402 | 1,701 | 1,701 | 2,092 | 993 | 1,099 | 5,494 | 2,694 | 2,800 |
| **Samfya District** | **96,515** | **48,523** | **47,992** | **61,263** | **28,450** | **32,813** | **157,778** | **76,973** | **80,805** |
| **Bangweulu Constituency** | **43,931** | **22,045** | **21,886** | **28,871** | **13,379** | **15,492** | **72,802** | **35,424** | **37,378** |
| Chimana | 8,836 | 4,418 | 4,418 | 7,112 | 3,384 | 3,728 | 15,948 | 7,802 | 8,146 |
| Mano | 8,652 | 4,368 | 4,284 | 5,415 | 2,556 | 2,859 | 14,067 | 6,924 | 7,143 |
| Katanshya | 5,020 | 2,549 | 2,471 | 2,916 | 1,257 | 1,659 | 7,936 | 3,806 | 4,130 |
| Isamba | 4,326 | 2,152 | 2,174 | 2,571 | 1,219 | 1,352 | 6,897 | 3,371 | 3,526 |
| Kapata | 7,747 | 3,883 | 3,864 | 5,122 | 2,313 | 2,809 | 12,869 | 6,196 | 6,673 |

## Table A11: Population (De Jure) by Age 0-14 Years (Children), 15-34 Years (Youths) and Total Population 0-34 Years by Sex, Province, District, Constituency and Ward, Zambia 2010

| Province,District,Constituency and Ward | 0 -14 Years | | | 15 - 34 Years | | | 0 - 34 Years | | |
|---|---|---|---|---|---|---|---|---|---|
| | Total | Male | Female | Total | Male | Female | Total | Male | Female |
| Musaba | 4,667 | 2,356 | 2,311 | 2,902 | 1,340 | 1,562 | 7,569 | 3,696 | 3,873 |
| Kapilibila | 1,822 | 893 | 929 | 1,116 | 524 | 592 | 2,938 | 1,417 | 1,521 |
| Lumamya | 2,861 | 1,426 | 1,435 | 1,717 | 786 | 931 | 4,578 | 2,212 | 2,366 |
| **Chifunabuli Constituency** | **41,161** | **20,797** | **20,364** | **24,754** | **11,529** | **13,225** | **65,915** | **32,326** | **33,589** |
| Masonde | 4,518 | 2,277 | 2,241 | 2,725 | 1,254 | 1,471 | 7,243 | 3,531 | 3,712 |
| Kasansa | 3,517 | 1,781 | 1,736 | 2,034 | 918 | 1,116 | 5,551 | 2,699 | 2,852 |
| Kasaba | 5,537 | 2,775 | 2,762 | 3,488 | 1,657 | 1,831 | 9,025 | 4,432 | 4,593 |
| Kapamba | 2,560 | 1,305 | 1,255 | 1,480 | 662 | 818 | 4,040 | 1,967 | 2,073 |
| Kasongele | 3,200 | 1,610 | 1,590 | 1,961 | 932 | 1,029 | 5,161 | 2,542 | 2,619 |
| Kafumbo | 3,822 | 1,909 | 1,913 | 2,254 | 1,028 | 1,226 | 6,076 | 2,937 | 3,139 |
| Chinkutila | 4,867 | 2,447 | 2,420 | 3,002 | 1,421 | 1,581 | 7,869 | 3,868 | 4,001 |
| Chishi | 2,803 | 1,415 | 1,388 | 1,432 | 666 | 766 | 4,235 | 2,081 | 2,154 |
| Chifunabuli | 7,736 | 3,934 | 3,802 | 4,875 | 2,310 | 2,565 | 12,611 | 6,244 | 6,367 |
| Mbambala | 2,601 | 1,344 | 1,257 | 1,503 | 681 | 822 | 4,104 | 2,025 | 2,079 |
| **Luapula Constituency** | **11,423** | **5,681** | **5,742** | **7,638** | **3,542** | **4,096** | **19,061** | **9,223** | **9,838** |
| Nkutila | 4,529 | 2,270 | 2,259 | 3,003 | 1,369 | 1,634 | 7,532 | 3,639 | 3,893 |
| Lunga | 1,608 | 805 | 803 | 1,226 | 537 | 689 | 2,834 | 1,342 | 1,492 |
| Ncheta | 2,539 | 1,271 | 1,268 | 1,546 | 792 | 754 | 4,085 | 2,063 | 2,022 |
| Nsalushi | 2,747 | 1,335 | 1,412 | 1,863 | 844 | 1,019 | 4,610 | 2,179 | 2,431 |
| **Lusaka Province** | **885,562** | **432,621** | **452,941** | **890,134** | **425,866** | **464,268** | **1,775,696** | **858,487** | **917,209** |
| **Chongwe District** | **87,081** | **43,332** | **43,749** | **67,001** | **33,349** | **33,652** | **154,082** | **76,681** | **77,401** |
| **Chongwe Constituency** | **63,078** | **31,140** | **31,938** | **51,085** | **25,289** | **25,796** | **114,163** | **56,429** | **57,734** |
| Kapwayambale | 7,905 | 3,849 | 4,056 | 6,512 | 3,139 | 3,373 | 14,417 | 6,988 | 7,429 |
| Chinkuli | 9,730 | 4,766 | 4,964 | 8,435 | 4,240 | 4,195 | 18,165 | 9,006 | 9,159 |
| Ntandabale | 8,637 | 4,259 | 4,378 | 7,503 | 3,720 | 3,783 | 16,140 | 7,979 | 8,161 |
| Chongwe | 11,314 | 5,607 | 5,707 | 9,938 | 4,853 | 5,085 | 21,252 | 10,460 | 10,792 |
| Kanakantapa | 6,415 | 3,232 | 3,183 | 4,532 | 2,338 | 2,194 | 10,947 | 5,570 | 5,377 |
| Chalimbana | 4,644 | 2,264 | 2,380 | 3,664 | 1,806 | 1,858 | 8,308 | 4,070 | 4,238 |
| Nakatindi | 2,935 | 1,420 | 1,515 | 2,505 | 1,143 | 1,362 | 5,440 | 2,563 | 2,877 |
| Lukoshi | 4,018 | 2,001 | 2,017 | 2,879 | 1,478 | 1,401 | 6,897 | 3,479 | 3,418 |
| Manyika | 5,049 | 2,508 | 2,541 | 3,414 | 1,707 | 1,707 | 8,463 | 4,215 | 4,248 |
| Lwimba | 2,431 | 1,234 | 1,197 | 1,703 | 865 | 838 | 4,134 | 2,099 | 2,035 |
| **Rufunsa Constituency** | **24,003** | **12,192** | **11,811** | **15,916** | **8,060** | **7,856** | **39,919** | **20,252** | **19,667** |
| Mwachilele | 1,957 | 951 | 1,006 | 1,391 | 740 | 651 | 3,348 | 1,691 | 1,657 |
| Nyangwena | 3,575 | 1,828 | 1,747 | 2,392 | 1,202 | 1,190 | 5,967 | 3,030 | 2,937 |
| Bunda Bunda | 6,570 | 3,375 | 3,195 | 4,529 | 2,321 | 2,208 | 11,099 | 5,696 | 5,403 |
| Nyamanongo | 1,520 | 791 | 729 | 921 | 460 | 461 | 2,441 | 1,251 | 1,190 |
| Rufunsa | 7,427 | 3,754 | 3,673 | 4,841 | 2,472 | 2,369 | 12,268 | 6,226 | 6,042 |
| Mankanda | 1,920 | 958 | 962 | 1,317 | 617 | 700 | 3,237 | 1,575 | 1,662 |
| Shikabeta | 1,034 | 535 | 499 | 525 | 248 | 277 | 1,559 | 783 | 776 |
| **Kafue District** | **96,970** | **47,851** | **49,119** | **85,010** | **41,771** | **43,239** | **181,980** | **89,622** | **92,358** |
| **Kafue Constituency** | **50,859** | **25,276** | **25,583** | **44,681** | **21,866** | **22,815** | **95,540** | **47,142** | **48,398** |
| Chiyaba | 2,555 | 1,287 | 1,268 | 2,177 | 1,185 | 992 | 4,732 | 2,472 | 2,260 |
| Kambale | 2,413 | 1,230 | 1,183 | 1,584 | 788 | 796 | 3,997 | 2,018 | 1,979 |
| Malundu | 4,532 | 2,291 | 2,241 | 3,138 | 1,598 | 1,540 | 7,670 | 3,889 | 3,781 |
| Chisankane | 3,878 | 1,921 | 1,957 | 3,002 | 1,502 | 1,500 | 6,880 | 3,423 | 3,457 |
| Lukolongo | 1,430 | 691 | 739 | 1,050 | 528 | 522 | 2,480 | 1,219 | 1,261 |
| Kafue | 3,310 | 1,617 | 1,693 | 3,158 | 1,589 | 1,569 | 6,468 | 3,206 | 3,262 |
| Matanda | 4,107 | 2,041 | 2,066 | 4,381 | 2,027 | 2,354 | 8,488 | 4,068 | 4,420 |
| Shabusele | 13,260 | 6,518 | 6,742 | 11,437 | 5,425 | 6,012 | 24,697 | 11,943 | 12,754 |
| Kasenje | 7,806 | 3,836 | 3,970 | 9,159 | 4,367 | 4,792 | 16,965 | 8,203 | 8,762 |
| Mungu | 7,101 | 3,608 | 3,493 | 5,234 | 2,657 | 2,577 | 12,335 | 6,265 | 6,070 |
| Chikupi | 467 | 236 | 231 | 361 | 200 | 161 | 828 | 436 | 392 |

## Table A11: Population (De Jure) by Age 0-14 Years (Children), 15-34 Years (Youths) and Total Population 0-34 Years by Sex, Province, District, Constituency and Ward, Zambia 2010

| Province,District,Constituency and Ward | 0 -14 Years | | | 15 - 34 Years | | | 0 - 34 Years | | |
|---|---|---|---|---|---|---|---|---|---|
| | Total | Male | Female | Total | Male | Female | Total | Male | Female |
| **Chilanga Constituency** | **46,111** | **22,575** | **23,536** | **40,329** | **19,905** | **20,424** | **86,440** | **42,480** | **43,960** |
| Chilanga | 13,005 | 6,350 | 6,655 | 12,593 | 6,174 | 6,419 | 25,598 | 12,524 | 13,074 |
| Chilongolo | 8,476 | 4,156 | 4,320 | 7,199 | 3,574 | 3,625 | 15,675 | 7,730 | 7,945 |
| Namalombwe | 13,724 | 6,697 | 7,027 | 12,309 | 6,046 | 6,263 | 26,033 | 12,743 | 13,290 |
| Nyemba | 4,361 | 2,174 | 2,187 | 3,632 | 1,801 | 1,831 | 7,993 | 3,975 | 4,018 |
| Nakachenje | 1,040 | 520 | 520 | 655 | 341 | 314 | 1,695 | 861 | 834 |
| Chinyanja | 5,505 | 2,678 | 2,827 | 3,941 | 1,969 | 1,972 | 9,446 | 4,647 | 4,799 |
| **Luangwa District** | **11,371** | **5,681** | **5,690** | **8,144** | **3,971** | **4,173** | **19,515** | **9,652** | **9,863** |
| **Feira Constituency** | **11,371** | **5,681** | **5,690** | **8,144** | **3,971** | **4,173** | **19,515** | **9,652** | **9,863** |
| Dzalo | 2,278 | 1,117 | 1,161 | 1,869 | 889 | 980 | 4,147 | 2,006 | 2,141 |
| M'kaliva | 426 | 224 | 202 | 277 | 134 | 143 | 703 | 358 | 345 |
| Mandombe | 313 | 165 | 148 | 268 | 130 | 138 | 581 | 295 | 286 |
| Phwazi | 446 | 210 | 236 | 290 | 145 | 145 | 736 | 355 | 381 |
| Mphuka | 537 | 262 | 275 | 358 | 168 | 190 | 895 | 430 | 465 |
| Kabowo | 94 | 56 | 38 | 60 | 24 | 36 | 154 | 80 | 74 |
| Kapoche | 726 | 352 | 374 | 436 | 228 | 208 | 1,162 | 580 | 582 |
| Chiriwe | 140 | 73 | 67 | 95 | 49 | 46 | 235 | 122 | 113 |
| Lunya | 312 | 153 | 159 | 189 | 86 | 103 | 501 | 239 | 262 |
| Katondwe | 1,113 | 567 | 546 | 969 | 468 | 501 | 2,082 | 1,035 | 1,047 |
| Chikoma | 1,091 | 529 | 562 | 699 | 361 | 338 | 1,790 | 890 | 900 |
| Mburuma | 1,365 | 690 | 675 | 958 | 462 | 496 | 2,323 | 1,152 | 1,171 |
| Mwalilia | 858 | 443 | 415 | 583 | 282 | 301 | 1,441 | 725 | 716 |
| Kaunga | 1,331 | 666 | 665 | 881 | 442 | 439 | 2,212 | 1,108 | 1,104 |
| Mankhokwe | 341 | 174 | 167 | 212 | 103 | 109 | 553 | 277 | 276 |
| **Lusaka District** | **690,140** | **335,757** | **354,383** | **729,979** | **346,775** | **383,204** | **1,420,119** | **682,532** | **737,587** |
| **Chawama Constituency** | **76,527** | **37,260** | **39,267** | **77,433** | **37,826** | **39,607** | **153,960** | **75,086** | **78,874** |
| Nkoloma | 31,805 | 15,412 | 16,393 | 30,185 | 14,830 | 15,355 | 61,990 | 30,242 | 31,748 |
| Chawama | 27,885 | 13,673 | 14,212 | 29,418 | 14,310 | 15,108 | 57,303 | 27,983 | 29,320 |
| John Howard | 11,483 | 5,587 | 5,896 | 12,305 | 6,017 | 6,288 | 23,788 | 11,604 | 12,184 |
| Lilayi | 5,354 | 2,588 | 2,766 | 5,525 | 2,669 | 2,856 | 10,879 | 5,257 | 5,622 |
| **Kabwata Constituency** | **60,079** | **28,955** | **31,124** | **80,194** | **36,540** | **43,654** | **140,273** | **65,495** | **74,778** |
| Kamwala | 19,260 | 9,316 | 9,944 | 22,426 | 10,448 | 11,978 | 41,686 | 19,764 | 21,922 |
| Kabwata | 6,345 | 3,011 | 3,334 | 11,492 | 5,193 | 6,299 | 17,837 | 8,204 | 9,633 |
| Libala | 7,108 | 3,388 | 3,720 | 10,628 | 4,705 | 5,923 | 17,736 | 8,093 | 9,643 |
| Chilenje | 16,569 | 7,951 | 8,618 | 24,923 | 11,094 | 13,829 | 41,492 | 19,045 | 22,447 |
| Kamulanga | 10,797 | 5,289 | 5,508 | 10,725 | 5,100 | 5,625 | 21,522 | 10,389 | 11,133 |
| **Kanyama Constituency** | **156,094** | **75,914** | **80,180** | **147,417** | **70,518** | **76,899** | **303,511** | **146,432** | **157,079** |
| Kanyama | 74,067 | 35,920 | 38,147 | 67,879 | 32,600 | 35,279 | 141,946 | 68,520 | 73,426 |
| Harry Mwaanga Nkumbula | 71,265 | 34,716 | 36,549 | 68,248 | 32,534 | 35,714 | 139,513 | 67,250 | 72,263 |
| Munkolo | 10,762 | 5,278 | 5,484 | 11,290 | 5,384 | 5,906 | 22,052 | 10,662 | 11,390 |
| **Lusaka Central Constituency** | **40,912** | **19,663** | **21,249** | **50,574** | **22,955** | **27,619** | **91,486** | **42,618** | **48,868** |
| Silwizya | 1,749 | 852 | 897 | 3,210 | 1,431 | 1,779 | 4,959 | 2,283 | 2,676 |
| Independence | 4,881 | 2,312 | 2,569 | 7,413 | 3,292 | 4,121 | 12,294 | 5,604 | 6,690 |
| Lubwa | 11,590 | 5,482 | 6,108 | 16,436 | 7,211 | 9,225 | 28,026 | 12,693 | 15,333 |
| Kabulonga | 22,692 | 11,017 | 11,675 | 23,515 | 11,021 | 12,494 | 46,207 | 22,038 | 24,169 |
| **Mandevu Constituency** | **148,302** | **72,490** | **75,812** | **143,527** | **69,255** | **74,272** | **291,829** | **141,745** | **150,084** |
| Roma | 27,630 | 13,467 | 14,163 | 26,166 | 12,532 | 13,634 | 53,796 | 25,999 | 27,797 |
| Mulungushi | 3,866 | 1,832 | 2,034 | 5,970 | 2,767 | 3,203 | 9,836 | 4,599 | 5,237 |
| Ngwerere | 26,067 | 12,724 | 13,343 | 28,053 | 13,606 | 14,447 | 54,120 | 26,330 | 27,790 |
| Chaisa | 8,058 | 3,884 | 4,174 | 8,112 | 4,069 | 4,043 | 16,170 | 7,953 | 8,217 |
| Justine Kabwe | 16,475 | 8,058 | 8,417 | 16,418 | 7,802 | 8,616 | 32,893 | 15,860 | 17,033 |
| Raphael Chota | 40,664 | 20,156 | 20,508 | 37,362 | 18,159 | 19,203 | 78,026 | 38,315 | 39,711 |
| Mpulungu | 25,542 | 12,369 | 13,173 | 21,446 | 10,320 | 11,126 | 46,988 | 22,689 | 24,299 |

## Table A11: Population (De Jure) by Age 0-14 Years (Children), 15-34 Years (Youths) and Total Population 0-34 Years by Sex, Province, District, Constituency and Ward, Zambia 2010

| Province,District,Constituency and Ward | 0 -14 Years | | | 15 - 34 Years | | | 0 - 34 Years | | |
|---|---|---|---|---|---|---|---|---|---|
| | Total | Male | Female | Total | Male | Female | Total | Male | Female |
| **Matero Constituency** | **111,071** | **54,271** | **56,800** | **117,317** | **56,668** | **60,649** | **228,388** | **110,939** | **117,449** |
| Muchinga | 14,637 | 7,070 | 7,567 | 18,163 | 8,675 | 9,488 | 32,800 | 15,745 | 17,055 |
| Kapwepwe | 21,833 | 10,500 | 11,333 | 22,442 | 10,889 | 11,553 | 44,275 | 21,389 | 22,886 |
| Lima | 26,236 | 12,900 | 13,336 | 24,549 | 12,167 | 12,382 | 50,785 | 25,067 | 25,718 |
| Mwembeshi | 28,236 | 13,864 | 14,372 | 27,905 | 13,264 | 14,641 | 56,141 | 27,128 | 29,013 |
| Matero | 20,129 | 9,937 | 10,192 | 24,258 | 11,673 | 12,585 | 44,387 | 21,610 | 22,777 |
| **Munali Constituency** | **97,155** | **47,204** | **49,951** | **113,517** | **53,013** | **60,504** | **210,672** | **100,217** | **110,455** |
| Chainda | 13,630 | 6,646 | 6,984 | 16,830 | 7,694 | 9,136 | 30,460 | 14,340 | 16,120 |
| Mtendere | 42,130 | 20,547 | 21,583 | 44,241 | 20,985 | 23,256 | 86,371 | 41,532 | 44,839 |
| Kalingalinga | 14,195 | 6,793 | 7,402 | 17,289 | 8,160 | 9,129 | 31,484 | 14,953 | 16,531 |
| Chakunkula | 11,184 | 5,444 | 5,740 | 14,638 | 6,688 | 7,950 | 25,822 | 12,132 | 13,690 |
| Munali | 16,016 | 7,774 | 8,242 | 20,519 | 9,486 | 11,033 | 36,535 | 17,260 | 19,275 |
| **Muchinga Province** | **346,014** | **173,033** | **172,981** | **231,448** | **110,133** | **121,315** | **577,462** | **283,166** | **294,296** |
| **Chama District** | **52,745** | **26,517** | **26,228** | **33,106** | **15,819** | **17,287** | **85,851** | **42,336** | **43,515** |
| **Chama North Constituency** | **27,101** | **13,674** | **13,427** | **17,169** | **8,264** | **8,905** | **44,270** | **21,938** | **22,332** |
| Mazonde | 859 | 405 | 454 | 455 | 221 | 234 | 1,314 | 626 | 688 |
| Nkhankha | 2,865 | 1,448 | 1,417 | 1,666 | 802 | 864 | 4,531 | 2,250 | 2,281 |
| Luangwa | 2,092 | 1,059 | 1,033 | 1,194 | 546 | 648 | 3,286 | 1,605 | 1,681 |
| Chisunga | 2,093 | 1,073 | 1,020 | 1,101 | 511 | 590 | 3,194 | 1,584 | 1,610 |
| Ndunda | 998 | 514 | 484 | 590 | 271 | 319 | 1,588 | 785 | 803 |
| Mbazi | 1,450 | 714 | 736 | 995 | 494 | 501 | 2,445 | 1,208 | 1,237 |
| Manthepa | 1,148 | 585 | 563 | 646 | 310 | 336 | 1,794 | 895 | 899 |
| Mphalausenga | 3,230 | 1,675 | 1,555 | 1,991 | 972 | 1,019 | 5,221 | 2,647 | 2,574 |
| Kalinkhu | 1,183 | 582 | 601 | 650 | 298 | 352 | 1,833 | 880 | 953 |
| Kamphemba | 6,373 | 3,183 | 3,190 | 4,814 | 2,376 | 2,438 | 11,187 | 5,559 | 5,628 |
| Mwalala | 3,282 | 1,669 | 1,613 | 2,036 | 963 | 1,073 | 5,318 | 2,632 | 2,686 |
| Muchinga | 1,528 | 767 | 761 | 1,031 | 500 | 531 | 2,559 | 1,267 | 1,292 |
| **Chama South Constituency** | **25,644** | **12,843** | **12,801** | **15,937** | **7,555** | **8,382** | **41,581** | **20,398** | **21,183** |
| Chipala | 1,306 | 658 | 648 | 836 | 381 | 455 | 2,142 | 1,039 | 1,103 |
| Bazimu | 2,902 | 1,446 | 1,456 | 1,932 | 931 | 1,001 | 4,834 | 2,377 | 2,457 |
| Mabinga | 2,813 | 1,435 | 1,378 | 1,756 | 824 | 932 | 4,569 | 2,259 | 2,310 |
| Lupamazi | 1,232 | 627 | 605 | 734 | 364 | 370 | 1,966 | 991 | 975 |
| Lumezi | 2,653 | 1,291 | 1,362 | 1,664 | 764 | 900 | 4,317 | 2,055 | 2,262 |
| Chibungwe | 2,650 | 1,310 | 1,340 | 1,688 | 816 | 872 | 4,338 | 2,126 | 2,212 |
| Lunzi | 3,850 | 1,910 | 1,940 | 2,513 | 1,218 | 1,295 | 6,363 | 3,128 | 3,235 |
| Vilimukulu | 2,835 | 1,430 | 1,405 | 1,694 | 804 | 890 | 4,529 | 2,234 | 2,295 |
| Chilenje | 3,172 | 1,601 | 1,571 | 1,907 | 893 | 1,014 | 5,079 | 2,494 | 2,585 |
| Mapamba | 2,231 | 1,135 | 1,096 | 1,213 | 560 | 653 | 3,444 | 1,695 | 1,749 |
| **Chinsali District** | **71,760** | **35,937** | **35,823** | **46,115** | **22,116** | **23,999** | **117,875** | **58,053** | **59,822** |
| **Chinsali Constituency** | **42,132** | **21,082** | **21,050** | **27,688** | **13,267** | **14,421** | **69,820** | **34,349** | **35,471** |
| Itapa | 4,998 | 2,475 | 2,523 | 3,018 | 1,441 | 1,577 | 8,016 | 3,916 | 4,100 |
| Chilunda | 647 | 320 | 327 | 393 | 189 | 204 | 1,040 | 509 | 531 |
| Chilinda | 4,450 | 2,259 | 2,191 | 2,742 | 1,358 | 1,384 | 7,192 | 3,617 | 3,575 |
| Kaunga | 4,577 | 2,318 | 2,259 | 2,665 | 1,250 | 1,415 | 7,242 | 3,568 | 3,674 |
| Malalo | 2,135 | 1,047 | 1,088 | 1,392 | 687 | 705 | 3,527 | 1,734 | 1,793 |
| Chipanga | 5,175 | 2,561 | 2,614 | 3,262 | 1,551 | 1,711 | 8,437 | 4,112 | 4,325 |
| Chambeshi | 846 | 419 | 427 | 557 | 271 | 286 | 1,403 | 690 | 713 |
| Lubwa | 5,016 | 2,593 | 2,423 | 3,281 | 1,581 | 1,700 | 8,297 | 4,174 | 4,123 |
| Ichinga | 7,518 | 3,703 | 3,815 | 5,494 | 2,593 | 2,901 | 13,012 | 6,296 | 6,716 |
| Nkakula | 1,990 | 985 | 1,005 | 1,885 | 911 | 974 | 3,875 | 1,896 | 1,979 |
| Mwanwakubili | 4,780 | 2,402 | 2,378 | 2,999 | 1,435 | 1,564 | 7,779 | 3,837 | 3,942 |

## Table A11: Population (De Jure) by Age 0-14 Years (Children), 15-34 Years (Youths) and Total Population 0-34 Years by Sex, Province, District, Constituency and Ward, Zambia 2010

| Province,District,Constituency and Ward | 0 -14 Years | | | 15 - 34 Years | | | 0 - 34 Years | | |
|---|---|---|---|---|---|---|---|---|---|
| | Total | Male | Female | Total | Male | Female | Total | Male | Female |
| **Shiwang'andu Constituency** | **29,628** | **14,855** | **14,773** | **18,427** | **8,849** | **9,578** | **48,055** | **23,704** | **24,351** |
| Chamusenga | 3,983 | 2,002 | 1,981 | 2,451 | 1,172 | 1,279 | 6,434 | 3,174 | 3,260 |
| Muchinga | 3,039 | 1,507 | 1,532 | 1,991 | 964 | 1,027 | 5,030 | 2,471 | 2,559 |
| Chandaula | 2,094 | 1,027 | 1,067 | 1,221 | 568 | 653 | 3,315 | 1,595 | 1,720 |
| Mukumbi | 3,263 | 1,659 | 1,604 | 2,229 | 1,095 | 1,134 | 5,492 | 2,754 | 2,738 |
| Mwila Kabuswe | 3,616 | 1,813 | 1,803 | 2,099 | 979 | 1,120 | 5,715 | 2,792 | 2,923 |
| Chibinda | 576 | 293 | 283 | 349 | 161 | 188 | 925 | 454 | 471 |
| Mayembe | 1,454 | 733 | 721 | 912 | 428 | 484 | 2,366 | 1,161 | 1,205 |
| Mwiche | 1,764 | 909 | 855 | 1,094 | 535 | 559 | 2,858 | 1,444 | 1,414 |
| Ichingo | 2,293 | 1,172 | 1,121 | 1,348 | 648 | 700 | 3,641 | 1,820 | 1,821 |
| Chimpunda | 4,171 | 2,079 | 2,092 | 2,759 | 1,352 | 1,407 | 6,930 | 3,431 | 3,499 |
| Nkulungwe | 3,375 | 1,661 | 1,714 | 1,974 | 947 | 1,027 | 5,349 | 2,608 | 2,741 |
| **Isoka District** | **33,868** | **16,917** | **16,951** | **23,706** | **11,319** | **12,387** | **57,574** | **28,236** | **29,338** |
| **Isoka Constituency** | **33,868** | **16,917** | **16,951** | **23,706** | **11,319** | **12,387** | **57,574** | **28,236** | **29,338** |
| Kasoka | 7,524 | 3,756 | 3,768 | 6,362 | 2,961 | 3,401 | 13,886 | 6,717 | 7,169 |
| Kantenshya | 3,506 | 1,724 | 1,782 | 2,387 | 1,165 | 1,222 | 5,893 | 2,889 | 3,004 |
| Sasamwenje | 6,269 | 3,149 | 3,120 | 3,929 | 1,881 | 2,048 | 10,198 | 5,030 | 5,168 |
| Kapililonga | 3,792 | 1,911 | 1,881 | 2,961 | 1,436 | 1,525 | 6,753 | 3,347 | 3,406 |
| Itukuta | 2,631 | 1,269 | 1,362 | 1,822 | 926 | 896 | 4,453 | 2,195 | 2,258 |
| Milongo | 976 | 483 | 493 | 677 | 324 | 353 | 1,653 | 807 | 846 |
| Nkombwa | 3,293 | 1,646 | 1,647 | 1,948 | 946 | 1,002 | 5,241 | 2,592 | 2,649 |
| Luangwa | 1,932 | 996 | 936 | 1,073 | 489 | 584 | 3,005 | 1,485 | 1,520 |
| Mpungu | 3,945 | 1,983 | 1,962 | 2,547 | 1,191 | 1,356 | 6,492 | 3,174 | 3,318 |
| **Mafinga District** | **32,877** | **16,488** | **16,389** | **20,938** | **9,856** | **11,082** | **53,815** | **26,344** | **27,471** |
| **Mafinga Constituency** | **32,877** | **16,488** | **16,389** | **20,938** | **9,856** | **11,082** | **53,815** | **26,344** | **27,471** |
| Mafinga | 3,068 | 1,520 | 1,548 | 1,779 | 813 | 966 | 4,847 | 2,333 | 2,514 |
| Ntonga | 5,740 | 2,944 | 2,796 | 3,613 | 1,717 | 1,896 | 9,353 | 4,661 | 4,692 |
| Mukutu | 3,489 | 1,788 | 1,701 | 2,278 | 1,056 | 1,222 | 5,767 | 2,844 | 2,923 |
| Thendere | 5,093 | 2,502 | 2,591 | 3,238 | 1,537 | 1,701 | 8,331 | 4,039 | 4,292 |
| Bemba | 5,171 | 2,541 | 2,630 | 3,120 | 1,415 | 1,705 | 8,291 | 3,956 | 4,335 |
| Kakoma | 2,094 | 1,078 | 1,016 | 1,322 | 606 | 716 | 3,416 | 1,684 | 1,732 |
| Luhoka | 2,064 | 1,006 | 1,058 | 1,383 | 675 | 708 | 3,447 | 1,681 | 1,766 |
| Kalanga | 6,158 | 3,109 | 3,049 | 4,205 | 2,037 | 2,168 | 10,363 | 5,146 | 5,217 |
| **Mpika District** | **97,726** | **48,872** | **48,854** | **66,441** | **31,438** | **35,003** | **164,167** | **80,310** | **83,857** |
| **Kanchibiya Constituency** | **41,270** | **20,705** | **20,565** | **25,077** | **11,770** | **13,307** | **66,347** | **32,475** | **33,872** |
| Chambeshi | 3,142 | 1,599 | 1,543 | 1,875 | 868 | 1,007 | 5,017 | 2,467 | 2,550 |
| Mansha | 3,145 | 1,626 | 1,519 | 1,964 | 914 | 1,050 | 5,109 | 2,540 | 2,569 |
| Mumbubu | 4,180 | 2,051 | 2,129 | 2,521 | 1,215 | 1,306 | 6,701 | 3,266 | 3,435 |
| Lubaleshi | 6,214 | 3,051 | 3,163 | 3,807 | 1,801 | 2,006 | 10,021 | 4,852 | 5,169 |
| Lulingila | 5,019 | 2,496 | 2,523 | 3,203 | 1,452 | 1,751 | 8,222 | 3,948 | 4,274 |
| Munikashi | 3,948 | 2,026 | 1,922 | 2,343 | 1,092 | 1,251 | 6,291 | 3,118 | 3,173 |
| Chinama | 3,043 | 1,520 | 1,523 | 1,684 | 771 | 913 | 4,727 | 2,291 | 2,436 |
| Lukulu | 4,378 | 2,207 | 2,171 | 2,518 | 1,184 | 1,334 | 6,896 | 3,391 | 3,505 |
| Lulimala | 5,281 | 2,657 | 2,624 | 3,276 | 1,558 | 1,718 | 8,557 | 4,215 | 4,342 |
| Chibwa | 2,920 | 1,472 | 1,448 | 1,886 | 915 | 971 | 4,806 | 2,387 | 2,419 |
| **Mfuwe Constituency** | **13,198** | **6,675** | **6,523** | **8,449** | **3,961** | **4,488** | **21,647** | **10,636** | **11,011** |
| Chifungwe | 3,068 | 1,511 | 1,557 | 1,847 | 851 | 996 | 4,915 | 2,362 | 2,553 |
| Muchinga | 2,216 | 1,110 | 1,106 | 1,366 | 607 | 759 | 3,582 | 1,717 | 1,865 |
| Chikanda | 3,891 | 1,985 | 1,906 | 2,634 | 1,253 | 1,381 | 6,525 | 3,238 | 3,287 |
| Mupamadzi | 4,023 | 2,069 | 1,954 | 2,602 | 1,250 | 1,352 | 6,625 | 3,319 | 3,306 |

## Table A11: Population (De Jure) by Age 0-14 Years (Children), 15-34 Years (Youths) and Total Population 0-34 Years by Sex, Province, District, Constituency and Ward, Zambia 2010

| Province,District,Constituency and Ward | 0 -14 Years | | | 15 - 34 Years | | | 0 - 34 Years | | |
|---|---|---|---|---|---|---|---|---|---|
| | Total | Male | Female | Total | Male | Female | Total | Male | Female |
| **Mpika Central Constituency** | **43,258** | **21,492** | **21,766** | **32,915** | **15,707** | **17,208** | **76,173** | **37,199** | **38,974** |
| Mukungwa | 3,758 | 1,903 | 1,855 | 2,463 | 1,167 | 1,296 | 6,221 | 3,070 | 3,151 |
| Lwitikila | 4,796 | 2,363 | 2,433 | 3,530 | 1,699 | 1,831 | 8,326 | 4,062 | 4,264 |
| Musakanya | 9,528 | 4,670 | 4,858 | 9,148 | 4,418 | 4,730 | 18,676 | 9,088 | 9,588 |
| Lubambala | 7,162 | 3,490 | 3,672 | 5,988 | 2,743 | 3,245 | 13,150 | 6,233 | 6,917 |
| Chishibe Isonde | 7,189 | 3,596 | 3,593 | 4,932 | 2,358 | 2,574 | 12,121 | 5,954 | 6,167 |
| Nachikufu | 3,950 | 2,032 | 1,918 | 2,387 | 1,156 | 1,231 | 6,337 | 3,188 | 3,149 |
| Mutekwe | 2,504 | 1,290 | 1,214 | 1,690 | 811 | 879 | 4,194 | 2,101 | 2,093 |
| Chipembele | 4,371 | 2,148 | 2,223 | 2,777 | 1,355 | 1,422 | 7,148 | 3,503 | 3,645 |
| **Nakonde District** | **57,038** | **28,302** | **28,736** | **41,142** | **19,585** | **21,557** | **98,180** | **47,887** | **50,293** |
| **Nakonde Constituency** | **57,038** | **28,302** | **28,736** | **41,142** | **19,585** | **21,557** | **98,180** | **47,887** | **50,293** |
| Mulalo | 4,817 | 2,298 | 2,519 | 3,003 | 1,419 | 1,584 | 7,820 | 3,717 | 4,103 |
| Luchinde | 2,920 | 1,423 | 1,497 | 1,750 | 804 | 946 | 4,670 | 2,227 | 2,443 |
| Ng'umba | 3,236 | 1,618 | 1,618 | 2,203 | 1,086 | 1,117 | 5,439 | 2,704 | 2,735 |
| Musyani | 3,416 | 1,707 | 1,709 | 2,394 | 1,180 | 1,214 | 5,810 | 2,887 | 2,923 |
| Popomozi | 3,113 | 1,627 | 1,486 | 1,982 | 950 | 1,032 | 5,095 | 2,577 | 2,518 |
| Chiwaza | 3,842 | 1,842 | 2,000 | 2,408 | 1,138 | 1,270 | 6,250 | 2,980 | 3,270 |
| Ilonda | 2,338 | 1,158 | 1,180 | 1,621 | 782 | 839 | 3,959 | 1,940 | 2,019 |
| Isunda | 3,175 | 1,608 | 1,567 | 2,051 | 1,002 | 1,049 | 5,226 | 2,610 | 2,616 |
| Nakonde | 17,774 | 8,840 | 8,934 | 14,829 | 7,011 | 7,818 | 32,603 | 15,851 | 16,752 |
| Old Fife | 5,706 | 2,785 | 2,921 | 4,442 | 2,089 | 2,353 | 10,148 | 4,874 | 5,274 |
| Musele | 1,954 | 975 | 979 | 1,251 | 606 | 645 | 3,205 | 1,581 | 1,624 |
| Mpande | 3,607 | 1,838 | 1,769 | 2,310 | 1,093 | 1,217 | 5,917 | 2,931 | 2,986 |
| Mukulika | 1,140 | 583 | 557 | 898 | 425 | 473 | 2,038 | 1,008 | 1,030 |
| **Northern Province** | **538,126** | **269,359** | **268,767** | **355,868** | **169,887** | **185,981** | **893,994** | **439,246** | **454,748** |
| **Chilubi District** | **40,198** | **20,249** | **19,949** | **24,478** | **11,494** | **12,984** | **64,676** | **31,743** | **32,933** |
| **Chilubi Constituency** | **40,198** | **20,249** | **19,949** | **24,478** | **11,494** | **12,984** | **64,676** | **31,743** | **32,933** |
| Mulanda | 2,842 | 1,451 | 1,391 | 1,585 | 729 | 856 | 4,427 | 2,180 | 2,247 |
| Chifwenge | 4,094 | 2,087 | 2,007 | 2,346 | 1,118 | 1,228 | 6,440 | 3,205 | 3,235 |
| Bulilo | 3,220 | 1,651 | 1,569 | 2,062 | 931 | 1,131 | 5,282 | 2,582 | 2,700 |
| Chisupa | 1,817 | 911 | 906 | 1,147 | 548 | 599 | 2,964 | 1,459 | 1,505 |
| Ndela | 1,572 | 783 | 789 | 992 | 470 | 522 | 2,564 | 1,253 | 1,311 |
| Katamba | 1,606 | 790 | 816 | 969 | 459 | 510 | 2,575 | 1,249 | 1,326 |
| Muteka | 1,419 | 754 | 665 | 944 | 460 | 484 | 2,363 | 1,214 | 1,149 |
| Lwenda | 1,875 | 956 | 919 | 1,065 | 485 | 580 | 2,940 | 1,441 | 1,499 |
| Mofu | 1,519 | 774 | 745 | 867 | 377 | 490 | 2,386 | 1,151 | 1,235 |
| Mpanshya | 797 | 384 | 413 | 530 | 268 | 262 | 1,327 | 652 | 675 |
| Kashitu | 1,257 | 625 | 632 | 808 | 383 | 425 | 2,065 | 1,008 | 1,057 |
| Kambashi | 1,529 | 740 | 789 | 863 | 395 | 468 | 2,392 | 1,135 | 1,257 |
| Nguni | 567 | 298 | 269 | 371 | 175 | 196 | 938 | 473 | 465 |
| Chinkundu | 799 | 397 | 402 | 483 | 219 | 264 | 1,282 | 616 | 666 |
| Chiloba | 2,209 | 1,124 | 1,085 | 1,345 | 591 | 754 | 3,554 | 1,715 | 1,839 |
| Kanchindi | 3,457 | 1,723 | 1,734 | 2,276 | 1,082 | 1,194 | 5,733 | 2,805 | 2,928 |
| Kapoka | 1,090 | 537 | 553 | 731 | 363 | 368 | 1,821 | 900 | 921 |
| Kawena | 2,770 | 1,358 | 1,412 | 1,627 | 783 | 844 | 4,397 | 2,141 | 2,256 |
| Kanama | 721 | 355 | 366 | 430 | 196 | 234 | 1,151 | 551 | 600 |
| Bumba | 3,107 | 1,584 | 1,523 | 1,721 | 795 | 926 | 4,828 | 2,379 | 2,449 |
| Mubemba | 959 | 473 | 486 | 575 | 276 | 299 | 1,534 | 749 | 785 |
| Luangwa | 972 | 494 | 478 | 741 | 391 | 350 | 1,713 | 885 | 828 |

## Table A11: Population (De Jure) by Age 0-14 Years (Children), 15-34 Years (Youths) and Total Population 0-34 Years by Sex, Province, District, Constituency and Ward, Zambia 2010

| Province,District,Constituency and Ward | 0 -14 Years | | | 15 - 34 Years | | | 0 - 34 Years | | |
|---|---|---|---|---|---|---|---|---|---|
| | Total | Male | Female | Total | Male | Female | Total | Male | Female |
| **Kaputa District** | **59,803** | **29,828** | **29,975** | **38,160** | **17,978** | **20,182** | **97,963** | **47,806** | **50,157** |
| **Chimbamilonga Constituency** | **24,286** | **12,062** | **12,224** | **15,420** | **7,250** | **8,170** | **39,706** | **19,312** | **20,394** |
| Kapisha | 4,430 | 2,244 | 2,186 | 3,095 | 1,441 | 1,654 | 7,525 | 3,685 | 3,840 |
| Nsumbu | 2,072 | 1,008 | 1,064 | 1,558 | 754 | 804 | 3,630 | 1,762 | 1,868 |
| Chishela | 1,037 | 501 | 536 | 614 | 300 | 314 | 1,651 | 801 | 850 |
| Munwa | 3,938 | 1,983 | 1,955 | 2,377 | 1,110 | 1,267 | 6,315 | 3,093 | 3,222 |
| Kampinda | 3,429 | 1,718 | 1,711 | 2,097 | 985 | 1,112 | 5,526 | 2,703 | 2,823 |
| Kakusu | 599 | 288 | 311 | 394 | 185 | 209 | 993 | 473 | 520 |
| Kashikishi | 2,046 | 1,027 | 1,019 | 1,251 | 600 | 651 | 3,297 | 1,627 | 1,670 |
| Mwambeshi | 872 | 437 | 435 | 548 | 254 | 294 | 1,420 | 691 | 729 |
| Chubo | 1,548 | 748 | 800 | 947 | 459 | 488 | 2,495 | 1,207 | 1,288 |
| Fungwa | 2,265 | 1,112 | 1,153 | 1,353 | 629 | 724 | 3,618 | 1,741 | 1,877 |
| Mukubwe | 2,050 | 996 | 1,054 | 1,186 | 533 | 653 | 3,236 | 1,529 | 1,707 |
| **Kaputa Constituency** | **35,517** | **17,766** | **17,751** | **22,740** | **10,728** | **12,012** | **58,257** | **28,494** | **29,763** |
| Chiyilunda | 1,476 | 771 | 705 | 935 | 437 | 498 | 2,411 | 1,208 | 1,203 |
| Mofwe | 713 | 362 | 351 | 470 | 221 | 249 | 1,183 | 583 | 600 |
| Kalungwishi | 2,586 | 1,247 | 1,339 | 1,555 | 737 | 818 | 4,141 | 1,984 | 2,157 |
| Nkota | 2,868 | 1,430 | 1,438 | 1,763 | 839 | 924 | 4,631 | 2,269 | 2,362 |
| Chipili | 5,018 | 2,593 | 2,425 | 3,186 | 1,555 | 1,631 | 8,204 | 4,148 | 4,056 |
| Choma | 4,405 | 2,096 | 2,309 | 3,346 | 1,604 | 1,742 | 7,751 | 3,700 | 4,051 |
| Mowa | 6,352 | 3,184 | 3,168 | 4,131 | 1,905 | 2,226 | 10,483 | 5,089 | 5,394 |
| Kaleulu | 4,828 | 2,442 | 2,386 | 2,960 | 1,368 | 1,592 | 7,788 | 3,810 | 3,978 |
| Mwawe | 1,254 | 643 | 611 | 783 | 357 | 426 | 2,037 | 1,000 | 1,037 |
| Kapulwa | 2,854 | 1,415 | 1,439 | 1,681 | 793 | 888 | 4,535 | 2,208 | 2,327 |
| Munkonge | 3,163 | 1,583 | 1,580 | 1,930 | 912 | 1,018 | 5,093 | 2,495 | 2,598 |
| **Kasama District** | **107,872** | **53,462** | **54,410** | **78,527** | **37,740** | **40,787** | **186,399** | **91,202** | **95,197** |
| **Kasama Central Constituency** | **73,054** | **35,928** | **37,126** | **56,326** | **27,003** | **29,323** | **129,380** | **62,931** | **66,449** |
| Kasenga | 6,250 | 3,077 | 3,173 | 3,902 | 1,917 | 1,985 | 10,152 | 4,994 | 5,158 |
| Bululu | 9,398 | 4,830 | 4,568 | 5,776 | 2,773 | 3,003 | 15,174 | 7,603 | 7,571 |
| Chilunga | 5,983 | 2,852 | 3,131 | 3,766 | 1,778 | 1,988 | 9,749 | 4,630 | 5,119 |
| Lukulu | 1,981 | 994 | 987 | 1,173 | 562 | 611 | 3,154 | 1,556 | 1,598 |
| Julia Chikamoneka | 1,317 | 632 | 685 | 857 | 407 | 450 | 2,174 | 1,039 | 1,135 |
| Lukupa | 4,271 | 2,156 | 2,115 | 3,140 | 1,541 | 1,599 | 7,411 | 3,697 | 3,714 |
| Mulilansolo | 14,574 | 7,126 | 7,448 | 14,719 | 7,102 | 7,617 | 29,293 | 14,228 | 15,065 |
| Buseko | 29,280 | 14,261 | 15,019 | 22,993 | 10,923 | 12,070 | 52,273 | 25,184 | 27,089 |
| **Lukashya Constituency** | **34,818** | **17,534** | **17,284** | **22,201** | **10,737** | **11,464** | **57,019** | **28,271** | **28,748** |
| Lusenga | 5,523 | 2,857 | 2,666 | 3,373 | 1,634 | 1,739 | 8,896 | 4,491 | 4,405 |
| Mukanga | 3,729 | 1,817 | 1,912 | 2,541 | 1,253 | 1,288 | 6,270 | 3,070 | 3,200 |
| Lualuo | 4,152 | 2,116 | 2,036 | 2,655 | 1,291 | 1,364 | 6,807 | 3,407 | 3,400 |
| Chiba | 3,614 | 1,774 | 1,840 | 2,471 | 1,202 | 1,269 | 6,085 | 2,976 | 3,109 |
| Kapumaula | 4,642 | 2,405 | 2,237 | 2,954 | 1,464 | 1,490 | 7,596 | 3,869 | 3,727 |
| Chibundu | 4,439 | 2,177 | 2,262 | 2,788 | 1,322 | 1,466 | 7,227 | 3,499 | 3,728 |
| Kapongolo | 3,293 | 1,694 | 1,599 | 2,100 | 1,018 | 1,082 | 5,393 | 2,712 | 2,681 |
| Musowa | 2,935 | 1,433 | 1,502 | 1,773 | 826 | 947 | 4,708 | 2,259 | 2,449 |
| Chumba | 2,491 | 1,261 | 1,230 | 1,546 | 727 | 819 | 4,037 | 1,988 | 2,049 |
| **Luwingu District** | **59,217** | **29,800** | **29,417** | **38,865** | **18,354** | **20,511** | **98,082** | **48,154** | **49,928** |
| **Lubansenshi Constituency** | **25,090** | **12,614** | **12,476** | **17,312** | **8,232** | **9,080** | **42,402** | **20,846** | **21,556** |
| Ipusukilo | 1,990 | 990 | 1,000 | 1,236 | 562 | 674 | 3,226 | 1,552 | 1,674 |
| Katopola | 4,573 | 2,257 | 2,316 | 4,192 | 2,029 | 2,163 | 8,765 | 4,286 | 4,479 |
| Namukolo | 464 | 239 | 225 | 410 | 201 | 209 | 874 | 440 | 434 |
| Chulungoma | 3,020 | 1,515 | 1,505 | 2,242 | 1,080 | 1,162 | 5,262 | 2,595 | 2,667 |
| Masonde | 3,112 | 1,631 | 1,481 | 2,136 | 1,038 | 1,098 | 5,248 | 2,669 | 2,579 |
| Chifwile | 2,340 | 1,197 | 1,143 | 1,441 | 669 | 772 | 3,781 | 1,866 | 1,915 |
| Mushitu-Wamboo | 3,236 | 1,621 | 1,615 | 1,903 | 943 | 960 | 5,139 | 2,564 | 2,575 |

## Table A11: Population (De Jure) by Age 0-14 Years (Children), 15-34 Years (Youths) and Total Population 0-34 Years by Sex, Province, District, Constituency and Ward, Zambia 2010

| Province,District,Constituency and Ward | 0 -14 Years | | | 15 - 34 Years | | | 0 - 34 Years | | |
|---|---|---|---|---|---|---|---|---|---|
| | Total | Male | Female | Total | Male | Female | Total | Male | Female |
| Lwata | 2,609 | 1,259 | 1,350 | 1,598 | 747 | 851 | 4,207 | 2,006 | 2,201 |
| Isangano | 3,746 | 1,905 | 1,841 | 2,154 | 963 | 1,191 | 5,900 | 2,868 | 3,032 |
| **Lupososhi Constituency** | **34,127** | **17,186** | **16,941** | **21,553** | **10,122** | **11,431** | **55,680** | **27,308** | **28,372** |
| Ntandashi | 2,473 | 1,210 | 1,263 | 1,680 | 795 | 885 | 4,153 | 2,005 | 2,148 |
| Kaela | 2,824 | 1,410 | 1,414 | 1,878 | 901 | 977 | 4,702 | 2,311 | 2,391 |
| Munshishinga | 2,831 | 1,414 | 1,417 | 1,748 | 831 | 917 | 4,579 | 2,245 | 2,334 |
| Katilye | 2,008 | 973 | 1,035 | 1,193 | 536 | 657 | 3,201 | 1,509 | 1,692 |
| Kafinsa | 1,721 | 873 | 848 | 1,067 | 471 | 596 | 2,788 | 1,344 | 1,444 |
| Kampemba | 3,332 | 1,664 | 1,668 | 2,077 | 995 | 1,082 | 5,409 | 2,659 | 2,750 |
| Mulalashi | 1,291 | 616 | 675 | 852 | 397 | 455 | 2,143 | 1,013 | 1,130 |
| Mufili | 2,431 | 1,240 | 1,191 | 1,486 | 714 | 772 | 3,917 | 1,954 | 1,963 |
| Ilambo | 2,134 | 1,042 | 1,092 | 1,224 | 559 | 665 | 3,358 | 1,601 | 1,757 |
| Ibale | 1,328 | 687 | 641 | 795 | 376 | 419 | 2,123 | 1,063 | 1,060 |
| Bwalinde | 4,855 | 2,485 | 2,370 | 3,066 | 1,416 | 1,650 | 7,921 | 3,901 | 4,020 |
| Mwelawamangu | 4,271 | 2,206 | 2,065 | 2,813 | 1,349 | 1,464 | 7,084 | 3,555 | 3,529 |
| Isansa | 2,628 | 1,366 | 1,262 | 1,674 | 782 | 892 | 4,302 | 2,148 | 2,154 |
| **Mbala District** | **100,165** | **50,204** | **49,961** | **65,607** | **31,618** | **33,989** | **165,772** | **81,822** | **83,950** |
| **Mbala Constituency** | **53,865** | **26,695** | **27,170** | **36,534** | **17,642** | **18,892** | **90,399** | **44,337** | **46,062** |
| Intala | 11,603 | 5,783 | 5,820 | 7,355 | 3,560 | 3,795 | 18,958 | 9,343 | 9,615 |
| Motomoto | 1,375 | 684 | 691 | 1,048 | 503 | 545 | 2,423 | 1,187 | 1,236 |
| Kazimolwa | 10,998 | 5,395 | 5,603 | 8,817 | 4,269 | 4,548 | 19,815 | 9,664 | 10,151 |
| Mwambezi | 3,934 | 1,984 | 1,950 | 2,590 | 1,243 | 1,347 | 6,524 | 3,227 | 3,297 |
| Nsunzu | 7,167 | 3,524 | 3,643 | 5,098 | 2,433 | 2,665 | 12,265 | 5,957 | 6,308 |
| Kawimbe | 4,328 | 2,110 | 2,218 | 2,710 | 1,301 | 1,409 | 7,038 | 3,411 | 3,627 |
| Mwamba | 3,085 | 1,551 | 1,534 | 1,905 | 942 | 963 | 4,990 | 2,493 | 2,497 |
| Luandi | 11,375 | 5,664 | 5,711 | 7,011 | 3,391 | 3,620 | 18,386 | 9,055 | 9,331 |
| **Senga Hill Constituency** | **46,300** | **23,509** | **22,791** | **29,073** | **13,976** | **15,097** | **75,373** | **37,485** | **37,888** |
| Mukololo | 6,663 | 3,394 | 3,269 | 4,103 | 1,959 | 2,144 | 10,766 | 5,353 | 5,413 |
| Lapisha | 6,201 | 3,191 | 3,010 | 4,016 | 1,940 | 2,076 | 10,217 | 5,131 | 5,086 |
| Malamba | 4,072 | 2,116 | 1,956 | 2,490 | 1,178 | 1,312 | 6,562 | 3,294 | 3,268 |
| Chimbili | 6,347 | 3,160 | 3,187 | 3,988 | 1,917 | 2,071 | 10,335 | 5,077 | 5,258 |
| Chela | 8,088 | 4,126 | 3,962 | 5,076 | 2,500 | 2,576 | 13,164 | 6,626 | 6,538 |
| Mwiluzi | 3,294 | 1,723 | 1,571 | 2,172 | 1,059 | 1,113 | 5,466 | 2,782 | 2,684 |
| Chinyika | 4,758 | 2,400 | 2,358 | 3,017 | 1,413 | 1,604 | 7,775 | 3,813 | 3,962 |
| Ipembe | 3,346 | 1,658 | 1,688 | 1,978 | 948 | 1,030 | 5,324 | 2,606 | 2,718 |
| Chozi | 3,531 | 1,741 | 1,790 | 2,233 | 1,062 | 1,171 | 5,764 | 2,803 | 2,961 |
| **Mporokoso District** | **48,816** | **24,499** | **24,317** | **30,965** | **14,976** | **15,989** | **79,781** | **39,475** | **40,306** |
| **Lunte Constituency** | **29,039** | **14,569** | **14,470** | **17,693** | **8,647** | **9,046** | **46,732** | **23,216** | **23,516** |
| Kasanshi | 1,793 | 918 | 875 | 1,132 | 557 | 575 | 2,925 | 1,475 | 1,450 |
| Isenga | 2,449 | 1,234 | 1,215 | 1,555 | 754 | 801 | 4,004 | 1,988 | 2,016 |
| Nchelenge | 2,937 | 1,498 | 1,439 | 1,755 | 826 | 929 | 4,692 | 2,324 | 2,368 |
| Malambwa | 1,795 | 888 | 907 | 1,066 | 525 | 541 | 2,861 | 1,413 | 1,448 |
| Malaila | 2,145 | 1,105 | 1,040 | 1,330 | 671 | 659 | 3,475 | 1,776 | 1,699 |
| Bwandela | 2,532 | 1,244 | 1,288 | 1,449 | 714 | 735 | 3,981 | 1,958 | 2,023 |
| Masonde | 821 | 398 | 423 | 571 | 296 | 275 | 1,392 | 694 | 698 |
| Luangwa | 3,397 | 1,751 | 1,646 | 1,883 | 903 | 980 | 5,280 | 2,654 | 2,626 |
| Kalungwishi | 3,771 | 1,869 | 1,902 | 2,408 | 1,180 | 1,228 | 6,179 | 3,049 | 3,130 |
| Lunte | 3,609 | 1,812 | 1,797 | 2,188 | 1,062 | 1,126 | 5,797 | 2,874 | 2,923 |
| Kanyanta | 2,239 | 1,093 | 1,146 | 1,412 | 686 | 726 | 3,651 | 1,779 | 1,872 |
| Lubushi | 1,551 | 759 | 792 | 944 | 473 | 471 | 2,495 | 1,232 | 1,263 |

## Table A11: Population (De Jure) by Age 0-14 Years (Children), 15-34 Years (Youths) and Total Population 0-34 Years by Sex, Province, District, Constituency and Ward, Zambia 2010

| Province,District,Constituency and Ward | 0 -14 Years | | | 15 - 34 Years | | | 0 - 34 Years | | |
|---|---|---|---|---|---|---|---|---|---|
| | Total | Male | Female | Total | Male | Female | Total | Male | Female |
| **Mporokoso Constituency** | **19,777** | **9,930** | **9,847** | **13,272** | **6,329** | **6,943** | **33,049** | **16,259** | **16,790** |
| Lumangwe | 2,301 | 1,241 | 1,060 | 1,347 | 609 | 738 | 3,648 | 1,850 | 1,798 |
| Chikulu | 1,173 | 596 | 577 | 645 | 320 | 325 | 1,818 | 916 | 902 |
| Mumbuluma | 1,252 | 622 | 630 | 767 | 374 | 393 | 2,019 | 996 | 1,023 |
| Chisha Mwamba | 4,125 | 2,085 | 2,040 | 2,625 | 1,247 | 1,378 | 6,750 | 3,332 | 3,418 |
| Mikomba | 2,261 | 1,117 | 1,144 | 2,177 | 1,048 | 1,129 | 4,438 | 2,165 | 2,273 |
| Kapumo | 1,751 | 836 | 915 | 1,259 | 624 | 635 | 3,010 | 1,460 | 1,550 |
| Muchinga | 2,117 | 1,065 | 1,052 | 1,427 | 674 | 753 | 3,544 | 1,739 | 1,805 |
| Chimpolonge | 1,761 | 812 | 949 | 1,199 | 599 | 600 | 2,960 | 1,411 | 1,549 |
| Mutotoshi | 1,509 | 779 | 730 | 904 | 396 | 508 | 2,413 | 1,175 | 1,238 |
| Mabale | 1,527 | 777 | 750 | 922 | 438 | 484 | 2,449 | 1,215 | 1,234 |
| **Mpulungu District** | **47,555** | **23,751** | **23,804** | **32,854** | **15,591** | **17,263** | **80,409** | **39,342** | **41,067** |
| **Mpulungu Constituency** | **47,555** | **23,751** | **23,804** | **32,854** | **15,591** | **17,263** | **80,409** | **39,342** | **41,067** |
| Kapembwa | 1,065 | 512 | 553 | 716 | 359 | 357 | 1,781 | 871 | 910 |
| Katwe | 1,671 | 849 | 822 | 1,038 | 486 | 552 | 2,709 | 1,335 | 1,374 |
| Chibulula | 11,503 | 5,789 | 5,714 | 8,201 | 3,892 | 4,309 | 19,704 | 9,681 | 10,023 |
| Mpulungu Central | 9,653 | 4,726 | 4,927 | 8,224 | 3,819 | 4,405 | 17,877 | 8,545 | 9,332 |
| Tanganyika | 1,398 | 716 | 682 | 949 | 435 | 514 | 2,347 | 1,151 | 1,196 |
| Chilumba | 385 | 196 | 189 | 242 | 122 | 120 | 627 | 318 | 309 |
| Isoko | 4,471 | 2,250 | 2,221 | 2,866 | 1,356 | 1,510 | 7,337 | 3,606 | 3,731 |
| Iyendwe | 2,086 | 1,005 | 1,081 | 1,241 | 578 | 663 | 3,327 | 1,583 | 1,744 |
| Mumila | 2,214 | 1,118 | 1,096 | 1,320 | 639 | 681 | 3,534 | 1,757 | 1,777 |
| Itimbwe | 1,213 | 610 | 603 | 771 | 361 | 410 | 1,984 | 971 | 1,013 |
| Vyamba | 5,204 | 2,599 | 2,605 | 3,287 | 1,622 | 1,665 | 8,491 | 4,221 | 4,270 |
| Chisha | 5,140 | 2,579 | 2,561 | 3,076 | 1,468 | 1,608 | 8,216 | 4,047 | 4,169 |
| Isunga | 1,552 | 802 | 750 | 923 | 454 | 469 | 2,475 | 1,256 | 1,219 |
| **Mungwi District** | **74,500** | **37,566** | **36,934** | **46,412** | **22,136** | **24,276** | **120,912** | **59,702** | **61,210** |
| **Malole Constituency** | **74,500** | **37,566** | **36,934** | **46,412** | **22,136** | **24,276** | **120,912** | **59,702** | **61,210** |
| Lubala | 6,807 | 3,462 | 3,345 | 4,244 | 2,013 | 2,231 | 11,051 | 5,475 | 5,576 |
| Mpanda | 6,305 | 3,181 | 3,124 | 3,970 | 1,900 | 2,070 | 10,275 | 5,081 | 5,194 |
| Kabisha | 4,418 | 2,175 | 2,243 | 2,615 | 1,275 | 1,340 | 7,033 | 3,450 | 3,583 |
| Fibwe | 5,397 | 2,818 | 2,579 | 3,217 | 1,523 | 1,694 | 8,614 | 4,341 | 4,273 |
| Chibamba | 8,661 | 4,365 | 4,296 | 5,422 | 2,588 | 2,834 | 14,083 | 6,953 | 7,130 |
| Iyaya | 5,884 | 2,956 | 2,928 | 3,465 | 1,648 | 1,817 | 9,349 | 4,604 | 4,745 |
| Kalungu | 7,217 | 3,606 | 3,611 | 4,421 | 2,060 | 2,361 | 11,638 | 5,666 | 5,972 |
| Fube | 6,897 | 3,422 | 3,475 | 4,360 | 2,119 | 2,241 | 11,257 | 5,541 | 5,716 |
| Ngulula | 4,740 | 2,394 | 2,346 | 2,976 | 1,382 | 1,594 | 7,716 | 3,776 | 3,940 |
| Mungwi | 6,051 | 3,064 | 2,987 | 4,275 | 2,065 | 2,210 | 10,326 | 5,129 | 5,197 |
| Chambeshi | 7,018 | 3,533 | 3,485 | 4,243 | 2,022 | 2,221 | 11,261 | 5,555 | 5,706 |
| Mabula | 2,513 | 1,281 | 1,232 | 1,653 | 787 | 866 | 4,166 | 2,068 | 2,098 |
| Musensenshe | 2,592 | 1,309 | 1,283 | 1,551 | 754 | 797 | 4,143 | 2,063 | 2,080 |
| **North Western Province** | **354,494** | **177,019** | **177,475** | **239,522** | **115,472** | **124,050** | **594,016** | **292,491** | **301,525** |
| **Chavuma District** | **17,041** | **8,516** | **8,525** | **11,278** | **5,426** | **5,852** | **28,319** | **13,942** | **14,377** |
| **Chavuma Constituency** | **17,041** | **8,516** | **8,525** | **11,278** | **5,426** | **5,852** | **28,319** | **13,942** | **14,377** |
| Chambi Mandalo | 1,246 | 617 | 629 | 756 | 370 | 386 | 2,002 | 987 | 1,015 |
| Sewe | 1,231 | 617 | 614 | 772 | 378 | 394 | 2,003 | 995 | 1,008 |
| Lingelengenda | 882 | 446 | 436 | 510 | 239 | 271 | 1,392 | 685 | 707 |
| Chiyeke | 2,674 | 1,375 | 1,299 | 1,982 | 942 | 1,040 | 4,656 | 2,317 | 2,339 |
| Kalombo Kamisamba | 1,045 | 529 | 516 | 755 | 343 | 412 | 1,800 | 872 | 928 |
| Chivombo Mbalango | 717 | 352 | 365 | 406 | 200 | 206 | 1,123 | 552 | 571 |
| Chavuma Central | 3,364 | 1,657 | 1,707 | 2,476 | 1,183 | 1,293 | 5,840 | 2,840 | 3,000 |
| Sanjongo | 662 | 321 | 341 | 449 | 236 | 213 | 1,111 | 557 | 554 |
| Lingundu | 530 | 264 | 266 | 342 | 170 | 172 | 872 | 434 | 438 |
| Lukolwe Musumba | 590 | 299 | 291 | 350 | 183 | 167 | 940 | 482 | 458 |

## Table A11: Population (De Jure) by Age 0-14 Years (Children), 15-34 Years (Youths) and Total Population 0-34 Years by Sex, Province, District, Constituency and Ward, Zambia 2010

| Province,District,Constituency and Ward | 0 -14 Years | | | 15 - 34 Years | | | 0 - 34 Years | | |
|---|---|---|---|---|---|---|---|---|---|
| | Total | Male | Female | Total | Male | Female | Total | Male | Female |
| Kambuya Mukelangombe | 1,950 | 984 | 966 | 1,228 | 596 | 632 | 3,178 | 1,580 | 1,598 |
| Nyatanda Nyambingila | 1,403 | 690 | 713 | 802 | 384 | 418 | 2,205 | 1,074 | 1,131 |
| Nguvu | 747 | 365 | 382 | 450 | 202 | 248 | 1,197 | 567 | 630 |
| **Ikelenge District** | **16,264** | **8,222** | **8,042** | **10,360** | **5,068** | **5,292** | **26,624** | **13,290** | **13,334** |
| **Ikelenge Constituency** | **16,264** | **8,222** | **8,042** | **10,360** | **5,068** | **5,292** | **26,624** | **13,290** | **13,334** |
| Chana Chamuhinga | 1,643 | 812 | 831 | 1,020 | 504 | 516 | 2,663 | 1,316 | 1,347 |
| Jimbe | 1,923 | 974 | 949 | 1,177 | 578 | 599 | 3,100 | 1,552 | 1,548 |
| Nyakaseya | 4,029 | 2,066 | 1,963 | 2,588 | 1,220 | 1,368 | 6,617 | 3,286 | 3,331 |
| Ikeleng'i | 3,944 | 2,021 | 1,923 | 2,593 | 1,291 | 1,302 | 6,537 | 3,312 | 3,225 |
| Mwininyilamba | 2,439 | 1,207 | 1,232 | 1,555 | 779 | 776 | 3,994 | 1,986 | 2,008 |
| Mukangala | 2,286 | 1,142 | 1,144 | 1,427 | 696 | 731 | 3,713 | 1,838 | 1,875 |
| **Kabompo District** | **44,821** | **22,512** | **22,309** | **29,395** | **14,119** | **15,276** | **74,216** | **36,631** | **37,585** |
| **Kabompo East Constituency** | **23,628** | **11,916** | **11,712** | **15,115** | **7,315** | **7,800** | **38,743** | **19,231** | **19,512** |
| Dihamba | 1,294 | 649 | 645 | 748 | 361 | 387 | 2,042 | 1,010 | 1,032 |
| Lunsona | 2,714 | 1,366 | 1,348 | 1,615 | 794 | 821 | 4,329 | 2,160 | 2,169 |
| Kashinakaji | 274 | 145 | 129 | 136 | 64 | 72 | 410 | 209 | 201 |
| Chiteve | 703 | 334 | 369 | 400 | 199 | 201 | 1,103 | 533 | 570 |
| Manyinga | 734 | 361 | 373 | 422 | 225 | 197 | 1,156 | 586 | 570 |
| Loloma | 5,782 | 2,915 | 2,867 | 4,285 | 2,047 | 2,238 | 10,067 | 4,962 | 5,105 |
| Kawanda | 3,298 | 1,705 | 1,593 | 1,980 | 922 | 1,058 | 5,278 | 2,627 | 2,651 |
| Kaula | 7,828 | 3,942 | 3,886 | 4,945 | 2,423 | 2,522 | 12,773 | 6,365 | 6,408 |
| Chongo | 1,001 | 499 | 502 | 584 | 280 | 304 | 1,585 | 779 | 806 |
| **Kabompo West Constituency** | **21,193** | **10,596** | **10,597** | **14,280** | **6,804** | **7,476** | **35,473** | **17,400** | **18,073** |
| Kamafwafwa | 1,838 | 930 | 908 | 1,154 | 534 | 620 | 2,992 | 1,464 | 1,528 |
| Kabompo | 4,100 | 2,049 | 2,051 | 3,742 | 1,797 | 1,945 | 7,842 | 3,846 | 3,996 |
| Litoya | 765 | 383 | 382 | 480 | 225 | 255 | 1,245 | 608 | 637 |
| Kamisombo | 1,984 | 1,014 | 970 | 1,272 | 628 | 644 | 3,256 | 1,642 | 1,614 |
| Kabulamema | 2,034 | 970 | 1,064 | 1,328 | 606 | 722 | 3,362 | 1,576 | 1,786 |
| Mumbeji | 1,995 | 1,024 | 971 | 1,201 | 546 | 655 | 3,196 | 1,570 | 1,626 |
| Luli | 1,291 | 629 | 662 | 812 | 373 | 439 | 2,103 | 1,002 | 1,101 |
| Katuva | 1,214 | 614 | 600 | 742 | 379 | 363 | 1,956 | 993 | 963 |
| Maveve | 911 | 458 | 453 | 531 | 260 | 271 | 1,442 | 718 | 724 |
| Chikenge | 1,447 | 734 | 713 | 832 | 382 | 450 | 2,279 | 1,116 | 1,163 |
| Lunyiwe | 741 | 387 | 354 | 488 | 233 | 255 | 1,229 | 620 | 609 |
| Kayombo | 970 | 452 | 518 | 599 | 299 | 300 | 1,569 | 751 | 818 |
| Chikonkwelo | 1,903 | 952 | 951 | 1,099 | 542 | 557 | 3,002 | 1,494 | 1,508 |
| **Kasempa District** | **34,501** | **17,464** | **17,037** | **22,083** | **10,503** | **11,580** | **56,584** | **27,967** | **28,617** |
| **Kasempa Constituency** | **34,501** | **17,464** | **17,037** | **22,083** | **10,503** | **11,580** | **56,584** | **27,967** | **28,617** |
| Kamakuku | 522 | 264 | 258 | 339 | 168 | 171 | 861 | 432 | 429 |
| Nselauke | 1,765 | 860 | 905 | 1,163 | 557 | 606 | 2,928 | 1,417 | 1,511 |
| Ingwe | 298 | 134 | 164 | 186 | 95 | 91 | 484 | 229 | 255 |
| Kaimbwe | 1,538 | 799 | 739 | 1,038 | 503 | 535 | 2,576 | 1,302 | 1,274 |
| Mukema | 2,301 | 1,229 | 1,072 | 1,304 | 599 | 705 | 3,605 | 1,828 | 1,777 |
| Kamatete | 2,214 | 1,098 | 1,116 | 1,276 | 642 | 634 | 3,490 | 1,740 | 1,750 |
| Dengwe | 1,753 | 894 | 859 | 1,181 | 561 | 620 | 2,934 | 1,455 | 1,479 |
| Njenga | 3,151 | 1,611 | 1,540 | 1,847 | 903 | 944 | 4,998 | 2,514 | 2,484 |
| Kalombe | 1,842 | 931 | 911 | 1,146 | 539 | 607 | 2,988 | 1,470 | 1,518 |
| Kamusongolwa | 419 | 216 | 203 | 544 | 264 | 280 | 963 | 480 | 483 |
| Kikonkomene | 1,956 | 924 | 1,032 | 1,732 | 805 | 927 | 3,688 | 1,729 | 1,959 |
| Nkenyauna | 3,097 | 1,567 | 1,530 | 2,326 | 1,070 | 1,256 | 5,423 | 2,637 | 2,786 |
| Mukinge | 701 | 342 | 359 | 433 | 190 | 243 | 1,134 | 532 | 602 |
| Mutenda | 2,424 | 1,238 | 1,186 | 1,504 | 730 | 774 | 3,928 | 1,968 | 1,960 |
| Lubofu | 1,166 | 595 | 571 | 659 | 307 | 352 | 1,825 | 902 | 923 |
| Mpungu | 669 | 335 | 334 | 439 | 220 | 219 | 1,108 | 555 | 553 |
| Nyoka | 1,959 | 967 | 992 | 1,137 | 524 | 613 | 3,096 | 1,491 | 1,605 |

## Table A11: Population (De Jure) by Age 0-14 Years (Children), 15-34 Years (Youths) and Total Population 0-34 Years by Sex, Province, District, Constituency and Ward, Zambia 2010

| Province,District,Constituency and Ward | 0 -14 Years | | | 15 - 34 Years | | | 0 - 34 Years | | |
|---|---|---|---|---|---|---|---|---|---|
| | Total | Male | Female | Total | Male | Female | Total | Male | Female |
| Kelongwa | 1,528 | 814 | 714 | 778 | 377 | 401 | 2,306 | 1,191 | 1,115 |
| Mukunanshi | 1,890 | 988 | 902 | 1,080 | 523 | 557 | 2,970 | 1,511 | 1,459 |
| Kanongo | 863 | 428 | 435 | 500 | 242 | 258 | 1,363 | 670 | 693 |
| Kamankechi | 1,884 | 946 | 938 | 1,100 | 485 | 615 | 2,984 | 1,431 | 1,553 |
| Jifumpa | 561 | 284 | 277 | 371 | 199 | 172 | 932 | 483 | 449 |
| **Mufumbwe District** | **29,200** | **14,493** | **14,707** | **18,564** | **9,004** | **9,560** | **47,764** | **23,497** | **24,267** |
| **Mufumbwe Constituency** | **29,200** | **14,493** | **14,707** | **18,564** | **9,004** | **9,560** | **47,764** | **23,497** | **24,267** |
| Kashima West | 3,450 | 1,652 | 1,798 | 2,233 | 1,067 | 1,166 | 5,683 | 2,719 | 2,964 |
| Kashima East | 1,014 | 492 | 522 | 638 | 348 | 290 | 1,652 | 840 | 812 |
| Matushi | 4,418 | 2,220 | 2,198 | 2,742 | 1,315 | 1,427 | 7,160 | 3,535 | 3,625 |
| Kamabuta | 1,650 | 831 | 819 | 1,034 | 508 | 526 | 2,684 | 1,339 | 1,345 |
| Kalambu | 5,122 | 2,535 | 2,587 | 3,966 | 1,905 | 2,061 | 9,088 | 4,440 | 4,648 |
| Chizela | 1,537 | 723 | 814 | 949 | 431 | 518 | 2,486 | 1,154 | 1,332 |
| Shukwe | 1,204 | 656 | 548 | 721 | 361 | 360 | 1,925 | 1,017 | 908 |
| Kikonge | 935 | 488 | 447 | 520 | 254 | 266 | 1,455 | 742 | 713 |
| Munyambala | 886 | 463 | 423 | 452 | 211 | 241 | 1,338 | 674 | 664 |
| Kalengwa | 1,092 | 525 | 567 | 614 | 301 | 313 | 1,706 | 826 | 880 |
| Kabipupu | 748 | 360 | 388 | 426 | 209 | 217 | 1,174 | 569 | 605 |
| Mushima | 2,312 | 1,137 | 1,175 | 1,359 | 654 | 705 | 3,671 | 1,791 | 1,880 |
| Musonweji | 1,070 | 548 | 522 | 641 | 331 | 310 | 1,711 | 879 | 832 |
| Kaminzekenzeke | 1,092 | 529 | 563 | 646 | 309 | 337 | 1,738 | 838 | 900 |
| Lalafuta | 518 | 261 | 257 | 286 | 127 | 159 | 804 | 388 | 416 |
| Miluji | 2,152 | 1,073 | 1,079 | 1,337 | 673 | 664 | 3,489 | 1,746 | 1,743 |
| **Mwinilunga District** | **52,431** | **26,250** | **26,181** | **33,143** | **15,994** | **17,149** | **85,574** | **42,244** | **43,330** |
| **Mwinilunga Constituency** | **52,431** | **26,250** | **26,181** | **33,143** | **15,994** | **17,149** | **85,574** | **42,244** | **43,330** |
| Kanongesha | 6,681 | 3,378 | 3,303 | 4,195 | 2,016 | 2,179 | 10,876 | 5,394 | 5,482 |
| Kawiku | 1,570 | 783 | 787 | 968 | 449 | 519 | 2,538 | 1,232 | 1,306 |
| Mulumbi | 7,284 | 3,522 | 3,762 | 5,611 | 2,673 | 2,938 | 12,895 | 6,195 | 6,700 |
| Mundwinji | 4,057 | 2,038 | 2,019 | 2,614 | 1,286 | 1,328 | 6,671 | 3,324 | 3,347 |
| Kapundu | 2,701 | 1,368 | 1,333 | 1,497 | 684 | 813 | 4,198 | 2,052 | 2,146 |
| Kanyama | 2,392 | 1,215 | 1,177 | 1,461 | 720 | 741 | 3,853 | 1,935 | 1,918 |
| Kakoma | 3,813 | 1,919 | 1,894 | 2,108 | 1,003 | 1,105 | 5,921 | 2,922 | 2,999 |
| Kasampula | 572 | 289 | 283 | 339 | 147 | 192 | 911 | 436 | 475 |
| Lumwana | 2,529 | 1,316 | 1,213 | 1,584 | 767 | 817 | 4,113 | 2,083 | 2,030 |
| Sailunga | 1,505 | 761 | 744 | 989 | 460 | 529 | 2,494 | 1,221 | 1,273 |
| Chisasa | 2,000 | 1,014 | 986 | 1,228 | 605 | 623 | 3,228 | 1,619 | 1,609 |
| Ntambu | 4,764 | 2,360 | 2,404 | 2,912 | 1,416 | 1,496 | 7,676 | 3,776 | 3,900 |
| Samuteba | 3,444 | 1,716 | 1,728 | 2,141 | 1,053 | 1,088 | 5,585 | 2,769 | 2,816 |
| Mudyanyama | 1,616 | 824 | 792 | 931 | 469 | 462 | 2,547 | 1,293 | 1,254 |
| Chibwika | 5,060 | 2,520 | 2,540 | 3,080 | 1,513 | 1,567 | 8,140 | 4,033 | 4,107 |
| Kamapanda | 2,443 | 1,227 | 1,216 | 1,485 | 733 | 752 | 3,928 | 1,960 | 1,968 |
| **Solwezi District** | **120,738** | **59,766** | **60,972** | **89,184** | **43,141** | **46,043** | **209,922** | **102,907** | **107,015** |
| **Solwezi Central Constituency** | **59,911** | **29,250** | **30,661** | **50,725** | **24,668** | **26,057** | **110,636** | **53,918** | **56,718** |
| Kapijimpanga | 8,904 | 4,416 | 4,488 | 6,082 | 2,978 | 3,104 | 14,986 | 7,394 | 7,592 |
| Sandangombe | 8,905 | 4,358 | 4,547 | 6,085 | 2,914 | 3,171 | 14,990 | 7,272 | 7,718 |
| Kamalamba | 8,106 | 3,968 | 4,138 | 7,658 | 3,695 | 3,963 | 15,764 | 7,663 | 8,101 |
| Tumvwanganai | 18,001 | 8,755 | 9,246 | 16,163 | 7,893 | 8,270 | 34,164 | 16,648 | 17,516 |
| Kimasala | 15,995 | 7,753 | 8,242 | 14,737 | 7,188 | 7,549 | 30,732 | 14,941 | 15,791 |
| **Solwezi East Constituency** | **17,974** | **9,021** | **8,953** | **11,213** | **5,399** | **5,814** | **29,187** | **14,420** | **14,767** |
| Musaka | 4,479 | 2,264 | 2,215 | 2,639 | 1,265 | 1,374 | 7,118 | 3,529 | 3,589 |
| Chikola | 1,975 | 1,006 | 969 | 1,229 | 621 | 608 | 3,204 | 1,627 | 1,577 |
| Kangwena | 2,003 | 965 | 1,038 | 1,255 | 582 | 673 | 3,258 | 1,547 | 1,711 |
| Kalilele | 1,486 | 740 | 746 | 943 | 444 | 499 | 2,429 | 1,184 | 1,245 |
| Mulonga | 4,755 | 2,414 | 2,341 | 3,090 | 1,485 | 1,605 | 7,845 | 3,899 | 3,946 |

## Table A11: Population (De Jure) by Age 0-14 Years (Children), 15-34 Years (Youths) and Total Population 0-34 Years by Sex, Province, District, Constituency and Ward, Zambia 2010

| Province,District,Constituency and Ward | 0 -14 Years | | | 15 - 34 Years | | | 0 - 34 Years | | |
|---|---|---|---|---|---|---|---|---|---|
| | Total | Male | Female | Total | Male | Female | Total | Male | Female |
| Mapunga | 1,830 | 948 | 882 | 1,253 | 606 | 647 | 3,083 | 1,554 | 1,529 |
| Mujimanzovu | 1,446 | 684 | 762 | 804 | 396 | 408 | 2,250 | 1,080 | 1,170 |
| **Solwezi West Constituency** | **42,853** | **21,495** | **21,358** | **27,246** | **13,074** | **14,172** | **70,099** | **34,569** | **35,530** |
| Mumena | 5,892 | 3,035 | 2,857 | 3,560 | 1,733 | 1,827 | 9,452 | 4,768 | 4,684 |
| Mwajimambwe | 6,205 | 3,148 | 3,057 | 4,051 | 1,942 | 2,109 | 10,256 | 5,090 | 5,166 |
| Kibanza | 2,719 | 1,390 | 1,329 | 1,545 | 777 | 768 | 4,264 | 2,167 | 2,097 |
| Mukumbi | 4,441 | 2,230 | 2,211 | 3,086 | 1,486 | 1,600 | 7,527 | 3,716 | 3,811 |
| Matebo | 1,213 | 643 | 570 | 727 | 368 | 359 | 1,940 | 1,011 | 929 |
| Shilenda | 8,184 | 3,962 | 4,222 | 5,419 | 2,598 | 2,821 | 13,603 | 6,560 | 7,043 |
| Lumwana | 3,738 | 1,848 | 1,890 | 2,478 | 1,169 | 1,309 | 6,216 | 3,017 | 3,199 |
| Mumbezhi | 718 | 382 | 336 | 592 | 277 | 315 | 1,310 | 659 | 651 |
| Musele | 6,174 | 3,111 | 3,063 | 3,784 | 1,769 | 2,015 | 9,958 | 4,880 | 5,078 |
| Chovwe | 3,569 | 1,746 | 1,823 | 2,004 | 955 | 1,049 | 5,573 | 2,701 | 2,872 |
| **Zambezi District** | **39,498** | **19,796** | **19,702** | **25,515** | **12,217** | **13,298** | **65,013** | **32,013** | **33,000** |
| **Zambezi East Constituency** | **28,410** | **14,257** | **14,153** | **18,617** | **8,838** | **9,779** | **47,027** | **23,095** | **23,932** |
| Lunkunyi | 2,286 | 1,158 | 1,128 | 1,360 | 623 | 737 | 3,646 | 1,781 | 1,865 |
| Nyakuleng'a | 2,033 | 1,031 | 1,002 | 1,176 | 571 | 605 | 3,209 | 1,602 | 1,607 |
| Dipalata | 2,963 | 1,443 | 1,520 | 1,743 | 844 | 899 | 4,706 | 2,287 | 2,419 |
| Mukanda Nkunda | 3,807 | 1,875 | 1,932 | 2,249 | 1,076 | 1,173 | 6,056 | 2,951 | 3,105 |
| Chileng'a Chizenzi | 2,518 | 1,279 | 1,239 | 1,500 | 720 | 780 | 4,018 | 1,999 | 2,019 |
| Lwitadi Lwatembo | 1,620 | 826 | 794 | 947 | 463 | 484 | 2,567 | 1,289 | 1,278 |
| Mpidi Kakong'a | 3,996 | 2,078 | 1,918 | 2,229 | 1,052 | 1,177 | 6,225 | 3,130 | 3,095 |
| Chitokoloki | 1,927 | 963 | 964 | 1,432 | 697 | 735 | 3,359 | 1,660 | 1,699 |
| Chivweji Kasesi | 2,872 | 1,436 | 1,436 | 2,025 | 949 | 1,076 | 4,897 | 2,385 | 2,512 |
| Zambezi | 4,388 | 2,168 | 2,220 | 3,956 | 1,843 | 2,113 | 8,344 | 4,011 | 4,333 |
| **Zambezi West Constituency** | **11,088** | **5,539** | **5,549** | **6,898** | **3,379** | **3,519** | **17,986** | **8,918** | **9,068** |
| Mapachi Chinyingi | 1,811 | 921 | 890 | 1,199 | 594 | 605 | 3,010 | 1,515 | 1,495 |
| Likungu | 2,365 | 1,183 | 1,182 | 1,460 | 689 | 771 | 3,825 | 1,872 | 1,953 |
| Liyovu | 2,570 | 1,291 | 1,279 | 1,568 | 772 | 796 | 4,138 | 2,063 | 2,075 |
| Muyembe | 1,077 | 531 | 546 | 595 | 269 | 326 | 1,672 | 800 | 872 |
| Mwange Nyawanda | 1,935 | 967 | 968 | 1,178 | 587 | 591 | 3,113 | 1,554 | 1,559 |
| Matondo Nyachikayi | 1,330 | 646 | 684 | 898 | 468 | 430 | 2,228 | 1,114 | 1,114 |
| **Southern Province** | **754,569** | **375,728** | **378,841** | **556,276** | **269,219** | **287,057** | **1,310,845** | **644,947** | **665,898** |
| **Choma District** | **119,090** | **59,172** | **59,918** | **84,644** | **40,915** | **43,729** | **203,734** | **100,087** | **103,647** |
| **Choma Central Constituency** | **56,156** | **27,891** | **28,265** | **44,966** | **21,667** | **23,299** | **101,122** | **49,558** | **51,564** |
| Batoka | 3,530 | 1,818 | 1,712 | 2,191 | 1,041 | 1,150 | 5,721 | 2,859 | 2,862 |
| Sikalongo | 4,010 | 2,022 | 1,988 | 2,560 | 1,229 | 1,331 | 6,570 | 3,251 | 3,319 |
| Simamvwa | 5,674 | 2,900 | 2,774 | 3,842 | 1,906 | 1,936 | 9,516 | 4,806 | 4,710 |
| Stateland | 2,994 | 1,450 | 1,544 | 2,426 | 1,221 | 1,205 | 5,420 | 2,671 | 2,749 |
| Nakeempa | 2,820 | 1,418 | 1,402 | 1,723 | 834 | 889 | 4,543 | 2,252 | 2,291 |
| Moomba | 1,678 | 818 | 860 | 1,112 | 536 | 576 | 2,790 | 1,354 | 1,436 |
| Kalundana | 5,298 | 2,545 | 2,753 | 5,420 | 2,568 | 2,852 | 10,718 | 5,113 | 5,605 |
| Simacheche | 3,696 | 1,807 | 1,889 | 3,290 | 1,657 | 1,633 | 6,986 | 3,464 | 3,522 |
| Sikalundu | 3,349 | 1,653 | 1,696 | 4,354 | 1,989 | 2,365 | 7,703 | 3,642 | 4,061 |
| Mubula | 8,671 | 4,161 | 4,510 | 8,770 | 4,175 | 4,595 | 17,441 | 8,336 | 9,105 |
| Singani | 4,443 | 2,273 | 2,170 | 2,905 | 1,455 | 1,450 | 7,348 | 3,728 | 3,620 |
| Siasikabole | 4,232 | 2,179 | 2,053 | 2,684 | 1,278 | 1,406 | 6,916 | 3,457 | 3,459 |
| Namuswa | 5,761 | 2,847 | 2,914 | 3,689 | 1,778 | 1,911 | 9,450 | 4,625 | 4,825 |
| **Mbabala Constituency** | **28,962** | **14,481** | **14,481** | **18,544** | **9,075** | **9,469** | **47,506** | **23,556** | **23,950** |
| Simaubi | 6,099 | 3,056 | 3,043 | 3,673 | 1,833 | 1,840 | 9,772 | 4,889 | 4,883 |
| Mapanza | 2,956 | 1,480 | 1,476 | 1,915 | 957 | 958 | 4,871 | 2,437 | 2,434 |
| Mang'unza | 3,198 | 1,573 | 1,625 | 1,871 | 934 | 937 | 5,069 | 2,507 | 2,562 |
| Chilalantambo | 4,760 | 2,404 | 2,356 | 3,021 | 1,470 | 1,551 | 7,781 | 3,874 | 3,907 |
| Macha | 3,575 | 1,803 | 1,772 | 2,628 | 1,234 | 1,394 | 6,203 | 3,037 | 3,166 |
| Kabimba | 1,015 | 487 | 528 | 674 | 339 | 335 | 1,689 | 826 | 863 |

## Table A11: Population (De Jure) by Age 0-14 Years (Children), 15-34 Years (Youths) and Total Population 0-34 Years by Sex, Province, District, Constituency and Ward, Zambia 2010

| Province,District,Constituency and Ward | 0 -14 Years | | | 15 - 34 Years | | | 0 - 34 Years | | |
|---|---|---|---|---|---|---|---|---|---|
| | Total | Male | Female | Total | Male | Female | Total | Male | Female |
| Mbabala | 7,359 | 3,678 | 3,681 | 4,762 | 2,308 | 2,454 | 12,121 | 5,986 | 6,135 |
| **Pemba Constituency** | **33,972** | **16,800** | **17,172** | **21,134** | **10,173** | **10,961** | **55,106** | **26,973** | **28,133** |
| Kasiya | 6,619 | 3,317 | 3,302 | 4,021 | 2,012 | 2,009 | 10,640 | 5,329 | 5,311 |
| Pemba | 974 | 456 | 518 | 939 | 459 | 480 | 1,913 | 915 | 998 |
| Hamaundu | 8,962 | 4,425 | 4,537 | 6,029 | 2,935 | 3,094 | 14,991 | 7,360 | 7,631 |
| Maambo | 6,790 | 3,382 | 3,408 | 3,706 | 1,753 | 1,953 | 10,496 | 5,135 | 5,361 |
| Kauba | 3,657 | 1,794 | 1,863 | 2,267 | 1,084 | 1,183 | 5,924 | 2,878 | 3,046 |
| Habunkululu | 2,223 | 1,077 | 1,146 | 1,355 | 638 | 717 | 3,578 | 1,715 | 1,863 |
| Nachibanga | 4,747 | 2,349 | 2,398 | 2,817 | 1,292 | 1,525 | 7,564 | 3,641 | 3,923 |
| **Gwembe District** | **26,381** | **13,181** | **13,200** | **17,551** | **8,426** | **9,125** | **43,932** | **21,607** | **22,325** |
| **Gwembe Constituency** | **26,381** | **13,181** | **13,200** | **17,551** | **8,426** | **9,125** | **43,932** | **21,607** | **22,325** |
| Chisanga | 1,560 | 781 | 779 | 838 | 406 | 432 | 2,398 | 1,187 | 1,211 |
| Sinafala | 1,049 | 546 | 503 | 633 | 299 | 334 | 1,682 | 845 | 837 |
| Jumbo | 1,248 | 644 | 604 | 802 | 380 | 422 | 2,050 | 1,024 | 1,026 |
| Kkoma | 2,789 | 1,366 | 1,423 | 2,007 | 990 | 1,017 | 4,796 | 2,356 | 2,440 |
| Chibuwe | 2,520 | 1,212 | 1,308 | 2,034 | 1,003 | 1,031 | 4,554 | 2,215 | 2,339 |
| Siampande | 773 | 425 | 348 | 439 | 213 | 226 | 1,212 | 638 | 574 |
| Kota Kota | 567 | 283 | 284 | 370 | 186 | 184 | 937 | 469 | 468 |
| Luumbo | 1,882 | 959 | 923 | 1,191 | 560 | 631 | 3,073 | 1,519 | 1,554 |
| Kkole | 1,411 | 696 | 715 | 861 | 406 | 455 | 2,272 | 1,102 | 1,170 |
| Bbondo | 4,478 | 2,190 | 2,288 | 2,734 | 1,327 | 1,407 | 7,212 | 3,517 | 3,695 |
| Chaamwe | 1,441 | 720 | 721 | 931 | 452 | 479 | 2,372 | 1,172 | 1,200 |
| Fumbo | 4,408 | 2,251 | 2,157 | 2,940 | 1,385 | 1,555 | 7,348 | 3,636 | 3,712 |
| Jongola | 354 | 178 | 176 | 196 | 84 | 112 | 550 | 262 | 288 |
| Lukonde | 1,901 | 930 | 971 | 1,575 | 735 | 840 | 3,476 | 1,665 | 1,811 |
| **Itezhi Tezhi District** | **33,396** | **16,666** | **16,730** | **22,809** | **11,068** | **11,741** | **56,205** | **27,734** | **28,471** |
| **Itezhi Tezhi Constituency** | **33,396** | **16,666** | **16,730** | **22,809** | **11,068** | **11,741** | **56,205** | **27,734** | **28,471** |
| Itezhi Tezhi | 3,485 | 1,654 | 1,831 | 3,367 | 1,599 | 1,768 | 6,852 | 3,253 | 3,599 |
| Itumbi | 2,890 | 1,434 | 1,456 | 1,756 | 837 | 919 | 4,646 | 2,271 | 2,375 |
| Kaanzwa | 3,236 | 1,609 | 1,627 | 1,936 | 927 | 1,009 | 5,172 | 2,536 | 2,636 |
| Banamwaze | 1,404 | 698 | 706 | 932 | 469 | 463 | 2,336 | 1,167 | 1,169 |
| Makunku | 1,845 | 955 | 890 | 1,198 | 564 | 634 | 3,043 | 1,519 | 1,524 |
| Nyambo | 1,250 | 657 | 593 | 818 | 404 | 414 | 2,068 | 1,061 | 1,007 |
| Kabulungwe | 806 | 409 | 397 | 505 | 259 | 246 | 1,311 | 668 | 643 |
| Lubanda | 3,156 | 1,589 | 1,567 | 2,307 | 1,170 | 1,137 | 5,463 | 2,759 | 2,704 |
| Masemu | 4,556 | 2,292 | 2,264 | 3,305 | 1,586 | 1,719 | 7,861 | 3,878 | 3,983 |
| Luubwe | 1,672 | 874 | 798 | 1,088 | 545 | 543 | 2,760 | 1,419 | 1,341 |
| Basanga | 3,589 | 1,756 | 1,833 | 2,403 | 1,162 | 1,241 | 5,992 | 2,918 | 3,074 |
| Luchena | 1,161 | 614 | 547 | 643 | 300 | 343 | 1,804 | 914 | 890 |
| Mbila | 4,346 | 2,125 | 2,221 | 2,551 | 1,246 | 1,305 | 6,897 | 3,371 | 3,526 |
| **Kalomo District** | **131,800** | **65,462** | **66,338** | **86,011** | **41,418** | **44,593** | **217,811** | **106,880** | **110,931** |
| **Dundumwezi Constituency** | **43,559** | **21,722** | **21,837** | **25,418** | **12,202** | **13,216** | **68,977** | **33,924** | **35,053** |
| Chikanta | 11,105 | 5,610 | 5,495 | 6,487 | 3,121 | 3,366 | 17,592 | 8,731 | 8,861 |
| Chamuka | 5,383 | 2,641 | 2,742 | 3,207 | 1,500 | 1,707 | 8,590 | 4,141 | 4,449 |
| Kasukwe | 8,171 | 4,116 | 4,055 | 4,889 | 2,297 | 2,592 | 13,060 | 6,413 | 6,647 |
| Omba | 6,014 | 2,964 | 3,050 | 3,287 | 1,570 | 1,717 | 9,301 | 4,534 | 4,767 |
| Bbilili | 5,490 | 2,736 | 2,754 | 3,140 | 1,534 | 1,606 | 8,630 | 4,270 | 4,360 |
| Naluja | 7,396 | 3,655 | 3,741 | 4,408 | 2,180 | 2,228 | 11,804 | 5,835 | 5,969 |
| **Kalomo Central Constituency** | **53,225** | **26,394** | **26,831** | **37,214** | **17,973** | **19,241** | **90,439** | **44,367** | **46,072** |
| Siachitema | 14,049 | 7,083 | 6,966 | 8,438 | 4,049 | 4,389 | 22,487 | 11,132 | 11,355 |
| Kalonda | 6,966 | 3,365 | 3,601 | 4,412 | 2,166 | 2,246 | 11,378 | 5,531 | 5,847 |
| Choonga | 9,076 | 4,371 | 4,705 | 8,976 | 4,279 | 4,697 | 18,052 | 8,650 | 9,402 |
| Mayoba | 5,630 | 2,811 | 2,819 | 3,720 | 1,835 | 1,885 | 9,350 | 4,646 | 4,704 |
| Namwianga | 4,578 | 2,320 | 2,258 | 3,558 | 1,685 | 1,873 | 8,136 | 4,005 | 4,131 |

## Table A11: Population (De Jure) by Age 0-14 Years (Children), 15-34 Years (Youths) and Total Population 0-34 Years by Sex, Province, District, Constituency and Ward, Zambia 2010

| Province,District,Constituency and Ward | 0 -14 Years | | | 15 - 34 Years | | | 0 - 34 Years | | |
|---|---|---|---|---|---|---|---|---|---|
| | Total | Male | Female | Total | Male | Female | Total | Male | Female |
| Simayakwe | 2,647 | 1,320 | 1,327 | 1,589 | 779 | 810 | 4,236 | 2,099 | 2,137 |
| Chawila | 4,142 | 2,033 | 2,109 | 2,522 | 1,240 | 1,282 | 6,664 | 3,273 | 3,391 |
| Sipatunyana | 1,905 | 990 | 915 | 1,234 | 613 | 621 | 3,139 | 1,603 | 1,536 |
| Nachikungu | 4,232 | 2,101 | 2,131 | 2,765 | 1,327 | 1,438 | 6,997 | 3,428 | 3,569 |
| **Mapatizya Constituency** | **35,016** | **17,346** | **17,670** | **23,379** | **11,243** | **12,136** | **58,395** | **28,589** | **29,806** |
| Chidi | 5,153 | 2,485 | 2,668 | 3,579 | 1,697 | 1,882 | 8,732 | 4,182 | 4,550 |
| Mulamfu | 3,458 | 1,718 | 1,740 | 2,038 | 940 | 1,098 | 5,496 | 2,658 | 2,838 |
| Simwatachela | 2,476 | 1,252 | 1,224 | 1,754 | 865 | 889 | 4,230 | 2,117 | 2,113 |
| Luyaba | 7,759 | 3,874 | 3,885 | 4,995 | 2,400 | 2,595 | 12,754 | 6,274 | 6,480 |
| Zimba | 6,192 | 3,042 | 3,150 | 4,522 | 2,205 | 2,317 | 10,714 | 5,247 | 5,467 |
| Siamafumba | 5,323 | 2,693 | 2,630 | 3,447 | 1,675 | 1,772 | 8,770 | 4,368 | 4,402 |
| Mbwiko | 4,655 | 2,282 | 2,373 | 3,044 | 1,461 | 1,583 | 7,699 | 3,743 | 3,956 |
| **Kazungula District** | **51,821** | **26,183** | **25,638** | **33,396** | **16,318** | **17,078** | **85,217** | **42,501** | **42,716** |
| **Katombola Constituency** | **51,821** | **26,183** | **25,638** | **33,396** | **16,318** | **17,078** | **85,217** | **42,501** | **42,716** |
| Moomba | 1,161 | 603 | 558 | 734 | 366 | 368 | 1,895 | 969 | 926 |
| Chooma | 3,973 | 2,017 | 1,956 | 2,240 | 1,049 | 1,191 | 6,213 | 3,066 | 3,147 |
| Nguba | 6,275 | 3,119 | 3,156 | 3,493 | 1,651 | 1,842 | 9,768 | 4,770 | 4,998 |
| Kauwe | 3,425 | 1,795 | 1,630 | 2,020 | 1,021 | 999 | 5,445 | 2,816 | 2,629 |
| Nyawa | 5,749 | 2,883 | 2,866 | 3,397 | 1,621 | 1,776 | 9,146 | 4,504 | 4,642 |
| Ngwezi | 5,136 | 2,633 | 2,503 | 3,141 | 1,567 | 1,574 | 8,277 | 4,200 | 4,077 |
| Sikaunzwe | 3,474 | 1,765 | 1,709 | 2,478 | 1,251 | 1,227 | 5,952 | 3,016 | 2,936 |
| Mandia | 4,167 | 2,074 | 2,093 | 3,322 | 1,563 | 1,759 | 7,489 | 3,637 | 3,852 |
| Sekute | 1,459 | 742 | 717 | 1,106 | 570 | 536 | 2,565 | 1,312 | 1,253 |
| Kanchele | 5,442 | 2,670 | 2,772 | 3,253 | 1,587 | 1,666 | 8,695 | 4,257 | 4,438 |
| Simango | 2,327 | 1,201 | 1,126 | 1,535 | 770 | 765 | 3,862 | 1,971 | 1,891 |
| Musokotwane | 2,275 | 1,174 | 1,101 | 1,528 | 753 | 775 | 3,803 | 1,927 | 1,876 |
| Katapazi | 3,203 | 1,598 | 1,605 | 2,089 | 1,018 | 1,071 | 5,292 | 2,616 | 2,676 |
| Mukuni | 3,755 | 1,909 | 1,846 | 3,060 | 1,531 | 1,529 | 6,815 | 3,440 | 3,375 |
| **Livingstone District** | **52,486** | **25,519** | **26,967** | **60,420** | **29,087** | **31,333** | **112,906** | **54,606** | **58,300** |
| **Livingstone Constituency** | **52,486** | **25,519** | **26,967** | **60,420** | **29,087** | **31,333** | **112,906** | **54,606** | **58,300** |
| Freedom | 4,415 | 2,219 | 2,196 | 5,173 | 2,408 | 2,765 | 9,588 | 4,627 | 4,961 |
| Musi-oa-tunya | 2,523 | 1,169 | 1,354 | 3,932 | 1,827 | 2,105 | 6,455 | 2,996 | 3,459 |
| Dr. Mubitana | 2,315 | 1,114 | 1,201 | 2,923 | 1,444 | 1,479 | 5,238 | 2,558 | 2,680 |
| Namatama | 4,615 | 2,243 | 2,372 | 4,849 | 2,458 | 2,391 | 9,464 | 4,701 | 4,763 |
| Kasiya | 3,919 | 1,959 | 1,960 | 3,514 | 1,724 | 1,790 | 7,433 | 3,683 | 3,750 |
| Libuyu | 3,047 | 1,507 | 1,540 | 3,151 | 1,588 | 1,563 | 6,198 | 3,095 | 3,103 |
| Mwalibonena | 3,864 | 1,836 | 2,028 | 4,347 | 2,125 | 2,222 | 8,211 | 3,961 | 4,250 |
| Mulungushi | 3,169 | 1,523 | 1,646 | 3,458 | 1,661 | 1,797 | 6,627 | 3,184 | 3,443 |
| Maramba | 3,541 | 1,667 | 1,874 | 4,309 | 2,006 | 2,303 | 7,850 | 3,673 | 4,177 |
| Akapelwa | 914 | 415 | 499 | 1,495 | 679 | 816 | 2,409 | 1,094 | 1,315 |
| Lizuma | 901 | 453 | 448 | 1,287 | 574 | 713 | 2,188 | 1,027 | 1,161 |
| Simonga | 5,187 | 2,545 | 2,642 | 5,420 | 2,627 | 2,793 | 10,607 | 5,172 | 5,435 |
| Dambwa Central | 2,133 | 996 | 1,137 | 2,903 | 1,374 | 1,529 | 5,036 | 2,370 | 2,666 |
| Zambezi | 6,175 | 3,003 | 3,172 | 7,133 | 3,336 | 3,797 | 13,308 | 6,339 | 6,969 |
| Kariba | 1,591 | 812 | 779 | 2,064 | 989 | 1,075 | 3,655 | 1,801 | 1,854 |
| Nansanzu | 2,730 | 1,334 | 1,396 | 3,095 | 1,577 | 1,518 | 5,825 | 2,911 | 2,914 |
| Shungu | 1,447 | 724 | 723 | 1,367 | 690 | 677 | 2,814 | 1,414 | 1,400 |
| **Mazabuka District** | **103,535** | **51,580** | **51,955** | **86,278** | **42,231** | **44,047** | **189,813** | **93,811** | **96,002** |
| **Chikankanta Constituency** | **27,370** | **13,756** | **13,614** | **21,344** | **10,531** | **10,813** | **48,714** | **24,287** | **24,427** |
| Kasengo | 3,185 | 1,556 | 1,629 | 2,271 | 1,155 | 1,116 | 5,456 | 2,711 | 2,745 |
| Namalundu | 3,343 | 1,668 | 1,675 | 3,202 | 1,557 | 1,645 | 6,545 | 3,225 | 3,320 |
| Musaya | 516 | 251 | 265 | 406 | 216 | 190 | 922 | 467 | 455 |
| Nansenga | 1,253 | 656 | 597 | 938 | 457 | 481 | 2,191 | 1,113 | 1,078 |
| Chitete | 4,876 | 2,422 | 2,454 | 3,716 | 1,833 | 1,883 | 8,592 | 4,255 | 4,337 |

## Table A11: Population (De Jure) by Age 0-14 Years (Children), 15-34 Years (Youths) and Total Population 0-34 Years by Sex, Province, District, Constituency and Ward, Zambia 2010

| Province,District,Constituency and Ward | 0 -14 Years | | | 15 - 34 Years | | | 0 - 34 Years | | |
|---|---|---|---|---|---|---|---|---|---|
| | Total | Male | Female | Total | Male | Female | Total | Male | Female |
| Malala | 3,018 | 1,578 | 1,440 | 2,227 | 1,104 | 1,123 | 5,245 | 2,682 | 2,563 |
| Mabwe Atuba | 5,903 | 2,972 | 2,931 | 4,609 | 2,259 | 2,350 | 10,512 | 5,231 | 5,281 |
| Upper Kaleya | 5,276 | 2,653 | 2,623 | 3,975 | 1,950 | 2,025 | 9,251 | 4,603 | 4,648 |
| **Magoye Constituency** | **34,184** | **17,214** | **16,970** | **24,353** | **11,919** | **12,434** | **58,537** | **29,133** | **29,404** |
| Konkola | 3,107 | 1,577 | 1,530 | 2,138 | 1,048 | 1,090 | 5,245 | 2,625 | 2,620 |
| Chivuna | 9,656 | 4,805 | 4,851 | 6,572 | 3,153 | 3,419 | 16,228 | 7,958 | 8,270 |
| Musuma | 2,649 | 1,333 | 1,316 | 1,886 | 949 | 937 | 4,535 | 2,282 | 2,253 |
| Munjile | 1,208 | 632 | 576 | 826 | 425 | 401 | 2,034 | 1,057 | 977 |
| Ngwezi | 7,762 | 3,862 | 3,900 | 5,804 | 2,845 | 2,959 | 13,566 | 6,707 | 6,859 |
| Kalama | 1,579 | 810 | 769 | 1,169 | 576 | 593 | 2,748 | 1,386 | 1,362 |
| Munenga | 1,328 | 701 | 627 | 939 | 461 | 478 | 2,267 | 1,162 | 1,105 |
| Mwanachingwala | 5,565 | 2,828 | 2,737 | 4,092 | 1,981 | 2,111 | 9,657 | 4,809 | 4,848 |
| Itebe | 1,330 | 666 | 664 | 927 | 481 | 446 | 2,257 | 1,147 | 1,110 |
| **Mazabuka Central Constituency** | **41,981** | **20,610** | **21,371** | **40,581** | **19,781** | **20,800** | **82,562** | **40,391** | **42,171** |
| Mazabuka Central | 23,906 | 11,650 | 12,256 | 25,134 | 12,255 | 12,879 | 49,040 | 23,905 | 25,135 |
| Nakambala | 3,644 | 1,778 | 1,866 | 3,300 | 1,576 | 1,724 | 6,944 | 3,354 | 3,590 |
| Chizobo | 2,895 | 1,424 | 1,471 | 2,260 | 1,092 | 1,168 | 5,155 | 2,516 | 2,639 |
| Lubombo | 9,282 | 4,567 | 4,715 | 8,198 | 4,031 | 4,167 | 17,480 | 8,598 | 8,882 |
| Nega Nega | 2,254 | 1,191 | 1,063 | 1,689 | 827 | 862 | 3,943 | 2,018 | 1,925 |
| **Monze District** | **93,150** | **46,945** | **46,205** | **64,620** | **31,336** | **33,284** | **157,770** | **78,281** | **79,489** |
| **Bweengwa Constituency** | **30,847** | **15,469** | **15,378** | **18,968** | **9,165** | **9,803** | **49,815** | **24,634** | **25,181** |
| Malundu | 4,087 | 2,042 | 2,045 | 2,599 | 1,288 | 1,311 | 6,686 | 3,330 | 3,356 |
| Kaila | 3,934 | 1,899 | 2,035 | 2,274 | 1,088 | 1,186 | 6,208 | 2,987 | 3,221 |
| Keemba | 6,354 | 3,242 | 3,112 | 3,887 | 1,876 | 2,011 | 10,241 | 5,118 | 5,123 |
| Choongo West | 1,790 | 883 | 907 | 1,130 | 525 | 605 | 2,920 | 1,408 | 1,512 |
| Bweengwa | 3,885 | 1,940 | 1,945 | 2,252 | 1,083 | 1,169 | 6,137 | 3,023 | 3,114 |
| Hamangaba | 4,593 | 2,304 | 2,289 | 2,485 | 1,209 | 1,276 | 7,078 | 3,513 | 3,565 |
| Choongo East | 6,204 | 3,159 | 3,045 | 4,341 | 2,096 | 2,245 | 10,545 | 5,255 | 5,290 |
| **Monze Central Constituency** | **47,118** | **23,789** | **23,329** | **36,178** | **17,521** | **18,657** | **83,296** | **41,310** | **41,986** |
| Chipembele | 3,505 | 1,807 | 1,698 | 2,262 | 1,108 | 1,154 | 5,767 | 2,915 | 2,852 |
| Ufwenuka | 4,516 | 2,302 | 2,214 | 3,259 | 1,581 | 1,678 | 7,775 | 3,883 | 3,892 |
| Manungu | 15,891 | 7,880 | 8,011 | 16,087 | 7,668 | 8,419 | 31,978 | 15,548 | 16,430 |
| Chisekesi | 3,279 | 1,649 | 1,630 | 2,480 | 1,215 | 1,265 | 5,759 | 2,864 | 2,895 |
| Mayaba | 2,232 | 1,104 | 1,128 | 1,558 | 734 | 824 | 3,790 | 1,838 | 1,952 |
| Hufwa/Hamapande | 1,829 | 954 | 875 | 1,058 | 505 | 553 | 2,887 | 1,459 | 1,428 |
| Hamamvwa | 2,257 | 1,182 | 1,075 | 1,305 | 621 | 684 | 3,562 | 1,803 | 1,759 |
| Katimba | 3,723 | 1,857 | 1,866 | 2,159 | 1,057 | 1,102 | 5,882 | 2,914 | 2,968 |
| Hatontola | 6,223 | 3,184 | 3,039 | 3,697 | 1,847 | 1,850 | 9,920 | 5,031 | 4,889 |
| Bbombo | 2,968 | 1,491 | 1,477 | 1,785 | 905 | 880 | 4,753 | 2,396 | 2,357 |
| Monze Urban | 695 | 379 | 316 | 528 | 280 | 248 | 1,223 | 659 | 564 |
| **Moomba Constituency** | **15,185** | **7,687** | **7,498** | **9,474** | **4,650** | **4,824** | **24,659** | **12,337** | **12,322** |
| Mwanza West | 6,490 | 3,281 | 3,209 | 4,206 | 2,070 | 2,136 | 10,696 | 5,351 | 5,345 |
| Chona | 4,921 | 2,479 | 2,442 | 3,088 | 1,544 | 1,544 | 8,009 | 4,023 | 3,986 |
| Mwanza East | 3,233 | 1,657 | 1,576 | 1,880 | 893 | 987 | 5,113 | 2,550 | 2,563 |
| Moomba | 541 | 270 | 271 | 300 | 143 | 157 | 841 | 413 | 428 |
| **Namwala District** | **52,277** | **26,043** | **26,234** | **33,859** | **16,238** | **17,621** | **86,136** | **42,281** | **43,855** |
| **Namwala Constituency** | **52,277** | **26,043** | **26,234** | **33,859** | **16,238** | **17,621** | **86,136** | **42,281** | **43,855** |
| Namwala Central | 4,236 | 2,040 | 2,196 | 4,077 | 1,974 | 2,103 | 8,313 | 4,014 | 4,299 |
| Ngabo | 1,382 | 719 | 663 | 893 | 466 | 427 | 2,275 | 1,185 | 1,090 |
| Baambwe | 1,946 | 992 | 954 | 1,426 | 731 | 695 | 3,372 | 1,723 | 1,649 |
| Maala | 2,664 | 1,308 | 1,356 | 2,127 | 982 | 1,145 | 4,791 | 2,290 | 2,501 |
| Kantengwa | 2,198 | 1,108 | 1,090 | 1,577 | 794 | 783 | 3,775 | 1,902 | 1,873 |
| Kabulamwanda | 3,630 | 1,856 | 1,774 | 2,440 | 1,169 | 1,271 | 6,070 | 3,025 | 3,045 |
| Chitongo | 2,538 | 1,258 | 1,280 | 1,573 | 715 | 858 | 4,111 | 1,973 | 2,138 |

## Table A11: Population (De Jure) by Age 0-14 Years (Children), 15-34 Years (Youths) and Total Population 0-34 Years by Sex, Province, District, Constituency and Ward, Zambia 2010

| Province,District,Constituency and Ward | 0 -14 Years | | | 15 - 34 Years | | | 0 - 34 Years | | |
|---|---|---|---|---|---|---|---|---|---|
| | Total | Male | Female | Total | Male | Female | Total | Male | Female |
| Mandondo | 2,342 | 1,162 | 1,180 | 1,412 | 680 | 732 | 3,754 | 1,842 | 1,912 |
| Nakamboma | 7,037 | 3,482 | 3,555 | 4,255 | 2,073 | 2,182 | 11,292 | 5,555 | 5,737 |
| Mbeza | 2,637 | 1,286 | 1,351 | 1,653 | 808 | 845 | 4,290 | 2,094 | 2,196 |
| Ndema | 5,052 | 2,507 | 2,545 | 2,853 | 1,330 | 1,523 | 7,905 | 3,837 | 4,068 |
| Namakube | 6,019 | 3,001 | 3,018 | 3,357 | 1,582 | 1,775 | 9,376 | 4,583 | 4,793 |
| Itapa | 3,946 | 1,974 | 1,972 | 2,174 | 1,015 | 1,159 | 6,120 | 2,989 | 3,131 |
| Moobola | 6,650 | 3,350 | 3,300 | 4,042 | 1,919 | 2,123 | 10,692 | 5,269 | 5,423 |
| **Siavonga District** | **42,423** | **21,174** | **21,249** | **31,275** | **15,267** | **16,008** | **73,698** | **36,441** | **37,257** |
| **Siavonga Constituency** | **42,423** | **21,174** | **21,249** | **31,275** | **15,267** | **16,008** | **73,698** | **36,441** | **37,257** |
| Ibwemunyama | 1,669 | 819 | 850 | 1,072 | 535 | 537 | 2,741 | 1,354 | 1,387 |
| Musaya | 2,476 | 1,263 | 1,213 | 1,793 | 891 | 902 | 4,269 | 2,154 | 2,115 |
| Chirundu | 6,568 | 3,094 | 3,474 | 5,952 | 2,754 | 3,198 | 12,520 | 5,848 | 6,672 |
| Ng'ombe Ilede | 5,860 | 3,011 | 2,849 | 3,648 | 1,770 | 1,878 | 9,508 | 4,781 | 4,727 |
| Sikoongo | 1,956 | 985 | 971 | 1,152 | 577 | 575 | 3,108 | 1,562 | 1,546 |
| Lusitu | 3,868 | 1,995 | 1,873 | 2,613 | 1,325 | 1,288 | 6,481 | 3,320 | 3,161 |
| Nanyanga | 1,221 | 619 | 602 | 846 | 424 | 422 | 2,067 | 1,043 | 1,024 |
| Kariba | 6,967 | 3,371 | 3,596 | 6,787 | 3,295 | 3,492 | 13,754 | 6,666 | 7,088 |
| Simamba | 2,879 | 1,458 | 1,421 | 2,066 | 1,035 | 1,031 | 4,945 | 2,493 | 2,452 |
| Mulimya | 2,704 | 1,401 | 1,303 | 1,645 | 825 | 820 | 4,349 | 2,226 | 2,123 |
| Manchamvwa | 2,360 | 1,187 | 1,173 | 1,515 | 763 | 752 | 3,875 | 1,950 | 1,925 |
| Sinadambwe | 2,238 | 1,148 | 1,090 | 1,282 | 636 | 646 | 3,520 | 1,784 | 1,736 |
| Lusangazi | 1,657 | 823 | 834 | 904 | 437 | 467 | 2,561 | 1,260 | 1,301 |
| **Sinazongwe District** | **48,210** | **23,803** | **24,407** | **35,413** | **16,915** | **18,498** | **83,623** | **40,718** | **42,905** |
| **Sinazongwe Constituency** | **48,210** | **23,803** | **24,407** | **35,413** | **16,915** | **18,498** | **83,623** | **40,718** | **42,905** |
| Mabinga | 386 | 186 | 200 | 259 | 118 | 141 | 645 | 304 | 341 |
| Namazambwe | 2,594 | 1,298 | 1,296 | 1,704 | 825 | 879 | 4,298 | 2,123 | 2,175 |
| Mweenda | 2,677 | 1,316 | 1,361 | 1,692 | 804 | 888 | 4,369 | 2,120 | 2,249 |
| Muuka | 2,394 | 1,228 | 1,166 | 1,511 | 737 | 774 | 3,905 | 1,965 | 1,940 |
| Tekelo | 979 | 472 | 507 | 573 | 284 | 289 | 1,552 | 756 | 796 |
| Mweemba | 5,230 | 2,603 | 2,627 | 3,971 | 1,935 | 2,036 | 9,201 | 4,538 | 4,663 |
| Muchekwa | 3,303 | 1,680 | 1,623 | 2,257 | 1,051 | 1,206 | 5,560 | 2,731 | 2,829 |
| Maamba | 4,138 | 1,978 | 2,160 | 4,234 | 1,991 | 2,243 | 8,372 | 3,969 | 4,403 |
| Mweezya | 8,017 | 3,906 | 4,111 | 6,086 | 2,923 | 3,163 | 14,103 | 6,829 | 7,274 |
| Nkamdabwe | 3,038 | 1,459 | 1,579 | 2,271 | 1,078 | 1,193 | 5,309 | 2,537 | 2,772 |
| Sinazongwe | 5,176 | 2,576 | 2,600 | 3,779 | 1,775 | 2,004 | 8,955 | 4,351 | 4,604 |
| Nang'ombe | 3,068 | 1,486 | 1,582 | 2,187 | 1,047 | 1,140 | 5,255 | 2,533 | 2,722 |
| Sinenge | 4,121 | 2,055 | 2,066 | 2,816 | 1,351 | 1,465 | 6,937 | 3,406 | 3,531 |
| Malima | 3,089 | 1,560 | 1,529 | 2,073 | 996 | 1,077 | 5,162 | 2,556 | 2,606 |
| **Western Province** | **420,229** | **210,543** | **209,686** | **297,401** | **138,447** | **158,954** | **717,630** | **348,990** | **368,640** |
| **Kalabo District** | **60,274** | **30,072** | **30,202** | **41,280** | **18,563** | **22,717** | **101,554** | **48,635** | **52,919** |
| **Kalabo Central Constituency** | **26,263** | **13,111** | **13,152** | **18,543** | **8,528** | **10,015** | **44,806** | **21,639** | **23,167** |
| Mapungu | 1,699 | 849 | 850 | 1,204 | 570 | 634 | 2,903 | 1,419 | 1,484 |
| Luanginga | 3,617 | 1,789 | 1,828 | 3,474 | 1,713 | 1,761 | 7,091 | 3,502 | 3,589 |
| Liumba | 1,751 | 893 | 858 | 1,163 | 531 | 632 | 2,914 | 1,424 | 1,490 |
| Yuka | 3,251 | 1,651 | 1,600 | 2,173 | 947 | 1,226 | 5,424 | 2,598 | 2,826 |
| Buleya | 1,866 | 949 | 917 | 1,313 | 578 | 735 | 3,179 | 1,527 | 1,652 |
| Lutwi | 2,797 | 1,403 | 1,394 | 1,663 | 722 | 941 | 4,460 | 2,125 | 2,335 |
| Ndoka | 3,610 | 1,784 | 1,826 | 2,445 | 1,079 | 1,366 | 6,055 | 2,863 | 3,192 |
| Namulilo | 3,670 | 1,800 | 1,870 | 2,605 | 1,238 | 1,367 | 6,275 | 3,038 | 3,237 |
| Nguma | 2,129 | 1,077 | 1,052 | 1,305 | 595 | 710 | 3,434 | 1,672 | 1,762 |
| Kandambo | 1,873 | 916 | 957 | 1,198 | 555 | 643 | 3,071 | 1,471 | 1,600 |

## Table A11: Population (De Jure) by Age 0-14 Years (Children), 15-34 Years (Youths) and Total Population 0-34 Years by Sex, Province, District, Constituency and Ward, Zambia 2010

| Province,District,Constituency and Ward | 0 -14 Years | | | 15 - 34 Years | | | 0 - 34 Years | | |
|---|---|---|---|---|---|---|---|---|---|
| | Total | Male | Female | Total | Male | Female | Total | Male | Female |
| **Liuwa Constituency** | **12,384** | **6,216** | **6,168** | **8,415** | **3,928** | **4,487** | **20,799** | **10,144** | **10,655** |
| Siluwe | 981 | 491 | 490 | 698 | 319 | 379 | 1,679 | 810 | 869 |
| Likulundundu | 1,104 | 555 | 549 | 721 | 325 | 396 | 1,825 | 880 | 945 |
| Luola | 818 | 400 | 418 | 575 | 254 | 321 | 1,393 | 654 | 739 |
| Salunda | 1,416 | 711 | 705 | 907 | 407 | 500 | 2,323 | 1,118 | 1,205 |
| Sishekanu | 2,648 | 1,291 | 1,357 | 1,738 | 793 | 945 | 4,386 | 2,084 | 2,302 |
| Kuuli | 1,870 | 956 | 914 | 1,205 | 571 | 634 | 3,075 | 1,527 | 1,548 |
| Libonda | 3,547 | 1,812 | 1,735 | 2,571 | 1,259 | 1,312 | 6,118 | 3,071 | 3,047 |
| **Sikongo Constituency** | **21,627** | **10,745** | **10,882** | **14,322** | **6,107** | **8,215** | **35,949** | **16,852** | **19,097** |
| Lueti | 4,280 | 2,124 | 2,156 | 2,865 | 1,250 | 1,615 | 7,145 | 3,374 | 3,771 |
| Lulan'gunyi | 1,113 | 547 | 566 | 763 | 323 | 440 | 1,876 | 870 | 1,006 |
| Tuuwa | 2,221 | 1,112 | 1,109 | 1,465 | 583 | 882 | 3,686 | 1,695 | 1,991 |
| Maala | 2,752 | 1,403 | 1,349 | 1,787 | 767 | 1,020 | 4,539 | 2,170 | 2,369 |
| Liumena | 3,229 | 1,611 | 1,618 | 1,993 | 839 | 1,154 | 5,222 | 2,450 | 2,772 |
| Licha | 3,850 | 1,906 | 1,944 | 2,590 | 1,064 | 1,526 | 6,440 | 2,970 | 3,470 |
| Lwambi | 822 | 405 | 417 | 521 | 241 | 280 | 1,343 | 646 | 697 |
| Nengu | 1,105 | 530 | 575 | 740 | 319 | 421 | 1,845 | 849 | 996 |
| Mutala | 699 | 359 | 340 | 468 | 223 | 245 | 1,167 | 582 | 585 |
| Mwenyi | 1,556 | 748 | 808 | 1,130 | 498 | 632 | 2,686 | 1,246 | 1,440 |
| **Kaoma District** | **88,044** | **44,137** | **43,907** | **61,868** | **28,980** | **32,888** | **149,912** | **73,117** | **76,795** |
| **Kaoma Central Constituency** | **39,198** | **19,890** | **19,308** | **29,961** | **14,606** | **15,355** | **69,159** | **34,496** | **34,663** |
| Lalafuta | 3,597 | 1,844 | 1,753 | 2,327 | 1,150 | 1,177 | 5,924 | 2,994 | 2,930 |
| Shitwa | 4,054 | 2,076 | 1,978 | 2,684 | 1,307 | 1,377 | 6,738 | 3,383 | 3,355 |
| Namilangi | 9,716 | 4,941 | 4,775 | 6,822 | 3,282 | 3,540 | 16,538 | 8,223 | 8,315 |
| Nkeyema | 5,445 | 2,739 | 2,706 | 4,126 | 2,095 | 2,031 | 9,571 | 4,834 | 4,737 |
| Litoya | 4,032 | 2,101 | 1,931 | 3,098 | 1,637 | 1,461 | 7,130 | 3,738 | 3,392 |
| Mulamatila | 8,929 | 4,404 | 4,525 | 8,578 | 3,993 | 4,585 | 17,507 | 8,397 | 9,110 |
| Longe | 3,425 | 1,785 | 1,640 | 2,326 | 1,142 | 1,184 | 5,751 | 2,927 | 2,824 |
| **Luampa Constituency** | **21,035** | **10,449** | **10,586** | **13,790** | **6,216** | **7,574** | **34,825** | **16,665** | **18,160** |
| Naliele | 3,210 | 1,616 | 1,594 | 2,300 | 1,054 | 1,246 | 5,510 | 2,670 | 2,840 |
| Namando | 585 | 291 | 294 | 383 | 176 | 207 | 968 | 467 | 501 |
| Nyambi | 3,102 | 1,572 | 1,530 | 1,870 | 885 | 985 | 4,972 | 2,457 | 2,515 |
| Mulwa | 2,119 | 1,066 | 1,053 | 1,262 | 558 | 704 | 3,381 | 1,624 | 1,757 |
| Lui | 3,708 | 1,810 | 1,898 | 2,495 | 1,120 | 1,375 | 6,203 | 2,930 | 3,273 |
| Nkenga | 4,171 | 2,062 | 2,109 | 2,847 | 1,241 | 1,606 | 7,018 | 3,303 | 3,715 |
| Mbanyutu | 978 | 463 | 515 | 649 | 281 | 368 | 1,627 | 744 | 883 |
| Luampa | 3,162 | 1,569 | 1,593 | 1,984 | 901 | 1,083 | 5,146 | 2,470 | 2,676 |
| **Mangango Constituency** | **27,811** | **13,798** | **14,013** | **18,117** | **8,158** | **9,959** | **45,928** | **21,956** | **23,972** |
| Namafulo | 7,873 | 3,901 | 3,972 | 4,911 | 2,165 | 2,746 | 12,784 | 6,066 | 6,718 |
| Luambuwa | 3,752 | 1,861 | 1,891 | 2,411 | 1,052 | 1,359 | 6,163 | 2,913 | 3,250 |
| Mushwala | 8,072 | 3,942 | 4,130 | 5,053 | 2,231 | 2,822 | 13,125 | 6,173 | 6,952 |
| Kapili | 1,063 | 542 | 521 | 699 | 341 | 358 | 1,762 | 883 | 879 |
| Mangango | 2,291 | 1,150 | 1,141 | 1,906 | 884 | 1,022 | 4,197 | 2,034 | 2,163 |
| Kanabilumbu | 1,548 | 776 | 772 | 927 | 438 | 489 | 2,475 | 1,214 | 1,261 |
| Shikombwe | 3,212 | 1,626 | 1,586 | 2,210 | 1,047 | 1,163 | 5,422 | 2,673 | 2,749 |
| **Lukulu District** | **42,145** | **21,297** | **20,848** | **27,132** | **12,457** | **14,675** | **69,277** | **33,754** | **35,523** |
| **Lukulu East Constituency** | **28,413** | **14,481** | **13,932** | **18,492** | **8,519** | **9,973** | **46,905** | **23,000** | **23,905** |
| Simakumba | 2,485 | 1,268 | 1,217 | 1,372 | 630 | 742 | 3,857 | 1,898 | 1,959 |
| Kamilende | 1,333 | 693 | 640 | 863 | 427 | 436 | 2,196 | 1,120 | 1,076 |
| Dongwe | 1,268 | 641 | 627 | 741 | 355 | 386 | 2,009 | 996 | 1,013 |
| Kashamba | 1,242 | 613 | 629 | 847 | 412 | 435 | 2,089 | 1,025 | 1,064 |
| Mwito | 1,975 | 969 | 1,006 | 1,215 | 540 | 675 | 3,190 | 1,509 | 1,681 |
| Kang'oti | 3,303 | 1,663 | 1,640 | 1,953 | 880 | 1,073 | 5,256 | 2,543 | 2,713 |
| Lukau | 1,877 | 958 | 919 | 1,008 | 451 | 557 | 2,885 | 1,409 | 1,476 |

## Table A11: Population (De Jure) by Age 0-14 Years (Children), 15-34 Years (Youths) and Total Population 0-34 Years by Sex, Province, District, Constituency and Ward, Zambia 2010

| Province,District,Constituency and Ward | 0 -14 Years | | | 15 - 34 Years | | | 0 - 34 Years | | |
|---|---|---|---|---|---|---|---|---|---|
| | Total | Male | Female | Total | Male | Female | Total | Male | Female |
| Likapai | 827 | 434 | 393 | 471 | 227 | 244 | 1,298 | 661 | 637 |
| Mbanga | 2,422 | 1,239 | 1,183 | 1,590 | 762 | 828 | 4,012 | 2,001 | 2,011 |
| Kawaya | 1,957 | 1,034 | 923 | 1,232 | 550 | 682 | 3,189 | 1,584 | 1,605 |
| Mwandi | 4,656 | 2,344 | 2,312 | 3,935 | 1,790 | 2,145 | 8,591 | 4,134 | 4,457 |
| Namayula | 1,902 | 998 | 904 | 1,170 | 528 | 642 | 3,072 | 1,526 | 1,546 |
| Luanchuma | 3,166 | 1,627 | 1,539 | 2,095 | 967 | 1,128 | 5,261 | 2,594 | 2,667 |
| **Lukulu West Constituency** | **13,732** | **6,816** | **6,916** | **8,640** | **3,938** | **4,702** | **22,372** | **10,754** | **11,618** |
| Muyondoti | 1,450 | 716 | 734 | 961 | 458 | 503 | 2,411 | 1,174 | 1,237 |
| Nyaala | 1,604 | 816 | 788 | 1,057 | 495 | 562 | 2,661 | 1,311 | 1,350 |
| Mataba | 1,801 | 860 | 941 | 1,165 | 543 | 622 | 2,966 | 1,403 | 1,563 |
| Lupui | 1,378 | 689 | 689 | 877 | 386 | 491 | 2,255 | 1,075 | 1,180 |
| Kakwacha | 843 | 411 | 432 | 528 | 229 | 299 | 1,371 | 640 | 731 |
| Lutembwe | 520 | 277 | 243 | 296 | 133 | 163 | 816 | 410 | 406 |
| Mitete | 1,590 | 792 | 798 | 1,067 | 455 | 612 | 2,657 | 1,247 | 1,410 |
| Kashizhi | 3,306 | 1,640 | 1,666 | 1,980 | 934 | 1,046 | 5,286 | 2,574 | 2,712 |
| Chin'onwe | 1,240 | 615 | 625 | 709 | 305 | 404 | 1,949 | 920 | 1,029 |
| **Mongu District** | **79,694** | **39,857** | **39,837** | **61,908** | **28,951** | **32,957** | **141,602** | **68,808** | **72,794** |
| **Luena Constituency** | **23,561** | **11,855** | **11,706** | **16,065** | **7,563** | **8,502** | **39,626** | **19,418** | **20,208** |
| Limulunga | 6,041 | 3,006 | 3,035 | 4,837 | 2,262 | 2,575 | 10,878 | 5,268 | 5,610 |
| Mabili | 1,910 | 998 | 912 | 1,215 | 610 | 605 | 3,125 | 1,608 | 1,517 |
| Ikwichi | 1,615 | 810 | 805 | 981 | 461 | 520 | 2,596 | 1,271 | 1,325 |
| Namboma | 2,393 | 1,204 | 1,189 | 1,589 | 760 | 829 | 3,982 | 1,964 | 2,018 |
| Nangula | 5,807 | 2,952 | 2,855 | 3,773 | 1,766 | 2,007 | 9,580 | 4,718 | 4,862 |
| Ushaa | 2,587 | 1,284 | 1,303 | 1,649 | 781 | 868 | 4,236 | 2,065 | 2,171 |
| Simaa | 2,470 | 1,240 | 1,230 | 1,539 | 713 | 826 | 4,009 | 1,953 | 2,056 |
| Ndanda | 738 | 361 | 377 | 482 | 210 | 272 | 1,220 | 571 | 649 |
| **Mongu Central Constituency** | **36,825** | **18,242** | **18,583** | **33,509** | **15,740** | **17,769** | **70,334** | **33,982** | **36,352** |
| Namushakende | 2,377 | 1,186 | 1,191 | 1,748 | 793 | 955 | 4,125 | 1,979 | 2,146 |
| Yeta | 2,914 | 1,454 | 1,460 | 2,195 | 1,061 | 1,134 | 5,109 | 2,515 | 2,594 |
| Kama | 1,301 | 668 | 633 | 959 | 467 | 492 | 2,260 | 1,135 | 1,125 |
| Lumbo | 2,167 | 1,050 | 1,117 | 1,441 | 674 | 767 | 3,608 | 1,724 | 1,884 |
| Katongo | 4,186 | 1,990 | 2,196 | 3,508 | 1,605 | 1,903 | 7,694 | 3,595 | 4,099 |
| Kanyonyo | 4,567 | 2,339 | 2,228 | 4,482 | 2,072 | 2,410 | 9,049 | 4,411 | 4,638 |
| Kambule | 3,714 | 1,811 | 1,903 | 4,025 | 1,888 | 2,137 | 7,739 | 3,699 | 4,040 |
| Lewanika | 1,128 | 561 | 567 | 1,280 | 613 | 667 | 2,408 | 1,174 | 1,234 |
| Mulambwa | 3,848 | 1,856 | 1,992 | 4,049 | 1,882 | 2,167 | 7,897 | 3,738 | 4,159 |
| Imwiko | 6,729 | 3,276 | 3,453 | 6,881 | 3,234 | 3,647 | 13,610 | 6,510 | 7,100 |
| Lealui | 2,157 | 1,113 | 1,044 | 1,665 | 821 | 844 | 3,822 | 1,934 | 1,888 |
| Mabumbu | 964 | 526 | 438 | 693 | 336 | 357 | 1,657 | 862 | 795 |
| Kaande | 773 | 412 | 361 | 583 | 294 | 289 | 1,356 | 706 | 650 |
| **Nalikwanda Constituency** | **19,308** | **9,760** | **9,548** | **12,334** | **5,648** | **6,686** | **31,642** | **15,408** | **16,234** |
| Lui | 4,984 | 2,462 | 2,522 | 3,255 | 1,453 | 1,802 | 8,239 | 3,915 | 4,324 |
| Imalyo | 3,011 | 1,512 | 1,499 | 1,964 | 898 | 1,066 | 4,975 | 2,410 | 2,565 |
| Mutondo | 1,751 | 938 | 813 | 985 | 456 | 529 | 2,736 | 1,394 | 1,342 |
| Namengo | 1,576 | 797 | 779 | 1,030 | 467 | 563 | 2,606 | 1,264 | 1,342 |
| Nakanyaa | 3,282 | 1,707 | 1,575 | 2,052 | 939 | 1,113 | 5,334 | 2,646 | 2,688 |
| Mbekise | 1,960 | 994 | 966 | 1,242 | 587 | 655 | 3,202 | 1,581 | 1,621 |
| Nakato | 2,744 | 1,350 | 1,394 | 1,806 | 848 | 958 | 4,550 | 2,198 | 2,352 |
| **Senanga District** | **59,009** | **29,584** | **29,425** | **41,369** | **19,177** | **22,192** | **100,378** | **48,761** | **51,617** |
| **Nalolo Constituency** | **26,046** | **13,134** | **12,912** | **17,694** | **8,256** | **9,438** | **43,740** | **21,390** | **22,350** |
| Lyamakumba | 5,703 | 2,836 | 2,867 | 3,732 | 1,735 | 1,997 | 9,435 | 4,571 | 4,864 |
| Silowana | 3,300 | 1,671 | 1,629 | 2,179 | 1,022 | 1,157 | 5,479 | 2,693 | 2,786 |
| Shekela | 3,477 | 1,815 | 1,662 | 2,365 | 1,130 | 1,235 | 5,842 | 2,945 | 2,897 |
| Makoka | 3,123 | 1,577 | 1,546 | 2,044 | 903 | 1,141 | 5,167 | 2,480 | 2,687 |

## Table A11: Population (De Jure) by Age 0-14 Years (Children), 15-34 Years (Youths) and Total Population 0-34 Years by Sex, Province, District, Constituency and Ward, Zambia 2010

| Province,District,Constituency and Ward | 0 -14 Years | | | 15 - 34 Years | | | 0 - 34 Years | | |
|---|---|---|---|---|---|---|---|---|---|
| | Total | Male | Female | Total | Male | Female | Total | Male | Female |
| Kambai | 3,145 | 1,581 | 1,564 | 2,103 | 970 | 1,133 | 5,248 | 2,551 | 2,697 |
| Kataba | 2,151 | 1,098 | 1,053 | 1,396 | 656 | 740 | 3,547 | 1,754 | 1,793 |
| Muoyo | 2,130 | 1,041 | 1,089 | 1,687 | 823 | 864 | 3,817 | 1,864 | 1,953 |
| Nanjucha | 3,017 | 1,515 | 1,502 | 2,188 | 1,017 | 1,171 | 5,205 | 2,532 | 2,673 |
| **Senanga Constituency** | **32,963** | **16,450** | **16,513** | **23,675** | **10,921** | **12,754** | **56,638** | **27,371** | **29,267** |
| Mwanambuyu | 5,412 | 2,683 | 2,729 | 3,518 | 1,638 | 1,880 | 8,930 | 4,321 | 4,609 |
| Imatongo | 3,344 | 1,686 | 1,658 | 2,502 | 1,160 | 1,342 | 5,846 | 2,846 | 3,000 |
| Imatanda | 7,108 | 3,552 | 3,556 | 6,910 | 3,201 | 3,709 | 14,018 | 6,753 | 7,265 |
| Wanyau | 2,317 | 1,125 | 1,192 | 1,520 | 703 | 817 | 3,837 | 1,828 | 2,009 |
| Lipuwe | 4,447 | 2,177 | 2,270 | 2,829 | 1,256 | 1,573 | 7,276 | 3,433 | 3,843 |
| Naluywa | 3,088 | 1,599 | 1,489 | 1,878 | 907 | 971 | 4,966 | 2,506 | 2,460 |
| Lumbe | 2,054 | 1,036 | 1,018 | 1,317 | 594 | 723 | 3,371 | 1,630 | 1,741 |
| Sibukali | 2,504 | 1,241 | 1,263 | 1,544 | 710 | 834 | 4,048 | 1,951 | 2,097 |
| Mata | 2,689 | 1,351 | 1,338 | 1,657 | 752 | 905 | 4,346 | 2,103 | 2,243 |
| **Sesheke District** | **45,594** | **22,785** | **22,809** | **33,393** | **16,315** | **17,078** | **78,987** | **39,100** | **39,887** |
| **Mulobezi Constituency** | **14,507** | **7,183** | **7,324** | **9,629** | **4,686** | **4,943** | **24,136** | **11,869** | **12,267** |
| Nawinda | 2,555 | 1,303 | 1,252 | 1,673 | 823 | 850 | 4,228 | 2,126 | 2,102 |
| Kamanga | 1,810 | 853 | 957 | 1,016 | 475 | 541 | 2,826 | 1,328 | 1,498 |
| Luamuloba | 1,929 | 983 | 946 | 1,253 | 633 | 620 | 3,182 | 1,616 | 1,566 |
| Sichili | 4,610 | 2,300 | 2,310 | 3,064 | 1,452 | 1,612 | 7,674 | 3,752 | 3,922 |
| Mulobezi | 1,930 | 954 | 976 | 1,527 | 769 | 758 | 3,457 | 1,723 | 1,734 |
| Machile | 1,673 | 790 | 883 | 1,096 | 534 | 562 | 2,769 | 1,324 | 1,445 |
| **Mwandi Constituency** | **11,645** | **5,884** | **5,761** | **8,118** | **3,939** | **4,179** | **19,763** | **9,823** | **9,940** |
| Magumwi | 1,539 | 788 | 751 | 994 | 486 | 508 | 2,533 | 1,274 | 1,259 |
| Sankolonga | 760 | 387 | 373 | 505 | 256 | 249 | 1,265 | 643 | 622 |
| Mabumbu | 1,791 | 928 | 863 | 1,255 | 622 | 633 | 3,046 | 1,550 | 1,496 |
| Mwandi | 1,782 | 879 | 903 | 1,666 | 794 | 872 | 3,448 | 1,673 | 1,775 |
| Simungoma | 502 | 257 | 245 | 295 | 135 | 160 | 797 | 392 | 405 |
| Loanja | 1,455 | 683 | 772 | 955 | 479 | 476 | 2,410 | 1,162 | 1,248 |
| Lwazamba | 2,208 | 1,129 | 1,079 | 1,428 | 666 | 762 | 3,636 | 1,795 | 1,841 |
| Mushukula | 1,608 | 833 | 775 | 1,020 | 501 | 519 | 2,628 | 1,334 | 1,294 |
| **Sesheke Constituency** | **19,442** | **9,718** | **9,724** | **15,646** | **7,690** | **7,956** | **35,088** | **17,408** | **17,680** |
| Maondo | 3,858 | 1,926 | 1,932 | 2,795 | 1,402 | 1,393 | 6,653 | 3,328 | 3,325 |
| Mulimambango | 8,263 | 4,114 | 4,149 | 7,824 | 3,796 | 4,028 | 16,087 | 7,910 | 8,177 |
| Lusu | 1,360 | 699 | 661 | 1,010 | 493 | 517 | 2,370 | 1,192 | 1,178 |
| Luampungu | 2,613 | 1,301 | 1,312 | 1,590 | 794 | 796 | 4,203 | 2,095 | 2,108 |
| Kalobolelwa | 2,366 | 1,182 | 1,184 | 1,728 | 886 | 842 | 4,094 | 2,068 | 2,026 |
| Imusho | 982 | 496 | 486 | 699 | 319 | 380 | 1,681 | 815 | 866 |
| **Shang'ombo District** | **45,469** | **22,811** | **22,658** | **30,451** | **14,004** | **16,447** | **75,920** | **36,815** | **39,105** |
| **Sinjembela Constituency** | **45,469** | **22,811** | **22,658** | **30,451** | **14,004** | **16,447** | **75,920** | **36,815** | **39,105** |
| Mambolomoka | 5,337 | 2,650 | 2,687 | 3,605 | 1,588 | 2,017 | 8,942 | 4,238 | 4,704 |
| Keyana | 2,980 | 1,471 | 1,509 | 2,124 | 984 | 1,140 | 5,104 | 2,455 | 2,649 |
| Nalwashi | 1,678 | 849 | 829 | 1,090 | 502 | 588 | 2,768 | 1,351 | 1,417 |
| Kalongola | 1,321 | 698 | 623 | 955 | 452 | 503 | 2,276 | 1,150 | 1,126 |
| Mbeta | 6,056 | 2,984 | 3,072 | 3,962 | 1,888 | 2,074 | 10,018 | 4,872 | 5,146 |
| Sioma | 2,658 | 1,330 | 1,328 | 1,962 | 955 | 1,007 | 4,620 | 2,285 | 2,335 |
| Sikabenga | 4,595 | 2,340 | 2,255 | 2,992 | 1,426 | 1,566 | 7,587 | 3,766 | 3,821 |
| Mutomena | 5,424 | 2,660 | 2,764 | 3,491 | 1,616 | 1,875 | 8,915 | 4,276 | 4,639 |
| Mulamba | 560 | 270 | 290 | 417 | 209 | 208 | 977 | 479 | 498 |
| Kaunga Mashi | 2,187 | 1,115 | 1,072 | 1,547 | 709 | 838 | 3,734 | 1,824 | 1,910 |
| Beshe | 1,391 | 682 | 709 | 857 | 396 | 461 | 2,248 | 1,078 | 1,170 |
| Sipuma | 3,571 | 1,783 | 1,788 | 2,432 | 1,057 | 1,375 | 6,003 | 2,840 | 3,163 |
| Mulonga | 4,245 | 2,175 | 2,070 | 2,782 | 1,239 | 1,543 | 7,027 | 3,414 | 3,613 |
| Simu | 3,466 | 1,804 | 1,662 | 2,235 | 983 | 1,252 | 5,701 | 2,787 | 2,914 |

# Social Tables

## Table B1: Population 15 Years and Older by 5 Year Age Group, Sex and Marital Status, Rural/Urban, Zambia 2010

| Sex and Age Group | Total Population 15 Years and Older | Marital Status | | | | |
|---|---|---|---|---|---|---|
| | | Never married | Married/Cohabiting | Divorced | Separated | Widowed |
| **Zambia Total** | **6,756,049** | **2,278,080** | **3,773,165** | **189,705** | **141,348** | **373,751** |
| Male | 3,249,752 | 1,330,819 | 1,786,279 | 45,675 | 39,771 | 47,208 |
| Female | 3,506,297 | 947,261 | 1,986,886 | 144,030 | 101,577 | 326,543 |
| **15 - 19** | **1,427,884** | **1,262,552** | **154,997** | **3,404** | **5,892** | **1,039** |
| Male | 692,859 | 679,165 | 12,546 | 296 | 647 | 205 |
| Female | 735,025 | 583,387 | 142,451 | 3,108 | 5,245 | 834 |
| **20 - 24** | **1,117,476** | **581,096** | **496,530** | **16,411** | **18,925** | **4,514** |
| Male | 504,876 | 370,229 | 128,848 | 2,180 | 3,122 | 497 |
| Female | 612,600 | 210,867 | 367,682 | 14,231 | 15,803 | 4,017 |
| **25 - 29** | **1,006,244** | **247,273** | **692,538** | **28,163** | **26,747** | **11,523** |
| Male | 464,493 | 165,424 | 285,529 | 5,622 | 6,570 | 1,348 |
| Female | 541,751 | 81,849 | 407,009 | 22,541 | 20,177 | 10,175 |
| **30 - 34** | **800,770** | **92,471** | **630,482** | **31,137** | **24,776** | **21,904** |
| Male | 397,694 | 59,941 | 319,511 | 7,970 | 7,577 | 2,695 |
| Female | 403,076 | 32,530 | 310,971 | 23,167 | 17,199 | 19,209 |
| **35 - 39** | **647,813** | **43,797** | **529,185** | **26,987** | **19,472** | **28,372** |
| Male | 332,961 | 27,874 | 287,880 | 7,366 | 6,284 | 3,557 |
| Female | 314,852 | 15,923 | 241,305 | 19,621 | 13,188 | 24,815 |
| **40 - 44** | **447,584** | **19,123** | **362,126** | **20,573** | **12,981** | **32,781** |
| Male | 232,253 | 11,863 | 206,157 | 5,608 | 4,364 | 4,261 |
| Female | 215,331 | 7,260 | 155,969 | 14,965 | 8,617 | 28,520 |
| **45 - 49** | **354,989** | **10,716** | **282,052** | **16,889** | **9,803** | **35,529** |
| Male | 174,833 | 6,272 | 156,959 | 4,215 | 3,098 | 4,289 |
| Female | 180,156 | 4,444 | 125,093 | 12,674 | 6,705 | 31,240 |
| **50 - 54** | **270,254** | **6,154** | **203,429** | **13,359** | **6,884** | **40,428** |
| Male | 128,696 | 3,399 | 115,515 | 3,210 | 2,201 | 4,371 |
| Female | 141,558 | 2,755 | 87,914 | 10,149 | 4,683 | 36,057 |
| **55 - 59** | **184,828** | **3,755** | **133,762** | **9,233** | **4,570** | **33,508** |
| Male | 90,037 | 1,941 | 80,605 | 2,290 | 1,536 | 3,665 |
| Female | 94,791 | 1,814 | 53,157 | 6,943 | 3,034 | 29,843 |
| **60 - 64** | **161,875** | **2,949** | **105,870** | **8,278** | **4,025** | **40,753** |
| Male | 73,419 | 1,359 | 64,351 | 2,039 | 1,323 | 4,347 |
| Female | 88,456 | 1,590 | 41,519 | 6,239 | 2,702 | 36,406 |
| **65 - 69** | **118,582** | **2,267** | **72,342** | **5,928** | **2,800** | **35,245** |
| Male | 53,695 | 949 | 46,290 | 1,574 | 1,050 | 3,832 |
| Female | 64,887 | 1,318 | 26,052 | 4,354 | 1,750 | 31,413 |
| **70 - 74** | **90,802** | **2,165** | **49,174** | **4,091** | **1,972** | **33,400** |
| Male | 41,929 | 911 | 34,681 | 1,300 | 787 | 4,250 |
| Female | 48,873 | 1,254 | 14,493 | 2,791 | 1,185 | 29,150 |
| **75+** | **126,948** | **3,762** | **60,678** | **5,252** | **2,501** | **54,755** |
| Male | 62,007 | 1,492 | 47,407 | 2,005 | 1,212 | 9,891 |
| Female | 64,941 | 2,270 | 13,271 | 3,247 | 1,289 | 44,864 |

## Table B1: Population 15 Years and Older by 5 Year Age Group, Sex and Marital Status, Rural/Urban, Zambia 2010

| Sex and Age Group | Total Population 15 Years and Older | Marital Status | | | | |
|---|---|---|---|---|---|---|
| | | Never married | Married/Cohabiting | Divorced | Separated | Widowed |
| **Zambia Rural** | **3,792,683** | **1,086,634** | **2,296,104** | **116,922** | **79,175** | **213,848** |
| Male | 1,801,972 | 659,849 | 1,071,803 | 25,686 | 19,683 | 24,951 |
| Female | 1,990,711 | 426,785 | 1,224,301 | 91,236 | 59,492 | 188,897 |
| **15 - 19** | **802,997** | **680,394** | **115,562** | **2,548** | **3,817** | **676** |
| Male | 397,738 | 387,101 | 9,861 | 215 | 430 | 131 |
| Female | 405,259 | 293,293 | 105,701 | 2,333 | 3,387 | 545 |
| **20 - 24** | **598,270** | **247,456** | **326,558** | **10,798** | **10,991** | **2,467** |
| Male | 269,368 | 169,546 | 96,400 | 1,395 | 1,727 | 300 |
| Female | 328,902 | 77,910 | 230,158 | 9,403 | 9,264 | 2,167 |
| **25 - 29** | **529,481** | **83,896** | **410,045** | **16,456** | **13,659** | **5,425** |
| Male | 241,136 | 57,060 | 177,422 | 3,088 | 2,888 | 678 |
| Female | 288,345 | 26,836 | 232,623 | 13,368 | 10,771 | 4,747 |
| **30 - 34** | **419,932** | **30,979** | **350,239** | **17,093** | **12,055** | **9,566** |
| Male | 203,507 | 20,096 | 175,208 | 3,896 | 3,094 | 1,213 |
| Female | 216,425 | 10,883 | 175,031 | 13,197 | 8,961 | 8,353 |
| **35 - 39** | **351,970** | **16,763** | **297,888** | **15,189** | **10,006** | **12,124** |
| Male | 173,650 | 10,835 | 155,133 | 3,530 | 2,684 | 1,468 |
| Female | 178,320 | 5,928 | 142,755 | 11,659 | 7,322 | 10,656 |
| **40 - 44** | **254,964** | **8,599** | **211,770** | **12,171** | **7,298** | **15,126** |
| Male | 127,044 | 5,556 | 114,786 | 2,873 | 2,060 | 1,769 |
| Female | 127,920 | 3,043 | 96,984 | 9,298 | 5,238 | 13,357 |
| **45 - 49** | **209,868** | **5,484** | **170,619** | **10,552** | **5,837** | **17,376** |
| Male | 100,903 | 3,293 | 91,714 | 2,384 | 1,636 | 1,876 |
| Female | 108,965 | 2,191 | 78,905 | 8,168 | 4,201 | 15,500 |
| **50 - 54** | **162,395** | **3,540** | **124,475** | **8,578** | **4,313** | **21,489** |
| Male | 75,868 | 2,037 | 68,691 | 1,893 | 1,232 | 2,015 |
| Female | 86,527 | 1,503 | 55,784 | 6,685 | 3,081 | 19,474 |
| **55 - 59** | **111,511** | **2,249** | **81,755** | **6,097** | **2,936** | **18,474** |
| Male | 52,225 | 1,181 | 47,055 | 1,414 | 919 | 1,656 |
| Female | 59,286 | 1,068 | 34,700 | 4,683 | 2,017 | 16,818 |
| **60 - 64** | **107,038** | **1,877** | **70,686** | **5,845** | **2,816** | **25,814** |
| Male | 45,761 | 884 | 40,399 | 1,359 | 854 | 2,265 |
| Female | 61,277 | 993 | 30,287 | 4,486 | 1,962 | 23,549 |
| **65 - 69** | **83,157** | **1,518** | **51,749** | **4,333** | **2,065** | **23,492** |
| Male | 36,611 | 655 | 31,927 | 1,085 | 718 | 2,226 |
| Female | 46,546 | 863 | 19,822 | 3,248 | 1,347 | 21,266 |
| **70 - 74** | **66,388** | **1,441** | **37,108** | **3,127** | **1,442** | **23,270** |
| Male | 30,553 | 622 | 25,759 | 968 | 540 | 2,664 |
| Female | 35,835 | 819 | 11,349 | 2,159 | 902 | 20,606 |
| **75+** | **94,712** | **2,438** | **47,650** | **4,135** | **1,940** | **38,549** |
| Male | 47,608 | 983 | 37,448 | 1,586 | 901 | 6,690 |
| Female | 47,104 | 1,455 | 10,202 | 2,549 | 1,039 | 31,859 |

| Table B1: Population 15 Years and Older by 5 Year Age Group, Sex and Marital Status, Rural/Urban, Zambia 2010 | | | | | | |
|---|---|---|---|---|---|---|
| Sex and Age Group | Total Population 15 Years and Older | Marital Status | | | | |
| | | Never married | Married/Cohabiting | Divorced | Separated | Widowed |
| **Zambia Urban** | **2,963,366** | **1,191,446** | **1,477,061** | **72,783** | **62,173** | **159,903** |
| Male | 1,447,780 | 670,970 | 714,476 | 19,989 | 20,088 | 22,257 |
| Female | 1,515,586 | 520,476 | 762,585 | 52,794 | 42,085 | 137,646 |
| **15 - 19** | **624,887** | **582,158** | **39,435** | **856** | **2,075** | **363** |
| Male | 295,121 | 292,064 | 2,685 | 81 | 217 | 74 |
| Female | 329,766 | 290,094 | 36,750 | 775 | 1,858 | 289 |
| **20 - 24** | **519,206** | **333,640** | **169,972** | **5,613** | **7,934** | **2,047** |
| Male | 235,508 | 200,683 | 32,448 | 785 | 1,395 | 197 |
| Female | 283,698 | 132,957 | 137,524 | 4,828 | 6,539 | 1,850 |
| **25 - 29** | **476,763** | **163,377** | **282,493** | **11,707** | **13,088** | **6,098** |
| Male | 223,357 | 108,364 | 108,107 | 2,534 | 3,682 | 670 |
| Female | 253,406 | 55,013 | 174,386 | 9,173 | 9,406 | 5,428 |
| **30 - 34** | **380,838** | **61,492** | **280,243** | **14,044** | **12,721** | **12,338** |
| Male | 194,187 | 39,845 | 144,303 | 4,074 | 4,483 | 1,482 |
| Female | 186,651 | 21,647 | 135,940 | 9,970 | 8,238 | 10,856 |
| **35 - 39** | **295,843** | **27,034** | **231,297** | **11,798** | **9,466** | **16,248** |
| Male | 159,311 | 17,039 | 132,747 | 3,836 | 3,600 | 2,089 |
| Female | 136,532 | 9,995 | 98,550 | 7,962 | 5,866 | 14,159 |
| **40 - 44** | **192,620** | **10,524** | **150,356** | **8,402** | **5,683** | **17,655** |
| Male | 105,209 | 6,307 | 91,371 | 2,735 | 2,304 | 2,492 |
| Female | 87,411 | 4,217 | 58,985 | 5,667 | 3,379 | 15,163 |
| **45 - 49** | **145,121** | **5,232** | **111,433** | **6,337** | **3,966** | **18,153** |
| Male | 73,930 | 2,979 | 65,245 | 1,831 | 1,462 | 2,413 |
| Female | 71,191 | 2,253 | 46,188 | 4,506 | 2,504 | 15,740 |
| **50 - 54** | **107,859** | **2,614** | **78,954** | **4,781** | **2,571** | **18,939** |
| Male | 52,828 | 1,362 | 46,824 | 1,317 | 969 | 2,356 |
| Female | 55,031 | 1,252 | 32,130 | 3,464 | 1,602 | 16,583 |
| **55 - 59** | **73,317** | **1,506** | **52,007** | **3,136** | **1,634** | **15,034** |
| Male | 37,812 | 760 | 33,550 | 876 | 617 | 2,009 |
| Female | 35,505 | 746 | 18,457 | 2,260 | 1,017 | 13,025 |
| **60 - 64** | **54,837** | **1,072** | **35,184** | **2,433** | **1,209** | **14,939** |
| Male | 27,658 | 475 | 23,952 | 680 | 469 | 2,082 |
| Female | 27,179 | 597 | 11,232 | 1,753 | 740 | 12,857 |
| **65 - 69** | **35,425** | **749** | **20,593** | **1,595** | **735** | **11,753** |
| Male | 17,084 | 294 | 14,363 | 489 | 332 | 1,606 |
| Female | 18,341 | 455 | 6,230 | 1,106 | 403 | 10,147 |
| **70 - 74** | **24,414** | **724** | **12,066** | **964** | **530** | **10,130** |
| Male | 11,376 | 289 | 8,922 | 332 | 247 | 1,586 |
| Female | 13,038 | 435 | 3,144 | 632 | 283 | 8,544 |
| **75+** | **32,236** | **1,324** | **13,028** | **1,117** | **561** | **16,206** |
| Male | 14,399 | 509 | 9,959 | 419 | 311 | 3,201 |
| Female | 17,837 | 815 | 3,069 | 698 | 250 | 13,005 |

## Table B2: Percent Distribution of Population 15 Years and Above by 5 Year Age Group, Sex, Selected Exact Ages at First Marriage, Never Married and Median Age at First Marriage, Rural/Urban, Zambia 2010

| Age Group and Sex | Exact Age at First Marriage | | | | | Never Married | | Median Age at First Marriage |
|---|---|---|---|---|---|---|---|---|
| | 15 | 18 | 20 | 22 | 25 | Never married | Percent Never Married | |
| **Zambia Total** | **100** | **100** | **100** | **100** | **100** | **2,278,080** | **100** | **20.7** |
| Male | 6.1 | 14.5 | 42.1 | 60.1 | 73.7 | 1,330,819 | 58.4 | 24.2 |
| Female | 93.9 | 85.5 | 57.9 | 39.9 | 26.3 | 947,261 | 41.6 | 18.8 |
| **15 - 19** | **13.2** | **6.1** | **-** | **-** | **-** | **1,262,552** | **55.4** | **16.9** |
| Male | 0.9 | 0.7 | - | - | - | 679,165 | 29.8 | 17.6 |
| Female | 12.3 | 5.4 | - | - | - | 583,387 | 25.6 | 16.9 |
| **20 - 24** | **16.4** | **18.4** | **15.6** | **12.9** | **-** | **581,096** | **25.5** | **19.1** |
| Male | 0.8 | 2.4 | 6.1 | 7.3 | - | 370,229 | 16.3 | 20.8 |
| Female | 15.6 | 16 | 9.5 | 5.6 | - | 210,867 | 9.3 | 18.5 |
| **25 - 29** | **17.6** | **18** | **17.3** | **20.9** | **18.6** | **247,273** | **10.9** | **20.6** |
| Male | 1 | 2.9 | 7.4 | 12.2 | 12.7 | 165,424 | 7.3 | 23 |
| Female | 16.6 | 15.1 | 9.9 | 8.7 | 5.9 | 81,849 | 3.6 | 19 |
| **30 - 34** | **12.5** | **13.7** | **16** | **17.6** | **17.9** | **92,471** | **4.1** | **21.5** |
| Male | 0.9 | 2.2 | 7.2 | 10.8 | 13.1 | 59,941 | 2.6 | 24.5 |
| Female | 11.7 | 11.5 | 8.8 | 6.7 | 4.7 | 32,530 | 1.4 | 19.4 |
| **35 - 39** | **10.1** | **11.5** | **12.6** | **13.4** | **16.8** | **43,797** | **1.9** | **22** |
| Male | 0.7 | 1.8 | 5.7 | 8.5 | 12.7 | 27,874 | 1.2 | 25.1 |
| Female | 9.4 | 9.7 | 6.9 | 4.9 | 4 | 15,923 | 0.7 | 19.3 |
| **40 - 44** | **6.3** | **7.8** | **9.6** | **10.1** | **11.3** | **19,123** | **0.8** | **22.1** |
| Male | 0.4 | 1.2 | 4.3 | 6.3 | 8.6 | 11,863 | 0.5 | 25.2 |
| Female | 5.8 | 6.6 | 5.4 | 3.8 | 2.6 | 7,260 | 0.3 | 19.5 |
| **45 - 49** | **5.7** | **6.6** | **7.4** | **7.3** | **9.8** | **10,716** | **0.5** | **22.1** |
| Male | 0.4 | 0.9 | 3 | 4.4 | 7.3 | 6,272 | 0.3 | 25.5 |
| Female | 5.3 | 5.8 | 4.4 | 2.9 | 2.5 | 4,444 | 0.2 | 19.4 |
| **50 - 54** | **4.7** | **5.1** | **5.9** | **5.4** | **7.2** | **6,154** | **0.3** | **21.9** |
| Male | 0.3 | 0.6 | 2.3 | 3.2 | 5.3 | 3,399 | 0.1 | 25.6 |
| Female | 4.4 | 4.4 | 3.6 | 2.2 | 1.9 | 2,755 | 0.1 | 19.3 |
| **55+** | **13.6** | **12.9** | **15.6** | **12.4** | **18.4** | **14,898** | **0.7** | **21.4** |
| Male | 0.8 | 1.8 | 6.2 | 7.5 | 13.8 | 6,652 | 0.3 | 25.5 |
| Female | 12.8 | 11.1 | 9.4 | 4.9 | 4.6 | 8,246 | 0.4 | 19 |

## Table B2: Percent Distribution of Population 15 Years and Above by 5 Year Age Group, Sex, Selected Exact Ages at First Marriage, Never Married and Median Age at First Marriage, Rural/Urban, Zambia 2010

| Age Group and Sex | Exact Age at First Marriage | | | | | Never Married | | Median Age at First Marriage |
|---|---|---|---|---|---|---|---|---|
| | 15 | 18 | 20 | 22 | 25 | Never married | Percent Never Married | |
| **Zambia Rural** | **100.0** | **100.0** | **100.0** | **100.0** | **100.0** | **1,086,634** | **100.0** | **20.3** |
| Male | 6.7 | 16.8 | 47.9 | 66.9 | 77.2 | 659,849 | 60.7 | 23.1 |
| Female | 93.3 | 83.2 | 52.1 | 33.1 | 22.8 | 426,785 | 39.3 | 18.5 |
| **15 - 19** | **14.2** | **6.5** | **0.0** | **0.0** | **0.0** | **680,394** | **62.6** | **16.8** |
| Male | 0.9 | 0.9 | 0.0 | 0.0 | 0.0 | 387,101 | 35.6 | 17.6 |
| Female | 13.2 | 5.6 | 0.0 | 0.0 | 0.0 | 293,293 | 27.0 | 16.8 |
| **20 - 24** | **16.9** | **18.5** | **15.1** | **12.7** | **0.0** | **247,456** | **22.8** | **18.9** |
| Male | 0.9 | 2.9 | 7.1 | 8.5 | 0.0 | 169,546 | 15.6 | 20.7 |
| Female | 16.0 | 15.6 | 7.9 | 4.1 | 0.0 | 77,910 | 7.2 | 18.2 |
| **25 - 29** | **17.8** | **17.6** | **16.5** | **19.8** | **15.6** | **83,896** | **7.7** | **20.1** |
| Male | 1.2 | 3.4 | 8.4 | 13.4 | 11.9 | 57,060 | 5.3 | 22.4 |
| Female | 16.7 | 14.2 | 8.1 | 6.3 | 3.7 | 26,836 | 2.5 | 18.5 |
| **30 - 34** | **12.2** | **13.1** | **15.3** | **16.6** | **16.2** | **30,979** | **2.9** | **20.7** |
| Male | 0.9 | 2.4 | 7.9 | 11.5 | 12.9 | 20,096 | 1.8 | 23.1 |
| Female | 11.3 | 10.6 | 7.4 | 5.1 | 3.4 | 10,883 | 1.0 | 18.7 |
| **35 - 39** | **9.9** | **11.1** | **12.3** | **13.1** | **16.1** | **16,763** | **1.5** | **20.9** |
| Male | 0.8 | 2.0 | 6.3 | 9.0 | 12.9 | 10,835 | 1.0 | 23.8 |
| Female | 9.2 | 9.1 | 6.0 | 4.0 | 3.2 | 5,928 | 0.5 | 18.8 |
| **40 - 44** | **6.2** | **7.8** | **9.6** | **10.1** | **11.2** | **8,599** | **0.8** | **21.1** |
| Male | 0.5 | 1.4 | 4.7 | 6.8 | 8.9 | 5,556 | 0.5 | 24.0 |
| Female | 5.7 | 6.4 | 4.9 | 3.3 | 2.4 | 3,043 | 0.3 | 19.0 |
| **45 - 49** | **5.5** | **6.6** | **7.5** | **7.6** | **10.2** | **5,484** | **0.5** | **21.2** |
| Male | 0.4 | 1.0 | 3.5 | 5.0 | 7.8 | 3,293 | 0.3 | 24.6 |
| Female | 5.1 | 5.6 | 4.0 | 2.6 | 2.3 | 2,191 | 0.2 | 19.0 |
| **50 - 54** | **4.4** | **5.0** | **5.9** | **5.7** | **7.7** | **3,540** | **0.3** | **21.2** |
| Male | 0.3 | 0.7 | 2.6 | 3.6 | 5.8 | 2,037 | 0.2 | 25.0 |
| Female | 4.1 | 4.2 | 3.4 | 2.1 | 1.9 | 1,503 | 0.1 | 19.1 |
| **55+** | **13.0** | **13.9** | **17.7** | **14.5** | **22.9** | **9,523** | **0.9** | **21.0** |
| Male | 0.9 | 2.1 | 7.3 | 9.0 | 17.1 | 4,325 | 0.4 | 25.2 |
| Female | 12.1 | 11.8 | 10.4 | 5.6 | 5.9 | 5,198 | 0.5 | 19.0 |

## Table B2: Percent Distribution of Population 15 Years and Above by 5 Year Age Group, Sex, Selected Exact Ages at First Marriage, Never Married and Median Age at First Marriage, Rural/Urban, Zambia 2010

| Age Group and Sex | Exact Age at First Marriage | | | | | Never Married | | Median Age at First Marriage |
|---|---|---|---|---|---|---|---|---|
| | 15 | 18 | 20 | 22 | 25 | Never married | Percent Never Married | |
| **Zambia Urban** | **100.0** | **100.0** | **100.0** | **100.0** | **100.0** | **1,191,446** | **100.0** | **21.9** |
| Male | 4.7 | 10.1 | 31.4 | 50.2 | 69.4 | 670,970 | 56.3 | 25.5 |
| Female | 95.3 | 89.9 | 68.6 | 49.8 | 30.6 | 520,476 | 43.7 | 19.6 |
| **15 - 19** | **10.7** | **5.4** | **0.0** | **0.0** | **0.0** | **582,158** | **48.9** | **17.1** |
| Male | 0.8 | 0.4 | 0.0 | 0.0 | 0.0 | 292,064 | 24.5 | 17.5 |
| Female | 9.9 | 4.9 | 0.0 | 0.0 | 0.0 | 290,094 | 24.3 | 17.1 |
| **20 - 24** | **14.9** | **18.1** | **16.6** | **13.3** | **0.0** | **333,640** | **28.0** | **19.5** |
| Male | 0.5 | 1.5 | 4.1 | 5.4 | 0.0 | 200,683 | 16.8 | 21.2 |
| Female | 14.4 | 16.7 | 12.5 | 7.9 | 0.0 | 132,957 | 11.2 | 19.0 |
| **25 - 29** | **17.0** | **18.7** | **18.7** | **22.4** | **22.3** | **163,377** | **13.7** | **21.6** |
| Male | 0.7 | 1.9 | 5.5 | 10.3 | 13.7 | 108,364 | 9.1 | 24.1 |
| Female | 16.4 | 16.8 | 13.2 | 12.1 | 8.5 | 55,013 | 4.6 | 20.1 |
| **30 - 34** | **13.5** | **14.8** | **17.2** | **19.0** | **19.8** | **61,492** | **5.2** | **23.0** |
| Male | 0.7 | 1.7 | 5.8 | 10.0 | 13.4 | 39,845 | 3.3 | 25.8 |
| Female | 12.8 | 13.1 | 11.4 | 9.1 | 6.4 | 21,647 | 1.8 | 20.3 |
| **35 - 39** | **10.4** | **12.3** | **13.2** | **13.9** | **17.6** | **27,034** | **2.3** | **23.7** |
| Male | 0.6 | 1.4 | 4.6 | 7.7 | 12.6 | 17,039 | 1.4 | 26.3 |
| Female | 9.9 | 10.9 | 8.5 | 6.2 | 5.0 | 9,995 | 0.8 | 20.1 |
| **40 - 44** | **6.5** | **7.9** | **9.7** | **10.1** | **11.3** | **10,524** | **0.9** | **23.7** |
| Male | 0.4 | 0.9 | 3.4 | 5.6 | 8.4 | 6,307 | 0.5 | 26.5 |
| Female | 6.1 | 7.0 | 6.4 | 4.5 | 2.9 | 4,217 | 0.4 | 20.1 |
| **45 - 49** | **6.1** | **6.7** | **7.3** | **6.9** | **9.3** | **5,232** | **0.4** | **23.5** |
| Male | 0.2 | 0.6 | 2.2 | 3.4 | 6.6 | 2,979 | 0.3 | 26.6 |
| Female | 5.9 | 6.1 | 5.0 | 3.5 | 2.7 | 2,253 | 0.2 | 19.9 |
| **50 - 54** | **5.4** | **5.2** | **5.7** | **5.0** | **6.7** | **2,614** | **0.2** | **22.8** |
| Male | 0.2 | 0.5 | 1.7 | 2.5 | 4.8 | 1,362 | 0.1 | 26.2 |
| Female | 5.2 | 4.8 | 4.0 | 2.5 | 1.9 | 1,252 | 0.1 | 19.6 |
| **55+** | **15.4** | **10.9** | **11.7** | **9.3** | **13.0** | **5,375** | **0.5** | **22.1** |
| Male | 0.6 | 1.2 | 4.2 | 5.3 | 9.9 | 2,327 | 0.2 | 26.0 |
| Female | 14.7 | 9.7 | 7.5 | 4.0 | 3.1 | 3,048 | 0.3 | 18.9 |

# Table B3: Population Aged 0-17 Years With and Without Birth Certificates by Sex and Age (Single and Grouped), Rural/Urban, Zambia 2010

| Age | Total Population 0-17 years | Total | | | Male | | | Female | | |
|---|---|---|---|---|---|---|---|---|---|---|
| | | With Birth Certificates | Without Birth Certificates | Don't know | With Birth Certificates | Without Birth Certificates | Don't know | With Birth Certificates | Without Birth Certificates | Don't know |
| **Zambia Total** | **6,638,301** | **1,128,011** | **5,098,619** | **411,671** | **557,742** | **2,541,064** | **193,816** | **570,269** | **2,557,555** | **217,855** |
| **0 - 4** | **2,214,887** | **378,806** | **1,737,154** | **98,927** | **188,680** | **864,672** | **48,920** | **190,126** | **872,482** | **50,007** |
| 0 | 436,304 | 71,384 | 345,066 | 19,854 | 35,753 | 172,196 | 9,941 | 35,631 | 172,870 | 9,913 |
| 1 | 437,960 | 77,985 | 341,483 | 18,492 | 38,875 | 170,176 | 9,206 | 39,110 | 171,307 | 9,286 |
| 2 | 452,387 | 78,243 | 354,318 | 19,826 | 39,142 | 176,346 | 9,811 | 39,101 | 177,972 | 10,015 |
| 3 | 442,255 | 76,211 | 346,110 | 19,934 | 37,509 | 171,388 | 9,745 | 38,702 | 174,722 | 10,189 |
| 4 | 445,981 | 74,983 | 350,177 | 20,821 | 37,401 | 174,566 | 10,217 | 37,582 | 175,611 | 10,604 |
| **5 - 9** | **1,856,336** | **311,076** | **1,451,446** | **93,814** | **154,737** | **724,286** | **45,372** | **156,339** | **727,160** | **48,442** |
| 5 | 406,833 | 68,454 | 319,566 | 18,813 | 34,434 | 160,620 | 9,196 | 34,020 | 158,946 | 9,617 |
| 6 | 396,794 | 65,672 | 312,294 | 18,828 | 32,830 | 155,574 | 9,077 | 32,842 | 156,720 | 9,751 |
| 7 | 388,305 | 64,571 | 304,169 | 19,565 | 32,393 | 152,738 | 9,570 | 32,178 | 151,431 | 9,995 |
| 8 | 351,465 | 58,802 | 274,011 | 18,652 | 28,752 | 135,528 | 8,943 | 30,050 | 138,483 | 9,709 |
| 9 | 312,939 | 53,577 | 241,406 | 17,956 | 26,328 | 119,826 | 8,586 | 27,249 | 121,580 | 9,370 |
| **10 - 14** | **1,699,042** | **288,403** | **1,288,029** | **122,610** | **141,633** | **641,850** | **57,351** | **146,770** | **646,179** | **65,259** |
| 10 | 449,492 | 72,096 | 349,859 | 27,537 | 35,722 | 175,540 | 13,206 | 36,374 | 174,319 | 14,331 |
| 11 | 290,577 | 49,540 | 221,376 | 19,661 | 24,187 | 110,184 | 9,126 | 25,353 | 111,192 | 10,535 |
| 12 | 351,053 | 59,689 | 265,756 | 25,608 | 29,578 | 132,783 | 12,110 | 30,111 | 132,973 | 13,498 |
| 13 | 305,128 | 53,576 | 227,561 | 23,991 | 26,225 | 112,577 | 10,916 | 27,351 | 114,984 | 13,075 |
| 14 | 302,792 | 53,502 | 223,477 | 25,813 | 25,921 | 110,766 | 11,993 | 27,581 | 112,711 | 13,820 |
| **15 - 17** | **868,036** | **149,726** | **621,990** | **96,320** | **72,692** | **310,256** | **42,173** | **77,034** | **311,734** | **54,147** |
| 15 | 312,338 | 53,135 | 229,465 | 29,738 | 26,046 | 115,243 | 13,858 | 27,089 | 114,222 | 15,880 |
| 16 | 295,552 | 51,107 | 212,510 | 31,935 | 24,486 | 105,257 | 13,897 | 26,621 | 107,253 | 18,038 |
| 17 | 260,146 | 45,484 | 180,015 | 34,647 | 22,160 | 89,756 | 14,418 | 23,324 | 90,259 | 20,229 |
| | | | | | | | | | | |
| **Zambia Rural** | **4,208,117** | **359,391** | **3,650,226** | **198,500** | **180,485** | **1,836,337** | **95,714** | **178,906** | **1,813,889** | **102,786** |
| **0 - 4** | **1,454,385** | **134,313** | **1,264,096** | **55,976** | **66,778** | **629,497** | **27,883** | **67,535** | **634,599** | **28,093** |
| 0 | 288,243 | 26,546 | 249,465 | 12,232 | 13,356 | 124,373 | 6,145 | 13,190 | 125,092 | 6,087 |
| 1 | 280,428 | 26,978 | 242,969 | 10,481 | 13,377 | 121,283 | 5,261 | 13,601 | 121,686 | 5,220 |
| 2 | 300,708 | 28,336 | 261,018 | 11,354 | 13,971 | 129,714 | 5,590 | 14,365 | 131,304 | 5,764 |
| 3 | 290,561 | 26,503 | 253,299 | 10,759 | 13,114 | 125,730 | 5,346 | 13,389 | 127,569 | 5,413 |
| 4 | 294,445 | 25,950 | 257,345 | 11,150 | 12,960 | 128,397 | 5,541 | 12,990 | 128,948 | 5,609 |
| **5- 9** | **1,212,247** | **103,139** | **1,062,209** | **46,899** | **51,794** | **533,330** | **23,117** | **51,345** | **528,879** | **23,782** |
| 5 | 268,012 | 23,405 | 234,895 | 9,712 | 11,835 | 118,567 | 4,773 | 11,570 | 116,328 | 4,939 |
| 6 | 262,858 | 22,459 | 230,708 | 9,691 | 11,310 | 115,563 | 4,695 | 11,149 | 115,145 | 4,996 |
| 7 | 253,186 | 21,264 | 222,179 | 9,743 | 10,745 | 112,080 | 4,764 | 10,519 | 110,099 | 4,979 |
| 8 | 229,514 | 19,505 | 200,744 | 9,265 | 9,679 | 99,982 | 4,618 | 9,826 | 100,762 | 4,647 |
| 9 | 198,677 | 16,506 | 173,683 | 8,488 | 8,225 | 87,138 | 4,267 | 8,281 | 86,545 | 4,221 |
| **10 - 14** | **1,045,977** | **84,113** | **908,116** | **53,748** | **42,469** | **460,791** | **26,718** | **41,644** | **447,325** | **27,030** |
| 10 | 291,852 | 23,165 | 255,318 | 13,369 | 11,636 | 129,442 | 6,655 | 11,529 | 125,876 | 6,714 |
| 11 | 179,931 | 14,286 | 156,753 | 8,892 | 7,140 | 79,156 | 4,407 | 7,146 | 77,597 | 4,485 |
| 12 | 216,717 | 17,618 | 187,735 | 11,364 | 9,083 | 95,661 | 5,651 | 8,535 | 92,074 | 5,713 |
| 13 | 180,849 | 14,664 | 156,363 | 9,822 | 7,471 | 79,134 | 4,844 | 7,193 | 77,229 | 4,978 |
| 14 | 176,628 | 14,380 | 151,947 | 10,301 | 7,139 | 77,398 | 5,161 | 7,241 | 74,549 | 5,140 |
| **15 - 17** | **495,508** | **37,826** | **415,805** | **41,877** | **19,444** | **212,719** | **17,996** | **18,382** | **203,086** | **23,881** |
| 15 | 184,001 | 14,424 | 156,777 | 12,800 | 7,493 | 80,802 | 6,210 | 6,931 | 75,975 | 6,590 |
| 16 | 169,390 | 12,907 | 142,706 | 13,777 | 6,512 | 72,763 | 5,888 | 6,395 | 69,943 | 7,889 |
| 17 | 142,117 | 10,495 | 116,322 | 15,300 | 5,439 | 59,154 | 5,898 | 5,056 | 57,168 | 9,402 |

## Table B3. Population Aged 0-17 Years With and Without Birth Certificates by Sex and Age (Single and Grouped), Rural/Urban, Zambia 2010

| Age | Total Population 0-17 years | Total | | | Male | | | Female | | |
|---|---|---|---|---|---|---|---|---|---|---|
| | | With Birth Certificates | Without Birth Certificates | Don't know | With Birth Certificates | Without Birth Certificates | Don't know | With Birth Certificates | Without Birth Certificates | Don't know |
| **Zambia Urban** | **2,430,184** | **768,620** | **1,448,393** | **213,171** | **377,257** | **704,727** | **98,102** | **391,363** | **743,666** | **115,069** |
| **0 - 4** | **760,502** | **244,493** | **473,058** | **42,951** | **121,902** | **235,175** | **21,037** | **122,591** | **237,883** | **21,914** |
| 0 | 148,061 | 44,838 | 95,601 | 7,622 | 22,397 | 47,823 | 3,796 | 22,441 | 47,778 | 3,826 |
| 1 | 157,532 | 51,007 | 98,514 | 8,011 | 25,498 | 48,893 | 3,945 | 25,509 | 49,621 | 4,066 |
| 2 | 151,679 | 49,907 | 93,300 | 8,472 | 25,171 | 46,632 | 4,221 | 24,736 | 46,668 | 4,251 |
| 3 | 151,694 | 49,708 | 92,811 | 9,175 | 24,395 | 45,658 | 4,399 | 25,313 | 47,153 | 4,776 |
| 4 | 151,536 | 49,033 | 92,832 | 9,671 | 24,441 | 46,169 | 4,676 | 24,592 | 46,663 | 4,995 |
| **5- 9** | **644,089** | **207,937** | **389,237** | **46,915** | **102,943** | **190,956** | **22,255** | **104,994** | **198,281** | **24,660** |
| 5 | 138,821 | 45,049 | 84,671 | 9,101 | 22,599 | 42,053 | 4,423 | 22,450 | 42,618 | 4,678 |
| 6 | 133,936 | 43,213 | 81,586 | 9,137 | 21,520 | 40,011 | 4,382 | 21,693 | 41,575 | 4,755 |
| 7 | 135,119 | 43,307 | 81,990 | 9,822 | 21,648 | 40,658 | 4,806 | 21,659 | 41,332 | 5,016 |
| 8 | 121,951 | 39,297 | 73,267 | 9,387 | 19,073 | 35,546 | 4,325 | 20,224 | 37,721 | 5,062 |
| 9 | 114,262 | 37,071 | 67,723 | 9,468 | 18,103 | 32,688 | 4,319 | 18,968 | 35,035 | 5,149 |
| **10 - 14** | **653,065** | **204,290** | **379,913** | **68,862** | **99,164** | **181,059** | **30,633** | **105,126** | **198,854** | **38,229** |
| 10 | 157,640 | 48,931 | 94,541 | 14,168 | 24,086 | 46,098 | 6,551 | 24,845 | 48,443 | 7,617 |
| 11 | 110,646 | 35,254 | 64,623 | 10,769 | 17,047 | 31,028 | 4,719 | 18,207 | 33,595 | 6,050 |
| 12 | 134,336 | 42,071 | 78,021 | 14,244 | 20,495 | 37,122 | 6,459 | 21,576 | 40,899 | 7,785 |
| 13 | 124,279 | 38,912 | 71,198 | 14,169 | 18,754 | 33,443 | 6,072 | 20,158 | 37,755 | 8,097 |
| 14 | 126,164 | 39,122 | 71,530 | 15,512 | 18,782 | 33,368 | 6,832 | 20,340 | 38,162 | 8,680 |
| **15 - 17** | **372,528** | **111,900** | **206,185** | **54,443** | **53,248** | **97,537** | **24,177** | **58,652** | **108,648** | **30,266** |
| 15 | 128,337 | 38,711 | 72,688 | 16,938 | 18,553 | 34,441 | 7,648 | 20,158 | 38,247 | 9,290 |
| 16 | 126,162 | 38,200 | 69,804 | 18,158 | 17,974 | 32,494 | 8,009 | 20,226 | 37,310 | 10,149 |
| 17 | 118,029 | 34,989 | 63,693 | 19,347 | 16,721 | 30,602 | 8,520 | 18,268 | 33,091 | 10,827 |

## Table B4i. Population (De facto) 3 Years and Above (Excluding the Deaf and Dumb and Persons with a Speech Impairment) by Sex and Predominant Language of Communication, Rural/Urban, Zambia 2010

| Predominant Language of Communication | Total | | | Rural | | | Urban | | |
|---|---|---|---|---|---|---|---|---|---|
| | Total | Male | Female | Total | Male | Female | Total | Male | Female |
| **Zambia** | | | | | | | | | |
| **Total** | **11,174,260** | **5,442,014** | **5,732,246** | **6,617,226** | **3,221,164** | **3,396,062** | **4,557,034** | **2,220,850** | **2,336,184** |
| Ambo | 747 | 370 | 377 | 660 | 331 | 329 | 87 | 39 | 48 |
| Bemba | 3,729,828 | 1,827,141 | 1,902,687 | 1,481,193 | 728,106 | 753,087 | 2,248,635 | 1,099,035 | 1,149,600 |
| Bisa | 112,016 | 52,615 | 59,401 | 107,953 | 50,746 | 57,207 | 4,063 | 1,869 | 2,194 |
| Bwile | 19,564 | 9,502 | 10,062 | 18,767 | 9,121 | 9,646 | 797 | 381 | 416 |
| Chewa | 499,671 | 245,838 | 253,833 | 447,433 | 220,059 | 227,374 | 52,238 | 25,779 | 26,459 |
| Chikunda | 6,759 | 3,281 | 3,478 | 5,687 | 2,777 | 2,910 | 1,072 | 504 | 568 |
| Chishinga | 12,760 | 6,244 | 6,516 | 9,974 | 4,875 | 5,099 | 2,786 | 1,369 | 1,417 |
| Chokwe | 17,651 | 8,537 | 9,114 | 13,226 | 6,389 | 6,837 | 4,425 | 2,148 | 2,277 |
| Gowa | 12,675 | 6,153 | 6,522 | 10,800 | 5,284 | 5,516 | 1,875 | 869 | 1,006 |
| Ila | 82,940 | 40,244 | 42,696 | 74,734 | 36,469 | 38,265 | 8,206 | 3,775 | 4,431 |
| Imilangu | 1,982 | 943 | 1,039 | 1,745 | 821 | 924 | 237 | 122 | 115 |
| Kabende | 37,929 | 18,002 | 19,927 | 35,631 | 16,887 | 18,744 | 2,298 | 1,115 | 1,183 |
| Kaonde | 206,182 | 99,730 | 106,452 | 155,734 | 75,649 | 80,085 | 50,448 | 24,081 | 26,367 |
| Koma | 9,302 | 4,138 | 5,164 | 9,199 | 4,087 | 5,112 | 103 | 51 | 52 |
| Kunda | 40,029 | 19,272 | 20,757 | 35,578 | 17,179 | 18,399 | 4,451 | 2,093 | 2,358 |
| Kwandi | 997 | 489 | 508 | 964 | 473 | 491 | 33 | 16 | 17 |
| Kwangwa | 1,405 | 654 | 751 | 1,367 | 634 | 733 | 38 | 20 | 18 |
| Lala | 200,772 | 99,031 | 101,741 | 189,313 | 93,398 | 95,915 | 11,459 | 5,633 | 5,826 |
| Lamba | 198,390 | 96,979 | 101,411 | 179,701 | 88,155 | 91,546 | 18,689 | 8,824 | 9,865 |
| Lenje | 128,269 | 63,086 | 65,183 | 117,586 | 58,056 | 59,530 | 10,683 | 5,030 | 5,653 |
| Lima | 2,254 | 1,099 | 1,155 | 1,850 | 904 | 946 | 404 | 195 | 209 |
| Lozi | 611,920 | 290,597 | 321,323 | 466,559 | 221,263 | 245,296 | 145,361 | 69,334 | 76,027 |
| Luano | 1,623 | 800 | 823 | 1,569 | 773 | 796 | 54 | 27 | 27 |
| Luchazi | 29,578 | 14,290 | 15,288 | 25,388 | 12,319 | 13,069 | 4,190 | 1,971 | 2,219 |
| Lunda (Luapula) | 15,009 | 7,299 | 7,710 | 8,404 | 4,197 | 4,207 | 6,605 | 3,102 | 3,503 |
| Lunda (North Western) | 213,719 | 103,520 | 110,199 | 171,920 | 83,293 | 88,627 | 41,799 | 20,227 | 21,572 |
| Lungu | 64,542 | 31,127 | 33,415 | 49,730 | 24,056 | 25,674 | 14,812 | 7,071 | 7,741 |
| Luvale | 170,815 | 82,120 | 88,695 | 128,478 | 61,776 | 66,702 | 42,337 | 20,344 | 21,993 |
| Luyana | 481 | 211 | 270 | 437 | 188 | 249 | 44 | 23 | 21 |
| Mambwe | 142,175 | 69,095 | 73,080 | 118,751 | 57,779 | 60,972 | 23,424 | 11,316 | 12,108 |
| Mashasha | 1,186 | 577 | 609 | 1,114 | 546 | 568 | 72 | 31 | 41 |
| Mashi | 18,805 | 8,419 | 10,386 | 18,156 | 8,123 | 10,033 | 649 | 296 | 353 |
| Mbowe | 464 | 233 | 231 | 239 | 124 | 115 | 225 | 109 | 116 |
| Mbunda | 82,980 | 38,625 | 44,355 | 74,230 | 34,512 | 39,718 | 8,750 | 4,113 | 4,637 |
| Mukulu | 1,679 | 824 | 855 | 1,473 | 718 | 755 | 206 | 106 | 100 |
| Mwenyi | 2,868 | 1,298 | 1,570 | 2,802 | 1,265 | 1,537 | 66 | 33 | 33 |

## Table B4i. Population (De facto) 3 Years and Above (Excluding the Deaf and Dumb and Persons with a Speech Impairment) by Sex and Predominant Language of Communication, Rural/Urban, Zambia 2010

| Predominant Language of Communication | Total | | | Rural | | | Urban | | |
|---|---|---|---|---|---|---|---|---|---|
| | Total | Male | Female | Total | Male | Female | Total | Male | Female |
| Namwanga | 135,382 | 65,886 | 69,496 | 104,950 | 51,188 | 53,762 | 30,432 | 14,698 | 15,734 |
| Ndembu | 10,913 | 5,290 | 5,623 | 9,678 | 4,709 | 4,969 | 1,235 | 581 | 654 |
| Ngoni | 75,945 | 36,603 | 39,342 | 58,945 | 28,657 | 30,288 | 17,000 | 7,946 | 9,054 |
| Ngumbo | 43,547 | 20,821 | 22,726 | 42,736 | 20,406 | 22,330 | 811 | 415 | 396 |
| Nkoya | 34,994 | 16,497 | 18,497 | 32,337 | 15,239 | 17,098 | 2,657 | 1,258 | 1,399 |
| Nsenga | 328,793 | 159,224 | 169,569 | 282,199 | 137,205 | 144,994 | 46,594 | 22,019 | 24,575 |
| Nyanja | 1,643,686 | 809,718 | 833,968 | 372,876 | 186,610 | 186,266 | 1,270,810 | 623,108 | 647,702 |
| Nyengo | 11,487 | 5,052 | 6,435 | 11,188 | 4,909 | 6,279 | 299 | 143 | 156 |
| Sala | 23,544 | 11,434 | 12,110 | 21,960 | 10,683 | 11,277 | 1,584 | 751 | 833 |
| Senga | 79,546 | 37,870 | 41,676 | 72,949 | 34,665 | 38,284 | 6,597 | 3,205 | 3,392 |
| Shila | 4,117 | 2,052 | 2,065 | 2,753 | 1,379 | 1,374 | 1,364 | 673 | 691 |
| Simaa | 2,529 | 1,098 | 1,431 | 2,508 | 1,091 | 1,417 | 21 | 7 | 14 |
| Soli | 34,075 | 16,922 | 17,153 | 30,554 | 15,254 | 15,300 | 3,521 | 1,668 | 1,853 |
| Subiya | 1,350 | 648 | 702 | 1,202 | 582 | 620 | 148 | 66 | 82 |
| Swaka | 37,653 | 18,688 | 18,965 | 33,451 | 16,581 | 16,870 | 4,202 | 2,107 | 2,095 |
| Tabwa | 27,450 | 13,221 | 14,229 | 25,619 | 12,333 | 13,286 | 1,831 | 888 | 943 |
| Tambo | 4,223 | 2,030 | 2,193 | 4,028 | 1,946 | 2,082 | 195 | 84 | 111 |
| Toka-Leya | 55,644 | 27,198 | 28,446 | 49,658 | 24,416 | 25,242 | 5,986 | 2,782 | 3,204 |
| Tonga | 1,271,993 | 615,472 | 656,521 | 1,050,305 | 510,100 | 540,205 | 221,688 | 105,372 | 116,316 |
| Totela | 1,118 | 522 | 596 | 888 | 406 | 482 | 230 | 116 | 114 |
| Tumbuka | 284,917 | 137,498 | 147,419 | 264,288 | 127,531 | 136,757 | 20,629 | 9,967 | 10,662 |
| Unga | 11,518 | 5,377 | 6,141 | 11,411 | 5,321 | 6,090 | 107 | 56 | 51 |
| Ushi | 100,352 | 48,342 | 52,010 | 89,629 | 43,171 | 46,458 | 10,723 | 5,171 | 5,552 |
| Wina | 4,189 | 2,054 | 2,135 | 3,775 | 1,812 | 1,963 | 414 | 242 | 172 |
| Yombe | 2,001 | 954 | 1,047 | 591 | 280 | 311 | 1,410 | 674 | 736 |
| English language | 184,453 | 90,683 | 93,770 | 11,281 | 5,986 | 5,295 | 173,172 | 84,697 | 88,475 |
| Other Language groups | 31,537 | 15,470 | 16,067 | 26,079 | 12,644 | 13,435 | 5,458 | 2,826 | 2,632 |
| Language Not Applicable | 12,812 | 5,473 | 7,339 | 8,481 | 3,618 | 4,863 | 4,331 | 1,855 | 2,476 |
| Language Not Stated | 13,449 | 6,994 | 6,455 | 9,340 | 4,706 | 4,634 | 4,109 | 2,288 | 1,821 |
| African | 17,163 | 8,384 | 8,779 | 12,344 | 5,944 | 6,400 | 4,819 | 2,440 | 2,379 |
| American | 417 | 204 | 213 | 189 | 89 | 100 | 228 | 115 | 113 |
| Asian | 2,701 | 1,548 | 1,153 | 459 | 255 | 204 | 2,242 | 1,293 | 949 |
| European | 796 | 404 | 392 | 230 | 116 | 114 | 566 | 288 | 278 |

## Table B4 ii. Population (De facto) by Sex and Ethnicity, Rural/Urban, Zambia 2010

| Ethnicity | Total | | | Rural | | | Urban | | |
|---|---|---|---|---|---|---|---|---|---|
| | Total | Male | Female | Total | Male | Female | Total | Male | Female |
| **Zambia** | | | | | | | | | |
| **Total** | **12,526,314** | **6,117,253** | **6,409,061** | **7,505,292** | **3,664,349** | **3,840,943** | **5,021,022** | **2,452,904** | **2,568,118** |
| Ambo | 2,445 | 1,193 | 1,252 | 1,476 | 710 | 766 | 969 | 483 | 486 |
| Bemba | 2,629,710 | 1,285,174 | 1,344,536 | 1,197,328 | 587,535 | 609,793 | 1,432,382 | 697,639 | 734,743 |
| Bisa | 197,744 | 94,379 | 103,365 | 146,481 | 69,667 | 76,814 | 51,263 | 24,712 | 26,551 |
| Bwile | 55,598 | 27,025 | 28,573 | 51,347 | 24,938 | 26,409 | 4,251 | 2,087 | 2,164 |
| Chewa | 929,842 | 458,418 | 471,424 | 604,038 | 297,768 | 306,270 | 325,804 | 160,650 | 165,154 |
| Chikunda | 31,291 | 15,338 | 15,953 | 13,954 | 6,907 | 7,047 | 17,337 | 8,431 | 8,906 |
| Chishinga | 64,417 | 31,452 | 32,965 | 48,669 | 23,800 | 24,869 | 15,748 | 7,652 | 8,096 |
| Chokwe | 61,890 | 30,748 | 31,142 | 35,689 | 17,643 | 18,046 | 26,201 | 13,105 | 13,096 |
| Gowa | 21,942 | 10,730 | 11,212 | 14,473 | 7,051 | 7,422 | 7,469 | 3,679 | 3,790 |
| Ila | 97,411 | 47,861 | 49,550 | 68,694 | 33,980 | 34,714 | 28,717 | 13,881 | 14,836 |
| Imilangu | 3,633 | 1,669 | 1,964 | 2,877 | 1,322 | 1,555 | 756 | 347 | 409 |
| Kabende | 44,955 | 21,476 | 23,479 | 38,653 | 18,381 | 20,272 | 6,302 | 3,095 | 3,207 |
| Kaonde | 366,441 | 178,674 | 187,767 | 208,022 | 100,950 | 107,072 | 158,419 | 77,724 | 80,695 |
| Koma | 14,342 | 6,566 | 7,776 | 13,424 | 6,133 | 7,291 | 918 | 433 | 485 |
| Kunda | 83,467 | 39,948 | 43,519 | 46,302 | 22,347 | 23,955 | 37,165 | 17,601 | 19,564 |
| Kwandi | 5,702 | 2,916 | 2,786 | 5,353 | 2,732 | 2,621 | 349 | 184 | 165 |
| Kwangwa | 24,113 | 11,419 | 12,694 | 22,855 | 10,801 | 12,054 | 1,258 | 618 | 640 |
| Lala | 391,883 | 190,796 | 201,087 | 251,925 | 123,365 | 128,560 | 139,958 | 67,431 | 72,527 |
| Lamba | 257,225 | 124,269 | 132,956 | 163,066 | 78,853 | 84,213 | 94,159 | 45,416 | 48,743 |
| Lenje | 196,892 | 95,837 | 101,055 | 128,320 | 63,198 | 65,122 | 68,572 | 32,639 | 35,933 |
| Lima | 5,715 | 2,724 | 2,991 | 3,945 | 1,873 | 2,072 | 1,770 | 851 | 919 |
| Lozi | 717,015 | 346,812 | 370,203 | 454,938 | 219,080 | 235,858 | 262,077 | 127,732 | 134,345 |
| Luano | 1,680 | 813 | 867 | 1,318 | 619 | 699 | 362 | 194 | 168 |
| Luchazi | 51,897 | 25,491 | 26,406 | 39,001 | 19,140 | 19,861 | 12,896 | 6,351 | 6,545 |
| Lunda (Luapula) | 118,562 | 58,656 | 59,906 | 55,527 | 27,451 | 28,076 | 63,035 | 31,205 | 31,830 |
| Lunda (North Western) | 325,301 | 160,139 | 165,162 | 215,716 | 105,619 | 110,097 | 109,585 | 54,520 | 55,065 |
| Lungu | 100,357 | 49,325 | 51,032 | 72,871 | 35,784 | 37,087 | 27,486 | 13,541 | 13,945 |
| Luvale | 272,931 | 134,838 | 138,093 | 161,083 | 78,947 | 82,136 | 111,848 | 55,891 | 55,957 |
| Luyana | 1,140 | 535 | 605 | 928 | 434 | 494 | 212 | 101 | 111 |
| Mambwe | 316,131 | 155,656 | 160,475 | 168,113 | 82,860 | 85,253 | 148,018 | 72,796 | 75,222 |
| Mashasha | 1,163 | 580 | 583 | 695 | 365 | 330 | 468 | 215 | 253 |
| Mashi | 43,771 | 20,215 | 23,556 | 40,372 | 18,597 | 21,775 | 3,399 | 1,618 | 1,781 |
| Mbowe | 1,622 | 837 | 785 | 1,082 | 561 | 521 | 540 | 276 | 264 |
| Mbunda | 152,512 | 73,466 | 79,046 | 122,573 | 58,627 | 63,946 | 29,939 | 14,839 | 15,100 |
| Mukulu | 5,132 | 2,469 | 2,663 | 4,020 | 1,898 | 2,122 | 1,112 | 571 | 541 |
| Mwenyi | 4,595 | 2,082 | 2,513 | 4,308 | 1,969 | 2,339 | 287 | 113 | 174 |
| Namwanga | 351,895 | 173,031 | 178,864 | 163,296 | 80,470 | 82,826 | 188,599 | 92,561 | 96,038 |

## Table B4 ii. Population (De facto) by Sex and Ethnicity, Rural/Urban, Zambia 2010

| Ethnicity | Total | | | Rural | | | Urban | | |
|---|---|---|---|---|---|---|---|---|---|
| | Total | Male | Female | Total | Male | Female | Total | Male | Female |
| Ndembu | 13,978 | 6,858 | 7,120 | 10,511 | 5,179 | 5,332 | 3,467 | 1,679 | 1,788 |
| Ngoni | 503,276 | 247,561 | 255,715 | 235,912 | 117,180 | 118,732 | 267,364 | 130,381 | 136,983 |
| Ngumbo | 81,112 | 39,289 | 41,823 | 64,118 | 30,878 | 33,240 | 16,994 | 8,411 | 8,583 |
| Nkoya | 60,937 | 29,719 | 31,218 | 48,344 | 23,355 | 24,989 | 12,593 | 6,364 | 6,229 |
| Nsenga | 660,947 | 319,937 | 341,010 | 348,063 | 169,583 | 178,480 | 312,884 | 150,354 | 162,530 |
| Nyanja | 50,761 | 25,264 | 25,497 | 23,884 | 11,926 | 11,958 | 26,877 | 13,338 | 13,539 |
| Nyengo | 16,639 | 7,482 | 9,157 | 15,406 | 6,900 | 8,506 | 1,233 | 582 | 651 |
| Sala | 16,733 | 8,228 | 8,505 | 13,134 | 6,475 | 6,659 | 3,599 | 1,753 | 1,846 |
| Senga | 112,118 | 53,887 | 58,231 | 83,924 | 40,257 | 43,667 | 28,194 | 13,630 | 14,564 |
| Shila | 21,170 | 10,433 | 10,737 | 19,452 | 9,551 | 9,901 | 1,718 | 882 | 836 |
| Simaa | 4,996 | 2,198 | 2,798 | 4,868 | 2,135 | 2,733 | 128 | 63 | 65 |
| Soli | 87,778 | 42,545 | 45,233 | 49,009 | 23,951 | 25,058 | 38,769 | 18,594 | 20,175 |
| Subiya | 5,873 | 2,652 | 3,221 | 4,887 | 2,222 | 2,665 | 986 | 430 | 556 |
| Swaka | 43,830 | 21,249 | 22,581 | 33,285 | 16,201 | 17,084 | 10,545 | 5,048 | 5,497 |
| Tabwa | 90,730 | 44,066 | 46,664 | 77,816 | 37,670 | 40,146 | 12,914 | 6,396 | 6,518 |
| Tambo | 5,569 | 2,738 | 2,831 | 3,934 | 1,924 | 2,010 | 1,635 | 814 | 821 |
| Toka-Leya | 56,357 | 27,210 | 29,147 | 40,373 | 19,713 | 20,660 | 15,984 | 7,497 | 8,487 |
| Tonga | 1,698,108 | 826,995 | 871,113 | 1,258,939 | 615,322 | 643,617 | 439,169 | 211,673 | 227,496 |
| Totela | 8,983 | 4,234 | 4,749 | 7,347 | 3,413 | 3,934 | 1,636 | 821 | 815 |
| Tumbuka | 549,665 | 271,403 | 278,262 | 307,012 | 149,977 | 157,035 | 242,653 | 121,426 | 121,227 |
| Unga | 24,892 | 11,673 | 13,219 | 23,856 | 11,155 | 12,701 | 1,036 | 518 | 518 |
| Ushi | 233,590 | 113,771 | 119,819 | 145,108 | 70,455 | 74,653 | 88,482 | 43,316 | 45,166 |
| Wina | 2,572 | 1,263 | 1,309 | 1,712 | 840 | 872 | 860 | 423 | 437 |
| Yombe | 1,486 | 755 | 731 | 535 | 260 | 275 | 951 | 495 | 456 |
| English language | 2,741 | 1,433 | 1,308 | 957 | 507 | 450 | 1,784 | 926 | 858 |
| Other Language groups | 73,299 | 36,469 | 36,830 | 41,366 | 20,443 | 20,923 | 31,933 | 16,026 | 15,907 |
| Language Not Stated | 47,587 | 24,068 | 23,519 | 26,389 | 13,174 | 13,215 | 21,198 | 10,894 | 10,304 |
| African | 81,462 | 41,099 | 40,363 | 40,426 | 20,222 | 20,204 | 41,036 | 20,877 | 20,159 |
| American | 1,658 | 846 | 812 | 465 | 248 | 217 | 1,193 | 598 | 595 |
| Asian | 11,606 | 6,480 | 5,126 | 838 | 484 | 354 | 10,768 | 5,996 | 4,772 |
| European | 3,499 | 1,891 | 1,608 | 690 | 374 | 316 | 2,809 | 1,517 | 1,292 |

## Table B5: Population (De facto) by Religion, 5 Year Age Group and Sex, Rural/Urban, Zambia 2010

| Age Group and Sex | Total | Religion | | | | | | | |
|---|---|---|---|---|---|---|---|---|---|
| | | Catholic | Protestant | Muslim | Hindu | Buddist | Bahai faith | Other | None |
| **Zambia Total** | **12,526,314** | **2,532,858** | **9,436,231** | **61,412** | **4,383** | **9,623** | **3,891** | **253,621** | **224,295** |
| Male | 6,117,253 | 1,238,491 | 4,567,759 | 32,082 | 2,497 | 4,786 | 1,997 | 123,631 | 146,010 |
| Female | 6,409,061 | 1,294,367 | 4,868,472 | 29,330 | 1,886 | 4,837 | 1,894 | 129,990 | 78,285 |
| **0 - 4** | **2,214,887** | **417,196** | **1,706,918** | **10,202** | **373** | **1,788** | **696** | **44,485** | **33,229** |
| Male | 1,102,272 | 207,464 | 849,493 | 5,183 | 197 | 892 | 360 | 21,945 | 16,738 |
| Female | 1,112,615 | 209,732 | 857,425 | 5,019 | 176 | 896 | 336 | 22,540 | 16,491 |
| **5 - 9** | **1,856,336** | **362,683** | **1,423,050** | **8,427** | **327** | **1,423** | **566** | **37,392** | **22,468** |
| Male | 924,395 | 180,388 | 707,971 | 4,323 | 170 | 694 | 293 | 18,449 | 12,107 |
| Female | 931,941 | 182,295 | 715,079 | 4,104 | 157 | 729 | 273 | 18,943 | 10,361 |
| **10 - 14** | **1,699,042** | **346,602** | **1,291,103** | **8,070** | **277** | **1,285** | **539** | **33,374** | **17,792** |
| Male | 840,834 | 171,062 | 637,193 | 4,106 | 148 | 653 | 276 | 16,732 | 10,664 |
| Female | 858,208 | 175,540 | 653,910 | 3,964 | 129 | 632 | 263 | 16,642 | 7,128 |
| **15 - 19** | **1,427,884** | **300,508** | **1,074,558** | **6,526** | **211** | **1,035** | **413** | **28,071** | **16,562** |
| Male | 692,859 | 146,575 | 517,616 | 3,384 | 115 | 498 | 207 | 13,697 | 10,767 |
| Female | 735,025 | 153,933 | 556,942 | 3,142 | 96 | 537 | 206 | 14,374 | 5,795 |
| **20 - 24** | **1,117,476** | **226,668** | **842,947** | **5,088** | **234** | **850** | **318** | **22,596** | **18,775** |
| Male | 504,876 | 105,480 | 373,805 | 2,502 | 135 | 384 | 155 | 10,166 | 12,249 |
| Female | 612,600 | 121,188 | 469,142 | 2,586 | 99 | 466 | 163 | 12,430 | 6,526 |
| **25 - 29** | **1,006,244** | **197,307** | **758,639** | **4,666** | **421** | **830** | **271** | **20,816** | **23,294** |
| Male | 464,493 | 94,178 | 341,491 | 2,338 | 246 | 410 | 126 | 9,484 | 16,220 |
| Female | 541,751 | 103,129 | 417,148 | 2,328 | 175 | 420 | 145 | 11,332 | 7,074 |
| **30 - 34** | **800,770** | **153,007** | **604,419** | **4,036** | **486** | **637** | **226** | **16,594** | **21,365** |
| Male | 397,694 | 76,792 | 293,720 | 2,127 | 273 | 300 | 121 | 8,376 | 15,985 |
| Female | 403,076 | 76,215 | 310,699 | 1,909 | 213 | 337 | 105 | 8,218 | 5,380 |
| **35 - 39** | **647,813** | **126,074** | **484,462** | **3,618** | **425** | **502** | **186** | **13,696** | **18,850** |
| Male | 332,961 | 64,434 | 244,528 | 1,985 | 259 | 273 | 104 | 6,906 | 14,472 |
| Female | 314,852 | 61,640 | 239,934 | 1,633 | 166 | 229 | 82 | 6,790 | 4,378 |
| **40 - 44** | **447,584** | **91,181** | **330,249** | **2,700** | **346** | **386** | **139** | **9,397** | **13,186** |
| Male | 232,253 | 46,433 | 168,758 | 1,628 | 220 | 214 | 77 | 4,804 | 10,119 |
| Female | 215,331 | 44,748 | 161,491 | 1,072 | 126 | 172 | 62 | 4,593 | 3,067 |
| **45 - 49** | **354,989** | **76,626** | **257,930** | **2,139** | **276** | **299** | **129** | **7,370** | **10,220** |
| Male | 174,833 | 36,290 | 125,549 | 1,204 | 167 | 169 | 69 | 3,659 | 7,726 |
| Female | 180,156 | 40,336 | 132,381 | 935 | 109 | 130 | 60 | 3,711 | 2,494 |
| **50 - 54** | **270,254** | **62,945** | **191,814** | **1,666** | **282** | **194** | **106** | **5,640** | **7,607** |
| Male | 128,696 | 29,242 | 90,054 | 945 | 161 | 92 | 54 | 2,699 | 5,449 |
| Female | 141,558 | 33,703 | 101,760 | 721 | 121 | 102 | 52 | 2,941 | 2,158 |
| **55 - 59** | **184,828** | **45,438** | **128,916** | **1,064** | **228** | **122** | **73** | **3,847** | **5,140** |
| Male | 90,037 | 21,603 | 62,071 | 611 | 121 | 65 | 37 | 1,871 | 3,658 |
| Female | 94,791 | 23,835 | 66,845 | 453 | 107 | 57 | 36 | 1,976 | 1,482 |
| **60 - 64** | **161,875** | **41,550** | **111,132** | **958** | **199** | **86** | **82** | **3,340** | **4,528** |
| Male | 73,419 | 18,837 | 49,452 | 472 | 111 | 48 | 42 | 1,501 | 2,956 |
| Female | 88,456 | 22,713 | 61,680 | 486 | 88 | 38 | 40 | 1,839 | 1,572 |
| **65 - 69** | **118,582** | **30,875** | **80,853** | **705** | **121** | **73** | **49** | **2,430** | **3,476** |
| Male | 53,695 | 14,047 | 35,825 | 364 | 74 | 43 | 23 | 1,114 | 2,205 |
| Female | 64,887 | 16,828 | 45,028 | 341 | 47 | 30 | 26 | 1,316 | 1,271 |
| **70 - 74** | **90,802** | **23,165** | **61,973** | **618** | **80** | **44** | **30** | **1,954** | **2,938** |
| Male | 41,929 | 10,816 | 28,014 | 347 | 44 | 20 | 12 | 921 | 1,755 |
| Female | 48,873 | 12,349 | 33,959 | 271 | 36 | 24 | 18 | 1,033 | 1,183 |
| **75+** | **126,948** | **31,033** | **87,268** | **929** | **97** | **69** | **68** | **2,619** | **4,865** |
| Male | 62,007 | 14,850 | 42,219 | 563 | 56 | 31 | 41 | 1,307 | 2,940 |
| Female | 64,941 | 16,183 | 45,049 | 366 | 41 | 38 | 27 | 1,312 | 1,925 |

## Table B5: Population (De facto) by Religion, 5 Year Age Group and Sex, Rural/Urban, Zambia 2010

| Age Group and Sex | Total | Religion | | | | | | | |
|---|---|---|---|---|---|---|---|---|---|
| | | Catholic | Protestant | Muslim | Hindu | Buddist | Bahai faith | Other | None |
| **Zambia Rural** | **7,505,292** | **1,507,000** | **5,663,569** | **24,379** | **401** | **6,073** | **2,790** | **140,451** | **160,629** |
| Male | 3,664,349 | 732,370 | 2,748,526 | 12,341 | 212 | 2,969 | 1,448 | 68,229 | 98,254 |
| Female | 3,840,943 | 774,630 | 2,915,043 | 12,038 | 189 | 3,104 | 1,342 | 72,222 | 62,375 |
| **0 - 4** | **1,454,385** | **276,805** | **1,116,913** | **4,797** | **71** | **1,237** | **524** | **27,111** | **26,927** |
| Male | 724,158 | 137,573 | 556,194 | 2,457 | 37 | 610 | 271 | 13,452 | 13,564 |
| Female | 730,227 | 139,232 | 560,719 | 2,340 | 34 | 627 | 253 | 13,659 | 13,363 |
| **5 - 9** | **1,212,247** | **238,768** | **926,491** | **3,859** | **61** | **1,009** | **413** | **22,695** | **18,951** |
| Male | 608,241 | 119,450 | 464,710 | 1,920 | 30 | 498 | 211 | 11,189 | 10,233 |
| Female | 604,006 | 119,318 | 461,781 | 1,939 | 31 | 511 | 202 | 11,506 | 8,718 |
| **10 - 14** | **1,045,977** | **212,923** | **794,977** | **3,353** | **45** | **821** | **410** | **18,868** | **14,580** |
| Male | 529,978 | 107,413 | 401,773 | 1,727 | 24 | 414 | 217 | 9,695 | 8,715 |
| Female | 515,999 | 105,510 | 393,204 | 1,626 | 21 | 407 | 193 | 9,173 | 5,865 |
| **15 - 19** | **802,997** | **165,864** | **607,652** | **2,352** | **43** | **604** | **297** | **14,487** | **11,698** |
| Male | 397,738 | 82,055 | 299,325 | 1,260 | 20 | 284 | 154 | 7,222 | 7,418 |
| Female | 405,259 | 83,809 | 308,327 | 1,092 | 23 | 320 | 143 | 7,265 | 4,280 |
| **20 - 24** | **598,270** | **119,036** | **454,530** | **1,597** | **25** | **510** | **192** | **11,117** | **11,263** |
| Male | 269,368 | 53,891 | 202,769 | 760 | 13 | 228 | 96 | 4,973 | 6,638 |
| Female | 328,902 | 65,145 | 251,761 | 837 | 12 | 282 | 96 | 6,144 | 4,625 |
| **25 - 29** | **529,481** | **102,263** | **401,433** | **1,440** | **36** | **477** | **182** | **9,904** | **13,746** |
| Male | 241,136 | 47,226 | 179,975 | 628 | 18 | 238 | 85 | 4,442 | 8,524 |
| Female | 288,345 | 55,037 | 221,458 | 812 | 18 | 239 | 97 | 5,462 | 5,222 |
| **30 - 34** | **419,932** | **79,007** | **318,346** | **1,208** | **27** | **361** | **151** | **8,001** | **12,831** |
| Male | 203,507 | 38,250 | 151,697 | 588 | 18 | 158 | 76 | 3,879 | 8,841 |
| Female | 216,425 | 40,757 | 166,649 | 620 | 9 | 203 | 75 | 4,122 | 3,990 |
| **35 - 39** | **351,970** | **67,904** | **263,631** | **1,243** | **22** | **301** | **117** | **6,734** | **12,018** |
| Male | 173,650 | 32,964 | 128,115 | 607 | 13 | 157 | 67 | 3,201 | 8,526 |
| Female | 178,320 | 34,940 | 135,516 | 636 | 9 | 144 | 50 | 3,533 | 3,492 |
| **40 - 44** | **254,964** | **51,412** | **188,358** | **962** | **18** | **224** | **105** | **4,991** | **8,894** |
| Male | 127,044 | 24,963 | 92,522 | 520 | 11 | 117 | 61 | 2,422 | 6,428 |
| Female | 127,920 | 26,449 | 95,836 | 442 | 7 | 107 | 44 | 2,569 | 2,466 |
| **45 - 49** | **209,868** | **44,960** | **152,438** | **779** | **9** | **169** | **92** | **4,105** | **7,316** |
| Male | 100,903 | 20,950 | 72,171 | 416 | 4 | 96 | 48 | 1,960 | 5,258 |
| Female | 108,965 | 24,010 | 80,267 | 363 | 5 | 73 | 44 | 2,145 | 2,058 |
| **50 - 54** | **162,395** | **36,423** | **116,225** | **687** | **7** | **108** | **77** | **3,216** | **5,652** |
| Male | 75,868 | 16,866 | 53,270 | 349 | 4 | 44 | 41 | 1,507 | 3,787 |
| Female | 86,527 | 19,557 | 62,955 | 338 | 3 | 64 | 36 | 1,709 | 1,865 |
| **55 - 59** | **111,511** | **26,092** | **78,765** | **451** | **6** | **71** | **54** | **2,240** | **3,832** |
| Male | 52,225 | 11,885 | 36,419 | 231 | 2 | 36 | 25 | 1,052 | 2,575 |
| Female | 59,286 | 14,207 | 42,346 | 220 | 4 | 35 | 29 | 1,188 | 1,257 |
| **60 - 64** | **107,038** | **26,130** | **74,608** | **467** | **10** | **53** | **62** | **2,163** | **3,545** |
| Male | 45,761 | 11,108 | 31,247 | 215 | 5 | 27 | 33 | 935 | 2,191 |
| Female | 61,277 | 15,022 | 43,361 | 252 | 5 | 26 | 29 | 1,228 | 1,354 |
| **65 - 69** | **83,157** | **20,766** | **57,523** | **374** | **4** | **44** | **37** | **1,626** | **2,783** |
| Male | 36,611 | 9,126 | 24,862 | 185 | 2 | 25 | 19 | 708 | 1,684 |
| Female | 46,546 | 11,640 | 32,661 | 189 | 2 | 19 | 18 | 918 | 1,099 |
| **70 - 74** | **66,388** | **16,313** | **45,928** | **340** | **12** | **31** | **24** | **1,330** | **2,410** |
| Male | 30,553 | 7,589 | 20,740 | 190 | 7 | 16 | 10 | 632 | 1,369 |
| Female | 35,835 | 8,724 | 25,188 | 150 | 5 | 15 | 14 | 698 | 1,041 |
| **75+** | **94,712** | **22,334** | **65,751** | **470** | **5** | **53** | **53** | **1,863** | **4,183** |
| Male | 47,608 | 11,061 | 32,737 | 288 | 4 | 21 | 34 | 960 | 2,503 |
| Female | 47,104 | 11,273 | 33,014 | 182 | 1 | 32 | 19 | 903 | 1,680 |

## Table B5: Population (De facto) by Religion, 5 Year Age Group and Sex, Rural/Urban, Zambia 2010

| Age Group and Sex | Total | Religion | | | | | | | |
|---|---|---|---|---|---|---|---|---|---|
| | | Catholic | Protestant | Muslim | Hindu | Buddist | Bahai faith | Other | None |
| **Zambia Urban** | **5,021,022** | **1,025,858** | **3,772,662** | **37,033** | **3,982** | **3,550** | **1,101** | **113,170** | **63,666** |
| Male | 2,452,904 | 506,121 | 1,819,233 | 19,741 | 2,285 | 1,817 | 549 | 55,402 | 47,756 |
| Female | 2,568,118 | 519,737 | 1,953,429 | 17,292 | 1,697 | 1,733 | 552 | 57,768 | 15,910 |
| **0 - 4** | **760,502** | **140,391** | **590,005** | **5,405** | **302** | **551** | **172** | **17,374** | **6,302** |
| Male | 378,114 | 69,891 | 293,299 | 2,726 | 160 | 282 | 89 | 8,493 | 3,174 |
| Female | 382,388 | 70,500 | 296,706 | 2,679 | 142 | 269 | 83 | 8,881 | 3,128 |
| **5 - 9** | **644,089** | **123,915** | **496,559** | **4,568** | **266** | **414** | **153** | **14,697** | **3,517** |
| Male | 316,154 | 60,938 | 243,261 | 2,403 | 140 | 196 | 82 | 7,260 | 1,874 |
| Female | 327,935 | 62,977 | 253,298 | 2,165 | 126 | 218 | 71 | 7,437 | 1,643 |
| **10 - 14** | **653,065** | **133,679** | **496,126** | **4,717** | **232** | **464** | **129** | **14,506** | **3,212** |
| Male | 310,856 | 63,649 | 235,420 | 2,379 | 124 | 239 | 59 | 7,037 | 1,949 |
| Female | 342,209 | 70,030 | 260,706 | 2,338 | 108 | 225 | 70 | 7,469 | 1,263 |
| **15 - 19** | **624,887** | **134,644** | **466,906** | **4,174** | **168** | **431** | **116** | **13,584** | **4,864** |
| Male | 295,121 | 64,520 | 218,291 | 2,124 | 95 | 214 | 53 | 6,475 | 3,349 |
| Female | 329,766 | 70,124 | 248,615 | 2,050 | 73 | 217 | 63 | 7,109 | 1,515 |
| **20 - 24** | **519,206** | **107,632** | **388,417** | **3,491** | **209** | **340** | **126** | **11,479** | **7,512** |
| Male | 235,508 | 51,589 | 171,036 | 1,742 | 122 | 156 | 59 | 5,193 | 5,611 |
| Female | 283,698 | 56,043 | 217,381 | 1,749 | 87 | 184 | 67 | 6,286 | 1,901 |
| **25 - 29** | **476,763** | **95,044** | **357,206** | **3,226** | **385** | **353** | **89** | **10,912** | **9,548** |
| Male | 223,357 | 46,952 | 161,516 | 1,710 | 228 | 172 | 41 | 5,042 | 7,696 |
| Female | 253,406 | 48,092 | 195,690 | 1,516 | 157 | 181 | 48 | 5,870 | 1,852 |
| **30 - 34** | **380,838** | **74,000** | **286,073** | **2,828** | **459** | **276** | **75** | **8,593** | **8,534** |
| Male | 194,187 | 38,542 | 142,023 | 1,539 | 255 | 142 | 45 | 4,497 | 7,144 |
| Female | 186,651 | 35,458 | 144,050 | 1,289 | 204 | 134 | 30 | 4,096 | 1,390 |
| **35 - 39** | **295,843** | **58,170** | **220,831** | **2,375** | **403** | **201** | **69** | **6,962** | **6,832** |
| Male | 159,311 | 31,470 | 116,413 | 1,378 | 246 | 116 | 37 | 3,705 | 5,946 |
| Female | 136,532 | 26,700 | 104,418 | 997 | 157 | 85 | 32 | 3,257 | 886 |
| **40 - 44** | **192,620** | **39,769** | **141,891** | **1,738** | **328** | **162** | **34** | **4,406** | **4,292** |
| Male | 105,209 | 21,470 | 76,236 | 1,108 | 209 | 97 | 16 | 2,382 | 3,691 |
| Female | 87,411 | 18,299 | 65,655 | 630 | 119 | 65 | 18 | 2,024 | 601 |
| **45 - 49** | **145,121** | **31,666** | **105,492** | **1,360** | **267** | **130** | **37** | **3,265** | **2,904** |
| Male | 73,930 | 15,340 | 53,378 | 788 | 163 | 73 | 21 | 1,699 | 2,468 |
| Female | 71,191 | 16,326 | 52,114 | 572 | 104 | 57 | 16 | 1,566 | 436 |
| **50 - 54** | **107,859** | **26,522** | **75,589** | **979** | **275** | **86** | **29** | **2,424** | **1,955** |
| Male | 52,828 | 12,376 | 36,784 | 596 | 157 | 48 | 13 | 1,192 | 1,662 |
| Female | 55,031 | 14,146 | 38,805 | 383 | 118 | 38 | 16 | 1,232 | 293 |
| **55 - 59** | **73,317** | **19,346** | **50,151** | **613** | **222** | **51** | **19** | **1,607** | **1,308** |
| Male | 37,812 | 9,718 | 25,652 | 380 | 119 | 29 | 12 | 819 | 1,083 |
| Female | 35,505 | 9,628 | 24,499 | 233 | 103 | 22 | 7 | 788 | 225 |
| **60 - 64** | **54,837** | **15,420** | **36,524** | **491** | **189** | **33** | **20** | **1,177** | **983** |
| Male | 27,658 | 7,729 | 18,205 | 257 | 106 | 21 | 9 | 566 | 765 |
| Female | 27,179 | 7,691 | 18,319 | 234 | 83 | 12 | 11 | 611 | 218 |
| **65 - 69** | **35,425** | **10,109** | **23,330** | **331** | **117** | **29** | **12** | **804** | **693** |
| Male | 17,084 | 4,921 | 10,963 | 179 | 72 | 18 | 4 | 406 | 521 |
| Female | 18,341 | 5,188 | 12,367 | 152 | 45 | 11 | 8 | 398 | 172 |
| **70 - 74** | **24,414** | **6,852** | **16,045** | **278** | **68** | **13** | **6** | **624** | **528** |
| Male | 11,376 | 3,227 | 7,274 | 157 | 37 | 4 | 2 | 289 | 386 |
| Female | 13,038 | 3,625 | 8,771 | 121 | 31 | 9 | 4 | 335 | 142 |
| **75+** | **32,236** | **8,699** | **21,517** | **459** | **92** | **16** | **15** | **756** | **682** |
| Male | 14,399 | 3,789 | 9,482 | 275 | 52 | 10 | 7 | 347 | 437 |
| Female | 17,837 | 4,910 | 12,035 | 184 | 40 | 6 | 8 | 409 | 245 |

## Table B6: Population (De Facto) Aged 0-17 Years by 5 Year Age Group; Survival Status of Parents; Province and Rural/Urban, Zambia 2010

| Province and Age Group | Population 0 - 17 years | Survival Status of Mother | | | Survival Status of Father | | |
|---|---|---|---|---|---|---|---|
| | | Alive | Dead | Dont know | Alive | Dead | Don't know |
| **Zambia Total** | **6,638,301** | **6,184,229** | **321,206** | **132,866** | **5,815,404** | **723,982** | **98,915** |
| 0 - 4 | 2,214,887 | 2,138,331 | 35,348 | 41,208 | 2,085,275 | 106,637 | 22,975 |
| 5 - 9 | 1,856,336 | 1,761,799 | 67,807 | 26,730 | 1,671,696 | 165,758 | 18,882 |
| 10 - 14 | 1,699,042 | 1,546,101 | 121,891 | 31,050 | 1,410,864 | 262,256 | 25,922 |
| 15 - 17 | 868,036 | 737,998 | 96,160 | 33,878 | 647,569 | 189,331 | 31,136 |
| | | | | | | | |
| **Central** | **679,174** | **629,101** | **35,181** | **14,892** | **591,046** | **76,957** | **11,171** |
| 0 - 4 | 224,289 | 215,421 | 4,022 | 4,846 | 209,540 | 11,865 | 2,884 |
| 5 - 9 | 190,254 | 179,724 | 7,445 | 3,085 | 170,448 | 17,622 | 2,184 |
| 10 - 14 | 175,827 | 159,077 | 13,311 | 3,439 | 145,286 | 27,683 | 2,858 |
| 15 - 17 | 88,804 | 74,879 | 10,403 | 3,522 | 65,772 | 19,787 | 3,245 |
| | | | | | | | |
| **Copperbelt** | **943,568** | **867,787** | **57,883** | **17,898** | **804,672** | **125,053** | **13,843** |
| 0 - 4 | 288,184 | 277,785 | 5,390 | 5,009 | 269,401 | 15,798 | 2,985 |
| 5 - 9 | 248,404 | 233,655 | 11,162 | 3,587 | 219,349 | 26,409 | 2,646 |
| 10 - 14 | 258,545 | 231,426 | 22,569 | 4,550 | 207,809 | 46,843 | 3,893 |
| 15 - 17 | 148,435 | 124,921 | 18,762 | 4,752 | 108,113 | 36,003 | 4,319 |
| | | | | | | | |
| **Eastern** | **834,968** | **785,845** | **34,605** | **14,518** | **742,491** | **81,569** | **10,908** |
| 0 - 4 | 280,797 | 272,240 | 3,922 | 4,635 | 265,983 | 12,353 | 2,461 |
| 5 - 9 | 239,952 | 229,794 | 7,358 | 2,800 | 218,818 | 19,120 | 2,014 |
| 10 - 14 | 212,911 | 196,179 | 13,499 | 3,233 | 180,123 | 29,959 | 2,829 |
| 15 - 17 | 101,308 | 87,632 | 9,826 | 3,850 | 77,567 | 20,137 | 3,604 |
| | | | | | | | |
| **Luapula** | **520,045** | **486,535** | **24,276** | **9,234** | **460,299** | **53,846** | **5,900** |
| 0 - 4 | 178,815 | 172,622 | 3,158 | 3,035 | 167,741 | 9,716 | 1,358 |
| 5 - 9 | 149,455 | 141,638 | 6,022 | 1,795 | 134,724 | 13,693 | 1,038 |
| 10 - 14 | 130,510 | 119,410 | 8,978 | 2,122 | 110,387 | 18,601 | 1,522 |
| 15 - 17 | 61,265 | 52,865 | 6,118 | 2,282 | 47,447 | 11,836 | 1,982 |
| | | | | | | | |
| **Lusaka** | **1,019,184** | **941,850** | **55,807** | **21,527** | **875,909** | **126,725** | **16,550** |
| 0 - 4 | 330,249 | 319,242 | 5,042 | 5,965 | 311,283 | 15,588 | 3,378 |
| 5 - 9 | 274,057 | 259,639 | 10,141 | 4,277 | 244,975 | 26,066 | 3,016 |
| 10 - 14 | 267,942 | 240,912 | 21,555 | 5,475 | 215,877 | 47,384 | 4,681 |
| 15 - 17 | 146,936 | 122,057 | 19,069 | 5,810 | 103,774 | 37,687 | 5,475 |
| | | | | | | | |
| **Muchinga** | **380,193** | **356,405** | **15,610** | **8,178** | **338,634** | **36,400** | **5,159** |
| 0 - 4 | 130,097 | 125,421 | 1,919 | 2,757 | 123,290 | 5,665 | 1,142 |
| 5 - 9 | 111,376 | 105,811 | 3,888 | 1,677 | 101,124 | 9,271 | 981 |
| 10 - 14 | 94,047 | 86,518 | 5,808 | 1,721 | 79,874 | 12,920 | 1,253 |
| 15 - 17 | 44,673 | 38,655 | 3,995 | 2,023 | 34,346 | 8,544 | 1,783 |
| | | | | | | | |
| **Northern** | **587,352** | **552,758** | **22,850** | **11,744** | **525,505** | **54,516** | **7,331** |
| 0 - 4 | 205,958 | 199,103 | 3,030 | 3,825 | 195,095 | 9,218 | 1,645 |
| 5 - 9 | 169,675 | 161,872 | 5,496 | 2,307 | 154,561 | 13,796 | 1,318 |
| 10 - 14 | 144,083 | 133,127 | 8,444 | 2,512 | 123,298 | 19,109 | 1,676 |
| 15 - 17 | 67,636 | 58,656 | 5,880 | 3,100 | 52,551 | 12,393 | 2,692 |

## Table B6: Population (De Facto) Aged 0-17 Years by 5 Year Age Group; Survival Status of Parents; Province and Rural/Urban, Zambia 2010

| Province and Age Group | Population 0 - 17 years | Survival Status of Mother | | | Survival Status of Father | | |
|---|---|---|---|---|---|---|---|
| | | Alive | Dead | Dont know | Alive | Dead | Don't know |
| **North Western** | **384,686** | **362,834** | **12,586** | **9,266** | **346,942** | **30,877** | **6,867** |
| 0 - 4 | 132,975 | 128,491 | 1,497 | 2,987 | 125,726 | 5,410 | 1,839 |
| 5 - 9 | 109,165 | 104,315 | 2,823 | 2,027 | 100,283 | 7,482 | 1,400 |
| 10 - 14 | 95,597 | 88,842 | 4,595 | 2,160 | 83,353 | 10,475 | 1,769 |
| 15 - 17 | 46,949 | 41,186 | 3,671 | 2,092 | 37,580 | 7,510 | 1,859 |
| | | | | | | | |
| **Southern** | **839,690** | **783,566** | **38,310** | **17,814** | **742,861** | **82,571** | **14,258** |
| 0 - 4 | 285,543 | 275,780 | 4,012 | 5,751 | 270,754 | 11,294 | 3,495 |
| 5 - 9 | 236,602 | 224,975 | 7,941 | 3,686 | 215,127 | 18,558 | 2,917 |
| 10 - 14 | 209,728 | 191,354 | 14,381 | 3,993 | 175,923 | 30,157 | 3,648 |
| 15 - 17 | 107,817 | 91,457 | 11,976 | 4,384 | 81,057 | 22,562 | 4,198 |
| | | | | | | | |
| **Western** | **449,441** | **417,548** | **24,098** | **7,795** | **387,045** | **55,468** | **6,928** |
| 0 - 4 | 157,980 | 152,226 | 3,356 | 2,398 | 146,462 | 9,730 | 1,788 |
| 5 - 9 | 127,396 | 120,376 | 5,531 | 1,489 | 112,287 | 13,741 | 1,368 |
| 10 - 14 | 109,852 | 99,256 | 8,751 | 1,845 | 88,934 | 19,125 | 1,793 |
| 15 - 17 | 54,213 | 45,690 | 6,460 | 2,063 | 39,362 | 12,872 | 1,979 |

## Table B6: Population (De Facto) Aged 0-17 Years by 5 Year Age Group; Survival Status of Parents; Province and Rural/Urban, Zambia 2010

| Province and Age Group | Population 0 - 17 years | Survival Status of Mother | | | Survival Status of Father | | |
|---|---|---|---|---|---|---|---|
| | | Alive | Dead | Dont know | Alive | Dead | Don't know |
| **Zambia Rural** | **4,208,117** | **3,950,554** | **177,920** | **79,643** | **3,745,986** | **403,873** | **58,258** |
| 0 - 4 | 1,454,385 | 1,405,703 | 22,424 | 26,258 | 1,373,280 | 66,835 | 14,270 |
| 5 - 9 | 1,212,247 | 1,154,866 | 41,222 | 16,159 | 1,101,030 | 99,809 | 11,408 |
| 10 - 14 | 1,045,977 | 961,613 | 66,764 | 17,600 | 888,157 | 143,146 | 14,674 |
| 15 - 17 | 495,508 | 428,372 | 47,510 | 19,626 | 383,519 | 94,083 | 17,906 |
| | | | | | | | |
| **Central** | **522,248** | **486,371** | **24,890** | **10,987** | **460,485** | **53,493** | **8,270** |
| 0 - 4 | 177,233 | 170,483 | 3,101 | 3,649 | 166,268 | 8,791 | 2,174 |
| 5 - 9 | 149,075 | 141,257 | 5,497 | 2,321 | 134,547 | 12,837 | 1,691 |
| 10 - 14 | 132,583 | 120,732 | 9,379 | 2,472 | 111,411 | 19,106 | 2,066 |
| 15 - 17 | 63,357 | 53,899 | 6,913 | 2,545 | 48,259 | 12,759 | 2,339 |
| | | | | | | | |
| **Copperbelt** | **193,493** | **180,309** | **9,602** | **3,582** | **169,730** | **21,091** | **2,672** |
| 0 - 4 | 63,624 | 61,264 | 1,195 | 1,165 | 59,632 | 3,358 | 634 |
| 5 - 9 | 55,166 | 52,184 | 2,220 | 762 | 49,355 | 5,253 | 558 |
| 10 - 14 | 50,152 | 45,734 | 3,614 | 804 | 41,966 | 7,497 | 689 |
| 15 - 17 | 24,551 | 21,127 | 2,573 | 851 | 18,777 | 4,983 | 791 |
| | | | | | | | |
| **Eastern** | **738,526** | **696,880** | **29,350** | **12,296** | **659,718** | **69,614** | **9,194** |
| 0 - 4 | 250,475 | 243,054 | 3,435 | 3,986 | 237,527 | 10,857 | 2,091 |
| 5 - 9 | 213,530 | 204,770 | 6,424 | 2,336 | 195,175 | 16,664 | 1,691 |
| 10 - 14 | 187,059 | 172,911 | 11,447 | 2,701 | 159,194 | 25,469 | 2,396 |
| 15 - 17 | 87,462 | 76,145 | 8,044 | 3,273 | 67,822 | 16,624 | 3,016 |
| | | | | | | | |
| **Luapula** | **423,749** | **397,993** | **18,623** | **7,133** | **378,062** | **41,251** | **4,436** |
| 0 - 4 | 148,488 | 143,534 | 2,542 | 2,412 | 139,736 | 7,730 | 1,022 |
| 5 - 9 | 122,910 | 116,743 | 4,772 | 1,395 | 111,255 | 10,873 | 782 |
| 10 - 14 | 104,481 | 96,058 | 6,837 | 1,586 | 89,371 | 13,998 | 1,112 |
| 15 - 17 | 47,870 | 41,658 | 4,472 | 1,740 | 37,700 | 8,650 | 1,520 |
| | | | | | | | |
| **Lusaka** | **169,038** | **156,764** | **8,329** | **3,945** | **147,448** | **18,695** | **2,895** |
| 0 - 4 | 55,680 | 53,710 | 823 | 1,147 | 52,493 | 2,527 | 660 |
| 5 - 9 | 47,160 | 44,683 | 1,645 | 832 | 42,505 | 4,076 | 579 |
| 10 - 14 | 44,071 | 39,750 | 3,303 | 1,018 | 36,158 | 7,072 | 841 |
| 15 - 17 | 22,127 | 18,621 | 2,558 | 948 | 16,292 | 5,020 | 815 |
| | | | | | | | |
| **Muchinga** | **319,849** | **300,742** | **12,492** | **6,615** | **286,721** | **29,065** | **4,063** |
| 0 - 4 | 110,413 | 106,578 | 1,580 | 2,255 | 104,873 | 4,645 | 895 |
| 5 - 9 | 94,562 | 89,962 | 3,227 | 1,373 | 86,182 | 7,607 | 773 |
| 10 - 14 | 78,518 | 72,497 | 4,640 | 1,381 | 67,268 | 10,259 | 991 |
| 15 - 17 | 36,356 | 31,705 | 3,045 | 1,606 | 28,398 | 6,554 | 1,404 |
| | | | | | | | |
| **Northern** | **485,922** | **459,016** | **17,718** | **9,188** | **438,413** | **41,876** | **5,633** |
| 0 - 4 | 173,968 | 168,387 | 2,494 | 3,087 | 165,152 | 7,547 | 1,269 |
| 5 - 9 | 141,524 | 135,275 | 4,467 | 1,782 | 129,542 | 10,960 | 1,022 |
| 10 - 14 | 117,198 | 108,833 | 6,494 | 1,871 | 101,487 | 14,479 | 1,232 |
| 15 - 17 | 53,232 | 46,521 | 4,263 | 2,448 | 42,232 | 8,890 | 2,110 |

## Table B6: Population (De Facto) Aged 0-17 Years by 5 Year Age Group; Survival Status of Parents; Province and Rural/Urban, Zambia 2010

| Province and Age Group | Population 0 - 17 years | Survival Status of Mother | | | Survival Status of Father | | |
|---|---|---|---|---|---|---|---|
| | | Alive | Dead | Dont know | Alive | Dead | Don't know |
| **North Western** | **302,697** | **286,909** | **9,153** | **6,635** | **274,551** | **23,171** | **4,975** |
| 0 - 4 | 105,731 | 102,298 | 1,195 | 2,238 | 100,089 | 4,259 | 1,383 |
| 5 - 9 | 87,038 | 83,426 | 2,149 | 1,463 | 80,195 | 5,817 | 1,026 |
| 10 - 14 | 74,508 | 69,712 | 3,311 | 1,485 | 65,446 | 7,801 | 1,261 |
| 15 - 17 | 35,420 | 31,473 | 2,498 | 1,449 | 28,821 | 5,294 | 1,305 |
| | | | | | | | |
| **Southern** | **659,128** | **618,642** | **27,676** | **12,810** | **589,871** | **58,882** | **10,375** |
| 0 - 4 | 228,258 | 220,854 | 3,140 | 4,264 | 217,034 | 8,611 | 2,613 |
| 5 - 9 | 188,394 | 179,690 | 6,059 | 2,645 | 172,470 | 13,782 | 2,142 |
| 10 - 14 | 162,652 | 149,395 | 10,456 | 2,801 | 138,524 | 21,488 | 2,640 |
| 15 - 17 | 79,824 | 68,703 | 8,021 | 3,100 | 61,843 | 15,001 | 2,980 |
| | | | | | | | |
| ***Western*** | ***393,467*** | ***366,928*** | ***20,087*** | ***6,452*** | ***340,987*** | ***46,735*** | ***5,745*** |
| 0 - 4 | 140,515 | 135,541 | 2,919 | 2,055 | 130,476 | 8,510 | 1,529 |
| 5 - 9 | 112,888 | 106,876 | 4,762 | 1,250 | 99,804 | 11,940 | 1,144 |
| 10 - 14 | 94,755 | 85,991 | 7,283 | 1,481 | 77,332 | 15,977 | 1,446 |
| 15 - 17 | 45,309 | 38,520 | 5,123 | 1,666 | 33,375 | 10,308 | 1,626 |

## Table B6: Population (De Facto) Aged 0-17 Years by 5 Year Age Group; Survival Status of Parents; Province and Rural/Urban, Zambia 2010

| Province and Age Group | Population 0 - 17 years | Survival Status of Mother | | | Survival Status of Father | | |
|---|---|---|---|---|---|---|---|
| | | Alive | Dead | Dont know | Alive | Dead | Don't know |
| **Zambia Urban** | **2,430,184** | **2,233,675** | **143,286** | **53,223** | **2,069,418** | **320,109** | **40,657** |
| 0 - 4 | 760,502 | 732,628 | 12,924 | 14,950 | 711,995 | 39,802 | 8,705 |
| 5 - 9 | 644,089 | 606,933 | 26,585 | 10,571 | 570,666 | 65,949 | 7,474 |
| 10 - 14 | 653,065 | 584,488 | 55,127 | 13,450 | 522,707 | 119,110 | 11,248 |
| 15 - 17 | 372,528 | 309,626 | 48,650 | 14,252 | 264,050 | 95,248 | 13,230 |
| | | | | | | | |
| **Central** | **156,926** | **142,730** | **10,291** | **3,905** | **130,561** | **23,464** | **2,901** |
| 0 - 4 | 47,056 | 44,938 | 921 | 1,197 | 43,272 | 3,074 | 710 |
| 5 - 9 | 41,179 | 38,467 | 1,948 | 764 | 35,901 | 4,785 | 493 |
| 10 - 14 | 43,244 | 38,345 | 3,932 | 967 | 33,875 | 8,577 | 792 |
| 15 - 17 | 25,447 | 20,980 | 3,490 | 977 | 17,513 | 7,028 | 906 |
| | | | | | | | |
| **Copperbelt** | **750,075** | **687,478** | **48,281** | **14,316** | **634,942** | **103,962** | **11,171** |
| 0 - 4 | 224,560 | 216,521 | 4,195 | 3,844 | 209,769 | 12,440 | 2,351 |
| 5 - 9 | 193,238 | 181,471 | 8,942 | 2,825 | 169,994 | 21,156 | 2,088 |
| 10 - 14 | 208,393 | 185,692 | 18,955 | 3,746 | 165,843 | 39,346 | 3,204 |
| 15 - 17 | 123,884 | 103,794 | 16,189 | 3,901 | 89,336 | 31,020 | 3,528 |
| | | | | | | | |
| **Eastern** | **96,442** | **88,965** | **5,255** | **2,222** | **82,773** | **11,955** | **1,714** |
| 0 - 4 | 30,322 | 29,186 | 487 | 649 | 28,456 | 1,496 | 370 |
| 5 - 9 | 26,422 | 25,024 | 934 | 464 | 23,643 | 2,456 | 323 |
| 10 - 14 | 25,852 | 23,268 | 2,052 | 532 | 20,929 | 4,490 | 433 |
| 15 - 17 | 13,846 | 11,487 | 1,782 | 577 | 9,745 | 3,513 | 588 |
| | | | | | | | |
| **Luapula** | **96,296** | **88,542** | **5,653** | **2,101** | **82,237** | **12,595** | **1,464** |
| 0 - 4 | 30,327 | 29,088 | 616 | 623 | 28,005 | 1,986 | 336 |
| 5 - 9 | 26,545 | 24,895 | 1,250 | 400 | 23,469 | 2,820 | 256 |
| 10 - 14 | 26,029 | 23,352 | 2,141 | 536 | 21,016 | 4,603 | 410 |
| 15 - 17 | 13,395 | 11,207 | 1,646 | 542 | 9,747 | 3,186 | 462 |
| | | | | | | | |
| **Lusaka** | **850,146** | **785,086** | **47,478** | **17,582** | **728,461** | **108,030** | **13,655** |
| 0 - 4 | 274,569 | 265,532 | 4,219 | 4,818 | 258,790 | 13,061 | 2,718 |
| 5 - 9 | 226,897 | 214,956 | 8,496 | 3,445 | 202,470 | 21,990 | 2,437 |
| 10 - 14 | 223,871 | 201,162 | 18,252 | 4,457 | 179,719 | 40,312 | 3,840 |
| 15 - 17 | 124,809 | 103,436 | 16,511 | 4,862 | 87,482 | 32,667 | 4,660 |
| | | | | | | | |
| **Muchinga** | **60,344** | **55,663** | **3,118** | **1,563** | **51,913** | **7,335** | **1,096** |
| 0 - 4 | 19,684 | 18,843 | 339 | 502 | 18,417 | 1,020 | 247 |
| 5 - 9 | 16,814 | 15,849 | 661 | 304 | 14,942 | 1,664 | 208 |
| 10 - 14 | 15,529 | 14,021 | 1,168 | 340 | 12,606 | 2,661 | 262 |
| 15 - 17 | 8,317 | 6,950 | 950 | 417 | 5,948 | 1,990 | 379 |
| | | | | | | | |
| **Northern** | **101,430** | **93,742** | **5,132** | **2,556** | **87,092** | **12,640** | **1,698** |
| 0 - 4 | 31,990 | 30,716 | 536 | 738 | 29,943 | 1,671 | 376 |
| 5 - 9 | 28,151 | 26,597 | 1,029 | 525 | 25,019 | 2,836 | 296 |
| 10 - 14 | 26,885 | 24,294 | 1,950 | 641 | 21,811 | 4,630 | 444 |
| 15 - 17 | 14,404 | 12,135 | 1,617 | 652 | 10,319 | 3,503 | 582 |

| Table B6: Population (De Facto) Aged 0-17 Years by 5 Year Age Group; Survival Status of Parents; Province and Rural/Urban, Zambia 2010 | | | | | | | |
|---|---|---|---|---|---|---|---|
| Province and Age Group | Population 0 - 17 years | Survival Status of Mother | | | Survival Status of Father | | |
| | | Alive | Dead | Dont know | Alive | Dead | Don't know |
| **North Western** | **81,989** | **75,925** | **3,433** | **2,631** | **72,391** | **7,706** | **1,892** |
| 0 - 4 | 27,244 | 26,193 | 302 | 749 | 25,637 | 1,151 | 456 |
| 5 - 9 | 22,127 | 20,889 | 674 | 564 | 20,088 | 1,665 | 374 |
| 10 - 14 | 21,089 | 19,130 | 1,284 | 675 | 17,907 | 2,674 | 508 |
| 15 - 17 | 11,529 | 9,713 | 1,173 | 643 | 8,759 | 2,216 | 554 |
| | | | | | | | |
| **Southern** | **180,562** | **164,924** | **10,634** | **5,004** | **152,990** | **23,689** | **3,883** |
| 0 - 4 | 57,285 | 54,926 | 872 | 1,487 | 53,720 | 2,683 | 882 |
| 5 - 9 | 48,208 | 45,285 | 1,882 | 1,041 | 42,657 | 4,776 | 775 |
| 10 - 14 | 47,076 | 41,959 | 3,925 | 1,192 | 37,399 | 8,669 | 1,008 |
| 15 - 17 | 27,993 | 22,754 | 3,955 | 1,284 | 19,214 | 7,561 | 1,218 |
| | | | | | | | |
| **Western** | **55,974** | **50,620** | **4,011** | **1,343** | **46,058** | **8,733** | **1,183** |
| 0 - 4 | 17,465 | 16,685 | 437 | 343 | 15,986 | 1,220 | 259 |
| 5 - 9 | 14,508 | 13,500 | 769 | 239 | 12,483 | 1,801 | 224 |
| 10 - 14 | 15,097 | 13,265 | 1,468 | 364 | 11,602 | 3,148 | 347 |
| 15 - 17 | 8,904 | 7,170 | 1,337 | 397 | 5,987 | 2,564 | 353 |

## Table B7: Population (De Facto) Aged 0-17 Years by 5 Year Age Group; Survival Status of Parents; Province and Rural/Urban, Zambia 2010

| Province and Age Group | Survival Status of Parents | | | | | | | | | |
|---|---|---|---|---|---|---|---|---|---|---|
| | Population 0 - 17 Years | Both Parents Alive | Mother Alive, Father Dead | Father Alive, Mother Dead | Both Parents Dead | Father Dead, Status of Mother Unknown | Mother Dead, Status of Father Unknown | Status of Both Parents Unknown | Father Alive, Status of Mother Unknown | Mother Alive, Status of Father Unknown |
| **Zambia Total** | **6,638,301** | **5,608,343** | **536,282** | **130,482** | **184,546** | **3,154** | **6,178** | **53,133** | **76,579** | **39,604** |
| 0 - 4 | 2,214,887 | 2,040,240 | 88,075 | 16,532 | 18,085 | 477 | 731 | 12,228 | 28,503 | 10,016 |
| 5 - 9 | 1,856,336 | 1,622,764 | 129,660 | 31,091 | 35,354 | 744 | 1,362 | 8,145 | 17,841 | 9,375 |
| 10 - 14 | 1,699,042 | 1,344,079 | 190,505 | 48,976 | 70,626 | 1,125 | 2,289 | 12,116 | 17,809 | 11,517 |
| 15 - 17 | 868,036 | 601,260 | 128,042 | 33,883 | 60,481 | 808 | 1,796 | 20,644 | 12,426 | 8,696 |
| | | | | | | | | | | |
| **Central** | **679,174** | **569,142** | **55,612** | **13,424** | **20,897** | **448** | **860** | **5,964** | **8,480** | **4,347** |
| 0 - 4 | 224,289 | 204,709 | 9,579 | 1,695 | 2,226 | 60 | 101 | 1,650 | 3,136 | 1,133 |
| 5 - 9 | 190,254 | 165,255 | 13,415 | 3,163 | 4,092 | 115 | 190 | 940 | 2,030 | 1,054 |
| 10 - 14 | 175,827 | 138,216 | 19,629 | 5,084 | 7,907 | 147 | 320 | 1,306 | 1,986 | 1,232 |
| 15 - 17 | 88,804 | 60,962 | 12,989 | 3,482 | 6,672 | 126 | 249 | 2,068 | 1,328 | 928 |
| | | | | | | | | | | |
| **Copperbelt** | **943,568** | **771,912** | **90,447** | **22,763** | **34,216** | **390** | **904** | **7,511** | **9,997** | **5,428** |
| 0 - 4 | 288,184 | 263,523 | 13,004 | 2,543 | 2,741 | 53 | 106 | 1,621 | 3,335 | 1,258 |
| 5 - 9 | 248,404 | 211,994 | 20,447 | 5,098 | 5,876 | 86 | 188 | 1,244 | 2,257 | 1,214 |
| 10 - 14 | 258,545 | 196,613 | 33,174 | 8,726 | 13,520 | 149 | 323 | 1,931 | 2,470 | 1,639 |
| 15 - 17 | 148,435 | 99,782 | 23,822 | 6,396 | 12,079 | 102 | 287 | 2,715 | 1,935 | 1,317 |
| | | | | | | | | | | |
| **Eastern** | **834,968** | **720,820** | **60,647** | **13,406** | **20,549** | **373** | **650** | **5,880** | **8,265** | **4,378** |
| 0 - 4 | 280,797 | 260,977 | 10,227 | 1,790 | 2,058 | 68 | 74 | 1,351 | 3,216 | 1,036 |
| 5 - 9 | 239,952 | 213,706 | 15,108 | 3,282 | 3,926 | 86 | 150 | 884 | 1,830 | 980 |
| 10 - 14 | 212,911 | 173,198 | 21,637 | 5,060 | 8,208 | 114 | 231 | 1,254 | 1,865 | 1,344 |
| 15 - 17 | 101,308 | 72,939 | 13,675 | 3,274 | 6,357 | 105 | 195 | 2,391 | 1,354 | 1,018 |
| | | | | | | | | | | |
| **Luapula** | **520,045** | **445,023** | **39,223** | **9,488** | **14,395** | **228** | **393** | **3,218** | **5,788** | **2,289** |
| 0 - 4 | 178,815 | 164,030 | 7,975 | 1,397 | 1,700 | 41 | 61 | 680 | 2,314 | 617 |
| 5 - 9 | 149,455 | 130,890 | 10,247 | 2,533 | 3,396 | 50 | 93 | 444 | 1,301 | 501 |
| 10 - 14 | 130,510 | 105,666 | 13,101 | 3,424 | 5,405 | 95 | 149 | 730 | 1,297 | 643 |
| 15 - 17 | 61,265 | 44,437 | 7,900 | 2,134 | 3,894 | 42 | 90 | 1,364 | 876 | 528 |
| | | | | | | | | | | |
| **Lusaka** | **1,019,184** | **841,133** | **94,350** | **22,743** | **31,906** | **469** | **1,158** | **9,025** | **12,033** | **6,367** |
| 0 - 4 | 330,249 | 304,671 | 13,176 | 2,581 | 2,350 | 62 | 111 | 1,872 | 4,031 | 1,395 |
| 5 - 9 | 274,057 | 237,203 | 20,990 | 4,938 | 4,975 | 101 | 228 | 1,342 | 2,834 | 1,446 |
| 10 - 14 | 267,942 | 204,138 | 34,842 | 8,716 | 12,367 | 175 | 472 | 2,277 | 3,023 | 1,932 |
| 15 - 17 | 146,936 | 95,121 | 25,342 | 6,508 | 12,214 | 131 | 347 | 3,534 | 2,145 | 1,594 |
| | | | | | | | | | | |
| **Muchinga** | **380,193** | **327,638** | **26,673** | **5,834** | **9,519** | **208** | **257** | **2,808** | **5,162** | **2,094** |
| 0 - 4 | 130,097 | 120,290 | 4,572 | 817 | 1,067 | 26 | 35 | 548 | 2,183 | 559 |
| 5 - 9 | 111,376 | 98,276 | 7,015 | 1,626 | 2,198 | 58 | 64 | 397 | 1,222 | 520 |
| 10 - 14 | 94,047 | 76,671 | 9,257 | 2,130 | 3,590 | 73 | 88 | 575 | 1,073 | 590 |
| 15 - 17 | 44,673 | 32,401 | 5,829 | 1,261 | 2,664 | 51 | 70 | 1,288 | 684 | 425 |
| | | | | | | | | | | |
| **Northern** | **587,352** | **509,499** | **40,469** | **8,673** | **13,791** | **256** | **386** | **4,155** | **7,333** | **2,790** |
| 0 - 4 | 205,958 | 190,817 | 7,508 | 1,303 | 1,664 | 46 | 63 | 804 | 2,975 | 778 |
| 5 - 9 | 169,675 | 150,588 | 10,590 | 2,271 | 3,142 | 64 | 83 | 541 | 1,702 | 694 |
| 10 - 14 | 144,083 | 118,520 | 13,838 | 3,126 | 5,177 | 94 | 141 | 766 | 1,652 | 769 |
| 15 - 17 | 67,636 | 49,574 | 8,533 | 1,973 | 3,808 | 52 | 99 | 2,044 | 1,004 | 549 |

## Table B7: Population (De Facto) Aged 0-17 Years by 5 Year Age Group; Survival Status of Parents; Province and Rural/Urban, Zambia 2010

| Province and Age Group | Survival Status of Parents | | | | | | | | | |
|---|---|---|---|---|---|---|---|---|---|---|
| | Population 0 - 17 Years | Both Parents Alive | Mother Alive, Father Dead | Father Alive, Mother Dead | Both Parents Dead | Father Dead, Status of Mother Unknown | Mother Dead, Status of Father Unknown | Status of Both Parents Unknown | Father Alive, Status of Mother Unknown | Mother Alive, Status of Father Unknown |
| **North Western** | **384,686** | **335,321** | **24,447** | **6,027** | **6,261** | **169** | **298** | **3,503** | **5,594** | **3,066** |
| 0 - 4 | 132,975 | 122,860 | 4,704 | 784 | 679 | 27 | 34 | 878 | 2,082 | 927 |
| 5 - 9 | 109,165 | 97,420 | 6,146 | 1,465 | 1,292 | 44 | 66 | 585 | 1,398 | 749 |
| 10 - 14 | 95,597 | 79,840 | 8,162 | 2,241 | 2,252 | 61 | 102 | 827 | 1,272 | 840 |
| 15 - 17 | 46,949 | 35,201 | 5,435 | 1,537 | 2,038 | 37 | 96 | 1,213 | 842 | 550 |
| | | | | | | | | | | |
| **Southern** | **839,690** | **716,321** | **61,394** | **16,650** | **20,791** | **386** | **869** | **7,538** | **9,890** | **5,851** |
| 0 - 4 | 285,543 | 265,044 | 9,252 | 1,945 | 1,980 | 62 | 87 | 1,924 | 3,765 | 1,484 |
| 5 - 9 | 236,602 | 208,851 | 14,616 | 3,886 | 3,850 | 92 | 205 | 1,204 | 2,390 | 1,508 |
| 10 - 14 | 209,728 | 167,444 | 22,246 | 6,273 | 7,782 | 129 | 326 | 1,658 | 2,206 | 1,664 |
| 15 - 17 | 107,817 | 74,982 | 15,280 | 4,546 | 7,179 | 103 | 251 | 2,752 | 1,529 | 1,195 |
| | | | | | | | | | | |
| **Western** | **449,441** | **371,534** | **43,020** | **11,474** | **12,221** | **227** | **403** | **3,531** | **4,037** | **2,994** |
| 0 - 4 | 157,980 | 143,319 | 8,078 | 1,677 | 1,620 | 32 | 59 | 900 | 1,466 | 829 |
| 5 - 9 | 127,396 | 108,581 | 11,086 | 2,829 | 2,607 | 48 | 95 | 564 | 877 | 709 |
| 10 - 14 | 109,852 | 83,773 | 14,619 | 4,196 | 4,418 | 88 | 137 | 792 | 965 | 864 |
| 15 - 17 | 54,213 | 35,861 | 9,237 | 2,772 | 3,576 | 59 | 112 | 1,275 | 729 | 592 |

## Table B7: Population (De Facto) Aged 0-17 Years by 5 Year Age Group; Survival Status of Parents; Province and Rural/Urban, Zambia 2010

| Province and Age Group | Survival Status of Parents | | | | | | | | | |
|---|---|---|---|---|---|---|---|---|---|---|
| | Population 0 - 17 Years | Both Parents Alive | Mother Alive, Father Dead | Father Alive, Mother Dead | Both Parents Dead | Father Dead, Status of Mother Unknown | Mother Dead, Status of Father Unknown | Status of Both Parents Unknown | Father Alive, Status of Mother Unknown | Mother Alive, Status of Father Unknown |
| **Zambia Rural** | **4,208,117** | **3,625,407** | **300,811** | **73,288** | **100,998** | **2,064** | **3,634** | **30,288** | **47,291** | **24,336** |
| 0 - 4 | 1,454,385 | 1,344,374 | 54,779 | 10,237 | 11,717 | 339 | 470 | 7,250 | 18,669 | 6,550 |
| 5 - 9 | 1,212,247 | 1,071,408 | 77,522 | 18,600 | 21,774 | 513 | 848 | 4,624 | 11,022 | 5,936 |
| 10 - 14 | 1,045,977 | 850,523 | 104,139 | 27,161 | 38,292 | 715 | 1,311 | 6,412 | 10,473 | 6,951 |
| 15 - 17 | 495,508 | 359,102 | 64,371 | 17,290 | 29,215 | 497 | 1,005 | 12,002 | 7,127 | 4,899 |
| **Central** | **522,248** | **444,464** | **38,619** | **9,678** | **14,546** | **328** | **666** | **4,316** | **6,343** | **3,288** |
| 0 - 4 | 177,233 | 162,586 | 7,035 | 1,304 | 1,717 | 39 | 80 | 1,232 | 2,378 | 862 |
| 5 - 9 | 149,075 | 130,663 | 9,752 | 2,357 | 2,994 | 91 | 146 | 703 | 1,527 | 842 |
| 10 - 14 | 132,583 | 106,308 | 13,497 | 3,630 | 5,505 | 104 | 244 | 895 | 1,473 | 927 |
| 15 - 17 | 63,357 | 44,907 | 8,335 | 2,387 | 4,330 | 94 | 196 | 1,486 | 965 | 657 |
| **Copperbelt** | **193,493** | **163,926** | **15,205** | **3,657** | **5,756** | **130** | **189** | **1,305** | **2,147** | **1,178** |
| 0 - 4 | 63,624 | 58,289 | 2,667 | 499 | 672 | 19 | 24 | 302 | 844 | 308 |
| 5 - 9 | 55,166 | 47,938 | 3,958 | 907 | 1,266 | 29 | 47 | 223 | 510 | 288 |
| 10 - 14 | 50,152 | 40,111 | 5,293 | 1,388 | 2,159 | 45 | 67 | 292 | 467 | 330 |
| 15 - 17 | 24,551 | 17,588 | 3,287 | 863 | 1,659 | 37 | 51 | 488 | 326 | 252 |
| **Eastern** | **738,526** | **641,332** | **51,787** | **11,297** | **17,496** | **331** | **557** | **4,876** | **7,089** | **3,761** |
| 0 - 4 | 250,475 | 233,168 | 8,972 | 1,554 | 1,820 | 65 | 61 | 1,116 | 2,805 | 914 |
| 5 - 9 | 213,530 | 190,827 | 13,108 | 2,806 | 3,478 | 78 | 140 | 716 | 1,542 | 835 |
| 10 - 14 | 187,059 | 153,367 | 18,383 | 4,258 | 6,986 | 100 | 203 | 1,032 | 1,569 | 1,161 |
| 15 - 17 | 87,462 | 63,970 | 11,324 | 2,679 | 5,212 | 88 | 153 | 2,012 | 1,173 | 851 |
| **Luapula** | **423,749** | **366,092** | **30,142** | **7,390** | **10,929** | **180** | **304** | **2,373** | **4,580** | **1,759** |
| 0 - 4 | 148,488 | 136,729 | 6,321 | 1,118 | 1,370 | 39 | 54 | 484 | 1,889 | 484 |
| 5 - 9 | 122,910 | 108,233 | 8,119 | 1,992 | 2,712 | 42 | 68 | 323 | 1,030 | 391 |
| 10 - 14 | 104,481 | 85,707 | 9,876 | 2,669 | 4,052 | 70 | 116 | 521 | 995 | 475 |
| 15 - 17 | 47,870 | 35,423 | 5,826 | 1,611 | 2,795 | 29 | 66 | 1,045 | 666 | 409 |
| **Lusaka** | **169,038** | **141,935** | **13,682** | **3,233** | **4,907** | **106** | **189** | **1,559** | **2,280** | **1,147** |
| 0 - 4 | 55,680 | 51,341 | 2,106 | 400 | 406 | 15 | 17 | 380 | 752 | 263 |
| 5 - 9 | 47,160 | 41,172 | 3,224 | 771 | 831 | 21 | 43 | 249 | 562 | 287 |
| 10 - 14 | 44,071 | 34,312 | 5,062 | 1,264 | 1,964 | 46 | 75 | 390 | 582 | 376 |
| 15 - 17 | 22,127 | 15,110 | 3,290 | 798 | 1,706 | 24 | 54 | 540 | 384 | 221 |
| **Muchinga** | **319,849** | **277,688** | **21,391** | **4,769** | **7,507** | **167** | **216** | **2,184** | **4,264** | **1,663** |
| 0 - 4 | 110,413 | 102,389 | 3,744 | 673 | 879 | 22 | 28 | 422 | 1,811 | 445 |
| 5 - 9 | 94,562 | 83,786 | 5,764 | 1,378 | 1,796 | 47 | 53 | 308 | 1,018 | 412 |
| 10 - 14 | 78,518 | 64,650 | 7,374 | 1,737 | 2,826 | 59 | 77 | 441 | 881 | 473 |
| 15 - 17 | 36,356 | 26,863 | 4,509 | 981 | 2,006 | 39 | 58 | 1,013 | 554 | 333 |
| **Northern** | **485,922** | **425,742** | **31,087** | **6,833** | **10,581** | **208** | **304** | **3,142** | **5,838** | **2,187** |
| 0 - 4 | 173,968 | 161,623 | 6,126 | 1,065 | 1,379 | 42 | 50 | 581 | 2,464 | 638 |
| 5 - 9 | 141,524 | 126,345 | 8,368 | 1,861 | 2,540 | 52 | 66 | 394 | 1,336 | 562 |
| 10 - 14 | 117,198 | 97,785 | 10,456 | 2,438 | 3,947 | 76 | 109 | 531 | 1,264 | 592 |
| 15 - 17 | 53,232 | 39,989 | 6,137 | 1,469 | 2,715 | 38 | 79 | 1,636 | 774 | 395 |

## Table B7: Population (De Facto) Aged 0-17 Years by 5 Year Age Group; Survival Status of Parents; Province and Rural/Urban, Zambia 2010

| Province and Age Group | Survival Status of Parents | | | | | | | | | |
|---|---|---|---|---|---|---|---|---|---|---|
| | Population 0 - 17 Years | Both Parents Alive | Mother Alive, Father Dead | Father Alive, Mother Dead | Both Parents Dead | Father Dead, Status of Mother Unknown | Mother Dead, Status of Father Unknown | Status of Both Parents Unknown | Father Alive, Status of Mother Unknown | Mother Alive, Status of Father Unknown |
| **North Western** | **302,697** | **266,028** | **18,540** | **4,422** | **4,516** | **115** | **215** | **2,419** | **4,101** | **2,341** |
| 0 - 4 | 105,731 | 97,865 | 3,701 | 626 | 542 | 16 | 27 | 624 | 1,598 | 732 |
| 5 - 9 | 87,038 | 78,058 | 4,804 | 1,121 | 977 | 36 | 51 | 411 | 1,016 | 564 |
| 10 - 14 | 74,508 | 62,954 | 6,121 | 1,602 | 1,641 | 39 | 68 | 556 | 890 | 637 |
| 15 - 17 | 35,420 | 27,151 | 3,914 | 1,073 | 1,356 | 24 | 69 | 828 | 597 | 408 |
| | | | | | | | | | | |
| **Southern** | **659,128** | **570,424** | **43,746** | **12,173** | **14,841** | **295** | **662** | **5,241** | **7,274** | **4,472** |
| 0 - 4 | 228,258 | 212,687 | 6,989 | 1,493 | 1,571 | 51 | 76 | 1,359 | 2,854 | 1,178 |
| 5 - 9 | 188,394 | 167,784 | 10,749 | 2,939 | 2,960 | 73 | 160 | 825 | 1,747 | 1,157 |
| 10 - 14 | 162,652 | 132,367 | 15,770 | 4,590 | 5,622 | 96 | 244 | 1,138 | 1,567 | 1,258 |
| 15 - 17 | 79,824 | 57,586 | 10,238 | 3,151 | 4,688 | 75 | 182 | 1,919 | 1,106 | 879 |
| | | | | | | | | | | |
| **Western** | **393,467** | **327,776** | **36,612** | **9,836** | **9,919** | **204** | **332** | **2,873** | **3,375** | **2,540** |
| 0 - 4 | 140,515 | 127,697 | 7,118 | 1,505 | 1,361 | 31 | 53 | 750 | 1,274 | 726 |
| 5 - 9 | 112,888 | 96,602 | 9,676 | 2,468 | 2,220 | 44 | 74 | 472 | 734 | 598 |
| 10 - 14 | 94,755 | 72,962 | 12,307 | 3,585 | 3,590 | 80 | 108 | 616 | 785 | 722 |
| 15 - 17 | 45,309 | 30,515 | 7,511 | 2,278 | 2,748 | 49 | 97 | 1,035 | 582 | 494 |

## Table B7: Population (De Facto) Aged 0-17 Years by 5 Year Age Group; Survival Status of Parents; Province and Rural/Urban, Zambia 2010

| Province and Age Group | Survival Status of Parents | | | | | | | | |
|---|---|---|---|---|---|---|---|---|---|
| | Population 0 - 17 Years | Both Parents Alive | Mother Alive, Father Dead | Father Alive, Mother Dead | Both Parents Dead | Father Dead, Status of Mother Unknown | Mother Dead, Status of Father Unknown | Status of Both Parents Unknown | Father Alive, Status of Mother Unknown | Mother Alive, Status of Father Unknown |
| **Zambia Urban** | **2,430,184** | **1,982,936** | **235,471** | **57,194** | **83,548** | **1,090** | **2,544** | **22,845** | **29,288** | **15,268** |
| 0 - 4 | 760,502 | 695,866 | 33,296 | 6,295 | 6,368 | 138 | 261 | 4,978 | 9,834 | 3,466 |
| 5 - 9 | 644,089 | 551,356 | 52,138 | 12,491 | 13,580 | 231 | 514 | 3,521 | 6,819 | 3,439 |
| 10 - 14 | 653,065 | 493,556 | 86,366 | 21,815 | 32,334 | 410 | 978 | 5,704 | 7,336 | 4,566 |
| 15 - 17 | 372,528 | 242,158 | 63,671 | 16,593 | 31,266 | 311 | 791 | 8,642 | 5,299 | 3,797 |
| | | | | | | | | | | |
| **Central** | **156,926** | **124,678** | **16,993** | **3,746** | **6,351** | **120** | **194** | **1,648** | **2,137** | **1,059** |
| 0 - 4 | 47,056 | 42,123 | 2,544 | 391 | 509 | 21 | 21 | 418 | 758 | 271 |
| 5 - 9 | 41,179 | 34,592 | 3,663 | 806 | 1,098 | 24 | 44 | 237 | 503 | 212 |
| 10 - 14 | 43,244 | 31,908 | 6,132 | 1,454 | 2,402 | 43 | 76 | 411 | 513 | 305 |
| 15 - 17 | 25,447 | 16,055 | 4,654 | 1,095 | 2,342 | 32 | 53 | 582 | 363 | 271 |
| | | | | | | | | | | |
| **Copperbelt** | **750,075** | **607,986** | **75,242** | **19,106** | **28,460** | **260** | **715** | **6,206** | **7,850** | **4,250** |
| 0 - 4 | 224,560 | 205,234 | 10,337 | 2,044 | 2,069 | 34 | 82 | 1,319 | 2,491 | 950 |
| 5 - 9 | 193,238 | 164,056 | 16,489 | 4,191 | 4,610 | 57 | 141 | 1,021 | 1,747 | 926 |
| 10 - 14 | 208,393 | 156,502 | 27,881 | 7,338 | 11,361 | 104 | 256 | 1,639 | 2,003 | 1,309 |
| 15 - 17 | 123,884 | 82,194 | 20,535 | 5,533 | 10,420 | 65 | 236 | 2,227 | 1,609 | 1,065 |
| | | | | | | | | | | |
| **Eastern** | **96,442** | **79,488** | **8,860** | **2,109** | **3,053** | **42** | **93** | **1,004** | **1,176** | **617** |
| 0 - 4 | 30,322 | 27,809 | 1,255 | 236 | 238 | 3 | 13 | 235 | 411 | 122 |
| 5 - 9 | 26,422 | 22,879 | 2,000 | 476 | 448 | 8 | 10 | 168 | 288 | 145 |
| 10 - 14 | 25,852 | 19,831 | 3,254 | 802 | 1,222 | 14 | 28 | 222 | 296 | 183 |
| 15 - 17 | 13,846 | 8,969 | 2,351 | 595 | 1,145 | 17 | 42 | 379 | 181 | 167 |
| | | | | | | | | | | |
| **Luapula** | **96,296** | **78,931** | **9,081** | **2,098** | **3,466** | **48** | **89** | **845** | **1,208** | **530** |
| 0 - 4 | 30,327 | 27,301 | 1,654 | 279 | 330 | 2 | 7 | 196 | 425 | 133 |
| 5 - 9 | 26,545 | 22,657 | 2,128 | 541 | 684 | 8 | 25 | 121 | 271 | 110 |
| 10 - 14 | 26,029 | 19,959 | 3,225 | 755 | 1,353 | 25 | 33 | 209 | 302 | 168 |
| 15 - 17 | 13,395 | 9,014 | 2,074 | 523 | 1,099 | 13 | 24 | 319 | 210 | 119 |
| | | | | | | | | | | |
| **Lusaka** | **850,146** | **699,198** | **80,668** | **19,510** | **26,999** | **363** | **969** | **7,466** | **9,753** | **5,220** |
| 0 - 4 | 274,569 | 253,330 | 11,070 | 2,181 | 1,944 | 47 | 94 | 1,492 | 3,279 | 1,132 |
| 5 - 9 | 226,897 | 196,031 | 17,766 | 4,167 | 4,144 | 80 | 185 | 1,093 | 2,272 | 1,159 |
| 10 - 14 | 223,871 | 169,826 | 29,780 | 7,452 | 10,403 | 129 | 397 | 1,887 | 2,441 | 1,556 |
| 15 - 17 | 124,809 | 80,011 | 22,052 | 5,710 | 10,508 | 107 | 293 | 2,994 | 1,761 | 1,373 |
| | | | | | | | | | | |
| **Muchinga** | **60,344** | **49,950** | **5,282** | **1,065** | **2,012** | **41** | **41** | **624** | **898** | **431** |
| 0 - 4 | 19,684 | 17,901 | 828 | 144 | 188 | 4 | 7 | 126 | 372 | 114 |
| 5 - 9 | 16,814 | 14,490 | 1,251 | 248 | 402 | 11 | 11 | 89 | 204 | 108 |
| 10 - 14 | 15,529 | 12,021 | 1,883 | 393 | 764 | 14 | 11 | 134 | 192 | 117 |
| 15 - 17 | 8,317 | 5,538 | 1,320 | 280 | 658 | 12 | 12 | 275 | 130 | 92 |
| | | | | | | | | | | |
| **Northern** | **101,430** | **83,757** | **9,382** | **1,840** | **3,210** | **48** | **82** | **1,013** | **1,495** | **603** |
| 0 - 4 | 31,990 | 29,194 | 1,382 | 238 | 285 | 4 | 13 | 223 | 511 | 140 |
| 5 - 9 | 28,151 | 24,243 | 2,222 | 410 | 602 | 12 | 17 | 147 | 366 | 132 |
| 10 - 14 | 26,885 | 20,735 | 3,382 | 688 | 1,230 | 18 | 32 | 235 | 388 | 177 |
| 15 - 17 | 14,404 | 9,585 | 2,396 | 504 | 1,093 | 14 | 20 | 408 | 230 | 154 |

## Table B7: Population (De Facto) Aged 0-17 Years by 5 Year Age Group; Survival Status of Parents; Province and Rural/Urban, Zambia 2010

| Province and Age Group | Survival Status of Parents | | | | | | | | | |
|---|---|---|---|---|---|---|---|---|---|---|
| | Population 0 - 17 Years | Both Parents Alive | Mother Alive, Father Dead | Father Alive, Mother Dead | Both Parents Dead | Father Dead, Status of Mother Unknown | Mother Dead, Status of Father Unknown | Status of Both Parents Unknown | Father Alive, Status of Mother Unknown | Mother Alive, Status of Father Unknown |
| **North Western** | **81,989** | **69,293** | **5,907** | **1,605** | **1,745** | **54** | **83** | **1,084** | **1,493** | **725** |
| 0 - 4 | 27,244 | 24,995 | 1,003 | 158 | 137 | 11 | 7 | 254 | 484 | 195 |
| 5 - 9 | 22,127 | 19,362 | 1,342 | 344 | 315 | 8 | 15 | 174 | 382 | 185 |
| 10 - 14 | 21,089 | 16,886 | 2,041 | 639 | 611 | 22 | 34 | 271 | 382 | 203 |
| 15 - 17 | 11,529 | 8,050 | 1,521 | 464 | 682 | 13 | 27 | 385 | 245 | 142 |
| | | | | | | | | | | |
| **Southern** | **180,562** | **145,897** | **17,648** | **4,477** | **5,950** | **91** | **207** | **2,297** | **2,616** | **1,379** |
| 0 - 4 | 57,285 | 52,357 | 2,263 | 452 | 409 | 11 | 11 | 565 | 911 | 306 |
| 5 - 9 | 48,208 | 41,067 | 3,867 | 947 | 890 | 19 | 45 | 379 | 643 | 351 |
| 10 - 14 | 47,076 | 35,077 | 6,476 | 1,683 | 2,160 | 33 | 82 | 520 | 639 | 406 |
| 15 - 17 | 27,993 | 17,396 | 5,042 | 1,395 | 2,491 | 28 | 69 | 833 | 423 | 316 |
| | | | | | | | | | | |
| **Western** | **55,974** | **43,758** | **6,408** | **1,638** | **2,302** | **23** | **71** | **658** | **662** | **454** |
| 0 - 4 | 17,465 | 15,622 | 960 | 172 | 259 | 1 | 6 | 150 | 192 | 103 |
| 5 - 9 | 14,508 | 11,979 | 1,410 | 361 | 387 | 4 | 21 | 92 | 143 | 111 |
| 10 - 14 | 15,097 | 10,811 | 2,312 | 611 | 828 | 8 | 29 | 176 | 180 | 142 |
| 15 - 17 | 8,904 | 5,346 | 1,726 | 494 | 828 | 10 | 15 | 240 | 147 | 98 |

## Table B8: Population (De Facto) Aged 0-17 Years by 5 Years Age Groups; Survival and Household Living Status of Parents; Province and Rural/Urban, Zambia 2010

| Province and Age Group | Population 0-17 Years | Survival and Household Living Status of Mother | | | | Survival and Household Living Status of Father | | | |
|---|---|---|---|---|---|---|---|---|---|
| | | Total with Mother Alive | Alive and in Household | Alive and not in Household | Dead / Don't Know | Total with Father Alive | Alive and in Household | Alive and not in Household | Dead / Don't Know |
| **Zambia Total** | **6,638,301** | **6,184,229** | **5,470,301** | **713,928** | **454,072** | **5,815,404** | **4,608,517** | **1,206,887** | **822,897** |
| 0 - 4 | 2,214,887 | 2,138,331 | 2,027,776 | 110,555 | 76,556 | 2,085,275 | 1,709,647 | 375,628 | 129,612 |
| 5 - 9 | 1,856,336 | 1,761,799 | 1,575,622 | 186,177 | 94,537 | 1,671,696 | 1,346,085 | 325,611 | 184,640 |
| 10 - 14 | 1,699,042 | 1,546,101 | 1,297,328 | 248,773 | 152,941 | 1,410,864 | 1,088,490 | 322,374 | 288,178 |
| 15 - 17 | 868,036 | 737,998 | 569,575 | 168,423 | 130,038 | 647,569 | 464,295 | 183,274 | 220,467 |
| | | | | | | | | | |
| **Central** | **679,174** | **629,101** | **550,652** | **78,449** | **50,073** | **591,046** | **472,718** | **118,328** | **88,128** |
| 0 - 4 | 224,289 | 215,421 | 202,755 | 12,666 | 8,868 | 209,540 | 173,444 | 36,096 | 14,749 |
| 5 - 9 | 190,254 | 179,724 | 159,085 | 20,639 | 10,530 | 170,448 | 138,596 | 31,852 | 19,806 |
| 10 - 14 | 175,827 | 159,077 | 131,886 | 27,191 | 16,750 | 145,286 | 113,240 | 32,046 | 30,541 |
| 15 - 17 | 88,804 | 74,879 | 56,926 | 17,953 | 13,925 | 65,772 | 47,438 | 18,334 | 23,032 |
| | | | | | | | | | |
| **Copperbelt** | **943,568** | **867,787** | **757,016** | **110,771** | **75,781** | **804,672** | **633,208** | **171,464** | **138,896** |
| 0 - 4 | 288,184 | 277,785 | 262,079 | 15,706 | 10,399 | 269,401 | 218,186 | 51,215 | 18,783 |
| 5 - 9 | 248,404 | 233,655 | 206,904 | 26,751 | 14,749 | 219,349 | 175,631 | 43,718 | 29,055 |
| 10 - 14 | 258,545 | 231,426 | 191,409 | 40,017 | 27,119 | 207,809 | 160,081 | 47,728 | 50,736 |
| 15 - 17 | 148,435 | 124,921 | 96,624 | 28,297 | 23,514 | 108,113 | 79,310 | 28,803 | 40,322 |
| | | | | | | | | | |
| **Eastern** | **834,968** | **785,845** | **715,406** | **70,439** | **49,123** | **742,491** | **606,985** | **135,506** | **92,477** |
| 0 - 4 | 280,797 | 272,240 | 261,899 | 10,341 | 8,557 | 265,983 | 226,337 | 39,646 | 14,814 |
| 5 - 9 | 239,952 | 229,794 | 211,129 | 18,665 | 10,158 | 218,818 | 180,838 | 37,980 | 21,134 |
| 10 - 14 | 212,911 | 196,179 | 171,150 | 25,029 | 16,732 | 180,123 | 142,499 | 37,624 | 32,788 |
| 15 - 17 | 101,308 | 87,632 | 71,228 | 16,404 | 13,676 | 77,567 | 57,311 | 20,256 | 23,741 |
| | | | | | | | | | |
| **Luapula** | **520,045** | **486,535** | **443,078** | **43,457** | **33,510** | **460,299** | **375,091** | **85,208** | **59,746** |
| 0 - 4 | 178,815 | 172,622 | 165,614 | 7,008 | 6,193 | 167,741 | 140,773 | 26,968 | 11,074 |
| 5 - 9 | 149,455 | 141,638 | 130,148 | 11,490 | 7,817 | 134,724 | 111,186 | 23,538 | 14,731 |
| 10 - 14 | 130,510 | 119,410 | 104,169 | 15,241 | 11,100 | 110,387 | 87,671 | 22,716 | 20,123 |
| 15 - 17 | 61,265 | 52,865 | 43,147 | 9,718 | 8,400 | 47,447 | 35,461 | 11,986 | 13,818 |
| | | | | | | | | | |
| **Lusaka** | **1,019,184** | **941,850** | **826,349** | **115,501** | **77,334** | **875,909** | **696,210** | **179,699** | **143,275** |
| 0 - 4 | 330,249 | 319,242 | 302,666 | 16,576 | 11,007 | 311,283 | 257,447 | 53,836 | 18,966 |
| 5 - 9 | 274,057 | 259,639 | 231,591 | 28,048 | 14,418 | 244,975 | 198,999 | 45,976 | 29,082 |
| 10 - 14 | 267,942 | 240,912 | 199,781 | 41,131 | 27,030 | 215,877 | 166,079 | 49,798 | 52,065 |
| 15 - 17 | 146,936 | 122,057 | 92,311 | 29,746 | 24,879 | 103,774 | 73,685 | 30,089 | 43,162 |
| | | | | | | | | | |
| **Muchinga** | **380,193** | **356,405** | **327,828** | **28,577** | **23,788** | **338,634** | **286,667** | **51,967** | **41,559** |
| 0 - 4 | 130,097 | 125,421 | 121,229 | 4,192 | 4,676 | 123,290 | 108,460 | 14,830 | 6,807 |
| 5 - 9 | 111,376 | 105,811 | 98,164 | 7,647 | 5,565 | 101,124 | 86,403 | 14,721 | 10,252 |
| 10 - 14 | 94,047 | 86,518 | 76,851 | 9,667 | 7,529 | 79,874 | 65,577 | 14,297 | 14,173 |
| 15 - 17 | 44,673 | 38,655 | 31,584 | 7,071 | 6,018 | 34,346 | 26,227 | 8,119 | 10,327 |
| | | | | | | | | | |
| **Northern** | **587,352** | **552,758** | **507,806** | **44,952** | **34,594** | **525,505** | **442,829** | **82,676** | **61,847** |
| 0 - 4 | 205,958 | 199,103 | 192,118 | 6,985 | 6,855 | 195,095 | 170,217 | 24,878 | 10,863 |
| 5 - 9 | 169,675 | 161,872 | 149,856 | 12,016 | 7,803 | 154,561 | 131,582 | 22,979 | 15,114 |
| 10 - 14 | 144,083 | 133,127 | 117,916 | 15,211 | 10,956 | 123,298 | 101,183 | 22,115 | 20,785 |
| 15 - 17 | 67,636 | 58,656 | 47,916 | 10,740 | 8,980 | 52,551 | 39,847 | 12,704 | 15,085 |

## Table B8: Population (De Facto) Aged 0-17 Years by 5 Years Age Groups and Survival; Household Living Status of Parents; Province and Rural/Urban, Zambia 2010

| Province and Age Group | Population 0-17 Years | Survival and Household Living Status of Mother | | | | Survival and Household Living Status of Father | | | |
|---|---|---|---|---|---|---|---|---|---|
| | | Total with Mother Alive | Alive and in Household | Alive and not in Household | Dead / Don't Know | Total with Father Alive | Alive and in Household | Alive and not in Household | Dead / Don't Know |
| **North Western** | **384,686** | **362,834** | **318,400** | **44,434** | **21,852** | **346,942** | **263,896** | **83,046** | **37,744** |
| 0 - 4 | 132,975 | 128,491 | 121,715 | 6,776 | 4,484 | 125,726 | 98,990 | 26,736 | 7,249 |
| 5 - 9 | 109,165 | 104,315 | 92,802 | 11,513 | 4,850 | 100,283 | 77,902 | 22,381 | 8,882 |
| 10 - 14 | 95,597 | 88,842 | 73,251 | 15,591 | 6,755 | 83,353 | 61,650 | 21,703 | 12,244 |
| 15 - 17 | 46,949 | 41,186 | 30,632 | 10,554 | 5,763 | 37,580 | 25,354 | 12,226 | 9,369 |
| | | | | | | | | | |
| **Southern** | **839,690** | **783,566** | **668,521** | **115,045** | **56,124** | **742,861** | **569,966** | **172,895** | **96,829** |
| 0 - 4 | 285,543 | 275,780 | 256,611 | 19,169 | 9,763 | 270,754 | 215,925 | 54,829 | 14,789 |
| 5 - 9 | 236,602 | 224,975 | 193,527 | 31,448 | 11,627 | 215,127 | 167,962 | 47,165 | 21,475 |
| 10 - 14 | 209,728 | 191,354 | 152,671 | 38,683 | 18,374 | 175,923 | 131,152 | 44,771 | 33,805 |
| 15 - 17 | 107,817 | 91,457 | 65,712 | 25,745 | 16,360 | 81,057 | 54,927 | 26,130 | 26,760 |
| | | | | | | | | | |
| **Western** | **449,441** | **417,548** | **355,245** | **62,303** | **31,893** | **387,045** | **260,947** | **126,098** | **62,396** |
| 0 - 4 | 157,980 | 152,226 | 141,090 | 11,136 | 5,754 | 146,462 | 99,868 | 46,594 | 11,518 |
| 5 - 9 | 127,396 | 120,376 | 102,416 | 17,960 | 7,020 | 112,287 | 76,986 | 35,301 | 15,109 |
| 10 - 14 | 109,852 | 99,256 | 78,244 | 21,012 | 10,596 | 88,934 | 59,358 | 29,576 | 20,918 |
| 15 - 17 | 54,213 | 45,690 | 33,495 | 12,195 | 8,523 | 39,362 | 24,735 | 14,627 | 14,851 |

## Table B8: Population (De Facto) Aged 0-17 Years by 5 Years Age Groups and Survival; Household Living Status of Parents; Province and Rural, Zambia 2010

| Province and Age Group | Population 0-17 Years | Survival and Household Living Status of Mother | | | | Survival and Household Living Status of Father | | | |
|---|---|---|---|---|---|---|---|---|---|
| | | Total with Mother Alive | Alive and in Household | Alive and not in Household | Dead / Don't Know | Total with Father Alive | Alive and in Household | Alive and not in Household | Dead / Don't Know |
| **Zambia Rural** | **4,208,117** | **3,950,554** | **3,532,999** | **417,555** | **257,563** | **3,745,986** | **3,000,479** | **745,507** | **462,131** |
| 0 - 4 | 1,454,385 | 1,405,703 | 1,336,577 | 69,126 | 48,682 | 1,373,280 | 1,136,989 | 236,291 | 81,105 |
| 5 - 9 | 1,212,247 | 1,154,866 | 1,038,599 | 116,267 | 57,381 | 1,101,030 | 891,672 | 209,358 | 111,217 |
| 10 - 14 | 1,045,977 | 961,613 | 818,234 | 143,379 | 84,364 | 888,157 | 691,930 | 196,227 | 157,820 |
| 15 - 17 | 495,508 | 428,372 | 339,589 | 88,783 | 67,136 | 383,519 | 279,888 | 103,631 | 111,989 |
| | | | | | | | | | |
| **Central** | **522,248** | **486,371** | **429,079** | **57,292** | **35,877** | **460,485** | **373,061** | **87,424** | **61,763** |
| 0 - 4 | 177,233 | 170,483 | 160,697 | 9,786 | 6,750 | 166,268 | 139,180 | 27,088 | 10,965 |
| 5 - 9 | 149,075 | 141,257 | 125,404 | 15,853 | 7,818 | 134,547 | 110,246 | 24,301 | 14,528 |
| 10 - 14 | 132,583 | 120,732 | 100,948 | 19,784 | 11,851 | 111,411 | 87,867 | 23,544 | 21,172 |
| 15 - 17 | 63,357 | 53,899 | 42,030 | 11,869 | 9,458 | 48,259 | 35,768 | 12,491 | 15,098 |
| | | | | | | | | | |
| **Copperbelt** | **193,493** | **180,309** | **160,212** | **20,097** | **13,184** | **169,730** | **137,429** | **32,301** | **23,763** |
| 0 - 4 | 63,624 | 61,264 | 58,211 | 3,053 | 2,360 | 59,632 | 50,385 | 9,247 | 3,992 |
| 5 - 9 | 55,166 | 52,184 | 46,844 | 5,340 | 2,982 | 49,355 | 40,446 | 8,909 | 5,811 |
| 10 - 14 | 50,152 | 45,734 | 38,572 | 7,162 | 4,418 | 41,966 | 32,827 | 9,139 | 8,186 |
| 15 - 17 | 24,551 | 21,127 | 16,585 | 4,542 | 3,424 | 18,777 | 13,771 | 5,006 | 5,774 |
| | | | | | | | | | |
| **Eastern** | **738,526** | **696,880** | **638,066** | **58,814** | **41,646** | **659,718** | **542,466** | **117,252** | **78,808** |
| 0 - 4 | 250,475 | 243,054 | 234,274 | 8,780 | 7,421 | 237,527 | 203,136 | 34,391 | 12,948 |
| 5 - 9 | 213,530 | 204,770 | 188,788 | 15,982 | 8,760 | 195,175 | 161,795 | 33,380 | 18,355 |
| 10 - 14 | 187,059 | 172,911 | 151,998 | 20,913 | 14,148 | 159,194 | 126,683 | 32,511 | 27,865 |
| 15 - 17 | 87,462 | 76,145 | 63,006 | 13,139 | 11,317 | 67,822 | 50,852 | 16,970 | 19,640 |
| | | | | | | | | | |
| **Luapula** | **423,749** | **397,993** | **365,660** | **32,333** | **25,756** | **378,062** | **311,592** | **66,470** | **45,687** |
| 0 - 4 | 148,488 | 143,534 | 138,155 | 5,379 | 4,954 | 139,736 | 118,582 | 21,154 | 8,752 |
| 5 - 9 | 122,910 | 116,743 | 107,974 | 8,769 | 6,167 | 111,255 | 92,662 | 18,593 | 11,655 |
| 10 - 14 | 104,481 | 96,058 | 84,780 | 11,278 | 8,423 | 89,371 | 71,717 | 17,654 | 15,110 |
| 15 - 17 | 47,870 | 41,658 | 34,751 | 6,907 | 6,212 | 37,700 | 28,631 | 9,069 | 10,170 |
| | | | | | | | | | |
| **Lusaka** | **169,038** | **156,764** | **136,445** | **20,319** | **12,274** | **147,448** | **117,235** | **30,213** | **21,590** |
| 0 - 4 | 55,680 | 53,710 | 50,613 | 3,097 | 1,970 | 52,493 | 43,535 | 8,958 | 3,187 |
| 5 - 9 | 47,160 | 44,683 | 39,210 | 5,473 | 2,477 | 42,505 | 34,293 | 8,212 | 4,655 |
| 10 - 14 | 44,071 | 39,750 | 32,523 | 7,227 | 4,321 | 36,158 | 27,712 | 8,446 | 7,913 |
| 15 - 17 | 22,127 | 18,621 | 14,099 | 4,522 | 3,506 | 16,292 | 11,695 | 4,597 | 5,835 |
| | | | | | | | | | |
| **Muchinga** | **319,849** | **300,742** | **278,972** | **21,770** | **19,107** | **286,721** | **244,612** | **42,109** | **33,128** |
| 0 - 4 | 110,413 | 106,578 | 103,350 | 3,228 | 3,835 | 104,873 | 92,751 | 12,122 | 5,540 |
| 5 - 9 | 94,562 | 89,962 | 83,895 | 6,067 | 4,600 | 86,182 | 73,993 | 12,189 | 8,380 |
| 10 - 14 | 78,518 | 72,497 | 65,146 | 7,351 | 6,021 | 67,268 | 55,711 | 11,557 | 11,250 |
| 15 - 17 | 36,356 | 31,705 | 26,581 | 5,124 | 4,651 | 28,398 | 22,157 | 6,241 | 7,958 |
| | | | | | | | | | |
| **Northern** | **485,922** | **459,016** | **425,489** | **33,527** | **26,906** | **438,413** | **372,878** | **65,535** | **47,509** |
| 0 - 4 | 173,968 | 168,387 | 162,957 | 5,430 | 5,581 | 165,152 | 145,054 | 20,098 | 8,816 |
| 5 - 9 | 141,524 | 135,275 | 125,917 | 9,358 | 6,249 | 129,542 | 110,908 | 18,634 | 11,982 |
| 10 - 14 | 117,198 | 108,833 | 97,628 | 11,205 | 8,365 | 101,487 | 84,178 | 17,309 | 15,711 |
| 15 - 17 | 53,232 | 46,521 | 38,987 | 7,534 | 6,711 | 42,232 | 32,738 | 9,494 | 11,000 |

## Table B8: Population (De Facto) Aged 0-17 Years by 5 Years Age Groups and Survival; Household Living Status of Parents; Province and Rural, Zambia 2010

| Province and Age Group | Population 0-17 Years | Survival and Household Living Status of Mother | | | | Survival and Household Living Status of Father | | | |
|---|---|---|---|---|---|---|---|---|---|
| | | Total with Mother Alive | Alive and in Household | Alive and not in Household | Dead / Don't Know | Total with Father Alive | Alive and in Household | Alive and not in Household | Dead / Don't Know |
| **North Western** | **302,697** | **286,909** | **254,066** | **32,843** | **15,788** | **274,551** | **211,018** | **63,533** | **28,146** |
| 0 - 4 | 105,731 | 102,298 | 97,012 | 5,286 | 3,433 | 100,089 | 79,377 | 20,712 | 5,642 |
| 5 - 9 | 87,038 | 83,426 | 74,607 | 8,819 | 3,612 | 80,195 | 62,674 | 17,521 | 6,843 |
| 10 - 14 | 74,508 | 69,712 | 58,370 | 11,342 | 4,796 | 65,446 | 49,064 | 16,382 | 9,062 |
| 15 - 17 | 35,420 | 31,473 | 24,077 | 7,396 | 3,947 | 28,821 | 19,903 | 8,918 | 6,599 |
| | | | | | | | | | |
| **Southern** | **659,128** | **618,642** | **530,297** | **88,345** | **40,486** | **589,871** | **458,326** | **131,545** | **69,257** |
| 0 - 4 | 228,258 | 220,854 | 205,431 | 15,423 | 7,404 | 217,034 | 175,194 | 41,840 | 11,224 |
| 5 - 9 | 188,394 | 179,690 | 154,604 | 25,086 | 8,704 | 172,470 | 135,824 | 36,646 | 15,924 |
| 10 - 14 | 162,652 | 149,395 | 119,755 | 29,640 | 13,257 | 138,524 | 104,275 | 34,249 | 24,128 |
| 15 - 17 | 79,824 | 68,703 | 50,507 | 18,196 | 11,121 | 61,843 | 43,033 | 18,810 | 17,981 |
| | | | | | | | | | |
| **Western** | **393,467** | **366,928** | **314,713** | **52,215** | **26,539** | **340,987** | **231,862** | **109,125** | **52,480** |
| 0 - 4 | 140,515 | 135,541 | 125,877 | 9,664 | 4,974 | 130,476 | 89,795 | 40,681 | 10,039 |
| 5 - 9 | 112,888 | 106,876 | 91,356 | 15,520 | 6,012 | 99,804 | 68,831 | 30,973 | 13,084 |
| 10 - 14 | 94,755 | 85,991 | 68,514 | 17,477 | 8,764 | 77,332 | 51,896 | 25,436 | 17,423 |
| 15 - 17 | 45,309 | 38,520 | 28,966 | 9,554 | 6,789 | 33,375 | 21,340 | 12,035 | 11,934 |

## Table B8: Population (De Facto) Aged 0-17 Years by 5 Years Age Groups and Survival; Household Living Status of Parents; Province and Urban, Zambia 2010

| Province and Age Group | Population 0-17 Years | Survival and Household Living Status of Mother | | | | Survival and Household Living Status of Father | | | |
|---|---|---|---|---|---|---|---|---|---|
| | | Total with Mother Alive | Alive and in Household | Alive and not in Household | Dead / Don't Know | Total with Father Alive | Alive and in Household | Alive and not in Household | Dead / Don't Know |
| **Zambia Urban** | **2,430,184** | **2,233,675** | **1,937,302** | **296,373** | **196,509** | **2,069,418** | **1,608,038** | **461,380** | **360,766** |
| 0 - 4 | 760,502 | 732,628 | 691,199 | 41,429 | 27,874 | 711,995 | 572,658 | 139,337 | 48,507 |
| 5 - 9 | 644,089 | 606,933 | 537,023 | 69,910 | 37,156 | 570,666 | 454,413 | 116,253 | 73,423 |
| 10 - 14 | 653,065 | 584,488 | 479,094 | 105,394 | 68,577 | 522,707 | 396,560 | 126,147 | 130,358 |
| 15 - 17 | 372,528 | 309,626 | 229,986 | 79,640 | 62,902 | 264,050 | 184,407 | 79,643 | 108,478 |
| | | | | | | | | | |
| **Central** | **156,926** | **142,730** | **121,573** | **21,157** | **14,196** | **130,561** | **99,657** | **30,904** | **26,365** |
| 0 - 4 | 47,056 | 44,938 | 42,058 | 2,880 | 2,118 | 43,272 | 34,264 | 9,008 | 3,784 |
| 5 - 9 | 41,179 | 38,467 | 33,681 | 4,786 | 2,712 | 35,901 | 28,350 | 7,551 | 5,278 |
| 10 - 14 | 43,244 | 38,345 | 30,938 | 7,407 | 4,899 | 33,875 | 25,373 | 8,502 | 9,369 |
| 15 - 17 | 25,447 | 20,980 | 14,896 | 6,084 | 4,467 | 17,513 | 11,670 | 5,843 | 7,934 |
| | | | | | | | | | |
| **Copperbelt** | **750,075** | **687,478** | **596,804** | **90,674** | **62,597** | **634,942** | **495,779** | **139,163** | **115,133** |
| 0 - 4 | 224,560 | 216,521 | 203,868 | 12,653 | 8,039 | 209,769 | 167,801 | 41,968 | 14,791 |
| 5 - 9 | 193,238 | 181,471 | 160,060 | 21,411 | 11,767 | 169,994 | 135,185 | 34,809 | 23,244 |
| 10 - 14 | 208,393 | 185,692 | 152,837 | 32,855 | 22,701 | 165,843 | 127,254 | 38,589 | 42,550 |
| 15 - 17 | 123,884 | 103,794 | 80,039 | 23,755 | 20,090 | 89,336 | 65,539 | 23,797 | 34,548 |
| | | | | | | | | | |
| **Eastern** | **96,442** | **88,965** | **77,340** | **11,625** | **7,477** | **82,773** | **64,519** | **18,254** | **13,669** |
| 0 - 4 | 30,322 | 29,186 | 27,625 | 1,561 | 1,136 | 28,456 | 23,201 | 5,255 | 1,866 |
| 5 - 9 | 26,422 | 25,024 | 22,341 | 2,683 | 1,398 | 23,643 | 19,043 | 4,600 | 2,779 |
| 10 - 14 | 25,852 | 23,268 | 19,152 | 4,116 | 2,584 | 20,929 | 15,816 | 5,113 | 4,923 |
| 15 - 17 | 13,846 | 11,487 | 8,222 | 3,265 | 2,359 | 9,745 | 6,459 | 3,286 | 4,101 |
| | | | | | | | | | |
| **Luapula** | **96,296** | **88,542** | **77,418** | **11,124** | **7,754** | **82,237** | **63,499** | **18,738** | **14,059** |
| 0 - 4 | 30,327 | 29,088 | 27,459 | 1,629 | 1,239 | 28,005 | 22,191 | 5,814 | 2,322 |
| 5 - 9 | 26,545 | 24,895 | 22,174 | 2,721 | 1,650 | 23,469 | 18,524 | 4,945 | 3,076 |
| 10 - 14 | 26,029 | 23,352 | 19,389 | 3,963 | 2,677 | 21,016 | 15,954 | 5,062 | 5,013 |
| 15 - 17 | 13,395 | 11,207 | 8,396 | 2,811 | 2,188 | 9,747 | 6,830 | 2,917 | 3,648 |
| | | | | | | | | | |
| **Lusaka** | **850,146** | **785,086** | **689,904** | **95,182** | **65,060** | **728,461** | **578,975** | **149,486** | **121,685** |
| 0 - 4 | 274,569 | 265,532 | 252,053 | 13,479 | 9,037 | 258,790 | 213,912 | 44,878 | 15,779 |
| 5 - 9 | 226,897 | 214,956 | 192,381 | 22,575 | 11,941 | 202,470 | 164,706 | 37,764 | 24,427 |
| 10 - 14 | 223,871 | 201,162 | 167,258 | 33,904 | 22,709 | 179,719 | 138,367 | 41,352 | 44,152 |
| 15 - 17 | 124,809 | 103,436 | 78,212 | 25,224 | 21,373 | 87,482 | 61,990 | 25,492 | 37,327 |
| | | | | | | | | | |
| **Muchinga** | **60,344** | **55,663** | **48,856** | **6,807** | **4,681** | **51,913** | **42,055** | **9,858** | **8,431** |
| 0 - 4 | 19,684 | 18,843 | 17,879 | 964 | 841 | 18,417 | 15,709 | 2,708 | 1,267 |
| 5 - 9 | 16,814 | 15,849 | 14,269 | 1,580 | 965 | 14,942 | 12,410 | 2,532 | 1,872 |
| 10 - 14 | 15,529 | 14,021 | 11,705 | 2,316 | 1,508 | 12,606 | 9,866 | 2,740 | 2,923 |
| 15 - 17 | 8,317 | 6,950 | 5,003 | 1,947 | 1,367 | 5,948 | 4,070 | 1,878 | 2,369 |
| | | | | | | | | | |
| **Northern** | **101,430** | **93,742** | **82,317** | **11,425** | **7,688** | **87,092** | **69,951** | **17,141** | **14,338** |
| 0 - 4 | 31,990 | 30,716 | 29,161 | 1,555 | 1,274 | 29,943 | 25,163 | 4,780 | 2,047 |
| 5 - 9 | 28,151 | 26,597 | 23,939 | 2,658 | 1,554 | 25,019 | 20,674 | 4,345 | 3,132 |
| 10 - 14 | 26,885 | 24,294 | 20,288 | 4,006 | 2,591 | 21,811 | 17,005 | 4,806 | 5,074 |
| 15 - 17 | 14,404 | 12,135 | 8,929 | 3,206 | 2,269 | 10,319 | 7,109 | 3,210 | 4,085 |

## Table B8: Population (De Facto) Aged 0-17 Years by 5 Years Age Groups and Survival; Household Living Status of Parents; Province and Urban, Zambia 2010

| Province and Age Group | Population 0-17 Years | Survival and Household Living Status of Mother | | | | Survival and Household Living Status of Father | | | |
|---|---|---|---|---|---|---|---|---|---|
| | | Total with Mother Alive | Alive and in Household | Alive and not in Household | Dead / Don't Know | Total with Father Alive | Alive and in Household | Alive and not in Household | Dead / Don't Know |
| **North Western** | **81,989** | **75,925** | **64,334** | **11,591** | **6,064** | **72,391** | **52,878** | **19,513** | **9,598** |
| 0 - 4 | 27,244 | 26,193 | 24,703 | 1,490 | 1,051 | 25,637 | 19,613 | 6,024 | 1,607 |
| 5 - 9 | 22,127 | 20,889 | 18,195 | 2,694 | 1,238 | 20,088 | 15,228 | 4,860 | 2,039 |
| 10 - 14 | 21,089 | 19,130 | 14,881 | 4,249 | 1,959 | 17,907 | 12,586 | 5,321 | 3,182 |
| 15 - 17 | 11,529 | 9,713 | 6,555 | 3,158 | 1,816 | 8,759 | 5,451 | 3,308 | 2,770 |
| | | | | | | | | | |
| **Southern** | **180,562** | **164,924** | **138,224** | **26,700** | **15,638** | **152,990** | **111,640** | **41,350** | **27,572** |
| 0 - 4 | 57,285 | 54,926 | 51,180 | 3,746 | 2,359 | 53,720 | 40,731 | 12,989 | 3,565 |
| 5 - 9 | 48,208 | 45,285 | 38,923 | 6,362 | 2,923 | 42,657 | 32,138 | 10,519 | 5,551 |
| 10 - 14 | 47,076 | 41,959 | 32,916 | 9,043 | 5,117 | 37,399 | 26,877 | 10,522 | 9,677 |
| 15 - 17 | 27,993 | 22,754 | 15,205 | 7,549 | 5,239 | 19,214 | 11,894 | 7,320 | 8,779 |
| | | | | | | | | | |
| **Western** | **55,974** | **50,620** | **40,532** | **10,088** | **5,354** | **46,058** | **29,085** | **16,973** | **9,916** |
| 0 - 4 | 17,465 | 16,685 | 15,213 | 1,472 | 780 | 15,986 | 10,073 | 5,913 | 1,479 |
| 5 - 9 | 14,508 | 13,500 | 11,060 | 2,440 | 1,008 | 12,483 | 8,155 | 4,328 | 2,025 |
| 10 - 14 | 15,097 | 13,265 | 9,730 | 3,535 | 1,832 | 11,602 | 7,462 | 4,140 | 3,495 |
| 15 - 17 | 8,904 | 7,170 | 4,529 | 2,641 | 1,734 | 5,987 | 3,395 | 2,592 | 2,917 |

## Table B9: Type of Orphan as a Percent of Population Aged 0-17 Years by Province and Rural/Urban, Zambia 2010

| Province and Rural/ Urban | Population 0-17 Years | Orphans by Type as a Percentage of Population Aged 0-17 Years | | | | | | | |
|---|---|---|---|---|---|---|---|---|---|
| | | Paternal Orphans | | Maternal Orphans | | Double Orphans | | Total Orphans | |
| | | Number | Percent | Number | Percent | Number | Percent | Number | Percent |
| **Zambia Total** | **6,638,301** | **539,436** | **8.1** | **136,660** | **2.1** | **184,546** | **2.8** | **860,642** | **13.0** |
| Rural | 4,208,117 | 302,875 | 7.2 | 76,922 | 1.8 | 100,998 | 2.4 | 480,795 | 11.4 |
| Urban | 2,430,184 | 236,561 | 9.7 | 59,738 | 2.5 | 83,548 | 3.4 | 379,847 | 15.6 |
| **Central** | **679,174** | **56,060** | **8.3** | **14,284** | **2.1** | **20,897** | **3.1** | **91,241** | **13.4** |
| Rural | 522,248 | 38,947 | 7.5 | 10,344 | 2.0 | 14,546 | 2.8 | 63,837 | 12.2 |
| Urban | 156,926 | 17,113 | 10.9 | 3,940 | 2.5 | 6,351 | 4.0 | 27,404 | 17.5 |
| **Copperbelt** | **943,568** | **90,837** | **9.6** | **23,667** | **2.5** | **34,216** | **3.6** | **148,720** | **15.8** |
| Rural | 193,493 | 15,335 | 7.9 | 3,846 | 2.0 | 5,756 | 3.0 | 24,937 | 12.9 |
| Urban | 750,075 | 75,502 | 10.1 | 19,821 | 2.6 | 28,460 | 3.8 | 123,783 | 16.5 |
| **Eastern** | **834,968** | **61,020** | **7.3** | **14,056** | **1.7** | **20,549** | **2.5** | **95,625** | **11.5** |
| Rural | 738,526 | 52,118 | 7.1 | 11,854 | 1.6 | 17,496 | 2.4 | 81,468 | 11.0 |
| Urban | 96,442 | 8,902 | 9.2 | 2,202 | 2.3 | 3,053 | 3.2 | 14,157 | 14.7 |
| **Luapula** | **520,045** | **39,451** | **7.6** | **9,881** | **1.9** | **14,395** | **2.8** | **63,727** | **12.3** |
| Rural | 423,749 | 30,322 | 7.2 | 7,694 | 1.8 | 10,929 | 2.6 | 48,945 | 11.6 |
| Urban | 96,296 | 9,129 | 9.5 | 2,187 | 2.3 | 3,466 | 3.6 | 14,782 | 15.4 |
| **Lusaka** | **1,019,184** | **94,819** | **9.3** | **23,901** | **2.3** | **31,906** | **3.1** | **150,626** | **14.8** |
| Rural | 169,038 | 13,788 | 8.2 | 3,422 | 2.0 | 4,907 | 2.9 | 22,117 | 13.1 |
| Urban | 850,146 | 81,031 | 9.5 | 20,479 | 2.4 | 26,999 | 3.2 | 128,509 | 15.1 |
| **Muchinga** | **380,193** | **26,881** | **7.1** | **6,091** | **1.6** | **9,519** | **2.5** | **42,491** | **11.2** |
| Rural | 319,849 | 21,558 | 6.7 | 4,985 | 1.6 | 7,507 | 2.3 | 34,050 | 10.6 |
| Urban | 60,344 | 5,323 | 8.8 | 1,106 | 1.8 | 2,012 | 3.3 | 8,441 | 14.0 |
| **Northern** | **587,352** | **40,725** | **6.9** | **9,059** | **1.5** | **13,791** | **2.3** | **63,575** | **10.8** |
| Rural | 485,922 | 31,295 | 6.4 | 7,137 | 1.5 | 10,581 | 2.2 | 49,013 | 10.1 |
| Urban | 101,430 | 9,430 | 9.3 | 1,922 | 1.9 | 3,210 | 3.2 | 14,562 | 14.4 |
| **North Western** | **384,686** | **24,616** | **6.4** | **6,325** | **1.6** | **6,261** | **1.6** | **37,202** | **9.7** |
| Rural | 302,697 | 18,655 | 6.2 | 4,637 | 1.5 | 4,516 | 1.5 | 27,808 | 9.2 |
| Urban | 81,989 | 5,961 | 7.3 | 1,688 | 2.1 | 1,745 | 2.1 | 9,394 | 11.5 |
| **Southern** | **839,690** | **61,780** | **7.4** | **17,519** | **2.1** | **20,791** | **2.5** | **100,090** | **11.9** |
| Rural | 659,128 | 44,041 | 6.7 | 12,835 | 1.9 | 14,841 | 2.3 | 71,717 | 10.9 |
| Urban | 180,562 | 17,739 | 9.8 | 4,684 | 2.6 | 5,950 | 3.3 | 28,373 | 15.7 |
| **Western** | **449,441** | **43,247** | **9.6** | **11,877** | **2.6** | **12,221** | **2.7** | **67,345** | **15.0** |
| Rural | 393,467 | 36,816 | 9.4 | 10,168 | 2.6 | 9,919 | 2.5 | 56,903 | 14.5 |
| Urban | 55,974 | 6,431 | 11.5 | 1,709 | 3.1 | 2,302 | 4.1 | 10,442 | 18.7 |

## Table B10: Type of Orphan as a Percent of Population Aged 0-17 Years by 5 Year Age Group and Sex, Rural/Urban, Zambia 2010

| Age Group and Sex | Population 0-17 Years | Orphans by Type, as a Percent of Population Aged 0-17 Years | | | | | | | |
|---|---|---|---|---|---|---|---|---|---|
| | | Paternal Orphans | | Maternal Orphans | | Double Orphans | | Total Orphans | |
| | | Total | Percent | Total | Percent | Total | Percent | Total | Percent |
| **Zambia Total** | **6,638,301** | **539,436** | **8.1** | **136,660** | **2.1** | **184,546** | **2.8** | **860,642** | **13.0** |
| 0 - 4 | 2,214,887 | 88,552 | 4.0 | 17,263 | 0.8 | 18,085 | 0.8 | 123,900 | 5.6 |
| 5 - 9 | 1,856,336 | 130,404 | 7.0 | 32,453 | 1.7 | 35,354 | 1.9 | 198,211 | 10.7 |
| 10 - 14 | 1,699,042 | 191,630 | 11.3 | 51,265 | 3.0 | 70,626 | 4.2 | 313,521 | 18.5 |
| 15 - 17 | 868,036 | 128,850 | 14.8 | 35,679 | 4.1 | 60,481 | 7.0 | 225,010 | 25.9 |
| | | | | | | | | | |
| **Male** | **3,292,622** | **265,738** | **8.1** | **67,400** | **2.0** | **91,044** | **2.8** | **424,182** | **12.9** |
| 0 - 4 | 1,102,272 | 44,063 | 4.0 | 8,533 | 0.8 | 8,912 | 0.8 | 61,508 | 5.6 |
| 5 - 9 | 924,395 | 64,707 | 7.0 | 16,149 | 1.7 | 17,462 | 1.9 | 98,318 | 10.6 |
| 10 - 14 | 840,834 | 94,057 | 11.2 | 25,223 | 3.0 | 34,760 | 4.1 | 154,040 | 18.3 |
| 15 - 17 | 425,121 | 62,911 | 14.8 | 17,495 | 4.1 | 29,910 | 7.0 | 110,316 | 25.9 |
| | | | | | | | | | |
| **Female** | **3,345,679** | **273,698** | **8.2** | **69,260** | **2.1** | **93,502** | **2.8** | **436,460** | **13.0** |
| 0 - 4 | 1,112,615 | 44,489 | 4.0 | 8,730 | 0.8 | 9,173 | 0.8 | 62,392 | 5.6 |
| 5 - 9 | 931,941 | 65,697 | 7.0 | 16,304 | 1.7 | 17,892 | 1.9 | 99,893 | 10.7 |
| 10 - 14 | 858,208 | 97,573 | 11.4 | 26,042 | 3.0 | 35,866 | 4.2 | 159,481 | 18.6 |
| 15 - 17 | 442,915 | 65,939 | 14.9 | 18,184 | 4.1 | 30,571 | 6.9 | 114,694 | 25.9 |
| | | | | | | | | | |
| **Zambia Rural** | **4,208,117** | **302,875** | **7.2** | **76,922** | **1.8** | **100,998** | **2.4** | **480,795** | **11.4** |
| 0 - 4 | 1,454,385 | 55,118 | 3.8 | 10,707 | 0.7 | 11,717 | 0.8 | 77,542 | 5.3 |
| 5 - 9 | 1,212,247 | 78,035 | 6.4 | 19,448 | 1.6 | 21,774 | 1.8 | 119,257 | 9.8 |
| 10 - 14 | 1,045,977 | 104,854 | 10.0 | 28,472 | 2.7 | 38,292 | 3.7 | 171,618 | 16.4 |
| 15 - 17 | 495,508 | 64,868 | 13.1 | 18,295 | 3.7 | 29,215 | 5.9 | 112,378 | 22.7 |
| | | | | | | | | | |
| **Male** | **2,112,536** | **153,133** | **7.2** | **39,275** | **1.9** | **51,795** | **2.5** | **244,203** | **11.6** |
| 0 - 4 | 724,158 | 27,636 | 3.8 | 5,316 | 0.7 | 5,823 | 0.8 | 38,775 | 5.4 |
| 5 - 9 | 608,241 | 39,126 | 6.4 | 9,891 | 1.6 | 10,925 | 1.8 | 59,942 | 9.9 |
| 10 - 14 | 529,978 | 53,290 | 10.1 | 14,671 | 2.8 | 19,882 | 3.8 | 87,843 | 16.6 |
| 15 - 17 | 250,159 | 33,081 | 13.2 | 9,397 | 3.8 | 15,165 | 6.1 | 57,643 | 23.0 |
| | | | | | | | | | |
| **Female** | **2,095,581** | **149,742** | **7.1** | **37,647** | **1.8** | **49,203** | **2.3** | **236,592** | **11.3** |
| 0 - 4 | 730,227 | 27,482 | 3.8 | 5,391 | 0.7 | 5,894 | 0.8 | 38,767 | 5.3 |
| 5 - 9 | 604,006 | 38,909 | 6.4 | 9,557 | 1.6 | 10,849 | 1.8 | 59,315 | 9.8 |
| 10 - 14 | 515,999 | 51,564 | 10.0 | 13,801 | 2.7 | 18,410 | 3.6 | 83,775 | 16.2 |
| 15 - 17 | 245,349 | 31,787 | 13.0 | 8,898 | 3.6 | 14,050 | 5.7 | 54,735 | 22.3 |
| | | | | | | | | | |
| **Zambia Urban** | **2,430,184** | **236,561** | **9.7** | **59,738** | **2.5** | **83,548** | **3.4** | **379,847** | **15.6** |
| 0 - 4 | 760,502 | 33,434 | 4.4 | 6,556 | 0.9 | 6,368 | 0.8 | 46,358 | 6.1 |
| 5 - 9 | 644,089 | 52,369 | 8.1 | 13,005 | 2.0 | 13,580 | 2.1 | 78,954 | 12.3 |
| 10 - 14 | 653,065 | 86,776 | 13.3 | 22,793 | 3.5 | 32,334 | 5.0 | 141,903 | 21.7 |
| 15 - 17 | 372,528 | 63,982 | 17.2 | 17,384 | 4.7 | 31,266 | 8.4 | 112,632 | 30.2 |
| | | | | | | | | | |
| **Male** | **1,180,086** | **112,605** | **9.5** | **28,125** | **2.4** | **39,249** | **3.3** | **179,979** | **15.3** |
| 0 - 4 | 378,114 | 16,427 | 4.3 | 3,217 | 0.9 | 3,089 | 0.8 | 22,733 | 6.0 |
| 5 - 9 | 316,154 | 25,581 | 8.1 | 6,258 | 2.0 | 6,537 | 2.1 | 38,376 | 12.1 |
| 10 - 14 | 310,856 | 40,767 | 13.1 | 10,552 | 3.4 | 14,878 | 4.8 | 66,197 | 21.3 |
| 15 - 17 | 174,962 | 29,830 | 17.0 | 8,098 | 4.6 | 14,745 | 8.4 | 52,673 | 30.1 |
| | | | | | | | | | |
| **Female** | **1,250,098** | **123,956** | **9.9** | **31,613** | **2.5** | **44,299** | **3.5** | **199,868** | **16.0** |
| 0 - 4 | 382,388 | 17,007 | 4.4 | 3,339 | 0.9 | 3,279 | 0.9 | 23,625 | 6.2 |
| 5 - 9 | 327,935 | 26,788 | 8.2 | 6,747 | 2.1 | 7,043 | 2.1 | 40,578 | 12.4 |
| 10 - 14 | 342,209 | 46,009 | 13.4 | 12,241 | 3.6 | 17,456 | 5.1 | 75,706 | 22.1 |
| 15 - 17 | 197,566 | 34,152 | 17.3 | 9,286 | 4.7 | 16,521 | 8.4 | 59,959 | 30.3 |

## Table B11: Population of Orphans Aged 5-17 Years by Age (Single and Grouped), Sex and School Attendance, Rural/Urban, Zambia 2010

| Age | Population 5 -17 Years | Never Attended | | | Ever Attended | | | | | |
|---|---|---|---|---|---|---|---|---|---|---|
| | | | | | Not Currently Attending | | | Currently Attending | | |
| | | Total | Male | Female | Total | Male | Female | Total | Male | Female |
| **Zambia Total** | **736,742** | **146,094** | **74,652** | **71,442** | **70,395** | **30,215** | **40,180** | **520,253** | **257,807** | **262,446** |
| **5 - 9** | **198,211** | **94,081** | **48,043** | **46,038** | **5,919** | **2,925** | **2,994** | **98,211** | **47,350** | **50,861** |
| 5 | 34,617 | 27,935 | 14,031 | 13,904 | 703 | 342 | 361 | 5,979 | 2,865 | 3,114 |
| 6 | 37,029 | 25,272 | 12,790 | 12,482 | 853 | 433 | 420 | 10,904 | 5,174 | 5,730 |
| 7 | 41,922 | 19,198 | 9,961 | 9,237 | 1,215 | 601 | 614 | 21,509 | 10,468 | 11,041 |
| 8 | 42,244 | 12,921 | 6,659 | 6,262 | 1,466 | 702 | 764 | 27,857 | 13,331 | 14,526 |
| 9 | 42,399 | 8,755 | 4,602 | 4,153 | 1,682 | 847 | 835 | 31,962 | 15,512 | 16,450 |
| **10 - 14** | **313,521** | **34,847** | **18,479** | **16,368** | **20,702** | **9,965** | **10,737** | **257,972** | **125,596** | **132,376** |
| 10 | 68,227 | 11,830 | 6,365 | 5,465 | 3,191 | 1,600 | 1,591 | 53,206 | 25,953 | 27,253 |
| 11 | 49,867 | 6,116 | 3,153 | 2,963 | 2,769 | 1,370 | 1,399 | 40,982 | 19,676 | 21,306 |
| 12 | 65,101 | 6,573 | 3,602 | 2,971 | 4,006 | 1,984 | 2,022 | 54,522 | 26,842 | 27,680 |
| 13 | 62,340 | 5,119 | 2,652 | 2,467 | 4,398 | 2,086 | 2,312 | 52,823 | 25,596 | 27,227 |
| 14 | 67,986 | 5,209 | 2,707 | 2,502 | 6,338 | 2,925 | 3,413 | 56,439 | 27,529 | 28,910 |
| **15 - 17** | **225,010** | **17,166** | **8,130** | **9,036** | **43,774** | **17,325** | **26,449** | **164,070** | **84,861** | **79,209** |
| 15 | 76,997 | 6,217 | 3,087 | 3,130 | 10,446 | 4,513 | 5,933 | 60,334 | 30,505 | 29,829 |
| 16 | 75,780 | 5,581 | 2,613 | 2,968 | 13,995 | 5,419 | 8,576 | 56,204 | 28,509 | 27,695 |
| 17 | 72,233 | 5,368 | 2,430 | 2,938 | 19,333 | 7,393 | 11,940 | 47,532 | 25,847 | 21,685 |
| | | | | | | | | | | |
| **Zambia Rural** | **403,253** | **110,570** | **57,106** | **53,464** | **39,419** | **17,249** | **22,170** | **253,264** | **131,073** | **122,191** |
| **5 - 9** | **119,257** | **68,375** | **35,106** | **33,269** | **3,061** | **1,536** | **1,525** | **47,821** | **23,300** | **24,521** |
| 5 | 21,239 | 19,025 | 9,558 | 9,467 | 318 | 160 | 158 | 1,896 | 925 | 971 |
| 6 | 22,664 | 17,872 | 9,108 | 8,764 | 394 | 202 | 192 | 4,398 | 2,087 | 2,311 |
| 7 | 25,220 | 14,273 | 7,441 | 6,832 | 618 | 310 | 308 | 10,329 | 5,009 | 5,320 |
| 8 | 25,431 | 10,200 | 5,309 | 4,891 | 807 | 399 | 408 | 14,424 | 7,002 | 7,422 |
| 9 | 24,703 | 7,005 | 3,690 | 3,315 | 924 | 465 | 459 | 16,774 | 8,277 | 8,497 |
| **10 - 14** | **171,618** | **28,259** | **15,273** | **12,986** | **11,812** | **5,936** | **5,876** | **131,547** | **66,634** | **64,913** |
| 10 | 40,424 | 9,630 | 5,234 | 4,396 | 1,848 | 939 | 909 | 28,946 | 14,472 | 14,474 |
| 11 | 27,885 | 5,017 | 2,639 | 2,378 | 1,595 | 811 | 784 | 21,273 | 10,562 | 10,711 |
| 12 | 35,756 | 5,267 | 2,938 | 2,329 | 2,316 | 1,213 | 1,103 | 28,173 | 14,426 | 13,747 |
| 13 | 32,687 | 4,133 | 2,210 | 1,923 | 2,476 | 1,239 | 1,237 | 26,078 | 13,207 | 12,871 |
| 14 | 34,866 | 4,212 | 2,252 | 1,960 | 3,577 | 1,734 | 1,843 | 27,077 | 13,967 | 13,110 |
| **15 - 17** | **112,378** | **13,936** | **6,727** | **7,209** | **24,546** | **9,777** | **14,769** | **73,896** | **41,139** | **32,757** |
| 15 | 40,184 | 5,031 | 2,567 | 2,464 | 6,078 | 2,702 | 3,376 | 29,075 | 15,620 | 13,455 |
| 16 | 37,910 | 4,568 | 2,181 | 2,387 | 7,924 | 3,094 | 4,830 | 25,418 | 13,923 | 11,495 |
| 17 | 34,284 | 4,337 | 1,979 | 2,358 | 10,544 | 3,981 | 6,563 | 19,403 | 11,596 | 7,807 |
| | | | | | | | | | | |
| **Zambia Urban** | **333,489** | **35,524** | **17,546** | **17,978** | **30,976** | **12,966** | **18,010** | **266,989** | **126,734** | **140,255** |
| **5 - 9** | **78,954** | **25,706** | **12,937** | **12,769** | **2,858** | **1,389** | **1,469** | **50,390** | **24,050** | **26,340** |
| 5 | 13,378 | 8,910 | 4,473 | 4,437 | 385 | 182 | 203 | 4,083 | 1,940 | 2,143 |
| 6 | 14,365 | 7,400 | 3,682 | 3,718 | 459 | 231 | 228 | 6,506 | 3,087 | 3,419 |
| 7 | 16,702 | 4,925 | 2,520 | 2,405 | 597 | 291 | 306 | 11,180 | 5,459 | 5,721 |
| 8 | 16,813 | 2,721 | 1,350 | 1,371 | 659 | 303 | 356 | 13,433 | 6,329 | 7,104 |
| 9 | 17,696 | 1,750 | 912 | 838 | 758 | 382 | 376 | 15,188 | 7,235 | 7,953 |
| **10 - 14** | **141,903** | **6,588** | **3,206** | **3,382** | **8,890** | **4,029** | **4,861** | **126,425** | **58,962** | **67,463** |
| 10 | 27,803 | 2,200 | 1,131 | 1,069 | 1,343 | 661 | 682 | 24,260 | 11,481 | 12,779 |
| 11 | 21,982 | 1,099 | 514 | 585 | 1,174 | 559 | 615 | 19,709 | 9,114 | 10,595 |
| 12 | 29,345 | 1,306 | 664 | 642 | 1,690 | 771 | 919 | 26,349 | 12,416 | 13,933 |
| 13 | 29,653 | 986 | 442 | 544 | 1,922 | 847 | 1,075 | 26,745 | 12,389 | 14,356 |
| 14 | 33,120 | 997 | 455 | 542 | 2,761 | 1,191 | 1,570 | 29,362 | 13,562 | 15,800 |
| **15 - 17** | **112,632** | **3,230** | **1,403** | **1,827** | **19,228** | **7,548** | **11,680** | **90,174** | **43,722** | **46,452** |
| 15 | 36,813 | 1,186 | 520 | 666 | 4,368 | 1,811 | 2,557 | 31,259 | 14,885 | 16,374 |
| 16 | 37,870 | 1,013 | 432 | 581 | 6,071 | 2,325 | 3,746 | 30,786 | 14,586 | 16,200 |
| 17 | 37,949 | 1,031 | 451 | 580 | 8,789 | 3,412 | 5,377 | 28,129 | 14,251 | 13,878 |

## Table B12: Population of Orphans Aged 12-17 Years by Age, Sex and Economic Activity Status, Rural/Urban, Zambia 2010

| Sex and Age | Population 12-17 Years | Economically Active | | | | | Economically Inactive | | | |
|---|---|---|---|---|---|---|---|---|---|---|
| | | Total Labour Force Aged 12-17 Years | Employed | Unemployed | | | Total | Full Time House Wife/Home Maker | Full Time Student | Other |
| | | | | Total | Seeking Work | Not Seeking Work But Available for Work | | | | |
| **Zambia Total** | **420,437** | **73,704** | **58,315** | **15,389** | **7,287** | **8,102** | **346,733** | **13,778** | **294,566** | **38,389** |
| 12 | 65,101 | 9,051 | 7,615 | 1,436 | 539 | 897 | 56,050 | 1,398 | 48,371 | 6,281 |
| 13 | 62,340 | 8,233 | 6,859 | 1,374 | 522 | 852 | 54,107 | 1,396 | 47,105 | 5,606 |
| 14 | 67,986 | 9,952 | 8,124 | 1,828 | 758 | 1,070 | 58,034 | 1,642 | 50,352 | 6,040 |
| 15 | 76,997 | 13,653 | 10,852 | 2,801 | 1,255 | 1,546 | 63,344 | 2,264 | 54,181 | 6,899 |
| 16 | 75,780 | 14,928 | 11,520 | 3,408 | 1,748 | 1,660 | 60,852 | 3,098 | 50,935 | 6,819 |
| 17 | 72,233 | 17,887 | 13,345 | 4,542 | 2,465 | 2,077 | 54,346 | 3,980 | 43,622 | 6,744 |
| **Male** | **206,239** | **36,752** | **29,156** | **7,596** | **3,695** | **3,901** | **169,487** | **3,912** | **147,091** | **18,484** |
| 12 | 32,428 | 4,814 | 4,067 | 747 | 292 | 455 | 27,614 | 586 | 23,782 | 3,246 |
| 13 | 30,334 | 4,318 | 3,611 | 707 | 261 | 446 | 26,016 | 544 | 22,708 | 2,764 |
| 14 | 33,161 | 5,166 | 4,242 | 924 | 389 | 535 | 27,995 | 604 | 24,417 | 2,974 |
| 15 | 38,105 | 6,953 | 5,547 | 1,406 | 657 | 749 | 31,152 | 729 | 27,131 | 3,292 |
| 16 | 36,541 | 7,030 | 5,429 | 1,601 | 828 | 773 | 29,511 | 734 | 25,644 | 3,133 |
| 17 | 35,670 | 8,471 | 6,260 | 2,211 | 1,268 | 943 | 27,199 | 715 | 23,409 | 3,075 |
| **Female** | **214,198** | **36,952** | **29,159** | **7,793** | **3,592** | **4,201** | **177,246** | **9,866** | **147,475** | **19,905** |
| 12 | 32,673 | 4,237 | 3,548 | 689 | 247 | 442 | 28,436 | 812 | 24,589 | 3,035 |
| 13 | 32,006 | 3,915 | 3,248 | 667 | 261 | 406 | 28,091 | 852 | 24,397 | 2,842 |
| 14 | 34,825 | 4,786 | 3,882 | 904 | 369 | 535 | 30,039 | 1,038 | 25,935 | 3,066 |
| 15 | 38,892 | 6,700 | 5,305 | 1,395 | 598 | 797 | 32,192 | 1,535 | 27,050 | 3,607 |
| 16 | 39,239 | 7,898 | 6,091 | 1,807 | 920 | 887 | 31,341 | 2,364 | 25,291 | 3,686 |
| 17 | 36,563 | 9,416 | 7,085 | 2,331 | 1,197 | 1,134 | 27,147 | 3,265 | 20,213 | 3,669 |
| | | | | | | | | | | |
| **Zambia Rural** | **215,687** | **58,930** | **51,935** | **6,995** | **2,558** | **4,437** | **156,757** | **7,071** | **130,222** | **19,464** |
| 12 | 35,756 | 7,739 | 6,890 | 849 | 253 | 596 | 28,017 | 792 | 23,604 | 3,621 |
| 13 | 32,687 | 7,014 | 6,212 | 802 | 246 | 556 | 25,673 | 759 | 21,831 | 3,083 |
| 14 | 34,866 | 8,261 | 7,305 | 956 | 323 | 633 | 26,605 | 817 | 22,639 | 3,149 |
| 15 | 40,184 | 11,136 | 9,785 | 1,351 | 484 | 867 | 29,048 | 1,235 | 24,330 | 3,483 |
| 16 | 37,910 | 11,641 | 10,241 | 1,400 | 562 | 838 | 26,269 | 1,569 | 21,492 | 3,208 |
| 17 | 34,284 | 13,139 | 11,502 | 1,637 | 690 | 947 | 21,145 | 1,899 | 16,326 | 2,920 |
| **Male** | **110,829** | **29,533** | **25,918** | **3,615** | **1,323** | **2,292** | **81,296** | **2,076** | **69,346** | **9,874** |
| 12 | 18,577 | 4,174 | 3,717 | 457 | 138 | 319 | 14,403 | 344 | 12,156 | 1,903 |
| 13 | 16,656 | 3,718 | 3,295 | 423 | 121 | 302 | 12,938 | 314 | 11,057 | 1,567 |
| 14 | 17,953 | 4,333 | 3,829 | 504 | 168 | 336 | 13,620 | 311 | 11,660 | 1,649 |
| 15 | 20,889 | 5,691 | 4,991 | 700 | 258 | 442 | 15,198 | 415 | 13,024 | 1,759 |
| 16 | 19,198 | 5,490 | 4,793 | 697 | 277 | 420 | 13,708 | 379 | 11,764 | 1,565 |
| 17 | 17,556 | 6,127 | 5,293 | 834 | 361 | 473 | 11,429 | 313 | 9,685 | 1,431 |
| **Female** | **104,858** | **29,397** | **26,017** | **3,380** | **1,235** | **2,145** | **75,461** | **4,995** | **60,876** | **9,590** |
| 12 | 17,179 | 3,565 | 3,173 | 392 | 115 | 277 | 13,614 | 448 | 11,448 | 1,718 |
| 13 | 16,031 | 3,296 | 2,917 | 379 | 125 | 254 | 12,735 | 445 | 10,774 | 1,516 |
| 14 | 16,913 | 3,928 | 3,476 | 452 | 155 | 297 | 12,985 | 506 | 10,979 | 1,500 |
| 15 | 19,295 | 5,445 | 4,794 | 651 | 226 | 425 | 13,850 | 820 | 11,306 | 1,724 |
| 16 | 18,712 | 6,151 | 5,448 | 703 | 285 | 418 | 12,561 | 1,190 | 9,728 | 1,643 |
| 17 | 16,728 | 7,012 | 6,209 | 803 | 329 | 474 | 9,716 | 1,586 | 6,641 | 1,489 |

## Table B12: Population of Orphans Aged 12-17 Years by Age, Sex and Economic Activity Status, Rural/Urban, Zambia 2010

| Sex and Age | Population 12-17 Years | Economically Active | | | | | Economically Inactive | | | |
|---|---|---|---|---|---|---|---|---|---|---|
| | | Total Labour Force Aged 12-17 Years | Employed | Unemployed | | | Total | Full Time House Wife/Home Maker | Full Time Student | Other |
| | | | | Total | Seeking Work | Not Seeking Work But Available for Work | | | | |
| **Zambia Urban** | **204,750** | **14,774** | **6,380** | **8,394** | **4,729** | **3,665** | **189,976** | **6,707** | **164,344** | **18,925** |
| 12 | 29,345 | 1,312 | 725 | 587 | 286 | 301 | 28,033 | 606 | 24,767 | 2,660 |
| 13 | 29,653 | 1,219 | 647 | 572 | 276 | 296 | 28,434 | 637 | 25,274 | 2,523 |
| 14 | 33,120 | 1,691 | 819 | 872 | 435 | 437 | 31,429 | 825 | 27,713 | 2,891 |
| 15 | 36,813 | 2,517 | 1,067 | 1,450 | 771 | 679 | 34,296 | 1,029 | 29,851 | 3,416 |
| 16 | 37,870 | 3,287 | 1,279 | 2,008 | 1,186 | 822 | 34,583 | 1,529 | 29,443 | 3,611 |
| 17 | 37,949 | 4,748 | 1,843 | 2,905 | 1,775 | 1,130 | 33,201 | 2,081 | 27,296 | 3,824 |
| **Male** | **95,410** | **7,219** | **3,238** | **3,981** | **2,372** | **1,609** | **88,191** | **1,836** | **77,745** | **8,610** |
| 12 | 13,851 | 640 | 350 | 290 | 154 | 136 | 13,211 | 242 | 11,626 | 1,343 |
| 13 | 13,678 | 600 | 316 | 284 | 140 | 144 | 13,078 | 230 | 11,651 | 1,197 |
| 14 | 15,208 | 833 | 413 | 420 | 221 | 199 | 14,375 | 293 | 12,757 | 1,325 |
| 15 | 17,216 | 1,262 | 556 | 706 | 399 | 307 | 15,954 | 314 | 14,107 | 1,533 |
| 16 | 17,343 | 1,540 | 636 | 904 | 551 | 353 | 15,803 | 355 | 13,880 | 1,568 |
| 17 | 18,114 | 2,344 | 967 | 1,377 | 907 | 470 | 15,770 | 402 | 13,724 | 1,644 |
| **Female** | **109,340** | **7,555** | **3,142** | **4,413** | **2,357** | **2,056** | **101,785** | **4,871** | **86,599** | **10,315** |
| 12 | 15,494 | 672 | 375 | 297 | 132 | 165 | 14,822 | 364 | 13,141 | 1,317 |
| 13 | 15,975 | 619 | 331 | 288 | 136 | 152 | 15,356 | 407 | 13,623 | 1,326 |
| 14 | 17,912 | 858 | 406 | 452 | 214 | 238 | 17,054 | 532 | 14,956 | 1,566 |
| 15 | 19,597 | 1,255 | 511 | 744 | 372 | 372 | 18,342 | 715 | 15,744 | 1,883 |
| 16 | 20,527 | 1,747 | 643 | 1,104 | 635 | 469 | 18,780 | 1,174 | 15,563 | 2,043 |
| 17 | 19,835 | 2,404 | 876 | 1,528 | 868 | 660 | 17,431 | 1,679 | 13,572 | 2,180 |

## Table B13: Population 16 Years and Older with National Registration Cards (NRCs) by Sex, Province and Constituency, Zambia 2010

| Province | Constituency | Population 16 Years and Older | | | Population 16 Years and Older with Green NRCs | | | Percent with Green NRCs | | |
|---|---|---|---|---|---|---|---|---|---|---|
| | | Total | Male | Female | Total | Male | Female | Total | Male | Female |
| Central | **Total** | **620,462** | **301,919** | **318,543** | **499,902** | **243,042** | **256,860** | **80.6** | **80.5** | **80.6** |
| | Chisamba | 47,882 | 23,567 | 24,315 | 36,911 | 18,165 | 18,746 | 77.1 | 77.1 | 77.1 |
| | Katuba | 38,316 | 18,858 | 19,458 | 30,743 | 15,093 | 15,650 | 80.2 | 80.0 | 80.4 |
| | Keembe | 54,744 | 26,609 | 28,135 | 43,516 | 20,988 | 22,528 | 79.5 | 78.9 | 80.1 |
| | Bwacha | 44,247 | 21,519 | 22,728 | 36,113 | 17,589 | 18,524 | 81.6 | 81.7 | 81.5 |
| | Kabwe | 66,447 | 31,631 | 34,816 | 55,750 | 26,724 | 29,026 | 83.9 | 84.5 | 83.4 |
| | Kapiri Mposhi | 119,103 | 57,998 | 61,105 | 95,845 | 46,656 | 49,189 | 80.5 | 80.4 | 80.5 |
| | Mkushi North | 61,191 | 30,861 | 30,330 | 50,189 | 25,629 | 24,560 | 82.0 | 83.0 | 81.0 |
| | Mkushi South | 11,622 | 5,812 | 5,810 | 8,628 | 4,279 | 4,349 | 74.2 | 73.6 | 74.9 |
| | Mwembeshi | 24,633 | 12,107 | 12,526 | 19,651 | 9,649 | 10,002 | 79.8 | 79.7 | 79.8 |
| | Mumbwa | 40,291 | 19,372 | 20,919 | 32,295 | 15,429 | 16,866 | 80.2 | 79.6 | 80.6 |
| | Nangoma | 36,192 | 17,436 | 18,756 | 27,824 | 13,348 | 14,476 | 76.9 | 76.6 | 77.2 |
| | Chitambo | 21,497 | 10,013 | 11,484 | 17,752 | 8,158 | 9,594 | 82.6 | 81.5 | 83.5 |
| | Muchinga | 24,076 | 11,559 | 12,517 | 19,786 | 9,417 | 10,369 | 82.2 | 81.5 | 82.8 |
| | Serenje | 30,221 | 14,577 | 15,644 | 24,899 | 11,918 | 12,981 | 82.4 | 81.8 | 83.0 |
| Copperbelt | **Total** | **1,069,122** | **530,734** | **538,388** | **937,166** | **470,813** | **466,353** | **87.7** | **88.7** | **86.6** |
| | Chililabombwe | 50,222 | 25,891 | 24,331 | 46,442 | 24,141 | 22,301 | 92.5 | 93.2 | 91.7 |
| | Chingola | 68,158 | 33,467 | 34,691 | 62,195 | 30,875 | 31,320 | 91.3 | 92.3 | 90.3 |
| | Nchanga | 49,968 | 25,360 | 24,608 | 45,104 | 23,181 | 21,923 | 90.3 | 91.4 | 89.1 |
| | Kalulushi | 54,091 | 27,209 | 26,882 | 48,297 | 24,574 | 23,723 | 89.3 | 90.3 | 88.2 |
| | Chimwemwe | 63,680 | 31,319 | 32,361 | 53,607 | 26,936 | 26,671 | 84.2 | 86.0 | 82.4 |
| | Kamfinsa | 47,965 | 23,617 | 24,348 | 40,296 | 20,218 | 20,078 | 84.0 | 85.6 | 82.5 |
| | Kwacha | 73,949 | 36,858 | 37,091 | 63,670 | 32,403 | 31,267 | 86.1 | 87.9 | 84.3 |
| | Nkana | 48,170 | 24,108 | 24,062 | 42,771 | 21,738 | 21,033 | 88.8 | 90.2 | 87.4 |
| | Wusakile | 53,188 | 26,805 | 26,383 | 45,523 | 23,495 | 22,028 | 85.6 | 87.7 | 83.5 |
| | Luanshya | 54,627 | 26,674 | 27,953 | 48,231 | 23,742 | 24,489 | 88.3 | 89.0 | 87.6 |
| | Roan | 32,261 | 15,855 | 16,406 | 28,791 | 14,257 | 14,534 | 89.2 | 89.9 | 88.6 |
| | Kankoyo | 24,364 | 12,245 | 12,119 | 21,850 | 11,067 | 10,783 | 89.7 | 90.4 | 89.0 |
| | Kantanshi | 32,539 | 16,396 | 16,143 | 29,461 | 14,935 | 14,526 | 90.5 | 91.1 | 90.0 |
| | Mufulira | 33,605 | 16,320 | 17,285 | 29,802 | 14,568 | 15,234 | 88.7 | 89.3 | 88.1 |
| | Kafulafuta | 20,795 | 10,323 | 10,472 | 18,353 | 9,016 | 9,337 | 88.3 | 87.3 | 89.2 |
| | Lufwanyama | 37,231 | 18,413 | 18,818 | 32,642 | 15,994 | 16,648 | 87.7 | 86.9 | 88.5 |
| | Masaiti | 30,423 | 14,954 | 15,469 | 26,988 | 13,159 | 13,829 | 88.7 | 88.0 | 89.4 |
| | Mpongwe | 44,189 | 21,892 | 22,297 | 37,901 | 18,765 | 19,136 | 85.8 | 85.7 | 85.8 |
| | Bwana Mkubwa | 63,967 | 31,748 | 32,219 | 55,052 | 27,811 | 27,241 | 86.1 | 87.6 | 84.5 |
| | Chifubu | 55,090 | 27,028 | 28,062 | 47,396 | 23,530 | 23,866 | 86.0 | 87.1 | 85.0 |
| | Kabushi | 53,854 | 26,393 | 27,461 | 46,417 | 23,299 | 23,118 | 86.2 | 88.3 | 84.2 |
| | Ndola | 76,786 | 37,859 | 38,927 | 66,377 | 33,109 | 33,268 | 86.4 | 87.5 | 85.5 |

## Table B13: Population 16 Years and Older with National Registration Cards (NRCs) by Sex, Province and Constituency, Zambia 2010

| Province | Constituency | Population 16 Years and Older | | | Population 16 Years and Older with Green NRCs | | | Percent with Green NRCs | | |
|---|---|---|---|---|---|---|---|---|---|---|
| | | Total | Male | Female | Total | Male | Female | Total | Male | Female |
| Eastern | **Total** | **752,990** | **357,509** | **395,481** | **609,506** | **282,602** | **326,904** | **80.9** | **79.0** | **82.7** |
| | Chadiza | 28,541 | 13,606 | 14,935 | 24,037 | 11,131 | 12,906 | 84.2 | 81.8 | 86.4 |
| | Vubwi | 20,485 | 10,024 | 10,461 | 16,778 | 7,958 | 8,820 | 81.9 | 79.4 | 84.3 |
| | Chipangali | 57,714 | 28,299 | 29,415 | 42,798 | 20,130 | 22,668 | 74.2 | 71.1 | 77.1 |
| | Chipata Central | 82,243 | 39,963 | 42,280 | 65,917 | 31,976 | 33,941 | 80.1 | 80.0 | 80.3 |
| | Kasenengwa | 45,227 | 21,037 | 24,190 | 36,274 | 16,342 | 19,932 | 80.2 | 77.7 | 82.4 |
| | Luangeni | 36,689 | 17,278 | 19,411 | 29,724 | 13,550 | 16,174 | 81.0 | 78.4 | 83.3 |
| | Milanzi | 30,315 | 14,382 | 15,933 | 25,367 | 11,650 | 13,717 | 83.7 | 81.0 | 86.1 |
| | Mkaika | 45,687 | 21,870 | 23,817 | 37,698 | 17,609 | 20,089 | 82.5 | 80.5 | 84.3 |
| | Sinda | 38,503 | 18,102 | 20,401 | 32,840 | 14,949 | 17,891 | 85.3 | 82.6 | 87.7 |
| | Chasefu | 45,600 | 21,272 | 24,328 | 34,311 | 15,951 | 18,360 | 75.2 | 75.0 | 75.5 |
| | Lumezi | 41,615 | 19,439 | 22,176 | 34,052 | 15,469 | 18,583 | 81.8 | 79.6 | 83.8 |
| | Lundazi | 62,342 | 29,276 | 33,066 | 50,460 | 23,665 | 26,795 | 80.9 | 80.8 | 81.0 |
| | Malambo | 31,845 | 15,170 | 16,675 | 26,877 | 12,503 | 14,374 | 84.4 | 82.4 | 86.2 |
| | Nyimba | 40,006 | 18,822 | 21,184 | 33,628 | 15,335 | 18,293 | 84.1 | 81.5 | 86.4 |
| | Kapoche | 56,547 | 26,409 | 30,138 | 45,362 | 20,418 | 24,944 | 80.2 | 77.3 | 82.8 |
| | Petauke | 58,764 | 27,651 | 31,113 | 47,715 | 21,830 | 25,885 | 81.2 | 78.9 | 83.2 |
| | Msanzala | 30,867 | 14,909 | 15,958 | 25,668 | 12,136 | 13,532 | 83.2 | 81.4 | 84.8 |
| Luapula | **Total** | **456,630** | **213,884** | **242,746** | **395,076** | **182,023** | **213,053** | **86.5** | **85.1** | **87.8** |
| | Kawambwa | 22,367 | 10,686 | 11,681 | 19,373 | 9,114 | 10,259 | 86.6 | 85.3 | 87.8 |
| | Mwansabombwe | 20,762 | 9,591 | 11,171 | 18,481 | 8,291 | 10,190 | 89.0 | 86.4 | 91.2 |
| | Pambashe | 18,753 | 8,941 | 9,812 | 16,144 | 7,627 | 8,517 | 86.1 | 85.3 | 86.8 |
| | Bahati | 42,076 | 19,734 | 22,342 | 36,885 | 17,165 | 19,720 | 87.7 | 87.0 | 88.3 |
| | Chembe | 18,523 | 8,708 | 9,815 | 16,408 | 7,546 | 8,862 | 88.6 | 86.7 | 90.3 |
| | Mansa | 62,426 | 29,284 | 33,142 | 54,637 | 25,404 | 29,233 | 87.5 | 86.8 | 88.2 |
| | Chipili | 15,580 | 7,471 | 8,109 | 14,136 | 6,717 | 7,419 | 90.7 | 89.9 | 91.5 |
| | Mambilima | 12,379 | 5,823 | 6,556 | 11,181 | 5,164 | 6,017 | 90.3 | 88.7 | 91.8 |
| | Mwense | 29,281 | 13,570 | 15,711 | 26,391 | 12,037 | 14,354 | 90.1 | 88.7 | 91.4 |
| | Chienge | 53,101 | 24,918 | 28,183 | 45,183 | 20,900 | 24,283 | 85.1 | 83.9 | 86.2 |
| | Nchelenge | 71,170 | 34,252 | 36,918 | 60,547 | 28,437 | 32,110 | 85.1 | 83.0 | 87.0 |
| | Bangweulu | 42,037 | 19,130 | 22,907 | 35,391 | 15,780 | 19,611 | 84.2 | 82.5 | 85.6 |
| | Chifunabuli | 37,459 | 17,030 | 20,429 | 31,162 | 13,855 | 17,307 | 83.2 | 81.4 | 84.7 |
| | Luapula | 10,716 | 4,746 | 5,970 | 9,157 | 3,986 | 5,171 | 85.5 | 84.0 | 86.6 |
| Lusaka | **Total** | **1,202,706** | **592,320** | **610,386** | **1,022,432** | **515,910** | **506,522** | **85.0** | **87.1** | **83.0** |
| | Kafue | 62,574 | 30,755 | 31,819 | 54,008 | 26,878 | 27,130 | 86.3 | 87.4 | 85.3 |
| | Feira | 11,399 | 5,378 | 6,021 | 9,853 | 4,543 | 5,310 | 86.4 | 84.5 | 88.2 |
| | Chilanga | 55,416 | 28,282 | 27,134 | 47,622 | 24,723 | 22,899 | 85.9 | 87.4 | 84.4 |
| | Chongwe | 69,604 | 35,016 | 34,588 | 59,997 | 30,484 | 29,513 | 86.2 | 87.1 | 85.3 |
| | Rufunsa | 23,123 | 11,423 | 11,700 | 19,396 | 9,374 | 10,022 | 83.9 | 82.1 | 85.7 |
| | Chawama | 104,569 | 52,522 | 52,047 | 89,292 | 46,172 | 43,120 | 85.4 | 87.9 | 82.8 |
| | Kabwata | 105,575 | 49,453 | 56,122 | 93,649 | 44,633 | 49,016 | 88.7 | 90.3 | 87.3 |
| | Kanyama | 194,386 | 97,525 | 96,861 | 159,469 | 83,125 | 76,344 | 82.0 | 85.2 | 78.8 |
| | Lusaka Central | 68,471 | 32,272 | 36,199 | 60,152 | 28,817 | 31,335 | 87.9 | 89.3 | 86.6 |
| | Mandevu | 195,575 | 97,564 | 98,011 | 160,007 | 82,844 | 77,163 | 81.8 | 84.9 | 78.7 |
| | Matero | 157,996 | 78,156 | 79,840 | 134,833 | 68,577 | 66,256 | 85.3 | 87.7 | 83.0 |
| | Munali | 154,018 | 73,974 | 80,044 | 134,154 | 65,740 | 68,414 | 87.1 | 88.9 | 85.5 |

## Table B13: Population 16 Years and Older with National Registration Cards (NRCs) by Sex, Province and Constituency, Zambia 2010

| Province | Constituency | Population 16 Years and Older | | | Population 16 Years and Older with Green NRCs | | | Percent with Green NRCs | | |
|---|---|---|---|---|---|---|---|---|---|---|
| | | Total | Male | Female | Total | Male | Female | Total | Male | Female |
| Muchinga | **Total** | **324,048** | **152,013** | **172,035** | **263,225** | **122,629** | **140,596** | **81.2** | **80.7** | **81.7** |
| | Chama North | 23,260 | 10,771 | 12,489 | 19,357 | 8,686 | 10,671 | 83.2 | 80.6 | 85.4 |
| | Chama South | 22,190 | 9,974 | 12,216 | 18,849 | 8,139 | 10,710 | 84.9 | 81.6 | 87.7 |
| | Chinsali | 38,951 | 18,348 | 20,603 | 31,335 | 14,665 | 16,670 | 80.4 | 79.9 | 80.9 |
| | Mafinga | 29,325 | 13,302 | 16,023 | 23,465 | 10,511 | 12,954 | 80.0 | 79.0 | 80.8 |
| | Isoka West | 33,714 | 15,788 | 17,926 | 28,142 | 13,079 | 15,063 | 83.5 | 82.8 | 84.0 |
| | Kanchibiya | 36,121 | 16,870 | 19,251 | 28,530 | 13,311 | 15,219 | 79.0 | 78.9 | 79.1 |
| | Mfuwe | 11,735 | 5,289 | 6,446 | 9,248 | 4,114 | 5,134 | 78.8 | 77.8 | 79.6 |
| | Mpika | 44,916 | 21,601 | 23,315 | 35,799 | 17,289 | 18,510 | 79.7 | 80.0 | 79.4 |
| | Nakonde | 57,281 | 27,474 | 29,807 | 46,953 | 22,664 | 24,289 | 82.0 | 82.5 | 81.5 |
| | Shiwang'andu | 26,555 | 12,596 | 13,959 | 21,547 | 10,171 | 11,376 | 81.1 | 80.7 | 81.5 |
| Northern | **Total** | **499,575** | **235,203** | **264,372** | **413,443** | **193,406** | **220,037** | **82.8** | **82.2** | **83.2** |
| | Chilubi | 34,785 | 15,210 | 19,575 | 29,448 | 12,703 | 16,745 | 84.7 | 83.5 | 85.5 |
| | Chimbamilonga | 21,084 | 9,823 | 11,261 | 17,497 | 7,915 | 9,582 | 83.0 | 80.6 | 85.1 |
| | Kaputa | 30,639 | 14,117 | 16,522 | 25,858 | 11,707 | 14,151 | 84.4 | 82.9 | 85.6 |
| | Kasama | 78,114 | 37,695 | 40,419 | 65,433 | 31,659 | 33,774 | 83.8 | 84.0 | 83.6 |
| | Lubansenshi | 23,913 | 11,249 | 12,664 | 20,543 | 9,591 | 10,952 | 85.9 | 85.3 | 86.5 |
| | Lukashya | 33,618 | 16,297 | 17,321 | 28,505 | 13,761 | 14,744 | 84.8 | 84.4 | 85.1 |
| | Lunte | 26,347 | 12,825 | 13,522 | 21,780 | 10,472 | 11,308 | 82.7 | 81.7 | 83.6 |
| | Lupososhi | 31,046 | 14,395 | 16,651 | 26,473 | 12,141 | 14,332 | 85.3 | 84.3 | 86.1 |
| | Malole | 66,566 | 31,002 | 35,564 | 54,629 | 25,301 | 29,328 | 82.1 | 81.6 | 82.5 |
| | Mbala | 50,050 | 23,802 | 26,248 | 39,818 | 18,972 | 20,846 | 79.6 | 79.7 | 79.4 |
| | Mporokoso | 19,312 | 9,315 | 9,997 | 16,813 | 7,982 | 8,831 | 87.1 | 85.7 | 88.3 |
| | Mpulungu | 43,427 | 20,279 | 23,148 | 34,403 | 15,938 | 18,465 | 79.2 | 78.6 | 79.8 |
| | Senga Hill | 40,674 | 19,194 | 21,480 | 32,243 | 15,264 | 16,979 | 79.3 | 79.5 | 79.0 |
| North Western | **Total** | **323,125** | **153,202** | **169,923** | **267,853** | **124,399** | **143,454** | **82.9** | **81.2** | **84.4** |
| | Chavuma | 16,377 | 7,510 | 8,867 | 13,418 | 5,937 | 7,481 | 81.9 | 79.1 | 84.4 |
| | Kabompo East | 21,609 | 10,114 | 11,495 | 18,609 | 8,415 | 10,194 | 86.1 | 83.2 | 88.7 |
| | Kabompo West | 20,285 | 9,515 | 10,770 | 16,761 | 7,599 | 9,162 | 82.6 | 79.9 | 85.1 |
| | Kasempa | 29,332 | 13,483 | 15,849 | 25,036 | 11,246 | 13,790 | 85.4 | 83.4 | 87.0 |
| | Mufumbwe | 24,892 | 11,828 | 13,064 | 20,682 | 9,570 | 11,112 | 83.1 | 80.9 | 85.1 |
| | Mwinilunga East | 45,800 | 21,395 | 24,405 | 38,280 | 17,269 | 21,011 | 83.6 | 80.7 | 86.1 |
| | Mwinilunga West | 15,213 | 7,093 | 8,120 | 12,898 | 5,791 | 7,107 | 84.8 | 81.6 | 87.5 |
| | Solwezi Central | 64,236 | 31,810 | 32,426 | 54,306 | 27,175 | 27,131 | 84.5 | 85.4 | 83.7 |
| | Solwezi East | 15,261 | 7,284 | 7,977 | 12,301 | 5,732 | 6,569 | 80.6 | 78.7 | 82.3 |
| | Solwezi West | 33,524 | 16,020 | 17,504 | 25,493 | 12,012 | 13,481 | 76.0 | 75.0 | 77.0 |
| | Zambezi East | 26,820 | 12,522 | 14,298 | 21,874 | 9,899 | 11,975 | 81.6 | 79.1 | 83.8 |
| | Zambezi West | 9,776 | 4,628 | 5,148 | 8,195 | 3,754 | 4,441 | 83.8 | 81.1 | 86.3 |

## Table B13: Population 16 Years and Older with National Registration Cards (NRCs) by Sex, Province and Constituency, Zambia 2010

| Province | Constituency | Population 16 Years and Older | | | Population 16 Years and Older with Green NRCs | | | Percent with Green NRCs | | |
|---|---|---|---|---|---|---|---|---|---|---|
| | | Total | Male | Female | Total | Male | Female | Total | Male | Female |
| Southern | **Total** | **743,872** | **353,469** | **390,403** | **610,469** | **287,744** | **322,725** | **82.1** | **81.4** | **82.7** |
| | Choma | 59,822 | 28,602 | 31,220 | 48,642 | 23,265 | 25,377 | 81.3 | 81.3 | 81.3 |
| | Mbabala | 25,773 | 11,980 | 13,793 | 20,671 | 9,500 | 11,171 | 80.2 | 79.3 | 81.0 |
| | Pemba | 29,454 | 13,550 | 15,904 | 22,956 | 10,341 | 12,615 | 77.9 | 76.3 | 79.3 |
| | Gwembe | 23,389 | 10,858 | 12,531 | 19,046 | 8,538 | 10,508 | 81.4 | 78.6 | 83.9 |
| | Dundumwenze | 32,586 | 15,022 | 17,564 | 24,891 | 11,183 | 13,708 | 76.4 | 74.4 | 78.0 |
| | Kalomo | 48,740 | 23,062 | 25,678 | 39,563 | 18,477 | 21,086 | 81.2 | 80.1 | 82.1 |
| | Katombola | 47,609 | 22,833 | 24,776 | 40,425 | 19,072 | 21,353 | 84.9 | 83.5 | 86.2 |
| | Mapatizya | 30,435 | 14,056 | 16,379 | 24,871 | 11,081 | 13,790 | 81.7 | 78.8 | 84.2 |
| | Livingstone | 78,989 | 38,879 | 40,110 | 69,053 | 34,253 | 34,800 | 87.4 | 88.1 | 86.8 |
| | Chikankata | 28,949 | 14,038 | 14,911 | 23,673 | 11,494 | 12,179 | 81.8 | 81.9 | 81.7 |
| | Magoye | 33,506 | 16,140 | 17,366 | 26,636 | 12,733 | 13,903 | 79.5 | 78.9 | 80.1 |
| | Mazabuka | 52,901 | 26,608 | 26,293 | 43,409 | 22,372 | 21,037 | 82.1 | 84.1 | 80.0 |
| | Bweengwa | 26,183 | 12,115 | 14,068 | 20,989 | 9,530 | 11,459 | 80.2 | 78.7 | 81.5 |
| | Monze | 48,624 | 22,771 | 25,853 | 40,421 | 18,717 | 21,704 | 83.1 | 82.2 | 84.0 |
| | Moomba | 13,142 | 6,101 | 7,041 | 11,285 | 5,164 | 6,121 | 85.9 | 84.6 | 86.9 |
| | Itezhi Tezhi | 29,992 | 14,409 | 15,583 | 23,622 | 11,187 | 12,435 | 78.8 | 77.6 | 79.8 |
| | Namwala | 43,355 | 20,061 | 23,294 | 35,068 | 15,933 | 19,135 | 80.9 | 79.4 | 82.1 |
| | Siavonga | 42,203 | 19,913 | 22,290 | 35,334 | 16,530 | 18,804 | 83.7 | 83.0 | 84.4 |
| | Sinazongwe | 48,220 | 22,471 | 25,749 | 39,914 | 18,374 | 21,540 | 82.8 | 81.8 | 83.7 |
| Western | **Total** | **419,760** | **187,237** | **232,523** | **339,533** | **147,091** | **192,442** | **80.9** | **78.6** | **82.8** |
| | Kalabo | 26,683 | 11,211 | 15,472 | 21,925 | 8,887 | 13,038 | 82.2 | 79.3 | 84.3 |
| | Liuwa | 12,823 | 5,653 | 7,170 | 11,058 | 4,714 | 6,344 | 86.2 | 83.4 | 88.5 |
| | Sikongo | 20,910 | 8,347 | 12,563 | 17,825 | 6,939 | 10,886 | 85.2 | 83.1 | 86.7 |
| | Kaoma | 40,984 | 19,345 | 21,639 | 32,212 | 14,829 | 17,383 | 78.6 | 76.7 | 80.3 |
| | Luampa | 19,744 | 8,594 | 11,150 | 16,512 | 6,998 | 9,514 | 83.6 | 81.4 | 85.3 |
| | Mangango | 24,199 | 10,647 | 13,552 | 18,897 | 8,155 | 10,742 | 78.1 | 76.6 | 79.3 |
| | Lukulu East | 27,287 | 12,328 | 14,959 | 22,087 | 9,629 | 12,458 | 80.9 | 78.1 | 83.3 |
| | Lukulu West | 12,368 | 5,478 | 6,890 | 9,855 | 4,180 | 5,675 | 79.7 | 76.3 | 82.4 |
| | Luena | 24,427 | 10,844 | 13,583 | 18,902 | 7,975 | 10,927 | 77.4 | 73.5 | 80.4 |
| | Mongu | 46,267 | 21,277 | 24,990 | 36,804 | 16,691 | 20,113 | 79.5 | 78.4 | 80.5 |
| | Nalikwanda | 17,906 | 7,694 | 10,212 | 14,933 | 6,129 | 8,804 | 83.4 | 79.7 | 86.2 |
| | Nalolo | 25,701 | 11,322 | 14,379 | 19,537 | 8,197 | 11,340 | 76.0 | 72.4 | 78.9 |
| | Senanga | 33,323 | 14,832 | 18,491 | 26,931 | 11,644 | 15,287 | 80.8 | 78.5 | 82.7 |
| | Sinjembela | 40,571 | 17,718 | 22,853 | 31,799 | 13,566 | 18,233 | 78.4 | 76.6 | 79.8 |
| | Mulobezi | 13,625 | 6,305 | 7,320 | 11,811 | 5,346 | 6,465 | 86.7 | 84.8 | 88.3 |
| | Mwandi | 11,672 | 5,517 | 6,155 | 10,118 | 4,652 | 5,466 | 86.7 | 84.3 | 88.8 |
| | Sesheke | 21,270 | 10,125 | 11,145 | 18,327 | 8,560 | 9,767 | 86.2 | 84.5 | 87.6 |
| **Zambia Total** | | **6,412,290** | **3,077,490** | **3,334,800** | **5,358,605** | **2,569,659** | **2,788,946** | **83.6** | **83.5** | **83.6** |

## Table B14: Population 18 Years and Older (Eligible Voters) and Registered Voters at the time of the Census by Sex, Province and Constituency, Zambia 2010

| Province | Constituency | Population 18 Years and Older | | | Population 18 Years and Older Registered as Voters | | | Percent Registered as Voters | | |
|---|---|---|---|---|---|---|---|---|---|---|
| | | Total | Male | Female | Total | Male | Female | Total | Male | Female |
| Central | **Total** | **563,460** | **273,812** | **289,648** | **305,901** | **154,016** | **151,885** | **54.3** | **56.2** | **52.4** |
| | Bwacha | 40,086 | 19,489 | 20,597 | 24,822 | 12,524 | 12,298 | 61.9 | 64.3 | 59.7 |
| | Chisamba | 43,463 | 21,313 | 22,150 | 23,934 | 12,160 | 11,774 | 55.1 | 57.1 | 53.2 |
| | Chitambo | 19,539 | 9,054 | 10,485 | 11,040 | 5,196 | 5,844 | 56.5 | 57.4 | 55.7 |
| | Kabwe | 59,863 | 28,642 | 31,221 | 37,037 | 18,543 | 18,494 | 61.9 | 64.7 | 59.2 |
| | Kapiri Mposhi | 108,638 | 52,803 | 55,835 | 55,180 | 27,985 | 27,195 | 50.8 | 53.0 | 48.7 |
| | Katuba | 34,877 | 17,111 | 17,766 | 20,373 | 10,203 | 10,170 | 58.4 | 59.6 | 57.2 |
| | Keembe | 49,447 | 23,938 | 25,509 | 29,228 | 14,428 | 14,800 | 59.1 | 60.3 | 58.0 |
| | Mkushi North | 55,871 | 28,355 | 27,516 | 25,900 | 13,974 | 11,926 | 46.4 | 49.3 | 43.3 |
| | Mkushi South | 10,597 | 5,296 | 5,301 | 5,729 | 2,916 | 2,813 | 54.1 | 55.1 | 53.1 |
| | Muchinga | 21,878 | 10,493 | 11,385 | 8,982 | 4,537 | 4,445 | 41.1 | 43.2 | 39.0 |
| | Mwembeshi | 22,508 | 11,026 | 11,482 | 12,994 | 6,436 | 6,558 | 57.7 | 58.4 | 57.1 |
| | Mumbwa | 36,451 | 17,414 | 19,037 | 19,629 | 9,623 | 10,006 | 53.9 | 55.3 | 52.6 |
| | Nangoma | 32,779 | 15,668 | 17,111 | 18,622 | 9,202 | 9,420 | 56.8 | 58.7 | 55.1 |
| | Serenje | 27,463 | 13,210 | 14,253 | 12,431 | 6,289 | 6,142 | 45.3 | 47.6 | 43.1 |
| Copperbelt | **Total** | **971,599** | **484,115** | **487,484** | **642,210** | **338,084** | **304,126** | **66.1** | **69.8** | **62.4** |
| | Bwana Mkubwa | 57,930 | 28,906 | 29,024 | 36,414 | 19,301 | 17,113 | 62.9 | 66.8 | 59.0 |
| | Chifubu | 50,067 | 24,643 | 25,424 | 34,678 | 17,724 | 16,954 | 69.3 | 71.9 | 66.7 |
| | Chililabombwe | 45,641 | 23,709 | 21,932 | 32,180 | 17,729 | 14,451 | 70.5 | 74.8 | 65.9 |
| | Chimwemwe | 58,024 | 28,614 | 29,410 | 38,002 | 20,068 | 17,934 | 65.5 | 70.1 | 61.0 |
| | Chingola | 62,362 | 30,737 | 31,625 | 42,461 | 22,218 | 20,243 | 68.1 | 72.3 | 64.0 |
| | Kabushi | 49,017 | 24,149 | 24,868 | 31,538 | 16,792 | 14,746 | 64.3 | 69.5 | 59.3 |
| | Kafulafuta | 18,922 | 9,388 | 9,534 | 12,955 | 6,471 | 6,484 | 68.5 | 68.9 | 68.0 |
| | Kalulushi | 49,241 | 24,907 | 24,334 | 33,713 | 17,948 | 15,765 | 68.5 | 72.1 | 64.8 |
| | Kamfinsa | 43,642 | 21,590 | 22,052 | 27,617 | 14,426 | 13,191 | 63.3 | 66.8 | 59.8 |
| | Kankoyo | 22,033 | 11,073 | 10,960 | 16,284 | 8,591 | 7,693 | 73.9 | 77.6 | 70.2 |
| | Kantanshi | 29,202 | 14,770 | 14,432 | 21,009 | 11,193 | 9,816 | 71.9 | 75.8 | 68.0 |
| | Kwacha | 67,765 | 33,907 | 33,858 | 43,556 | 23,349 | 20,207 | 64.3 | 68.9 | 59.7 |
| | Luanshya | 49,614 | 24,236 | 25,378 | 32,783 | 16,752 | 16,031 | 66.1 | 69.1 | 63.2 |
| | Lufwanyama | 33,844 | 16,760 | 17,084 | 20,737 | 10,596 | 10,141 | 61.3 | 63.2 | 59.4 |
| | Masaiti | 27,732 | 13,585 | 14,147 | 17,335 | 8,667 | 8,668 | 62.5 | 63.8 | 61.3 |
| | Mpongwe | 40,190 | 19,890 | 20,300 | 21,420 | 11,202 | 10,218 | 53.3 | 56.3 | 50.3 |
| | Mufulira | 30,512 | 14,842 | 15,670 | 21,358 | 10,882 | 10,476 | 70.0 | 73.3 | 66.9 |
| | Nchanga | 45,151 | 23,035 | 22,116 | 32,584 | 17,549 | 15,035 | 72.2 | 76.2 | 68.0 |
| | Ndola | 70,266 | 34,781 | 35,485 | 44,387 | 22,982 | 21,405 | 63.2 | 66.1 | 60.3 |
| | Nkana | 44,060 | 22,218 | 21,842 | 28,973 | 15,637 | 13,336 | 65.8 | 70.4 | 61.1 |
| | Roan | 28,328 | 13,982 | 14,346 | 20,264 | 10,583 | 9,681 | 71.5 | 75.7 | 67.5 |
| | Wusakile | 48,056 | 24,393 | 23,663 | 31,962 | 17,424 | 14,538 | 66.5 | 71.4 | 61.4 |
| Eastern | **Total** | **689,672** | **325,492** | **364,180** | **416,050** | **195,068** | **220,982** | **60.3** | **59.9** | **60.7** |
| | Chadiza | 25,921 | 12,260 | 13,661 | 17,340 | 8,006 | 9,334 | 66.9 | 65.3 | 68.3 |
| | Chasefu | 41,758 | 19,305 | 22,453 | 26,172 | 12,392 | 13,780 | 62.7 | 64.2 | 61.4 |
| | Chipangali | 52,853 | 25,734 | 27,119 | 25,441 | 12,233 | 13,208 | 48.1 | 47.5 | 48.7 |
| | Chipata Central | 75,293 | 36,592 | 38,701 | 39,138 | 19,662 | 19,476 | 52.0 | 53.7 | 50.3 |
| | Kapoche | 51,831 | 24,010 | 27,821 | 29,261 | 13,247 | 16,014 | 56.5 | 55.2 | 57.6 |
| | Kasenengwa | 41,496 | 19,170 | 22,326 | 24,546 | 11,323 | 13,223 | 59.2 | 59.1 | 59.2 |
| | Luangeni | 33,602 | 15,734 | 17,868 | 20,762 | 9,491 | 11,271 | 61.8 | 60.3 | 63.1 |
| | Lumezi | 38,007 | 17,648 | 20,359 | 24,510 | 11,221 | 13,289 | 64.5 | 63.6 | 65.3 |
| | Lundazi | 57,356 | 26,796 | 30,560 | 37,763 | 18,176 | 19,587 | 65.8 | 67.8 | 64.1 |
| | Malambo | 29,023 | 13,729 | 15,294 | 19,204 | 9,023 | 10,181 | 66.2 | 65.7 | 66.6 |
| | Milanzi | 27,752 | 13,058 | 14,694 | 20,769 | 9,471 | 11,298 | 74.8 | 72.5 | 76.9 |
| | Mkaika | 41,806 | 19,861 | 21,945 | 26,103 | 12,306 | 13,797 | 62.4 | 62.0 | 62.9 |
| | Msanzala | 28,250 | 13,607 | 14,643 | 18,253 | 8,642 | 9,611 | 64.6 | 63.5 | 65.6 |
| | Nyimba | 36,530 | 17,018 | 19,512 | 21,950 | 9,976 | 11,974 | 60.1 | 58.6 | 61.4 |
| | Petauke | 53,904 | 25,203 | 28,701 | 30,242 | 13,946 | 16,296 | 56.1 | 55.3 | 56.8 |
| | Sinda | 35,484 | 16,585 | 18,899 | 22,644 | 10,247 | 12,397 | 63.8 | 61.8 | 65.6 |
| | Vubwi | 18,806 | 9,182 | 9,624 | 11,952 | 5,706 | 6,246 | 63.6 | 62.1 | 64.9 |

## Table B14: Population 18 Years and Older (Eligible Voters) and Registered Voters at the time of the Census by Sex, Province and Constituency, Zambia 2010

| Province | Constituency | Population 18 Years and Older | | | Population 18 Years and Older Registered as Voters | | | Percent Registered as Voters | | |
|---|---|---|---|---|---|---|---|---|---|---|
| | | Total | Male | Female | Total | Male | Female | Total | Male | Female |
| Luapula | **Total** | **417,745** | **194,930** | **222,815** | **273,141** | **130,077** | **143,064** | **65.4** | **66.7** | **64.2** |
| | Bahati | 38,296 | 17,868 | 20,428 | 23,705 | 11,603 | 12,102 | 61.9 | 64.9 | 59.2 |
| | Bangweulu | 38,367 | 17,358 | 21,009 | 25,685 | 11,667 | 14,018 | 66.9 | 67.2 | 66.7 |
| | Chembe | 16,730 | 7,862 | 8,868 | 11,156 | 5,291 | 5,865 | 66.7 | 67.3 | 66.1 |
| | Chienge | 49,130 | 23,025 | 26,105 | 26,070 | 12,743 | 13,327 | 53.1 | 55.3 | 51.1 |
| | Chifunabuli | 34,344 | 15,476 | 18,868 | 23,825 | 10,789 | 13,036 | 69.4 | 69.7 | 69.1 |
| | Chipili | 14,228 | 6,823 | 7,405 | 10,834 | 5,304 | 5,530 | 76.1 | 77.7 | 74.7 |
| | Kawambwa | 20,280 | 9,656 | 10,624 | 14,887 | 7,130 | 7,757 | 73.4 | 73.8 | 73.0 |
| | Luapula | 9,883 | 4,373 | 5,510 | 7,398 | 3,263 | 4,135 | 74.9 | 74.6 | 75.0 |
| | Mambilima | 11,354 | 5,302 | 6,052 | 8,883 | 4,173 | 4,710 | 78.2 | 78.7 | 77.8 |
| | Mansa | 56,871 | 26,647 | 30,224 | 35,860 | 17,632 | 18,228 | 63.1 | 66.2 | 60.3 |
| | Mwansabombwe | 18,878 | 8,642 | 10,236 | 14,650 | 6,675 | 7,975 | 77.6 | 77.2 | 77.9 |
| | Mwense | 26,912 | 12,380 | 14,532 | 19,535 | 9,089 | 10,446 | 72.6 | 73.4 | 71.9 |
| | Nchelenge | 65,341 | 31,393 | 33,948 | 37,843 | 18,520 | 19,323 | 57.9 | 59.0 | 56.9 |
| | Pambashe | 17,131 | 8,125 | 9,006 | 12,810 | 6,198 | 6,612 | 74.8 | 76.3 | 73.4 |
| Lusaka | **Total** | **1,107,167** | **547,786** | **559,381** | **631,816** | **337,880** | **293,936** | **57.1** | **61.7** | **52.5** |
| | Chawama | 96,748 | 48,866 | 47,882 | 54,759 | 29,965 | 24,794 | 56.6 | 61.3 | 51.8 |
| | Chilanga | 50,931 | 26,166 | 24,765 | 29,474 | 15,853 | 13,621 | 57.9 | 60.6 | 55.0 |
| | Chongwe | 63,513 | 31,974 | 31,539 | 37,615 | 19,594 | 18,021 | 59.2 | 61.3 | 57.1 |
| | Feira | 10,264 | 4,815 | 5,449 | 7,912 | 3,637 | 4,275 | 77.1 | 75.5 | 78.5 |
| | Kabwata | 97,445 | 45,986 | 51,459 | 60,020 | 30,660 | 29,360 | 61.6 | 66.7 | 57.1 |
| | Kafue | 56,842 | 27,985 | 28,857 | 33,991 | 17,607 | 16,384 | 59.8 | 62.9 | 56.8 |
| | Kanyama | 179,847 | 90,825 | 89,022 | 90,507 | 50,740 | 39,767 | 50.3 | 55.9 | 44.7 |
| | Lusaka Central | 63,220 | 29,983 | 33,237 | 37,843 | 19,298 | 18,545 | 59.9 | 64.4 | 55.8 |
| | Mandevu | 180,187 | 90,252 | 89,935 | 96,457 | 53,582 | 42,875 | 53.5 | 59.4 | 47.7 |
| | Matero | 145,287 | 72,214 | 73,073 | 85,248 | 46,187 | 39,061 | 58.7 | 64.0 | 53.5 |
| | Munali | 141,901 | 68,425 | 73,476 | 85,003 | 44,449 | 40,554 | 59.9 | 65.0 | 55.2 |
| | Rufunsa | 20,982 | 10,295 | 10,687 | 12,987 | 6,308 | 6,679 | 61.9 | 61.3 | 62.5 |
| Muchinga | **Total** | **296,682** | **138,575** | **158,107** | **189,189** | **91,042** | **98,147** | **63.8** | **65.7** | **62.1** |
| | Chama North | 21,391 | 9,829 | 11,562 | 16,090 | 7,330 | 8,760 | 75.2 | 74.6 | 75.8 |
| | Chama South | 20,328 | 9,032 | 11,296 | 16,481 | 7,128 | 9,353 | 81.1 | 78.9 | 82.8 |
| | Chinsali | 35,689 | 16,680 | 19,009 | 20,975 | 10,218 | 10,757 | 58.8 | 61.3 | 56.6 |
| | Mafinga | 26,785 | 12,081 | 14,704 | 19,529 | 8,898 | 10,631 | 72.9 | 73.7 | 72.3 |
| | Isoka West | 30,788 | 14,392 | 16,396 | 19,296 | 9,381 | 9,915 | 62.7 | 65.2 | 60.5 |
| | Kanchibiya | 33,234 | 15,467 | 17,767 | 19,597 | 9,483 | 10,114 | 59.0 | 61.3 | 56.9 |
| | Mfuwe | 10,824 | 4,859 | 5,965 | 6,091 | 2,776 | 3,315 | 56.3 | 57.1 | 55.6 |
| | Mpika | 40,855 | 19,605 | 21,250 | 24,283 | 12,179 | 12,104 | 59.4 | 62.1 | 57.0 |
| | Nakonde | 52,506 | 25,195 | 27,311 | 31,481 | 16,104 | 15,377 | 60.0 | 63.9 | 56.3 |
| | Shiwang'andu | 24,282 | 11,435 | 12,847 | 15,366 | 7,545 | 7,821 | 63.3 | 66.0 | 60.9 |
| Northern | **Total** | **457,232** | **214,571** | **242,661** | **308,313** | **149,707** | **158,606** | **67.4** | **69.8** | **65.4** |
| | Chilubi | 31,805 | 13,698 | 18,107 | 22,708 | 9,922 | 12,786 | 71.4 | 72.4 | 70.6 |
| | Chimbamilonga | 19,451 | 9,064 | 10,387 | 13,273 | 6,190 | 7,083 | 68.2 | 68.3 | 68.2 |
| | Kaputa | 28,304 | 13,021 | 15,283 | 18,354 | 8,638 | 9,716 | 64.8 | 66.3 | 63.6 |
| | Kasama | 71,072 | 34,210 | 36,862 | 49,707 | 25,094 | 24,613 | 69.9 | 73.4 | 66.8 |
| | Lubansenshi | 21,867 | 10,262 | 11,605 | 15,103 | 7,326 | 7,777 | 69.1 | 71.4 | 67.0 |
| | Lukashya | 30,794 | 14,941 | 15,853 | 21,728 | 10,871 | 10,857 | 70.6 | 72.8 | 68.5 |
| | Lunte | 24,109 | 11,685 | 12,424 | 16,216 | 8,124 | 8,092 | 67.3 | 69.5 | 65.1 |
| | Lupososhi | 28,429 | 13,130 | 15,299 | 19,671 | 9,320 | 10,351 | 69.2 | 71.0 | 67.7 |
| | Malole | 61,315 | 28,409 | 32,906 | 40,944 | 19,851 | 21,093 | 66.8 | 69.9 | 64.1 |
| | Mbala | 45,472 | 21,605 | 23,867 | 29,157 | 14,375 | 14,782 | 64.1 | 66.5 | 61.9 |
| | Mporokoso | 17,583 | 8,464 | 9,119 | 12,139 | 5,968 | 6,171 | 69.0 | 70.5 | 67.7 |
| | Mpulungu | 39,830 | 18,606 | 21,224 | 24,261 | 11,821 | 12,440 | 60.9 | 63.5 | 58.6 |
| | Senga Hill | 37,201 | 17,476 | 19,725 | 25,052 | 12,207 | 12,845 | 67.3 | 69.9 | 65.1 |

## Table B14: Population 18 Years and Older (Eligible Voters) and Registered Voters at the time of the Census by Sex, Province and Constituency, Zambia 2010

| Province | Constituency | Population 18 Years and Older | | | Population 18 Years and Older Registered as Voters | | | Percent Registered as Voters | | |
|---|---|---|---|---|---|---|---|---|---|---|
| | | Total | Male | Female | Total | Male | Female | Total | Male | Female |
| | **Total** | **293,285** | **138,428** | **154,857** | **211,765** | **100,835** | **110,930** | **72.2** | **72.8** | **71.6** |
| | Chavuma | 14,904 | 6,794 | 8,110 | 11,796 | 5,291 | 6,505 | 79.1 | 77.9 | 80.2 |
| | Kabompo East | 19,676 | 9,107 | 10,569 | 15,369 | 7,043 | 8,326 | 78.1 | 77.3 | 78.8 |
| | Kabompo West | 18,454 | 8,620 | 9,834 | 12,787 | 5,951 | 6,836 | 69.3 | 69.0 | 69.5 |
| | Kasempa | 26,396 | 12,064 | 14,332 | 18,014 | 8,379 | 9,635 | 68.2 | 69.5 | 67.2 |
| | Mufumbwe | 22,465 | 10,607 | 11,858 | 18,114 | 8,438 | 9,676 | 80.6 | 79.6 | 81.6 |
| **North Western** | Mwinilunga East | 41,334 | 19,166 | 22,168 | 32,587 | 14,874 | 17,713 | 78.8 | 77.6 | 79.9 |
| | Mwinilunga West | 13,733 | 6,307 | 7,426 | 11,341 | 5,152 | 6,189 | 82.6 | 81.7 | 83.3 |
| | Solwezi Central | 58,699 | 29,164 | 29,535 | 38,509 | 20,444 | 18,065 | 65.6 | 70.1 | 61.2 |
| | Solwezi East | 13,904 | 6,604 | 7,300 | 8,220 | 4,014 | 4,206 | 59.1 | 60.8 | 57.6 |
| | Solwezi West | 30,421 | 14,496 | 15,925 | 19,244 | 9,428 | 9,816 | 63.3 | 65.0 | 61.6 |
| | Zambezi East | 24,382 | 11,312 | 13,070 | 18,831 | 8,596 | 10,235 | 77.2 | 76.0 | 78.3 |
| | Zambezi West | 8,917 | 4,187 | 4,730 | 6,953 | 3,225 | 3,728 | 78.0 | 77.0 | 78.8 |
| | **Total** | **675,974** | **320,078** | **355,896** | **433,601** | **207,489** | **226,112** | **64.1** | **64.8** | **63.5** |
| | Bweengwa | 23,774 | 10,940 | 12,834 | 16,873 | 7,723 | 9,150 | 71.0 | 70.6 | 71.3 |
| | Chikankata | 26,254 | 12,668 | 13,586 | 16,608 | 8,256 | 8,352 | 63.3 | 65.2 | 61.5 |
| | Choma | 54,310 | 25,911 | 28,399 | 31,882 | 15,654 | 16,228 | 58.7 | 60.4 | 57.1 |
| | Dundumwenze | 29,346 | 13,371 | 15,975 | 16,467 | 7,621 | 8,846 | 56.1 | 57.0 | 55.4 |
| | Gwembe | 21,142 | 9,734 | 11,408 | 15,814 | 7,057 | 8,757 | 74.8 | 72.5 | 76.8 |
| | Itezhi Tezhi | 27,208 | 13,029 | 14,179 | 15,959 | 7,622 | 8,337 | 58.7 | 58.5 | 58.8 |
| | Kalomo | 43,942 | 20,733 | 23,209 | 28,136 | 13,291 | 14,845 | 64.0 | 64.1 | 64.0 |
| | Katombola | 43,520 | 20,724 | 22,796 | 29,637 | 13,973 | 15,664 | 68.1 | 67.4 | 68.7 |
| | Livingstone | 72,772 | 35,979 | 36,793 | 47,878 | 24,427 | 23,451 | 65.8 | 67.9 | 63.7 |
| **Southern** | Magoye | 30,469 | 14,610 | 15,859 | 18,369 | 9,053 | 9,316 | 60.3 | 62.0 | 58.7 |
| | Mapatizya | 27,567 | 12,632 | 14,935 | 19,092 | 8,486 | 10,606 | 69.3 | 67.2 | 71.0 |
| | Mazabuka | 48,357 | 24,473 | 23,884 | 28,694 | 15,451 | 13,243 | 59.3 | 63.1 | 55.4 |
| | Mbabala | 23,303 | 10,754 | 12,549 | 14,367 | 6,734 | 7,633 | 61.7 | 62.6 | 60.8 |
| | Monze | 44,248 | 20,619 | 23,629 | 29,958 | 14,100 | 15,858 | 67.7 | 68.4 | 67.1 |
| | Moomba | 11,861 | 5,458 | 6,403 | 8,556 | 3,974 | 4,582 | 72.1 | 72.8 | 71.6 |
| | Namwala | 39,325 | 18,083 | 21,242 | 24,317 | 11,135 | 13,182 | 61.8 | 61.6 | 62.1 |
| | Pemba | 26,491 | 12,089 | 14,402 | 17,475 | 7,923 | 9,552 | 66.0 | 65.5 | 66.3 |
| | Siavonga | 38,472 | 18,017 | 20,455 | 25,107 | 11,783 | 13,324 | 65.3 | 65.4 | 65.1 |
| | Sinazongwe | 43,613 | 20,254 | 23,359 | 28,412 | 13,226 | 15,186 | 65.1 | 65.3 | 65.0 |
| | **Total** | **384,990** | **170,311** | **214,679** | **265,106** | **114,319** | **150,787** | **68.9** | **67.1** | **70.2** |
| | Kalabo | 24,494 | 10,112 | 14,382 | 18,092 | 7,282 | 10,810 | 73.9 | 72.0 | 75.2 |
| | Kaoma | 37,319 | 17,535 | 19,784 | 22,910 | 10,453 | 12,457 | 61.4 | 59.6 | 63.0 |
| | Liuwa | 11,801 | 5,125 | 6,676 | 9,615 | 4,052 | 5,563 | 81.5 | 79.1 | 83.3 |
| | Luampa | 18,155 | 7,822 | 10,333 | 13,247 | 5,592 | 7,655 | 73.0 | 71.5 | 74.1 |
| | Luena | 22,433 | 9,867 | 12,566 | 16,323 | 6,805 | 9,518 | 72.8 | 69.0 | 75.7 |
| | Lukulu East | 25,002 | 11,194 | 13,808 | 17,170 | 7,500 | 9,670 | 68.7 | 67.0 | 70.0 |
| | Lukulu West | 11,353 | 5,022 | 6,331 | 8,542 | 3,627 | 4,915 | 75.2 | 72.2 | 77.6 |
| | Mangango | 22,221 | 9,695 | 12,526 | 14,523 | 6,281 | 8,242 | 65.4 | 64.8 | 65.8 |
| **Western** | Mongu | 42,340 | 19,436 | 22,904 | 26,411 | 11,985 | 14,426 | 62.4 | 61.7 | 63.0 |
| | Mulobezi | 12,456 | 5,710 | 6,746 | 9,102 | 4,104 | 4,998 | 73.1 | 71.9 | 74.1 |
| | Mwandi | 10,761 | 5,043 | 5,718 | 8,000 | 3,637 | 4,363 | 74.3 | 72.1 | 76.3 |
| | Nalikwanda | 16,395 | 6,953 | 9,442 | 11,564 | 4,802 | 6,762 | 70.5 | 69.1 | 71.6 |
| | Nalolo | 23,592 | 10,284 | 13,308 | 15,327 | 6,397 | 8,930 | 65.0 | 62.2 | 67.1 |
| | Senanga | 30,570 | 13,522 | 17,048 | 20,333 | 8,781 | 11,552 | 66.5 | 64.9 | 67.8 |
| | Sesheke | 19,536 | 9,274 | 10,262 | 13,254 | 6,195 | 7,059 | 67.8 | 66.8 | 68.8 |
| | Sikongo | 19,174 | 7,523 | 11,651 | 14,784 | 5,722 | 9,062 | 77.1 | 76.1 | 77.8 |
| | Sinjembela | 37,388 | 16,194 | 21,194 | 25,909 | 11,104 | 14,805 | 69.3 | 68.6 | 69.9 |
| **Zambia Total** | **All Constituencies** | **5,857,806** | **2,808,098** | **3,049,708** | **3,677,092** | **1,818,517** | **1,858,575** | **62.8** | **64.8** | **60.9** |

# Fertility Tables

## Table C1: Population of Women 12 Years and Older by Average Number of Children Ever Born, Children Ever Born Alive, Children Still Living, Children Dead, Sex of Child, Province and Rural/Urban, Zambia 2010

| Province and Rural/Urban | Number of Women 12 Years and Older | Average Number of Children Ever Born | Children Ever Born Alive | | | Children still Living | | | Children Dead | | |
|---|---|---|---|---|---|---|---|---|---|---|---|
| | | | Total | Male | Female | Total | Male | Female | Total | Male | Female |
| **Zambia Total** | **3,992,401** | **2.7** | **10,970,814** | **5,464,308** | **5,506,506** | **9,000,504** | **4,469,438** | **4,531,066** | **1,970,310** | **994,870** | **975,440** |
| Rural | 2,273,363 | 3.1 | 7,145,714 | 3,565,538 | 3,580,176 | 5,736,895 | 2,853,935 | 2,882,960 | 1,408,819 | 711,603 | 697,216 |
| Urban | 1,719,038 | 2.2 | 3,825,100 | 1,898,770 | 1,926,330 | 3,263,609 | 1,615,503 | 1,648,106 | 561,491 | 283,267 | 278,224 |
| **Central** | | | | | | | | | | | |
| **Total** | **386,021** | **2.8** | **1,084,458** | **538,919** | **545,539** | **899,774** | **446,814** | **452,960** | **184,684** | **92,105** | **92,579** |
| Rural | 276,841 | 3.0 | 840,090 | 418,093 | 421,997 | 695,084 | 345,875 | 349,209 | 145,006 | 72,218 | 72,788 |
| Urban | 109,180 | 2.2 | 244,368 | 120,826 | 123,542 | 204,690 | 100,939 | 103,751 | 39,678 | 19,887 | 19,791 |
| **Copperbelt** | | | | | | | | | | | |
| **Total** | **645,888** | **2.5** | **1,596,106** | **794,444** | **801,662** | **1,348,125** | **670,163** | **677,962** | **247,981** | **124,281** | **123,700** |
| Rural | 109,856 | 3.2 | 349,693 | 174,627 | 175,066 | 285,598 | 142,716 | 142,882 | 64,095 | 31,911 | 32,184 |
| Urban | 536,032 | 2.3 | 1,246,413 | 619,817 | 626,596 | 1,062,527 | 527,447 | 535,080 | 183,886 | 92,370 | 91,516 |
| **Eastern** | | | | | | | | | | | |
| **Total** | **471,841** | **3.1** | **1,471,563** | **733,697** | **737,866** | **1,153,965** | **573,013** | **580,952** | **317,598** | **160,684** | **156,914** |
| Rural | 407,337 | 3.2 | 1,320,399 | 658,742 | 661,657 | 1,027,534 | 510,525 | 517,009 | 292,865 | 148,217 | 144,648 |
| Urban | 64,504 | 2.3 | 151,164 | 74,955 | 76,209 | 126,431 | 62,488 | 63,943 | 24,733 | 12,467 | 12,266 |
| **Luapula** | | | | | | | | | | | |
| **Total** | **288,885** | **3.1** | **908,593** | **452,967** | **455,626** | **701,261** | **348,605** | **352,656** | **207,332** | **104,362** | **102,970** |
| Rural | 229,058 | 3.3 | 746,238 | 372,816 | 373,422 | 572,318 | 285,061 | 287,257 | 173,920 | 87,755 | 86,165 |
| Urban | 59,827 | 2.7 | 162,355 | 80,151 | 82,204 | 128,943 | 63,544 | 65,399 | 33,412 | 16,607 | 16,805 |
| **Lusaka** | | | | | | | | | | | |
| **Total** | **727,097** | **2.2** | **1,575,862** | **783,761** | **792,101** | **1,356,193** | **672,230** | **683,963** | **219,669** | **111,531** | **108,138** |
| Rural | 98,312 | 2.8 | 273,863 | 136,457 | 137,406 | 228,866 | 113,834 | 115,032 | 44,997 | 22,623 | 22,374 |
| Urban | 628,785 | 2.1 | 1,301,999 | 647,304 | 654,695 | 1,127,327 | 558,396 | 568,931 | 174,672 | 88,908 | 85,764 |
| **Muchinga** | | | | | | | | | | | |
| **Total** | **206,469** | **3.1** | **635,128** | **318,258** | **316,870** | **515,417** | **257,034** | **258,383** | **119,711** | **61,224** | **58,487** |
| Rural | 168,964 | 3.2 | 544,463 | 273,179 | 271,284 | 437,607 | 218,542 | 219,065 | 106,856 | 54,637 | 52,219 |
| Urban | 37,505 | 2.4 | 90,665 | 45,079 | 45,586 | 77,810 | 38,492 | 39,318 | 12,855 | 6,587 | 6,268 |
| **Northern** | | | | | | | | | | | |
| **Total** | **315,375** | **3.2** | **993,623** | **497,129** | **496,494** | **796,337** | **396,666** | **399,671** | **197,286** | **100,463** | **96,823** |
| Rural | 252,707 | 3.3 | 832,762 | 417,299 | 415,463 | 662,636 | 330,703 | 331,933 | 170,126 | 86,596 | 83,530 |
| Urban | 62,668 | 2.6 | 160,861 | 79,830 | 81,031 | 133,701 | 65,963 | 67,738 | 27,160 | 13,867 | 13,293 |
| **North-Western** | | | | | | | | | | | |
| **Total** | **206,434** | **2.9** | **601,412** | **299,257** | **302,155** | **511,558** | **254,078** | **257,480** | **89,854** | **45,179** | **44,675** |
| Rural | 155,829 | 3.1 | 486,553 | 242,678 | 243,875 | 410,731 | 204,468 | 206,263 | 75,822 | 38,210 | 37,612 |
| Urban | 50,605 | 2.3 | 114,859 | 56,579 | 58,280 | 100,827 | 49,610 | 51,217 | 14,032 | 6,969 | 7,063 |
| **Southern** | | | | | | | | | | | |
| **Total** | **470,300** | **2.8** | **1,323,348** | **659,493** | **663,855** | **1,111,794** | **552,322** | **559,472** | **211,554** | **107,171** | **104,383** |
| Rural | 340,709 | 3.1 | 1,057,117 | 527,728 | 529,389 | 881,344 | 438,523 | 442,821 | 175,773 | 89,205 | 86,568 |
| Urban | 129,591 | 2.1 | 266,231 | 131,765 | 134,466 | 230,450 | 113,799 | 116,651 | 35,781 | 17,966 | 17,815 |
| **Western** | | | | | | | | | | | |
| **Total** | **274,091** | **2.8** | **780,721** | **386,383** | **394,338** | **606,080** | **298,513** | **307,567** | **174,641** | **87,870** | **86,771** |
| Rural | 233,750 | 3.0 | 694,536 | 343,919 | 350,617 | 535,177 | 263,688 | 271,489 | 159,359 | 80,231 | 79,128 |
| Urban | 40,341 | 2.1 | 86,185 | 42,464 | 43,721 | 70,903 | 34,825 | 36,078 | 15,282 | 7,639 | 7,643 |

## Table C2: Population of Women aged 12 Years and Older by Average Number of Children Ever Born Alive, Children Ever Born Alive, Children Still Living, Children Dead, Sex of Child, 5 Year Age Group and Rural/Urban, Zambia 2010

| Age Group and Rural/Urban | Number of Women 12 Years and Older | Average Number of Children Ever Born | Children Ever Born | | | Children Still Living | | | Children Dead | | |
|---|---|---|---|---|---|---|---|---|---|---|---|
| | | | Total | Male | Female | Total | Male | Female | Total | Male | Female |
| **Zambia Total** | **3,992,401** | **2.7** | **10,970,814** | **5,464,308** | **5,506,506** | **9,000,504** | **4,469,438** | **4,531,066** | **1,970,310** | **994,870** | **975,440** |
| 12 - 14 | 486,104 | 0.0 | 7,947 | 4,000 | 3,947 | 6,924 | 3,431 | 3,493 | 1,023 | 569 | 454 |
| 15 - 19 | 735,025 | 0.2 | 175,811 | 87,622 | 88,189 | 161,182 | 79,880 | 81,302 | 14,629 | 7,742 | 6,887 |
| 20 - 24 | 612,600 | 1.3 | 822,610 | 409,373 | 413,237 | 752,608 | 372,507 | 380,101 | 70,002 | 36,866 | 33,136 |
| 25 - 29 | 541,751 | 2.6 | 1,419,661 | 706,979 | 712,682 | 1,279,281 | 633,721 | 645,560 | 140,380 | 73,258 | 67,122 |
| 30 - 34 | 403,076 | 3.9 | 1,553,530 | 774,823 | 778,707 | 1,371,087 | 680,133 | 690,954 | 182,443 | 94,690 | 87,753 |
| 35 - 39 | 314,852 | 4.8 | 1,525,804 | 763,380 | 762,424 | 1,319,083 | 657,096 | 661,987 | 206,721 | 106,284 | 100,437 |
| 40 - 44 | 215,331 | 5.6 | 1,210,821 | 606,130 | 604,691 | 1,013,909 | 505,437 | 508,472 | 196,912 | 100,693 | 96,219 |
| 45 - 49 | 180,156 | 6.0 | 1,080,764 | 540,626 | 540,138 | 881,305 | 440,076 | 441,229 | 199,459 | 100,550 | 98,909 |
| 50 - 54 | 141,558 | 6.2 | 879,792 | 439,039 | 440,753 | 683,742 | 342,048 | 341,694 | 196,050 | 96,991 | 99,059 |
| 55 - 59 | 94,791 | 6.4 | 603,169 | 299,663 | 303,506 | 449,826 | 224,820 | 225,006 | 153,343 | 74,843 | 78,500 |
| 60 - 64 | 88,456 | 6.5 | 573,756 | 284,473 | 289,283 | 393,365 | 196,162 | 197,203 | 180,391 | 88,311 | 92,080 |
| 65+ | 178,701 | 6.3 | 1,117,149 | 548,200 | 568,949 | 688,192 | 334,127 | 354,065 | 428,957 | 214,073 | 214,884 |
| | | | | | | | | | | | |
| **Zambia Rural** | **2,273,363** | **3.1** | **7,145,714** | **3,565,538** | **3,580,176** | **5,736,895** | **2,853,935** | **2,882,960** | **1,408,819** | **711,603** | **697,216** |
| 12 - 14 | 282,652 | 0.0 | 4,987 | 2,570 | 2,417 | 4,307 | 2,190 | 2,117 | 680 | 380 | 300 |
| 15 - 19 | 405,259 | 0.3 | 121,237 | 60,581 | 60,656 | 110,453 | 54,881 | 55,572 | 10,784 | 5,700 | 5,084 |
| 20 - 24 | 328,902 | 1.6 | 539,787 | 268,274 | 271,513 | 490,061 | 242,063 | 247,998 | 49,726 | 26,211 | 23,515 |
| 25 - 29 | 288,345 | 3.1 | 897,227 | 446,891 | 450,336 | 798,681 | 395,600 | 403,081 | 98,546 | 51,291 | 47,255 |
| 30 - 34 | 216,425 | 4.5 | 970,606 | 484,771 | 485,835 | 842,695 | 418,765 | 423,930 | 127,911 | 66,006 | 61,905 |
| 35 - 39 | 178,320 | 5.5 | 977,140 | 490,345 | 486,795 | 828,790 | 414,237 | 414,553 | 148,350 | 76,108 | 72,242 |
| 40 - 44 | 127,920 | 6.2 | 790,430 | 397,927 | 392,503 | 646,028 | 324,016 | 322,012 | 144,402 | 73,911 | 70,491 |
| 45 - 49 | 108,965 | 6.4 | 695,798 | 349,456 | 346,342 | 551,315 | 276,443 | 274,872 | 144,483 | 73,013 | 71,470 |
| 50 - 54 | 86,527 | 6.4 | 556,535 | 278,388 | 278,147 | 419,006 | 210,069 | 208,937 | 137,529 | 68,319 | 69,210 |
| 55 - 59 | 59,286 | 6.5 | 385,233 | 191,460 | 193,773 | 279,684 | 139,796 | 139,888 | 105,549 | 51,664 | 53,885 |
| 60 - 64 | 61,277 | 6.5 | 400,026 | 198,647 | 201,379 | 270,834 | 135,041 | 135,793 | 129,192 | 63,606 | 65,586 |
| 65+ | 129,485 | 6.2 | 806,708 | 396,228 | 410,480 | 495,041 | 240,834 | 254,207 | 311,667 | 155,394 | 156,273 |
| | | | | | | | | | | | |
| **Zambia Urban** | **1,719,038** | **2.2** | **3,825,100** | **1,898,770** | **1,926,330** | **3,263,609** | **1,615,503** | **1,648,106** | **561,491** | **283,267** | **278,224** |
| 12 - 14 | 203,452 | 0.0 | 2,960 | 1,430 | 1,530 | 2,617 | 1,241 | 1,376 | 343 | 189 | 154 |
| 15 - 19 | 329,766 | 0.2 | 54,574 | 27,041 | 27,533 | 50,729 | 24,999 | 25,730 | 3,845 | 2,042 | 1,803 |
| 20 - 24 | 283,698 | 1.0 | 282,823 | 141,099 | 141,724 | 262,547 | 130,444 | 132,103 | 20,276 | 10,655 | 9,621 |
| 25 - 29 | 253,406 | 2.1 | 522,434 | 260,088 | 262,346 | 480,600 | 238,121 | 242,479 | 41,834 | 21,967 | 19,867 |
| 30 - 34 | 186,651 | 3.1 | 582,924 | 290,052 | 292,872 | 528,392 | 261,368 | 267,024 | 54,532 | 28,684 | 25,848 |
| 35 - 39 | 136,532 | 4.0 | 548,664 | 273,035 | 275,629 | 490,293 | 242,859 | 247,434 | 58,371 | 30,176 | 28,195 |
| 40 - 44 | 87,411 | 4.8 | 420,391 | 208,203 | 212,188 | 367,881 | 181,421 | 186,460 | 52,510 | 26,782 | 25,728 |
| 45 - 49 | 71,191 | 5.4 | 384,966 | 191,170 | 193,796 | 329,990 | 163,633 | 166,357 | 54,976 | 27,537 | 27,439 |
| 50 - 54 | 55,031 | 5.9 | 323,257 | 160,651 | 162,606 | 264,736 | 131,979 | 132,757 | 58,521 | 28,672 | 29,849 |
| 55 - 59 | 35,505 | 6.1 | 217,936 | 108,203 | 109,733 | 170,142 | 85,024 | 85,118 | 47,794 | 23,179 | 24,615 |
| 60 - 64 | 27,179 | 6.4 | 173,730 | 85,826 | 87,904 | 122,531 | 61,121 | 61,410 | 51,199 | 24,705 | 26,494 |
| 65+ | 49,216 | 6.3 | 310,441 | 151,972 | 158,469 | 193,151 | 93,293 | 99,858 | 117,290 | 58,679 | 58,611 |

## Table C2i. Population of Women 12-49 Years by Age Group, Number of Children Born Alive 12 Months Prior to the Census, Children Still Living, Children Dead, Sex of Child and Rural/Urban, Zambia 2010

| Age Group and Rural/Urban | Number of Women 12 Years and Older | Children Born Alive 12 Months Prior to the Census | | | Children Still Living | | | Children Dead | | |
|---|---|---|---|---|---|---|---|---|---|---|
| | | Total | Male | Female | Total | Male | Female | Total | Male | Female |
| **Zambia Total** | **3,488,895** | **442,998** | **224,756** | **218,242** | **423,116** | **214,441** | **208,675** | **19,882** | **10,315** | **9,567** |
| 12 - 14 | 486,104 | 921 | 470 | 451 | 870 | 447 | 423 | 51 | 23 | 28 |
| 15 - 19 | 735,025 | 58,999 | 29,701 | 29,298 | 56,523 | 28,406 | 28,117 | 2,476 | 1,295 | 1,181 |
| 20 - 24 | 612,600 | 128,270 | 65,048 | 63,222 | 123,381 | 62,419 | 60,962 | 4,889 | 2,629 | 2,260 |
| 25 - 29 | 541,751 | 114,701 | 58,366 | 56,335 | 109,784 | 55,861 | 53,923 | 4,917 | 2,505 | 2,412 |
| 30 - 34 | 403,076 | 74,066 | 37,666 | 36,400 | 70,501 | 35,842 | 34,659 | 3,565 | 1,824 | 1,741 |
| 35 - 39 | 314,852 | 45,452 | 23,072 | 22,380 | 42,958 | 21,789 | 21,169 | 2,494 | 1,283 | 1,211 |
| 40 - 44 | 215,331 | 16,150 | 8,181 | 7,969 | 15,087 | 7,635 | 7,452 | 1,063 | 546 | 517 |
| 45 - 49 | 180,156 | 4,439 | 2,252 | 2,187 | 4,012 | 2,042 | 1,970 | 427 | 210 | 217 |
| **Zambia Rural** | **1,936,788** | **294,803** | **149,436** | **145,367** | **281,341** | **142,357** | **138,984** | **13,462** | **7,079** | **6,383** |
| 12 - 14 | 282,652 | 672 | 342 | 330 | 630 | 323 | 307 | 42 | 19 | 23 |
| 15 - 19 | 405,259 | 41,899 | 21,054 | 20,845 | 40,055 | 20,075 | 19,980 | 1,844 | 979 | 865 |
| 20 - 24 | 328,902 | 84,736 | 42,823 | 41,913 | 81,446 | 41,041 | 40,405 | 3,290 | 1,782 | 1,508 |
| 25 - 29 | 288,345 | 72,639 | 37,041 | 35,598 | 69,506 | 35,413 | 34,093 | 3,133 | 1,628 | 1,505 |
| 30 - 34 | 216,425 | 47,489 | 24,077 | 23,412 | 45,182 | 22,876 | 22,306 | 2,307 | 1,201 | 1,106 |
| 35 - 39 | 178,320 | 31,641 | 16,135 | 15,506 | 29,939 | 15,251 | 14,688 | 1,702 | 884 | 818 |
| 40 - 44 | 127,920 | 12,286 | 6,213 | 6,073 | 11,479 | 5,802 | 5,677 | 807 | 411 | 396 |
| 45 - 49 | 108,965 | 3,441 | 1,751 | 1,690 | 3,104 | 1,576 | 1,528 | 337 | 175 | 162 |
| **Zambia Urban** | **1,552,107** | **148,195** | **75,320** | **72,875** | **141,775** | **72,084** | **69,691** | **6,420** | **3,236** | **3,184** |
| 12 - 14 | 203,452 | 249 | 128 | 121 | 240 | 124 | 116 | 9 | 4 | 5 |
| 15 - 19 | 329,766 | 17,100 | 8,647 | 8,453 | 16,468 | 8,331 | 8,137 | 632 | 316 | 316 |
| 20 - 24 | 283,698 | 43,534 | 22,225 | 21,309 | 41,935 | 21,378 | 20,557 | 1,599 | 847 | 752 |
| 25 - 29 | 253,406 | 42,062 | 21,325 | 20,737 | 40,278 | 20,448 | 19,830 | 1,784 | 877 | 907 |
| 30 - 34 | 186,651 | 26,577 | 13,589 | 12,988 | 25,319 | 12,966 | 12,353 | 1,258 | 623 | 635 |
| 35 - 39 | 136,532 | 13,811 | 6,937 | 6,874 | 13,019 | 6,538 | 6,481 | 792 | 399 | 393 |
| 40 - 44 | 87,411 | 3,864 | 1,968 | 1,896 | 3,608 | 1,833 | 1,775 | 256 | 135 | 121 |
| 45 - 49 | 71,191 | 998 | 501 | 497 | 908 | 466 | 442 | 90 | 35 | 55 |

## Table C3: Population of Women 12 Years and Older by Number of Children Ever Born Alive, Children Still Living, Children Dead, Sex of Child, Age Group and Marital Status, Rural/Urban, Zambia 2010

| Age Group and Marital Status | Number of Women 12 Years and Older | Children Ever Born Alive | | | Children Still Living | | | Children Dead | | |
|---|---|---|---|---|---|---|---|---|---|---|
| | | Total | Male | Female | Total | Male | Female | Total | Male | Female |
| **Zambia Total** | **3,992,401** | **10,970,814** | **5,464,308** | **5,506,506** | **9,000,504** | **4,469,438** | **4,531,066** | **1,970,310** | **994,870** | **975,440** |
| 12 - 14 | 486,104 | 7,947 | 4,000 | 3,947 | 6,924 | 3,431 | 3,493 | 1,023 | 569 | 454 |
| 15 - 19 | 735,025 | 175,811 | 87,622 | 88,189 | 161,182 | 79,880 | 81,302 | 14,629 | 7,742 | 6,887 |
| 20 - 24 | 612,600 | 822,610 | 409,373 | 413,237 | 752,608 | 372,507 | 380,101 | 70,002 | 36,866 | 33,136 |
| 25 - 29 | 541,751 | 1,419,661 | 706,979 | 712,682 | 1,279,281 | 633,721 | 645,560 | 140,380 | 73,258 | 67,122 |
| 30 - 34 | 403,076 | 1,553,530 | 774,823 | 778,707 | 1,371,087 | 680,133 | 690,954 | 182,443 | 94,690 | 87,753 |
| 35 - 39 | 314,852 | 1,525,804 | 763,380 | 762,424 | 1,319,083 | 657,096 | 661,987 | 206,721 | 106,284 | 100,437 |
| 40 - 44 | 215,331 | 1,210,821 | 606,130 | 604,691 | 1,013,909 | 505,437 | 508,472 | 196,912 | 100,693 | 96,219 |
| 45 - 49 | 180,156 | 1,080,764 | 540,626 | 540,138 | 881,305 | 440,076 | 441,229 | 199,459 | 100,550 | 98,909 |
| 50 - 54 | 141,558 | 879,792 | 439,039 | 440,753 | 683,742 | 342,048 | 341,694 | 196,050 | 96,991 | 99,059 |
| 55 - 59 | 94,791 | 603,169 | 299,663 | 303,506 | 449,826 | 224,820 | 225,006 | 153,343 | 74,843 | 78,500 |
| 60 - 64 | 88,456 | 573,756 | 284,473 | 289,283 | 393,365 | 196,162 | 197,203 | 180,391 | 88,311 | 92,080 |
| 65+ | 178,701 | 1,117,149 | 548,200 | 568,949 | 688,192 | 334,127 | 354,065 | 428,957 | 214,073 | 214,884 |
| **Never married** | | | | | | | | | | |
| **Total** | **1,426,636** | **263,214** | **129,335** | **133,879** | **233,650** | **114,624** | **119,026** | **29,564** | **14,711** | **14,853** |
| 12 - 14 | 479,375 | 6,277 | 3,162 | 3,115 | 5,408 | 2,682 | 2,726 | 869 | 480 | 389 |
| 15 - 19 | 583,387 | 43,689 | 21,516 | 22,173 | 40,582 | 19,910 | 20,672 | 3,107 | 1,606 | 1,501 |
| 20 - 24 | 210,867 | 65,919 | 32,526 | 33,393 | 61,399 | 30,231 | 31,168 | 4,520 | 2,295 | 2,225 |
| 25 - 29 | 81,849 | 49,234 | 24,352 | 24,882 | 45,054 | 22,324 | 22,730 | 4,180 | 2,028 | 2,152 |
| 30 - 34 | 32,530 | 31,889 | 15,767 | 16,122 | 28,290 | 13,905 | 14,385 | 3,599 | 1,862 | 1,737 |
| 35 - 39 | 15,923 | 20,700 | 10,039 | 10,661 | 18,035 | 8,770 | 9,265 | 2,665 | 1,269 | 1,396 |
| 40 - 44 | 7,260 | 11,820 | 5,765 | 6,055 | 9,896 | 4,800 | 5,096 | 1,924 | 965 | 959 |
| 45 - 49 | 4,444 | 8,600 | 4,224 | 4,376 | 7,129 | 3,515 | 3,614 | 1,471 | 709 | 762 |
| 50 - 54 | 2,755 | 6,416 | 3,121 | 3,295 | 4,968 | 2,394 | 2,574 | 1,448 | 727 | 721 |
| 55 - 59 | 1,814 | 4,325 | 2,137 | 2,188 | 3,288 | 1,625 | 1,663 | 1,037 | 512 | 525 |
| 60 - 64 | 1,590 | 4,031 | 1,916 | 2,115 | 2,773 | 1,335 | 1,438 | 1,258 | 581 | 677 |
| 65+ | 4,842 | 10,314 | 4,810 | 5,504 | 6,828 | 3,133 | 3,695 | 3,486 | 1,677 | 1,809 |
| **Married** | | | | | | | | | | |
| **Total** | **1,883,324** | **7,683,212** | **3,843,430** | **3,839,782** | **6,512,650** | **3,247,922** | **3,264,728** | **1,170,562** | **595,508** | **575,054** |
| 12 - 14 | 5,571 | 1,179 | 595 | 584 | 1,083 | 534 | 549 | 96 | 61 | 35 |
| 15 - 19 | 124,519 | 108,870 | 54,471 | 54,399 | 99,355 | 49,422 | 49,933 | 9,515 | 5,049 | 4,466 |
| 20 - 24 | 338,806 | 649,991 | 324,114 | 325,877 | 594,725 | 294,871 | 299,854 | 55,266 | 29,243 | 26,023 |
| 25 - 29 | 384,492 | 1,175,681 | 586,088 | 589,593 | 1,062,514 | 526,677 | 535,837 | 113,167 | 59,411 | 53,756 |
| 30 - 34 | 296,937 | 1,268,349 | 633,456 | 634,893 | 1,124,960 | 558,879 | 566,081 | 143,389 | 74,577 | 68,812 |
| 35 - 39 | 231,830 | 1,227,750 | 615,930 | 611,820 | 1,067,549 | 533,381 | 534,168 | 160,201 | 82,549 | 77,652 |
| 40 - 44 | 150,699 | 921,482 | 462,885 | 458,597 | 777,998 | 389,391 | 388,607 | 143,484 | 73,494 | 69,990 |
| 45 - 49 | 121,278 | 786,128 | 395,110 | 391,018 | 646,940 | 324,659 | 322,281 | 139,188 | 70,451 | 68,737 |
| 50 - 54 | 85,306 | 568,120 | 285,136 | 282,984 | 447,759 | 225,518 | 222,241 | 120,361 | 59,618 | 60,743 |
| 55 - 59 | 51,598 | 350,219 | 174,855 | 175,364 | 265,865 | 133,595 | 132,270 | 84,354 | 41,260 | 43,094 |
| 60 - 64 | 40,271 | 277,942 | 138,559 | 139,383 | 196,760 | 98,745 | 98,015 | 81,182 | 39,814 | 41,368 |
| 65+ | 52,017 | 347,501 | 172,231 | 175,270 | 227,142 | 112,250 | 114,892 | 120,359 | 59,981 | 60,378 |
| **Divorced** | | | | | | | | | | |
| **Total** | **144,135** | **563,741** | **278,390** | **285,351** | **440,780** | **216,542** | **224,238** | **122,961** | **61,848** | **61,113** |
| 12 - 14 | 105 | 81 | 39 | 42 | 76 | 37 | 39 | 5 | 2 | 3 |
| 15 - 19 | 3,108 | 3,163 | 1,623 | 1,540 | 2,806 | 1,433 | 1,373 | 357 | 190 | 167 |
| 20 - 24 | 14,231 | 25,623 | 12,699 | 12,924 | 22,787 | 11,213 | 11,574 | 2,836 | 1,486 | 1,350 |
| 25 - 29 | 22,541 | 57,612 | 28,522 | 29,090 | 50,158 | 24,753 | 25,405 | 7,454 | 3,769 | 3,685 |
| 30 - 34 | 23,167 | 77,163 | 38,211 | 38,952 | 65,783 | 32,344 | 33,439 | 11,380 | 5,867 | 5,513 |
| 35 - 39 | 19,621 | 78,694 | 38,917 | 39,777 | 65,676 | 32,216 | 33,460 | 13,018 | 6,701 | 6,317 |
| 40 - 44 | 14,965 | 70,026 | 34,675 | 35,351 | 56,347 | 27,643 | 28,704 | 13,679 | 7,032 | 6,647 |
| 45 - 49 | 12,674 | 63,687 | 31,092 | 32,595 | 49,839 | 24,227 | 25,612 | 13,848 | 6,865 | 6,983 |
| 50 - 54 | 10,149 | 54,053 | 26,794 | 27,259 | 40,401 | 19,966 | 20,435 | 13,652 | 6,828 | 6,824 |
| 55 - 59 | 6,943 | 38,547 | 19,132 | 19,415 | 27,686 | 13,789 | 13,897 | 10,861 | 5,343 | 5,518 |
| 60 - 64 | 6,239 | 35,656 | 17,657 | 17,999 | 23,473 | 11,694 | 11,779 | 12,183 | 5,963 | 6,220 |
| 65+ | 10,392 | 59,436 | 29,029 | 30,407 | 35,748 | 17,227 | 18,521 | 23,688 | 11,802 | 11,886 |

## Table C3: Population of Women 12 Years and Older by Number of Children Ever Born Alive, Children Still Living, Children Dead, Sex of Child, Age Group and Marital Status, Rural/Urban, Zambia 2010

| Age Group and Marital Status | Number of Women 12 Years and Older | Children Ever Born Alive | | | Children Still Living | | | Children Dead | | |
|---|---|---|---|---|---|---|---|---|---|---|
| | | Total | Male | Female | Total | Male | Female | Total | Male | Female |
| **Separated** | | | | | | | | | | |
| **Total** | **101,716** | **352,384** | **174,134** | **178,250** | **288,302** | **141,848** | **146,454** | **64,082** | **32,286** | **31,796** |
| 12 - 14 | 139 | 85 | 45 | 40 | 73 | 39 | 34 | 12 | 6 | 6 |
| 15 - 19 | 5,245 | 5,231 | 2,616 | 2,615 | 4,756 | 2,353 | 2,403 | 475 | 263 | 212 |
| 20 - 24 | 15,803 | 27,090 | 13,207 | 13,883 | 24,571 | 11,917 | 12,654 | 2,519 | 1,290 | 1,229 |
| 25 - 29 | 20,177 | 50,140 | 24,601 | 25,539 | 44,535 | 21,733 | 22,802 | 5,605 | 2,868 | 2,737 |
| 30 - 34 | 17,199 | 57,166 | 28,430 | 28,736 | 49,649 | 24,579 | 25,070 | 7,517 | 3,851 | 3,666 |
| 35 - 39 | 13,188 | 53,861 | 26,711 | 27,150 | 45,325 | 22,331 | 22,994 | 8,536 | 4,380 | 4,156 |
| 40 - 44 | 8,617 | 41,438 | 20,493 | 20,945 | 33,835 | 16,639 | 17,196 | 7,603 | 3,854 | 3,749 |
| 45 - 49 | 6,705 | 34,563 | 17,118 | 17,445 | 27,434 | 13,553 | 13,881 | 7,129 | 3,565 | 3,564 |
| 50 - 54 | 4,683 | 25,908 | 12,880 | 13,028 | 19,643 | 9,719 | 9,924 | 6,265 | 3,161 | 3,104 |
| 55 - 59 | 3,034 | 17,326 | 8,512 | 8,814 | 12,794 | 6,291 | 6,503 | 4,532 | 2,221 | 2,311 |
| 60 - 64 | 2,702 | 16,005 | 7,920 | 8,085 | 10,812 | 5,408 | 5,404 | 5,193 | 2,512 | 2,681 |
| 65+ | 4,224 | 23,571 | 11,601 | 11,970 | 14,875 | 7,286 | 7,589 | 8,696 | 4,315 | 4,381 |
| **Widowed** | | | | | | | | | | |
| **Total** | **326,684** | **1,798,493** | **884,471** | **914,022** | **1,260,355** | **617,033** | **643,322** | **538,138** | **267,438** | **270,700** |
| 12 - 14 | 141 | 98 | 45 | 53 | 74 | 33 | 41 | 24 | 12 | 12 |
| 15 - 19 | 834 | 990 | 470 | 520 | 823 | 376 | 447 | 167 | 94 | 73 |
| 20 - 24 | 4,017 | 8,082 | 4,007 | 4,075 | 7,052 | 3,484 | 3,568 | 1,030 | 523 | 507 |
| 25 - 29 | 10,175 | 27,480 | 13,576 | 13,904 | 23,702 | 11,638 | 12,064 | 3,778 | 1,938 | 1,840 |
| 30 - 34 | 19,209 | 65,881 | 32,491 | 33,390 | 56,031 | 27,443 | 28,588 | 9,850 | 5,048 | 4,802 |
| 35 - 39 | 24,815 | 100,485 | 49,648 | 50,837 | 84,645 | 41,633 | 43,012 | 15,840 | 8,015 | 7,825 |
| 40 - 44 | 28,520 | 137,004 | 67,868 | 69,136 | 111,780 | 55,063 | 56,717 | 25,224 | 12,805 | 12,419 |
| 45 - 49 | 31,240 | 166,253 | 82,316 | 83,937 | 132,553 | 65,451 | 67,102 | 33,700 | 16,865 | 16,835 |
| 50 - 54 | 36,057 | 209,662 | 103,184 | 106,478 | 159,023 | 78,413 | 80,610 | 50,639 | 24,771 | 25,868 |
| 55 - 59 | 29,843 | 183,750 | 90,533 | 93,217 | 133,401 | 66,144 | 67,257 | 50,349 | 24,389 | 25,960 |
| 60 - 64 | 36,406 | 232,847 | 114,850 | 117,997 | 154,392 | 76,408 | 77,984 | 78,455 | 38,442 | 40,013 |
| 65+ | 105,427 | 665,961 | 325,483 | 340,478 | 396,879 | 190,947 | 205,932 | 269,082 | 134,536 | 134,546 |
| **Cohabiting** | | | | | | | | | | |
| **Total** | **109,906** | **309,770** | **154,548** | **155,222** | **264,767** | **131,469** | **133,298** | **45,003** | **23,079** | **21,924** |
| 12 - 14 | 773 | 227 | 114 | 113 | 210 | 106 | 104 | 17 | 8 | 9 |
| 15 - 19 | 17,932 | 13,868 | 6,926 | 6,942 | 12,860 | 6,386 | 6,474 | 1,008 | 540 | 468 |
| 20 - 24 | 28,876 | 45,905 | 22,820 | 23,085 | 42,074 | 20,791 | 21,283 | 3,831 | 2,029 | 1,802 |
| 25 - 29 | 22,517 | 59,514 | 29,840 | 29,674 | 53,318 | 26,596 | 26,722 | 6,196 | 3,244 | 2,952 |
| 30 - 34 | 14,034 | 53,082 | 26,468 | 26,614 | 46,374 | 22,983 | 23,391 | 6,708 | 3,485 | 3,223 |
| 35 - 39 | 9,475 | 44,314 | 22,135 | 22,179 | 37,853 | 18,765 | 19,088 | 6,461 | 3,370 | 3,091 |
| 40 - 44 | 5,270 | 29,051 | 14,444 | 14,607 | 24,053 | 11,901 | 12,152 | 4,998 | 2,543 | 2,455 |
| 45 - 49 | 3,815 | 21,533 | 10,766 | 10,767 | 17,410 | 8,671 | 8,739 | 4,123 | 2,095 | 2,028 |
| 50 - 54 | 2,608 | 15,633 | 7,924 | 7,709 | 11,948 | 6,038 | 5,910 | 3,685 | 1,886 | 1,799 |
| 55 - 59 | 1,559 | 9,002 | 4,494 | 4,508 | 6,792 | 3,376 | 3,416 | 2,210 | 1,118 | 1,092 |
| 60 - 64 | 1,248 | 7,275 | 3,571 | 3,704 | 5,155 | 2,572 | 2,583 | 2,120 | 999 | 1,121 |
| 65+ | 1,799 | 10,366 | 5,046 | 5,320 | 6,720 | 3,284 | 3,436 | 3,646 | 1,762 | 1,884 |

## Table C3: Population of Women 12 Years and Older by Number of Children Ever Born Alive, Children Still Living, Children Dead, Sex of Child, Age Group and Marital Status, Rural, Zambia 2010

| Age Group and Marital Status | Number of Women 12 Years and Older | Children Ever Born Alive | | | Children Still Living | | | Children Dead | | |
|---|---|---|---|---|---|---|---|---|---|---|
| | | Total | Male | Female | Total | Male | Female | Total | Male | Female |
| **Zambia Rural** | | | | | | | | | | |
| **Total** | **2,273,363** | **7,145,714** | **3,565,538** | **3,580,176** | **5,736,895** | **2,853,935** | **2,882,960** | **1,408,819** | **711,603** | **697,216** |
| 12 - 14 | 282,652 | 4,987 | 2,570 | 2,417 | 4,307 | 2,190 | 2,117 | 680 | 380 | 300 |
| 15 - 19 | 405,259 | 121,237 | 60,581 | 60,656 | 110,453 | 54,881 | 55,572 | 10,784 | 5,700 | 5,084 |
| 20 - 24 | 328,902 | 539,787 | 268,274 | 271,513 | 490,061 | 242,063 | 247,998 | 49,726 | 26,211 | 23,515 |
| 25 - 29 | 288,345 | 897,227 | 446,891 | 450,336 | 798,681 | 395,600 | 403,081 | 98,546 | 51,291 | 47,255 |
| 30 - 34 | 216,425 | 970,606 | 484,771 | 485,835 | 842,695 | 418,765 | 423,930 | 127,911 | 66,006 | 61,905 |
| 35 - 39 | 178,320 | 977,140 | 490,345 | 486,795 | 828,790 | 414,237 | 414,553 | 148,350 | 76,108 | 72,242 |
| 40 - 44 | 127,920 | 790,430 | 397,927 | 392,503 | 646,028 | 324,016 | 322,012 | 144,402 | 73,911 | 70,491 |
| 45 - 49 | 108,965 | 695,798 | 349,456 | 346,342 | 551,315 | 276,443 | 274,872 | 144,483 | 73,013 | 71,470 |
| 50 - 54 | 86,527 | 556,535 | 278,388 | 278,147 | 419,006 | 210,069 | 208,937 | 137,529 | 68,319 | 69,210 |
| 55 - 59 | 59,286 | 385,233 | 191,460 | 193,773 | 279,684 | 139,796 | 139,888 | 105,549 | 51,664 | 53,885 |
| 60 - 64 | 61,277 | 400,026 | 198,647 | 201,379 | 270,834 | 135,041 | 135,793 | 129,192 | 63,606 | 65,586 |
| 65+ | 129,485 | 806,708 | 396,228 | 410,480 | 495,041 | 240,834 | 254,207 | 311,667 | 155,394 | 156,273 |
| **Never married** | | | | | | | | | | |
| **Total** | **704,599** | **135,744** | **66,662** | **69,082** | **117,032** | **57,322** | **59,710** | **18,712** | **9,340** | **9,372** |
| 12 - 14 | 277,814 | 3,762 | 1,944 | 1,818 | 3,201 | 1,634 | 1,567 | 561 | 310 | 251 |
| 15 - 19 | 293,293 | 23,236 | 11,528 | 11,708 | 21,411 | 10,576 | 10,835 | 1,825 | 952 | 873 |
| 20 - 24 | 77,910 | 31,485 | 15,444 | 16,041 | 28,991 | 14,190 | 14,801 | 2,494 | 1,254 | 1,240 |
| 25 - 29 | 26,836 | 22,579 | 11,175 | 11,404 | 20,212 | 10,024 | 10,188 | 2,367 | 1,151 | 1,216 |
| 30 - 34 | 10,883 | 15,400 | 7,571 | 7,829 | 13,307 | 6,496 | 6,811 | 2,093 | 1,075 | 1,018 |
| 35 - 39 | 5,928 | 10,986 | 5,358 | 5,628 | 9,278 | 4,532 | 4,746 | 1,708 | 826 | 882 |
| 40 - 44 | 3,043 | 6,657 | 3,206 | 3,451 | 5,363 | 2,563 | 2,800 | 1,294 | 643 | 651 |
| 45 - 49 | 2,191 | 5,352 | 2,650 | 2,702 | 4,247 | 2,121 | 2,126 | 1,105 | 529 | 576 |
| 50 - 54 | 1,503 | 4,011 | 1,955 | 2,056 | 2,957 | 1,413 | 1,544 | 1,054 | 542 | 512 |
| 55 - 59 | 1,068 | 2,669 | 1,326 | 1,343 | 1,930 | 956 | 974 | 739 | 370 | 369 |
| 60 - 64 | 993 | 2,692 | 1,291 | 1,401 | 1,793 | 859 | 934 | 899 | 432 | 467 |
| 65+ | 3,137 | 6,915 | 3,214 | 3,701 | 4,342 | 1,958 | 2,384 | 2,573 | 1,256 | 1,317 |
| **Married** | | | | | | | | | | |
| **Total** | **1,166,790** | **5,112,515** | **2,560,867** | **2,551,648** | **4,246,873** | **2,120,909** | **2,125,964** | **865,642** | **439,958** | **425,684** |
| 12 - 14 | 4,048 | 905 | 472 | 433 | 824 | 420 | 404 | 81 | 52 | 29 |
| 15 - 19 | 93,884 | 82,457 | 41,270 | 41,187 | 74,892 | 37,274 | 37,618 | 7,565 | 3,996 | 3,569 |
| 20 - 24 | 214,561 | 443,057 | 220,610 | 222,447 | 402,526 | 199,142 | 203,384 | 40,531 | 21,468 | 19,063 |
| 25 - 29 | 221,288 | 759,803 | 378,644 | 381,159 | 678,508 | 336,120 | 342,388 | 81,295 | 42,524 | 38,771 |
| 30 - 34 | 167,938 | 808,641 | 404,495 | 404,146 | 705,665 | 351,215 | 354,450 | 102,976 | 53,280 | 49,696 |
| 35 - 39 | 137,516 | 802,897 | 403,544 | 399,353 | 685,132 | 343,043 | 342,089 | 117,765 | 60,501 | 57,264 |
| 40 - 44 | 93,816 | 617,604 | 311,857 | 305,747 | 509,344 | 256,354 | 252,990 | 108,260 | 55,503 | 52,757 |
| 45 - 49 | 76,553 | 521,761 | 263,088 | 258,673 | 417,494 | 210,151 | 207,343 | 104,267 | 52,937 | 51,330 |
| 50 - 54 | 54,120 | 371,136 | 186,373 | 184,763 | 283,598 | 142,992 | 140,606 | 87,538 | 43,381 | 44,157 |
| 55 - 59 | 33,686 | 232,375 | 116,159 | 116,216 | 171,859 | 86,499 | 85,360 | 60,516 | 29,660 | 30,856 |
| 60 - 64 | 29,396 | 203,839 | 101,689 | 102,150 | 142,611 | 71,513 | 71,098 | 61,228 | 30,176 | 31,052 |
| 65+ | 39,984 | 268,040 | 132,666 | 135,374 | 174,420 | 86,186 | 88,234 | 93,620 | 46,480 | 47,140 |
| **Divorced** | | | | | | | | | | |
| **Total** | **91,318** | **387,004** | **191,488** | **195,516** | **295,831** | **145,535** | **150,296** | **91,173** | **45,953** | **45,220** |
| 12 - 14 | 82 | 62 | 29 | 33 | 58 | 27 | 31 | 4 | 2 | 2 |
| 15 - 19 | 2,333 | 2,356 | 1,214 | 1,142 | 2,083 | 1,066 | 1,017 | 273 | 148 | 125 |
| 20 - 24 | 9,403 | 17,702 | 8,709 | 8,993 | 15,645 | 7,629 | 8,016 | 2,057 | 1,080 | 977 |
| 25 - 29 | 13,368 | 37,672 | 18,642 | 19,030 | 32,475 | 16,028 | 16,447 | 5,197 | 2,614 | 2,583 |
| 30 - 34 | 13,197 | 49,562 | 24,560 | 25,002 | 41,536 | 20,459 | 21,077 | 8,026 | 4,101 | 3,925 |
| 35 - 39 | 11,659 | 52,672 | 26,233 | 26,439 | 43,202 | 21,315 | 21,887 | 9,470 | 4,918 | 4,552 |
| 40 - 44 | 9,298 | 47,949 | 23,875 | 24,074 | 37,785 | 18,636 | 19,149 | 10,164 | 5,239 | 4,925 |
| 45 - 49 | 8,168 | 43,842 | 21,423 | 22,419 | 33,483 | 16,311 | 17,172 | 10,359 | 5,112 | 5,247 |
| 50 - 54 | 6,685 | 37,239 | 18,571 | 18,668 | 27,108 | 13,453 | 13,655 | 10,131 | 5,118 | 5,013 |
| 55 - 59 | 4,683 | 26,832 | 13,311 | 13,521 | 18,852 | 9,363 | 9,489 | 7,980 | 3,948 | 4,032 |
| 60 - 64 | 4,486 | 25,740 | 12,753 | 12,987 | 16,504 | 8,207 | 8,297 | 9,236 | 4,546 | 4,690 |
| 65+ | 7,956 | 45,376 | 22,168 | 23,208 | 27,100 | 13,041 | 14,059 | 18,276 | 9,127 | 9,149 |

## Table C3: Population of Women 12 Years and Older by Number of Children Ever Born Alive, Children Still Living, Children Dead, Sex of Child, Age Group and Marital Status, Rural, Zambia 2010

| Age Group and Marital Status | Number of Women 12 Years and Older | Children Ever Born Alive | | | Children Still Living | | | Children Dead | | |
|---|---|---|---|---|---|---|---|---|---|---|
| | | Total | Male | Female | Total | Male | Female | Total | Male | Female |
| **Separated** | | | | | | | | | | |
| **Total** | **59,582** | **226,558** | **112,267** | **114,291** | **180,625** | **89,120** | **91,505** | **45,933** | **23,147** | **22,786** |
| 12 - 14 | 90 | 47 | 24 | 23 | 39 | 21 | 18 | 8 | 3 | 5 |
| 15 - 19 | 3,387 | 3,289 | 1,650 | 1,639 | 2,979 | 1,473 | 1,506 | 310 | 177 | 133 |
| 20 - 24 | 9,264 | 16,593 | 8,085 | 8,508 | 14,964 | 7,248 | 7,716 | 1,629 | 837 | 792 |
| 25 - 29 | 10,771 | 29,562 | 14,574 | 14,988 | 25,848 | 12,654 | 13,194 | 3,714 | 1,920 | 1,794 |
| 30 - 34 | 8,961 | 33,992 | 16,991 | 17,001 | 29,020 | 14,442 | 14,578 | 4,972 | 2,549 | 2,423 |
| 35 - 39 | 7,322 | 33,774 | 16,887 | 16,887 | 27,759 | 13,829 | 13,930 | 6,015 | 3,058 | 2,957 |
| 40 - 44 | 5,238 | 27,595 | 13,698 | 13,897 | 22,018 | 10,867 | 11,151 | 5,577 | 2,831 | 2,746 |
| 45 - 49 | 4,201 | 22,757 | 11,289 | 11,468 | 17,570 | 8,702 | 8,868 | 5,187 | 2,587 | 2,600 |
| 50 - 54 | 3,081 | 17,282 | 8,543 | 8,739 | 12,719 | 6,225 | 6,494 | 4,563 | 2,318 | 2,245 |
| 55 - 59 | 2,017 | 11,780 | 5,802 | 5,978 | 8,507 | 4,188 | 4,319 | 3,273 | 1,614 | 1,659 |
| 60 - 64 | 1,962 | 11,595 | 5,695 | 5,900 | 7,705 | 3,821 | 3,884 | 3,890 | 1,874 | 2,016 |
| 65+ | 3,288 | 18,292 | 9,029 | 9,263 | 11,497 | 5,650 | 5,847 | 6,795 | 3,379 | 3,416 |
| **Widowed** | | | | | | | | | | |
| **Total** | **188,966** | **1,096,735** | **540,862** | **555,873** | **739,563** | **363,061** | **376,502** | **357,172** | **177,801** | **179,371** |
| 12 - 14 | 69 | 43 | 21 | 22 | 30 | 14 | 16 | 13 | 7 | 6 |
| 15 - 19 | 545 | 631 | 314 | 317 | 503 | 241 | 262 | 128 | 73 | 55 |
| 20 - 24 | 2,167 | 4,608 | 2,280 | 2,328 | 3,975 | 1,958 | 2,017 | 633 | 322 | 311 |
| 25 - 29 | 4,747 | 14,581 | 7,211 | 7,370 | 12,354 | 6,085 | 6,269 | 2,227 | 1,126 | 1,101 |
| 30 - 34 | 8,353 | 33,133 | 16,335 | 16,798 | 27,488 | 13,482 | 14,006 | 5,645 | 2,853 | 2,792 |
| 35 - 39 | 10,656 | 50,112 | 24,941 | 25,171 | 41,068 | 20,410 | 20,658 | 9,044 | 4,531 | 4,513 |
| 40 - 44 | 13,357 | 71,968 | 35,981 | 35,987 | 56,428 | 28,099 | 28,329 | 15,540 | 7,882 | 7,658 |
| 45 - 49 | 15,500 | 88,194 | 44,058 | 44,136 | 67,511 | 33,683 | 33,828 | 20,683 | 10,375 | 10,308 |
| 50 - 54 | 19,474 | 116,613 | 57,773 | 58,840 | 84,940 | 42,134 | 42,806 | 31,673 | 15,639 | 16,034 |
| 55 - 59 | 16,818 | 105,651 | 51,931 | 53,720 | 74,172 | 36,634 | 37,538 | 31,479 | 15,297 | 16,182 |
| 60 - 64 | 23,549 | 150,993 | 74,720 | 76,273 | 98,575 | 48,856 | 49,719 | 52,418 | 25,864 | 26,554 |
| 65+ | 73,731 | 460,208 | 225,297 | 234,911 | 272,519 | 131,465 | 141,054 | 187,689 | 93,832 | 93,857 |
| **Cohabiting** | | | | | | | | | | |
| **Total** | **62,108** | **187,158** | **93,392** | **93,766** | **156,971** | **77,988** | **78,983** | **30,187** | **15,404** | **14,783** |
| 12 - 14 | 549 | 168 | 80 | 88 | 155 | 74 | 81 | 13 | 6 | 7 |
| 15 - 19 | 11,817 | 9,268 | 4,605 | 4,663 | 8,585 | 4,251 | 4,334 | 683 | 354 | 329 |
| 20 - 24 | 15,597 | 26,342 | 13,146 | 13,196 | 23,960 | 11,896 | 12,064 | 2,382 | 1,250 | 1,132 |
| 25 - 29 | 11,335 | 33,030 | 16,645 | 16,385 | 29,284 | 14,689 | 14,595 | 3,746 | 1,956 | 1,790 |
| 30 - 34 | 7,093 | 29,878 | 14,819 | 15,059 | 25,679 | 12,671 | 13,008 | 4,199 | 2,148 | 2,051 |
| 35 - 39 | 5,239 | 26,699 | 13,382 | 13,317 | 22,351 | 11,108 | 11,243 | 4,348 | 2,274 | 2,074 |
| 40 - 44 | 3,168 | 18,657 | 9,310 | 9,347 | 15,090 | 7,497 | 7,593 | 3,567 | 1,813 | 1,754 |
| 45 - 49 | 2,352 | 13,892 | 6,948 | 6,944 | 11,010 | 5,475 | 5,535 | 2,882 | 1,473 | 1,409 |
| 50 - 54 | 1,664 | 10,254 | 5,173 | 5,081 | 7,684 | 3,852 | 3,832 | 2,570 | 1,321 | 1,249 |
| 55 - 59 | 1,014 | 5,926 | 2,931 | 2,995 | 4,364 | 2,156 | 2,208 | 1,562 | 775 | 787 |
| 60 - 64 | 891 | 5,167 | 2,499 | 2,668 | 3,646 | 1,785 | 1,861 | 1,521 | 714 | 807 |
| 65+ | 1,389 | 7,877 | 3,854 | 4,023 | 5,163 | 2,534 | 2,629 | 2,714 | 1,320 | 1,394 |

## Table C3: Population of Women 12 Years and Older by Number of Children Ever Born Alive, Children Still Living, Children Dead, Sex of Child, Age Group and Marital Status, Urban, Zambia 2010

| Age Group and Marital Status | Number of Women 12 Years and Older | Children Ever Born Alive | | | Children Still Living | | | Children Dead | | |
|---|---|---|---|---|---|---|---|---|---|---|
| | | Total | Male | Female | Total | Male | Female | Total | Male | Female |
| **Zambia Urban** | | | | | | | | | | |
| **Total** | **1,719,038** | **3,825,100** | **1,898,770** | **1,926,330** | **3,263,609** | **1,615,503** | **1,648,106** | **561,491** | **283,267** | **278,224** |
| 12 - 14 | 203,452 | 2,960 | 1,430 | 1,530 | 2,617 | 1,241 | 1,376 | 343 | 189 | 154 |
| 15 - 19 | 329,766 | 54,574 | 27,041 | 27,533 | 50,729 | 24,999 | 25,730 | 3,845 | 2,042 | 1,803 |
| 20 - 24 | 283,698 | 282,823 | 141,099 | 141,724 | 262,547 | 130,444 | 132,103 | 20,276 | 10,655 | 9,621 |
| 25 - 29 | 253,406 | 522,434 | 260,088 | 262,346 | 480,600 | 238,121 | 242,479 | 41,834 | 21,967 | 19,867 |
| 30 - 34 | 186,651 | 582,924 | 290,052 | 292,872 | 528,392 | 261,368 | 267,024 | 54,532 | 28,684 | 25,848 |
| 35 - 39 | 136,532 | 548,664 | 273,035 | 275,629 | 490,293 | 242,859 | 247,434 | 58,371 | 30,176 | 28,195 |
| 40 - 44 | 87,411 | 420,391 | 208,203 | 212,188 | 367,881 | 181,421 | 186,460 | 52,510 | 26,782 | 25,728 |
| 45 - 49 | 71,191 | 384,966 | 191,170 | 193,796 | 329,990 | 163,633 | 166,357 | 54,976 | 27,537 | 27,439 |
| 50 - 54 | 55,031 | 323,257 | 160,651 | 162,606 | 264,736 | 131,979 | 132,757 | 58,521 | 28,672 | 29,849 |
| 55 - 59 | 35,505 | 217,936 | 108,203 | 109,733 | 170,142 | 85,024 | 85,118 | 47,794 | 23,179 | 24,615 |
| 60 - 64 | 27,179 | 173,730 | 85,826 | 87,904 | 122,531 | 61,121 | 61,410 | 51,199 | 24,705 | 26,494 |
| 65+ | 49,216 | 310,441 | 151,972 | 158,469 | 193,151 | 93,293 | 99,858 | 117,290 | 58,679 | 58,611 |
| **Never married** | | | | | | | | | | |
| **Total** | **722,037** | **127,470** | **62,673** | **64,797** | **116,618** | **57,302** | **59,316** | **10,852** | **5,371** | **5,481** |
| 12 - 14 | 201,561 | 2,515 | 1,218 | 1,297 | 2,207 | 1,048 | 1,159 | 308 | 170 | 138 |
| 15 - 19 | 290,094 | 20,453 | 9,988 | 10,465 | 19,171 | 9,334 | 9,837 | 1,282 | 654 | 628 |
| 20 - 24 | 132,957 | 34,434 | 17,082 | 17,352 | 32,408 | 16,041 | 16,367 | 2,026 | 1,041 | 985 |
| 25 - 29 | 55,013 | 26,655 | 13,177 | 13,478 | 24,842 | 12,300 | 12,542 | 1,813 | 877 | 936 |
| 30 - 34 | 21,647 | 16,489 | 8,196 | 8,293 | 14,983 | 7,409 | 7,574 | 1,506 | 787 | 719 |
| 35 - 39 | 9,995 | 9,714 | 4,681 | 5,033 | 8,757 | 4,238 | 4,519 | 957 | 443 | 514 |
| 40 - 44 | 4,217 | 5,163 | 2,559 | 2,604 | 4,533 | 2,237 | 2,296 | 630 | 322 | 308 |
| 45 - 49 | 2,253 | 3,248 | 1,574 | 1,674 | 2,882 | 1,394 | 1,488 | 366 | 180 | 186 |
| 50 - 54 | 1,252 | 2,405 | 1,166 | 1,239 | 2,011 | 981 | 1,030 | 394 | 185 | 209 |
| 55 - 59 | 746 | 1,656 | 811 | 845 | 1,358 | 669 | 689 | 298 | 142 | 156 |
| 60 - 64 | 597 | 1,339 | 625 | 714 | 980 | 476 | 504 | 359 | 149 | 210 |
| 65+ | 1,705 | 3,399 | 1,596 | 1,803 | 2,486 | 1,175 | 1,311 | 913 | 421 | 492 |
| **Married** | | | | | | | | | | |
| **Total** | **716,534** | **2,570,697** | **1,282,563** | **1,288,134** | **2,265,777** | **1,127,013** | **1,138,764** | **304,920** | **155,550** | **149,370** |
| 12 - 14 | 1,523 | 274 | 123 | 151 | 259 | 114 | 145 | 15 | 9 | 6 |
| 15 - 19 | 30,635 | 26,413 | 13,201 | 13,212 | 24,463 | 12,148 | 12,315 | 1,950 | 1,053 | 897 |
| 20 - 24 | 124,245 | 206,934 | 103,504 | 103,430 | 192,199 | 95,729 | 96,470 | 14,735 | 7,775 | 6,960 |
| 25 - 29 | 163,204 | 415,878 | 207,444 | 208,434 | 384,006 | 190,557 | 193,449 | 31,872 | 16,887 | 14,985 |
| 30 - 34 | 128,999 | 459,708 | 228,961 | 230,747 | 419,295 | 207,664 | 211,631 | 40,413 | 21,297 | 19,116 |
| 35 - 39 | 94,314 | 424,853 | 212,386 | 212,467 | 382,417 | 190,338 | 192,079 | 42,436 | 22,048 | 20,388 |
| 40 - 44 | 56,883 | 303,878 | 151,028 | 152,850 | 268,654 | 133,037 | 135,617 | 35,224 | 17,991 | 17,233 |
| 45 - 49 | 44,725 | 264,367 | 132,022 | 132,345 | 229,446 | 114,508 | 114,938 | 34,921 | 17,514 | 17,407 |
| 50 - 54 | 31,186 | 196,984 | 98,763 | 98,221 | 164,161 | 82,526 | 81,635 | 32,823 | 16,237 | 16,586 |
| 55 - 59 | 17,912 | 117,844 | 58,696 | 59,148 | 94,006 | 47,096 | 46,910 | 23,838 | 11,600 | 12,238 |
| 60 - 64 | 10,875 | 74,103 | 36,870 | 37,233 | 54,149 | 27,232 | 26,917 | 19,954 | 9,638 | 10,316 |
| 65+ | 12,033 | 79,461 | 39,565 | 39,896 | 52,722 | 26,064 | 26,658 | 26,739 | 13,501 | 13,238 |
| **Divorced** | | | | | | | | | | |
| **Total** | **52,817** | **176,737** | **86,902** | **89,835** | **144,949** | **71,007** | **73,942** | **31,788** | **15,895** | **15,893** |
| 12 - 14 | 23 | 19 | 10 | 9 | 18 | 10 | 8 | 1 | - | 1 |
| 15 - 19 | 775 | 807 | 409 | 398 | 723 | 367 | 356 | 84 | 42 | 42 |
| 20 - 24 | 4,828 | 7,921 | 3,990 | 3,931 | 7,142 | 3,584 | 3,558 | 779 | 406 | 373 |
| 25 - 29 | 9,173 | 19,940 | 9,880 | 10,060 | 17,683 | 8,725 | 8,958 | 2,257 | 1,155 | 1,102 |
| 30 - 34 | 9,970 | 27,601 | 13,651 | 13,950 | 24,247 | 11,885 | 12,362 | 3,354 | 1,766 | 1,588 |
| 35 - 39 | 7,962 | 26,022 | 12,684 | 13,338 | 22,474 | 10,901 | 11,573 | 3,548 | 1,783 | 1,765 |
| 40 - 44 | 5,667 | 22,077 | 10,800 | 11,277 | 18,562 | 9,007 | 9,555 | 3,515 | 1,793 | 1,722 |
| 45 - 49 | 4,506 | 19,845 | 9,669 | 10,176 | 16,356 | 7,916 | 8,440 | 3,489 | 1,753 | 1,736 |
| 50 - 54 | 3,464 | 16,814 | 8,223 | 8,591 | 13,293 | 6,513 | 6,780 | 3,521 | 1,710 | 1,811 |
| 55 - 59 | 2,260 | 11,715 | 5,821 | 5,894 | 8,834 | 4,426 | 4,408 | 2,881 | 1,395 | 1,486 |
| 60 - 64 | 1,753 | 9,916 | 4,904 | 5,012 | 6,969 | 3,487 | 3,482 | 2,947 | 1,417 | 1,530 |
| 65+ | 2,436 | 14,060 | 6,861 | 7,199 | 8,648 | 4,186 | 4,462 | 5,412 | 2,675 | 2,737 |

## Table C3: Population of Women 12 Years and Older by Number of Children Ever Born Alive, Children Still Living, Children Dead, Sex of Child, Age Group and Marital Status, Urban, Zambia 2010

| Age Group and Marital Status | Number of Women 12 Years and Older | Children Ever Born Alive | | | Children Still Living | | | Children Dead | | |
|---|---|---|---|---|---|---|---|---|---|---|
| | | Total | Male | Female | Total | Male | Female | Total | Male | Female |
| **Separated** | | | | | | | | | | |
| **Total** | **42,134** | **125,826** | **61,867** | **63,959** | **107,677** | **52,728** | **54,949** | **18,149** | **9,139** | **9,010** |
| 12 - 14 | 49 | 38 | 21 | 17 | 34 | 18 | 16 | 4 | 3 | 1 |
| 15 - 19 | 1,858 | 1,942 | 966 | 976 | 1,777 | 880 | 897 | 165 | 86 | 79 |
| 20 - 24 | 6,539 | 10,497 | 5,122 | 5,375 | 9,607 | 4,669 | 4,938 | 890 | 453 | 437 |
| 25 - 29 | 9,406 | 20,578 | 10,027 | 10,551 | 18,687 | 9,079 | 9,608 | 1,891 | 948 | 943 |
| 30 - 34 | 8,238 | 23,174 | 11,439 | 11,735 | 20,629 | 10,137 | 10,492 | 2,545 | 1,302 | 1,243 |
| 35 - 39 | 5,866 | 20,087 | 9,824 | 10,263 | 17,566 | 8,502 | 9,064 | 2,521 | 1,322 | 1,199 |
| 40 - 44 | 3,379 | 13,843 | 6,795 | 7,048 | 11,817 | 5,772 | 6,045 | 2,026 | 1,023 | 1,003 |
| 45 - 49 | 2,504 | 11,806 | 5,829 | 5,977 | 9,864 | 4,851 | 5,013 | 1,942 | 978 | 964 |
| 50 - 54 | 1,602 | 8,626 | 4,337 | 4,289 | 6,924 | 3,494 | 3,430 | 1,702 | 843 | 859 |
| 55 - 59 | 1,017 | 5,546 | 2,710 | 2,836 | 4,287 | 2,103 | 2,184 | 1,259 | 607 | 652 |
| 60 - 64 | 740 | 4,410 | 2,225 | 2,185 | 3,107 | 1,587 | 1,520 | 1,303 | 638 | 665 |
| 65+ | 936 | 5,279 | 2,572 | 2,707 | 3,378 | 1,636 | 1,742 | 1,901 | 936 | 965 |
| **Widowed** | | | | | | | | | | |
| **Total** | **137,718** | **701,758** | **343,609** | **358,149** | **520,792** | **253,972** | **266,820** | **180,966** | **89,637** | **91,329** |
| 12 - 14 | 72 | 55 | 24 | 31 | 44 | 19 | 25 | 11 | 5 | 6 |
| 15 - 19 | 289 | 359 | 156 | 203 | 320 | 135 | 185 | 39 | 21 | 18 |
| 20 - 24 | 1,850 | 3,474 | 1,727 | 1,747 | 3,077 | 1,526 | 1,551 | 397 | 201 | 196 |
| 25 - 29 | 5,428 | 12,899 | 6,365 | 6,534 | 11,348 | 5,553 | 5,795 | 1,551 | 812 | 739 |
| 30 - 34 | 10,856 | 32,748 | 16,156 | 16,592 | 28,543 | 13,961 | 14,582 | 4,205 | 2,195 | 2,010 |
| 35 - 39 | 14,159 | 50,373 | 24,707 | 25,666 | 43,577 | 21,223 | 22,354 | 6,796 | 3,484 | 3,312 |
| 40 - 44 | 15,163 | 65,036 | 31,887 | 33,149 | 55,352 | 26,964 | 28,388 | 9,684 | 4,923 | 4,761 |
| 45 - 49 | 15,740 | 78,059 | 38,258 | 39,801 | 65,042 | 31,768 | 33,274 | 13,017 | 6,490 | 6,527 |
| 50 - 54 | 16,583 | 93,049 | 45,411 | 47,638 | 74,083 | 36,279 | 37,804 | 18,966 | 9,132 | 9,834 |
| 55 - 59 | 13,025 | 78,099 | 38,602 | 39,497 | 59,229 | 29,510 | 29,719 | 18,870 | 9,092 | 9,778 |
| 60 - 64 | 12,857 | 81,854 | 40,130 | 41,724 | 55,817 | 27,552 | 28,265 | 26,037 | 12,578 | 13,459 |
| 65+ | 31,696 | 205,753 | 100,186 | 105,567 | 124,360 | 59,482 | 64,878 | 81,393 | 40,704 | 40,689 |
| **Cohabiting** | | | | | | | | | | |
| **Total** | **47,798** | **122,612** | **61,156** | **61,456** | **107,796** | **53,481** | **54,315** | **14,816** | **7,675** | **7,141** |
| 12 - 14 | 224 | 59 | 34 | 25 | 55 | 32 | 23 | 4 | 2 | 2 |
| 15 - 19 | 6,115 | 4,600 | 2,321 | 2,279 | 4,275 | 2,135 | 2,140 | 325 | 186 | 139 |
| 20 - 24 | 13,279 | 19,563 | 9,674 | 9,889 | 18,114 | 8,895 | 9,219 | 1,449 | 779 | 670 |
| 25 - 29 | 11,182 | 26,484 | 13,195 | 13,289 | 24,034 | 11,907 | 12,127 | 2,450 | 1,288 | 1,162 |
| 30 - 34 | 6,941 | 23,204 | 11,649 | 11,555 | 20,695 | 10,312 | 10,383 | 2,509 | 1,337 | 1,172 |
| 35 - 39 | 4,236 | 17,615 | 8,753 | 8,862 | 15,502 | 7,657 | 7,845 | 2,113 | 1,096 | 1,017 |
| 40 - 44 | 2,102 | 10,394 | 5,134 | 5,260 | 8,963 | 4,404 | 4,559 | 1,431 | 730 | 701 |
| 45 - 49 | 1,463 | 7,641 | 3,818 | 3,823 | 6,400 | 3,196 | 3,204 | 1,241 | 622 | 619 |
| 50 - 54 | 944 | 5,379 | 2,751 | 2,628 | 4,264 | 2,186 | 2,078 | 1,115 | 565 | 550 |
| 55 - 59 | 545 | 3,076 | 1,563 | 1,513 | 2,428 | 1,220 | 1,208 | 648 | 343 | 305 |
| 60 - 64 | 357 | 2,108 | 1,072 | 1,036 | 1,509 | 787 | 722 | 599 | 285 | 314 |
| 65+ | 410 | 2,489 | 1,192 | 1,297 | 1,557 | 750 | 807 | 932 | 442 | 490 |

## Table C3i: Population of Women 12-49 Years by Number of Children Born Alive 12 Months Prior to the Census, Children Still Living, Children Dead, Sex of Child, Age Group and Marital Status, Rural/Urban, Zambia 2010

| Age Group and Marital Status | Number of Women 12 Years and Older | Children Born Alive 12 Months Prior to Census | | | Children Still Living | | | Children Dead | | |
|---|---|---|---|---|---|---|---|---|---|---|
| | | Total | Male | Female | Total | Male | Female | Total | Male | Female |
| **Zambia Total** | | | | | | | | | | |
| **Total** | **3,488,895** | **442,998** | **224,756** | **218,242** | **423,116** | **214,441** | **208,675** | **19,882** | **10,315** | **9,567** |
| 12 - 14 | 486,104 | 921 | 470 | 451 | 870 | 447 | 423 | 51 | 23 | 28 |
| 15 - 19 | 735,025 | 58,999 | 29,701 | 29,298 | 56,523 | 28,406 | 28,117 | 2,476 | 1,295 | 1,181 |
| 20 - 24 | 612,600 | 128,270 | 65,048 | 63,222 | 123,381 | 62,419 | 60,962 | 4,889 | 2,629 | 2,260 |
| 25 - 29 | 541,751 | 114,701 | 58,366 | 56,335 | 109,784 | 55,861 | 53,923 | 4,917 | 2,505 | 2,412 |
| 30 - 34 | 403,076 | 74,066 | 37,666 | 36,400 | 70,501 | 35,842 | 34,659 | 3,565 | 1,824 | 1,741 |
| 35 - 39 | 314,852 | 45,452 | 23,072 | 22,380 | 42,958 | 21,789 | 21,169 | 2,494 | 1,283 | 1,211 |
| 40 - 44 | 215,331 | 16,150 | 8,181 | 7,969 | 15,087 | 7,635 | 7,452 | 1,063 | 546 | 517 |
| 45 - 49 | 180,156 | 4,439 | 2,252 | 2,187 | 4,012 | 2,042 | 1,970 | 427 | 210 | 217 |
| **Never married** | | | | | | | | | | |
| **Total** | **1,415,635** | **31,280** | **15,760** | **15,520** | **30,177** | **15,209** | **14,968** | **1,103** | **551** | **552** |
| 12 - 14 | 479,375 | 434 | 217 | 217 | 414 | 211 | 203 | 20 | 6 | 14 |
| 15 - 19 | 583,387 | 13,535 | 6,885 | 6,650 | 13,102 | 6,680 | 6,422 | 433 | 205 | 228 |
| 20 - 24 | 210,867 | 10,928 | 5,458 | 5,470 | 10,580 | 5,268 | 5,312 | 348 | 190 | 158 |
| 25 - 29 | 81,849 | 3,974 | 1,990 | 1,984 | 3,788 | 1,898 | 1,890 | 186 | 92 | 94 |
| 30 - 34 | 32,530 | 1,564 | 792 | 772 | 1,487 | 756 | 731 | 77 | 36 | 41 |
| 35 - 39 | 15,923 | 640 | 314 | 326 | 612 | 297 | 315 | 28 | 17 | 11 |
| 40 - 44 | 7,260 | 149 | 70 | 79 | 142 | 66 | 76 | 7 | 4 | 3 |
| 45 - 49 | 4,444 | 56 | 34 | 22 | 52 | 33 | 19 | 4 | 1 | 3 |
| **Married** | | | | | | | | | | |
| **Total** | **1,654,132** | **357,803** | **181,747** | **176,056** | **341,915** | **173,515** | **168,400** | **15,888** | **8,232** | **7,656** |
| 12 - 14 | 5,571 | 338 | 175 | 163 | 315 | 162 | 153 | 23 | 13 | 10 |
| 15 - 19 | 124,519 | 37,561 | 18,875 | 18,686 | 35,844 | 17,968 | 17,876 | 1,717 | 907 | 810 |
| 20 - 24 | 338,806 | 102,719 | 52,197 | 50,522 | 98,818 | 50,118 | 48,700 | 3,901 | 2,079 | 1,822 |
| 25 - 29 | 384,492 | 97,830 | 49,892 | 47,938 | 93,791 | 47,822 | 45,969 | 4,039 | 2,070 | 1,969 |
| 30 - 34 | 296,937 | 63,223 | 32,167 | 31,056 | 60,294 | 30,668 | 29,626 | 2,929 | 1,499 | 1,430 |
| 35 - 39 | 231,830 | 38,876 | 19,693 | 19,183 | 36,789 | 18,633 | 18,156 | 2,087 | 1,060 | 1,027 |
| 40 - 44 | 150,699 | 13,615 | 6,897 | 6,718 | 12,753 | 6,454 | 6,299 | 862 | 443 | 419 |
| 45 - 49 | 121,278 | 3,641 | 1,851 | 1,790 | 3,311 | 1,690 | 1,621 | 330 | 161 | 169 |
| **Divorced** | | | | | | | | | | |
| **Total** | **110,412** | **11,456** | **5,832** | **5,624** | **10,703** | **5,440** | **5,263** | **753** | **392** | **361** |
| 12 - 14 | 105 | 12 | 7 | 5 | 10 | 6 | 4 | 2 | 1 | 1 |
| 15 - 19 | 3,108 | 666 | 349 | 317 | 628 | 328 | 300 | 38 | 21 | 17 |
| 20 - 24 | 14,231 | 2,578 | 1,285 | 1,293 | 2,451 | 1,219 | 1,232 | 127 | 66 | 61 |
| 25 - 29 | 22,541 | 3,116 | 1,618 | 1,498 | 2,905 | 1,506 | 1,399 | 211 | 112 | 99 |
| 30 - 34 | 23,167 | 2,609 | 1,317 | 1,292 | 2,427 | 1,225 | 1,202 | 182 | 92 | 90 |
| 35 - 39 | 19,621 | 1,650 | 847 | 803 | 1,538 | 784 | 754 | 112 | 63 | 49 |
| 40 - 44 | 14,965 | 637 | 308 | 329 | 586 | 287 | 299 | 51 | 21 | 30 |
| 45 - 49 | 12,674 | 188 | 101 | 87 | 158 | 85 | 73 | 30 | 16 | 14 |

## Table C3i: Population of Women 12-49 Years by Number of Children Born Alive 12 Months Prior to the Census, Children Still Living, Children Dead, Sex of Child, Age Group and Marital Status, Rural/Urban, Zambia 2010

| Age Group and Marital Status | Number of Women 12 Years and Older | Children Born Alive 12 Months Prior to Census | | | Children Still Living | | | Children Dead | | |
|---|---|---|---|---|---|---|---|---|---|---|
| | | Total | Male | Female | Total | Male | Female | Total | Male | Female |
| **Separated** | | | | | | | | | | |
| **Total** | **87,073** | **12,907** | **6,466** | **6,441** | **12,259** | **6,131** | **6,128** | **648** | **335** | **313** |
| 12 - 14 | 139 | 23 | 14 | 9 | 23 | 14 | 9 | - | - | - |
| 15 - 19 | 5,245 | 1,567 | 770 | 797 | 1,489 | 726 | 763 | 78 | 44 | 34 |
| 20 - 24 | 15,803 | 3,493 | 1,750 | 1,743 | 3,332 | 1,666 | 1,666 | 161 | 84 | 77 |
| 25 - 29 | 20,177 | 3,346 | 1,660 | 1,686 | 3,176 | 1,577 | 1,599 | 170 | 83 | 87 |
| 30 - 34 | 17,199 | 2,351 | 1,190 | 1,161 | 2,234 | 1,136 | 1,098 | 117 | 54 | 63 |
| 35 - 39 | 13,188 | 1,447 | 740 | 707 | 1,366 | 692 | 674 | 81 | 48 | 33 |
| 40 - 44 | 8,617 | 542 | 278 | 264 | 513 | 261 | 252 | 29 | 17 | 12 |
| 45 - 49 | 6,705 | 138 | 64 | 74 | 126 | 59 | 67 | 12 | 5 | 7 |
| **Widowed** | | | | | | | | | | |
| **Total** | **118,951** | **6,096** | **3,118** | **2,978** | **5,634** | **2,877** | **2,757** | **462** | **241** | **221** |
| 12 - 14 | 141 | 3 | 1 | 2 | 3 | 1 | 2 | - | - | - |
| 15 - 19 | 834 | 142 | 80 | 62 | 136 | 76 | 60 | 6 | 4 | 2 |
| 20 - 24 | 4,017 | 675 | 358 | 317 | 638 | 338 | 300 | 37 | 20 | 17 |
| 25 - 29 | 10,175 | 1,227 | 618 | 609 | 1,142 | 572 | 570 | 85 | 46 | 39 |
| 30 - 34 | 19,209 | 1,622 | 820 | 802 | 1,507 | 760 | 747 | 115 | 60 | 55 |
| 35 - 39 | 24,815 | 1,385 | 727 | 658 | 1,281 | 674 | 607 | 104 | 53 | 51 |
| 40 - 44 | 28,520 | 737 | 376 | 361 | 663 | 337 | 326 | 74 | 39 | 35 |
| 45 - 49 | 31,240 | 305 | 138 | 167 | 264 | 119 | 145 | 41 | 19 | 22 |
| **Cohabiting** | | | | | | | | | | |
| **Total** | **102,692** | **23,456** | **11,833** | **11,623** | **22,428** | **11,269** | **11,159** | **1,028** | **564** | **464** |
| 12 - 14 | 773 | 111 | 56 | 55 | 105 | 53 | 52 | 6 | 3 | 3 |
| 15 - 19 | 17,932 | 5,528 | 2,742 | 2,786 | 5,324 | 2,628 | 2,696 | 204 | 114 | 90 |
| 20 - 24 | 28,876 | 7,877 | 4,000 | 3,877 | 7,562 | 3,810 | 3,752 | 315 | 190 | 125 |
| 25 - 29 | 22,517 | 5,208 | 2,588 | 2,620 | 4,982 | 2,486 | 2,496 | 226 | 102 | 124 |
| 30 - 34 | 14,034 | 2,697 | 1,380 | 1,317 | 2,552 | 1,297 | 1,255 | 145 | 83 | 62 |
| 35 - 39 | 9,475 | 1,454 | 751 | 703 | 1,372 | 709 | 663 | 82 | 42 | 40 |
| 40 - 44 | 5,270 | 470 | 252 | 218 | 430 | 230 | 200 | 40 | 22 | 18 |
| 45 - 49 | 3,815 | 111 | 64 | 47 | 101 | 56 | 45 | 10 | 8 | 2 |

## Table C3i: Population of Women 12-49 Years by Number of Children Born Alive 12 Months Prior to the Census, Children Still Living, Children Dead, Sex of Child, Age Group and Marital Status, Rural, Zambia 2010

| Age Group and Marital Status | Number of Women 12 Years and Older | Children Born Alive 12 Months Prior to Census | | | Children Still Living | | | Children Dead | | |
|---|---|---|---|---|---|---|---|---|---|---|
| | | Total | Male | Female | Total | Male | Female | Total | Male | Female |
| **Zambia Rural** | | | | | | | | | | |
| **Total** | **1,936,788** | **294,803** | **149,436** | **145,367** | **281,341** | **142,357** | **138,984** | **13,462** | **7,079** | **6,383** |
| 12 - 14 | 282,652 | 672 | 342 | 330 | 630 | 323 | 307 | 42 | 19 | 23 |
| 15 - 19 | 405,259 | 41,899 | 21,054 | 20,845 | 40,055 | 20,075 | 19,980 | 1,844 | 979 | 865 |
| 20 - 24 | 328,902 | 84,736 | 42,823 | 41,913 | 81,446 | 41,041 | 40,405 | 3,290 | 1,782 | 1,508 |
| 25 - 29 | 288,345 | 72,639 | 37,041 | 35,598 | 69,506 | 35,413 | 34,093 | 3,133 | 1,628 | 1,505 |
| 30 - 34 | 216,425 | 47,489 | 24,077 | 23,412 | 45,182 | 22,876 | 22,306 | 2,307 | 1,201 | 1,106 |
| 35 - 39 | 178,320 | 31,641 | 16,135 | 15,506 | 29,939 | 15,251 | 14,688 | 1,702 | 884 | 818 |
| 40 - 44 | 127,920 | 12,286 | 6,213 | 6,073 | 11,479 | 5,802 | 5,677 | 807 | 411 | 396 |
| 45 - 49 | 108,965 | 3,441 | 1,751 | 1,690 | 3,104 | 1,576 | 1,528 | 337 | 175 | 162 |
| **Never married** | | | | | | | | | | |
| **Total** | **697,898** | **16,892** | **8,494** | **8,398** | **16,283** | **8,178** | **8,105** | **609** | **316** | **293** |
| 12 - 14 | 277,814 | 273 | 140 | 133 | 260 | 136 | 124 | 13 | 4 | 9 |
| 15 - 19 | 293,293 | 7,723 | 3,906 | 3,817 | 7,456 | 3,774 | 3,682 | 267 | 132 | 135 |
| 20 - 24 | 77,910 | 5,558 | 2,772 | 2,786 | 5,362 | 2,665 | 2,697 | 196 | 107 | 89 |
| 25 - 29 | 26,836 | 2,017 | 1,030 | 987 | 1,935 | 983 | 952 | 82 | 47 | 35 |
| 30 - 34 | 10,883 | 816 | 409 | 407 | 789 | 396 | 393 | 27 | 13 | 14 |
| 35 - 39 | 5,928 | 368 | 171 | 197 | 351 | 162 | 189 | 17 | 9 | 8 |
| 40 - 44 | 3,043 | 95 | 43 | 52 | 91 | 40 | 51 | 4 | 3 | 1 |
| 45 - 49 | 2,191 | 42 | 23 | 19 | 39 | 22 | 17 | 3 | 1 | 2 |
| **Married** | | | | | | | | | | |
| **Total** | **1,009,604** | **242,361** | **122,923** | **119,438** | **231,360** | **117,152** | **114,208** | **11,001** | **5,771** | **5,230** |
| 12 - 14 | 4,048 | 293 | 151 | 142 | 271 | 139 | 132 | 22 | 12 | 10 |
| 15 - 19 | 93,884 | 28,745 | 14,452 | 14,293 | 27,393 | 13,730 | 13,663 | 1,352 | 722 | 630 |
| 20 - 24 | 214,561 | 69,809 | 35,328 | 34,481 | 67,131 | 33,888 | 33,243 | 2,678 | 1,440 | 1,238 |
| 25 - 29 | 221,288 | 62,363 | 31,816 | 30,547 | 59,727 | 30,453 | 29,274 | 2,636 | 1,363 | 1,273 |
| 30 - 34 | 167,938 | 40,667 | 20,626 | 20,041 | 38,729 | 19,611 | 19,118 | 1,938 | 1,015 | 923 |
| 35 - 39 | 137,516 | 27,172 | 13,808 | 13,364 | 25,739 | 13,072 | 12,667 | 1,433 | 736 | 697 |
| 40 - 44 | 93,816 | 10,446 | 5,273 | 5,173 | 9,777 | 4,933 | 4,844 | 669 | 340 | 329 |
| 45 - 49 | 76,553 | 2,866 | 1,469 | 1,397 | 2,593 | 1,326 | 1,267 | 273 | 143 | 130 |
| **Divorced** | | | | | | | | | | |
| **Total** | **67,508** | **8,792** | **4,453** | **4,339** | **8,223** | **4,161** | **4,062** | **569** | **292** | **277** |
| 12 - 14 | 82 | 8 | 5 | 3 | 6 | 4 | 2 | 2 | 1 | 1 |
| 15 - 19 | 2,333 | 544 | 281 | 263 | 515 | 266 | 249 | 29 | 15 | 14 |
| 20 - 24 | 9,403 | 1,972 | 965 | 1,007 | 1,878 | 917 | 961 | 94 | 48 | 46 |
| 25 - 29 | 13,368 | 2,345 | 1,222 | 1,123 | 2,194 | 1,140 | 1,054 | 151 | 82 | 69 |
| 30 - 34 | 13,197 | 1,952 | 983 | 969 | 1,818 | 917 | 901 | 134 | 66 | 68 |
| 35 - 39 | 11,659 | 1,306 | 672 | 634 | 1,215 | 622 | 593 | 91 | 50 | 41 |
| 40 - 44 | 9,298 | 515 | 252 | 263 | 473 | 235 | 238 | 42 | 17 | 25 |
| 45 - 49 | 8,168 | 150 | 73 | 77 | 124 | 60 | 64 | 26 | 13 | 13 |

## Table C3i: Population of Women 12-49 Years by Number of Children Born Alive 12 Months Prior to the Census, Children Still Living, Children Dead, Sex of Child, Age Group and Marital Status, Rural/Urban, Zambia 2010

| Age Group and Marital Status | Number of Women 12 Years and Older | Children Born Alive 12 Months Prior to Census | | | Children Still Living | | | Children Dead | | |
|---|---|---|---|---|---|---|---|---|---|---|
| | | Total | Male | Female | Total | Male | Female | Total | Male | Female |
| **Separated** | | | | | | | | | | |
| **Total** | **49,234** | **8,630** | **4,374** | **4,256** | **8,223** | **4,158** | **4,065** | **407** | **216** | **191** |
| 12 - 14 | 90 | 10 | 5 | 5 | 10 | 5 | 5 | - | - | - |
| 15 - 19 | 3,387 | 1,028 | 511 | 517 | 973 | 481 | 492 | 55 | 30 | 25 |
| 20 - 24 | 9,264 | 2,310 | 1,167 | 1,143 | 2,204 | 1,108 | 1,096 | 106 | 59 | 47 |
| 25 - 29 | 10,771 | 2,206 | 1,106 | 1,100 | 2,108 | 1,054 | 1,054 | 98 | 52 | 46 |
| 30 - 34 | 8,961 | 1,546 | 791 | 755 | 1,479 | 762 | 717 | 67 | 29 | 38 |
| 35 - 39 | 7,322 | 1,015 | 532 | 483 | 962 | 501 | 461 | 53 | 31 | 22 |
| 40 - 44 | 5,238 | 410 | 212 | 198 | 389 | 200 | 189 | 21 | 12 | 9 |
| 45 - 49 | 4,201 | 105 | 50 | 55 | 98 | 47 | 51 | 7 | 3 | 4 |
| **Widowed** | | | | | | | | | | |
| **Total** | **55,394** | **3,740** | **1,954** | **1,786** | **3,477** | **1,807** | **1,670** | **263** | **147** | **116** |
| 12 - 14 | 69 | 3 | 1 | 2 | 3 | 1 | 2 | - | - | - |
| 15 - 19 | 545 | 101 | 56 | 45 | 96 | 53 | 43 | 5 | 3 | 2 |
| 20 - 24 | 2,167 | 420 | 226 | 194 | 396 | 210 | 186 | 24 | 16 | 8 |
| 25 - 29 | 4,747 | 734 | 376 | 358 | 686 | 348 | 338 | 48 | 28 | 20 |
| 30 - 34 | 8,353 | 939 | 473 | 466 | 873 | 437 | 436 | 66 | 36 | 30 |
| 35 - 39 | 10,656 | 864 | 472 | 392 | 808 | 442 | 366 | 56 | 30 | 26 |
| 40 - 44 | 13,357 | 487 | 260 | 227 | 443 | 235 | 208 | 44 | 25 | 19 |
| 45 - 49 | 15,500 | 192 | 90 | 102 | 172 | 81 | 91 | 20 | 9 | 11 |
| **Cohabiting** | | | | | | | | | | |
| **Total** | **57,150** | **14,388** | **7,238** | **7,150** | **13,775** | **6,901** | **6,874** | **613** | **337** | **276** |
| 12 - 14 | 549 | 85 | 40 | 45 | 80 | 38 | 42 | 5 | 2 | 3 |
| 15 - 19 | 11,817 | 3,758 | 1,848 | 1,910 | 3,622 | 1,771 | 1,851 | 136 | 77 | 59 |
| 20 - 24 | 15,597 | 4,667 | 2,365 | 2,302 | 4,475 | 2,253 | 2,222 | 192 | 112 | 80 |
| 25 - 29 | 11,335 | 2,974 | 1,491 | 1,483 | 2,856 | 1,435 | 1,421 | 118 | 56 | 62 |
| 30 - 34 | 7,093 | 1,569 | 795 | 774 | 1,494 | 753 | 741 | 75 | 42 | 33 |
| 35 - 39 | 5,239 | 916 | 480 | 436 | 864 | 452 | 412 | 52 | 28 | 24 |
| 40 - 44 | 3,168 | 333 | 173 | 160 | 306 | 159 | 147 | 27 | 14 | 13 |
| 45 - 49 | 2,352 | 86 | 46 | 40 | 78 | 40 | 38 | 8 | 6 | 2 |

## Table C3i: Population of Women 12-49 Years by Number of Children Born Alive 12 Months Prior to the Census, Children Still Living, Children Dead, Sex of Child, Age Group and Marital Status, Urban, Zambia 2010

| Age Group and Marital Status | Number of Women 12 Years and Older | Children Born Alive 12 Months Prior to Census | | | Children Still Living | | | Children Dead | | |
|---|---|---|---|---|---|---|---|---|---|---|
| | | Total | Male | Female | Total | Male | Female | Total | Male | Female |
| **Zambia Urban** | | | | | | | | | | |
| **Total** | **1,552,107** | **148,195** | **75,320** | **72,875** | **141,775** | **72,084** | **69,691** | **6,420** | **3,236** | **3,184** |
| 12 - 14 | 203,452 | 249 | 128 | 121 | 240 | 124 | 116 | 9 | 4 | 5 |
| 15 - 19 | 329,766 | 17,100 | 8,647 | 8,453 | 16,468 | 8,331 | 8,137 | 632 | 316 | 316 |
| 20 - 24 | 283,698 | 43,534 | 22,225 | 21,309 | 41,935 | 21,378 | 20,557 | 1,599 | 847 | 752 |
| 25 - 29 | 253,406 | 42,062 | 21,325 | 20,737 | 40,278 | 20,448 | 19,830 | 1,784 | 877 | 907 |
| 30 - 34 | 186,651 | 26,577 | 13,589 | 12,988 | 25,319 | 12,966 | 12,353 | 1,258 | 623 | 635 |
| 35 - 39 | 136,532 | 13,811 | 6,937 | 6,874 | 13,019 | 6,538 | 6,481 | 792 | 399 | 393 |
| 40 - 44 | 87,411 | 3,864 | 1,968 | 1,896 | 3,608 | 1,833 | 1,775 | 256 | 135 | 121 |
| 45 - 49 | 71,191 | 998 | 501 | 497 | 908 | 466 | 442 | 90 | 35 | 55 |
| **Never married** | | | | | | | | | | |
| **Total** | **717,737** | **14,388** | **7,266** | **7,122** | **13,894** | **7,031** | **6,863** | **494** | **235** | **259** |
| 12 - 14 | 201,561 | 161 | 77 | 84 | 154 | 75 | 79 | 7 | 2 | 5 |
| 15 - 19 | 290,094 | 5,812 | 2,979 | 2,833 | 5,646 | 2,906 | 2,740 | 166 | 73 | 93 |
| 20 - 24 | 132,957 | 5,370 | 2,686 | 2,684 | 5,218 | 2,603 | 2,615 | 152 | 83 | 69 |
| 25 - 29 | 55,013 | 1,957 | 960 | 997 | 1,853 | 915 | 938 | 104 | 45 | 59 |
| 30 - 34 | 21,647 | 748 | 383 | 365 | 698 | 360 | 338 | 50 | 23 | 27 |
| 35 - 39 | 9,995 | 272 | 143 | 129 | 261 | 135 | 126 | 11 | 8 | 3 |
| 40 - 44 | 4,217 | 54 | 27 | 27 | 51 | 26 | 25 | 3 | 1 | 2 |
| 45 - 49 | 2,253 | 14 | 11 | 3 | 13 | 11 | 2 | 1 | - | 1 |
| **Married** | | | | | | | | | | |
| **Total** | **644,528** | **115,442** | **58,824** | **56,618** | **110,555** | **56,363** | **54,192** | **4,887** | **2,461** | **2,426** |
| 12 - 14 | 1,523 | 45 | 24 | 21 | 44 | 23 | 21 | 1 | 1 | - |
| 15 - 19 | 30,635 | 8,816 | 4,423 | 4,393 | 8,451 | 4,238 | 4,213 | 365 | 185 | 180 |
| 20 - 24 | 124,245 | 32,910 | 16,869 | 16,041 | 31,687 | 16,230 | 15,457 | 1,223 | 639 | 584 |
| 25 - 29 | 163,204 | 35,467 | 18,076 | 17,391 | 34,064 | 17,369 | 16,695 | 1,403 | 707 | 696 |
| 30 - 34 | 128,999 | 22,556 | 11,541 | 11,015 | 21,565 | 11,057 | 10,508 | 991 | 484 | 507 |
| 35 - 39 | 94,314 | 11,704 | 5,885 | 5,819 | 11,050 | 5,561 | 5,489 | 654 | 324 | 330 |
| 40 - 44 | 56,883 | 3,169 | 1,624 | 1,545 | 2,976 | 1,521 | 1,455 | 193 | 103 | 90 |
| 45 - 49 | 44,725 | 775 | 382 | 393 | 718 | 364 | 354 | 57 | 18 | 39 |
| **Divorced** | | | | | | | | | | |
| **Total** | **42,904** | **2,664** | **1,379** | **1,285** | **2,480** | **1,279** | **1,201** | **184** | **100** | **84** |
| 12 - 14 | 23 | 4 | 2 | 2 | 4 | 2 | 2 | - | - | - |
| 15 - 19 | 775 | 122 | 68 | 54 | 113 | 62 | 51 | 9 | 6 | 3 |
| 20 - 24 | 4,828 | 606 | 320 | 286 | 573 | 302 | 271 | 33 | 18 | 15 |
| 25 - 29 | 9,173 | 771 | 396 | 375 | 711 | 366 | 345 | 60 | 30 | 30 |
| 30 - 34 | 9,970 | 657 | 334 | 323 | 609 | 308 | 301 | 48 | 26 | 22 |
| 35 - 39 | 7,962 | 344 | 175 | 169 | 323 | 162 | 161 | 21 | 13 | 8 |
| 40 - 44 | 5,667 | 122 | 56 | 66 | 113 | 52 | 61 | 9 | 4 | 5 |
| 45 - 49 | 4,506 | 38 | 28 | 10 | 34 | 25 | 9 | 4 | 3 | 1 |

## Table C3i: Population of Women 12-49 Years by Number of Children Born Alive 12 Months Prior to the Census, Children Still Living, Children Dead, Sex of Child, Age Group and Marital Status, Urban, Zambia 2010

| Age Group and Marital Status | Number of Women 12 Years and Older | Children Born Alive 12 Months Prior to Census | | | Children Still Living | | | Children Dead | | |
|---|---|---|---|---|---|---|---|---|---|---|
| | | Total | Male | Female | Total | Male | Female | Total | Male | Female |
| **Separated** | | | | | | | | | | |
| **Total** | **37,839** | **4,277** | **2,092** | **2,185** | **4,036** | **1,973** | **2,063** | **241** | **119** | **122** |
| 12 - 14 | 49 | 13 | 9 | 4 | 13 | 9 | 4 | - | - | - |
| 15 - 19 | 1,858 | 539 | 259 | 280 | 516 | 245 | 271 | 23 | 14 | 9 |
| 20 - 24 | 6,539 | 1,183 | 583 | 600 | 1,128 | 558 | 570 | 55 | 25 | 30 |
| 25 - 29 | 9,406 | 1,140 | 554 | 586 | 1,068 | 523 | 545 | 72 | 31 | 41 |
| 30 - 34 | 8,238 | 805 | 399 | 406 | 755 | 374 | 381 | 50 | 25 | 25 |
| 35 - 39 | 5,866 | 432 | 208 | 224 | 404 | 191 | 213 | 28 | 17 | 11 |
| 40 - 44 | 3,379 | 132 | 66 | 66 | 124 | 61 | 63 | 8 | 5 | 3 |
| 45 - 49 | 2,504 | 33 | 14 | 19 | 28 | 12 | 16 | 5 | 2 | 3 |
| **Widowed** | | | | | | | | | | |
| **Total** | **63,557** | **2,356** | **1,164** | **1,192** | **2,157** | **1,070** | **1,087** | **199** | **94** | **105** |
| 12 - 14 | 72 | - | - | - | - | - | - | - | - | - |
| 15 - 19 | 289 | 41 | 24 | 17 | 40 | 23 | 17 | 1 | 1 | - |
| 20 - 24 | 1,850 | 255 | 132 | 123 | 242 | 128 | 114 | 13 | 4 | 9 |
| 25 - 29 | 5,428 | 493 | 242 | 251 | 456 | 224 | 232 | 37 | 18 | 19 |
| 30 - 34 | 10,856 | 683 | 347 | 336 | 634 | 323 | 311 | 49 | 24 | 25 |
| 35 - 39 | 14,159 | 521 | 255 | 266 | 473 | 232 | 241 | 48 | 23 | 25 |
| 40 - 44 | 15,163 | 250 | 116 | 134 | 220 | 102 | 118 | 30 | 14 | 16 |
| 45 - 49 | 15,740 | 113 | 48 | 65 | 92 | 38 | 54 | 21 | 10 | 11 |
| **Cohabiting** | | | | | | | | | | |
| **Total** | **45,542** | **9,068** | **4,595** | **4,473** | **8,653** | **4,368** | **4,285** | **415** | **227** | **188** |
| 12 - 14 | 224 | 26 | 16 | 10 | 25 | 15 | 10 | 1 | 1 | - |
| 15 - 19 | 6,115 | 1,770 | 894 | 876 | 1,702 | 857 | 845 | 68 | 37 | 31 |
| 20 - 24 | 13,279 | 3,210 | 1,635 | 1,575 | 3,087 | 1,557 | 1,530 | 123 | 78 | 45 |
| 25 - 29 | 11,182 | 2,234 | 1,097 | 1,137 | 2,126 | 1,051 | 1,075 | 108 | 46 | 62 |
| 30 - 34 | 6,941 | 1,128 | 585 | 543 | 1,058 | 544 | 514 | 70 | 41 | 29 |
| 35 - 39 | 4,236 | 538 | 271 | 267 | 508 | 257 | 251 | 30 | 14 | 16 |
| 40 - 44 | 2,102 | 137 | 79 | 58 | 124 | 71 | 53 | 13 | 8 | 5 |
| 45 - 49 | 1,463 | 25 | 18 | 7 | 23 | 16 | 7 | 2 | 2 | - |

**Table C4: Population of Women 12 Years and Older by Age Group, Average Number of Children Ever Born, Number of Children Ever Born Alive, Children Still Living, Children Dead, Sex of Child and Highest Educational Attainment, Rural/Urban, Zambia 2010**

| Age Group and Highest Level of Education | Number of Women 12 Years and Older | Average Number of Children Ever Born | Children Ever Born Alive | | | Children Still Living | | | Children Dead | | |
|---|---|---|---|---|---|---|---|---|---|---|---|
| | | | Total | Male | Female | Total | Male | Female | Total | Male | Female |
| **Zambia Total** | | | | | | | | | | | |
| **Total** | **3,992,401** | **2.7** | **10,970,814** | **5,464,308** | **5,506,506** | **9,000,504** | **4,469,438** | **4,531,066** | **1,970,310** | **994,870** | **975,440** |
| 12 - 14 | 486,104 | * | 7,947 | 4,000 | 3,947 | 6,924 | 3,431 | 3,493 | 1,023 | 569 | 454 |
| 15 - 19 | 735,025 | 0.2 | 175,811 | 87,622 | 88,189 | 161,182 | 79,880 | 81,302 | 14,629 | 7,742 | 6,887 |
| 20 - 24 | 612,600 | 1.3 | 822,610 | 409,373 | 413,237 | 752,608 | 372,507 | 380,101 | 70,002 | 36,866 | 33,136 |
| 25 - 29 | 541,751 | 2.6 | 1,419,661 | 706,979 | 712,682 | 1,279,281 | 633,721 | 645,560 | 140,380 | 73,258 | 67,122 |
| 30 - 34 | 403,076 | 3.9 | 1,553,530 | 774,823 | 778,707 | 1,371,087 | 680,133 | 690,954 | 182,443 | 94,690 | 87,753 |
| 35 - 39 | 314,852 | 4.8 | 1,525,804 | 763,380 | 762,424 | 1,319,083 | 657,096 | 661,987 | 206,721 | 106,284 | 100,437 |
| 40 - 44 | 215,331 | 5.6 | 1,210,821 | 606,130 | 604,691 | 1,013,909 | 505,437 | 508,472 | 196,912 | 100,693 | 96,219 |
| 45 - 49 | 180,156 | 6.0 | 1,080,764 | 540,626 | 540,138 | 881,305 | 440,076 | 441,229 | 199,459 | 100,550 | 98,909 |
| 50 - 54 | 141,558 | 6.2 | 879,792 | 439,039 | 440,753 | 683,742 | 342,048 | 341,694 | 196,050 | 96,991 | 99,059 |
| 55 - 59 | 94,791 | 6.4 | 603,169 | 299,663 | 303,506 | 449,826 | 224,820 | 225,006 | 153,343 | 74,843 | 78,500 |
| 60 - 64 | 88,456 | 6.5 | 573,756 | 284,473 | 289,283 | 393,365 | 196,162 | 197,203 | 180,391 | 88,311 | 92,080 |
| 65+ | 178,701 | 6.3 | 1,117,149 | 548,200 | 568,949 | 688,192 | 334,127 | 354,065 | 428,957 | 214,073 | 214,884 |
| **Never Attended** | | | | | | | | | | | |
| **Total** | **751,606** | **4.1** | **3,070,469** | **1,524,901** | **1,545,568** | **2,320,926** | **1,149,347** | **1,171,579** | **749,543** | **375,554** | **373,989** |
| 12 - 14 | 41,432 | 0.0 | 1,166 | 600 | 566 | 1,000 | 515 | 485 | 166 | 85 | 81 |
| 15 - 19 | 70,114 | 0.5 | 31,918 | 15,955 | 15,963 | 28,720 | 14,268 | 14,452 | 3,198 | 1,687 | 1,511 |
| 20 - 24 | 94,326 | 1.8 | 170,954 | 84,658 | 86,296 | 153,241 | 75,518 | 77,723 | 17,713 | 9,140 | 8,573 |
| 25 - 29 | 98,807 | 3.1 | 309,052 | 153,655 | 155,397 | 271,529 | 134,187 | 137,342 | 37,523 | 19,468 | 18,055 |
| 30 - 34 | 73,562 | 4.4 | 324,399 | 161,961 | 162,438 | 277,060 | 137,561 | 139,499 | 47,339 | 24,400 | 22,939 |
| 35 - 39 | 63,011 | 5.3 | 334,330 | 167,454 | 166,876 | 279,716 | 139,734 | 139,982 | 54,614 | 27,720 | 26,894 |
| 40 - 44 | 47,836 | 5.9 | 282,958 | 142,279 | 140,679 | 226,595 | 113,712 | 112,883 | 56,363 | 28,567 | 27,796 |
| 45 - 49 | 43,228 | 6.1 | 263,819 | 132,217 | 131,602 | 204,533 | 102,423 | 102,110 | 59,286 | 29,794 | 29,492 |
| 50 - 54 | 39,496 | 6.1 | 242,875 | 120,533 | 122,342 | 176,777 | 88,037 | 88,740 | 66,098 | 32,496 | 33,602 |
| 55 - 59 | 30,437 | 6.2 | 188,726 | 93,679 | 95,047 | 132,937 | 66,288 | 66,649 | 55,789 | 27,391 | 28,398 |
| 60 - 64 | 40,426 | 6.3 | 255,202 | 126,065 | 129,137 | 168,219 | 83,384 | 84,835 | 86,983 | 42,681 | 44,302 |
| 65+ | 108,931 | 6.1 | 665,070 | 325,845 | 339,225 | 400,599 | 193,720 | 206,879 | 264,471 | 132,125 | 132,346 |
| **Primary Level** | | | | | | | | | | | |
| **Total** | **1,857,338** | **2.8** | **5,276,358** | **2,634,741** | **2,641,617** | **4,323,666** | **2,151,841** | **2,171,825** | **952,692** | **482,900** | **469,792** |
| 12 - 14 | 403,162 | * | 5,974 | 3,016 | 2,958 | 5,189 | 2,576 | 2,613 | 785 | 440 | 345 |
| 15 - 19 | 317,116 | 0.3 | 87,255 | 43,468 | 43,787 | 79,413 | 39,301 | 40,112 | 7,842 | 4,167 | 3,675 |
| 20 - 24 | 214,871 | 1.8 | 386,521 | 192,386 | 194,135 | 350,662 | 173,346 | 177,316 | 35,859 | 19,040 | 16,819 |
| 25 - 29 | 215,864 | 3.2 | 685,950 | 342,010 | 343,940 | 612,274 | 303,552 | 308,722 | 73,676 | 38,458 | 35,218 |
| 30 - 34 | 171,082 | 4.5 | 763,144 | 381,050 | 382,094 | 665,728 | 330,382 | 335,346 | 97,416 | 50,668 | 46,748 |
| 35 - 39 | 141,829 | 5.5 | 773,900 | 388,175 | 385,725 | 659,879 | 329,229 | 330,650 | 114,021 | 58,946 | 55,075 |
| 40 - 44 | 101,051 | 6.2 | 628,666 | 315,732 | 312,934 | 519,762 | 259,765 | 259,997 | 108,904 | 55,967 | 52,937 |
| 45 - 49 | 87,204 | 6.5 | 566,996 | 284,370 | 282,626 | 456,035 | 228,405 | 227,630 | 110,961 | 55,965 | 54,996 |
| 50 - 54 | 67,012 | 6.6 | 445,560 | 223,305 | 222,255 | 342,609 | 172,114 | 170,495 | 102,951 | 51,191 | 51,760 |
| 55 - 59 | 43,267 | 6.8 | 294,602 | 146,276 | 148,326 | 217,568 | 108,736 | 108,832 | 77,034 | 37,540 | 39,494 |
| 60 - 64 | 36,915 | 6.9 | 253,009 | 125,871 | 127,138 | 174,276 | 87,337 | 86,939 | 78,733 | 38,534 | 40,199 |
| 65+ | 57,965 | 6.6 | 384,781 | 189,082 | 195,699 | 240,271 | 117,098 | 123,173 | 144,510 | 71,984 | 72,526 |

## Table C4: Population of Women 12 Years and Older by Age Group, Average Number of Children Ever Born, Number of Children Ever Born Alive, Children Still Living, Children Dead, Sex of Child and Highest Educational Attainment, Rural/Urban, Zambia 2010

| Age Group and Highest Level of Education | Number of Women 12 Years and Older | Average Number of Children Ever Born | Children Ever Born Alive | | | Children Still Living | | | Children Dead | | |
|---|---|---|---|---|---|---|---|---|---|---|---|
| | | | Total | Male | Female | Total | Male | Female | Total | Male | Female |
| **Secondary Level** | | | | | | | | | | | |
| **Total** | **1,165,720** | **1.8** | **2,130,869** | **1,058,768** | **1,072,101** | **1,908,386** | **945,711** | **962,675** | **222,483** | **113,057** | **109,426** |
| 12 - 14 | 41,510 | * | 807 | 384 | 423 | 735 | 340 | 395 | 72 | 44 | 28 |
| 15 - 19 | 344,096 | 0.2 | 55,995 | 27,872 | 28,123 | 52,456 | 26,015 | 26,441 | 3,539 | 1,857 | 1,682 |
| 20 - 24 | 271,859 | 0.9 | 250,618 | 125,064 | 125,554 | 234,901 | 116,762 | 118,139 | 15,717 | 8,302 | 7,415 |
| 25 - 29 | 174,263 | 2.1 | 365,733 | 181,831 | 183,902 | 339,528 | 168,064 | 171,464 | 26,205 | 13,767 | 12,438 |
| 30 - 34 | 116,837 | 3.3 | 382,919 | 190,414 | 192,505 | 349,978 | 173,324 | 176,654 | 32,941 | 17,090 | 15,851 |
| 35 - 39 | 81,756 | 4.2 | 339,458 | 168,814 | 170,644 | 306,641 | 151,941 | 154,700 | 32,817 | 16,873 | 15,944 |
| 40 - 44 | 48,289 | 4.9 | 236,961 | 117,194 | 119,767 | 210,336 | 103,696 | 106,640 | 26,625 | 13,498 | 13,127 |
| 45 - 49 | 35,644 | 5.4 | 193,213 | 95,827 | 97,386 | 169,051 | 83,641 | 85,410 | 24,162 | 12,186 | 11,976 |
| 50 - 54 | 23,324 | 5.8 | 136,393 | 67,831 | 68,562 | 115,182 | 57,399 | 57,783 | 21,211 | 10,432 | 10,779 |
| 55 - 59 | 13,078 | 6.1 | 79,258 | 39,293 | 39,965 | 64,305 | 32,147 | 32,158 | 14,953 | 7,146 | 7,807 |
| 60 - 64 | 6,840 | 6.2 | 42,119 | 20,823 | 21,296 | 32,067 | 16,019 | 16,048 | 10,052 | 4,804 | 5,248 |
| 65+ | 8,224 | 5.8 | 47,395 | 23,421 | 23,974 | 33,206 | 16,363 | 16,843 | 14,189 | 7,058 | 7,131 |
| **Tertiary Level** | | | | | | | | | | | |
| **Total** | **217,737** | **2.3** | **493,118** | **245,898** | **247,220** | **447,526** | **222,539** | **224,987** | **45,592** | **23,359** | **22,233** |
| 12 - 14 | - | - | - | - | - | - | - | - | - | - | - |
| 15 - 19 | 3,699 | 0.2 | 643 | 327 | 316 | 593 | 296 | 297 | 50 | 31 | 19 |
| 20 - 24 | 31,544 | 0.5 | 14,517 | 7,265 | 7,252 | 13,804 | 6,881 | 6,923 | 713 | 384 | 329 |
| 25 - 29 | 52,817 | 1.1 | 58,926 | 29,483 | 29,443 | 55,950 | 27,918 | 28,032 | 2,976 | 1,565 | 1,411 |
| 30 - 34 | 41,595 | 2.0 | 83,068 | 41,398 | 41,670 | 78,321 | 38,866 | 39,455 | 4,747 | 2,532 | 2,215 |
| 35 - 39 | 28,256 | 2.8 | 78,116 | 38,937 | 39,179 | 72,847 | 36,192 | 36,655 | 5,269 | 2,745 | 2,524 |
| 40 - 44 | 18,155 | 3.4 | 62,236 | 30,925 | 31,311 | 57,216 | 28,264 | 28,952 | 5,020 | 2,661 | 2,359 |
| 45 - 49 | 14,080 | 4.0 | 56,736 | 28,212 | 28,524 | 51,686 | 25,607 | 26,079 | 5,050 | 2,605 | 2,445 |
| 50 - 54 | 11,726 | 4.7 | 54,964 | 27,370 | 27,594 | 49,174 | 24,498 | 24,676 | 5,790 | 2,872 | 2,918 |
| 55 - 59 | 8,009 | 5.1 | 40,583 | 20,415 | 20,168 | 35,016 | 17,649 | 17,367 | 5,567 | 2,766 | 2,801 |
| 60 - 64 | 4,275 | 5.5 | 23,426 | 11,714 | 11,712 | 18,803 | 9,422 | 9,381 | 4,623 | 2,292 | 2,331 |
| 65+ | 3,581 | 5.6 | 19,903 | 9,852 | 10,051 | 14,116 | 6,946 | 7,170 | 5,787 | 2,906 | 2,881 |

## Table C4: Population of Women 12 Years and Older by Age Group, Average Number of Children Ever Born, Number of Children Ever Born Alive, Children Still Living, Children Dead, Sex of Child and Highest Educational Attainment, Rural, Zambia 2010

| Age Group and Highest Level of Education | Number of Women 12 Years and Older | Average Number of Children Ever Born | Children Ever Born Alive | | | Children Still Living | | | Children Dead | | |
|---|---|---|---|---|---|---|---|---|---|---|---|
| | | | Total | Male | Female | Total | Male | Female | Total | Male | Female |
| **Zambia Rural** | | | | | | | | | | | |
| **Total** | **2,273,363** | **3.1** | **7,145,714** | **3,565,538** | **3,580,176** | **5,736,895** | **2,853,935** | **2,882,960** | **1,408,819** | **711,603** | **697,216** |
| 12 - 14 | 282,652 | * | 4,987 | 2,570 | 2,417 | 4,307 | 2,190 | 2,117 | 680 | 380 | 300 |
| 15 - 19 | 405,259 | 0.3 | 121,237 | 60,581 | 60,656 | 110,453 | 54,881 | 55,572 | 10,784 | 5,700 | 5,084 |
| 20 - 24 | 328,902 | 1.6 | 539,787 | 268,274 | 271,513 | 490,061 | 242,063 | 247,998 | 49,726 | 26,211 | 23,515 |
| 25 - 29 | 288,345 | 3.1 | 897,227 | 446,891 | 450,336 | 798,681 | 395,600 | 403,081 | 98,546 | 51,291 | 47,255 |
| 30 - 34 | 216,425 | 4.5 | 970,606 | 484,771 | 485,835 | 842,695 | 418,765 | 423,930 | 127,911 | 66,006 | 61,905 |
| 35 - 39 | 178,320 | 5.5 | 977,140 | 490,345 | 486,795 | 828,790 | 414,237 | 414,553 | 148,350 | 76,108 | 72,242 |
| 40 - 44 | 127,920 | 6.2 | 790,430 | 397,927 | 392,503 | 646,028 | 324,016 | 322,012 | 144,402 | 73,911 | 70,491 |
| 45 - 49 | 108,965 | 6.4 | 695,798 | 349,456 | 346,342 | 551,315 | 276,443 | 274,872 | 144,483 | 73,013 | 71,470 |
| 50 - 54 | 86,527 | 6.4 | 556,535 | 278,388 | 278,147 | 419,006 | 210,069 | 208,937 | 137,529 | 68,319 | 69,210 |
| 55 - 59 | 59,286 | 6.5 | 385,233 | 191,460 | 193,773 | 279,684 | 139,796 | 139,888 | 105,549 | 51,664 | 53,885 |
| 60 - 64 | 61,277 | 6.5 | 400,026 | 198,647 | 201,379 | 270,834 | 135,041 | 135,793 | 129,192 | 63,606 | 65,586 |
| 65+ | 129,485 | 6.2 | 806,708 | 396,228 | 410,480 | 495,041 | 240,834 | 254,207 | 311,667 | 155,394 | 156,273 |
| **Never Attended** | | | | | | | | | | | |
| **Total** | **623,422** | **4.1** | **2,549,268** | **1,267,581** | **1,281,687** | **1,933,298** | **958,570** | **974,728** | **615,970** | **309,011** | **306,959** |
| 12 - 14 | 34,669 | 0.0 | 1,006 | 527 | 479 | 863 | 453 | 410 | 143 | 74 | 69 |
| 15 - 19 | 59,905 | 0.5 | 28,276 | 14,148 | 14,128 | 25,415 | 12,641 | 12,774 | 2,861 | 1,507 | 1,354 |
| 20 - 24 | 79,999 | 1.9 | 148,722 | 73,665 | 75,057 | 133,086 | 65,582 | 67,504 | 15,636 | 8,083 | 7,553 |
| 25 - 29 | 83,393 | 3.2 | 267,971 | 133,136 | 134,835 | 234,865 | 115,977 | 118,888 | 33,106 | 17,159 | 15,947 |
| 30 - 34 | 61,496 | 4.5 | 278,798 | 139,272 | 139,526 | 237,385 | 117,954 | 119,431 | 41,413 | 21,318 | 20,095 |
| 35 - 39 | 52,883 | 5.4 | 287,529 | 144,139 | 143,390 | 239,652 | 119,828 | 119,824 | 47,877 | 24,311 | 23,566 |
| 40 - 44 | 40,296 | 6.0 | 242,486 | 122,198 | 120,288 | 193,350 | 97,294 | 96,056 | 49,136 | 24,904 | 24,232 |
| 45 - 49 | 36,011 | 6.2 | 221,592 | 111,251 | 110,341 | 170,689 | 85,619 | 85,070 | 50,903 | 25,632 | 25,271 |
| 50 - 54 | 32,180 | 6.2 | 198,601 | 98,469 | 100,132 | 143,932 | 71,543 | 72,389 | 54,669 | 26,926 | 27,743 |
| 55 - 59 | 24,284 | 6.2 | 150,521 | 74,809 | 75,712 | 105,611 | 52,727 | 52,884 | 44,910 | 22,082 | 22,828 |
| 60 - 64 | 32,595 | 6.3 | 204,666 | 101,160 | 103,506 | 134,896 | 66,809 | 68,087 | 69,770 | 34,351 | 35,419 |
| 65+ | 85,711 | 6.1 | 519,100 | 254,807 | 264,293 | 313,554 | 152,143 | 161,411 | 205,546 | 102,664 | 102,882 |
| **Primary Level** | | | | | | | | | | | |
| **Total** | **1,232,633** | **3.0** | **3,710,394** | **1,855,806** | **1,854,588** | **3,025,244** | **1,507,917** | **1,517,327** | **685,150** | **347,889** | **337,261** |
| 12 - 14 | 237,902 | * | 3,719 | 1,915 | 1,804 | 3,207 | 1,623 | 1,584 | 512 | 292 | 220 |
| 15 - 19 | 223,885 | 0.3 | 66,723 | 33,270 | 33,453 | 60,530 | 29,980 | 30,550 | 6,193 | 3,290 | 2,903 |
| 20 - 24 | 151,813 | 1.9 | 284,433 | 141,484 | 142,949 | 257,502 | 127,164 | 130,338 | 26,931 | 14,320 | 12,611 |
| 25 - 29 | 144,834 | 3.4 | 488,031 | 243,562 | 244,469 | 433,984 | 215,434 | 218,550 | 54,047 | 28,128 | 25,919 |
| 30 - 34 | 113,049 | 4.8 | 539,251 | 269,396 | 269,855 | 467,678 | 232,426 | 235,252 | 71,573 | 36,970 | 34,603 |
| 35 - 39 | 96,046 | 5.8 | 555,307 | 279,005 | 276,302 | 469,917 | 234,924 | 234,993 | 85,390 | 44,081 | 41,309 |
| 40 - 44 | 69,190 | 6.5 | 450,311 | 226,952 | 223,359 | 368,170 | 184,593 | 183,577 | 82,141 | 42,359 | 39,782 |
| 45 - 49 | 58,739 | 6.7 | 394,457 | 198,247 | 196,210 | 312,762 | 156,916 | 155,846 | 81,695 | 41,331 | 40,364 |
| 50 - 54 | 44,242 | 6.8 | 298,947 | 150,248 | 148,699 | 226,451 | 113,996 | 112,455 | 72,496 | 36,252 | 36,244 |
| 55 - 59 | 28,745 | 6.8 | 196,850 | 97,834 | 99,016 | 143,846 | 71,950 | 71,896 | 53,004 | 25,884 | 27,120 |
| 60 - 64 | 24,994 | 6.9 | 172,047 | 85,800 | 86,247 | 118,369 | 59,337 | 59,032 | 53,678 | 26,463 | 27,215 |
| 65+ | 39,194 | 6.6 | 260,318 | 128,093 | 132,225 | 162,828 | 79,574 | 83,254 | 97,490 | 48,519 | 48,971 |

## Table C4: Population of Women 12 Years and Older by Age Group, Average Number of Children Ever Born, Number of Children Ever Born Alive, Children Still Living, Children Dead, Sex of Child and Highest Educational Attainment, Rural, Zambia 2010

| Age Group and Highest Level of Education | Number of Women 12 Years and Older | Average Number of Children Ever Born | Children Ever Born Alive | | | Children Still Living | | | Children Dead | | |
|---|---|---|---|---|---|---|---|---|---|---|---|
| | | | Total | Male | Female | Total | Male | Female | Total | Male | Female |
| **Secondary Level** | | | | | | | | | | | |
| **Total** | **383,473** | **2.1** | **792,577** | **395,256** | **397,321** | **696,833** | **346,690** | **350,143** | **95,744** | **48,566** | **47,178** |
| 12 - 14 | 10,081 | * | 262 | 128 | 134 | 237 | 114 | 123 | 25 | 14 | 11 |
| 15 - 19 | 120,572 | 0.2 | 25,965 | 13,027 | 12,938 | 24,254 | 12,136 | 12,118 | 1,711 | 891 | 820 |
| 20 - 24 | 93,120 | 1.1 | 103,891 | 51,748 | 52,143 | 96,895 | 48,031 | 48,864 | 6,996 | 3,717 | 3,279 |
| 25 - 29 | 52,012 | 2.5 | 130,482 | 64,863 | 65,619 | 119,757 | 59,223 | 60,534 | 10,725 | 5,640 | 5,085 |
| 30 - 34 | 35,522 | 3.9 | 137,986 | 68,877 | 69,109 | 124,129 | 61,705 | 62,424 | 13,857 | 7,172 | 6,685 |
| 35 - 39 | 25,408 | 4.8 | 121,776 | 60,888 | 60,888 | 107,757 | 53,728 | 54,029 | 14,019 | 7,160 | 6,859 |
| 40 - 44 | 15,857 | 5.5 | 87,150 | 43,472 | 43,678 | 75,202 | 37,460 | 37,742 | 11,948 | 6,012 | 5,936 |
| 45 - 49 | 11,902 | 5.8 | 69,051 | 34,586 | 34,465 | 58,481 | 29,233 | 29,248 | 10,570 | 5,353 | 5,217 |
| 50 - 54 | 8,005 | 6.0 | 48,181 | 24,233 | 23,948 | 39,360 | 19,853 | 19,507 | 8,821 | 4,380 | 4,441 |
| 55 - 59 | 4,769 | 6.2 | 29,502 | 14,604 | 14,898 | 23,289 | 11,616 | 11,673 | 6,213 | 2,988 | 3,225 |
| 60 - 64 | 2,739 | 6.4 | 17,589 | 8,727 | 8,862 | 13,156 | 6,619 | 6,537 | 4,433 | 2,108 | 2,325 |
| 65+ | 3,486 | 6.0 | 20,742 | 10,103 | 10,639 | 14,316 | 6,972 | 7,344 | 6,426 | 3,131 | 3,295 |
| **Tertiary Level** | | | | | | | | | | | |
| **Total** | **33,835** | **2.8** | **93,475** | **46,895** | **46,580** | **81,520** | **40,758** | **40,762** | **11,955** | **6,137** | **5,818** |
| 12 - 14 | - | - | - | - | - | - | - | - | - | - | - |
| 15 - 19 | 897 | 0.3 | 273 | 136 | 137 | 254 | 124 | 130 | 19 | 12 | 7 |
| 20 - 24 | 3,970 | 0.7 | 2,741 | 1,377 | 1,364 | 2,578 | 1,286 | 1,292 | 163 | 91 | 72 |
| 25 - 29 | 8,106 | 1.3 | 10,743 | 5,330 | 5,413 | 10,075 | 4,966 | 5,109 | 668 | 364 | 304 |
| 30 - 34 | 6,358 | 2.3 | 14,571 | 7,226 | 7,345 | 13,503 | 6,680 | 6,823 | 1,068 | 546 | 522 |
| 35 - 39 | 3,983 | 3.1 | 12,528 | 6,313 | 6,215 | 11,464 | 5,757 | 5,707 | 1,064 | 556 | 508 |
| 40 - 44 | 2,577 | 4.1 | 10,483 | 5,305 | 5,178 | 9,306 | 4,669 | 4,637 | 1,177 | 636 | 541 |
| 45 - 49 | 2,313 | 4.6 | 10,698 | 5,372 | 5,326 | 9,383 | 4,675 | 4,708 | 1,315 | 697 | 618 |
| 50 - 54 | 2,100 | 5.1 | 10,806 | 5,438 | 5,368 | 9,263 | 4,677 | 4,586 | 1,543 | 761 | 782 |
| 55 - 59 | 1,488 | 5.6 | 8,360 | 4,213 | 4,147 | 6,938 | 3,503 | 3,435 | 1,422 | 710 | 712 |
| 60 - 64 | 949 | 6.0 | 5,724 | 2,960 | 2,764 | 4,413 | 2,276 | 2,137 | 1,311 | 684 | 627 |
| 65+ | 1,094 | 6.0 | 6,548 | 3,225 | 3,323 | 4,343 | 2,145 | 2,198 | 2,205 | 1,080 | 1,125 |

## Table C4: Population of Women 12 Years and Older by Age Group, Average Number of Children Ever Born, Number of Children Ever Born Alive, Children Still Living, Children Dead, Sex of Child and Highest Educational Attainment, Urban, Zambia 2010

| Age Group and Highest Level of Education | Number of Women 12 Years and Older | Average Number of Children Ever Born | Children Ever Born Alive | | | Children Still Living | | | Children Dead | | |
|---|---|---|---|---|---|---|---|---|---|---|---|
| | | | Total | Male | Female | Total | Male | Female | Total | Male | Female |
| **Zambia Urban** | | | | | | | | | | | |
| **Total** | **1,719,038** | **2.2** | **3,825,100** | **1,898,770** | **1,926,330** | **3,263,609** | **1,615,503** | **1,648,106** | **561,491** | **283,267** | **278,224** |
| 12 - 14 | 203,452 | * | 2,960 | 1,430 | 1,530 | 2,617 | 1,241 | 1,376 | 343 | 189 | 154 |
| 15 - 19 | 329,766 | 0.2 | 54,574 | 27,041 | 27,533 | 50,729 | 24,999 | 25,730 | 3,845 | 2,042 | 1,803 |
| 20 - 24 | 283,698 | 1.0 | 282,823 | 141,099 | 141,724 | 262,547 | 130,444 | 132,103 | 20,276 | 10,655 | 9,621 |
| 25 - 29 | 253,406 | 2.1 | 522,434 | 260,088 | 262,346 | 480,600 | 238,121 | 242,479 | 41,834 | 21,967 | 19,867 |
| 30 - 34 | 186,651 | 3.1 | 582,924 | 290,052 | 292,872 | 528,392 | 261,368 | 267,024 | 54,532 | 28,684 | 25,848 |
| 35 - 39 | 136,532 | 4.0 | 548,664 | 273,035 | 275,629 | 490,293 | 242,859 | 247,434 | 58,371 | 30,176 | 28,195 |
| 40 - 44 | 87,411 | 4.8 | 420,391 | 208,203 | 212,188 | 367,881 | 181,421 | 186,460 | 52,510 | 26,782 | 25,728 |
| 45 - 49 | 71,191 | 5.4 | 384,966 | 191,170 | 193,796 | 329,990 | 163,633 | 166,357 | 54,976 | 27,537 | 27,439 |
| 50 - 54 | 55,031 | 5.9 | 323,257 | 160,651 | 162,606 | 264,736 | 131,979 | 132,757 | 58,521 | 28,672 | 29,849 |
| 55 - 59 | 35,505 | 6.1 | 217,936 | 108,203 | 109,733 | 170,142 | 85,024 | 85,118 | 47,794 | 23,179 | 24,615 |
| 60 - 64 | 27,179 | 6.4 | 173,730 | 85,826 | 87,904 | 122,531 | 61,121 | 61,410 | 51,199 | 24,705 | 26,494 |
| 65+ | 49,216 | 6.3 | 310,441 | 151,972 | 158,469 | 193,151 | 93,293 | 99,858 | 117,290 | 58,679 | 58,611 |
| **Never Attended** | | | | | | | | | | | |
| **Total** | **128,184** | **4.1** | **521,201** | **257,320** | **263,881** | **387,628** | **190,777** | **196,851** | **133,573** | **66,543** | **67,030** |
| 12 - 14 | 6,763 | 0.0 | 160 | 73 | 87 | 137 | 62 | 75 | 23 | 11 | 12 |
| 15 - 19 | 10,209 | 0.4 | 3,642 | 1,807 | 1,835 | 3,305 | 1,627 | 1,678 | 337 | 180 | 157 |
| 20 - 24 | 14,327 | 1.6 | 22,232 | 10,993 | 11,239 | 20,155 | 9,936 | 10,219 | 2,077 | 1,057 | 1,020 |
| 25 - 29 | 15,414 | 2.7 | 41,081 | 20,519 | 20,562 | 36,664 | 18,210 | 18,454 | 4,417 | 2,309 | 2,108 |
| 30 - 34 | 12,066 | 3.8 | 45,601 | 22,689 | 22,912 | 39,675 | 19,607 | 20,068 | 5,926 | 3,082 | 2,844 |
| 35 - 39 | 10,128 | 4.6 | 46,801 | 23,315 | 23,486 | 40,064 | 19,906 | 20,158 | 6,737 | 3,409 | 3,328 |
| 40 - 44 | 7,540 | 5.4 | 40,472 | 20,081 | 20,391 | 33,245 | 16,418 | 16,827 | 7,227 | 3,663 | 3,564 |
| 45 - 49 | 7,217 | 5.9 | 42,227 | 20,966 | 21,261 | 33,844 | 16,804 | 17,040 | 8,383 | 4,162 | 4,221 |
| 50 - 54 | 7,316 | 6.1 | 44,274 | 22,064 | 22,210 | 32,845 | 16,494 | 16,351 | 11,429 | 5,570 | 5,859 |
| 55 - 59 | 6,153 | 6.2 | 38,205 | 18,870 | 19,335 | 27,326 | 13,561 | 13,765 | 10,879 | 5,309 | 5,570 |
| 60 - 64 | 7,831 | 6.5 | 50,536 | 24,905 | 25,631 | 33,323 | 16,575 | 16,748 | 17,213 | 8,330 | 8,883 |
| 65+ | 23,220 | 6.3 | 145,970 | 71,038 | 74,932 | 87,045 | 41,577 | 45,468 | 58,925 | 29,461 | 29,464 |
| **Primary Level** | | | | | | | | | | | |
| **Total** | **624,705** | **2.5** | **1,565,964** | **778,935** | **787,029** | **1,298,422** | **643,924** | **654,498** | **267,542** | **135,011** | **132,531** |
| 12 - 14 | 165,260 | * | 2,255 | 1,101 | 1,154 | 1,982 | 953 | 1,029 | 273 | 148 | 125 |
| 15 - 19 | 93,231 | 0.2 | 20,532 | 10,198 | 10,334 | 18,883 | 9,321 | 9,562 | 1,649 | 877 | 772 |
| 20 - 24 | 63,058 | 1.6 | 102,088 | 50,902 | 51,186 | 93,160 | 46,182 | 46,978 | 8,928 | 4,720 | 4,208 |
| 25 - 29 | 71,030 | 2.8 | 197,919 | 98,448 | 99,471 | 178,290 | 88,118 | 90,172 | 19,629 | 10,330 | 9,299 |
| 30 - 34 | 58,033 | 3.9 | 223,893 | 111,654 | 112,239 | 198,050 | 97,956 | 100,094 | 25,843 | 13,698 | 12,145 |
| 35 - 39 | 45,783 | 4.8 | 218,593 | 109,170 | 109,423 | 189,962 | 94,305 | 95,657 | 28,631 | 14,865 | 13,766 |
| 40 - 44 | 31,861 | 5.6 | 178,355 | 88,780 | 89,575 | 151,592 | 75,172 | 76,420 | 26,763 | 13,608 | 13,155 |
| 45 - 49 | 28,465 | 6.1 | 172,539 | 86,123 | 86,416 | 143,273 | 71,489 | 71,784 | 29,266 | 14,634 | 14,632 |
| 50 - 54 | 22,770 | 6.4 | 146,613 | 73,057 | 73,556 | 116,158 | 58,118 | 58,040 | 30,455 | 14,939 | 15,516 |
| 55 - 59 | 14,522 | 6.7 | 97,752 | 48,442 | 49,310 | 73,722 | 36,786 | 36,936 | 24,030 | 11,656 | 12,374 |
| 60 - 64 | 11,921 | 6.8 | 80,962 | 40,071 | 40,891 | 55,907 | 28,000 | 27,907 | 25,055 | 12,071 | 12,984 |
| 65+ | 18,771 | 6.6 | 124,463 | 60,989 | 63,474 | 77,443 | 37,524 | 39,919 | 47,020 | 23,465 | 23,555 |

## Table C4: Population of Women 12 Years and Older by Age Group, Average Number of Children Ever Born, Number of Children Ever Born Alive, Children Still Living, Children Dead, Sex of Child and Highest Educational Attainment, Urban, Zambia 2010

| Age Group and Highest Level of Education | Number of Women 12 Years and Older | Average Number of Children Ever Born | Children Ever Born Alive | | | Children Still Living | | | Children Dead | | |
|---|---|---|---|---|---|---|---|---|---|---|---|
| | | | Total | Male | Female | Total | Male | Female | Total | Male | Female |
| **Secondary Level** | | | | | | | | | | | |
| **Total** | **782,247** | **1.7** | **1,338,292** | **663,512** | **674,780** | **1,211,553** | **599,021** | **612,532** | **126,739** | **64,491** | **62,248** |
| 12 - 14 | 31,429 | * | 545 | 256 | 289 | 498 | 226 | 272 | 47 | 30 | 17 |
| 15 - 19 | 223,524 | 0.1 | 30,030 | 14,845 | 15,185 | 28,202 | 13,879 | 14,323 | 1,828 | 966 | 862 |
| 20 - 24 | 178,739 | 0.8 | 146,727 | 73,316 | 73,411 | 138,006 | 68,731 | 69,275 | 8,721 | 4,585 | 4,136 |
| 25 - 29 | 122,251 | 1.9 | 235,251 | 116,968 | 118,283 | 219,771 | 108,841 | 110,930 | 15,480 | 8,127 | 7,353 |
| 30 - 34 | 81,315 | 3.0 | 244,933 | 121,537 | 123,396 | 225,849 | 111,619 | 114,230 | 19,084 | 9,918 | 9,166 |
| 35 - 39 | 56,348 | 3.9 | 217,682 | 107,926 | 109,756 | 198,884 | 98,213 | 100,671 | 18,798 | 9,713 | 9,085 |
| 40 - 44 | 32,432 | 4.6 | 149,811 | 73,722 | 76,089 | 135,134 | 66,236 | 68,898 | 14,677 | 7,486 | 7,191 |
| 45 - 49 | 23,742 | 5.2 | 124,162 | 61,241 | 62,921 | 110,570 | 54,408 | 56,162 | 13,592 | 6,833 | 6,759 |
| 50 - 54 | 15,319 | 5.8 | 88,212 | 43,598 | 44,614 | 75,822 | 37,546 | 38,276 | 12,390 | 6,052 | 6,338 |
| 55 - 59 | 8,309 | 6.0 | 49,756 | 24,689 | 25,067 | 41,016 | 20,531 | 20,485 | 8,740 | 4,158 | 4,582 |
| 60 - 64 | 4,101 | 6.0 | 24,530 | 12,096 | 12,434 | 18,911 | 9,400 | 9,511 | 5,619 | 2,696 | 2,923 |
| 65+ | 4,738 | 5.6 | 26,653 | 13,318 | 13,335 | 18,890 | 9,391 | 9,499 | 7,763 | 3,927 | 3,836 |
| **Tertiary Level** | | | | | | | | | | | |
| **Total** | **183,902** | **2.2** | **399,643** | **199,003** | **200,640** | **366,006** | **181,781** | **184,225** | **33,637** | **17,222** | **16,415** |
| 12 - 14 | - | - | - | - | - | - | - | - | - | - | - |
| 15 - 19 | 2,802 | 0.1 | 370 | 191 | 179 | 339 | 172 | 167 | 31 | 19 | 12 |
| 20 - 24 | 27,574 | 0.4 | 11,776 | 5,888 | 5,888 | 11,226 | 5,595 | 5,631 | 550 | 293 | 257 |
| 25 - 29 | 44,711 | 1.1 | 48,183 | 24,153 | 24,030 | 45,875 | 22,952 | 22,923 | 2,308 | 1,201 | 1,107 |
| 30 - 34 | 35,237 | 1.9 | 68,497 | 34,172 | 34,325 | 64,818 | 32,186 | 32,632 | 3,679 | 1,986 | 1,693 |
| 35 - 39 | 24,273 | 2.7 | 65,588 | 32,624 | 32,964 | 61,383 | 30,435 | 30,948 | 4,205 | 2,189 | 2,016 |
| 40 - 44 | 15,578 | 3.3 | 51,753 | 25,620 | 26,133 | 47,910 | 23,595 | 24,315 | 3,843 | 2,025 | 1,818 |
| 45 - 49 | 11,767 | 3.9 | 46,038 | 22,840 | 23,198 | 42,303 | 20,932 | 21,371 | 3,735 | 1,908 | 1,827 |
| 50 - 54 | 9,626 | 4.6 | 44,158 | 21,932 | 22,226 | 39,911 | 19,821 | 20,090 | 4,247 | 2,111 | 2,136 |
| 55 - 59 | 6,521 | 4.9 | 32,223 | 16,202 | 16,021 | 28,078 | 14,146 | 13,932 | 4,145 | 2,056 | 2,089 |
| 60 - 64 | 3,326 | 5.3 | 17,702 | 8,754 | 8,948 | 14,390 | 7,146 | 7,244 | 3,312 | 1,608 | 1,704 |
| 65+ | 2,487 | 5.4 | 13,355 | 6,627 | 6,728 | 9,773 | 4,801 | 4,972 | 3,582 | 1,826 | 1,756 |

## Table C4i: Population of Women 12-49 Years by Age Group, Number of Children Born Alive 12 Months Prior to the Census, Children Still Living, Children Dead, Sex of Child and Educational Attainment, Rural/Urban, Zambia 2010

| Age Group and Highest Level of Education | Number of Women 12 Years and Older | Children Born 12 Months Prior to the Census | | | Children Still Living | | | Children Dead | | |
|---|---|---|---|---|---|---|---|---|---|---|
| | | Total | Male | Female | Total | Male | Female | Total | Male | Female |
| **Zambia Total** | | | | | | | | | | |
| **Total** | **3,488,895** | **442,998** | **224,756** | **218,242** | **423,116** | **214,441** | **208,675** | **19,882** | **10,315** | **9,567** |
| 12 - 14 | 486,104 | 921 | 470 | 451 | 870 | 447 | 423 | 51 | 23 | 28 |
| 15 - 19 | 735,025 | 58,999 | 29,701 | 29,298 | 56,523 | 28,406 | 28,117 | 2,476 | 1,295 | 1,181 |
| 20 - 24 | 612,600 | 128,270 | 65,048 | 63,222 | 123,381 | 62,419 | 60,962 | 4,889 | 2,629 | 2,260 |
| 25 - 29 | 541,751 | 114,701 | 58,366 | 56,335 | 109,784 | 55,861 | 53,923 | 4,917 | 2,505 | 2,412 |
| 30 - 34 | 403,076 | 74,066 | 37,666 | 36,400 | 70,501 | 35,842 | 34,659 | 3,565 | 1,824 | 1,741 |
| 35 - 39 | 314,852 | 45,452 | 23,072 | 22,380 | 42,958 | 21,789 | 21,169 | 2,494 | 1,283 | 1,211 |
| 40 - 44 | 215,331 | 16,150 | 8,181 | 7,969 | 15,087 | 7,635 | 7,452 | 1,063 | 546 | 517 |
| 45 - 49 | 180,156 | 4,439 | 2,252 | 2,187 | 4,012 | 2,042 | 1,970 | 427 | 210 | 217 |
| **Never Attended** | | | | | | | | | | |
| **Total** | **532,316** | **89,850** | **45,596** | **44,254** | **85,545** | **43,383** | **42,162** | **4,305** | **2,213** | **2,092** |
| 12 - 14 | 41,432 | 184 | 97 | 87 | 171 | 91 | 80 | 13 | 6 | 7 |
| 15 - 19 | 70,114 | 9,621 | 4,819 | 4,802 | 9,163 | 4,581 | 4,582 | 458 | 238 | 220 |
| 20 - 24 | 94,326 | 24,150 | 12,286 | 11,864 | 23,176 | 11,777 | 11,399 | 974 | 509 | 465 |
| 25 - 29 | 98,807 | 24,154 | 12,337 | 11,817 | 23,042 | 11,755 | 11,287 | 1,112 | 582 | 530 |
| 30 - 34 | 73,562 | 15,340 | 7,788 | 7,552 | 14,583 | 7,418 | 7,165 | 757 | 370 | 387 |
| 35 - 39 | 63,011 | 10,741 | 5,400 | 5,341 | 10,173 | 5,109 | 5,064 | 568 | 291 | 277 |
| 40 - 44 | 47,836 | 4,256 | 2,177 | 2,079 | 3,971 | 2,024 | 1,947 | 285 | 153 | 132 |
| 45 - 49 | 43,228 | 1,404 | 692 | 712 | 1,266 | 628 | 638 | 138 | 64 | 74 |
| **Primary Level** | | | | | | | | | | |
| **Total** | **1,652,179** | **210,096** | **106,510** | **103,586** | **200,094** | **101,220** | **98,874** | **10,002** | **5,290** | **4,712** |
| 12 - 14 | 403,162 | 616 | 313 | 303 | 583 | 298 | 285 | 33 | 15 | 18 |
| 15 - 19 | 317,116 | 29,552 | 14,836 | 14,716 | 28,182 | 14,099 | 14,083 | 1,370 | 737 | 633 |
| 20 - 24 | 214,871 | 56,824 | 28,666 | 28,158 | 54,514 | 27,400 | 27,114 | 2,310 | 1,266 | 1,044 |
| 25 - 29 | 215,864 | 52,603 | 26,889 | 25,714 | 50,276 | 25,687 | 24,589 | 2,327 | 1,202 | 1,125 |
| 30 - 34 | 171,082 | 35,622 | 18,081 | 17,541 | 33,836 | 17,142 | 16,694 | 1,786 | 939 | 847 |
| 35 - 39 | 141,829 | 23,635 | 12,035 | 11,600 | 22,290 | 11,346 | 10,944 | 1,345 | 689 | 656 |
| 40 - 44 | 101,051 | 8,947 | 4,505 | 4,442 | 8,347 | 4,188 | 4,159 | 600 | 317 | 283 |
| 45 - 49 | 87,204 | 2,297 | 1,185 | 1,112 | 2,066 | 1,060 | 1,006 | 231 | 125 | 106 |
| **Secondary Level** | | | | | | | | | | |
| **Total** | **1,114,254** | **122,640** | **62,302** | **60,338** | **117,826** | **59,845** | **57,981** | **4,814** | **2,457** | **2,357** |
| 12 - 14 | 41,510 | 121 | 60 | 61 | 116 | 58 | 58 | 5 | 2 | 3 |
| 15 - 19 | 344,096 | 19,649 | 9,959 | 9,690 | 19,009 | 9,642 | 9,367 | 640 | 317 | 323 |
| 20 - 24 | 271,859 | 44,171 | 22,486 | 21,685 | 42,675 | 21,690 | 20,985 | 1,496 | 796 | 700 |
| 25 - 29 | 174,263 | 29,893 | 15,074 | 14,819 | 28,691 | 14,478 | 14,213 | 1,202 | 596 | 606 |
| 30 - 34 | 116,837 | 17,335 | 8,889 | 8,446 | 16,514 | 8,466 | 8,048 | 821 | 423 | 398 |
| 35 - 39 | 81,756 | 8,539 | 4,332 | 4,207 | 8,076 | 4,085 | 3,991 | 463 | 247 | 216 |
| 40 - 44 | 48,289 | 2,329 | 1,190 | 1,139 | 2,189 | 1,130 | 1,059 | 140 | 60 | 80 |
| 45 - 49 | 35,644 | 603 | 312 | 291 | 556 | 296 | 260 | 47 | 16 | 31 |
| **Tertiary Level** | | | | | | | | | | |
| **Total** | **190,146** | **20,412** | **10,348** | **10,064** | **19,651** | **9,993** | **9,658** | **761** | **355** | **406** |
| 12 - 14 | - | - | - | - | - | - | - | - | - | - |
| 15 - 19 | 3,699 | 177 | 87 | 90 | 169 | 84 | 85 | 8 | 3 | 5 |
| 20 - 24 | 31,544 | 3,125 | 1,610 | 1,515 | 3,016 | 1,552 | 1,464 | 109 | 58 | 51 |
| 25 - 29 | 52,817 | 8,051 | 4,066 | 3,985 | 7,775 | 3,941 | 3,834 | 276 | 125 | 151 |
| 30 - 34 | 41,595 | 5,769 | 2,908 | 2,861 | 5,568 | 2,816 | 2,752 | 201 | 92 | 109 |
| 35 - 39 | 28,256 | 2,537 | 1,305 | 1,232 | 2,419 | 1,249 | 1,170 | 118 | 56 | 62 |
| 40 - 44 | 18,155 | 618 | 309 | 309 | 580 | 293 | 287 | 38 | 16 | 22 |
| 45 - 49 | 14,080 | 135 | 63 | 72 | 124 | 58 | 66 | 11 | 5 | 6 |

## Table C4i: Population of Women 12-49 Years by Age Group, Number of Children Born Alive 12 Months Prior to the Census, Children Still Living, Children Dead, Sex of Child and Educational Attainment, Rural, Zambia 2010

| Age Group and Highest Level of Education | Number of Women 12 Years and Older | Children Born 12 Months Prior to the Census | | | Children Still Living | | | Children Dead | | |
|---|---|---|---|---|---|---|---|---|---|---|
| | | Total | Male | Female | Total | Male | Female | Total | Male | Female |
| **Zambia Rural** | | | | | | | | | | |
| **Total** | **1,936,788** | **294,803** | **149,436** | **145,367** | **281,341** | **142,357** | **138,984** | **13,462** | **7,079** | **6,383** |
| 12 - 14 | 282,652 | 672 | 342 | 330 | 630 | 323 | 307 | 42 | 19 | 23 |
| 15 - 19 | 405,259 | 41,899 | 21,054 | 20,845 | 40,055 | 20,075 | 19,980 | 1,844 | 979 | 865 |
| 20 - 24 | 328,902 | 84,736 | 42,823 | 41,913 | 81,446 | 41,041 | 40,405 | 3,290 | 1,782 | 1,508 |
| 25 - 29 | 288,345 | 72,639 | 37,041 | 35,598 | 69,506 | 35,413 | 34,093 | 3,133 | 1,628 | 1,505 |
| 30 - 34 | 216,425 | 47,489 | 24,077 | 23,412 | 45,182 | 22,876 | 22,306 | 2,307 | 1,201 | 1,106 |
| 35 - 39 | 178,320 | 31,641 | 16,135 | 15,506 | 29,939 | 15,251 | 14,688 | 1,702 | 884 | 818 |
| 40 - 44 | 127,920 | 12,286 | 6,213 | 6,073 | 11,479 | 5,802 | 5,677 | 807 | 411 | 396 |
| 45 - 49 | 108,965 | 3,441 | 1,751 | 1,690 | 3,104 | 1,576 | 1,528 | 337 | 175 | 162 |
| **Never Attended** | | | | | | | | | | |
| **Total** | **448,652** | **79,357** | **40,265** | **39,092** | **75,550** | **38,290** | **37,260** | **3,807** | **1,975** | **1,832** |
| 12 - 14 | 34,669 | 170 | 88 | 82 | 157 | 82 | 75 | 13 | 6 | 7 |
| 15 - 19 | 59,905 | 8,621 | 4,312 | 4,309 | 8,201 | 4,092 | 4,109 | 420 | 220 | 200 |
| 20 - 24 | 79,999 | 21,281 | 10,792 | 10,489 | 20,427 | 10,339 | 10,088 | 854 | 453 | 401 |
| 25 - 29 | 83,393 | 21,330 | 10,891 | 10,439 | 20,336 | 10,362 | 9,974 | 994 | 529 | 465 |
| 30 - 34 | 61,496 | 13,410 | 6,808 | 6,602 | 12,749 | 6,483 | 6,266 | 661 | 325 | 336 |
| 35 - 39 | 52,883 | 9,460 | 4,793 | 4,667 | 8,973 | 4,540 | 4,433 | 487 | 253 | 234 |
| 40 - 44 | 40,296 | 3,835 | 1,968 | 1,867 | 3,586 | 1,839 | 1,747 | 249 | 129 | 120 |
| 45 - 49 | 36,011 | 1,250 | 613 | 637 | 1,121 | 553 | 568 | 129 | 60 | 69 |
| **Primary Level** | | | | | | | | | | |
| **Total** | **1,095,458** | **159,645** | **80,926** | **78,719** | **152,188** | **76,947** | **75,241** | **7,457** | **3,979** | **3,478** |
| 12 - 14 | 237,902 | 436 | 221 | 215 | 409 | 208 | 201 | 27 | 13 | 14 |
| 15 - 19 | 223,885 | 23,304 | 11,687 | 11,617 | 22,205 | 11,091 | 11,114 | 1,099 | 596 | 503 |
| 20 - 24 | 151,813 | 43,561 | 21,975 | 21,586 | 41,814 | 21,014 | 20,800 | 1,747 | 961 | 786 |
| 25 - 29 | 144,834 | 38,895 | 19,939 | 18,956 | 37,265 | 19,090 | 18,175 | 1,630 | 849 | 781 |
| 30 - 34 | 113,049 | 26,285 | 13,310 | 12,975 | 24,996 | 12,622 | 12,374 | 1,289 | 688 | 601 |
| 35 - 39 | 96,046 | 18,123 | 9,242 | 8,881 | 17,122 | 8,725 | 8,397 | 1,001 | 517 | 484 |
| 40 - 44 | 69,190 | 7,179 | 3,582 | 3,597 | 6,700 | 3,334 | 3,366 | 479 | 248 | 231 |
| 45 - 49 | 58,739 | 1,862 | 970 | 892 | 1,677 | 863 | 814 | 185 | 107 | 78 |
| **Secondary Level** | | | | | | | | | | |
| **Total** | **364,474** | **52,035** | **26,300** | **25,735** | **50,009** | **25,257** | **24,752** | **2,026** | **1,043** | **983** |
| 12 - 14 | 10,081 | 66 | 33 | 33 | 64 | 33 | 31 | 2 | - | 2 |
| 15 - 19 | 120,572 | 9,901 | 5,019 | 4,882 | 9,576 | 4,856 | 4,720 | 325 | 163 | 162 |
| 20 - 24 | 93,120 | 19,302 | 9,731 | 9,571 | 18,641 | 9,378 | 9,263 | 661 | 353 | 308 |
| 25 - 29 | 52,012 | 10,966 | 5,485 | 5,481 | 10,523 | 5,263 | 5,260 | 443 | 222 | 221 |
| 30 - 34 | 35,522 | 6,721 | 3,403 | 3,318 | 6,407 | 3,241 | 3,166 | 314 | 162 | 152 |
| 35 - 39 | 25,408 | 3,636 | 1,879 | 1,757 | 3,442 | 1,773 | 1,669 | 194 | 106 | 88 |
| 40 - 44 | 15,857 | 1,147 | 599 | 548 | 1,081 | 570 | 511 | 66 | 29 | 37 |
| 45 - 49 | 11,902 | 296 | 151 | 145 | 275 | 143 | 132 | 21 | 8 | 13 |
| **Tertiary Level** | | | | | | | | | | |
| **Total** | **28,204** | **3,766** | **1,945** | **1,821** | **3,594** | **1,863** | **1,731** | **172** | **82** | **90** |
| 12 - 14 | - | - | - | - | - | - | - | - | - | - |
| 15 - 19 | 897 | 73 | 36 | 37 | 73 | 36 | 37 | - | - | - |
| 20 - 24 | 3,970 | 592 | 325 | 267 | 564 | 310 | 254 | 28 | 15 | 13 |
| 25 - 29 | 8,106 | 1,448 | 726 | 722 | 1,382 | 698 | 684 | 66 | 28 | 38 |
| 30 - 34 | 6,358 | 1,073 | 556 | 517 | 1,030 | 530 | 500 | 43 | 26 | 17 |
| 35 - 39 | 3,983 | 422 | 221 | 201 | 402 | 213 | 189 | 20 | 8 | 12 |
| 40 - 44 | 2,577 | 125 | 64 | 61 | 112 | 59 | 53 | 13 | 5 | 8 |
| 45 - 49 | 2,313 | 33 | 17 | 16 | 31 | 17 | 14 | 2 | - | 2 |

## Table C4i: Population of Women 12-49 Years by Age Group, Number of Children Born Alive 12 Months Prior to the Census, Children Still Living, Children Dead, Sex of Child and Educational Attainment, Urban, Zambia 2010

| Age Group and Highest Level of Education | Number of Women 12 Years and Older | Children Born 12 Months Prior to the Census | | | Children Still Living | | | Children Dead | | |
|---|---|---|---|---|---|---|---|---|---|---|
| | | Total | Male | Female | Total | Male | Female | Total | Male | Female |
| **Zambia Urban** | | | | | | | | | | |
| **Total** | **1,552,107** | **148,195** | **75,320** | **72,875** | **141,775** | **72,084** | **69,691** | **6,420** | **3,236** | **3,184** |
| 12 - 14 | 203,452 | 249 | 128 | 121 | 240 | 124 | 116 | 9 | 4 | 5 |
| 15 - 19 | 329,766 | 17,100 | 8,647 | 8,453 | 16,468 | 8,331 | 8,137 | 632 | 316 | 316 |
| 20 - 24 | 283,698 | 43,534 | 22,225 | 21,309 | 41,935 | 21,378 | 20,557 | 1,599 | 847 | 752 |
| 25 - 29 | 253,406 | 42,062 | 21,325 | 20,737 | 40,278 | 20,448 | 19,830 | 1,784 | 877 | 907 |
| 30 - 34 | 186,651 | 26,577 | 13,589 | 12,988 | 25,319 | 12,966 | 12,353 | 1,258 | 623 | 635 |
| 35 - 39 | 136,532 | 13,811 | 6,937 | 6,874 | 13,019 | 6,538 | 6,481 | 792 | 399 | 393 |
| 40 - 44 | 87,411 | 3,864 | 1,968 | 1,896 | 3,608 | 1,833 | 1,775 | 256 | 135 | 121 |
| 45 - 49 | 71,191 | 998 | 501 | 497 | 908 | 466 | 442 | 90 | 35 | 55 |
| **None** | | | | | | | | | | |
| **Total** | **83,664** | **10,493** | **5,331** | **5,162** | **9,995** | **5,093** | **4,902** | **498** | **238** | **260** |
| 12 - 14 | 6,763 | 14 | 9 | 5 | 14 | 9 | 5 | 0 | 0 | 0 |
| 15 - 19 | 10,209 | 1,000 | 507 | 493 | 962 | 489 | 473 | 38 | 18 | 20 |
| 20 - 24 | 14,327 | 2,869 | 1,494 | 1,375 | 2,749 | 1,438 | 1,311 | 120 | 56 | 64 |
| 25 - 29 | 15,414 | 2,824 | 1,446 | 1,378 | 2,706 | 1,393 | 1,313 | 118 | 53 | 65 |
| 30 - 34 | 12,066 | 1,930 | 980 | 950 | 1,834 | 935 | 899 | 96 | 45 | 51 |
| 35 - 39 | 10,128 | 1,281 | 607 | 674 | 1,200 | 569 | 631 | 81 | 38 | 43 |
| 40 - 44 | 7,540 | 421 | 209 | 212 | 385 | 185 | 200 | 36 | 24 | 12 |
| 45 - 49 | 7,217 | 154 | 79 | 75 | 145 | 75 | 70 | 9 | 4 | 5 |
| **Primary Level** | | | | | | | | | | |
| **Total** | **556,721** | **50,451** | **25,584** | **24,867** | **47,906** | **24,273** | **23,633** | **2,545** | **1,311** | **1,234** |
| 12 - 14 | 165,260 | 180 | 92 | 88 | 174 | 90 | 84 | 6 | 2 | 4 |
| 15 - 19 | 93,231 | 6,248 | 3,149 | 3,099 | 5,977 | 3,008 | 2,969 | 271 | 141 | 130 |
| 20 - 24 | 63,058 | 13,263 | 6,691 | 6,572 | 12,700 | 6,386 | 6,314 | 563 | 305 | 258 |
| 25 - 29 | 71,030 | 13,708 | 6,950 | 6,758 | 13,011 | 6,597 | 6,414 | 697 | 353 | 344 |
| 30 - 34 | 58,033 | 9,337 | 4,771 | 4,566 | 8,840 | 4,520 | 4,320 | 497 | 251 | 246 |
| 35 - 39 | 45,783 | 5,512 | 2,793 | 2,719 | 5,168 | 2,621 | 2,547 | 344 | 172 | 172 |
| 40 - 44 | 31,861 | 1,768 | 923 | 845 | 1,647 | 854 | 793 | 121 | 69 | 52 |
| 45 - 49 | 28,465 | 435 | 215 | 220 | 389 | 197 | 192 | 46 | 18 | 28 |
| **Secondary Level** | | | | | | | | | | |
| **Total** | **749,780** | **70,605** | **36,002** | **34,603** | **67,817** | **34,588** | **33,229** | **2,788** | **1,414** | **1,374** |
| 12 - 14 | 31,429 | 55 | 27 | 28 | 52 | 25 | 27 | 3 | 2 | 1 |
| 15 - 19 | 223,524 | 9,748 | 4,940 | 4,808 | 9,433 | 4,786 | 4,647 | 315 | 154 | 161 |
| 20 - 24 | 178,739 | 24,869 | 12,755 | 12,114 | 24,034 | 12,312 | 11,722 | 835 | 443 | 392 |
| 25 - 29 | 122,251 | 18,927 | 9,589 | 9,338 | 18,168 | 9,215 | 8,953 | 759 | 374 | 385 |
| 30 - 34 | 81,315 | 10,614 | 5,486 | 5,128 | 10,107 | 5,225 | 4,882 | 507 | 261 | 246 |
| 35 - 39 | 56,348 | 4,903 | 2,453 | 2,450 | 4,634 | 2,312 | 2,322 | 269 | 141 | 128 |
| 40 - 44 | 32,432 | 1,182 | 591 | 591 | 1,108 | 560 | 548 | 74 | 31 | 43 |
| 45 - 49 | 23,742 | 307 | 161 | 146 | 281 | 153 | 128 | 26 | 8 | 18 |
| **Tertiary Level** | | | | | | | | | | |
| **Total** | **161,942** | **16,646** | **8,403** | **8,243** | **16,057** | **8,130** | **7,927** | **589** | **273** | **316** |
| 12 - 14 | - | - | - | - | - | - | - | - | - | - |
| 15 - 19 | 2,802 | 104 | 51 | 53 | 96 | 48 | 48 | 8 | 3 | 5 |
| 20 - 24 | 27,574 | 2,533 | 1,285 | 1,248 | 2,452 | 1,242 | 1,210 | 81 | 43 | 38 |
| 25 - 29 | 44,711 | 6,603 | 3,340 | 3,263 | 6,393 | 3,243 | 3,150 | 210 | 97 | 113 |
| 30 - 34 | 35,237 | 4,696 | 2,352 | 2,344 | 4,538 | 2,286 | 2,252 | 158 | 66 | 92 |
| 35 - 39 | 24,273 | 2,115 | 1,084 | 1,031 | 2,017 | 1,036 | 981 | 98 | 48 | 50 |
| 40 - 44 | 15,578 | 493 | 245 | 248 | 468 | 234 | 234 | 25 | 11 | 14 |
| 45 - 49 | 11,767 | 102 | 46 | 56 | 93 | 41 | 52 | 9 | 5 | 4 |

## Table C5: Population of Women 12 years and Older by Age Group, Number of Children Ever Born Alive, Children Still Living, Children Dead, Sex of Child and Economic Activity Status, Rural/Urban, Zambia 2010

| Age Group and Economic Activity Status | Number of Women 12 Years and Older | Children Ever Born Alive | | | Children Still Living | | | Children Dead | | |
|---|---|---|---|---|---|---|---|---|---|---|
| | | Total | Male | Female | Total | Male | Female | Total | Male | Female |
| **Zambia Total** | | | | | | | | | | |
| **Total** | **3,992,401** | **10,970,814** | **5,464,308** | **5,506,506** | **9,000,504** | **4,469,438** | **4,531,066** | **1,970,310** | **994,870** | **975,440** |
| 12 - 14 | 486,104 | 7,947 | 4,000 | 3,947 | 6,924 | 3,431 | 3,493 | 1,023 | 569 | 454 |
| 15 - 19 | 735,025 | 175,811 | 87,622 | 88,189 | 161,182 | 79,880 | 81,302 | 14,629 | 7,742 | 6,887 |
| 20 - 24 | 612,600 | 822,610 | 409,373 | 413,237 | 752,608 | 372,507 | 380,101 | 70,002 | 36,866 | 33,136 |
| 25 - 29 | 541,751 | 1,419,661 | 706,979 | 712,682 | 1,279,281 | 633,721 | 645,560 | 140,380 | 73,258 | 67,122 |
| 30 - 34 | 403,076 | 1,553,530 | 774,823 | 778,707 | 1,371,087 | 680,133 | 690,954 | 182,443 | 94,690 | 87,753 |
| 35 - 39 | 314,852 | 1,525,804 | 763,380 | 762,424 | 1,319,083 | 657,096 | 661,987 | 206,721 | 106,284 | 100,437 |
| 40 - 44 | 215,331 | 1,210,821 | 606,130 | 604,691 | 1,013,909 | 505,437 | 508,472 | 196,912 | 100,693 | 96,219 |
| 45 - 49 | 180,156 | 1,080,764 | 540,626 | 540,138 | 881,305 | 440,076 | 441,229 | 199,459 | 100,550 | 98,909 |
| 50 - 54 | 141,558 | 879,792 | 439,039 | 440,753 | 683,742 | 342,048 | 341,694 | 196,050 | 96,991 | 99,059 |
| 55 - 59 | 94,791 | 603,169 | 299,663 | 303,506 | 449,826 | 224,820 | 225,006 | 153,343 | 74,843 | 78,500 |
| 60 - 64 | 88,456 | 573,756 | 284,473 | 289,283 | 393,365 | 196,162 | 197,203 | 180,391 | 88,311 | 92,080 |
| 65+ | 178,701 | 1,117,149 | 548,200 | 568,949 | 688,192 | 334,127 | 354,065 | 428,957 | 214,073 | 214,884 |
| **Economically Active** | | | | | | | | | | |
| **Total** | **1,973,795** | **6,958,301** | **3,465,372** | **3,492,929** | **5,694,233** | **2,826,735** | **2,867,498** | **1,264,068** | **638,637** | **625,431** |
| 12 - 14 | 67,434 | 2,056 | 1,073 | 983 | 1,796 | 923 | 873 | 260 | 150 | 110 |
| 15 - 19 | 206,994 | 91,702 | 45,713 | 45,989 | 83,657 | 41,513 | 42,144 | 8,045 | 4,200 | 3,845 |
| 20 - 24 | 326,392 | 473,247 | 235,060 | 238,187 | 430,175 | 212,346 | 217,829 | 43,072 | 22,714 | 20,358 |
| 25 - 29 | 329,156 | 866,879 | 431,192 | 435,687 | 775,908 | 383,822 | 392,086 | 90,971 | 47,370 | 43,601 |
| 30 - 34 | 261,589 | 1,009,056 | 502,620 | 506,436 | 884,032 | 437,916 | 446,116 | 125,024 | 64,704 | 60,320 |
| 35 - 39 | 212,281 | 1,030,120 | 515,015 | 515,105 | 883,459 | 439,511 | 443,948 | 146,661 | 75,504 | 71,157 |
| 40 - 44 | 148,555 | 837,371 | 419,268 | 418,103 | 694,659 | 346,072 | 348,587 | 142,712 | 73,196 | 69,516 |
| 45 - 49 | 124,787 | 751,469 | 375,687 | 375,782 | 606,491 | 302,425 | 304,066 | 144,978 | 73,262 | 71,716 |
| 50 - 54 | 95,316 | 597,044 | 297,337 | 299,707 | 458,851 | 228,951 | 229,900 | 138,193 | 68,386 | 69,807 |
| 55 - 59 | 62,119 | 400,505 | 199,120 | 201,385 | 295,213 | 147,444 | 147,769 | 105,292 | 51,676 | 53,616 |
| 60 - 64 | 54,997 | 362,890 | 179,757 | 183,133 | 247,411 | 123,229 | 124,182 | 115,479 | 56,528 | 58,951 |
| 65+ | 84,175 | 535,962 | 263,530 | 272,432 | 332,581 | 162,583 | 169,998 | 203,381 | 100,947 | 102,434 |
| **Economically inactive** | | | | | | | | | | |
| **Total** | **2,018,606** | **4,012,513** | **1,998,936** | **2,013,577** | **3,306,271** | **1,642,703** | **1,663,568** | **706,242** | **356,233** | **350,009** |
| 12 - 14 | 418,670 | 5,891 | 2,927 | 2,964 | 5,128 | 2,508 | 2,620 | 763 | 419 | 344 |
| 15 - 19 | 528,031 | 84,109 | 41,909 | 42,200 | 77,525 | 38,367 | 39,158 | 6,584 | 3,542 | 3,042 |
| 20 - 24 | 286,208 | 349,363 | 174,313 | 175,050 | 322,433 | 160,161 | 162,272 | 26,930 | 14,152 | 12,778 |
| 25 - 29 | 212,595 | 552,782 | 275,787 | 276,995 | 503,373 | 249,899 | 253,474 | 49,409 | 25,888 | 23,521 |
| 30 - 34 | 141,487 | 544,474 | 272,203 | 272,271 | 487,055 | 242,217 | 244,838 | 57,419 | 29,986 | 27,433 |
| 35 - 39 | 102,571 | 495,684 | 248,365 | 247,319 | 435,624 | 217,585 | 218,039 | 60,060 | 30,780 | 29,280 |
| 40 - 44 | 66,776 | 373,450 | 186,862 | 186,588 | 319,250 | 159,365 | 159,885 | 54,200 | 27,497 | 26,703 |
| 45 - 49 | 55,369 | 329,295 | 164,939 | 164,356 | 274,814 | 137,651 | 137,163 | 54,481 | 27,288 | 27,193 |
| 50 - 54 | 46,242 | 282,748 | 141,702 | 141,046 | 224,891 | 113,097 | 111,794 | 57,857 | 28,605 | 29,252 |
| 55 - 59 | 32,672 | 202,664 | 100,543 | 102,121 | 154,613 | 77,376 | 77,237 | 48,051 | 23,167 | 24,884 |
| 60 - 64 | 33,459 | 210,866 | 104,716 | 106,150 | 145,954 | 72,933 | 73,021 | 64,912 | 31,783 | 33,129 |
| 65+ | 94,526 | 581,187 | 284,670 | 296,517 | 355,611 | 171,544 | 184,067 | 225,576 | 113,126 | 112,450 |

## Table C5: Population of Women 12 years and Older by Age Group, Number of Children Ever Born Alive, Children Still Living, Children Dead, Sex of Child and Economic Activity Status, Rural, Zambia 2010

| Age Group and Economic Activity Status | Number of Women 12 Years and Older | Children Ever Born Alive | | | Children Still Living | | | Children Dead | | |
|---|---|---|---|---|---|---|---|---|---|---|
| | | Total | Male | Female | Total | Male | Female | Total | Male | Female |
| **Zambia Rural** | | | | | | | | | | |
| **Total** | **2,273,363** | **7,145,714** | **3,565,538** | **3,580,176** | **5,736,895** | **2,853,935** | **2,882,960** | **1,408,819** | **711,603** | **697,216** |
| 12 - 14 | 282,652 | 4,987 | 2,570 | 2,417 | 4,307 | 2,190 | 2,117 | 680 | 380 | 300 |
| 15 - 19 | 405,259 | 121,237 | 60,581 | 60,656 | 110,453 | 54,881 | 55,572 | 10,784 | 5,700 | 5,084 |
| 20 - 24 | 328,902 | 539,787 | 268,274 | 271,513 | 490,061 | 242,063 | 247,998 | 49,726 | 26,211 | 23,515 |
| 25 - 29 | 288,345 | 897,227 | 446,891 | 450,336 | 798,681 | 395,600 | 403,081 | 98,546 | 51,291 | 47,255 |
| 30 - 34 | 216,425 | 970,606 | 484,771 | 485,835 | 842,695 | 418,765 | 423,930 | 127,911 | 66,006 | 61,905 |
| 35 - 39 | 178,320 | 977,140 | 490,345 | 486,795 | 828,790 | 414,237 | 414,553 | 148,350 | 76,108 | 72,242 |
| 40 - 44 | 127,920 | 790,430 | 397,927 | 392,503 | 646,028 | 324,016 | 322,012 | 144,402 | 73,911 | 70,491 |
| 45 - 49 | 108,965 | 695,798 | 349,456 | 346,342 | 551,315 | 276,443 | 274,872 | 144,483 | 73,013 | 71,470 |
| 50 - 54 | 86,527 | 556,535 | 278,388 | 278,147 | 419,006 | 210,069 | 208,937 | 137,529 | 68,319 | 69,210 |
| 55 - 59 | 59,286 | 385,233 | 191,460 | 193,773 | 279,684 | 139,796 | 139,888 | 105,549 | 51,664 | 53,885 |
| 60 - 64 | 61,277 | 400,026 | 198,647 | 201,379 | 270,834 | 135,041 | 135,793 | 129,192 | 63,606 | 65,586 |
| 65+ | 129,485 | 806,708 | 396,228 | 410,480 | 495,041 | 240,834 | 254,207 | 311,667 | 155,394 | 156,273 |
| **Economically Active** | | | | | | | | | | |
| **Total** | **1,351,536** | **5,219,508** | **2,605,023** | **2,614,485** | **4,201,013** | **2,090,407** | **2,110,606** | **1,018,495** | **514,616** | **503,879** |
| 12 - 14 | 59,232 | 1,784 | 941 | 843 | 1,552 | 809 | 743 | 232 | 132 | 100 |
| 15 - 19 | 163,252 | 77,730 | 38,770 | 38,960 | 70,718 | 35,110 | 35,608 | 7,012 | 3,660 | 3,352 |
| 20 - 24 | 217,747 | 377,617 | 187,437 | 190,180 | 341,889 | 168,599 | 173,290 | 35,728 | 18,838 | 16,890 |
| 25 - 29 | 205,677 | 649,045 | 323,273 | 325,772 | 576,437 | 285,427 | 291,010 | 72,608 | 37,846 | 34,762 |
| 30 - 34 | 159,817 | 723,188 | 361,013 | 362,175 | 626,046 | 310,926 | 315,120 | 97,142 | 50,087 | 47,055 |
| 35 - 39 | 134,296 | 741,764 | 372,276 | 369,488 | 627,000 | 313,358 | 313,642 | 114,764 | 58,918 | 55,846 |
| 40 - 44 | 97,966 | 610,411 | 307,273 | 303,138 | 497,055 | 249,152 | 247,903 | 113,356 | 58,121 | 55,235 |
| 45 - 49 | 84,500 | 544,224 | 273,371 | 270,853 | 429,720 | 215,380 | 214,340 | 114,504 | 57,991 | 56,513 |
| 50 - 54 | 66,565 | 433,763 | 216,693 | 217,070 | 325,418 | 162,837 | 162,581 | 108,345 | 53,856 | 54,489 |
| 55 - 59 | 45,515 | 300,250 | 149,443 | 150,807 | 217,560 | 108,785 | 108,775 | 82,690 | 40,658 | 42,032 |
| 60 - 64 | 44,677 | 297,212 | 147,276 | 149,936 | 201,196 | 100,172 | 101,024 | 96,016 | 47,104 | 48,912 |
| 65+ | 72,292 | 462,520 | 227,257 | 235,263 | 286,422 | 139,852 | 146,570 | 176,098 | 87,405 | 88,693 |
| **Economically inactive** | | | | | | | | | | |
| **Total** | **921,827** | **1,926,206** | **960,515** | **965,691** | **1,535,882** | **763,528** | **772,354** | **390,324** | **196,987** | **193,337** |
| 12 - 14 | 223,420 | 3,203 | 1,629 | 1,574 | 2,755 | 1,381 | 1,374 | 448 | 248 | 200 |
| 15 - 19 | 242,007 | 43,507 | 21,811 | 21,696 | 39,735 | 19,771 | 19,964 | 3,772 | 2,040 | 1,732 |
| 20 - 24 | 111,155 | 162,170 | 80,837 | 81,333 | 148,172 | 73,464 | 74,708 | 13,998 | 7,373 | 6,625 |
| 25 - 29 | 82,668 | 248,182 | 123,618 | 124,564 | 222,244 | 110,173 | 112,071 | 25,938 | 13,445 | 12,493 |
| 30 - 34 | 56,608 | 247,418 | 123,758 | 123,660 | 216,649 | 107,839 | 108,810 | 30,769 | 15,919 | 14,850 |
| 35 - 39 | 44,024 | 235,376 | 118,069 | 117,307 | 201,790 | 100,879 | 100,911 | 33,586 | 17,190 | 16,396 |
| 40 - 44 | 29,954 | 180,019 | 90,654 | 89,365 | 148,973 | 74,864 | 74,109 | 31,046 | 15,790 | 15,256 |
| 45 - 49 | 24,465 | 151,574 | 76,085 | 75,489 | 121,595 | 61,063 | 60,532 | 29,979 | 15,022 | 14,957 |
| 50 - 54 | 19,962 | 122,772 | 61,695 | 61,077 | 93,588 | 47,232 | 46,356 | 29,184 | 14,463 | 14,721 |
| 55 - 59 | 13,771 | 84,983 | 42,017 | 42,966 | 62,124 | 31,011 | 31,113 | 22,859 | 11,006 | 11,853 |
| 60 - 64 | 16,600 | 102,814 | 51,371 | 51,443 | 69,638 | 34,869 | 34,769 | 33,176 | 16,502 | 16,674 |
| 65+ | 57,193 | 344,188 | 168,971 | 175,217 | 208,619 | 100,982 | 107,637 | 135,569 | 67,989 | 67,580 |

## Table C5: Population of Women 12 years and Older by Age Group, Number of Children Ever Born Alive, Children Still Living, Children Dead, Sex of Child and Economic Activity Status, Urban, Zambia 2010

| Age Group and Economic Activity Status | Number of Women 12 Years and Older | Children Ever Born Alive | | | Children Still Living | | | Children Dead | | |
|---|---|---|---|---|---|---|---|---|---|---|
| | | Total | Male | Female | Total | Male | Female | Total | Male | Female |
| **Zambia Urban** | | | | | | | | | | |
| **Total** | **1,719,038** | **3,825,100** | **1,898,770** | **1,926,330** | **3,263,609** | **1,615,503** | **1,648,106** | **561,491** | **283,267** | **278,224** |
| 12 - 14 | 203,452 | 2,960 | 1,430 | 1,530 | 2,617 | 1,241 | 1,376 | 343 | 189 | 154 |
| 15 - 19 | 329,766 | 54,574 | 27,041 | 27,533 | 50,729 | 24,999 | 25,730 | 3,845 | 2,042 | 1,803 |
| 20 - 24 | 283,698 | 282,823 | 141,099 | 141,724 | 262,547 | 130,444 | 132,103 | 20,276 | 10,655 | 9,621 |
| 25 - 29 | 253,406 | 522,434 | 260,088 | 262,346 | 480,600 | 238,121 | 242,479 | 41,834 | 21,967 | 19,867 |
| 30 - 34 | 186,651 | 582,924 | 290,052 | 292,872 | 528,392 | 261,368 | 267,024 | 54,532 | 28,684 | 25,848 |
| 35 - 39 | 136,532 | 548,664 | 273,035 | 275,629 | 490,293 | 242,859 | 247,434 | 58,371 | 30,176 | 28,195 |
| 40 - 44 | 87,411 | 420,391 | 208,203 | 212,188 | 367,881 | 181,421 | 186,460 | 52,510 | 26,782 | 25,728 |
| 45 - 49 | 71,191 | 384,966 | 191,170 | 193,796 | 329,990 | 163,633 | 166,357 | 54,976 | 27,537 | 27,439 |
| 50 - 54 | 55,031 | 323,257 | 160,651 | 162,606 | 264,736 | 131,979 | 132,757 | 58,521 | 28,672 | 29,849 |
| 55 - 59 | 35,505 | 217,936 | 108,203 | 109,733 | 170,142 | 85,024 | 85,118 | 47,794 | 23,179 | 24,615 |
| 60 - 64 | 27,179 | 173,730 | 85,826 | 87,904 | 122,531 | 61,121 | 61,410 | 51,199 | 24,705 | 26,494 |
| 65+ | 49,216 | 310,441 | 151,972 | 158,469 | 193,151 | 93,293 | 99,858 | 117,290 | 58,679 | 58,611 |
| **Economically Active** | | | | | | | | | | |
| **Total** | **622,259** | **1,738,793** | **860,349** | **878,444** | **1,493,220** | **736,328** | **756,892** | **245,573** | **124,021** | **121,552** |
| 12 - 14 | 8,202 | 272 | 132 | 140 | 244 | 114 | 130 | 28 | 18 | 10 |
| 15 - 19 | 43,742 | 13,972 | 6,943 | 7,029 | 12,939 | 6,403 | 6,536 | 1,033 | 540 | 493 |
| 20 - 24 | 108,645 | 95,630 | 47,623 | 48,007 | 88,286 | 43,747 | 44,539 | 7,344 | 3,876 | 3,468 |
| 25 - 29 | 123,479 | 217,834 | 107,919 | 109,915 | 199,471 | 98,395 | 101,076 | 18,363 | 9,524 | 8,839 |
| 30 - 34 | 101,772 | 285,868 | 141,607 | 144,261 | 257,986 | 126,990 | 130,996 | 27,882 | 14,617 | 13,265 |
| 35 - 39 | 77,985 | 288,356 | 142,739 | 145,617 | 256,459 | 126,153 | 130,306 | 31,897 | 16,586 | 15,311 |
| 40 - 44 | 50,589 | 226,960 | 111,995 | 114,965 | 197,604 | 96,920 | 100,684 | 29,356 | 15,075 | 14,281 |
| 45 - 49 | 40,287 | 207,245 | 102,316 | 104,929 | 176,771 | 87,045 | 89,726 | 30,474 | 15,271 | 15,203 |
| 50 - 54 | 28,751 | 163,281 | 80,644 | 82,637 | 133,433 | 66,114 | 67,319 | 29,848 | 14,530 | 15,318 |
| 55 - 59 | 16,604 | 100,255 | 49,677 | 50,578 | 77,653 | 38,659 | 38,994 | 22,602 | 11,018 | 11,584 |
| 60 - 64 | 10,320 | 65,678 | 32,481 | 33,197 | 46,215 | 23,057 | 23,158 | 19,463 | 9,424 | 10,039 |
| 65+ | 11,883 | 73,442 | 36,273 | 37,169 | 46,159 | 22,731 | 23,428 | 27,283 | 13,542 | 13,741 |
| **Economically Inactive** | | | | | | | | | | |
| **Total** | **1,096,779** | **2,086,307** | **1,038,421** | **1,047,886** | **1,770,389** | **879,175** | **891,214** | **315,918** | **159,246** | **156,672** |
| 12 - 14 | 195,250 | 2,688 | 1,298 | 1,390 | 2,373 | 1,127 | 1,246 | 315 | 171 | 144 |
| 15 - 19 | 286,024 | 40,602 | 20,098 | 20,504 | 37,790 | 18,596 | 19,194 | 2,812 | 1,502 | 1,310 |
| 20 - 24 | 175,053 | 187,193 | 93,476 | 93,717 | 174,261 | 86,697 | 87,564 | 12,932 | 6,779 | 6,153 |
| 25 - 29 | 129,927 | 304,600 | 152,169 | 152,431 | 281,129 | 139,726 | 141,403 | 23,471 | 12,443 | 11,028 |
| 30 - 34 | 84,879 | 297,056 | 148,445 | 148,611 | 270,406 | 134,378 | 136,028 | 26,650 | 14,067 | 12,583 |
| 35 - 39 | 58,547 | 260,308 | 130,296 | 130,012 | 233,834 | 116,706 | 117,128 | 26,474 | 13,590 | 12,884 |
| 40 - 44 | 36,822 | 193,431 | 96,208 | 97,223 | 170,277 | 84,501 | 85,776 | 23,154 | 11,707 | 11,447 |
| 45 - 49 | 30,904 | 177,721 | 88,854 | 88,867 | 153,219 | 76,588 | 76,631 | 24,502 | 12,266 | 12,236 |
| 50 - 54 | 26,280 | 159,976 | 80,007 | 79,969 | 131,303 | 65,865 | 65,438 | 28,673 | 14,142 | 14,531 |
| 55 - 59 | 18,901 | 117,681 | 58,526 | 59,155 | 92,489 | 46,365 | 46,124 | 25,192 | 12,161 | 13,031 |
| 60 - 64 | 16,859 | 108,052 | 53,345 | 54,707 | 76,316 | 38,064 | 38,252 | 31,736 | 15,281 | 16,455 |
| 65+ | 37,333 | 236,999 | 115,699 | 121,300 | 146,992 | 70,562 | 76,430 | 90,007 | 45,137 | 44,870 |

## Table C5i: Population of Women 12-49 Years by Age Group, Number of Children Born Alive 12 Months Prior to the Census, Children Still Living, Children Dead, Sex of Child and Economic Activity Status, Rural/Urban, Zambia 2010

| Age Group and Economic Activity Status | Number of Women 12 Years and Older | Children Born Alive 12 Months Prior to the Census | | | Children Still Living | | | Children Dead | | |
|---|---|---|---|---|---|---|---|---|---|---|
| | | Total | Male | Female | Total | Male | Female | Total | Male | Female |
| **Zambia Total** | | | | | | | | | | |
| **Total** | **3,488,895** | **442,998** | **224,756** | **218,242** | **423,116** | **214,441** | **208,675** | **19,882** | **10,315** | **9,567** |
| 12 - 14 | 486,104 | 921 | 470 | 451 | 870 | 447 | 423 | 51 | 23 | 28 |
| 15 - 19 | 735,025 | 58,999 | 29,701 | 29,298 | 56,523 | 28,406 | 28,117 | 2,476 | 1,295 | 1,181 |
| 20 - 24 | 612,600 | 128,270 | 65,048 | 63,222 | 123,381 | 62,419 | 60,962 | 4,889 | 2,629 | 2,260 |
| 25 - 29 | 541,751 | 114,701 | 58,366 | 56,335 | 109,784 | 55,861 | 53,923 | 4,917 | 2,505 | 2,412 |
| 30 - 34 | 403,076 | 74,066 | 37,666 | 36,400 | 70,501 | 35,842 | 34,659 | 3,565 | 1,824 | 1,741 |
| 35 - 39 | 314,852 | 45,452 | 23,072 | 22,380 | 42,958 | 21,789 | 21,169 | 2,494 | 1,283 | 1,211 |
| 40 - 44 | 215,331 | 16,150 | 8,181 | 7,969 | 15,087 | 7,635 | 7,452 | 1,063 | 546 | 517 |
| 45 - 49 | 180,156 | 4,439 | 2,252 | 2,187 | 4,012 | 2,042 | 1,970 | 427 | 210 | 217 |
| **Economically Active** | | | | | | | | | | |
| **Total** | **1,677,188** | **263,047** | **133,086** | **129,961** | **250,571** | **126,602** | **123,969** | **12,476** | **6,484** | **5,992** |
| 12 - 14 | 67,434 | 403 | 204 | 199 | 377 | 190 | 187 | 26 | 14 | 12 |
| 15 - 19 | 206,994 | 30,907 | 15,530 | 15,377 | 29,486 | 14,798 | 14,688 | 1,421 | 732 | 689 |
| 20 - 24 | 326,392 | 71,077 | 35,860 | 35,217 | 68,225 | 34,309 | 33,916 | 2,852 | 1,551 | 1,301 |
| 25 - 29 | 329,156 | 68,562 | 34,809 | 33,753 | 65,472 | 33,232 | 32,240 | 3,090 | 1,577 | 1,513 |
| 30 - 34 | 261,589 | 47,321 | 23,934 | 23,387 | 44,993 | 22,754 | 22,239 | 2,328 | 1,180 | 1,148 |
| 35 - 39 | 212,281 | 30,484 | 15,527 | 14,957 | 28,791 | 14,644 | 14,147 | 1,693 | 883 | 810 |
| 40 - 44 | 148,555 | 11,162 | 5,641 | 5,521 | 10,418 | 5,262 | 5,156 | 744 | 379 | 365 |
| 45 - 49 | 124,787 | 3,131 | 1,581 | 1,550 | 2,809 | 1,413 | 1,396 | 322 | 168 | 154 |
| **Economically Inactive** | | | | | | | | | | |
| **Total** | **1,811,707** | **179,951** | **91,670** | **88,281** | **172,545** | **87,839** | **84,706** | **7,406** | **3,831** | **3,575** |
| 12 - 14 | 418,670 | 518 | 266 | 252 | 493 | 257 | 236 | 25 | 9 | 16 |
| 15 - 19 | 528,031 | 28,092 | 14,171 | 13,921 | 27,037 | 13,608 | 13,429 | 1,055 | 563 | 492 |
| 20 - 24 | 286,208 | 57,193 | 29,188 | 28,005 | 55,156 | 28,110 | 27,046 | 2,037 | 1,078 | 959 |
| 25 - 29 | 212,595 | 46,139 | 23,557 | 22,582 | 44,312 | 22,629 | 21,683 | 1,827 | 928 | 899 |
| 30 - 34 | 141,487 | 26,745 | 13,732 | 13,013 | 25,508 | 13,088 | 12,420 | 1,237 | 644 | 593 |
| 35 - 39 | 102,571 | 14,968 | 7,545 | 7,423 | 14,167 | 7,145 | 7,022 | 801 | 400 | 401 |
| 40 - 44 | 66,776 | 4,988 | 2,540 | 2,448 | 4,669 | 2,373 | 2,296 | 319 | 167 | 152 |
| 45 - 49 | 55,369 | 1,308 | 671 | 637 | 1,203 | 629 | 574 | 105 | 42 | 63 |

## Table C5i: Population of Women 12-49 Years by Age Group, Number of Children Born Alive 12 Months Prior to the Census, Children Still Living, Children Dead, Sex of Child and Economic Activity Status, Rural, Zambia 2010

| Age Group and Economic Activity Status | Number of Women 12 Years and Older | Children Born Alive 12 Months Prior to the Census | | | Children Still Living | | | Children Dead | | |
|---|---|---|---|---|---|---|---|---|---|---|
| | | Total | Male | Female | Total | Male | Female | Total | Male | Female |
| **Zambia Rural** | | | | | | | | | | |
| **Total** | **1,936,788** | **294,803** | **149,436** | **145,367** | **281,341** | **142,357** | **138,984** | **13,462** | **7,079** | **6,383** |
| 12 - 14 | 282,652 | 672 | 342 | 330 | 630 | 323 | 307 | 42 | 19 | 23 |
| 15 - 19 | 405,259 | 41,899 | 21,054 | 20,845 | 40,055 | 20,075 | 19,980 | 1,844 | 979 | 865 |
| 20 - 24 | 328,902 | 84,736 | 42,823 | 41,913 | 81,446 | 41,041 | 40,405 | 3,290 | 1,782 | 1,508 |
| 25 - 29 | 288,345 | 72,639 | 37,041 | 35,598 | 69,506 | 35,413 | 34,093 | 3,133 | 1,628 | 1,505 |
| 30 - 34 | 216,425 | 47,489 | 24,077 | 23,412 | 45,182 | 22,876 | 22,306 | 2,307 | 1,201 | 1,106 |
| 35 - 39 | 178,320 | 31,641 | 16,135 | 15,506 | 29,939 | 15,251 | 14,688 | 1,702 | 884 | 818 |
| 40 - 44 | 127,920 | 12,286 | 6,213 | 6,073 | 11,479 | 5,802 | 5,677 | 807 | 411 | 396 |
| 45 - 49 | 108,965 | 3,441 | 1,751 | 1,690 | 3,104 | 1,576 | 1,528 | 337 | 175 | 162 |
| **Economically Active** | | | | | | | | | | |
| **Total** | **1,122,487** | **209,057** | **105,814** | **103,243** | **199,341** | **100,728** | **98,613** | **9,716** | **5,086** | **4,630** |
| 12 - 14 | 59,232 | 361 | 185 | 176 | 338 | 173 | 165 | 23 | 12 | 11 |
| 15 - 19 | 163,252 | 26,865 | 13,499 | 13,366 | 25,639 | 12,859 | 12,780 | 1,226 | 640 | 586 |
| 20 - 24 | 217,747 | 58,395 | 29,403 | 28,992 | 56,072 | 28,143 | 27,929 | 2,323 | 1,260 | 1,063 |
| 25 - 29 | 205,677 | 52,290 | 26,674 | 25,616 | 50,023 | 25,494 | 24,529 | 2,267 | 1,180 | 1,087 |
| 30 - 34 | 159,817 | 35,301 | 17,818 | 17,483 | 33,610 | 16,948 | 16,662 | 1,691 | 870 | 821 |
| 35 - 39 | 134,296 | 23,871 | 12,184 | 11,687 | 22,566 | 11,512 | 11,054 | 1,305 | 672 | 633 |
| 40 - 44 | 97,966 | 9,337 | 4,714 | 4,623 | 8,725 | 4,405 | 4,320 | 612 | 309 | 303 |
| 45 - 49 | 84,500 | 2,637 | 1,337 | 1,300 | 2,368 | 1,194 | 1,174 | 269 | 143 | 126 |
| **Economically Inactive** | | | | | | | | | | |
| **Total** | **814,301** | **85,746** | **43,622** | **42,124** | **82,000** | **41,629** | **40,371** | **3,746** | **1,993** | **1,753** |
| 12 - 14 | 223,420 | 311 | 157 | 154 | 292 | 150 | 142 | 19 | 7 | 12 |
| 15 - 19 | 242,007 | 15,034 | 7,555 | 7,479 | 14,416 | 7,216 | 7,200 | 618 | 339 | 279 |
| 20 - 24 | 111,155 | 26,341 | 13,420 | 12,921 | 25,374 | 12,898 | 12,476 | 967 | 522 | 445 |
| 25 - 29 | 82,668 | 20,349 | 10,367 | 9,982 | 19,483 | 9,919 | 9,564 | 866 | 448 | 418 |
| 30 - 34 | 56,608 | 12,188 | 6,259 | 5,929 | 11,572 | 5,928 | 5,644 | 616 | 331 | 285 |
| 35 - 39 | 44,024 | 7,770 | 3,951 | 3,819 | 7,373 | 3,739 | 3,634 | 397 | 212 | 185 |
| 40 - 44 | 29,954 | 2,949 | 1,499 | 1,450 | 2,754 | 1,397 | 1,357 | 195 | 102 | 93 |
| 45 - 49 | 24,465 | 804 | 414 | 390 | 736 | 382 | 354 | 68 | 32 | 36 |

## Table C5i: Population of Women 12-49 Years by Age Group, Number of Children Born Alive 12 Months Prior to the Census, Children Still Living, Children Dead, Sex of Child and Economic Activity Status, Urban, Zambia 2010

| Age Group and Economic Activity Status | Number of Women 12 Years and Older | Children Born Alive 12 Months Prior to the Census | | | Children Still Living | | | Children Dead | | |
|---|---|---|---|---|---|---|---|---|---|---|
| | | Total | Male | Female | Total | Male | Female | Total | Male | Female |
| **Zambia Urban** | | | | | | | | | | |
| **Total** | **1,552,107** | **148,195** | **75,320** | **72,875** | **141,775** | **72,084** | **69,691** | **6,420** | **3,236** | **3,184** |
| 12 - 14 | 203,452 | 249 | 128 | 121 | 240 | 124 | 116 | 9 | 4 | 5 |
| 15 - 19 | 329,766 | 17,100 | 8,647 | 8,453 | 16,468 | 8,331 | 8,137 | 632 | 316 | 316 |
| 20 - 24 | 283,698 | 43,534 | 22,225 | 21,309 | 41,935 | 21,378 | 20,557 | 1,599 | 847 | 752 |
| 25 - 29 | 253,406 | 42,062 | 21,325 | 20,737 | 40,278 | 20,448 | 19,830 | 1,784 | 877 | 907 |
| 30 - 34 | 186,651 | 26,577 | 13,589 | 12,988 | 25,319 | 12,966 | 12,353 | 1,258 | 623 | 635 |
| 35 - 39 | 136,532 | 13,811 | 6,937 | 6,874 | 13,019 | 6,538 | 6,481 | 792 | 399 | 393 |
| 40 - 44 | 87,411 | 3,864 | 1,968 | 1,896 | 3,608 | 1,833 | 1,775 | 256 | 135 | 121 |
| 45 - 49 | 71,191 | 998 | 501 | 497 | 908 | 466 | 442 | 90 | 35 | 55 |
| **Economically Active** | | | | | | | | | | |
| **Total** | **554,701** | **53,990** | **27,272** | **26,718** | **51,230** | **25,874** | **25,356** | **2,760** | **1,398** | **1,362** |
| 12 - 14 | 8,202 | 42 | 19 | 23 | 39 | 17 | 22 | 3 | 2 | 1 |
| 15 - 19 | 43,742 | 4,042 | 2,031 | 2,011 | 3,847 | 1,939 | 1,908 | 195 | 92 | 103 |
| 20 - 24 | 108,645 | 12,682 | 6,457 | 6,225 | 12,153 | 6,166 | 5,987 | 529 | 291 | 238 |
| 25 - 29 | 123,479 | 16,272 | 8,135 | 8,137 | 15,449 | 7,738 | 7,711 | 823 | 397 | 426 |
| 30 - 34 | 101,772 | 12,020 | 6,116 | 5,904 | 11,383 | 5,806 | 5,577 | 637 | 310 | 327 |
| 35 - 39 | 77,985 | 6,613 | 3,343 | 3,270 | 6,225 | 3,132 | 3,093 | 388 | 211 | 177 |
| 40 - 44 | 50,589 | 1,825 | 927 | 898 | 1,693 | 857 | 836 | 132 | 70 | 62 |
| 45 - 49 | 40,287 | 494 | 244 | 250 | 441 | 219 | 222 | 53 | 25 | 28 |
| **Economically Inactive** | | | | | | | | | | |
| **Total** | **997,406** | **94,205** | **48,048** | **46,157** | **90,545** | **46,210** | **44,335** | **3,660** | **1,838** | **1,822** |
| 12 - 14 | 195,250 | 207 | 109 | 98 | 201 | 107 | 94 | 6 | 2 | 4 |
| 15 - 19 | 286,024 | 13,058 | 6,616 | 6,442 | 12,621 | 6,392 | 6,229 | 437 | 224 | 213 |
| 20 - 24 | 175,053 | 30,852 | 15,768 | 15,084 | 29,782 | 15,212 | 14,570 | 1,070 | 556 | 514 |
| 25 - 29 | 129,927 | 25,790 | 13,190 | 12,600 | 24,829 | 12,710 | 12,119 | 961 | 480 | 481 |
| 30 - 34 | 84,879 | 14,557 | 7,473 | 7,084 | 13,936 | 7,160 | 6,776 | 621 | 313 | 308 |
| 35 - 39 | 58,547 | 7,198 | 3,594 | 3,604 | 6,794 | 3,406 | 3,388 | 404 | 188 | 216 |
| 40 - 44 | 36,822 | 2,039 | 1,041 | 998 | 1,915 | 976 | 939 | 124 | 65 | 59 |
| 45 - 49 | 30,904 | 504 | 257 | 247 | 467 | 247 | 220 | 37 | 10 | 27 |

## Table C6: Population of Women 12 Years and Older by Age Group, Number of Children Ever Born, Total Children Ever Born and Averag Number of Children Ever Born and Rural/Urban, Zambia 2010

| Age Group | Number of Women 12 Years and Older | Number of children ever born | | | | | | | | | | | Number of Children Ever Born | Average number of Children Ever Born |
|---|---|---|---|---|---|---|---|---|---|---|---|---|---|---|
| | | 0 | 1 | 2 | 3 | 4 | 5 | 6 | 7 | 8 | 9 | 10+ | | |
| **Zambia Total** | | | | | | | | | | | | | | |
| **Total** | **3,992,401** | **1,594,933** | **386,647** | **356,167** | **325,273** | **288,701** | **244,105** | **216,511** | **160,875** | **130,482** | **97,685** | **191,022** | **10,970,814** | **2.7** |
| 12 - 14 | 486,104 | 480,099 | 4,781 | 682 | 366 | 176 | - | - | - | - | - | - | 7,947 | * |
| 15 - 19 | 735,025 | 602,488 | 100,587 | 24,400 | 5,007 | 1,619 | 617 | 307 | - | - | - | - | 175,811 | 0.2 |
| 20 - 24 | 612,600 | 224,301 | 140,969 | 131,564 | 71,629 | 27,564 | 9,826 | 3,958 | 1,820 | 969 | - | - | 822,610 | 1.3 |
| 25 - 29 | 541,751 | 102,329 | 66,909 | 91,704 | 105,620 | 87,517 | 49,536 | 22,567 | 8,814 | 4,037 | 1,840 | 878 | 1,419,661 | 2.6 |
| 30 - 34 | 403,076 | 49,853 | 28,241 | 42,757 | 55,203 | 63,855 | 61,074 | 49,161 | 26,576 | 13,924 | 6,647 | 5,785 | 1,553,530 | 3.9 |
| 35 - 39 | 314,852 | 31,598 | 14,452 | 22,776 | 31,496 | 38,147 | 41,815 | 45,820 | 33,914 | 24,519 | 14,660 | 15,655 | 1,525,804 | 4.8 |
| 40 - 44 | 215,331 | 20,301 | 8,157 | 12,371 | 16,798 | 20,850 | 23,685 | 27,550 | 22,857 | 20,447 | 16,006 | 26,309 | 1,210,821 | 5.6 |
| 45 - 49 | 180,156 | 16,708 | 6,162 | 9,504 | 12,802 | 15,875 | 18,251 | 20,762 | 18,411 | 17,341 | 14,479 | 29,861 | 1,080,764 | 6.0 |
| 50 - 54 | 141,558 | 15,090 | 4,495 | 6,643 | 9,090 | 11,245 | 12,661 | 14,518 | 14,078 | 13,845 | 11,941 | 27,952 | 879,792 | 6.2 |
| 55 - 59 | 94,791 | 10,752 | 2,942 | 4,232 | 5,310 | 6,770 | 7,835 | 9,217 | 9,412 | 9,219 | 8,404 | 20,698 | 603,169 | 6.4 |
| 60 - 64 | 88,456 | 11,583 | 2,820 | 3,384 | 4,227 | 5,306 | 6,453 | 7,868 | 8,552 | 8,774 | 7,928 | 21,561 | 573,756 | 6.5 |
| 65+ | 178,701 | 29,831 | 6,132 | 6,150 | 7,725 | 9,777 | 12,352 | 14,783 | 16,441 | 17,407 | 15,780 | 42,323 | 1,117,149 | 6.3 |
| **Zambia Rural** | | | | | | | | | | | | | | |
| **Total** | **2,273,363** | **845,126** | **191,406** | **181,235** | **176,765** | **167,655** | **152,320** | **143,542** | **109,684** | **92,124** | **70,617** | **142,889** | **7,145,714** | **3.1** |
| 12 - 14 | 282,652 | 278,826 | 3,059 | 478 | 184 | 105 | - | - | - | - | - | - | 4,987 | * |
| 15 - 19 | 405,259 | 315,987 | 65,895 | 17,661 | 3,788 | 1,219 | 474 | 235 | - | - | - | - | 121,237 | 0.3 |
| 20 - 24 | 328,902 | 94,260 | 69,370 | 80,771 | 50,569 | 20,748 | 7,828 | 3,196 | 1,420 | 740 | - | - | 539,787 | 1.6 |
| 25 - 29 | 288,345 | 43,840 | 21,702 | 37,865 | 58,593 | 58,783 | 37,168 | 17,913 | 7,114 | 3,229 | 1,444 | 694 | 897,227 | 3.1 |
| 30 - 34 | 216,425 | 23,817 | 8,540 | 13,693 | 21,707 | 32,230 | 38,707 | 35,784 | 20,748 | 11,067 | 5,387 | 4,745 | 970,606 | 4.5 |
| 35 - 39 | 178,320 | 17,156 | 5,089 | 7,955 | 11,915 | 16,808 | 22,211 | 29,489 | 24,152 | 18,982 | 11,779 | 12,784 | 977,140 | 5.5 |
| 40 - 44 | 127,920 | 12,277 | 3,551 | 5,136 | 7,153 | 9,619 | 12,002 | 16,145 | 14,567 | 14,408 | 12,072 | 20,990 | 790,430 | 6.2 |
| 45 - 49 | 108,965 | 10,747 | 3,191 | 4,694 | 6,419 | 7,950 | 9,694 | 11,653 | 10,823 | 11,192 | 9,938 | 22,664 | 695,798 | 6.4 |
| 50 - 54 | 86,527 | 9,944 | 2,692 | 3,730 | 4,974 | 6,057 | 6,899 | 8,169 | 8,105 | 8,439 | 7,640 | 19,878 | 556,535 | 6.4 |
| 55 - 59 | 59,286 | 7,288 | 1,856 | 2,505 | 3,082 | 3,730 | 4,381 | 5,360 | 5,558 | 5,728 | 5,442 | 14,356 | 385,233 | 6.5 |
| 60 - 64 | 61,277 | 8,495 | 1,989 | 2,314 | 2,833 | 3,428 | 4,176 | 5,201 | 5,665 | 5,962 | 5,524 | 15,690 | 400,026 | 6.5 |
| 65+ | 129,485 | 22,489 | 4,472 | 4,433 | 5,548 | 6,978 | 8,780 | 10,397 | 11,532 | 12,377 | 11,391 | 31,088 | 806,708 | 6.2 |
| **Zambia Urban** | | | | | | | | | | | | | | |
| **Total** | **1,719,038** | **749,807** | **195,241** | **174,932** | **148,508** | **121,046** | **91,785** | **72,969** | **51,191** | **38,358** | **27,068** | **48,133** | **3,825,100** | **2.2** |
| 12 - 14 | 203,452 | 201,273 | 1,722 | 204 | 182 | 71 | - | - | - | - | - | - | 2,960 | * |
| 15 - 19 | 329,766 | 286,501 | 34,692 | 6,739 | 1,219 | 400 | 143 | 72 | - | - | - | - | 54,574 | 0.2 |
| 20 - 24 | 283,698 | 130,041 | 71,599 | 50,793 | 21,060 | 6,816 | 1,998 | 762 | 400 | 229 | - | - | 282,823 | 1.0 |
| 25 - 29 | 253,406 | 58,489 | 45,207 | 53,839 | 47,027 | 28,734 | 12,368 | 4,654 | 1,700 | 808 | 396 | 184 | 522,434 | 2.1 |
| 30 - 34 | 186,651 | 26,036 | 19,701 | 29,064 | 33,496 | 31,625 | 22,367 | 13,377 | 5,828 | 2,857 | 1,260 | 1,040 | 582,924 | 3.1 |
| 35 - 39 | 136,532 | 14,442 | 9,363 | 14,821 | 19,581 | 21,339 | 19,604 | 16,331 | 9,762 | 5,537 | 2,881 | 2,871 | 548,664 | 4.0 |
| 40 - 44 | 87,411 | 8,024 | 4,606 | 7,235 | 9,645 | 11,231 | 11,683 | 11,405 | 8,290 | 6,039 | 3,934 | 5,319 | 420,391 | 4.8 |
| 45 - 49 | 71,191 | 5,961 | 2,971 | 4,810 | 6,383 | 7,925 | 8,557 | 9,109 | 7,588 | 6,149 | 4,541 | 7,197 | 384,966 | 5.4 |
| 50 - 54 | 55,031 | 5,146 | 1,803 | 2,913 | 4,116 | 5,188 | 5,762 | 6,349 | 5,973 | 5,406 | 4,301 | 8,074 | 323,257 | 5.9 |
| 55 - 59 | 35,505 | 3,464 | 1,086 | 1,727 | 2,228 | 3,040 | 3,454 | 3,857 | 3,854 | 3,491 | 2,962 | 6,342 | 217,936 | 6.1 |
| 60 - 64 | 27,179 | 3,088 | 831 | 1,070 | 1,394 | 1,878 | 2,277 | 2,667 | 2,887 | 2,812 | 2,404 | 5,871 | 173,730 | 6.4 |
| 65+ | 49,216 | 7,342 | 1,660 | 1,717 | 2,177 | 2,799 | 3,572 | 4,386 | 4,909 | 5,030 | 4,389 | 11,235 | 310,441 | 6.3 |

## Table C7: Population of Women 12 Years and Older by Age Group, Average Number of Children Ever Born Alive, Children Ever Born Alive, Children Still Living, Children Dead, Sex of Child and Religion, Rural/Urban, Zambia 2010

| Age Group and Religion | Number of Women 12 Years and Older | Average Number of Children Ever Born | Children Ever Born Alive | | | Children Still Living | | | Children Dead | | |
|---|---|---|---|---|---|---|---|---|---|---|---|
| | | | Total | Male | Female | Total | Male | Female | Total | Male | Female |
| **Zambia Total** | | | | | | | | | | | |
| **Total** | **3,992,401** | **2.7** | **10,970,814** | **5,464,308** | **5,506,506** | **9,000,504** | **4,469,438** | **4,531,066** | **1,970,310** | **994,870** | **975,440** |
| 12 - 14 | 486,104 | 0.0 | 7,947 | 4,000 | 3,947 | 6,924 | 3,431 | 3,493 | 1,023 | 569 | 454 |
| 15 - 19 | 735,025 | 0.2 | 175,811 | 87,622 | 88,189 | 161,182 | 79,880 | 81,302 | 14,629 | 7,742 | 6,887 |
| 20 - 24 | 612,600 | 1.3 | 822,610 | 409,373 | 413,237 | 752,608 | 372,507 | 380,101 | 70,002 | 36,866 | 33,136 |
| 25 - 29 | 541,751 | 2.6 | 1,419,661 | 706,979 | 712,682 | 1,279,281 | 633,721 | 645,560 | 140,380 | 73,258 | 67,122 |
| 30 - 34 | 403,076 | 3.9 | 1,553,530 | 774,823 | 778,707 | 1,371,087 | 680,133 | 690,954 | 182,443 | 94,690 | 87,753 |
| 35 - 39 | 314,852 | 4.8 | 1,525,804 | 763,380 | 762,424 | 1,319,083 | 657,096 | 661,987 | 206,721 | 106,284 | 100,437 |
| 40 - 44 | 215,331 | 5.6 | 1,210,821 | 606,130 | 604,691 | 1,013,909 | 505,437 | 508,472 | 196,912 | 100,693 | 96,219 |
| 45 - 49 | 180,156 | 6.0 | 1,080,764 | 540,626 | 540,138 | 881,305 | 440,076 | 441,229 | 199,459 | 100,550 | 98,909 |
| 50 - 54 | 141,558 | 6.2 | 879,792 | 439,039 | 440,753 | 683,742 | 342,048 | 341,694 | 196,050 | 96,991 | 99,059 |
| 55 - 59 | 94,791 | 6.4 | 603,169 | 299,663 | 303,506 | 449,826 | 224,820 | 225,006 | 153,343 | 74,843 | 78,500 |
| 60 - 64 | 88,456 | 6.5 | 573,756 | 284,473 | 289,283 | 393,365 | 196,162 | 197,203 | 180,391 | 88,311 | 92,080 |
| 65+ | 178,701 | 6.3 | 1,117,149 | 548,200 | 568,949 | 688,192 | 334,127 | 354,065 | 428,957 | 214,073 | 214,884 |
| **Catholic** | | | | | | | | | | | |
| **Total** | **826,986** | **2.8** | **2,329,859** | **1,159,372** | **1,170,487** | **1,880,105** | **933,202** | **946,903** | **449,754** | **226,170** | **223,584** |
| 12 - 14 | 100,186 | 0.0 | 1,495 | 744 | 751 | 1,285 | 630 | 655 | 210 | 114 | 96 |
| 15 - 19 | 153,933 | 0.2 | 33,992 | 16,966 | 17,026 | 31,021 | 15,407 | 15,614 | 2,971 | 1,559 | 1,412 |
| 20 - 24 | 121,188 | 1.3 | 153,117 | 76,103 | 77,014 | 139,775 | 69,054 | 70,721 | 13,342 | 7,049 | 6,293 |
| 25 - 29 | 103,129 | 2.5 | 261,616 | 129,891 | 131,725 | 235,605 | 116,382 | 119,223 | 26,011 | 13,509 | 12,502 |
| 30 - 34 | 76,215 | 3.8 | 286,970 | 142,848 | 144,122 | 253,081 | 125,329 | 127,752 | 33,889 | 17,519 | 16,370 |
| 35 - 39 | 61,640 | 4.8 | 294,153 | 147,204 | 146,949 | 254,296 | 126,668 | 127,628 | 39,857 | 20,536 | 19,321 |
| 40 - 44 | 44,748 | 5.6 | 251,103 | 125,462 | 125,641 | 209,470 | 104,255 | 105,215 | 41,633 | 21,207 | 20,426 |
| 45 - 49 | 40,336 | 6.0 | 242,692 | 121,640 | 121,052 | 197,262 | 98,737 | 98,525 | 45,430 | 22,903 | 22,527 |
| 50 - 54 | 33,703 | 6.2 | 210,118 | 104,922 | 105,196 | 163,684 | 81,953 | 81,731 | 46,434 | 22,969 | 23,465 |
| 55 - 59 | 23,835 | 6.4 | 153,195 | 76,174 | 77,021 | 114,287 | 57,293 | 56,994 | 38,908 | 18,881 | 20,027 |
| 60 - 64 | 22,713 | 6.6 | 149,561 | 73,983 | 75,578 | 102,104 | 50,836 | 51,268 | 47,457 | 23,147 | 24,310 |
| 65+ | 45,360 | 6.4 | 291,847 | 143,435 | 148,412 | 178,235 | 86,658 | 91,577 | 113,612 | 56,777 | 56,835 |
| **Protestant** | | | | | | | | | | | |
| **Total** | **3,011,912** | **2.7** | **8,192,681** | **4,080,793** | **4,111,888** | **6,765,145** | **3,359,133** | **3,406,012** | **1,427,536** | **721,660** | **705,876** |
| 12 - 14 | 369,854 | 0.0 | 6,174 | 3,117 | 3,057 | 5,408 | 2,696 | 2,712 | 766 | 421 | 345 |
| 15 - 19 | 556,942 | 0.2 | 135,335 | 67,465 | 67,870 | 124,286 | 61,608 | 62,678 | 11,049 | 5,857 | 5,192 |
| 20 - 24 | 469,142 | 1.4 | 638,550 | 317,597 | 320,953 | 584,965 | 289,438 | 295,527 | 53,585 | 28,159 | 25,426 |
| 25 - 29 | 417,148 | 2.6 | 1,100,722 | 548,300 | 552,422 | 993,059 | 492,132 | 500,927 | 107,663 | 56,168 | 51,495 |
| 30 - 34 | 310,699 | 3.9 | 1,204,844 | 601,099 | 603,745 | 1,064,769 | 528,314 | 536,455 | 140,075 | 72,785 | 67,290 |
| 35 - 39 | 239,934 | 4.9 | 1,168,912 | 584,647 | 584,265 | 1,011,972 | 504,001 | 507,971 | 156,940 | 80,646 | 76,294 |
| 40 - 44 | 161,491 | 5.6 | 910,422 | 455,961 | 454,461 | 764,692 | 381,280 | 383,412 | 145,730 | 74,681 | 71,049 |
| 45 - 49 | 132,381 | 6.0 | 795,194 | 397,545 | 397,649 | 650,255 | 324,514 | 325,741 | 144,939 | 73,031 | 71,908 |
| 50 - 54 | 101,760 | 6.2 | 634,791 | 316,780 | 318,011 | 494,204 | 247,195 | 247,009 | 140,587 | 69,585 | 71,002 |
| 55 - 59 | 66,845 | 6.4 | 425,729 | 211,280 | 214,449 | 318,204 | 158,753 | 159,451 | 107,525 | 52,527 | 54,998 |
| 60 - 64 | 61,680 | 6.5 | 400,007 | 198,496 | 201,511 | 275,470 | 137,406 | 138,064 | 124,537 | 61,090 | 63,447 |
| 65+ | 124,036 | 6.2 | 772,001 | 378,506 | 393,495 | 477,861 | 231,796 | 246,065 | 294,140 | 146,710 | 147,430 |

## Table C7: Population of Women 12 Years and Older by Age Group, Average Number of Children Ever Born Alive, Children Ever Born Alive, Children Still Living, Children Dead, Sex of Child and Religion, Rural/Urban, Zambia 2010

| Age Group and Religion | Number of Women 12 Years and Older | Average Number of Children Ever Born | Children Ever Born Alive | | | Children Still Living | | | Children Dead | | |
|---|---|---|---|---|---|---|---|---|---|---|---|
| | | | Total | Male | Female | Total | Male | Female | Total | Male | Female |
| **Muslim** | | | | | | | | | | | |
| **Total** | **18,519** | **2.7** | **50,568** | **25,694** | **24,874** | **40,663** | **20,687** | **19,976** | **9,905** | **5,007** | **4,898** |
| 12 - 14 | 2,276 | 0.0 | 43 | 26 | 17 | 35 | 20 | 15 | 8 | 6 | 2 |
| 15 - 19 | 3,142 | 0.2 | 644 | 319 | 325 | 585 | 284 | 301 | 59 | 35 | 24 |
| 20 - 24 | 2,586 | 1.2 | 3,024 | 1,591 | 1,433 | 2,747 | 1,446 | 1,301 | 277 | 145 | 132 |
| 25 - 29 | 2,328 | 2.5 | 5,798 | 2,964 | 2,834 | 5,227 | 2,658 | 2,569 | 571 | 306 | 265 |
| 30 - 34 | 1,909 | 3.7 | 7,032 | 3,533 | 3,499 | 6,232 | 3,093 | 3,139 | 800 | 440 | 360 |
| 35 - 39 | 1,633 | 4.6 | 7,467 | 3,800 | 3,667 | 6,374 | 3,263 | 3,111 | 1,093 | 537 | 556 |
| 40 - 44 | 1,072 | 5.3 | 5,630 | 2,849 | 2,781 | 4,651 | 2,366 | 2,285 | 979 | 483 | 496 |
| 45 - 49 | 935 | 5.5 | 5,125 | 2,581 | 2,544 | 4,188 | 2,093 | 2,095 | 937 | 488 | 449 |
| 50 - 54 | 721 | 5.6 | 4,050 | 2,094 | 1,956 | 3,032 | 1,564 | 1,468 | 1,018 | 530 | 488 |
| 55 - 59 | 453 | 6.3 | 2,858 | 1,502 | 1,356 | 2,020 | 1,060 | 960 | 838 | 442 | 396 |
| 60 - 64 | 486 | 5.9 | 2,860 | 1,429 | 1,431 | 1,882 | 965 | 917 | 978 | 464 | 514 |
| 65+ | 978 | 6.2 | 6,037 | 3,006 | 3,031 | 3,690 | 1,875 | 1,815 | 2,347 | 1,131 | 1,216 |
| **Hindu** | | | | | | | | | | | |
| **Total** | **1,494** | **1.6** | **2,386** | **1,256** | **1,130** | **2,252** | **1,189** | **1,063** | **134** | **67** | **67** |
| 12 - 14 | 70 | - | - | - | - | - | - | - | - | - | - |
| 15 - 19 | 96 | 0.1 | 7 | 2 | 5 | 7 | 2 | 5 | - | - | - |
| 20 - 24 | 99 | 0.5 | 46 | 26 | 20 | 43 | 23 | 20 | 3 | 3 | - |
| 25 - 29 | 175 | 1.0 | 171 | 81 | 90 | 167 | 78 | 89 | 4 | 3 | 1 |
| 30 - 34 | 213 | 1.4 | 306 | 172 | 134 | 294 | 167 | 127 | 12 | 5 | 7 |
| 35 - 39 | 166 | 1.7 | 288 | 151 | 137 | 276 | 146 | 130 | 12 | 5 | 7 |
| 40 - 44 | 126 | 1.9 | 244 | 127 | 117 | 236 | 125 | 111 | 8 | 2 | 6 |
| 45 - 49 | 109 | 2.0 | 217 | 113 | 104 | 214 | 110 | 104 | 3 | 3 | - |
| 50 - 54 | 121 | 2.1 | 257 | 145 | 112 | 245 | 138 | 107 | 12 | 7 | 5 |
| 55 - 59 | 107 | 2.1 | 224 | 121 | 103 | 214 | 117 | 97 | 10 | 4 | 6 |
| 60 - 64 | 88 | 2.6 | 225 | 110 | 115 | 209 | 100 | 109 | 16 | 10 | 6 |
| 65+ | 124 | 3.2 | 401 | 208 | 193 | 347 | 183 | 164 | 54 | 25 | 29 |
| **Other** | | | | | | | | | | | |
| **Total** | **85,525** | **2.7** | **231,456** | **115,296** | **116,160** | **189,033** | **93,721** | **95,312** | **42,423** | **21,575** | **20,848** |
| 12 - 14 | 10,058 | 0.0 | 164 | 76 | 88 | 135 | 57 | 78 | 29 | 19 | 10 |
| 15 - 19 | 15,117 | 0.2 | 3,478 | 1,723 | 1,755 | 3,187 | 1,570 | 1,617 | 291 | 153 | 138 |
| 20 - 24 | 13,059 | 1.3 | 17,104 | 8,582 | 8,522 | 15,578 | 7,761 | 7,817 | 1,526 | 821 | 705 |
| 25 - 29 | 11,897 | 2.5 | 30,326 | 15,100 | 15,226 | 27,254 | 13,490 | 13,764 | 3,072 | 1,610 | 1,462 |
| 30 - 34 | 8,660 | 3.7 | 32,322 | 16,099 | 16,223 | 28,332 | 14,049 | 14,283 | 3,990 | 2,050 | 1,940 |
| 35 - 39 | 7,101 | 4.7 | 33,191 | 16,674 | 16,517 | 28,578 | 14,243 | 14,335 | 4,613 | 2,431 | 2,182 |
| 40 - 44 | 4,827 | 5.5 | 26,591 | 13,330 | 13,261 | 21,910 | 10,940 | 10,970 | 4,681 | 2,390 | 2,291 |
| 45 - 49 | 3,901 | 5.8 | 22,758 | 11,283 | 11,475 | 18,382 | 9,064 | 9,318 | 4,376 | 2,219 | 2,157 |
| 50 - 54 | 3,095 | 6.0 | 18,574 | 9,166 | 9,408 | 14,357 | 7,066 | 7,291 | 4,217 | 2,100 | 2,117 |
| 55 - 59 | 2,069 | 6.1 | 12,666 | 6,334 | 6,332 | 9,430 | 4,746 | 4,684 | 3,236 | 1,588 | 1,648 |
| 60 - 64 | 1,917 | 6.2 | 11,888 | 5,846 | 6,042 | 8,067 | 4,000 | 4,067 | 3,821 | 1,846 | 1,975 |
| 65+ | 3,824 | 5.9 | 22,394 | 11,083 | 11,311 | 13,823 | 6,735 | 7,088 | 8,571 | 4,348 | 4,223 |

## Table C7: Population of Women 12 Years and Older by Age Group, Average Number of Children Ever Born Alive, Children Ever Born Alive, Children Still Living, Children Dead, Sex of Child and Religion, Rural/Urban, Zambia 2010

| Age Group and Religion | Number of Women 12 Years and Older | Average Number of Children Ever Born | Children Ever Born Alive | | | Children Still Living | | | Children Dead | | |
|---|---|---|---|---|---|---|---|---|---|---|---|
| | | | Total | Male | Female | Total | Male | Female | Total | Male | Female |
| **None** | | | | | | | | | | | |
| **Total** | **47,965** | **3.4** | **163,864** | **81,897** | **81,967** | **123,306** | **61,506** | **61,800** | **40,558** | **20,391** | **20,167** |
| 12 - 14 | 3,660 | 0.0 | 71 | 37 | 34 | 61 | 28 | 33 | 10 | 9 | 1 |
| 15 - 19 | 5,795 | 0.4 | 2,355 | 1,147 | 1,208 | 2,096 | 1,009 | 1,087 | 259 | 138 | 121 |
| 20 - 24 | 6,526 | 1.7 | 10,769 | 5,474 | 5,295 | 9,500 | 4,785 | 4,715 | 1,269 | 689 | 580 |
| 25 - 29 | 7,074 | 3.0 | 21,028 | 10,643 | 10,385 | 17,969 | 8,981 | 8,988 | 3,059 | 1,662 | 1,397 |
| 30 - 34 | 5,380 | 4.1 | 22,056 | 11,072 | 10,984 | 18,379 | 9,181 | 9,198 | 3,677 | 1,891 | 1,786 |
| 35 - 39 | 4,378 | 5.0 | 21,793 | 10,904 | 10,889 | 17,587 | 8,775 | 8,812 | 4,206 | 2,129 | 2,077 |
| 40 - 44 | 3,067 | 5.5 | 16,831 | 8,401 | 8,430 | 12,950 | 6,471 | 6,479 | 3,881 | 1,930 | 1,951 |
| 45 - 49 | 2,494 | 5.9 | 14,778 | 7,464 | 7,314 | 11,004 | 5,558 | 5,446 | 3,774 | 1,906 | 1,868 |
| 50 - 54 | 2,158 | 5.6 | 12,002 | 5,932 | 6,070 | 8,220 | 4,132 | 4,088 | 3,782 | 1,800 | 1,982 |
| 55 - 59 | 1,482 | 5.7 | 8,497 | 4,252 | 4,245 | 5,671 | 2,851 | 2,820 | 2,826 | 1,401 | 1,425 |
| 60 - 64 | 1,572 | 5.9 | 9,215 | 4,609 | 4,606 | 5,633 | 2,855 | 2,778 | 3,582 | 1,754 | 1,828 |
| 65+ | 4,379 | 5.6 | 24,469 | 11,962 | 12,507 | 14,236 | 6,880 | 7,356 | 10,233 | 5,082 | 5,151 |

## Table C7: Population of Women 12 Years and Older by Age Group, Average Number of Children Ever Born Alive, Children Ever Born Alive, Children Still Living, Children Dead, Sex of Child and Religion, Rural, Zambia 2010

| Age Group and Religion | Number of Women 12 Years and Older | Average Number of Children Ever Born | Children Ever Born Alive | | | Children Still Living | | | Children Dead | | |
|---|---|---|---|---|---|---|---|---|---|---|---|
| | | | Total | Male | Female | Total | Male | Female | Total | Male | Female |
| **Zambia Rural** | | | | | | | | | | | |
| **Total** | **2,273,363** | **3.1** | **7,145,714** | **3,565,538** | **3,580,176** | **5,736,895** | **2,853,935** | **2,882,960** | **1,408,819** | **711,603** | **697,216** |
| 12 - 14 | 282,652 | 0.0 | 4,987 | 2,570 | 2,417 | 4,307 | 2,190 | 2,117 | 680 | 380 | 300 |
| 15 - 19 | 405,259 | 0.3 | 121,237 | 60,581 | 60,656 | 110,453 | 54,881 | 55,572 | 10,784 | 5,700 | 5,084 |
| 20 - 24 | 328,902 | 1.6 | 539,787 | 268,274 | 271,513 | 490,061 | 242,063 | 247,998 | 49,726 | 26,211 | 23,515 |
| 25 - 29 | 288,345 | 3.1 | 897,227 | 446,891 | 450,336 | 798,681 | 395,600 | 403,081 | 98,546 | 51,291 | 47,255 |
| 30 - 34 | 216,425 | 4.5 | 970,606 | 484,771 | 485,835 | 842,695 | 418,765 | 423,930 | 127,911 | 66,006 | 61,905 |
| 35 - 39 | 178,320 | 5.5 | 977,140 | 490,345 | 486,795 | 828,790 | 414,237 | 414,553 | 148,350 | 76,108 | 72,242 |
| 40 - 44 | 127,920 | 6.2 | 790,430 | 397,927 | 392,503 | 646,028 | 324,016 | 322,012 | 144,402 | 73,911 | 70,491 |
| 45 - 49 | 108,965 | 6.4 | 695,798 | 349,456 | 346,342 | 551,315 | 276,443 | 274,872 | 144,483 | 73,013 | 71,470 |
| 50 - 54 | 86,527 | 6.4 | 556,535 | 278,388 | 278,147 | 419,006 | 210,069 | 208,937 | 137,529 | 68,319 | 69,210 |
| 55 - 59 | 59,286 | 6.5 | 385,233 | 191,460 | 193,773 | 279,684 | 139,796 | 139,888 | 105,549 | 51,664 | 53,885 |
| 60 - 64 | 61,277 | 6.5 | 400,026 | 198,647 | 201,379 | 270,834 | 135,041 | 135,793 | 129,192 | 63,606 | 65,586 |
| 65+ | 129,485 | 6.2 | 806,708 | 396,228 | 410,480 | 495,041 | 240,834 | 254,207 | 311,667 | 155,394 | 156,273 |
| **Catholic** | | | | | | | | | | | |
| **Total** | **468,457** | **3.2** | **1,499,463** | **747,327** | **752,136** | **1,184,502** | **588,414** | **596,088** | **314,961** | **158,913** | **156,048** |
| 12 - 14 | 57,887 | 0.0 | 935 | 476 | 459 | 801 | 403 | 398 | 134 | 73 | 61 |
| 15 - 19 | 83,809 | 0.3 | 23,143 | 11,638 | 11,505 | 20,974 | 10,482 | 10,492 | 2,169 | 1,156 | 1,013 |
| 20 - 24 | 65,145 | 1.6 | 101,541 | 50,264 | 51,277 | 91,927 | 45,181 | 46,746 | 9,614 | 5,083 | 4,531 |
| 25 - 29 | 55,037 | 3.1 | 168,249 | 83,425 | 84,824 | 149,615 | 73,739 | 75,876 | 18,634 | 9,686 | 8,948 |
| 30 - 34 | 40,757 | 4.4 | 180,477 | 89,884 | 90,593 | 156,398 | 77,455 | 78,943 | 24,079 | 12,429 | 11,650 |
| 35 - 39 | 34,940 | 5.4 | 189,625 | 95,208 | 94,417 | 160,835 | 80,318 | 80,517 | 28,790 | 14,890 | 13,900 |
| 40 - 44 | 26,449 | 6.2 | 162,840 | 81,911 | 80,929 | 132,257 | 66,294 | 65,963 | 30,583 | 15,617 | 14,966 |
| 45 - 49 | 24,010 | 6.4 | 153,365 | 76,975 | 76,390 | 120,770 | 60,476 | 60,294 | 32,595 | 16,499 | 16,096 |
| 50 - 54 | 19,557 | 6.4 | 125,272 | 62,760 | 62,512 | 94,233 | 47,276 | 46,957 | 31,039 | 15,484 | 15,555 |
| 55 - 59 | 14,207 | 6.5 | 92,707 | 46,246 | 46,461 | 67,060 | 33,704 | 33,356 | 25,647 | 12,542 | 13,105 |
| 60 - 64 | 15,022 | 6.6 | 99,142 | 49,063 | 50,079 | 66,540 | 33,019 | 33,521 | 32,602 | 16,044 | 16,558 |
| 65+ | 31,637 | 6.4 | 202,167 | 99,477 | 102,690 | 123,092 | 60,067 | 63,025 | 79,075 | 39,410 | 39,665 |
| **Protestant** | | | | | | | | | | | |
| **Total** | **1,714,824** | **3.1** | **5,338,321** | **2,664,225** | **2,674,096** | **4,315,843** | **2,147,659** | **2,168,184** | **1,022,478** | **516,566** | **505,912** |
| 12 - 14 | 215,485 | 0.0 | 3,879 | 2,012 | 1,867 | 3,357 | 1,722 | 1,635 | 522 | 290 | 232 |
| 15 - 19 | 308,327 | 0.3 | 93,707 | 46,754 | 46,953 | 85,544 | 42,448 | 43,096 | 8,163 | 4,306 | 3,857 |
| 20 - 24 | 251,761 | 1.7 | 417,815 | 207,642 | 210,173 | 379,952 | 187,743 | 192,209 | 37,863 | 19,899 | 17,964 |
| 25 - 29 | 221,458 | 3.1 | 691,186 | 344,456 | 346,730 | 616,354 | 305,564 | 310,790 | 74,832 | 38,892 | 35,940 |
| 30 - 34 | 166,649 | 4.5 | 749,753 | 374,680 | 375,073 | 652,295 | 324,407 | 327,888 | 97,458 | 50,273 | 47,185 |
| 35 - 39 | 135,516 | 5.5 | 745,216 | 373,914 | 371,302 | 633,299 | 316,597 | 316,702 | 111,917 | 57,317 | 54,600 |
| 40 - 44 | 95,836 | 6.2 | 593,850 | 299,103 | 294,747 | 487,441 | 244,536 | 242,905 | 106,409 | 54,567 | 51,842 |
| 45 - 49 | 80,267 | 6.4 | 513,092 | 257,765 | 255,327 | 408,208 | 204,801 | 203,407 | 104,884 | 52,964 | 51,920 |
| 50 - 54 | 62,955 | 6.5 | 406,684 | 203,452 | 203,232 | 307,339 | 154,133 | 153,206 | 99,345 | 49,319 | 50,026 |
| 55 - 59 | 42,346 | 6.5 | 275,647 | 136,727 | 138,920 | 201,025 | 100,239 | 100,786 | 74,622 | 36,488 | 38,134 |
| 60 - 64 | 43,361 | 6.5 | 283,131 | 140,746 | 142,385 | 192,915 | 96,316 | 96,599 | 90,216 | 44,430 | 45,786 |
| 65+ | 90,863 | 6.2 | 564,361 | 276,974 | 287,387 | 348,114 | 169,153 | 178,961 | 216,247 | 107,821 | 108,426 |

## Table C7: Population of Women 12 Years and Older by Age Group, Average Number of Children Ever Born Alive, Children Ever Born Alive, Children Still Living, Children Dead, Sex of Child and Religion, Rural, Zambia 2010

| Age Group and Religion | Number of Women 12 Years and Older | Average Number of Children Ever Born | Children Ever Born Alive | | | Children Still Living | | | Children Dead | | |
|---|---|---|---|---|---|---|---|---|---|---|---|
| | | | Total | Male | Female | Total | Male | Female | Total | Male | Female |
| **Muslim** | | | | | | | | | | | |
| **Total** | **7,018** | **3.5** | **24,901** | **12,600** | **12,301** | **18,561** | **9,393** | **9,168** | **6,340** | **3,207** | **3,133** |
| 12 - 14 | 885 | * | 16 | 8 | 8 | 14 | 6 | 8 | 2 | 2 | - |
| 15 - 19 | 1,092 | 0.3 | 345 | 171 | 174 | 310 | 152 | 158 | 35 | 19 | 16 |
| 20 - 24 | 837 | 1.6 | 1,326 | 703 | 623 | 1,174 | 622 | 552 | 152 | 81 | 71 |
| 25 - 29 | 812 | 3.3 | 2,639 | 1,363 | 1,276 | 2,269 | 1,175 | 1,094 | 370 | 188 | 182 |
| 30 - 34 | 620 | 4.8 | 2,945 | 1,474 | 1,471 | 2,469 | 1,214 | 1,255 | 476 | 260 | 216 |
| 35 - 39 | 636 | 5.7 | 3,605 | 1,837 | 1,768 | 2,877 | 1,482 | 1,395 | 728 | 355 | 373 |
| 40 - 44 | 442 | 6.6 | 2,910 | 1,471 | 1,439 | 2,241 | 1,132 | 1,109 | 669 | 339 | 330 |
| 45 - 49 | 363 | 6.5 | 2,372 | 1,177 | 1,195 | 1,792 | 879 | 913 | 580 | 298 | 282 |
| 50 - 54 | 338 | 6.5 | 2,187 | 1,097 | 1,090 | 1,463 | 725 | 738 | 724 | 372 | 352 |
| 55 - 59 | 220 | 6.8 | 1,495 | 786 | 709 | 964 | 497 | 467 | 531 | 289 | 242 |
| 60 - 64 | 252 | 6.4 | 1,622 | 807 | 815 | 1,006 | 515 | 491 | 616 | 292 | 324 |
| 65+ | 521 | 6.6 | 3,439 | 1,706 | 1,733 | 1,982 | 994 | 988 | 1,457 | 712 | 745 |
| **Hindu** | | | | | | | | | | | |
| **Total** | **117** | **2.4** | **275** | **135** | **140** | **219** | **106** | **113** | **56** | **29** | **27** |
| 12 - 14 | 14 | - | - | - | - | - | - | - | - | - | - |
| 15 - 19 | 23 | 0.1 | 3 | 1 | 2 | 3 | 1 | 2 | - | - | - |
| 20 - 24 | 12 | 1.5 | 18 | 9 | 9 | 15 | 6 | 9 | 3 | 3 | - |
| 25 - 29 | 18 | 2.0 | 36 | 14 | 22 | 32 | 11 | 21 | 4 | 3 | 1 |
| 30 - 34 | 9 | 2.0 | 18 | 11 | 7 | 17 | 11 | 6 | 1 | - | 1 |
| 35 - 39 | 9 | 3.3 | 30 | 19 | 11 | 27 | 17 | 10 | 3 | 2 | 1 |
| 40 - 44 | 7 | 5.4 | 38 | 18 | 20 | 34 | 17 | 17 | 4 | 1 | 3 |
| 45 - 49 | 5 | 4.4 | 22 | 9 | 13 | 21 | 8 | 13 | 1 | 1 | - |
| 50 - 54 | 3 | 1.3 | 4 | 2 | 2 | 3 | 2 | 1 | 1 | - | 1 |
| 55 - 59 | 4 | 5.0 | 20 | 10 | 10 | 17 | 9 | 8 | 3 | 1 | 2 |
| 60 - 64 | 5 | 4.6 | 23 | 11 | 12 | 18 | 9 | 9 | 5 | 2 | 3 |
| 65+ | 8 | 7.9 | 63 | 31 | 32 | 32 | 15 | 17 | 31 | 16 | 15 |
| **Other** | | | | | | | | | | | |
| **Total** | **45,557** | **3.2** | **144,261** | **72,005** | **72,256** | **114,537** | **56,900** | **57,637** | **29,724** | **15,105** | **14,619** |
| 12 - 14 | 5,420 | * | 99 | 45 | 54 | 84 | 36 | 48 | 15 | 9 | 6 |
| 15 - 19 | 7,728 | 0.3 | 2,208 | 1,119 | 1,089 | 2,008 | 1,011 | 997 | 200 | 108 | 92 |
| 20 - 24 | 6,522 | 1.6 | 10,661 | 5,357 | 5,304 | 9,584 | 4,770 | 4,814 | 1,077 | 587 | 490 |
| 25 - 29 | 5,798 | 3.1 | 17,973 | 8,953 | 9,020 | 15,860 | 7,834 | 8,026 | 2,113 | 1,119 | 994 |
| 30 - 34 | 4,400 | 4.4 | 19,490 | 9,723 | 9,767 | 16,715 | 8,296 | 8,419 | 2,775 | 1,427 | 1,348 |
| 35 - 39 | 3,727 | 5.4 | 20,051 | 10,058 | 9,993 | 16,815 | 8,383 | 8,432 | 3,236 | 1,675 | 1,561 |
| 40 - 44 | 2,720 | 6.1 | 16,507 | 8,288 | 8,219 | 13,161 | 6,593 | 6,568 | 3,346 | 1,695 | 1,651 |
| 45 - 49 | 2,262 | 6.3 | 14,226 | 7,109 | 7,117 | 11,098 | 5,527 | 5,571 | 3,128 | 1,582 | 1,546 |
| 50 - 54 | 1,809 | 6.4 | 11,617 | 5,750 | 5,867 | 8,646 | 4,247 | 4,399 | 2,971 | 1,503 | 1,468 |
| 55 - 59 | 1,252 | 6.4 | 7,968 | 3,996 | 3,972 | 5,704 | 2,891 | 2,813 | 2,264 | 1,105 | 1,159 |
| 60 - 64 | 1,283 | 6.3 | 8,126 | 4,017 | 4,109 | 5,437 | 2,689 | 2,748 | 2,689 | 1,328 | 1,361 |
| 65+ | 2,636 | 5.8 | 15,335 | 7,590 | 7,745 | 9,425 | 4,623 | 4,802 | 5,910 | 2,967 | 2,943 |

## Table C7: Population of Women 12 Years and Older by Age Group, Average Number of Children Ever Born Alive, Children Ever Born Alive, Children Still Living, Children Dead, Sex of Child and Religion, Rural, Zambia 2010

| Age Group and Religion | Number of Women 12 Years and Older | Average Number of Children Ever Born | Children Ever Born Alive | | | Children Still Living | | | Children Dead | | |
|---|---|---|---|---|---|---|---|---|---|---|---|
| | | | Total | Male | Female | Total | Male | Female | Total | Male | Female |
| **None** | | | | | | | | | | | |
| **Total** | **37,390** | **3.7** | **138,493** | **69,246** | **69,247** | **103,233** | **51,463** | **51,770** | **35,260** | **17,783** | **17,477** |
| 12 - 14 | 2,961 | 0.0 | 58 | 29 | 29 | 51 | 23 | 28 | 7 | 6 | 1 |
| 15 - 19 | 4,280 | 0.4 | 1,831 | 898 | 933 | 1,614 | 787 | 827 | 217 | 111 | 106 |
| 20 - 24 | 4,625 | 1.8 | 8,426 | 4,299 | 4,127 | 7,409 | 3,741 | 3,668 | 1,017 | 558 | 459 |
| 25 - 29 | 5,222 | 3.3 | 17,144 | 8,680 | 8,464 | 14,551 | 7,277 | 7,274 | 2,593 | 1,403 | 1,190 |
| 30 - 34 | 3,990 | 4.5 | 17,923 | 8,999 | 8,924 | 14,801 | 7,382 | 7,419 | 3,122 | 1,617 | 1,505 |
| 35 - 39 | 3,492 | 5.3 | 18,613 | 9,309 | 9,304 | 14,937 | 7,440 | 7,497 | 3,676 | 1,869 | 1,807 |
| 40 - 44 | 2,466 | 5.8 | 14,285 | 7,136 | 7,149 | 10,894 | 5,444 | 5,450 | 3,391 | 1,692 | 1,699 |
| 45 - 49 | 2,058 | 6.2 | 12,721 | 6,421 | 6,300 | 9,426 | 4,752 | 4,674 | 3,295 | 1,669 | 1,626 |
| 50 - 54 | 1,865 | 5.8 | 10,771 | 5,327 | 5,444 | 7,322 | 3,686 | 3,636 | 3,449 | 1,641 | 1,808 |
| 55 - 59 | 1,257 | 5.9 | 7,396 | 3,695 | 3,701 | 4,914 | 2,456 | 2,458 | 2,482 | 1,239 | 1,243 |
| 60 - 64 | 1,354 | 5.9 | 7,982 | 4,003 | 3,979 | 4,918 | 2,493 | 2,425 | 3,064 | 1,510 | 1,554 |
| 65+ | 3,820 | 5.6 | 21,343 | 10,450 | 10,893 | 12,396 | 5,982 | 6,414 | 8,947 | 4,468 | 4,479 |

## Table C7: Population of Women 12 Years and Older by Age Group, Average Number of Children Ever Born Alive, Children Ever Born Alive, Children Still Living, Children Dead, Sex of Child and Religion, Urban, Zambia 2010

| Age Group and Religion | Number of Women 12 Years and Older | Average Number of Children Ever Born | Children Ever Born Alive | | | Children Still Living | | | Children Dead | | |
|---|---|---|---|---|---|---|---|---|---|---|---|
| | | | Total | Male | Female | Total | Male | Female | Total | Male | Female |
| **Zambia Urban** | | | | | | | | | | | |
| **Total** | **1,719,038** | **2.2** | **3,825,100** | **1,898,770** | **1,926,330** | **3,263,609** | **1,615,503** | **1,648,106** | **561,491** | **283,267** | **278,224** |
| 12 - 14 | 203,452 | 0.0 | 2,960 | 1,430 | 1,530 | 2,617 | 1,241 | 1,376 | 343 | 189 | 154 |
| 15 - 19 | 329,766 | 0.2 | 54,574 | 27,041 | 27,533 | 50,729 | 24,999 | 25,730 | 3,845 | 2,042 | 1,803 |
| 20 - 24 | 283,698 | 1.0 | 282,823 | 141,099 | 141,724 | 262,547 | 130,444 | 132,103 | 20,276 | 10,655 | 9,621 |
| 25 - 29 | 253,406 | 2.1 | 522,434 | 260,088 | 262,346 | 480,600 | 238,121 | 242,479 | 41,834 | 21,967 | 19,867 |
| 30 - 34 | 186,651 | 3.1 | 582,924 | 290,052 | 292,872 | 528,392 | 261,368 | 267,024 | 54,532 | 28,684 | 25,848 |
| 35 - 39 | 136,532 | 4.0 | 548,664 | 273,035 | 275,629 | 490,293 | 242,859 | 247,434 | 58,371 | 30,176 | 28,195 |
| 40 - 44 | 87,411 | 4.8 | 420,391 | 208,203 | 212,188 | 367,881 | 181,421 | 186,460 | 52,510 | 26,782 | 25,728 |
| 45 - 49 | 71,191 | 5.4 | 384,966 | 191,170 | 193,796 | 329,990 | 163,633 | 166,357 | 54,976 | 27,537 | 27,439 |
| 50 - 54 | 55,031 | 5.9 | 323,257 | 160,651 | 162,606 | 264,736 | 131,979 | 132,757 | 58,521 | 28,672 | 29,849 |
| 55 - 59 | 35,505 | 6.1 | 217,936 | 108,203 | 109,733 | 170,142 | 85,024 | 85,118 | 47,794 | 23,179 | 24,615 |
| 60 - 64 | 27,179 | 6.4 | 173,730 | 85,826 | 87,904 | 122,531 | 61,121 | 61,410 | 51,199 | 24,705 | 26,494 |
| 65+ | 49,216 | 6.3 | 310,441 | 151,972 | 158,469 | 193,151 | 93,293 | 99,858 | 117,290 | 58,679 | 58,611 |
| **Catholic** | | | | | | | | | | | |
| **Total** | **358,529** | **2.3** | **830,396** | **412,045** | **418,351** | **695,603** | **344,788** | **350,815** | **134,793** | **67,257** | **67,536** |
| 12 - 14 | 42,299 | 0.0 | 560 | 268 | 292 | 484 | 227 | 257 | 76 | 41 | 35 |
| 15 - 19 | 70,124 | 0.2 | 10,849 | 5,328 | 5,521 | 10,047 | 4,925 | 5,122 | 802 | 403 | 399 |
| 20 - 24 | 56,043 | 0.9 | 51,576 | 25,839 | 25,737 | 47,848 | 23,873 | 23,975 | 3,728 | 1,966 | 1,762 |
| 25 - 29 | 48,092 | 1.9 | 93,367 | 46,466 | 46,901 | 85,990 | 42,643 | 43,347 | 7,377 | 3,823 | 3,554 |
| 30 - 34 | 35,458 | 3.0 | 106,493 | 52,964 | 53,529 | 96,683 | 47,874 | 48,809 | 9,810 | 5,090 | 4,720 |
| 35 - 39 | 26,700 | 3.9 | 104,528 | 51,996 | 52,532 | 93,461 | 46,350 | 47,111 | 11,067 | 5,646 | 5,421 |
| 40 - 44 | 18,299 | 4.8 | 88,263 | 43,551 | 44,712 | 77,213 | 37,961 | 39,252 | 11,050 | 5,590 | 5,460 |
| 45 - 49 | 16,326 | 5.5 | 89,327 | 44,665 | 44,662 | 76,492 | 38,261 | 38,231 | 12,835 | 6,404 | 6,431 |
| 50 - 54 | 14,146 | 6.0 | 84,846 | 42,162 | 42,684 | 69,451 | 34,677 | 34,774 | 15,395 | 7,485 | 7,910 |
| 55 - 59 | 9,628 | 6.3 | 60,488 | 29,928 | 30,560 | 47,227 | 23,589 | 23,638 | 13,261 | 6,339 | 6,922 |
| 60 - 64 | 7,691 | 6.6 | 50,419 | 24,920 | 25,499 | 35,564 | 17,817 | 17,747 | 14,855 | 7,103 | 7,752 |
| 65+ | 13,723 | 6.5 | 89,680 | 43,958 | 45,722 | 55,143 | 26,591 | 28,552 | 34,537 | 17,367 | 17,170 |
| **Protestant** | | | | | | | | | | | |
| **Total** | **1,297,088** | **2.2** | **2,854,360** | **1,416,568** | **1,437,792** | **2,449,302** | **1,211,474** | **1,237,828** | **405,058** | **205,094** | **199,964** |
| 12 - 14 | 154,369 | 0.0 | 2,295 | 1,105 | 1,190 | 2,051 | 974 | 1,077 | 244 | 131 | 113 |
| 15 - 19 | 248,615 | 0.2 | 41,628 | 20,711 | 20,917 | 38,742 | 19,160 | 19,582 | 2,886 | 1,551 | 1,335 |
| 20 - 24 | 217,381 | 1.0 | 220,735 | 109,955 | 110,780 | 205,013 | 101,695 | 103,318 | 15,722 | 8,260 | 7,462 |
| 25 - 29 | 195,690 | 2.1 | 409,536 | 203,844 | 205,692 | 376,705 | 186,568 | 190,137 | 32,831 | 17,276 | 15,555 |
| 30 - 34 | 144,050 | 3.2 | 455,091 | 226,419 | 228,672 | 412,474 | 203,907 | 208,567 | 42,617 | 22,512 | 20,105 |
| 35 - 39 | 104,418 | 4.1 | 423,696 | 210,733 | 212,963 | 378,673 | 187,404 | 191,269 | 45,023 | 23,329 | 21,694 |
| 40 - 44 | 65,655 | 4.8 | 316,572 | 156,858 | 159,714 | 277,251 | 136,744 | 140,507 | 39,321 | 20,114 | 19,207 |
| 45 - 49 | 52,114 | 5.4 | 282,102 | 139,780 | 142,322 | 242,047 | 119,713 | 122,334 | 40,055 | 20,067 | 19,988 |
| 50 - 54 | 38,805 | 5.9 | 228,107 | 113,328 | 114,779 | 186,865 | 93,062 | 93,803 | 41,242 | 20,266 | 20,976 |
| 55 - 59 | 24,499 | 6.1 | 150,082 | 74,553 | 75,529 | 117,179 | 58,514 | 58,665 | 32,903 | 16,039 | 16,864 |
| 60 - 64 | 18,319 | 6.4 | 116,876 | 57,750 | 59,126 | 82,555 | 41,090 | 41,465 | 34,321 | 16,660 | 17,661 |
| 65+ | 33,173 | 6.3 | 207,640 | 101,532 | 106,108 | 129,747 | 62,643 | 67,104 | 77,893 | 38,889 | 39,004 |

## Table C7: Population of Women 12 Years and Older by Age Group, Average Number of Children Ever Born Alive, Children Ever Born Alive, Children Still Living, Children Dead, Sex of Child and Religion, Urban, Zambia 2010

| Age Group and Religion | Number of Women 12 Years and Older | Average Number of Children Ever Born | Children Ever Born Alive | | | Children Still Living | | | Children Dead | | |
|---|---|---|---|---|---|---|---|---|---|---|---|
| | | | Total | Male | Female | Total | Male | Female | Total | Male | Female |
| **Muslim** | | | | | | | | | | | |
| **Total** | **11,501** | **2.2** | **25,667** | **13,094** | **12,573** | **22,102** | **11,294** | **10,808** | **3,565** | **1,800** | **1,765** |
| 12 - 14 | 1,391 | 0.0 | 27 | 18 | 9 | 21 | 14 | 7 | 6 | 4 | 2 |
| 15 - 19 | 2,050 | 0.1 | 299 | 148 | 151 | 275 | 132 | 143 | 24 | 16 | 8 |
| 20 - 24 | 1,749 | 1.0 | 1,698 | 888 | 810 | 1,573 | 824 | 749 | 125 | 64 | 61 |
| 25 - 29 | 1,516 | 2.1 | 3,159 | 1,601 | 1,558 | 2,958 | 1,483 | 1,475 | 201 | 118 | 83 |
| 30 - 34 | 1,289 | 3.2 | 4,087 | 2,059 | 2,028 | 3,763 | 1,879 | 1,884 | 324 | 180 | 144 |
| 35 - 39 | 997 | 3.9 | 3,862 | 1,963 | 1,899 | 3,497 | 1,781 | 1,716 | 365 | 182 | 183 |
| 40 - 44 | 630 | 4.3 | 2,720 | 1,378 | 1,342 | 2,410 | 1,234 | 1,176 | 310 | 144 | 166 |
| 45 - 49 | 572 | 4.8 | 2,753 | 1,404 | 1,349 | 2,396 | 1,214 | 1,182 | 357 | 190 | 167 |
| 50 - 54 | 383 | 4.9 | 1,863 | 997 | 866 | 1,569 | 839 | 730 | 294 | 158 | 136 |
| 55 - 59 | 233 | 5.8 | 1,363 | 716 | 647 | 1,056 | 563 | 493 | 307 | 153 | 154 |
| 60 - 64 | 234 | 5.3 | 1,238 | 622 | 616 | 876 | 450 | 426 | 362 | 172 | 190 |
| 65+ | 457 | 5.7 | 2,598 | 1,300 | 1,298 | 1,708 | 881 | 827 | 890 | 419 | 471 |
| **Hindu** | | | | | | | | | | | |
| **Total** | **1,377** | **1.5** | **2,111** | **1,121** | **990** | **2,033** | **1,083** | **950** | **78** | **38** | **40** |
| 12 - 14 | 56 | - | - | - | - | - | - | - | - | - | - |
| 15 - 19 | 73 | 0.1 | 4 | 1 | 3 | 4 | 1 | 3 | - | - | - |
| 20 - 24 | 87 | 0.3 | 28 | 17 | 11 | 28 | 17 | 11 | - | - | - |
| 25 - 29 | 157 | 0.9 | 135 | 67 | 68 | 135 | 67 | 68 | - | - | - |
| 30 - 34 | 204 | 1.4 | 288 | 161 | 127 | 277 | 156 | 121 | 11 | 5 | 6 |
| 35 - 39 | 157 | 1.6 | 258 | 132 | 126 | 249 | 129 | 120 | 9 | 3 | 6 |
| 40 - 44 | 119 | 1.7 | 206 | 109 | 97 | 202 | 108 | 94 | 4 | 1 | 3 |
| 45 - 49 | 104 | 1.9 | 195 | 104 | 91 | 193 | 102 | 91 | 2 | 2 | - |
| 50 - 54 | 118 | 2.1 | 253 | 143 | 110 | 242 | 136 | 106 | 11 | 7 | 4 |
| 55 - 59 | 103 | 2.0 | 204 | 111 | 93 | 197 | 108 | 89 | 7 | 3 | 4 |
| 60 - 64 | 83 | 2.4 | 202 | 99 | 103 | 191 | 91 | 100 | 11 | 8 | 3 |
| 65+ | 116 | 2.9 | 338 | 177 | 161 | 315 | 168 | 147 | 23 | 9 | 14 |
| **Other** | | | | | | | | | | | |
| **Total** | **39,968** | **2.2** | **87,195** | **43,291** | **43,904** | **74,496** | **36,821** | **37,675** | **12,699** | **6,470** | **6,229** |
| 12 - 14 | 4,638 | 0.0 | 65 | 31 | 34 | 51 | 21 | 30 | 14 | 10 | 4 |
| 15 - 19 | 7,389 | 0.2 | 1,270 | 604 | 666 | 1,179 | 559 | 620 | 91 | 45 | 46 |
| 20 - 24 | 6,537 | 1.0 | 6,443 | 3,225 | 3,218 | 5,994 | 2,991 | 3,003 | 449 | 234 | 215 |
| 25 - 29 | 6,099 | 2.0 | 12,353 | 6,147 | 6,206 | 11,394 | 5,656 | 5,738 | 959 | 491 | 468 |
| 30 - 34 | 4,260 | 3.0 | 12,832 | 6,376 | 6,456 | 11,617 | 5,753 | 5,864 | 1,215 | 623 | 592 |
| 35 - 39 | 3,374 | 3.9 | 13,140 | 6,616 | 6,524 | 11,763 | 5,860 | 5,903 | 1,377 | 756 | 621 |
| 40 - 44 | 2,107 | 4.8 | 10,084 | 5,042 | 5,042 | 8,749 | 4,347 | 4,402 | 1,335 | 695 | 640 |
| 45 - 49 | 1,639 | 5.2 | 8,532 | 4,174 | 4,358 | 7,284 | 3,537 | 3,747 | 1,248 | 637 | 611 |
| 50 - 54 | 1,286 | 5.4 | 6,957 | 3,416 | 3,541 | 5,711 | 2,819 | 2,892 | 1,246 | 597 | 649 |
| 55 - 59 | 817 | 5.8 | 4,698 | 2,338 | 2,360 | 3,726 | 1,855 | 1,871 | 972 | 483 | 489 |
| 60 - 64 | 634 | 5.9 | 3,762 | 1,829 | 1,933 | 2,630 | 1,311 | 1,319 | 1,132 | 518 | 614 |
| 65+ | 1,188 | 5.9 | 7,059 | 3,493 | 3,566 | 4,398 | 2,112 | 2,286 | 2,661 | 1,381 | 1,280 |

## Table C7: Population of Women 12 Years and Older by Age Group, Average Number of Children Ever Born Alive, Children Ever Born Alive, Children Still Living, Children Dead, Sex of Child and Religion, Urban, Zambia 2010

| Age Group and Religion | Number of Women 12 Years and Older | Average Number of Children Ever Born | Children Ever Born Alive | | | Children Still Living | | | Children Dead | | |
|---|---|---|---|---|---|---|---|---|---|---|---|
| | | | Total | Male | Female | Total | Male | Female | Total | Male | Female |
| **None** | | | | | | | | | | | |
| **Total** | **10,575** | **2.4** | **25,371** | **12,651** | **12,720** | **20,073** | **10,043** | **10,030** | **5,298** | **2,608** | **2,690** |
| 12 - 14 | 699 | 0.0 | 13 | 8 | 5 | 10 | 5 | 5 | 3 | 3 | - |
| 15 - 19 | 1,515 | 0.3 | 524 | 249 | 275 | 482 | 222 | 260 | 42 | 27 | 15 |
| 20 - 24 | 1,901 | 1.2 | 2,343 | 1,175 | 1,168 | 2,091 | 1,044 | 1,047 | 252 | 131 | 121 |
| 25 - 29 | 1,852 | 2.1 | 3,884 | 1,963 | 1,921 | 3,418 | 1,704 | 1,714 | 466 | 259 | 207 |
| 30 - 34 | 1,390 | 3.0 | 4,133 | 2,073 | 2,060 | 3,578 | 1,799 | 1,779 | 555 | 274 | 281 |
| 35 - 39 | 886 | 3.6 | 3,180 | 1,595 | 1,585 | 2,650 | 1,335 | 1,315 | 530 | 260 | 270 |
| 40 - 44 | 601 | 4.2 | 2,546 | 1,265 | 1,281 | 2,056 | 1,027 | 1,029 | 490 | 238 | 252 |
| 45 - 49 | 436 | 4.7 | 2,057 | 1,043 | 1,014 | 1,578 | 806 | 772 | 479 | 237 | 242 |
| 50 - 54 | 293 | 4.2 | 1,231 | 605 | 626 | 898 | 446 | 452 | 333 | 159 | 174 |
| 55 - 59 | 225 | 4.9 | 1,101 | 557 | 544 | 757 | 395 | 362 | 344 | 162 | 182 |
| 60 - 64 | 218 | 5.7 | 1,233 | 606 | 627 | 715 | 362 | 353 | 518 | 244 | 274 |
| 65+ | 559 | 5.6 | 3,126 | 1,512 | 1,614 | 1,840 | 898 | 942 | 1,286 | 614 | 672 |

## Table C7i: Popuplation of Women Aged 12-49 Years By Age Group, Number of Children Born Alive 12 Months Prior to the Census, Children still Living, Children Dead and Religion, Rural/Urban, Zambia 2010

| Age Group and Religion | Number of Women 12 Years and Older | Children Born Alive 12 Months Prior to the Census | | | Children Still Living | | | Children Dead | | |
|---|---|---|---|---|---|---|---|---|---|---|
| | | Total | Male | Female | Total | Male | Female | Total | Male | Female |
| **Total** | **3,488,895** | **442,998** | **224,756** | **218,242** | **423,116** | **214,441** | **208,675** | **19,882** | **10,315** | **9,567** |
| 12 - 14 | 486,104 | 921 | 470 | 451 | 870 | 447 | 423 | 51 | 23 | 28 |
| 15 - 19 | 735,025 | 58,999 | 29,701 | 29,298 | 56,523 | 28,406 | 28,117 | 2,476 | 1,295 | 1,181 |
| 20 - 24 | 612,600 | 128,270 | 65,048 | 63,222 | 123,381 | 62,419 | 60,962 | 4,889 | 2,629 | 2,260 |
| 25 - 29 | 541,751 | 114,701 | 58,366 | 56,335 | 109,784 | 55,861 | 53,923 | 4,917 | 2,505 | 2,412 |
| 30 - 34 | 403,076 | 74,066 | 37,666 | 36,400 | 70,501 | 35,842 | 34,659 | 3,565 | 1,824 | 1,741 |
| 35 - 39 | 314,852 | 45,452 | 23,072 | 22,380 | 42,958 | 21,789 | 21,169 | 2,494 | 1,283 | 1,211 |
| 40 - 44 | 215,331 | 16,150 | 8,181 | 7,969 | 15,087 | 7,635 | 7,452 | 1,063 | 546 | 517 |
| 45 - 49 | 180,156 | 4,439 | 2,252 | 2,187 | 4,012 | 2,042 | 1,970 | 427 | 210 | 217 |
| **Catholic** | | | | | | | | | | |
| **Total** | **701,375** | **82,691** | **41,912** | **40,779** | **78,846** | **39,889** | **38,957** | **3,845** | **2,023** | **1,822** |
| 12 - 14 | 100,186 | 169 | 96 | 73 | 159 | 93 | 66 | 10 | 3 | 7 |
| 15 - 19 | 153,933 | 11,705 | 5,893 | 5,812 | 11,173 | 5,621 | 5,552 | 532 | 272 | 260 |
| 20 - 24 | 121,188 | 24,033 | 12,166 | 11,867 | 23,110 | 11,654 | 11,456 | 923 | 512 | 411 |
| 25 - 29 | 103,129 | 20,964 | 10,614 | 10,350 | 20,009 | 10,133 | 9,876 | 955 | 481 | 474 |
| 30 - 34 | 76,215 | 13,313 | 6,747 | 6,566 | 12,665 | 6,411 | 6,254 | 648 | 336 | 312 |
| 35 - 39 | 61,640 | 8,451 | 4,303 | 4,148 | 7,975 | 4,045 | 3,930 | 476 | 258 | 218 |
| 40 - 44 | 44,748 | 3,104 | 1,603 | 1,501 | 2,885 | 1,483 | 1,402 | 219 | 120 | 99 |
| 45 - 49 | 40,336 | 952 | 490 | 462 | 870 | 449 | 421 | 82 | 41 | 41 |
| **Protestant** | | | | | | | | | | |
| **Total** | **2,657,591** | **342,573** | **173,818** | **168,755** | **327,468** | **165,998** | **161,470** | **15,105** | **7,820** | **7,285** |
| 12 - 14 | 369,854 | 720 | 359 | 361 | 681 | 339 | 342 | 39 | 20 | 19 |
| 15 - 19 | 556,942 | 45,188 | 22,728 | 22,460 | 43,349 | 21,760 | 21,589 | 1,839 | 968 | 871 |
| 20 - 24 | 469,142 | 99,368 | 50,387 | 48,981 | 95,610 | 48,380 | 47,230 | 3,758 | 2,007 | 1,751 |
| 25 - 29 | 417,148 | 89,065 | 45,362 | 43,703 | 85,330 | 43,456 | 41,874 | 3,735 | 1,906 | 1,829 |
| 30 - 34 | 310,699 | 57,686 | 29,361 | 28,325 | 54,945 | 27,957 | 26,988 | 2,741 | 1,404 | 1,337 |
| 35 - 39 | 239,934 | 34,957 | 17,772 | 17,185 | 33,070 | 16,813 | 16,257 | 1,887 | 959 | 928 |
| 40 - 44 | 161,491 | 12,329 | 6,200 | 6,129 | 11,538 | 5,799 | 5,739 | 791 | 401 | 390 |
| 45 - 49 | 132,381 | 3,260 | 1,649 | 1,611 | 2,945 | 1,494 | 1,451 | 315 | 155 | 160 |
| **Muslim** | | | | | | | | | | |
| **Total** | **15,881** | **1,738** | **936** | **802** | **1,660** | **896** | **764** | **78** | **40** | **38** |
| 12 - 14 | 2,276 | 2 | 1 | 1 | 2 | 1 | 1 | - | - | - |
| 15 - 19 | 3,142 | 197 | 105 | 92 | 189 | 101 | 88 | 8 | 4 | 4 |
| 20 - 24 | 2,586 | 446 | 251 | 195 | 429 | 242 | 187 | 17 | 9 | 8 |
| 25 - 29 | 2,328 | 444 | 246 | 198 | 426 | 236 | 190 | 18 | 10 | 8 |
| 30 - 34 | 1,909 | 329 | 164 | 165 | 312 | 157 | 155 | 17 | 7 | 10 |
| 35 - 39 | 1,633 | 219 | 119 | 100 | 208 | 113 | 95 | 11 | 6 | 5 |
| 40 - 44 | 1,072 | 67 | 34 | 33 | 62 | 31 | 31 | 5 | 3 | 2 |
| 45 - 49 | 935 | 34 | 16 | 18 | 32 | 15 | 17 | 2 | 1 | 1 |
| **Hindu** | | | | | | | | | | |
| **Total** | **1,054** | **69** | **36** | **33** | **68** | **36** | **32** | **1** | **-** | **1** |
| 12 - 14 | 70 | - | - | - | - | - | - | - | - | - |
| 15 - 19 | 96 | 4 | 1 | 3 | 4 | 1 | 3 | - | - | - |
| 20 - 24 | 99 | 10 | 4 | 6 | 10 | 4 | 6 | - | - | - |
| 25 - 29 | 175 | 24 | 11 | 13 | 24 | 11 | 13 | - | - | - |
| 30 - 34 | 213 | 22 | 14 | 8 | 22 | 14 | 8 | - | - | - |
| 35 - 39 | 166 | 5 | 4 | 1 | 4 | 4 | - | 1 | - | 1 |
| 40 - 44 | 126 | 4 | 2 | 2 | 4 | 2 | 2 | - | - | - |
| 45 - 49 | 109 | - | - | - | - | - | - | - | - | - |

## Table C7i: Popuplation of Women Aged 12-49 Years By Age Group, Number of Children Born Alive 12 Months Prior to the Census, Children still Living, Children Dead and Religion, Rural/Urban, Zambia 2010

| Age Group and Religion | Number of Women 12 Years and Older | Children Born Alive 12 Months Prior to the Census | | | Children Still Living | | | Children Dead | | |
|---|---|---|---|---|---|---|---|---|---|---|
| | | Total | Male | Female | Total | Male | Female | Total | Male | Female |
| **Other** | | | | | | | | | | |
| **Total** | **74,620** | **9,365** | **4,770** | **4,595** | **8,903** | **4,540** | **4,363** | **462** | **230** | **232** |
| 12 - 14 | 10,058 | 17 | 7 | 10 | 15 | 7 | 8 | 2 | - | 2 |
| 15 - 19 | 15,117 | 1,149 | 602 | 547 | 1,095 | 572 | 523 | 54 | 30 | 24 |
| 20 - 24 | 13,059 | 2,741 | 1,380 | 1,361 | 2,626 | 1,321 | 1,305 | 115 | 59 | 56 |
| 25 - 29 | 11,897 | 2,461 | 1,282 | 1,179 | 2,357 | 1,228 | 1,129 | 104 | 54 | 50 |
| 30 - 34 | 8,660 | 1,538 | 778 | 760 | 1,458 | 745 | 713 | 80 | 33 | 47 |
| 35 - 39 | 7,101 | 1,021 | 492 | 529 | 953 | 456 | 497 | 68 | 36 | 32 |
| 40 - 44 | 4,827 | 354 | 183 | 171 | 329 | 174 | 155 | 25 | 9 | 16 |
| 45 - 49 | 3,901 | 84 | 46 | 38 | 70 | 37 | 33 | 14 | 9 | 5 |
| **None** | | | | | | | | | | |
| **Total** | **38,374** | **6,562** | **3,284** | **3,278** | **6,171** | **3,082** | **3,089** | **391** | **202** | **189** |
| 12 - 14 | 3,660 | 13 | 7 | 6 | 13 | 7 | 6 | - | - | - |
| 15 - 19 | 5,795 | 756 | 372 | 384 | 713 | 351 | 362 | 43 | 21 | 22 |
| 20 - 24 | 6,526 | 1,672 | 860 | 812 | 1,596 | 818 | 778 | 76 | 42 | 34 |
| 25 - 29 | 7,074 | 1,743 | 851 | 892 | 1,638 | 797 | 841 | 105 | 54 | 51 |
| 30 - 34 | 5,380 | 1,178 | 602 | 576 | 1,099 | 558 | 541 | 79 | 44 | 35 |
| 35 - 39 | 4,378 | 799 | 382 | 417 | 748 | 358 | 390 | 51 | 24 | 27 |
| 40 - 44 | 3,067 | 292 | 159 | 133 | 269 | 146 | 123 | 23 | 13 | 10 |
| 45 - 49 | 2,494 | 109 | 51 | 58 | 95 | 47 | 48 | 14 | 4 | 10 |

# Education Characteristics Tables

## Table D1: Population (De Facto) 5 Years and Older by 5 Year Age Group, School Attendance and Province, Zambia 2010

| Province and Age Group | Population 5 Year and Older | Never Attended | Ever Attended | | |
|---|---|---|---|---|---|
| | | | Total | Currently Attending | Not Currently Attending |
| **Zambia Total** | **10,311,427** | **2,186,558** | **8,124,869** | **3,522,038** | **4,602,831** |
| 5 - 9 | 1,856,336 | 953,860 | 902,476 | 857,354 | 45,122 |
| 10 - 14 | 1,699,042 | 186,449 | 1,512,593 | 1,423,205 | 89,388 |
| 15 - 19 | 1,427,884 | 118,138 | 1,309,746 | 921,525 | 388,221 |
| 20 - 24 | 1,117,476 | 138,744 | 978,732 | 195,228 | 783,504 |
| 25 - 29 | 1,006,244 | 147,279 | 858,965 | 50,048 | 808,917 |
| 30 - 34 | 800,770 | 114,851 | 685,919 | 25,714 | 660,205 |
| 35 - 39 | 647,813 | 97,423 | 550,390 | 17,575 | 532,815 |
| 40 - 44 | 447,584 | 72,378 | 375,206 | 10,243 | 364,963 |
| 45 - 49 | 354,989 | 62,442 | 292,547 | 6,784 | 285,763 |
| 50 - 54 | 270,254 | 53,832 | 216,422 | 4,537 | 211,885 |
| 55 + | 683,035 | 241,162 | 441,873 | 9,825 | 432,048 |
| **Central Province** | | | | | |
| **Total** | **1,020,800** | **211,102** | **809,698** | **359,125** | **450,573** |
| 5 - 9 | 190,254 | 97,673 | 92,581 | 87,651 | 4,930 |
| 10 - 14 | 175,827 | 17,780 | 158,047 | 148,886 | 9,161 |
| 15 - 19 | 144,976 | 11,365 | 133,611 | 92,669 | 40,942 |
| 20 - 24 | 108,067 | 12,962 | 95,105 | 18,615 | 76,490 |
| 25 - 29 | 91,398 | 12,562 | 78,836 | 4,376 | 74,460 |
| 30 - 34 | 74,143 | 10,078 | 64,065 | 2,257 | 61,808 |
| 35 - 39 | 61,248 | 8,501 | 52,747 | 1,557 | 51,190 |
| 40 - 44 | 43,287 | 6,536 | 36,751 | 949 | 35,802 |
| 45 - 49 | 34,498 | 5,696 | 28,802 | 654 | 28,148 |
| 50 - 54 | 27,078 | 5,061 | 22,017 | 436 | 21,581 |
| 55 + | 70,024 | 22,888 | 47,136 | 1,075 | 46,061 |
| **Copperbelt Province** | | | | | |
| **Total** | **1,632,427** | **163,899** | **1,468,528** | **629,019** | **839,509** |
| 5 - 9 | 248,404 | 84,992 | 163,412 | 156,827 | 6,585 |
| 10 - 14 | 258,545 | 10,277 | 248,268 | 236,626 | 11,642 |
| 15 - 19 | 244,789 | 6,449 | 238,340 | 177,496 | 60,844 |
| 20 - 24 | 183,304 | 7,304 | 176,000 | 34,629 | 141,371 |
| 25 - 29 | 167,226 | 7,789 | 159,437 | 9,643 | 149,794 |
| 30 - 34 | 133,289 | 6,267 | 127,022 | 4,928 | 122,094 |
| 35 - 39 | 106,800 | 5,573 | 101,227 | 3,355 | 97,872 |
| 40 - 44 | 75,181 | 4,456 | 70,725 | 1,953 | 68,772 |
| 45 - 49 | 60,717 | 4,203 | 56,514 | 1,217 | 55,297 |
| 50 - 54 | 47,907 | 4,145 | 43,762 | 801 | 42,961 |
| 55 + | 106,265 | 22,444 | 83,821 | 1,544 | 82,277 |
| **Eastern Province** | | | | | |
| **Total** | **1,244,326** | **458,750** | **785,576** | **342,761** | **442,815** |
| 5 - 9 | 239,952 | 164,765 | 75,187 | 70,797 | 4,390 |
| 10 - 14 | 212,911 | 49,454 | 163,457 | 151,014 | 12,443 |
| 15 - 19 | 166,676 | 31,118 | 135,558 | 92,175 | 43,383 |
| 20 - 24 | 127,428 | 34,460 | 92,968 | 18,904 | 74,064 |
| 25 - 29 | 112,688 | 35,958 | 76,730 | 3,936 | 72,794 |
| 30 - 34 | 88,441 | 28,190 | 60,251 | 1,773 | 58,478 |
| 35 - 39 | 74,483 | 24,371 | 50,112 | 1,257 | 48,855 |
| 40 - 44 | 51,632 | 16,869 | 34,763 | 762 | 34,001 |
| 45 - 49 | 42,696 | 14,180 | 28,516 | 592 | 27,924 |
| 50 - 54 | 32,008 | 11,125 | 20,883 | 452 | 20,431 |
| 55 + | 95,411 | 48,260 | 47,151 | 1,099 | 46,052 |

## Table D1: Population (De Facto) 5 Years and Older by 5 Year Age Group, School Attendance and Province, Zambia 2010

| Province and Age Group | Population 5 Year and Older | Never Attended | Ever Attended | | |
|---|---|---|---|---|---|
| | | | Total | Currently Attending | Not Currently Attending |
| **Luapula Province** | | | | | |
| **Total** | **759,576** | **207,757** | **551,819** | **236,071** | **315,748** |
| 5 - 9 | 149,455 | 95,859 | 53,596 | 50,280 | 3,316 |
| 10 - 14 | 130,510 | 20,925 | 109,585 | 101,632 | 7,953 |
| 15 - 19 | 100,669 | 11,053 | 89,616 | 62,902 | 26,714 |
| 20 - 24 | 73,509 | 12,840 | 60,669 | 13,937 | 46,732 |
| 25 - 29 | 67,803 | 13,998 | 53,805 | 2,880 | 50,925 |
| 30 - 34 | 54,625 | 10,521 | 44,104 | 1,331 | 42,773 |
| 35 - 39 | 45,626 | 8,869 | 36,757 | 887 | 35,870 |
| 40 - 44 | 34,119 | 6,782 | 27,337 | 665 | 26,672 |
| 45 - 49 | 27,403 | 5,398 | 22,005 | 447 | 21,558 |
| 50 - 54 | 21,820 | 4,619 | 17,201 | 330 | 16,871 |
| 55 + | 54,037 | 16,893 | 37,144 | 780 | 36,364 |
| **Lusaka Province** | | | | | |
| **Total** | **1,808,658** | **175,723** | **1,632,935** | **667,403** | **965,532** |
| 5 - 9 | 274,057 | 85,330 | 188,727 | 178,450 | 10,277 |
| 10 - 14 | 267,942 | 11,116 | 256,826 | 241,471 | 15,355 |
| 15 - 19 | 246,301 | 7,211 | 239,090 | 169,909 | 69,181 |
| 20 - 24 | 224,293 | 10,029 | 214,264 | 42,381 | 171,883 |
| 25 - 29 | 214,183 | 11,115 | 203,068 | 14,144 | 188,924 |
| 30 - 34 | 174,999 | 9,341 | 165,658 | 8,123 | 157,535 |
| 35 - 39 | 135,925 | 7,637 | 128,288 | 5,567 | 122,721 |
| 40 - 44 | 84,105 | 5,307 | 78,798 | 2,946 | 75,852 |
| 45 - 49 | 59,683 | 4,693 | 54,990 | 1,711 | 53,279 |
| 50 - 54 | 42,330 | 4,407 | 37,923 | 994 | 36,929 |
| 55 + | 84,840 | 19,537 | 65,303 | 1,707 | 63,596 |
| **Muchinga Province** | | | | | |
| **Total** | **547,410** | **141,922** | **405,488** | **179,316** | **226,172** |
| 5 - 9 | 111,376 | 64,592 | 46,784 | 44,360 | 2,424 |
| 10 - 14 | 94,047 | 11,986 | 82,061 | 77,206 | 4,855 |
| 15 - 19 | 72,759 | 7,841 | 64,918 | 44,292 | 20,626 |
| 20 - 24 | 55,816 | 9,173 | 46,643 | 8,340 | 38,303 |
| 25 - 29 | 49,634 | 9,372 | 40,262 | 1,936 | 38,326 |
| 30 - 34 | 37,396 | 6,817 | 30,579 | 926 | 29,653 |
| 35 - 39 | 31,699 | 5,936 | 25,763 | 659 | 25,104 |
| 40 - 44 | 22,491 | 4,438 | 18,053 | 423 | 17,630 |
| 45 - 49 | 18,634 | 3,874 | 14,760 | 336 | 14,424 |
| 50 - 54 | 13,900 | 3,200 | 10,700 | 247 | 10,453 |
| 55 + | 39,658 | 14,693 | 24,965 | 591 | 24,374 |
| **Northern Province** | | | | | |
| **Total** | **838,997** | **235,102** | **603,895** | **259,651** | **344,244** |
| 5 - 9 | 169,675 | 106,534 | 63,141 | 59,551 | 3,590 |
| 10 - 14 | 144,083 | 20,785 | 123,298 | 114,747 | 8,551 |
| 15 - 19 | 111,259 | 13,191 | 98,068 | 64,960 | 33,108 |
| 20 - 24 | 84,699 | 15,820 | 68,879 | 12,746 | 56,133 |
| 25 - 29 | 76,556 | 16,622 | 59,934 | 2,882 | 57,052 |
| 30 - 34 | 56,707 | 11,377 | 45,330 | 1,430 | 43,900 |
| 35 - 39 | 49,211 | 9,789 | 39,422 | 1,047 | 38,375 |
| 40 - 44 | 34,969 | 7,179 | 27,790 | 622 | 27,168 |
| 45 - 49 | 29,770 | 6,330 | 23,440 | 451 | 22,989 |
| 50 - 54 | 22,143 | 5,328 | 16,815 | 346 | 16,469 |
| 55 + | 59,925 | 22,147 | 37,778 | 869 | 36,909 |

## Table D1: Population (De Facto) 5 Years and Older by 5 Year Age Group, School Attendance and Province, Zambia 2010

| Province and Age Group | Population 5 Year and Older | Never Attended | Ever Attended | | |
|---|---|---|---|---|---|
| | | | Total | Currently Attending | Not Currently Attending |
| **North Western Province** | | | | | |
| **Total** | **548,723** | **146,112** | **402,611** | **197,558** | **205,053** |
| 5 - 9 | 109,165 | 59,988 | 49,177 | 46,817 | 2,360 |
| 10 - 14 | 95,597 | 11,211 | 84,386 | 79,828 | 4,558 |
| 15 - 19 | 76,074 | 6,979 | 69,095 | 51,589 | 17,506 |
| 20 - 24 | 55,377 | 8,349 | 47,028 | 12,786 | 34,242 |
| 25 - 29 | 49,985 | 9,878 | 40,107 | 2,998 | 37,109 |
| 30 - 34 | 38,742 | 8,014 | 30,728 | 1,243 | 29,485 |
| 35 - 39 | 31,051 | 6,741 | 24,310 | 797 | 23,513 |
| 40 - 44 | 22,881 | 5,712 | 17,169 | 458 | 16,711 |
| 45 - 49 | 18,163 | 4,956 | 13,207 | 351 | 12,856 |
| 50 - 54 | 13,384 | 4,248 | 9,136 | 215 | 8,921 |
| 55 + | 38,304 | 20,036 | 18,268 | 476 | 17,792 |
| **Southern Province** | | | | | |
| **Total** | **1,231,545** | **233,771** | **997,774** | **450,212** | **547,562** |
| 5 - 9 | 236,602 | 115,870 | 120,732 | 115,701 | 5,031 |
| 10 - 14 | 209,728 | 15,409 | 194,319 | 185,070 | 9,249 |
| 15 - 19 | 175,472 | 10,726 | 164,746 | 114,510 | 50,236 |
| 20 - 24 | 134,896 | 13,192 | 121,704 | 22,378 | 99,326 |
| 25 - 29 | 115,241 | 14,092 | 101,149 | 4,857 | 96,292 |
| 30 - 34 | 93,577 | 11,762 | 81,815 | 2,677 | 79,138 |
| 35 - 39 | 73,027 | 9,794 | 63,233 | 1,747 | 61,486 |
| 40 - 44 | 50,447 | 7,204 | 43,243 | 1,047 | 42,196 |
| 45 - 49 | 39,587 | 6,108 | 33,479 | 704 | 32,775 |
| 50 - 54 | 29,666 | 5,252 | 24,414 | 478 | 23,936 |
| 55 + | 73,302 | 24,362 | 48,940 | 1,043 | 47,897 |
| **Western Province** | | | | | |
| **Total** | **678,965** | **212,420** | **466,545** | **200,922** | **265,623** |
| 5 - 9 | 127,396 | 78,257 | 49,139 | 46,920 | 2,219 |
| 10 - 14 | 109,852 | 17,506 | 92,346 | 86,725 | 5,621 |
| 15 - 19 | 88,909 | 12,205 | 76,704 | 51,023 | 25,681 |
| 20 - 24 | 70,087 | 14,615 | 55,472 | 10,512 | 44,960 |
| 25 - 29 | 61,530 | 15,893 | 45,637 | 2,396 | 43,241 |
| 30 - 34 | 48,851 | 12,484 | 36,367 | 1,026 | 35,341 |
| 35 - 39 | 38,743 | 10,212 | 28,531 | 702 | 27,829 |
| 40 - 44 | 28,472 | 7,895 | 20,577 | 418 | 20,159 |
| 45 - 49 | 23,838 | 7,004 | 16,834 | 321 | 16,513 |
| 50 - 54 | 20,018 | 6,447 | 13,571 | 238 | 13,333 |
| 55 + | 61,269 | 29,902 | 31,367 | 641 | 30,726 |

## Table D2: Population (De facto) 5 Years and Older by 5 Year Age Group, Sex and School Attendance, Rural/Urban, Zambia 2010

| Age Group and Sex | Population 5 Years and Older | Never Attended | Ever Attended | | |
|---|---|---|---|---|---|
| | | | Total | Currently Attending | Not Currently Attending |
| **Zambia Total** | **10,311,427** | **2,186,558** | **8,124,869** | **3,522,038** | **4,602,831** |
| 5 - 9 | 1,856,336 | 953,860 | 902,476 | 857,354 | 45,122 |
| 10 - 14 | 1,699,042 | 186,449 | 1,512,593 | 1,423,205 | 89,388 |
| 15 - 19 | 1,427,884 | 118,138 | 1,309,746 | 921,525 | 388,221 |
| 20 - 24 | 1,117,476 | 138,744 | 978,732 | 195,228 | 783,504 |
| 25 - 29 | 1,006,244 | 147,279 | 858,965 | 50,048 | 808,917 |
| 30 - 34 | 800,770 | 114,851 | 685,919 | 25,714 | 660,205 |
| 35 - 39 | 647,813 | 97,423 | 550,390 | 17,575 | 532,815 |
| 40 - 44 | 447,584 | 72,378 | 375,206 | 10,243 | 364,963 |
| 45 - 49 | 354,989 | 62,442 | 292,547 | 6,784 | 285,763 |
| 50 - 54 | 270,254 | 53,832 | 216,422 | 4,537 | 211,885 |
| 55 + | 683,035 | 241,162 | 441,873 | 9,825 | 432,048 |
| **Male** | | | | | |
| **Total** | **5,014,981** | **932,565** | **4,082,416** | **1,801,506** | **2,280,910** |
| 5 - 9 | 924,395 | 487,237 | 437,158 | 414,763 | 22,395 |
| 10 - 14 | 840,834 | 97,706 | 743,128 | 700,359 | 42,769 |
| 15 - 19 | 692,859 | 49,837 | 643,022 | 498,680 | 144,342 |
| 20 - 24 | 504,876 | 45,944 | 458,932 | 122,776 | 336,156 |
| 25 - 29 | 464,493 | 50,231 | 414,262 | 25,846 | 388,416 |
| 30 - 34 | 397,694 | 42,470 | 355,224 | 12,614 | 342,610 |
| 35 - 39 | 332,961 | 35,366 | 297,595 | 9,073 | 288,522 |
| 40 - 44 | 232,253 | 25,243 | 207,010 | 5,792 | 201,218 |
| 45 - 49 | 174,833 | 19,855 | 154,978 | 3,701 | 151,277 |
| 50 - 54 | 128,696 | 14,895 | 113,801 | 2,427 | 111,374 |
| 55 + | 321,087 | 63,781 | 257,306 | 5,475 | 251,831 |
| **Female** | | | | | |
| **Total** | **5,296,446** | **1,253,993** | **4,042,453** | **1,720,532** | **2,321,921** |
| 5 - 9 | 931,941 | 466,623 | 465,318 | 442,591 | 22,727 |
| 10 - 14 | 858,208 | 88,743 | 769,465 | 722,846 | 46,619 |
| 15 - 19 | 735,025 | 68,301 | 666,724 | 422,845 | 243,879 |
| 20 - 24 | 612,600 | 92,800 | 519,800 | 72,452 | 447,348 |
| 25 - 29 | 541,751 | 97,048 | 444,703 | 24,202 | 420,501 |
| 30 - 34 | 403,076 | 72,381 | 330,695 | 13,100 | 317,595 |
| 35 - 39 | 314,852 | 62,057 | 252,795 | 8,502 | 244,293 |
| 40 - 44 | 215,331 | 47,135 | 168,196 | 4,451 | 163,745 |
| 45 - 49 | 180,156 | 42,587 | 137,569 | 3,083 | 134,486 |
| 50 - 54 | 141,558 | 38,937 | 102,621 | 2,110 | 100,511 |
| 55 + | 361,948 | 177,381 | 184,567 | 4,350 | 180,217 |

## Table D2: Population (De facto) 5 Years and Older by 5 Year Age Group, Sex and School Attendance, Rural, Zambia 2010

| Age Group and Sex | Population 5 Years and Older | Never Attended | Ever Attended | | |
|---|---|---|---|---|---|
| | | | Total | Currently Attending | Not Currently Attending |
| **Zambia Rural** | **6,050,907** | **1,779,136** | **4,271,771** | **1,883,424** | **2,388,347** |
| 5 - 9 | 1,212,247 | 739,002 | 473,245 | 447,959 | 25,286 |
| 10 - 14 | 1,045,977 | 160,997 | 884,980 | 827,669 | 57,311 |
| 15 - 19 | 802,997 | 102,017 | 700,980 | 470,339 | 230,641 |
| 20 - 24 | 598,270 | 118,003 | 480,267 | 87,061 | 393,206 |
| 25 - 29 | 529,481 | 125,064 | 404,417 | 19,114 | 385,303 |
| 30 - 34 | 419,932 | 97,101 | 322,831 | 9,244 | 313,587 |
| 35 - 39 | 351,970 | 82,414 | 269,556 | 6,604 | 262,952 |
| 40 - 44 | 254,964 | 61,561 | 193,403 | 4,111 | 189,292 |
| 45 - 49 | 209,868 | 52,823 | 157,045 | 3,113 | 153,932 |
| 50 - 54 | 162,395 | 44,677 | 117,718 | 2,329 | 115,389 |
| 55 + | 462,806 | 195,477 | 267,329 | 5,881 | 261,448 |
| **Male** | | | | | |
| **Total** | **2,940,191** | **762,704** | **2,177,487** | **991,386** | **1,186,101** |
| 5 - 9 | 608,241 | 378,568 | 229,673 | 217,035 | 12,638 |
| 10 - 14 | 529,978 | 85,470 | 444,508 | 416,204 | 28,304 |
| 15 - 19 | 397,738 | 43,328 | 354,410 | 270,079 | 84,331 |
| 20 - 24 | 269,368 | 39,164 | 230,204 | 60,088 | 170,116 |
| 25 - 29 | 241,136 | 42,940 | 198,196 | 10,841 | 187,355 |
| 30 - 34 | 203,507 | 36,460 | 167,047 | 4,904 | 162,143 |
| 35 - 39 | 173,650 | 30,272 | 143,378 | 3,471 | 139,907 |
| 40 - 44 | 127,044 | 21,800 | 105,244 | 2,388 | 102,856 |
| 45 - 49 | 100,903 | 17,293 | 83,610 | 1,733 | 81,877 |
| 50 - 54 | 75,868 | 12,888 | 62,980 | 1,258 | 61,722 |
| 55 + | 212,758 | 54,521 | 158,237 | 3,385 | 154,852 |
| **Female** | | | | | |
| **Total** | **3,110,716** | **1,016,432** | **2,094,284** | **892,038** | **1,202,246** |
| 5 - 9 | 604,006 | 360,434 | 243,572 | 230,924 | 12,648 |
| 10 - 14 | 515,999 | 75,527 | 440,472 | 411,465 | 29,007 |
| 15 - 19 | 405,259 | 58,689 | 346,570 | 200,260 | 146,310 |
| 20 - 24 | 328,902 | 78,839 | 250,063 | 26,973 | 223,090 |
| 25 - 29 | 288,345 | 82,124 | 206,221 | 8,273 | 197,948 |
| 30 - 34 | 216,425 | 60,641 | 155,784 | 4,340 | 151,444 |
| 35 - 39 | 178,320 | 52,142 | 126,178 | 3,133 | 123,045 |
| 40 - 44 | 127,920 | 39,761 | 88,159 | 1,723 | 86,436 |
| 45 - 49 | 108,965 | 35,530 | 73,435 | 1,380 | 72,055 |
| 50 - 54 | 86,527 | 31,789 | 54,738 | 1,071 | 53,667 |
| 55 + | 250,048 | 140,956 | 109,092 | 2,496 | 106,596 |

## Table D2: Population (De facto) 5 Years and Older by 5 Year Age Group, Sex and School Attendance, Urban, Zambia 2010

| Age Group and Sex | Population 5 Years and Older | Never Attended | Ever Attended | | |
|---|---|---|---|---|---|
| | | | Total | Currently Attending | Not Currently Attending |
| **Zambia Urban** | **4,260,520** | **407,422** | **3,853,098** | **1,638,614** | **2,214,484** |
| 5 - 9 | 644,089 | 214,858 | 429,231 | 409,395 | 19,836 |
| 10 - 14 | 653,065 | 25,452 | 627,613 | 595,536 | 32,077 |
| 15 - 19 | 624,887 | 16,121 | 608,766 | 451,186 | 157,580 |
| 20 - 24 | 519,206 | 20,741 | 498,465 | 108,167 | 390,298 |
| 25 - 29 | 476,763 | 22,215 | 454,548 | 30,934 | 423,614 |
| 30 - 34 | 380,838 | 17,750 | 363,088 | 16,470 | 346,618 |
| 35 - 39 | 295,843 | 15,009 | 280,834 | 10,971 | 269,863 |
| 40 - 44 | 192,620 | 10,817 | 181,803 | 6,132 | 175,671 |
| 45 - 49 | 145,121 | 9,619 | 135,502 | 3,671 | 131,831 |
| 50 - 54 | 107,859 | 9,155 | 98,704 | 2,208 | 96,496 |
| 55 + | 220,229 | 45,685 | 174,544 | 3,944 | 170,600 |
| **Male** | | | | | |
| **Total** | **2,074,790** | **169,861** | **1,904,929** | **810,120** | **1,094,809** |
| 5 - 9 | 316,154 | 108,669 | 207,485 | 197,728 | 9,757 |
| 10 - 14 | 310,856 | 12,236 | 298,620 | 284,155 | 14,465 |
| 15 - 19 | 295,121 | 6,509 | 288,612 | 228,601 | 60,011 |
| 20 - 24 | 235,508 | 6,780 | 228,728 | 62,688 | 166,040 |
| 25 - 29 | 223,357 | 7,291 | 216,066 | 15,005 | 201,061 |
| 30 - 34 | 194,187 | 6,010 | 188,177 | 7,710 | 180,467 |
| 35 - 39 | 159,311 | 5,094 | 154,217 | 5,602 | 148,615 |
| 40 - 44 | 105,209 | 3,443 | 101,766 | 3,404 | 98,362 |
| 45 - 49 | 73,930 | 2,562 | 71,368 | 1,968 | 69,400 |
| 50 - 54 | 52,828 | 2,007 | 50,821 | 1,169 | 49,652 |
| 55 + | 108,329 | 9,260 | 99,069 | 2,090 | 96,979 |
| **Female** | | | | | |
| **Total** | **2,185,730** | **237,561** | **1,948,169** | **828,494** | **1,119,675** |
| 5 - 9 | 327,935 | 106,189 | 221,746 | 211,667 | 10,079 |
| 10 - 14 | 342,209 | 13,216 | 328,993 | 311,381 | 17,612 |
| 15 - 19 | 329,766 | 9,612 | 320,154 | 222,585 | 97,569 |
| 20 - 24 | 283,698 | 13,961 | 269,737 | 45,479 | 224,258 |
| 25 - 29 | 253,406 | 14,924 | 238,482 | 15,929 | 222,553 |
| 30 - 34 | 186,651 | 11,740 | 174,911 | 8,760 | 166,151 |
| 35 - 39 | 136,532 | 9,915 | 126,617 | 5,369 | 121,248 |
| 40 - 44 | 87,411 | 7,374 | 80,037 | 2,728 | 77,309 |
| 45 - 49 | 71,191 | 7,057 | 64,134 | 1,703 | 62,431 |
| 50 - 54 | 55,031 | 7,148 | 47,883 | 1,039 | 46,844 |
| 55 + | 111,900 | 36,425 | 75,475 | 1,854 | 73,621 |

## Table D2i: Population (De Facto) 5 Years and Older by Age (Single and Grouped) School Attendance and Rural/Urban, Zambia 2010

| Age and Sex | Population 5 Years and Older | Never Attended | Ever Attended | | |
|---|---|---|---|---|---|
| | | | Total | Currently Attending | Not Currently Attending |
| **Zambia Total** | **10,311,427** | **2,186,558** | **8,124,869** | **3,522,038** | **4,602,831** |
| 5 | 406,833 | 328,978 | 77,855 | 71,138 | 6,717 |
| 6 | 396,794 | 274,684 | 122,110 | 114,076 | 8,034 |
| 7 | 388,305 | 181,100 | 207,205 | 197,484 | 9,721 |
| 8 | 351,465 | 107,352 | 244,113 | 233,718 | 10,395 |
| 9 | 312,939 | 61,746 | 251,193 | 240,938 | 10,255 |
| 5 - 9 | 1,856,336 | 953,860 | 902,476 | 857,354 | 45,122 |
| 10 | 449,492 | 73,438 | 376,054 | 358,915 | 17,139 |
| 11 | 290,577 | 33,140 | 257,437 | 244,830 | 12,607 |
| 12 | 351,053 | 33,496 | 317,557 | 299,775 | 17,782 |
| 13 | 305,128 | 23,920 | 281,208 | 263,290 | 17,918 |
| 14 | 302,792 | 22,455 | 280,337 | 256,395 | 23,942 |
| 10 - 14 | 1,699,042 | 186,449 | 1,512,593 | 1,423,205 | 89,388 |
| 15 | 312,338 | 24,549 | 287,789 | 251,323 | 36,466 |
| 16 | 295,552 | 21,820 | 273,732 | 224,867 | 48,865 |
| 17 | 260,146 | 19,732 | 240,414 | 174,971 | 65,443 |
| 18 | 309,090 | 27,573 | 281,517 | 167,593 | 113,924 |
| 19 | 250,758 | 24,464 | 226,294 | 102,771 | 123,523 |
| 15 - 19 | 1,427,884 | 118,138 | 1,309,746 | 921,525 | 388,221 |
| 20 - 24 | 1,117,476 | 138,744 | 978,732 | 195,228 | 783,504 |
| 25 - 29 | 1,006,244 | 147,279 | 858,965 | 50,048 | 808,917 |
| 30 - 34 | 800,770 | 114,851 | 685,919 | 25,714 | 660,205 |
| 35 - 39 | 647,813 | 97,423 | 550,390 | 17,575 | 532,815 |
| 40 - 44 | 447,584 | 72,378 | 375,206 | 10,243 | 364,963 |
| 45 - 49 | 354,989 | 62,442 | 292,547 | 6,784 | 285,763 |
| 50 - 54 | 270,254 | 53,832 | 216,422 | 4,537 | 211,885 |
| 55 - 59 | 184,828 | 41,022 | 143,806 | 2,800 | 141,006 |
| 60 - 64 | 161,875 | 51,643 | 110,232 | 2,088 | 108,144 |
| 65 + | 336,332 | 148,497 | 187,835 | 4,937 | 182,898 |

## Table D2i: Population (De Facto) 5 Years and Older by Age (Single and Grouped) School Attendance and Rural/Urban, Zambia 2010

| Age and Sex | Population 5 Years and Older | Never Attended | Ever Attended | | |
|---|---|---|---|---|---|
| | | | Total | Currently Attending | Not Currently Attending |
| **Male** | **5,014,981** | **932,565** | **4,082,416** | **1,801,506** | **2,280,910** |
| 5 | 204,250 | 166,377 | 37,873 | 34,506 | 3,367 |
| 6 | 197,481 | 139,442 | 58,039 | 53,994 | 4,045 |
| 7 | 194,701 | 93,884 | 100,817 | 96,006 | 4,811 |
| 8 | 173,223 | 55,323 | 117,900 | 112,827 | 5,073 |
| 9 | 154,740 | 32,211 | 122,529 | 117,430 | 5,099 |
| 5 - 9 | 924,395 | 487,237 | 437,158 | 414,763 | 22,395 |
| 10 | 224,468 | 38,853 | 185,615 | 176,931 | 8,684 |
| 11 | 143,497 | 17,455 | 126,042 | 119,892 | 6,150 |
| 12 | 174,471 | 17,736 | 156,735 | 148,085 | 8,650 |
| 13 | 149,718 | 12,301 | 137,417 | 128,932 | 8,485 |
| 14 | 148,680 | 11,361 | 137,319 | 126,519 | 10,800 |
| 10 - 14 | 840,834 | 97,706 | 743,128 | 700,359 | 42,769 |
| 15 | 155,147 | 12,118 | 143,029 | 127,681 | 15,348 |
| 16 | 143,640 | 9,912 | 133,728 | 115,478 | 18,250 |
| 17 | 126,334 | 8,239 | 118,095 | 94,672 | 23,423 |
| 18 | 149,250 | 10,779 | 138,471 | 97,402 | 41,069 |
| 19 | 118,488 | 8,789 | 109,699 | 63,447 | 46,252 |
| 15 - 19 | 692,859 | 49,837 | 643,022 | 498,680 | 144,342 |
| 20 - 24 | 504,876 | 45,944 | 458,932 | 122,776 | 336,156 |
| 25 - 29 | 464,493 | 50,231 | 414,262 | 25,846 | 388,416 |
| 30 - 34 | 397,694 | 42,470 | 355,224 | 12,614 | 342,610 |
| 35 - 39 | 332,961 | 35,366 | 297,595 | 9,073 | 288,522 |
| 40 - 44 | 232,253 | 25,243 | 207,010 | 5,792 | 201,218 |
| 45 - 49 | 174,833 | 19,855 | 154,978 | 3,701 | 151,277 |
| 50 - 54 | 128,696 | 14,895 | 113,801 | 2,427 | 111,374 |
| 55 - 59 | 90,037 | 11,024 | 79,013 | 1,565 | 77,448 |
| 60 - 64 | 73,419 | 11,746 | 61,673 | 1,125 | 60,548 |
| 65 + | 157,631 | 41,011 | 116,620 | 2,785 | 113,835 |

## Table D2 i: Population (De Facto) 5 Years and Older by Age (Single and Grouped) School Attendance and Rural/Urban, Zambia 2010

| Age and Sex | Population 5 Years and Older | Never Attended | Ever Attended | | |
|---|---|---|---|---|---|
| | | | Total | Currently Attending | Not Currently Attending |
| **Female** | **5,296,446** | **1,253,993** | **4,042,453** | **1,720,532** | **2,321,921** |
| 5 | 202,583 | 162,601 | 39,982 | 36,632 | 3,350 |
| 6 | 199,313 | 135,242 | 64,071 | 60,082 | 3,989 |
| 7 | 193,604 | 87,216 | 106,388 | 101,478 | 4,910 |
| 8 | 178,242 | 52,029 | 126,213 | 120,891 | 5,322 |
| 9 | 158,199 | 29,535 | 128,664 | 123,508 | 5,156 |
| 5 - 9 | 931,941 | 466,623 | 465,318 | 442,591 | 22,727 |
| 10 | 225,024 | 34,585 | 190,439 | 181,984 | 8,455 |
| 11 | 147,080 | 15,685 | 131,395 | 124,938 | 6,457 |
| 12 | 176,582 | 15,760 | 160,822 | 151,690 | 9,132 |
| 13 | 155,410 | 11,619 | 143,791 | 134,358 | 9,433 |
| 14 | 154,112 | 11,094 | 143,018 | 129,876 | 13,142 |
| 10 - 14 | 858,208 | 88,743 | 769,465 | 722,846 | 46,619 |
| 15 | 157,191 | 12,431 | 144,760 | 123,642 | 21,118 |
| 16 | 151,912 | 11,908 | 140,004 | 109,389 | 30,615 |
| 17 | 133,812 | 11,493 | 122,319 | 80,299 | 42,020 |
| 18 | 159,840 | 16,794 | 143,046 | 70,191 | 72,855 |
| 19 | 132,270 | 15,675 | 116,595 | 39,324 | 77,271 |
| 15 - 19 | 735,025 | 68,301 | 666,724 | 422,845 | 243,879 |
| 20 - 24 | 612,600 | 92,800 | 519,800 | 72,452 | 447,348 |
| 25 - 29 | 541,751 | 97,048 | 444,703 | 24,202 | 420,501 |
| 30 - 34 | 403,076 | 72,381 | 330,695 | 13,100 | 317,595 |
| 35 - 39 | 314,852 | 62,057 | 252,795 | 8,502 | 244,293 |
| 40 - 44 | 215,331 | 47,135 | 168,196 | 4,451 | 163,745 |
| 45 - 49 | 180,156 | 42,587 | 137,569 | 3,083 | 134,486 |
| 50 - 54 | 141,558 | 38,937 | 102,621 | 2,110 | 100,511 |
| 55 - 59 | 94,791 | 29,998 | 64,793 | 1,235 | 63,558 |
| 60 - 64 | 88,456 | 39,897 | 48,559 | 963 | 47,596 |
| 65 + | 178,701 | 107,486 | 71,215 | 2,152 | 69,063 |

## Table D2 i: Population (De Facto) 5 Years and Older by Age (Single and Grouped), School Attendance and Rural, Zambia 2010

| Age and Sex | Population 5 Years and Older | Never Attended | Ever Attended | | |
|---|---|---|---|---|---|
| | | | Total | Currently Attending | Not Currently Attending |
| **Zambia Rural** | **6,050,907** | **1,779,136** | **4,271,771** | **1,883,424** | **2,388,347** |
| 5 | 268,012 | 242,172 | 25,840 | 22,757 | 3,083 |
| 6 | 262,858 | 210,258 | 52,600 | 48,563 | 4,037 |
| 7 | 253,186 | 144,019 | 109,167 | 103,675 | 5,492 |
| 8 | 229,514 | 90,120 | 139,394 | 133,063 | 6,331 |
| 9 | 198,677 | 52,433 | 146,244 | 139,901 | 6,343 |
| 5 - 9 | 1,212,247 | 739,002 | 473,245 | 447,959 | 25,286 |
| 10 | 291,852 | 63,290 | 228,562 | 217,465 | 11,097 |
| 11 | 179,931 | 28,762 | 151,169 | 143,046 | 8,123 |
| 12 | 216,717 | 28,936 | 187,781 | 176,272 | 11,509 |
| 13 | 180,849 | 20,608 | 160,241 | 148,953 | 11,288 |
| 14 | 176,628 | 19,401 | 157,227 | 141,933 | 15,294 |
| 10 - 14 | 1,045,977 | 160,997 | 884,980 | 827,669 | 57,311 |
| 15 | 184,001 | 21,264 | 162,737 | 138,856 | 23,881 |
| 16 | 169,390 | 18,971 | 150,419 | 118,597 | 31,822 |
| 17 | 142,117 | 16,997 | 125,120 | 84,271 | 40,849 |
| 18 | 172,292 | 23,791 | 148,501 | 81,138 | 67,363 |
| 19 | 135,197 | 20,994 | 114,203 | 47,477 | 66,726 |
| 15 - 19 | 802,997 | 102,017 | 700,980 | 470,339 | 230,641 |
| 20 - 24 | 598,270 | 118,003 | 480,267 | 87,061 | 393,206 |
| 25 - 29 | 529,481 | 125,064 | 404,417 | 19,114 | 385,303 |
| 30 - 34 | 419,932 | 97,101 | 322,831 | 9,244 | 313,587 |
| 35 - 39 | 351,970 | 82,414 | 269,556 | 6,604 | 262,952 |
| 40 - 44 | 254,964 | 61,561 | 193,403 | 4,111 | 189,292 |
| 45 - 49 | 209,868 | 52,823 | 157,045 | 3,113 | 153,932 |
| 50 - 54 | 162,395 | 44,677 | 117,718 | 2,329 | 115,389 |
| 55 - 59 | 111,511 | 33,543 | 77,968 | 1,473 | 76,495 |
| 60 - 64 | 107,038 | 42,286 | 64,752 | 1,213 | 63,539 |
| 65 + | 244,257 | 119,648 | 124,609 | 3,195 | 121,414 |

## Table D2i: Population (De Facto) 5 Years and Older by Age (Single and Grouped), School Attendance and Rural, Zambia 2010

| Age and Sex | Population 5 Years and Older | Never Attended | Ever Attended | | |
|---|---|---|---|---|---|
| | | | Total | Currently Attending | Not Currently Attending |
| **Male** | | | | | |
| **TOTAL** | **2,940,191** | **762,704** | **2,177,487** | **991,386** | **1,186,101** |
| 5 | 135,175 | 122,692 | 12,483 | 10,914 | 1,569 |
| 6 | 131,568 | 106,824 | 24,744 | 22,734 | 2,010 |
| 7 | 127,589 | 74,860 | 52,729 | 49,984 | 2,745 |
| 8 | 114,279 | 46,661 | 67,618 | 64,494 | 3,124 |
| 9 | 99,630 | 27,531 | 72,099 | 68,909 | 3,190 |
| 5 - 9 | 608,241 | 378,568 | 229,673 | 217,035 | 12,638 |
| 10 | 147,733 | 33,734 | 113,999 | 108,275 | 5,724 |
| 11 | 90,703 | 15,339 | 75,364 | 71,290 | 4,074 |
| 12 | 110,395 | 15,539 | 94,856 | 88,993 | 5,863 |
| 13 | 91,449 | 10,857 | 80,592 | 75,046 | 5,546 |
| 14 | 89,698 | 10,001 | 79,697 | 72,600 | 7,097 |
| 10 - 14 | 529,978 | 85,470 | 444,508 | 416,204 | 28,304 |
| 15 | 94,505 | 10,666 | 83,839 | 73,613 | 10,226 |
| 16 | 85,163 | 8,727 | 76,436 | 64,483 | 11,953 |
| 17 | 70,491 | 7,138 | 63,353 | 49,051 | 14,302 |
| 18 | 83,910 | 9,288 | 74,622 | 50,979 | 23,643 |
| 19 | 63,669 | 7,509 | 56,160 | 31,953 | 24,207 |
| 15 - 19 | 397,738 | 43,328 | 354,410 | 270,079 | 84,331 |
| 20 - 24 | 269,368 | 39,164 | 230,204 | 60,088 | 170,116 |
| 25 - 29 | 241,136 | 42,940 | 198,196 | 10,841 | 187,355 |
| 30 - 34 | 203,507 | 36,460 | 167,047 | 4,904 | 162,143 |
| 35 - 39 | 173,650 | 30,272 | 143,378 | 3,471 | 139,907 |
| 40 - 44 | 127,044 | 21,800 | 105,244 | 2,388 | 102,856 |
| 45 - 49 | 100,903 | 17,293 | 83,610 | 1,733 | 81,877 |
| 50 - 54 | 75,868 | 12,888 | 62,980 | 1,258 | 61,722 |
| 55 - 59 | 52,225 | 9,560 | 42,665 | 816 | 41,849 |
| 60 - 64 | 45,761 | 10,065 | 35,696 | 663 | 35,033 |
| 65 + | 114,772 | 34,896 | 79,876 | 1,906 | 77,970 |

## Table D2i: Population (De Facto) 5 Years and Older by Age (Single and Grouped), School Attendance and Rural, Zambia 2010

| Age and Sex | Population 5 Years and Older | Never Attended | Ever Attended | | |
|---|---|---|---|---|---|
| | | | Total | Currently Attending | Not Currently Attending |
| **Female** | | | | | |
| **TOTAL** | **3,110,716** | **1,016,432** | **2,094,284** | **892,038** | **1,202,246** |
| 5 | 132,837 | 119,480 | 13,357 | 11,843 | 1,514 |
| 6 | 131,290 | 103,434 | 27,856 | 25,829 | 2,027 |
| 7 | 125,597 | 69,159 | 56,438 | 53,691 | 2,747 |
| 8 | 115,235 | 43,459 | 71,776 | 68,569 | 3,207 |
| 9 | 99,047 | 24,902 | 74,145 | 70,992 | 3,153 |
| 5 - 9 | 604,006 | 360,434 | 243,572 | 230,924 | 12,648 |
| 10 | 144,119 | 29,556 | 114,563 | 109,190 | 5,373 |
| 11 | 89,228 | 13,423 | 75,805 | 71,756 | 4,049 |
| 12 | 106,322 | 13,397 | 92,925 | 87,279 | 5,646 |
| 13 | 89,400 | 9,751 | 79,649 | 73,907 | 5,742 |
| 14 | 86,930 | 9,400 | 77,530 | 69,333 | 8,197 |
| 10 - 14 | 515,999 | 75,527 | 440,472 | 411,465 | 29,007 |
| 15 | 89,496 | 10,598 | 78,898 | 65,243 | 13,655 |
| 16 | 84,227 | 10,244 | 73,983 | 54,114 | 19,869 |
| 17 | 71,626 | 9,859 | 61,767 | 35,220 | 26,547 |
| 18 | 88,382 | 14,503 | 73,879 | 30,159 | 43,720 |
| 19 | 71,528 | 13,485 | 58,043 | 15,524 | 42,519 |
| 15 - 19 | 405,259 | 58,689 | 346,570 | 200,260 | 146,310 |
| 20 - 24 | 328,902 | 78,839 | 250,063 | 26,973 | 223,090 |
| 25 - 29 | 288,345 | 82,124 | 206,221 | 8,273 | 197,948 |
| 30 - 34 | 216,425 | 60,641 | 155,784 | 4,340 | 151,444 |
| 35 - 39 | 178,320 | 52,142 | 126,178 | 3,133 | 123,045 |
| 40 - 44 | 127,920 | 39,761 | 88,159 | 1,723 | 86,436 |
| 45 - 49 | 108,965 | 35,530 | 73,435 | 1,380 | 72,055 |
| 50 - 54 | 86,527 | 31,789 | 54,738 | 1,071 | 53,667 |
| 55 - 59 | 59,286 | 23,983 | 35,303 | 657 | 34,646 |
| 60 - 64 | 61,277 | 32,221 | 29,056 | 550 | 28,506 |
| 65 + | 129,485 | 84,752 | 44,733 | 1,289 | 43,444 |

## Table D2i: Population (De Facto) 5 Years and Older by Age (Single and Grouped), School Attendance and Urban/Rural, Zambia 2010

| Age and Sex | Population 5 Years and Older | Never Attended | Ever Attended | | |
|---|---|---|---|---|---|
| | | | Total | Currently Attending | Not Currently Attending |
| **Zambia Urban** | **4,260,520** | **407,422** | **3,853,098** | **1,638,614** | **2,214,484** |
| 5 | 138,821 | 86,806 | 52,015 | 48,381 | 3,634 |
| 6 | 133,936 | 64,426 | 69,510 | 65,513 | 3,997 |
| 7 | 135,119 | 37,081 | 98,038 | 93,809 | 4,229 |
| 8 | 121,951 | 17,232 | 104,719 | 100,655 | 4,064 |
| 9 | 114,262 | 9,313 | 104,949 | 101,037 | 3,912 |
| 5 - 9 | 644,089 | 214,858 | 429,231 | 409,395 | 19,836 |
| 10 | 157,640 | 10,148 | 147,492 | 141,450 | 6,042 |
| 11 | 110,646 | 4,378 | 106,268 | 101,784 | 4,484 |
| 12 | 134,336 | 4,560 | 129,776 | 123,503 | 6,273 |
| 13 | 124,279 | 3,312 | 120,967 | 114,337 | 6,630 |
| 14 | 126,164 | 3,054 | 123,110 | 114,462 | 8,648 |
| 10 - 14 | 653,065 | 25,452 | 627,613 | 595,536 | 32,077 |
| 15 | 128,337 | 3,285 | 125,052 | 112,467 | 12,585 |
| 16 | 126,162 | 2,849 | 123,313 | 106,270 | 17,043 |
| 17 | 118,029 | 2,735 | 115,294 | 90,700 | 24,594 |
| 18 | 136,798 | 3,782 | 133,016 | 86,455 | 46,561 |
| 19 | 115,561 | 3,470 | 112,091 | 55,294 | 56,797 |
| 15 - 19 | 624,887 | 16,121 | 608,766 | 451,186 | 157,580 |
| 20 - 24 | 519,206 | 20,741 | 498,465 | 108,167 | 390,298 |
| 25 - 29 | 476,763 | 22,215 | 454,548 | 30,934 | 423,614 |
| 30 - 34 | 380,838 | 17,750 | 363,088 | 16,470 | 346,618 |
| 35 - 39 | 295,843 | 15,009 | 280,834 | 10,971 | 269,863 |
| 40 - 44 | 192,620 | 10,817 | 181,803 | 6,132 | 175,671 |
| 45 - 49 | 145,121 | 9,619 | 135,502 | 3,671 | 131,831 |
| 50 - 54 | 107,859 | 9,155 | 98,704 | 2,208 | 96,496 |
| 55 - 59 | 73,317 | 7,479 | 65,838 | 1,327 | 64,511 |
| 60 - 64 | 54,837 | 9,357 | 45,480 | 875 | 44,605 |
| 65 + | 92,075 | 28,849 | 63,226 | 1,742 | 61,484 |

## Table D2i: Population (De Facto) 5 Years and Older by Age (Single and Grouped), School Attendance and Urban, Zambia 2010

| Age and Sex | Population 5 Years and Older | Never Attended | Ever Attended | | |
|---|---|---|---|---|---|
| | | | Total | Currently Attending | Not Currently Attending |
| **Male** | | | | | |
| **TOTAL** | **2,074,790** | **169,861** | **1,904,929** | **810,120** | **1,094,809** |
| 5 | 69,075 | 43,685 | 25,390 | 23,592 | 1,798 |
| 6 | 65,913 | 32,618 | 33,295 | 31,260 | 2,035 |
| 7 | 67,112 | 19,024 | 48,088 | 46,022 | 2,066 |
| 8 | 58,944 | 8,662 | 50,282 | 48,333 | 1,949 |
| 9 | 55,110 | 4,680 | 50,430 | 48,521 | 1,909 |
| 5 - 9 | 316,154 | 108,669 | 207,485 | 197,728 | 9,757 |
| 10 | 76,735 | 5,119 | 71,616 | 68,656 | 2,960 |
| 11 | 52,794 | 2,116 | 50,678 | 48,602 | 2,076 |
| 12 | 64,076 | 2,197 | 61,879 | 59,092 | 2,787 |
| 13 | 58,269 | 1,444 | 56,825 | 53,886 | 2,939 |
| 14 | 58,982 | 1,360 | 57,622 | 53,919 | 3,703 |
| 10 - 14 | 310,856 | 12,236 | 298,620 | 284,155 | 14,465 |
| 15 | 60,642 | 1,452 | 59,190 | 54,068 | 5,122 |
| 16 | 58,477 | 1,185 | 57,292 | 50,995 | 6,297 |
| 17 | 55,843 | 1,101 | 54,742 | 45,621 | 9,121 |
| 18 | 65,340 | 1,491 | 63,849 | 46,423 | 17,426 |
| 19 | 54,819 | 1,280 | 53,539 | 31,494 | 22,045 |
| 15 - 19 | 295,121 | 6,509 | 288,612 | 228,601 | 60,011 |
| 20 - 24 | 235,508 | 6,780 | 228,728 | 62,688 | 166,040 |
| 25 - 29 | 223,357 | 7,291 | 216,066 | 15,005 | 201,061 |
| 30 - 34 | 194,187 | 6,010 | 188,177 | 7,710 | 180,467 |
| 35 - 39 | 159,311 | 5,094 | 154,217 | 5,602 | 148,615 |
| 40 - 44 | 105,209 | 3,443 | 101,766 | 3,404 | 98,362 |
| 45 - 49 | 73,930 | 2,562 | 71,368 | 1,968 | 69,400 |
| 50 - 54 | 52,828 | 2,007 | 50,821 | 1,169 | 49,652 |
| 55 - 59 | 37,812 | 1,464 | 36,348 | 749 | 35,599 |
| 60 - 64 | 27,658 | 1,681 | 25,977 | 462 | 25,515 |
| 65 + | 42,859 | 6,115 | 36,744 | 879 | 35,865 |

## Table D2 i: Population (De Facto) 5 Years and Older by Age (Single and Grouped), School Attendance and Urban, Zambia 2010

| Age and Sex | Population 5 Years and Older | Never Attended | Ever Attended | | |
|---|---|---|---|---|---|
| | | | Total | Currently Attending | Not Currently Attending |
| **Female** | | | | | |
| **TOTAL** | **2,185,730** | **237,561** | **1,948,169** | **828,494** | **1,119,675** |
| 5 | 69,746 | 43,121 | 26,625 | 24,789 | 1,836 |
| 6 | 68,023 | 31,808 | 36,215 | 34,253 | 1,962 |
| 7 | 68,007 | 18,057 | 49,950 | 47,787 | 2,163 |
| 8 | 63,007 | 8,570 | 54,437 | 52,322 | 2,115 |
| 9 | 59,152 | 4,633 | 54,519 | 52,516 | 2,003 |
| 5 - 9 | 327,935 | 106,189 | 221,746 | 211,667 | 10,079 |
| 10 | 80,905 | 5,029 | 75,876 | 72,794 | 3,082 |
| 11 | 57,852 | 2,262 | 55,590 | 53,182 | 2,408 |
| 12 | 70,260 | 2,363 | 67,897 | 64,411 | 3,486 |
| 13 | 66,010 | 1,868 | 64,142 | 60,451 | 3,691 |
| 14 | 67,182 | 1,694 | 65,488 | 60,543 | 4,945 |
| 10 - 14 | 342,209 | 13,216 | 328,993 | 311,381 | 17,612 |
| 15 | 67,695 | 1,833 | 65,862 | 58,399 | 7,463 |
| 16 | 67,685 | 1,664 | 66,021 | 55,275 | 10,746 |
| 17 | 62,186 | 1,634 | 60,552 | 45,079 | 15,473 |
| 18 | 71,458 | 2,291 | 69,167 | 40,032 | 29,135 |
| 19 | 60,742 | 2,190 | 58,552 | 23,800 | 34,752 |
| 15 - 19 | 329,766 | 9,612 | 320,154 | 222,585 | 97,569 |
| 20 - 24 | 283,698 | 13,961 | 269,737 | 45,479 | 224,258 |
| 25 - 29 | 253,406 | 14,924 | 238,482 | 15,929 | 222,553 |
| 30 - 34 | 186,651 | 11,740 | 174,911 | 8,760 | 166,151 |
| 35 - 39 | 136,532 | 9,915 | 126,617 | 5,369 | 121,248 |
| 40 - 44 | 87,411 | 7,374 | 80,037 | 2,728 | 77,309 |
| 45 - 49 | 71,191 | 7,057 | 64,134 | 1,703 | 62,431 |
| 50 - 54 | 55,031 | 7,148 | 47,883 | 1,039 | 46,844 |
| 55 - 59 | 35,505 | 6,015 | 29,490 | 578 | 28,912 |
| 60 - 64 | 27,179 | 7,676 | 19,503 | 413 | 19,090 |
| 65 + | 49,216 | 22,734 | 26,482 | 863 | 25,619 |

## Table D3: Population (De Facto) 5 Years and Older by Age (Single and Grouped), Sex, Literacy Rate and Rural/Urban, Zambia 2010

| Age | Population 5 Years and Older | Can Read & Write | | | Cannot Read & Write | | |
|---|---|---|---|---|---|---|---|
| | | Total | Male | Female | Total | Male | Female |
| **Zambia Total** | **10,311,427** | **7,234,619** | **3,670,314** | **3,564,305** | **3,076,808** | **1,344,667** | **1,732,141** |
| 5 | 406,833 | 22,923 | 11,124 | 11,799 | 383,910 | 193,126 | 190,784 |
| 6 | 396,794 | 34,379 | 16,460 | 17,919 | 362,415 | 181,021 | 181,394 |
| 7 | 388,305 | 59,997 | 29,193 | 30,804 | 328,308 | 165,508 | 162,800 |
| 8 | 351,465 | 90,424 | 42,780 | 47,644 | 261,041 | 130,443 | 130,598 |
| 9 | 312,939 | 132,378 | 63,071 | 69,307 | 180,561 | 91,669 | 88,892 |
| 5 - 9 | 1,856,336 | 340,101 | 162,628 | 177,473 | 1,516,235 | 761,767 | 754,468 |
| 10 | 449,492 | 251,325 | 121,927 | 129,398 | 198,167 | 102,541 | 95,626 |
| 11 | 290,577 | 212,594 | 102,758 | 109,836 | 77,983 | 40,739 | 37,244 |
| 12 | 351,053 | 285,127 | 139,469 | 145,658 | 65,926 | 35,002 | 30,924 |
| 13 | 305,128 | 265,567 | 128,860 | 136,707 | 39,561 | 20,858 | 18,703 |
| 14 | 302,792 | 271,866 | 132,661 | 139,205 | 30,926 | 16,019 | 14,907 |
| 10 - 14 | 1,699,042 | 1,286,479 | 625,675 | 660,804 | 412,563 | 215,159 | 197,404 |
| 15 | 312,338 | 282,236 | 139,945 | 142,291 | 30,102 | 15,202 | 14,900 |
| 16 | 295,552 | 270,500 | 132,052 | 138,448 | 25,052 | 11,588 | 13,464 |
| 17 | 260,146 | 237,909 | 117,070 | 120,839 | 22,237 | 9,264 | 12,973 |
| 18 | 309,090 | 278,883 | 137,622 | 141,261 | 30,207 | 11,628 | 18,579 |
| 19 | 250,758 | 224,064 | 109,165 | 114,899 | 26,694 | 9,323 | 17,371 |
| 15 - 19 | 1,427,884 | 1,293,592 | 635,854 | 657,738 | 134,292 | 57,005 | 77,287 |
| 20 - 24 | 1,117,476 | 964,513 | 456,428 | 508,085 | 152,963 | 48,448 | 104,515 |
| 25 - 29 | 1,006,244 | 840,510 | 410,872 | 429,638 | 165,734 | 53,621 | 112,113 |
| 30 - 34 | 800,770 | 673,721 | 352,732 | 320,989 | 127,049 | 44,962 | 82,087 |
| 35 - 39 | 647,813 | 541,253 | 295,834 | 245,419 | 106,560 | 37,127 | 69,433 |
| 40 - 44 | 447,584 | 369,981 | 206,427 | 163,554 | 77,603 | 25,826 | 51,777 |
| 45 - 49 | 354,989 | 288,808 | 154,804 | 134,004 | 66,181 | 20,029 | 46,152 |
| 50 - 54 | 270,254 | 213,161 | 113,867 | 99,294 | 57,093 | 14,829 | 42,264 |
| 55 - 59 | 184,828 | 140,814 | 78,969 | 61,845 | 44,014 | 11,068 | 32,946 |
| 60 - 64 | 161,875 | 105,635 | 61,354 | 44,281 | 56,240 | 12,065 | 44,175 |
| 65 + | 336,332 | 176,051 | 114,870 | 61,181 | 160,281 | 42,761 | 117,520 |

## Table D3: Population (De Facto) 5 Years and Older by Age (Single and Grouped), Sex, Literacy Rate and Rural, Zambia 2010

| Age | Population 5 Years and Older | Can Read & Write | | | Cannot Read & Write | | |
|---|---|---|---|---|---|---|---|
| | | Total | Male | Female | Total | Male | Female |
| **Zambia Rural** | **6,050,907** | **3,662,469** | **1,899,222** | **1,763,247** | **2,388,438** | **1,040,969** | **1,347,469** |
| 5 | 268,012 | 7,306 | 3,586 | 3,720 | 260,706 | 131,589 | 129,117 |
| 6 | 262,858 | 11,351 | 5,451 | 5,900 | 251,507 | 126,117 | 125,390 |
| 7 | 253,186 | 22,831 | 11,178 | 11,653 | 230,355 | 116,411 | 113,944 |
| 8 | 229,514 | 38,162 | 18,245 | 19,917 | 191,352 | 96,034 | 95,318 |
| 9 | 198,677 | 60,875 | 29,422 | 31,453 | 137,802 | 70,208 | 67,594 |
| 5 - 9 | 1,212,247 | 140,525 | 67,882 | 72,643 | 1,071,722 | 540,359 | 531,363 |
| 10 | 291,852 | 131,066 | 64,109 | 66,957 | 160,786 | 83,624 | 77,162 |
| 11 | 179,931 | 114,318 | 56,141 | 58,177 | 65,613 | 34,562 | 31,051 |
| 12 | 216,717 | 160,316 | 80,150 | 80,166 | 56,401 | 30,245 | 26,156 |
| 13 | 180,849 | 146,623 | 73,054 | 73,569 | 34,226 | 18,395 | 15,831 |
| 14 | 176,628 | 149,701 | 75,536 | 74,165 | 26,927 | 14,162 | 12,765 |
| 10 - 14 | 1,045,977 | 702,024 | 348,990 | 353,034 | 343,953 | 180,988 | 162,965 |
| 15 | 184,001 | 157,700 | 80,993 | 76,707 | 26,301 | 13,512 | 12,789 |
| 16 | 169,390 | 147,442 | 74,871 | 72,571 | 21,948 | 10,292 | 11,656 |
| 17 | 142,117 | 122,793 | 62,414 | 60,379 | 19,324 | 8,077 | 11,247 |
| 18 | 172,292 | 145,936 | 73,785 | 72,151 | 26,356 | 10,125 | 16,231 |
| 19 | 135,197 | 112,065 | 55,594 | 56,471 | 23,132 | 8,075 | 15,057 |
| 15 - 19 | 802,997 | 685,936 | 347,657 | 338,279 | 117,061 | 50,081 | 66,980 |
| 20 - 24 | 598,270 | 466,722 | 227,556 | 239,166 | 131,548 | 41,812 | 89,736 |
| 25 - 29 | 529,481 | 387,414 | 194,623 | 192,791 | 142,067 | 46,513 | 95,554 |
| 30 - 34 | 419,932 | 311,351 | 164,430 | 146,921 | 108,581 | 39,077 | 69,504 |
| 35 - 39 | 351,970 | 260,745 | 141,395 | 119,350 | 91,225 | 32,255 | 58,970 |
| 40 - 44 | 254,964 | 188,202 | 104,407 | 83,795 | 66,762 | 22,637 | 44,125 |
| 45 - 49 | 209,868 | 153,355 | 83,235 | 70,120 | 56,513 | 17,668 | 38,845 |
| 50 - 54 | 162,395 | 114,713 | 62,887 | 51,826 | 47,682 | 12,981 | 34,701 |
| 55 - 59 | 111,511 | 75,459 | 42,539 | 32,920 | 36,052 | 9,686 | 26,366 |
| 60 - 64 | 107,038 | 60,941 | 35,322 | 25,619 | 46,097 | 10,439 | 35,658 |
| 65 + | 244,257 | 115,082 | 78,299 | 36,783 | 129,175 | 36,473 | 92,702 |

## Table D3: Population (De Facto) 5 Years and Older by Age (Single and Grouped), Sex, Literacy Rate and Urban, Zambia 2010

| Age | Population 5 Years and Older | Can Read & Write | | | Cannot Read & Write | | |
|---|---|---|---|---|---|---|---|
| | | Total | Male | Female | Total | Male | Female |
| **Zambia Urban** | **4,260,520** | **3,572,150** | **1,771,092** | **1,801,058** | **688,370** | **303,698** | **384,672** |
| 5 | 138,821 | 15,617 | 7,538 | 8,079 | 123,204 | 61,537 | 61,667 |
| 6 | 133,936 | 23,028 | 11,009 | 12,019 | 110,908 | 54,904 | 56,004 |
| 7 | 135,119 | 37,166 | 18,015 | 19,151 | 97,953 | 49,097 | 48,856 |
| 8 | 121,951 | 52,262 | 24,535 | 27,727 | 69,689 | 34,409 | 35,280 |
| 9 | 114,262 | 71,503 | 33,649 | 37,854 | 42,759 | 21,461 | 21,298 |
| 5 - 9 | 644,089 | 199,576 | 94,746 | 104,830 | 444,513 | 221,408 | 223,105 |
| 10 | 157,640 | 120,259 | 57,818 | 62,441 | 37,381 | 18,917 | 18,464 |
| 11 | 110,646 | 98,276 | 46,617 | 51,659 | 12,370 | 6,177 | 6,193 |
| 12 | 134,336 | 124,811 | 59,319 | 65,492 | 9,525 | 4,757 | 4,768 |
| 13 | 124,279 | 118,944 | 55,806 | 63,138 | 5,335 | 2,463 | 2,872 |
| 14 | 126,164 | 122,165 | 57,125 | 65,040 | 3,999 | 1,857 | 2,142 |
| 10 - 14 | 653,065 | 584,455 | 276,685 | 307,770 | 68,610 | 34,171 | 34,439 |
| 15 | 128,337 | 124,536 | 58,952 | 65,584 | 3,801 | 1,690 | 2,111 |
| 16 | 126,162 | 123,058 | 57,181 | 65,877 | 3,104 | 1,296 | 1,808 |
| 17 | 118,029 | 115,116 | 54,656 | 60,460 | 2,913 | 1,187 | 1,726 |
| 18 | 136,798 | 132,947 | 63,837 | 69,110 | 3,851 | 1,503 | 2,348 |
| 19 | 115,561 | 111,999 | 53,571 | 58,428 | 3,562 | 1,248 | 2,314 |
| 15 - 19 | 624,887 | 607,656 | 288,197 | 319,459 | 17,231 | 6,924 | 10,307 |
| 20 - 24 | 519,206 | 497,791 | 228,872 | 268,919 | 21,415 | 6,636 | 14,779 |
| 25 - 29 | 476,763 | 453,096 | 216,249 | 236,847 | 23,667 | 7,108 | 16,559 |
| 30 - 34 | 380,838 | 362,370 | 188,302 | 174,068 | 18,468 | 5,885 | 12,583 |
| 35 - 39 | 295,843 | 280,508 | 154,439 | 126,069 | 15,335 | 4,872 | 10,463 |
| 40 - 44 | 192,620 | 181,779 | 102,020 | 79,759 | 10,841 | 3,189 | 7,652 |
| 45 - 49 | 145,121 | 135,453 | 71,569 | 63,884 | 9,668 | 2,361 | 7,307 |
| 50 - 54 | 107,859 | 98,448 | 50,980 | 47,468 | 9,411 | 1,848 | 7,563 |
| 55 - 59 | 73,317 | 65,355 | 36,430 | 28,925 | 7,962 | 1,382 | 6,580 |
| 60 - 64 | 54,837 | 44,694 | 26,032 | 18,662 | 10,143 | 1,626 | 8,517 |
| 65 + | 92,075 | 60,969 | 36,571 | 24,398 | 31,106 | 6,288 | 24,818 |

## Table D4: Population (De Facto) 5 Years and Older by Age (Single and Grouped), Sex, Highest Level of Education Completed and Rural/Urban, Zambia 2010

| Age and Sex | Population 5 Years and Older | Total Never Attended | Total Ever Attended | Highest Level of Education Completed (Among those Ever Attended School) | | | | | | | | | | | | | | | | |
|---|---|---|---|---|---|---|---|---|---|---|---|---|---|---|---|---|---|---|---|---|
| | | | | | Primary School Grade 1 - 7 | | | | | | | | Secondary School Grade 8 - 12 | | | | | | |
| | | | | None | Total | 1 | 2 | 3 | 4 | 5 | 6 | 7 | Total | 8 | 9 | 10 | 11 | 12 | Tertiary |
| **Zambia Total** | | | | | | | | | | | | | | | | | | | | |
| **Total** | **10,311,427** | **2,186,558** | **8,124,869** | **276,965** | **4,709,131** | **498,893** | **552,736** | **569,068** | **621,868** | **619,734** | **703,846** | **1,142,986** | **2,577,104** | **602,677** | **849,771** | **285,069** | **205,883** | **633,704** | **561,669** |
| Male | 5,014,981 | 932,565 | 4,082,416 | 135,182 | 2,191,918 | 238,492 | 255,600 | 259,508 | 281,328 | 281,353 | 328,627 | 547,010 | 1,411,384 | 300,502 | 441,109 | 162,652 | 118,452 | 388,669 | 343,932 |
| Female | 5,296,446 | 1,253,993 | 4,042,453 | 141,783 | 2,517,213 | 260,401 | 297,136 | 309,560 | 340,540 | 338,381 | 375,219 | 595,976 | 1,165,720 | 302,175 | 408,662 | 122,417 | 87,431 | 245,035 | 217,737 |
| **5** | | | | | | | | | | | | | | | | | | | | |
| **Total** | **406,833** | **328,978** | **77,855** | **56,827** | **21,028** | **21,028** | **-** | **-** | **-** | **-** | **-** | **-** | **-** | **-** | **-** | **-** | **-** | **-** | **-** |
| Male | 204,250 | 166,377 | 37,873 | 27,721 | 10,152 | 10,152 | - | - | - | - | - | - | - | - | - | - | - | - | - |
| Female | 202,583 | 162,601 | 39,982 | 29,106 | 10,876 | 10,876 | - | - | - | - | - | - | - | - | - | - | - | - | - |
| **6** | | | | | | | | | | | | | | | | | | | | |
| **Total** | **396,794** | **274,684** | **122,110** | **59,890** | **62,220** | **52,319** | **9,901** | **-** | **-** | **-** | **-** | **-** | **-** | **-** | **-** | **-** | **-** | **-** | **-** |
| Male | 197,481 | 139,442 | 58,039 | 29,099 | 28,940 | 24,540 | 4,400 | - | - | - | - | - | - | - | - | - | - | - | - |
| Female | 199,313 | 135,242 | 64,071 | 30,791 | 33,280 | 27,779 | 5,501 | - | - | - | - | - | - | - | - | - | - | - | - |
| **7** | | | | | | | | | | | | | | | | | | | | |
| **Total** | **388,305** | **181,100** | **207,205** | **61,579** | **145,626** | **100,544** | **36,549** | **8,533** | **-** | **-** | **-** | **-** | **-** | **-** | **-** | **-** | **-** | **-** | **-** |
| Male | 194,701 | 93,884 | 100,817 | 30,596 | 70,221 | 49,246 | 16,961 | 4,014 | - | - | - | - | - | - | - | - | - | - | - |
| Female | 193,604 | 87,216 | 106,388 | 30,983 | 75,405 | 51,298 | 19,588 | 4,519 | - | - | - | - | - | - | - | - | - | - | - |
| **8** | | | | | | | | | | | | | | | | | | | | |
| **Total** | **351,465** | **107,352** | **244,113** | **37,074** | **207,039** | **95,435** | **75,474** | **28,751** | **7,379** | **-** | **-** | **-** | **-** | **-** | **-** | **-** | **-** | **-** | **-** |
| Male | 173,223 | 55,323 | 117,900 | 18,483 | 99,417 | 47,339 | 35,767 | 12,990 | 3,321 | - | - | - | - | - | - | - | - | - | - |
| Female | 178,242 | 52,029 | 126,213 | 18,591 | 107,622 | 48,096 | 39,707 | 15,761 | 4,058 | - | - | - | - | - | - | - | - | - | - |
| **9** | | | | | | | | | | | | | | | | | | | | |
| **Total** | **312,939** | **61,746** | **251,193** | **17,864** | **233,329** | **60,659** | **83,260** | **59,281** | **23,995** | **6,134** | **-** | **-** | **-** | **-** | **-** | **-** | **-** | **-** | **-** |
| Male | 154,740 | 32,211 | 122,529 | 9,219 | 113,310 | 30,864 | 40,905 | 27,769 | 10,942 | 2,830 | - | - | - | - | - | - | - | - | - |
| Female | 158,199 | 29,535 | 128,664 | 8,645 | 120,019 | 29,795 | 42,355 | 31,512 | 13,053 | 3,304 | - | - | - | - | - | - | - | - | - |
| **5 - 9** | | | | | | | | | | | | | | | | | | | | |
| **Total** | **1,856,336** | **953,860** | **902,476** | **233,234** | **669,242** | **329,985** | **205,184** | **96,565** | **31,374** | **6,134** | **-** | **-** | **-** | **-** | **-** | **-** | **-** | **-** | **-** |
| Male | 924,395 | 487,237 | 437,158 | 115,118 | 322,040 | 162,141 | 98,033 | 44,773 | 14,263 | 2,830 | - | - | - | - | - | - | - | - | - |
| Female | 931,941 | 466,623 | 465,318 | 118,116 | 347,202 | 167,844 | 107,151 | 51,792 | 17,111 | 3,304 | - | - | - | - | - | - | - | - | - |
| **10** | | | | | | | | | | | | | | | | | | | | |
| **Total** | **449,492** | **73,438** | **376,054** | **14,826** | **361,228** | **54,608** | **98,130** | **106,223** | **67,524** | **26,968** | **7,775** | **-** | **-** | **-** | **-** | **-** | **-** | **-** | **-** |
| Male | 224,468 | 38,853 | 185,615 | 7,706 | 177,909 | 28,200 | 49,449 | 52,028 | 32,160 | 12,400 | 3,672 | - | - | - | - | - | - | - | - |
| Female | 225,024 | 34,585 | 190,439 | 7,120 | 183,319 | 26,408 | 48,681 | 54,195 | 35,364 | 14,568 | 4,103 | - | - | - | - | - | - | - | - |
| **11** | | | | | | | | | | | | | | | | | | | | |
| **Total** | **290,577** | **33,140** | **257,437** | **4,338** | **253,099** | **17,213** | **39,721** | **62,424** | **65,440** | **43,796** | **18,659** | **5,846** | **-** | **-** | **-** | **-** | **-** | **-** | **-** |
| Male | 143,497 | 17,455 | 126,042 | 2,297 | 123,745 | 9,121 | 20,589 | 31,125 | 31,569 | 20,414 | 8,336 | 2,591 | - | - | - | - | - | - | - |
| Female | 147,080 | 15,685 | 131,395 | 2,041 | 129,354 | 8,092 | 19,132 | 31,299 | 33,871 | 23,382 | 10,323 | 3,255 | - | - | - | - | - | - | - |
| **12** | | | | | | | | | | | | | | | | | | | | |
| **Total** | **351,053** | **33,496** | **317,557** | **3,222** | **309,248** | **12,129** | **30,163** | **54,756** | **73,649** | **70,872** | **46,102** | **21,577** | **5,087** | **5,087** | **-** | **-** | **-** | **-** | **-** |
| Male | 174,471 | 17,736 | 156,735 | 1,687 | 152,775 | 6,600 | 16,031 | 27,972 | 36,858 | 34,217 | 21,351 | 9,746 | 2,273 | 2,273 | - | - | - | - | - |
| Female | 176,582 | 15,760 | 160,822 | 1,535 | 156,473 | 5,529 | 14,132 | 26,784 | 36,791 | 36,655 | 24,751 | 11,831 | 2,814 | 2,814 | - | - | - | - | - |
| **13** | | | | | | | | | | | | | | | | | | | | |
| **Total** | **305,128** | **23,920** | **281,208** | **1,761** | **260,908** | **5,802** | **15,212** | **30,905** | **49,597** | **62,481** | **57,378** | **39,533** | **18,539** | **14,512** | **4,027** | **-** | **-** | **-** | **-** |
| Male | 149,718 | 12,301 | 137,417 | 897 | 128,644 | 3,207 | 8,311 | 16,289 | 25,387 | 30,699 | 26,860 | 17,891 | 7,876 | 6,140 | 1,736 | - | - | - | - |
| Female | 155,410 | 11,619 | 143,791 | 864 | 132,264 | 2,595 | 6,901 | 14,616 | 24,210 | 31,782 | 30,518 | 21,642 | 10,663 | 8,372 | 2,291 | - | - | - | - |
| **14** | | | | | | | | | | | | | | | | | | | | |
| **Total** | **302,792** | **22,455** | **280,337** | **1,189** | **229,575** | **3,543** | **8,669** | **18,213** | **32,789** | **49,948** | **58,727** | **57,686** | **49,573** | **33,137** | **13,748** | **2,688** | **-** | **-** | **-** |
| Male | 148,680 | 11,361 | 137,319 | 629 | 115,150 | 1,987 | 4,787 | 10,124 | 17,428 | 25,391 | 28,596 | 26,837 | 21,540 | 14,519 | 5,876 | 1,145 | - | - | - |
| Female | 154,112 | 11,094 | 143,018 | 560 | 114,425 | 1,556 | 3,882 | 8,089 | 15,361 | 24,557 | 30,131 | 30,849 | 28,033 | 18,618 | 7,872 | 1,543 | - | - | - |
| **10 - 14** | | | | | | | | | | | | | | | | | | | | |
| **Total** | **1,699,042** | **186,449** | **1,512,593** | **25,336** | **1,414,058** | **93,295** | **191,895** | **272,521** | **288,999** | **254,065** | **188,641** | **124,642** | **73,199** | **52,736** | **17,775** | **2,688** | **-** | **-** | **-** |
| Male | 840,834 | 97,706 | 743,128 | 13,216 | 698,223 | 49,115 | 99,167 | 137,538 | 143,402 | 123,121 | 88,815 | 57,065 | 31,689 | 22,932 | 7,612 | 1,145 | - | - | - |
| Female | 858,208 | 88,743 | 769,465 | 12,120 | 715,835 | 44,180 | 92,728 | 134,983 | 145,597 | 130,944 | 99,826 | 67,577 | 41,510 | 29,804 | 10,163 | 1,543 | - | - | - |

## Table D4: Population (De Facto) 5 Years and Older by Age (Single and Grouped), Sex, Highest Level of Education Completed and Rural/Urban, Zambia 2010

| Age and Sex | Population 5 Years and Older | Total Never Attended | Total Ever Attended | Highest Level of Education Completed (Among those Ever Attended School) | | | | | | | | | | | | | | | |
|---|---|---|---|---|---|---|---|---|---|---|---|---|---|---|---|---|---|---|---|
| | | | | None | Primary School Grade 1 - 7 | | | | | | | | Secondary School Grade 8 - 12 | | | | | | Tertiary |
| | | | | | Total | 1 | 2 | 3 | 4 | 5 | 6 | 7 | Total | 8 | 9 | 10 | 11 | 12 | |
| **15** | | | | | | | | | | | | | | | | | | | |
| **Total** | **312,338** | **24,549** | **287,789** | **1,014** | **197,396** | **2,681** | **6,074** | **11,983** | **22,561** | **38,461** | **52,068** | **63,568** | **89,379** | **48,712** | **29,281** | **9,109** | **2,277** | **-** | **-** |
| Male | 155,147 | 12,118 | 143,029 | 553 | 102,480 | 1,481 | 3,391 | 6,717 | 12,525 | 20,564 | 26,680 | 31,122 | 39,996 | 22,130 | 13,007 | 3,941 | 918 | - | - |
| Female | 157,191 | 12,431 | 144,760 | 461 | 94,916 | 1,200 | 2,683 | 5,266 | 10,036 | 17,897 | 25,388 | 32,446 | 49,383 | 26,582 | 16,274 | 5,168 | 1,359 | - | - |
| **16** | | | | | | | | | | | | | | | | | | | |
| **Total** | **295,552** | **21,820** | **273,732** | **764** | **148,893** | **1,917** | **3,911** | **7,554** | **14,149** | **24,350** | **39,714** | **57,298** | **124,019** | **53,078** | **41,687** | **19,191** | **7,899** | **2,164** | **56** |
| Male | 143,640 | 9,912 | 133,728 | 376 | 76,935 | 955 | 2,049 | 3,999 | 7,760 | 13,110 | 20,774 | 28,288 | 56,399 | 24,808 | 18,905 | 8,529 | 3,247 | 910 | 18 |
| Female | 151,912 | 11,908 | 140,004 | 388 | 71,958 | 962 | 1,862 | 3,555 | 6,389 | 11,240 | 18,940 | 29,010 | 67,620 | 28,270 | 22,782 | 10,662 | 4,652 | 1,254 | 38 |
| **17** | | | | | | | | | | | | | | | | | | | |
| **Total** | **260,146** | **19,732** | **240,414** | **628** | **103,759** | **1,422** | **3,215** | **5,439** | **8,859** | **15,310** | **25,638** | **43,876** | **135,073** | **44,912** | **42,617** | **23,098** | **16,910** | **7,536** | **954** |
| Male | 126,334 | 8,239 | 118,095 | 331 | 52,851 | 634 | 1,417 | 2,616 | 4,489 | 7,900 | 13,569 | 22,226 | 64,498 | 22,162 | 20,103 | 11,438 | 7,687 | 3,108 | 415 |
| Female | 133,812 | 11,493 | 122,319 | 297 | 50,908 | 788 | 1,798 | 2,823 | 4,370 | 7,410 | 12,069 | 21,650 | 70,575 | 22,750 | 22,514 | 11,660 | 9,223 | 4,428 | 539 |
| **18** | | | | | | | | | | | | | | | | | | | |
| **Total** | **309,090** | **27,573** | **281,517** | **642** | **107,520** | **1,822** | **3,864** | **5,997** | **9,860** | **15,130** | **24,921** | **45,926** | **171,372** | **47,837** | **51,084** | **25,502** | **25,037** | **21,912** | **1,983** |
| Male | 149,250 | 10,779 | 138,471 | 294 | 51,852 | 756 | 1,594 | 2,564 | 4,420 | 7,131 | 12,437 | 22,950 | 85,481 | 24,700 | 24,991 | 13,630 | 12,570 | 9,590 | 844 |
| Female | 159,840 | 16,794 | 143,046 | 348 | 55,668 | 1,066 | 2,270 | 3,433 | 5,440 | 7,999 | 12,484 | 22,976 | 85,891 | 23,137 | 26,093 | 11,872 | 12,467 | 12,322 | 1,139 |
| **19** | | | | | | | | | | | | | | | | | | | |
| **Total** | **250,758** | **24,464** | **226,294** | **571** | **78,514** | **1,545** | **3,164** | **4,855** | **7,521** | **10,940** | **17,379** | **33,110** | **143,793** | **32,738** | **40,724** | **18,430** | **20,661** | **31,240** | **3,416** |
| Male | 118,488 | 8,789 | 109,699 | 252 | 34,848 | 561 | 1,153 | 1,833 | 2,987 | 4,644 | 8,071 | 15,599 | 73,166 | 16,926 | 20,020 | 10,271 | 11,324 | 14,625 | 1,433 |
| Female | 132,270 | 15,675 | 116,595 | 319 | 43,666 | 984 | 2,011 | 3,022 | 4,534 | 6,296 | 9,308 | 17,511 | 70,627 | 15,812 | 20,704 | 8,159 | 9,337 | 16,615 | 1,983 |
| **15 - 19** | | | | | | | | | | | | | | | | | | | |
| **Total** | **1,427,884** | **118,138** | **1,309,746** | **3,619** | **636,082** | **9,387** | **20,228** | **35,828** | **62,950** | **104,191** | **159,720** | **243,778** | **663,636** | **227,277** | **205,393** | **95,330** | **72,784** | **62,852** | **6,409** |
| Male | 692,859 | 49,837 | 643,022 | 1,806 | 318,966 | 4,387 | 9,604 | 17,729 | 32,181 | 53,349 | 81,531 | 120,185 | 319,540 | 110,726 | 97,026 | 47,809 | 35,746 | 28,233 | 2,710 |
| Female | 735,025 | 68,301 | 666,724 | 1,813 | 317,116 | 5,000 | 10,624 | 18,099 | 30,769 | 50,842 | 78,189 | 123,593 | 344,096 | 116,551 | 108,367 | 47,521 | 37,038 | 34,619 | 3,699 |
| **20 - 24** | | | | | | | | | | | | | | | | | | | |
| **Total** | **1,117,476** | **138,744** | **978,732** | **2,463** | **353,232** | **8,792** | **18,572** | **26,394** | **38,950** | **50,231** | **71,040** | **139,253** | **565,347** | **101,031** | **157,756** | **50,505** | **57,505** | **198,550** | **57,690** |
| Male | 504,876 | 45,944 | 458,932 | 937 | 138,361 | 2,888 | 5,766 | 8,384 | 13,449 | 18,097 | 28,527 | 61,250 | 293,488 | 49,406 | 77,181 | 28,483 | 33,242 | 105,176 | 26,146 |
| Female | 612,600 | 92,800 | 519,800 | 1,526 | 214,871 | 5,904 | 12,806 | 18,010 | 25,501 | 32,134 | 42,513 | 78,003 | 271,859 | 51,625 | 80,575 | 22,022 | 24,263 | 93,374 | 31,544 |
| **25 - 29** | | | | | | | | | | | | | | | | | | | |
| **Total** | **1,006,244** | **147,279** | **858,965** | **2,536** | **358,408** | **10,197** | **21,833** | **28,889** | **41,597** | **51,284** | **66,487** | **138,121** | **386,564** | **68,703** | **124,365** | **29,322** | **24,871** | **139,303** | **111,457** |
| Male | 464,493 | 50,231 | 414,262 | 777 | 142,544 | 3,263 | 7,174 | 9,334 | 14,714 | 19,183 | 27,194 | 61,682 | 212,301 | 33,052 | 61,970 | 16,785 | 14,967 | 85,527 | 58,640 |
| Female | 541,751 | 97,048 | 444,703 | 1,759 | 215,864 | 6,934 | 14,659 | 19,555 | 26,883 | 32,101 | 39,293 | 76,439 | 174,263 | 35,651 | 62,395 | 12,537 | 9,904 | 53,776 | 52,817 |
| **30 - 34** | | | | | | | | | | | | | | | | | | | |
| **Total** | **800,770** | **114,851** | **685,919** | **1,896** | **297,577** | **7,698** | **16,564** | **22,011** | **32,199** | **39,741** | **54,717** | **124,647** | **282,395** | **52,614** | **111,740** | **22,840** | **15,914** | **79,287** | **104,051** |
| Male | 397,694 | 42,470 | 355,224 | 715 | 126,495 | 2,874 | 5,979 | 7,721 | 11,943 | 15,720 | 23,947 | 58,311 | 165,558 | 27,298 | 58,709 | 14,269 | 10,403 | 54,879 | 62,456 |
| Female | 403,076 | 72,381 | 330,695 | 1,181 | 171,082 | 4,824 | 10,585 | 14,290 | 20,256 | 24,021 | 30,770 | 66,336 | 116,837 | 25,316 | 53,031 | 8,571 | 5,511 | 24,408 | 41,595 |
| **35 - 39** | | | | | | | | | | | | | | | | | | | |
| **Total** | **647,813** | **97,423** | **550,390** | **1,512** | **251,704** | **6,199** | **13,691** | **18,617** | **27,941** | **32,494** | **46,026** | **106,736** | **215,640** | **38,455** | **90,930** | **18,673** | **11,624** | **55,958** | **81,534** |
| Male | 332,961 | 35,366 | 297,595 | 558 | 109,875 | 2,310 | 5,140 | 6,706 | 10,711 | 13,187 | 20,683 | 51,138 | 133,884 | 21,276 | 51,455 | 11,905 | 7,989 | 41,259 | 53,278 |
| Female | 314,852 | 62,057 | 252,795 | 954 | 141,829 | 3,889 | 8,551 | 11,911 | 17,230 | 19,307 | 25,343 | 55,598 | 81,756 | 17,179 | 39,475 | 6,768 | 3,635 | 14,699 | 28,256 |
| **40 - 44** | | | | | | | | | | | | | | | | | | | |
| **Total** | **447,584** | **72,378** | **375,206** | **1,063** | **181,745** | **4,349** | **9,397** | **12,550** | **20,109** | **21,017** | **32,462** | **81,861** | **134,369** | **22,262** | **55,335** | **16,063** | **7,165** | **33,544** | **58,029** |
| Male | 232,253 | 25,243 | 207,010 | 362 | 80,694 | 1,554 | 3,403 | 4,508 | 7,499 | 8,483 | 14,876 | 40,371 | 86,080 | 12,921 | 33,539 | 9,612 | 4,772 | 25,236 | 39,874 |
| Female | 215,331 | 47,135 | 168,196 | 701 | 101,051 | 2,795 | 5,994 | 8,042 | 12,610 | 12,534 | 17,586 | 41,490 | 48,289 | 9,341 | 21,796 | 6,451 | 2,393 | 8,308 | 18,155 |
| **45 - 49** | | | | | | | | | | | | | | | | | | | |
| **Total** | **354,989** | **62,442** | **292,547** | **965** | **152,160** | **3,681** | **8,042** | **11,344** | **17,614** | **16,669** | **26,320** | **68,490** | **95,073** | **14,101** | **32,354** | **19,410** | **5,724** | **23,484** | **44,349** |
| Male | 174,833 | 19,855 | 154,978 | 324 | 64,956 | 1,144 | 2,651 | 3,912 | 6,178 | 6,341 | 11,708 | 33,022 | 59,429 | 7,813 | 19,216 | 11,487 | 3,696 | 17,217 | 30,269 |
| Female | 180,156 | 42,587 | 137,569 | 641 | 87,204 | 2,537 | 5,391 | 7,432 | 11,436 | 10,328 | 14,612 | 35,468 | 35,644 | 6,288 | 13,138 | 7,923 | 2,028 | 6,267 | 14,080 |
| **50 - 54** | | | | | | | | | | | | | | | | | | | |
| **Total** | **270,254** | **53,832** | **216,422** | **797** | **116,036** | **3,771** | **7,561** | **9,614** | **14,446** | **12,519** | **18,696** | **49,429** | **63,605** | **9,040** | **20,248** | **14,969** | **3,894** | **15,454** | **35,984** |
| Male | 128,696 | 14,895 | 113,801 | 238 | 49,024 | 1,072 | 2,201 | 3,320 | 4,837 | 4,663 | 8,279 | 24,652 | 40,281 | 4,710 | 11,476 | 9,857 | 2,709 | 11,529 | 24,258 |
| Female | 141,558 | 38,937 | 102,621 | 559 | 67,012 | 2,699 | 5,360 | 6,294 | 9,609 | 7,856 | 10,417 | 24,777 | 23,324 | 4,330 | 8,772 | 5,112 | 1,185 | 3,925 | 11,726 |

## Table D4: Population (De Facto) 5 Years and Older by Age (Single and Grouped), Sex, Highest Level of Education Completed and Rural/Urban, Zambia 2010

| Age and Sex | Population 5 Years and Older | Total Never Attended | Total Ever Attended | Highest Level of Education Completed (Among those Ever Attended School) | | | | | | | | | | | | | | | |
|---|---|---|---|---|---|---|---|---|---|---|---|---|---|---|---|---|---|---|---|
| | | | | None | Primary School Grade 1 - 7 | | | | | | | | Secondary School Grade 8 - 12 | | | | | | Tertiary |
| | | | | | Total | 1 | 2 | 3 | 4 | 5 | 6 | 7 | Total | 8 | 9 | 10 | 11 | 12 | |
| **55 - 59** | | | | | | | | | | | | | | | | | | | |
| **Total** | **184,828** | **41,022** | **143,806** | **610** | **77,596** | **3,256** | **6,889** | **7,270** | **10,702** | **8,604** | **12,300** | **28,575** | **39,773** | **6,041** | **13,534** | **7,629** | **2,436** | **10,133** | **25,827** |
| Male | 90,037 | 11,024 | 79,013 | 171 | 34,329 | 897 | 2,152 | 2,587 | 3,857 | 3,567 | 5,836 | 15,433 | 26,695 | 3,378 | 8,253 | 5,561 | 1,821 | 7,682 | 17,818 |
| Female | 94,791 | 29,998 | 64,793 | 439 | 43,267 | 2,359 | 4,737 | 4,683 | 6,845 | 5,037 | 6,464 | 13,142 | 13,078 | 2,663 | 5,281 | 2,068 | 615 | 2,451 | 8,009 |
| **60 - 64** | | | | | | | | | | | | | | | | | | | |
| **Total** | **161,875** | **51,643** | **110,232** | **752** | **67,455** | **4,656** | **9,425** | **8,330** | **11,452** | **7,716** | **9,899** | **15,977** | **25,108** | **4,184** | **8,941** | **3,659** | **1,682** | **6,642** | **16,917** |
| Male | 73,419 | 11,746 | 61,673 | 223 | 30,540 | 1,267 | 3,077 | 3,020 | 4,562 | 3,615 | 5,311 | 9,688 | 18,268 | 2,682 | 6,265 | 2,714 | 1,326 | 5,281 | 12,642 |
| Female | 88,456 | 39,897 | 48,559 | 529 | 36,915 | 3,389 | 6,348 | 5,310 | 6,890 | 4,101 | 4,588 | 6,289 | 6,840 | 1,502 | 2,676 | 945 | 356 | 1,361 | 4,275 |
| **65 +** | | | | | | | | | | | | | | | | | | | |
| **Total** | **336,332** | **148,497** | **187,835** | **2,182** | **133,836** | **13,627** | **23,455** | **19,135** | **23,535** | **15,069** | **17,538** | **21,477** | **32,395** | **6,233** | **11,400** | **3,981** | **2,284** | **8,497** | **19,422** |
| Male | 157,631 | 41,011 | 116,620 | 737 | 75,871 | 5,580 | 11,253 | 9,976 | 13,732 | 9,197 | 11,920 | 14,213 | 24,171 | 4,308 | 8,407 | 3,025 | 1,781 | 6,650 | 15,841 |
| Female | 178,701 | 107,486 | 71,215 | 1,445 | 57,965 | 8,047 | 12,202 | 9,159 | 9,803 | 5,872 | 5,618 | 7,264 | 8,224 | 1,925 | 2,993 | 956 | 503 | 1,847 | 3,581 |

## Table D4: Population (De Facto) 5 Years and Older by Age (Single and Grouped), Sex, Highest Level of Education Completed and Rural, Zambia 2010

| Age and Sex | Population 5 Years and Older | Total Never Attended | Total Ever Attended | Highest Level of Education Completed (Among those Ever Attended School) | | | | | | | | | | | | | | | |
|---|---|---|---|---|---|---|---|---|---|---|---|---|---|---|---|---|---|---|---|
| | | | | None | Primary School Grade 1 - 7 | | | | | | | | Secondary School Grade 8 - 12 | | | | | | Tertiary |
| | | | | | Total | 1 | 2 | 3 | 4 | 5 | 6 | 7 | Total | 8 | 9 | 10 | 11 | 12 | |
| **Zambia Rural** | | | | | | | | | | | | | | | | | | | |
| **Total** | **6,050,907** | **1,779,136** | **4,271,771** | **142,934** | **3,081,774** | **334,070** | **382,985** | **387,955** | **425,072** | **409,126** | **456,244** | **686,322** | **938,172** | **315,575** | **353,667** | **88,501** | **54,460** | **125,969** | **108,891** |
| Male | 2,940,191 | 762,704 | 2,177,487 | 69,292 | 1,478,440 | 159,364 | 177,652 | 177,923 | 194,850 | 190,792 | 223,226 | 354,633 | 554,699 | 171,806 | 203,737 | 57,287 | 35,695 | 86,174 | 75,056 |
| Female | 3,110,716 | 1,016,432 | 2,094,284 | 73,642 | 1,603,334 | 174,706 | 205,333 | 210,032 | 230,222 | 218,334 | 233,018 | 331,689 | 383,473 | 143,769 | 149,930 | 31,214 | 18,765 | 39,795 | 33,835 |
| **5** | | | | | | | | | | | | | | | | | | | |
| **Total** | **268,012** | **242,172** | **25,840** | **15,279** | **10,561** | **10,561** | **-** | **-** | **-** | **-** | **-** | **-** | **-** | **-** | **-** | **-** | **-** | **-** | **-** |
| Male | 135,175 | 122,692 | 12,483 | 7,323 | 5,160 | 5,160 | - | - | - | - | - | - | - | - | - | - | - | - | - |
| Female | 132,837 | 119,480 | 13,357 | 7,956 | 5,401 | 5,401 | - | - | - | - | - | - | - | - | - | - | - | - | - |
| **6** | | | | | | | | | | | | | | | | | | | |
| **Total** | **262,858** | **210,258** | **52,600** | **22,705** | **29,895** | **25,080** | **4,815** | **-** | **-** | **-** | **-** | **-** | **-** | **-** | **-** | **-** | **-** | **-** | **-** |
| Male | 131,568 | 106,824 | 24,744 | 10,678 | 14,066 | 11,846 | 2,220 | - | - | - | - | - | - | - | - | - | - | - | - |
| Female | 131,290 | 103,434 | 27,856 | 12,027 | 15,829 | 13,234 | 2,595 | - | - | - | - | - | - | - | - | - | - | - | - |
| **7** | | | | | | | | | | | | | | | | | | | |
| **Total** | **253,186** | **144,019** | **109,167** | **33,727** | **75,440** | **55,607** | **16,071** | **3,762** | **-** | **-** | **-** | **-** | **-** | **-** | **-** | **-** | **-** | **-** | **-** |
| Male | 127,589 | 74,860 | 52,729 | 16,531 | 36,198 | 26,834 | 7,553 | 1,811 | - | - | - | - | - | - | - | - | - | - | - |
| Female | 125,597 | 69,159 | 56,438 | 17,196 | 39,242 | 28,773 | 8,518 | 1,951 | - | - | - | - | - | - | - | - | - | - | - |
| **8** | | | | | | | | | | | | | | | | | | | |
| **Total** | **229,514** | **90,120** | **139,394** | **24,878** | **114,516** | **61,379** | **38,812** | **11,250** | **3,075** | **-** | **-** | **-** | **-** | **-** | **-** | **-** | **-** | **-** | **-** |
| Male | 114,279 | 46,661 | 67,618 | 12,357 | 55,261 | 30,252 | 18,359 | 5,175 | 1,475 | - | - | - | - | - | - | - | - | - | - |
| Female | 115,235 | 43,459 | 71,776 | 12,521 | 59,255 | 31,127 | 20,453 | 6,075 | 1,600 | - | - | - | - | - | - | - | - | - | - |
| **9** | | | | | | | | | | | | | | | | | | | |
| **Total** | **198,677** | **52,433** | **146,244** | **13,317** | **132,927** | **44,067** | **50,697** | **27,399** | **8,586** | **2,178** | **-** | **-** | **-** | **-** | **-** | **-** | **-** | **-** | **-** |
| Male | 99,630 | 27,531 | 72,099 | 6,864 | 65,235 | 22,376 | 24,805 | 13,003 | 4,033 | 1,018 | - | - | - | - | - | - | - | - | - |
| Female | 99,047 | 24,902 | 74,145 | 6,453 | 67,692 | 21,691 | 25,892 | 14,396 | 4,553 | 1,160 | - | - | - | - | - | - | - | - | - |
| **5 - 9** | | | | | | | | | | | | | | | | | | | |
| **Total** | **1,212,247** | **739,002** | **473,245** | **109,906** | **363,339** | **196,694** | **110,395** | **42,411** | **11,661** | **2,178** | **-** | **-** | **-** | **-** | **-** | **-** | **-** | **-** | **-** |
| Male | 608,241 | 378,568 | 229,673 | 53,753 | 175,920 | 96,468 | 52,937 | 19,989 | 5,508 | 1,018 | - | - | - | - | - | - | - | - | - |
| Female | 604,006 | 360,434 | 243,572 | 56,153 | 187,419 | 100,226 | 57,458 | 22,422 | 6,153 | 1,160 | - | - | - | - | - | - | - | - | - |
| **10** | | | | | | | | | | | | | | | | | | | |
| **Total** | **291,852** | **63,290** | **228,562** | **11,635** | **216,927** | **43,270** | **70,047** | **61,769** | **29,720** | **9,262** | **2,859** | **-** | **-** | **-** | **-** | **-** | **-** | **-** | **-** |
| Male | 147,733 | 33,734 | 113,999 | 6,082 | 107,917 | 22,468 | 35,279 | 30,117 | 14,272 | 4,341 | 1,440 | - | - | - | - | - | - | - | - |
| Female | 144,119 | 29,556 | 114,563 | 5,553 | 109,010 | 20,802 | 34,768 | 31,652 | 15,448 | 4,921 | 1,419 | - | - | - | - | - | - | - | - |
| **11** | | | | | | | | | | | | | | | | | | | |
| **Total** | **179,931** | **28,762** | **151,169** | **3,355** | **147,814** | **14,312** | **30,751** | **42,374** | **35,682** | **17,205** | **5,615** | **1,875** | **-** | **-** | **-** | **-** | **-** | **-** | **-** |
| Male | 90,703 | 15,339 | 75,364 | 1,822 | 73,542 | 7,630 | 15,965 | 21,167 | 17,121 | 8,093 | 2,647 | 919 | - | - | - | - | - | - | - |
| Female | 89,228 | 13,423 | 75,805 | 1,533 | 74,272 | 6,682 | 14,786 | 21,207 | 18,561 | 9,112 | 2,968 | 956 | - | - | - | - | - | - | - |
| **12** | | | | | | | | | | | | | | | | | | | |
| **Total** | **216,717** | **28,936** | **187,781** | **2,462** | **183,868** | **10,259** | **24,489** | **41,046** | **47,991** | **36,572** | **17,275** | **6,236** | **1,451** | **1,451** | **-** | **-** | **-** | **-** | **-** |
| Male | 110,395 | 15,539 | 94,856 | 1,345 | 92,763 | 5,600 | 13,159 | 21,135 | 24,089 | 17,669 | 8,149 | 2,962 | 748 | 748 | - | - | - | - | - |
| Female | 106,322 | 13,397 | 92,925 | 1,117 | 91,105 | 4,659 | 11,330 | 19,911 | 23,902 | 18,903 | 9,126 | 3,274 | 703 | 703 | - | - | - | - | - |
| **13** | | | | | | | | | | | | | | | | | | | |
| **Total** | **180,849** | **20,608** | **160,241** | **1,285** | **154,790** | **4,948** | **12,751** | **24,286** | **35,661** | **38,165** | **26,583** | **12,396** | **4,166** | **3,254** | **912** | **-** | **-** | **-** | **-** |
| Male | 91,449 | 10,857 | 80,592 | 662 | 78,064 | 2,790 | 7,089 | 12,944 | 18,444 | 18,825 | 12,331 | 5,641 | 1,866 | 1,434 | 432 | - | - | - | - |
| Female | 89,400 | 9,751 | 79,649 | 623 | 76,726 | 2,158 | 5,662 | 11,342 | 17,217 | 19,340 | 14,252 | 6,755 | 2,300 | 1,820 | 480 | - | - | - | - |
| **14** | | | | | | | | | | | | | | | | | | | |
| **Total** | **176,628** | **19,401** | **157,227** | **851** | **143,393** | **2,987** | **7,392** | **14,916** | **25,378** | **34,490** | **33,987** | **24,243** | **12,983** | **9,646** | **2,976** | **361** | **-** | **-** | **-** |
| Male | 89,698 | 10,001 | 79,697 | 470 | 73,322 | 1,723 | 4,127 | 8,377 | 13,547 | 17,681 | 16,566 | 11,301 | 5,905 | 4,364 | 1,375 | 166 | - | - | - |
| Female | 86,930 | 9,400 | 77,530 | 381 | 70,071 | 1,264 | 3,265 | 6,539 | 11,831 | 16,809 | 17,421 | 12,942 | 7,078 | 5,282 | 1,601 | 195 | - | - | - |
| **10 - 14** | | | | | | | | | | | | | | | | | | | |
| **Total** | **1,045,977** | **160,997** | **884,980** | **19,588** | **846,792** | **75,776** | **145,430** | **184,391** | **174,432** | **135,694** | **86,319** | **44,750** | **18,600** | **14,351** | **3,888** | **361** | **-** | **-** | **-** |
| Male | 529,978 | 85,470 | 444,508 | 10,381 | 425,608 | 40,211 | 75,619 | 93,740 | 87,473 | 66,609 | 41,133 | 20,823 | 8,519 | 6,546 | 1,807 | 166 | - | - | - |
| Female | 515,999 | 75,527 | 440,472 | 9,207 | 421,184 | 35,565 | 69,811 | 90,651 | 86,959 | 69,085 | 45,186 | 23,927 | 10,081 | 7,805 | 2,081 | 195 | - | - | - |

## Table D4: Population (De Facto) 5 Years and Older by Age (Single and Grouped), Sex, Highest Level of Education Completed and Rural, Zambia 2010

| Age and Sex | Population 5 Years and Older | Total Never Attended | Total Ever Attended | Highest Level of Education Completed (Among those Ever Attended School) | | | | | | | | | | | | | | | |
|---|---|---|---|---|---|---|---|---|---|---|---|---|---|---|---|---|---|---|---|
| | | | | None | Primary School Grade 1 - 7 | | | | | | | | Secondary School Grade 8 - 12 | | | | | | Tertiary |
| | | | | | Total | 1 | 2 | 3 | 4 | 5 | 6 | 7 | Total | 8 | 9 | 10 | 11 | 12 | |
| **15** | | | | | | | | | | | | | | | | | | | |
| **Total** | **184,001** | **21,264** | **162,737** | **700** | **133,881** | **2,258** | **5,227** | **10,039** | **18,214** | **28,812** | **34,880** | **34,451** | **28,156** | **19,138** | **7,498** | **1,197** | **323** | **-** | **-** |
| Male | 94,505 | 10,666 | 83,839 | 409 | 70,601 | 1,265 | 2,981 | 5,708 | 10,217 | 15,573 | 17,867 | 16,990 | 12,829 | 8,702 | 3,371 | 590 | 166 | - | - |
| Female | 89,496 | 10,598 | 78,898 | 291 | 63,280 | 993 | 2,246 | 4,331 | 7,997 | 13,239 | 17,013 | 17,461 | 15,327 | 10,436 | 4,127 | 607 | 157 | - | - |
| **16** | | | | | | | | | | | | | | | | | | | |
| **Total** | **169,390** | **18,971** | **150,419** | **526** | **106,415** | **1,588** | **3,356** | **6,393** | **11,648** | **19,199** | **28,784** | **35,447** | **43,474** | **25,767** | **13,551** | **2,901** | **951** | **304** | **4** |
| Male | 85,163 | 8,727 | 76,436 | 268 | 55,944 | 816 | 1,798 | 3,430 | 6,472 | 10,499 | 15,250 | 17,679 | 20,222 | 12,114 | 6,137 | 1,387 | 435 | 149 | 2 |
| Female | 84,227 | 10,244 | 73,983 | 258 | 50,471 | 772 | 1,558 | 2,963 | 5,176 | 8,700 | 13,534 | 17,768 | 23,252 | 13,653 | 7,414 | 1,514 | 516 | 155 | 2 |
| **17** | | | | | | | | | | | | | | | | | | | |
| **Total** | **142,117** | **16,997** | **125,120** | **418** | **75,828** | **1,170** | **2,741** | **4,603** | **7,310** | **12,167** | **19,106** | **28,731** | **48,492** | **24,530** | **16,479** | **4,367** | **2,227** | **889** | **382** |
| Male | 70,491 | 7,138 | 63,353 | 231 | 39,139 | 505 | 1,212 | 2,210 | 3,769 | 6,419 | 10,205 | 14,819 | 23,813 | 12,268 | 7,826 | 2,270 | 1,055 | 394 | 170 |
| Female | 71,626 | 9,859 | 61,767 | 187 | 36,689 | 665 | 1,529 | 2,393 | 3,541 | 5,748 | 8,901 | 13,912 | 24,679 | 12,262 | 8,653 | 2,097 | 1,172 | 495 | 212 |
| **18** | | | | | | | | | | | | | | | | | | | |
| **Total** | **172,292** | **23,791** | **148,501** | **473** | **80,852** | **1,492** | **3,333** | **5,023** | **8,138** | **12,052** | **19,108** | **31,706** | **66,580** | **29,195** | **23,318** | **6,547** | **4,393** | **3,127** | **596** |
| Male | 83,910 | 9,288 | 74,622 | 217 | 39,558 | 619 | 1,368 | 2,137 | 3,696 | 5,796 | 9,760 | 16,182 | 34,574 | 15,569 | 11,549 | 3,655 | 2,348 | 1,453 | 273 |
| Female | 88,382 | 14,503 | 73,879 | 256 | 41,294 | 873 | 1,965 | 2,886 | 4,442 | 6,256 | 9,348 | 15,524 | 32,006 | 13,626 | 11,769 | 2,892 | 2,045 | 1,674 | 323 |
| **19** | | | | | | | | | | | | | | | | | | | |
| **Total** | **135,197** | **20,994** | **114,203** | **406** | **58,670** | **1,269** | **2,683** | **4,050** | **6,152** | **8,678** | **13,166** | **22,672** | **54,429** | **20,439** | **19,318** | **5,523** | **4,374** | **4,775** | **698** |
| Male | 63,669 | 7,509 | 56,160 | 182 | 26,519 | 448 | 979 | 1,511 | 2,457 | 3,721 | 6,288 | 11,115 | 29,121 | 11,102 | 9,830 | 3,211 | 2,540 | 2,438 | 338 |
| Female | 71,528 | 13,485 | 58,043 | 224 | 32,151 | 821 | 1,704 | 2,539 | 3,695 | 4,957 | 6,878 | 11,557 | 25,308 | 9,337 | 9,488 | 2,312 | 1,834 | 2,337 | 360 |
| **15 - 19** | | | | | | | | | | | | | | | | | | | |
| **Total** | **802,997** | **102,017** | **700,980** | **2,523** | **455,646** | **7,777** | **17,340** | **30,108** | **51,462** | **80,908** | **115,044** | **153,007** | **241,131** | **119,069** | **80,164** | **20,535** | **12,268** | **9,095** | **1,680** |
| Male | 397,738 | 43,328 | 354,410 | 1,307 | 231,761 | 3,653 | 8,338 | 14,996 | 26,611 | 42,008 | 59,370 | 76,785 | 120,559 | 59,755 | 38,713 | 11,113 | 6,544 | 4,434 | 783 |
| Female | 405,259 | 58,689 | 346,570 | 1,216 | 223,885 | 4,124 | 9,002 | 15,112 | 24,851 | 38,900 | 55,674 | 76,222 | 120,572 | 59,314 | 41,451 | 9,422 | 5,724 | 4,661 | 897 |
| **20 - 24** | | | | | | | | | | | | | | | | | | | |
| **Total** | **598,270** | **118,003** | **480,267** | **1,853** | **256,113** | **7,347** | **15,699** | **21,710** | **30,893** | **37,846** | **51,749** | **90,869** | **213,977** | **62,313** | **76,240** | **18,447** | **17,534** | **39,443** | **8,324** |
| Male | 269,368 | 39,164 | 230,204 | 693 | 104,300 | 2,390 | 4,821 | 6,972 | 10,919 | 14,271 | 22,078 | 42,849 | 120,857 | 33,609 | 41,389 | 11,695 | 11,102 | 23,062 | 4,354 |
| Female | 328,902 | 78,839 | 250,063 | 1,160 | 151,813 | 4,957 | 10,878 | 14,738 | 19,974 | 23,575 | 29,671 | 48,020 | 93,120 | 28,704 | 34,851 | 6,752 | 6,432 | 16,381 | 3,970 |
| **25 - 29** | | | | | | | | | | | | | | | | | | | |
| **Total** | **529,481** | **125,064** | **404,417** | **1,860** | **250,994** | **8,626** | **18,278** | **23,652** | **32,760** | **37,965** | **46,425** | **83,288** | **132,502** | **36,921** | **50,156** | **9,373** | **7,598** | **28,454** | **19,061** |
| Male | 241,136 | 42,940 | 198,196 | 591 | 106,160 | 2,791 | 6,079 | 7,853 | 12,037 | 15,262 | 20,828 | 41,310 | 80,490 | 20,508 | 29,011 | 6,151 | 5,212 | 19,608 | 10,955 |
| Female | 288,345 | 82,124 | 206,221 | 1,269 | 144,834 | 5,835 | 12,199 | 15,799 | 20,723 | 22,703 | 25,597 | 41,978 | 52,012 | 16,413 | 21,145 | 3,222 | 2,386 | 8,846 | 8,106 |
| **30 - 34** | | | | | | | | | | | | | | | | | | | |
| **Total** | **419,932** | **97,101** | **322,831** | **1,399** | **206,620** | **6,456** | **13,900** | **18,082** | **25,353** | **29,570** | **38,352** | **74,907** | **95,717** | **27,179** | **42,682** | **6,694** | **4,492** | **14,670** | **19,095** |
| Male | 203,507 | 36,460 | 167,047 | 544 | 93,571 | 2,405 | 5,095 | 6,550 | 9,864 | 12,545 | 18,259 | 38,853 | 60,195 | 15,984 | 25,353 | 4,635 | 3,248 | 10,975 | 12,737 |
| Female | 216,425 | 60,641 | 155,784 | 855 | 113,049 | 4,051 | 8,805 | 11,532 | 15,489 | 17,025 | 20,093 | 36,054 | 35,522 | 11,195 | 17,329 | 2,059 | 1,244 | 3,695 | 6,358 |
| **35 - 39** | | | | | | | | | | | | | | | | | | | |
| **Total** | **351,970** | **82,414** | **269,556** | **1,168** | **178,167** | **5,202** | **11,539** | **15,361** | **22,364** | **24,779** | **33,227** | **65,695** | **76,031** | **20,348** | **36,130** | **5,551** | **3,385** | **10,617** | **14,190** |
| Male | 173,650 | 30,272 | 143,378 | 427 | 82,121 | 1,946 | 4,415 | 5,704 | 8,925 | 10,626 | 16,035 | 34,470 | 50,623 | 12,586 | 23,102 | 3,859 | 2,555 | 8,521 | 10,207 |
| Female | 178,320 | 52,142 | 126,178 | 741 | 96,046 | 3,256 | 7,124 | 9,657 | 13,439 | 14,153 | 17,192 | 31,225 | 25,408 | 7,762 | 13,028 | 1,692 | 830 | 2,096 | 3,983 |
| **40 - 44** | | | | | | | | | | | | | | | | | | | |
| **Total** | **254,964** | **61,561** | **193,403** | **814** | **131,499** | **3,679** | **7,810** | **10,218** | **16,257** | **16,089** | **24,079** | **53,367** | **50,742** | **12,319** | **23,629** | **5,718** | **2,333** | **6,743** | **10,348** |
| Male | 127,044 | 21,800 | 105,244 | 279 | 62,309 | 1,336 | 2,897 | 3,802 | 6,386 | 6,937 | 11,966 | 28,985 | 34,885 | 7,901 | 16,031 | 3,753 | 1,719 | 5,481 | 7,771 |
| Female | 127,920 | 39,761 | 88,159 | 535 | 69,190 | 2,343 | 4,913 | 6,416 | 9,871 | 9,152 | 12,113 | 24,382 | 15,857 | 4,418 | 7,598 | 1,965 | 614 | 1,262 | 2,577 |
| **45 - 49** | | | | | | | | | | | | | | | | | | | |
| **Total** | **209,868** | **52,823** | **157,045** | **739** | **109,721** | **3,039** | **6,518** | **8,981** | **14,097** | **12,733** | **19,603** | **44,750** | **37,755** | **7,981** | **14,439** | **7,945** | **2,147** | **5,243** | **8,830** |
| Male | 100,903 | 17,293 | 83,610 | 258 | 50,982 | 998 | 2,248 | 3,270 | 5,292 | 5,282 | 9,567 | 24,325 | 25,853 | 5,034 | 9,773 | 5,348 | 1,539 | 4,159 | 6,517 |
| Female | 108,965 | 35,530 | 73,435 | 481 | 58,739 | 2,041 | 4,270 | 5,711 | 8,805 | 7,451 | 10,036 | 20,425 | 11,902 | 2,947 | 4,666 | 2,597 | 608 | 1,084 | 2,313 |
| **50 - 54** | | | | | | | | | | | | | | | | | | | |
| **Total** | **162,395** | **44,677** | **117,718** | **567** | **82,375** | **2,992** | **5,832** | **7,260** | **11,215** | **9,240** | **13,553** | **32,283** | **26,375** | **5,095** | **9,233** | **6,667** | **1,634** | **3,746** | **8,401** |
| Male | 75,868 | 12,888 | 62,980 | 176 | 38,133 | 905 | 1,808 | 2,679 | 4,042 | 3,801 | 6,678 | 18,220 | 18,370 | 3,085 | 6,030 | 4,899 | 1,239 | 3,117 | 6,301 |
| Female | 86,527 | 31,789 | 54,738 | 391 | 44,242 | 2,087 | 4,024 | 4,581 | 7,173 | 5,439 | 6,875 | 14,063 | 8,005 | 2,010 | 3,203 | 1,768 | 395 | 629 | 2,100 |

## Table D4: Population (De Facto) 5 Years and Older by Age (Single and Grouped), Sex, Highest Level of Education Completed and Rural, Zambia 2010

| Age and Sex | Total | Total Never Attended | Total Ever Attended | Highest Level of Education Completed (Among those Ever Attended School) | | | | | | | | | | | | | | | |
|---|---|---|---|---|---|---|---|---|---|---|---|---|---|---|---|---|---|---|---|
| | | | | None | Primary School Grade 1 - 7 | | | | | | | | Secondary School Grade 8 - 12 | | | | | | Tertiary |
| | | | | | Total | 1 | 2 | 3 | 4 | 5 | 6 | 7 | Total | 8 | 9 | 10 | 11 | 12 | |
| **55 - 59** | | | | | | | | | | | | | | | | | | | |
| **Total** | **111,511** | **33,543** | **77,968** | **437** | **54,723** | **2,459** | **5,122** | **5,422** | **8,209** | **6,164** | **8,735** | **18,612** | **16,894** | **3,436** | **6,275** | **3,426** | **1,079** | **2,678** | **5,914** |
| Male | 52,225 | 9,560 | 42,665 | 136 | 25,978 | 711 | 1,708 | 2,049 | 3,163 | 2,783 | 4,540 | 11,024 | 12,125 | 2,126 | 4,218 | 2,661 | 868 | 2,252 | 4,426 |
| Female | 59,286 | 23,983 | 35,303 | 301 | 28,745 | 1,748 | 3,414 | 3,373 | 5,046 | 3,381 | 4,195 | 7,588 | 4,769 | 1,310 | 2,057 | 765 | 211 | 426 | 1,488 |
| **60 - 64** | | | | | | | | | | | | | | | | | | | |
| **Total** | **107,038** | **42,286** | **64,752** | **543** | **47,965** | **3,615** | **7,036** | **6,083** | **8,573** | **5,299** | **6,810** | **10,549** | **11,509** | **2,532** | **4,482** | **1,685** | **767** | **2,043** | **4,735** |
| Male | 45,761 | 10,065 | 35,696 | 169 | 22,971 | 1,039 | 2,439 | 2,333 | 3,628 | 2,703 | 3,966 | 6,863 | 8,770 | 1,736 | 3,312 | 1,316 | 648 | 1,758 | 3,786 |
| Female | 61,277 | 32,221 | 29,056 | 374 | 24,994 | 2,576 | 4,597 | 3,750 | 4,945 | 2,596 | 2,844 | 3,686 | 2,739 | 796 | 1,170 | 369 | 119 | 285 | 949 |
| **65 +** | | | | | | | | | | | | | | | | | | | |
| **Total** | **244,257** | **119,648** | **124,609** | **1,537** | **97,820** | **10,408** | **18,086** | **14,276** | **17,796** | **10,661** | **12,348** | **14,245** | **16,939** | **4,031** | **6,349** | **2,099** | **1,223** | **3,237** | **8,313** |
| Male | 114,772 | 34,896 | 79,876 | 578 | 58,626 | 4,511 | 9,248 | 7,986 | 11,002 | 6,947 | 8,806 | 10,126 | 13,453 | 2,936 | 4,998 | 1,691 | 1,021 | 2,807 | 7,219 |
| Female | 129,485 | 84,752 | 44,733 | 959 | 39,194 | 5,897 | 8,838 | 6,290 | 6,794 | 3,714 | 3,542 | 4,119 | 3,486 | 1,095 | 1,351 | 408 | 202 | 430 | 1,094 |

# Table D4: Population (De Facto) 5 Years and Older by Age (Single and Grouped), Sex, Highest Level of Education Completed and Urban, Zambia 2010

| Age and Sex | Population 5 Years and Older | Total Never Attended | Total Ever Attended | Highest Level of Education Completed (Among those Ever Attended School) | | | | | | | | | | | | | | | |
|---|---|---|---|---|---|---|---|---|---|---|---|---|---|---|---|---|---|---|---|
| | | | | None | Primary School Grade 1 - 7 | | | | | | | | Secondary School Grade 8 - 12 | | | | | | Tertiary |
| | | | | | Total | 1 | 2 | 3 | 4 | 5 | 6 | 7 | Total | 8 | 9 | 10 | 11 | 12 | |
| **Zambia Urban** | | | | | | | | | | | | | | | | | | | |
| **Total** | **4,260,520** | **407,422** | **3,853,098** | **134,031** | **1,627,357** | **164,823** | **169,751** | **181,113** | **196,796** | **210,608** | **247,602** | **456,664** | **1,638,932** | **287,102** | **496,104** | **196,568** | **151,423** | **507,735** | **452,778** |
| Male | 2,074,790 | 169,861 | 1,904,929 | 65,890 | 713,478 | 79,128 | 77,948 | 81,585 | 86,478 | 90,561 | 105,401 | 192,377 | 856,685 | 128,696 | 237,372 | 105,365 | 82,757 | 302,495 | 268,876 |
| Female | 2,185,730 | 237,561 | 1,948,169 | 68,141 | 913,879 | 85,695 | 91,803 | 99,528 | 110,318 | 120,047 | 142,201 | 264,287 | 782,247 | 158,406 | 258,732 | 91,203 | 68,666 | 205,240 | 183,902 |
| **5** | | | | | | | | | | | | | | | | | | | |
| **Total** | **138,821** | **86,806** | **52,015** | **41,548** | **10,467** | **10,467** | **-** | **-** | **-** | **-** | **-** | **-** | **-** | **-** | **-** | **-** | **-** | **-** | **-** |
| Male | 69,075 | 43,685 | 25,390 | 20,398 | 4,992 | 4,992 | - | - | - | - | - | - | - | - | - | - | - | - | - |
| Female | 69,746 | 43,121 | 26,625 | 21,150 | 5,475 | 5,475 | - | - | - | - | - | - | - | - | - | - | - | - | - |
| **6** | | | | | | | | | | | | | | | | | | | |
| **Total** | **133,936** | **64,426** | **69,510** | **37,185** | **32,325** | **27,239** | **5,086** | **-** | **-** | **-** | **-** | **-** | **-** | **-** | **-** | **-** | **-** | **-** | **-** |
| Male | 65,913 | 32,618 | 33,295 | 18,421 | 14,874 | 12,694 | 2,180 | - | - | - | - | - | - | - | - | - | - | - | - |
| Female | 68,023 | 31,808 | 36,215 | 18,764 | 17,451 | 14,545 | 2,906 | - | - | - | - | - | - | - | - | - | - | - | - |
| **7** | | | | | | | | | | | | | | | | | | | |
| **Total** | **135,119** | **37,081** | **98,038** | **27,852** | **70,186** | **44,937** | **20,478** | **4,771** | **-** | **-** | **-** | **-** | **-** | **-** | **-** | **-** | **-** | **-** | **-** |
| Male | 67,112 | 19,024 | 48,088 | 14,065 | 34,023 | 22,412 | 9,408 | 2,203 | - | - | - | - | - | - | - | - | - | - | - |
| Female | 68,007 | 18,057 | 49,950 | 13,787 | 36,163 | 22,525 | 11,070 | 2,568 | - | - | - | - | - | - | - | - | - | - | - |
| **8** | | | | | | | | | | | | | | | | | | | |
| **Total** | **121,951** | **17,232** | **104,719** | **12,196** | **92,523** | **34,056** | **36,662** | **17,501** | **4,304** | **-** | **-** | **-** | **-** | **-** | **-** | **-** | **-** | **-** | **-** |
| Male | 58,944 | 8,662 | 50,282 | 6,126 | 44,156 | 17,087 | 17,408 | 7,815 | 1,846 | - | - | - | - | - | - | - | - | - | - |
| Female | 63,007 | 8,570 | 54,437 | 6,070 | 48,367 | 16,969 | 19,254 | 9,686 | 2,458 | - | - | - | - | - | - | - | - | - | - |
| **9** | | | | | | | | | | | | | | | | | | | |
| **Total** | **114,262** | **9,313** | **104,949** | **4,547** | **100,402** | **16,592** | **32,563** | **31,882** | **15,409** | **3,956** | **-** | **-** | **-** | **-** | **-** | **-** | **-** | **-** | **-** |
| Male | 55,110 | 4,680 | 50,430 | 2,355 | 48,075 | 8,488 | 16,100 | 14,766 | 6,909 | 1,812 | - | - | - | - | - | - | - | - | - |
| Female | 59,152 | 4,633 | 54,519 | 2,192 | 52,327 | 8,104 | 16,463 | 17,116 | 8,500 | 2,144 | - | - | - | - | - | - | - | - | - |
| **5 - 9** | | | | | | | | | | | | | | | | | | | |
| **Total** | **644,089** | **214,858** | **429,231** | **123,328** | **305,903** | **133,291** | **94,789** | **54,154** | **19,713** | **3,956** | **-** | **-** | **-** | **-** | **-** | **-** | **-** | **-** | **-** |
| Male | 316,154 | 108,669 | 207,485 | 61,365 | 146,120 | 65,673 | 45,096 | 24,784 | 8,755 | 1,812 | - | - | - | - | - | - | - | - | - |
| Female | 327,935 | 106,189 | 221,746 | 61,963 | 159,783 | 67,618 | 49,693 | 29,370 | 10,958 | 2,144 | - | - | - | - | - | - | - | - | - |
| **10** | | | | | | | | | | | | | | | | | | | |
| **Total** | **157,640** | **10,148** | **147,492** | **3,191** | **144,301** | **11,338** | **28,083** | **44,454** | **37,804** | **17,706** | **4,916** | **-** | **-** | **-** | **-** | **-** | **-** | **-** | **-** |
| Male | 76,735 | 5,119 | 71,616 | 1,624 | 69,992 | 5,732 | 14,170 | 21,911 | 17,888 | 8,059 | 2,232 | - | - | - | - | - | - | - | - |
| Female | 80,905 | 5,029 | 75,876 | 1,567 | 74,309 | 5,606 | 13,913 | 22,543 | 19,916 | 9,647 | 2,684 | - | - | - | - | - | - | - | - |
| **11** | | | | | | | | | | | | | | | | | | | |
| **Total** | **110,646** | **4,378** | **106,268** | **983** | **105,285** | **2,901** | **8,970** | **20,050** | **29,758** | **26,591** | **13,044** | **3,971** | **-** | **-** | **-** | **-** | **-** | **-** | **-** |
| Male | 52,794 | 2,116 | 50,678 | 475 | 50,203 | 1,491 | 4,624 | 9,958 | 14,448 | 12,321 | 5,689 | 1,672 | - | - | - | - | - | - | - |
| Female | 57,852 | 2,262 | 55,590 | 508 | 55,082 | 1,410 | 4,346 | 10,092 | 15,310 | 14,270 | 7,355 | 2,299 | - | - | - | - | - | - | - |
| **12** | | | | | | | | | | | | | | | | | | | |
| **Total** | **134,336** | **4,560** | **129,776** | **760** | **125,380** | **1,870** | **5,674** | **13,710** | **25,658** | **34,300** | **28,827** | **15,341** | **3,636** | **3,636** | **-** | **-** | **-** | **-** | **-** |
| Male | 64,076 | 2,197 | 61,879 | 342 | 60,012 | 1,000 | 2,872 | 6,837 | 12,769 | 16,548 | 13,202 | 6,784 | 1,525 | 1,525 | - | - | - | - | - |
| Female | 70,260 | 2,363 | 67,897 | 418 | 65,368 | 870 | 2,802 | 6,873 | 12,889 | 17,752 | 15,625 | 8,557 | 2,111 | 2,111 | - | - | - | - | - |
| **13** | | | | | | | | | | | | | | | | | | | |
| **Total** | **124,279** | **3,312** | **120,967** | **476** | **106,118** | **854** | **2,461** | **6,619** | **13,936** | **24,316** | **30,795** | **27,137** | **14,373** | **11,258** | **3,115** | **-** | **-** | **-** | **-** |
| Male | 58,269 | 1,444 | 56,825 | 235 | 50,580 | 417 | 1,222 | 3,345 | 6,943 | 11,874 | 14,529 | 12,250 | 6,010 | 4,706 | 1,304 | - | - | - | - |
| Female | 66,010 | 1,868 | 64,142 | 241 | 55,538 | 437 | 1,239 | 3,274 | 6,993 | 12,442 | 16,266 | 14,887 | 8,363 | 6,552 | 1,811 | - | - | - | - |
| **14** | | | | | | | | | | | | | | | | | | | |
| **Total** | **126,164** | **3,054** | **123,110** | **338** | **86,182** | **556** | **1,277** | **3,297** | **7,411** | **15,458** | **24,740** | **33,443** | **36,590** | **23,491** | **10,772** | **2,327** | **-** | **-** | **-** |
| Male | 58,982 | 1,360 | 57,622 | 159 | 41,828 | 264 | 660 | 1,747 | 3,881 | 7,710 | 12,030 | 15,536 | 15,635 | 10,155 | 4,501 | 979 | - | - | - |
| Female | 67,182 | 1,694 | 65,488 | 179 | 44,354 | 292 | 617 | 1,550 | 3,530 | 7,748 | 12,710 | 17,907 | 20,955 | 13,336 | 6,271 | 1,348 | - | - | - |
| **10 - 14** | | | | | | | | | | | | | | | | | | | |
| **Total** | **653,065** | **25,452** | **627,613** | **5,748** | **567,266** | **17,519** | **46,465** | **88,130** | **114,567** | **118,371** | **102,322** | **79,892** | **54,599** | **38,385** | **13,887** | **2,327** | **-** | **-** | **-** |
| Male | 310,856 | 12,236 | 298,620 | 2,835 | 272,615 | 8,904 | 23,548 | 43,798 | 55,929 | 56,512 | 47,682 | 36,242 | 23,170 | 16,386 | 5,805 | 979 | - | - | - |
| Female | 342,209 | 13,216 | 328,993 | 2,913 | 294,651 | 8,615 | 22,917 | 44,332 | 58,638 | 61,859 | 54,640 | 43,650 | 31,429 | 21,999 | 8,082 | 1,348 | - | - | - |

## Table D4: Population (De Facto) 5 Years and Older by Age (Single and Grouped), Sex, Highest Level of Education Completed and Urban, Zambia 2010

| Age and Sex | Population 5 Years and Older | Total Never Attended | Total Ever Attended | Highest Level of Education Completed (Among those Ever Attended School) | | | | | | | | | | | | | | | |
|---|---|---|---|---|---|---|---|---|---|---|---|---|---|---|---|---|---|---|---|
| | | | | None | Primary School Grade 1 - 7 | | | | | | | | Secondary School Grade 8 - 12 | | | | | | Tertiary |
| | | | | | Total | 1 | 2 | 3 | 4 | 5 | 6 | 7 | Total | 8 | 9 | 10 | 11 | 12 | |
| **15** | | | | | | | | | | | | | | | | | | | |
| **Total** | **128,337** | **3,285** | **125,052** | **314** | **63,515** | **423** | **847** | **1,944** | **4,347** | **9,649** | **17,188** | **29,117** | **61,223** | **29,574** | **21,783** | **7,912** | **1,954** | **-** | **-** |
| Male | 60,642 | 1,452 | 59,190 | 144 | 31,879 | 216 | 410 | 1,009 | 2,308 | 4,991 | 8,813 | 14,132 | 27,167 | 13,428 | 9,636 | 3,351 | 752 | - | - |
| Female | 67,695 | 1,833 | 65,862 | 170 | 31,636 | 207 | 437 | 935 | 2,039 | 4,658 | 8,375 | 14,985 | 34,056 | 16,146 | 12,147 | 4,561 | 1,202 | - | - |
| **16** | | | | | | | | | | | | | | | | | | | |
| **Total** | **126,162** | **2,849** | **123,313** | **238** | **42,478** | **329** | **555** | **1,161** | **2,501** | **5,151** | **10,930** | **21,851** | **80,545** | **27,311** | **28,136** | **16,290** | **6,948** | **1,860** | **52** |
| Male | 58,477 | 1,185 | 57,292 | 108 | 20,991 | 139 | 251 | 569 | 1,288 | 2,611 | 5,524 | 10,609 | 36,177 | 12,694 | 12,768 | 7,142 | 2,812 | 761 | 16 |
| Female | 67,685 | 1,664 | 66,021 | 130 | 21,487 | 190 | 304 | 592 | 1,213 | 2,540 | 5,406 | 11,242 | 44,368 | 14,617 | 15,368 | 9,148 | 4,136 | 1,099 | 36 |
| **17** | | | | | | | | | | | | | | | | | | | |
| **Total** | **118,029** | **2,735** | **115,294** | **210** | **27,931** | **252** | **474** | **836** | **1,549** | **3,143** | **6,532** | **15,145** | **86,581** | **20,382** | **26,138** | **18,731** | **14,683** | **6,647** | **572** |
| Male | 55,843 | 1,101 | 54,742 | 100 | 13,712 | 129 | 205 | 406 | 720 | 1,481 | 3,364 | 7,407 | 40,685 | 9,894 | 12,277 | 9,168 | 6,632 | 2,714 | 245 |
| Female | 62,186 | 1,634 | 60,552 | 110 | 14,219 | 123 | 269 | 430 | 829 | 1,662 | 3,168 | 7,738 | 45,896 | 10,488 | 13,861 | 9,563 | 8,051 | 3,933 | 327 |
| **18** | | | | | | | | | | | | | | | | | | | |
| **Total** | **136,798** | **3,782** | **133,016** | **169** | **26,668** | **330** | **531** | **974** | **1,722** | **3,078** | **5,813** | **14,220** | **104,792** | **18,642** | **27,766** | **18,955** | **20,644** | **18,785** | **1,387** |
| Male | 65,340 | 1,491 | 63,849 | 77 | 12,294 | 137 | 226 | 427 | 724 | 1,335 | 2,677 | 6,768 | 50,907 | 9,131 | 13,442 | 9,975 | 10,222 | 8,137 | 571 |
| Female | 71,458 | 2,291 | 69,167 | 92 | 14,374 | 193 | 305 | 547 | 998 | 1,743 | 3,136 | 7,452 | 53,885 | 9,511 | 14,324 | 8,980 | 10,422 | 10,648 | 816 |
| **19** | | | | | | | | | | | | | | | | | | | |
| **Total** | **115,561** | **3,470** | **112,091** | **165** | **19,844** | **276** | **481** | **805** | **1,369** | **2,262** | **4,213** | **10,438** | **89,364** | **12,299** | **21,406** | **12,907** | **16,287** | **26,465** | **2,718** |
| Male | 54,819 | 1,280 | 53,539 | 70 | 8,329 | 113 | 174 | 322 | 530 | 923 | 1,783 | 4,484 | 44,045 | 5,824 | 10,190 | 7,060 | 8,784 | 12,187 | 1,095 |
| Female | 60,742 | 2,190 | 58,552 | 95 | 11,515 | 163 | 307 | 483 | 839 | 1,339 | 2,430 | 5,954 | 45,319 | 6,475 | 11,216 | 5,847 | 7,503 | 14,278 | 1,623 |
| **15 - 19** | | | | | | | | | | | | | | | | | | | |
| **Total** | **624,887** | **16,121** | **608,766** | **1,096** | **180,436** | **1,610** | **2,888** | **5,720** | **11,488** | **23,283** | **44,676** | **90,771** | **422,505** | **108,208** | **125,229** | **74,795** | **60,516** | **53,757** | **4,729** |
| Male | 295,121 | 6,509 | 288,612 | 499 | 87,205 | 734 | 1,266 | 2,733 | 5,570 | 11,341 | 22,161 | 43,400 | 198,981 | 50,971 | 58,313 | 36,696 | 29,202 | 23,799 | 1,927 |
| Female | 329,766 | 9,612 | 320,154 | 597 | 93,231 | 876 | 1,622 | 2,987 | 5,918 | 11,942 | 22,515 | 47,371 | 223,524 | 57,237 | 66,916 | 38,099 | 31,314 | 29,958 | 2,802 |
| **20 - 24** | | | | | | | | | | | | | | | | | | | |
| **Total** | **519,206** | **20,741** | **498,465** | **610** | **97,119** | **1,445** | **2,873** | **4,684** | **8,057** | **12,385** | **19,291** | **48,384** | **351,370** | **38,718** | **81,516** | **32,058** | **39,971** | **159,107** | **49,366** |
| Male | 235,508 | 6,780 | 228,728 | 244 | 34,061 | 498 | 945 | 1,412 | 2,530 | 3,826 | 6,449 | 18,401 | 172,631 | 15,797 | 35,792 | 16,788 | 22,140 | 82,114 | 21,792 |
| Female | 283,698 | 13,961 | 269,737 | 366 | 63,058 | 947 | 1,928 | 3,272 | 5,527 | 8,559 | 12,842 | 29,983 | 178,739 | 22,921 | 45,724 | 15,270 | 17,831 | 76,993 | 27,574 |
| **25 - 29** | | | | | | | | | | | | | | | | | | | |
| **Total** | **476,763** | **22,215** | **454,548** | **676** | **107,414** | **1,571** | **3,555** | **5,237** | **8,837** | **13,319** | **20,062** | **54,833** | **254,062** | **31,782** | **74,209** | **19,949** | **17,273** | **110,849** | **92,396** |
| Male | 223,357 | 7,291 | 216,066 | 186 | 36,384 | 472 | 1,095 | 1,481 | 2,677 | 3,921 | 6,366 | 20,372 | 131,811 | 12,544 | 32,959 | 10,634 | 9,755 | 65,919 | 47,685 |
| Female | 253,406 | 14,924 | 238,482 | 490 | 71,030 | 1,099 | 2,460 | 3,756 | 6,160 | 9,398 | 13,696 | 34,461 | 122,251 | 19,238 | 41,250 | 9,315 | 7,518 | 44,930 | 44,711 |
| **30 - 34** | | | | | | | | | | | | | | | | | | | |
| **Total** | **380,838** | **17,750** | **363,088** | **497** | **90,957** | **1,242** | **2,664** | **3,929** | **6,846** | **10,171** | **16,365** | **49,740** | **186,678** | **25,435** | **69,058** | **16,146** | **11,422** | **64,617** | **84,956** |
| Male | 194,187 | 6,010 | 188,177 | 171 | 32,924 | 469 | 884 | 1,171 | 2,079 | 3,175 | 5,688 | 19,458 | 105,363 | 11,314 | 33,356 | 9,634 | 7,155 | 43,904 | 49,719 |
| Female | 186,651 | 11,740 | 174,911 | 326 | 58,033 | 773 | 1,780 | 2,758 | 4,767 | 6,996 | 10,677 | 30,282 | 81,315 | 14,121 | 35,702 | 6,512 | 4,267 | 20,713 | 35,237 |
| **35 - 39** | | | | | | | | | | | | | | | | | | | |
| **Total** | **295,843** | **15,009** | **280,834** | **344** | **73,537** | **997** | **2,152** | **3,256** | **5,577** | **7,715** | **12,799** | **41,041** | **139,609** | **18,107** | **54,800** | **13,122** | **8,239** | **45,341** | **67,344** |
| Male | 159,311 | 5,094 | 154,217 | 131 | 27,754 | 364 | 725 | 1,002 | 1,786 | 2,561 | 4,648 | 16,668 | 83,261 | 8,690 | 28,353 | 8,046 | 5,434 | 32,738 | 43,071 |
| Female | 136,532 | 9,915 | 126,617 | 213 | 45,783 | 633 | 1,427 | 2,254 | 3,791 | 5,154 | 8,151 | 24,373 | 56,348 | 9,417 | 26,447 | 5,076 | 2,805 | 12,603 | 24,273 |
| **40 - 44** | | | | | | | | | | | | | | | | | | | |
| **Total** | **192,620** | **10,817** | **181,803** | **249** | **50,246** | **670** | **1,587** | **2,332** | **3,852** | **4,928** | **8,383** | **28,494** | **83,627** | **9,943** | **31,706** | **10,345** | **4,832** | **26,801** | **47,681** |
| Male | 105,209 | 3,443 | 101,766 | 83 | 18,385 | 218 | 506 | 706 | 1,113 | 1,546 | 2,910 | 11,386 | 51,195 | 5,020 | 17,508 | 5,859 | 3,053 | 19,755 | 32,103 |
| Female | 87,411 | 7,374 | 80,037 | 166 | 31,861 | 452 | 1,081 | 1,626 | 2,739 | 3,382 | 5,473 | 17,108 | 32,432 | 4,923 | 14,198 | 4,486 | 1,779 | 7,046 | 15,578 |
| **45 - 49** | | | | | | | | | | | | | | | | | | | |
| **Total** | **145,121** | **9,619** | **135,502** | **226** | **42,439** | **642** | **1,524** | **2,363** | **3,517** | **3,936** | **6,717** | **23,740** | **57,318** | **6,120** | **17,915** | **11,465** | **3,577** | **18,241** | **35,519** |
| Male | 73,930 | 2,562 | 71,368 | 66 | 13,974 | 146 | 403 | 642 | 886 | 1,059 | 2,141 | 8,697 | 33,576 | 2,779 | 9,443 | 6,139 | 2,157 | 13,058 | 23,752 |
| Female | 71,191 | 7,057 | 64,134 | 160 | 28,465 | 496 | 1,121 | 1,721 | 2,631 | 2,877 | 4,576 | 15,043 | 23,742 | 3,341 | 8,472 | 5,326 | 1,420 | 5,183 | 11,767 |
| **50 - 54** | | | | | | | | | | | | | | | | | | | |
| **Total** | **107,859** | **9,155** | **98,704** | **230** | **33,661** | **779** | **1,729** | **2,354** | **3,231** | **3,279** | **5,143** | **17,146** | **37,230** | **3,945** | **11,015** | **8,302** | **2,260** | **11,708** | **27,583** |
| Male | 52,828 | 2,007 | 50,821 | 62 | 10,891 | 167 | 393 | 641 | 795 | 862 | 1,601 | 6,432 | 21,911 | 1,625 | 5,446 | 4,958 | 1,470 | 8,412 | 17,957 |
| Female | 55,031 | 7,148 | 47,883 | 168 | 22,770 | 612 | 1,336 | 1,713 | 2,436 | 2,417 | 3,542 | 10,714 | 15,319 | 2,320 | 5,569 | 3,344 | 790 | 3,296 | 9,626 |

## Table D4: Population (De Facto) 5 Years and Older by Age (Single and Grouped), Sex, Highest Level of Education Completed and Urban, Zambia 2010

| Age and Sex | Population 5 Years and Older | Total Never Attended | Total Ever Attended | Highest Level of Education Completed (Among those Ever Attended School) | | | | | | | | | | | | | | | |
|---|---|---|---|---|---|---|---|---|---|---|---|---|---|---|---|---|---|---|---|
| | | | | None | Primary School Grade 1 - 7 | | | | | | | | Secondary School Grade 8 - 12 | | | | | | Tertiary |
| | | | | | Total | 1 | 2 | 3 | 4 | 5 | 6 | 7 | Total | 8 | 9 | 10 | 11 | 12 | |
| **55 - 59** | | | | | | | | | | | | | | | | | | | |
| **Total** | **73,317** | **7,479** | **65,838** | **173** | **22,873** | **797** | **1,767** | **1,848** | **2,493** | **2,440** | **3,565** | **9,963** | **22,879** | **2,605** | **7,259** | **4,203** | **1,357** | **7,455** | **19,913** |
| Male | 37,812 | 1,464 | 36,348 | 35 | 8,351 | 186 | 444 | 538 | 694 | 784 | 1,296 | 4,409 | 14,570 | 1,252 | 4,035 | 2,900 | 953 | 5,430 | 13,392 |
| Female | 35,505 | 6,015 | 29,490 | 138 | 14,522 | 611 | 1,323 | 1,310 | 1,799 | 1,656 | 2,269 | 5,554 | 8,309 | 1,353 | 3,224 | 1,303 | 404 | 2,025 | 6,521 |
| **60 - 64** | | | | | | | | | | | | | | | | | | | |
| **Total** | **54,837** | **9,357** | **45,480** | **209** | **19,490** | **1,041** | **2,389** | **2,247** | **2,879** | **2,417** | **3,089** | **5,428** | **13,599** | **1,652** | **4,459** | **1,974** | **915** | **4,599** | **12,182** |
| Male | 27,658 | 1,681 | 25,977 | 54 | 7,569 | 228 | 638 | 687 | 934 | 912 | 1,345 | 2,825 | 9,498 | 946 | 2,953 | 1,398 | 678 | 3,523 | 8,856 |
| Female | 27,179 | 7,676 | 19,503 | 155 | 11,921 | 813 | 1,751 | 1,560 | 1,945 | 1,505 | 1,744 | 2,603 | 4,101 | 706 | 1,506 | 576 | 237 | 1,076 | 3,326 |
| **65 +** | | | | | | | | | | | | | | | | | | | |
| **Total** | **92,075** | **28,849** | **63,226** | **645** | **36,016** | **3,219** | **5,369** | **4,859** | **5,739** | **4,408** | **5,190** | **7,232** | **15,456** | **2,202** | **5,051** | **1,882** | **1,061** | **5,260** | **11,109** |
| Male | 42,859 | 6,115 | 36,744 | 159 | 17,245 | 1,069 | 2,005 | 1,990 | 2,730 | 2,250 | 3,114 | 4,087 | 10,718 | 1,372 | 3,409 | 1,334 | 760 | 3,843 | 8,622 |
| Female | 49,216 | 22,734 | 26,482 | 486 | 18,771 | 2,150 | 3,364 | 2,869 | 3,009 | 2,158 | 2,076 | 3,145 | 4,738 | 830 | 1,642 | 548 | 301 | 1,417 | 2,487 |

## Table D5: Population (De Facto) 5 Years and Older by Field of Study, Highest Professional/Vocational Qualification Completed and Sex, Zambia 2010

| Field of Study | Population 5 Years and Older | Highest Profession/Vocational Qualification Completed | | | | | |
|---|---|---|---|---|---|---|---|
| | | None | Certificate | Diploma | Bachelor's Degree | Master's Degree | PhD |
| **Zambia Total** | **10,311,427** | **9,764,742** | **328,972** | **164,858** | **41,740** | **10,037** | **1,078** |
| Natural science (e.g. biological science programme chemistry programme geological programme etc). | 4,040 | - | 934 | 1,265 | 1,358 | 385 | 98 |
| Civil engineering | 5,116 | - | 2,266 | 1,442 | 1,159 | 231 | 18 |
| Electrical and electronics engineering | 23,819 | 357 | 15,590 | 6,140 | 1,491 | 225 | 16 |
| Mechanical engineering | 29,590 | 595 | 20,690 | 6,615 | 1,456 | 207 | 27 |
| Chemical engineering | 1,428 | - | 534 | 429 | 388 | 72 | 5 |
| Mining engineering | 4,275 | 205 | 2,316 | 1,053 | 590 | 102 | 9 |
| Industrial engineering | 1,978 | - | 1,477 | 382 | 93 | 26 | - |
| Metallurgical engineering | 1,534 | - | 796 | 383 | 314 | 32 | 9 |
| Architectural and town planning engineering | 1,665 | - | 730 | 411 | 426 | 89 | 9 |
| Other engineering | 8,276 | 182 | 5,383 | 1,991 | 567 | 134 | 19 |
| Medicine and surgery | 3,908 | - | 570 | 1,385 | 1,265 | 535 | 153 |
| Pharmacy | 3,448 | - | 1,662 | 1,305 | 395 | 81 | 5 |
| Dentistry | 1,217 | - | 704 | 395 | 94 | 22 | 2 |
| Nursing | 24,940 | - | 15,529 | 8,832 | 464 | 107 | 8 |
| Medical technology | 2,979 | - | 747 | 1,535 | 541 | 131 | 25 |
| X-Ray technology | 427 | - | 122 | 179 | 54 | 62 | 10 |
| Veterinary | 1,482 | - | 886 | 335 | 167 | 70 | 24 |
| Statistics | 541 | - | 190 | 169 | 138 | 37 | 7 |
| Mathematics | 1,062 | - | 211 | 486 | 301 | 53 | 11 |
| Computer science/Economics | 23,253 | - | 11,925 | 7,062 | 3,260 | 915 | 91 |
| Accountancy | 39,747 | - | 14,292 | 19,318 | 5,146 | 922 | 69 |
| Teacher training | 121,681 | 1,774 | 72,734 | 39,815 | 6,303 | 981 | 74 |
| Law and jurisprudence (includes magistrates and judges) | 8,160 | - | 3,312 | 2,744 | 1,723 | 352 | 29 |
| Journalism | 3,189 | - | 1,027 | 1,849 | 243 | 63 | 7 |
| Fine arts | 2,585 | 98 | 1,548 | 451 | 440 | 46 | 2 |
| Physical education | 870 | 30 | 438 | 258 | 108 | 31 | 5 |
| Library science | 1,154 | - | 503 | 397 | 212 | 38 | 4 |
| Social welfare | 9,956 | 164 | 4,688 | 4,019 | 878 | 194 | 13 |
| Criminology | 3,638 | - | 3,130 | 440 | 53 | 13 | 2 |
| Business administration and related programmes | 38,580 | - | 13,065 | 19,245 | 4,560 | 1,655 | 55 |
| Secretarial training | 15,764 | - | 11,948 | 3,644 | 147 | 23 | 2 |
| shorthand typing | 3,006 | 101 | 2,545 | 327 | 24 | 8 | 1 |
| Clerical Typing | 2,543 | 96 | 2,146 | 280 | 18 | 3 | - |
| Operating of office machines | 1,304 | 43 | 993 | 238 | 23 | 7 | - |
| Service trade (e.g. cooking tourist trade etc.) | 12,800 | 419 | 10,445 | 1,816 | 102 | 16 | 2 |
| Radio and television broadcasting | 836 | 15 | 384 | 311 | 106 | 20 | - |
| Fire protection and fire fighting | 1,160 | 240 | 769 | 134 | 9 | 8 | - |
| Agriculture forestry and fishery | 15,171 | 1,028 | 9,166 | 3,475 | 1,072 | 369 | 61 |
| Food and drinks processing trades programmes | 6,807 | 232 | 5,635 | 856 | 68 | 15 | 1 |
| Wood working | 10,265 | 565 | 9,282 | 371 | 37 | 10 | - |
| Textile trades | 8,091 | 393 | 7,404 | 260 | 31 | 3 | - |
| Leather trades | 707 | 43 | 604 | 46 | 9 | 5 | - |
| Other programmes | 104,995 | 6,221 | 68,153 | 22,770 | 5,907 | 1,739 | 205 |
| None | 9,753,440 | 9,751,941 | 1,499 | - | - | - | - |

## Table D5: Population (De Facto) 5 Years and Older by Field of Study, Highest Professional/Vocational Qualification Completed and Sex, Zambia 2010

| Field of Study | Population 5 Years and Older | Highest Profession/Vocational Qualification Completed | | | | | |
|---|---|---|---|---|---|---|---|
| | | None | Certificate | Diploma | Bachelor's Degree | Master's Degree | PhD |
| **MALE** | **5,014,981** | **4,678,948** | **196,748** | **101,269** | **29,837** | **7,295** | **884** |
| Natural science (e.g. biological science programme chemistry programme geological programme etc). | 3,008 | - | 758 | 914 | 968 | 283 | 85 |
| Civil engineering | 4,869 | - | 2,163 | 1,365 | 1,101 | 222 | 18 |
| Electrical and electronics engineering | 21,985 | 303 | 14,273 | 5,771 | 1,411 | 212 | 15 |
| Mechanical engineering | 28,812 | 565 | 20,109 | 6,474 | 1,436 | 202 | 26 |
| Chemical engineering | 1,317 | - | 506 | 397 | 348 | 61 | 5 |
| Mining engineering | 4,117 | 168 | 2,236 | 1,028 | 575 | 101 | 9 |
| Industrial engineering | 1,758 | - | 1,290 | 357 | 88 | 23 | - |
| Metallurgical engineering | 1,455 | - | 746 | 371 | 301 | 29 | 8 |
| Architectural and town planning engineering | 1,411 | - | 588 | 383 | 356 | 75 | 9 |
| Other engineering | 7,814 | 161 | 5,113 | 1,879 | 516 | 126 | 19 |
| Medicine and surgery | 2,988 | - | 461 | 1,090 | 934 | 392 | 111 |
| Pharmacy | 2,185 | - | 1,048 | 825 | 262 | 47 | 3 |
| Dentistry | 826 | - | 474 | 263 | 70 | 17 | 2 |
| Nursing | 8,792 | - | 5,948 | 2,711 | 106 | 23 | 4 |
| Medical technology | 2,221 | - | 549 | 1,160 | 406 | 83 | 23 |
| X-Ray technology | 296 | - | 86 | 118 | 41 | 45 | 6 |
| Veterinary | 1,275 | - | 783 | 278 | 133 | 60 | 21 |
| Statistics | 432 | - | 154 | 133 | 108 | 30 | 7 |
| Mathematics | 820 | - | 149 | 379 | 248 | 33 | 11 |
| Computer science/Economics | 13,391 | - | 5,667 | 4,522 | 2,431 | 693 | 78 |
| Accountancy | 28,703 | - | 10,149 | 13,970 | 3,813 | 714 | 57 |
| Teacher training | 55,870 | 818 | 29,913 | 20,546 | 3,902 | 632 | 59 |
| Law and jurisprudence (includes magistrates and judges) | 5,847 | - | 2,594 | 1,829 | 1,174 | 229 | 21 |
| Journalism | 1,666 | - | 553 | 936 | 126 | 45 | 6 |
| Fine arts | 1,739 | 60 | 1,102 | 332 | 223 | 20 | 2 |
| Physical education | 507 | 18 | 240 | 154 | 69 | 22 | 4 |
| Library science | 560 | - | 263 | 175 | 101 | 18 | 3 |
| Social welfare | 3,890 | 72 | 1,815 | 1,435 | 453 | 105 | 10 |
| Criminology | 3,049 | - | 2,614 | 379 | 45 | 9 | 2 |
| Business administration and related programmes | 23,063 | - | 7,832 | 11,030 | 2,944 | 1,216 | 41 |
| Secretarial training | 1,096 | - | 829 | 231 | 28 | 6 | 2 |
| shorthand typing | 720 | 26 | 552 | 127 | 11 | 4 | - |
| Clerical Typing | 952 | 44 | 741 | 151 | 13 | 3 | - |
| Operating of office machines | 941 | 28 | 718 | 171 | 20 | 4 | - |
| Service trade (e.g. cooking tourist trade etc.) | 4,559 | 168 | 3,703 | 635 | 45 | 7 | 1 |
| Radio and television broadcasting | 603 | 8 | 280 | 223 | 77 | 15 | - |
| Fire protection and fire fighting | 938 | 118 | 684 | 120 | 8 | 8 | - |
| Agriculture forestry and fishery | 12,245 | 691 | 7,406 | 2,927 | 874 | 297 | 50 |
| Food and drinks processing trades programmes | 2,302 | 89 | 1,857 | 316 | 32 | 8 | - |
| Wood working | 9,829 | 541 | 8,926 | 322 | 33 | 7 | - |
| Textile trades | 1,594 | 86 | 1,396 | 94 | 17 | 1 | - |
| Leather trades | 274 | 18 | 217 | 27 | 7 | 5 | - |
| Other programmes | 72,960 | 4,363 | 48,564 | 14,721 | 3,983 | 1,163 | 166 |
| None | 4,671,302 | 4,670,603 | 699 | - | - | - | - |

## Table D5: Population (De Facto) 5 Years and Older by Field of Study, Highest Professional/Vocational Qualification Completed and Sex, Zambia 2010

| Field of Study | Population 5 Years and Older | Highest Profession/Vocational Qualification Completed | | | | | |
|---|---|---|---|---|---|---|---|
| | | None | Certificate | Diploma | Bachelor's Degree | Master's Degree | PhD |
| **FEMALE** | **5,296,446** | **5,085,794** | **132,224** | **63,589** | **11,903** | **2,742** | **194** |
| Natural science (e.g. biological science programme chemistry programme geological programme etc). | 1,032 | - | 176 | 351 | 390 | 102 | 13 |
| Civil engineering | 247 | - | 103 | 77 | 58 | 9 | - |
| Electrical and electronics engineering | 1,834 | 54 | 1,317 | 369 | 80 | 13 | 1 |
| Mechanical engineering | 778 | 30 | 581 | 141 | 20 | 5 | 1 |
| Chemical engineering | 111 | - | 28 | 32 | 40 | 11 | - |
| Mining engineering | 158 | 37 | 80 | 25 | 15 | 1 | - |
| Industrial engineering | 220 | - | 187 | 25 | 5 | 3 | - |
| Metallurgical engineering | 79 | - | 50 | 12 | 13 | 3 | 1 |
| Architectural and town planning engineering | 254 | - | 142 | 28 | 70 | 14 | - |
| Other engineering | 462 | 21 | 270 | 112 | 51 | 8 | - |
| Medicine and surgery | 920 | - | 109 | 295 | 331 | 143 | 42 |
| Pharmacy | 1,263 | - | 614 | 480 | 133 | 34 | 2 |
| Dentistry | 391 | - | 230 | 132 | 24 | 5 | - |
| Nursing | 16,148 | - | 9,581 | 6,121 | 358 | 84 | 4 |
| Medical technology | 758 | - | 198 | 375 | 135 | 48 | 2 |
| X-Ray technology | 131 | - | 36 | 61 | 13 | 17 | 4 |
| Veterinary | 207 | - | 103 | 57 | 34 | 10 | 3 |
| Statistics | 109 | - | 36 | 36 | 30 | 7 | - |
| Mathematics | 242 | - | 62 | 107 | 53 | 20 | - |
| Computer science/Economics | 9,862 | - | 6,258 | 2,540 | 829 | 222 | 13 |
| Accountancy | 11,044 | - | 4,143 | 5,348 | 1,333 | 208 | 12 |
| Teacher training | 65,811 | 956 | 42,821 | 19,269 | 2,401 | 349 | 15 |
| Law and jurisprudence (includes magistrates and judges) | 2,313 | - | 718 | 915 | 549 | 123 | 8 |
| Journalism | 1,523 | - | 474 | 913 | 117 | 18 | 1 |
| Fine arts | 846 | 38 | 446 | 119 | 217 | 26 | - |
| Physical education | 363 | 12 | 198 | 104 | 39 | 9 | 1 |
| Library science | 594 | - | 240 | 222 | 111 | 20 | 1 |
| Social welfare | 6,066 | 92 | 2,873 | 2,584 | 425 | 89 | 3 |
| Criminology | 589 | - | 516 | 61 | 8 | 4 | - |
| Business administration and related programmes | 15,517 | - | 5,233 | 8,215 | 1,616 | 439 | 14 |
| Secretarial training | 14,668 | - | 11,119 | 3,413 | 119 | 17 | - |
| shorthand typing | 2,286 | 75 | 1,993 | 200 | 13 | 4 | 1 |
| Clerical Typing | 1,591 | 52 | 1,405 | 129 | 5 | - | - |
| Operating of office machines | 363 | 15 | 275 | 67 | 3 | 3 | - |
| Service trade (e.g. cooking tourist trade etc.) | 8,241 | 251 | 6,742 | 1,181 | 57 | 9 | 1 |
| Radio and television broadcasting | 233 | 7 | 104 | 88 | 29 | 5 | - |
| Fire protection and fire fighting | 222 | 122 | 85 | 14 | 1 | - | - |
| Agriculture forestry and fishery | 2,926 | 337 | 1,760 | 548 | 198 | 72 | 11 |
| Food and drinks processing trades programmes | 4,505 | 143 | 3,778 | 540 | 36 | 7 | 1 |
| Wood working | 436 | 24 | 356 | 49 | 4 | 3 | - |
| Textile trades | 6,497 | 307 | 6,008 | 166 | 14 | 2 | - |
| Leather trades | 433 | 25 | 387 | 19 | 2 | - | - |
| Other programmes | 32,035 | 1,858 | 19,589 | 8,049 | 1,924 | 576 | 39 |
| None | 5,082,138 | 5,081,338 | 800 | - | - | - | - |

## Table D6: Population (De Facto) by Sex, Current Primary School Attendance, Net and Gross Attendance Ratios and Rural/Urban, Zambia 2010

| Primary School Attendance | Total | | | Rural | | | Urban | | |
|---|---|---|---|---|---|---|---|---|---|
| | Population | Male | Female | Total | Male | Female | Total | Male | Female |
| **Zambia Total** | **12,526,314** | **6,117,253** | **6,409,061** | **7,505,292** | **3,664,349** | **3,840,943** | **5,021,022** | **2,452,904** | **2,568,118** |
| Total Population Aged 7-13 Years | 2,448,959 | 1,214,818 | 1,234,141 | 1,550,726 | 781,778 | 768,948 | 898,233 | 433,040 | 465,193 |
| Total Population Currently Attending Primary School | 2,372,844 | 1,190,512 | 1,182,332 | 1,431,252 | 731,468 | 699,784 | 941,592 | 459,044 | 482,548 |
| Total Population Aged 7-13 Years & Currently Attending Primary School | 1,753,042 | 861,835 | 891,207 | 1,038,095 | 515,757 | 522,338 | 714,947 | 346,078 | 368,869 |
| Gross Attendance Ratio | 0.9689 | 0.9800 | 0.9580 | 0.9230 | 0.9356 | 0.9101 | 1.0483 | 1.0600 | 1.0373 |
| Net Attendance Ratio | 0.7158 | 0.7094 | 0.7221 | 0.6694 | 0.6597 | 0.6793 | 0.7959 | 0.7992 | 0.7929 |

## Table D7: Population (De Facto) by Sex, Current Secondary School Attendance, Net and Gross Attendance Ratios and Rural/Urban, Zambia 2010

| Secondary School Attendance | Total | | | Rural | | | Urban | | |
|---|---|---|---|---|---|---|---|---|---|
| | Population | Male | Female | Total | Male | Female | Total | Male | Female |
| | | | | | | | | | |
| **Zambia Total** | **12,526,314** | **6,117,253** | **6,409,061** | **7,505,292** | **3,664,349** | **3,840,943** | **5,021,022** | **2,452,904** | **2,568,118** |
| Total Population Aged 14-18 Years | 1,479,918 | 723,051 | 756,867 | 844,428 | 423,767 | 420,661 | 635,490 | 299,284 | 336,206 |
| Total Population Currently Attending Secondary School | 1,017,251 | 539,112 | 478,139 | 429,632 | 245,731 | 183,901 | 587,619 | 293,381 | 294,238 |
| Total Population Aged 14-18 Years & Currently Attending Primary School | 674,102 | 339,713 | 334,389 | 278,749 | 148,277 | 130,472 | 395,353 | 191,436 | 203,917 |
| Gross Attendance Ratio | 0.6874 | 0.7456 | 0.6317 | 0.5088 | 0.5799 | 0.4372 | 0.9247 | 0.9803 | 0.8752 |
| Net Attendance Ratio | 0.4555 | 0.4698 | 0.4418 | 0.3301 | 0.3499 | 0.3102 | 0.6221 | 0.6396 | 0.6065 |

# Economic Activity Tables

## Table E1: Usually Working Population (De Facto) 12 Years and Older by Employment Status, Sex and Province, Zambia 2010

| Province and Sex | Population 12 Years and Older | Usually Working Population | Employment Status | | | |
|---|---|---|---|---|---|---|
| | | | Employer | Employee | Self Employed | Unpaid Family Worker |
| **Zambia Total** | | | | | | |
| **Total** | **7,715,022** | **3,887,052** | **29,033** | **864,942** | **1,715,082** | **1,277,995** |
| Male | 3,722,621 | 2,140,733 | 21,227 | 629,825 | 1,004,229 | 485,452 |
| Female | 3,992,401 | 1,746,319 | 7,806 | 235,117 | 710,853 | 792,543 |
| **Central Province** | | | | | | |
| **Total** | **754,850** | **362,862** | **2,510** | **66,901** | **168,940** | **124,511** |
| Male | 368,829 | 206,181 | 1,843 | 48,843 | 105,297 | 50,198 |
| Female | 386,021 | 156,681 | 667 | 18,058 | 63,643 | 74,313 |
| **Copperbelt Province** | | | | | | |
| **Total** | **1,278,711** | **512,184** | **6,113** | **227,400** | **183,554** | **95,117** |
| Male | 632,823 | 318,536 | 4,498 | 171,895 | 104,441 | 37,702 |
| Female | 645,888 | 193,648 | 1,615 | 55,505 | 79,113 | 57,415 |
| **Eastern Province** | | | | | | |
| **Total** | **908,683** | **572,288** | **2,072** | **39,291** | **293,732** | **237,193** |
| Male | 436,842 | 292,678 | 1,530 | 29,195 | 173,414 | 88,539 |
| Female | 471,841 | 279,610 | 542 | 10,096 | 120,318 | 148,654 |
| **Luapula Province** | | | | | | |
| **Total** | **549,692** | **326,321** | **1,544** | **26,025** | **159,178** | **139,574** |
| Male | 260,807 | 168,851 | 1,204 | 19,482 | 97,465 | 50,700 |
| Female | 288,885 | 157,470 | 340 | 6,543 | 61,713 | 88,874 |
| **Lusaka Province** | | | | | | |
| **Total** | **1,423,981** | **569,351** | **9,792** | **331,209** | **198,827** | **29,523** |
| Male | 696,884 | 369,218 | 6,993 | 234,266 | 114,938 | 13,021 |
| Female | 727,097 | 200,133 | 2,799 | 96,943 | 83,889 | 16,502 |
| **Muchinga Province** | | | | | | |
| **Total** | **394,054** | **248,373** | **820** | **15,872** | **118,113** | **113,568** |
| Male | 187,585 | 124,049 | 625 | 11,837 | 72,414 | 39,173 |
| Female | 206,469 | 124,324 | 195 | 4,035 | 45,699 | 74,395 |
| **Northern Province** | | | | | | |
| **Total** | **603,022** | **350,998** | **1,770** | **25,549** | **162,867** | **160,812** |
| Male | 287,647 | 181,367 | 1,330 | 19,412 | 93,766 | 66,859 |
| Female | 315,375 | 169,631 | 440 | 6,137 | 69,101 | 93,953 |
| **North-Western Province** | | | | | | |
| **Total** | **396,298** | **208,269** | **1,276** | **26,404** | **106,704** | **73,885** |
| Male | 189,864 | 105,185 | 959 | 20,135 | 55,402 | 28,689 |
| Female | 206,434 | 103,084 | 317 | 6,269 | 51,302 | 45,196 |
| **Southern Province** | | | | | | |
| **Total** | **903,297** | **430,858** | **2,284** | **85,350** | **185,864** | **157,360** |
| Male | 432,997 | 232,877 | 1,659 | 60,628 | 115,274 | 55,316 |
| Female | 470,300 | 197,981 | 625 | 24,722 | 70,590 | 102,044 |
| **Western Province** | | | | | | |
| **Total** | **502,434** | **305,548** | **852** | **20,941** | **137,303** | **146,452** |
| Male | 228,343 | 141,791 | 586 | 14,132 | 71,818 | 55,255 |
| Female | 274,091 | 163,757 | 266 | 6,809 | 65,485 | 91,197 |

## Table E2: Usually Working Population (De Facto) 12 Years and Older by Employment Status, Age Group, Sex and Rural/Urban, Zambia 2010

| Age Group and Sex | Population 12 Years and Older | Usually Working Population | Employment Status | | | |
|---|---|---|---|---|---|---|
| | | | Employer | Employee | Self Employed | Unpaid Family Worker |
| **Zambia Total** | | | | | | |
| **Total** | **7,715,022** | **3,887,052** | **29,033** | **864,942** | **1,715,082** | **1,277,995** |
| 12 - 14 | 958,973 | 118,554 | 94 | 2,301 | 20,023 | 96,136 |
| 15 - 19 | 1,427,884 | 297,932 | 550 | 19,671 | 72,588 | 205,123 |
| 20 - 24 | 1,117,476 | 513,734 | 2,396 | 99,875 | 189,362 | 222,101 |
| 25 - 29 | 1,006,244 | 643,955 | 4,643 | 184,674 | 270,810 | 183,828 |
| 30 - 34 | 800,770 | 577,219 | 5,005 | 175,396 | 260,556 | 136,262 |
| 35 - 39 | 647,813 | 487,876 | 4,598 | 138,219 | 232,857 | 112,202 |
| 40 - 44 | 447,584 | 344,306 | 3,392 | 90,155 | 170,614 | 80,145 |
| 45 - 49 | 354,989 | 272,125 | 2,615 | 64,943 | 137,965 | 66,602 |
| 50 - 54 | 270,254 | 202,063 | 2,033 | 44,583 | 104,685 | 50,762 |
| 55 - 59 | 184,828 | 132,250 | 1,433 | 22,181 | 73,492 | 35,144 |
| 60 - 64 | 161,875 | 109,614 | 974 | 11,495 | 64,766 | 32,379 |
| 65 - 69 | 118,582 | 77,547 | 580 | 6,036 | 47,328 | 23,603 |
| 70 - 74 | 90,802 | 51,849 | 362 | 2,900 | 32,883 | 15,704 |
| 75+ | 126,948 | 58,028 | 358 | 2,513 | 37,153 | 18,004 |
| **Male** | | | | | | |
| **Total** | **3,722,621** | **2,140,733** | **21,227** | **629,825** | **1,004,229** | **485,452** |
| 12 - 14 | 472,869 | 60,665 | 63 | 1,410 | 10,163 | 49,029 |
| 15 - 19 | 692,859 | 133,822 | 350 | 12,152 | 31,119 | 90,201 |
| 20 - 24 | 504,876 | 254,713 | 1,547 | 66,910 | 100,986 | 85,270 |
| 25 - 29 | 464,493 | 357,104 | 3,203 | 128,373 | 162,763 | 62,765 |
| 30 - 34 | 397,694 | 338,696 | 3,640 | 127,265 | 161,874 | 45,917 |
| 35 - 39 | 332,961 | 290,449 | 3,488 | 103,805 | 145,265 | 37,891 |
| 40 - 44 | 232,253 | 204,350 | 2,594 | 69,169 | 104,972 | 27,615 |
| 45 - 49 | 174,833 | 154,049 | 1,983 | 49,511 | 80,538 | 22,017 |
| 50 - 54 | 128,696 | 111,207 | 1,524 | 34,207 | 58,735 | 16,741 |
| 55 - 59 | 90,037 | 72,933 | 1,107 | 17,625 | 41,870 | 12,331 |
| 60 - 64 | 73,419 | 56,629 | 729 | 9,539 | 35,324 | 11,037 |
| 65 - 69 | 53,695 | 40,855 | 432 | 5,176 | 26,496 | 8,751 |
| 70 - 74 | 41,929 | 29,265 | 286 | 2,557 | 19,623 | 6,799 |
| 75+ | 62,007 | 35,996 | 281 | 2,126 | 24,501 | 9,088 |
| **Female** | | | | | | |
| **Total** | **3,992,401** | **1,746,319** | **7,806** | **235,117** | **710,853** | **792,543** |
| 12 - 14 | 486,104 | 57,889 | 31 | 891 | 9,860 | 47,107 |
| 15 - 19 | 735,025 | 164,110 | 200 | 7,519 | 41,469 | 114,922 |
| 20 - 24 | 612,600 | 259,021 | 849 | 32,965 | 88,376 | 136,831 |
| 25 - 29 | 541,751 | 286,851 | 1,440 | 56,301 | 108,047 | 121,063 |
| 30 - 34 | 403,076 | 238,523 | 1,365 | 48,131 | 98,682 | 90,345 |
| 35 - 39 | 314,852 | 197,427 | 1,110 | 34,414 | 87,592 | 74,311 |
| 40 - 44 | 215,331 | 139,956 | 798 | 20,986 | 65,642 | 52,530 |
| 45 - 49 | 180,156 | 118,076 | 632 | 15,432 | 57,427 | 44,585 |
| 50 - 54 | 141,558 | 90,856 | 509 | 10,376 | 45,950 | 34,021 |
| 55 - 59 | 94,791 | 59,317 | 326 | 4,556 | 31,622 | 22,813 |
| 60 - 64 | 88,456 | 52,985 | 245 | 1,956 | 29,442 | 21,342 |
| 65 - 69 | 64,887 | 36,692 | 148 | 860 | 20,832 | 14,852 |
| 70 - 74 | 48,873 | 22,584 | 76 | 343 | 13,260 | 8,905 |
| 75+ | 64,941 | 22,032 | 77 | 387 | 12,652 | 8,916 |

## Table E2: Usually Working Population (De Facto) 12 Years and Older by Employment Status, Age Group, Sex and Rural, Zambia 2010

| Age Group and Sex | Population 12 Years and Older | Usually Working Population | Employment Status | | | |
|---|---|---|---|---|---|---|
| | | | Employer | Employee | Self Employed | Unpaid Family Worker |
| **Zambia Rural** | | | | | | |
| **Total** | **4,366,877** | **2,624,368** | **9,434** | **194,631** | **1,241,111** | **1,179,192** |
| 12 - 14 | 574,194 | 109,491 | 62 | 1,485 | 17,740 | 90,204 |
| 15 - 19 | 802,997 | 262,207 | 234 | 7,247 | 61,981 | 192,745 |
| 20 - 24 | 598,270 | 375,523 | 690 | 24,059 | 144,457 | 206,317 |
| 25 - 29 | 529,481 | 401,585 | 1,439 | 40,497 | 190,092 | 169,557 |
| 30 - 34 | 419,932 | 337,115 | 1,538 | 37,745 | 172,810 | 125,022 |
| 35 - 39 | 351,970 | 287,729 | 1,331 | 28,835 | 154,785 | 102,778 |
| 40 - 44 | 254,964 | 211,016 | 1,027 | 18,923 | 117,707 | 73,359 |
| 45 - 49 | 209,868 | 174,484 | 855 | 14,158 | 98,848 | 60,623 |
| 50 - 54 | 162,395 | 133,872 | 672 | 10,369 | 77,036 | 45,795 |
| 55 - 59 | 111,511 | 91,214 | 477 | 4,902 | 54,610 | 31,225 |
| 60 - 64 | 107,038 | 83,565 | 392 | 2,768 | 51,224 | 29,181 |
| 65 - 69 | 83,157 | 62,429 | 280 | 1,744 | 39,023 | 21,382 |
| 70 - 74 | 66,388 | 43,838 | 208 | 939 | 28,304 | 14,387 |
| 75+ | 94,712 | 50,300 | 229 | 960 | 32,494 | 16,617 |
| **Male** | | | | | | |
| **Total** | **2,093,514** | **1,341,036** | **7,043** | **148,546** | **738,866** | **446,581** |
| 12 - 14 | 291,542 | 56,266 | 43 | 1,012 | 9,106 | 46,105 |
| 15 - 19 | 397,738 | 115,324 | 149 | 4,789 | 25,931 | 84,455 |
| 20 - 24 | 269,368 | 173,886 | 459 | 17,536 | 77,293 | 78,598 |
| 25 - 29 | 241,136 | 205,566 | 1,003 | 29,658 | 117,370 | 57,535 |
| 30 - 34 | 203,507 | 183,042 | 1,183 | 28,926 | 111,026 | 41,907 |
| 35 - 39 | 173,650 | 157,697 | 1,070 | 22,881 | 99,185 | 34,561 |
| 40 - 44 | 127,044 | 115,773 | 802 | 15,178 | 74,558 | 25,235 |
| 45 - 49 | 100,903 | 92,222 | 686 | 11,207 | 60,193 | 20,136 |
| 50 - 54 | 75,868 | 68,891 | 499 | 8,206 | 44,985 | 15,201 |
| 55 - 59 | 52,225 | 46,714 | 348 | 3,933 | 31,593 | 10,840 |
| 60 - 64 | 45,761 | 39,769 | 277 | 2,213 | 27,591 | 9,688 |
| 65 - 69 | 36,611 | 31,186 | 198 | 1,420 | 21,743 | 7,825 |
| 70 - 74 | 30,553 | 23,857 | 154 | 793 | 16,762 | 6,148 |
| 75+ | 47,608 | 30,843 | 172 | 794 | 21,530 | 8,347 |
| **Female** | | | | | | |
| **Total** | **2,273,363** | **1,283,332** | **2,391** | **46,085** | **502,245** | **732,611** |
| 12 - 14 | 282,652 | 53,225 | 19 | 473 | 8,634 | 44,099 |
| 15 - 19 | 405,259 | 146,883 | 85 | 2,458 | 36,050 | 108,290 |
| 20 - 24 | 328,902 | 201,637 | 231 | 6,523 | 67,164 | 127,719 |
| 25 - 29 | 288,345 | 196,019 | 436 | 10,839 | 72,722 | 112,022 |
| 30 - 34 | 216,425 | 154,073 | 355 | 8,819 | 61,784 | 83,115 |
| 35 - 39 | 178,320 | 130,032 | 261 | 5,954 | 55,600 | 68,217 |
| 40 - 44 | 127,920 | 95,243 | 225 | 3,745 | 43,149 | 48,124 |
| 45 - 49 | 108,965 | 82,262 | 169 | 2,951 | 38,655 | 40,487 |
| 50 - 54 | 86,527 | 64,981 | 173 | 2,163 | 32,051 | 30,594 |
| 55 - 59 | 59,286 | 44,500 | 129 | 969 | 23,017 | 20,385 |
| 60 - 64 | 61,277 | 43,796 | 115 | 555 | 23,633 | 19,493 |
| 65 - 69 | 46,546 | 31,243 | 82 | 324 | 17,280 | 13,557 |
| 70 - 74 | 35,835 | 19,981 | 54 | 146 | 11,542 | 8,239 |
| 75+ | 47,104 | 19,457 | 57 | 166 | 10,964 | 8,270 |

## Table E2: Usually Working Population (De Facto) 12 Years and Older by Employment Status, Age Group, Sex and Urban, Zambia 2010

| Age Group and Sex | Population 12 Years and Older | Usually Working Population | Employment Status | | | |
|---|---|---|---|---|---|---|
| | | | Employer | Employee | Self Employed | Unpaid Family Worker |
| **Zambia Urban** | | | | | | |
| **Total** | **3,348,145** | **1,262,684** | **19,599** | **670,311** | **473,971** | **98,803** |
| 12 - 14 | 384,779 | 9,063 | 32 | 816 | 2,283 | 5,932 |
| 15 - 19 | 624,887 | 35,725 | 316 | 12,424 | 10,607 | 12,378 |
| 20 - 24 | 519,206 | 138,211 | 1,706 | 75,816 | 44,905 | 15,784 |
| 25 - 29 | 476,763 | 242,370 | 3,204 | 144,177 | 80,718 | 14,271 |
| 30 - 34 | 380,838 | 240,104 | 3,467 | 137,651 | 87,746 | 11,240 |
| 35 - 39 | 295,843 | 200,147 | 3,267 | 109,384 | 78,072 | 9,424 |
| 40 - 44 | 192,620 | 133,290 | 2,365 | 71,232 | 52,907 | 6,786 |
| 45 - 49 | 145,121 | 97,641 | 1,760 | 50,785 | 39,117 | 5,979 |
| 50 - 54 | 107,859 | 68,191 | 1,361 | 34,214 | 27,649 | 4,967 |
| 55 - 59 | 73,317 | 41,036 | 956 | 17,279 | 18,882 | 3,919 |
| 60 - 64 | 54,837 | 26,049 | 582 | 8,727 | 13,542 | 3,198 |
| 65 - 69 | 35,425 | 15,118 | 300 | 4,292 | 8,305 | 2,221 |
| 70 - 74 | 24,414 | 8,011 | 154 | 1,961 | 4,579 | 1,317 |
| 75+ | 32,236 | 7,728 | 129 | 1,553 | 4,659 | 1,387 |
| **Male** | | | | | | |
| **Total** | **1,629,107** | **799,697** | **14,184** | **481,279** | **265,363** | **38,871** |
| 12 - 14 | 181,327 | 4,399 | 20 | 398 | 1,057 | 2,924 |
| 15 - 19 | 295,121 | 18,498 | 201 | 7,363 | 5,188 | 5,746 |
| 20 - 24 | 235,508 | 80,827 | 1,088 | 49,374 | 23,693 | 6,672 |
| 25 - 29 | 223,357 | 151,538 | 2,200 | 98,715 | 45,393 | 5,230 |
| 30 - 34 | 194,187 | 155,654 | 2,457 | 98,339 | 50,848 | 4,010 |
| 35 - 39 | 159,311 | 132,752 | 2,418 | 80,924 | 46,080 | 3,330 |
| 40 - 44 | 105,209 | 88,577 | 1,792 | 53,991 | 30,414 | 2,380 |
| 45 - 49 | 73,930 | 61,827 | 1,297 | 38,304 | 20,345 | 1,881 |
| 50 - 54 | 52,828 | 42,316 | 1,025 | 26,001 | 13,750 | 1,540 |
| 55 - 59 | 37,812 | 26,219 | 759 | 13,692 | 10,277 | 1,491 |
| 60 - 64 | 27,658 | 16,860 | 452 | 7,326 | 7,733 | 1,349 |
| 65 - 69 | 17,084 | 9,669 | 234 | 3,756 | 4,753 | 926 |
| 70 - 74 | 11,376 | 5,408 | 132 | 1,764 | 2,861 | 651 |
| 75+ | 14,399 | 5,153 | 109 | 1,332 | 2,971 | 741 |
| **Female** | | | | | | |
| **Total** | **1,719,038** | **462,987** | **5,415** | **189,032** | **208,608** | **59,932** |
| 12 - 14 | 203,452 | 4,664 | 12 | 418 | 1,226 | 3,008 |
| 15 - 19 | 329,766 | 17,227 | 115 | 5,061 | 5,419 | 6,632 |
| 20 - 24 | 283,698 | 57,384 | 618 | 26,442 | 21,212 | 9,112 |
| 25 - 29 | 253,406 | 90,832 | 1,004 | 45,462 | 35,325 | 9,041 |
| 30 - 34 | 186,651 | 84,450 | 1,010 | 39,312 | 36,898 | 7,230 |
| 35 - 39 | 136,532 | 67,395 | 849 | 28,460 | 31,992 | 6,094 |
| 40 - 44 | 87,411 | 44,713 | 573 | 17,241 | 22,493 | 4,406 |
| 45 - 49 | 71,191 | 35,814 | 463 | 12,481 | 18,772 | 4,098 |
| 50 - 54 | 55,031 | 25,875 | 336 | 8,213 | 13,899 | 3,427 |
| 55 - 59 | 35,505 | 14,817 | 197 | 3,587 | 8,605 | 2,428 |
| 60 - 64 | 27,179 | 9,189 | 130 | 1,401 | 5,809 | 1,849 |
| 65 - 69 | 18,341 | 5,449 | 66 | 536 | 3,552 | 1,295 |
| 70 - 74 | 13,038 | 2,603 | 22 | 197 | 1,718 | 666 |
| 75+ | 17,837 | 2,575 | 20 | 221 | 1,688 | 646 |

# Table E3: Population (De Facto) 12 Years and Older by Economic Activity Status, Age Group, Sex and Rural/Urban, Zambia 2010

| Age Group, Sex and Rural/Urban | Population 12 Years and Older | Economically Active | | | | | Economically Inactive | | | |
|---|---|---|---|---|---|---|---|---|---|---|
| | | Total Labour Force | Employed | Unemployed | | | Total | Full time Housewife/ homemaker | Full time student | Other |
| | | | | Total | Seeking work | Not seeking work but availabe for work | | | | |
| **Zambia Total** | | | | | | | | | | |
| **Total** | **7,715,022** | **4,430,542** | **3,887,052** | **543,490** | **370,059** | **173,431** | **3,284,480** | **880,506** | **1,792,900** | **611,074** |
| 12 - 14 | 958,973 | 137,835 | 118,554 | 19,281 | 7,106 | 12,175 | 821,138 | 19,072 | 720,543 | 81,523 |
| 15 - 19 | 1,427,884 | 382,796 | 297,932 | 84,864 | 47,538 | 37,326 | 1,045,088 | 86,833 | 833,075 | 125,180 |
| 20 - 24 | 1,117,476 | 666,110 | 513,734 | 152,376 | 109,058 | 43,318 | 451,366 | 183,454 | 182,914 | 84,998 |
| 25 - 29 | 1,006,244 | 748,108 | 643,955 | 104,153 | 78,353 | 25,800 | 258,136 | 179,181 | 33,711 | 45,244 |
| 30 - 34 | 800,770 | 638,897 | 577,219 | 61,678 | 46,203 | 15,475 | 161,873 | 125,367 | 8,816 | 27,690 |
| 35 - 39 | 647,813 | 530,440 | 487,876 | 42,564 | 31,390 | 11,174 | 117,373 | 92,187 | 4,402 | 20,784 |
| 40 - 44 | 447,584 | 370,024 | 344,306 | 25,718 | 18,362 | 7,356 | 77,560 | 58,235 | 2,205 | 17,120 |
| 45 - 49 | 354,989 | 290,689 | 272,125 | 18,564 | 12,743 | 5,821 | 64,300 | 46,226 | 1,429 | 16,645 |
| 50 - 54 | 270,254 | 214,018 | 202,063 | 11,955 | 7,444 | 4,511 | 56,236 | 32,845 | 1,105 | 22,286 |
| 55 - 59 | 184,828 | 140,558 | 132,250 | 8,308 | 4,871 | 3,437 | 44,270 | 19,697 | 848 | 23,725 |
| 60 - 64 | 161,875 | 115,535 | 109,614 | 5,921 | 3,141 | 2,780 | 46,340 | 15,117 | 825 | 30,398 |
| 65 - 69 | 118,582 | 81,198 | 77,547 | 3,651 | 1,808 | 1,843 | 37,384 | 9,656 | 665 | 27,063 |
| 70 - 74 | 90,802 | 53,933 | 51,849 | 2,084 | 985 | 1,099 | 36,869 | 5,896 | 861 | 30,112 |
| 75+ | 126,948 | 60,401 | 58,028 | 2,373 | 1,057 | 1,316 | 66,547 | 6,740 | 1,501 | 58,306 |
| **Male** | | | | | | | | | | |
| Total | 3,722,621 | 2,456,747 | 2,140,733 | 316,014 | 225,747 | 90,267 | 1,265,874 | 52,292 | 938,888 | 274,694 |
| 12 - 14 | 472,869 | 70,401 | 60,665 | 9,736 | 3,665 | 6,071 | 402,468 | 7,676 | 354,442 | 40,350 |
| 15 - 19 | 692,859 | 175,802 | 133,822 | 41,980 | 24,492 | 17,488 | 517,057 | 13,045 | 445,950 | 58,062 |
| 20 - 24 | 504,876 | 339,718 | 254,713 | 85,005 | 63,117 | 21,888 | 165,158 | 10,367 | 113,403 | 41,388 |
| 25 - 29 | 464,493 | 418,952 | 357,104 | 61,848 | 48,207 | 13,641 | 45,541 | 5,857 | 17,676 | 22,008 |
| 30 - 34 | 397,694 | 377,308 | 338,696 | 38,612 | 30,077 | 8,535 | 20,386 | 3,608 | 3,210 | 13,568 |
| 35 - 39 | 332,961 | 318,159 | 290,449 | 27,710 | 21,381 | 6,329 | 14,802 | 2,723 | 1,484 | 10,595 |
| 40 - 44 | 232,253 | 221,469 | 204,350 | 17,119 | 12,838 | 4,281 | 10,784 | 1,851 | 705 | 8,228 |
| 45 - 49 | 174,833 | 165,902 | 154,049 | 11,853 | 8,647 | 3,206 | 8,931 | 1,412 | 366 | 7,153 |
| 50 - 54 | 128,696 | 118,702 | 111,207 | 7,495 | 5,016 | 2,479 | 9,994 | 1,208 | 243 | 8,543 |
| 55 - 59 | 90,037 | 78,439 | 72,933 | 5,506 | 3,426 | 2,080 | 11,598 | 1,021 | 212 | 10,365 |
| 60 - 64 | 73,419 | 60,538 | 56,629 | 3,909 | 2,188 | 1,721 | 12,881 | 969 | 198 | 11,714 |
| 65 - 69 | 53,695 | 43,253 | 40,855 | 2,398 | 1,288 | 1,110 | 10,442 | 745 | 190 | 9,507 |
| 70 - 74 | 41,929 | 30,603 | 29,265 | 1,338 | 682 | 656 | 11,326 | 663 | 259 | 10,404 |
| 75+ | 62,007 | 37,501 | 35,996 | 1,505 | 723 | 782 | 24,506 | 1,147 | 550 | 22,809 |
| **Female** | | | | | | | | | | |
| **Total** | **3,992,401** | **1,973,795** | **1,746,319** | **227,476** | **144,312** | **83,164** | **2,018,606** | **828,214** | **854,012** | **336,380** |
| 12 - 14 | 486,104 | 67,434 | 57,889 | 9,545 | 3,441 | 6,104 | 418,670 | 11,396 | 366,101 | 41,173 |
| 15 - 19 | 735,025 | 206,994 | 164,110 | 42,884 | 23,046 | 19,838 | 528,031 | 73,788 | 387,125 | 67,118 |
| 20 - 24 | 612,600 | 326,392 | 259,021 | 67,371 | 45,941 | 21,430 | 286,208 | 173,087 | 69,511 | 43,610 |
| 25 - 29 | 541,751 | 329,156 | 286,851 | 42,305 | 30,146 | 12,159 | 212,595 | 173,324 | 16,035 | 23,236 |
| 30 - 34 | 403,076 | 261,589 | 238,523 | 23,066 | 16,126 | 6,940 | 141,487 | 121,759 | 5,606 | 14,122 |
| 35 - 39 | 314,852 | 212,281 | 197,427 | 14,854 | 10,009 | 4,845 | 102,571 | 89,464 | 2,918 | 10,189 |
| 40 - 44 | 215,331 | 148,555 | 139,956 | 8,599 | 5,524 | 3,075 | 66,776 | 56,384 | 1,500 | 8,892 |
| 45 - 49 | 180,156 | 124,787 | 118,076 | 6,711 | 4,096 | 2,615 | 55,369 | 44,814 | 1,063 | 9,492 |
| 50 - 54 | 141,558 | 95,316 | 90,856 | 4,460 | 2,428 | 2,032 | 46,242 | 31,637 | 862 | 13,743 |
| 55 - 59 | 94,791 | 62,119 | 59,317 | 2,802 | 1,445 | 1,357 | 32,672 | 18,676 | 636 | 13,360 |
| 60 - 64 | 88,456 | 54,997 | 52,985 | 2,012 | 953 | 1,059 | 33,459 | 14,148 | 627 | 18,684 |
| 65 - 69 | 64,887 | 37,945 | 36,692 | 1,253 | 520 | 733 | 26,942 | 8,911 | 475 | 17,556 |
| 70 - 74 | 48,873 | 23,330 | 22,584 | 746 | 303 | 443 | 25,543 | 5,233 | 602 | 19,708 |
| 75+ | 64,941 | 22,900 | 22,032 | 868 | 334 | 534 | 42,041 | 5,593 | 951 | 35,497 |

## Table E3: Population (De Facto) 12 Years and Older by Economic Activity Status, Age Group, Sex and Rural, Zambia 2010

| Age Group, Sex and Rural/Urban | Population 12 Years and Older | Economically Active | | | | | Economically Inactive | | | |
|---|---|---|---|---|---|---|---|---|---|---|
| | | Total Labour Force | Employed | Unemployed | | | Total | Full time Housewife/ homemaker | Full time student | Other |
| | | | | Total | Seeking work | Not seeking work but availabe for work | | | | |
| **Zambia Rural** | | | | | | | | | | |
| **Total** | **4,366,877** | **2,796,175** | **2,624,368** | **171,807** | **86,901** | **84,906** | **1,570,702** | **387,335** | **873,898** | **309,469** |
| 12 - 14 | 574,194 | 121,949 | 109,491 | 12,458 | 3,823 | 8,635 | 452,245 | 11,614 | 389,764 | 50,867 |
| 15 - 19 | 802,997 | 295,824 | 262,207 | 33,617 | 13,975 | 19,642 | 507,173 | 45,809 | 396,835 | 64,529 |
| 20 - 24 | 598,270 | 415,454 | 375,523 | 39,931 | 22,092 | 17,839 | 182,816 | 76,803 | 70,238 | 35,775 |
| 25 - 29 | 529,481 | 428,427 | 401,585 | 26,842 | 15,480 | 11,362 | 101,054 | 71,021 | 9,794 | 20,239 |
| 30 - 34 | 419,932 | 354,097 | 337,115 | 16,982 | 9,827 | 7,155 | 65,835 | 50,441 | 2,280 | 13,114 |
| 35 - 39 | 351,970 | 300,784 | 287,729 | 13,055 | 7,440 | 5,615 | 51,186 | 39,706 | 1,276 | 10,204 |
| 40 - 44 | 254,964 | 219,632 | 211,016 | 8,616 | 4,808 | 3,808 | 35,332 | 26,220 | 692 | 8,420 |
| 45 - 49 | 209,868 | 181,017 | 174,484 | 6,533 | 3,435 | 3,098 | 28,851 | 20,742 | 489 | 7,620 |
| 50 - 54 | 162,395 | 138,212 | 133,872 | 4,340 | 2,144 | 2,196 | 24,183 | 14,701 | 423 | 9,059 |
| 55 - 59 | 111,511 | 94,086 | 91,214 | 2,872 | 1,359 | 1,513 | 17,425 | 9,007 | 290 | 8,128 |
| 60 - 64 | 107,038 | 85,915 | 83,565 | 2,350 | 965 | 1,385 | 21,123 | 8,062 | 313 | 12,748 |
| 65 - 69 | 83,157 | 64,174 | 62,429 | 1,745 | 681 | 1,064 | 18,983 | 5,588 | 271 | 13,124 |
| 70 - 74 | 66,388 | 44,949 | 43,838 | 1,111 | 402 | 709 | 21,439 | 3,541 | 438 | 17,460 |
| 75+ | 94,712 | 51,655 | 50,300 | 1,355 | 470 | 885 | 43,057 | 4,080 | 795 | 38,182 |
| **Male** | | | | | | | | | | |
| **Total** | **2,093,514** | **1,444,639** | **1,341,036** | **103,603** | **56,104** | **47,499** | **648,875** | **27,026** | **481,520** | **140,329** |
| 12 - 14 | 291,542 | 62,717 | 56,266 | 6,451 | 1,983 | 4,468 | 228,825 | 4,828 | 197,958 | 26,039 |
| 15 - 19 | 397,738 | 132,572 | 115,324 | 17,248 | 7,442 | 9,806 | 265,166 | 7,021 | 226,608 | 31,537 |
| 20 - 24 | 269,368 | 197,707 | 173,886 | 23,821 | 13,852 | 9,969 | 71,661 | 4,443 | 48,956 | 18,262 |
| 25 - 29 | 241,136 | 222,750 | 205,566 | 17,184 | 10,495 | 6,689 | 18,386 | 2,727 | 5,683 | 9,976 |
| 30 - 34 | 203,507 | 194,280 | 183,042 | 11,238 | 6,913 | 4,325 | 9,227 | 1,818 | 841 | 6,568 |
| 35 - 39 | 173,650 | 166,488 | 157,697 | 8,791 | 5,375 | 3,416 | 7,162 | 1,452 | 434 | 5,276 |
| 40 - 44 | 127,044 | 121,666 | 115,773 | 5,893 | 3,493 | 2,400 | 5,378 | 988 | 226 | 4,164 |
| 45 - 49 | 100,903 | 96,517 | 92,222 | 4,295 | 2,415 | 1,880 | 4,386 | 795 | 114 | 3,477 |
| 50 - 54 | 75,868 | 71,647 | 68,891 | 2,756 | 1,487 | 1,269 | 4,221 | 620 | 85 | 3,516 |
| 55 - 59 | 52,225 | 48,571 | 46,714 | 1,857 | 939 | 918 | 3,654 | 471 | 57 | 3,126 |
| 60 - 64 | 45,761 | 41,238 | 39,769 | 1,469 | 648 | 821 | 4,523 | 440 | 59 | 4,024 |
| 65 - 69 | 36,611 | 32,241 | 31,186 | 1,055 | 464 | 591 | 4,370 | 381 | 54 | 3,935 |
| 70 - 74 | 30,553 | 24,527 | 23,857 | 670 | 262 | 408 | 6,026 | 353 | 144 | 5,529 |
| 75+ | 47,608 | 31,718 | 30,843 | 875 | 336 | 539 | 15,890 | 689 | 301 | 14,900 |
| **Female** | | | | | | | | | | |
| **Total** | **2,273,363** | **1,351,536** | **1,283,332** | **68,204** | **30,797** | **37,407** | **921,827** | **360,309** | **392,378** | **169,140** |
| 12 - 14 | 282,652 | 59,232 | 53,225 | 6,007 | 1,840 | 4,167 | 223,420 | 6,786 | 191,806 | 24,828 |
| 15 - 19 | 405,259 | 163,252 | 146,883 | 16,369 | 6,533 | 9,836 | 242,007 | 38,788 | 170,227 | 32,992 |
| 20 - 24 | 328,902 | 217,747 | 201,637 | 16,110 | 8,240 | 7,870 | 111,155 | 72,360 | 21,282 | 17,513 |
| 25 - 29 | 288,345 | 205,677 | 196,019 | 9,658 | 4,985 | 4,673 | 82,668 | 68,294 | 4,111 | 10,263 |
| 30 - 34 | 216,425 | 159,817 | 154,073 | 5,744 | 2,914 | 2,830 | 56,608 | 48,623 | 1,439 | 6,546 |
| 35 - 39 | 178,320 | 134,296 | 130,032 | 4,264 | 2,065 | 2,199 | 44,024 | 38,254 | 842 | 4,928 |
| 40 - 44 | 127,920 | 97,966 | 95,243 | 2,723 | 1,315 | 1,408 | 29,954 | 25,232 | 466 | 4,256 |
| 45 - 49 | 108,965 | 84,500 | 82,262 | 2,238 | 1,020 | 1,218 | 24,465 | 19,947 | 375 | 4,143 |
| 50 - 54 | 86,527 | 66,565 | 64,981 | 1,584 | 657 | 927 | 19,962 | 14,081 | 338 | 5,543 |
| 55 - 59 | 59,286 | 45,515 | 44,500 | 1,015 | 420 | 595 | 13,771 | 8,536 | 233 | 5,002 |
| 60 - 64 | 61,277 | 44,677 | 43,796 | 881 | 317 | 564 | 16,600 | 7,622 | 254 | 8,724 |
| 65 - 69 | 46,546 | 31,933 | 31,243 | 690 | 217 | 473 | 14,613 | 5,207 | 217 | 9,189 |
| 70 - 74 | 35,835 | 20,422 | 19,981 | 441 | 140 | 301 | 15,413 | 3,188 | 294 | 11,931 |
| 75+ | 47,104 | 19,937 | 19,457 | 480 | 134 | 346 | 27,167 | 3,391 | 494 | 23,282 |

## Table E3: Population (De Facto) 12 Years and Older by Economic Activity Status, Age Group, Sex and Urban, Zambia 2010

| Age Group, Sex and Rural/Urban | Population 12 Years and Older | Economically Active | | | | | Economically Inactive | | | |
|---|---|---|---|---|---|---|---|---|---|---|
| | | Total Labour Force | Employed | Unemployed | | | Total | Full time Housewife/ homemaker | Full time student | Other |
| | | | | Total | Seeking work | Not seeking work but availabe for work | | | | |
| **Zambia Urban** | | | | | | | | | | |
| **Total** | **3,348,145** | **1,634,367** | **1,262,684** | **371,683** | **283,158** | **88,525** | **1,713,778** | **493,171** | **919,002** | **301,605** |
| 12 - 14 | 384,779 | 15,886 | 9,063 | 6,823 | 3,283 | 3,540 | 368,893 | 7,458 | 330,779 | 30,656 |
| 15 - 19 | 624,887 | 86,972 | 35,725 | 51,247 | 33,563 | 17,684 | 537,915 | 41,024 | 436,240 | 60,651 |
| 20 - 24 | 519,206 | 250,656 | 138,211 | 112,445 | 86,966 | 25,479 | 268,550 | 106,651 | 112,676 | 49,223 |
| 25 - 29 | 476,763 | 319,681 | 242,370 | 77,311 | 62,873 | 14,438 | 157,082 | 108,160 | 23,917 | 25,005 |
| 30 - 34 | 380,838 | 284,800 | 240,104 | 44,696 | 36,376 | 8,320 | 96,038 | 74,926 | 6,536 | 14,576 |
| 35 - 39 | 295,843 | 229,656 | 200,147 | 29,509 | 23,950 | 5,559 | 66,187 | 52,481 | 3,126 | 10,580 |
| 40 - 44 | 192,620 | 150,392 | 133,290 | 17,102 | 13,554 | 3,548 | 42,228 | 32,015 | 1,513 | 8,700 |
| 45 - 49 | 145,121 | 109,672 | 97,641 | 12,031 | 9,308 | 2,723 | 35,449 | 25,484 | 940 | 9,025 |
| 50 - 54 | 107,859 | 75,806 | 68,191 | 7,615 | 5,300 | 2,315 | 32,053 | 18,144 | 682 | 13,227 |
| 55 - 59 | 73,317 | 46,472 | 41,036 | 5,436 | 3,512 | 1,924 | 26,845 | 10,690 | 558 | 15,597 |
| 60 - 64 | 54,837 | 29,620 | 26,049 | 3,571 | 2,176 | 1,395 | 25,217 | 7,055 | 512 | 17,650 |
| 65 - 69 | 35,425 | 17,024 | 15,118 | 1,906 | 1,127 | 779 | 18,401 | 4,068 | 394 | 13,939 |
| 70 - 74 | 24,414 | 8,984 | 8,011 | 973 | 583 | 390 | 15,430 | 2,355 | 423 | 12,652 |
| 75+ | 32,236 | 8,746 | 7,728 | 1,018 | 587 | 431 | 23,490 | 2,660 | 706 | 20,124 |
| **Male** | | | | | | | | | | |
| **Total** | **1,629,107** | **1,012,108** | **799,697** | **212,411** | **169,643** | **42,768** | **616,999** | **25,266** | **457,368** | **134,365** |
| 12 - 14 | 181,327 | 7,684 | 4,399 | 3,285 | 1,682 | 1,603 | 173,643 | 2,848 | 156,484 | 14,311 |
| 15 - 19 | 295,121 | 43,230 | 18,498 | 24,732 | 17,050 | 7,682 | 251,891 | 6,024 | 219,342 | 26,525 |
| 20 - 24 | 235,508 | 142,011 | 80,827 | 61,184 | 49,265 | 11,919 | 93,497 | 5,924 | 64,447 | 23,126 |
| 25 - 29 | 223,357 | 196,202 | 151,538 | 44,664 | 37,712 | 6,952 | 27,155 | 3,130 | 11,993 | 12,032 |
| 30 - 34 | 194,187 | 183,028 | 155,654 | 27,374 | 23,164 | 4,210 | 11,159 | 1,790 | 2,369 | 7,000 |
| 35 - 39 | 159,311 | 151,671 | 132,752 | 18,919 | 16,006 | 2,913 | 7,640 | 1,271 | 1,050 | 5,319 |
| 40 - 44 | 105,209 | 99,803 | 88,577 | 11,226 | 9,345 | 1,881 | 5,406 | 863 | 479 | 4,064 |
| 45 - 49 | 73,930 | 69,385 | 61,827 | 7,558 | 6,232 | 1,326 | 4,545 | 617 | 252 | 3,676 |
| 50 - 54 | 52,828 | 47,055 | 42,316 | 4,739 | 3,529 | 1,210 | 5,773 | 588 | 158 | 5,027 |
| 55 - 59 | 37,812 | 29,868 | 26,219 | 3,649 | 2,487 | 1,162 | 7,944 | 550 | 155 | 7,239 |
| 60 - 64 | 27,658 | 19,300 | 16,860 | 2,440 | 1,540 | 900 | 8,358 | 529 | 139 | 7,690 |
| 65 - 69 | 17,084 | 11,012 | 9,669 | 1,343 | 824 | 519 | 6,072 | 364 | 136 | 5,572 |
| 70 - 74 | 11,376 | 6,076 | 5,408 | 668 | 420 | 248 | 5,300 | 310 | 115 | 4,875 |
| 75+ | 14,399 | 5,783 | 5,153 | 630 | 387 | 243 | 8,616 | 458 | 249 | 7,909 |
| **Female** | | | | | | | | | | |
| **Total** | **1,719,038** | **622,259** | **462,987** | **159,272** | **113,515** | **45,757** | **1,096,779** | **467,905** | **461,634** | **167,240** |
| 12 - 14 | 203,452 | 8,202 | 4,664 | 3,538 | 1,601 | 1,937 | 195,250 | 4,610 | 174,295 | 16,345 |
| 15 - 19 | 329,766 | 43,742 | 17,227 | 26,515 | 16,513 | 10,002 | 286,024 | 35,000 | 216,898 | 34,126 |
| 20 - 24 | 283,698 | 108,645 | 57,384 | 51,261 | 37,701 | 13,560 | 175,053 | 100,727 | 48,229 | 26,097 |
| 25 - 29 | 253,406 | 123,479 | 90,832 | 32,647 | 25,161 | 7,486 | 129,927 | 105,030 | 11,924 | 12,973 |
| 30 - 34 | 186,651 | 101,772 | 84,450 | 17,322 | 13,212 | 4,110 | 84,879 | 73,136 | 4,167 | 7,576 |
| 35 - 39 | 136,532 | 77,985 | 67,395 | 10,590 | 7,944 | 2,646 | 58,547 | 51,210 | 2,076 | 5,261 |
| 40 - 44 | 87,411 | 50,589 | 44,713 | 5,876 | 4,209 | 1,667 | 36,822 | 31,152 | 1,034 | 4,636 |
| 45 - 49 | 71,191 | 40,287 | 35,814 | 4,473 | 3,076 | 1,397 | 30,904 | 24,867 | 688 | 5,349 |
| 50 - 54 | 55,031 | 28,751 | 25,875 | 2,876 | 1,771 | 1,105 | 26,280 | 17,556 | 524 | 8,200 |
| 55 - 59 | 35,505 | 16,604 | 14,817 | 1,787 | 1,025 | 762 | 18,901 | 10,140 | 403 | 8,358 |
| 60 - 64 | 27,179 | 10,320 | 9,189 | 1,131 | 636 | 495 | 16,859 | 6,526 | 373 | 9,960 |
| 65 - 69 | 18,341 | 6,012 | 5,449 | 563 | 303 | 260 | 12,329 | 3,704 | 258 | 8,367 |
| 70 - 74 | 13,038 | 2,908 | 2,603 | 305 | 163 | 142 | 10,130 | 2,045 | 308 | 7,777 |
| 75+ | 17,837 | 2,963 | 2,575 | 388 | 200 | 188 | 14,874 | 2,202 | 457 | 12,215 |

## Table E4: Usually Working Population (De Facto) 12 Years and Older by Industry of Employment, Age Group, Sex and Rural/Urban, Zambia 2010

| Age Group, Sex and Rural/ Urban | Population 12 Years and Older | Usual Working Population | Industry | | | | | | | | | | | | | |
|---|---|---|---|---|---|---|---|---|---|---|---|---|---|---|---|---|
| | | | Agri-culture Hunting Forestry and Fish-ing | Mining and Quarrying | Manufac-turing | Electricity Gas Steam and Air condition-ing supply | Water Supply | Construc-tion and Allied Repairs | Wholesale & Retail Trade Res-taurants and Hotel | Transport and Storage | Acco-modation and food services activities | Informa-tion and Communi-cation | Finance and Insurance | Real Estate Activies | Com-munity Social and Personal Services | Not Stated |
| **Zambia Total** | | | | | | | | | | | | | | | | |
| **Total** | **7,715,022** | **3,887,052** | **2,440,748** | **69,922** | **132,292** | **8,578** | **6,729** | **110,991** | **361,660** | **91,113** | **39,220** | **23,337** | **17,228** | **1,307** | **366,193** | **217,734** |
| 12 - 14 | 958,973 | 118,554 | 89,777 | 120 | 219 | - | 4 | 98 | 1,302 | 48 | 56 | 304 | 4 | - | 960 | 25,662 |
| 15 - 19 | 1,427,884 | 297,932 | 239,699 | 956 | 2,527 | 38 | 69 | 1,929 | 10,422 | 1,187 | 932 | 949 | 81 | 24 | 6,208 | 32,911 |
| 20 - 24 | 1,117,476 | 513,734 | 348,739 | 6,684 | 13,239 | 671 | 598 | 10,811 | 46,279 | 9,565 | 5,897 | 3,223 | 1,390 | 96 | 30,685 | 35,857 |
| 25 - 29 | 1,006,244 | 643,955 | 365,732 | 14,689 | 24,503 | 1,759 | 1,197 | 19,854 | 74,297 | 20,320 | 9,285 | 5,037 | 4,080 | 210 | 70,989 | 32,003 |
| 30 - 34 | 800,770 | 577,219 | 305,857 | 14,009 | 25,029 | 1,900 | 1,173 | 21,356 | 71,922 | 21,190 | 7,767 | 4,183 | 3,712 | 227 | 74,731 | 24,163 |
| 35 - 39 | 647,813 | 487,876 | 264,886 | 10,427 | 22,375 | 1,504 | 1,082 | 19,754 | 58,907 | 16,408 | 6,002 | 3,028 | 2,883 | 206 | 61,329 | 19,085 |
| 40 - 44 | 447,584 | 344,306 | 196,935 | 7,949 | 15,420 | 1,024 | 843 | 13,450 | 36,072 | 9,369 | 3,667 | 2,244 | 2,189 | 168 | 41,958 | 13,018 |
| 45 - 49 | 354,989 | 272,125 | 165,952 | 6,430 | 10,915 | 750 | 698 | 9,469 | 25,072 | 5,579 | 2,439 | 1,625 | 1,328 | 138 | 31,701 | 10,029 |
| 50 - 54 | 270,254 | 202,063 | 129,373 | 4,461 | 7,461 | 543 | 524 | 5,729 | 15,874 | 3,372 | 1,479 | 1,110 | 843 | 79 | 23,683 | 7,532 |
| 55 - 59 | 184,828 | 132,250 | 91,792 | 2,367 | 4,435 | 226 | 273 | 3,376 | 9,409 | 1,965 | 774 | 606 | 422 | 63 | 11,658 | 4,884 |
| 60 - 64 | 161,875 | 109,614 | 85,096 | 1,052 | 2,701 | 95 | 142 | 2,310 | 6,026 | 1,142 | 465 | 409 | 193 | 50 | 5,975 | 3,958 |
| 65 - 69 | 118,582 | 77,547 | 63,454 | 463 | 1,676 | 40 | 75 | 1,395 | 3,253 | 556 | 258 | 271 | 61 | 19 | 3,250 | 2,776 |
| 70 - 74 | 90,802 | 51,849 | 44,179 | 179 | 887 | 18 | 27 | 774 | 1,460 | 231 | 104 | 153 | 20 | 12 | 1,624 | 2,181 |
| 75+ | 126,948 | 58,028 | 49,277 | 136 | 905 | 10 | 24 | 686 | 1,365 | 181 | 95 | 195 | 22 | 15 | 1,442 | 3,675 |
| **Male** | | | | | | | | | | | | | | | | |
| **Total** | **3,722,621** | **2,140,733** | **1,227,232** | **63,840** | **103,436** | **7,563** | **5,407** | **107,979** | **171,651** | **87,203** | **18,146** | **14,287** | **10,875** | **898** | **213,519** | **108,697** |
| 12 - 14 | 472,869 | 60,665 | 46,590 | 54 | 137 | - | 2 | 79 | 576 | 38 | 17 | 161 | 4 | - | 376 | 12,631 |
| 15 - 19 | 692,859 | 133,822 | 104,451 | 704 | 1,676 | 34 | 45 | 1,825 | 4,591 | 1,083 | 356 | 420 | 34 | 12 | 2,309 | 16,282 |
| 20 - 24 | 504,876 | 254,713 | 159,500 | 5,823 | 9,960 | 563 | 427 | 10,411 | 21,902 | 9,027 | 2,351 | 1,701 | 621 | 58 | 14,454 | 17,915 |
| 25 - 29 | 464,493 | 357,104 | 184,808 | 13,256 | 19,191 | 1,504 | 936 | 19,231 | 37,671 | 19,450 | 4,088 | 3,020 | 2,288 | 126 | 36,086 | 15,449 |
| 30 - 34 | 397,694 | 338,696 | 162,822 | 12,999 | 19,913 | 1,644 | 932 | 20,826 | 36,398 | 20,426 | 3,613 | 2,609 | 2,346 | 151 | 41,821 | 12,196 |
| 35 - 39 | 332,961 | 290,449 | 141,800 | 9,664 | 17,572 | 1,333 | 893 | 19,340 | 29,519 | 15,851 | 2,917 | 2,014 | 1,918 | 160 | 37,455 | 10,013 |
| 40 - 44 | 232,253 | 204,350 | 105,305 | 7,411 | 12,092 | 947 | 704 | 13,176 | 16,830 | 8,956 | 1,832 | 1,550 | 1,527 | 131 | 27,010 | 6,879 |
| 45 - 49 | 174,833 | 154,049 | 85,004 | 6,013 | 8,464 | 675 | 582 | 9,200 | 9,918 | 5,275 | 1,254 | 1,067 | 931 | 95 | 20,453 | 5,118 |
| 50 - 54 | 128,696 | 111,207 | 64,113 | 4,140 | 5,878 | 489 | 434 | 5,577 | 5,708 | 3,180 | 734 | 713 | 614 | 51 | 15,681 | 3,895 |
| 55 - 59 | 90,037 | 72,933 | 46,147 | 2,217 | 3,582 | 218 | 226 | 3,275 | 3,626 | 1,885 | 423 | 410 | 331 | 44 | 8,082 | 2,467 |
| 60 - 64 | 73,419 | 56,629 | 40,402 | 944 | 2,162 | 91 | 114 | 2,263 | 2,417 | 1,106 | 275 | 232 | 176 | 40 | 4,523 | 1,884 |
| 65 - 69 | 53,695 | 40,855 | 31,587 | 380 | 1,337 | 39 | 67 | 1,352 | 1,253 | 538 | 154 | 167 | 54 | 13 | 2,656 | 1,258 |
| 70 - 74 | 41,929 | 29,265 | 24,163 | 135 | 723 | 18 | 23 | 753 | 630 | 217 | 73 | 85 | 14 | 6 | 1,398 | 1,027 |
| 75+ | 62,007 | 35,996 | 30,540 | 100 | 749 | 8 | 22 | 671 | 612 | 171 | 59 | 138 | 17 | 11 | 1,215 | 1,683 |
| **Female** | | | | | | | | | | | | | | | | |
| **Total** | **3,992,401** | **1,746,319** | **1,213,516** | **6,082** | **28,856** | **1,015** | **1,322** | **3,012** | **190,009** | **3,910** | **21,074** | **9,050** | **6,353** | **409** | **152,674** | **109,037** |
| 12 - 14 | 486,104 | 57,889 | 43,187 | 66 | 82 | - | 2 | 19 | 726 | 10 | 39 | 143 | - | - | 584 | 13,031 |
| 15 - 19 | 735,025 | 164,110 | 135,248 | 252 | 851 | 4 | 24 | 104 | 5,831 | 104 | 576 | 529 | 47 | 12 | 3,899 | 16,629 |
| 20 - 24 | 612,600 | 259,021 | 189,239 | 861 | 3,279 | 108 | 171 | 400 | 24,377 | 538 | 3,546 | 1,522 | 769 | 38 | 16,231 | 17,942 |
| 25 - 29 | 541,751 | 286,851 | 180,924 | 1,433 | 5,312 | 255 | 261 | 623 | 36,626 | 870 | 5,197 | 2,017 | 1,792 | 84 | 34,903 | 16,554 |
| 30 - 34 | 403,076 | 238,523 | 143,035 | 1,010 | 5,116 | 256 | 241 | 530 | 35,524 | 764 | 4,154 | 1,574 | 1,366 | 76 | 32,910 | 11,967 |
| 35 - 39 | 314,852 | 197,427 | 123,086 | 763 | 4,803 | 171 | 189 | 414 | 29,388 | 557 | 3,085 | 1,014 | 965 | 46 | 23,874 | 9,072 |
| 40 - 44 | 215,331 | 139,956 | 91,630 | 538 | 3,328 | 77 | 139 | 274 | 19,242 | 413 | 1,835 | 694 | 662 | 37 | 14,948 | 6,139 |
| 45 - 49 | 180,156 | 118,076 | 80,948 | 417 | 2,451 | 75 | 116 | 269 | 15,154 | 304 | 1,185 | 558 | 397 | 43 | 11,248 | 4,911 |
| 50 - 54 | 141,558 | 90,856 | 65,260 | 321 | 1,583 | 54 | 90 | 152 | 10,166 | 192 | 745 | 397 | 229 | 28 | 8,002 | 3,637 |
| 55 - 59 | 94,791 | 59,317 | 45,645 | 150 | 853 | 8 | 47 | 101 | 5,783 | 80 | 351 | 196 | 91 | 19 | 3,576 | 2,417 |
| 60 - 64 | 88,456 | 52,985 | 44,694 | 108 | 539 | 4 | 28 | 47 | 3,609 | 36 | 190 | 177 | 17 | 10 | 1,452 | 2,074 |
| 65 - 69 | 64,887 | 36,692 | 31,867 | 83 | 339 | 1 | 8 | 43 | 2,000 | 18 | 104 | 104 | 7 | 6 | 594 | 1,518 |
| 70 - 74 | 48,873 | 22,584 | 20,016 | 44 | 164 | - | 4 | 21 | 830 | 14 | 31 | 68 | 6 | 6 | 226 | 1,154 |
| 75+ | 64,941 | 22,032 | 18,737 | 36 | 156 | 2 | 2 | 15 | 753 | 10 | 36 | 57 | 5 | 4 | 227 | 1,992 |

## Table E4: Usually Working Population (De Facto) 12 Years and Older by Industry of Employment, Age Group, Sex and Rural, Zambia 2010

| Age Group, Sex and Rural/ Urban | Population 12 Years and Older | Usual Working Population | Industry | | | | | | | | | | | | | |
|---|---|---|---|---|---|---|---|---|---|---|---|---|---|---|---|---|
| | | | Agri-culture Hunting Forestry and Fish-ing | Mining and Quarrying | Manufac-turing | Electricity Gas Steam and Air condition-ing supply | Water Supply | Construc-tion and Allied Repairs | Wholesale & Retail Trade Res-taurants and Hotel | Transport and Storage | Acco-modation and food services activities | Informa-tion and Communi-cation | Finance and Insurance | Real Estate Activies | Com-munity Social and Personal Services | Not Stated |
| **Zambia Rural** | | | | | | | | | | | | | | | | |
| **Total** | **4,366,877** | **2,624,368** | **2,276,211** | **9,173** | **27,930** | **869** | **505** | **26,002** | **54,243** | **8,251** | **5,069** | **8,270** | **813** | **251** | **76,707** | **130,074** |
| 12 - 14 | 574,194 | 109,491 | 86,431 | 83 | 155 | - | 2 | 48 | 601 | 26 | 25 | 296 | - | - | 528 | 21,296 |
| 15 - 19 | 802,997 | 262,207 | 229,726 | 459 | 928 | 6 | 9 | 552 | 3,093 | 152 | 212 | 722 | 7 | 11 | 1,483 | 24,847 |
| 20 - 24 | 598,270 | 375,523 | 330,892 | 1,316 | 2,900 | 76 | 47 | 2,618 | 8,642 | 912 | 723 | 1,284 | 79 | 36 | 5,407 | 20,591 |
| 25 - 29 | 529,481 | 401,585 | 343,814 | 1,932 | 4,608 | 166 | 72 | 4,376 | 11,112 | 1,704 | 1,035 | 1,334 | 153 | 51 | 15,239 | 15,989 |
| 30 - 34 | 419,932 | 337,115 | 284,713 | 1,536 | 4,603 | 176 | 72 | 4,581 | 9,808 | 1,724 | 964 | 1,061 | 155 | 41 | 16,398 | 11,283 |
| 35 - 39 | 351,970 | 287,729 | 245,660 | 1,212 | 4,218 | 152 | 73 | 4,377 | 7,775 | 1,402 | 761 | 881 | 123 | 36 | 11,838 | 9,221 |
| 40 - 44 | 254,964 | 211,016 | 181,966 | 840 | 3,202 | 115 | 74 | 3,054 | 4,715 | 891 | 475 | 715 | 99 | 26 | 8,255 | 6,589 |
| 45 - 49 | 209,868 | 174,484 | 152,362 | 609 | 2,395 | 65 | 60 | 2,422 | 3,250 | 560 | 378 | 538 | 76 | 25 | 6,569 | 5,175 |
| 50 - 54 | 162,395 | 133,872 | 117,418 | 433 | 1,748 | 71 | 40 | 1,502 | 2,053 | 379 | 224 | 438 | 65 | 10 | 5,503 | 3,988 |
| 55 - 59 | 111,511 | 91,214 | 82,136 | 274 | 1,037 | 21 | 23 | 851 | 1,182 | 223 | 122 | 274 | 27 | 9 | 2,396 | 2,639 |
| 60 - 64 | 107,038 | 83,565 | 76,761 | 200 | 754 | 13 | 14 | 614 | 906 | 137 | 69 | 255 | 16 | 2 | 1,307 | 2,517 |
| 65 - 69 | 83,157 | 62,429 | 57,716 | 136 | 597 | 5 | 8 | 461 | 544 | 75 | 49 | 192 | 8 | 3 | 797 | 1,838 |
| 70 - 74 | 66,388 | 43,838 | 40,697 | 75 | 347 | 1 | 5 | 283 | 271 | 40 | 20 | 119 | 1 | 1 | 465 | 1,513 |
| 75+ | 94,712 | 50,300 | 45,919 | 68 | 438 | 2 | 6 | 263 | 291 | 26 | 12 | 161 | 4 | - | 522 | 2,588 |
| **Male** | | | | | | | | | | | | | | | | |
| **Total** | **2,093,514** | **1,341,036** | **1,136,245** | **7,128** | **19,357** | **789** | **433** | **25,281** | **22,344** | **7,735** | **2,520** | **4,457** | **548** | **192** | **50,856** | **63,151** |
| 12 - 14 | 291,542 | 56,266 | 44,896 | 34 | 87 | - | 1 | 37 | 229 | 19 | 7 | 158 | - | - | 234 | 10,564 |
| 15 - 19 | 397,738 | 115,324 | 99,660 | 287 | 467 | 5 | 6 | 501 | 981 | 113 | 70 | 304 | 4 | 8 | 569 | 12,349 |
| 20 - 24 | 269,368 | 173,886 | 150,369 | 1,002 | 1,866 | 65 | 36 | 2,523 | 3,307 | 831 | 314 | 640 | 44 | 30 | 2,821 | 10,038 |
| 25 - 29 | 241,136 | 205,566 | 172,635 | 1,548 | 3,164 | 144 | 63 | 4,247 | 4,752 | 1,618 | 524 | 740 | 101 | 42 | 8,673 | 7,315 |
| 30 - 34 | 203,507 | 183,042 | 150,323 | 1,265 | 3,310 | 162 | 61 | 4,487 | 4,321 | 1,635 | 508 | 592 | 109 | 29 | 10,901 | 5,339 |
| 35 - 39 | 173,650 | 157,697 | 130,327 | 990 | 3,019 | 139 | 63 | 4,266 | 3,480 | 1,334 | 429 | 489 | 78 | 29 | 8,472 | 4,582 |
| 40 - 44 | 127,044 | 115,773 | 96,638 | 674 | 2,268 | 106 | 62 | 2,990 | 2,004 | 841 | 230 | 432 | 68 | 21 | 6,092 | 3,347 |
| 45 - 49 | 100,903 | 92,222 | 77,888 | 470 | 1,666 | 61 | 56 | 2,348 | 1,303 | 526 | 201 | 303 | 53 | 16 | 4,811 | 2,520 |
| 50 - 54 | 75,868 | 68,891 | 58,235 | 333 | 1,243 | 67 | 33 | 1,472 | 729 | 351 | 87 | 238 | 47 | 6 | 4,087 | 1,963 |
| 55 - 59 | 52,225 | 46,714 | 40,957 | 207 | 742 | 21 | 20 | 836 | 448 | 211 | 61 | 165 | 19 | 6 | 1,747 | 1,274 |
| 60 - 64 | 45,761 | 39,769 | 35,746 | 135 | 509 | 12 | 14 | 600 | 325 | 130 | 39 | 121 | 15 | 2 | 994 | 1,127 |
| 65 - 69 | 36,611 | 31,186 | 28,349 | 85 | 417 | 5 | 7 | 444 | 206 | 69 | 30 | 99 | 7 | 2 | 645 | 821 |
| 70 - 74 | 30,553 | 23,857 | 21,958 | 50 | 261 | 1 | 5 | 273 | 124 | 34 | 11 | 62 | - | 1 | 377 | 700 |
| 75+ | 47,608 | 30,843 | 28,264 | 48 | 338 | 1 | 6 | 257 | 135 | 23 | 9 | 114 | 3 | - | 433 | 1,212 |
| **Female** | | | | | | | | | | | | | | | | |
| **Total** | **2,273,363** | **1,283,332** | **1,139,966** | **2,045** | **8,573** | **80** | **72** | **721** | **31,899** | **516** | **2,549** | **3,813** | **265** | **59** | **25,851** | **66,923** |
| 12 - 14 | 282,652 | 53,225 | 41,535 | 49 | 68 | - | 1 | 11 | 372 | 7 | 18 | 138 | - | - | 294 | 10,732 |
| 15 - 19 | 405,259 | 146,883 | 130,066 | 172 | 461 | 1 | 3 | 51 | 2,112 | 39 | 142 | 418 | 3 | 3 | 914 | 12,498 |
| 20 - 24 | 328,902 | 201,637 | 180,523 | 314 | 1,034 | 11 | 11 | 95 | 5,335 | 81 | 409 | 644 | 35 | 6 | 2,586 | 10,553 |
| 25 - 29 | 288,345 | 196,019 | 171,179 | 384 | 1,444 | 22 | 9 | 129 | 6,360 | 86 | 511 | 594 | 52 | 9 | 6,566 | 8,674 |
| 30 - 34 | 216,425 | 154,073 | 134,390 | 271 | 1,293 | 14 | 11 | 94 | 5,487 | 89 | 456 | 469 | 46 | 12 | 5,497 | 5,944 |
| 35 - 39 | 178,320 | 130,032 | 115,333 | 222 | 1,199 | 13 | 10 | 111 | 4,295 | 68 | 332 | 392 | 45 | 7 | 3,366 | 4,639 |
| 40 - 44 | 127,920 | 95,243 | 85,328 | 166 | 934 | 9 | 12 | 64 | 2,711 | 50 | 245 | 283 | 31 | 5 | 2,163 | 3,242 |
| 45 - 49 | 108,965 | 82,262 | 74,474 | 139 | 729 | 4 | 4 | 74 | 1,947 | 34 | 177 | 235 | 23 | 9 | 1,758 | 2,655 |
| 50 - 54 | 86,527 | 64,981 | 59,183 | 100 | 505 | 4 | 7 | 30 | 1,324 | 28 | 137 | 200 | 18 | 4 | 1,416 | 2,025 |
| 55 - 59 | 59,286 | 44,500 | 41,179 | 67 | 295 | - | 3 | 15 | 734 | 12 | 61 | 109 | 8 | 3 | 649 | 1,365 |
| 60 - 64 | 61,277 | 43,796 | 41,015 | 65 | 245 | 1 | - | 14 | 581 | 7 | 30 | 134 | 1 | - | 313 | 1,390 |
| 65 - 69 | 46,546 | 31,243 | 29,367 | 51 | 180 | - | 1 | 17 | 338 | 6 | 19 | 93 | 1 | 1 | 152 | 1,017 |
| 70 - 74 | 35,835 | 19,981 | 18,739 | 25 | 86 | - | - | 10 | 147 | 6 | 9 | 57 | 1 | - | 88 | 813 |
| 75+ | 47,104 | 19,457 | 17,655 | 20 | 100 | 1 | - | 6 | 156 | 3 | 3 | 47 | 1 | - | 89 | 1,376 |

## Table E4: Usually Working Population (De Facto) 12 Years and Older by Industry of Employment, Age Group, Sex and Urban, Zambia 2010

| Age Group, Sex and Rural/ Urban | Population 12 Years and Older | Usual Working Population | Industry | | | | | | | | | | | | | |
|---|---|---|---|---|---|---|---|---|---|---|---|---|---|---|---|---|
| | | | Agri-culture Hunting Forestry and Fish-ing | Mining and Quarrying | Manufac-turing | Electricity Gas Steam and Air condition-ing supply | Water Supply | Construc-tion and Allied Repairs | Wholesale & Retail Trade Res-taurants and Hotel | Transport and Storage | Acco-modation and food services activities | Informa-tion and Communi-cation | Finance and Insurance | Real Estate Activies | Com-munity Social and Personal Services | Not Stated |
| **Zambia Urban** | | | | | | | | | | | | | | | | |
| **Total** | **3,348,145** | **1,262,684** | **164,537** | **60,749** | **104,362** | **7,709** | **6,224** | **84,989** | **307,417** | **82,862** | **34,151** | **15,067** | **16,415** | **1,056** | **289,486** | **87,660** |
| 12 - 14 | 384,779 | 9,063 | 3,346 | 37 | 64 | - | 2 | 50 | 701 | 22 | 31 | 8 | 4 | - | 432 | 4,366 |
| 15 - 19 | 624,887 | 35,725 | 9,973 | 497 | 1,599 | 32 | 60 | 1,377 | 7,329 | 1,035 | 720 | 227 | 74 | 13 | 4,725 | 8,064 |
| 20 - 24 | 519,206 | 138,211 | 17,847 | 5,368 | 10,339 | 595 | 551 | 8,193 | 37,637 | 8,653 | 5,174 | 1,939 | 1,311 | 60 | 25,278 | 15,266 |
| 25 - 29 | 476,763 | 242,370 | 21,918 | 12,757 | 19,895 | 1,593 | 1,125 | 15,478 | 63,185 | 18,616 | 8,250 | 3,703 | 3,927 | 159 | 55,750 | 16,014 |
| 30 - 34 | 380,838 | 240,104 | 21,144 | 12,473 | 20,426 | 1,724 | 1,101 | 16,775 | 62,114 | 19,466 | 6,803 | 3,122 | 3,557 | 186 | 58,333 | 12,880 |
| 35 - 39 | 295,843 | 200,147 | 19,226 | 9,215 | 18,157 | 1,352 | 1,009 | 15,377 | 51,132 | 15,006 | 5,241 | 2,147 | 2,760 | 170 | 49,491 | 9,864 |
| 40 - 44 | 192,620 | 133,290 | 14,969 | 7,109 | 12,218 | 909 | 769 | 10,396 | 31,357 | 8,478 | 3,192 | 1,529 | 2,090 | 142 | 33,703 | 6,429 |
| 45 - 49 | 145,121 | 97,641 | 13,590 | 5,821 | 8,520 | 685 | 638 | 7,047 | 21,822 | 5,019 | 2,061 | 1,087 | 1,252 | 113 | 25,132 | 4,854 |
| 50 - 54 | 107,859 | 68,191 | 11,955 | 4,028 | 5,713 | 472 | 484 | 4,227 | 13,821 | 2,993 | 1,255 | 672 | 778 | 69 | 18,180 | 3,544 |
| 55 - 59 | 73,317 | 41,036 | 9,656 | 2,093 | 3,398 | 205 | 250 | 2,525 | 8,227 | 1,742 | 652 | 332 | 395 | 54 | 9,262 | 2,245 |
| 60 - 64 | 54,837 | 26,049 | 8,335 | 852 | 1,947 | 82 | 128 | 1,696 | 5,120 | 1,005 | 396 | 154 | 177 | 48 | 4,668 | 1,441 |
| 65 - 69 | 35,425 | 15,118 | 5,738 | 327 | 1,079 | 35 | 67 | 934 | 2,709 | 481 | 209 | 79 | 53 | 16 | 2,453 | 938 |
| 70 - 74 | 24,414 | 8,011 | 3,482 | 104 | 540 | 17 | 22 | 491 | 1,189 | 191 | 84 | 34 | 19 | 11 | 1,159 | 668 |
| 75+ | 32,236 | 7,728 | 3,358 | 68 | 467 | 8 | 18 | 423 | 1,074 | 155 | 83 | 34 | 18 | 15 | 920 | 1,087 |
| **Male** | | | | | | | | | | | | | | | | |
| **Total** | **1,629,107** | **799,697** | **90,987** | **56,712** | **84,079** | **6,774** | **4,974** | **82,698** | **149,307** | **79,468** | **15,626** | **9,830** | **10,327** | **706** | **162,663** | **45,546** |
| 12 - 14 | 181,327 | 4,399 | 1,694 | 20 | 50 | - | 1 | 42 | 347 | 19 | 10 | 3 | 4 | - | 142 | 2,067 |
| 15 - 19 | 295,121 | 18,498 | 4,791 | 417 | 1,209 | 29 | 39 | 1,324 | 3,610 | 970 | 286 | 116 | 30 | 4 | 1,740 | 3,933 |
| 20 - 24 | 235,508 | 80,827 | 9,131 | 4,821 | 8,094 | 498 | 391 | 7,888 | 18,595 | 8,196 | 2,037 | 1,061 | 577 | 28 | 11,633 | 7,877 |
| 25 - 29 | 223,357 | 151,538 | 12,173 | 11,708 | 16,027 | 1,360 | 873 | 14,984 | 32,919 | 17,832 | 3,564 | 2,280 | 2,187 | 84 | 27,413 | 8,134 |
| 30 - 34 | 194,187 | 155,654 | 12,499 | 11,734 | 16,603 | 1,482 | 871 | 16,339 | 32,077 | 18,791 | 3,105 | 2,017 | 2,237 | 122 | 30,920 | 6,857 |
| 35 - 39 | 159,311 | 132,752 | 11,473 | 8,674 | 14,553 | 1,194 | 830 | 15,074 | 26,039 | 14,517 | 2,488 | 1,525 | 1,840 | 131 | 28,983 | 5,431 |
| 40 - 44 | 105,209 | 88,577 | 8,667 | 6,737 | 9,824 | 841 | 642 | 10,186 | 14,826 | 8,115 | 1,602 | 1,118 | 1,459 | 110 | 20,918 | 3,532 |
| 45 - 49 | 73,930 | 61,827 | 7,116 | 5,543 | 6,798 | 614 | 526 | 6,852 | 8,615 | 4,749 | 1,053 | 764 | 878 | 79 | 15,642 | 2,598 |
| 50 - 54 | 52,828 | 42,316 | 5,878 | 3,807 | 4,635 | 422 | 401 | 4,105 | 4,979 | 2,829 | 647 | 475 | 567 | 45 | 11,594 | 1,932 |
| 55 - 59 | 37,812 | 26,219 | 5,190 | 2,010 | 2,840 | 197 | 206 | 2,439 | 3,178 | 1,674 | 362 | 245 | 312 | 38 | 6,335 | 1,193 |
| 60 - 64 | 27,658 | 16,860 | 4,656 | 809 | 1,653 | 79 | 100 | 1,663 | 2,092 | 976 | 236 | 111 | 161 | 38 | 3,529 | 757 |
| 65 - 69 | 17,084 | 9,669 | 3,238 | 295 | 920 | 34 | 60 | 908 | 1,047 | 469 | 124 | 68 | 47 | 11 | 2,011 | 437 |
| 70 - 74 | 11,376 | 5,408 | 2,205 | 85 | 462 | 17 | 18 | 480 | 506 | 183 | 62 | 23 | 14 | 5 | 1,021 | 327 |
| 75+ | 14,399 | 5,153 | 2,276 | 52 | 411 | 7 | 16 | 414 | 477 | 148 | 50 | 24 | 14 | 11 | 782 | 471 |
| **Female** | | | | | | | | | | | | | | | | |
| **Total** | **1,719,038** | **462,987** | **73,550** | **4,037** | **20,283** | **935** | **1,250** | **2,291** | **158,110** | **3,394** | **18,525** | **5,237** | **6,088** | **350** | **126,823** | **42,114** |
| 12 - 14 | 203,452 | 4,664 | 1,652 | 17 | 14 | - | 1 | 8 | 354 | 3 | 21 | 5 | - | - | 290 | 2,299 |
| 15 - 19 | 329,766 | 17,227 | 5,182 | 80 | 390 | 3 | 21 | 53 | 3,719 | 65 | 434 | 111 | 44 | 9 | 2,985 | 4,131 |
| 20 - 24 | 283,698 | 57,384 | 8,716 | 547 | 2,245 | 97 | 160 | 305 | 19,042 | 457 | 3,137 | 878 | 734 | 32 | 13,645 | 7,389 |
| 25 - 29 | 253,406 | 90,832 | 9,745 | 1,049 | 3,868 | 233 | 252 | 494 | 30,266 | 784 | 4,686 | 1,423 | 1,740 | 75 | 28,337 | 7,880 |
| 30 - 34 | 186,651 | 84,450 | 8,645 | 739 | 3,823 | 242 | 230 | 436 | 30,037 | 675 | 3,698 | 1,105 | 1,320 | 64 | 27,413 | 6,023 |
| 35 - 39 | 136,532 | 67,395 | 7,753 | 541 | 3,604 | 158 | 179 | 303 | 25,093 | 489 | 2,753 | 622 | 920 | 39 | 20,508 | 4,433 |
| 40 - 44 | 87,411 | 44,713 | 6,302 | 372 | 2,394 | 68 | 127 | 210 | 16,531 | 363 | 1,590 | 411 | 631 | 32 | 12,785 | 2,897 |
| 45 - 49 | 71,191 | 35,814 | 6,474 | 278 | 1,722 | 71 | 112 | 195 | 13,207 | 270 | 1,008 | 323 | 374 | 34 | 9,490 | 2,256 |
| 50 - 54 | 55,031 | 25,875 | 6,077 | 221 | 1,078 | 50 | 83 | 122 | 8,842 | 164 | 608 | 197 | 211 | 24 | 6,586 | 1,612 |
| 55 - 59 | 35,505 | 14,817 | 4,466 | 83 | 558 | 8 | 44 | 86 | 5,049 | 68 | 290 | 87 | 83 | 16 | 2,927 | 1,052 |
| 60 - 64 | 27,179 | 9,189 | 3,679 | 43 | 294 | 3 | 28 | 33 | 3,028 | 29 | 160 | 43 | 16 | 10 | 1,139 | 684 |
| 65 - 69 | 18,341 | 5,449 | 2,500 | 32 | 159 | 1 | 7 | 26 | 1,662 | 12 | 85 | 11 | 6 | 5 | 442 | 501 |
| 70 - 74 | 13,038 | 2,603 | 1,277 | 19 | 78 | - | 4 | 11 | 683 | 8 | 22 | 11 | 5 | 6 | 138 | 341 |
| 75+ | 17,837 | 2,575 | 1,082 | 16 | 56 | 1 | 2 | 9 | 597 | 7 | 33 | 10 | 4 | 4 | 138 | 616 |

## Table E5: Population (De Facto) 12 Years and Older by Age Group, Sex and Occupation Category, Rural/Urban, Zambia 2010

| Age Group, Sex and Rural/ Urban | Population 12 Years and Older | Usual Working Population | Occupation | | | | | | | | | |
|---|---|---|---|---|---|---|---|---|---|---|---|---|
| | | | Managers | Professionals | Technicians and Associate Professionals | Clerical Support Workers | Service and Sales Workers | Skilled Agricultural Forestry and Fishery Workers | Craft and Related Trades Workers | Plant and Machine Operators and Assemblers | Elementary Occupations | Not Stated |
| **Zambia Total** | | | | | | | | | | | | |
| **Total** | **7,715,022** | **3,887,052** | **35,898** | **177,334** | **84,637** | **37,323** | **388,270** | **2,092,815** | **267,053** | **123,689** | **497,112** | **182,921** |
| 12 - 14 | 958,973 | 118,554 | 107 | 32 | 92 | 31 | 1,204 | 78,578 | 646 | 154 | 13,469 | 24,241 |
| 15 - 19 | 1,427,884 | 297,932 | 465 | 606 | 908 | 520 | 11,135 | 204,301 | 5,689 | 1,555 | 43,602 | 29,151 |
| 20 - 24 | 1,117,476 | 513,734 | 1,933 | 10,382 | 7,004 | 4,950 | 50,952 | 294,524 | 26,685 | 11,970 | 76,019 | 29,315 |
| 25 - 29 | 1,006,244 | 643,955 | 4,766 | 37,229 | 16,831 | 8,800 | 80,415 | 309,713 | 48,375 | 26,095 | 86,215 | 25,516 |
| 30 - 34 | 800,770 | 577,219 | 6,037 | 39,746 | 17,210 | 7,142 | 76,169 | 260,297 | 50,665 | 27,781 | 72,623 | 19,549 |
| 35 - 39 | 647,813 | 487,876 | 5,926 | 30,280 | 13,917 | 5,598 | 62,382 | 225,881 | 45,168 | 22,196 | 60,527 | 16,001 |
| 40 - 44 | 447,584 | 344,306 | 5,040 | 20,744 | 9,800 | 3,891 | 38,828 | 168,975 | 31,795 | 13,226 | 41,127 | 10,880 |
| 45 - 49 | 354,989 | 272,125 | 4,001 | 15,553 | 7,374 | 2,870 | 27,181 | 142,960 | 22,831 | 8,720 | 32,469 | 8,166 |
| 50 - 54 | 270,254 | 202,063 | 3,077 | 12,145 | 5,517 | 2,086 | 17,532 | 112,488 | 14,619 | 5,445 | 23,080 | 6,074 |
| 55 - 59 | 184,828 | 132,250 | 1,928 | 5,852 | 2,957 | 880 | 9,914 | 80,211 | 8,409 | 3,059 | 15,228 | 3,812 |
| 60 - 64 | 161,875 | 109,614 | 1,168 | 2,619 | 1,549 | 307 | 6,201 | 75,039 | 5,345 | 1,794 | 12,551 | 3,041 |
| 65 - 69 | 118,582 | 77,547 | 674 | 1,228 | 774 | 145 | 3,358 | 56,209 | 3,245 | 910 | 8,781 | 2,223 |
| 70 - 74 | 90,802 | 51,849 | 384 | 503 | 366 | 56 | 1,607 | 39,381 | 1,755 | 430 | 5,559 | 1,808 |
| 75+ | 126,948 | 58,028 | 392 | 415 | 338 | 47 | 1,392 | 44,258 | 1,826 | 354 | 5,862 | 3,144 |
| **Male** | | | | | | | | | | | | |
| **Total** | **3,722,621** | **2,140,733** | **25,304** | **103,222** | **58,056** | **16,847** | **195,092** | **1,046,240** | **225,488** | **114,539** | **261,492** | **94,453** |
| 12 - 14 | 472,869 | 60,665 | 58 | 15 | 46 | 20 | 514 | 40,637 | 344 | 82 | 7,031 | 11,918 |
| 15 - 19 | 692,859 | 133,822 | 234 | 272 | 499 | 175 | 4,568 | 88,396 | 3,735 | 1,171 | 20,217 | 14,555 |
| 20 - 24 | 504,876 | 254,713 | 1,083 | 4,573 | 4,122 | 1,793 | 23,949 | 133,228 | 21,393 | 10,726 | 39,055 | 14,791 |
| 25 - 29 | 464,493 | 357,104 | 2,980 | 18,498 | 10,697 | 3,654 | 40,951 | 155,443 | 40,670 | 24,430 | 47,244 | 12,537 |
| 30 - 34 | 397,694 | 338,696 | 4,019 | 22,227 | 11,844 | 3,231 | 39,355 | 137,935 | 43,435 | 26,303 | 39,746 | 10,601 |
| 35 - 39 | 332,961 | 290,449 | 4,278 | 18,448 | 10,004 | 2,694 | 32,793 | 120,210 | 38,716 | 20,857 | 33,039 | 9,410 |
| 40 - 44 | 232,253 | 204,350 | 3,746 | 13,644 | 7,128 | 1,964 | 20,086 | 89,829 | 27,335 | 12,320 | 22,080 | 6,218 |
| 45 - 49 | 174,833 | 154,049 | 2,988 | 10,091 | 5,278 | 1,420 | 12,850 | 72,905 | 19,570 | 7,961 | 16,437 | 4,549 |
| 50 - 54 | 128,696 | 111,207 | 2,311 | 7,863 | 3,963 | 1,024 | 8,391 | 55,398 | 12,499 | 4,972 | 11,499 | 3,287 |
| 55 - 59 | 90,037 | 72,933 | 1,519 | 3,991 | 2,167 | 482 | 4,887 | 40,190 | 7,235 | 2,771 | 7,712 | 1,979 |
| 60 - 64 | 73,419 | 56,629 | 902 | 1,923 | 1,176 | 210 | 3,206 | 35,533 | 4,652 | 1,568 | 6,028 | 1,431 |
| 65 - 69 | 53,695 | 40,855 | 546 | 965 | 583 | 109 | 1,792 | 27,875 | 2,756 | 765 | 4,480 | 984 |
| 70 - 74 | 41,929 | 29,265 | 316 | 403 | 288 | 42 | 945 | 21,377 | 1,547 | 339 | 3,195 | 813 |
| 75+ | 62,007 | 35,996 | 324 | 309 | 261 | 29 | 805 | 27,284 | 1,601 | 274 | 3,729 | 1,380 |
| **Female** | | | | | | | | | | | | |
| **Total** | **3,992,401** | **1,746,319** | **10,594** | **74,112** | **26,581** | **20,476** | **193,178** | **1,046,575** | **41,565** | **9,150** | **235,620** | **88,468** |
| 12 - 14 | 486,104 | 57,889 | 49 | 17 | 46 | 11 | 690 | 37,941 | 302 | 72 | 6,438 | 12,323 |
| 15 - 19 | 735,025 | 164,110 | 231 | 334 | 409 | 345 | 6,567 | 115,905 | 1,954 | 384 | 23,385 | 14,596 |
| 20 - 24 | 612,600 | 259,021 | 850 | 5,809 | 2,882 | 3,157 | 27,003 | 161,296 | 5,292 | 1,244 | 36,964 | 14,524 |
| 25 - 29 | 541,751 | 286,851 | 1,786 | 18,731 | 6,134 | 5,146 | 39,464 | 154,270 | 7,705 | 1,665 | 38,971 | 12,979 |
| 30 - 34 | 403,076 | 238,523 | 2,018 | 17,519 | 5,366 | 3,911 | 36,814 | 122,362 | 7,230 | 1,478 | 32,877 | 8,948 |
| 35 - 39 | 314,852 | 197,427 | 1,648 | 11,832 | 3,913 | 2,904 | 29,589 | 105,671 | 6,452 | 1,339 | 27,488 | 6,591 |
| 40 - 44 | 215,331 | 139,956 | 1,294 | 7,100 | 2,672 | 1,927 | 18,742 | 79,146 | 4,460 | 906 | 19,047 | 4,662 |
| 45 - 49 | 180,156 | 118,076 | 1,013 | 5,462 | 2,096 | 1,450 | 14,331 | 70,055 | 3,261 | 759 | 16,032 | 3,617 |
| 50 - 54 | 141,558 | 90,856 | 766 | 4,282 | 1,554 | 1,062 | 9,141 | 57,090 | 2,120 | 473 | 11,581 | 2,787 |
| 55 - 59 | 94,791 | 59,317 | 409 | 1,861 | 790 | 398 | 5,027 | 40,021 | 1,174 | 288 | 7,516 | 1,833 |
| 60 - 64 | 88,456 | 52,985 | 266 | 696 | 373 | 97 | 2,995 | 39,506 | 693 | 226 | 6,523 | 1,610 |
| 65 - 69 | 64,887 | 36,692 | 128 | 263 | 191 | 36 | 1,566 | 28,334 | 489 | 145 | 4,301 | 1,239 |
| 70 - 74 | 48,873 | 22,584 | 68 | 100 | 78 | 14 | 662 | 18,004 | 208 | 91 | 2,364 | 995 |
| 75+ | 64,941 | 22,032 | 68 | 106 | 77 | 18 | 587 | 16,974 | 225 | 80 | 2,133 | 1,764 |

## Table E5: Population (De Facto) 12 Years and Older by Age Group, Sex and Occupation Category, Rural, Zambia 2010

| Age Group, Sex and Rural/ Urban | Population 12 Years and Older | Usual Working Population | Occupation | | | | | | | | | |
|---|---|---|---|---|---|---|---|---|---|---|---|---|
| | | | Managers | Professionals | Technicians and Associate Professionals | Clerical Support Workers | Service and Sales Workers | Skilled Agricultural Forestry and Fishery Workers | Craft and Related Trades Workers | Plant and Machine Operators and Assemblers | Elementary Occupations | Not Stated |
| **Zambia Rural** | | | | | | | | | | | | |
| **Total** | **4,366,877** | **2,624,368** | **8,145** | **45,490** | **16,739** | **3,306** | **54,317** | **1,954,531** | **67,757** | **18,465** | **346,715** | **108,903** |
| 12 - 14 | 574,194 | 109,491 | 95 | 16 | 69 | 20 | 527 | 75,580 | 399 | 132 | 12,670 | 19,983 |
| 15 - 19 | 802,997 | 262,207 | 381 | 269 | 379 | 92 | 2,574 | 195,844 | 2,611 | 545 | 37,501 | 22,011 |
| 20 - 24 | 598,270 | 375,523 | 740 | 2663 | 1,434 | 435 | 7,823 | 279,638 | 7,838 | 2,073 | 55,820 | 17,059 |
| 25 - 29 | 529,481 | 401,585 | 1,068 | 10327 | 3,062 | 598 | 10,648 | 291,646 | 11,704 | 3,447 | 56,306 | 12,779 |
| 30 - 34 | 419,932 | 337,115 | 1,061 | 10885 | 3,135 | 540 | 9,880 | 242,928 | 11,489 | 3,445 | 44,842 | 8,910 |
| 35 - 39 | 351,970 | 287,729 | 935 | 7124 | 2,472 | 500 | 8,153 | 210,041 | 10,321 | 2,990 | 37,951 | 7,242 |
| 40 - 44 | 254,964 | 211,016 | 876 | 4550 | 1,821 | 382 | 5,342 | 156,578 | 7,466 | 1,931 | 26,952 | 5,118 |
| 45 - 49 | 209,868 | 174,484 | 750 | 3677 | 1,485 | 281 | 3,714 | 131,672 | 5,679 | 1,431 | 21,774 | 4,021 |
| 50 - 54 | 162,395 | 133,872 | 659 | 3309 | 1,229 | 252 | 2,362 | 102,352 | 3,773 | 978 | 15,939 | 3,019 |
| 55 - 59 | 111,511 | 91,214 | 427 | 1389 | 663 | 92 | 1,277 | 71,832 | 2,145 | 551 | 10,858 | 1,980 |
| 60 - 64 | 107,038 | 83,565 | 361 | 594 | 419 | 56 | 954 | 67,708 | 1,599 | 399 | 9,571 | 1,904 |
| 65 - 69 | 83,157 | 62,429 | 297 | 335 | 251 | 34 | 507 | 51,168 | 1,188 | 246 | 6,957 | 1,446 |
| 70 - 74 | 66,388 | 43,838 | 226 | 178 | 155 | 9 | 271 | 36,298 | 720 | 155 | 4,587 | 1,239 |
| 75+ | 94,712 | 50,300 | 269 | 174 | 165 | 15 | 285 | 41,246 | 825 | 142 | 4,987 | 2,192 |
| **Male** | | | | | | | | | | | | |
| **Total** | **2,093,514** | **1,341,036** | **5,630** | **28,938** | **11,127** | **1,884** | **27,897** | **970,705** | **49,448** | **13,951** | **178,531** | **52,925** |
| 12 - 14 | 291,542 | 56,266 | 54 | 10 | 34 | 12 | 217 | 39,119 | 190 | 65 | 6,670 | 9,895 |
| 15 - 19 | 397,738 | 115,324 | 194 | 130 | 162 | 32 | 884 | 84,327 | 1,225 | 264 | 17,099 | 11,007 |
| 20 - 24 | 269,368 | 173,886 | 429 | 1279 | 807 | 222 | 3,429 | 125,662 | 4,975 | 1,441 | 27,340 | 8,302 |
| 25 - 29 | 241,136 | 205,566 | 693 | 5491 | 1,881 | 317 | 5,466 | 145,452 | 8,189 | 2,714 | 29,588 | 5,775 |
| 30 - 34 | 203,507 | 183,042 | 752 | 6930 | 2,148 | 322 | 5,267 | 127,802 | 8,646 | 2,787 | 24,082 | 4,306 |
| 35 - 39 | 173,650 | 157,697 | 685 | 4928 | 1,720 | 294 | 4,509 | 110,914 | 7,953 | 2,361 | 20,637 | 3,696 |
| 40 - 44 | 127,044 | 115,773 | 626 | 3285 | 1,311 | 231 | 3,000 | 82,808 | 5,846 | 1,489 | 14,615 | 2,562 |
| 45 - 49 | 100,903 | 92,222 | 554 | 2583 | 1,027 | 154 | 2,078 | 67,156 | 4,435 | 1,069 | 11,197 | 1,969 |
| 50 - 54 | 75,868 | 68,891 | 465 | 2382 | 870 | 157 | 1,252 | 50,609 | 2,917 | 744 | 8,031 | 1,464 |
| 55 - 59 | 52,225 | 46,714 | 307 | 969 | 471 | 60 | 691 | 35,737 | 1,637 | 408 | 5,500 | 934 |
| 60 - 64 | 45,761 | 39,769 | 256 | 433 | 282 | 40 | 493 | 31,429 | 1,240 | 261 | 4,499 | 836 |
| 65 - 69 | 36,611 | 31,186 | 218 | 253 | 177 | 27 | 290 | 25,009 | 917 | 157 | 3,503 | 635 |
| 70 - 74 | 30,553 | 23,857 | 179 | 136 | 116 | 6 | 158 | 19,419 | 592 | 100 | 2,600 | 551 |
| 75+ | 47,608 | 30,843 | 218 | 129 | 121 | 10 | 163 | 25,262 | 686 | 91 | 3,170 | 993 |
| **Female** | | | | | | | | | | | | |
| **Total** | **2,273,363** | **1,283,332** | **2,515** | **16,552** | **5,612** | **1,422** | **26,420** | **983,826** | **18,309** | **4,514** | **168,184** | **55,978** |
| 12 - 14 | 282,652 | 53,225 | 41 | 6 | 35 | 8 | 310 | 36,461 | 209 | 67 | 6,000 | 10,088 |
| 15 - 19 | 405,259 | 146,883 | 187 | 139 | 217 | 60 | 1,690 | 111,517 | 1,386 | 281 | 20,402 | 11,004 |
| 20 - 24 | 328,902 | 201,637 | 311 | 1384 | 627 | 213 | 4,394 | 153,976 | 2,863 | 632 | 28,480 | 8,757 |
| 25 - 29 | 288,345 | 196,019 | 375 | 4836 | 1,181 | 281 | 5,182 | 146,194 | 3,515 | 733 | 26,718 | 7,004 |
| 30 - 34 | 216,425 | 154,073 | 309 | 3955 | 987 | 218 | 4,613 | 115,126 | 2,843 | 658 | 20,760 | 4,604 |
| 35 - 39 | 178,320 | 130,032 | 250 | 2196 | 752 | 206 | 3,644 | 99,127 | 2,368 | 629 | 17,314 | 3,546 |
| 40 - 44 | 127,920 | 95,243 | 250 | 1265 | 510 | 151 | 2,342 | 73,770 | 1,620 | 442 | 12,337 | 2,556 |
| 45 - 49 | 108,965 | 82,262 | 196 | 1094 | 458 | 127 | 1,636 | 64,516 | 1,244 | 362 | 10,577 | 2,052 |
| 50 - 54 | 86,527 | 64,981 | 194 | 927 | 359 | 95 | 1,110 | 51,743 | 856 | 234 | 7,908 | 1,555 |
| 55 - 59 | 59,286 | 44,500 | 120 | 420 | 192 | 32 | 586 | 36,095 | 508 | 143 | 5,358 | 1,046 |
| 60 - 64 | 61,277 | 43,796 | 105 | 161 | 137 | 16 | 461 | 36,279 | 359 | 138 | 5,072 | 1,068 |
| 65 - 69 | 46,546 | 31,243 | 79 | 82 | 74 | 7 | 217 | 26,159 | 271 | 89 | 3,454 | 811 |
| 70 - 74 | 35,835 | 19,981 | 47 | 42 | 39 | 3 | 113 | 16,879 | 128 | 55 | 1,987 | 688 |
| 75+ | 47,104 | 19,457 | 51 | 45 | 44 | 5 | 122 | 15,984 | 139 | 51 | 1,817 | 1,199 |

## Table E5: Population (De Facto) 12 Years and Older by Age Group, Sex and Occupation Category, Urban, Zambia 2010

| Age Group, Sex and Rural/ Urban | Population 12 Years and Older | Usual Working Population | Occupation | | | | | | | | | |
|---|---|---|---|---|---|---|---|---|---|---|---|---|
| | | | Managers | Professionals | Technicians and Associate Professionals | Clerical Support Workers | Service and Sales Workers | Skilled Agricultural Forestry and Fishery Workers | Craft and Related Trades Workers | Plant and Machine Operators and Assemblers | Elementary Occupations | Not Stated |
| **Zambia Urban** | | | | | | | | | | | | |
| **Total** | **3,348,145** | **1,262,684** | **27,753** | **131,844** | **67,898** | **34,017** | **333,953** | **138,284** | **199,296** | **105,224** | **150,397** | **74,018** |
| 12 - 14 | 384,779 | 9,063 | 12 | 16 | 23 | 11 | 677 | 2998 | 247 | 22 | 799 | 4,258 |
| 15 - 19 | 624,887 | 35,725 | 84 | 337 | 529 | 428 | 8,561 | 8457 | 3,078 | 1,010 | 6,101 | 7,140 |
| 20 - 24 | 519,206 | 138,211 | 1,193 | 7,719 | 5,570 | 4,515 | 43,129 | 14886 | 18,847 | 9,897 | 20,199 | 12,256 |
| 25 - 29 | 476,763 | 242,370 | 3,698 | 26,902 | 13,769 | 8,202 | 69,767 | 18067 | 36,671 | 22,648 | 29,909 | 12,737 |
| 30 - 34 | 380,838 | 240,104 | 4,976 | 28,861 | 14,075 | 6,602 | 66,289 | 17369 | 39,176 | 24,336 | 27,781 | 10,639 |
| 35 - 39 | 295,843 | 200,147 | 4,991 | 23,156 | 11,445 | 5,098 | 54,229 | 15840 | 34,847 | 19,206 | 22,576 | 8,759 |
| 40 - 44 | 192,620 | 133,290 | 4,164 | 16,194 | 7,979 | 3,509 | 33,486 | 12397 | 24,329 | 11,295 | 14,175 | 5,762 |
| 45 - 49 | 145,121 | 97,641 | 3,251 | 11,876 | 5,889 | 2,589 | 23,467 | 11288 | 17,152 | 7,289 | 10,695 | 4,145 |
| 50 - 54 | 107,859 | 68,191 | 2,418 | 8,836 | 4,288 | 1,834 | 15,170 | 10136 | 10,846 | 4,467 | 7,141 | 3,055 |
| 55 - 59 | 73,317 | 41,036 | 1,501 | 4,463 | 2,294 | 788 | 8,637 | 8379 | 6,264 | 2,508 | 4,370 | 1,832 |
| 60 - 64 | 54,837 | 26,049 | 807 | 2,025 | 1,130 | 251 | 5,247 | 7331 | 3,746 | 1,395 | 2,980 | 1,137 |
| 65 - 69 | 35,425 | 15,118 | 377 | 893 | 523 | 111 | 2,851 | 5041 | 2,057 | 664 | 1,824 | 777 |
| 70 - 74 | 24,414 | 8,011 | 158 | 325 | 211 | 47 | 1,336 | 3083 | 1,035 | 275 | 972 | 569 |
| 75+ | 32,236 | 7,728 | 123 | 241 | 173 | 32 | 1,107 | 3012 | 1,001 | 212 | 875 | 952 |
| **Male** | | | | | | | | | | | | |
| **Total** | **1,629,107** | **799,697** | **19,674** | **74,284** | **46,929** | **14,963** | **167,195** | **75,535** | **176,040** | **100,588** | **82,961** | **41,528** |
| 12 - 14 | 181,327 | 4,399 | 4 | 5 | 12 | 8 | 297 | 1518 | 154 | 17 | 361 | 2,023 |
| 15 - 19 | 295,121 | 18,498 | 40 | 142 | 337 | 143 | 3,684 | 4069 | 2,510 | 907 | 3,118 | 3,548 |
| 20 - 24 | 235,508 | 80,827 | 654 | 3,294 | 3,315 | 1,571 | 20,520 | 7566 | 16,418 | 9,285 | 11,715 | 6,489 |
| 25 - 29 | 223,357 | 151,538 | 2,287 | 13,007 | 8,816 | 3,337 | 35,485 | 9991 | 32,481 | 21,716 | 17,656 | 6,762 |
| 30 - 34 | 194,187 | 155,654 | 3,267 | 15,297 | 9,696 | 2,909 | 34,088 | 10133 | 34,789 | 23,516 | 15,664 | 6,295 |
| 35 - 39 | 159,311 | 132,752 | 3,593 | 13,520 | 8,284 | 2,400 | 28,284 | 9296 | 30,763 | 18,496 | 12,402 | 5,714 |
| 40 - 44 | 105,209 | 88,577 | 3,120 | 10,359 | 5,817 | 1,733 | 17,086 | 7021 | 21,489 | 10,831 | 7,465 | 3,656 |
| 45 - 49 | 73,930 | 61,827 | 2,434 | 7,508 | 4,251 | 1,266 | 10,772 | 5749 | 15,135 | 6,892 | 5,240 | 2,580 |
| 50 - 54 | 52,828 | 42,316 | 1,846 | 5,481 | 3,093 | 867 | 7,139 | 4789 | 9,582 | 4,228 | 3,468 | 1,823 |
| 55 - 59 | 37,812 | 26,219 | 1,212 | 3,022 | 1,696 | 422 | 4,196 | 4453 | 5,598 | 2,363 | 2,212 | 1,045 |
| 60 - 64 | 27,658 | 16,860 | 646 | 1,490 | 894 | 170 | 2,713 | 4104 | 3,412 | 1,307 | 1,529 | 595 |
| 65 - 69 | 17,084 | 9,669 | 328 | 712 | 406 | 82 | 1,502 | 2866 | 1,839 | 608 | 977 | 349 |
| 70 - 74 | 11,376 | 5,408 | 137 | 267 | 172 | 36 | 787 | 1958 | 955 | 239 | 595 | 262 |
| 75+ | 14,399 | 5,153 | 106 | 180 | 140 | 19 | 642 | 2022 | 915 | 183 | 559 | 387 |
| **Female** | | | | | | | | | | | | |
| **Total** | **1,719,038** | **462,987** | **8,079** | **57,560** | **20,969** | **19,054** | **166,758** | **62,749** | **23,256** | **4,636** | **67,436** | **32,490** |
| 12 - 14 | 203,452 | 4,664 | 8 | 11 | 11 | 3 | 380 | 1480 | 93 | 5 | 438 | 2,235 |
| 15 - 19 | 329,766 | 17,227 | 44 | 195 | 192 | 285 | 4,877 | 4388 | 568 | 103 | 2,983 | 3,592 |
| 20 - 24 | 283,698 | 57,384 | 539 | 4,425 | 2,255 | 2,944 | 22,609 | 7320 | 2,429 | 612 | 8,484 | 5,767 |
| 25 - 29 | 253,406 | 90,832 | 1,411 | 13,895 | 4,953 | 4,865 | 34,282 | 8076 | 4,190 | 932 | 12,253 | 5,975 |
| 30 - 34 | 186,651 | 84,450 | 1,709 | 13,564 | 4,379 | 3,693 | 32,201 | 7236 | 4,387 | 820 | 12,117 | 4,344 |
| 35 - 39 | 136,532 | 67,395 | 1,398 | 9,636 | 3,161 | 2,698 | 25,945 | 6544 | 4,084 | 710 | 10,174 | 3,045 |
| 40 - 44 | 87,411 | 44,713 | 1,044 | 5,835 | 2,162 | 1,776 | 16,400 | 5376 | 2,840 | 464 | 6,710 | 2,106 |
| 45 - 49 | 71,191 | 35,814 | 817 | 4,368 | 1,638 | 1,323 | 12,695 | 5539 | 2,017 | 397 | 5,455 | 1,565 |
| 50 - 54 | 55,031 | 25,875 | 572 | 3,355 | 1,195 | 967 | 8,031 | 5347 | 1,264 | 239 | 3,673 | 1,232 |
| 55 - 59 | 35,505 | 14,817 | 289 | 1,441 | 598 | 366 | 4,441 | 3926 | 666 | 145 | 2,158 | 787 |
| 60 - 64 | 27,179 | 9,189 | 161 | 535 | 236 | 81 | 2,534 | 3227 | 334 | 88 | 1,451 | 542 |
| 65 - 69 | 18,341 | 5,449 | 49 | 181 | 117 | 29 | 1,349 | 2175 | 218 | 56 | 847 | 428 |
| 70 - 74 | 13,038 | 2,603 | 21 | 58 | 39 | 11 | 549 | 1125 | 80 | 36 | 377 | 307 |
| 75+ | 17,837 | 2,575 | 17 | 61 | 33 | 13 | 465 | 990 | 86 | 29 | 316 | 565 |

## Table E6: Usually Working Population (De Facto) 12 Years and Older by Industry, Sex and Highest Level of Education Completed, Rural/Urban, Zambia 2010

| Industry, Sex and Rural/Urban | Usually Working Population | Never Atended School | Highest Level of Education Completed | | | |
|---|---|---|---|---|---|---|
| | | | None | Primary | Secondary | Tertiary |
| **Zambia Total** | | | | | | |
| **Total** | **3,887,052** | **718,558** | **12,015** | **1,614,242** | **1,107,961** | **434,276** |
| Agriculture Hunting Forestry and Fishing | 2,440,748 | 628,384 | 9,136 | 1,259,619 | 500,924 | 42,685 |
| Mining and Quarrying | 69,922 | 1,821 | 25 | 7,842 | 37,572 | 22,662 |
| Manufacturing | 132,292 | 6,650 | 158 | 30,417 | 60,448 | 34,619 |
| Electricity Gas Steam and Air conditioning supply | 8,578 | 44 | 3 | 390 | 2,361 | 5,780 |
| Water Supply | 6,729 | 210 | 2 | 1,151 | 2,919 | 2,447 |
| Construction and Allied Repairs | 110,991 | 5,272 | 130 | 31,918 | 54,388 | 19,283 |
| Wholesale & Retail Trade Restaurants and Hotel | 361,660 | 22,693 | 515 | 111,894 | 186,893 | 39,665 |
| Transport and Storage | 91,113 | 2,030 | 52 | 14,770 | 57,649 | 16,612 |
| Accomodation and food services activities | 39,220 | 1,541 | 29 | 8,303 | 18,466 | 10,881 |
| Information and Communication | 23,337 | 1,827 | 35 | 4,533 | 5,316 | 11,626 |
| Finance and Insurance | 17,228 | 55 | - | 406 | 2,608 | 14,159 |
| Real Estate Activies | 1,307 | 43 | 3 | 236 | 454 | 571 |
| Community Social and Personal Services | 366,193 | 9,935 | 242 | 53,393 | 109,523 | 193,100 |
| Not Stated | 217,734 | 38,053 | 1,685 | 89,370 | 68,440 | 20,186 |
| **Male** | | | | | | |
| **Total** | **2,140,733** | **281,216** | **4,964** | **807,836** | **760,036** | **286,681** |
| Agriculture Hunting Forestry and Fishing | 1,227,232 | 242,494 | 3,713 | 620,032 | 328,026 | 32,967 |
| Mining and Quarrying | 63,840 | 1,091 | 17 | 6,183 | 35,890 | 20,659 |
| Manufacturing | 103,436 | 3,637 | 105 | 22,073 | 50,828 | 26,793 |
| Electricity Gas Steam and Air conditioning supply | 7,563 | 39 | 3 | 369 | 2,184 | 4,968 |
| Water Supply | 5,407 | 144 | 1 | 877 | 2,496 | 1,889 |
| Construction and Allied Repairs | 107,979 | 5,021 | 117 | 30,959 | 53,514 | 18,368 |
| Wholesale & Retail Trade Restaurants and Hotel | 171,651 | 5,819 | 155 | 37,475 | 102,458 | 25,744 |
| Transport and Storage | 87,203 | 1,852 | 45 | 14,046 | 56,272 | 14,988 |
| Accomodation and food services activities | 18,146 | 448 | 6 | 3,227 | 9,360 | 5,105 |
| Information and Communication | 14,287 | 768 | 16 | 2,352 | 3,802 | 7,349 |
| Finance and Insurance | 10,875 | 39 | - | 276 | 1,729 | 8,831 |
| Real Estate Activies | 898 | 20 | 1 | 159 | 356 | 362 |
| Community Social and Personal Services | 213,519 | 4,937 | 124 | 28,484 | 73,892 | 106,082 |
| Not Stated | 108,697 | 14,907 | 661 | 41,324 | 39,229 | 12,576 |
| **Female** | | | | | | |
| **Total** | **1,746,319** | **437,342** | **7,051** | **806,406** | **347,925** | **147,595** |
| Agriculture Hunting Forestry and Fishing | 1,213,516 | 385,890 | 5,423 | 639,587 | 172,898 | 9,718 |
| Mining and Quarrying | 6,082 | 730 | 8 | 1,659 | 1,682 | 2,003 |
| Manufacturing | 28,856 | 3,013 | 53 | 8,344 | 9,620 | 7,826 |
| Electricity Gas Steam and Air conditioning supply | 1,015 | 5 | - | 21 | 177 | 812 |
| Water Supply | 1,322 | 66 | 1 | 274 | 423 | 558 |
| Construction and Allied Repairs | 3,012 | 251 | 13 | 959 | 874 | 915 |
| Wholesale & Retail Trade Restaurants and Hotel | 190,009 | 16,874 | 360 | 74,419 | 84,435 | 13,921 |
| Transport and Storage | 3,910 | 178 | 7 | 724 | 1,377 | 1,624 |
| Accomodation and food services activities | 21,074 | 1,093 | 23 | 5,076 | 9,106 | 5,776 |
| Information and Communication | 9,050 | 1,059 | 19 | 2,181 | 1,514 | 4,277 |
| Finance and Insurance | 6,353 | 16 | - | 130 | 879 | 5,328 |
| Real Estate Activies | 409 | 23 | 2 | 77 | 98 | 209 |
| Community Social and Personal Services | 152,674 | 4,998 | 118 | 24,909 | 35,631 | 87,018 |
| Not Stated | 109,037 | 23,146 | 1,024 | 48,046 | 29,211 | 7,610 |

## Table E6: Usually Working Population (De Facto) 12 Years and Older by Industry, Sex and Highest Level of Education Completed, Rural, Zambia 2010

| Industry, Sex and Rural/Urban | Usually Working Population | Never Atended School | Highest Level of Education Completed | | | |
|---|---|---|---|---|---|---|
| | | | None | Primary | Secondary | Tertiary |
| **Zambia Rural** | | | | | | |
| **Total** | **2,624,368** | **659,219** | **10,315** | **1,326,436** | **533,724** | **94,674** |
| Agriculture Hunting Forestry and Fishing | 2,276,211 | 605,975 | 8,743 | 1,189,270 | 442,510 | 29,713 |
| Mining and Quarrying | 9,173 | 1,264 | 15 | 3,753 | 3,091 | 1,050 |
| Manufacturing | 27,930 | 3,964 | 72 | 11,751 | 8,330 | 3,813 |
| Electricity Gas Steam and Air conditioning supply | 869 | 14 | - | 97 | 327 | 431 |
| Water Supply | 505 | 36 | - | 164 | 193 | 112 |
| Construction and Allied Repairs | 26,002 | 2,333 | 59 | 11,220 | 9,741 | 2,649 |
| Wholesale & Retail Trade Restaurants and Hotel | 54,243 | 7,608 | 154 | 25,200 | 19,117 | 2,164 |
| Transport and Storage | 8,251 | 529 | 9 | 2,445 | 3,977 | 1,291 |
| Accomodation and food services activities | 5,069 | 507 | 11 | 1,955 | 1,960 | 636 |
| Information and Communication | 8,270 | 1,717 | 33 | 3,904 | 1,843 | 773 |
| Finance and Insurance | 813 | 18 | - | 58 | 187 | 550 |
| Real Estate Activies | 251 | 24 | 1 | 114 | 82 | 30 |
| Community Social and Personal Services | 76,707 | 3,151 | 73 | 11,306 | 15,319 | 46,858 |
| Not Stated | 130,074 | 32,079 | 1,145 | 65,199 | 27,047 | 4,604 |
| **Male** | | | | | | |
| **Total** | **1,341,036** | **256,847** | **4,260** | **659,955** | **352,445** | **67,529** |
| Agriculture Hunting Forestry and Fishing | 1,136,245 | 234,121 | 3,547 | 585,969 | 289,307 | 23,301 |
| Mining and Quarrying | 7,128 | 713 | 9 | 2,765 | 2,732 | 909 |
| Manufacturing | 19,357 | 1,933 | 41 | 7,831 | 6,625 | 2,927 |
| Electricity Gas Steam and Air conditioning supply | 789 | 13 | - | 93 | 305 | 378 |
| Water Supply | 433 | 27 | - | 130 | 174 | 102 |
| Construction and Allied Repairs | 25,281 | 2,197 | 51 | 10,830 | 9,607 | 2,596 |
| Wholesale & Retail Trade Restaurants and Hotel | 22,344 | 1,983 | 44 | 8,858 | 10,027 | 1,432 |
| Transport and Storage | 7,735 | 442 | 7 | 2,247 | 3,831 | 1,208 |
| Accomodation and food services activities | 2,520 | 149 | 1 | 859 | 1,135 | 376 |
| Information and Communication | 4,457 | 719 | 14 | 1,960 | 1,255 | 509 |
| Finance and Insurance | 548 | 12 | - | 46 | 141 | 349 |
| Real Estate Activies | 192 | 15 | 1 | 85 | 73 | 18 |
| Community Social and Personal Services | 50,856 | 1,686 | 44 | 7,421 | 11,421 | 30,284 |
| Not Stated | 63,151 | 12,837 | 501 | 30,861 | 15,812 | 3,140 |
| **Female** | | | | | | |
| **Total** | **1,283,332** | **402,372** | **6,055** | **666,481** | **181,279** | **27,145** |
| Agriculture Hunting Forestry and Fishing | 1,139,966 | 371,854 | 5,196 | 603,301 | 153,203 | 6,412 |
| Mining and Quarrying | 2,045 | 551 | 6 | 988 | 359 | 141 |
| Manufacturing | 8,573 | 2,031 | 31 | 3,920 | 1,705 | 886 |
| Electricity Gas Steam and Air conditioning supply | 80 | 1 | - | 4 | 22 | 53 |
| Water Supply | 72 | 9 | - | 34 | 19 | 10 |
| Construction and Allied Repairs | 721 | 136 | 8 | 390 | 134 | 53 |
| Wholesale & Retail Trade Restaurants and Hotel | 31,899 | 5,625 | 110 | 16,342 | 9,090 | 732 |
| Transport and Storage | 516 | 87 | 2 | 198 | 146 | 83 |
| Accomodation and food services activities | 2,549 | 358 | 10 | 1,096 | 825 | 260 |
| Information and Communication | 3,813 | 998 | 19 | 1,944 | 588 | 264 |
| Finance and Insurance | 265 | 6 | - | 12 | 46 | 201 |
| Real Estate Activies | 59 | 9 | - | 29 | 9 | 12 |
| Community Social and Personal Services | 25,851 | 1,465 | 29 | 3,885 | 3,898 | 16,574 |
| Not Stated | 66,923 | 19,242 | 644 | 34,338 | 11,235 | 1,464 |

## Table E6: Usually Working Population (De Facto) 12 Years and Older by Industry, Sex and Highest Level of Education Completed, Urban, Zambia 2010

| Industry, Sex and Rural/Urban | Usually Working Population | Never Atended School | Highest Level of Education Completed | | | |
|---|---|---|---|---|---|---|
| | | | None | Primary | Secondary | Tertiary |
| **Zambia Urban** | | | | | | |
| **Total** | **1,262,684** | **59,339** | **1,700** | **287,806** | **574,237** | **339,602** |
| Agriculture Hunting Forestry and Fishing | 164,537 | 22,409 | 393 | 70,349 | 58,414 | 12,972 |
| Mining and Quarrying | 60,749 | 557 | 10 | 4,089 | 34,481 | 21,612 |
| Manufacturing | 104,362 | 2,686 | 86 | 18,666 | 52,118 | 30,806 |
| Electricity Gas Steam and Air conditioning supply | 7,709 | 30 | 3 | 293 | 2,034 | 5,349 |
| Water Supply | 6,224 | 174 | 2 | 987 | 2,726 | 2,335 |
| Construction and Allied Repairs | 84,989 | 2,939 | 71 | 20,698 | 44,647 | 16,634 |
| Wholesale & Retail Trade Restaurants and Hotel | 307,417 | 15,085 | 361 | 86,694 | 167,776 | 37,501 |
| Transport and Storage | 82,862 | 1,501 | 43 | 12,325 | 53,672 | 15,321 |
| Accomodation and food services activities | 34,151 | 1,034 | 18 | 6,348 | 16,506 | 10,245 |
| Information and Communication | 15,067 | 110 | 2 | 629 | 3,473 | 10,853 |
| Finance and Insurance | 16,415 | 37 | - | 348 | 2,421 | 13,609 |
| Real Estate Activies | 1,056 | 19 | 2 | 122 | 372 | 541 |
| Community Social and Personal Services | 289,486 | 6,784 | 169 | 42,087 | 94,204 | 146,242 |
| Not Stated | 87,660 | 5,974 | 540 | 24,171 | 41,393 | 15,582 |
| **Male** | | | | | | |
| **Total** | **799,697** | **24,369** | **704** | **147,881** | **407,591** | **219,152** |
| Agriculture Hunting Forestry and Fishing | 90,987 | 8,373 | 166 | 34,063 | 38,719 | 9,666 |
| Mining and Quarrying | 56,712 | 378 | 8 | 3,418 | 33,158 | 19,750 |
| Manufacturing | 84,079 | 1,704 | 64 | 14,242 | 44,203 | 23,866 |
| Electricity Gas Steam and Air conditioning supply | 6,774 | 26 | 3 | 276 | 1,879 | 4,590 |
| Water Supply | 4,974 | 117 | 1 | 747 | 2,322 | 1,787 |
| Construction and Allied Repairs | 82,698 | 2,824 | 66 | 20,129 | 43,907 | 15,772 |
| Wholesale & Retail Trade Restaurants and Hotel | 149,307 | 3,836 | 111 | 28,617 | 92,431 | 24,312 |
| Transport and Storage | 79,468 | 1,410 | 38 | 11,799 | 52,441 | 13,780 |
| Accomodation and food services activities | 15,626 | 299 | 5 | 2,368 | 8,225 | 4,729 |
| Information and Communication | 9,830 | 49 | 2 | 392 | 2,547 | 6,840 |
| Finance and Insurance | 10,327 | 27 | - | 230 | 1,588 | 8,482 |
| Real Estate Activies | 706 | 5 | - | 74 | 283 | 344 |
| Community Social and Personal Services | 162,663 | 3,251 | 80 | 21,063 | 62,471 | 75,798 |
| Not Stated | 45,546 | 2,070 | 160 | 10,463 | 23,417 | 9,436 |
| **Female** | | | | | | |
| **Total** | **462,987** | **34,970** | **996** | **139,925** | **166,646** | **120,450** |
| Agriculture Hunting Forestry and Fishing | 73,550 | 14,036 | 227 | 36,286 | 19,695 | 3,306 |
| Mining and Quarrying | 4,037 | 179 | 2 | 671 | 1,323 | 1,862 |
| Manufacturing | 20,283 | 982 | 22 | 4,424 | 7,915 | 6,940 |
| Electricity Gas Steam and Air conditioning supply | 935 | 4 | - | 17 | 155 | 759 |
| Water Supply | 1,250 | 57 | 1 | 240 | 404 | 548 |
| Construction and Allied Repairs | 2,291 | 115 | 5 | 569 | 740 | 862 |
| Wholesale & Retail Trade Restaurants and Hotel | 158,110 | 11,249 | 250 | 58,077 | 75,345 | 13,189 |
| Transport and Storage | 3,394 | 91 | 5 | 526 | 1,231 | 1,541 |
| Accomodation and food services activities | 18,525 | 735 | 13 | 3,980 | 8,281 | 5,516 |
| Information and Communication | 5,237 | 61 | - | 237 | 926 | 4,013 |
| Finance and Insurance | 6,088 | 10 | - | 118 | 833 | 5,127 |
| Real Estate Activies | 350 | 14 | 2 | 48 | 89 | 197 |
| Community Social and Personal Services | 126,823 | 3,533 | 89 | 21,024 | 31,733 | 70,444 |
| Not Stated | 42,114 | 3,904 | 380 | 13,708 | 17,976 | 6,146 |

## Table E7: Usually Working Population (De Facto) 12 Years and Older by Occupation, Sex and Highest Level of Education Completed, Rural/Urban, Zambia 2010

| Occupation, Sex and Rural/Urban | Usually Working Population | Never Atended School | Highest Level of Education Completed | | | |
|---|---|---|---|---|---|---|
| | | | None | Primary | Secondary | Tertiary |
| **Zambia Total** | | | | | | |
| **Total** | **3,887,052** | **718,558** | **12,015** | **1,614,242** | **1,107,961** | **434,276** |
| Managers | 35,898 | 1,327 | 26 | 3,952 | 7,391 | 23,202 |
| Professionals | 177,334 | 1,091 | 24 | 3,583 | 13,971 | 158,665 |
| Technicians and Associate Professionals | 84,637 | 1,882 | 49 | 6,786 | 21,325 | 54,595 |
| Clerical Support Workers | 37,323 | 286 | 6 | 1,682 | 13,421 | 21,928 |
| Service and Sales Workers | 388,270 | 19,792 | 481 | 110,522 | 212,698 | 44,777 |
| Skilled Agricultural Forestry and Fishery Workers | 2,092,815 | 553,128 | 7,963 | 1,077,111 | 422,603 | 32,010 |
| Craft and Related Trades Workers | 267,053 | 16,345 | 337 | 72,099 | 128,952 | 49,320 |
| Plant and Machine Operators and Assemblers | 123,689 | 4,048 | 83 | 20,732 | 77,994 | 20,832 |
| Elementary Occupations | 497,112 | 88,919 | 1,470 | 244,134 | 150,035 | 12,554 |
| Not Stated | 182,921 | 31,740 | 1,576 | 73,641 | 59,571 | 16,393 |
| **Male** | | | | | | |
| **Total** | **2,140,733** | **281,216** | **4,964** | **807,836** | **760,036** | **286,681** |
| Managers | 25,304 | 706 | 18 | 2,430 | 5,434 | 16,716 |
| Professionals | 103,222 | 531 | 13 | 2,107 | 9,391 | 91,180 |
| Technicians and Associate Professionals | 58,056 | 846 | 37 | 4,392 | 16,318 | 36,463 |
| Clerical Support Workers | 16,847 | 145 | 4 | 987 | 7,550 | 8,161 |
| Service and Sales Workers | 195,092 | 6,101 | 159 | 41,969 | 120,136 | 26,727 |
| Skilled Agricultural Forestry and Fishery Workers | 1,046,240 | 212,087 | 3,208 | 529,842 | 276,350 | 24,753 |
| Craft and Related Trades Workers | 225,488 | 9,967 | 235 | 56,287 | 116,358 | 42,641 |
| Plant and Machine Operators and Assemblers | 114,539 | 2,332 | 62 | 17,112 | 75,217 | 19,816 |
| Elementary Occupations | 261,492 | 36,045 | 616 | 118,626 | 97,355 | 8,850 |
| Not Stated | 94,453 | 12,456 | 612 | 34,084 | 35,927 | 11,374 |
| **Female** | | | | | | |
| **Total** | **1,746,319** | **437,342** | **7,051** | **806,406** | **347,925** | **147,595** |
| Managers | 10,594 | 621 | 8 | 1,522 | 1,957 | 6,486 |
| Professionals | 74,112 | 560 | 11 | 1,476 | 4,580 | 67,485 |
| Technicians and Associate Professionals | 26,581 | 1,036 | 12 | 2,394 | 5,007 | 18,132 |
| Clerical Support Workers | 20,476 | 141 | 2 | 695 | 5,871 | 13,767 |
| Service and Sales Workers | 193,178 | 13,691 | 322 | 68,553 | 92,562 | 18,050 |
| Skilled Agricultural Forestry and Fishery Workers | 1,046,575 | 341,041 | 4,755 | 547,269 | 146,253 | 7,257 |
| Craft and Related Trades Workers | 41,565 | 6,378 | 102 | 15,812 | 12,594 | 6,679 |
| Plant and Machine Operators and Assemblers | 9,150 | 1,716 | 21 | 3,620 | 2,777 | 1,016 |
| Elementary Occupations | 235,620 | 52,874 | 854 | 125,508 | 52,680 | 3,704 |
| Not Stated | 88,468 | 19,284 | 964 | 39,557 | 23,644 | 5,019 |

## Table E7: Usually Working Population (De Facto) 12 Years and Older by Occupation, Sex and Highest Level of Education Completed, Rural, Zambia 2010

| Occupation, Sex and Rural/Urban | Usually Working Population | Never Atended School | Highest Level of Education Completed | | | |
|---|---|---|---|---|---|---|
| | | | None | Primary | Secondary | Tertiary |
| **Zambia Rural** | | | | | | |
| **Total** | **2,624,368** | **659,219** | **10,315** | **1,326,436** | **533,724** | **94,674** |
| Managers | 8,145 | 1,149 | 20 | 2,968 | 1,931 | 2,077 |
| Professionals | 45,490 | 922 | 6 | 2,016 | 3,580 | 38,966 |
| Technicians and Associate Professionals | 16,739 | 1,353 | 22 | 3,202 | 3,645 | 8,517 |
| Clerical Support Workers | 3,306 | 153 | - | 509 | 1,161 | 1,483 |
| Service and Sales Workers | 54,317 | 5,641 | 129 | 22,330 | 22,320 | 3,897 |
| Skilled Agricultural Forestry and Fishery Workers | 1,954,531 | 533,727 | 7,625 | 1,017,095 | 372,730 | 23,354 |
| Craft and Related Trades Workers | 67,757 | 10,123 | 177 | 30,627 | 21,071 | 5,759 |
| Plant and Machine Operators and Assemblers | 18,465 | 2,301 | 34 | 6,611 | 7,291 | 2,228 |
| Elementary Occupations | 346,715 | 77,044 | 1,234 | 186,341 | 77,230 | 4,866 |
| Not Stated | 108,903 | 26,806 | 1,068 | 54,737 | 22,765 | 3,527 |
| **Male** | | | | | | |
| **Total** | **1,341,036** | **256,847** | **4,260** | **659,955** | **352,445** | **67,529** |
| Managers | 5,630 | 607 | 13 | 1,905 | 1,474 | 1,631 |
| Professionals | 28,938 | 436 | 3 | 1,169 | 2,467 | 24,863 |
| Technicians and Associate Professionals | 11,127 | 548 | 14 | 2,004 | 2,726 | 5,835 |
| Clerical Support Workers | 1,884 | 72 | - | 306 | 798 | 708 |
| Service and Sales Workers | 27,897 | 1,886 | 47 | 9,697 | 13,475 | 2,792 |
| Skilled Agricultural Forestry and Fishery Workers | 970,705 | 204,881 | 3,072 | 500,812 | 243,578 | 18,362 |
| Craft and Related Trades Workers | 49,448 | 5,238 | 104 | 21,438 | 17,599 | 5,069 |
| Plant and Machine Operators and Assemblers | 13,951 | 1,018 | 19 | 4,346 | 6,503 | 2,065 |
| Elementary Occupations | 178,531 | 31,418 | 522 | 92,423 | 50,494 | 3,674 |
| Not Stated | 52,925 | 10,743 | 466 | 25,855 | 13,331 | 2,530 |
| **Female** | | | | | | |
| **Total** | **1,283,332** | **402,372** | **6,055** | **666,481** | **181,279** | **27,145** |
| Managers | 2,515 | 542 | 7 | 1,063 | 457 | 446 |
| Professionals | 16,552 | 486 | 3 | 847 | 1,113 | 14,103 |
| Technicians and Associate Professionals | 5,612 | 805 | 8 | 1,198 | 919 | 2,682 |
| Clerical Support Workers | 1,422 | 81 | - | 203 | 363 | 775 |
| Service and Sales Workers | 26,420 | 3,755 | 82 | 12,633 | 8,845 | 1,105 |
| Skilled Agricultural Forestry and Fishery Workers | 983,826 | 328,846 | 4,553 | 516,283 | 129,152 | 4,992 |
| Craft and Related Trades Workers | 18,309 | 4,885 | 73 | 9,189 | 3,472 | 690 |
| Plant and Machine Operators and Assemblers | 4,514 | 1,283 | 15 | 2,265 | 788 | 163 |
| Elementary Occupations | 168,184 | 45,626 | 712 | 93,918 | 26,736 | 1,192 |
| Not Stated | 55,978 | 16,063 | 602 | 28,882 | 9,434 | 997 |

## Table E7: Usually Working Population (De Facto) 12 Years and Older by Occupation, Sex and Highest Level of Education Completed, Urban, Zambia 2010

| Occupation, Sex and Rural/Urban | Usually Working Population | Never Atended School | Highest Level of Education Completed | | | |
|---|---|---|---|---|---|---|
| | | | None | Primary | Secondary | Tertiary |
| **Zambia Urban** | | | | | | |
| **Total** | **1,262,684** | **59,339** | **1,700** | **287,806** | **574,237** | **339,602** |
| Managers | 27,753 | 178 | 6 | 984 | 5,460 | 21,125 |
| Professionals | 131,844 | 169 | 18 | 1,567 | 10,391 | 119,699 |
| Technicians and Associate Professionals | 67,898 | 529 | 27 | 3,584 | 17,680 | 46,078 |
| Clerical Support Workers | 34,017 | 133 | 6 | 1,173 | 12,260 | 20,445 |
| Service and Sales Workers | 333,953 | 14,151 | 352 | 88,192 | 190,378 | 40,880 |
| Skilled Agricultural Forestry and Fishery Workers | 138,284 | 19,401 | 338 | 60,016 | 49,873 | 8,656 |
| Craft and Related Trades Workers | 199,296 | 6,222 | 160 | 41,472 | 107,881 | 43,561 |
| Plant and Machine Operators and Assemblers | 105,224 | 1,747 | 49 | 14,121 | 70,703 | 18,604 |
| Elementary Occupations | 150,397 | 11,875 | 236 | 57,793 | 72,805 | 7,688 |
| Not Stated | 74,018 | 4,934 | 508 | 18,904 | 36,806 | 12,866 |
| **Male** | | | | | | |
| **Total** | **799,697** | **24,369** | **704** | **147,881** | **407,591** | **219,152** |
| Managers | 19,674 | 99 | 5 | 525 | 3,960 | 15,085 |
| Professionals | 74,284 | 95 | 10 | 938 | 6,924 | 66,317 |
| Technicians and Associate Professionals | 46,929 | 298 | 23 | 2,388 | 13,592 | 30,628 |
| Clerical Support Workers | 14,963 | 73 | 4 | 681 | 6,752 | 7,453 |
| Service and Sales Workers | 167,195 | 4,215 | 112 | 32,272 | 106,661 | 23,935 |
| Skilled Agricultural Forestry and Fishery Workers | 75,535 | 7,206 | 136 | 29,030 | 32,772 | 6,391 |
| Craft and Related Trades Workers | 176,040 | 4,729 | 131 | 34,849 | 98,759 | 37,572 |
| Plant and Machine Operators and Assemblers | 100,588 | 1,314 | 43 | 12,766 | 68,714 | 17,751 |
| Elementary Occupations | 82,961 | 4,627 | 94 | 26,203 | 46,861 | 5,176 |
| Not Stated | 41,528 | 1,713 | 146 | 8,229 | 22,596 | 8,844 |
| **Female** | | | | | | |
| **Total** | **462,987** | **34,970** | **996** | **139,925** | **166,646** | **120,450** |
| Managers | 8,079 | 79 | 1 | 459 | 1,500 | 6,040 |
| Professionals | 57,560 | 74 | 8 | 629 | 3,467 | 53,382 |
| Technicians and Associate Professionals | 20,969 | 231 | 4 | 1,196 | 4,088 | 15,450 |
| Clerical Support Workers | 19,054 | 60 | 2 | 492 | 5,508 | 12,992 |
| Service and Sales Workers | 166,758 | 9,936 | 240 | 55,920 | 83,717 | 16,945 |
| Skilled Agricultural Forestry and Fishery Workers | 62,749 | 12,195 | 202 | 30,986 | 17,101 | 2,265 |
| Craft and Related Trades Workers | 23,256 | 1,493 | 29 | 6,623 | 9,122 | 5,989 |
| Plant and Machine Operators and Assemblers | 4,636 | 433 | 6 | 1,355 | 1,989 | 853 |
| Elementary Occupations | 67,436 | 7,248 | 142 | 31,590 | 25,944 | 2,512 |
| Not Stated | 32,490 | 3,221 | 362 | 10,675 | 14,210 | 4,022 |

## Table E8: Usually Working Population (De Facto) 12 Years and Older by Age Group, Sex and Highest Level of Education Completed, Rural/Urban, Zambia 2010

| Age Group, Sex and Rural/ Urban | Usually Working Population | Never Attended | None | Primary School Grade | | | | | | | | Secondary School Grade | | | | | | Tertiary |
|---|---|---|---|---|---|---|---|---|---|---|---|---|---|---|---|---|---|---|
| | | | | Total 1-7 | 1 | 2 | 3 | 4 | 5 | 6 | 7 | Total 8-12 | 8 | 9 | 10 | 11 | 12 | |
| **Zambia Total** | | | | | | | | | | | | | | | | | | |
| **Total** | **3,887,052** | **718,558** | **12,015** | **1,614,242** | **54,431** | **113,756** | **144,430** | **207,358** | **221,215** | **293,573** | **579,479** | **1,107,961** | **232,415** | **404,424** | **112,573** | **64,111** | **294,438** | **434,276** |
| 12 - 14 | 118,554 | 36,022 | 1,133 | 78,444 | 4,060 | 8,926 | 13,804 | 17,167 | 16,464 | 11,334 | 6,689 | 2,955 | 2,229 | 668 | 58 | - | - | - |
| 15 - 19 | 297,932 | 64,852 | 1,216 | 161,341 | 4,113 | 8,927 | 13,578 | 20,637 | 28,073 | 36,038 | 49,975 | 69,191 | 30,548 | 24,906 | 4,793 | 2,903 | 6,041 | 1,332 |
| 20 - 24 | 513,734 | 88,279 | 1,445 | 207,401 | 5,792 | 12,376 | 17,236 | 24,660 | 30,245 | 41,215 | 75,877 | 191,306 | 46,404 | 65,799 | 13,980 | 11,430 | 53,693 | 25,303 |
| 25 - 29 | 643,955 | 101,394 | 1,662 | 240,267 | 7,429 | 15,807 | 20,513 | 29,248 | 34,652 | 44,453 | 88,165 | 218,559 | 43,127 | 73,340 | 17,029 | 13,362 | 71,701 | 82,073 |
| 30 - 34 | 577,219 | 82,896 | 1,340 | 213,078 | 5,865 | 12,600 | 16,475 | 23,758 | 28,655 | 39,339 | 86,386 | 190,676 | 36,563 | 73,924 | 15,850 | 10,972 | 53,367 | 89,229 |
| 35 - 39 | 487,876 | 71,524 | 1,155 | 186,547 | 4,803 | 10,624 | 14,360 | 21,220 | 24,402 | 34,236 | 76,902 | 156,098 | 28,304 | 64,363 | 13,709 | 8,562 | 41,160 | 72,552 |
| 40 - 44 | 344,306 | 53,556 | 775 | 137,958 | 3,436 | 7,265 | 9,770 | 15,465 | 15,896 | 24,964 | 61,162 | 99,892 | 16,868 | 40,750 | 11,616 | 5,411 | 25,247 | 52,125 |
| 45 - 49 | 272,125 | 45,783 | 703 | 115,884 | 2,797 | 6,172 | 8,761 | 13,636 | 12,669 | 20,364 | 51,485 | 70,424 | 10,633 | 23,968 | 14,175 | 4,327 | 17,321 | 39,331 |
| 50 - 54 | 202,063 | 38,312 | 554 | 86,620 | 2,813 | 5,549 | 7,202 | 10,782 | 9,256 | 14,218 | 36,800 | 45,943 | 6,582 | 14,442 | 11,066 | 2,876 | 10,977 | 30,634 |
| 55 - 59 | 132,250 | 28,681 | 409 | 57,385 | 2,357 | 4,999 | 5,394 | 7,913 | 6,363 | 9,216 | 21,143 | 27,248 | 4,324 | 9,329 | 5,385 | 1,777 | 6,433 | 18,527 |
| 60 - 64 | 109,614 | 33,726 | 497 | 47,613 | 3,200 | 6,458 | 5,853 | 8,118 | 5,375 | 7,064 | 11,545 | 16,659 | 2,950 | 6,000 | 2,549 | 1,151 | 4,009 | 11,119 |
| 65 - 69 | 77,547 | 27,754 | 405 | 34,153 | 2,764 | 5,537 | 4,536 | 6,234 | 3,844 | 4,956 | 6,282 | 9,257 | 1,851 | 3,359 | 1,219 | 607 | 2,221 | 5,978 |
| 70 - 74 | 51,849 | 20,959 | 309 | 22,629 | 2,214 | 3,931 | 3,210 | 4,054 | 2,568 | 3,109 | 3,543 | 4,832 | 997 | 1,775 | 567 | 368 | 1,125 | 3,120 |
| 75+ | 58,028 | 24,820 | 412 | 24,922 | 2,788 | 4,585 | 3,738 | 4,466 | 2,753 | 3,067 | 3,525 | 4,921 | 1,035 | 1,801 | 577 | 365 | 1,143 | 2,953 |
| **Male** | | | | | | | | | | | | | | | | | | |
| **Total** | **2,140,733** | **281,216** | **4,964** | **807,836** | **22,280** | **48,483** | **62,130** | **93,443** | **104,415** | **151,459** | **325,626** | **760,036** | **139,199** | **263,584** | **81,221** | **47,524** | **228,508** | **286,681** |
| 12 - 14 | 60,665 | 19,275 | 593 | 39,363 | 2,170 | 4,764 | 7,094 | 8,665 | 8,052 | 5,405 | 3,213 | 1,434 | 1,065 | 330 | 39 | - | - | - |
| 15 - 19 | 133,822 | 28,167 | 557 | 72,385 | 1,770 | 3,880 | 6,104 | 9,357 | 12,673 | 16,306 | 22,295 | 32,141 | 13,621 | 11,263 | 2,524 | 1,499 | 3,234 | 572 |
| 20 - 24 | 254,713 | 33,014 | 567 | 92,576 | 2,065 | 4,235 | 6,145 | 9,619 | 12,584 | 19,042 | 38,886 | 116,178 | 25,554 | 38,763 | 9,088 | 7,398 | 35,375 | 12,378 |
| 25 - 29 | 357,104 | 40,500 | 578 | 117,496 | 2,776 | 6,116 | 7,883 | 12,420 | 15,934 | 22,591 | 49,776 | 151,765 | 26,443 | 47,917 | 12,542 | 10,139 | 54,724 | 46,765 |
| 30 - 34 | 338,696 | 35,708 | 580 | 109,153 | 2,511 | 5,264 | 6,791 | 10,456 | 13,702 | 20,791 | 49,638 | 136,373 | 23,469 | 49,005 | 11,952 | 8,578 | 43,369 | 56,882 |
| 35 - 39 | 290,449 | 29,982 | 480 | 96,297 | 2,029 | 4,554 | 5,998 | 9,513 | 11,702 | 18,250 | 44,251 | 113,982 | 18,651 | 44,075 | 10,227 | 6,786 | 34,243 | 49,708 |
| 40 - 44 | 204,350 | 21,381 | 321 | 71,587 | 1,375 | 3,030 | 4,025 | 6,707 | 7,514 | 13,351 | 35,585 | 73,818 | 11,412 | 28,925 | 8,258 | 4,100 | 21,123 | 37,243 |
| 45 - 49 | 154,049 | 16,862 | 280 | 57,987 | 1,008 | 2,375 | 3,495 | 5,544 | 5,699 | 10,538 | 29,328 | 50,860 | 6,899 | 16,632 | 9,873 | 3,190 | 14,266 | 28,060 |
| 50 - 54 | 111,207 | 12,397 | 202 | 43,133 | 949 | 1,915 | 2,916 | 4,244 | 4,098 | 7,409 | 21,602 | 33,732 | 4,107 | 9,780 | 8,372 | 2,222 | 9,251 | 21,743 |
| 55 - 59 | 72,933 | 9,071 | 142 | 29,340 | 769 | 1,857 | 2,204 | 3,309 | 3,072 | 5,019 | 13,110 | 20,553 | 2,820 | 6,500 | 4,335 | 1,453 | 5,445 | 13,827 |
| 60 - 64 | 56,629 | 9,168 | 184 | 24,986 | 1,033 | 2,518 | 2,492 | 3,748 | 2,938 | 4,355 | 7,902 | 13,335 | 2,146 | 4,674 | 2,038 | 989 | 3,488 | 8,956 |
| 65 - 69 | 40,855 | 8,051 | 147 | 20,026 | 1,112 | 2,583 | 2,314 | 3,598 | 2,434 | 3,461 | 4,524 | 7,553 | 1,371 | 2,706 | 990 | 520 | 1,966 | 5,078 |
| 70 - 74 | 29,265 | 7,113 | 122 | 15,134 | 1,057 | 2,254 | 1,991 | 2,791 | 1,871 | 2,420 | 2,750 | 4,110 | 802 | 1,499 | 478 | 324 | 1,007 | 2,786 |
| 75+ | 35,996 | 10,527 | 211 | 18,373 | 1,656 | 3,138 | 2,678 | 3,472 | 2,142 | 2,521 | 2,766 | 4,202 | 839 | 1,515 | 505 | 326 | 1,017 | 2,683 |
| **Female** | | | | | | | | | | | | | | | | | | |
| **Total** | **1,746,319** | **437,342** | **7,051** | **806,406** | **32,151** | **65,273** | **82,300** | **113,915** | **116,800** | **142,114** | **253,853** | **347,925** | **93,216** | **140,840** | **31,352** | **16,587** | **65,930** | **147,595** |
| 12 - 14 | 57,889 | 16,747 | 540 | 39,081 | 1,890 | 4,162 | 6,710 | 8,502 | 8,412 | 5,929 | 3,476 | 1,521 | 1,164 | 338 | 19 | - | - | - |
| 15 - 19 | 164,110 | 36,685 | 659 | 88,956 | 2,343 | 5,047 | 7,474 | 11,280 | 15,400 | 19,732 | 27,680 | 37,050 | 16,927 | 13,643 | 2,269 | 1,404 | 2,807 | 760 |
| 20 - 24 | 259,021 | 55,265 | 878 | 114,825 | 3,727 | 8,141 | 11,091 | 15,041 | 17,661 | 22,173 | 36,991 | 75,128 | 20,850 | 27,036 | 4,892 | 4,032 | 18,318 | 12,925 |
| 25 - 29 | 286,851 | 60,894 | 1,084 | 122,771 | 4,653 | 9,691 | 12,630 | 16,828 | 18,718 | 21,862 | 38,389 | 66,794 | 16,684 | 25,423 | 4,487 | 3,223 | 16,977 | 35,308 |
| 30 - 34 | 238,523 | 47,188 | 760 | 103,925 | 3,354 | 7,336 | 9,684 | 13,302 | 14,953 | 18,548 | 36,748 | 54,303 | 13,094 | 24,919 | 3,898 | 2,394 | 9,998 | 32,347 |
| 35 - 39 | 197,427 | 41,542 | 675 | 90,250 | 2,774 | 6,070 | 8,362 | 11,707 | 12,700 | 15,986 | 32,651 | 42,116 | 9,653 | 20,288 | 3,482 | 1,776 | 6,917 | 22,844 |
| 40 - 44 | 139,956 | 32,175 | 454 | 66,371 | 2,061 | 4,235 | 5,745 | 8,758 | 8,382 | 11,613 | 25,577 | 26,074 | 5,456 | 11,825 | 3,358 | 1,311 | 4,124 | 14,882 |
| 45 - 49 | 118,076 | 28,921 | 423 | 57,897 | 1,789 | 3,797 | 5,266 | 8,092 | 6,970 | 9,826 | 22,157 | 19,564 | 3,734 | 7,336 | 4,302 | 1,137 | 3,055 | 11,271 |
| 50 - 54 | 90,856 | 25,915 | 352 | 43,487 | 1,864 | 3,634 | 4,286 | 6,538 | 5,158 | 6,809 | 15,198 | 12,211 | 2,475 | 4,662 | 2,694 | 654 | 1,726 | 8,891 |
| 55 - 59 | 59,317 | 19,610 | 267 | 28,045 | 1,588 | 3,142 | 3,190 | 4,604 | 3,291 | 4,197 | 8,033 | 6,695 | 1,504 | 2,829 | 1,050 | 324 | 988 | 4,700 |
| 60 - 64 | 52,985 | 24,558 | 313 | 22,627 | 2,167 | 3,940 | 3,361 | 4,370 | 2,437 | 2,709 | 3,643 | 3,324 | 804 | 1,326 | 511 | 162 | 521 | 2,163 |
| 65 - 69 | 36,692 | 19,703 | 258 | 14,127 | 1,652 | 2,954 | 2,222 | 2,636 | 1,410 | 1,495 | 1,758 | 1,704 | 480 | 653 | 229 | 87 | 255 | 900 |
| 70 - 74 | 22,584 | 13,846 | 187 | 7,495 | 1,157 | 1,677 | 1,219 | 1,263 | 697 | 689 | 793 | 722 | 195 | 276 | 89 | 44 | 118 | 334 |
| 75+ | 22,032 | 14,293 | 201 | 6,549 | 1,132 | 1,447 | 1,060 | 994 | 611 | 546 | 759 | 719 | 196 | 286 | 72 | 39 | 126 | 270 |

## Table E8: Usually Working Population (De Facto) 12 Years and Older by Age Group, Sex and Highest Level of Education Completed, Rural, Zambia 2010

| Age Group, Sex and Rural/ Urban | Usually Working Population | Never Attended | None | Primary School Grade | | | | | | | | Secondary School Grade | | | | | | Tertiary |
|---|---|---|---|---|---|---|---|---|---|---|---|---|---|---|---|---|---|---|
| | | | | Total 1-7 | 1 | 2 | 3 | 4 | 5 | 6 | 7 | Total 8-12 | 8 | 9 | 10 | 11 | 12 | |
| **Zambia Rural** | | | | | | | | | | | | | | | | | | |
| **Total** | **2,624,368** | **659,219** | **10,315** | **1,326,436** | **48,675** | **101,828** | **128,064** | **182,004** | **188,584** | **242,654** | **434,627** | **533,724** | **160,844** | **219,233** | **50,358** | **26,132** | **77,157** | **94,674** |
| 12 - 14 | 109,491 | 35,024 | 1,083 | 71,138 | 3,875 | 8,478 | 12,970 | 15,815 | 14,789 | 9,802 | 5,409 | 2,246 | 1,714 | 498 | 34 | - | - | - |
| 15 - 19 | 262,207 | 62,226 | 1,125 | 146,956 | 3,866 | 8,490 | 12,787 | 19,337 | 25,914 | 32,812 | 43,750 | 51,265 | 25,783 | 18,946 | 2,941 | 1,525 | 2,070 | 635 |
| 20 - 24 | 375,523 | 82,429 | 1,270 | 177,514 | 5,354 | 11,473 | 15,755 | 22,091 | 26,387 | 35,344 | 61,110 | 109,072 | 35,622 | 43,351 | 7,218 | 5,604 | 17,277 | 5,238 |
| 25 - 29 | 401,585 | 93,022 | 1,374 | 194,283 | 6,694 | 14,260 | 18,360 | 25,468 | 29,181 | 35,999 | 64,321 | 96,396 | 28,507 | 37,827 | 6,639 | 4,941 | 18,482 | 16,510 |
| 30 - 34 | 337,115 | 75,129 | 1,095 | 166,572 | 5,190 | 11,218 | 14,469 | 20,376 | 23,630 | 31,022 | 60,667 | 76,650 | 22,247 | 34,206 | 5,436 | 3,556 | 11,205 | 17,669 |
| 35 - 39 | 287,729 | 64,348 | 956 | 145,983 | 4,256 | 9,471 | 12,563 | 18,229 | 20,247 | 27,219 | 53,998 | 63,274 | 17,197 | 30,067 | 4,624 | 2,760 | 8,626 | 13,168 |
| 40 - 44 | 211,016 | 48,327 | 648 | 109,392 | 3,045 | 6,383 | 8,471 | 13,364 | 13,223 | 20,184 | 44,722 | 42,969 | 10,487 | 20,087 | 4,776 | 1,982 | 5,637 | 9,680 |
| 45 - 49 | 174,484 | 41,265 | 584 | 92,039 | 2,447 | 5,336 | 7,468 | 11,749 | 10,556 | 16,574 | 37,909 | 32,362 | 6,884 | 12,388 | 6,862 | 1,858 | 4,370 | 8,234 |
| 50 - 54 | 133,872 | 34,326 | 442 | 68,686 | 2,401 | 4,681 | 5,969 | 9,209 | 7,570 | 11,470 | 27,386 | 22,627 | 4,327 | 7,902 | 5,822 | 1,406 | 3,170 | 7,791 |
| 55 - 59 | 91,214 | 25,612 | 347 | 45,627 | 1,968 | 4,169 | 4,461 | 6,700 | 5,134 | 7,361 | 15,834 | 14,432 | 2,926 | 5,369 | 2,969 | 934 | 2,234 | 5,196 |
| 60 - 64 | 83,565 | 30,606 | 425 | 38,810 | 2,785 | 5,462 | 4,870 | 6,886 | 4,283 | 5,647 | 8,877 | 9,685 | 2,141 | 3,751 | 1,465 | 654 | 1,674 | 4,039 |
| 65 - 69 | 62,429 | 25,107 | 342 | 28,378 | 2,385 | 4,806 | 3,854 | 5,301 | 3,139 | 4,014 | 4,879 | 5,815 | 1,387 | 2,228 | 743 | 370 | 1,087 | 2,787 |
| 70 - 74 | 43,838 | 19,134 | 265 | 19,305 | 1,939 | 3,473 | 2,775 | 3,514 | 2,142 | 2,597 | 2,865 | 3,328 | 779 | 1,263 | 404 | 262 | 620 | 1,806 |
| 75+ | 50,300 | 22,664 | 359 | 21,753 | 2,470 | 4,128 | 3,292 | 3,965 | 2,389 | 2,609 | 2,900 | 3,603 | 843 | 1,350 | 425 | 280 | 705 | 1,921 |
| **Male** | | | | | | | | | | | | | | | | | | |
| **Total** | **1,341,036** | **256,847** | **4,260** | **659,955** | **19,791** | **43,185** | **54,937** | **82,008** | **89,158** | **125,824** | **245,052** | **352,445** | **95,860** | **141,034** | **36,210** | **19,281** | **60,060** | **67,529** |
| 12 - 14 | 56,266 | 18,812 | 568 | 35,793 | 2,078 | 4,528 | 6,672 | 7,973 | 7,276 | 4,678 | 2,588 | 1,093 | 825 | 244 | 24 | - | - | - |
| 15 - 19 | 115,324 | 26,950 | 515 | 64,919 | 1,657 | 3,674 | 5,702 | 8,672 | 11,553 | 14,609 | 19,052 | 22,672 | 11,243 | 8,064 | 1,496 | 758 | 1,111 | 268 |
| 20 - 24 | 173,886 | 30,279 | 499 | 77,057 | 1,859 | 3,825 | 5,496 | 8,466 | 10,783 | 16,120 | 30,508 | 63,199 | 19,329 | 24,527 | 4,539 | 3,494 | 11,310 | 2,852 |
| 25 - 29 | 205,566 | 36,533 | 488 | 93,576 | 2,474 | 5,425 | 6,957 | 10,716 | 13,414 | 18,377 | 36,213 | 65,183 | 17,692 | 24,634 | 4,863 | 3,739 | 14,255 | 9,786 |
| 30 - 34 | 183,042 | 32,021 | 473 | 85,103 | 2,164 | 4,633 | 5,972 | 9,000 | 11,413 | 16,649 | 35,272 | 53,329 | 14,527 | 22,713 | 4,108 | 2,808 | 9,173 | 12,116 |
| 35 - 39 | 157,697 | 26,728 | 385 | 75,172 | 1,767 | 4,022 | 5,242 | 8,179 | 9,771 | 14,710 | 31,481 | 45,686 | 11,599 | 20,997 | 3,489 | 2,241 | 7,360 | 9,726 |
| 40 - 44 | 115,773 | 19,161 | 251 | 57,235 | 1,204 | 2,645 | 3,506 | 5,866 | 6,322 | 11,053 | 26,639 | 31,690 | 7,290 | 14,620 | 3,388 | 1,557 | 4,835 | 7,436 |
| 45 - 49 | 92,222 | 15,197 | 233 | 46,983 | 898 | 2,068 | 3,007 | 4,872 | 4,867 | 8,844 | 22,427 | 23,586 | 4,664 | 8,960 | 4,900 | 1,414 | 3,648 | 6,223 |
| 50 - 54 | 68,891 | 11,183 | 152 | 34,845 | 823 | 1,629 | 2,424 | 3,670 | 3,453 | 6,182 | 16,664 | 16,746 | 2,838 | 5,536 | 4,493 | 1,119 | 2,760 | 5,965 |
| 55 - 59 | 46,714 | 8,187 | 123 | 23,543 | 637 | 1,558 | 1,836 | 2,840 | 2,534 | 4,124 | 10,014 | 10,848 | 1,940 | 3,789 | 2,402 | 781 | 1,936 | 4,013 |
| 60 - 64 | 39,769 | 8,316 | 151 | 20,241 | 893 | 2,125 | 2,048 | 3,172 | 2,367 | 3,519 | 6,117 | 7,731 | 1,563 | 2,930 | 1,173 | 570 | 1,495 | 3,330 |
| 65 - 69 | 31,186 | 7,338 | 126 | 16,578 | 961 | 2,245 | 1,977 | 3,083 | 1,976 | 2,786 | 3,550 | 4,737 | 1,034 | 1,806 | 612 | 319 | 966 | 2,407 |
| 70 - 74 | 23,857 | 6,470 | 103 | 12,840 | 910 | 1,977 | 1,715 | 2,412 | 1,575 | 2,019 | 2,232 | 2,825 | 624 | 1,064 | 345 | 233 | 559 | 1,619 |
| 75+ | 30,843 | 9,672 | 193 | 16,070 | 1,466 | 2,831 | 2,383 | 3,087 | 1,854 | 2,154 | 2,295 | 3,120 | 692 | 1,150 | 378 | 248 | 652 | 1,788 |
| **Female** | | | | | | | | | | | | | | | | | | |
| **Total** | **1,283,332** | **402,372** | **6,055** | **666,481** | **28,884** | **58,643** | **73,127** | **99,996** | **99,426** | **116,830** | **189,575** | **181,279** | **64,984** | **78,199** | **14,148** | **6,851** | **17,097** | **27,145** |
| 12 - 14 | 53,225 | 16,212 | 515 | 35,345 | 1,797 | 3,950 | 6,298 | 7,842 | 7,513 | 5,124 | 2,821 | 1,153 | 889 | 254 | 10 | - | - | - |
| 15 - 19 | 146,883 | 35,276 | 610 | 82,037 | 2,209 | 4,816 | 7,085 | 10,665 | 14,361 | 18,203 | 24,698 | 28,593 | 14,540 | 10,882 | 1,445 | 767 | 959 | 367 |
| 20 - 24 | 201,637 | 52,150 | 771 | 100,457 | 3,495 | 7,648 | 10,259 | 13,625 | 15,604 | 19,224 | 30,602 | 45,873 | 16,293 | 18,824 | 2,679 | 2,110 | 5,967 | 2,386 |
| 25 - 29 | 196,019 | 56,489 | 886 | 100,707 | 4,220 | 8,835 | 11,403 | 14,752 | 15,767 | 17,622 | 28,108 | 31,213 | 10,815 | 13,193 | 1,776 | 1,202 | 4,227 | 6,724 |
| 30 - 34 | 154,073 | 43,108 | 622 | 81,469 | 3,026 | 6,585 | 8,497 | 11,376 | 12,217 | 14,373 | 25,395 | 23,321 | 7,720 | 11,493 | 1,328 | 748 | 2,032 | 5,553 |
| 35 - 39 | 130,032 | 37,620 | 571 | 70,811 | 2,489 | 5,449 | 7,321 | 10,050 | 10,476 | 12,509 | 22,517 | 17,588 | 5,598 | 9,070 | 1,135 | 519 | 1,266 | 3,442 |
| 40 - 44 | 95,243 | 29,166 | 397 | 52,157 | 1,841 | 3,738 | 4,965 | 7,498 | 6,901 | 9,131 | 18,083 | 11,279 | 3,197 | 5,467 | 1,388 | 425 | 802 | 2,244 |
| 45 - 49 | 82,262 | 26,068 | 351 | 45,056 | 1,549 | 3,268 | 4,461 | 6,877 | 5,689 | 7,730 | 15,482 | 8,776 | 2,220 | 3,428 | 1,962 | 444 | 722 | 2,011 |
| 50 - 54 | 64,981 | 23,143 | 290 | 33,841 | 1,578 | 3,052 | 3,545 | 5,539 | 4,117 | 5,288 | 10,722 | 5,881 | 1,489 | 2,366 | 1,329 | 287 | 410 | 1,826 |
| 55 - 59 | 44,500 | 17,425 | 224 | 22,084 | 1,331 | 2,611 | 2,625 | 3,860 | 2,600 | 3,237 | 5,820 | 3,584 | 986 | 1,580 | 567 | 153 | 298 | 1,183 |
| 60 - 64 | 43,796 | 22,290 | 274 | 18,569 | 1,892 | 3,337 | 2,822 | 3,714 | 1,916 | 2,128 | 2,760 | 1,954 | 578 | 821 | 292 | 84 | 179 | 709 |
| 65 - 69 | 31,243 | 17,769 | 216 | 11,800 | 1,424 | 2,561 | 1,877 | 2,218 | 1,163 | 1,228 | 1,329 | 1,078 | 353 | 422 | 131 | 51 | 121 | 380 |
| 70 - 74 | 19,981 | 12,664 | 162 | 6,465 | 1,029 | 1,496 | 1,060 | 1,102 | 567 | 578 | 633 | 503 | 155 | 199 | 59 | 29 | 61 | 187 |
| 75+ | 19,457 | 12,992 | 166 | 5,683 | 1,004 | 1,297 | 909 | 878 | 535 | 455 | 605 | 483 | 151 | 200 | 47 | 32 | 53 | 133 |

## Table E8: Usually Working Population (De Facto) 12 Years and Older by Age Group, Sex and Highest Level of Education Completed, Urban, Zambia 2010

| Age Group, Sex and Rural/ Urban | Usually Working Population | Never Attended | None | Primary School Grade | | | | | | | | Secondary School Grade | | | | | | Tertiary |
|---|---|---|---|---|---|---|---|---|---|---|---|---|---|---|---|---|---|---|
| | | | | Total 1-7 | 1 | 2 | 3 | 4 | 5 | 6 | 7 | Total 8-12 | 8 | 9 | 10 | 11 | 12 | |
| **Zambia Urban** | | | | | | | | | | | | | | | | | | |
| **Total** | **1,262,684** | **59,339** | **1,700** | **287,806** | **5,756** | **11,928** | **16,366** | **25,354** | **32,631** | **50,919** | **144,852** | **574,237** | **71,571** | **185,191** | **62,215** | **37,979** | **217,281** | **339,602** |
| 12 - 14 | 9,063 | 998 | 50 | 7,306 | 185 | 448 | 834 | 1,352 | 1,675 | 1,532 | 1,280 | 709 | 515 | 170 | 24 | - | - | - |
| 15 - 19 | 35,725 | 2,626 | 91 | 14,385 | 247 | 437 | 791 | 1,300 | 2,159 | 3,226 | 6,225 | 17,926 | 4,765 | 5,960 | 1,852 | 1,378 | 3,971 | 697 |
| 20 - 24 | 138,211 | 5,850 | 175 | 29,887 | 438 | 903 | 1,481 | 2,569 | 3,858 | 5,871 | 14,767 | 82,234 | 10,782 | 22,448 | 6,762 | 5,826 | 36,416 | 20,065 |
| 25 - 29 | 242,370 | 8,372 | 288 | 45,984 | 735 | 1,547 | 2,153 | 3,780 | 5,471 | 8,454 | 23,844 | 122,163 | 14,620 | 35,513 | 10,390 | 8,421 | 53,219 | 65,563 |
| 30 - 34 | 240,104 | 7,767 | 245 | 46,506 | 675 | 1,382 | 2,006 | 3,382 | 5,025 | 8,317 | 25,719 | 114,026 | 14,316 | 39,718 | 10,414 | 7,416 | 42,162 | 71,560 |
| 35 - 39 | 200,147 | 7,176 | 199 | 40,564 | 547 | 1,153 | 1,797 | 2,991 | 4,155 | 7,017 | 22,904 | 92,824 | 11,107 | 34,296 | 9,085 | 5,802 | 32,534 | 59,384 |
| 40 - 44 | 133,290 | 5,229 | 127 | 28,566 | 391 | 882 | 1,299 | 2,101 | 2,673 | 4,780 | 16,440 | 56,923 | 6,381 | 20,663 | 6,840 | 3,429 | 19,610 | 42,445 |
| 45 - 49 | 97,641 | 4,518 | 119 | 23,845 | 350 | 836 | 1,293 | 1,887 | 2,113 | 3,790 | 13,576 | 38,062 | 3,749 | 11,580 | 7,313 | 2,469 | 12,951 | 31,097 |
| 50 - 54 | 68,191 | 3,986 | 112 | 17,934 | 412 | 868 | 1,233 | 1,573 | 1,686 | 2,748 | 9,414 | 23,316 | 2,255 | 6,540 | 5,244 | 1,470 | 7,807 | 22,843 |
| 55 - 59 | 41,036 | 3,069 | 62 | 11,758 | 389 | 830 | 933 | 1,213 | 1,229 | 1,855 | 5,309 | 12,816 | 1,398 | 3,960 | 2,416 | 843 | 4,199 | 13,331 |
| 60 - 64 | 26,049 | 3,120 | 72 | 8,803 | 415 | 996 | 983 | 1,232 | 1,092 | 1,417 | 2,668 | 6,974 | 809 | 2,249 | 1,084 | 497 | 2,335 | 7,080 |
| 65 - 69 | 15,118 | 2,647 | 63 | 5,775 | 379 | 731 | 682 | 933 | 705 | 942 | 1,403 | 3,442 | 464 | 1,131 | 476 | 237 | 1,134 | 3,191 |
| 70 - 74 | 8,011 | 1,825 | 44 | 3,324 | 275 | 458 | 435 | 540 | 426 | 512 | 678 | 1,504 | 218 | 512 | 163 | 106 | 505 | 1,314 |
| 75+ | 7,728 | 2,156 | 53 | 3,169 | 318 | 457 | 446 | 501 | 364 | 458 | 625 | 1,318 | 192 | 451 | 152 | 85 | 438 | 1,032 |
| **Male** | | | | | | | | | | | | | | | | | | |
| **Total** | **799,697** | **24,369** | **704** | **147,881** | **2,489** | **5,298** | **7,193** | **11,435** | **15,257** | **25,635** | **80,574** | **407,591** | **43,339** | **122,550** | **45,011** | **28,243** | **168,448** | **219,152** |
| 12 - 14 | 4,399 | 463 | 25 | 3,570 | 92 | 236 | 422 | 692 | 776 | 727 | 625 | 341 | 240 | 86 | 15 | - | - | - |
| 15 - 19 | 18,498 | 1,217 | 42 | 7,466 | 113 | 206 | 402 | 685 | 1,120 | 1,697 | 3,243 | 9,469 | 2,378 | 3,199 | 1,028 | 741 | 2,123 | 304 |
| 20 - 24 | 80,827 | 2,735 | 68 | 15,519 | 206 | 410 | 649 | 1,153 | 1,801 | 2,922 | 8,378 | 52,979 | 6,225 | 14,236 | 4,549 | 3,904 | 24,065 | 9,526 |
| 25 - 29 | 151,538 | 3,967 | 90 | 23,920 | 302 | 691 | 926 | 1,704 | 2,520 | 4,214 | 13,563 | 86,582 | 8,751 | 23,283 | 7,679 | 6,400 | 40,469 | 36,979 |
| 30 - 34 | 155,654 | 3,687 | 107 | 24,050 | 347 | 631 | 819 | 1,456 | 2,289 | 4,142 | 14,366 | 83,044 | 8,942 | 26,292 | 7,844 | 5,770 | 34,196 | 44,766 |
| 35 - 39 | 132,752 | 3,254 | 95 | 21,125 | 262 | 532 | 756 | 1,334 | 1,931 | 3,540 | 12,770 | 68,296 | 7,052 | 23,078 | 6,738 | 4,545 | 26,883 | 39,982 |
| 40 - 44 | 88,577 | 2,220 | 70 | 14,352 | 171 | 385 | 519 | 841 | 1,192 | 2,298 | 8,946 | 42,128 | 4,122 | 14,305 | 4,870 | 2,543 | 16,288 | 29,807 |
| 45 - 49 | 61,827 | 1,665 | 47 | 11,004 | 110 | 307 | 488 | 672 | 832 | 1,694 | 6,901 | 27,274 | 2,235 | 7,672 | 4,973 | 1,776 | 10,618 | 21,837 |
| 50 - 54 | 42,316 | 1,214 | 50 | 8,288 | 126 | 286 | 492 | 574 | 645 | 1,227 | 4,938 | 16,986 | 1,269 | 4,244 | 3,879 | 1,103 | 6,491 | 15,778 |
| 55 - 59 | 26,219 | 884 | 19 | 5,797 | 132 | 299 | 368 | 469 | 538 | 895 | 3,096 | 9,705 | 880 | 2,711 | 1,933 | 672 | 3,509 | 9,814 |
| 60 - 64 | 16,860 | 852 | 33 | 4,745 | 140 | 393 | 444 | 576 | 571 | 836 | 1,785 | 5,604 | 583 | 1,744 | 865 | 419 | 1,993 | 5,626 |
| 65 - 69 | 9,669 | 713 | 21 | 3,448 | 151 | 338 | 337 | 515 | 458 | 675 | 974 | 2,816 | 337 | 900 | 378 | 201 | 1,000 | 2,671 |
| 70 - 74 | 5,408 | 643 | 19 | 2,294 | 147 | 277 | 276 | 379 | 296 | 401 | 518 | 1,285 | 178 | 435 | 133 | 91 | 448 | 1,167 |
| 75+ | 5,153 | 855 | 18 | 2,303 | 190 | 307 | 295 | 385 | 288 | 367 | 471 | 1,082 | 147 | 365 | 127 | 78 | 365 | 895 |
| **Female** | | | | | | | | | | | | | | | | | | |
| **Total** | **462,987** | **34,970** | **996** | **139,925** | **3,267** | **6,630** | **9,173** | **13,919** | **17,374** | **25,284** | **64,278** | **166,646** | **28,232** | **62,641** | **17,204** | **9,736** | **48,833** | **120,450** |
| 12 - 14 | 4,664 | 535 | 25 | 3,736 | 93 | 212 | 412 | 660 | 899 | 805 | 655 | 368 | 275 | 84 | 9 | - | - | - |
| 15 - 19 | 17,227 | 1,409 | 49 | 6,919 | 134 | 231 | 389 | 615 | 1,039 | 1,529 | 2,982 | 8,457 | 2,387 | 2,761 | 824 | 637 | 1,848 | 393 |
| 20 - 24 | 57,384 | 3,115 | 107 | 14,368 | 232 | 493 | 832 | 1,416 | 2,057 | 2,949 | 6,389 | 29,255 | 4,557 | 8,212 | 2,213 | 1,922 | 12,351 | 10,539 |
| 25 - 29 | 90,832 | 4,405 | 198 | 22,064 | 433 | 856 | 1,227 | 2,076 | 2,951 | 4,240 | 10,281 | 35,581 | 5,869 | 12,230 | 2,711 | 2,021 | 12,750 | 28,584 |
| 30 - 34 | 84,450 | 4,080 | 138 | 22,456 | 328 | 751 | 1,187 | 1,926 | 2,736 | 4,175 | 11,353 | 30,982 | 5,374 | 13,426 | 2,570 | 1,646 | 7,966 | 26,794 |
| 35 - 39 | 67,395 | 3,922 | 104 | 19,439 | 285 | 621 | 1,041 | 1,657 | 2,224 | 3,477 | 10,134 | 24,528 | 4,055 | 11,218 | 2,347 | 1,257 | 5,651 | 19,402 |
| 40 - 44 | 44,713 | 3,009 | 57 | 14,214 | 220 | 497 | 780 | 1,260 | 1,481 | 2,482 | 7,494 | 14,795 | 2,259 | 6,358 | 1,970 | 886 | 3,322 | 12,638 |
| 45 - 49 | 35,814 | 2,853 | 72 | 12,841 | 240 | 529 | 805 | 1,215 | 1,281 | 2,096 | 6,675 | 10,788 | 1,514 | 3,908 | 2,340 | 693 | 2,333 | 9,260 |
| 50 - 54 | 25,875 | 2,772 | 62 | 9,646 | 286 | 582 | 741 | 999 | 1,041 | 1,521 | 4,476 | 6,330 | 986 | 2,296 | 1,365 | 367 | 1,316 | 7,065 |
| 55 - 59 | 14,817 | 2,185 | 43 | 5,961 | 257 | 531 | 565 | 744 | 691 | 960 | 2,213 | 3,111 | 518 | 1,249 | 483 | 171 | 690 | 3,517 |
| 60 - 64 | 9,189 | 2,268 | 39 | 4,058 | 275 | 603 | 539 | 656 | 521 | 581 | 883 | 1,370 | 226 | 505 | 219 | 78 | 342 | 1,454 |
| 65 - 69 | 5,449 | 1,934 | 42 | 2,327 | 228 | 393 | 345 | 418 | 247 | 267 | 429 | 626 | 127 | 231 | 98 | 36 | 134 | 520 |
| 70 - 74 | 2,603 | 1,182 | 25 | 1,030 | 128 | 181 | 159 | 161 | 130 | 111 | 160 | 219 | 40 | 77 | 30 | 15 | 57 | 147 |
| 75+ | 2,575 | 1,301 | 35 | 866 | 128 | 150 | 151 | 116 | 76 | 91 | 154 | 236 | 45 | 86 | 25 | 7 | 73 | 137 |

## Table E9: Usually Unemployed Population (De Facto) 12 Years and Older by Age Group, Sex and Highest Level of Education Completed, Rural/Urban, Zambia 2010

| Age Group, Sex and Rural/ Urban | Total | Never Atended School | Total Ever Attended School | | | | | | | | | | | | | | | |
|---|---|---|---|---|---|---|---|---|---|---|---|---|---|---|---|---|---|---|
| | | | Primary School Grade | | | | | | | | | Secondary School Grade | | | | | | Tertiary |
| | | | None | Total 1-7 | 1 | 2 | 3 | 4 | 5 | 6 | 7 | Total 8-12 | 8 | 9 | 10 | 11 | 12 | |
| **Zambia Total** | | | | | | | | | | | | | | | | | | |
| **Total** | **543,490** | **60,553** | **1,152** | **162,084** | **3,482** | **7,729** | **10,986** | **16,781** | **21,320** | **30,657** | **71,129** | **272,861** | **35,460** | **75,934** | **19,833** | **15,102** | **126,532** | **46,840** |
| 12 - 14 | 19,281 | 6,719 | 162 | 11,496 | 488 | 1,090 | 1,799 | 2,260 | 2,389 | 2,013 | 1,457 | 904 | 597 | 281 | 26 | - | - | - |
| 15 - 19 | 84,864 | 11,431 | 248 | 33,344 | 591 | 1,423 | 2,225 | 3,568 | 5,089 | 7,375 | 13,073 | 39,021 | 8,295 | 11,183 | 2,580 | 2,049 | 14,914 | 820 |
| 20 - 24 | 152,376 | 10,150 | 206 | 31,756 | 535 | 1,219 | 1,784 | 2,871 | 4,019 | 6,333 | 14,995 | 98,606 | 10,143 | 21,787 | 5,180 | 5,428 | 56,068 | 11,658 |
| 25 - 29 | 104,153 | 8,628 | 151 | 25,327 | 462 | 1,025 | 1,469 | 2,277 | 3,211 | 4,646 | 12,237 | 55,951 | 6,254 | 14,344 | 3,538 | 3,167 | 28,648 | 14,096 |
| 30 - 34 | 61,678 | 6,013 | 104 | 18,043 | 302 | 668 | 913 | 1,513 | 2,100 | 3,315 | 9,232 | 30,224 | 4,115 | 10,787 | 2,296 | 1,675 | 11,351 | 7,294 |
| 35 - 39 | 42,564 | 4,679 | 80 | 13,827 | 261 | 547 | 687 | 1,196 | 1,562 | 2,412 | 7,162 | 19,707 | 2,664 | 7,752 | 1,651 | 1,105 | 6,535 | 4,271 |
| 40 - 44 | 25,718 | 3,200 | 36 | 8,707 | 155 | 348 | 457 | 796 | 948 | 1,419 | 4,584 | 11,090 | 1,455 | 4,273 | 1,273 | 596 | 3,493 | 2,685 |
| 45 - 49 | 18,564 | 2,607 | 45 | 6,598 | 125 | 275 | 423 | 625 | 633 | 1,057 | 3,460 | 7,361 | 800 | 2,362 | 1,425 | 434 | 2,340 | 1,953 |
| 50 - 54 | 11,955 | 1,857 | 23 | 4,513 | 124 | 251 | 300 | 473 | 426 | 697 | 2,242 | 4,221 | 440 | 1,231 | 954 | 300 | 1,296 | 1,341 |
| 55 - 59 | 8,308 | 1,259 | 24 | 2,999 | 76 | 228 | 241 | 351 | 300 | 514 | 1,289 | 2,787 | 305 | 893 | 512 | 164 | 913 | 1,239 |
| 60 - 64 | 5,921 | 1,289 | 20 | 2,249 | 125 | 230 | 254 | 333 | 277 | 350 | 680 | 1,521 | 179 | 496 | 237 | 97 | 512 | 842 |
| 65 - 69 | 3,651 | 1,009 | 21 | 1,482 | 84 | 199 | 195 | 228 | 161 | 254 | 361 | 773 | 102 | 290 | 91 | 44 | 246 | 366 |
| 70 - 74 | 2,084 | 748 | 15 | 865 | 75 | 105 | 117 | 135 | 104 | 140 | 189 | 311 | 53 | 110 | 27 | 20 | 101 | 145 |
| 75+ | 2,373 | 964 | 17 | 878 | 79 | 121 | 122 | 155 | 101 | 132 | 168 | 384 | 58 | 145 | 43 | 23 | 115 | 130 |
| **Male** | | | | | | | | | | | | | | | | | | |
| **Total** | **316,014** | **32,348** | **614** | **91,994** | **1,839** | **4,144** | **5,921** | **9,091** | **11,754** | **17,286** | **41,959** | **166,371** | **20,320** | **45,919** | **12,712** | **9,612** | **77,808** | **24,687** |
| 12 - 14 | 9,736 | 3,476 | 89 | 5,774 | 270 | 572 | 942 | 1,129 | 1,180 | 987 | 694 | 397 | 248 | 140 | 9 | - | - | - |
| 15 - 19 | 41,980 | 5,993 | 142 | 17,203 | 309 | 764 | 1,174 | 1,847 | 2,706 | 3,801 | 6,602 | 18,330 | 3,900 | 5,349 | 1,242 | 972 | 6,867 | 312 |
| 20 - 24 | 85,005 | 5,451 | 112 | 17,965 | 279 | 629 | 950 | 1,587 | 2,198 | 3,561 | 8,761 | 56,416 | 5,826 | 12,524 | 2,960 | 3,216 | 31,890 | 5,061 |
| 25 - 29 | 61,848 | 4,855 | 73 | 14,477 | 247 | 536 | 765 | 1,261 | 1,783 | 2,618 | 7,267 | 35,580 | 3,722 | 8,700 | 2,312 | 2,126 | 18,720 | 6,863 |
| 30 - 34 | 38,612 | 3,507 | 55 | 10,857 | 176 | 381 | 526 | 864 | 1,256 | 2,011 | 5,643 | 20,316 | 2,564 | 6,832 | 1,617 | 1,196 | 8,107 | 3,877 |
| 35 - 39 | 27,710 | 2,743 | 44 | 8,411 | 144 | 323 | 383 | 673 | 913 | 1,460 | 4,515 | 14,013 | 1,769 | 5,255 | 1,215 | 818 | 4,956 | 2,499 |
| 40 - 44 | 17,119 | 1,788 | 18 | 5,382 | 92 | 191 | 276 | 459 | 545 | 861 | 2,958 | 8,217 | 985 | 3,110 | 895 | 446 | 2,781 | 1,714 |
| 45 - 49 | 11,853 | 1,355 | 22 | 3,844 | 61 | 141 | 205 | 323 | 328 | 623 | 2,163 | 5,336 | 525 | 1,633 | 992 | 313 | 1,873 | 1,296 |
| 50 - 54 | 7,495 | 854 | 10 | 2,579 | 49 | 122 | 148 | 204 | 224 | 386 | 1,446 | 3,100 | 274 | 847 | 712 | 234 | 1,033 | 952 |
| 55 - 59 | 5,506 | 551 | 12 | 1,827 | 31 | 105 | 125 | 192 | 181 | 332 | 861 | 2,207 | 209 | 681 | 430 | 136 | 751 | 909 |
| 60 - 64 | 3,909 | 545 | 10 | 1,415 | 58 | 113 | 140 | 190 | 171 | 242 | 501 | 1,269 | 134 | 406 | 202 | 81 | 446 | 670 |
| 65 - 69 | 2,398 | 421 | 11 | 1,017 | 40 | 117 | 122 | 153 | 112 | 192 | 281 | 638 | 75 | 242 | 75 | 38 | 208 | 311 |
| 70 - 74 | 1,338 | 328 | 8 | 621 | 34 | 67 | 87 | 100 | 85 | 106 | 142 | 264 | 45 | 93 | 21 | 16 | 89 | 117 |
| 75+ | 1,505 | 481 | 8 | 622 | 49 | 83 | 78 | 109 | 72 | 106 | 125 | 288 | 44 | 107 | 30 | 20 | 87 | 106 |
| **Female** | | | | | | | | | | | | | | | | | | |
| **Total** | **227,476** | **28,205** | **538** | **70,090** | **1,643** | **3,585** | **5,065** | **7,690** | **9,566** | **13,371** | **29,170** | **106,490** | **15,140** | **30,015** | **7,121** | **5,490** | **48,724** | **22,153** |
| 12 - 14 | 9,545 | 3,243 | 73 | 5,722 | 218 | 518 | 857 | 1,131 | 1,209 | 1,026 | 763 | 507 | 349 | 141 | 17 | - | - | - |
| 15 - 19 | 42,884 | 5,438 | 106 | 16,141 | 282 | 659 | 1,051 | 1,721 | 2,383 | 3,574 | 6,471 | 20,691 | 4,395 | 5,834 | 1,338 | 1,077 | 8,047 | 508 |
| 20 - 24 | 67,371 | 4,699 | 94 | 13,791 | 256 | 590 | 834 | 1,284 | 1,821 | 2,772 | 6,234 | 42,190 | 4,317 | 9,263 | 2,220 | 2,212 | 24,178 | 6,597 |
| 25 - 29 | 42,305 | 3,773 | 78 | 10,850 | 215 | 489 | 704 | 1,016 | 1,428 | 2,028 | 4,970 | 20,371 | 2,532 | 5,644 | 1,226 | 1,041 | 9,928 | 7,233 |
| 30 - 34 | 23,066 | 2,506 | 49 | 7,186 | 126 | 287 | 387 | 649 | 844 | 1,304 | 3,589 | 9,908 | 1,551 | 3,955 | 679 | 479 | 3,244 | 3,417 |
| 35 - 39 | 14,854 | 1,936 | 36 | 5,416 | 117 | 224 | 304 | 523 | 649 | 952 | 2,647 | 5,694 | 895 | 2,497 | 436 | 287 | 1,579 | 1,772 |
| 40 - 44 | 8,599 | 1,412 | 18 | 3,325 | 63 | 157 | 181 | 337 | 403 | 558 | 1,626 | 2,873 | 470 | 1,163 | 378 | 150 | 712 | 971 |
| 45 - 49 | 6,711 | 1,252 | 23 | 2,754 | 64 | 134 | 218 | 302 | 305 | 434 | 1,297 | 2,025 | 275 | 729 | 433 | 121 | 467 | 657 |
| 50 - 54 | 4,460 | 1,003 | 13 | 1,934 | 75 | 129 | 152 | 269 | 202 | 311 | 796 | 1,121 | 166 | 384 | 242 | 66 | 263 | 389 |
| 55 - 59 | 2,802 | 708 | 12 | 1,172 | 45 | 123 | 116 | 159 | 119 | 182 | 428 | 580 | 96 | 212 | 82 | 28 | 162 | 330 |
| 60 - 64 | 2,012 | 744 | 10 | 834 | 67 | 117 | 114 | 143 | 106 | 108 | 179 | 252 | 45 | 90 | 35 | 16 | 66 | 172 |
| 65 - 69 | 1,253 | 588 | 10 | 465 | 44 | 82 | 73 | 75 | 49 | 62 | 80 | 135 | 27 | 48 | 16 | 6 | 38 | 55 |
| 70 - 74 | 746 | 420 | 7 | 244 | 41 | 38 | 30 | 35 | 19 | 34 | 47 | 47 | 8 | 17 | 6 | 4 | 12 | 28 |
| 75+ | 868 | 483 | 9 | 256 | 30 | 38 | 44 | 46 | 29 | 26 | 43 | 96 | 14 | 38 | 13 | 3 | 28 | 24 |

## Table E9: Usually Unemployed Population (De Facto) 12 Years and Older by Age Group, Sex and Highest Level of Education Completed, Rural, Zambia 2010

| Age Group, Sex and Rural/ Urban | Total | Never Atended School | Total Ever Attended School | | | | | | | | | | | | | | | |
|---|---|---|---|---|---|---|---|---|---|---|---|---|---|---|---|---|---|---|
| | | | Primary School Grade | | | | | | | | | Secondary School Grade | | | | | | Tertiary |
| | | | None | Total 1-7 | 1 | 2 | 3 | 4 | 5 | 6 | 7 | Total 8-12 | 8 | 9 | 10 | 11 | 12 | |
| **Zambia Rural** | | | | | | | | | | | | | | | | | | |
| **Total** | **171,807** | **39,181** | **623** | **72,788** | **2,152** | **4,626** | **6,366** | **9,160** | **10,627** | **13,979** | **25,878** | **54,367** | **11,728** | **17,623** | **3,827** | **2,665** | **18,524** | **4,848** |
| 12 - 14 | 12,458 | 5,263 | 116 | 6,800 | 382 | 754 | 1,234 | 1,453 | 1,377 | 1,041 | 559 | 279 | 183 | 90 | 6 | - | - | - |
| 15 - 19 | 33,617 | 8,023 | 144 | 16,309 | 398 | 940 | 1,382 | 2,066 | 2,667 | 3,600 | 5,256 | 8,994 | 3,207 | 2,867 | 497 | 345 | 2,078 | 147 |
| 20 - 24 | 39,931 | 6,229 | 93 | 13,328 | 277 | 684 | 978 | 1,439 | 1,894 | 2,749 | 5,307 | 19,052 | 3,407 | 5,286 | 985 | 902 | 8,472 | 1,229 |
| 25 - 29 | 26,842 | 5,152 | 73 | 10,059 | 269 | 558 | 765 | 1,137 | 1,451 | 1,942 | 3,937 | 10,343 | 1,851 | 3,061 | 631 | 558 | 4,242 | 1,215 |
| 30 - 34 | 16,982 | 3,527 | 54 | 7,176 | 174 | 391 | 521 | 774 | 998 | 1,344 | 2,974 | 5,573 | 1,178 | 2,217 | 378 | 297 | 1,503 | 652 |
| 35 - 39 | 13,055 | 2,787 | 39 | 5,920 | 157 | 334 | 391 | 632 | 769 | 1,108 | 2,529 | 3,835 | 774 | 1,662 | 290 | 221 | 888 | 474 |
| 40 - 44 | 8,616 | 1,972 | 21 | 4,015 | 100 | 210 | 250 | 447 | 502 | 699 | 1,807 | 2,340 | 468 | 1,027 | 267 | 111 | 467 | 268 |
| 45 - 49 | 6,533 | 1,635 | 20 | 3,072 | 83 | 152 | 220 | 340 | 323 | 514 | 1,440 | 1,574 | 242 | 576 | 325 | 84 | 347 | 232 |
| 50 - 54 | 4,340 | 1,157 | 12 | 2,075 | 66 | 128 | 149 | 245 | 211 | 342 | 934 | 924 | 158 | 292 | 229 | 59 | 186 | 172 |
| 55 - 59 | 2,872 | 755 | 11 | 1,337 | 41 | 109 | 129 | 177 | 128 | 228 | 525 | 607 | 98 | 216 | 114 | 38 | 141 | 162 |
| 60 - 64 | 2,350 | 814 | 7 | 1,017 | 65 | 121 | 115 | 156 | 127 | 156 | 277 | 373 | 68 | 139 | 55 | 22 | 89 | 139 |
| 65 - 69 | 1,745 | 699 | 13 | 727 | 54 | 110 | 95 | 124 | 73 | 118 | 153 | 234 | 37 | 99 | 23 | 16 | 59 | 72 |
| 70 - 74 | 1,111 | 516 | 11 | 445 | 37 | 60 | 66 | 77 | 53 | 62 | 90 | 100 | 23 | 40 | 10 | 6 | 21 | 39 |
| 75+ | 1,355 | 652 | 9 | 508 | 49 | 75 | 71 | 93 | 54 | 76 | 90 | 139 | 34 | 51 | 17 | 6 | 31 | 47 |
| **Male** | | | | | | | | | | | | | | | | | | |
| **Total** | **103,603** | **21,042** | **345** | **43,600** | **1,172** | **2,551** | **3,538** | **5,148** | **6,197** | **8,412** | **16,582** | **35,592** | **7,229** | **11,567** | **2,737** | **1,851** | **12,208** | **3,024** |
| 12 - 14 | 6,451 | 2,811 | 63 | 3,447 | 214 | 390 | 643 | 744 | 674 | 498 | 284 | 130 | 76 | 52 | 2 | - | - | - |
| 15 - 19 | 17,248 | 4,233 | 90 | 8,567 | 212 | 524 | 743 | 1,122 | 1,434 | 1,864 | 2,668 | 4,299 | 1,521 | 1,348 | 251 | 180 | 999 | 59 |
| 20 - 24 | 23,821 | 3,371 | 51 | 7,953 | 146 | 347 | 537 | 813 | 1,124 | 1,642 | 3,344 | 11,788 | 2,140 | 3,324 | 635 | 572 | 5,117 | 658 |
| 25 - 29 | 17,184 | 2,874 | 38 | 6,259 | 148 | 298 | 427 | 639 | 870 | 1,247 | 2,630 | 7,300 | 1,238 | 2,104 | 464 | 422 | 3,072 | 713 |
| 30 - 34 | 11,238 | 2,061 | 30 | 4,665 | 100 | 230 | 311 | 452 | 655 | 901 | 2,016 | 4,075 | 822 | 1,560 | 300 | 229 | 1,164 | 407 |
| 35 - 39 | 8,791 | 1,612 | 16 | 3,886 | 84 | 208 | 228 | 375 | 485 | 730 | 1,776 | 2,967 | 587 | 1,256 | 237 | 169 | 718 | 310 |
| 40 - 44 | 5,893 | 1,099 | 13 | 2,725 | 65 | 119 | 157 | 281 | 318 | 475 | 1,310 | 1,860 | 350 | 809 | 215 | 89 | 397 | 196 |
| 45 - 49 | 4,295 | 874 | 10 | 1,997 | 45 | 87 | 114 | 184 | 195 | 363 | 1,009 | 1,251 | 192 | 448 | 261 | 66 | 284 | 163 |
| 50 - 54 | 2,756 | 547 | 7 | 1,343 | 29 | 73 | 83 | 121 | 126 | 222 | 689 | 718 | 104 | 223 | 185 | 48 | 158 | 141 |
| 55 - 59 | 1,857 | 361 | 6 | 870 | 20 | 54 | 73 | 104 | 92 | 152 | 375 | 497 | 69 | 178 | 98 | 31 | 121 | 123 |
| 60 - 64 | 1,469 | 346 | 4 | 689 | 33 | 65 | 70 | 101 | 90 | 116 | 214 | 312 | 55 | 109 | 50 | 21 | 77 | 118 |
| 65 - 69 | 1,055 | 292 | 7 | 503 | 27 | 68 | 62 | 86 | 49 | 89 | 122 | 193 | 27 | 82 | 16 | 14 | 54 | 60 |
| 70 - 74 | 670 | 225 | 6 | 320 | 18 | 35 | 46 | 55 | 46 | 47 | 73 | 86 | 19 | 36 | 8 | 4 | 19 | 33 |
| 75+ | 875 | 336 | 4 | 376 | 31 | 53 | 44 | 71 | 39 | 66 | 72 | 116 | 29 | 38 | 15 | 6 | 28 | 43 |
| **Female** | | | | | | | | | | | | | | | | | | |
| **Total** | **68,204** | **18,139** | **278** | **29,188** | **980** | **2,075** | **2,828** | **4,012** | **4,430** | **5,567** | **9,296** | **18,775** | **4,499** | **6,056** | **1,090** | **814** | **6,316** | **1,824** |
| 12 - 14 | 6,007 | 2,452 | 53 | 3,353 | 168 | 364 | 591 | 709 | 703 | 543 | 275 | 149 | 107 | 38 | 4 | - | - | - |
| 15 - 19 | 16,369 | 3,790 | 54 | 7,742 | 186 | 416 | 639 | 944 | 1,233 | 1,736 | 2,588 | 4,695 | 1,686 | 1,519 | 246 | 165 | 1,079 | 88 |
| 20 - 24 | 16,110 | 2,858 | 42 | 5,375 | 131 | 337 | 441 | 626 | 770 | 1,107 | 1,963 | 7,264 | 1,267 | 1,962 | 350 | 330 | 3,355 | 571 |
| 25 - 29 | 9,658 | 2,278 | 35 | 3,800 | 121 | 260 | 338 | 498 | 581 | 695 | 1,307 | 3,043 | 613 | 957 | 167 | 136 | 1,170 | 502 |
| 30 - 34 | 5,744 | 1,466 | 24 | 2,511 | 74 | 161 | 210 | 322 | 343 | 443 | 958 | 1,498 | 356 | 657 | 78 | 68 | 339 | 245 |
| 35 - 39 | 4,264 | 1,175 | 23 | 2,034 | 73 | 126 | 163 | 257 | 284 | 378 | 753 | 868 | 187 | 406 | 53 | 52 | 170 | 164 |
| 40 - 44 | 2,723 | 873 | 8 | 1,290 | 35 | 91 | 93 | 166 | 184 | 224 | 497 | 480 | 118 | 218 | 52 | 22 | 70 | 72 |
| 45 - 49 | 2,238 | 761 | 10 | 1,075 | 38 | 65 | 106 | 156 | 128 | 151 | 431 | 323 | 50 | 128 | 64 | 18 | 63 | 69 |
| 50 - 54 | 1,584 | 610 | 5 | 732 | 37 | 55 | 66 | 124 | 85 | 120 | 245 | 206 | 54 | 69 | 44 | 11 | 28 | 31 |
| 55 - 59 | 1,015 | 394 | 5 | 467 | 21 | 55 | 56 | 73 | 36 | 76 | 150 | 110 | 29 | 38 | 16 | 7 | 20 | 39 |
| 60 - 64 | 881 | 468 | 3 | 328 | 32 | 56 | 45 | 55 | 37 | 40 | 63 | 61 | 13 | 30 | 5 | 1 | 12 | 21 |
| 65 - 69 | 690 | 407 | 6 | 224 | 27 | 42 | 33 | 38 | 24 | 29 | 31 | 41 | 10 | 17 | 7 | 2 | 5 | 12 |
| 70 - 74 | 441 | 291 | 5 | 125 | 19 | 25 | 20 | 22 | 7 | 15 | 17 | 14 | 4 | 4 | 2 | 2 | 2 | 6 |
| 75+ | 480 | 316 | 5 | 132 | 18 | 22 | 27 | 22 | 15 | 10 | 18 | 23 | 5 | 13 | 2 | - | 3 | 4 |

## Table E9: Usually Unemployed Population (De Facto) 12 Years and Older by Age Group, Sex and Highest Level of Education Completed, Urban, Zambia 2010

| Age Group, Sex and Rural/ Urban | Total | Never Atended School | Total Ever Attended School | | | | | | | | | | | | | | | |
|---|---|---|---|---|---|---|---|---|---|---|---|---|---|---|---|---|---|---|
| | | | Primary School Grade | | | | | | | | | Secondary School Grade | | | | | | Tertiary |
| | | | None | Total 1-7 | 1 | 2 | 3 | 4 | 5 | 6 | 7 | Total 8-12 | 8 | 9 | 10 | 11 | 12 | |
| **Zambia Urban** | | | | | | | | | | | | | | | | | | |
| **Total** | **371,683** | **21,372** | **529** | **89,296** | **1,330** | **3,103** | **4,620** | **7,621** | **10,693** | **16,678** | **45,251** | **218,494** | **23,732** | **58,311** | **16,006** | **12,437** | **108,008** | **41,992** |
| 12 - 14 | 6,823 | 1,456 | 46 | 4,696 | 106 | 336 | 565 | 807 | 1,012 | 972 | 898 | 625 | 414 | 191 | 20 | - | - | - |
| 15 - 19 | 51,247 | 3,408 | 104 | 17,035 | 193 | 483 | 843 | 1,502 | 2,422 | 3,775 | 7,817 | 30,027 | 5,088 | 8,316 | 2,083 | 1,704 | 12,836 | 673 |
| 20 - 24 | 112,445 | 3,921 | 113 | 18,428 | 258 | 535 | 806 | 1,432 | 2,125 | 3,584 | 9,688 | 79,554 | 6,736 | 16,501 | 4,195 | 4,526 | 47,596 | 10,429 |
| 25 - 29 | 77,311 | 3,476 | 78 | 15,268 | 193 | 467 | 704 | 1,140 | 1,760 | 2,704 | 8,300 | 45,608 | 4,403 | 11,283 | 2,907 | 2,609 | 24,406 | 12,881 |
| 30 - 34 | 44,696 | 2,486 | 50 | 10,867 | 128 | 277 | 392 | 739 | 1,102 | 1,971 | 6,258 | 24,651 | 2,937 | 8,570 | 1,918 | 1,378 | 9,848 | 6,642 |
| 35 - 39 | 29,509 | 1,892 | 41 | 7,907 | 104 | 213 | 296 | 564 | 793 | 1,304 | 4,633 | 15,872 | 1,890 | 6,090 | 1,361 | 884 | 5,647 | 3,797 |
| 40 - 44 | 17,102 | 1,228 | 15 | 4,692 | 55 | 138 | 207 | 349 | 446 | 720 | 2,777 | 8,750 | 987 | 3,246 | 1,006 | 485 | 3,026 | 2,417 |
| 45 - 49 | 12,031 | 972 | 25 | 3,526 | 42 | 123 | 203 | 285 | 310 | 543 | 2,020 | 5,787 | 558 | 1,786 | 1,100 | 350 | 1,993 | 1,721 |
| 50 - 54 | 7,615 | 700 | 11 | 2,438 | 58 | 123 | 151 | 228 | 215 | 355 | 1,308 | 3,297 | 282 | 939 | 725 | 241 | 1,110 | 1,169 |
| 55 - 59 | 5,436 | 504 | 13 | 1,662 | 35 | 119 | 112 | 174 | 172 | 286 | 764 | 2,180 | 207 | 677 | 398 | 126 | 772 | 1,077 |
| 60 - 64 | 3,571 | 475 | 13 | 1,232 | 60 | 109 | 139 | 177 | 150 | 194 | 403 | 1,148 | 111 | 357 | 182 | 75 | 423 | 703 |
| 65 - 69 | 1,906 | 310 | 8 | 755 | 30 | 89 | 100 | 104 | 88 | 136 | 208 | 539 | 65 | 191 | 68 | 28 | 187 | 294 |
| 70 - 74 | 973 | 232 | 4 | 420 | 38 | 45 | 51 | 58 | 51 | 78 | 99 | 211 | 30 | 70 | 17 | 14 | 80 | 106 |
| 75+ | 1,018 | 312 | 8 | 370 | 30 | 46 | 51 | 62 | 47 | 56 | 78 | 245 | 24 | 94 | 26 | 17 | 84 | 83 |
| **Male** | | | | | | | | | | | | | | | | | | |
| **Total** | **212,411** | **11,306** | **269** | **48,394** | **667** | **1,593** | **2,383** | **3,943** | **5,557** | **8,874** | **25,377** | **130,779** | **13,091** | **34,352** | **9,975** | **7,761** | **65,600** | **21,663** |
| 12 - 14 | 3,285 | 665 | 26 | 2,327 | 56 | 182 | 299 | 385 | 506 | 489 | 410 | 267 | 172 | 88 | 7 | - | - | - |
| 15 - 19 | 24,732 | 1,760 | 52 | 8,636 | 97 | 240 | 431 | 725 | 1,272 | 1,937 | 3,934 | 14,031 | 2,379 | 4,001 | 991 | 792 | 5,868 | 253 |
| 20 - 24 | 61,184 | 2,080 | 61 | 10,012 | 133 | 282 | 413 | 774 | 1,074 | 1,919 | 5,417 | 44,628 | 3,686 | 9,200 | 2,325 | 2,644 | 26,773 | 4,403 |
| 25 - 29 | 44,664 | 1,981 | 35 | 8,218 | 99 | 238 | 338 | 622 | 913 | 1,371 | 4,637 | 28,280 | 2,484 | 6,596 | 1,848 | 1,704 | 15,648 | 6,150 |
| 30 - 34 | 27,374 | 1,446 | 25 | 6,192 | 76 | 151 | 215 | 412 | 601 | 1,110 | 3,627 | 16,241 | 1,742 | 5,272 | 1,317 | 967 | 6,943 | 3,470 |
| 35 - 39 | 18,919 | 1,131 | 28 | 4,525 | 60 | 115 | 155 | 298 | 428 | 730 | 2,739 | 11,046 | 1,182 | 3,999 | 978 | 649 | 4,238 | 2,189 |
| 40 - 44 | 11,226 | 689 | 5 | 2,657 | 27 | 72 | 119 | 178 | 227 | 386 | 1,648 | 6,357 | 635 | 2,301 | 680 | 357 | 2,384 | 1,518 |
| 45 - 49 | 7,558 | 481 | 12 | 1,847 | 16 | 54 | 91 | 139 | 133 | 260 | 1,154 | 4,085 | 333 | 1,185 | 731 | 247 | 1,589 | 1,133 |
| 50 - 54 | 4,739 | 307 | 3 | 1,236 | 20 | 49 | 65 | 83 | 98 | 164 | 757 | 2,382 | 170 | 624 | 527 | 186 | 875 | 811 |
| 55 - 59 | 3,649 | 190 | 6 | 957 | 11 | 51 | 52 | 88 | 89 | 180 | 486 | 1,710 | 140 | 503 | 332 | 105 | 630 | 786 |
| 60 - 64 | 2,440 | 199 | 6 | 726 | 25 | 48 | 70 | 89 | 81 | 126 | 287 | 957 | 79 | 297 | 152 | 60 | 369 | 552 |
| 65 - 69 | 1,343 | 129 | 4 | 514 | 13 | 49 | 60 | 67 | 63 | 103 | 159 | 445 | 48 | 160 | 59 | 24 | 154 | 251 |
| 70 - 74 | 668 | 103 | 2 | 301 | 16 | 32 | 41 | 45 | 39 | 59 | 69 | 178 | 26 | 57 | 13 | 12 | 70 | 84 |
| 75+ | 630 | 145 | 4 | 246 | 18 | 30 | 34 | 38 | 33 | 40 | 53 | 172 | 15 | 69 | 15 | 14 | 59 | 63 |
| **Female** | | | | | | | | | | | | | | | | | | |
| **Total** | **159,272** | **10,066** | **260** | **40,902** | **663** | **1,510** | **2,237** | **3,678** | **5,136** | **7,804** | **19,874** | **87,715** | **10,641** | **23,959** | **6,031** | **4,676** | **42,408** | **20,329** |
| 12 - 14 | 3,538 | 791 | 20 | 2,369 | 50 | 154 | 266 | 422 | 506 | 483 | 488 | 358 | 242 | 103 | 13 | - | - | - |
| 15 - 19 | 26,515 | 1,648 | 52 | 8,399 | 96 | 243 | 412 | 777 | 1,150 | 1,838 | 3,883 | 15,996 | 2,709 | 4,315 | 1,092 | 912 | 6,968 | 420 |
| 20 - 24 | 51,261 | 1,841 | 52 | 8,416 | 125 | 253 | 393 | 658 | 1,051 | 1,665 | 4,271 | 34,926 | 3,050 | 7,301 | 1,870 | 1,882 | 20,823 | 6,026 |
| 25 - 29 | 32,647 | 1,495 | 43 | 7,050 | 94 | 229 | 366 | 518 | 847 | 1,333 | 3,663 | 17,328 | 1,919 | 4,687 | 1,059 | 905 | 8,758 | 6,731 |
| 30 - 34 | 17,322 | 1,040 | 25 | 4,675 | 52 | 126 | 177 | 327 | 501 | 861 | 2,631 | 8,410 | 1,195 | 3,298 | 601 | 411 | 2,905 | 3,172 |
| 35 - 39 | 10,590 | 761 | 13 | 3,382 | 44 | 98 | 141 | 266 | 365 | 574 | 1,894 | 4,826 | 708 | 2,091 | 383 | 235 | 1,409 | 1,608 |
| 40 - 44 | 5,876 | 539 | 10 | 2,035 | 28 | 66 | 88 | 171 | 219 | 334 | 1,129 | 2,393 | 352 | 945 | 326 | 128 | 642 | 899 |
| 45 - 49 | 4,473 | 491 | 13 | 1,679 | 26 | 69 | 112 | 146 | 177 | 283 | 866 | 1,702 | 225 | 601 | 369 | 103 | 404 | 588 |
| 50 - 54 | 2,876 | 393 | 8 | 1,202 | 38 | 74 | 86 | 145 | 117 | 191 | 551 | 915 | 112 | 315 | 198 | 55 | 235 | 358 |
| 55 - 59 | 1,787 | 314 | 7 | 705 | 24 | 68 | 60 | 86 | 83 | 106 | 278 | 470 | 67 | 174 | 66 | 21 | 142 | 291 |
| 60 - 64 | 1,131 | 276 | 7 | 506 | 35 | 61 | 69 | 88 | 69 | 68 | 116 | 191 | 32 | 60 | 30 | 15 | 54 | 151 |
| 65 - 69 | 563 | 181 | 4 | 241 | 17 | 40 | 40 | 37 | 25 | 33 | 49 | 94 | 17 | 31 | 9 | 4 | 33 | 43 |
| 70 - 74 | 305 | 129 | 2 | 119 | 22 | 13 | 10 | 13 | 12 | 19 | 30 | 33 | 4 | 13 | 4 | 2 | 10 | 22 |
| 75+ | 388 | 167 | 4 | 124 | 12 | 16 | 17 | 24 | 14 | 16 | 25 | 73 | 9 | 25 | 11 | 3 | 25 | 20 |

## Table E10: Usually Working Population (De Facto) 12 Years and Older by Employment Status, Sex and Highest Professional/Vocational Qualification Completed, Rural/Urban, Zambia 2010

| Employment Status, Sex and Rural/Urban | Usually Working Population | Highest Profession/Vocational Qualification | | | | | |
|---|---|---|---|---|---|---|---|
| | | None | Certificate | Diploma | Bachelor's Degree | Master's Degree | PhD |
| **Zambia Total** | | | | | | | |
| **Total** | **3,887,052** | **3,455,191** | **253,164** | **133,547** | **35,223** | **8,956** | **971** |
| Employer | 29,033 | 17,494 | 5,532 | 3,900 | 1,590 | 467 | 50 |
| Employee | 864,942 | 544,060 | 173,498 | 110,039 | 29,112 | 7,453 | 780 |
| Self Employed | 1,715,082 | 1,629,821 | 62,132 | 17,836 | 4,197 | 965 | 131 |
| Unpaid family worker | 1,277,995 | 1,263,816 | 12,002 | 1,772 | 324 | 71 | 10 |
| **Male** | | | | | | | |
| **Total** | **2,140,733** | **1,855,705** | **165,089** | **86,306** | **26,161** | **6,664** | **808** |
| Employer | 21,227 | 13,040 | 3,748 | 2,750 | 1,277 | 366 | 46 |
| Employee | 629,825 | 422,897 | 110,604 | 68,981 | 21,237 | 5,451 | 655 |
| Self Employed | 1,004,229 | 942,916 | 43,652 | 13,357 | 3,412 | 792 | 100 |
| Unpaid family worker | 485,452 | 476,852 | 7,085 | 1,218 | 235 | 55 | 7 |
| **Female** | | | | | | | |
| **Total** | **1,746,319** | **1,599,486** | **88,075** | **47,241** | **9,062** | **2,292** | **163** |
| Employer | 7,806 | 4,454 | 1,784 | 1,150 | 313 | 101 | 4 |
| Employee | 235,117 | 121,163 | 62,894 | 41,058 | 7,875 | 2,002 | 125 |
| Self Employed | 710,853 | 686,905 | 18,480 | 4,479 | 785 | 173 | 31 |
| Unpaid family worker | 792,543 | 786,964 | 4,917 | 554 | 89 | 16 | 3 |
| **Zambia Rural** | | | | | | | |
| **Total** | **2,624,368** | **2,530,003** | **68,314** | **21,803** | **3,251** | **862** | **135** |
| Employer | 9,434 | 6,423 | 1,837 | 844 | 246 | 74 | 10 |
| Employee | 194,631 | 135,896 | 38,197 | 17,412 | 2,394 | 638 | 94 |
| Self Employed | 1,241,111 | 1,218,106 | 19,649 | 2,723 | 491 | 120 | 22 |
| Unpaid family worker | 1,179,192 | 1,169,578 | 8,631 | 824 | 120 | 30 | 9 |
| **Male** | | | | | | | |
| **Total** | **1,341,036** | **1,273,726** | **48,079** | **15,970** | **2,490** | **657** | **114** |
| Employer | 7,043 | 4,852 | 1,304 | 641 | 182 | 55 | 9 |
| Employee | 148,546 | 108,081 | 25,653 | 12,441 | 1,811 | 478 | 82 |
| Self Employed | 738,866 | 720,274 | 15,794 | 2,272 | 411 | 99 | 16 |
| Unpaid family worker | 446,581 | 440,519 | 5,328 | 616 | 86 | 25 | 7 |
| **Female** | | | | | | | |
| **Total** | **1,283,332** | **1,256,277** | **20,235** | **5,833** | **761** | **205** | **21** |
| Employer | 2,391 | 1,571 | 533 | 203 | 64 | 19 | 1 |
| Employee | 46,085 | 27,815 | 12,544 | 4,971 | 583 | 160 | 12 |
| Self Employed | 502,245 | 497,832 | 3,855 | 451 | 80 | 21 | 6 |
| Unpaid family worker | 732,611 | 729,059 | 3,303 | 208 | 34 | 5 | 2 |

## Table E10: Usually Working Population (De Facto) 12 Years and Older by Employment Status, Sex and Highest Professional/Vocational Qualification Completed, Rural/Urban, Zambia 2010

| Employment Status, Sex and Rural/Urban | Usually Working Population | Highest Profession/Vocational Qualification | | | | | |
|---|---|---|---|---|---|---|---|
| | | None | Certificate | Diploma | Bachelor's Degree | Master's Degree | PhD |
| **Zambia Urban** | | | | | | | |
| **Total** | **1,262,684** | **925,188** | **184,850** | **111,744** | **31,972** | **8,094** | **836** |
| Employer | 19,599 | 11,071 | 3,695 | 3,056 | 1,344 | 393 | 40 |
| Employee | 670,311 | 408,164 | 135,301 | 92,627 | 26,718 | 6,815 | 686 |
| Self Employed | 473,971 | 411,715 | 42,483 | 15,113 | 3,706 | 845 | 109 |
| Unpaid Family Worker | 98,803 | 94,238 | 3,371 | 948 | 204 | 41 | 1 |
| **Male** | | | | | | | |
| **Total** | **799,697** | **581,979** | **117,010** | **70,336** | **23,671** | **6,007** | **694** |
| Employer | 14,184 | 8,188 | 2,444 | 2,109 | 1,095 | 311 | 37 |
| Employee | 481,279 | 314,816 | 84,951 | 56,540 | 19,426 | 4,973 | 573 |
| Self Employed | 265,363 | 222,642 | 27,858 | 11,085 | 3,001 | 693 | 84 |
| Unpaid Family Worker | 38,871 | 36,333 | 1,757 | 602 | 149 | 30 | - |
| **Female** | | | | | | | |
| **Total** | **462,987** | **343,209** | **67,840** | **41,408** | **8,301** | **2,087** | **142** |
| Employer | 5,415 | 2,883 | 1,251 | 947 | 249 | 82 | 3 |
| Employee | 189,032 | 93,348 | 50,350 | 36,087 | 7,292 | 1,842 | 113 |
| Self Employed | 208,608 | 189,073 | 14,625 | 4,028 | 705 | 152 | 25 |
| Unpaid Family Worker | 59,932 | 57,905 | 1,614 | 346 | 55 | 11 | 1 |

## Table E11: Usually Working Population (De Facto) 12 Years and Older by Field of Study, Sex and Highest Professional/Vocational Qualification Completed, Rural/Urban, Zambia 2010

| Field of Study, Sex and Rural/Urban | Usually Working Population | Highest Profession/Vocational Qualification | | | | | |
|---|---|---|---|---|---|---|---|
| | | None | Certificate | Diploma | Bachelor's Degree | Master's Degree | PhD |
| **Zambia Total** | **3,885,049** | **3,455,191** | **252,186** | **132,796** | **35,004** | **8,902** | **970** |
| Natural Science | 3,246 | - | 731 | 1,031 | 1,058 | 335 | 91 |
| Civil engineering | 4,429 | - | 1,903 | 1,238 | 1,059 | 213 | 16 |
| Electrical and Electronics Engineering | 19,288 | 248 | 12,317 | 5,191 | 1,311 | 205 | 16 |
| Mechanical Engineering | 25,198 | 467 | 17,445 | 5,770 | 1,301 | 191 | 24 |
| Chemical Engineering | 1,192 | - | 437 | 349 | 337 | 65 | 4 |
| Mining Engineering | 3,664 | 176 | 1,946 | 902 | 534 | 97 | 9 |
| Industrial Engineering | 1,537 | - | 1,128 | 304 | 82 | 23 | - |
| Metallurgical Engineering | 1,253 | - | 628 | 307 | 278 | 31 | 9 |
| Architectural and Town Planning Engineering | 1,392 | - | 584 | 348 | 368 | 84 | 8 |
| Other Engineering | 6,701 | 127 | 4,311 | 1,641 | 479 | 124 | 19 |
| Medicine and Surgery | 3,447 | - | 469 | 1,210 | 1,121 | 507 | 140 |
| Pharmacy | 2,718 | - | 1,211 | 1,092 | 343 | 67 | 5 |
| Dentistry | 991 | - | 541 | 343 | 86 | 20 | 1 |
| Nursing | 20,950 | - | 12,792 | 7,660 | 393 | 99 | 6 |
| Medical Technology | 2,565 | - | 601 | 1,358 | 462 | 122 | 22 |
| X-Ray Technology | 363 | - | 88 | 163 | 44 | 60 | 8 |
| Veterinary | 1,288 | - | 757 | 294 | 147 | 67 | 23 |
| Statistics | 445 | - | 149 | 137 | 118 | 34 | 7 |
| Mathematics | 934 | - | 180 | 453 | 254 | 39 | 8 |
| Computer Science/Economics | 13,201 | - | 5,870 | 4,116 | 2,389 | 746 | 80 |
| Accountancy | 30,970 | - | 10,064 | 15,500 | 4,491 | 852 | 63 |
| Teacher Training | 104,929 | 1,432 | 60,796 | 36,255 | 5,515 | 863 | 68 |
| Law and Jurisprudence | 6,418 | - | 2,717 | 1,958 | 1,411 | 305 | 27 |
| Journalism | 2,298 | - | 662 | 1,381 | 194 | 54 | 7 |
| Fine Arts | 1,938 | 71 | 1,175 | 353 | 308 | 29 | 2 |
| Physical Education | 705 | 26 | 350 | 213 | 86 | 26 | 4 |
| Library Science | 869 | - | 374 | 296 | 164 | 31 | 4 |
| Social Welfare | 6,645 | 91 | 3,078 | 2,619 | 689 | 158 | 10 |
| Criminology | 3,253 | - | 2,814 | 383 | 42 | 12 | 2 |
| Business Administration and Related Programmes | 28,130 | - | 8,909 | 13,904 | 3,782 | 1,490 | 45 |
| Secretarial Training | 10,259 | - | 7,524 | 2,600 | 115 | 18 | 2 |
| Shorthand Typing | 1,904 | 59 | 1,581 | 238 | 19 | 6 | 1 |
| Clerical Typing | 1,757 | 78 | 1,452 | 210 | 14 | 3 | - |
| Operating of Office Machines | 1,003 | 32 | 753 | 193 | 19 | 6 | - |
| Service Trade | 8,845 | 317 | 7,121 | 1,311 | 82 | 12 | 2 |
| Radio and Television Broadcasting | 600 | 9 | 271 | 219 | 84 | 17 | - |
| Fire Protection and Fire Fighting | 928 | 142 | 663 | 111 | 6 | 6 | - |
| Agriculture Forestry and Fishery | 12,601 | 909 | 7,522 | 2,890 | 896 | 334 | 50 |
| Food and Drink Processing | 4,430 | 164 | 3,596 | 603 | 52 | 14 | 1 |
| Wood Working | 8,948 | 526 | 8,064 | 319 | 31 | 8 | - |
| Textile Trades | 5,732 | 287 | 5,233 | 186 | 23 | 3 | - |
| Leather Trades | 520 | 35 | 435 | 37 | 8 | 5 | - |
| Other Programmes | 81,172 | 4,925 | 52,621 | 17,110 | 4,809 | 1,521 | 186 |
| None | 3,445,393 | 3,445,070 | 323 | - | - | - | - |

## Table E11: Usually Working Population (De Facto) 12 Years and Older by Field of Study, Sex and Highest Professional/Vocational Qualification Completed, Rural/Urban, Zambia 2010

| Field of Study, Sex and Rural/Urban | Usually Working Population | Highest Profession/Vocational Qualification | | | | | |
|---|---|---|---|---|---|---|---|
| | | None | Certificate | Diploma | Bachelor's Degree | Master's Degree | PhD |
| **Male Total** | **2,139,473** | **1,855,705** | **164,576** | **85,781** | **25,985** | **6,619** | **807** |
| Natural Science | 2,562 | - | 623 | 783 | 818 | 256 | 82 |
| Civil engineering | 4,237 | - | 1,827 | 1,178 | 1,011 | 205 | 16 |
| Electrical and Electronics Engineering | 18,294 | 221 | 11,674 | 4,935 | 1,254 | 195 | 15 |
| Mechanical Engineering | 24,711 | 452 | 17,095 | 5,669 | 1,283 | 188 | 24 |
| Chemical Engineering | 1,110 | - | 418 | 329 | 305 | 54 | 4 |
| Mining Engineering | 3,541 | 146 | 1,890 | 881 | 519 | 96 | 9 |
| Industrial Engineering | 1,405 | - | 1,015 | 291 | 79 | 20 | - |
| Metallurgical Engineering | 1,208 | - | 601 | 303 | 268 | 28 | 8 |
| Architectural and Town Planning Engineering | 1,204 | - | 486 | 324 | 314 | 72 | 8 |
| Other Engineering | 6,445 | 119 | 4,178 | 1,566 | 445 | 118 | 19 |
| Medicine and Surgery | 2,691 | - | 392 | 970 | 854 | 371 | 104 |
| Pharmacy | 1,816 | - | 825 | 717 | 231 | 40 | 3 |
| Dentistry | 709 | - | 394 | 234 | 65 | 15 | 1 |
| Nursing | 7,777 | - | 5,162 | 2,491 | 99 | 22 | 3 |
| Medical Technology | 1,956 | - | 451 | 1,043 | 364 | 78 | 20 |
| X-Ray Technology | 256 | - | 66 | 108 | 34 | 43 | 5 |
| Veterinary | 1,132 | - | 685 | 250 | 120 | 57 | 20 |
| Statistics | 359 | - | 121 | 108 | 95 | 28 | 7 |
| Mathematics | 735 | - | 125 | 360 | 215 | 27 | 8 |
| Computer Science/Economics | 8,757 | - | 3,392 | 2,871 | 1,846 | 579 | 69 |
| Accountancy | 23,314 | - | 7,617 | 11,553 | 3,423 | 669 | 52 |
| Teacher Training | 50,783 | 704 | 26,783 | 19,152 | 3,515 | 573 | 56 |
| Law and Jurisprudence | 4,936 | - | 2,231 | 1,486 | 1,002 | 198 | 19 |
| Journalism | 1,335 | - | 428 | 760 | 101 | 40 | 6 |
| Fine Arts | 1,437 | 50 | 903 | 279 | 186 | 17 | 2 |
| Physical Education | 442 | 18 | 206 | 137 | 60 | 18 | 3 |
| Library Science | 452 | - | 210 | 144 | 79 | 16 | 3 |
| Social Welfare | 3,121 | 54 | 1,461 | 1,122 | 385 | 91 | 8 |
| Criminology | 2,759 | - | 2,378 | 333 | 37 | 9 | 2 |
| Business Administration and Related Programmes | 18,630 | - | 6,051 | 8,855 | 2,572 | 1,119 | 33 |
| Secretarial Training | 877 | - | 655 | 195 | 19 | 6 | 2 |
| Shorthand Typing | 578 | 17 | 442 | 105 | 10 | 4 | - |
| Clerical Typing | 805 | 40 | 630 | 122 | 10 | 3 | - |
| Operating of Office Machines | 789 | 24 | 594 | 149 | 18 | 4 | - |
| Service Trade | 3,909 | 149 | 3,175 | 540 | 38 | 6 | 1 |
| Radio and Television Broadcasting | 466 | 7 | 215 | 169 | 62 | 13 | - |
| Fire Protection and Fire Fighting | 799 | 82 | 604 | 101 | 6 | 6 | - |
| Agriculture Forestry and Fishery | 10,549 | 627 | 6,343 | 2,508 | 753 | 275 | 43 |
| Food and Drink Processing | 1,946 | 86 | 1,569 | 260 | 24 | 7 | - |
| Wood Working | 8,659 | 507 | 7,838 | 280 | 28 | 6 | - |
| Textile Trades | 1,412 | 77 | 1,241 | 78 | 15 | 1 | - |
| Leather Trades | 246 | 17 | 192 | 25 | 7 | 5 | - |
| Other Programmes | 61,629 | 3,747 | 41,256 | 12,017 | 3,416 | 1,041 | 152 |
| None | 1,848,695 | 1,848,561 | 134 | - | - | - | - |

## Table E11: Usually Working Population (De Facto) 12 Years and Older by Field of Study, Sex and Highest Professional/Vocational Qualification Completed, Rural/Urban, Zambia 2010

| Field of Study, Sex and Rural/Urban | Usually Working Population | Highest Profession/Vocational Qualification | | | | | |
|---|---|---|---|---|---|---|---|
| | | None | Certificate | Diploma | Bachelor's Degree | Master's Degree | PhD |
| **Female Total** | **1,745,576** | **1,599,486** | **87,610** | **47,015** | **9,019** | **2,283** | **163** |
| Natural Science | 684 | - | 108 | 248 | 240 | 79 | 9 |
| Civil engineering | 192 | - | 76 | 60 | 48 | 8 | - |
| Electrical and Electronics Engineering | 994 | 27 | 643 | 256 | 57 | 10 | 1 |
| Mechanical Engineering | 487 | 15 | 350 | 101 | 18 | 3 | - |
| Chemical Engineering | 82 | - | 19 | 20 | 32 | 11 | - |
| Mining Engineering | 123 | 30 | 56 | 21 | 15 | 1 | - |
| Industrial Engineering | 132 | - | 113 | 13 | 3 | 3 | - |
| Metallurgical Engineering | 45 | - | 27 | 4 | 10 | 3 | 1 |
| Architectural and Town Planning Engineering | 188 | - | 98 | 24 | 54 | 12 | - |
| Other Engineering | 256 | 8 | 133 | 75 | 34 | 6 | - |
| Medicine and Surgery | 756 | - | 77 | 240 | 267 | 136 | 36 |
| Pharmacy | 902 | - | 386 | 375 | 112 | 27 | 2 |
| Dentistry | 282 | - | 147 | 109 | 21 | 5 | - |
| Nursing | 13,173 | - | 7,630 | 5,169 | 294 | 77 | 3 |
| Medical Technology | 609 | - | 150 | 315 | 98 | 44 | 2 |
| X-Ray Technology | 107 | - | 22 | 55 | 10 | 17 | 3 |
| Veterinary | 156 | - | 72 | 44 | 27 | 10 | 3 |
| Statistics | 86 | - | 28 | 29 | 23 | 6 | - |
| Mathematics | 199 | - | 55 | 93 | 39 | 12 | - |
| Computer Science/Economics | 4,444 | - | 2,478 | 1,245 | 543 | 167 | 11 |
| Accountancy | 7,656 | - | 2,447 | 3,947 | 1,068 | 183 | 11 |
| Teacher Training | 54,146 | 728 | 34,013 | 17,103 | 2,000 | 290 | 12 |
| Law and Jurisprudence | 1,482 | - | 486 | 472 | 409 | 107 | 8 |
| Journalism | 963 | - | 234 | 621 | 93 | 14 | 1 |
| Fine Arts | 501 | 21 | 272 | 74 | 122 | 12 | - |
| Physical Education | 263 | 8 | 144 | 76 | 26 | 8 | 1 |
| Library Science | 417 | - | 164 | 152 | 85 | 15 | 1 |
| Social Welfare | 3,524 | 37 | 1,617 | 1,497 | 304 | 67 | 2 |
| Criminology | 494 | - | 436 | 50 | 5 | 3 | - |
| Business Administration and Related Programmes | 9,500 | - | 2,858 | 5,049 | 1,210 | 371 | 12 |
| Secretarial Training | 9,382 | - | 6,869 | 2,405 | 96 | 12 | - |
| Shorthand Typing | 1,326 | 42 | 1,139 | 133 | 9 | 2 | 1 |
| Clerical Typing | 952 | 38 | 822 | 88 | 4 | - | - |
| Operating of Office Machines | 214 | 8 | 159 | 44 | 1 | 2 | - |
| Service Trade | 4,936 | 168 | 3,946 | 771 | 44 | 6 | 1 |
| Radio and Television Broadcasting | 134 | 2 | 56 | 50 | 22 | 4 | - |
| Fire Protection and Fire Fighting | 129 | 60 | 59 | 10 | - | - | - |
| Agriculture Forestry and Fishery | 2,052 | 282 | 1,179 | 382 | 143 | 59 | 7 |
| Food and Drink Processing | 2,484 | 78 | 2,027 | 343 | 28 | 7 | 1 |
| Wood Working | 289 | 19 | 226 | 39 | 3 | 2 | - |
| Textile Trades | 4,320 | 210 | 3,992 | 108 | 8 | 2 | - |
| Leather Trades | 274 | 18 | 243 | 12 | 1 | - | - |
| Other Programmes | 19,543 | 1,178 | 11,365 | 5,093 | 1,393 | 480 | 34 |
| None | 1,596,698 | 1,596,509 | 189 | - | - | - | - |

## Table E11: Usually Working Population (De Facto) 12 Years and Older by Field of Study, Sex and Highest Professional/Vocational Qualification Completed, Rural, Zambia 2010

| Field of Study, Sex and Rural/Urban | Usually Working Population | Highest Profession/Vocational Qualification | | | | | |
|---|---|---|---|---|---|---|---|
| | | None | Certificate | Diploma | Bachelor's Degree | Master's Degree | PhD |
| **Zambia Rural** | **2,624,232** | **2,530,003** | **68,233** | **21,757** | **3,246** | **858** | **135** |
| Natural science | 724 | - | 333 | 229 | 110 | 43 | 9 |
| Civil engineering | 719 | - | 517 | 110 | 77 | 15 | - |
| Electrical and electronics engineering | 1,612 | 50 | 1,156 | 338 | 53 | 14 | 1 |
| Mechanical engineering | 2,872 | 109 | 2,243 | 426 | 83 | 10 | 1 |
| Chemical engineering | 149 | - | 107 | 18 | 19 | 5 | - |
| Mining engineering | 618 | 50 | 469 | 78 | 17 | 4 | - |
| Industrial engineering | 358 | - | 319 | 27 | 9 | 3 | - |
| Metallurgical engineering | 159 | - | 117 | 28 | 10 | 4 | - |
| Architectural and town planning engineering | 299 | - | 255 | 27 | 9 | 7 | 1 |
| Other engineering | 761 | 38 | 574 | 105 | 32 | 9 | 3 |
| Medicine and surgery | 517 | - | 169 | 212 | 83 | 30 | 23 |
| Pharmacy | 765 | - | 603 | 138 | 18 | 6 | - |
| Dentistry | 291 | - | 240 | 44 | 5 | 2 | - |
| Nursing | 7,303 | - | 5,499 | 1,730 | 58 | 14 | 2 |
| Medical technology | 772 | - | 248 | 376 | 126 | 19 | 3 |
| X-Ray technology | 74 | - | 26 | 20 | 6 | 18 | 4 |
| Veterinary | 628 | - | 476 | 104 | 26 | 17 | 5 |
| Statistics | 72 | - | 41 | 16 | 6 | 8 | 1 |
| Mathematics | 338 | - | 96 | 197 | 41 | 3 | 1 |
| Computer science/Economics | 1,256 | - | 775 | 260 | 145 | 63 | 13 |
| Accountancy | 2,713 | - | 1,374 | 1,024 | 251 | 58 | 6 |
| Teacher training | 40,573 | 609 | 27,256 | 11,557 | 1,016 | 128 | 7 |
| Law and jurisprudence | 1,084 | - | 758 | 193 | 102 | 28 | 3 |
| Journalism | 210 | - | 105 | 89 | 10 | 5 | 1 |
| Fine arts | 342 | 13 | 245 | 55 | 27 | 2 | - |
| Physical education | 253 | 9 | 160 | 68 | 13 | 3 | - |
| Library science | 159 | - | 102 | 41 | 13 | 3 | - |
| Social welfare | 1,144 | 34 | 751 | 279 | 55 | 23 | 2 |
| Criminology | 710 | - | 648 | 52 | 8 | 2 | - |
| Business administration and related programmes | 2,121 | - | 1,021 | 810 | 195 | 90 | 5 |
| Secretarial training | 1,109 | - | 891 | 209 | 6 | 3 | - |
| shorthand typing | 641 | 26 | 527 | 80 | 6 | 2 | - |
| Clerical Typing | 489 | 25 | 422 | 41 | - | 1 | - |
| Operating of office machines | 189 | 9 | 162 | 16 | 2 | - | - |
| Service trade | 929 | 52 | 777 | 91 | 6 | 2 | 1 |
| Radio and television broadcasting | 101 | 5 | 68 | 20 | 7 | 1 | - |
| Fire protection and fire fighting | 209 | 84 | 119 | 6 | - | - | - |
| Agriculture forestry and fishery | 6,143 | 641 | 4,156 | 994 | 237 | 102 | 13 |
| Food and drinks processing trades programmes | 431 | 28 | 351 | 46 | 5 | 1 | - |
| Wood working | 2,645 | 176 | 2,389 | 73 | 6 | 1 | - |
| Textile trades | 1,007 | 57 | 922 | 24 | 4 | - | - |
| Leather trades | 124 | 14 | 102 | 6 | 1 | 1 | - |
| Other programmes | 13,681 | 1,309 | 10,391 | 1,500 | 343 | 108 | 30 |
| None | 2,526,938 | 2,526,665 | 273 | - | - | - | - |

## Table E11: Usually Working Population (De Facto) 12 Years and Older by Field of Study, Sex and Highest Professional/Vocational Qualification Completed, Rural, Zambia 2010

| Field of Study, Sex and Rural/Urban | Usually Working Population | Highest Profession/Vocational Qualification | | | | | |
|---|---|---|---|---|---|---|---|
| | | None | Certificate | Diploma | Bachelor's Degree | Master's Degree | PhD |
| **Male Rural** | **1,340,950** | **1,273,726** | **48,032** | **15,939** | **2,485** | **654** | **114** |
| Natural science | 603 | - | 294 | 188 | 81 | 31 | 9 |
| Civil engineering | 680 | - | 482 | 107 | 76 | 15 | - |
| Electrical and electronics engineering | 1,533 | 35 | 1,114 | 319 | 50 | 14 | 1 |
| Mechanical engineering | 2,810 | 102 | 2,197 | 417 | 83 | 10 | 1 |
| Chemical engineering | 134 | - | 96 | 17 | 17 | 4 | - |
| Mining engineering | 587 | 29 | 462 | 76 | 16 | 4 | - |
| Industrial engineering | 266 | - | 232 | 23 | 8 | 3 | - |
| Metallurgical engineering | 145 | - | 103 | 28 | 10 | 4 | - |
| Architectural and town planning engineering | 205 | - | 170 | 24 | 5 | 5 | 1 |
| Other engineering | 738 | 34 | 560 | 100 | 32 | 9 | 3 |
| Medicine and surgery | 446 | - | 151 | 190 | 69 | 22 | 14 |
| Pharmacy | 554 | - | 436 | 103 | 10 | 5 | - |
| Dentistry | 221 | - | 181 | 34 | 4 | 2 | - |
| Nursing | 4,239 | - | 3,130 | 1,080 | 21 | 6 | 2 |
| Medical technology | 648 | - | 207 | 324 | 100 | 15 | 2 |
| X-Ray technology | 59 | - | 23 | 16 | 5 | 13 | 2 |
| Veterinary | 584 | - | 454 | 91 | 21 | 15 | 3 |
| Statistics | 60 | - | 36 | 12 | 4 | 7 | 1 |
| Mathematics | 277 | - | 66 | 174 | 33 | 3 | 1 |
| Computer science/Economics | 808 | - | 456 | 179 | 110 | 51 | 12 |
| Accountancy | 2,201 | - | 1,118 | 836 | 190 | 51 | 6 |
| Teacher training | 25,758 | 368 | 16,469 | 8,066 | 762 | 86 | 7 |
| Law and jurisprudence | 926 | - | 666 | 157 | 82 | 20 | 1 |
| Journalism | 152 | - | 74 | 66 | 6 | 5 | 1 |
| Fine arts | 267 | 9 | 198 | 45 | 15 | - | - |
| Physical education | 170 | 7 | 101 | 48 | 13 | 1 | - |
| Library science | 106 | - | 72 | 26 | 7 | 1 | - |
| Social welfare | 709 | 21 | 477 | 168 | 27 | 14 | 2 |
| Criminology | 666 | - | 612 | 47 | 6 | 1 | - |
| Business administration and related programmes | 1,553 | - | 778 | 579 | 131 | 61 | 4 |
| Secretarial training | 237 | - | 195 | 35 | 4 | 3 | - |
| shorthand typing | 283 | 12 | 211 | 55 | 3 | 2 | - |
| Clerical Typing | 294 | 14 | 247 | 32 | - | 1 | - |
| Operating of office machines | 162 | 8 | 138 | 14 | 2 | - | - |
| Service trade | 513 | 32 | 427 | 49 | 3 | 1 | 1 |
| Radio and television broadcasting | 85 | 5 | 56 | 16 | 7 | 1 | - |
| Fire protection and fire fighting | 155 | 40 | 109 | 6 | - | - | - |
| Agriculture forestry and fishery | 5,234 | 430 | 3,612 | 896 | 200 | 86 | 10 |
| Food and drinks processing trades programmes | 241 | 21 | 199 | 18 | 3 | - | - |
| Wood working | 2,557 | 167 | 2,319 | 65 | 5 | 1 | - |
| Textile trades | 306 | 21 | 274 | 8 | 3 | - | - |
| Leather trades | 81 | 10 | 64 | 5 | 1 | 1 | - |
| Other programmes | 11,235 | 1,009 | 8,656 | 1,200 | 260 | 80 | 30 |
| None | 1,271,462 | 1,271,352 | 110 | - | - | - | - |

## Table E11: Usually Working Population (De Facto) 12 Years and Older by Field of Study, Sex and Highest Professional/Vocational Qualification Completed, Rural, Zambia 2010

| Field of Study/Sex/Rural/Urban | Usually Working Population | Highest Profession/Vocational Qualification | | | | | |
|---|---|---|---|---|---|---|---|
| | | None | Certificate | Diploma | Bachelor's Degree | Master's Degree | PhD |
| **Female Rural** | **1,283,282** | **1,256,277** | **20,201** | **5,818** | **761** | **204** | **21** |
| Natural science | 121 | - | 39 | 41 | 29 | 12 | - |
| Civil engineering | 39 | - | 35 | 3 | 1 | - | - |
| Electrical and electronics engineering | 79 | 15 | 42 | 19 | 3 | - | - |
| Mechanical engineering | 62 | 7 | 46 | 9 | - | - | - |
| Chemical engineering | 15 | - | 11 | 1 | 2 | 1 | - |
| Mining engineering | 31 | 21 | 7 | 2 | 1 | - | - |
| Industrial engineering | 92 | - | 87 | 4 | 1 | - | - |
| Metallurgical engineering | 14 | - | 14 | - | - | - | - |
| Architectural and town planning engineering | 94 | - | 85 | 3 | 4 | 2 | - |
| Other engineering | 23 | 4 | 14 | 5 | - | - | - |
| Medicine and surgery | 71 | - | 18 | 22 | 14 | 8 | 9 |
| Pharmacy | 211 | - | 167 | 35 | 8 | 1 | - |
| Dentistry | 70 | - | 59 | 10 | 1 | - | - |
| Nursing | 3,064 | - | 2,369 | 650 | 37 | 8 | - |
| Medical technology | 124 | - | 41 | 52 | 26 | 4 | 1 |
| X-Ray technology | 15 | - | 3 | 4 | 1 | 5 | 2 |
| Veterinary | 44 | - | 22 | 13 | 5 | 2 | 2 |
| Statistics | 12 | - | 5 | 4 | 2 | 1 | - |
| Mathematics | 61 | - | 30 | 23 | 8 | - | - |
| Computer science/Economics | 448 | - | 319 | 81 | 35 | 12 | 1 |
| Accountancy | 512 | - | 256 | 188 | 61 | 7 | - |
| Teacher training | 14,815 | 241 | 10,787 | 3,491 | 254 | 42 | - |
| Law and jurisprudence | 158 | - | 92 | 36 | 20 | 8 | 2 |
| Journalism | 58 | - | 31 | 23 | 4 | - | - |
| Fine arts | 75 | 4 | 47 | 10 | 12 | 2 | - |
| Physical education | 83 | 2 | 59 | 20 | - | 2 | - |
| Library science | 53 | - | 30 | 15 | 6 | 2 | - |
| Social welfare | 435 | 13 | 274 | 111 | 28 | 9 | - |
| Criminology | 44 | - | 36 | 5 | 2 | 1 | - |
| Business administration and related programmes | 568 | - | 243 | 231 | 64 | 29 | 1 |
| Secretarial training | 872 | - | 696 | 174 | 2 | - | - |
| shorthand typing | 358 | 14 | 316 | 25 | 3 | - | - |
| Clerical Typing | 195 | 11 | 175 | 9 | - | - | - |
| Operating of office machines | 27 | 1 | 24 | 2 | - | - | - |
| Service trade | 416 | 20 | 350 | 42 | 3 | 1 | - |
| Radio and television broadcasting | 16 | - | 12 | 4 | - | - | - |
| Fire protection and fire fighting | 54 | 44 | 10 | - | - | - | - |
| Agriculture forestry and fishery | 909 | 211 | 544 | 98 | 37 | 16 | 3 |
| Food and drinks processing trades programmes | 190 | 7 | 152 | 28 | 2 | 1 | - |
| Wood working | 88 | 9 | 70 | 8 | 1 | - | - |
| Textile trades | 701 | 36 | 648 | 16 | 1 | - | - |
| Leather trades | 43 | 4 | 38 | 1 | - | - | - |
| Other programmes | 2,446 | 300 | 1,735 | 300 | 83 | 28 | - |
| None | 1,255,476 | 1,255,313 | 163 | - | - | - | - |

## Table E11: Usually Working Population (De Facto) 12 Years and Older by Field of Study, Sex and Highest Professional/Vocational Qualification Completed, Urban, Zambia 2010

| Field of Study, Sex and Rural/Urban | Usually Working Population | Highest Profession/Vocational Qualification | | | | | |
|---|---|---|---|---|---|---|---|
| | | None | Certificate | Diploma | Bachelor's Degree | Master's Degree | PhD |
| **Zambia Urban** | **1,260,817** | **925,188** | **183,953** | **111,039** | **31,758** | **8,044** | **835** |
| Natural science | 2,522 | - | 398 | 802 | 948 | 292 | 82 |
| Civil engineering | 3,710 | - | 1,386 | 1,128 | 982 | 198 | 16 |
| Electrical and electronics engineering | 17,676 | 198 | 11,161 | 4,853 | 1,258 | 191 | 15 |
| Mechanical engineering | 22,326 | 358 | 15,202 | 5,344 | 1,218 | 181 | 23 |
| Chemical engineering | 1,043 | - | 330 | 331 | 318 | 60 | 4 |
| Mining engineering | 3,046 | 126 | 1,477 | 824 | 517 | 93 | 9 |
| Industrial engineering | 1,179 | - | 809 | 277 | 73 | 20 | - |
| Metallurgical engineering | 1,094 | - | 511 | 279 | 268 | 27 | 9 |
| Architectural and town planning engineering | 1,093 | - | 329 | 321 | 359 | 77 | 7 |
| Other engineering | 5,940 | 89 | 3,737 | 1,536 | 447 | 115 | 16 |
| Medicine and surgery | 2,930 | - | 300 | 998 | 1,038 | 477 | 117 |
| Pharmacy | 1,953 | - | 608 | 954 | 325 | 61 | 5 |
| Dentistry | 700 | - | 301 | 299 | 81 | 18 | 1 |
| Nursing | 13,647 | - | 7,293 | 5,930 | 335 | 85 | 4 |
| Medical technology | 1,793 | - | 353 | 982 | 336 | 103 | 19 |
| X-Ray technology | 289 | - | 62 | 143 | 38 | 42 | 4 |
| Veterinary | 660 | - | 281 | 190 | 121 | 50 | 18 |
| Statistics | 373 | - | 108 | 121 | 112 | 26 | 6 |
| Mathematics | 596 | - | 84 | 256 | 213 | 36 | 7 |
| Computer science/Economics | 11,945 | - | 5,095 | 3,856 | 2,244 | 683 | 67 |
| Accountancy | 28,257 | - | 8,690 | 14,476 | 4,240 | 794 | 57 |
| Teacher training | 64,356 | 823 | 33,540 | 24,698 | 4,499 | 735 | 61 |
| Law and jurisprudence | 5,334 | - | 1,959 | 1,765 | 1,309 | 277 | 24 |
| Journalism | 2,088 | - | 557 | 1,292 | 184 | 49 | 6 |
| Fine arts | 1,596 | 58 | 930 | 298 | 281 | 27 | 2 |
| Physical education | 452 | 17 | 190 | 145 | 73 | 23 | 4 |
| Library science | 710 | - | 272 | 255 | 151 | 28 | 4 |
| Social welfare | 5,501 | 57 | 2,327 | 2,340 | 634 | 135 | 8 |
| Criminology | 2,543 | - | 2,166 | 331 | 34 | 10 | 2 |
| Business administration and related programmes | 26,009 | - | 7,888 | 13,094 | 3,587 | 1,400 | 40 |
| Secretarial training | 9,150 | - | 6,633 | 2,391 | 109 | 15 | 2 |
| shorthand typing | 1,263 | 33 | 1,054 | 158 | 13 | 4 | 1 |
| Clerical Typing | 1,268 | 53 | 1,030 | 169 | 14 | 2 | - |
| Operating of office machines | 814 | 23 | 591 | 177 | 17 | 6 | - |
| Service trade | 7,916 | 265 | 6,344 | 1,220 | 76 | 10 | 1 |
| Radio and television broadcasting | 499 | 4 | 203 | 199 | 77 | 16 | - |
| Fire protection and fire fighting | 719 | 58 | 544 | 105 | 6 | 6 | - |
| Agriculture forestry and fishery | 6,458 | 268 | 3,366 | 1,896 | 659 | 232 | 37 |
| Food and drinks processing trades programmes | 3,999 | 136 | 3,245 | 557 | 47 | 13 | 1 |
| Wood working | 6,303 | 350 | 5,675 | 246 | 25 | 7 | - |
| Textile trades | 4,725 | 230 | 4,311 | 162 | 19 | 3 | - |
| Leather trades | 396 | 21 | 333 | 31 | 7 | 4 | - |
| Other programmes | 67,491 | 3,616 | 42,230 | 15,610 | 4,466 | 1,413 | 156 |
| None | 918,455 | 918,405 | 50 | - | - | - | - |

## Table E11: Usually Working Population (De Facto) 12 Years and Older by Field of Study, Sex and Highest Professional/Vocational Qualification Completed, Urban, Zambia 2010

| Field of Study, Sex and Rural/Urban | Usually Working Population | Highest Profession/Vocational Qualification | | | | | |
|---|---|---|---|---|---|---|---|
| | | None | Certificate | Diploma | Bachelor's Degree | Master's Degree | PhD |
| **Male Urban** | **798,523** | **581,979** | **116,544** | **69,842** | **23,500** | **5,965** | **693** |
| Natural science | 1,959 | - | 329 | 595 | 737 | 225 | 73 |
| Civil engineering | 3,557 | - | 1,345 | 1,071 | 935 | 190 | 16 |
| Electrical and electronics engineering | 16,761 | 186 | 10,560 | 4,616 | 1,204 | 181 | 14 |
| Mechanical engineering | 21,901 | 350 | 14,898 | 5,252 | 1,200 | 178 | 23 |
| Chemical engineering | 976 | - | 322 | 312 | 288 | 50 | 4 |
| Mining engineering | 2,954 | 117 | 1,428 | 805 | 503 | 92 | 9 |
| Industrial engineering | 1,139 | - | 783 | 268 | 71 | 17 | - |
| Metallurgical engineering | 1,063 | - | 498 | 275 | 258 | 24 | 8 |
| Architectural and town planning engineering | 999 | - | 316 | 300 | 309 | 67 | 7 |
| Other engineering | 5,707 | 85 | 3,618 | 1,466 | 413 | 109 | 16 |
| Medicine and surgery | 2,245 | - | 241 | 780 | 785 | 349 | 90 |
| Pharmacy | 1,262 | - | 389 | 614 | 221 | 35 | 3 |
| Dentistry | 488 | - | 213 | 200 | 61 | 13 | 1 |
| Nursing | 3,538 | - | 2,032 | 1,411 | 78 | 16 | 1 |
| Medical technology | 1,308 | - | 244 | 719 | 264 | 63 | 18 |
| X-Ray technology | 197 | - | 43 | 92 | 29 | 30 | 3 |
| Veterinary | 548 | - | 231 | 159 | 99 | 42 | 17 |
| Statistics | 299 | - | 85 | 96 | 91 | 21 | 6 |
| Mathematics | 458 | - | 59 | 186 | 182 | 24 | 7 |
| Computer science/Economics | 7,949 | - | 2,936 | 2,692 | 1,736 | 528 | 57 |
| Accountancy | 21,113 | - | 6,499 | 10,717 | 3,233 | 618 | 46 |
| Teacher training | 25,025 | 336 | 10,314 | 11,086 | 2,753 | 487 | 49 |
| Law and jurisprudence | 4,010 | - | 1,565 | 1,329 | 920 | 178 | 18 |
| Journalism | 1,183 | - | 354 | 694 | 95 | 35 | 5 |
| Fine arts | 1,170 | 41 | 705 | 234 | 171 | 17 | 2 |
| Physical education | 272 | 11 | 105 | 89 | 47 | 17 | 3 |
| Library science | 346 | - | 138 | 118 | 72 | 15 | 3 |
| Social welfare | 2,412 | 33 | 984 | 954 | 358 | 77 | 6 |
| Criminology | 2,093 | - | 1,766 | 286 | 31 | 8 | 2 |
| Business administration and related programmes | 17,077 | - | 5,273 | 8,276 | 2,441 | 1,058 | 29 |
| Secretarial training | 640 | - | 460 | 160 | 15 | 3 | 2 |
| shorthand typing | 295 | 5 | 231 | 50 | 7 | 2 | - |
| Clerical Typing | 511 | 26 | 383 | 90 | 10 | 2 | - |
| Operating of office machines | 627 | 16 | 456 | 135 | 16 | 4 | - |
| Service trade | 3,396 | 117 | 2,748 | 491 | 35 | 5 | - |
| Radio and television broadcasting | 381 | 2 | 159 | 153 | 55 | 12 | - |
| Fire protection and fire fighting | 644 | 42 | 495 | 95 | 6 | 6 | - |
| Agriculture forestry and fishery | 5,315 | 197 | 2,731 | 1,612 | 553 | 189 | 33 |
| Food and drinks processing trades programmes | 1,705 | 65 | 1,370 | 242 | 21 | 7 | - |
| Wood working | 6,102 | 340 | 5,519 | 215 | 23 | 5 | - |
| Textile trades | 1,106 | 56 | 967 | 70 | 12 | 1 | - |
| Leather trades | 165 | 7 | 128 | 20 | 6 | 4 | - |
| Other programmes | 50,394 | 2,738 | 32,600 | 10,817 | 3,156 | 961 | 122 |
| None | 577,233 | 577,209 | 24 | - | - | - | - |

## Table E11: Usually Working Population (De Facto) 12 Years and Older by Field of Study, Sex and Highest Professional/Vocational Qualification Completed, Urban, Zambia 2010

| Field of Study, Sex and Rural/Urban | Usually Working Population | Highest Profession/Vocational Qualification | | | | | |
|---|---|---|---|---|---|---|---|
| | | None | Certificate | Diploma | Bachelor's Degree | Master's Degree | PhD |
| **Female Urban** | **462,294** | **343,209** | **67,409** | **41,197** | **8,258** | **2,079** | **142** |
| Natural science | 563 | - | 69 | 207 | 211 | 67 | 9 |
| Civil engineering | 153 | - | 41 | 57 | 47 | 8 | - |
| Electrical and electronics engineering | 915 | 12 | 601 | 237 | 54 | 10 | 1 |
| Mechanical engineering | 425 | 8 | 304 | 92 | 18 | 3 | - |
| Chemical engineering | 67 | - | 8 | 19 | 30 | 10 | - |
| Mining engineering | 92 | 9 | 49 | 19 | 14 | 1 | - |
| Industrial engineering | 40 | - | 26 | 9 | 2 | 3 | - |
| Metallurgical engineering | 31 | - | 13 | 4 | 10 | 3 | 1 |
| Architectural and town planning engineering | 94 | - | 13 | 21 | 50 | 10 | - |
| Other engineering | 233 | 4 | 119 | 70 | 34 | 6 | - |
| Medicine and surgery | 685 | - | 59 | 218 | 253 | 128 | 27 |
| Pharmacy | 691 | - | 219 | 340 | 104 | 26 | 2 |
| Dentistry | 212 | - | 88 | 99 | 20 | 5 | - |
| Nursing | 10,109 | - | 5,261 | 4,519 | 257 | 69 | 3 |
| Medical technology | 485 | - | 109 | 263 | 72 | 40 | 1 |
| X-Ray technology | 92 | - | 19 | 51 | 9 | 12 | 1 |
| Veterinary | 112 | - | 50 | 31 | 22 | 8 | 1 |
| Statistics | 74 | - | 23 | 25 | 21 | 5 | - |
| Mathematics | 138 | - | 25 | 70 | 31 | 12 | - |
| Computer science/Economics | 3,996 | - | 2,159 | 1,164 | 508 | 155 | 10 |
| Accountancy | 7,144 | - | 2,191 | 3,759 | 1,007 | 176 | 11 |
| Teacher training | 39,331 | 487 | 23,226 | 13,612 | 1,746 | 248 | 12 |
| Law and jurisprudence | 1,324 | - | 394 | 436 | 389 | 99 | 6 |
| Journalism | 905 | - | 203 | 598 | 89 | 14 | 1 |
| Fine arts | 426 | 17 | 225 | 64 | 110 | 10 | - |
| Physical education | 180 | 6 | 85 | 56 | 26 | 6 | 1 |
| Library science | 364 | - | 134 | 137 | 79 | 13 | 1 |
| Social welfare | 3,089 | 24 | 1,343 | 1,386 | 276 | 58 | 2 |
| Criminology | 450 | - | 400 | 45 | 3 | 2 | - |
| Business administration and related programmes | 8,932 | - | 2,615 | 4,818 | 1,146 | 342 | 11 |
| Secretarial training | 8,510 | - | 6,173 | 2,231 | 94 | 12 | - |
| shorthand typing | 968 | 28 | 823 | 108 | 6 | 2 | 1 |
| Clerical Typing | 757 | 27 | 647 | 79 | 4 | - | - |
| Operating of office machines | 187 | 7 | 135 | 42 | 1 | 2 | - |
| Service trade | 4,520 | 148 | 3,596 | 729 | 41 | 5 | 1 |
| Radio and television broadcasting | 118 | 2 | 44 | 46 | 22 | 4 | - |
| Fire protection and fire fighting | 75 | 16 | 49 | 10 | - | - | - |
| Agriculture forestry and fishery | 1,143 | 71 | 635 | 284 | 106 | 43 | 4 |
| Food and drinks processing trades programmes | 2,294 | 71 | 1,875 | 315 | 26 | 6 | 1 |
| Wood working | 201 | 10 | 156 | 31 | 2 | 2 | - |
| Textile trades | 3,619 | 174 | 3,344 | 92 | 7 | 2 | - |
| Leather trades | 231 | 14 | 205 | 11 | 1 | - | - |
| Other programmes | 17,097 | 878 | 9,630 | 4,793 | 1,310 | 452 | 34 |

## Table E12: Usually Working Population (De Facto) 12 Years and Older by Industry, Sex and Employment Status, Rural/Urban, Zambia 2010

| Sex and Industry of Employment | Usually Working Population | Employment Status | | | |
|---|---|---|---|---|---|
| | | Employer | Employee | Self Employed | Unpaid Family Worker |
| **Zambia Total** | | | | | |
| **Total** | **3,887,052** | **29,033** | **864,942** | **1,715,082** | **1,277,995** |
| Agriculture Hunting Forestry and Fishing | 2,440,748 | 6,168 | 101,381 | 1,196,816 | 1,136,383 |
| Mining and Quarrying | 69,922 | 1,240 | 60,407 | 6,954 | 1,321 |
| Manufacturing | 132,292 | 2,155 | 67,373 | 57,451 | 5,313 |
| Electricity Gas Steam and Air conditioning supply | 8,578 | 168 | 7,462 | 921 | 27 |
| Water Supply | 6,729 | 170 | 5,794 | 735 | 30 |
| Construction and Allied Repairs | 110,991 | 2,066 | 59,098 | 47,347 | 2,480 |
| Wholesale & Retail Trade Restaurants and Hotel | 361,660 | 3,669 | 72,049 | 254,382 | 31,560 |
| Transport and Storage | 91,113 | 2,016 | 74,405 | 13,550 | 1,142 |
| Accomodation and food services activities | 39,220 | 965 | 28,710 | 8,351 | 1,194 |
| Information and Communication | 23,337 | 378 | 13,394 | 5,902 | 3,663 |
| Finance and Insurance | 17,228 | 356 | 15,780 | 1,040 | 52 |
| Real Estate Activies | 1,307 | 48 | 799 | 424 | 36 |
| Community Social and Personal Services | 366,193 | 7,453 | 314,113 | 38,694 | 5,933 |
| Not Stated | 217,734 | 2,181 | 44,177 | 82,515 | 88,861 |
| **Male** | | | | | |
| **Total** | **2,140,733** | **21,227** | **629,825** | **1,004,229** | **485,452** |
| Agriculture Hunting Forestry and Fishing | 1,227,232 | 4,603 | 80,811 | 714,351 | 427,467 |
| Mining and Quarrying | 63,840 | 1,148 | 57,054 | 5,013 | 625 |
| Manufacturing | 103,436 | 1,805 | 57,921 | 41,099 | 2,611 |
| Electricity Gas Steam and Air conditioning supply | 7,563 | 150 | 6,504 | 885 | 24 |
| Water Supply | 5,407 | 144 | 4,614 | 627 | 22 |
| Construction and Allied Repairs | 107,979 | 1,988 | 57,440 | 46,322 | 2,229 |
| Wholesale & Retail Trade Restaurants and Hotel | 171,651 | 2,455 | 48,189 | 111,770 | 9,237 |
| Transport and Storage | 87,203 | 1,915 | 71,676 | 12,623 | 989 |
| Accomodation and food services activities | 18,146 | 451 | 14,728 | 2,667 | 300 |
| Information and Communication | 14,287 | 272 | 8,533 | 3,888 | 1,594 |
| Finance and Insurance | 10,875 | 245 | 9,856 | 745 | 29 |
| Real Estate Activies | 898 | 37 | 550 | 289 | 22 |
| Community Social and Personal Services | 213,519 | 4,570 | 183,288 | 23,148 | 2,513 |
| Not Stated | 108,697 | 1,444 | 28,661 | 40,802 | 37,790 |
| **Female** | | | | | |
| **Total** | **1,746,319** | **7,806** | **235,117** | **710,853** | **792,543** |
| Agriculture Hunting Forestry and Fishing | 1,213,516 | 1,565 | 20,570 | 482,465 | 708,916 |
| Mining and Quarrying | 6,082 | 92 | 3,353 | 1,941 | 696 |
| Manufacturing | 28,856 | 350 | 9,452 | 16,352 | 2,702 |
| Electricity Gas Steam and Air conditioning supply | 1,015 | 18 | 958 | 36 | 3 |
| Water Supply | 1,322 | 26 | 1,180 | 108 | 8 |
| Construction and Allied Repairs | 3,012 | 78 | 1,658 | 1,025 | 251 |
| Wholesale & Retail Trade Restaurants and Hotel | 190,009 | 1,214 | 23,860 | 142,612 | 22,323 |
| Transport and Storage | 3,910 | 101 | 2,729 | 927 | 153 |
| Accomodation and food services activities | 21,074 | 514 | 13,982 | 5,684 | 894 |
| Information and Communication | 9,050 | 106 | 4,861 | 2,014 | 2,069 |
| Finance and Insurance | 6,353 | 111 | 5,924 | 295 | 23 |
| Real Estate Activies | 409 | 11 | 249 | 135 | 14 |
| Community Social and Personal Services | 152,674 | 2,883 | 130,825 | 15,546 | 3,420 |
| Not Stated | 109,037 | 737 | 15,516 | 41,713 | 51,071 |

## Table E12: Usually Working Population (De Facto) 12 Years and Older by Industry, Sex and Employment Status, Rural, Zambia 2010

| Sex and Industry of Employment | Usually Working Population | Employment Status | | | |
|---|---|---|---|---|---|
| | | Employer | Employee | Self Employed | Unpaid Family Worker |
| **Zambia Rural** | | | | | |
| **Total** | **2,624,368** | **9,434** | **194,631** | **1,241,111** | **1,179,192** |
| Agriculture Hunting Forestry and Fishing | 2,276,211 | 4,901 | 77,273 | 1,113,857 | 1,080,180 |
| Mining and Quarrying | 9,173 | 209 | 4,885 | 2,939 | 1,140 |
| Manufacturing | 27,930 | 318 | 7,982 | 16,355 | 3,275 |
| Electricity Gas Steam and Air conditioning supply | 869 | 20 | 771 | 66 | 12 |
| Water Supply | 505 | 15 | 390 | 82 | 18 |
| Construction and Allied Repairs | 26,002 | 480 | 12,422 | 11,495 | 1,605 |
| Wholesale & Retail Trade Restaurants and Hotel | 54,243 | 322 | 5,370 | 37,353 | 11,198 |
| Transport and Storage | 8,251 | 182 | 5,778 | 1,880 | 411 |
| Accomodation and food services activities | 5,069 | 86 | 3,087 | 1,458 | 438 |
| Information and Communication | 8,270 | 46 | 1,315 | 3,609 | 3,300 |
| Finance and Insurance | 813 | 27 | 671 | 103 | 12 |
| Real Estate Activies | 251 | 1 | 223 | 18 | 9 |
| Community Social and Personal Services | 76,707 | 2,065 | 63,418 | 7,970 | 3,254 |
| Not Stated | 130,074 | 762 | 11,046 | 43,926 | 74,340 |
| **Male** | | | | | |
| **Total** | **1,341,036** | **7,043** | **148,546** | **738,866** | **446,581** |
| Agriculture Hunting Forestry and Fishing | 1,136,245 | 3,637 | 61,292 | 666,155 | 405,161 |
| Mining and Quarrying | 7,128 | 188 | 4,470 | 1,954 | 516 |
| Manufacturing | 19,357 | 256 | 6,632 | 10,957 | 1,512 |
| Electricity Gas Steam and Air conditioning supply | 789 | 20 | 694 | 63 | 12 |
| Water Supply | 433 | 14 | 336 | 70 | 13 |
| Construction and Allied Repairs | 25,281 | 462 | 12,147 | 11,223 | 1,449 |
| Wholesale & Retail Trade Restaurants and Hotel | 22,344 | 210 | 3,286 | 15,977 | 2,871 |
| Transport and Storage | 7,735 | 174 | 5,575 | 1,654 | 332 |
| Accomodation and food services activities | 2,520 | 46 | 2,008 | 385 | 81 |
| Information and Communication | 4,457 | 33 | 938 | 2,087 | 1,399 |
| Finance and Insurance | 548 | 21 | 443 | 76 | 8 |
| Real Estate Activies | 192 | 1 | 174 | 10 | 7 |
| Community Social and Personal Services | 50,856 | 1,437 | 42,736 | 5,260 | 1,423 |
| Not Stated | 63,151 | 544 | 7,815 | 22,995 | 31,797 |
| **Female** | | | | | |
| **Total** | **1,283,332** | **2,391** | **46,085** | **502,245** | **732,611** |
| Agriculture Hunting Forestry and Fishing | 1,139,966 | 1,264 | 15,981 | 447,702 | 675,019 |
| Mining and Quarrying | 2,045 | 21 | 415 | 985 | 624 |
| Manufacturing | 8,573 | 62 | 1,350 | 5,398 | 1,763 |
| Electricity Gas Steam and Air conditioning supply | 80 | - | 77 | 3 | - |
| Water Supply | 72 | 1 | 54 | 12 | 5 |
| Construction and Allied Repairs | 721 | 18 | 275 | 272 | 156 |
| Wholesale & Retail Trade Restaurants and Hotel | 31,899 | 112 | 2,084 | 21,376 | 8,327 |
| Transport and Storage | 516 | 8 | 203 | 226 | 79 |
| Accomodation and food services activities | 2,549 | 40 | 1,079 | 1,073 | 357 |
| Information and Communication | 3,813 | 13 | 377 | 1,522 | 1,901 |
| Finance and Insurance | 265 | 6 | 228 | 27 | 4 |
| Real Estate Activies | 59 | - | 49 | 8 | 2 |
| Community Social and Personal Services | 25,851 | 628 | 20,682 | 2,710 | 1,831 |
| Not Stated | 66,923 | 218 | 3,231 | 20,931 | 42,543 |

## Table E12: Usually Working Population (De Facto) 12 Years and Older by Industry, Sex and Employment Status, Urban, Zambia 2010

| Sex and Industry of Employment | Usually Working Population | Employment Status | | | |
|---|---|---|---|---|---|
| | | Employer | Employee | Self Employed | Unpaid Family Worker |
| **Zambia Urban** | | | | | |
| **Total** | **1,262,684** | **19,599** | **670,311** | **473,971** | **98,803** |
| Agriculture Hunting Forestry and Fishing | 164,537 | 1,267 | 24,108 | 82,959 | 56,203 |
| Mining and Quarrying | 60,749 | 1,031 | 55,522 | 4,015 | 181 |
| Manufacturing | 104,362 | 1,837 | 59,391 | 41,096 | 2,038 |
| Electricity Gas Steam and Air conditioning supply | 7,709 | 148 | 6,691 | 855 | 15 |
| Water Supply | 6,224 | 155 | 5,404 | 653 | 12 |
| Construction and Allied Repairs | 84,989 | 1,586 | 46,676 | 35,852 | 875 |
| Wholesale & Retail Trade Restaurants and Hotel | 307,417 | 3,347 | 66,679 | 217,029 | 20,362 |
| Transport and Storage | 82,862 | 1,834 | 68,627 | 11,670 | 731 |
| Accomodation and food services activities | 34,151 | 879 | 25,623 | 6,893 | 756 |
| Information and Communication | 15,067 | 332 | 12,079 | 2,293 | 363 |
| Finance and Insurance | 16,415 | 329 | 15,109 | 937 | 40 |
| Real Estate Activies | 1,056 | 47 | 576 | 406 | 27 |
| Community Social and Personal Services | 289,486 | 5,388 | 250,695 | 30,724 | 2,679 |
| Not Stated | 87,660 | 1,419 | 33,131 | 38,589 | 14,521 |
| **Male** | | | | | |
| **Total** | **799,697** | **14,184** | **481,279** | **265,363** | **38,871** |
| Agriculture Hunting Forestry and Fishing | 90,987 | 966 | 19,519 | 48,196 | 22,306 |
| Mining and Quarrying | 56,712 | 960 | 52,584 | 3,059 | 109 |
| Manufacturing | 84,079 | 1,549 | 51,289 | 30,142 | 1,099 |
| Electricity Gas Steam and Air conditioning supply | 6,774 | 130 | 5,810 | 822 | 12 |
| Water Supply | 4,974 | 130 | 4,278 | 557 | 9 |
| Construction and Allied Repairs | 82,698 | 1,526 | 45,293 | 35,099 | 780 |
| Wholesale & Retail Trade Restaurants and Hotel | 149,307 | 2,245 | 44,903 | 95,793 | 6,366 |
| Transport and Storage | 79,468 | 1,741 | 66,101 | 10,969 | 657 |
| Accomodation and food services activities | 15,626 | 405 | 12,720 | 2,282 | 219 |
| Information and Communication | 9,830 | 239 | 7,595 | 1,801 | 195 |
| Finance and Insurance | 10,327 | 224 | 9,413 | 669 | 21 |
| Real Estate Activies | 706 | 36 | 376 | 279 | 15 |
| Community Social and Personal Services | 162,663 | 3,133 | 140,552 | 17,888 | 1,090 |
| Not Stated | 45,546 | 900 | 20,846 | 17,807 | 5,993 |
| **Female** | | | | | |
| **Total** | **462,987** | **5,415** | **189,032** | **208,608** | **59,932** |
| Agriculture Hunting Forestry and Fishing | 73,550 | 301 | 4,589 | 34,763 | 33,897 |
| Mining and Quarrying | 4,037 | 71 | 2,938 | 956 | 72 |
| Manufacturing | 20,283 | 288 | 8,102 | 10,954 | 939 |
| Electricity Gas Steam and Air conditioning supply | 935 | 18 | 881 | 33 | 3 |
| Water Supply | 1,250 | 25 | 1,126 | 96 | 3 |
| Construction and Allied Repairs | 2,291 | 60 | 1,383 | 753 | 95 |
| Wholesale & Retail Trade Restaurants and Hotel | 158,110 | 1,102 | 21,776 | 121,236 | 13,996 |
| Transport and Storage | 3,394 | 93 | 2,526 | 701 | 74 |
| Accomodation and food services activities | 18,525 | 474 | 12,903 | 4,611 | 537 |
| Information and Communication | 5,237 | 93 | 4,484 | 492 | 168 |
| Finance and Insurance | 6,088 | 105 | 5,696 | 268 | 19 |
| Real Estate Activies | 350 | 11 | 200 | 127 | 12 |
| Community Social and Personal Services | 126,823 | 2,255 | 110,143 | 12,836 | 1,589 |
| Not Stated | 42,114 | 519 | 12,285 | 20,782 | 8,528 |

## Table E13: Usually Working Population (De Facto) 12 Years and Older by Occupation, Sex and Employment Status, Rural/Urban, Zambia 2010

| Sex and Occupation | Usually Working Population | Employment Status | | | |
|---|---|---|---|---|---|
| | | Employer | Employee | Self Employed | Unpaid Family Worker |
| **ZambiaTotal** | | | | | |
| **Total** | **3,887,052** | **29,033** | **864,942** | **1,715,082** | **1,277,995** |
| Managers | 35,898 | 3,225 | 21,358 | 8,877 | 2,438 |
| Professionals | 177,334 | 4,281 | 154,367 | 16,362 | 2,324 |
| Technicians and Associate Professionals | 84,637 | 1,820 | 61,762 | 18,992 | 2,063 |
| Clerical Support Workers | 37,323 | 588 | 33,895 | 2,361 | 479 |
| Service and Sales Workers | 388,270 | 4,273 | 159,601 | 198,876 | 25,520 |
| Skilled Agricultural Forestry and Fishery Workers | 2,092,815 | 4,592 | 44,468 | 1,070,787 | 972,968 |
| Craft and Related Trades Workers | 267,053 | 3,408 | 121,956 | 129,747 | 11,942 |
| Plant and Machine Operators and Assemblers | 123,689 | 2,246 | 98,919 | 18,984 | 3,540 |
| Elementary Occupations | 497,112 | 2,857 | 129,578 | 185,375 | 179,302 |
| Not Stated | 182,921 | 1,743 | 39,038 | 64,721 | 77,419 |
| **Male** | | | | | |
| **Total** | **2,140,733** | **21,227** | **629,825** | **1,004,229** | **485,452** |
| Managers | 25,304 | 2,370 | 15,782 | 5,804 | 1,348 |
| Professionals | 103,222 | 2,738 | 88,006 | 11,318 | 1,160 |
| Technicians and Associate Professionals | 58,056 | 1,332 | 42,217 | 13,451 | 1,056 |
| Clerical Support Workers | 16,847 | 287 | 15,109 | 1,242 | 209 |
| Service and Sales Workers | 195,092 | 2,533 | 101,074 | 83,662 | 7,823 |
| Skilled Agricultural Forestry and Fishery Workers | 1,046,240 | 3,413 | 37,001 | 639,171 | 366,655 |
| Craft and Related Trades Workers | 225,488 | 3,164 | 115,831 | 100,800 | 5,693 |
| Plant and Machine Operators and Assemblers | 114,539 | 2,179 | 96,516 | 14,102 | 1,742 |
| Elementary Occupations | 261,492 | 2,016 | 89,876 | 102,999 | 66,601 |
| Not Stated | 94,453 | 1,195 | 28,413 | 31,680 | 33,165 |
| **Female** | | | | | |
| **Total** | **1,746,319** | **7,806** | **235,117** | **710,853** | **792,543** |
| Managers | 10,594 | 855 | 5,576 | 3,073 | 1,090 |
| Professionals | 74,112 | 1,543 | 66,361 | 5,044 | 1,164 |
| Technicians and Associate Professionals | 26,581 | 488 | 19,545 | 5,541 | 1,007 |
| Clerical Support Workers | 20,476 | 301 | 18,786 | 1,119 | 270 |
| Service and Sales Workers | 193,178 | 1,740 | 58,527 | 115,214 | 17,697 |
| Skilled Agricultural Forestry and Fishery Workers | 1,046,575 | 1,179 | 7,467 | 431,616 | 606,313 |
| Craft and Related Trades Workers | 41,565 | 244 | 6,125 | 28,947 | 6,249 |
| Plant and Machine Operators and Assemblers | 9,150 | 67 | 2,403 | 4,882 | 1,798 |
| Elementary Occupations | 235,620 | 841 | 39,702 | 82,376 | 112,701 |
| Not Stated | 88,468 | 548 | 10,625 | 33,041 | 44,254 |

## Table E13: Usually Working Population (De Facto) 12 Years and Older by Occupation, Sex and Employment Status, Rural, Zambia 2010

| Sex and Occupation | Usually Working Population | Employment Status | | | |
|---|---|---|---|---|---|
| | | Employer | Employee | Self Employed | Unpaid Family Worker |
| **Zambia Rural** | | | | | |
| **Total** | **2,624,368** | **9,434** | **194,631** | **1,241,111** | **1,179,192** |
| Managers | 8,145 | 355 | 2,784 | 2,869 | 2,137 |
| Professionals | 45,490 | 1,448 | 38,758 | 3,596 | 1,688 |
| Technicians and Associate Professionals | 16,739 | 475 | 10,051 | 4,898 | 1,315 |
| Clerical Support Workers | 3,306 | 65 | 2,462 | 491 | 288 |
| Service and Sales Workers | 54,317 | 573 | 19,535 | 25,786 | 8,423 |
| Skilled Agricultural Forestry and Fishery Workers | 1,954,531 | 3,714 | 30,165 | 996,187 | 924,465 |
| Craft and Related Trades Workers | 67,757 | 686 | 18,402 | 40,683 | 7,986 |
| Plant and Machine Operators and Assemblers | 18,465 | 274 | 10,158 | 5,600 | 2,433 |
| Elementary Occupations | 346,715 | 1,295 | 53,514 | 125,999 | 165,907 |
| Not Stated | 108,903 | 549 | 8,802 | 35,002 | 64,550 |
| **Male** | | | | | |
| **Total** | **1,341,036** | **7,043** | **148,546** | **738,866** | **446,581** |
| Managers | 5,630 | 272 | 2,321 | 1,843 | 1,194 |
| Professionals | 28,938 | 981 | 24,792 | 2,330 | 835 |
| Technicians and Associate Professionals | 11,127 | 355 | 7,040 | 3,086 | 646 |
| Clerical Support Workers | 1,884 | 37 | 1,431 | 287 | 129 |
| Service and Sales Workers | 27,897 | 390 | 13,680 | 11,429 | 2,398 |
| Skilled Agricultural Forestry and Fishery Workers | 970,705 | 2,754 | 24,596 | 596,027 | 347,328 |
| Craft and Related Trades Workers | 49,448 | 638 | 17,407 | 27,872 | 3,531 |
| Plant and Machine Operators and Assemblers | 13,951 | 260 | 9,832 | 2,901 | 958 |
| Elementary Occupations | 178,531 | 978 | 40,938 | 74,888 | 61,727 |
| Not Stated | 52,925 | 378 | 6,509 | 18,203 | 27,835 |
| **Female** | | | | | |
| **Total** | **1,283,332** | **2,391** | **46,085** | **502,245** | **732,611** |
| Managers | 2,515 | 83 | 463 | 1,026 | 943 |
| Professionals | 16,552 | 467 | 13,966 | 1,266 | 853 |
| Technicians and Associate Professionals | 5,612 | 120 | 3,011 | 1,812 | 669 |
| Clerical Support Workers | 1,422 | 28 | 1,031 | 204 | 159 |
| Service and Sales Workers | 26,420 | 183 | 5,855 | 14,357 | 6,025 |
| Skilled Agricultural Forestry and Fishery Workers | 983,826 | 960 | 5,569 | 400,160 | 577,137 |
| Craft and Related Trades Workers | 18,309 | 48 | 995 | 12,811 | 4,455 |
| Plant and Machine Operators and Assemblers | 4,514 | 14 | 326 | 2,699 | 1,475 |
| Elementary Occupations | 168,184 | 317 | 12,576 | 51,111 | 104,180 |
| Not Stated | 55,978 | 171 | 2,293 | 16,799 | 36,715 |

## Table E13: Usually Working Population (De Facto) 12 Years and Older by Occupation, Sex and Employment Status, Urban, Zambia 2010

| Sex and Occupation | Usually Working Population | Employment Status | | | |
|---|---|---|---|---|---|
| | | Employer | Employee | Self Employed | Unpaid Family Worker |
| **Zambia Urban** | | | | | |
| **Total** | **1,262,684** | **19,599** | **670,311** | **473,971** | **98,803** |
| Managers | 27,753 | 2,870 | 18,574 | 6,008 | 301 |
| Professionals | 131,844 | 2,833 | 115,609 | 12,766 | 636 |
| Technicians and Associate Professionals | 67,898 | 1,345 | 51,711 | 14,094 | 748 |
| Clerical Support Workers | 34,017 | 523 | 31,433 | 1,870 | 191 |
| Service and Sales Workers | 333,953 | 3,700 | 140,066 | 173,090 | 17,097 |
| Skilled Agricultural Forestry and Fishery Workers | 138,284 | 878 | 14,303 | 74,600 | 48,503 |
| Craft and Related Trades Workers | 199,296 | 2,722 | 103,554 | 89,064 | 3,956 |
| Plant and Machine Operators and Assemblers | 105,224 | 1,972 | 88,761 | 13,384 | 1,107 |
| Elementary Occupations | 150,397 | 1,562 | 76,064 | 59,376 | 13,395 |
| Not Stated | 74,018 | 1,194 | 30,236 | 29,719 | 12,869 |
| **Male** | | | | | |
| **Total** | **799,697** | **14,184** | **481,279** | **265,363** | **38,871** |
| Managers | 19,674 | 2,098 | 13,461 | 3,961 | 154 |
| Professionals | 74,284 | 1,757 | 63,214 | 8,988 | 325 |
| Technicians and Associate Professionals | 46,929 | 977 | 35,177 | 10,365 | 410 |
| Clerical Support Workers | 14,963 | 250 | 13,678 | 955 | 80 |
| Service and Sales Workers | 167,195 | 2,143 | 87,394 | 72,233 | 5,425 |
| Skilled Agricultural Forestry and Fishery Workers | 75,535 | 659 | 12,405 | 43,144 | 19,327 |
| Craft and Related Trades Workers | 176,040 | 2,526 | 98,424 | 72,928 | 2,162 |
| Plant and Machine Operators and Assemblers | 100,588 | 1,919 | 86,684 | 11,201 | 784 |
| Elementary Occupations | 82,961 | 1,038 | 48,938 | 28,111 | 4,874 |
| Not Stated | 41,528 | 817 | 21,904 | 13,477 | 5,330 |
| **Female** | | | | | |
| **Total** | **462,987** | **5,415** | **189,032** | **208,608** | **59,932** |
| Managers | 8,079 | 772 | 5,113 | 2,047 | 147 |
| Professionals | 57,560 | 1,076 | 52,395 | 3,778 | 311 |
| Technicians and Associate Professionals | 20,969 | 368 | 16,534 | 3,729 | 338 |
| Clerical Support Workers | 19,054 | 273 | 17,755 | 915 | 111 |
| Service and Sales Workers | 166,758 | 1,557 | 52,672 | 100,857 | 11,672 |
| Skilled Agricultural Forestry and Fishery Workers | 62,749 | 219 | 1,898 | 31,456 | 29,176 |
| Craft and Related Trades Workers | 23,256 | 196 | 5,130 | 16,136 | 1,794 |
| Plant and Machine Operators and Assemblers | 4,636 | 53 | 2,077 | 2,183 | 323 |
| Elementary Occupations | 67,436 | 524 | 27,126 | 31,265 | 8,521 |
| Not Stated | 32,490 | 377 | 8,332 | 16,242 | 7,539 |

# Disability Tables

## Table F1: Population (De Facto) and Total Disabled Population by Sex, Province and Rural/Urban, Zambia 2010

| Province and Sex | Population | | | Disabled Population | | | Percent Disabled | | |
|---|---|---|---|---|---|---|---|---|---|
| | Total | Rural | Urban | Total | Rural | Urban | Total | Rural | Urban |
| **Zambia** | | | | | | | | | |
| **Total** | **12,526,314** | **7,505,292** | **5,021,022** | **251,427** | **181,856** | **69,571** | **2.0** | **2.4** | **1.4** |
| Male | 6,117,253 | 3,664,349 | 2,452,904 | 130,302 | 94,268 | 36,034 | 2.1 | 2.6 | 1.5 |
| Female | 6,409,061 | 3,840,943 | 2,568,118 | 121,125 | 87,588 | 33,537 | 1.9 | 2.3 | 1.3 |
| **Central** | | | | | | | | | |
| **Total** | **1,245,089** | **929,042** | **316,047** | **25,943** | **20,566** | **5,377** | **2.1** | **2.2** | **1.7** |
| Male | 613,372 | 460,146 | 153,226 | 13,750 | 10,992 | 2,758 | 2.2 | 2.4 | 1.8 |
| Female | 631,717 | 468,896 | 162,821 | 12,193 | 9,574 | 2,619 | 1.9 | 2.0 | 1.6 |
| **Copperbelt** | | | | | | | | | |
| **Total** | **1,920,611** | **361,652** | **1,558,959** | **30,727** | **8,653** | **22,074** | **1.6** | **2.4** | **1.4** |
| Male | 950,775 | 181,734 | 769,041 | 16,167 | 4,631 | 11,536 | 1.7 | 2.5 | 1.5 |
| Female | 969,836 | 179,918 | 789,918 | 14,560 | 4,022 | 10,538 | 1.5 | 2.2 | 1.3 |
| **Eastern** | | | | | | | | | |
| **Total** | **1,525,123** | **1,333,443** | **191,680** | **31,838** | **29,105** | **2,733** | **2.1** | **2.2** | **1.4** |
| Male | 744,354 | 651,737 | 92,617 | 16,656 | 15,196 | 1,460 | 2.2 | 2.3 | 1.6 |
| Female | 780,769 | 681,706 | 99,063 | 15,182 | 13,909 | 1,273 | 1.9 | 2.0 | 1.3 |
| **Luapula** | | | | | | | | | |
| **Total** | **938,391** | **756,351** | **182,040** | **26,326** | **22,377** | **3,949** | **2.8** | **3.0** | **2.2** |
| Male | 455,046 | 367,462 | 87,584 | 13,306 | 11,364 | 1,942 | 2.9 | 3.1 | 2.2 |
| Female | 483,345 | 388,889 | 94,456 | 13,020 | 11,013 | 2,007 | 2.7 | 2.8 | 2.1 |
| **Lusaka** | | | | | | | | | |
| **Total** | **2,138,907** | **319,732** | **1,819,175** | **27,350** | **6,079** | **21,271** | **1.3** | **1.9** | **1.2** |
| Male | 1,049,215 | 160,216 | 888,999 | 14,470 | 3,331 | 11,139 | 1.4 | 2.1 | 1.3 |
| Female | 1,089,692 | 159,516 | 930,176 | 12,880 | 2,748 | 10,132 | 1.2 | 1.7 | 1.1 |
| **Muchinga** | | | | | | | | | |
| **Total** | **677,507** | **562,025** | **115,482** | **15,110** | **13,338** | **1,772** | **2.2** | **2.4** | **1.5** |
| Male | 328,980 | 272,884 | 56,096 | 7,843 | 6,966 | 877 | 2.4 | 2.6 | 1.6 |
| Female | 348,527 | 289,141 | 59,386 | 7,267 | 6,372 | 895 | 2.1 | 2.2 | 1.5 |
| **Northern** | | | | | | | | | |
| **Total** | **1,044,955** | **852,963** | **191,992** | **24,688** | **21,549** | **3,139** | **2.4** | **2.5** | **1.6** |
| Male | 508,226 | 415,508 | 92,718 | 12,572 | 10,966 | 1,606 | 2.5 | 2.6 | 1.7 |
| Female | 536,729 | 437,455 | 99,274 | 12,116 | 10,583 | 1,533 | 2.3 | 2.4 | 1.5 |
| **North-Western** | | | | | | | | | |
| **Total** | **681,698** | **525,156** | **156,542** | **18,495** | **15,728** | **2,767** | **2.7** | **3.0** | **1.8** |
| Male | 332,376 | 256,167 | 76,209 | 9,549 | 8,176 | 1,373 | 2.9 | 3.2 | 1.8 |
| Female | 349,322 | 268,989 | 80,333 | 8,946 | 7,552 | 1,394 | 2.6 | 2.8 | 1.7 |
| **Southern** | | | | | | | | | |
| **Total** | **1,517,088** | **1,141,540** | **375,548** | **27,133** | **22,536** | **4,597** | **1.8** | **2.0** | **1.2** |
| Male | 738,996 | 556,592 | 182,404 | 14,072 | 11,665 | 2,407 | 1.9 | 2.1 | 1.3 |
| Female | 778,092 | 584,948 | 193,144 | 13,061 | 10,871 | 2,190 | 1.7 | 1.9 | 1.1 |
| **Western** | | | | | | | | | |
| **Total** | **836,945** | **723,388** | **113,557** | **23,817** | **21,925** | **1,892** | **2.8** | **3.0** | **1.7** |
| Male | 395,913 | 341,903 | 54,010 | 11,917 | 10,981 | 936 | 3.0 | 3.2 | 1.7 |
| Female | 441,032 | 381,485 | 59,547 | 11,900 | 10,944 | 956 | 2.7 | 2.9 | 1.6 |

## Table F2: Population (De facto) and Total Disabled Population by 5 Year Age Group, Sex and Rural/Urban, Zambia, 2010

| Age Group and Rural/Urban | Population | | | Disabled population | | | Percent Disabled | | |
|---|---|---|---|---|---|---|---|---|---|
| | Total | Male | Female | Total | Male | Female | Total | Male | Female |
| **Zambia Total** | | | | | | | | | |
| **Total** | **12,526,314** | **6,117,253** | **6,409,061** | **251,427** | **130,302** | **121,125** | **2.0** | **2.1** | **1.9** |
| 0 - 4 | 2,214,887 | 1,102,272 | 1,112,615 | 12,971 | 7,463 | 5,508 | 0.6 | 0.7 | 0.5 |
| 5-9 | 1,856,336 | 924,395 | 931,941 | 20,134 | 11,403 | 8,731 | 1.1 | 1.2 | 0.9 |
| 10-14 | 1,699,042 | 840,834 | 858,208 | 24,630 | 13,507 | 11,123 | 1.4 | 1.6 | 1.3 |
| 15 - 19 | 1,427,884 | 692,859 | 735,025 | 21,279 | 11,688 | 9,591 | 1.5 | 1.7 | 1.3 |
| 20 - 24 | 1,117,476 | 504,876 | 612,600 | 15,835 | 8,369 | 7,466 | 1.4 | 1.7 | 1.2 |
| 25 - 29 | 1,006,244 | 464,493 | 541,751 | 15,115 | 8,010 | 7,105 | 1.5 | 1.7 | 1.3 |
| 30 - 34 | 800,770 | 397,694 | 403,076 | 14,742 | 8,017 | 6,725 | 1.8 | 2.0 | 1.7 |
| 35 - 39 | 647,813 | 332,961 | 314,852 | 14,847 | 8,078 | 6,769 | 2.3 | 2.4 | 2.1 |
| 40 - 44 | 447,584 | 232,253 | 215,331 | 14,146 | 7,545 | 6,601 | 3.2 | 3.2 | 3.1 |
| 45 - 49 | 354,989 | 174,833 | 180,156 | 14,095 | 6,979 | 7,116 | 4.0 | 4.0 | 3.9 |
| 50 - 54 | 270,254 | 128,696 | 141,558 | 13,639 | 6,546 | 7,093 | 5.0 | 5.1 | 5.0 |
| 55 - 59 | 184,828 | 90,037 | 94,791 | 11,083 | 5,254 | 5,829 | 6.0 | 5.8 | 6.1 |
| 60 - 64 | 161,875 | 73,419 | 88,456 | 12,894 | 5,613 | 7,281 | 8.0 | 7.6 | 8.2 |
| 65 - 69 | 118,582 | 53,695 | 64,887 | 11,669 | 5,077 | 6,592 | 9.8 | 9.5 | 10.2 |
| 70 - 74 | 90,802 | 41,929 | 48,873 | 11,862 | 5,356 | 6,506 | 13.1 | 12.8 | 13.3 |
| 75 - 79 | 61,420 | 30,662 | 30,758 | 9,452 | 4,843 | 4,609 | 15.4 | 15.8 | 15.0 |
| 80 - 84 | 32,821 | 16,045 | 16,776 | 6,047 | 3,085 | 2,962 | 18.4 | 19.2 | 17.7 |
| 85 - 89 | 18,655 | 9,347 | 9,308 | 3,679 | 1,979 | 1,700 | 19.7 | 21.2 | 18.3 |
| 90 - 94 | 6,308 | 3,152 | 3,156 | 1,457 | 784 | 673 | 23.1 | 24.9 | 21.3 |
| 95+ | 7,744 | 2,801 | 4,943 | 1,851 | 706 | 1,145 | 23.9 | 25.2 | 23.2 |
| **Zambia Rural** | | | | | | | | | |
| **Total** | **7,505,292** | **3,664,349** | **3,840,943** | **181,856** | **94,268** | **87,588** | **2.4** | **2.6** | **2.3** |
| 0 - 4 | 1,454,385 | 724,158 | 730,227 | 9,740 | 5,648 | 4,092 | 0.7 | 0.8 | 0.6 |
| 5-9 | 1,212,247 | 608,241 | 604,006 | 15,123 | 8,626 | 6,497 | 1.2 | 1.4 | 1.1 |
| 10-14 | 1,045,977 | 529,978 | 515,999 | 18,088 | 10,055 | 8,033 | 1.7 | 1.9 | 1.6 |
| 15 - 19 | 802,997 | 397,738 | 405,259 | 14,771 | 8,296 | 6,475 | 1.8 | 2.1 | 1.6 |
| 20 - 24 | 598,270 | 269,368 | 328,902 | 10,590 | 5,630 | 4,960 | 1.8 | 2.1 | 1.5 |
| 25 - 29 | 529,481 | 241,136 | 288,345 | 10,079 | 5,340 | 4,739 | 1.9 | 2.2 | 1.6 |
| 30 - 34 | 419,932 | 203,507 | 216,425 | 9,860 | 5,325 | 4,535 | 2.3 | 2.6 | 2.1 |
| 35 - 39 | 351,970 | 173,650 | 178,320 | 9,968 | 5,319 | 4,649 | 2.8 | 3.1 | 2.6 |
| 40 - 44 | 254,964 | 127,044 | 127,920 | 9,884 | 5,205 | 4,679 | 3.9 | 4.1 | 3.7 |
| 45 - 49 | 209,868 | 100,903 | 108,965 | 10,073 | 4,978 | 5,095 | 4.8 | 4.9 | 4.7 |
| 50 - 54 | 162,395 | 75,868 | 86,527 | 9,787 | 4,699 | 5,088 | 6.0 | 6.2 | 5.9 |
| 55 - 59 | 111,511 | 52,225 | 59,286 | 7,900 | 3,690 | 4,210 | 7.1 | 7.1 | 7.1 |
| 60 - 64 | 107,038 | 45,761 | 61,277 | 9,649 | 4,087 | 5,562 | 9.0 | 8.9 | 9.1 |
| 65 - 69 | 83,157 | 36,611 | 46,546 | 8,927 | 3,850 | 5,077 | 10.7 | 10.5 | 10.9 |
| 70 - 74 | 66,388 | 30,553 | 35,835 | 9,378 | 4,213 | 5,165 | 14.1 | 13.8 | 14.4 |
| 75 - 79 | 45,681 | 23,394 | 22,287 | 7,560 | 3,923 | 3,637 | 16.5 | 16.8 | 16.3 |
| 80 - 84 | 24,318 | 12,247 | 12,071 | 4,775 | 2,486 | 2,289 | 19.6 | 20.3 | 19.0 |
| 85 - 89 | 13,883 | 7,238 | 6,645 | 2,967 | 1,646 | 1,321 | 21.4 | 22.7 | 19.9 |
| 90 - 94 | 4,818 | 2,496 | 2,322 | 1,192 | 653 | 539 | 24.7 | 26.2 | 23.2 |
| 95+ | 6,012 | 2,233 | 3,779 | 1,545 | 599 | 946 | 25.7 | 26.8 | 25.0 |

## Table F2: Total Population (De facto) and Total Disabled Population by 5 Year Age Group, Sex and Rural/Urban, Zambia, 2010

| Age Group and Rural/Urban | Population | | | Disabled population | | | Percent Disabled | | |
|---|---|---|---|---|---|---|---|---|---|
| | Total | Male | Female | Total | Male | Female | Total | Male | Female |
| **Zambia Urban** | | | | | | | | | |
| **Total** | **5,021,022** | **2,452,904** | **2,568,118** | **69,571** | **36,034** | **33,537** | **1.4** | **1.5** | **1.3** |
| 0 - 4 | 760,502 | 378,114 | 382,388 | 3,231 | 1,815 | 1,416 | 0.4 | 0.5 | 0.4 |
| 5-9 | 644,089 | 316,154 | 327,935 | 5,011 | 2,777 | 2,234 | 0.8 | 0.9 | 0.7 |
| 10-14 | 653,065 | 310,856 | 342,209 | 6,542 | 3,452 | 3,090 | 1.0 | 1.1 | 0.9 |
| 15 - 19 | 624,887 | 295,121 | 329,766 | 6,508 | 3,392 | 3,116 | 1.0 | 1.1 | 0.9 |
| 20 - 24 | 519,206 | 235,508 | 283,698 | 5,245 | 2,739 | 2,506 | 1.0 | 1.2 | 0.9 |
| 25 - 29 | 476,763 | 223,357 | 253,406 | 5,036 | 2,670 | 2,366 | 1.1 | 1.2 | 0.9 |
| 30 - 34 | 380,838 | 194,187 | 186,651 | 4,882 | 2,692 | 2,190 | 1.3 | 1.4 | 1.2 |
| 35 - 39 | 295,843 | 159,311 | 136,532 | 4,879 | 2,759 | 2,120 | 1.6 | 1.7 | 1.6 |
| 40 - 44 | 192,620 | 105,209 | 87,411 | 4,262 | 2,340 | 1,922 | 2.2 | 2.2 | 2.2 |
| 45 - 49 | 145,121 | 73,930 | 71,191 | 4,022 | 2,001 | 2,021 | 2.8 | 2.7 | 2.8 |
| 50 - 54 | 107,859 | 52,828 | 55,031 | 3,852 | 1,847 | 2,005 | 3.6 | 3.5 | 3.6 |
| 55 - 59 | 73,317 | 37,812 | 35,505 | 3,183 | 1,564 | 1,619 | 4.3 | 4.1 | 4.6 |
| 60 - 64 | 54,837 | 27,658 | 27,179 | 3,245 | 1,526 | 1,719 | 5.9 | 5.5 | 6.3 |
| 65 - 69 | 35,425 | 17,084 | 18,341 | 2,742 | 1,227 | 1,515 | 7.7 | 7.2 | 8.3 |
| 70 - 74 | 24,414 | 11,376 | 13,038 | 2,484 | 1,143 | 1,341 | 10.2 | 10.0 | 10.3 |
| 75 - 79 | 15,739 | 7,268 | 8,471 | 1,892 | 920 | 972 | 12.0 | 12.7 | 11.5 |
| 80 - 84 | 8,503 | 3,798 | 4,705 | 1,272 | 599 | 673 | 15.0 | 15.8 | 14.3 |
| 85 - 89 | 4,772 | 2,109 | 2,663 | 712 | 333 | 379 | 14.9 | 15.8 | 14.2 |
| 90 - 94 | 1,490 | 656 | 834 | 265 | 131 | 134 | 17.8 | 20.0 | 16.1 |
| 95+ | 1,732 | 568 | 1,164 | 306 | 107 | 199 | 17.7 | 18.8 | 17.1 |

## Table F3: Disabled Household Heads by Sex of Household Head, Number of Household Members and Employment Status, Rural/Urban, Zambia 2010

| Sex of Household Head and Number of Household Members | Disabled Household Heads | Employment Status | | | |
|---|---|---|---|---|---|
| | | An employer | An employee | Self employed | An unpaid family worker |
| **Zambia Total** | | | | | |
| **Total** | **59,020** | **429** | **8,616** | **37,752** | **12,223** |
| 1 | 7,733 | 39 | 1,187 | 4,899 | 1,608 |
| 2 | 7,267 | 52 | 837 | 4,800 | 1,578 |
| 3 | 7,368 | 40 | 958 | 4,755 | 1,615 |
| 4 | 7,797 | 64 | 1,085 | 5,010 | 1,638 |
| 5 | 7,498 | 60 | 1,112 | 4,738 | 1,588 |
| 6 | 6,864 | 51 | 1,083 | 4,365 | 1,365 |
| 7 | 5,796 | 48 | 934 | 3,675 | 1,139 |
| 8 | 7,008 | 55 | 1,076 | 4,499 | 1,378 |
| 9+ | 1,689 | 20 | 344 | 1,011 | 314 |
| **Male** | | | | | |
| **Total** | **40,752** | **352** | **7,127** | **25,629** | **7,644** |
| 1 | 4,117 | 28 | 953 | 2,459 | 677 |
| 2 | 4,407 | 36 | 629 | 2,886 | 856 |
| 3 | 4,496 | 32 | 724 | 2,862 | 878 |
| 4 | 5,152 | 50 | 854 | 3,250 | 998 |
| 5 | 5,344 | 51 | 903 | 3,351 | 1,039 |
| 6 | 5,245 | 45 | 945 | 3,297 | 958 |
| 7 | 4,688 | 41 | 838 | 2,937 | 872 |
| 8 | 5,886 | 50 | 963 | 3,753 | 1,120 |
| 9+ | 1,417 | 19 | 318 | 834 | 246 |
| **Female** | | | | | |
| **Total** | **18,268** | **77** | **1,489** | **12,123** | **4,579** |
| 1 | 3,616 | 11 | 234 | 2,440 | 931 |
| 2 | 2,860 | 16 | 208 | 1,914 | 722 |
| 3 | 2,872 | 8 | 234 | 1,893 | 737 |
| 4 | 2,645 | 14 | 231 | 1,760 | 640 |
| 5 | 2,154 | 9 | 209 | 1,387 | 549 |
| 6 | 1,619 | 6 | 138 | 1,068 | 407 |
| 7 | 1,108 | 7 | 96 | 738 | 267 |
| 8 | 1,122 | 5 | 113 | 746 | 258 |
| 9+ | 272 | 1 | 26 | 177 | 68 |

## Table F3: Disabled Household Heads by Sex of Household Head, Number of Household Members and Employment Status, Rural, Zambia 2010

| Sex of Household Head and Number of Household Members | Disabled Household Heads | Employment Status | | | |
|---|---|---|---|---|---|
| | | An employer | An employee | Self employed | An unpaid family worker |
| **Zambia Rural** | | | | | |
| **Total** | **46,588** | **251** | **3,137** | **31,896** | **11,304** |
| 1 | 6,160 | 27 | 491 | 4,144 | 1,498 |
| 2 | 5,863 | 37 | 277 | 4,086 | 1,463 |
| 3 | 5,833 | 24 | 317 | 3,991 | 1,501 |
| 4 | 6,133 | 37 | 356 | 4,232 | 1,508 |
| 5 | 5,881 | 24 | 409 | 3,985 | 1,463 |
| 6 | 5,354 | 33 | 369 | 3,689 | 1,263 |
| 7 | 4,525 | 29 | 353 | 3,094 | 1,049 |
| 8 | 5,606 | 33 | 448 | 3,851 | 1,274 |
| 9+ | 1,233 | 7 | 117 | 824 | 285 |
| **Male** | | | | | |
| **Total** | **31,709** | **204** | **2,696** | **21,725** | **7,084** |
| 1 | 3,050 | 19 | 416 | 1,988 | 627 |
| 2 | 3,497 | 26 | 210 | 2,469 | 792 |
| 3 | 3,494 | 19 | 257 | 2,410 | 808 |
| 4 | 3,994 | 29 | 295 | 2,752 | 918 |
| 5 | 4,148 | 19 | 347 | 2,820 | 962 |
| 6 | 4,077 | 28 | 335 | 2,822 | 892 |
| 7 | 3,659 | 27 | 324 | 2,504 | 804 |
| 8 | 4,745 | 30 | 404 | 3,262 | 1,049 |
| 9+ | 1,045 | 7 | 108 | 698 | 232 |
| **Female** | | | | | |
| **Total** | **14,879** | **47** | **441** | **10,171** | **4,220** |
| 1 | 3,110 | 8 | 75 | 2,156 | 871 |
| 2 | 2,366 | 11 | 67 | 1,617 | 671 |
| 3 | 2,339 | 5 | 60 | 1,581 | 693 |
| 4 | 2,139 | 8 | 61 | 1,480 | 590 |
| 5 | 1,733 | 5 | 62 | 1,165 | 501 |
| 6 | 1,277 | 5 | 34 | 867 | 371 |
| 7 | 866 | 2 | 29 | 590 | 245 |
| 8 | 861 | 3 | 44 | 589 | 225 |
| 9+ | 188 | - | 9 | 126 | 53 |

## Table F3: Disabled Household Heads by Sex of Household Head, Number of Household Members and Employment Status, Urban, Zambia 2010

| Sex of Household Head and Number of Household Members | Disabled Household Heads | Employment Status | | | |
|---|---|---|---|---|---|
| | | An employer | An employee | Self employed | An unpaid family worker |
| **Zambia Urban** | | | | | |
| **Total** | **12,432** | **178** | **5,479** | **5,856** | **919** |
| 1 | 1,573 | 12 | 696 | 755 | 110 |
| 2 | 1,404 | 15 | 560 | 714 | 115 |
| 3 | 1,535 | 16 | 641 | 764 | 114 |
| 4 | 1,664 | 27 | 729 | 778 | 130 |
| 5 | 1,617 | 36 | 703 | 753 | 125 |
| 6 | 1,510 | 18 | 714 | 676 | 102 |
| 7 | 1,271 | 19 | 581 | 581 | 90 |
| 8 | 1,402 | 22 | 628 | 648 | 104 |
| 9+ | 456 | 13 | 227 | 187 | 29 |
| **Male** | | | | | |
| **Total** | **9,043** | **148** | **4,431** | **3,904** | **560** |
| 1 | 1,067 | 9 | 537 | 471 | 50 |
| 2 | 910 | 10 | 419 | 417 | 64 |
| 3 | 1,002 | 13 | 467 | 452 | 70 |
| 4 | 1,158 | 21 | 559 | 498 | 80 |
| 5 | 1,196 | 32 | 556 | 531 | 77 |
| 6 | 1,168 | 17 | 610 | 475 | 66 |
| 7 | 1,029 | 14 | 514 | 433 | 68 |
| 8 | 1,141 | 20 | 559 | 491 | 71 |
| 9+ | 372 | 12 | 210 | 136 | 14 |
| **Female** | | | | | |
| **Total** | **3,389** | **30** | **1,048** | **1,952** | **359** |
| 1 | 506 | 3 | 159 | 284 | 60 |
| 2 | 494 | 5 | 141 | 297 | 51 |
| 3 | 533 | 3 | 174 | 312 | 44 |
| 4 | 506 | 6 | 170 | 280 | 50 |
| 5 | 421 | 4 | 147 | 222 | 48 |
| 6 | 342 | 1 | 104 | 201 | 36 |
| 7 | 242 | 5 | 67 | 148 | 22 |
| 8 | 261 | 2 | 69 | 157 | 33 |
| 9+ | 84 | 1 | 17 | 51 | 15 |

## Table F4: Disabled Population (De Facto) 5 Years and Older by 5 Year Age Group, Sex and School Attendance, Rural/Urban, Zambia 2010

| Age Group and Sex | Disabled Population 5 Years and Older | Never attended | Ever attended school | | |
|---|---|---|---|---|---|
| | | | Total | Currently attending | Currently not attending |
| **Zambia Total** | **238,456** | **82,143** | **156,313** | **39,469** | **116,844** |
| Male | 122,839 | 33,654 | 89,185 | 22,589 | 66,596 |
| Female | 115,617 | 48,489 | 67,128 | 16,880 | 50,248 |
| **5 - 9** | | | | | |
| Total | 20,134 | 12,268 | 7,866 | 7,221 | 645 |
| Male | 11,403 | 7,105 | 4,298 | 3,944 | 354 |
| Female | 8,731 | 5,163 | 3,568 | 3,277 | 291 |
| **10 - 14** | | | | | |
| Total | 24,630 | 6,255 | 18,375 | 16,435 | 1,940 |
| Male | 13,507 | 3,466 | 10,041 | 9,030 | 1,011 |
| Female | 11,123 | 2,789 | 8,334 | 7,405 | 929 |
| **15 - 19** | | | | | |
| Total | 21,279 | 5,038 | 16,241 | 10,705 | 5,536 |
| Male | 11,688 | 2,526 | 9,162 | 6,498 | 2,664 |
| Female | 9,591 | 2,512 | 7,079 | 4,207 | 2,872 |
| **20 - 24** | | | | | |
| Total | 15,835 | 4,590 | 11,245 | 2,367 | 8,878 |
| Male | 8,369 | 2,108 | 6,261 | 1,589 | 4,672 |
| Female | 7,466 | 2,482 | 4,984 | 778 | 4,206 |
| **25 - 29** | | | | | |
| Total | 15,115 | 4,652 | 10,463 | 618 | 9,845 |
| Male | 8,010 | 2,137 | 5,873 | 358 | 5,515 |
| Female | 7,105 | 2,515 | 4,590 | 260 | 4,330 |
| **30 - 34** | | | | | |
| Total | 14,742 | 4,036 | 10,706 | 396 | 10,310 |
| Male | 8,017 | 1,854 | 6,163 | 214 | 5,949 |
| Female | 6,725 | 2,182 | 4,543 | 182 | 4,361 |
| **35 - 39** | | | | | |
| Total | 14,847 | 3,955 | 10,892 | 326 | 10,566 |
| Male | 8,078 | 1,688 | 6,390 | 162 | 6,228 |
| Female | 6,769 | 2,267 | 4,502 | 164 | 4,338 |
| **40 - 44** | | | | | |
| Total | 14,146 | 3,697 | 10,449 | 283 | 10,166 |
| Male | 7,545 | 1,574 | 5,971 | 162 | 5,809 |
| Female | 6,601 | 2,123 | 4,478 | 121 | 4,357 |
| **45 - 49** | | | | | |
| Total | 14,095 | 3,688 | 10,407 | 228 | 10,179 |
| Male | 6,979 | 1,388 | 5,591 | 127 | 5,464 |
| Female | 7,116 | 2,300 | 4,816 | 101 | 4,715 |
| **50 - 54** | | | | | |
| Total | 13,639 | 3,665 | 9,974 | 193 | 9,781 |
| Male | 6,546 | 1,121 | 5,425 | 103 | 5,322 |
| Female | 7,093 | 2,544 | 4,549 | 90 | 4,459 |
| **55 - 59** | | | | | |
| Total | 11,083 | 3,158 | 7,925 | 139 | 7,786 |
| Male | 5,254 | 917 | 4,337 | 83 | 4,254 |
| Female | 5,829 | 2,241 | 3,588 | 56 | 3,532 |
| **60 - 64** | | | | | |
| Total | 12,894 | 4,863 | 8,031 | 120 | 7,911 |
| Male | 5,613 | 1,158 | 4,455 | 67 | 4,388 |
| Female | 7,281 | 3,705 | 3,576 | 53 | 3,523 |
| **65 - 69** | | | | | |
| Total | 11,669 | 4,978 | 6,691 | 121 | 6,570 |
| Male | 5,077 | 1,202 | 3,875 | 64 | 3,811 |
| Female | 6,592 | 3,776 | 2,816 | 57 | 2,759 |
| **70 - 74** | | | | | |
| Total | 11,862 | 5,657 | 6,205 | 108 | 6,097 |
| Male | 5,356 | 1,538 | 3,818 | 60 | 3,758 |
| Female | 6,506 | 4,119 | 2,387 | 48 | 2,339 |
| **75+** | | | | | |
| Total | 22,486 | 11,643 | 10,843 | 209 | 10,634 |
| Male | 11,397 | 3,872 | 7,525 | 128 | 7,397 |
| Female | 11,089 | 7,771 | 3,318 | 81 | 3,237 |

## Table F4: Disabled Population (De Facto) 5 Years and Older by 5 Year Age Group, Sex and School Attendance, Rural, Zambia 2010

| Age Group and Sex | Disabled Population 5 Years and Older | Never attended | Ever attended school | | |
|---|---|---|---|---|---|
| | | | Total | Currently attending | Currently not attending |
| **Zambia Rural** | | | | | |
| **Total** | **172,116** | **68,412** | **103,704** | **26,308** | **77,396** |
| Male | 88,620 | 28,161 | 60,459 | 15,463 | 44,996 |
| Female | 83,496 | 40,251 | 43,245 | 10,845 | 32,400 |
| **5 - 9** | | | | | |
| **Total** | **15,123** | **9,901** | **5,222** | **4,830** | **392** |
| Male | 8,626 | 5,764 | 2,862 | 2,644 | 218 |
| Female | 6,497 | 4,137 | 2,360 | 2,186 | 174 |
| **10 - 14** | | | | | |
| **Total** | **18,088** | **5,061** | **13,027** | **11,657** | **1,370** |
| Male | 10,055 | 2,805 | 7,250 | 6,529 | 721 |
| Female | 8,033 | 2,256 | 5,777 | 5,128 | 649 |
| **15 - 19** | | | | | |
| **Total** | **14,771** | **4,076** | **10,695** | **6,902** | **3,793** |
| Male | 8,296 | 2,045 | 6,251 | 4,413 | 1,838 |
| Female | 6,475 | 2,031 | 4,444 | 2,489 | 1,955 |
| **20 - 24** | | | | | |
| **Total** | **10,590** | **3,739** | **6,851** | **1,333** | **5,518** |
| Male | 5,630 | 1,700 | 3,930 | 955 | 2,975 |
| Female | 4,960 | 2,039 | 2,921 | 378 | 2,543 |
| **25 - 29** | | | | | |
| **Total** | **10,079** | **3,855** | **6,224** | **314** | **5,910** |
| Male | 5,340 | 1,746 | 3,594 | 211 | 3,383 |
| Female | 4,739 | 2,109 | 2,630 | 103 | 2,527 |
| **30 - 34** | | | | | |
| **Total** | **9,860** | **3,429** | **6,431** | **192** | **6,239** |
| Male | 5,325 | 1,566 | 3,759 | 106 | 3,653 |
| Female | 4,535 | 1,863 | 2,672 | 86 | 2,586 |
| **35 - 39** | | | | | |
| **Total** | **9,968** | **3,321** | **6,647** | **185** | **6,462** |
| Male | 5,319 | 1,411 | 3,908 | 93 | 3,815 |
| Female | 4,649 | 1,910 | 2,739 | 92 | 2,647 |
| **40 - 44** | | | | | |
| **Total** | **9,884** | **3,160** | **6,724** | **152** | **6,572** |
| Male | 5,205 | 1,356 | 3,849 | 88 | 3,761 |
| Female | 4,679 | 1,804 | 2,875 | 64 | 2,811 |
| **45 - 49** | | | | | |
| **Total** | **10,073** | **3,190** | **6,883** | **129** | **6,754** |
| Male | 4,978 | 1,216 | 3,762 | 77 | 3,685 |
| Female | 5,095 | 1,974 | 3,121 | 52 | 3,069 |
| **50 - 54** | | | | | |
| **Total** | **9,787** | **3,142** | **6,645** | **123** | **6,522** |
| Male | 4,699 | 974 | 3,725 | 63 | 3,662 |
| Female | 5,088 | 2,168 | 2,920 | 60 | 2,860 |
| **55 - 59** | | | | | |
| **Total** | **7,900** | **2,664** | **5,236** | **82** | **5,154** |
| Male | 3,690 | 799 | 2,891 | 47 | 2,844 |
| Female | 4,210 | 1,865 | 2,345 | 35 | 2,310 |
| **60 - 64** | | | | | |
| **Total** | **9,649** | **4,131** | **5,518** | **80** | **5,438** |
| Male | 4,087 | 1,019 | 3,068 | 46 | 3,022 |
| Female | 5,562 | 3,112 | 2,450 | 34 | 2,416 |
| **65 - 69** | | | | | |
| **Total** | **8,927** | **4,196** | **4,731** | **87** | **4,644** |
| Male | 3,850 | 1,057 | 2,793 | 49 | 2,744 |
| Female | 5,077 | 3,139 | 1,938 | 38 | 1,900 |
| **70 - 74** | | | | | |
| **Total** | **9,378** | **4,781** | **4,597** | **80** | **4,517** |
| Male | 4,213 | 1,322 | 2,891 | 47 | 2,844 |
| Female | 5,165 | 3,459 | 1,706 | 33 | 1,673 |
| **75+** | | | | | |
| **Total** | **18,039** | **9,766** | **8,273** | **162** | **8,111** |
| Male | 9,307 | 3,381 | 5,926 | 95 | 5,831 |
| Female | 8,732 | 6,385 | 2,347 | 67 | 2,280 |

## Table F4: Disabled Population (De Facto) 5 Years and Older by 5 Year Age Group, Sex and School Attendance, Urban, Zambia 2010

| Age Group and Sex | Disabled Population 5 Years and Older | Never attended | Ever attended school | | |
|---|---|---|---|---|---|
| | | | Total | Currently attending | Currently not attending |
| **Zambia Urban** | | | | | |
| **Total** | **66,340** | **13,731** | **52,609** | **13,161** | **39,448** |
| Male | 34,219 | 5,493 | 28,726 | 7,126 | 21,600 |
| Female | 32,121 | 8,238 | 23,883 | 6,035 | 17,848 |
| **5-9** | | | | | |
| Total | 5,011 | 2,367 | 2,644 | 2,391 | 253 |
| Male | 2,777 | 1,341 | 1,436 | 1,300 | 136 |
| Female | 2,234 | 1,026 | 1,208 | 1,091 | 117 |
| **10 - 14** | | | | | |
| **Total** | **6,542** | **1,194** | **5,348** | **4,778** | **570** |
| Male | 3,452 | 661 | 2,791 | 2,501 | 290 |
| Female | 3,090 | 533 | 2,557 | 2,277 | 280 |
| **15 - 19** | | | | | |
| **Total** | **6,508** | **962** | **5,546** | **3,803** | **1,743** |
| Male | 3,392 | 481 | 2,911 | 2,085 | 826 |
| Female | 3,116 | 481 | 2,635 | 1,718 | 917 |
| **20 - 24** | | | | | |
| **Total** | **5,245** | **851** | **4,394** | **1,034** | **3,360** |
| Male | 2,739 | 408 | 2,331 | 634 | 1,697 |
| Female | 2,506 | 443 | 2,063 | 400 | 1,663 |
| **25 - 29** | | | | | |
| **Total** | **5,036** | **797** | **4,239** | **304** | **3,935** |
| Male | 2,670 | 391 | 2,279 | 147 | 2,132 |
| Female | 2,366 | 406 | 1,960 | 157 | 1,803 |
| **30 - 34** | | | | | |
| **Total** | **4,882** | **607** | **4,275** | **204** | **4,071** |
| Male | 2,692 | 288 | 2,404 | 108 | 2,296 |
| Female | 2,190 | 319 | 1,871 | 96 | 1,775 |
| **35 - 39** | | | | | |
| **Total** | **4,879** | **634** | **4,245** | **141** | **4,104** |
| Male | 2,759 | 277 | 2,482 | 69 | 2,413 |
| Female | 2,120 | 357 | 1,763 | 72 | 1,691 |
| **40 - 44** | | | | | |
| **Total** | **4,262** | **537** | **3,725** | **131** | **3,594** |
| Male | 2,340 | 218 | 2,122 | 74 | 2,048 |
| Female | 1,922 | 319 | 1,603 | 57 | 1,546 |
| **45 - 49** | | | | | |
| **Total** | **4,022** | **498** | **3,524** | **99** | **3,425** |
| Male | 2,001 | 172 | 1,829 | 50 | 1,779 |
| Female | 2,021 | 326 | 1,695 | 49 | 1,646 |
| **50 - 54** | | | | | |
| **Total** | **3,852** | **523** | **3,329** | **70** | **3,259** |
| Male | 1,847 | 147 | 1,700 | 40 | 1,660 |
| Female | 2,005 | 376 | 1,629 | 30 | 1,599 |
| **55 - 59** | | | | | |
| **Total** | **3,183** | **494** | **2,689** | **57** | **2,632** |
| Male | 1,564 | 118 | 1,446 | 36 | 1,410 |
| Female | 1,619 | 376 | 1,243 | 21 | 1,222 |
| **60 - 64** | | | | | |
| **Total** | **3,245** | **732** | **2,513** | **40** | **2,473** |
| Male | 1,526 | 139 | 1,387 | 21 | 1,366 |
| Female | 1,719 | 593 | 1,126 | 19 | 1,107 |
| **65 - 69** | | | | | |
| **Total** | **2,742** | **782** | **1,960** | **34** | **1,926** |
| Male | 1,227 | 145 | 1,082 | 15 | 1,067 |
| Female | 1,515 | 637 | 878 | 19 | 859 |
| **70 - 74** | | | | | |
| **Total** | **2,484** | **876** | **1,608** | **28** | **1,580** |
| Male | 1,143 | 216 | 927 | 13 | 914 |
| Female | 1,341 | 660 | 681 | 15 | 666 |
| **75+** | | | | | |
| **Total** | **4,447** | **1,877** | **2,570** | **47** | **2,523** |
| Male | 2,090 | 491 | 1,599 | 33 | 1,566 |
| Female | 2,357 | 1,386 | 971 | 14 | 957 |

## Table F5: Disabled Population (De Facto) 5 Years and Older by 5 Year Age Group and Highest Level of Education Completed, Rural/Urban, Zambia 2010

| Age Group and Rural/ Urban | Disabled Popula-tion 5 Years and Older | Never attended | Total Ever Attended | Highest Level of Eduction Completed | | | | | | | | | | | | | | | |
|---|---|---|---|---|---|---|---|---|---|---|---|---|---|---|---|---|---|---|---|
| | | | | None | Primary School | | | | | | | | Secondary School | | | | | | Tertiary |
| | | | | | 1 | 2 | 3 | 4 | 5 | 6 | 7 | Total 1-7 | 8 | 9 | 10 | 11 | 12 | Total 8-12 | |
| **Zambia Total** | | | | | | | | | | | | | | | | | | | |
| **Total** | **238,456** | **82,143** | **156,313** | **3,984** | **10,330** | **13,788** | **13,584** | **15,328** | **13,020** | **15,043** | **23,130** | **104,223** | **8,817** | **12,245** | **4,688** | **2,546** | **7,738** | **36,034** | **12,072** |
| 5-9 | 20,134 | 12,268 | 7,866 | 2,129 | 3,010 | 1,745 | 741 | 191 | 50 | 0 | 0 | 5,737 | 0 | 0 | 0 | 0 | 0 | 0 | 0 |
| 10-14 | 24,630 | 6,255 | 18,375 | 661 | 1,826 | 3,060 | 3,654 | 3,360 | 2,663 | 1,706 | 947 | 17,216 | 355 | 126 | 17 | 0 | 0 | 498 | 0 |
| 15-19 | 21,279 | 5,038 | 16,241 | 192 | 371 | 630 | 942 | 1,323 | 1,822 | 2,386 | 3,010 | 10,484 | 2,295 | 1,602 | 688 | 487 | 422 | 5,494 | 71 |
| 20 - 24 | 15,835 | 4,590 | 11,245 | 128 | 269 | 464 | 526 | 731 | 785 | 1,036 | 1,609 | 5,420 | 1,300 | 1,505 | 524 | 501 | 1,397 | 5,227 | 470 |
| 25 - 29 | 15,115 | 4,652 | 10,463 | 105 | 304 | 509 | 598 | 750 | 847 | 989 | 1,646 | 5,643 | 794 | 1,203 | 293 | 269 | 1,161 | 3,720 | 995 |
| 30 - 34 | 14,742 | 4,036 | 10,706 | 86 | 294 | 486 | 626 | 771 | 775 | 1,070 | 1,958 | 5,980 | 731 | 1,446 | 274 | 185 | 826 | 3,462 | 1,178 |
| 35 - 39 | 14,847 | 3,955 | 10,892 | 63 | 253 | 482 | 581 | 773 | 792 | 1,143 | 2,109 | 6,133 | 745 | 1,461 | 317 | 176 | 777 | 3,476 | 1,220 |
| 40 - 44 | 14,146 | 3,697 | 10,449 | 51 | 249 | 446 | 530 | 759 | 721 | 1,045 | 2,346 | 6,096 | 583 | 1,206 | 373 | 170 | 680 | 3,012 | 1,290 |
| 45 - 49 | 14,095 | 3,688 | 10,407 | 50 | 255 | 454 | 580 | 820 | 720 | 1,105 | 2,466 | 6,400 | 454 | 870 | 619 | 157 | 602 | 2,702 | 1,255 |
| 50 - 54 | 13,639 | 3,665 | 9,974 | 46 | 271 | 527 | 602 | 889 | 733 | 985 | 2,295 | 6,302 | 362 | 717 | 611 | 150 | 505 | 2,345 | 1,281 |
| 55 - 59 | 11,083 | 3,158 | 7,925 | 41 | 296 | 534 | 580 | 752 | 538 | 735 | 1,613 | 5,048 | 286 | 606 | 357 | 124 | 362 | 1,735 | 1,101 |
| 60 - 64 | 12,894 | 4,863 | 8,031 | 89 | 541 | 881 | 757 | 929 | 620 | 797 | 999 | 5,524 | 278 | 514 | 225 | 93 | 318 | 1,428 | 990 |
| 65 - 69 | 11,669 | 4,978 | 6,691 | 70 | 553 | 876 | 765 | 928 | 530 | 632 | 675 | 4,959 | 198 | 333 | 129 | 62 | 218 | 940 | 722 |
| 70 - 74 | 11,862 | 5,657 | 6,205 | 88 | 630 | 929 | 726 | 866 | 529 | 578 | 558 | 4,816 | 162 | 242 | 99 | 71 | 177 | 751 | 550 |
| 75+ | 22,486 | 11,643 | 11,843 | 185 | 1,208 | 1,765 | 1,376 | 1,486 | 895 | 836 | 899 | 8,465 | 274 | 414 | 162 | 101 | 293 | 1,244 | 949 |
| **Zambia Rural** | | | | | | | | | | | | | | | | | | | |
| **Total** | **172,116** | **68412** | **103,704** | **2,669** | **8,182** | **11,041** | **10,600** | **12,010** | **9,722** | **10,933** | **15,943** | **78,431** | **5,561** | **6,576** | **2,334** | **1,113** | **2,706** | **18,290** | **4,314** |
| 5-9 | 15,123 | 9901 | 5,222 | 1,323 | 2,142 | 1,192 | 425 | 109 | 31 | 0 | 0 | 3,899 | 0 | 0 | 0 | 0 | 0 | 0 | 0 |
| 10-14 | 18,088 | 5061 | 13,027 | 469 | 1,524 | 2,449 | 2,763 | 2,423 | 1,740 | 994 | 482 | 12,375 | 141 | 38 | 4 | 0 | 0 | 183 | 0 |
| 15-19 | 14,771 | 4076 | 10,695 | 117 | 300 | 522 | 755 | 1,064 | 1,427 | 1,773 | 2,085 | 7,926 | 1,446 | 771 | 209 | 100 | 102 | 2,628 | 24 |
| 20 - 24 | 10,590 | 3739 | 6,851 | 79 | 229 | 381 | 423 | 572 | 586 | 753 | 1,098 | 4,042 | 863 | 906 | 252 | 196 | 409 | 2,626 | 104 |
| 25 - 29 | 10,079 | 3855 | 6,224 | 63 | 265 | 430 | 479 | 566 | 625 | 717 | 1,062 | 4,144 | 505 | 637 | 143 | 121 | 352 | 1,758 | 259 |
| 30 - 34 | 9,860 | 3429 | 6,431 | 69 | 250 | 408 | 518 | 618 | 604 | 756 | 1,265 | 4,419 | 441 | 695 | 125 | 80 | 266 | 1,607 | 336 |
| 35 - 39 | 9,968 | 3321 | 6,647 | 49 | 217 | 424 | 477 | 626 | 629 | 842 | 1,390 | 4,605 | 445 | 737 | 134 | 92 | 243 | 1,651 | 342 |
| 40 - 44 | 9,884 | 3160 | 6,724 | 42 | 217 | 394 | 446 | 614 | 592 | 829 | 1,693 | 4,785 | 367 | 641 | 201 | 88 | 252 | 1,549 | 348 |
| 45 - 49 | 10,073 | 3190 | 6,883 | 40 | 214 | 391 | 477 | 690 | 570 | 864 | 1,800 | 5,006 | 300 | 498 | 352 | 77 | 239 | 1,466 | 371 |
| 50 - 54 | 9,787 | 3142 | 6,645 | 34 | 227 | 429 | 477 | 738 | 566 | 757 | 1,660 | 4,854 | 226 | 415 | 348 | 85 | 187 | 1,261 | 496 |
| 55 - 59 | 7,900 | 2664 | 5,236 | 36 | 222 | 428 | 485 | 610 | 420 | 556 | 1,146 | 3,867 | 192 | 322 | 193 | 80 | 155 | 942 | 391 |
| 60 - 64 | 9,649 | 4131 | 5,518 | 74 | 459 | 702 | 589 | 755 | 455 | 593 | 714 | 4,267 | 189 | 276 | 125 | 49 | 139 | 778 | 399 |
| 65 - 69 | 8,927 | 4196 | 4,731 | 57 | 440 | 694 | 592 | 732 | 382 | 454 | 480 | 3,774 | 126 | 214 | 80 | 36 | 109 | 565 | 335 |
| 70 - 74 | 9,378 | 4781 | 4,597 | 69 | 512 | 760 | 559 | 695 | 409 | 426 | 399 | 3,760 | 120 | 140 | 66 | 41 | 91 | 458 | 310 |
| 75+ | 18,039 | 9766 | 8,273 | 148 | 964 | 1,437 | 1,135 | 1,198 | 686 | 619 | 669 | 6,708 | 200 | 286 | 102 | 68 | 162 | 818 | 599 |

## Table F5: Disabled Population (De Facto) 5 Years and Older by 5 Year Age Group and Highest Level of Education Completed, Rural/Urban, Zambia 2010

| Age Group and Rural/ Urban | Disabled Popula- tion 5 Years and Older | Never attended | Total Ever Attended | Highest Level of Eduction Completed | | | | | | | | | | | | | | | |
|---|---|---|---|---|---|---|---|---|---|---|---|---|---|---|---|---|---|---|---|
| | | | | None | Primary School | | | | | | | | Secondary School | | | | | | Tertiary |
| | | | | | 1 | 2 | 3 | 4 | 5 | 6 | 7 | Total 1-7 | 8 | 9 | 10 | 11 | 12 | Total 8-12 | |
| **Zambia Urban** | | | | | | | | | | | | | | | | | | | |
| **Total** | **66,340** | **13,731** | **52,609** | **1,315** | **2,148** | **2,747** | **2,984** | **3,318** | **3,298** | **4,110** | **7,187** | **25,792** | **3,256** | **5,669** | **2,354** | **1,433** | **5,032** | **17,744** | **7,758** |
| 5-9 | 5,011 | 2,367 | 2,644 | 806 | 868 | 553 | 316 | 82 | 19 | 0 | 0 | 1,838 | 0 | 0 | 0 | 0 | 0 | 0 | 0 |
| 10-14 | 6,542 | 1,194 | 5,348 | 192 | 302 | 611 | 891 | 937 | 923 | 712 | 465 | 4,841 | 214 | 88 | 13 | 0 | 0 | 315 | 0 |
| 15-19 | 6,508 | 962 | 5,546 | 75 | 71 | 108 | 187 | 259 | 395 | 613 | 925 | 2,558 | 849 | 831 | 479 | 387 | 320 | 2,866 | 47 |
| 20 - 24 | 5,245 | 851 | 4,394 | 49 | 40 | 83 | 103 | 159 | 199 | 283 | 511 | 1,378 | 437 | 599 | 272 | 305 | 988 | 2,601 | 366 |
| 25 - 29 | 5,036 | 797 | 4,239 | 42 | 39 | 79 | 119 | 184 | 222 | 272 | 584 | 1,499 | 289 | 566 | 150 | 148 | 809 | 1,962 | 736 |
| 30 - 34 | 4,882 | 607 | 4,275 | 17 | 44 | 78 | 108 | 153 | 171 | 314 | 693 | 1,561 | 290 | 751 | 149 | 105 | 560 | 1,855 | 842 |
| 35 - 39 | 4,879 | 634 | 4,245 | 14 | 36 | 58 | 104 | 147 | 163 | 301 | 719 | 1,528 | 300 | 724 | 183 | 84 | 534 | 1,825 | 878 |
| 40 - 44 | 4,262 | 537 | 3,725 | 9 | 32 | 52 | 84 | 145 | 129 | 216 | 653 | 1,311 | 216 | 565 | 172 | 82 | 428 | 1,463 | 942 |
| 45 - 49 | 4,022 | 498 | 3,524 | 10 | 41 | 63 | 103 | 130 | 150 | 241 | 666 | 1,394 | 154 | 372 | 267 | 80 | 363 | 1,236 | 884 |
| 50 - 54 | 3,852 | 523 | 3,329 | 12 | 44 | 98 | 125 | 151 | 167 | 228 | 635 | 1,448 | 136 | 302 | 263 | 65 | 318 | 1,084 | 785 |
| 55 - 59 | 3,183 | 494 | 2,689 | 5 | 74 | 106 | 95 | 142 | 118 | 179 | 467 | 1,181 | 94 | 284 | 164 | 44 | 207 | 793 | 710 |
| 60 - 64 | 3,245 | 732 | 2,513 | 15 | 82 | 179 | 168 | 174 | 165 | 204 | 285 | 1,257 | 89 | 238 | 100 | 44 | 179 | 650 | 591 |
| 65 - 69 | 2,742 | 782 | 1,960 | 13 | 113 | 182 | 173 | 196 | 148 | 178 | 195 | 1,185 | 72 | 119 | 49 | 26 | 109 | 375 | 387 |
| 70 - 74 | 2,484 | 876 | 1,608 | 19 | 118 | 169 | 167 | 171 | 120 | 152 | 159 | 1,056 | 42 | 102 | 33 | 30 | 86 | 293 | 240 |
| 75+ | 4,447 | 1,877 | 2,570 | 37 | 244 | 328 | 241 | 288 | 209 | 217 | 230 | 1,757 | 74 | 128 | 60 | 33 | 131 | 426 | 350 |

## Table F5i: Disabled Population (De Facto) 5 Years and Older by 5 Year Age Group, Sex and Highest Level of Education Completed, Rural/Urban, Zambia 2010

| Age Group and Rural/ Urban | Disabled Popula- tion 5 Years and Older | Never attended | Total Ever Attended | Highest Level of Eduction Completed | | | | | | | | | | | | | | | |
|---|---|---|---|---|---|---|---|---|---|---|---|---|---|---|---|---|---|---|---|
| | | | | None | Primary School | | | | | | | | Secondary School | | | | | | Tertiary |
| | | | | | 1 | 2 | 3 | 4 | 5 | 6 | 7 | Total 1-7 | 8 | 9 | 10 | 11 | 12 | Total 8-12 | |
| **Zambia Total - Male** | | | | | | | | | | | | | | | | | | | |
| **Total** | **122,839** | **33,654** | **89,185** | **2,070** | **5,043** | **6,816** | **6,977** | **7,953** | **7,029** | **8,459** | **13,260** | **55,537** | **5,246** | **7,598** | **3,108** | **1,697** | **5,492** | **23,141** | **8,437** |
| 5-9 | 11,403 | 7,105 | 4,298 | 1,176 | 1,664 | 945 | 386 | 100 | 27 | - | - | 3,122 | - | - | - | - | - | - | - |
| 10-14 | 13,507 | 3,466 | 10,041 | 391 | 1,018 | 1,696 | 2,054 | 1,869 | 1,442 | 878 | 472 | 9,429 | 153 | 60 | 8 | - | - | 221 | - |
| 15-19 | 11,688 | 2,526 | 9,162 | 106 | 215 | 336 | 549 | 762 | 1,091 | 1,410 | 1,736 | 6,099 | 1,282 | 840 | 364 | 247 | 195 | 2,928 | 29 |
| 20 - 24 | 8,369 | 2,108 | 6,261 | 62 | 138 | 203 | 237 | 361 | 408 | 555 | 918 | 2,820 | 784 | 895 | 347 | 316 | 789 | 3,131 | 248 |
| 25 - 29 | 8,010 | 2,137 | 5,873 | 44 | 127 | 224 | 291 | 389 | 425 | 546 | 928 | 2,930 | 465 | 746 | 190 | 182 | 759 | 2,342 | 557 |
| 30 - 34 | 8,017 | 1,854 | 6,163 | 42 | 154 | 257 | 300 | 373 | 424 | 600 | 1,097 | 3,205 | 433 | 838 | 186 | 117 | 606 | 2,180 | 736 |
| 35 - 39 | 8,078 | 1,688 | 6,390 | 27 | 116 | 243 | 261 | 373 | 399 | 610 | 1,188 | 3,190 | 465 | 923 | 216 | 140 | 591 | 2,335 | 838 |
| 40 - 44 | 7,545 | 1,574 | 5,971 | 19 | 103 | 200 | 221 | 312 | 339 | 562 | 1,290 | 3,027 | 354 | 798 | 231 | 124 | 532 | 2,039 | 886 |
| 45 - 49 | 6,979 | 1,388 | 5,591 | 20 | 74 | 162 | 222 | 338 | 309 | 545 | 1,301 | 2,951 | 281 | 552 | 370 | 93 | 473 | 1,769 | 851 |
| 50 - 54 | 6,546 | 1,121 | 5,425 | 14 | 84 | 206 | 240 | 344 | 331 | 469 | 1,250 | 2,924 | 206 | 432 | 428 | 110 | 424 | 1,600 | 887 |
| 55 - 59 | 5,254 | 917 | 4,337 | 8 | 102 | 191 | 236 | 283 | 240 | 366 | 919 | 2,337 | 168 | 396 | 271 | 93 | 300 | 1,228 | 764 |
| 60 - 64 | 5,613 | 1,158 | 4,455 | 35 | 162 | 285 | 302 | 410 | 304 | 447 | 652 | 2,562 | 195 | 377 | 177 | 82 | 266 | 1,097 | 761 |
| 65 - 69 | 5,077 | 1,202 | 3,875 | 18 | 173 | 369 | 374 | 476 | 303 | 413 | 460 | 2,568 | 137 | 244 | 102 | 48 | 175 | 706 | 583 |
| 70 - 74 | 5,356 | 1,538 | 3,818 | 26 | 259 | 447 | 390 | 521 | 332 | 412 | 388 | 2,749 | 124 | 174 | 83 | 55 | 143 | 579 | 464 |
| 75+ | 11,397 | 3,872 | 7,525 | 82 | 654 | 1,052 | 914 | 1,042 | 655 | 646 | 661 | 5,624 | 199 | 323 | 135 | 90 | 239 | 986 | 833 |
| **Zambia Rural - Male** | | | | | | | | | | | | | | | | | | | |
| **Total** | **88,620** | **28,161** | **60,459** | **1,388** | **3,999** | **5,571** | **5,555** | **6,362** | **5,446** | **6,391** | **9,643** | **42,967** | **3,547** | **4,472** | **1,715** | **841** | **2,150** | **12,725** | **3,379** |
| 5-9 | 8,626 | 5,764 | 2,862 | 739 | 1,165 | 648 | 228 | 64 | 18 | - | - | 2,123 | - | - | - | - | - | - | - |
| 10-14 | 10,055 | 2,805 | 7,250 | 281 | 853 | 1,372 | 1,563 | 1,361 | 955 | 523 | 257 | 6,884 | 64 | 20 | 1 | - | - | 85 | - |
| 15-19 | 8,296 | 2,045 | 6,251 | 72 | 173 | 280 | 441 | 610 | 873 | 1,050 | 1,249 | 4,676 | 841 | 413 | 129 | 53 | 54 | 1,490 | 13 |
| 20 - 24 | 5,630 | 1,700 | 3,930 | 37 | 116 | 157 | 188 | 286 | 312 | 428 | 642 | 2,129 | 552 | 573 | 177 | 132 | 262 | 1,696 | 68 |
| 25 - 29 | 5,340 | 1,746 | 3,594 | 25 | 110 | 189 | 228 | 280 | 332 | 404 | 621 | 2,164 | 321 | 449 | 112 | 99 | 264 | 1,245 | 160 |
| 30 - 34 | 5,325 | 1,566 | 3,759 | 29 | 129 | 213 | 249 | 304 | 342 | 439 | 743 | 2,419 | 280 | 443 | 86 | 59 | 209 | 1,077 | 234 |
| 35 - 39 | 5,319 | 1,411 | 3,908 | 19 | 100 | 221 | 212 | 294 | 331 | 465 | 829 | 2,452 | 304 | 503 | 98 | 75 | 195 | 1,175 | 262 |
| 40 - 44 | 5,205 | 1,356 | 3,849 | 15 | 88 | 180 | 186 | 261 | 287 | 463 | 971 | 2,436 | 242 | 466 | 132 | 67 | 227 | 1,134 | 264 |
| 45 - 49 | 4,978 | 1,216 | 3,762 | 16 | 60 | 144 | 197 | 305 | 264 | 454 | 1,019 | 2,443 | 201 | 350 | 230 | 53 | 197 | 1,031 | 272 |
| 50 - 54 | 4,699 | 974 | 3,725 | 11 | 70 | 176 | 208 | 290 | 277 | 389 | 979 | 2,389 | 144 | 286 | 272 | 66 | 171 | 939 | 386 |
| 55 - 59 | 3,690 | 799 | 2,891 | 8 | 80 | 161 | 202 | 244 | 197 | 293 | 681 | 1,858 | 124 | 237 | 163 | 69 | 137 | 730 | 295 |
| 60 - 64 | 4,087 | 1,019 | 3,068 | 29 | 146 | 239 | 253 | 357 | 237 | 363 | 489 | 2,084 | 137 | 225 | 101 | 43 | 120 | 626 | 329 |
| 65 - 69 | 3,850 | 1,057 | 2,793 | 14 | 147 | 317 | 297 | 395 | 235 | 312 | 350 | 2,053 | 93 | 163 | 66 | 30 | 92 | 444 | 282 |
| 70 - 74 | 4,213 | 1,322 | 2,891 | 22 | 219 | 375 | 317 | 431 | 270 | 319 | 297 | 2,228 | 95 | 106 | 58 | 33 | 78 | 370 | 271 |
| 75+ | 9,307 | 3,381 | 5,926 | 71 | 543 | 899 | 786 | 880 | 516 | 489 | 516 | 4,629 | 149 | 238 | 90 | 62 | 144 | 683 | 543 |

## Table F5 i: Disabled Population (De Facto) 5 Years and Older by 5 Year Age Group, Sex and Highest Level of Education Completed, Rural/Urban, Zambia 2010

| Age Group and Rural/ Urban | Disabled Popula-tion 5 Years and Older | Never attended | Total Ever Attended | Highest Level of Eduction Completed | | | | | | | | | | | | | | | |
|---|---|---|---|---|---|---|---|---|---|---|---|---|---|---|---|---|---|---|---|
| | | | | None | Primary School | | | | | | | | Secondary School | | | | | | Tertiary |
| | | | | | 1 | 2 | 3 | 4 | 5 | 6 | 7 | Total 1-7 | 8 | 9 | 10 | 11 | 12 | Total 8-12 | |
| **Zambia Urban - Male** | | | | | | | | | | | | | | | | | | | |
| **Total** | **34,219** | **5,493** | **28,726** | **682** | **1,044** | **1,245** | **1,422** | **1,591** | **1,583** | **2,068** | **3,617** | **12,570** | **1,699** | **3,126** | **1,393** | **856** | **3,342** | **10,416** | **5,058** |
| 5-9 | 2,777 | 1,341 | 1,436 | 437 | 499 | 297 | 158 | 36 | 9 | 0 | 0 | 999 | 0 | 0 | 0 | 0 | 0 | 0 | 0 |
| 10-14 | 3,452 | 661 | 2,791 | 110 | 165 | 324 | 491 | 508 | 487 | 355 | 215 | 2,545 | 89 | 40 | 7 | 0 | 0 | 136 | 0 |
| 15-19 | 3,392 | 481 | 2,911 | 34 | 42 | 56 | 108 | 152 | 218 | 360 | 487 | 1,423 | 441 | 427 | 235 | 194 | 141 | 1,438 | 16 |
| 20 - 24 | 2,739 | 408 | 2,331 | 25 | 22 | 46 | 49 | 75 | 96 | 127 | 276 | 691 | 232 | 322 | 170 | 184 | 527 | 1,435 | 180 |
| 25 - 29 | 2,670 | 391 | 2,279 | 19 | 17 | 35 | 63 | 109 | 93 | 142 | 307 | 766 | 144 | 297 | 78 | 83 | 495 | 1,097 | 397 |
| 30 - 34 | 2,692 | 288 | 2,404 | 13 | 25 | 44 | 51 | 69 | 82 | 161 | 354 | 786 | 153 | 395 | 100 | 58 | 397 | 1,103 | 502 |
| 35 - 39 | 2,759 | 277 | 2,482 | 8 | 16 | 22 | 49 | 79 | 68 | 145 | 359 | 738 | 161 | 420 | 118 | 65 | 396 | 1,160 | 576 |
| 40 - 44 | 2,340 | 218 | 2,122 | 4 | 15 | 20 | 35 | 51 | 52 | 99 | 319 | 591 | 112 | 332 | 99 | 57 | 305 | 905 | 622 |
| 45 - 49 | 2,001 | 172 | 1,829 | 4 | 14 | 18 | 25 | 33 | 45 | 91 | 282 | 508 | 80 | 202 | 140 | 40 | 276 | 738 | 579 |
| 50 - 54 | 1,847 | 147 | 1,700 | 3 | 14 | 30 | 32 | 54 | 54 | 80 | 271 | 535 | 62 | 146 | 156 | 44 | 253 | 661 | 501 |
| 55 - 59 | 1,564 | 118 | 1,446 | - | 22 | 30 | 34 | 39 | 43 | 73 | 238 | 479 | 44 | 159 | 108 | 24 | 163 | 498 | 469 |
| 60 - 64 | 1,526 | 139 | 1,387 | 6 | 16 | 46 | 49 | 53 | 67 | 84 | 163 | 478 | 58 | 152 | 76 | 39 | 146 | 471 | 432 |
| 65 - 69 | 1,227 | 145 | 1,082 | 4 | 26 | 52 | 77 | 81 | 68 | 101 | 110 | 515 | 44 | 81 | 36 | 18 | 83 | 262 | 301 |
| 70 - 74 | 1,143 | 216 | 927 | 4 | 40 | 72 | 73 | 90 | 62 | 93 | 91 | 521 | 29 | 68 | 25 | 22 | 65 | 209 | 193 |
| 75+ | 2,090 | 491 | 1,599 | 11 | 111 | 153 | 128 | 162 | 139 | 157 | 145 | 995 | 50 | 85 | 45 | 28 | 95 | 303 | 290 |

## Table F5ii: Disabled Population (De Facto) 5 Years and Older by 5 Year Age Group, Sex and Highest Level of Education Completed, Rural/Urban, Zambia 2010

| Age Group and Rural/ Urban | Disabled Popula-tion 5 Years and Older | Never attended | Total Ever Attended | Highest Level of Eduction Completed | | | | | | | | | | | | | | | |
|---|---|---|---|---|---|---|---|---|---|---|---|---|---|---|---|---|---|---|---|
| | | | | None | Primary School | | | | | | | | Secondary School | | | | | | Tertiary |
| | | | | | 1 | 2 | 3 | 4 | 5 | 6 | 7 | Total 1-7 | 8 | 9 | 10 | 11 | 12 | Total 8-12 | |
| **Zambia Total - Female** | | | | | | | | | | | | | | | | | | | |
| **Total** | **115,617** | **48,489** | **67,128** | **1,914** | **5,287** | **6,972** | **6,607** | **7,375** | **5,991** | **6,584** | **9,870** | **48,686** | **3,571** | **4,647** | **1,580** | **849** | **2,246** | **12,893** | **3,635** |
| 5-9 | 8,731 | 5,163 | 3,568 | 953 | 1,346 | 800 | 355 | 91 | 23 | 0 | 0 | 2,615 | 0 | 0 | 0 | 0 | 0 | 0 | 0 |
| 10-14 | 11,123 | 2,789 | 8,334 | 270 | 808 | 1,364 | 1,600 | 1,491 | 1,221 | 828 | 475 | 7,787 | 202 | 66 | 9 | 0 | 0 | 277 | 0 |
| 15-19 | 9,591 | 2,512 | 7,079 | 86 | 156 | 294 | 393 | 561 | 731 | 976 | 1,274 | 4,385 | 1,013 | 762 | 324 | 240 | 227 | 2,566 | 42 |
| 20 - 24 | 7,466 | 2,482 | 4,984 | 66 | 131 | 261 | 289 | 370 | 377 | 481 | 691 | 2,600 | 516 | 610 | 177 | 185 | 608 | 2,096 | 222 |
| 25 - 29 | 7,105 | 2,515 | 4,590 | 61 | 177 | 285 | 307 | 361 | 422 | 443 | 718 | 2,713 | 329 | 457 | 103 | 87 | 402 | 1,378 | 438 |
| 30 - 34 | 6,725 | 2,182 | 4,543 | 44 | 140 | 229 | 326 | 398 | 351 | 470 | 861 | 2,775 | 298 | 608 | 88 | 68 | 220 | 1,282 | 442 |
| 35 - 39 | 6,769 | 2,267 | 4,502 | 36 | 137 | 239 | 320 | 400 | 393 | 533 | 921 | 2,943 | 280 | 538 | 101 | 36 | 186 | 1,141 | 382 |
| 40 - 44 | 6,601 | 2,123 | 4,478 | 32 | 146 | 246 | 309 | 447 | 382 | 483 | 1,056 | 3,069 | 229 | 408 | 142 | 46 | 148 | 973 | 404 |
| 45 - 49 | 7,116 | 2,300 | 4,816 | 30 | 181 | 292 | 358 | 482 | 411 | 560 | 1,165 | 3,449 | 173 | 318 | 249 | 64 | 129 | 933 | 404 |
| 50 - 54 | 7,093 | 2,544 | 4,549 | 32 | 187 | 321 | 362 | 545 | 402 | 516 | 1,045 | 3,378 | 156 | 285 | 183 | 40 | 81 | 745 | 394 |
| 55 - 59 | 5,829 | 2,241 | 3,588 | 33 | 194 | 343 | 344 | 469 | 298 | 369 | 694 | 2,711 | 118 | 210 | 86 | 31 | 62 | 507 | 337 |
| 60 - 64 | 7,281 | 3,705 | 3,576 | 54 | 379 | 596 | 455 | 519 | 316 | 350 | 347 | 2,962 | 83 | 137 | 48 | 11 | 52 | 331 | 229 |
| 65 - 69 | 6,592 | 3,776 | 2,816 | 52 | 380 | 507 | 391 | 452 | 227 | 219 | 215 | 2,391 | 61 | 89 | 27 | 14 | 43 | 234 | 139 |
| 70 - 74 | 6,506 | 4,119 | 2,387 | 62 | 371 | 482 | 336 | 345 | 197 | 166 | 170 | 2,067 | 38 | 68 | 16 | 16 | 34 | 172 | 86 |
| 75+ | 11,089 | 7,771 | 3,318 | 103 | 554 | 713 | 462 | 444 | 240 | 190 | 238 | 2,841 | 75 | 91 | 27 | 11 | 54 | 258 | 116 |
| **Zambia Rural - Female** | | | | | | | | | | | | | | | | | | | |
| **Total** | **83,496** | **40,251** | **43,245** | **1,281** | **4,183** | **5,470** | **5,045** | **5,648** | **4,276** | **4,542** | **6,300** | **35,464** | **2,014** | **2,104** | **619** | **272** | **556** | **5,565** | **935** |
| 5-9 | 6,497 | 4,137 | 2,360 | 584 | 977 | 544 | 197 | 45 | 13 | - | - | 1,776 | - | - | - | - | - | - | - |
| 10-14 | 8,033 | 2,256 | 5,777 | 188 | 671 | 1,077 | 1,200 | 1,062 | 785 | 471 | 225 | 5,491 | 77 | 18 | 3 | - | - | 98 | - |
| 15-19 | 6,475 | 2,031 | 4,444 | 45 | 127 | 242 | 314 | 454 | 554 | 723 | 836 | 3,250 | 605 | 358 | 80 | 47 | 48 | 1,138 | 11 |
| 20 - 24 | 4,960 | 2,039 | 2,921 | 42 | 113 | 224 | 235 | 286 | 274 | 325 | 456 | 1,913 | 311 | 333 | 75 | 64 | 147 | 930 | 36 |
| 25 - 29 | 4,739 | 2,109 | 2,630 | 38 | 155 | 241 | 251 | 286 | 293 | 313 | 441 | 1,980 | 184 | 188 | 31 | 22 | 88 | 513 | 99 |
| 30 - 34 | 4,535 | 1,863 | 2,672 | 40 | 121 | 195 | 269 | 314 | 262 | 317 | 522 | 2,000 | 161 | 252 | 39 | 21 | 57 | 530 | 102 |
| 35 - 39 | 4,649 | 1,910 | 2,739 | 30 | 117 | 203 | 265 | 332 | 298 | 377 | 561 | 2,153 | 141 | 234 | 36 | 17 | 48 | 476 | 80 |
| 40 - 44 | 4,679 | 1,804 | 2,875 | 27 | 129 | 214 | 260 | 353 | 305 | 366 | 722 | 2,349 | 125 | 175 | 69 | 21 | 25 | 415 | 84 |
| 45 - 49 | 5,095 | 1,974 | 3,121 | 24 | 154 | 247 | 280 | 385 | 306 | 410 | 781 | 2,563 | 99 | 148 | 122 | 24 | 42 | 435 | 99 |
| 50 - 54 | 5,088 | 2,168 | 2,920 | 23 | 157 | 253 | 269 | 448 | 289 | 368 | 681 | 2,465 | 82 | 129 | 76 | 19 | 16 | 322 | 110 |
| 55 - 59 | 4,210 | 1,865 | 2,345 | 28 | 142 | 267 | 283 | 366 | 223 | 263 | 465 | 2,009 | 68 | 85 | 30 | 11 | 18 | 212 | 96 |
| 60 - 64 | 5,562 | 3,112 | 2,450 | 45 | 313 | 463 | 336 | 398 | 218 | 230 | 225 | 2,183 | 52 | 51 | 24 | 6 | 19 | 152 | 70 |
| 65 - 69 | 5,077 | 3,139 | 1,938 | 43 | 293 | 377 | 295 | 337 | 147 | 142 | 130 | 1,721 | 33 | 51 | 14 | 6 | 17 | 121 | 53 |
| 70 - 74 | 5,165 | 3,459 | 1,706 | 47 | 293 | 385 | 242 | 264 | 139 | 107 | 102 | 1,532 | 25 | 34 | 8 | 8 | 13 | 88 | 39 |
| 75+ | 8,732 | 6,385 | 2,347 | 77 | 421 | 538 | 349 | 318 | 170 | 130 | 153 | 2,079 | 51 | 48 | 12 | 6 | 18 | 135 | 56 |

## Table F5 ii: Disabled Population (De Facto) 5 Years and Older by 5 Year Age Group, Sex and Highest Level of Education Completed, Rural/Urban, Zambia 2010

| Age Group and Rural/ Urban | Disabled Popula- tion 5 Years and Older | Never attended | Total Ever Attended | Highest Level of Eduction Completed | | | | | | | | | | | | | | |
|---|---|---|---|---|---|---|---|---|---|---|---|---|---|---|---|---|---|---|
| | | | | None | Primary School | | | | | | | | Secondary School | | | | | Tertiary |
| | | | | | 1 | 2 | 3 | 4 | 5 | 6 | 7 | Total 1-7 | 8 | 9 | 10 | 11 | 12 | Total 8-12 | |
| **Zambia Urban - Female** | | | | | | | | | | | | | | | | | | |
| **Total** | **32,121** | **8,238** | **23,883** | **633** | **1,104** | **1,502** | **1,562** | **1,727** | **1,715** | **2,042** | **3,570** | **13,222** | **1,557** | **2,543** | **961** | **577** | **1,690** | **7,328** | **2,700** |
| 5-9 | 2,234 | 1,026 | 1,208 | 369 | 369 | 256 | 158 | 46 | 10 | 0 | 0 | 839 | 0 | 0 | 0 | 0 | 0 | 0 | 0 |
| 10-14 | 3,090 | 533 | 2,557 | 82 | 137 | 287 | 400 | 429 | 436 | 357 | 250 | 2,296 | 125 | 48 | 6 | 0 | 0 | 179 | 0 |
| 15-19 | 3,116 | 481 | 2,635 | 41 | 29 | 52 | 79 | 107 | 177 | 253 | 438 | 1,135 | 408 | 404 | 244 | 193 | 179 | 1,428 | 31 |
| 20 - 24 | 2,506 | 443 | 2,063 | 24 | 18 | 37 | 54 | 84 | 103 | 156 | 235 | 687 | 205 | 277 | 102 | 121 | 461 | 1,166 | 186 |
| 25 - 29 | 2,366 | 406 | 1,960 | 23 | 22 | 44 | 56 | 75 | 129 | 130 | 277 | 733 | 145 | 269 | 72 | 65 | 314 | 865 | 339 |
| 30 - 34 | 2,190 | 319 | 1,871 | 4 | 19 | 34 | 57 | 84 | 89 | 153 | 339 | 775 | 137 | 356 | 49 | 47 | 163 | 752 | 340 |
| 35 - 39 | 2,120 | 357 | 1,763 | 6 | 20 | 36 | 55 | 68 | 95 | 156 | 360 | 790 | 139 | 304 | 65 | 19 | 138 | 665 | 302 |
| 40 - 44 | 1,922 | 319 | 1,603 | 5 | 17 | 32 | 49 | 94 | 77 | 117 | 334 | 720 | 104 | 233 | 73 | 25 | 123 | 558 | 320 |
| 45 - 49 | 2,021 | 326 | 1,695 | 6 | 27 | 45 | 78 | 97 | 105 | 150 | 384 | 886 | 74 | 170 | 127 | 40 | 87 | 498 | 305 |
| 50 - 54 | 2,005 | 376 | 1,629 | 9 | 30 | 68 | 93 | 97 | 113 | 148 | 364 | 913 | 74 | 156 | 107 | 21 | 65 | 423 | 284 |
| 55 - 59 | 1,619 | 376 | 1,243 | 5 | 52 | 76 | 61 | 103 | 75 | 106 | 229 | 702 | 50 | 125 | 56 | 20 | 44 | 295 | 241 |
| 60 - 64 | 1,719 | 593 | 1,126 | 9 | 66 | 133 | 119 | 121 | 98 | 120 | 122 | 779 | 31 | 86 | 24 | 5 | 33 | 179 | 159 |
| 65 - 69 | 1,515 | 637 | 878 | 9 | 87 | 130 | 96 | 115 | 80 | 77 | 85 | 670 | 28 | 38 | 13 | 8 | 26 | 113 | 86 |
| 70 - 74 | 1,341 | 660 | 681 | 15 | 78 | 97 | 94 | 81 | 58 | 59 | 68 | 535 | 13 | 34 | 8 | 8 | 21 | 84 | 47 |
| 75+ | 2,357 | 1,386 | 971 | 26 | 133 | 175 | 113 | 126 | 70 | 60 | 85 | 762 | 24 | 43 | 15 | 5 | 36 | 123 | 60 |

## Table F6: Disabled Population (De Facto) 12 Years and Older by 5 Year Age Group, Sex and Economic Activity, Rural/Urban, Zambia 2010

| Age Group and Sex | Disabled Population 12 Years and Older | Disabled labour force | Economically active | | | | Economically inactive | | | |
|---|---|---|---|---|---|---|---|---|---|---|
| | | | Employed | Unemployed | | | Total | Full time housewife/ homemaker | Full time student | Not available for work for other reasons |
| | | | | Total | Unemployed and seeking work | Not seeking work but availabe for work | | | | |
| **Zambia Total** | **207,996** | **104,052** | **94,628** | **9,424** | **5,206** | **4,218** | **103,944** | **18,712** | **20,571** | **64,661** |
| 12-14 | 14,304 | 2,462 | 2,179 | 283 | 90 | 193 | 11,842 | 377 | 8,381 | 3,084 |
| 15 - 19 | 21,279 | 5,844 | 4,861 | 983 | 458 | 525 | 15,435 | 1,043 | 9,245 | 5,147 |
| 20 - 24 | 15,835 | 7,941 | 6,486 | 1,455 | 869 | 586 | 7,894 | 1,737 | 2,029 | 4,128 |
| 25 - 29 | 15,115 | 8,990 | 7,719 | 1,271 | 822 | 449 | 6,125 | 1,960 | 381 | 3,784 |
| 30 - 34 | 14,742 | 9,378 | 8,383 | 995 | 602 | 393 | 5,364 | 1,788 | 136 | 3,440 |
| 35 - 39 | 14,847 | 9,687 | 8,805 | 882 | 548 | 334 | 5,160 | 1,757 | 80 | 3,323 |
| 40 - 44 | 14,146 | 9,499 | 8,734 | 765 | 492 | 273 | 4,647 | 1,595 | 57 | 2,995 |
| 45 - 49 | 14,095 | 9,468 | 8,762 | 706 | 397 | 309 | 4,627 | 1,717 | 46 | 2,864 |
| 50 - 54 | 13,639 | 8,868 | 8,353 | 515 | 274 | 241 | 4,771 | 1,514 | 41 | 3,216 |
| 55 - 59 | 11,083 | 6,760 | 6,356 | 404 | 197 | 207 | 4,323 | 1,152 | 21 | 3,150 |
| 60 - 64 | 12,894 | 7,089 | 6,714 | 375 | 168 | 207 | 5,805 | 1,194 | 20 | 4,591 |
| 65 - 69 | 11,669 | 6,003 | 5,725 | 278 | 103 | 175 | 5,666 | 929 | 27 | 4,710 |
| 70 - 74 | 11,862 | 5,013 | 4,787 | 226 | 89 | 137 | 6,849 | 819 | 38 | 5,992 |
| 75+ | 22,486 | 7,050 | 6,764 | 286 | 97 | 189 | 15,436 | 1,130 | 69 | 14,237 |
| **Male** | | | | | | | | | | |
| **Total** | **105,698** | **59,580** | **53,657** | **5,923** | **3,391** | **2,532** | **46,118** | **2,272** | **11,985** | **31,861** |
| 12-14 | 7,769 | 1,295 | 1,149 | 146 | 51 | 95 | 6,474 | 133 | 4,692 | 1,649 |
| 15 - 19 | 11,688 | 3,184 | 2,631 | 553 | 263 | 290 | 8,504 | 255 | 5,536 | 2,713 |
| 20 - 24 | 8,369 | 4,514 | 3,641 | 873 | 522 | 351 | 3,855 | 206 | 1,337 | 2,312 |
| 25 - 29 | 8,010 | 5,418 | 4,595 | 823 | 541 | 282 | 2,592 | 175 | 219 | 2,198 |
| 30 - 34 | 8,017 | 5,687 | 5,047 | 640 | 407 | 233 | 2,330 | 173 | 63 | 2,094 |
| 35 - 39 | 8,078 | 5,819 | 5,229 | 590 | 380 | 210 | 2,259 | 143 | 33 | 2,083 |
| 40 - 44 | 7,545 | 5,653 | 5,126 | 527 | 350 | 177 | 1,892 | 128 | 19 | 1,745 |
| 45 - 49 | 6,979 | 5,306 | 4,884 | 422 | 240 | 182 | 1,673 | 142 | 15 | 1,516 |
| 50 - 54 | 6,546 | 4,857 | 4,514 | 343 | 191 | 152 | 1,689 | 118 | 17 | 1,554 |
| 55 - 59 | 5,254 | 3,677 | 3,419 | 258 | 138 | 120 | 1,577 | 106 | 4 | 1,467 |
| 60 - 64 | 5,613 | 3,623 | 3,380 | 243 | 115 | 128 | 1,990 | 126 | 6 | 1,858 |
| 65 - 69 | 5,077 | 3,123 | 2,952 | 171 | 67 | 104 | 1,954 | 115 | 5 | 1,834 |
| 70 - 74 | 5,356 | 2,871 | 2,720 | 151 | 65 | 86 | 2,485 | 151 | 14 | 2,320 |
| 75+ | 11,397 | 4,553 | 4,370 | 183 | 61 | 122 | 6,844 | 301 | 25 | 6,518 |
| **Female** | | | | | | | | | | |
| **Total** | **102,298** | **44,472** | **40,971** | **3,501** | **1,815** | **1,686** | **57,826** | **16,440** | **8,586** | **32,800** |
| 12-14 | 6,535 | 1,167 | 1,030 | 137 | 39 | 98 | 5,368 | 244 | 3,689 | 1,435 |
| 15 - 19 | 9,591 | 2,660 | 2,230 | 430 | 195 | 235 | 6,931 | 788 | 3,709 | 2,434 |
| 20 - 24 | 7,466 | 3,427 | 2,845 | 582 | 347 | 235 | 4,039 | 1,531 | 692 | 1,816 |
| 25 - 29 | 7,105 | 3,572 | 3,124 | 448 | 281 | 167 | 3,533 | 1,785 | 162 | 1,586 |
| 30 - 34 | 6,725 | 3,691 | 3,336 | 355 | 195 | 160 | 3,034 | 1,615 | 73 | 1,346 |
| 35 - 39 | 6,769 | 3,868 | 3,576 | 292 | 168 | 124 | 2,901 | 1,614 | 47 | 1,240 |
| 40 - 44 | 6,601 | 3,846 | 3,608 | 238 | 142 | 96 | 2,755 | 1,467 | 38 | 1,250 |
| 45 - 49 | 7,116 | 4,162 | 3,878 | 284 | 157 | 127 | 2,954 | 1,575 | 31 | 1,348 |
| 50 - 54 | 7,093 | 4,011 | 3,839 | 172 | 83 | 89 | 3,082 | 1,396 | 24 | 1,662 |
| 55 - 59 | 5,829 | 3,083 | 2,937 | 146 | 59 | 87 | 2,746 | 1,046 | 17 | 1,683 |
| 60 - 64 | 7,281 | 3,466 | 3,334 | 132 | 53 | 79 | 3,815 | 1,068 | 14 | 2,733 |
| 65 - 69 | 6,592 | 2,880 | 2,773 | 107 | 36 | 71 | 3,712 | 814 | 22 | 2,876 |
| 70 - 74 | 6,506 | 2,142 | 2,067 | 75 | 24 | 51 | 4,364 | 668 | 24 | 3,672 |
| 75+ | 11,089 | 2,497 | 2,394 | 103 | 36 | 67 | 8,592 | 829 | 44 | 7,719 |

## Table F6: Disabled Population (De Facto) 12 Years and Older by 5 Year Age Group, Sex and Economic Activity, Rural, Zambia 2010

| Age Group and Sex | Disabled Population 12 Years and Older | Disabled labour force | Economically active | | | | Economically inactive | | | |
|---|---|---|---|---|---|---|---|---|---|---|
| | | | Employed | Unemployed | | | Total | Full time housewife/ homemaker | Full time student | Not available for work for other reasons |
| | | | | Total | Unemployed and seeking work | Not seeking work but availabe for work | | | | |
| **Zambia Rural** | **149,251** | **79,994** | **75,367** | **4,627** | **1,880** | **2,747** | **69,257** | **11,693** | **12,779** | **44,785** |
| 12-14 | 10,346 | 2,228 | 2,031 | 197 | 47 | 150 | 8,118 | 266 | 5,627 | 2,225 |
| 15 - 19 | 14,771 | 4,941 | 4,413 | 528 | 186 | 342 | 9,830 | 684 | 5,659 | 3,487 |
| 20 - 24 | 10,590 | 5,886 | 5,295 | 591 | 247 | 344 | 4,704 | 990 | 1,015 | 2,699 |
| 25 - 29 | 10,079 | 6,359 | 5,838 | 521 | 236 | 285 | 3,720 | 1,144 | 173 | 2,403 |
| 30 - 34 | 9,860 | 6,521 | 6,076 | 445 | 206 | 239 | 3,339 | 1,046 | 56 | 2,237 |
| 35 - 39 | 9,968 | 6,725 | 6,324 | 401 | 193 | 208 | 3,243 | 1,057 | 45 | 2,141 |
| 40 - 44 | 9,884 | 6,849 | 6,489 | 360 | 175 | 185 | 3,035 | 971 | 27 | 2,037 |
| 45 - 49 | 10,073 | 6,976 | 6,620 | 356 | 155 | 201 | 3,097 | 1,111 | 29 | 1,957 |
| 50 - 54 | 9,787 | 6,729 | 6,442 | 287 | 132 | 155 | 3,058 | 916 | 28 | 2,114 |
| 55 - 59 | 7,900 | 5,241 | 5,025 | 216 | 86 | 130 | 2,659 | 701 | 15 | 1,943 |
| 60 - 64 | 9,649 | 5,832 | 5,610 | 222 | 74 | 148 | 3,817 | 790 | 15 | 3,012 |
| 65 - 69 | 8,927 | 5,061 | 4,892 | 169 | 47 | 122 | 3,866 | 648 | 20 | 3,198 |
| 70 - 74 | 9,378 | 4,373 | 4,233 | 140 | 37 | 103 | 5,005 | 578 | 24 | 4,403 |
| 75+ | 18,039 | 6,273 | 6,079 | 194 | 59 | 135 | 11,766 | 791 | 46 | 10,929 |
| **Male** | | | | | | | | | | |
| **Total** | **75,667** | **44,682** | **41,704** | **2,978** | **1,282** | **1,696** | **30,985** | **1,540** | **7,820** | **21,625** |
| 12-14 | 5,728 | 1,171 | 1,071 | 100 | 27 | 73 | 4,557 | 91 | 3,267 | 1,199 |
| 15 - 19 | 8,296 | 2,678 | 2,381 | 297 | 112 | 185 | 5,618 | 158 | 3,588 | 1,872 |
| 20 - 24 | 5,630 | 3,278 | 2,890 | 388 | 168 | 220 | 2,352 | 112 | 739 | 1,501 |
| 25 - 29 | 5,340 | 3,749 | 3,392 | 357 | 167 | 190 | 1,591 | 105 | 113 | 1,373 |
| 30 - 34 | 5,325 | 3,858 | 3,562 | 296 | 152 | 144 | 1,467 | 117 | 24 | 1,326 |
| 35 - 39 | 5,319 | 3,905 | 3,619 | 286 | 143 | 143 | 1,414 | 103 | 16 | 1,295 |
| 40 - 44 | 5,205 | 3,953 | 3,695 | 258 | 130 | 128 | 1,252 | 85 | 8 | 1,159 |
| 45 - 49 | 4,978 | 3,827 | 3,610 | 217 | 92 | 125 | 1,151 | 108 | 12 | 1,031 |
| 50 - 54 | 4,699 | 3,589 | 3,394 | 195 | 96 | 99 | 1,110 | 90 | 12 | 1,008 |
| 55 - 59 | 3,690 | 2,738 | 2,601 | 137 | 63 | 74 | 952 | 76 | 4 | 872 |
| 60 - 64 | 4,087 | 2,854 | 2,721 | 133 | 46 | 87 | 1,233 | 84 | 4 | 1,145 |
| 65 - 69 | 3,850 | 2,569 | 2,468 | 101 | 26 | 75 | 1,281 | 80 | 5 | 1,196 |
| 70 - 74 | 4,213 | 2,457 | 2,367 | 90 | 26 | 64 | 1,756 | 110 | 11 | 1,635 |
| 75+ | 9,307 | 4,056 | 3,933 | 123 | 34 | 89 | 5,251 | 221 | 17 | 5,013 |
| **Female** | | | | | | | | | | |
| **Total** | **73,584** | **35,312** | **33,663** | **1,649** | **598** | **1,051** | **38,272** | **10,153** | **4,959** | **23,160** |
| 12-14 | 4,618 | 1,057 | 960 | 97 | 20 | 77 | 3,561 | 175 | 2,360 | 1,026 |
| 15 - 19 | 6,475 | 2,263 | 2,032 | 231 | 74 | 157 | 4,212 | 526 | 2,071 | 1,615 |
| 20 - 24 | 4,960 | 2,608 | 2,405 | 203 | 79 | 124 | 2,352 | 878 | 276 | 1,198 |
| 25 - 29 | 4,739 | 2,610 | 2,446 | 164 | 69 | 95 | 2,129 | 1,039 | 60 | 1,030 |
| 30 - 34 | 4,535 | 2,663 | 2,514 | 149 | 54 | 95 | 1,872 | 929 | 32 | 911 |
| 35 - 39 | 4,649 | 2,820 | 2,705 | 115 | 50 | 65 | 1,829 | 954 | 29 | 846 |
| 40 - 44 | 4,679 | 2,896 | 2,794 | 102 | 45 | 57 | 1,783 | 886 | 19 | 878 |
| 45 - 49 | 5,095 | 3,149 | 3,010 | 139 | 63 | 76 | 1,946 | 1,003 | 17 | 926 |
| 50 - 54 | 5,088 | 3,140 | 3,048 | 92 | 36 | 56 | 1,948 | 826 | 16 | 1,106 |
| 55 - 59 | 4,210 | 2,503 | 2,424 | 79 | 23 | 56 | 1,707 | 625 | 11 | 1,071 |
| 60 - 64 | 5,562 | 2,978 | 2,889 | 89 | 28 | 61 | 2,584 | 706 | 11 | 1,867 |
| 65 - 69 | 5,077 | 2,492 | 2,424 | 68 | 21 | 47 | 2,585 | 568 | 15 | 2,002 |
| 70 - 74 | 5,165 | 1,916 | 1,866 | 50 | 11 | 39 | 3,249 | 468 | 13 | 2,768 |
| 75+ | 8,732 | 2,217 | 2,146 | 71 | 25 | 46 | 6,515 | 570 | 29 | 5,916 |

## Table F6: Disabled Population (De Facto) 12 Years and Older by 5 Year Age Group, Sex and Economic Activity, Urban, Zambia 2010

| Age Group and Sex | Disabled Population 12 Years and Older | Disabled labour force | Economically active | | | | Economically inactive | | | |
|---|---|---|---|---|---|---|---|---|---|---|
| | | | Employed | Unemployed | | | Total | Full time housewife/ homemaker | Full time student | Not available for work for other reasons |
| | | | | Total | Unemployed and seeking work | Not seeking work but availabe for work | | | | |
| **Zambia Urban** | **58,745** | **24,058** | **19,261** | **4,797** | **3,326** | **1,471** | **34,687** | **7,019** | **7,792** | **19,876** |
| 12-14 | 3,958 | 234 | 148 | 86 | 43 | 43 | 3,724 | 111 | 2,754 | 859 |
| 15 - 19 | 6,508 | 903 | 448 | 455 | 272 | 183 | 5,605 | 359 | 3,586 | 1,660 |
| 20 - 24 | 5,245 | 2,055 | 1,191 | 864 | 622 | 242 | 3,190 | 747 | 1,014 | 1,429 |
| 25 - 29 | 5,036 | 2,631 | 1,881 | 750 | 586 | 164 | 2,405 | 816 | 208 | 1,381 |
| 30 - 34 | 4,882 | 2,857 | 2,307 | 550 | 396 | 154 | 2,025 | 742 | 80 | 1,203 |
| 35 - 39 | 4,879 | 2,962 | 2,481 | 481 | 355 | 126 | 1,917 | 700 | 35 | 1,182 |
| 40 - 44 | 4,262 | 2,650 | 2,245 | 405 | 317 | 88 | 1,612 | 624 | 30 | 958 |
| 45 - 49 | 4,022 | 2,492 | 2,142 | 350 | 242 | 108 | 1,530 | 606 | 17 | 907 |
| 50 - 54 | 3,852 | 2,139 | 1,911 | 228 | 142 | 86 | 1,713 | 598 | 13 | 1,102 |
| 55 - 59 | 3,183 | 1,519 | 1,331 | 188 | 111 | 77 | 1,664 | 451 | 6 | 1,207 |
| 60 - 64 | 3,245 | 1,257 | 1,104 | 153 | 94 | 59 | 1,988 | 404 | 5 | 1,579 |
| 65 - 69 | 2,742 | 942 | 833 | 109 | 56 | 53 | 1,800 | 281 | 7 | 1,512 |
| 70 - 74 | 2,484 | 640 | 554 | 86 | 52 | 34 | 1,844 | 241 | 14 | 1,589 |
| 75+ | 4,447 | 777 | 685 | 92 | 38 | 54 | 3,670 | 339 | 23 | 3,308 |
| **Male** | | | | | | | | | | |
| **Total** | **30,031** | **14,898** | **11,953** | **2,945** | **2,109** | **836** | **15,133** | **732** | **4,165** | **10,236** |
| 12-14 | 2,041 | 124 | 78 | 46 | 24 | 22 | 1,917 | 42 | 1,425 | 450 |
| 15 - 19 | 3,392 | 506 | 250 | 256 | 151 | 105 | 2,886 | 97 | 1,948 | 841 |
| 20 - 24 | 2,739 | 1,236 | 751 | 485 | 354 | 131 | 1,503 | 94 | 598 | 811 |
| 25 - 29 | 2,670 | 1,669 | 1,203 | 466 | 374 | 92 | 1,001 | 70 | 106 | 825 |
| 30 - 34 | 2,692 | 1,829 | 1,485 | 344 | 255 | 89 | 863 | 56 | 39 | 768 |
| 35 - 39 | 2,759 | 1,914 | 1,610 | 304 | 237 | 67 | 845 | 40 | 17 | 788 |
| 40 - 44 | 2,340 | 1,700 | 1,431 | 269 | 220 | 49 | 640 | 43 | 11 | 586 |
| 45 - 49 | 2,001 | 1,479 | 1,274 | 205 | 148 | 57 | 522 | 34 | 3 | 485 |
| 50 - 54 | 1,847 | 1,268 | 1,120 | 148 | 95 | 53 | 579 | 28 | 5 | 546 |
| 55 - 59 | 1,564 | 939 | 818 | 121 | 75 | 46 | 625 | 30 | - | 595 |
| 60 - 64 | 1,526 | 769 | 659 | 110 | 69 | 41 | 757 | 42 | 2 | 713 |
| 65 - 69 | 1,227 | 554 | 484 | 70 | 41 | 29 | 673 | 35 | - | 638 |
| 70 - 74 | 1,143 | 414 | 353 | 61 | 39 | 22 | 729 | 41 | 3 | 685 |
| 75+ | 2,090 | 497 | 437 | 60 | 27 | 33 | 1,593 | 80 | 8 | 1,505 |
| **Female** | | | | | | | | | | |
| **Total** | **28,714** | **9,160** | **7,308** | **1,852** | **1,217** | **635** | **19,554** | **6,287** | **3,627** | **9,640** |
| 12-14 | 1,917 | 110 | 70 | 40 | 19 | 21 | 1,807 | 69 | 1,329 | 409 |
| 15 - 19 | 3,116 | 397 | 198 | 199 | 121 | 78 | 2,719 | 262 | 1,638 | 819 |
| 20 - 24 | 2,506 | 819 | 440 | 379 | 268 | 111 | 1,687 | 653 | 416 | 618 |
| 25 - 29 | 2,366 | 962 | 678 | 284 | 212 | 72 | 1,404 | 746 | 102 | 556 |
| 30 - 34 | 2,190 | 1,028 | 822 | 206 | 141 | 65 | 1,162 | 686 | 41 | 435 |
| 35 - 39 | 2,120 | 1,048 | 871 | 177 | 118 | 59 | 1,072 | 660 | 18 | 394 |
| 40 - 44 | 1,922 | 950 | 814 | 136 | 97 | 39 | 972 | 581 | 19 | 372 |
| 45 - 49 | 2,021 | 1,013 | 868 | 145 | 94 | 51 | 1,008 | 572 | 14 | 422 |
| 50 - 54 | 2,005 | 871 | 791 | 80 | 47 | 33 | 1,134 | 570 | 8 | 556 |
| 55 - 59 | 1,619 | 580 | 513 | 67 | 36 | 31 | 1,039 | 421 | 6 | 612 |
| 60 - 64 | 1,719 | 488 | 445 | 43 | 25 | 18 | 1,231 | 362 | 3 | 866 |
| 65 - 69 | 1,515 | 388 | 349 | 39 | 15 | 24 | 1,127 | 246 | 7 | 874 |
| 70 - 74 | 1,341 | 226 | 201 | 25 | 13 | 12 | 1,115 | 200 | 11 | 904 |
| 75+ | 2,357 | 280 | 248 | 32 | 11 | 21 | 2,077 | 259 | 15 | 1,803 |

## Table F7: Population (De Facto) and Albino Population by 5 Year Age Group and Sex, Rural/Urban, Zambia 2010

| Age Group and Rural/Urban | Population | | | Albino Population | | | Percent Albinos | | |
|---|---|---|---|---|---|---|---|---|---|
| | Total | Male | Female | Total | Male | Female | Total | Male | Female |
| **Zambia Total** | | | | | | | | | |
| **Total** | **12,526,314** | **6,117,253** | **6,409,061** | **25,324** | **12,532** | **12,792** | **0.2** | **0.2** | **0.2** |
| 0 - 4 | 2,214,887 | 1,102,272 | 1,112,615 | 5,564 | 2,749 | 2,815 | 0.3 | 0.2 | 0.3 |
| 5-9 | 1,856,336 | 924,395 | 931,941 | 4,261 | 2,180 | 2,081 | 0.2 | 0.2 | 0.2 |
| 10-14 | 1,699,042 | 840,834 | 858,208 | 3,734 | 1,865 | 1,869 | 0.2 | 0.2 | 0.2 |
| 15 - 19 | 1,427,884 | 692,859 | 735,025 | 2,873 | 1,390 | 1,483 | 0.2 | 0.2 | 0.2 |
| 20 - 24 | 1,117,476 | 504,876 | 612,600 | 1,993 | 927 | 1,066 | 0.2 | 0.2 | 0.2 |
| 25 - 29 | 1,006,244 | 464,493 | 541,751 | 1,655 | 833 | 822 | 0.2 | 0.2 | 0.2 |
| 30 - 34 | 800,770 | 397,694 | 403,076 | 1,293 | 687 | 606 | 0.2 | 0.2 | 0.2 |
| 35 - 39 | 647,813 | 332,961 | 314,852 | 982 | 499 | 483 | 0.2 | 0.1 | 0.2 |
| 40 - 44 | 447,584 | 232,253 | 215,331 | 676 | 343 | 333 | 0.2 | 0.1 | 0.2 |
| 45 - 49 | 354,989 | 174,833 | 180,156 | 547 | 261 | 286 | 0.2 | 0.1 | 0.2 |
| 50 - 54 | 270,254 | 128,696 | 141,558 | 466 | 225 | 241 | 0.2 | 0.2 | 0.2 |
| 55 - 59 | 184,828 | 90,037 | 94,791 | 285 | 145 | 140 | 0.2 | 0.2 | 0.1 |
| 60 - 64 | 161,875 | 73,419 | 88,456 | 310 | 132 | 178 | 0.2 | 0.2 | 0.2 |
| 65 - 69 | 118,582 | 53,695 | 64,887 | 243 | 99 | 144 | 0.2 | 0.2 | 0.2 |
| 70 - 74 | 90,802 | 41,929 | 48,873 | 183 | 89 | 94 | 0.2 | 0.2 | 0.2 |
| 75+ | 126,948 | 62,007 | 64,941 | 259 | 108 | 151 | 0.2 | 0.2 | 0.2 |
| **Zambia Rural** | | | | | | | | | |
| **Total** | **7,505,292** | **3,664,349** | **3,840,943** | **16,937** | **8,335** | **8,602** | **0.2** | **0.2** | **0.2** |
| 0 - 4 | 1,454,385 | 724,158 | 730,227 | 3,948 | 1,964 | 1,984 | 0.3 | 0.3 | 0.3 |
| 0 - 4 | 1,212,247 | 608,241 | 604,006 | 2,966 | 1,519 | 1,447 | 0.2 | 0.2 | 0.2 |
| 5-9 | 1,045,977 | 529,978 | 515,999 | 2,556 | 1,280 | 1,276 | 0.2 | 0.2 | 0.2 |
| 15 - 19 | 802,997 | 397,738 | 405,259 | 1,839 | 887 | 952 | 0.2 | 0.2 | 0.2 |
| 20 - 24 | 598,270 | 269,368 | 328,902 | 1,243 | 588 | 655 | 0.2 | 0.2 | 0.2 |
| 25 - 29 | 529,481 | 241,136 | 288,345 | 1,006 | 502 | 504 | 0.2 | 0.2 | 0.2 |
| 30 - 34 | 419,932 | 203,507 | 216,425 | 731 | 367 | 364 | 0.2 | 0.2 | 0.2 |
| 35 - 39 | 351,970 | 173,650 | 178,320 | 590 | 280 | 310 | 0.2 | 0.2 | 0.2 |
| 40 - 44 | 254,964 | 127,044 | 127,920 | 466 | 233 | 233 | 0.2 | 0.2 | 0.2 |
| 45 - 49 | 209,868 | 100,903 | 108,965 | 366 | 164 | 202 | 0.2 | 0.2 | 0.2 |
| 50 - 54 | 162,395 | 75,868 | 86,527 | 300 | 146 | 154 | 0.2 | 0.2 | 0.2 |
| 55 - 59 | 111,511 | 52,225 | 59,286 | 183 | 92 | 91 | 0.2 | 0.2 | 0.2 |
| 60 - 64 | 107,038 | 45,761 | 61,277 | 207 | 84 | 123 | 0.2 | 0.2 | 0.2 |
| 65 - 69 | 83,157 | 36,611 | 46,546 | 191 | 77 | 114 | 0.2 | 0.2 | 0.2 |
| 70 - 74 | 66,388 | 30,553 | 35,835 | 135 | 67 | 68 | 0.2 | 0.2 | 0.2 |
| 75+ | 94,712 | 47,608 | 47,104 | 210 | 85 | 125 | 0.2 | 0.2 | 0.3 |

## Table F7: Population (De Facto) and Albino Population by 5 Year Age Group and Sex, Rural/Urban, Zambia 2010

| Age Group and Rural/Urban | Population | | | Albino Population | | | Percent Albinos | | |
|---|---|---|---|---|---|---|---|---|---|
| | Total | Male | Female | Total | Male | Female | Total | Male | Female |
| **Zambia Urban** | | | | | | | | | |
| **Total** | **5,021,022** | **2,452,904** | **2,568,118** | **8,387** | **4,197** | **4,190** | **0.2** | **0.2** | **0.2** |
| 0 - 4 | 760,502 | 378,114 | 382,388 | 1,616 | 785 | 831 | 0.2 | 0.2 | 0.2 |
| 0 - 4 | 644,089 | 316,154 | 327,935 | 1,295 | 661 | 634 | 0.2 | 0.2 | 0.2 |
| 5-9 | 653,065 | 310,856 | 342,209 | 1,178 | 585 | 593 | 0.2 | 0.2 | 0.2 |
| 15 - 19 | 624,887 | 295,121 | 329,766 | 1,034 | 503 | 531 | 0.2 | 0.2 | 0.2 |
| 20 - 24 | 519,206 | 235,508 | 283,698 | 750 | 339 | 411 | 0.1 | 0.1 | 0.1 |
| 25 - 29 | 476,763 | 223,357 | 253,406 | 649 | 331 | 318 | 0.1 | 0.1 | 0.1 |
| 30 - 34 | 380,838 | 194,187 | 186,651 | 562 | 320 | 242 | 0.1 | 0.2 | 0.1 |
| 35 - 39 | 295,843 | 159,311 | 136,532 | 392 | 219 | 173 | 0.1 | 0.1 | 0.1 |
| 40 - 44 | 192,620 | 105,209 | 87,411 | 210 | 110 | 100 | 0.1 | 0.1 | 0.1 |
| 45 - 49 | 145,121 | 73,930 | 71,191 | 181 | 97 | 84 | 0.1 | 0.1 | 0.1 |
| 50 - 54 | 107,859 | 52,828 | 55,031 | 166 | 79 | 87 | 0.2 | 0.1 | 0.2 |
| 55 - 59 | 73,317 | 37,812 | 35,505 | 102 | 53 | 49 | 0.1 | 0.1 | 0.1 |
| 60 - 64 | 54,837 | 27,658 | 27,179 | 103 | 48 | 55 | 0.2 | 0.2 | 0.2 |
| 65 - 69 | 35,425 | 17,084 | 18,341 | 52 | 22 | 30 | 0.1 | 0.1 | 0.2 |
| 70 - 74 | 24,414 | 11,376 | 13,038 | 48 | 22 | 26 | 0.2 | 0.2 | 0.2 |
| 75+ | 32,236 | 14,399 | 17,837 | 49 | 23 | 26 | 0.2 | 0.2 | 0.1 |

# Migration Tables

## Table G1: Population Born Outside Zambia by Country of Birth, Age Group and Sex, Zambia 2010

| Country of Birth | Total | | | 0 - 4 | | 5 - 9 | | 10 - 14 | | 15-44 | | 45 - 49 | | 50 - 54 | | 55 - 59 | | 60 - 64 | | 65+ | |
|---|---|---|---|---|---|---|---|---|---|---|---|---|---|---|---|---|---|---|---|---|---|
| | Total | Male | Female | Male | Female | Male | Female | Male | Female | Male | Female | Male | Female | Male | Female | Male | Female | Male | Female | Male | Female |
| **Zambia** | | | | | | | | | | | | | | | | | | | | | |
| **Total** | **101,780** | **51,367** | **50,413** | **2,508** | **2,545** | **2,811** | **3,037** | **3,345** | **3,555** | **23,627** | **23,153** | **3,922** | **3,190** | **3,423** | **3,170** | **2,708** | **2,569** | **2,357** | **2,552** | **6,666** | **6,642** |
| Algeria | 324 | 165 | 159 | 27 | 21 | 13 | 24 | 13 | 21 | 72 | 65 | 5 | 4 | 5 | 5 | 5 | 3 | 5 | 6 | 20 | 10 |
| Angola | 11,841 | 5,857 | 5,984 | 93 | 89 | 154 | 191 | 590 | 631 | 2,077 | 2,564 | 522 | 408 | 474 | 341 | 359 | 215 | 333 | 294 | 1,255 | 1,251 |
| Benin | 237 | 115 | 122 | 18 | 20 | 19 | 20 | 11 | 15 | 48 | 51 | 5 | 4 | 1 | 2 | 3 | - | 5 | 4 | 5 | 6 |
| Botswana | 918 | 423 | 495 | 50 | 57 | 91 | 110 | 74 | 92 | 153 | 146 | 16 | 11 | 14 | 10 | 10 | 12 | 3 | 12 | 12 | 45 |
| Burundi | 1,068 | 572 | 496 | 43 | 32 | 41 | 43 | 40 | 49 | 357 | 304 | 33 | 24 | 19 | 16 | 21 | 7 | 7 | 12 | 11 | 9 |
| Cameroon | 243 | 129 | 114 | 21 | 24 | 18 | 17 | 14 | 13 | 60 | 48 | 7 | 5 | 2 | 4 | 4 | - | 2 | 1 | 1 | 2 |
| Cape Verde Islands | 218 | 118 | 100 | 27 | 17 | 15 | 12 | 15 | 13 | 42 | 43 | 5 | 4 | 4 | 3 | 2 | - | 4 | 3 | 4 | 5 |
| Central African Republic | 31 | 16 | 15 | 1 | 1 | 2 | 2 | 1 | 2 | 10 | 5 | - | - | - | - | - | 4 | 1 | 1 | 1 | - |
| Chad | 34 | 13 | 21 | 1 | 3 | 2 | 3 | 4 | 1 | 5 | 10 | - | 1 | 1 | 1 | - | - | - | 1 | - | 1 |
| Comoros | 23 | 8 | 15 | 1 | 2 | - | - | 1 | 2 | 4 | 6 | - | 1 | - | - | - | - | 2 | - | - | 4 |
| Congo | 8,480 | 4,261 | 4,219 | 143 | 134 | 192 | 229 | 290 | 322 | 2,368 | 2,379 | 351 | 302 | 279 | 251 | 203 | 181 | 155 | 142 | 280 | 279 |
| Egypt | 164 | 104 | 60 | 1 | 5 | 9 | 6 | 4 | 2 | 60 | 28 | 7 | 8 | 9 | 3 | 4 | 1 | 4 | 2 | 6 | 5 |
| Equatorial Guinea | 3,129 | 1,624 | 1,505 | 108 | 108 | 96 | 101 | 127 | 104 | 939 | 857 | 112 | 80 | 78 | 81 | 56 | 50 | 43 | 37 | 65 | 87 |
| Ethiopia | 100 | 48 | 52 | 1 | 4 | 1 | 5 | 2 | 1 | 26 | 37 | 5 | 3 | 5 | 1 | 2 | 1 | 4 | - | 2 | - |
| Gabon | 27 | 24 | 3 | - | - | - | - | 1 | - | 16 | - | - | - | 2 | 2 | 3 | - | 1 | - | 1 | 1 |
| Gambia | 6 | 5 | 1 | - | - | 1 | - | - | - | 2 | - | - | - | - | - | 1 | - | - | 1 | 1 | - |
| Ghana | 175 | 119 | 56 | 3 | 1 | - | 4 | 1 | 5 | 45 | 27 | 20 | 5 | 23 | 10 | 15 | 2 | 5 | 2 | 7 | - |
| Guinea | 37 | 20 | 17 | 1 | - | - | - | 1 | - | 8 | 10 | 2 | 2 | - | 2 | 3 | - | 3 | 1 | 2 | 2 |
| Guinea  Bissau | 4 | 1 | 3 | - | - | - | - | - | 1 | 1 | 1 | - | - | - | - | - | - | - | 1 | - | - |
| Ivory Coast | 49 | 28 | 21 | - | - | 3 | 1 | - | 2 | 17 | 13 | 3 | 2 | 2 | 2 | 1 | - | - | 1 | 2 | - |
| Kenya | 535 | 293 | 242 | 18 | 15 | 24 | 18 | 19 | 13 | 152 | 145 | 21 | 12 | 18 | 10 | 13 | 16 | 7 | 8 | 21 | 5 |
| Lesotho | 75 | 41 | 34 | 2 | 2 | - | 1 | 4 | 2 | 24 | 19 | 4 | 1 | 2 | 3 | 2 | 3 | - | 1 | 3 | 2 |
| Liberia | 99 | 55 | 44 | 7 | 3 | 4 | 5 | 3 | 2 | 27 | 21 | 3 | 1 | 3 | - | 1 | 2 | 4 | 3 | 3 | 7 |
| Libya | 25 | 11 | 14 | - | - | 1 | 2 | 1 | - | 8 | 7 | - | 1 | - | 1 | - | - | - | 2 | 1 | 1 |
| Madagascar | 52 | 24 | 28 | - | - | - | 1 | 1 | 2 | 12 | 13 | 3 | 2 | 3 | 4 | 1 | - | - | 2 | 4 | 4 |
| Malawi | 13,467 | 6,627 | 6,840 | 418 | 442 | 503 | 527 | 474 | 508 | 2,906 | 3,401 | 321 | 311 | 257 | 290 | 241 | 267 | 237 | 269 | 1,270 | 825 |
| Mali | 217 | 142 | 75 | 3 | 3 | 1 | 3 | 3 | 7 | 46 | 50 | 26 | 2 | 28 | 3 | 20 | 2 | 9 | 1 | 6 | 4 |
| Mauritania | 3,360 | 1,995 | 1,365 | 122 | 126 | 136 | 165 | 141 | 155 | 1,020 | 680 | 149 | 41 | 110 | 43 | 72 | 25 | 69 | 41 | 176 | 89 |
| Mauritius | 59 | 28 | 31 | 1 | 2 | 2 | 2 | - | - | 14 | 12 | 2 | 3 | 4 | 2 | 2 | 5 | 2 | - | 1 | 5 |
| Morocco | 5 | 3 | 2 | - | - | - | - | - | - | 2 | 1 | - | - | - | 1 | - | - | - | - | 1 | - |
| Mozambique | 1,301 | 666 | 635 | 36 | 31 | 34 | 50 | 36 | 26 | 228 | 218 | 34 | 29 | 33 | 28 | 28 | 34 | 26 | 30 | 211 | 189 |
| Niger | 17 | 11 | 6 | 3 | - | 3 | - | - | - | 3 | 5 | - | - | 1 | 1 | - | - | - | - | 1 | - |
| Nigeria | 504 | 282 | 222 | 12 | 12 | 15 | 12 | 17 | 11 | 149 | 86 | 23 | 10 | 19 | 16 | 5 | 9 | 4 | 12 | 38 | 54 |
| Rwanda | 1,726 | 938 | 788 | 15 | 12 | 25 | 33 | 24 | 56 | 657 | 553 | 79 | 43 | 52 | 31 | 39 | 19 | 24 | 19 | 23 | 22 |
| Senegal | 142 | 107 | 35 | 2 | 1 | 1 | 2 | - | 1 | 36 | 21 | 15 | 2 | 18 | 1 | 19 | 3 | 10 | 2 | 6 | 2 |
| Sierra Leone | 62 | 39 | 23 | - | 2 | 1 | 1 | 2 | - | 19 | 10 | 2 | 2 | 4 | 2 | 5 | 1 | 2 | 1 | 4 | 4 |
| Somalia | 1,331 | 720 | 611 | 15 | 11 | 27 | 18 | 41 | 45 | 472 | 474 | 60 | 22 | 48 | 14 | 24 | 7 | 17 | 8 | 16 | 12 |
| South Africa | 3,068 | 1,560 | 1,508 | 180 | 216 | 147 | 130 | 103 | 107 | 550 | 477 | 121 | 95 | 142 | 117 | 116 | 101 | 67 | 77 | 134 | 188 |
| Namibia | 628 | 279 | 349 | 54 | 65 | 63 | 67 | 41 | 56 | 86 | 116 | 6 | 10 | 5 | 3 | 7 | 6 | 5 | 6 | 12 | 20 |
| Sudan | 85 | 46 | 39 | 4 | 5 | 1 | - | 2 | 1 | 27 | 18 | 1 | 4 | 2 | 3 | 3 | 2 | 2 | 1 | 4 | 5 |
| Swaziland | 111 | 53 | 58 | 5 | 2 | 6 | 2 | 5 | 8 | 29 | 24 | 3 | 5 | 1 | 3 | - | 1 | 2 | 3 | 2 | 10 |
| Tanzania | 5,917 | 2,964 | 2,953 | 159 | 160 | 159 | 207 | 171 | 221 | 1,567 | 1,539 | 180 | 155 | 134 | 144 | 131 | 126 | 120 | 107 | 343 | 294 |
| Togo | 53 | 30 | 23 | 3 | 4 | 1 | 2 | 2 | - | 14 | 13 | 3 | 2 | 3 | - | - | - | - | 2 | 4 | - |
| Tunisia | 278 | 132 | 146 | 11 | 14 | 12 | 11 | 4 | 6 | 40 | 50 | 9 | 10 | 25 | 12 | 12 | 11 | 1 | 6 | 18 | 26 |
| Uganda | 342 | 219 | 123 | 4 | 6 | 5 | 7 | 6 | 4 | 125 | 79 | 29 | 10 | 22 | 7 | 14 | 1 | 10 | 2 | 4 | 7 |
| Burkina Faso | 43 | 21 | 22 | 1 | - | 1 | - | 1 | 2 | 10 | 9 | - | 1 | 3 | - | 2 | 1 | 1 | 2 | 2 | 7 |
| Congo DR | 8,591 | 4,293 | 4,298 | 163 | 138 | 236 | 227 | 336 | 360 | 2,320 | 2,393 | 339 | 295 | 257 | 255 | 206 | 169 | 152 | 152 | 284 | 309 |
| Zimbabwe | 17,100 | 7,764 | 9,336 | 91 | 100 | 165 | 150 | 173 | 173 | 2,626 | 2,600 | 783 | 874 | 854 | 1,092 | 659 | 988 | 655 | 978 | 1,758 | 2,381 |
| Other Africa | 488 | 243 | 245 | 44 | 40 | 32 | 31 | 20 | 25 | 77 | 89 | 15 | 12 | 15 | 14 | 10 | 10 | 7 | 6 | 23 | 18 |
| Afghanistan | 579 | 292 | 287 | 45 | 44 | 44 | 36 | 31 | 46 | 144 | 127 | 7 | 15 | 5 | 4 | 4 | 4 | 7 | 3 | 5 | 8 |
| Australia | 264 | 136 | 128 | 23 | 23 | 16 | 16 | 10 | 17 | 64 | 54 | 9 | 6 | 7 | 5 | 6 | 2 | 1 | 2 | - | 3 |
| Bahrain | 81 | 38 | 43 | 5 | 5 | 13 | 7 | 7 | 8 | 11 | 18 | 1 | - | 1 | 3 | - | - | - | 2 | - | - |

## Table G1: Population Born Outside Zambia by Country of Birth, Age Group and Sex, Zambia 2010

| Country of Birth | Total | | | 0 - 4 | | 5 - 9 | | 10 - 14 | | 15-44 | | 45 - 49 | | 50 - 54 | | 55 - 59 | | 60 - 64 | | 65+ | |
|---|---|---|---|---|---|---|---|---|---|---|---|---|---|---|---|---|---|---|---|---|---|
| | Total | Male | Female | Male | Female | Male | Female | Male | Female | Male | Female | Male | Female | Male | Female | Male | Female | Male | Female | Male | Female |
| Bangladesh | 501 | 240 | 261 | 47 | 49 | 26 | 41 | 40 | 48 | 104 | 108 | 7 | 4 | 5 | 1 | 3 | 1 | 4 | 5 | 4 | 4 |
| Bhutan | 151 | 67 | 84 | 9 | 16 | 7 | 18 | 10 | 7 | 30 | 36 | 3 | 1 | 4 | 1 | 1 | - | 2 | 2 | 1 | 3 |
| Burma | 318 | 148 | 170 | 34 | 27 | 22 | 31 | 18 | 18 | 61 | 85 | 8 | 1 | 3 | 1 | - | 3 | 2 | 3 | - | 1 |
| China | 1,007 | 701 | 306 | 21 | 23 | 24 | 34 | 18 | 22 | 443 | 161 | 103 | 24 | 55 | 17 | 24 | 8 | 7 | 8 | 6 | 9 |
| Fiji | 305 | 153 | 152 | 24 | 16 | 15 | 19 | 17 | 16 | 76 | 73 | 5 | 7 | 3 | 7 | 4 | 4 | 1 | 5 | 8 | 5 |
| Hong Kong | 365 | 217 | 148 | 21 | 29 | 21 | 23 | 13 | 18 | 128 | 63 | 17 | 3 | 8 | 5 | 4 | 2 | 4 | 2 | 1 | 3 |
| India | 4,630 | 2,518 | 2,112 | 105 | 93 | 101 | 108 | 121 | 109 | 1,449 | 1,188 | 173 | 128 | 132 | 116 | 126 | 107 | 104 | 109 | 207 | 154 |
| Indonesia | 158 | 80 | 78 | 11 | 12 | 12 | 9 | 14 | 10 | 30 | 41 | 2 | - | 2 | - | 3 | 4 | 4 | 2 | 2 | - |
| Iran | 22 | 12 | 10 | - | - | 2 | 2 | 1 | - | 7 | 5 | - | 2 | 1 | - | 1 | - | - | 1 | - | - |
| Iraq | 467 | 256 | 211 | 3 | 4 | 9 | 6 | 13 | 9 | 122 | 110 | 18 | 24 | 19 | 15 | 15 | 8 | 17 | 9 | 40 | 26 |
| Israel | 24 | 13 | 11 | 1 | 1 | 2 | 1 | - | 1 | 8 | 4 | 1 | - | 1 | 3 | - | 1 | - | - | - | - |
| Japan | 100 | 50 | 50 | 4 | 1 | 2 | 4 | - | 2 | 29 | 35 | 4 | 4 | 5 | 1 | 2 | 1 | 2 | - | 2 | 2 |
| Jordan | 9 | 4 | 5 | - | - | - | 2 | - | 2 | 3 | 1 | 1 | - | - | - | - | - | - | - | - | - |
| Kampuchea | 10 | 6 | 4 | - | - | - | - | - | - | 3 | 2 | - | - | - | 1 | 2 | - | - | 1 | 1 | - |
| North Korea | 39 | 23 | 16 | 1 | 1 | - | - | 2 | 4 | 7 | 8 | 7 | 2 | 3 | 1 | 3 | - | - | - | - | - |
| South Korea | 49 | 27 | 22 | - | 1 | 2 | - | 5 | - | 10 | 12 | 5 | 3 | - | 3 | 3 | 1 | - | 1 | 2 | 1 |
| Kuwait | 15 | 11 | 4 | 1 | - | - | - | - | - | 9 | - | - | 3 | 1 | - | - | 1 | - | - | - | - |
| Lebanon | 383 | 229 | 154 | 22 | 24 | 12 | 18 | 20 | 5 | 146 | 94 | 12 | 8 | 4 | 1 | 9 | 1 | 2 | 1 | 2 | 2 |
| Malaysia | 12 | 8 | 4 | 1 | - | - | 1 | - | - | 3 | 2 | - | 1 | 2 | - | - | - | - | - | 2 | - |
| Mongolia | 3 | 1 | 2 | - | - | - | - | - | - | - | 2 | 1 | - | - | - | - | - | - | - | - | - |
| Nepal | 47 | 30 | 17 | 2 | 2 | 3 | 1 | 2 | 1 | 21 | 9 | 2 | 1 | - | 1 | - | 1 | - | 1 | - | - |
| New Zealand | 54 | 36 | 18 | 3 | 2 | 4 | 1 | 1 | 2 | 14 | 9 | 4 | - | 3 | 1 | 3 | 2 | 2 | - | 2 | 1 |
| Oman | 8 | 6 | 2 | 2 | 1 | - | - | - | - | 1 | 1 | - | - | 1 | - | - | - | 1 | - | 1 | - |
| Pakistan | 156 | 91 | 65 | 5 | 3 | 2 | 6 | 10 | 5 | 49 | 38 | 5 | 3 | 8 | 5 | 3 | 3 | 4 | 1 | 5 | 1 |
| Papua-New Guinea | 3 | 2 | 1 | - | 1 | - | - | 1 | - | 1 | - | - | - | - | - | - | - | - | - | - | - |
| Philippines | 96 | 51 | 45 | 1 | - | 4 | 1 | 1 | 2 | 23 | 22 | 6 | 5 | 5 | 5 | 6 | 4 | 3 | 5 | 2 | 1 |
| Saudi Arabia | 17 | 5 | 12 | - | - | - | - | - | - | 4 | 11 | 1 | 1 | - | - | - | - | - | - | - | - |
| Singapore | 8 | 5 | 3 | - | - | - | - | - | - | 3 | 3 | 1 | - | 1 | - | - | - | - | - | - | - |
| Sri Lanka | 142 | 79 | 63 | 2 | 4 | 2 | 5 | 5 | 3 | 32 | 27 | 10 | 6 | 9 | 7 | 5 | 3 | 7 | 3 | 7 | 5 |
| Syria | 22 | 17 | 5 | 3 | - | 3 | - | - | 1 | 8 | 3 | 1 | 1 | - | - | 2 | - | - | - | - | - |
| Taiwan | 8 | 6 | 2 | - | - | - | - | - | 2 | 1 | - | - | - | 1 | - | 2 | - | 2 | - | - | - |
| Thailand | 36 | 17 | 19 | 1 | 2 | 2 | 1 | 1 | 1 | 8 | 12 | 1 | - | 2 | 1 | - | - | 1 | 1 | 1 | 1 |
| United Arab Emirates | 8 | 4 | 4 | 1 | - | 1 | - | 2 | 3 | - | 1 | - | - | - | - | - | - | - | - | - | - |
| Vietnam | 4 | 1 | 3 | - | - | - | 1 | - | - | 1 | 2 | - | - | - | - | - | - | - | - | - | - |
| Yemen | 23 | 16 | 7 | 2 | - | 3 | 1 | - | - | 8 | 5 | 1 | - | - | - | 1 | - | 1 | 1 | - | - |
| Other Asia and Oceania | 86 | 45 | 41 | 6 | 3 | 2 | 3 | 5 | 1 | 15 | 18 | 6 | 5 | 2 | 1 | 1 | 2 | 3 | 3 | 5 | 5 |
| Argentina | 6 | 5 | 1 | - | - | - | - | 2 | - | 2 | 1 | - | - | - | - | - | - | - | - | 1 | - |
| Barbados | 15 | 4 | 11 | - | 2 | - | 2 | 1 | - | 3 | 6 | - | - | - | - | - | 1 | - | - | - | - |
| Bolivia | 13 | 7 | 6 | - | 3 | 1 | - | 1 | 1 | 4 | 2 | 1 | - | - | - | - | - | - | - | - | - |
| Brazil | 47 | 23 | 24 | - | 2 | 2 | 1 | - | 1 | 16 | 13 | - | 2 | 3 | 3 | 2 | 1 | - | - | - | 1 |
| Canada | 200 | 94 | 106 | 9 | 10 | 4 | 7 | 6 | 6 | 44 | 64 | 8 | - | 6 | 7 | 7 | 7 | 4 | - | 6 | 5 |
| Chile | 13 | 8 | 5 | - | 1 | 1 | - | - | 1 | 5 | 1 | 1 | 2 | - | - | - | - | 1 | - | - | - |
| Colombia | 12 | 5 | 7 | - | - | - | - | 1 | - | 1 | 2 | - | - | 1 | 2 | - | 1 | 1 | 1 | 1 | 1 |
| Dominican Republic | 3 | 1 | 2 | - | - | - | - | 1 | - | - | 2 | - | - | - | - | - | - | - | - | - | - |
| Ecuador | 3 | 2 | 1 | - | 1 | 1 | - | - | - | 1 | - | - | - | - | - | - | - | - | - | - | - |
| El Salvador | 1 | 1 | - | - | - | - | - | - | - | - | - | - | - | - | - | - | - | - | - | 1 | - |
| Guatemala | 4 | 2 | 2 | - | - | - | - | - | - | 2 | 2 | - | - | - | - | - | - | - | - | - | - |
| Guyana | 14 | 5 | 9 | - | - | 1 | 1 | - | 1 | 1 | 4 | - | - | - | 1 | - | 1 | - | 1 | 3 | - |
| Honduras | 1 | - | 1 | - | - | - | - | - | - | - | - | - | - | - | - | - | - | - | - | - | 1 |
| Jamaica | 9 | 5 | 4 | - | - | - | - | - | - | 2 | 1 | - | - | 2 | 1 | - | 1 | - | 1 | 1 | - |
| Mexico | 14 | 6 | 8 | 1 | 1 | 1 | - | - | 1 | 2 | 2 | - | - | - | 2 | 1 | - | - | 1 | 1 | 1 |
| Paraguay | 7 | 6 | 1 | - | - | - | - | 3 | - | 2 | - | 1 | 1 | - | - | - | - | - | - | - | - |
| Trinidad and Tobago | 2 | 1 | 1 | - | - | - | - | - | - | 1 | - | - | 1 | - | - | - | - | - | - | - | - |
| Uruguay | 5 | 2 | 3 | - | - | - | - | - | - | 2 | 1 | - | 1 | - | - | - | - | - | - | - | 1 |

## Table G1: Population Born Outside Zambia by Country of Birth, Age Group and Sex, Zambia 2010

| Country of Birth | Total | | | 0 - 4 | | 5 - 9 | | 10 - 14 | | 15-44 | | 45 - 49 | | 50 - 54 | | 55 - 59 | | 60 - 64 | | 65+ | |
|---|---|---|---|---|---|---|---|---|---|---|---|---|---|---|---|---|---|---|---|---|---|
| | Total | Male | Female | Male | Female | Male | Female | Male | Female | Male | Female | Male | Female | Male | Female | Male | Female | Male | Female | Male | Female |
| United States of America | 949 | 447 | 502 | 46 | 61 | 67 | 72 | 41 | 33 | 187 | 227 | 24 | 22 | 30 | 26 | 22 | 22 | 12 | 17 | 18 | 22 |
| Venezuela | 6 | 1 | 5 | - | - | - | 1 | - | 2 | 1 | 2 | - | - | - | - | - | - | - | - | - | - |
| Other Americas | 140 | 84 | 56 | 7 | 7 | 5 | 4 | 5 | 3 | 40 | 30 | 11 | 3 | 6 | 5 | 1 | 2 | 4 | 1 | 5 | 1 |
| Albania | 24 | 15 | 9 | 4 | 2 | 1 | - | - | 1 | 7 | 5 | 3 | 1 | - | - | - | - | - | - | - | - |
| Austria | 39 | 20 | 19 | 2 | 2 | - | 1 | - | 1 | 11 | 11 | 1 | - | 2 | 1 | - | 1 | 2 | 1 | 2 | 1 |
| Belgium | 54 | 30 | 24 | 4 | 2 | 1 | 3 | 1 | 2 | 16 | 13 | 3 | 3 | - | - | 3 | 1 | 2 | - | - | - |
| Bulgaria | 54 | 24 | 30 | 3 | 2 | - | 5 | 3 | 8 | 13 | 12 | 1 | - | 2 | 2 | 1 | 1 | 1 | - | - | - |
| Cyprus | 34 | 18 | 16 | - | 2 | 1 | - | 1 | - | 8 | 5 | - | - | - | 2 | 2 | 2 | - | 2 | 6 | 3 |
| Czechoslovakia | 32 | 19 | 13 | 4 | 2 | 2 | 3 | 1 | - | 8 | 4 | - | 1 | - | - | 1 | 1 | 1 | 2 | 2 | - |
| Denmark | 87 | 47 | 40 | 3 | 3 | 4 | 6 | 1 | 3 | 18 | 16 | 5 | 2 | 3 | 2 | 2 | 1 | 4 | - | 7 | 7 |
| Finland | 61 | 33 | 28 | 1 | - | 3 | 3 | 9 | 1 | 14 | 17 | 2 | - | - | 2 | 1 | 1 | 1 | 2 | 2 | 2 |
| France | 70 | 41 | 29 | 3 | 3 | 3 | 1 | - | 3 | 22 | 18 | 1 | 1 | 4 | - | 2 | 1 | 2 | 1 | 4 | 1 |
| Germany | 279 | 151 | 128 | 14 | 12 | 12 | 6 | 4 | 5 | 69 | 67 | 6 | 4 | 7 | 10 | 7 | 8 | 16 | 6 | 16 | 10 |
| Greece | 90 | 64 | 26 | 2 | 2 | 1 | 1 | - | 1 | 20 | 6 | 7 | 4 | 6 | 5 | 6 | 2 | 5 | 1 | 17 | 4 |
| Hungary | 10 | 4 | 6 | - | 2 | - | - | - | - | 4 | 2 | - | - | - | - | - | 2 | - | - | - | - |
| Ireland | 117 | 50 | 67 | - | 2 | 3 | 1 | 2 | 3 | 6 | 12 | 5 | 3 | 2 | 4 | 9 | 9 | 4 | 10 | 19 | 23 |
| Italy | 157 | 86 | 71 | - | 4 | 1 | 1 | 4 | 2 | 21 | 27 | 11 | 4 | 4 | 6 | 3 | 3 | 8 | 9 | 34 | 15 |
| Luxemburg | 11 | 6 | 5 | - | - | - | - | - | - | 4 | 2 | - | 2 | - | - | 1 | 1 | - | - | 1 | - |
| Malta | 39 | 20 | 19 | - | 2 | - | - | 1 | - | 8 | 12 | 4 | - | - | - | 3 | - | 3 | 2 | 1 | 3 |
| Netherlands | 109 | 62 | 47 | 4 | 6 | 5 | 2 | - | 3 | 16 | 21 | 5 | 6 | 5 | 1 | 7 | 2 | 10 | 3 | 10 | 3 |
| Norway | 39 | 18 | 21 | 1 | 2 | 2 | - | 2 | 1 | 6 | 14 | - | - | - | 1 | 1 | - | 1 | 1 | 5 | 2 |
| Poland | 59 | 35 | 24 | - | 2 | - | 1 | - | - | 15 | 13 | 5 | - | 4 | 3 | 3 | 2 | 4 | 1 | 4 | 2 |
| Portugal | 45 | 24 | 21 | - | 1 | 1 | - | 3 | 1 | 9 | 7 | 2 | 2 | 2 | 1 | 1 | 1 | 2 | 1 | 4 | 7 |
| Romania | 45 | 25 | 20 | 4 | 3 | 2 | 2 | - | - | 12 | 11 | 1 | 2 | 4 | 1 | - | - | - | 1 | 2 | - |
| Spain | 49 | 31 | 18 | 1 | 3 | 2 | 2 | - | - | 20 | 12 | 4 | - | 1 | 1 | 2 | - | 1 | - | - | - |
| Sweden | 50 | 24 | 26 | 3 | 1 | 1 | 2 | 1 | 1 | 6 | 17 | 4 | 2 | 1 | 1 | - | - | 5 | 1 | 3 | 1 |
| Switzerland | 57 | 29 | 28 | 2 | 2 | 2 | 4 | 2 | 1 | 11 | 10 | 1 | 2 | 3 | - | - | 1 | 1 | 1 | 7 | 7 |
| Turkey | 22 | 15 | 7 | - | 1 | 1 | 1 | 1 | 1 | 8 | 4 | 1 | - | 1 | - | 1 | - | - | - | 2 | - |
| United Kingdom | 1,286 | 680 | 606 | 59 | 68 | 56 | 60 | 53 | 33 | 243 | 254 | 49 | 28 | 37 | 27 | 38 | 33 | 59 | 39 | 86 | 64 |
| Russia | 74 | 31 | 43 | 2 | - | - | 1 | 2 | - | 18 | 25 | 2 | 4 | 2 | 6 | 3 | 1 | - | 2 | 2 | 4 |
| Yugoslavia | 26 | 15 | 11 | - | - | 1 | - | - | - | 1 | 5 | 1 | - | 2 | 2 | 3 | 3 | 3 | 1 | 4 | - |
| Other Europe | 298 | 164 | 134 | 11 | 9 | 5 | 5 | 6 | 5 | 65 | 64 | 6 | 10 | 9 | 5 | 18 | 8 | 9 | 10 | 35 | 18 |

## Table G2: Population (De facto) by Rural/Urban Part of District of Birth, Age, Sex and District of Residence, Zambia 2010

| Rural/Urban Part of District of Birth | Age and Sex | | | | | | | | | | | | | | | | | | | | |
|---|---|---|---|---|---|---|---|---|---|---|---|---|---|---|---|---|---|---|---|---|---|
| | Total | | | 0-4 | | 5-9 | | 10-14 | | 15-44 | | 45-49 | | 50-54 | | 55-59 | | 60-64 | | 65+ | |
| | Total | Male | Female | Male | Female | Male | Female | Male | Female | Male | Female | Male | Female | Male | Female | Male | Female | Male | Female | Male | Female |
| **BORN IN ZAMBIA** | | | | | | | | | | | | | | | | | | | | | |
| **Total** | **12,424,534** | **6,065,886** | **6,358,648** | **1,099,764** | **1,110,070** | **921,584** | **928,904** | **837,489** | **854,653** | **2,601,509** | **2,799,482** | **170,911** | **176,966** | **125,273** | **138,388** | **87,329** | **92,222** | **71,062** | **85,904** | **150,965** | **172,059** |
| | | | | | | | | | | | | | | | | | | | | | |
| **Central** | **1,148,590** | **561,533** | **587,057** | **109,620** | **110,892** | **92,032** | **92,391** | **83,389** | **85,378** | **231,038** | **248,691** | **13,018** | **13,489** | **9,334** | **10,452** | **6,332** | **6,771** | **5,344** | **6,379** | **11,426** | **12,614** |
| Chibombo | 216,129 | 107,373 | 108,756 | 24,485 | 24,583 | 19,639 | 19,519 | 17,404 | 17,090 | 39,281 | 40,245 | 1,954 | 1,995 | 1,291 | 1,443 | 852 | 940 | 752 | 907 | 1,715 | 2,034 |
| Kabwe | 252,585 | 120,818 | 131,767 | 16,500 | 16,659 | 14,846 | 15,331 | 16,156 | 17,518 | 63,526 | 71,240 | 3,226 | 3,454 | 2,201 | 2,752 | 1,487 | 1,606 | 1,111 | 1,235 | 1,765 | 1,972 |
| Kapiri-Mposhi | 168,559 | 82,795 | 85,764 | 19,931 | 20,226 | 16,341 | 16,483 | 14,316 | 14,306 | 28,058 | 30,118 | 1,187 | 1,207 | 819 | 931 | 553 | 648 | 464 | 612 | 1,126 | 1,233 |
| Mkushi | 136,324 | 66,756 | 69,568 | 13,591 | 13,866 | 10,997 | 10,980 | 9,522 | 9,970 | 25,765 | 27,799 | 1,836 | 1,753 | 1,416 | 1,374 | 905 | 916 | 832 | 973 | 1,892 | 1,937 |
| Mumbwa | 192,750 | 94,407 | 98,343 | 19,115 | 19,222 | 16,167 | 15,987 | 13,751 | 14,181 | 38,296 | 40,654 | 2,102 | 2,222 | 1,436 | 1,656 | 971 | 1,151 | 710 | 999 | 1,859 | 2,271 |
| Serenje | 182,243 | 89,384 | 92,859 | 15,998 | 16,336 | 14,042 | 14,091 | 12,240 | 12,313 | 36,112 | 38,635 | 2,713 | 2,858 | 2,171 | 2,296 | 1,564 | 1,510 | 1,475 | 1,653 | 3,069 | 3,167 |
| | | | | | | | | | | | | | | | | | | | | | |
| **Copper-belt** | **1,953,742** | **950,025** | **1,003,717** | **143,355** | **144,385** | **122,158** | **123,737** | **129,119** | **133,571** | **485,488** | **526,662** | **26,199** | **27,366** | **16,699** | **19,135** | **10,696** | **11,210** | **6,798** | **7,715** | **9,513** | **9,936** |
| Chilila-bombwe | 84,772 | 41,744 | 43,028 | 6,268 | 6,203 | 5,187 | 5,216 | 5,800 | 5,878 | 21,991 | 23,190 | 1,253 | 1,295 | 660 | 662 | 279 | 262 | 148 | 145 | 158 | 177 |
| Chingola | 228,245 | 110,878 | 117,367 | 15,871 | 16,030 | 13,663 | 13,784 | 14,935 | 15,249 | 58,709 | 64,227 | 3,073 | 3,239 | 2,038 | 2,301 | 1,246 | 1,175 | 663 | 688 | 680 | 674 |
| Kalulushi | 72,462 | 35,478 | 36,984 | 6,370 | 6,364 | 5,164 | 5,191 | 5,046 | 5,171 | 16,963 | 18,279 | 817 | 844 | 506 | 523 | 264 | 268 | 135 | 137 | 213 | 207 |
| Kitwe | 509,278 | 247,344 | 261,934 | 37,818 | 38,609 | 31,404 | 32,046 | 32,490 | 34,471 | 129,520 | 139,866 | 6,504 | 6,568 | 3,849 | 4,439 | 2,454 | 2,591 | 1,487 | 1,638 | 1,818 | 1,706 |
| Luanshya | 185,396 | 89,464 | 95,932 | 10,496 | 10,495 | 9,205 | 9,301 | 11,312 | 11,712 | 48,803 | 53,678 | 3,281 | 3,484 | 2,262 | 2,821 | 1,614 | 1,775 | 1,065 | 1,251 | 1,426 | 1,415 |
| Lufwan-yama | 58,151 | 28,270 | 29,881 | 6,282 | 6,336 | 5,160 | 5,250 | 4,366 | 4,475 | 10,059 | 11,150 | 627 | 686 | 494 | 535 | 381 | 392 | 285 | 355 | 616 | 702 |
| Masaiti | 80,235 | 39,714 | 40,521 | 7,603 | 7,803 | 6,860 | 6,687 | 6,203 | 5,996 | 15,359 | 15,884 | 921 | 1,053 | 710 | 804 | 523 | 601 | 495 | 566 | 1,040 | 1,127 |
| Mpongwe | 71,354 | 35,109 | 36,245 | 8,113 | 8,013 | 6,482 | 6,588 | 5,472 | 5,441 | 12,617 | 13,402 | 704 | 726 | 500 | 538 | 307 | 390 | 261 | 372 | 653 | 775 |
| Mufulira | 197,102 | 95,082 | 102,020 | 11,573 | 11,615 | 10,121 | 10,055 | 11,703 | 11,835 | 51,844 | 57,518 | 3,629 | 3,994 | 2,463 | 2,920 | 1,670 | 1,740 | 986 | 1,221 | 1,093 | 1,122 |
| Ndola | 466,747 | 226,942 | 239,805 | 32,961 | 32,917 | 28,912 | 29,619 | 31,792 | 33,343 | 119,623 | 129,468 | 5,390 | 5,477 | 3,217 | 3,592 | 1,958 | 2,016 | 1,273 | 1,342 | 1,816 | 2,031 |
| | | | | | | | | | | | | | | | | | | | | | |
| **Eastern** | **1,746,789** | **857,596** | **889,193** | **150,880** | **153,159** | **130,814** | **131,629** | **116,307** | **116,468** | **350,232** | **363,814** | **28,178** | **28,701** | **21,487** | **23,453** | **15,866** | **16,658** | **13,719** | **17,506** | **30,113** | **37,805** |
| Chadiza | 108,673 | 53,705 | 54,968 | 9,696 | 9,906 | 8,476 | 8,497 | 7,305 | 7,456 | 21,761 | 22,105 | 1,717 | 1,688 | 1,238 | 1,299 | 966 | 951 | 780 | 959 | 1,766 | 2,107 |
| Chama | 105,934 | 51,903 | 54,031 | 10,066 | 10,051 | 8,779 | 8,786 | 7,250 | 7,060 | 19,880 | 21,432 | 1,564 | 1,667 | 1,084 | 1,291 | 812 | 852 | 765 | 991 | 1,703 | 1,901 |
| Chipata | 512,922 | 253,237 | 259,685 | 39,642 | 40,449 | 34,610 | 34,912 | 32,314 | 33,455 | 111,909 | 112,093 | 8,962 | 8,884 | 6,955 | 7,265 | 5,050 | 5,192 | 4,296 | 5,173 | 9,499 | 12,262 |
| Katete | 253,023 | 123,906 | 129,117 | 22,329 | 22,733 | 19,313 | 19,460 | 17,428 | 17,024 | 50,330 | 53,029 | 4,049 | 4,128 | 2,803 | 3,072 | 2,193 | 2,290 | 1,826 | 2,599 | 3,635 | 4,782 |
| Lundazi | 327,261 | 160,663 | 166,598 | 28,879 | 29,506 | 25,127 | 25,494 | 21,972 | 22,005 | 62,812 | 66,492 | 5,381 | 5,407 | 4,249 | 4,569 | 3,264 | 3,212 | 2,851 | 3,421 | 6,128 | 6,492 |
| Mambwe | 52,446 | 25,530 | 26,916 | 5,323 | 5,363 | 4,613 | 4,644 | 3,891 | 3,826 | 9,300 | 10,143 | 584 | 638 | 464 | 511 | 329 | 349 | 270 | 463 | 756 | 979 |
| Nyimba | 77,971 | 38,222 | 39,749 | 7,433 | 7,373 | 6,385 | 6,369 | 5,609 | 5,341 | 14,859 | 15,825 | 1,054 | 1,125 | 825 | 935 | 477 | 587 | 498 | 639 | 1,082 | 1,555 |
| Petauke | 308,559 | 150,430 | 158,129 | 27,512 | 27,778 | 23,511 | 23,467 | 20,538 | 20,301 | 59,381 | 62,695 | 4,867 | 5,164 | 3,869 | 4,511 | 2,775 | 3,225 | 2,433 | 3,261 | 5,544 | 7,727 |

## Table G2: Population (De facto) by Rural/Urban Part of District of Birth, Age, Sex and District of Residence, Zambia 2010

| Rural/Urban Part of District of Birth | Age and Sex | | | | | | | | | | | | | | | | | | | | |
|---|---|---|---|---|---|---|---|---|---|---|---|---|---|---|---|---|---|---|---|---|---|
| | Total | | | 0-4 | | 5-9 | | 10-14 | | 15-44 | | 45-49 | | 50-54 | | 55-59 | | 60-64 | | 65+ | |
| | Total | Male | Female | Male | Female | Male | Female | Male | Female | Male | Female | Male | Female | Male | Female | Male | Female | Male | Female | Male | Female |
| **Luapula** | **1,015,686** | **496,335** | **519,351** | **90,829** | **92,124** | **77,680** | **77,984** | **67,919** | **68,499** | **196,754** | **215,170** | **16,621** | **16,811** | **13,672** | **13,880** | **9,403** | **9,831** | **8,158** | **9,113** | **15,299** | **15,939** |
| Chienge | 109,413 | 53,439 | 55,974 | 11,433 | 11,689 | 8,686 | 8,686 | 7,095 | 7,181 | 20,494 | 22,753 | 1,649 | 1,590 | 1,372 | 1,368 | 893 | 883 | 731 | 704 | 1,086 | 1,120 |
| Kawambwa | 151,612 | 74,107 | 77,505 | 12,214 | 12,587 | 11,104 | 11,049 | 10,152 | 9,982 | 29,653 | 32,309 | 2,562 | 2,652 | 2,197 | 2,305 | 1,587 | 1,681 | 1,471 | 1,648 | 3,167 | 3,292 |
| Mansa | 243,348 | 119,816 | 123,532 | 20,172 | 20,306 | 17,420 | 17,652 | 15,698 | 16,271 | 51,096 | 53,691 | 4,076 | 4,029 | 3,412 | 3,408 | 2,377 | 2,393 | 2,037 | 2,152 | 3,528 | 3,630 |
| Milenge | 38,734 | 18,633 | 20,101 | 4,021 | 4,168 | 3,450 | 3,441 | 2,870 | 2,916 | 6,569 | 7,585 | 466 | 526 | 404 | 401 | 225 | 273 | 208 | 303 | 420 | 488 |
| Mwense | 133,090 | 65,068 | 68,022 | 10,846 | 10,894 | 9,844 | 9,811 | 8,702 | 8,856 | 25,768 | 28,421 | 2,659 | 2,649 | 2,213 | 2,131 | 1,469 | 1,526 | 1,263 | 1,451 | 2,304 | 2,283 |
| Nchelenge | 130,596 | 64,241 | 66,355 | 13,328 | 13,685 | 11,054 | 11,191 | 9,855 | 9,655 | 24,400 | 26,223 | 1,709 | 1,635 | 1,236 | 1,163 | 863 | 930 | 641 | 696 | 1,155 | 1,177 |
| Samfya | 208,893 | 101,031 | 107,862 | 18,815 | 18,795 | 16,122 | 16,154 | 13,547 | 13,638 | 38,774 | 44,188 | 3,500 | 3,730 | 2,838 | 3,104 | 1,989 | 2,145 | 1,807 | 2,159 | 3,639 | 3,949 |
| | | | | | | | | | | | | | | | | | | | | | |
| **Lusaka** | **1,557,482** | **760,364** | **797,118** | **156,524** | **158,149** | **124,042** | **125,733** | **113,010** | **119,147** | **337,629** | **362,136** | **11,147** | **11,008** | **6,495** | **7,523** | **3,904** | **4,222** | **2,723** | **3,046** | **4,890** | **6,154** |
| Chongwe | 122,568 | 60,390 | 62,178 | 13,014 | 13,222 | 10,410 | 10,154 | 9,040 | 9,237 | 23,271 | 24,223 | 1,361 | 1,379 | 985 | 1,157 | 602 | 626 | 514 | 669 | 1,193 | 1,511 |
| Kafue | 118,677 | 58,294 | 60,383 | 12,991 | 13,018 | 10,002 | 10,002 | 9,207 | 9,259 | 23,859 | 25,506 | 718 | 822 | 506 | 572 | 316 | 318 | 214 | 274 | 481 | 612 |
| Luangwa | 30,028 | 14,325 | 15,703 | 2,075 | 2,144 | 1,984 | 2,019 | 1,924 | 1,895 | 5,899 | 6,539 | 554 | 673 | 461 | 547 | 343 | 376 | 331 | 412 | 754 | 1,098 |
| Lusaka | 1,286,209 | 627,355 | 658,854 | 128,444 | 129,765 | 101,646 | 103,558 | 92,839 | 98,756 | 284,600 | 305,868 | 8,514 | 8,134 | 4,543 | 5,247 | 2,643 | 2,902 | 1,664 | 1,691 | 2,462 | 2,933 |
| | | | | | | | | | | | | | | | | | | | | | |
| **Northern** | **1,794,363** | **880,395** | **913,968** | **159,755** | **161,065** | **136,301** | **137,885** | **117,965** | **118,717** | **351,560** | **376,006** | **29,368** | **29,741** | **22,297** | **23,440** | **16,387** | **16,741** | **14,334** | **16,661** | **32,428** | **33,712** |
| Chilubi | 83,435 | 39,740 | 43,695 | 8,348 | 8,315 | 6,876 | 6,897 | 5,438 | 5,403 | 14,471 | 17,502 | 1,203 | 1,474 | 922 | 1,040 | 658 | 784 | 568 | 805 | 1,256 | 1,475 |
| Chinsali | 173,273 | 84,660 | 88,613 | 13,789 | 14,193 | 12,749 | 12,845 | 11,383 | 11,325 | 34,065 | 36,334 | 3,139 | 3,209 | 2,355 | 2,492 | 1,756 | 1,926 | 1,546 | 2,017 | 3,878 | 4,272 |
| Isoka | 159,323 | 78,883 | 80,440 | 12,608 | 12,733 | 11,331 | 11,498 | 9,944 | 9,936 | 31,864 | 33,179 | 3,060 | 3,116 | 2,595 | 2,659 | 2,031 | 1,817 | 1,775 | 1,887 | 3,675 | 3,615 |
| Kaputa | 109,152 | 53,263 | 55,889 | 11,343 | 11,497 | 8,701 | 8,897 | 7,277 | 7,194 | 20,292 | 22,943 | 1,535 | 1,445 | 1,248 | 1,254 | 956 | 896 | 691 | 724 | 1,220 | 1,039 |
| Kasama | 265,820 | 131,370 | 134,450 | 20,616 | 20,712 | 18,213 | 18,815 | 16,734 | 17,575 | 58,733 | 59,337 | 4,555 | 4,491 | 3,272 | 3,368 | 2,381 | 2,520 | 2,080 | 2,419 | 4,786 | 5,213 |
| Luwingu | 129,383 | 63,138 | 66,245 | 11,173 | 11,225 | 9,737 | 9,729 | 8,605 | 8,412 | 24,182 | 26,811 | 2,334 | 2,421 | 1,834 | 1,954 | 1,308 | 1,393 | 1,234 | 1,373 | 2,731 | 2,927 |
| Mbala | 231,483 | 114,470 | 117,013 | 19,766 | 19,752 | 17,057 | 17,162 | 14,929 | 15,033 | 46,854 | 49,380 | 4,273 | 4,025 | 3,096 | 3,333 | 2,361 | 2,267 | 1,929 | 2,093 | 4,205 | 3,968 |
| Mpika | 201,977 | 98,466 | 103,511 | 18,168 | 18,290 | 15,798 | 15,856 | 13,677 | 14,111 | 39,663 | 43,121 | 2,845 | 2,926 | 2,184 | 2,226 | 1,486 | 1,633 | 1,415 | 1,721 | 3,230 | 3,627 |
| Mporokoso | 116,723 | 57,454 | 59,269 | 9,495 | 9,502 | 8,600 | 8,699 | 7,672 | 7,710 | 22,576 | 23,865 | 2,045 | 2,181 | 1,639 | 1,726 | 1,272 | 1,334 | 1,174 | 1,422 | 2,981 | 2,830 |
| Mpulungu | 82,874 | 40,521 | 42,353 | 9,331 | 9,355 | 6,951 | 7,204 | 5,813 | 5,661 | 14,976 | 16,592 | 1,063 | 1,034 | 719 | 740 | 489 | 457 | 347 | 437 | 832 | 873 |
| Mungwi | 128,514 | 62,578 | 65,936 | 14,181 | 14,233 | 11,279 | 11,195 | 9,190 | 8,973 | 21,452 | 24,199 | 1,642 | 1,843 | 1,222 | 1,402 | 799 | 896 | 783 | 948 | 2,030 | 2,247 |
| Nakonde | 112,406 | 55,852 | 56,554 | 10,937 | 11,258 | 9,009 | 9,088 | 7,303 | 7,384 | 22,432 | 22,743 | 1,674 | 1,576 | 1,211 | 1,246 | 890 | 818 | 792 | 815 | 1,604 | 1,626 |
| | | | | | | | | | | | | | | | | | | | | | |
| **North Western** | **692,105** | **340,297** | **351,808** | **65,619** | **65,974** | **53,627** | **53,921** | **46,934** | **47,397** | **132,586** | **142,009** | **10,752** | **10,554** | **8,111** | **8,268** | **5,934** | **5,837** | **4,746** | **5,595** | **11,988** | **12,253** |
| Chavuma | 37,470 | 18,048 | 19,422 | 3,479 | 3,437 | 2,790 | 2,856 | 2,363 | 2,455 | 7,181 | 8,013 | 571 | 591 | 360 | 461 | 262 | 307 | 233 | 338 | 809 | 964 |
| Kabompo | 91,500 | 44,869 | 46,631 | 8,328 | 8,252 | 7,132 | 7,123 | 6,396 | 6,489 | 17,502 | 18,993 | 1,351 | 1,353 | 993 | 1,115 | 806 | 808 | 600 | 749 | 1,761 | 1,749 |
| Kasempa | 76,581 | 37,207 | 39,374 | 6,861 | 6,954 | 5,899 | 5,795 | 5,190 | 5,066 | 14,500 | 16,517 | 1,214 | 1,272 | 923 | 1,018 | 679 | 692 | 563 | 690 | 1,378 | 1,370 |
| Mufumbwe | 40,133 | 19,694 | 20,439 | 5,097 | 5,102 | 3,950 | 3,951 | 3,041 | 3,161 | 6,256 | 6,856 | 388 | 376 | 239 | 275 | 191 | 188 | 164 | 164 | 368 | 366 |
| Mwinilunga | 159,166 | 78,874 | 80,292 | 13,672 | 13,576 | 11,598 | 11,708 | 10,712 | 10,622 | 31,925 | 33,277 | 2,875 | 2,808 | 2,299 | 2,179 | 1,575 | 1,567 | 1,327 | 1,510 | 2,891 | 3,045 |
| Solwezi | 192,558 | 95,015 | 97,543 | 20,603 | 21,024 | 15,711 | 15,943 | 13,391 | 13,656 | 36,528 | 38,323 | 2,529 | 2,356 | 1,854 | 1,780 | 1,338 | 1,204 | 1,016 | 1,116 | 2,045 | 2,141 |
| Zambezi | 94,697 | 46,590 | 48,107 | 7,579 | 7,629 | 6,547 | 6,545 | 5,841 | 5,948 | 18,694 | 20,030 | 1,824 | 1,798 | 1,443 | 1,440 | 1,083 | 1,071 | 843 | 1,028 | 2,736 | 2,618 |

## Table G2: Population (De facto) by Rural/Urban Part of District of Birth, Age, Sex and District of Residence, Zambia 2010

| Rural/Urban Part of District of Birth | Age and Sex | | | | | | | | | | | | | | | | | | | |
|---|---|---|---|---|---|---|---|---|---|---|---|---|---|---|---|---|---|---|---|---|
| | Total | | | 0-4 | | 5-9 | | 10-14 | | 15-44 | | 45-49 | | 50-54 | | 55-59 | | 60-64 | | 65+ | |
| | Total | Male | Female | Male | Female | Male | Female | Male | Female | Male | Female | Male | Female | Male | Female | Male | Female | Male | Female | Male | Female |
| **Southern** | **1,619,231** | **788,087** | **831,144** | **143,559** | **144,698** | **119,880** | **120,937** | **106,937** | **108,874** | **343,483** | **368,612** | **22,110** | **23,788** | **15,768** | **18,549** | **10,675** | **11,612** | **8,271** | **10,722** | **17,404** | **23,352** |
| Choma | 312,753 | 152,046 | 160,707 | 24,768 | 24,941 | 21,044 | 21,739 | 19,708 | 20,088 | 70,084 | 74,977 | 4,877 | 5,097 | 3,442 | 4,060 | 2,503 | 2,617 | 1,808 | 2,337 | 3,812 | 4,851 |
| Gwembe | 58,354 | 28,098 | 30,256 | 5,070 | 4,997 | 4,268 | 4,343 | 3,697 | 3,846 | 11,472 | 12,629 | 930 | 1,040 | 708 | 820 | 533 | 578 | 431 | 618 | 989 | 1,385 |
| Itezhi-tezhi | 41,651 | 20,484 | 21,167 | 5,491 | 5,452 | 4,083 | 4,103 | 3,156 | 3,165 | 6,704 | 7,163 | 275 | 281 | 173 | 226 | 124 | 172 | 132 | 166 | 346 | 439 |
| Kalomo | 245,921 | 119,919 | 126,002 | 25,780 | 26,414 | 20,945 | 20,990 | 17,660 | 17,810 | 46,810 | 50,203 | 2,599 | 2,882 | 2,022 | 2,330 | 1,162 | 1,259 | 961 | 1,389 | 1,980 | 2,725 |
| Kazungula | 82,970 | 40,792 | 42,178 | 8,857 | 8,869 | 7,389 | 7,442 | 6,212 | 5,997 | 14,853 | 15,568 | 957 | 989 | 676 | 856 | 479 | 536 | 370 | 594 | 999 | 1,327 |
| Livingstone | 111,662 | 53,618 | 58,044 | 9,451 | 9,559 | 7,210 | 7,092 | 6,148 | 6,801 | 27,224 | 30,302 | 1,347 | 1,445 | 880 | 1,064 | 515 | 621 | 309 | 437 | 534 | 723 |
| Mazabuka | 232,757 | 113,913 | 118,844 | 18,588 | 18,653 | 16,156 | 16,065 | 15,958 | 16,112 | 51,963 | 54,719 | 3,126 | 3,591 | 2,333 | 2,782 | 1,580 | 1,661 | 1,286 | 1,636 | 2,923 | 3,625 |
| Monze | 248,084 | 121,178 | 126,906 | 18,970 | 18,795 | 16,314 | 16,346 | 14,920 | 15,036 | 56,632 | 60,009 | 4,323 | 4,319 | 2,960 | 3,465 | 2,133 | 2,340 | 1,631 | 1,994 | 3,295 | 4,602 |
| Namwala | 96,186 | 46,580 | 49,606 | 9,706 | 9,868 | 7,936 | 8,090 | 6,712 | 6,863 | 18,783 | 20,595 | 1,019 | 1,162 | 745 | 906 | 464 | 551 | 382 | 476 | 833 | 1,095 |
| Siavonga | 84,303 | 41,223 | 43,080 | 7,984 | 8,043 | 6,646 | 6,544 | 5,662 | 5,858 | 17,154 | 18,377 | 1,217 | 1,360 | 823 | 870 | 536 | 537 | 416 | 427 | 785 | 1,064 |
| Sinazongwe | 104,590 | 50,236 | 54,354 | 8,894 | 9,107 | 7,889 | 8,183 | 7,104 | 7,298 | 21,804 | 24,070 | 1,440 | 1,622 | 1,006 | 1,170 | 646 | 740 | 545 | 648 | 908 | 1,516 |
| | | | | | | | | | | | | | | | | | | | | | |
| **Western** | **896,546** | **431,254** | **465,292** | **79,623** | **79,624** | **65,050** | **64,687** | **55,909** | **56,602** | **172,739** | **196,382** | **13,518** | **15,508** | **11,410** | **13,688** | **8,132** | **9,340** | **6,969** | **9,167** | **17,904** | **20,294** |
| Kalabo | 152,188 | 71,738 | 80,450 | 12,093 | 12,290 | 9,857 | 10,080 | 8,712 | 8,907 | 28,575 | 34,211 | 2,640 | 3,294 | 2,534 | 3,162 | 1,732 | 1,924 | 1,582 | 2,084 | 4,013 | 4,498 |
| Kaoma | 148,510 | 72,598 | 75,912 | 15,657 | 15,413 | 12,318 | 12,302 | 10,378 | 10,618 | 27,202 | 30,453 | 1,708 | 1,753 | 1,401 | 1,539 | 915 | 969 | 836 | 994 | 2,183 | 1,871 |
| Lukulu | 83,889 | 40,695 | 43,194 | 8,193 | 8,164 | 6,636 | 6,336 | 5,547 | 5,590 | 15,533 | 17,647 | 1,248 | 1,407 | 929 | 1,128 | 653 | 663 | 540 | 739 | 1,416 | 1,520 |
| Mongu | 205,055 | 99,057 | 105,998 | 15,839 | 15,898 | 13,270 | 13,406 | 12,297 | 12,386 | 43,600 | 47,510 | 3,245 | 3,635 | 2,698 | 3,278 | 2,082 | 2,446 | 1,667 | 2,193 | 4,359 | 5,246 |
| Senanga | 132,407 | 63,537 | 68,870 | 10,976 | 11,017 | 9,274 | 9,074 | 7,928 | 8,045 | 25,608 | 29,197 | 2,290 | 2,567 | 1,927 | 2,319 | 1,413 | 1,693 | 1,190 | 1,571 | 2,931 | 3,387 |
| Sesheke | 94,069 | 45,546 | 48,523 | 8,339 | 8,430 | 6,929 | 6,774 | 5,907 | 5,868 | 18,379 | 20,115 | 1,401 | 1,611 | 1,120 | 1,326 | 851 | 1,050 | 727 | 977 | 1,893 | 2,372 |
| Shang'ombo | 80,428 | 38,083 | 42,345 | 8,526 | 8,412 | 6,766 | 6,715 | 5,140 | 5,188 | 13,842 | 17,249 | 986 | 1,241 | 801 | 936 | 486 | 595 | 427 | 609 | 1,109 | 1,400 |

## Table G2: Population (De facto) by Rural Part of District of Birth, Age, Sex and District of Residence, Zambia 2010

| Rural/Urban Part of District of Birth | Age and Sex | | | | | | | | | | | | | | | | | | | | |
|---|---|---|---|---|---|---|---|---|---|---|---|---|---|---|---|---|---|---|---|---|---|
| | Total | | | 0-4 | | 5-9 | | 10-14 | | 15-44 | | 45-49 | | 50-54 | | 55-59 | | 60-64 | | 65+ | |
| | Total | Male | Female | Male | Female | Male | Female | Male | Female | Male | Female | Male | Female | Male | Female | Male | Female | Male | Female | Male | Female |
| **Zambia Rural** | | | | | | | | | | | | | | | | | | | | | |
| **Total** | **7,879,472** | **3,857,247** | **4,022,225** | **714,867** | **721,282** | **606,667** | **606,098** | **529,953** | **526,074** | **1,535,499** | **1,644,737** | **121,185** | **127,098** | **93,785** | **103,452** | **66,834** | **71,481** | **57,545** | **71,754** | **130,912** | **150,249** |
| | | | | | | | | | | | | | | | | | | | | | |
| **Central** | **823,403** | **405,705** | **417,698** | **83,974** | **84,977** | **70,210** | **69,562** | **61,113** | **60,990** | **372,450** | **596,400** | **9,542** | **9,861** | **7,060** | **7,690** | **4,773** | **5,164** | **4,231** | **5,188** | **9,698** | **10,747** |
| Chibombo | 207,665 | 103,303 | 104,362 | 23,971 | 24,031 | 19,133 | 18,952 | 16,857 | 16,474 | 37,124 | 37,934 | 1,838 | 1,877 | 1,210 | 1,374 | 797 | 886 | 719 | 867 | 1,654 | 1,967 |
| Kabwe | 39,947 | 19,069 | 20,878 | 1,705 | 1,622 | 1,839 | 1,908 | 2,143 | 2,454 | 10,523 | 11,669 | 774 | 836 | 626 | 712 | 428 | 458 | 350 | 440 | 681 | 779 |
| Kapiri-Mposhi | 133,620 | 65,924 | 67,696 | 15,602 | 15,929 | 12,921 | 13,012 | 11,333 | 11,007 | 22,310 | 23,513 | 1,052 | 1,060 | 743 | 842 | 488 | 600 | 421 | 572 | 1,054 | 1,161 |
| Mkushi | 116,504 | 57,225 | 59,279 | 11,745 | 12,057 | 9,602 | 9,463 | 8,110 | 8,392 | 21,639 | 23,117 | 1,583 | 1,550 | 1,263 | 1,222 | 797 | 810 | 755 | 882 | 1,731 | 1,786 |
| Mumbwa | 164,282 | 80,880 | 83,402 | 16,507 | 16,580 | 14,025 | 13,676 | 11,808 | 11,925 | 32,154 | 33,743 | 1,862 | 1,948 | 1,272 | 1,483 | 878 | 1,025 | 645 | 915 | 1,729 | 2,107 |
| Serenje | 161,385 | 79,304 | 82,081 | 14,444 | 14,758 | 12,690 | 12,551 | 10,862 | 10,738 | 31,354 | 33,543 | 2,433 | 2,590 | 1,946 | 2,057 | 1,385 | 1,385 | 1,341 | 1,512 | 2,849 | 2,947 |
| | | | | | | | | | | | | | | | | | | | | | |
| **Copper-belt** | **332,285** | **163,139** | **169,146** | **29,279** | **29,327** | **24,789** | **24,729** | **22,622** | **22,424** | **69,338** | **73,826** | **4,973** | **5,240** | **3,697** | **4,101** | **2,530** | **2,807** | **2,026** | **2,439** | **3,885** | **4,253** |
| Chilila-bombwe | 9,132 | 4,512 | 4,620 | 647 | 629 | 551 | 530 | 573 | 526 | 2,242 | 2,424 | 191 | 219 | 140 | 125 | 57 | 61 | 48 | 47 | 63 | 59 |
| Chingola | 18,924 | 9,290 | 9,634 | 1,459 | 1,443 | 1,173 | 1,213 | 1,128 | 1,125 | 4,540 | 4,786 | 337 | 351 | 251 | 299 | 157 | 180 | 108 | 111 | 137 | 126 |
| Kalulushi | 13,845 | 6,931 | 6,914 | 1,311 | 1,238 | 1,077 | 1,018 | 997 | 928 | 2,947 | 3,161 | 196 | 204 | 136 | 127 | 94 | 76 | 58 | 61 | 115 | 101 |
| Kitwe | 22,617 | 11,021 | 11,596 | 1,039 | 1,113 | 933 | 912 | 1,045 | 1,081 | 6,393 | 6,797 | 515 | 500 | 373 | 391 | 245 | 284 | 175 | 205 | 303 | 313 |
| Luanshya | 21,645 | 10,546 | 11,099 | 1,356 | 1,334 | 1,169 | 1,155 | 1,221 | 1,206 | 5,195 | 5,583 | 494 | 498 | 340 | 460 | 252 | 278 | 200 | 247 | 319 | 338 |
| Lufwan-yama | 55,093 | 26,812 | 28,281 | 6,080 | 6,126 | 4,975 | 5,080 | 4,192 | 4,284 | 9,315 | 10,297 | 572 | 626 | 459 | 502 | 352 | 356 | 264 | 337 | 603 | 673 |
| Masaiti | 77,845 | 38,570 | 39,275 | 7,471 | 7,669 | 6,743 | 6,541 | 6,059 | 5,829 | 14,731 | 15,226 | 877 | 1,009 | 685 | 768 | 503 | 584 | 483 | 548 | 1,018 | 1,101 |
| Mpongwe | 67,577 | 33,334 | 34,243 | 7,876 | 7,769 | 6,214 | 6,313 | 5,245 | 5,149 | 11,740 | 12,373 | 657 | 664 | 455 | 499 | 278 | 368 | 241 | 354 | 628 | 754 |
| Mufulira | 17,076 | 8,083 | 8,993 | 766 | 790 | 739 | 727 | 751 | 802 | 4,361 | 5,050 | 485 | 530 | 355 | 380 | 244 | 267 | 163 | 210 | 219 | 237 |
| Ndola | 28,531 | 14,040 | 14,491 | 1,274 | 1,216 | 1,215 | 1,240 | 1,411 | 1,494 | 7,874 | 8,129 | 649 | 639 | 503 | 550 | 348 | 353 | 286 | 319 | 480 | 551 |
| | | | | | | | | | | | | | | | | | | | | | |
| **Eastern** | **1,507,052** | **740,512** | **766,540** | **130,943** | **133,005** | **114,119** | **114,302** | **100,843** | **99,184** | **296,898** | **307,937** | **24,685** | **25,341** | **18,923** | **20,855** | **14,067** | **14,912** | **12,342** | **16,085** | **27,692** | **34,919** |
| Chadiza | 103,993 | 51,405 | 52,588 | 9,383 | 9,593 | 8,208 | 8,220 | 7,034 | 7,156 | 20,652 | 20,872 | 1,612 | 1,611 | 1,169 | 1,241 | 905 | 909 | 736 | 933 | 1,706 | 2,053 |
| Chama | 96,908 | 47,333 | 49,575 | 9,227 | 9,220 | 8,142 | 8,122 | 6,703 | 6,483 | 17,846 | 19,541 | 1,402 | 1,540 | 988 | 1,175 | 732 | 785 | 714 | 925 | 1,579 | 1,784 |
| Chipata | 377,222 | 186,830 | 190,392 | 29,061 | 29,694 | 25,542 | 25,477 | 23,589 | 23,621 | 80,237 | 79,465 | 7,042 | 7,006 | 5,530 | 5,872 | 4,080 | 4,227 | 3,562 | 4,399 | 8,187 | 10,631 |
| Katete | 229,286 | 112,399 | 116,887 | 20,174 | 20,608 | 17,565 | 17,710 | 15,924 | 15,326 | 45,212 | 47,359 | 3,756 | 3,805 | 2,577 | 2,841 | 2,040 | 2,161 | 1,694 | 2,486 | 3,457 | 4,591 |
| Lundazi | 303,713 | 149,156 | 154,557 | 26,954 | 27,490 | 23,499 | 23,715 | 20,451 | 20,305 | 57,832 | 61,273 | 4,956 | 5,065 | 3,957 | 4,269 | 3,030 | 3,001 | 2,655 | 3,259 | 5,822 | 6,180 |
| Mambwe | 50,729 | 24,754 | 25,975 | 5,223 | 5,275 | 4,511 | 4,528 | 3,805 | 3,692 | 8,903 | 9,656 | 560 | 608 | 443 | 477 | 314 | 338 | 262 | 445 | 733 | 956 |
| Nyimba | 72,412 | 35,516 | 36,896 | 6,810 | 6,753 | 5,923 | 5,863 | 5,214 | 4,973 | 13,790 | 14,659 | 1,003 | 1,076 | 783 | 887 | 454 | 563 | 477 | 615 | 1,062 | 1,507 |
| Petauke | 272,789 | 133,119 | 139,670 | 24,111 | 24,372 | 20,729 | 20,667 | 18,123 | 17,628 | 52,426 | 55,112 | 4,354 | 4,630 | 3,476 | 4,093 | 2,512 | 2,928 | 2,242 | 3,023 | 5,146 | 7,217 |

## Table G2: Population (De facto) by Rural Part of District of Birth, Age, Sex and District of Residence, Zambia 2010

| Rural/Urban Part of District of Birth | Age and Sex | | | | | | | | | | | | | | | | | | | | |
|---|---|---|---|---|---|---|---|---|---|---|---|---|---|---|---|---|---|---|---|---|---|
| | Total | | | 0-4 | | 5-9 | | 10-14 | | 15-44 | | 45-49 | | 50-54 | | 55-59 | | 60-64 | | 65+ | |
| | Total | Male | Female | Male | Female | Male | Female | Male | Female | Male | Female | Male | Female | Male | Female | Male | Female | Male | Female | Male | Female |
| **Luapula** | **860,494** | **420,190** | **440,304** | **77,320** | **78,375** | **66,385** | **66,158** | **57,564** | **57,223** | **163,186** | **180,450** | **14,447** | **14,626** | **11,990** | **12,205** | **8,211** | **8,711** | **7,241** | **8,199** | **13,846** | **14,357** |
| Chienge | 107,377 | 52,390 | 54,987 | 11,346 | 11,562 | 8,565 | 8,563 | 6,968 | 7,055 | 19,944 | 22,291 | 1,606 | 1,547 | 1,332 | 1,338 | 863 | 861 | 709 | 685 | 1,057 | 1,085 |
| Kawambwa | 126,471 | 61,864 | 64,607 | 10,072 | 10,372 | 9,253 | 9,076 | 8,504 | 8,247 | 24,467 | 26,929 | 2,187 | 2,270 | 1,922 | 1,950 | 1,360 | 1,462 | 1,271 | 1,435 | 2,828 | 2,866 |
| Mansa | 165,619 | 81,660 | 83,959 | 13,614 | 13,813 | 11,945 | 11,920 | 10,531 | 10,486 | 33,470 | 35,369 | 3,056 | 3,003 | 2,619 | 2,663 | 1,843 | 1,918 | 1,658 | 1,774 | 2,924 | 3,013 |
| Milenge | 37,301 | 17,907 | 19,394 | 3,942 | 4,088 | 3,372 | 3,380 | 2,772 | 2,834 | 6,185 | 7,185 | 437 | 501 | 389 | 382 | 209 | 262 | 199 | 292 | 402 | 470 |
| Mwense | 123,288 | 60,305 | 62,983 | 10,264 | 10,319 | 9,259 | 9,217 | 8,137 | 8,185 | 23,506 | 26,004 | 2,432 | 2,443 | 2,043 | 1,962 | 1,362 | 1,385 | 1,153 | 1,360 | 2,149 | 2,108 |
| Nchelenge | 106,532 | 52,428 | 54,104 | 10,258 | 10,442 | 8,745 | 8,784 | 7,969 | 7,820 | 20,468 | 22,032 | 1,504 | 1,445 | 1,096 | 1,057 | 750 | 838 | 580 | 632 | 1,058 | 1,054 |
| Samfya | 193,906 | 93,636 | 100,270 | 17,824 | 17,779 | 15,246 | 15,218 | 12,683 | 12,596 | 35,146 | 40,640 | 3,225 | 3,417 | 2,589 | 2,853 | 1,824 | 1,985 | 1,671 | 2,021 | 3,428 | 3,761 |
| | | | | | | | | | | | | | | | | | | | | | |
| **Lusaka** | **240,253** | **118,175** | **122,078** | **23,677** | **23,848** | **19,306** | **19,032** | **17,071** | **17,159** | **47,793** | **49,677** | **2,899** | **3,129** | **2,131** | **2,584** | **1,394** | **1,495** | **1,186** | **1,504** | **2,718** | **3,650** |
| Chongwe | 108,640 | 53,745 | 54,895 | 11,763 | 11,964 | 9,434 | 9,167 | 8,110 | 8,192 | 20,210 | 20,634 | 1,194 | 1,229 | 879 | 1,081 | 543 | 573 | 478 | 623 | 1,134 | 1,432 |
| Kafue | 57,716 | 28,678 | 29,038 | 6,915 | 6,884 | 5,343 | 5,263 | 4,513 | 4,425 | 10,357 | 10,639 | 441 | 521 | 351 | 389 | 211 | 207 | 160 | 200 | 387 | 510 |
| Luangwa | 25,236 | 12,021 | 13,215 | 1,752 | 1,803 | 1,717 | 1,731 | 1,619 | 1,608 | 4,829 | 5,312 | 468 | 595 | 378 | 466 | 299 | 317 | 285 | 365 | 674 | 1,018 |
| Lusaka | 48,661 | 23,731 | 24,930 | 3,247 | 3,197 | 2,812 | 2,871 | 2,829 | 2,934 | 12,397 | 13,092 | 796 | 784 | 523 | 648 | 341 | 398 | 263 | 316 | 523 | 690 |
| | | | | | | | | | | | | | | | | | | | | | |
| **Northern** | **1,520,205** | **746,204** | **774,001** | **135,808** | **136,837** | **116,508** | **116,991** | **100,207** | **99,222** | **291,995** | **313,811** | **25,445** | **25,978** | **19,527** | **20,655** | **14,338** | **14,789** | **12,749** | **15,081** | **29,627** | **30,637** |
| Chilubi | 81,647 | 38,851 | 42,796 | 8,273 | 8,224 | 6,789 | 6,796 | 5,361 | 5,304 | 13,968 | 17,044 | 1,163 | 1,427 | 894 | 1,002 | 623 | 760 | 547 | 789 | 1,233 | 1,450 |
| Chinsali | 156,747 | 76,744 | 80,003 | 12,879 | 13,194 | 11,839 | 11,831 | 10,452 | 10,269 | 30,097 | 32,095 | 2,814 | 2,884 | 2,102 | 2,225 | 1,571 | 1,708 | 1,386 | 1,833 | 3,604 | 3,964 |
| Isoka | 142,354 | 70,276 | 72,078 | 11,314 | 11,486 | 10,246 | 10,381 | 8,894 | 8,802 | 27,963 | 29,427 | 2,706 | 2,804 | 2,319 | 2,432 | 1,797 | 1,631 | 1,595 | 1,746 | 3,442 | 3,369 |
| Kaputa | 103,564 | 50,467 | 53,097 | 10,692 | 10,793 | 8,197 | 8,383 | 6,941 | 6,840 | 19,257 | 21,945 | 1,450 | 1,378 | 1,191 | 1,205 | 908 | 866 | 661 | 692 | 1,170 | 995 |
| Kasama | 163,881 | 81,587 | 82,294 | 12,046 | 11,994 | 10,663 | 10,927 | 9,828 | 10,043 | 36,017 | 35,647 | 3,259 | 3,201 | 2,445 | 2,450 | 1,761 | 1,932 | 1,640 | 1,918 | 3,928 | 4,182 |
| Luwingu | 119,504 | 58,382 | 61,122 | 10,559 | 10,579 | 9,167 | 9,114 | 8,065 | 7,765 | 21,926 | 24,402 | 2,136 | 2,197 | 1,696 | 1,768 | 1,180 | 1,270 | 1,108 | 1,287 | 2,545 | 2,740 |
| Mbala | 195,979 | 97,049 | 98,930 | 16,995 | 17,043 | 14,651 | 14,550 | 12,695 | 12,601 | 38,862 | 40,901 | 3,646 | 3,487 | 2,618 | 2,933 | 2,074 | 1,979 | 1,713 | 1,873 | 3,795 | 3,563 |
| Mpika | 166,834 | 81,337 | 85,497 | 14,869 | 15,003 | 13,273 | 13,246 | 11,341 | 11,440 | 32,018 | 34,999 | 2,459 | 2,539 | 1,905 | 1,980 | 1,287 | 1,432 | 1,247 | 1,578 | 2,938 | 3,280 |
| Mporokoso | 103,329 | 51,049 | 52,280 | 8,472 | 8,466 | 7,672 | 7,730 | 6,852 | 6,771 | 19,869 | 20,817 | 1,807 | 1,938 | 1,465 | 1,501 | 1,120 | 1,188 | 1,060 | 1,284 | 2,732 | 2,585 |
| Mpulungu | 67,822 | 33,157 | 34,665 | 7,369 | 7,370 | 5,571 | 5,677 | 4,624 | 4,494 | 12,490 | 13,892 | 944 | 930 | 643 | 673 | 436 | 422 | 308 | 404 | 772 | 803 |
| Mungwi | 125,207 | 60,968 | 64,239 | 13,814 | 13,879 | 10,944 | 10,849 | 8,967 | 8,707 | 20,867 | 23,618 | 1,612 | 1,799 | 1,202 | 1,379 | 783 | 874 | 771 | 925 | 2,008 | 2,209 |
| Nakonde | 93,337 | 46,337 | 47,000 | 8,526 | 8,806 | 7,496 | 7,507 | 6,187 | 6,186 | 18,661 | 19,024 | 1,449 | 1,394 | 1,047 | 1,107 | 798 | 727 | 713 | 752 | 1,460 | 1,497 |
| | | | | | | | | | | | | | | | | | | | | | |
| **North Western** | **577,663** | **283,910** | **293,753** | **53,439** | **53,708** | **44,946** | **44,985** | **39,170** | **38,797** | **109,461** | **117,821** | **9,259** | **9,302** | **7,103** | **7,375** | **5,217** | **5,278** | **4,275** | **5,143** | **11,040** | **11,344** |
| Chavuma | 35,804 | 17,248 | 18,556 | 3,387 | 3,365 | 2,715 | 2,767 | 2,298 | 2,337 | 6,748 | 7,570 | 534 | 542 | 335 | 436 | 245 | 287 | 219 | 326 | 767 | 926 |
| Kabompo | 74,731 | 36,688 | 38,043 | 6,515 | 6,495 | 5,775 | 5,726 | 5,119 | 5,169 | 14,355 | 15,527 | 1,150 | 1,170 | 878 | 981 | 734 | 720 | 548 | 675 | 1,614 | 1,580 |
| Kasempa | 69,088 | 33,613 | 35,475 | 6,220 | 6,278 | 5,392 | 5,296 | 4,796 | 4,530 | 12,842 | 14,661 | 1,076 | 1,147 | 833 | 946 | 614 | 657 | 533 | 647 | 1,307 | 1,313 |
| Mufumbwe | 37,630 | 18,471 | 19,159 | 4,898 | 4,933 | 3,766 | 3,764 | 2,870 | 2,940 | 5,677 | 6,230 | 353 | 351 | 220 | 252 | 178 | 178 | 155 | 158 | 354 | 353 |
| Mwinilunga | 141,247 | 69,885 | 71,362 | 12,138 | 12,018 | 10,311 | 10,342 | 9,523 | 9,261 | 27,960 | 29,493 | 2,557 | 2,577 | 2,081 | 2,004 | 1,404 | 1,427 | 1,213 | 1,411 | 2,698 | 2,829 |
| Solwezi | 133,274 | 65,750 | 67,524 | 13,228 | 13,530 | 10,917 | 11,043 | 9,187 | 9,172 | 25,217 | 26,472 | 1,980 | 1,907 | 1,476 | 1,476 | 1,097 | 1,041 | 859 | 971 | 1,789 | 1,912 |
| Zambezi | 85,889 | 42,255 | 43,634 | 7,053 | 7,089 | 6,070 | 6,047 | 5,377 | 5,388 | 16,662 | 17,868 | 1,609 | 1,608 | 1,280 | 1,280 | 945 | 968 | 748 | 955 | 2,511 | 2,431 |

# Table G2: Population (De facto) by Rural Part of District of Birth, Age, Sex and District of Residence, Zambia 2010

| Rural/Urban Part of District of Birth | Age and Sex | | | | | | | | | | | | | | | | | | | | |
|---|---|---|---|---|---|---|---|---|---|---|---|---|---|---|---|---|---|---|---|---|---|
| | Total | | | 0-4 | | 5-9 | | 10-14 | | 15-44 | | 45-49 | | 50-54 | | 55-59 | | 60-64 | | 65+ | |
| | Total | Male | Female | Male | Female | Male | Female | Male | Female | Male | Female | Male | Female | Male | Female | Male | Female | Male | Female | Male | Female |
| **Southern** | **1,264,923** | **617,684** | **647,239** | **113,316** | **114,231** | **95,652** | **95,974** | **84,697** | **84,303** | **260,005** | **275,718** | **18,316** | **19,953** | **13,365** | **15,825** | **9,137** | **9,991** | **7,286** | **9,644** | **15,910** | **21,600** |
| Choma | 250,418 | 122,186 | 128,232 | 19,786 | 20,035 | 17,097 | 17,397 | 15,892 | 15,810 | 54,856 | 57,970 | 4,153 | 4,445 | 3,021 | 3,562 | 2,197 | 2,325 | 1,630 | 2,144 | 3,554 | 4,544 |
| Gwembe | 55,169 | 26,604 | 28,565 | 4,866 | 4,768 | 4,088 | 4,134 | 3,500 | 3,632 | 10,718 | 11,741 | 892 | 996 | 668 | 780 | 497 | 550 | 412 | 601 | 963 | 1,363 |
| Itezhi-tezhi | 31,532 | 15,565 | 15,967 | 4,184 | 4,103 | 3,080 | 3,012 | 2,333 | 2,310 | 5,008 | 5,356 | 252 | 249 | 152 | 209 | 110 | 161 | 119 | 154 | 327 | 413 |
| Kalomo | 222,515 | 108,598 | 113,917 | 23,637 | 24,234 | 19,181 | 19,259 | 16,137 | 16,074 | 41,642 | 44,525 | 2,363 | 2,626 | 1,844 | 2,159 | 1,060 | 1,146 | 880 | 1,302 | 1,854 | 2,592 |
| Kazungula | 79,995 | 39,367 | 40,628 | 8,683 | 8,690 | 7,252 | 7,276 | 6,063 | 5,829 | 14,026 | 14,682 | 908 | 946 | 643 | 807 | 454 | 518 | 360 | 581 | 978 | 1,299 |
| Livingstone | 11,249 | 5,396 | 5,853 | 631 | 662 | 565 | 578 | 498 | 578 | 2,922 | 3,097 | 240 | 235 | 179 | 188 | 115 | 130 | 82 | 133 | 164 | 252 |
| Mazabuka | 166,120 | 81,550 | 84,570 | 13,088 | 13,090 | 11,308 | 11,173 | 10,993 | 10,908 | 36,611 | 37,966 | 2,546 | 2,936 | 1,932 | 2,329 | 1,346 | 1,397 | 1,103 | 1,471 | 2,623 | 3,300 |
| Monze | 196,281 | 96,371 | 99,910 | 15,097 | 15,026 | 13,159 | 13,016 | 11,899 | 11,690 | 43,635 | 45,277 | 3,657 | 3,700 | 2,586 | 3,035 | 1,843 | 2,053 | 1,457 | 1,798 | 3,038 | 4,315 |
| Namwala | 87,945 | 42,762 | 45,183 | 9,203 | 9,298 | 7,430 | 7,578 | 6,255 | 6,293 | 16,746 | 18,193 | 912 | 1,027 | 666 | 831 | 422 | 495 | 342 | 434 | 786 | 1,034 |
| Siavonga | 68,378 | 33,533 | 34,845 | 5,992 | 5,970 | 5,265 | 5,074 | 4,681 | 4,658 | 14,213 | 15,197 | 1,061 | 1,244 | 724 | 803 | 484 | 502 | 380 | 394 | 733 | 1,003 |
| Sinazongwe | 95,321 | 45,752 | 49,569 | 8,149 | 8,355 | 7,227 | 7,477 | 6,446 | 6,521 | 19,628 | 21,714 | 1,332 | 1,549 | 950 | 1,122 | 609 | 714 | 521 | 632 | 890 | 1,485 |
| | | | | | | | | | | | | | | | | | | | | | |
| **Western** | **753,194** | **361,728** | **391,466** | **67,111** | **66,974** | **54,752** | **54,365** | **46,666** | **46,772** | **141,719** | **161,978** | **11,619** | **13,668** | **9,989** | **12,162** | **7,167** | **8,334** | **6,209** | **8,471** | **16,496** | **18,742** |
| Kalabo | 138,989 | 65,260 | 73,729 | 11,081 | 11,241 | 8,954 | 9,264 | 7,999 | 8,125 | 25,692 | 31,013 | 2,389 | 3,061 | 2,306 | 2,961 | 1,573 | 1,781 | 1,475 | 1,976 | 3,791 | 4,307 |
| Kaoma | 123,578 | 60,443 | 63,135 | 13,254 | 12,984 | 10,285 | 10,203 | 8,525 | 8,665 | 22,124 | 24,803 | 1,457 | 1,556 | 1,240 | 1,375 | 815 | 884 | 746 | 934 | 1,997 | 1,731 |
| Lukulu | 75,465 | 36,560 | 38,905 | 7,123 | 7,074 | 5,828 | 5,570 | 4,981 | 4,977 | 14,126 | 16,109 | 1,160 | 1,330 | 865 | 1,057 | 615 | 629 | 501 | 714 | 1,361 | 1,445 |
| Mongu | 142,483 | 68,929 | 73,554 | 10,943 | 10,971 | 9,221 | 9,223 | 8,333 | 8,106 | 29,045 | 31,429 | 2,439 | 2,795 | 2,111 | 2,601 | 1,668 | 1,994 | 1,357 | 1,873 | 3,812 | 4,562 |
| Senanga | 112,278 | 53,753 | 58,525 | 9,371 | 9,399 | 7,923 | 7,728 | 6,661 | 6,756 | 21,232 | 24,344 | 1,953 | 2,243 | 1,673 | 2,042 | 1,241 | 1,494 | 1,045 | 1,442 | 2,654 | 3,077 |
| Sesheke | 81,560 | 39,502 | 42,058 | 7,018 | 7,088 | 5,943 | 5,827 | 5,158 | 5,096 | 15,903 | 17,282 | 1,252 | 1,449 | 1,005 | 1,192 | 776 | 966 | 664 | 928 | 1,783 | 2,230 |
| Shang'ombo | 78,841 | 37,281 | 41,560 | 8,321 | 8,217 | 6,598 | 6,550 | 5,009 | 5,047 | 13,597 | 16,998 | 969 | 1,234 | 789 | 934 | 479 | 586 | 421 | 604 | 1,098 | 1,390 |

## Table G2: Population (De facto) by Urban Part of District of Birth, Age, Sex and District of Residence, Zambia 2010

| Rural/Urban Part of District of Birth | Age and Sex | | | | | | | | | | | | | | | | | | | | |
|---|---|---|---|---|---|---|---|---|---|---|---|---|---|---|---|---|---|---|---|---|---|
| | Total | | | 0-4 | | 5-9 | | 10-14 | | 15-44 | | 45-49 | | 50-54 | | 55-59 | | 60-64 | | 65+ | |
| | Total | Male | Female | Male | Female | Male | Female | Male | Female | Male | Female | Male | Female | Male | Female | Male | Female | Male | Female | Male | Female |
| **Zambia Urban** | | | | | | | | | | | | | | | | | | | | | |
| **Total** | **4,545,062** | **2,208,639** | **2,336,423** | **384,897** | **388,788** | **314,917** | **322,806** | **307,536** | **328,579** | **1,066,010** | **1,154,745** | **49,726** | **49,868** | **31,488** | **34,936** | **20,495** | **20,741** | **13,517** | **14,150** | **20,053** | **21,810** |
| | | | | | | | | | | | | | | | | | | | | | |
| **Central** | **325,187** | **155,828** | **169,359** | **25,646** | **25,915** | **21,822** | **22,829** | **22,276** | **24,388** | **534,686** | **292,336** | **3,476** | **3,628** | **2,274** | **2,762** | **1,559** | **1,607** | **1,113** | **1,191** | **1,728** | **1,867** |
| Chibombo | 8,464 | 4,070 | 4,394 | 514 | 552 | 506 | 567 | 547 | 616 | 2,157 | 2,311 | 116 | 118 | 81 | 69 | 55 | 54 | 33 | 40 | 61 | 67 |
| Kabwe | 212,638 | 101,749 | 110,889 | 14,795 | 15,037 | 13,007 | 13,423 | 14,013 | 15,064 | 53,003 | 59,571 | 2,452 | 2,618 | 1,575 | 2,040 | 1,059 | 1,148 | 761 | 795 | 1,084 | 1,193 |
| Kapiri-Mposhi | 34,939 | 16,871 | 18,068 | 4,329 | 4,297 | 3,420 | 3,471 | 2,983 | 3,299 | 5,748 | 6,605 | 135 | 147 | 76 | 89 | 65 | 48 | 43 | 40 | 72 | 72 |
| Mkushi | 19,820 | 9,531 | 10,289 | 1,846 | 1,809 | 1,395 | 1,517 | 1,412 | 1,578 | 4,126 | 4,682 | 253 | 203 | 153 | 152 | 108 | 106 | 77 | 91 | 161 | 151 |
| Mumbwa | 28,468 | 13,527 | 14,941 | 2,608 | 2,642 | 2,142 | 2,311 | 1,943 | 2,256 | 6,142 | 6,911 | 240 | 274 | 164 | 173 | 93 | 126 | 65 | 84 | 130 | 164 |
| Serenje | 20,858 | 10,080 | 10,778 | 1,554 | 1,578 | 1,352 | 1,540 | 1,378 | 1,575 | 4,758 | 5,092 | 280 | 268 | 225 | 239 | 179 | 125 | 134 | 141 | 220 | 220 |
| | | | | | | | | | | | | | | | | | | | | | |
| **Copper-belt** | **1,621,457** | **786,886** | **834,571** | **114,076** | **115,058** | **97,369** | **99,008** | **106,497** | **111,147** | **416,150** | **452,836** | **21,226** | **22,126** | **13,002** | **15,034** | **8,166** | **8,403** | **4,772** | **5,276** | **5,628** | **5,683** |
| Chilila-bombwe | 75,640 | 37,232 | 38,408 | 5,621 | 5,574 | 4,636 | 4,686 | 5,227 | 5,352 | 19,749 | 20,766 | 1,062 | 1,076 | 520 | 537 | 222 | 201 | 100 | 98 | 95 | 118 |
| Chingola | 209,321 | 101,588 | 107,733 | 14,412 | 14,587 | 12,490 | 12,571 | 13,807 | 14,124 | 54,169 | 59,441 | 2,736 | 2,888 | 1,787 | 2,002 | 1,089 | 995 | 555 | 577 | 543 | 548 |
| Kalulushi | 58,617 | 28,547 | 30,070 | 5,059 | 5,126 | 4,087 | 4,173 | 4,049 | 4,243 | 14,016 | 15,118 | 621 | 640 | 370 | 396 | 170 | 192 | 77 | 76 | 98 | 106 |
| Kitwe | 486,661 | 236,323 | 250,338 | 36,779 | 37,496 | 30,471 | 31,134 | 31,445 | 33,390 | 123,127 | 133,069 | 5,989 | 6,068 | 3,476 | 4,048 | 2,209 | 2,307 | 1,312 | 1,433 | 1,515 | 1,393 |
| Luanshya | 163,751 | 78,918 | 84,833 | 9,140 | 9,161 | 8,036 | 8,146 | 10,091 | 10,506 | 43,608 | 48,095 | 2,787 | 2,986 | 1,922 | 2,361 | 1,362 | 1,497 | 865 | 1,004 | 1,107 | 1,077 |
| Lufwan-yama | 3,058 | 1,458 | 1,600 | 202 | 210 | 185 | 170 | 174 | 191 | 744 | 853 | 55 | 60 | 35 | 33 | 29 | 36 | 21 | 18 | 13 | 29 |
| Masaiti | 2,390 | 1,144 | 1,246 | 132 | 134 | 117 | 146 | 144 | 167 | 628 | 658 | 44 | 44 | 25 | 36 | 20 | 17 | 12 | 18 | 22 | 26 |
| Mpongwe | 3,777 | 1,775 | 2,002 | 237 | 244 | 268 | 275 | 227 | 292 | 877 | 1,029 | 47 | 62 | 45 | 39 | 29 | 22 | 20 | 18 | 25 | 21 |
| Mufulira | 180,026 | 86,999 | 93,027 | 10,807 | 10,825 | 9,382 | 9,328 | 10,952 | 11,033 | 47,483 | 52,468 | 3,144 | 3,464 | 2,108 | 2,540 | 1,426 | 1,473 | 823 | 1,011 | 874 | 885 |
| Ndola | 438,216 | 212,902 | 225,314 | 31,687 | 31,701 | 27,697 | 28,379 | 30,381 | 31,849 | 111,749 | 121,339 | 4,741 | 4,838 | 2,714 | 3,042 | 1,610 | 1,663 | 987 | 1,023 | 1,336 | 1,480 |
| | | | | | | | | | | | | | | | | | | | | | |
| **Eastern** | **239,737** | **117,084** | **122,653** | **19,937** | **20,154** | **16,695** | **17,327** | **15,464** | **17,284** | **53,334** | **55,877** | **3,493** | **3,360** | **2,564** | **2,598** | **1,799** | **1,746** | **1,377** | **1,421** | **2,421** | **2,886** |
| Chadiza | 4,680 | 2,300 | 2,380 | 313 | 313 | 268 | 277 | 271 | 300 | 1,109 | 1,233 | 105 | 77 | 69 | 58 | 61 | 42 | 44 | 26 | 60 | 54 |
| Chama | 9,026 | 4,570 | 4,456 | 839 | 831 | 637 | 664 | 547 | 577 | 2,034 | 1,891 | 162 | 127 | 96 | 116 | 80 | 67 | 51 | 66 | 124 | 117 |
| Chipata | 135,700 | 66,407 | 69,293 | 10,581 | 10,755 | 9,068 | 9,435 | 8,725 | 9,834 | 31,672 | 32,628 | 1,920 | 1,878 | 1,425 | 1,393 | 970 | 965 | 734 | 774 | 1,312 | 1,631 |
| Katete | 23,737 | 11,507 | 12,230 | 2,155 | 2,125 | 1,748 | 1,750 | 1,504 | 1,698 | 5,118 | 5,670 | 293 | 323 | 226 | 231 | 153 | 129 | 132 | 113 | 178 | 191 |
| Lundazi | 23,548 | 11,507 | 12,041 | 1,925 | 2,016 | 1,628 | 1,779 | 1,521 | 1,700 | 4,980 | 5,219 | 425 | 342 | 292 | 300 | 234 | 211 | 196 | 162 | 306 | 312 |
| Mambwe | 1,717 | 776 | 941 | 100 | 88 | 102 | 116 | 86 | 134 | 397 | 487 | 24 | 30 | 21 | 34 | 15 | 11 | 8 | 18 | 23 | 23 |
| Nyimba | 5,559 | 2,706 | 2,853 | 623 | 620 | 462 | 506 | 395 | 368 | 1,069 | 1,166 | 51 | 49 | 42 | 48 | 23 | 24 | 21 | 24 | 20 | 48 |
| Petauke | 35,770 | 17,311 | 18,459 | 3,401 | 3,406 | 2,782 | 2,800 | 2,415 | 2,673 | 6,955 | 7,583 | 513 | 534 | 393 | 418 | 263 | 297 | 191 | 238 | 398 | 510 |

## Table G2: Population (De facto) by Urban Part of District of Birth, Age, Sex and District of Residence, Zambia 2010

| Rural/Urban Part of District of Birth | Age and Sex | | | | | | | | | | | | | | | | | | | |
|---|---|---|---|---|---|---|---|---|---|---|---|---|---|---|---|---|---|---|---|---|
| | Total | | | 0-4 | | 5-9 | | 10-14 | | 15-44 | | 45-49 | | 50-54 | | 55-59 | | 60-64 | | 65+ | |
| | Total | Male | Female | Male | Female | Male | Female | Male | Female | Male | Female | Male | Female | Male | Female | Male | Female | Male | Female | Male | Female |
| **Luapula** | **155,192** | **76,145** | **79,047** | **13,509** | **13,749** | **11,295** | **11,826** | **10,355** | **11,276** | **33,568** | **34,720** | **2,174** | **2,185** | **1,682** | **1,675** | **1,192** | **1,120** | **917** | **914** | **1,453** | **1,582** |
| Chienge | 2,036 | 1,049 | 987 | 87 | 127 | 121 | 123 | 127 | 126 | 550 | 462 | 43 | 43 | 40 | 30 | 30 | 22 | 22 | 19 | 29 | 35 |
| Kawambwa | 25,141 | 12,243 | 12,898 | 2,142 | 2,215 | 1,851 | 1,973 | 1,648 | 1,735 | 5,186 | 5,380 | 375 | 382 | 275 | 355 | 227 | 219 | 200 | 213 | 339 | 426 |
| Mansa | 77,729 | 38,156 | 39,573 | 6,558 | 6,493 | 5,475 | 5,732 | 5,167 | 5,785 | 17,626 | 18,322 | 1,020 | 1,026 | 793 | 745 | 534 | 475 | 379 | 378 | 604 | 617 |
| Milenge | 1,433 | 726 | 707 | 79 | 80 | 78 | 61 | 98 | 82 | 384 | 400 | 29 | 25 | 15 | 19 | 16 | 11 | 9 | 11 | 18 | 18 |
| Mwense | 9,802 | 4,763 | 5,039 | 582 | 575 | 585 | 594 | 565 | 671 | 2,262 | 2,417 | 227 | 206 | 170 | 169 | 107 | 141 | 110 | 91 | 155 | 175 |
| Nchelenge | 24,064 | 11,813 | 12,251 | 3,070 | 3,243 | 2,309 | 2,407 | 1,886 | 1,835 | 3,932 | 4,191 | 205 | 190 | 140 | 106 | 113 | 92 | 61 | 64 | 97 | 123 |
| Samfya | 14,987 | 7,395 | 7,592 | 991 | 1,016 | 876 | 936 | 864 | 1,042 | 3,628 | 3,548 | 275 | 313 | 249 | 251 | 165 | 160 | 136 | 138 | 211 | 188 |
| | | | | | | | | | | | | | | | | | | | | | |
| **Lusaka** | **1,317,229** | **642,189** | **675,040** | **132,847** | **134,301** | **104,736** | **106,701** | **95,939** | **101,988** | **289,836** | **312,459** | **8,248** | **7,879** | **4,364** | **4,939** | **2,510** | **2,727** | **1,537** | **1,542** | **2,172** | **2,504** |
| Chongwe | 13,928 | 6,645 | 7,283 | 1,251 | 1,258 | 976 | 987 | 930 | 1,045 | 3,061 | 3,589 | 167 | 150 | 106 | 76 | 59 | 53 | 36 | 46 | 59 | 79 |
| Kafue | 60,961 | 29,616 | 31,345 | 6,076 | 6,134 | 4,659 | 4,739 | 4,694 | 4,834 | 13,502 | 14,867 | 277 | 301 | 155 | 183 | 105 | 111 | 54 | 74 | 94 | 102 |
| Luangwa | 4,792 | 2,304 | 2,488 | 323 | 341 | 267 | 288 | 305 | 287 | 1,070 | 1,227 | 86 | 78 | 83 | 81 | 44 | 59 | 46 | 47 | 80 | 80 |
| Lusaka | 1,237,548 | 603,624 | 633,924 | 125,197 | 126,568 | 98,834 | 100,687 | 90,010 | 95,822 | 272,203 | 292,776 | 7,718 | 7,350 | 4,020 | 4,599 | 2,302 | 2,504 | 1,401 | 1,375 | 1,939 | 2,243 |
| | | | | | | | | | | | | | | | | | | | | | |
| **Northern** | **274,158** | **134,191** | **139,967** | **23,947** | **24,228** | **19,793** | **20,894** | **17,758** | **19,495** | **59,565** | **62,195** | **3,923** | **3,763** | **2,770** | **2,785** | **2,049** | **1,952** | **1,585** | **1,580** | **2,801** | **3,075** |
| Chilubi | 1,788 | 889 | 899 | 75 | 91 | 87 | 101 | 77 | 99 | 503 | 458 | 40 | 47 | 28 | 38 | 35 | 24 | 21 | 16 | 23 | 25 |
| Chinsali | 16,526 | 7,916 | 8,610 | 910 | 999 | 910 | 1,014 | 931 | 1,056 | 3,968 | 4,239 | 325 | 325 | 253 | 267 | 185 | 218 | 160 | 184 | 274 | 308 |
| Isoka | 16,969 | 8,607 | 8,362 | 1,294 | 1,247 | 1,085 | 1,117 | 1,050 | 1,134 | 3,901 | 3,752 | 354 | 312 | 276 | 227 | 234 | 186 | 180 | 141 | 233 | 246 |
| Kaputa | 5,588 | 2,796 | 2,792 | 651 | 704 | 504 | 514 | 336 | 354 | 1,035 | 998 | 85 | 67 | 57 | 49 | 48 | 30 | 30 | 32 | 50 | 44 |
| Kasama | 101,939 | 49,783 | 52,156 | 8,570 | 8,718 | 7,550 | 7,888 | 6,906 | 7,532 | 22,716 | 23,690 | 1,296 | 1,290 | 827 | 918 | 620 | 588 | 440 | 501 | 858 | 1,031 |
| Luwingu | 9,879 | 4,756 | 5,123 | 614 | 646 | 570 | 615 | 540 | 647 | 2,256 | 2,409 | 198 | 224 | 138 | 186 | 128 | 123 | 126 | 86 | 186 | 187 |
| Mbala | 35,504 | 17,421 | 18,083 | 2,771 | 2,709 | 2,406 | 2,612 | 2,234 | 2,432 | 7,992 | 8,479 | 627 | 538 | 478 | 400 | 287 | 288 | 216 | 220 | 410 | 405 |
| Mpika | 35,143 | 17,129 | 18,014 | 3,299 | 3,287 | 2,525 | 2,610 | 2,336 | 2,671 | 7,645 | 8,122 | 386 | 387 | 279 | 246 | 199 | 201 | 168 | 143 | 292 | 347 |
| Mporokoso | 13,394 | 6,405 | 6,989 | 1,023 | 1,036 | 928 | 969 | 820 | 939 | 2,707 | 3,048 | 238 | 243 | 174 | 225 | 152 | 146 | 114 | 138 | 249 | 245 |
| Mpulungu | 15,052 | 7,364 | 7,688 | 1,962 | 1,985 | 1,380 | 1,527 | 1,189 | 1,167 | 2,486 | 2,700 | 119 | 104 | 76 | 67 | 53 | 35 | 39 | 33 | 60 | 70 |
| Mungwi | 3,307 | 1,610 | 1,697 | 367 | 354 | 335 | 346 | 223 | 266 | 585 | 581 | 30 | 44 | 20 | 23 | 16 | 22 | 12 | 23 | 22 | 38 |
| Nakonde | 19,069 | 9,515 | 9,554 | 2,411 | 2,452 | 1,513 | 1,581 | 1,116 | 1,198 | 3,771 | 3,719 | 225 | 182 | 164 | 139 | 92 | 91 | 79 | 63 | 144 | 129 |
| | | | | | | | | | | | | | | | | | | | | | |
| **North Western** | **114,442** | **56,387** | **58,055** | **12,180** | **12,266** | **8,681** | **8,936** | **7,764** | **8,600** | **23,125** | **24,188** | **1,493** | **1,252** | **1,008** | **893** | **717** | **559** | **471** | **452** | **948** | **909** |
| Chavuma | 1,666 | 800 | 866 | 92 | 72 | 75 | 89 | 65 | 118 | 433 | 443 | 37 | 49 | 25 | 25 | 17 | 20 | 14 | 12 | 42 | 38 |
| Kabompo | 16,769 | 8,181 | 8,588 | 1,813 | 1,757 | 1,357 | 1,397 | 1,277 | 1,320 | 3,147 | 3,466 | 201 | 183 | 115 | 134 | 72 | 88 | 52 | 74 | 147 | 169 |
| Kasempa | 7,493 | 3,594 | 3,899 | 641 | 676 | 507 | 499 | 394 | 536 | 1,658 | 1,856 | 138 | 125 | 90 | 72 | 65 | 35 | 30 | 43 | 71 | 57 |
| Mufumbwe | 2,503 | 1,223 | 1,280 | 199 | 169 | 184 | 187 | 171 | 221 | 579 | 626 | 35 | 25 | 19 | 23 | 13 | 10 | 9 | 6 | 14 | 13 |
| Mwinilunga | 17,919 | 8,989 | 8,930 | 1,534 | 1,558 | 1,287 | 1,366 | 1,189 | 1,361 | 3,965 | 3,784 | 318 | 231 | 218 | 175 | 171 | 140 | 114 | 99 | 193 | 216 |
| Solwezi | 59,284 | 29,265 | 30,019 | 7,375 | 7,494 | 4,794 | 4,900 | 4,204 | 4,484 | 11,311 | 11,851 | 549 | 449 | 378 | 304 | 241 | 163 | 157 | 145 | 256 | 229 |
| Zambezi | 8,808 | 4,335 | 4,473 | 526 | 540 | 477 | 498 | 464 | 560 | 2,032 | 2,162 | 215 | 190 | 163 | 160 | 138 | 103 | 95 | 73 | 225 | 187 |

## Table G2: Population (De facto) by Urban Part of District of Birth, Age, Sex and District of Residence, Zambia 2010

| Rural/Urban Part of District of Birth | Age and Sex | | | | | | | | | | | | | | | | | | | | |
|---|---|---|---|---|---|---|---|---|---|---|---|---|---|---|---|---|---|---|---|---|---|
| | Total | | | 0-4 | | 5-9 | | 10-14 | | 15-44 | | 45-49 | | 50-54 | | 55-59 | | 60-64 | | 65+ | |
| | Total | Male | Female | Male | Female | Male | Female | Male | Female | Male | Female | Male | Female | Male | Female | Male | Female | Male | Female | Male | Female |
| **Southern** | **354,308** | **170,403** | **183,905** | **30,243** | **30,467** | **24,228** | **24,963** | **22,240** | **24,571** | **83,478** | **92,894** | **3,794** | **3,835** | **2,403** | **2,724** | **1,538** | **1,621** | **985** | **1,078** | **1,494** | **1,752** |
| Choma | 62,335 | 29,860 | 32,475 | 4,982 | 4,906 | 3,947 | 4,342 | 3,816 | 4,278 | 15,228 | 17,007 | 724 | 652 | 421 | 498 | 306 | 292 | 178 | 193 | 258 | 307 |
| Gwembe | 3,185 | 1,494 | 1,691 | 204 | 229 | 180 | 209 | 197 | 214 | 754 | 888 | 38 | 44 | 40 | 40 | 36 | 28 | 19 | 17 | 26 | 22 |
| Itezhi-tezhi | 10,119 | 4,919 | 5,200 | 1,307 | 1,349 | 1,003 | 1,091 | 823 | 855 | 1,696 | 1,807 | 23 | 32 | 21 | 17 | 14 | 11 | 13 | 12 | 19 | 26 |
| Kalomo | 23,406 | 11,321 | 12,085 | 2,143 | 2,180 | 1,764 | 1,731 | 1,523 | 1,736 | 5,168 | 5,678 | 236 | 256 | 178 | 171 | 102 | 113 | 81 | 87 | 126 | 133 |
| Kazungula | 2,975 | 1,425 | 1,550 | 174 | 179 | 137 | 166 | 149 | 168 | 827 | 886 | 49 | 43 | 33 | 49 | 25 | 18 | 10 | 13 | 21 | 28 |
| Livingstone | 100,413 | 48,222 | 52,191 | 8,820 | 8,897 | 6,645 | 6,514 | 5,650 | 6,223 | 24,302 | 27,205 | 1,107 | 1,210 | 701 | 876 | 400 | 491 | 227 | 304 | 370 | 471 |
| Mazabuka | 66,637 | 32,363 | 34,274 | 5,500 | 5,563 | 4,848 | 4,892 | 4,965 | 5,204 | 15,352 | 16,753 | 580 | 655 | 401 | 453 | 234 | 264 | 183 | 165 | 300 | 325 |
| Monze | 51,803 | 24,807 | 26,996 | 3,873 | 3,769 | 3,155 | 3,330 | 3,021 | 3,346 | 12,997 | 14,732 | 666 | 619 | 374 | 430 | 290 | 287 | 174 | 196 | 257 | 287 |
| Namwala | 8,241 | 3,818 | 4,423 | 503 | 570 | 506 | 512 | 457 | 570 | 2,037 | 2,402 | 107 | 135 | 79 | 75 | 42 | 56 | 40 | 42 | 47 | 61 |
| Siavonga | 15,925 | 7,690 | 8,235 | 1,992 | 2,073 | 1,381 | 1,470 | 981 | 1,200 | 2,941 | 3,180 | 156 | 116 | 99 | 67 | 52 | 35 | 36 | 33 | 52 | 61 |
| Sinazongwe | 9,269 | 4,484 | 4,785 | 745 | 752 | 662 | 706 | 658 | 777 | 2,176 | 2,356 | 108 | 73 | 56 | 48 | 37 | 26 | 24 | 16 | 18 | 31 |
| | | | | | | | | | | | | | | | | | | | | | |
| **Western** | **143,352** | **69,526** | **73,826** | **12,512** | **12,650** | **10,298** | **10,322** | **9,243** | **9,830** | **31,020** | **34,404** | **1,899** | **1,840** | **1,421** | **1,526** | **965** | **1,006** | **760** | **696** | **1,408** | **1,552** |
| Kalabo | 13,199 | 6,478 | 6,721 | 1,012 | 1,049 | 903 | 816 | 713 | 782 | 2,883 | 3,198 | 251 | 233 | 228 | 201 | 159 | 143 | 107 | 108 | 222 | 191 |
| Kaoma | 24,932 | 12,155 | 12,777 | 2,403 | 2,429 | 2,033 | 2,099 | 1,853 | 1,953 | 5,078 | 5,650 | 251 | 197 | 161 | 164 | 100 | 85 | 90 | 60 | 186 | 140 |
| Lukulu | 8,424 | 4,135 | 4,289 | 1,070 | 1,090 | 808 | 766 | 566 | 613 | 1,407 | 1,538 | 88 | 77 | 64 | 71 | 38 | 34 | 39 | 25 | 55 | 75 |
| Mongu | 62,572 | 30,128 | 32,444 | 4,896 | 4,927 | 4,049 | 4,183 | 3,964 | 4,280 | 14,555 | 16,081 | 806 | 840 | 587 | 677 | 414 | 452 | 310 | 320 | 547 | 684 |
| Senanga | 20,129 | 9,784 | 10,345 | 1,605 | 1,618 | 1,351 | 1,346 | 1,267 | 1,289 | 4,376 | 4,853 | 337 | 324 | 254 | 277 | 172 | 199 | 145 | 129 | 277 | 310 |
| Sesheke | 12,509 | 6,044 | 6,465 | 1,321 | 1,342 | 986 | 947 | 749 | 772 | 2,476 | 2,833 | 149 | 162 | 115 | 134 | 75 | 84 | 63 | 49 | 110 | 142 |
| Shang'ombo | 1,587 | 802 | 785 | 205 | 195 | 168 | 165 | 131 | 141 | 245 | 251 | 17 | 7 | 12 | 2 | 7 | 9 | 6 | 5 | 11 | 10 |

## Table G3: Population Born in Zambia by Sex, Province of Birth and Province of Residence, Zambia 2010

| Province of Residence | Province of Birth | | | | | | | | | |
|---|---|---|---|---|---|---|---|---|---|---|
| | Population Born in Zambia | Central | Copperbelt | Eastern | Luapula | Lusaka | Northern | North Western | Southern | Western |
| **ZAMBIA TOTAL** | **12,424,534** | **1,148,590** | **1,953,742** | **1,746,789** | **1,015,686** | **1,557,482** | **1,794,363** | **692,105** | **1,619,231** | **896,546** |
| Male | 6,065,886 | 561,533 | 950,025 | 857,596 | 496,335 | 760,364 | 880,395 | 340,297 | 788,087 | 431,254 |
| Female | 6,358,648 | 587,057 | 1,003,717 | 889,193 | 519,351 | 797,118 | 913,968 | 351,808 | 831,144 | 465,292 |
| **Central Province** | | | | | | | | | | |
| **Total** | **1,239,961** | **934,283** | **64,394** | **21,786** | **12,425** | **62,267** | **48,057** | **6,486** | **73,310** | **16,953** |
| Male | 610,855 | 460,002 | 30,291 | 11,145 | 6,362 | 29,777 | 24,493 | 3,476 | 36,179 | 9,130 |
| Female | 629,106 | 474,281 | 34,103 | 10,641 | 6,063 | 32,490 | 23,564 | 3,010 | 37,131 | 7,823 |
| **Copperbelt Province** | | | | | | | | | | |
| **Total** | **1,906,628** | **54,531** | **1,487,913** | **38,717** | **75,808** | **51,520** | **105,906** | **51,517** | **29,597** | **11,119** |
| Male | 943,214 | 26,681 | 730,743 | 20,307 | 38,215 | 24,577 | 54,343 | 26,807 | 15,232 | 6,309 |
| Female | 963,414 | 27,850 | 757,170 | 18,410 | 37,593 | 26,943 | 51,563 | 24,710 | 14,365 | 4,810 |
| **Eastern Province** | | | | | | | | | | |
| **Total** | **1,608,004** | **8,087** | **31,994** | **1,504,229** | **1,857** | **43,704** | **6,874** | **1,314** | **8,005** | **1,940** |
| Male | 784,294 | 3,790 | 15,092 | 734,207 | 965 | 21,273 | 3,415 | 682 | 3,840 | 1,030 |
| Female | 823,710 | 4,297 | 16,902 | 770,022 | 892 | 22,431 | 3,459 | 632 | 4,165 | 910 |
| **Luapula Province** | | | | | | | | | | |
| Total | 931,898 | 3,680 | 37,057 | 1,979 | 857,590 | 6,405 | 21,581 | 1,088 | 1,815 | 703 |
| Male | 451,647 | 1,763 | 17,068 | 1,079 | 415,970 | 3,148 | 10,771 | 543 | 932 | 373 |
| Female | 480,251 | 1,917 | 19,989 | 900 | 441,620 | 3,257 | 10,810 | 545 | 883 | 330 |
| **Lusaka Province** | | | | | | | | | | |
| **Total** | **2,111,401** | **108,665** | **204,045** | **158,564** | **37,408** | **1,315,854** | **104,583** | **21,306** | **119,534** | **41,442** |
| Male | 1,034,841 | 50,719 | 96,511 | 79,680 | 19,046 | 644,401 | 53,231 | 11,465 | 57,664 | 22,124 |
| Female | 1,076,560 | 57,946 | 107,534 | 78,884 | 18,362 | 671,453 | 51,352 | 9,841 | 61,870 | 19,318 |
| **Northern Province** | | | | | | | | | | |
| **Total** | **1,617,064** | **12,535** | **57,990** | **5,904** | **21,484** | **19,294** | **1,492,462** | **1,408** | **4,632** | **1,355** |
| Male | 786,566 | 6,022 | 27,089 | 3,080 | 10,869 | 9,390 | 726,309 | 686 | 2,375 | 746 |
| Female | 830,498 | 6,513 | 30,901 | 2,824 | 10,615 | 9,904 | 766,153 | 722 | 2,257 | 609 |
| **North Western Province** | | | | | | | | | | |
| **Total** | **670,221** | **4,520** | **38,517** | **1,843** | **2,369** | **7,508** | **3,353** | **599,488** | **3,640** | **8,983** |
| Male | 326,549 | 2,202 | 18,245 | 1,013 | 1,255 | 3,645 | 1,792 | 292,022 | 1,886 | 4,489 |
| Female | 343,672 | 2,318 | 20,272 | 830 | 1,114 | 3,863 | 1,561 | 307,466 | 1,754 | 4,494 |
| **Southern Province** | | | | | | | | | | |
| **Total** | **1,510,924** | **18,044** | **23,963** | **12,174** | **5,144** | **41,071** | **10,179** | **3,569** | **1,366,480** | **30,300** |
| Male | 736,079 | 8,358 | 11,351 | 6,290 | 2,815 | 19,488 | 5,369 | 1,895 | 664,278 | 16,235 |
| Female | 774,845 | 9,686 | 12,612 | 5,884 | 2,329 | 21,583 | 4,810 | 1,674 | 702,202 | 14,065 |
| **Western Province** | | | | | | | | | | |
| **Total** | **828,433** | **4,245** | **7,869** | **1,593** | **1,601** | **9,859** | **1,368** | **5,929** | **12,218** | **783,751** |
| Male | 391,841 | 1,996 | 3,635 | 795 | 838 | 4,665 | 672 | 2,721 | 5,701 | 370,818 |
| Female | 436,592 | 2,249 | 4,234 | 798 | 763 | 5,194 | 696 | 3,208 | 6,517 | 412,933 |

## Table G4: Population (De Facto) and Immigrants by 5 Year Age Group, Duration of Stay in Zambia and Sex, Zambia 2010

| Age Group and Sex | Population | Total Immigrants | Less than 12 Months | 12-24 Months | 25-36 Months | 37-60 Months | 61 Months and Over |
|---|---|---|---|---|---|---|---|
| **Zambia Total** | **12,526,314** | **43,867** | **8,406** | **4,705** | **4,178** | **7,103** | **19,475** |
| 0 - 4 | 2,214,887 | 4,161 | 1,569 | 876 | 726 | 990 | - |
| 5 - 9 | 1,856,336 | 3,823 | 682 | 363 | 350 | 642 | 1,786 |
| 10 - 14 | 1,699,042 | 3,760 | 583 | 258 | 281 | 739 | 1,899 |
| 15 - 19 | 1,427,884 | 3,119 | 565 | 224 | 206 | 579 | 1,545 |
| 20 - 24 | 1,117,476 | 3,199 | 816 | 414 | 313 | 482 | 1,174 |
| 25 - 29 | 1,006,244 | 3,705 | 1,023 | 525 | 418 | 557 | 1,182 |
| 30 - 34 | 800,770 | 3,935 | 835 | 565 | 466 | 606 | 1,463 |
| 35 - 39 | 647,813 | 3,838 | 688 | 445 | 413 | 603 | 1,689 |
| 40 - 44 | 447,584 | 3,224 | 495 | 337 | 307 | 553 | 1,532 |
| 45 - 49 | 354,989 | 2,574 | 378 | 231 | 223 | 445 | 1,297 |
| 50 - 54 | 270,254 | 2,284 | 269 | 178 | 199 | 326 | 1,312 |
| 55 - 59 | 184,828 | 1,731 | 203 | 112 | 104 | 204 | 1,108 |
| 60 - 64 | 161,875 | 1,433 | 113 | 81 | 69 | 147 | 1,023 |
| 65+ | 336,332 | 3,081 | 187 | 96 | 103 | 230 | 2,465 |
| | | | | | | | |
| **Male** | **6,117,253** | **23,310** | **4,637** | **2,596** | **2,308** | **3,749** | **10,020** |
| 0 - 4 | 1,102,272 | 2,101 | 822 | 438 | 366 | 475 | - |
| 5 - 9 | 924,395 | 1,919 | 347 | 189 | 177 | 326 | 880 |
| 10 - 14 | 840,834 | 1,836 | 278 | 112 | 129 | 379 | 938 |
| 15 - 19 | 692,859 | 1,452 | 247 | 101 | 104 | 256 | 744 |
| 20 - 24 | 504,876 | 1,431 | 392 | 181 | 128 | 195 | 535 |
| 25 - 29 | 464,493 | 1,822 | 552 | 292 | 201 | 237 | 540 |
| 30 - 34 | 397,694 | 2,079 | 496 | 331 | 269 | 306 | 677 |
| 35 - 39 | 332,961 | 2,215 | 449 | 281 | 268 | 325 | 892 |
| 40 - 44 | 232,253 | 1,998 | 326 | 237 | 208 | 344 | 883 |
| 45 - 49 | 174,833 | 1,617 | 264 | 149 | 153 | 310 | 741 |
| 50 - 54 | 128,696 | 1,333 | 172 | 113 | 130 | 223 | 695 |
| 55 - 59 | 90,037 | 1,045 | 128 | 77 | 73 | 146 | 621 |
| 60 - 64 | 73,419 | 777 | 62 | 43 | 47 | 98 | 527 |
| 65+ | 157,631 | 1,685 | 102 | 52 | 55 | 129 | 1,347 |
| | | | | | | | |
| **Female** | **6,409,061** | **20,557** | **3,769** | **2,109** | **1,870** | **3,354** | **9,455** |
| 0 - 4 | 1,112,615 | 2,060 | 747 | 438 | 360 | 515 | - |
| 5 - 9 | 931,941 | 1,904 | 335 | 174 | 173 | 316 | 906 |
| 10 - 14 | 858,208 | 1,924 | 305 | 146 | 152 | 360 | 961 |
| 15 - 19 | 735,025 | 1,667 | 318 | 123 | 102 | 323 | 801 |
| 20 - 24 | 612,600 | 1,768 | 424 | 233 | 185 | 287 | 639 |
| 25 - 29 | 541,751 | 1,883 | 471 | 233 | 217 | 320 | 642 |
| 30 - 34 | 403,076 | 1,856 | 339 | 234 | 197 | 300 | 786 |
| 35 - 39 | 314,852 | 1,623 | 239 | 164 | 145 | 278 | 797 |
| 40 - 44 | 215,331 | 1,226 | 169 | 100 | 99 | 209 | 649 |
| 45 - 49 | 180,156 | 957 | 114 | 82 | 70 | 135 | 556 |
| 50 - 54 | 141558 | 951 | 97 | 65 | 69 | 103 | 617 |
| 55 - 59 | 94791 | 686 | 75 | 35 | 31 | 58 | 487 |
| 60 - 64 | 88456 | 656 | 51 | 38 | 22 | 49 | 496 |
| 65+ | 178,701 | 1,396 | 85 | 44 | 48 | 101 | 1,118 |

## Table G5: Migrant Population by Sex and Country of Citizenship, Rural/Urban, Zambian 2010

| Country of Citizenship | Total | | | Rural | | | Urban | | |
|---|---|---|---|---|---|---|---|---|---|
| | Total | Male | Female | Total | Male | Female | Total | Male | Female |
| **Immigrant Population** | **43,867** | **23,310** | **20,557** | **17,834** | **9,163** | **8,671** | **26,033** | **14,147** | **11,886** |
| Algeria | 5 | 2 | 3 | 2 | - | 2 | 3 | 2 | 1 |
| Angola | 5,574 | 2,831 | 2,743 | 5,436 | 2,756 | 2,680 | 138 | 75 | 63 |
| Benin | 1 | - | 1 | - | - | - | 1 | - | 1 |
| Botswana | 75 | 33 | 42 | 12 | 7 | 5 | 63 | 26 | 37 |
| Burundi | 831 | 463 | 368 | 312 | 168 | 144 | 519 | 295 | 224 |
| Cameroon | 37 | 21 | 16 | 1 | 1 | - | 36 | 20 | 16 |
| Cape Verde Islands | 5 | 3 | 2 | 3 | 2 | 1 | 2 | 1 | 1 |
| Central African Republic | 7 | 6 | 1 | - | - | - | 7 | 6 | 1 |
| Chad | 2 | 1 | 1 | - | - | - | 2 | 1 | 1 |
| Comoros | 21 | 8 | 13 | - | - | - | 21 | 8 | 13 |
| Congo | 3,092 | 1,548 | 1,544 | 850 | 452 | 398 | 2,242 | 1,096 | 1,146 |
| Egypt | 100 | 65 | 35 | 6 | 4 | 2 | 94 | 61 | 33 |
| Equatorial Guinea | 584 | 331 | 253 | 462 | 267 | 195 | 122 | 64 | 58 |
| Ethiopia | 70 | 36 | 34 | 5 | 5 | - | 65 | 31 | 34 |
| Gabon | 24 | 20 | 4 | - | - | - | 24 | 20 | 4 |
| Ghana | 132 | 85 | 47 | 2 | 2 | - | 130 | 83 | 47 |
| Guinea | 24 | 13 | 11 | 3 | 2 | 1 | 21 | 11 | 10 |
| Guinea – Bissau | 2 | 1 | 1 | 1 | - | 1 | 1 | 1 | - |
| Ivory Coast | 16 | 11 | 5 | - | - | - | 16 | 11 | 5 |
| Kenya | 288 | 164 | 124 | 11 | 6 | 5 | 277 | 158 | 119 |
| Lesotho | 28 | 25 | 3 | 1 | 1 | - | 27 | 24 | 3 |
| Liberia | 44 | 26 | 18 | - | - | - | 44 | 26 | 18 |
| Libya | 14 | 7 | 7 | 4 | 2 | 2 | 10 | 5 | 5 |
| Madagascar | 17 | 9 | 8 | 1 | 1 | - | 16 | 8 | 8 |
| Malawi | 579 | 331 | 248 | 230 | 126 | 104 | 349 | 205 | 144 |
| Mali | 174 | 104 | 70 | 5 | 4 | 1 | 169 | 100 | 69 |
| Mauritania | 104 | 69 | 35 | 52 | 38 | 14 | 52 | 31 | 21 |
| Mauritius | 27 | 11 | 16 | - | - | - | 27 | 11 | 16 |
| Morocco | 5 | 3 | 2 | - | - | - | 5 | 3 | 2 |
| Mozambique | 93 | 61 | 32 | 24 | 16 | 8 | 69 | 45 | 24 |
| Niger | 2 | 1 | 1 | 2 | 1 | 1 | - | - | - |
| Nigeria | 189 | 148 | 41 | 12 | 9 | 3 | 177 | 139 | 38 |
| Rwanda | 2,381 | 1,212 | 1,169 | 1,495 | 746 | 749 | 886 | 466 | 420 |
| Senegal | 118 | 91 | 27 | 1 | 1 | - | 117 | 90 | 27 |
| Sierra Leone | 34 | 23 | 11 | 2 | 1 | 1 | 32 | 22 | 10 |
| Somalia | 1,293 | 689 | 604 | 101 | 46 | 55 | 1,192 | 643 | 549 |
| South Africa | 1,574 | 868 | 706 | 303 | 165 | 138 | 1,271 | 703 | 568 |
| Namibia | 61 | 36 | 25 | 19 | 12 | 7 | 42 | 24 | 18 |
| Sudan | 67 | 38 | 29 | 7 | 7 | - | 60 | 31 | 29 |

## Table G5: Migrant Population by Sex and Country of Citizenship, Rural/Urban, Zambian 2010

| Country of Citizenship | Total | | | Rural | | | Urban | | |
|---|---|---|---|---|---|---|---|---|---|
| | Total | Male | Female | Total | Male | Female | Total | Male | Female |
| Swaziland | 24 | 9 | 15 | 9 | 1 | 8 | 15 | 8 | 7 |
| Tanzania | 2,322 | 1,206 | 1,116 | 457 | 218 | 239 | 1,865 | 988 | 877 |
| Togo | 33 | 23 | 10 | 1 | 1 | - | 32 | 22 | 10 |
| Tunisia | 136 | 72 | 64 | 11 | 6 | 5 | 125 | 66 | 59 |
| Uganda | 239 | 158 | 81 | 22 | 16 | 6 | 217 | 142 | 75 |
| Burkina Faso | 4 | 1 | 3 | 1 | - | 1 | 3 | 1 | 2 |
| Congo DR | 4,150 | 2,128 | 2,022 | 3,010 | 1,562 | 1,448 | 1,140 | 566 | 574 |
| Zimbabwe | 5,094 | 2,481 | 2,613 | 2,524 | 1,196 | 1,328 | 2,570 | 1,285 | 1,285 |
| Other Africa | 56 | 31 | 25 | - | - | - | 56 | 31 | 25 |
| Afghanistan | 7 | 4 | 3 | - | - | - | 7 | 4 | 3 |
| Australia | 139 | 81 | 58 | 84 | 51 | 33 | 55 | 30 | 25 |
| Bahrain | 6 | 1 | 5 | - | - | - | 6 | 1 | 5 |
| Bangladesh | 56 | 30 | 26 | 1 | 1 | - | 55 | 29 | 26 |
| Bhutan | 15 | 8 | 7 | - | - | - | 15 | 8 | 7 |
| Burma | 2 | 2 | - | - | - | - | 2 | 2 | - |
| China | 681 | 549 | 132 | 159 | 129 | 30 | 522 | 420 | 102 |
| Fiji | 6 | 2 | 4 | 1 | 1 | - | 5 | 1 | 4 |
| Hong Kong | 137 | 119 | 18 | 24 | 24 | - | 113 | 95 | 18 |
| India | 3,880 | 2,151 | 1,729 | 134 | 75 | 59 | 3,746 | 2,076 | 1,670 |
| Indonesia | 27 | 18 | 9 | - | - | - | 27 | 18 | 9 |
| Iran | 4 | 3 | 1 | 1 | - | 1 | 3 | 3 | - |
| Iraq | 331 | 180 | 151 | 12 | 7 | 5 | 319 | 173 | 146 |
| Israel | 18 | 10 | 8 | - | - | - | 18 | 10 | 8 |
| Japan | 69 | 32 | 37 | 5 | 2 | 3 | 64 | 30 | 34 |
| Jordan | 6 | 4 | 2 | - | - | - | 6 | 4 | 2 |
| Cambodia | 4 | 3 | 1 | 1 | 1 | - | 3 | 2 | 1 |
| Dem. P. R. of Korea | 32 | 20 | 12 | 1 | 1 | - | 31 | 19 | 12 |
| Korea Republic | 44 | 24 | 20 | 7 | 4 | 3 | 37 | 20 | 17 |
| Kuwait | 7 | 5 | 2 | - | - | - | 7 | 5 | 2 |
| Lebanon | 325 | 189 | 136 | 14 | 8 | 6 | 311 | 181 | 130 |
| Malaysia | 7 | 4 | 3 | - | - | - | 7 | 4 | 3 |
| Mongolia | 1 | - | 1 | - | - | - | 1 | - | 1 |
| Nepal | 58 | 42 | 16 | - | - | - | 58 | 42 | 16 |
| New Zealand | 46 | 28 | 18 | 25 | 17 | 8 | 21 | 11 | 10 |
| Oman | 5 | 5 | - | - | - | - | 5 | 5 | - |
| Pakistan | 123 | 71 | 52 | 5 | 5 | - | 118 | 66 | 52 |
| Papua-New Guinea | 2 | 1 | 1 | 2 | 1 | 1 | - | - | - |
| Philippines | 85 | 44 | 41 | 6 | 3 | 3 | 79 | 41 | 38 |
| Saudi Arabia | 5 | 2 | 3 | - | - | - | 5 | 2 | 3 |

## Table G5: Migrant Population by Sex and Country of Citizenship, Rural/Urban, Zambian 2010

| Country of Citizenship | Total | | | Rural | | | Urban | | |
|---|---|---|---|---|---|---|---|---|---|
| | Total | Male | Female | Total | Male | Female | Total | Male | Female |
| Singapore | 7 | 4 | 3 | 1 | - | 1 | 6 | 4 | 2 |
| Sri Lanka | 118 | 63 | 55 | 2 | 2 | - | 116 | 61 | 55 |
| Syria | 6 | 6 | - | 2 | 2 | - | 4 | 4 | - |
| Taiwan | 5 | 5 | - | - | - | - | 5 | 5 | - |
| Thailand | 26 | 12 | 14 | - | - | - | 26 | 12 | 14 |
| United Arab Emirates | 6 | 3 | 3 | - | - | - | 6 | 3 | 3 |
| Vietnam | 5 | 2 | 3 | - | - | - | 5 | 2 | 3 |
| Yemen Arab Rep of | 20 | 15 | 5 | 5 | 3 | 2 | 15 | 12 | 3 |
| Other Asia and Oceania | 87 | 47 | 40 | 10 | 3 | 7 | 77 | 44 | 33 |
| Barbados | 1 | 1 | - | - | - | - | 1 | 1 | - |
| Bolivia | 1 | - | 1 | - | - | - | 1 | - | 1 |
| Brazil | 23 | 14 | 9 | - | - | - | 23 | 14 | 9 |
| Canada | 135 | 69 | 66 | 38 | 16 | 22 | 97 | 53 | 44 |
| Chile | 9 | 6 | 3 | - | - | - | 9 | 6 | 3 |
| Colombia | 10 | 5 | 5 | 1 | 1 | - | 9 | 4 | 5 |
| Dominican Republic | 1 | - | 1 | - | - | - | 1 | - | 1 |
| El Salvador | 1 | 1 | - | 1 | 1 | - | - | - | - |
| Guyana | 5 | 1 | 4 | - | - | - | 5 | 1 | 4 |
| Jamaica | 6 | 3 | 3 | 1 | 1 | - | 5 | 2 | 3 |
| Mexico | 11 | 4 | 7 | 3 | 2 | 1 | 8 | 2 | 6 |
| Paraguay | 5 | 4 | 1 | - | - | - | 5 | 4 | 1 |
| Uruguay | 4 | 2 | 2 | - | - | - | 4 | 2 | 2 |
| United States of America (USA) | 643 | 305 | 338 | 127 | 60 | 67 | 516 | 245 | 271 |
| Venezuela | 1 | 1 | - | - | - | - | 1 | 1 | - |
| Other Americas | 96 | 63 | 33 | 5 | 1 | 4 | 91 | 62 | 29 |
| Albania | 7 | 2 | 5 | - | - | - | 7 | 2 | 5 |
| Austria | 17 | 10 | 7 | 7 | 5 | 2 | 10 | 5 | 5 |
| Belgium | 32 | 16 | 16 | 6 | 2 | 4 | 26 | 14 | 12 |
| Bulgaria | 9 | 4 | 5 | - | - | - | 9 | 4 | 5 |
| Cyprus | 22 | 13 | 9 | - | - | - | 22 | 13 | 9 |
| Czechoslovakia | 6 | 3 | 3 | 2 | 1 | 1 | 4 | 2 | 2 |
| Denmark | 56 | 33 | 23 | 15 | 12 | 3 | 41 | 21 | 20 |
| Finland | 22 | 8 | 14 | - | - | - | 22 | 8 | 14 |
| France | 46 | 28 | 18 | 4 | 3 | 1 | 42 | 25 | 17 |
| Germany | 161 | 81 | 80 | 21 | 9 | 12 | 140 | 72 | 68 |
| Greece | 70 | 47 | 23 | 10 | 8 | 2 | 60 | 39 | 21 |
| Hungary | 8 | 4 | 4 | 1 | 1 | - | 7 | 3 | 4 |
| Ireland | 85 | 36 | 49 | 17 | 9 | 8 | 68 | 27 | 41 |
| Italy | 126 | 69 | 57 | 13 | 8 | 5 | 113 | 61 | 52 |
| Luxemburg | 3 | 2 | 1 | - | - | - | 3 | 2 | 1 |
| Malta | 26 | 13 | 13 | 3 | 2 | 1 | 23 | 11 | 12 |

## Table G5: Migrant Population by Sex and Country of Citizenship, Rural/Urban, Zambian 2010

| Country of Citizenship | Total | | | Rural | | | Urban | | |
|---|---|---|---|---|---|---|---|---|---|
| | Total | Male | Female | Total | Male | Female | Total | Male | Female |
| Netherlands | 88 | 51 | 37 | 34 | 19 | 15 | 54 | 32 | 22 |
| Norway | 17 | 6 | 11 | 3 | - | 3 | 14 | 6 | 8 |
| Poland | 45 | 31 | 14 | 9 | 6 | 3 | 36 | 25 | 11 |
| Portugal | 39 | 20 | 19 | 10 | 5 | 5 | 29 | 15 | 14 |
| Romania | 43 | 26 | 17 | 2 | 1 | 1 | 41 | 25 | 16 |
| Spain | 22 | 15 | 7 | 2 | 2 | - | 20 | 13 | 7 |
| Sweden | 37 | 21 | 16 | 2 | 1 | 1 | 35 | 20 | 15 |
| Switzerland | 34 | 18 | 16 | 12 | 5 | 7 | 22 | 13 | 9 |
| Turkey | 12 | 7 | 5 | - | - | - | 12 | 7 | 5 |
| United Kingdom | 860 | 456 | 404 | 193 | 101 | 92 | 667 | 355 | 312 |
| Russian | 40 | 17 | 23 | 6 | 2 | 4 | 34 | 15 | 19 |
| Yugoslavia (Former) | 16 | 10 | 6 | - | - | - | 16 | 10 | 6 |
| Other Europe | 227 | 129 | 98 | 42 | 17 | 25 | 185 | 112 | 73 |
| Not Stated | 4,579 | 2,363 | 2,216 | 1,342 | 674 | 668 | 3,237 | 1,689 | 1,548 |

## Table G6: Migrant Population by Purpose of Stay in Zambia, 5 Year Age Group, Sex and Province, Zambia 2010

| Age Group, Sex and Province | Purpose Of Stay | | | | | | | |
|---|---|---|---|---|---|---|---|---|
| | Total | Employment | Family Reunification/ Formation | Education/ Training | Settlement | Refuge/ Asylum | Investor | Other |
| **Zambia** | | | | | | | | |
| **Total** | **43,867** | **8,095** | **8,278** | **1,131** | **8,868** | **12,893** | **1,357** | **3,245** |
| Male | 23,310 | 5,981 | 3,059 | 595 | 4,458 | 6,568 | 987 | 1,662 |
| Female | 20,557 | 2,114 | 5,219 | 536 | 4,410 | 6,325 | 370 | 1,583 |
| | | | | | | | | |
| **0 - 4** | **4,161** | **-** | **1,027** | **138** | **795** | **1,904** | **-** | **297** |
| Male | 2,101 | - | 516 | 68 | 421 | 945 | - | 151 |
| Female | 2,060 | - | 511 | 70 | 374 | 959 | - | 146 |
| | | | | | | | | |
| **5 - 9** | **3,823** | **-** | **943** | **193** | **714** | **1,740** | **-** | **233** |
| Male | 1,919 | - | 485 | 105 | 353 | 869 | - | 107 |
| Female | 1,904 | - | 458 | 88 | 361 | 871 | - | 126 |
| | | | | | | | | |
| **10 - 14** | **3,760** | **74** | **758** | **225** | **731** | **1,798** | **-** | **174** |
| Male | 1,836 | 40 | 373 | 112 | 370 | 855 | - | 86 |
| Female | 1,924 | 34 | 385 | 113 | 361 | 943 | - | 88 |
| | | | | | | | | |
| **15 - 19** | **3,119** | **133** | **566** | **181** | **620** | **1,422** | **35** | **162** |
| Male | 1,452 | 60 | 260 | 86 | 295 | 650 | 17 | 84 |
| Female | 1,667 | 73 | 306 | 95 | 325 | 772 | 18 | 78 |
| | | | | | | | | |
| **20 - 24** | **3,199** | **487** | **619** | **120** | **629** | **1,024** | **77** | **243** |
| Male | 1,431 | 309 | 195 | 62 | 251 | 457 | 52 | 105 |
| Female | 1,768 | 178 | 424 | 58 | 378 | 567 | 25 | 138 |
| | | | | | | | | |
| **25 - 29** | **3,705** | **1,008** | **737** | **77** | **693** | **689** | **146** | **355** |
| Male | 1,822 | 712 | 186 | 46 | 309 | 287 | 106 | 176 |
| Female | 1,883 | 296 | 551 | 31 | 384 | 402 | 40 | 179 |
| | | | | | | | | |
| **30 - 34** | **3,935** | **1,224** | **694** | **46** | **692** | **783** | **170** | **326** |
| Male | 2,079 | 907 | 148 | 31 | 348 | 349 | 113 | 183 |
| Female | 1,856 | 317 | 546 | 15 | 344 | 434 | 57 | 143 |
| | | | | | | | | |
| **35 - 39** | **3,838** | **1,259** | **570** | **28** | **634** | **852** | **181** | **314** |
| Male | 2,215 | 953 | 166 | 19 | 338 | 422 | 131 | 186 |
| Female | 1,623 | 306 | 404 | 9 | 296 | 430 | 50 | 128 |
| | | | | | | | | |
| **40 - 44** | **3,224** | **1,031** | **479** | **34** | **510** | **780** | **177** | **213** |
| Male | 1,998 | 812 | 147 | 20 | 304 | 453 | 141 | 121 |
| Female | 1,226 | 219 | 332 | 14 | 206 | 327 | 36 | 92 |
| | | | | | | | | |
| **45 - 49** | **2,574** | **832** | **343** | **21** | **412** | **606** | **169** | **191** |
| Male | 1,617 | 658 | 99 | 10 | 228 | 386 | 132 | 104 |
| Female | 957 | 174 | 244 | 11 | 184 | 220 | 37 | 87 |
| | | | | | | | | |
| **50 - 54** | **2,284** | **688** | **363** | **17** | **470** | **439** | **130** | **177** |
| Male | 1,333 | 518 | 106 | 12 | 227 | 293 | 94 | 83 |
| Female | 951 | 170 | 257 | 5 | 243 | 146 | 36 | 94 |

## Table G6: Migrant Population by Purpose of Stay in Zambia, 5 Year Age Group, Sex and Province, Zambia 2010

| Age Group, Sex and Province | Purpose Of Stay | | | | | | | |
|---|---|---|---|---|---|---|---|---|
| | Total | Employment | Family Reunification/ Formation | Education/ Training | Settlement | Refuge/ Asylum | Investor | Other |
| **55 - 59** | **1,731** | **512** | **294** | **19** | **406** | **266** | **97** | **137** |
| Male | 1,045 | 385 | 90 | 11 | 218 | 195 | 77 | 69 |
| Female | 686 | 127 | 204 | 8 | 188 | 71 | 20 | 68 |
| | | | | | | | | |
| **60 - 64** | **1,433** | **336** | **254** | **9** | **423** | **211** | **76** | **124** |
| Male | 777 | 249 | 81 | 2 | 196 | 143 | 54 | 52 |
| Female | 656 | 87 | 173 | 7 | 227 | 68 | 22 | 72 |
| | | | | | | | | |
| **65+** | **3,081** | **511** | **631** | **23** | **1,139** | **379** | **99** | **299** |
| Male | 1,685 | 378 | 207 | 11 | 600 | 264 | 70 | 155 |
| Female | 1,396 | 133 | 424 | 12 | 539 | 115 | 29 | 144 |
| **Central Province** | | | | | | | | |
| **Total** | **3,336** | **288** | **634** | **53** | **1,855** | **53** | **138** | **315** |
| Male | 1,647 | 194 | 258 | 25 | 890 | 25 | 91 | 164 |
| Female | 1,689 | 94 | 376 | 28 | 965 | 28 | 47 | 151 |
| | | | | | | | | |
| **0 - 4** | **272** | **-** | **48** | **6** | **178** | **7** | **-** | **33** |
| Male | 142 | - | 25 | 3 | 94 | 3 | - | 17 |
| Female | 130 | - | 23 | 3 | 84 | 4 | - | 16 |
| | | | | | | | | |
| **5 - 9** | **250** | **-** | **51** | **8** | **155** | **5** | **-** | **31** |
| Male | 122 | - | 27 | 2 | 76 | 2 | - | 15 |
| Female | 128 | - | 24 | 6 | 79 | 3 | - | 16 |
| | | | | | | | | |
| **10 - 14** | **231** | **1** | **40** | **6** | **162** | **5** | **-** | **17** |
| Male | 121 | - | 21 | 4 | 85 | 2 | - | 9 |
| Female | 110 | 1 | 19 | 2 | 77 | 3 | - | 8 |
| | | | | | | | | |
| **15 - 19** | **178** | **3** | **32** | **5** | **127** | **-** | **2** | **9** |
| Male | 102 | - | 21 | 1 | 72 | - | 2 | 6 |
| Female | 76 | 3 | 11 | 4 | 55 | - | - | 3 |
| | | | | | | | | |
| **20 - 24** | **165** | **18** | **32** | **2** | **84** | **4** | **1** | **24** |
| Male | 70 | 10 | 9 | 1 | 36 | 2 | 1 | 11 |
| Female | 95 | 8 | 23 | 1 | 48 | 2 | - | 13 |
| | | | | | | | | |
| **25 - 29** | **139** | **21** | **25** | **4** | **56** | **5** | **8** | **20** |
| Male | 64 | 16 | 9 | 2 | 22 | 3 | 6 | 6 |
| Female | 75 | 5 | 16 | 2 | 34 | 2 | 2 | 14 |
| | | | | | | | | |
| **30 - 34** | **157** | **25** | **25** | **2** | **70** | **2** | **16** | **17** |
| Male | 76 | 21 | 6 | 1 | 33 | 1 | 8 | 6 |
| Female | 81 | 4 | 19 | 1 | 37 | 1 | 8 | 11 |
| | | | | | | | | |
| **35 - 39** | **207** | **49** | **33** | **3** | **65** | **1** | **28** | **28** |
| Male | 121 | 33 | 18 | 2 | 31 | 1 | 20 | 16 |
| Female | 86 | 16 | 15 | 1 | 34 | - | 8 | 12 |
| | | | | | | | | |
| **40 - 44** | **182** | **43** | **20** | **3** | **80** | **-** | **21** | **15** |
| Male | 100 | 27 | 10 | 2 | 38 | - | 14 | 9 |
| Female | 82 | 16 | 10 | 1 | 42 | - | 7 | 6 |

## Table G6: Migrant Population by Purpose of Stay in Zambia, 5 Year Age Group, Sex and Province, Zambia 2010

| Age Group, Sex and Province | Purpose Of Stay | | | | | | | |
|---|---|---|---|---|---|---|---|---|
| | Total | Employment | Family Reunification/ Formation | Education/ Training | Settlement | Refuge/ Asylum | Investor | Other |
| **45 - 49** | **147** | **29** | **23** | **1** | **65** | **3** | **14** | **12** |
| Male | 74 | 18 | 4 | - | 34 | 1 | 11 | 6 |
| Female | 73 | 11 | 19 | 1 | 31 | 2 | 3 | 6 |
| | | | | | | | | |
| **50 - 54** | **231** | **30** | **50** | **4** | **123** | **5** | **10** | **9** |
| Male | 91 | 21 | 12 | 2 | 45 | 2 | 6 | 3 |
| Female | 140 | 9 | 38 | 2 | 78 | 3 | 4 | 6 |
| | | | | | | | | |
| **55 - 59** | **224** | **20** | **48** | **4** | **119** | **4** | **11** | **18** |
| Male | 103 | 14 | 16 | 2 | 51 | 2 | 6 | 12 |
| Female | 121 | 6 | 32 | 2 | 68 | 2 | 5 | 6 |
| | | | | | | | | |
| **60 - 64** | **226** | **10** | **58** | **1** | **123** | **1** | **14** | **19** |
| Male | 104 | 7 | 31 | - | 51 | - | 7 | 8 |
| Female | 122 | 3 | 27 | 1 | 72 | 1 | 7 | 11 |
| | | | | | | | | |
| **65+** | **727** | **39** | **149** | **4** | **448** | **11** | **13** | **63** |
| Male | 357 | 27 | 49 | 3 | 222 | 6 | 10 | 40 |
| Female | 370 | 12 | 100 | 1 | 226 | 5 | 3 | 23 |
| **Copperbelt Province** | | | | | | | | |
| **Total** | **6,831** | **2,091** | **1,903** | **221** | **1,391** | **151** | **307** | **767** |
| Male | 3,895 | 1,702 | 648 | 109 | 701 | 89 | 232 | 414 |
| Female | 2,936 | 389 | 1,255 | 112 | 690 | 62 | 75 | 353 |
| | | | | | | | | |
| **0 - 4** | **409** | **-** | **224** | **29** | **76** | **20** | **-** | **60** |
| Male | 198 | - | 107 | 14 | 41 | 8 | - | 28 |
| Female | 211 | - | 117 | 15 | 35 | 12 | - | 32 |
| | | | | | | | | |
| **5 - 9** | **420** | **-** | **217** | **51** | **84** | **15** | **-** | **53** |
| Male | 222 | - | 113 | 30 | 44 | 10 | - | 25 |
| Female | 198 | - | 104 | 21 | 40 | 5 | - | 28 |
| | | | | | | | | |
| **10 - 14** | **387** | **10** | **168** | **58** | **91** | **14** | **-** | **46** |
| Male | 176 | 4 | 70 | 24 | 42 | 9 | - | 27 |
| Female | 211 | 6 | 98 | 34 | 49 | 5 | - | 19 |
| | | | | | | | | |
| **15 - 19** | **303** | **14** | **114** | **32** | **79** | **4** | **17** | **43** |
| Male | 138 | 4 | 60 | 7 | 39 | 1 | 7 | 20 |
| Female | 165 | 10 | 54 | 25 | 40 | 3 | 10 | 23 |
| | | | | | | | | |
| **20 - 24** | **392** | **88** | **131** | **18** | **79** | **14** | **22** | **40** |
| Male | 183 | 64 | 45 | 11 | 27 | 5 | 15 | 16 |
| Female | 209 | 24 | 86 | 7 | 52 | 9 | 7 | 24 |
| | | | | | | | | |
| **25 - 29** | **645** | **230** | **174** | **3** | **112** | **20** | **37** | **69** |
| Male | 363 | 187 | 46 | 3 | 48 | 8 | 30 | 41 |
| Female | 282 | 43 | 128 | - | 64 | 12 | 7 | 28 |
| | | | | | | | | |
| **30 - 34** | **701** | **303** | **178** | **12** | **93** | **18** | **35** | **62** |
| Male | 408 | 252 | 33 | 7 | 48 | 10 | 25 | 33 |
| Female | 293 | 51 | 145 | 5 | 45 | 8 | 10 | 29 |

## Table G6: Migrant Population by Purpose of Stay in Zambia, 5 Year Age Group, Sex and Province, Zambia 2010

| Age Group, Sex and Province | Purpose Of Stay | | | | | | | |
|---|---|---|---|---|---|---|---|---|
| | Total | Employment | Family Reunification/ Formation | Education/ Training | Settlement | Refuge/ Asylum | Investor | Other |
| **35 - 39** | **649** | **296** | **140** | **1** | **92** | **14** | **30** | **76** |
| Male | 392 | 245 | 30 | 1 | 41 | 10 | 18 | 47 |
| Female | 257 | 51 | 110 | - | 51 | 4 | 12 | 29 |
| | | | | | | | | |
| **40 - 44** | **561** | **252** | **118** | **6** | **83** | **8** | **34** | **60** |
| Male | 382 | 219 | 34 | 4 | 57 | 8 | 30 | 30 |
| Female | 179 | 33 | 84 | 2 | 26 | - | 4 | 30 |
| | | | | | | | | |
| **45 - 49** | **495** | **231** | **83** | **2** | **73** | **9** | **46** | **51** |
| Male | 332 | 199 | 19 | 2 | 35 | 9 | 39 | 29 |
| Female | 163 | 32 | 64 | - | 38 | - | 7 | 22 |
| | | | | | | | | |
| **50 - 54** | **469** | **212** | **85** | **3** | **80** | **6** | **29** | **54** |
| Male | 262 | 155 | 14 | 3 | 36 | 5 | 23 | 26 |
| Female | 207 | 57 | 71 | - | 44 | 1 | 6 | 28 |
| | | | | | | | | |
| **55 - 59** | **385** | **168** | **70** | **1** | **82** | **4** | **19** | **41** |
| Male | 240 | 134 | 19 | 1 | 42 | 2 | 17 | 25 |
| Female | 145 | 34 | 51 | - | 40 | 2 | 2 | 16 |
| | | | | | | | | |
| **60 - 64** | **330** | **112** | **60** | **2** | **111** | **1** | **12** | **32** |
| Male | 192 | 100 | 10 | - | 53 | 1 | 9 | 19 |
| Female | 138 | 12 | 50 | 2 | 58 | - | 3 | 13 |
| | | | | | | | | |
| **65+** | **685** | **175** | **141** | **3** | **256** | **4** | **26** | **80** |
| Male | 407 | 139 | 48 | 2 | 148 | 3 | 19 | 48 |
| Female | 278 | 36 | 93 | 1 | 108 | 1 | 7 | 32 |
| **Eastern Province** | | | | | | | | |
| **Total** | **611** | **203** | **152** | **22** | **120** | **9** | **24** | **81** |
| Male | 339 | 149 | 49 | 12 | 63 | 5 | 17 | 44 |
| Female | 272 | 54 | 103 | 10 | 57 | 4 | 7 | 37 |
| | | | | | | | | |
| **0 - 4** | **40** | **-** | **17** | **1** | **13** | **-** | **-** | **9** |
| Male | 18 | - | 6 | - | 7 | - | - | 5 |
| Female | 22 | - | 11 | 1 | 6 | - | - | 4 |
| | | | | | | | | |
| **5 - 9** | **25** | **-** | **7** | **1** | **7** | **3** | **-** | **7** |
| Male | 7 | - | 2 | - | 1 | 2 | - | 2 |
| Female | 18 | - | 5 | 1 | 6 | 1 | - | 5 |
| | | | | | | | | |
| **10 - 14** | **26** | **2** | **8** | **2** | **8** | **1** | **-** | **5** |
| Male | 13 | 1 | 4 | 1 | 6 | 1 | - | - |
| Female | 13 | 1 | 4 | 1 | 2 | - | - | 5 |
| | | | | | | | | |
| **15 - 19** | **36** | **6** | **8** | **3** | **11** | **-** | **-** | **8** |
| Male | 18 | 3 | 3 | 2 | 5 | - | - | 5 |
| Female | 18 | 3 | 5 | 1 | 6 | - | - | 3 |
| | | | | | | | | |
| **20 - 24** | **54** | **18** | **16** | **4** | **7** | **-** | **3** | **6** |
| Male | 26 | 12 | 5 | 3 | 3 | - | 1 | 2 |
| Female | 28 | 6 | 11 | 1 | 4 | - | 2 | 4 |

## Table G6: Migrant Population by Purpose of Stay in Zambia, 5 Year Age Group, Sex and Province, Zambia 2010

| Age Group, Sex and Province | Purpose Of Stay | | | | | | | |
|---|---|---|---|---|---|---|---|---|
| | Total | Employment | Family Reunification/ Formation | Education/ Training | Settlement | Refuge/ Asylum | Investor | Other |
| **25 - 29** | **82** | **37** | **19** | **6** | **7** | **-** | **3** | **10** |
| Male | 43 | 25 | 4 | 2 | 3 | - | 2 | 7 |
| Female | 39 | 12 | 15 | 4 | 4 | - | 1 | 3 |
| | | | | | | | | |
| **30 - 34** | **61** | **25** | **16** | **1** | **8** | **-** | **3** | **8** |
| Male | 36 | 18 | 5 | 1 | 4 | - | 3 | 5 |
| Female | 25 | 7 | 11 | - | 4 | - | - | 3 |
| | | | | | | | | |
| **35 - 39** | **57** | **21** | **11** | **2** | **9** | **1** | **2** | **11** |
| Male | 36 | 15 | 5 | 2 | 5 | - | 2 | 7 |
| Female | 21 | 6 | 6 | - | 4 | 1 | - | 4 |
| | | | | | | | | |
| **40 - 44** | **57** | **28** | **13** | **1** | **12** | **-** | **1** | **2** |
| Male | 40 | 23 | 5 | 1 | 9 | - | - | 2 |
| Female | 17 | 5 | 8 | - | 3 | - | 1 | - |
| | | | | | | | | |
| **45 - 49** | **40** | **20** | **4** | **-** | **9** | **2** | **2** | **3** |
| Male | 31 | 18 | 2 | - | 7 | 1 | 1 | 2 |
| Female | 9 | 2 | 2 | - | 2 | 1 | 1 | 1 |
| | | | | | | | | |
| **50 - 54** | **32** | **16** | **6** | **-** | **5** | **-** | **2** | **3** |
| Male | 20 | 11 | 3 | - | 3 | - | 2 | 1 |
| Female | 12 | 5 | 3 | - | 2 | - | - | 2 |
| | | | | | | | | |
| **55 - 59** | **38** | **18** | **4** | **1** | **5** | **1** | **5** | **4** |
| Male | 25 | 14 | 1 | - | 2 | 1 | 4 | 3 |
| Female | 13 | 4 | 3 | 1 | 3 | - | 1 | 1 |
| | | | | | | | | |
| **60 - 64** | **21** | **7** | **8** | **-** | **4** | **-** | **2** | **-** |
| Male | 12 | 5 | 2 | - | 3 | - | 2 | - |
| Female | 9 | 2 | 6 | - | 1 | - | - | - |
| | | | | | | | | |
| **65+** | **42** | **5** | **15** | **0** | **15** | **1** | **1** | **5** |
| Male | 14 | 4 | 2 | 0 | 5 | 0 | 0 | 3 |
| Female | 28 | 1 | 13 | 0 | 10 | 1 | 1 | 2 |
| **Luapula Province** | | | | | | | | |
| **Total** | **882** | **75** | **127** | **10** | **460** | **56** | **19** | **135** |
| Male | 500 | 59 | 64 | 6 | 236 | 32 | 13 | 90 |
| Female | 382 | 16 | 63 | 4 | 224 | 24 | 6 | 45 |
| | | | | | | | | |
| **0 - 4** | **100** | **-** | **22** | **1** | **64** | **4** | **-** | **9** |
| Male | 62 | - | 13 | 1 | 37 | 4 | - | 7 |
| Female | 38 | - | 9 | - | 27 | - | - | 2 |
| | | | | | | | | |
| **5 - 9** | **73** | **-** | **19** | **2** | **45** | **4** | **-** | **3** |
| Male | 32 | - | 8 | 1 | 20 | 1 | - | 2 |
| Female | 41 | - | 11 | 1 | 25 | 3 | - | 1 |
| | | | | | | | | |
| **10 - 14** | **70** | **-** | **7** | **-** | **51** | **6** | **-** | **6** |
| Male | 37 | - | 5 | - | 25 | 3 | - | 4 |
| Female | 33 | - | 2 | - | 26 | 3 | - | 2 |

## Table G6: Migrant Population by Purpose of Stay in Zambia, 5 Year Age Group, Sex and Province, Zambia 2010

| Age Group, Sex and Province | Purpose Of Stay | | | | | | | |
|---|---|---|---|---|---|---|---|---|
| | Total | Employment | Family Reunification/ Formation | Education/ Training | Settlement | Refuge/ Asylum | Investor | Other |
| **15 - 19** | **66** | **5** | **13** | **-** | **36** | **6** | **-** | **6** |
| Male | 39 | 5 | 6 | - | 20 | 4 | - | 4 |
| Female | 27 | - | 7 | - | 16 | 2 | - | 2 |
| | | | | | | | | |
| **20 - 24** | **107** | **21** | **16** | **3** | **40** | **5** | **1** | **21** |
| Male | 54 | 16 | 4 | 1 | 14 | 2 | 1 | 16 |
| Female | 53 | 5 | 12 | 2 | 26 | 3 | - | 5 |
| | | | | | | | | |
| **25 - 29** | **86** | **14** | **11** | **1** | **44** | **3** | **-** | **13** |
| Male | 42 | 10 | 3 | 1 | 20 | 1 | - | 7 |
| Female | 44 | 4 | 8 | - | 24 | 2 | - | 6 |
| | | | | | | | | |
| **30 - 34** | **80** | **13** | **5** | **-** | **40** | **5** | **3** | **14** |
| Male | 42 | 10 | 2 | - | 19 | 3 | 1 | 7 |
| Female | 38 | 3 | 3 | - | 21 | 2 | 2 | 7 |
| | | | | | | | | |
| **35 - 39** | **82** | **6** | **6** | **1** | **40** | **3** | **9** | **17** |
| Male | 48 | 5 | 1 | - | 22 | 2 | 6 | 12 |
| Female | 34 | 1 | 5 | 1 | 18 | 1 | 3 | 5 |
| | | | | | | | | |
| **40 - 44** | **72** | **7** | **8** | **1** | **30** | **9** | **3** | **14** |
| Male | 51 | 7 | 6 | 1 | 19 | 5 | 3 | 10 |
| Female | 21 | - | 2 | - | 11 | 4 | - | 4 |
| | | | | | | | | |
| **45 - 49** | **58** | **-** | **5** | **1** | **30** | **5** | **1** | **16** |
| Male | 39 | - | 5 | 1 | 17 | 5 | 1 | 10 |
| Female | 19 | - | - | - | 13 | - | - | 6 |
| | | | | | | | | |
| **50 - 54** | **32** | **3** | **7** | **-** | **13** | **1** | **1** | **7** |
| Male | 22 | 3 | 6 | - | 7 | - | - | 6 |
| Female | 10 | - | 1 | - | 6 | 1 | 1 | 1 |
| | | | | | | | | |
| **55 - 59** | **13** | **3** | **1** | **-** | **7** | **-** | **-** | **2** |
| Male | 10 | 1 | 1 | - | 7 | - | - | 1 |
| Female | 3 | 2 | - | - | - | - | - | 1 |
| | | | | | | | | |
| **60 - 64** | **16** | **2** | **1** | **-** | **7** | **2** | **1** | **3** |
| Male | 8 | 1 | - | - | 3 | 1 | 1 | 2 |
| Female | 8 | 1 | 1 | - | 4 | 1 | - | 1 |
| | | | | | | | | |
| **65+** | **27** | **1** | **6** | **0** | **13** | **3** | **0** | **4** |
| Male | 14 | 1 | 4 | 0 | 6 | 1 | 0 | 2 |
| Female | 13 | 0 | 2 | 0 | 7 | 2 | 0 | 2 |

## Table G6: Migrant Population by Purpose of Stay in Zambia, 5 Year Age Group, Sex and Province, Zambia 2010

| Age Group, Sex and Province | Purpose Of Stay | | | | | | | |
|---|---|---|---|---|---|---|---|---|
| | Total | Employment | Family Reunification/ Formation | Education/ Training | Settlement | Refuge/ Asylum | Investor | Other |
| **Lusaka Province** | | | | | | | | |
| **Total** | **17,007** | **4,319** | **4,391** | **709** | **3,543** | **1,998** | **711** | **1,336** |
| Male | 9,142 | 3,102 | 1,652 | 388 | 1,793 | 1,036 | 511 | 660 |
| Female | 7,865 | 1,217 | 2,739 | 321 | 1,750 | 962 | 200 | 676 |
| | | | | | | | | |
| **0 - 4** | **1,353** | **-** | **585** | **87** | **299** | **244** | **-** | **138** |
| Male | 682 | - | 303 | 44 | 154 | 111 | - | 70 |
| Female | 671 | - | 282 | 43 | 145 | 133 | - | 68 |
| | | | | | | | | |
| **5 - 9** | **1,216** | **-** | **520** | **112** | **271** | **217** | **-** | **96** |
| Male | 609 | - | 260 | 65 | 139 | 104 | - | 41 |
| Female | 607 | - | 260 | 47 | 132 | 113 | - | 55 |
| | | | | | | | | |
| **10 - 14** | **1,218** | **50** | **464** | **134** | **300** | **191** | **-** | **79** |
| Male | 615 | 29 | 240 | 70 | 145 | 90 | - | 41 |
| Female | 603 | 21 | 224 | 64 | 155 | 101 | - | 38 |
| | | | | | | | | |
| **15 - 19** | **1,043** | **80** | **322** | **131** | **242** | **179** | **15** | **74** |
| Male | 493 | 37 | 148 | 71 | 108 | 84 | 7 | 38 |
| Female | 550 | 43 | 174 | 60 | 134 | 95 | 8 | 36 |
| | | | | | | | | |
| **20 - 24** | **1,279** | **258** | **327** | **82** | **265** | **200** | **40** | **107** |
| Male | 584 | 154 | 106 | 41 | 112 | 97 | 26 | 48 |
| Female | 695 | 104 | 221 | 41 | 153 | 103 | 14 | 59 |
| | | | | | | | | |
| **25 - 29** | **1,785** | **570** | **396** | **48** | **334** | **183** | **86** | **168** |
| Male | 896 | 394 | 98 | 33 | 151 | 80 | 60 | 80 |
| Female | 889 | 176 | 298 | 15 | 183 | 103 | 26 | 88 |
| | | | | | | | | |
| **30 - 34** | **1,901** | **680** | **380** | **28** | **355** | **198** | **104** | **156** |
| Male | 1,011 | 485 | 76 | 20 | 179 | 90 | 69 | 92 |
| Female | 890 | 195 | 304 | 8 | 176 | 108 | 35 | 64 |
| | | | | | | | | |
| **35 - 39** | **1,768** | **722** | **316** | **18** | **298** | **192** | **100** | **122** |
| Male | 1,061 | 532 | 96 | 13 | 159 | 118 | 75 | 68 |
| Female | 707 | 190 | 220 | 5 | 139 | 74 | 25 | 54 |
| | | | | | | | | |
| **40 - 44** | **1,402** | **559** | **263** | **21** | **226** | **164** | **98** | **71** |
| Male | 881 | 430 | 72 | 10 | 132 | 114 | 79 | 44 |
| Female | 521 | 129 | 191 | 11 | 94 | 50 | 19 | 27 |
| | | | | | | | | |
| **45 - 49** | **1,030** | **432** | **187** | **17** | **175** | **83** | **74** | **62** |
| Male | 633 | 328 | 52 | 7 | 98 | 59 | 56 | 33 |
| Female | 397 | 104 | 135 | 10 | 77 | 24 | 18 | 29 |
| | | | | | | | | |
| **50 - 54** | **922** | **335** | **175** | **6** | **200** | **67** | **71** | **68** |
| Male | 548 | 254 | 58 | 4 | 110 | 43 | 51 | 28 |
| Female | 374 | 81 | 117 | 2 | 90 | 24 | 20 | 40 |

## Table G6: Migrant Population by Purpose of Stay in Zambia, 5 Year Age Group, Sex and Province, Zambia 2010

| Age Group, Sex and Province | Purpose Of Stay | | | | | | | |
|---|---|---|---|---|---|---|---|---|
| | Total | Employment | Family Reunification/ Formation | Education/ Training | Settlement | Refuge/ Asylum | Investor | Other |
| **55 - 59** | **664** | **243** | **136** | **11** | **147** | **34** | **43** | **50** |
| Male | 385 | 180 | 40 | 6 | 83 | 23 | 34 | 19 |
| Female | 279 | 63 | 96 | 5 | 64 | 11 | 9 | 31 |
| | | | | | | | | |
| **60 - 64** | **510** | **168** | **98** | **6** | **138** | **19** | **35** | **46** |
| Male | 256 | 113 | 29 | 2 | 66 | 8 | 24 | 14 |
| Female | 254 | 55 | 69 | 4 | 72 | 11 | 11 | 32 |
| | | | | | | | | |
| **65+** | **916** | **222** | **222** | **8** | **293** | **27** | **45** | **99** |
| Male | 488 | 166 | 74 | 2 | 157 | 15 | 30 | 44 |
| Female | 428 | 56 | 148 | 6 | 136 | 12 | 15 | 55 |
| **Northern Province** | | | | | | | | |
| **Total** | **1,436** | **110** | **355** | **28** | **703** | **58** | **24** | **158** |
| Male | 682 | 79 | 129 | 9 | 342 | 33 | 18 | 72 |
| Female | 754 | 31 | 226 | 19 | 361 | 25 | 6 | 86 |
| | | | | | | | | |
| **0 - 4** | **169** | **-** | **43** | **4** | **100** | **5** | **-** | **17** |
| Male | 90 | - | 21 | 2 | 55 | 4 | - | 8 |
| Female | 79 | - | 22 | 2 | 45 | 1 | - | 9 |
| | | | | | | | | |
| **5 - 9** | **134** | **-** | **38** | **5** | **73** | **6** | **-** | **12** |
| Male | 67 | - | 20 | - | 38 | 5 | - | 4 |
| Female | 67 | - | 18 | 5 | 35 | 1 | - | 8 |
| | | | | | | | | |
| **10 - 14** | **95** | **3** | **22** | **7** | **43** | **10** | **-** | **10** |
| Male | 45 | 1 | 10 | 3 | 22 | 7 | - | 2 |
| Female | 50 | 2 | 12 | 4 | 21 | 3 | - | 8 |
| | | | | | | | | |
| **15 - 19** | **109** | **5** | **34** | **2** | **53** | **3** | **1** | **11** |
| Male | 41 | 5 | 11 | 1 | 17 | 2 | 1 | 4 |
| Female | 68 | - | 23 | 1 | 36 | 1 | - | 7 |
| | | | | | | | | |
| **20 - 24** | **181** | **12** | **47** | **2** | **94** | **8** | **2** | **16** |
| Male | 58 | 8 | 8 | 1 | 31 | 4 | - | 6 |
| Female | 123 | 4 | 39 | 1 | 63 | 4 | 2 | 10 |
| | | | | | | | | |
| **25 - 29** | **204** | **15** | **45** | **5** | **107** | **2** | **3** | **27** |
| Male | 84 | 7 | 11 | 1 | 50 | - | 3 | 12 |
| Female | 120 | 8 | 34 | 4 | 57 | 2 | - | 15 |
| | | | | | | | | |
| **30 - 34** | **150** | **20** | **29** | **2** | **75** | **1** | **4** | **19** |
| Male | 86 | 16 | 14 | 1 | 40 | - | 3 | 12 |
| Female | 64 | 4 | 15 | 1 | 35 | 1 | 1 | 7 |
| | | | | | | | | |
| **35 - 39** | **108** | **11** | **16** | **1** | **64** | **4** | **1** | **11** |
| Male | 64 | 8 | 4 | - | 42 | 2 | 1 | 7 |
| Female | 44 | 3 | 12 | 1 | 22 | 2 | - | 4 |
| | | | | | | | | |
| **40 - 44** | **68** | **10** | **17** | **-** | **23** | **4** | **2** | **12** |
| Male | 32 | 6 | 5 | - | 13 | 1 | 1 | 6 |
| Female | 36 | 4 | 12 | - | 10 | 3 | 1 | 6 |

## Table G6: Migrant Population by Purpose of Stay in Zambia, 5 Year Age Group, Sex and Province, Zambia 2010

| Age Group, Sex and Province | Purpose Of Stay | | | | | | | |
|---|---|---|---|---|---|---|---|---|
| | Total | Employment | Family Reunification/ Formation | Education/ Training | Settlement | Refuge/ Asylum | Investor | Other |
| **45 - 49** | **66** | **12** | **16** | **-** | **19** | **6** | **5** | **8** |
| Male | 35 | 10 | 5 | - | 10 | 4 | 3 | 3 |
| Female | 31 | 2 | 11 | - | 9 | 2 | 2 | 5 |
| | | | | | | | | |
| **50 - 54** | **47** | **11** | **13** | **-** | **15** | **3** | **2** | **3** |
| Male | 24 | 10 | 4 | - | 5 | 2 | 2 | 1 |
| Female | 23 | 1 | 9 | - | 10 | 1 | - | 2 |
| | | | | | | | | |
| **55 - 59** | **29** | **6** | **8** | **-** | **10** | **2** | **1** | **2** |
| Male | 19 | 4 | 3 | - | 9 | 1 | 1 | 1 |
| Female | 10 | 2 | 5 | - | 1 | 1 | - | 1 |
| | | | | | | | | |
| **60 - 64** | **19** | **2** | **8** | **-** | **4** | **1** | **1** | **3** |
| Male | 8 | 1 | 4 | - | - | 1 | 1 | 1 |
| Female | 11 | 1 | 4 | - | 4 | - | - | 2 |
| | | | | | | | | |
| **65+** | **57** | **3** | **19** | **0** | **23** | **3** | **2** | **7** |
| Male | 29 | 3 | 9 | 0 | 10 | 0 | 2 | 5 |
| Female | 28 | 0 | 10 | 0 | 13 | 3 | 0 | 2 |
| **North Western Province** | | | | | | | | |
| **Total** | **7,562** | **360** | **288** | **34** | **172** | **6,557** | **8** | **143** |
| Male | 3,943 | 267 | 127 | 17 | 87 | 3,364 | 7 | 74 |
| Female | 3,619 | 93 | 161 | 17 | 85 | 3,193 | 1 | 69 |
| | | | | | | | | |
| **0 - 4** | **1,282** | **-** | **51** | **5** | **17** | **1,205** | **-** | **4** |
| Male | 637 | - | 25 | 2 | 8 | 601 | - | 1 |
| Female | 645 | - | 26 | 3 | 9 | 604 | - | 3 |
| | | | | | | | | |
| **5 - 9** | **1,170** | **-** | **45** | **6** | **20** | **1,093** | **-** | **6** |
| Male | 590 | - | 32 | 2 | 7 | 549 | - | - |
| Female | 580 | - | 13 | 4 | 13 | 544 | - | 6 |
| | | | | | | | | |
| **10 - 14** | **968** | **3** | **25** | **7** | **13** | **915** | **-** | **5** |
| Male | 465 | 1 | 14 | 5 | 9 | 434 | - | 2 |
| Female | 503 | 2 | 11 | 2 | 4 | 481 | - | 3 |
| | | | | | | | | |
| **15 - 19** | **666** | **1** | **27** | **3** | **14** | **618** | **-** | **3** |
| Male | 318 | - | 8 | 1 | 7 | 300 | - | 2 |
| Female | 348 | 1 | 19 | 2 | 7 | 318 | - | 1 |
| | | | | | | | | |
| **20 - 24** | **566** | **27** | **18** | **4** | **14** | **495** | **1** | **7** |
| Male | 267 | 16 | 6 | 1 | 6 | 234 | 1 | 3 |
| Female | 299 | 11 | 12 | 3 | 8 | 261 | - | 4 |
| | | | | | | | | |
| **25 - 29** | **455** | **48** | **27** | **3** | **4** | **351** | **1** | **21** |
| Male | 213 | 27 | 8 | 3 | 1 | 165 | 1 | 8 |
| Female | 242 | 21 | 19 | - | 3 | 186 | - | 13 |
| | | | | | | | | |
| **30 - 34** | **477** | **47** | **25** | **-** | **11** | **380** | **-** | **14** |
| Male | 235 | 37 | 6 | - | 6 | 178 | - | 8 |
| Female | 242 | 10 | 19 | - | 5 | 202 | - | 6 |

| Age Group, Sex and Province | Purpose Of Stay | | | | | | | |
|---|---|---|---|---|---|---|---|---|
| | Total | Employment | Family Reunification/ Formation | Education/ Training | Settlement | Refuge/ Asylum | Investor | Other |
| **35 - 39** | **507** | **61** | **20** | **2** | **17** | **379** | **2** | **26** |
| Male | 279 | 48 | 5 | 1 | 10 | 196 | 2 | 17 |
| Female | 228 | 13 | 15 | 1 | 7 | 183 | - | 9 |
| | | | | | | | | |
| **40 - 44** | **430** | **67** | **20** | **-** | **12** | **313** | **1** | **17** |
| Male | 268 | 54 | 10 | - | 7 | 183 | 1 | 13 |
| Female | 162 | 13 | 10 | - | 5 | 130 | - | 4 |
| | | | | | | | | |
| **45 - 49** | **322** | **47** | **11** | **-** | **11** | **242** | **2** | **9** |
| Male | 204 | 38 | 4 | - | 6 | 150 | 2 | 4 |
| Female | 118 | 9 | 7 | - | 5 | 92 | - | 5 |
| | | | | | | | | |
| **50 - 54** | **266** | **27** | **10** | **1** | **12** | **209** | **-** | **7** |
| Male | 168 | 24 | 4 | 1 | 6 | 128 | - | 5 |
| Female | 98 | 3 | 6 | - | 6 | 81 | - | 2 |
| | | | | | | | | |
| **55 - 59** | **149** | **16** | **3** | **-** | **12** | **112** | **-** | **6** |
| Male | 106 | 11 | 2 | - | 6 | 85 | - | 2 |
| Female | 43 | 5 | 1 | - | 6 | 27 | - | 4 |
| | | | | | | | | |
| **60 - 64** | **122** | **9** | **3** | **-** | **6** | **95** | **-** | **9** |
| Male | 78 | 7 | 2 | - | 4 | 60 | - | 5 |
| Female | 44 | 2 | 1 | - | 2 | 35 | - | 4 |
| | | | | | | | | |
| **65+** | **182** | **7** | **3** | **3** | **9** | **150** | **1** | **9** |
| Male | 115 | 4 | 1 | 1 | 4 | 101 | 0 | 4 |
| Female | 67 | 3 | 2 | 2 | 5 | 49 | 1 | 5 |
| **Southern Province** | | | | | | | | |
| **Total** | **1,660** | **567** | **397** | **47** | **258** | **27** | **109** | **255** |
| Male | 858 | 377 | 114 | 24 | 130 | 18 | 83 | 112 |
| Female | 802 | 190 | 283 | 23 | 128 | 9 | 26 | 143 |
| | | | | | | | | |
| **0 - 4** | **73** | **-** | **33** | **4** | **14** | **-** | **-** | **22** |
| Male | 35 | - | 15 | 1 | 7 | - | - | 12 |
| Female | 38 | - | 18 | 3 | 7 | - | - | 10 |
| | | | | | | | | |
| **5 - 9** | **92** | **-** | **42** | **7** | **19** | **8** | **-** | **16** |
| Male | 48 | - | 20 | 4 | 7 | 4 | - | 13 |
| Female | 44 | - | 22 | 3 | 12 | 4 | - | 3 |
| | | | | | | | | |
| **10 - 14** | **52** | **5** | **21** | **7** | **13** | **2** | **-** | **4** |
| Male | 24 | 4 | 7 | 3 | 8 | 2 | - | - |
| Female | 28 | 1 | 14 | 4 | 5 | - | - | 4 |
| | | | | | | | | |
| **15 - 19** | **41** | **9** | **15** | **4** | **7** | **1** | **-** | **5** |
| Male | 14 | 4 | 3 | 2 | 1 | 1 | - | 3 |
| Female | 27 | 5 | 12 | 2 | 6 | - | - | 2 |
| | | | | | | | | |
| **20 - 24** | **108** | **40** | **30** | **5** | **10** | **1** | **4** | **18** |
| Male | 51 | 25 | 11 | 3 | 6 | 1 | 4 | 1 |
| Female | 57 | 15 | 19 | 2 | 4 | - | - | 17 |

## Table G6: Migrant Population by Purpose of Stay in Zambia, 5 Year Age Group, Sex and Province, Zambia 2010

| Age Group, Sex and Province | Purpose Of Stay | | | | | | | |
|---|---|---|---|---|---|---|---|---|
| | Total | Employment | Family Reunification/ Formation | Education/ Training | Settlement | Refuge/ Asylum | Investor | Other |
| **25 - 29** | **162** | **70** | **37** | **7** | **19** | **-** | **5** | **24** |
| Male | 73 | 44 | 6 | 1 | 8 | - | 1 | 13 |
| Female | 89 | 26 | 31 | 6 | 11 | - | 4 | 11 |
| | | | | | | | | |
| **30 - 34** | **194** | **97** | **36** | **1** | **18** | **5** | **4** | **33** |
| Male | 96 | 58 | 6 | 1 | 8 | 3 | 3 | 17 |
| Female | 98 | 39 | 30 | - | 10 | 2 | 1 | 16 |
| | | | | | | | | |
| **35 - 39** | **159** | **82** | **27** | **-** | **25** | **2** | **7** | **16** |
| Male | 94 | 60 | 6 | - | 14 | 1 | 5 | 8 |
| Female | 65 | 22 | 21 | - | 11 | 1 | 2 | 8 |
| | | | | | | | | |
| **40 - 44** | **126** | **55** | **17** | **2** | **17** | **-** | **15** | **20** |
| Male | 67 | 38 | 2 | 2 | 9 | - | 11 | 5 |
| Female | 59 | 17 | 15 | - | 8 | - | 4 | 15 |
| | | | | | | | | |
| **45 - 49** | **127** | **53** | **9** | **-** | **9** | **3** | **24** | **29** |
| Male | 86 | 40 | 4 | - | 5 | 3 | 18 | 16 |
| Female | 41 | 13 | 5 | - | 4 | - | 6 | 13 |
| | | | | | | | | |
| **50 - 54** | **111** | **48** | **17** | **3** | **6** | **-** | **13** | **24** |
| Male | 68 | 36 | 5 | 2 | 3 | - | 10 | 12 |
| Female | 43 | 12 | 12 | 1 | 3 | - | 3 | 12 |
| | | | | | | | | |
| **55 - 59** | **101** | **34** | **23** | **2** | **15** | **-** | **18** | **9** |
| Male | 61 | 24 | 8 | 2 | 9 | - | 15 | 3 |
| Female | 40 | 10 | 15 | - | 6 | - | 3 | 6 |
| | | | | | | | | |
| **60 - 64** | **78** | **23** | **17** | **-** | **19** | **1** | **8** | **10** |
| Male | 35 | 13 | 3 | - | 10 | - | 7 | 2 |
| Female | 43 | 10 | 14 | - | 9 | 1 | 1 | 8 |
| | | | | | | | | |
| **65+** | **236** | **51** | **73** | **5** | **67** | **4** | **11** | **25** |
| Male | 106 | 31 | 18 | 3 | 35 | 3 | 9 | 7 |
| Female | 130 | 20 | 55 | 2 | 32 | 1 | 2 | 18 |
| **Western Province** | | | | | | | | |
| **Total** | **4,542** | **82** | **31** | **7** | **366** | **3,984** | **17** | **55** |
| Male | 2,304 | 52 | 18 | 5 | 216 | 1,966 | 15 | 32 |
| Female | 2,238 | 30 | 13 | 2 | 150 | 2,018 | 2 | 23 |
| | | | | | | | | |
| **0 - 4** | **463** | **-** | **4** | **1** | **34** | **419** | **-** | **5** |
| Male | 237 | - | 1 | 1 | 18 | 214 | - | 3 |
| Female | 226 | - | 3 | - | 16 | 205 | - | 2 |
| | | | | | | | | |
| **5 - 9** | **443** | **-** | **4** | **1** | **40** | **389** | **-** | **9** |
| Male | 222 | - | 3 | 1 | 21 | 192 | - | 5 |
| Female | 221 | - | 1 | - | 19 | 197 | - | 4 |
| | | | | | | | | |
| **10 - 14** | **713** | **-** | **3** | **4** | **50** | **654** | **-** | **2** |
| Male | 340 | - | 2 | 2 | 28 | 307 | - | 1 |
| Female | 373 | - | 1 | 2 | 22 | 347 | - | 1 |

## Table G6: Migrant Population by Purpose of Stay in Zambia, 5 Year Age Group, Sex and Province, Zambia 2010

| Age Group, Sex and Province | Purpose Of Stay | | | | | | | |
|---|---|---|---|---|---|---|---|---|
| | Total | Employment | Family Reunification/ Formation | Education/ Training | Settlement | Refuge/ Asylum | Investor | Other |
| **15 - 19** | **677** | **10** | **1** | **1** | **51** | **611** | **-** | **3** |
| Male | 289 | 2 | - | 1 | 26 | 258 | - | 2 |
| Female | 388 | 8 | 1 | - | 25 | 353 | - | 1 |
| | | | | | | | | |
| **20 - 24** | **347** | **5** | **2** | **-** | **36** | **297** | **3** | **4** |
| Male | 138 | 4 | 1 | - | 16 | 112 | 3 | 2 |
| Female | 209 | 1 | 1 | - | 20 | 185 | - | 2 |
| | | | | | | | | |
| **25 - 29** | **147** | **3** | **3** | **-** | **10** | **125** | **3** | **3** |
| Male | 44 | 2 | 1 | - | 6 | 30 | 3 | 2 |
| Female | 103 | 1 | 2 | - | 4 | 95 | - | 1 |
| | | | | | | | | |
| **30 - 34** | **214** | **14** | **-** | **-** | **22** | **174** | **1** | **3** |
| Male | 89 | 10 | - | - | 11 | 64 | 1 | 3 |
| Female | 125 | 4 | - | - | 11 | 110 | - | - |
| | | | | | | | | |
| **35 - 39** | **301** | **11** | **1** | **-** | **24** | **256** | **2** | **7** |
| Male | 120 | 7 | 1 | - | 14 | 92 | 2 | 4 |
| Female | 181 | 4 | - | - | 10 | 164 | - | 3 |
| | | | | | | | | |
| **40 - 44** | **326** | **10** | **3** | **-** | **27** | **282** | **2** | **2** |
| Male | 177 | 8 | 3 | - | 20 | 142 | 2 | 2 |
| Female | 149 | 2 | - | - | 7 | 140 | - | - |
| | | | | | | | | |
| **45 - 49** | **289** | **8** | **5** | **-** | **21** | **253** | **1** | **1** |
| Male | 183 | 7 | 4 | - | 16 | 154 | 1 | 1 |
| Female | 106 | 1 | 1 | - | 5 | 99 | - | - |
| | | | | | | | | |
| **50 - 54** | **174** | **6** | **-** | **-** | **16** | **148** | **2** | **2** |
| Male | 130 | 4 | - | - | 12 | 113 | - | 1 |
| Female | 44 | 2 | - | - | 4 | 35 | 2 | 1 |
| | | | | | | | | |
| **55 - 59** | **128** | **4** | **1** | **-** | **9** | **109** | **-** | **5** |
| Male | 96 | 3 | - | - | 9 | 81 | - | 3 |
| Female | 32 | 1 | 1 | - | - | 28 | - | 2 |
| | | | | | | | | |
| **60 - 64** | **111** | **3** | **1** | **-** | **11** | **91** | **3** | **2** |
| Male | 84 | 2 | - | - | 6 | 72 | 3 | 1 |
| Female | 27 | 1 | 1 | - | 5 | 19 | - | 1 |
| | | | | | | | | |
| **65+** | **209** | **8** | **3** | **0** | **15** | **176** | **0** | **7** |
| Male | 155 | 3 | 2 | 0 | 13 | 135 | 0 | 2 |
| Female | 54 | 5 | 1 | 0 | 2 | 41 | 0 | 5 |

## Table G7: Migrant Population (5 Years and Older) by 5 Year Age Group , Sex and Educational Attainment, Rural/Urban, Zambia 2010

| Age Group, Sex and Rural/Urban | Immigrant Population 5 Years and Older | Total Never Attended | Total Ever Attended School | | | | | | | | | | | | | | |
|---|---|---|---|---|---|---|---|---|---|---|---|---|---|---|---|---|---|
| | | | Total 1-7 | Primary | | | | | | | | | Secondary | | | | Tertiary |
| | | | | 1 | 2 | 3 | 4 | 5 | 6 | 7 | Total 8-12 | 8 | 9 | 10 | 11 | 12 | |
| **Zambia Total** | | | | | | | | | | | | | | | | | |
| **Total** | **39,706** | **6,665** | **11,852** | **1,212** | **1,451** | **1,461** | **1,733** | **1,636** | **1,803** | **2,556** | **10,082** | **1,406** | **1,800** | **1,244** | **956** | **4,676** | **11,107** |
| Male | 21,209 | 2,987 | 5,685 | 609 | 651 | 658 | 876 | 765 | 891 | 1,235 | 5,479 | 684 | 980 | 668 | 534 | 2,613 | 7,058 |
| Female | 18,497 | 3,678 | 6,167 | 603 | 800 | 803 | 857 | 871 | 912 | 1,321 | 4,603 | 722 | 820 | 576 | 422 | 2,063 | 4,049 |
| **5 - 9** | | | | | | | | | | | | | | | | | |
| **Total** | **3,823** | **1,840** | **1,983** | **882** | **593** | **329** | **143** | **36** | **-** | **-** | **-** | **-** | **-** | **-** | **-** | **-** | **-** |
| Male | 1,919 | 958 | 961 | 445 | 267 | 153 | 79 | 17 | - | - | - | - | - | - | - | - | - |
| Female | 1,904 | 882 | 1,022 | 437 | 326 | 176 | 64 | 19 | - | - | - | - | - | - | - | - | - |
| **10 - 14** | | | | | | | | | | | | | | | | | |
| **Total** | **3,760** | **424** | **3,006** | **148** | **380** | **496** | **612** | **571** | **464** | **335** | **330** | **206** | **94** | **30** | **-** | **-** | **-** |
| Male | 1,836 | 206 | 1,462 | 85 | 178 | 235 | 310 | 289 | 208 | 157 | 168 | 97 | 53 | 18 | - | - | - |
| Female | 1,924 | 218 | 1,544 | 63 | 202 | 261 | 302 | 282 | 256 | 178 | 162 | 109 | 41 | 12 | - | - | - |
| **15 - 19** | | | | | | | | | | | | | | | | | |
| **Total** | **3,119** | **395** | **1,168** | **10** | **48** | **80** | **136** | **240** | **288** | **366** | **1,487** | **372** | **327** | **278** | **244** | **266** | **69** |
| Male | 1,452 | 168 | 516 | 3 | 24 | 32 | 55 | 124 | 121 | 157 | 732 | 181 | 150 | 138 | 131 | 132 | 36 |
| Female | 1,667 | 227 | 652 | 7 | 24 | 48 | 81 | 116 | 167 | 209 | 755 | 191 | 177 | 140 | 113 | 134 | 33 |
| **20 - 24** | | | | | | | | | | | | | | | | | |
| **Total** | **3,199** | **367** | **774** | **12** | **35** | **50** | **85** | **121** | **177** | **294** | **1,446** | **179** | **262** | **144** | **188** | **673** | **612** |
| Male | 1,431 | 130 | 303 | 8 | 13 | 12 | 40 | 35 | 73 | 122 | 684 | 67 | 129 | 62 | 87 | 339 | 314 |
| Female | 1,768 | 237 | 471 | 4 | 22 | 38 | 45 | 86 | 104 | 172 | 762 | 112 | 133 | 82 | 101 | 334 | 298 |
| **25 - 29** | | | | | | | | | | | | | | | | | |
| **Total** | **3,705** | **378** | **677** | **7** | **37** | **40** | **77** | **85** | **137** | **294** | **1,370** | **146** | **216** | **152** | **114** | **742** | **1,280** |
| Male | 1,822 | 133 | 270 | 4 | 12 | 11 | 22 | 32 | 57 | 132 | 708 | 64 | 100 | 83 | 64 | 397 | 711 |
| Female | 1,883 | 245 | 407 | 3 | 25 | 29 | 55 | 53 | 80 | 162 | 662 | 82 | 116 | 69 | 50 | 345 | 569 |
| **30 - 34** | | | | | | | | | | | | | | | | | |
| **Total** | **3,935** | **372** | **647** | **11** | **36** | **50** | **73** | **93** | **130** | **254** | **1,261** | **129** | **199** | **164** | **113** | **656** | **1,655** |
| Male | 2,079 | 135 | 274 | - | 12 | 16 | 29 | 38 | 61 | 118 | 691 | 59 | 107 | 84 | 66 | 375 | 979 |
| Female | 1,856 | 237 | 373 | 11 | 24 | 34 | 44 | 55 | 69 | 136 | 570 | 70 | 92 | 80 | 47 | 281 | 676 |
| **35 - 39** | | | | | | | | | | | | | | | | | |
| **Total** | **3,838** | **444** | **623** | **19** | **35** | **50** | **85** | **92** | **104** | **238** | **1,087** | **109** | **188** | **130** | **92** | **568** | **1,684** |
| Male | 2,215 | 173 | 295 | 8 | 13 | 16 | 45 | 35 | 58 | 120 | 662 | 67 | 111 | 85 | 56 | 343 | 1,085 |
| Female | 1,623 | 271 | 328 | 11 | 22 | 34 | 40 | 57 | 46 | 118 | 425 | 42 | 77 | 45 | 36 | 225 | 599 |
| **40 - 44** | | | | | | | | | | | | | | | | | |
| **Total** | **3,224** | **399** | **525** | **13** | **44** | **42** | **89** | **69** | **108** | **160** | **856** | **70** | **126** | **101** | **64** | **495** | **1,444** |
| Male | 1,998 | 197 | 280 | 3 | 27 | 18 | 54 | 33 | 69 | 76 | 528 | 40 | 76 | 58 | 43 | 311 | 993 |
| Female | 1,226 | 202 | 245 | 10 | 17 | 24 | 35 | 36 | 39 | 84 | 328 | 30 | 50 | 43 | 21 | 184 | 451 |
| **45 - 49** | | | | | | | | | | | | | | | | | |
| **Total** | **2,574** | **338** | **434** | **11** | **38** | **51** | **91** | **65** | **62** | **116** | **615** | **52** | **99** | **71** | **48** | **345** | **1,187** |
| Male | 1,617 | 173 | 254 | 7 | 17 | 30 | 60 | 31 | 38 | 71 | 379 | 33 | 58 | 40 | 28 | 220 | 811 |
| Female | 957 | 165 | 180 | 4 | 21 | 21 | 31 | 34 | 24 | 45 | 236 | 19 | 41 | 31 | 20 | 125 | 376 |
| **50 - 54** | | | | | | | | | | | | | | | | | |
| **Total** | **2,284** | **323** | **429** | **8** | **28** | **53** | **75** | **52** | **78** | **135** | **510** | **39** | **71** | **68** | **37** | **295** | **1,022** |
| Male | 1,333 | 129 | 227 | 4 | 13 | 27 | 46 | 19 | 47 | 71 | 295 | 23 | 47 | 41 | 23 | 161 | 682 |
| Female | 951 | 194 | 202 | 4 | 15 | 26 | 29 | 33 | 31 | 64 | 215 | 16 | 24 | 27 | 14 | 134 | 340 |
| **55 - 59** | | | | | | | | | | | | | | | | | |
| **Total** | **1,731** | **251** | **360** | **10** | **32** | **38** | **67** | **50** | **49** | **114** | **343** | **23** | **67** | **33** | **20** | **200** | **777** |
| Male | 1,045 | 119 | 191 | 5 | 13 | 22 | 33 | 25 | 29 | 64 | 191 | 11 | 40 | 19 | 12 | 109 | 544 |
| Female | 686 | 132 | 169 | 5 | 19 | 16 | 34 | 25 | 20 | 50 | 152 | 12 | 27 | 14 | 8 | 91 | 233 |
| **60 - 64** | | | | | | | | | | | | | | | | | |
| **Total** | **1,433** | **254** | **312** | **17** | **36** | **42** | **54** | **50** | **48** | **65** | **263** | **24** | **41** | **29** | **12** | **157** | **604** |
| Male | 777 | 90 | 163 | 8 | 10 | 24 | 25 | 27 | 29 | 40 | 138 | 13 | 28 | 14 | 7 | 76 | 386 |
| Female | 656 | 164 | 149 | 9 | 26 | 18 | 29 | 23 | 19 | 25 | 125 | 11 | 13 | 15 | 5 | 81 | 218 |
| **65+** | | | | | | | | | | | | | | | | | |
| **Total** | **3,081** | **880** | **914** | **64** | **109** | **140** | **146** | **112** | **158** | **185** | **514** | **57** | **110** | **44** | **24** | **279** | **773** |
| Male | 1,685 | 376 | 489 | 29 | 52 | 62 | 78 | 60 | 101 | 107 | 303 | 29 | 81 | 26 | 17 | 150 | 517 |
| Female | 1,396 | 504 | 425 | 35 | 57 | 78 | 68 | 52 | 57 | 78 | 211 | 28 | 29 | 18 | 7 | 129 | 256 |

# Table G7: Migrant Population (5 Years and Older) by 5 Year Age Group , Sex and Educational Attainment, Rural, Zambia 2010

| Age Group, Sex and Rural/Urban | Immigrant Population 5 Years and Older | Total Ever Attended School | | | | | | | | | | | | | | | |
|---|---|---|---|---|---|---|---|---|---|---|---|---|---|---|---|---|---|
| | | Total Never Attended | Total 1-7 | Primary | | | | | | | Total 8-12 | Secondary | | | | | Tertiary |
| | | | | 1 | 2 | 3 | 4 | 5 | 6 | 7 | | 8 | 9 | 10 | 11 | 12 | |
| **Zambia Rural** | | | | | | | | | | | | | | | | | |
| **Total** | **15,632** | **4,536** | **6,789** | **714** | **905** | **961** | **1,111** | **969** | **999** | **1,130** | **2,533** | **620** | **628** | **286** | **290** | **709** | **1,774** |
| Male | 8,029 | 2,060 | 3,303 | 335 | 415 | 416 | 582 | 464 | 520 | 571 | 1,546 | 340 | 387 | 163 | 190 | 466 | 1,120 |
| Female | 7,603 | 2,476 | 3,486 | 379 | 490 | 545 | 529 | 505 | 479 | 559 | 987 | 280 | 241 | 123 | 100 | 243 | 654 |
| **5 - 9** | | | | | | | | | | | | | | | | | |
| **Total** | **1,993** | **1,126** | **867** | **471** | **243** | **112** | **37** | **4** | **-** | **-** | **-** | **-** | **-** | **-** | **-** | **-** | **-** |
| Male | 986 | 590 | 396 | 220 | 112 | 43 | 19 | 2 | - | - | - | - | - | - | - | - | - |
| Female | 1,007 | 536 | 471 | 251 | 131 | 69 | 18 | 2 | - | - | - | - | - | - | - | - | - |
| **10 - 14** | | | | | | | | | | | | | | | | | |
| **Total** | **2,045** | **345** | **1,651** | **119** | **313** | **354** | **356** | **264** | **157** | **88** | **49** | **37** | **10** | **2** | **-** | **-** | **-** |
| Male | 991 | 172 | 799 | 66 | 146 | 163 | 180 | 133 | 63 | 48 | 20 | 15 | 5 | - | - | - | - |
| Female | 1,054 | 173 | 852 | 53 | 167 | 191 | 176 | 131 | 94 | 40 | 29 | 22 | 5 | 2 | - | - | - |
| **15 - 19** | | | | | | | | | | | | | | | | | |
| **Total** | **1,615** | **305** | **873** | **7** | **42** | **74** | **114** | **202** | **219** | **215** | **433** | **196** | **109** | **57** | **42** | **29** | **4** |
| Male | 761 | 137 | 392 | 2 | 22 | 29 | 48 | 106 | 95 | 90 | 231 | 109 | 50 | 34 | 24 | 14 | 1 |
| Female | 854 | 168 | 481 | 5 | 20 | 45 | 66 | 96 | 124 | 125 | 202 | 87 | 59 | 23 | 18 | 15 | 3 |
| **20 - 24** | | | | | | | | | | | | | | | | | |
| **Total** | **1,280** | **243** | **487** | **11** | **27** | **37** | **58** | **85** | **119** | **150** | **434** | **92** | **121** | **46** | **69** | **106** | **116** |
| Male | 570 | 89 | 190 | 8 | 9 | 8 | 29 | 21 | 53 | 62 | 233 | 41 | 73 | 18 | 37 | 64 | 58 |
| Female | 710 | 154 | 297 | 3 | 18 | 29 | 29 | 64 | 66 | 88 | 201 | 51 | 48 | 28 | 32 | 42 | 58 |
| **25 - 29** | | | | | | | | | | | | | | | | | |
| **Total** | **948** | **210** | **290** | **5** | **25** | **23** | **49** | **41** | **54** | **93** | **285** | **59** | **59** | **30** | **27** | **110** | **163** |
| Male | 421 | 63 | 102 | 3 | 6 | 5 | 12 | 17 | 22 | 37 | 167 | 36 | 33 | 20 | 17 | 61 | 89 |
| Female | 527 | 147 | 188 | 2 | 19 | 18 | 37 | 24 | 32 | 56 | 118 | 23 | 26 | 10 | 10 | 49 | 74 |
| **30 - 34** | | | | | | | | | | | | | | | | | |
| **Total** | **1,003** | **246** | **312** | **5** | **27** | **32** | **43** | **58** | **67** | **80** | **251** | **44** | **51** | **29** | **42** | **85** | **194** |
| Male | 480 | 82 | 125 | - | 9 | 5 | 18 | 26 | 34 | 33 | 158 | 23 | 31 | 14 | 29 | 61 | 115 |
| Female | 523 | 164 | 187 | 5 | 18 | 27 | 25 | 32 | 33 | 47 | 93 | 21 | 20 | 15 | 13 | 24 | 79 |
| **35 - 39** | | | | | | | | | | | | | | | | | |
| **Total** | **1,136** | **310** | **351** | **12** | **26** | **39** | **65** | **57** | **57** | **95** | **225** | **53** | **62** | **20** | **24** | **66** | **250** |
| Male | 581 | 111 | 171 | 4 | 10 | 12 | 32 | 20 | 40 | 53 | 148 | 36 | 33 | 15 | 16 | 48 | 151 |
| Female | 555 | 199 | 180 | 8 | 16 | 27 | 33 | 37 | 17 | 42 | 77 | 17 | 29 | 5 | 8 | 18 | 99 |
| **40 - 44** | | | | | | | | | | | | | | | | | |
| **Total** | **1,084** | **282** | **360** | **10** | **33** | **36** | **79** | **44** | **80** | **78** | **200** | **34** | **48** | **24** | **23** | **71** | **242** |
| Male | 620 | 128 | 194 | 1 | 21 | 13 | 48 | 24 | 52 | 35 | 132 | 14 | 34 | 13 | 20 | 51 | 166 |
| Female | 464 | 154 | 166 | 9 | 12 | 23 | 31 | 20 | 28 | 43 | 68 | 20 | 14 | 11 | 3 | 20 | 76 |
| **45 - 49** | | | | | | | | | | | | | | | | | |
| **Total** | **939** | **256** | **304** | **7** | **31** | **43** | **73** | **48** | **43** | **59** | **170** | **29** | **36** | **12** | **26** | **67** | **209** |
| Male | 582 | 128 | 189 | 3 | 15 | 25 | 55 | 22 | 27 | 42 | 123 | 18 | 27 | 11 | 18 | 49 | 142 |
| Female | 357 | 128 | 115 | 4 | 16 | 18 | 18 | 26 | 16 | 17 | 47 | 11 | 9 | 1 | 8 | 18 | 67 |
| **50 - 54** | | | | | | | | | | | | | | | | | |
| **Total** | **849** | **239** | **278** | **5** | **22** | **42** | **55** | **35** | **43** | **76** | **147** | **22** | **28** | **21** | **17** | **59** | **185** |
| Male | 482 | 98 | 156 | 3 | 10 | 25 | 37 | 13 | 26 | 42 | 101 | 14 | 22 | 14 | 10 | 41 | 127 |
| Female | 367 | 141 | 122 | 2 | 12 | 17 | 18 | 22 | 17 | 34 | 46 | 8 | 6 | 7 | 7 | 18 | 58 |
| **55 - 59** | | | | | | | | | | | | | | | | | |
| **Total** | **662** | **178** | **245** | **7** | **26** | **30** | **50** | **32** | **32** | **68** | **102** | **10** | **32** | **17** | **7** | **36** | **137** |
| Male | 399 | 100 | 143 | 4 | 11 | 19 | 25 | 21 | 22 | 41 | 63 | 7 | 19 | 11 | 6 | 20 | 93 |
| Female | 263 | 78 | 102 | 3 | 15 | 11 | 25 | 11 | 10 | 27 | 39 | 3 | 13 | 6 | 1 | 16 | 44 |
| **60 - 64** | | | | | | | | | | | | | | | | | |
| **Total** | **567** | **179** | **206** | **13** | **24** | **34** | **34** | **36** | **30** | **35** | **77** | **14** | **21** | **12** | **4** | **26** | **105** |
| Male | 322 | 74 | 125 | 6 | 10 | 21 | 23 | 21 | 20 | 24 | 60 | 11 | 19 | 5 | 4 | 21 | 63 |
| Female | 245 | 105 | 81 | 7 | 14 | 13 | 11 | 15 | 10 | 11 | 17 | 3 | 2 | 7 | - | 5 | 42 |
| **65+** | | | | | | | | | | | | | | | | | |
| **Total** | **1,511** | **617** | **565** | **42** | **66** | **105** | **98** | **63** | **98** | **93** | **160** | **30** | **51** | **16** | **9** | **54** | **169** |
| Male | 834 | 288 | 321 | 15 | 34 | 48 | 56 | 38 | 66 | 64 | 110 | 16 | 41 | 8 | 9 | 36 | 115 |
| Female | 677 | 329 | 244 | 27 | 32 | 57 | 42 | 25 | 32 | 29 | 50 | 14 | 10 | 8 | - | 18 | 54 |

## Table G7: Migrant Population (5 Years and Older) by 5 Year Age Group , Sex and Educational Attainment, Urban, Zambia 2010

| Age Group, Sex and Rural/Urban | Immigrant Population 5 Years and Older | Total Ever Attended School | | | | | | | | | | | | | | | |
|---|---|---|---|---|---|---|---|---|---|---|---|---|---|---|---|---|---|
| | | | Primary | | | | | | | | | | Secondary | | | | |
| | | Total Never Attended | Total 1-7 | 1 | 2 | 3 | 4 | 5 | 6 | 7 | Total 8-12 | 8 | 9 | 10 | 11 | 12 | Tertiary |
| **Zambia Urban** | | | | | | | | | | | | | | | | | |
| **Total** | **24,074** | **2,129** | **5,063** | **498** | **546** | **500** | **622** | **667** | **804** | **1,426** | **7,549** | **786** | **1,172** | **958** | **666** | **3,967** | **9,333** |
| Male | 13,180 | 927 | 2,382 | 274 | 236 | 242 | 294 | 301 | 371 | 664 | 3,933 | 344 | 593 | 505 | 344 | 2,147 | 5,938 |
| Female | 10,894 | 1,202 | 2,681 | 224 | 310 | 258 | 328 | 366 | 433 | 762 | 3,616 | 442 | 579 | 453 | 322 | 1,820 | 3,395 |
| **5 - 9** | | | | | | | | | | | | | | | | | |
| **Total** | **1,830** | **714** | **1,116** | **411** | **350** | **217** | **106** | **32** | **-** | **-** | **-** | **-** | **-** | **-** | **-** | **-** | **-** |
| Male | 933 | 368 | 565 | 225 | 155 | 110 | 60 | 15 | - | - | - | - | - | - | - | - | - |
| Female | 897 | 346 | 551 | 186 | 195 | 107 | 46 | 17 | - | - | - | - | - | - | - | - | - |
| **10 - 14** | | | | | | | | | | | | | | | | | |
| **Total** | **1,715** | **79** | **1,355** | **29** | **67** | **142** | **256** | **307** | **307** | **247** | **281** | **169** | **84** | **28** | **-** | **-** | **-** |
| Male | 845 | 34 | 663 | 19 | 32 | 72 | 130 | 156 | 145 | 109 | 148 | 82 | 48 | 18 | - | - | - |
| Female | 870 | 45 | 692 | 10 | 35 | 70 | 126 | 151 | 162 | 138 | 133 | 87 | 36 | 10 | - | - | - |
| **15 - 19** | | | | | | | | | | | | | | | | | |
| **Total** | **1,504** | **90** | **295** | **3** | **6** | **6** | **22** | **38** | **69** | **151** | **1,054** | **176** | **218** | **221** | **202** | **237** | **65** |
| Male | 691 | 31 | 124 | 1 | 2 | 3 | 7 | 18 | 26 | 67 | 501 | 72 | 100 | 104 | 107 | 118 | 35 |
| Female | 813 | 59 | 171 | 2 | 4 | 3 | 15 | 20 | 43 | 84 | 553 | 104 | 118 | 117 | 95 | 119 | 30 |
| **20 - 24** | | | | | | | | | | | | | | | | | |
| **Total** | **1,919** | **124** | **287** | **1** | **8** | **13** | **27** | **36** | **58** | **144** | **1,012** | **87** | **141** | **98** | **119** | **567** | **496** |
| Male | 861 | 41 | 113 | - | 4 | 4 | 11 | 14 | 20 | 60 | 451 | 26 | 56 | 44 | 50 | 275 | 256 |
| Female | 1,058 | 83 | 174 | 1 | 4 | 9 | 16 | 22 | 38 | 84 | 561 | 61 | 85 | 54 | 69 | 292 | 240 |
| **25 - 29** | | | | | | | | | | | | | | | | | |
| **Total** | **2,757** | **168** | **387** | **2** | **12** | **17** | **28** | **44** | **83** | **201** | **1,085** | **87** | **157** | **122** | **87** | **632** | **1,117** |
| Male | 1,401 | 70 | 168 | 1 | 6 | 6 | 10 | 15 | 35 | 95 | 541 | 28 | 67 | 63 | 47 | 336 | 622 |
| Female | 1,356 | 98 | 219 | 1 | 6 | 11 | 18 | 29 | 48 | 106 | 544 | 59 | 90 | 59 | 40 | 296 | 495 |
| **30 - 34** | | | | | | | | | | | | | | | | | |
| **Total** | **2,932** | **126** | **335** | **6** | **9** | **18** | **30** | **35** | **63** | **174** | **1,010** | **85** | **148** | **135** | **71** | **571** | **1,461** |
| Male | 1,599 | 53 | 149 | - | 3 | 11 | 11 | 12 | 27 | 85 | 533 | 36 | 76 | 70 | 37 | 314 | 864 |
| Female | 1,333 | 73 | 186 | 6 | 6 | 7 | 19 | 23 | 36 | 89 | 477 | 49 | 72 | 65 | 34 | 257 | 597 |
| **35 - 39** | | | | | | | | | | | | | | | | | |
| **Total** | **2,702** | **134** | **272** | **7** | **9** | **11** | **20** | **35** | **47** | **143** | **862** | **56** | **126** | **110** | **68** | **502** | **1,434** |
| Male | 1,634 | 62 | 124 | 4 | 3 | 4 | 13 | 15 | 18 | 67 | 514 | 31 | 78 | 70 | 40 | 295 | 934 |
| Female | 1,068 | 72 | 148 | 3 | 6 | 7 | 7 | 20 | 29 | 76 | 348 | 25 | 48 | 40 | 28 | 207 | 500 |
| **40 - 44** | | | | | | | | | | | | | | | | | |
| **Total** | **2,140** | **117** | **165** | **3** | **11** | **6** | **10** | **25** | **28** | **82** | **656** | **36** | **78** | **77** | **41** | **424** | **1,202** |
| Male | 1,378 | 69 | 86 | 2 | 6 | 5 | 6 | 9 | 17 | 41 | 396 | 26 | 42 | 45 | 23 | 260 | 827 |
| Female | 762 | 48 | 79 | 1 | 5 | 1 | 4 | 16 | 11 | 41 | 260 | 10 | 36 | 32 | 18 | 164 | 375 |
| **45 - 49** | | | | | | | | | | | | | | | | | |
| **Total** | **1,635** | **82** | **130** | **4** | **7** | **8** | **18** | **17** | **19** | **57** | **445** | **23** | **63** | **59** | **22** | **278** | **978** |
| Male | 1,035 | 45 | 65 | 4 | 2 | 5 | 5 | 9 | 11 | 29 | 256 | 15 | 31 | 29 | 10 | 171 | 669 |
| Female | 600 | 37 | 65 | - | 5 | 3 | 13 | 8 | 8 | 28 | 189 | 8 | 32 | 30 | 12 | 107 | 309 |
| **50 - 54** | | | | | | | | | | | | | | | | | |
| **Total** | **1,435** | **84** | **151** | **3** | **6** | **11** | **20** | **17** | **35** | **59** | **363** | **17** | **43** | **47** | **20** | **236** | **837** |
| Male | 851 | 31 | 71 | 1 | 3 | 2 | 9 | 6 | 21 | 29 | 194 | 9 | 25 | 27 | 13 | 120 | 555 |
| Female | 584 | 53 | 80 | 2 | 3 | 9 | 11 | 11 | 14 | 30 | 169 | 8 | 18 | 20 | 7 | 116 | 282 |
| **55 - 59** | | | | | | | | | | | | | | | | | |
| **Total** | **1,069** | **73** | **115** | **3** | **6** | **8** | **17** | **18** | **17** | **46** | **241** | **13** | **35** | **16** | **13** | **164** | **640** |
| Male | 646 | 19 | 48 | 1 | 2 | 3 | 8 | 4 | 7 | 23 | 128 | 4 | 21 | 8 | 6 | 89 | 451 |
| Female | 423 | 54 | 67 | 2 | 4 | 5 | 9 | 14 | 10 | 23 | 113 | 9 | 14 | 8 | 7 | 75 | 189 |
| **60 - 64** | | | | | | | | | | | | | | | | | |
| **Total** | **866** | **75** | **106** | **4** | **12** | **8** | **20** | **14** | **18** | **30** | **186** | **10** | **20** | **17** | **8** | **131** | **499** |
| Male | 455 | 16 | 38 | 2 | - | 3 | 2 | 6 | 9 | 16 | 78 | 2 | 9 | 9 | 3 | 55 | 323 |
| Female | 411 | 59 | 68 | 2 | 12 | 5 | 18 | 8 | 9 | 14 | 108 | 8 | 11 | 8 | 5 | 76 | 176 |
| **65+** | | | | | | | | | | | | | | | | | |
| **Total** | **1,570** | **263** | **349** | **22** | **43** | **35** | **48** | **49** | **60** | **92** | **354** | **27** | **59** | **28** | **15** | **225** | **604** |
| Male | 851 | 88 | 168 | 14 | 18 | 14 | 22 | 22 | 35 | 43 | 193 | 13 | 40 | 18 | 8 | 114 | 402 |
| Female | 719 | 175 | 181 | 8 | 25 | 21 | 26 | 27 | 25 | 49 | 161 | 14 | 19 | 10 | 7 | 111 | 202 |

## Table G8: Migrant Population 12 Years and Older by Employment Status, 5 Year Age Groups and Sex, Rural/Urban, Zambia 2010

| Age Group, Sex and Rural/Urban | Migant Population 12 Years and Older | Employment Status | | | |
|---|---|---|---|---|---|
| | | An employer | An employee | Self employed | An unpaid family worker |
| **Zambia Total** | | | | | |
| **Total** | **19,952** | **1,202** | **8,044** | **7,609** | **3,097** |
| Male | 13,600 | 971 | 6,122 | 5,077 | 1,430 |
| Female | 6,352 | 231 | 1,922 | 2,532 | 1,667 |
| | | | | | |
| **12 - 14** | **199** | **-** | **14** | **51** | **134** |
| Male | 89 | - | 7 | 20 | 62 |
| Female | 110 | - | 7 | 31 | 72 |
| | | | | | |
| **15 - 19** | **587** | **1** | **94** | **168** | **324** |
| Male | 261 | 1 | 58 | 80 | 122 |
| Female | 326 | - | 36 | 88 | 202 |
| | | | | | |
| **20 - 24** | **1,422** | **20** | **525** | **467** | **410** |
| Male | 821 | 15 | 354 | 268 | 184 |
| Female | 601 | 5 | 171 | 199 | 226 |
| | | | | | |
| **25 - 29** | **2,146** | **84** | **1,021** | **771** | **270** |
| Male | 1,461 | 66 | 775 | 499 | 121 |
| Female | 685 | 18 | 246 | 272 | 149 |
| | | | | | |
| **30 - 34** | **2,629** | **134** | **1,303** | **925** | **267** |
| Male | 1,824 | 106 | 1,002 | 612 | 104 |
| Female | 805 | 28 | 301 | 313 | 163 |
| | | | | | |
| **35 - 39** | **2,816** | **178** | **1,292** | **1,049** | **297** |
| Male | 1,988 | 146 | 989 | 742 | 111 |
| Female | 828 | 32 | 303 | 307 | 186 |
| | | | | | |
| **40 - 44** | **2,503** | **192** | **1,094** | **921** | **296** |
| Male | 1,810 | 166 | 860 | 653 | 131 |
| Female | 693 | 26 | 234 | 268 | 165 |
| | | | | | |
| **45 - 49** | **1,954** | **129** | **868** | **733** | **224** |
| Male | 1,436 | 103 | 686 | 526 | 121 |
| Female | 518 | 26 | 182 | 207 | 103 |
| | | | | | |
| **50 - 54** | **1,717** | **147** | **686** | **663** | **221** |
| Male | 1,181 | 120 | 520 | 436 | 105 |
| Female | 536 | 27 | 166 | 227 | 116 |
| | | | | | |
| **55 - 59** | **1,288** | **110** | **493** | **524** | **161** |
| Male | 912 | 88 | 387 | 354 | 83 |
| Female | 376 | 22 | 106 | 170 | 78 |
| | | | | | |
| **60 - 64** | **997** | **69** | **327** | **455** | **146** |
| Male | 655 | 56 | 234 | 282 | 83 |
| Female | 342 | 13 | 93 | 173 | 63 |
| | | | | | |
| **65+** | **1694** | **138** | **327** | **882** | **347** |
| Male | 1162 | 104 | 250 | 605 | 203 |
| Female | 532 | 34 | 77 | 277 | 144 |

## Table G8: Migrant Population 12 Years and Older by Employment Status, 5 Year Age Groups and Sex, Rural, Zambia 2010

| Age Group, Sex and Rural/Urban | Migant Population 12 Years and Older | Employment Status | | | |
|---|---|---|---|---|---|
| | | An employer | An employee | Self employed | An unpaid family worker |
| **Zambia Rural** | | | | | |
| **Total** | **7,743** | **316** | **1,451** | **3,241** | **2,735** |
| Male | 4,511 | 247 | 970 | 2,033 | 1,261 |
| Female | 3,232 | 69 | 481 | 1,208 | 1,474 |
| | | | | | |
| **12 - 14** | **188** | **-** | **13** | **47** | **128** |
| Male | 82 | - | 6 | 17 | 59 |
| Female | 106 | - | 7 | 30 | 69 |
| | | | | | |
| **15 - 19** | **496** | **1** | **60** | **127** | **308** |
| Male | 206 | 1 | 33 | 57 | 115 |
| Female | 290 | - | 27 | 70 | 193 |
| | | | | | |
| **20 - 24** | **731** | **5** | **163** | **201** | **362** |
| Male | 347 | 4 | 91 | 90 | 162 |
| Female | 384 | 1 | 72 | 111 | 200 |
| | | | | | |
| **25 - 29** | **633** | **15** | **164** | **251** | **203** |
| Male | 342 | 10 | 103 | 143 | 86 |
| Female | 291 | 5 | 61 | 108 | 117 |
| | | | | | |
| **30 - 34** | **709** | **24** | **173** | **289** | **223** |
| Male | 397 | 18 | 130 | 169 | 80 |
| Female | 312 | 6 | 43 | 120 | 143 |
| | | | | | |
| **35 - 39** | **887** | **46** | **214** | **369** | **258** |
| Male | 500 | 40 | 129 | 237 | 94 |
| Female | 387 | 6 | 85 | 132 | 164 |
| | | | | | |
| **40 - 44** | **862** | **55** | **190** | **355** | **262** |
| Male | 530 | 46 | 131 | 238 | 115 |
| Female | 332 | 9 | 59 | 117 | 147 |
| | | | | | |
| **45 - 49** | **720** | **39** | **167** | **314** | **200** |
| Male | 484 | 32 | 126 | 218 | 108 |
| Female | 236 | 7 | 41 | 96 | 92 |
| | | | | | |
| **50 - 54** | **655** | **42** | **121** | **291** | **201** |
| Male | 408 | 36 | 87 | 187 | 98 |
| Female | 247 | 6 | 34 | 104 | 103 |
| | | | | | |
| **55 - 59** | **510** | **30** | **79** | **257** | **144** |
| Male | 337 | 21 | 61 | 177 | 78 |
| Female | 173 | 9 | 18 | 80 | 66 |
| | | | | | |
| **60 - 64** | **411** | **14** | **51** | **216** | **130** |
| Male | 266 | 11 | 31 | 145 | 79 |
| Female | 145 | 3 | 20 | 71 | 51 |
| | | | | | |
| **65+** | **941** | **45** | **56** | **524** | **316** |
| Male | 612 | 28 | 42 | 355 | 187 |
| Female | 329 | 17 | 14 | 169 | 129 |

## Table G8: Migrant Population 12 Years and Older by Employment Status, 5 Year Age Groups and Sex, Urban, Zambia 2010

| Age Group, Sex and Rural/Urban | Migant Population 12 Years and Older | Employment Status | | | |
|---|---|---|---|---|---|
| | | An employer | An employee | Self employed | An unpaid family worker |
| **Zambia Urban** | | | | | |
| **Total** | **12,209** | **886** | **6,593** | **4,368** | **362** |
| Male | 9,089 | 724 | 5,152 | 3,044 | 169 |
| Female | 3,120 | 162 | 1,441 | 1,324 | 193 |
| | | | | | |
| **12 - 14** | **11** | **-** | **1** | **4** | **6** |
| Male | 7 | - | 1 | 3 | 3 |
| Female | 4 | - | - | 1 | 3 |
| | | | | | |
| **15 - 19** | **91** | **-** | **34** | **41** | **16** |
| Male | 55 | - | 25 | 23 | 7 |
| Female | 36 | - | 9 | 18 | 9 |
| | | | | | |
| **20 - 24** | **691** | **15** | **362** | **266** | **48** |
| Male | 474 | 11 | 263 | 178 | 22 |
| Female | 217 | 4 | 99 | 88 | 26 |
| | | | | | |
| **25 - 29** | **1,513** | **69** | **857** | **520** | **67** |
| Male | 1,119 | 56 | 672 | 356 | 35 |
| Female | 394 | 13 | 185 | 164 | 32 |
| | | | | | |
| **30 - 34** | **1,920** | **110** | **1,130** | **636** | **44** |
| Male | 1,427 | 88 | 872 | 443 | 24 |
| Female | 493 | 22 | 258 | 193 | 20 |
| | | | | | |
| **35 - 39** | **1,929** | **132** | **1,078** | **680** | **39** |
| Male | 1,488 | 106 | 860 | 505 | 17 |
| Female | 441 | 26 | 218 | 175 | 22 |
| | | | | | |
| **40 - 44** | **1,641** | **137** | **904** | **566** | **34** |
| Male | 1,280 | 120 | 729 | 415 | 16 |
| Female | 361 | 17 | 175 | 151 | 18 |
| | | | | | |
| **45 - 49** | **1,234** | **90** | **701** | **419** | **24** |
| Male | 952 | 71 | 560 | 308 | 13 |
| Female | 282 | 19 | 141 | 111 | 11 |
| | | | | | |
| **50 - 54** | **1,062** | **105** | **565** | **372** | **20** |
| Male | 773 | 84 | 433 | 249 | 7 |
| Female | 289 | 21 | 132 | 123 | 13 |
| | | | | | |
| **55 - 59** | **778** | **80** | **414** | **267** | **17** |
| Male | 575 | 67 | 326 | 177 | 5 |
| Female | 203 | 13 | 88 | 90 | 12 |
| | | | | | |
| **60 - 64** | **586** | **55** | **276** | **239** | **16** |
| Male | 389 | 45 | 203 | 137 | 4 |
| Female | 197 | 10 | 73 | 102 | 12 |
| | | | | | |
| **65+** | **753** | **93** | **271** | **358** | **31** |
| Male | 550 | 76 | 208 | 250 | 16 |
| Female | 203 | 17 | 63 | 108 | 15 |

# Housing Tables

## Table H1: Households by Sex of Household Head and Province, Rural/Urban, Zambia 2010

| Province | Sex of the Household Head | | | | | | | | | | | |
|---|---|---|---|---|---|---|---|---|---|---|---|---|
| | Total | | | | Rural | | | | Urban | | | |
| | Total Households | Male | Female | Percent Female Headed Households | Total | Male | Female | Percent Female Headed Households | Total | Male | Female | Percent Female Headed Households |
| **Zambia Total** | **2,513,768** | **1,947,501** | **566,267** | **22.5** | **1,495,861** | **1,150,794** | **345,067** | **23.1** | **1,017,907** | **796,707** | **221,200** | **21.7** |
| Central | 235,560 | 185,348 | 50,212 | 21.3 | 170,714 | 136,077 | 34,637 | 20.3 | 64,846 | 49,271 | 15,575 | 24.0 |
| Copperbelt | 371,125 | 295,018 | 76,107 | 20.5 | 74,541 | 59,705 | 14,836 | 19.9 | 296,584 | 235,313 | 61,271 | 20.7 |
| Eastern | 305,198 | 236,449 | 68,749 | 22.5 | 265,783 | 205,425 | 60,358 | 22.7 | 39,415 | 31,024 | 8,391 | 21.3 |
| Luapula | 194,962 | 151,913 | 43,049 | 22.1 | 157,432 | 123,717 | 33,715 | 21.4 | 37,530 | 28,196 | 9,334 | 24.9 |
| Lusaka | 444,418 | 353,797 | 90,621 | 20.4 | 64,518 | 52,539 | 11,979 | 18.6 | 379,900 | 301,258 | 78,642 | 20.7 |
| Muchinga | 138,783 | 108,853 | 29,930 | 21.6 | 114,762 | 89,937 | 24,825 | 21.6 | 24,021 | 18,916 | 5,105 | 21.3 |
| Northern | 220,561 | 175,037 | 45,524 | 20.6 | 180,442 | 143,941 | 36,501 | 20.2 | 40,119 | 31,096 | 9,023 | 22.5 |
| North-Western | 130,803 | 99,754 | 31,049 | 23.7 | 100,566 | 76,492 | 24,074 | 23.9 | 30,237 | 23,262 | 6,975 | 23.1 |
| Southern | 292,179 | 220,606 | 71,573 | 24.5 | 211,077 | 158,582 | 52,495 | 24.9 | 81,102 | 62,024 | 19,078 | 23.5 |
| Western | 180,179 | 120,726 | 59,453 | 33.0 | 156,026 | 104,379 | 51,647 | 33.1 | 24,153 | 16,347 | 7,806 | 32.3 |

## Table H2: Percent Distribution of Housing Units by Occupancy Status, Rural/Urban and Province, Zambia 2010

| Province and Rural/Urban | Total Housing Units | Percent Total | Occupancy Status | | | | | | | | | | | |
|---|---|---|---|---|---|---|---|---|---|---|---|---|---|---|
| | | | Single Household | Percent | One Household in Several Housing Units | Percent | Shared | Percent | Vacant | Percent | Non-Contact | Percent | Non-Residential | Percent |
| **Zambia** | **2,801,382** | **100** | **2,269,486** | **81.0** | **213,431** | **7.6** | **11,952** | **0.4** | **115,161** | **4.1** | **50,959** | **1.8** | **140,393** | **5.0** |
| Rural | 1,670,983 | 100 | 1,341,262 | 80.3 | 146,453 | 8.8 | 3,087 | 0.2 | 74,592 | 4.5 | 32,792 | 2 | 72,797 | 4.4 |
| Urban | 1,130,399 | 100 | 928,224 | 82.1 | 66,978 | 5.9 | 8,865 | 0.8 | 40,569 | 3.6 | 18,167 | 1.6 | 67,596 | 6.0 |
| **Province** | | | | | | | | | | | | | | |
| **Total** | **2,801,382** | **100** | **2,269,486** | **81.0** | **213,431** | **7.6** | **11,952** | **0.4** | **115,161** | **4.1** | **50,959** | **1.8** | **140,393** | **5.0** |
| Central | 271,375 | 100 | 201,823 | 74.4 | 31,773 | 11.7 | 758 | 0.3 | 10,701 | 3.9 | 6,950 | 2.6 | 19,370 | 7.1 |
| Copperbelt | 420,460 | 100 | 339,748 | 80.8 | 20,345 | 4.8 | 4,723 | 1.1 | 17,813 | 4.2 | 9,328 | 2.2 | 28,503 | 6.8 |
| Eastern | 341,664 | 100 | 276,997 | 81.1 | 26,157 | 7.7 | 727 | 0.2 | 14,440 | 4.2 | 6,516 | 1.9 | 16,827 | 4.9 |
| Luapula | 243,053 | 100 | 189,125 | 77.8 | 4,802 | 2.0 | 250 | 0.1 | 28,631 | 11.8 | 8,615 | 3.5 | 11,630 | 4.8 |
| Lusaka | 492,363 | 100 | 398,870 | 81.0 | 38,005 | 7.7 | 2,598 | 0.5 | 17,870 | 3.6 | 6,674 | 1.4 | 28,346 | 5.8 |
| Muchinga | 153,975 | 100 | 131,609 | 85.5 | 6,757 | 4.4 | 142 | 0.1 | 5,863 | 3.8 | 2,572 | 1.7 | 7,032 | 4.6 |
| Northern | 240,076 | 100 | 214,001 | 89.1 | 5,845 | 2.4 | 235 | 0.1 | 8,949 | 3.7 | 3,879 | 1.6 | 7,167 | 3.0 |
| North Western | 142,166 | 100 | 113,235 | 79.6 | 16,824 | 11.8 | 312 | 0.2 | 3,783 | 2.7 | 2,057 | 1.4 | 5,955 | 4.2 |
| Southern | 306,598 | 100 | 251,238 | 81.9 | 36,431 | 11.9 | 1,854 | 0.6 | 3,859 | 1.3 | 2,533 | 0.8 | 10,683 | 3.5 |
| Western | 189,652 | 100 | 152,840 | 80.6 | 26,492 | 14.0 | 353 | 0.2 | 3,252 | 1.7 | 1,835 | 1 | 4,880 | 2.6 |

## Table H3:Number Residential Housing Units by Type of Housing Unit, Province and Rural/Urban, Zambia 2010

| Type of Housing Unit and Residential Housing Units | Total | Province | | | | | | | | | |
|---|---|---|---|---|---|---|---|---|---|---|---|
| | | Central | Copperbelt | Eastern | Luapula | Lusaka | Muchinga | Northern | North Western | Southern | Western |
| **Zambia** | | | | | | | | | | | |
| Residential Housing Units | 2,660,989 | 252,005 | 391,957 | 324,837 | 231,423 | 464,017 | 146,943 | 232,909 | 136,211 | 295,915 | 184,772 |
| Rural | 1,598,186 | 182,869 | 82,929 | 282,323 | 188,720 | 70,147 | 121,935 | 190,512 | 104,968 | 213,639 | 160,144 |
| Urban | 1,062,803 | 69,136 | 309,028 | 42,514 | 42,703 | 393,870 | 25,008 | 42,397 | 31,243 | 82,276 | 24,628 |
| **Traditional** | | | | | | | | | | | |
| Residential Housing Units | 1,177,414 | 131,414 | 61,837 | 202,800 | 147,874 | 26,818 | 101,586 | 151,316 | 78,361 | 128,234 | 147,174 |
| Rural | 1,075,076 | 119,861 | 48,588 | 198,605 | 126,819 | 25,083 | 93,332 | 130,395 | 70,545 | 121,244 | 140,604 |
| Urban | 102,338 | 11,553 | 13,249 | 4,195 | 21,055 | 1,735 | 8,254 | 20,921 | 7,816 | 6,990 | 6,570 |
| **Improved Traditional** | | | | | | | | | | | |
| Residential Housing Units | 575,640 | 70,945 | 102,417 | 70,800 | 67,249 | 32,761 | 31,167 | 61,713 | 40,176 | 78,812 | 19,600 |
| Rural | 387,681 | 46,578 | 24,826 | 60,238 | 55,086 | 18,694 | 23,866 | 53,336 | 27,556 | 65,230 | 12,271 |
| Urban | 187,959 | 24,367 | 77,591 | 10,562 | 12,163 | 14,067 | 7,301 | 8,377 | 12,620 | 13,582 | 7,329 |
| **Mixed** | | | | | | | | | | | |
| Residential Housing Units | 82,803 | 7,909 | 29,749 | 9,591 | 1,912 | 16,792 | 2,197 | 1,998 | 2,484 | 7,645 | 2,526 |
| Rural | 26,117 | 3,939 | 2,021 | 6,253 | 1,087 | 3,064 | 1,086 | 1,196 | 1,253 | 4,706 | 1,512 |
| Urban | 56,686 | 3,970 | 27,728 | 3,338 | 825 | 13,728 | 1,111 | 802 | 1,231 | 2,939 | 1,014 |
| **Conventional Flat** | | | | | | | | | | | |
| Residential Housing Units | 245,016 | 9,328 | 30,601 | 7,333 | 1,031 | 166,160 | 2,828 | 2,390 | 1,813 | 20,285 | 3,247 |
| Rural | 17,012 | 2,090 | 1,182 | 1,556 | 328 | 6,118 | 572 | 677 | 478 | 3,123 | 888 |
| Urban | 228,004 | 7,238 | 29,419 | 5,777 | 703 | 160,042 | 2,256 | 1,713 | 1,335 | 17,162 | 2,359 |
| **Conventional House** | | | | | | | | | | | |
| Residential Housing Units | 536,681 | 27,259 | 160,281 | 29,082 | 10,513 | 211,722 | 7,971 | 13,828 | 11,126 | 55,004 | 9,895 |
| Rural | 70,221 | 6,637 | 5,192 | 11,913 | 3,041 | 15,681 | 2,186 | 3,671 | 3,397 | 15,121 | 3,382 |
| Urban | 466,460 | 20,622 | 155,089 | 17,169 | 7,472 | 196,041 | 5,785 | 10,157 | 7,729 | 39,883 | 6,513 |
| **Mobile** | | | | | | | | | | | |
| Residential Housing Units | 1,650 | 125 | 142 | 57 | 48 | 295 | 24 | 34 | 385 | 376 | 164 |
| Rural | 1,096 | 86 | 25 | 34 | 29 | 97 | 22 | 16 | 370 | 284 | 133 |
| Urban | 554 | 39 | 117 | 23 | 19 | 198 | 2 | 18 | 15 | 92 | 31 |
| **Part of commercial building** | | | | | | | | | | | |
| Residential Housing Units | 10,288 | 1,018 | 1,096 | 3,290 | 291 | 2,404 | 380 | 275 | 169 | 993 | 372 |
| Rural | 4,993 | 622 | 234 | 2,601 | 150 | 259 | 193 | 141 | 94 | 523 | 176 |
| Urban | 5,295 | 396 | 862 | 689 | 141 | 2,145 | 187 | 134 | 75 | 470 | 196 |
| **Improvised/Makeshift** | | | | | | | | | | | |
| Residential Housing Units | 12,992 | 1,935 | 2,680 | 311 | 1,699 | 1,894 | 385 | 692 | 491 | 2,342 | 563 |
| Rural | 8,242 | 1,736 | 435 | 251 | 1,637 | 377 | 362 | 666 | 406 | 1,975 | 397 |
| Urban | 4,750 | 199 | 2,245 | 60 | 62 | 1,517 | 23 | 26 | 85 | 367 | 166 |
| **Collective/Institutional quarters** | | | | | | | | | | | |
| Residential Housing Units | 4,645 | 680 | 602 | 387 | 208 | 1,432 | 144 | 255 | 173 | 553 | 211 |
| Rural | 1,934 | 463 | 110 | 299 | 144 | 135 | 116 | 135 | 118 | 300 | 114 |
| Urban | 2,711 | 217 | 492 | 88 | 64 | 1,297 | 28 | 120 | 55 | 253 | 97 |
| **Unintended** | | | | | | | | | | | |
| Residential Housing Units | 5,190 | 469 | 963 | 593 | 150 | 1,501 | 152 | 203 | 241 | 744 | 174 |
| Rural | 2,171 | 277 | 147 | 264 | 104 | 347 | 113 | 131 | 178 | 485 | 125 |
| Urban | 3,019 | 192 | 816 | 329 | 46 | 1,154 | 39 | 72 | 63 | 259 | 49 |
| **Other** | | | | | | | | | | | |
| Residential Housing Units | 8,670 | 923 | 1,589 | 593 | 448 | 2,238 | 109 | 205 | 792 | 927 | 846 |
| Rural | 3,643 | 580 | 169 | 309 | 295 | 292 | 87 | 148 | 573 | 648 | 542 |
| Urban | 5,027 | 343 | 1,420 | 284 | 153 | 1,946 | 22 | 57 | 219 | 279 | 304 |

## Table H4: Number of Residential Housing Units, Households and Population (De Jure) by Construction Material of Walls and Roof, Rural/Urban, Zambia 2010

| Construction Material of Walls, Housing Units Households and Population | Total | Construction Material of Roof | | | | | | | | | | | | |
|---|---|---|---|---|---|---|---|---|---|---|---|---|---|---|
| | | Thatch/ Palm Leaf | Rustic Mat | Palm/ Bamboo | Wood Planks | Cardboard | Metal/Iron Sheets | Wood | Asbestos | Ceramic Tiles/ Harvey Tile | Cement | Roofing Shingles | Mud Tiles | Other |
| **Zambia Total** | | | | | | | | | | | | | | |
| Residential Housing Units | 2,660,989 | 1,344,408 | 4,727 | 7,537 | 6,213 | 1,158 | 831,880 | 10,233 | 408,899 | 6,906 | 4,587 | 1,259 | 213 | 32,969 |
| Households | 2,513,768 | 1,253,288 | 4,441 | 6,991 | 5,836 | 1,063 | 791,746 | 9,698 | 398,414 | 5,702 | 4,347 | 1,141 | 190 | 30,911 |
| Population | 13,092,666 | 6,454,900 | 21,036 | 35,032 | 29,320 | 4,893 | 4,162,438 | 49,568 | 2,128,424 | 27,487 | 20,397 | 5,743 | 872 | 152,556 |
| **Burnt bricks** | | | | | | | | | | | | | | |
| Residential Housing Units | 646,788 | 275,663 | 562 | 1,021 | 1,603 | 192 | 318,864 | 1,220 | 40,028 | 1,929 | 725 | 348 | 74 | 4,559 |
| Households | 609,545 | 257,753 | 504 | 941 | 1,498 | 176 | 302,376 | 1,174 | 38,144 | 1,669 | 684 | 315 | 67 | 4,244 |
| Population | 3,389,110 | 1,418,579 | 2,660 | 5,188 | 8,567 | 952 | 1,700,627 | 6,500 | 209,876 | 8,093 | 3,084 | 1,628 | 320 | 23,036 |
| **Mud bricks** | | | | | | | | | | | | | | |
| Residential Housing Units | 822,564 | 619,260 | 2,938 | 4,486 | 3,207 | 718 | 162,973 | 4,632 | 10,378 | 17 | 7 | 40 | 9 | 13,899 |
| Households | 761,659 | 569,897 | 2,758 | 4,136 | 2,999 | 659 | 154,072 | 4,357 | 9,883 | 17 | 7 | 38 | 9 | 12,827 |
| Population | 3,903,713 | 2,925,845 | 13,057 | 20,872 | 14,549 | 2,970 | 790,898 | 22,458 | 48,202 | 103 | 42 | 145 | 33 | 64,539 |
| **Compressed Mud** | | | | | | | | | | | | | | |
| Residential Housing Units | 260,163 | 236,035 | 428 | 1,145 | 409 | 38 | 15,791 | 2,093 | 778 | 1 | 1 | 8 | 1 | 3,435 |
| Households | 245,975 | 223,048 | 399 | 1,091 | 392 | 35 | 15,012 | 1,979 | 747 | 1 | 1 | 8 | 1 | 3,261 |
| Population | 1,248,607 | 1,131,075 | 1,965 | 5,299 | 1,869 | 165 | 78,102 | 10,220 | 3,691 | 10 | 3 | 43 | 3 | 16,162 |
| **Compressed Cement/Bricks** | | | | | | | | | | | | | | |
| Residential Housing Units | 68,346 | 203 | 16 | 8 | 72 | 6 | 35,083 | 14 | 31,645 | 603 | 269 | 106 | 38 | 283 |
| Households | 65,777 | 185 | 16 | 8 | 69 | 6 | 33,527 | 14 | 30,823 | 504 | 257 | 98 | 34 | 236 |
| Population | 347,935 | 936 | 77 | 36 | 378 | 36 | 171,832 | 88 | 169,447 | 2,405 | 1,147 | 448 | 147 | 958 |
| **Concrete blocks/slab** | | | | | | | | | | | | | | |
| Residential Housing Units | 141,508 | 147 | 6 | 6 | 31 | 8 | 59,809 | 13 | 78,695 | 1,410 | 1,053 | 186 | 21 | 123 |
| Households | 137,650 | 130 | 6 | 6 | 28 | 7 | 57,556 | 13 | 77,451 | 1,150 | 1,012 | 161 | 16 | 114 |
| Population | 723,205 | 608 | 22 | 34 | 151 | 40 | 290,899 | 61 | 420,092 | 5,300 | 4,555 | 799 | 92 | 552 |
| **Cement blocks** | | | | | | | | | | | | | | |
| Residential Housing Units | 475,881 | 430 | 25 | 6 | 93 | 34 | 222,557 | 45 | 246,066 | 2,916 | 2,519 | 558 | 63 | 569 |
| Households | 459,661 | 367 | 26 | 5 | 89 | 32 | 213,172 | 44 | 240,132 | 2,334 | 2,373 | 508 | 56 | 523 |
| Population | 2,350,263 | 1,855 | 117 | 24 | 485 | 146 | 1,048,261 | 206 | 1,270,995 | 11,433 | 11,488 | 2,612 | 242 | 2,399 |
| **Stone** | | | | | | | | | | | | | | |
| Residential Housing Units | 703 | 36 | - | 1 | 2 | 1 | 389 | - | 247 | 8 | 7 | 3 | 1 | 8 |
| Households | 657 | 31 | - | 1 | 2 | - | 367 | - | 231 | 8 | 7 | 3 | 1 | 6 |
| Population | 3,296 | 127 | - | 7 | 6 | - | 1,834 | - | 1,200 | 36 | 40 | 12 | 2 | 32 |
| **Iron sheets** | | | | | | | | | | | | | | |
| Residential Housing Units | 1,961 | 54 | 10 | 3 | 7 | 11 | 1,688 | 3 | 106 | - | 3 | 2 | 2 | 72 |
| Households | 1,769 | 52 | 10 | 2 | 6 | 5 | 1,525 | 2 | 103 | - | 3 | 2 | 2 | 57 |
| Population | 7,817 | 294 | 37 | 9 | 31 | 20 | 6,616 | 6 | 536 | - | 19 | 11 | 11 | 227 |
| **Asbestos/hardboard/wood** | | | | | | | | | | | | | | |
| Residential Housing Units | 5,375 | 4,076 | 29 | 20 | 228 | 33 | 553 | 119 | 182 | 1 | - | - | - | 134 |
| Households | 4,993 | 3,842 | 27 | 20 | 220 | 32 | 446 | 112 | 167 | - | - | - | - | 127 |
| Population | 23,859 | 18,886 | 103 | 98 | 886 | 117 | 1,859 | 471 | 851 | - | - | - | - | 588 |
| **Pole and dagga/mud** | | | | | | | | | | | | | | |
| Residential Housing Units | 174,275 | 157,743 | 185 | 540 | 341 | 34 | 10,416 | 1,414 | 221 | - | 1 | 5 | 2 | 3,373 |
| Households | 166,245 | 150,272 | 181 | 507 | 320 | 32 | 10,093 | 1,347 | 214 | - | 1 | 5 | 2 | 3,271 |
| Population | 818,466 | 736,781 | 809 | 2,294 | 1,460 | 130 | 53,256 | 6,472 | 959 | - | 8 | 31 | 13 | 16,253 |
| **Grass** | | | | | | | | | | | | | | |
| Residential Housing Units | 49,084 | 44,338 | 315 | 242 | 118 | 43 | 138 | 537 | 7 | - | - | - | - | 3,346 |
| Households | 46,154 | 41,568 | 308 | 219 | 114 | 41 | 135 | 520 | 7 | - | - | - | - | 3,242 |
| Population | 210,805 | 190,114 | 1,331 | 914 | 504 | 181 | 721 | 2,466 | 30 | - | - | - | - | 14,544 |
| **Other** | | | | | | | | | | | | | | |
| Residential Housing Units | 14,341 | 6,423 | 213 | 59 | 102 | 40 | 3,619 | 143 | 546 | 21 | 2 | 3 | 2 | 3,168 |
| Households | 13,683 | 6,143 | 206 | 55 | 99 | 38 | 3,465 | 136 | 512 | 19 | 2 | 3 | 2 | 3,003 |
| Population | 65,590 | 29,800 | 858 | 257 | 434 | 136 | 17,533 | 620 | 2,545 | 107 | 11 | 14 | 9 | 13,266 |

## Table H4: Number of Residential Housing Units, Households and Population (De Jure) by Construction Material of Walls and Roof, Rural, Zambia 2010

| Construction Material of Walls, Housing Units Households and Population | Total | Construction Material of Roof | | | | | | | | | | | | |
|---|---|---|---|---|---|---|---|---|---|---|---|---|---|---|
| | | Thatch/ Palm Leaf | Rustic Mat | Palm/ Bamboo | Wood Planks | Cardboard | Metal/Iron Sheets | Wood | Asbestos | Ceramic Tiles/ Harvey Tile | Cement | Roofing Shingles | Mud Tiles | Other |
| **Zambia Rural** | | | | | | | | | | | | | | |
| Residential Housing Units | 1,598,186 | 1,231,222 | 2,273 | 5,943 | 3,929 | 355 | 285,019 | 9,158 | 34,546 | 556 | 186 | 252 | 42 | 24,705 |
| Households | 1,495,861 | 1,150,696 | 2,134 | 5,551 | 3,694 | 318 | 268,431 | 8,701 | 32,126 | 435 | 161 | 227 | 38 | 23,349 |
| Population | 7,919,216 | 5,965,894 | 10,681 | 28,211 | 19,461 | 1,618 | 1,552,865 | 45,039 | 173,049 | 2,206 | 838 | 1,305 | 162 | 117,887 |
| **Burnt bricks** | | | | | | | | | | | | | | |
| Residential Housing Units | 446,822 | 259,465 | 348 | 807 | 1,290 | 111 | 170,812 | 1,130 | 9,032 | 52 | 76 | 104 | 13 | 3,582 |
| Households | 420,110 | 242,997 | 318 | 750 | 1,203 | 101 | 161,658 | 1,092 | 8,408 | 46 | 63 | 94 | 12 | 3,368 |
| Population | 2,396,017 | 1,343,760 | 1,713 | 4,236 | 7,095 | 594 | 965,533 | 6,100 | 46,934 | 277 | 315 | 566 | 62 | 18,832 |
| **Mud bricks** | | | | | | | | | | | | | | |
| Residential Housing Units | 618,483 | 535,709 | 1,133 | 3,316 | 1,732 | 116 | 60,711 | 3,819 | 2,664 | 4 | 1 | 10 | 6 | 9,262 |
| Households | 572,456 | 494,580 | 1,063 | 3,080 | 1,627 | 105 | 57,312 | 3,605 | 2,469 | 4 | 1 | 9 | 6 | 8,595 |
| Population | 2,999,569 | 2,569,602 | 5,485 | 15,837 | 8,092 | 542 | 322,802 | 19,107 | 12,966 | 28 | 1 | 42 | 25 | 45,040 |
| **Compressed Mud** | | | | | | | | | | | | | | |
| Residential Housing Units | 244,092 | 229,291 | 294 | 1,059 | 313 | 10 | 7,702 | 2,065 | 246 | - | - | 5 | 1 | 3,106 |
| Households | 230,887 | 216,828 | 269 | 1,011 | 301 | 9 | 7,318 | 1,955 | 233 | - | - | 5 | 1 | 2,957 |
| Population | 1,175,697 | 1,101,215 | 1,351 | 4,910 | 1,542 | 46 | 40,532 | 10,080 | 1,227 | - | - | 28 | 3 | 14,763 |
| **Compressed Cement/Bricks** | | | | | | | | | | | | | | |
| Residential Housing Units | 8,525 | 159 | 4 | 2 | 35 | 1 | 6,098 | 2 | 2,125 | 48 | 11 | 26 | 1 | 13 |
| Households | 7,819 | 141 | 4 | 2 | 32 | 1 | 5,577 | 2 | 1,980 | 31 | 9 | 26 | 1 | 13 |
| Population | 42,571 | 713 | 16 | 10 | 161 | 8 | 30,428 | 17 | 10,813 | 153 | 50 | 137 | 1 | 64 |
| **Concrete blocks/slab** | | | | | | | | | | | | | | |
| Residential Housing Units | 14,096 | 108 | 1 | 3 | 21 | - | 8,178 | 2 | 5,503 | 210 | 23 | 21 | 5 | 21 |
| Households | 13,021 | 93 | 1 | 2 | 20 | - | 7,520 | 2 | 5,171 | 153 | 22 | 15 | 2 | 20 |
| Population | 69,201 | 436 | 2 | 17 | 119 | - | 39,935 | 9 | 27,720 | 699 | 98 | 72 | 7 | 87 |
| **Cement blocks** | | | | | | | | | | | | | | |
| Residential Housing Units | 37,703 | 314 | 7 | 1 | 31 | 8 | 22,284 | 5 | 14,561 | 240 | 71 | 77 | 13 | 91 |
| Households | 34,453 | 280 | 8 | 1 | 28 | 7 | 20,208 | 4 | 13,490 | 199 | 62 | 69 | 13 | 84 |
| Population | 181,685 | 1,473 | 44 | 7 | 187 | 39 | 106,220 | 20 | 71,493 | 1,036 | 346 | 412 | 45 | 363 |
| **Stone** | | | | | | | | | | | | | | |
| Residential Housing Units | 205 | 30 | - | 1 | 2 | 1 | 126 | - | 39 | 1 | - | 2 | - | 3 |
| Households | 192 | 25 | - | 1 | 2 | - | 123 | - | 35 | 1 | - | 2 | - | 3 |
| Population | 982 | 92 | - | 7 | 6 | - | 687 | - | 169 | 7 | - | 6 | - | 8 |
| **Iron sheets** | | | | | | | | | | | | | | |
| Residential Housing Units | 1,011 | 49 | 6 | 2 | 4 | 9 | 898 | - | 17 | - | 2 | 1 | 2 | 21 |
| Households | 932 | 47 | 6 | 1 | 3 | 3 | 833 | - | 14 | - | 2 | 1 | 2 | 20 |
| Population | 4,271 | 269 | 23 | 4 | 14 | 12 | 3,755 | - | 81 | - | 13 | 5 | 11 | 84 |
| **Asbestos/hardboard/wood** | | | | | | | | | | | | | | |
| Residential Housing Units | 4,616 | 3,980 | 21 | 17 | 93 | 10 | 243 | 92 | 63 | - | - | - | - | 97 |
| Households | 4,342 | 3,753 | 18 | 17 | 90 | 9 | 225 | 86 | 53 | - | - | - | - | 91 |
| Population | 21,250 | 18,527 | 80 | 82 | 440 | 35 | 962 | 382 | 288 | - | - | - | - | 454 |
| **Pole and dagga/mud** | | | | | | | | | | | | | | |
| Residential Housing Units | 164,595 | 152,866 | 133 | 456 | 253 | 31 | 6,340 | 1,393 | 89 | - | 1 | 4 | 1 | 3,028 |
| Households | 156,957 | 145,649 | 130 | 431 | 238 | 29 | 6,122 | 1,327 | 84 | - | 1 | 4 | 1 | 2,941 |
| Population | 775,682 | 716,072 | 595 | 2,000 | 1,144 | 121 | 34,110 | 6,392 | 452 | - | 8 | 25 | 8 | 14,755 |
| **Grass** | | | | | | | | | | | | | | |
| Residential Housing Units | 47,229 | 43,053 | 202 | 229 | 107 | 34 | 113 | 520 | 6 | - | - | - | - | 2,965 |
| Households | 44,398 | 40,368 | 197 | 208 | 103 | 32 | 110 | 503 | 6 | - | - | - | - | 2,871 |
| Population | 203,175 | 184,918 | 859 | 881 | 454 | 143 | 603 | 2,356 | 28 | - | - | - | - | 12,933 |
| **Other** | | | | | | | | | | | | | | |
| Residential Housing Units | 10,809 | 6,198 | 124 | 50 | 48 | 24 | 1,514 | 130 | 201 | 1 | 1 | 2 | - | 2,516 |
| Households | 10,294 | 5,935 | 120 | 47 | 47 | 22 | 1,425 | 125 | 183 | 1 | 1 | 2 | - | 2,386 |
| Population | 49,116 | 28,817 | 513 | 220 | 207 | 78 | 7,298 | 576 | 878 | 6 | 7 | 12 | - | 10,504 |

## Table H4: Number of Residential Housing Units, Households and Population (De Jure) by Construction Material of Walls and Roof, Urban, Zambia 2010

| Construction Material of Walls, Housing Units Households and Population | Total | Construction Material of Roof | | | | | | | | | | | | |
|---|---|---|---|---|---|---|---|---|---|---|---|---|---|---|
| | | Thatch/ Palm Leaf | Rustic Mat | Palm/ Bamboo | Wood Planks | Cardboard | Metal/Iron Sheets | Wood | Asbestos | Ceramic Tiles/ Harvey Tile | Cement | Roofing Shingles | Mud Tiles | Other |
| **Zambia Urban** | | | | | | | | | | | | | | |
| Residential Housing Units | 1,062,803 | 113,186 | 2,454 | 1,594 | 2,284 | 803 | 546,861 | 1,075 | 374,353 | 6,350 | 4,401 | 1,007 | 171 | 8,264 |
| Households | 1,017,907 | 102,592 | 2,307 | 1,440 | 2,142 | 745 | 523,315 | 997 | 366,288 | 5,267 | 4,186 | 914 | 152 | 7,562 |
| Population | 5,173,450 | 489,006 | 10,355 | 6,821 | 9,859 | 3,275 | 2,609,573 | 4,529 | 1,955,375 | 25,281 | 19,559 | 4,438 | 710 | 34,669 |
| **Burnt bricks** | | | | | | | | | | | | | | |
| Residential Housing Units | 199,966 | 16,198 | 214 | 214 | 313 | 81 | 148,052 | 90 | 30,996 | 1,877 | 649 | 244 | 61 | 977 |
| Households | 189,435 | 14,756 | 186 | 191 | 295 | 75 | 140,718 | 82 | 29,736 | 1,623 | 621 | 221 | 55 | 876 |
| Population | 993,093 | 74,819 | 947 | 952 | 1,472 | 358 | 735,094 | 400 | 162,942 | 7,816 | 2,769 | 1,062 | 258 | 4,204 |
| **Mud bricks** | | | | | | | | | | | | | | |
| Residential Housing Units | 204,081 | 83,551 | 1,805 | 1,170 | 1,475 | 602 | 102,262 | 813 | 7,714 | 13 | 6 | 30 | 3 | 4,637 |
| Households | 189,203 | 75,317 | 1,695 | 1,056 | 1,372 | 554 | 96,760 | 752 | 7,414 | 13 | 6 | 29 | 3 | 4,232 |
| Population | 904,144 | 356,243 | 7,572 | 5,035 | 6,457 | 2,428 | 468,096 | 3,351 | 35,236 | 75 | 41 | 103 | 8 | 19,499 |
| **Compressed Mud** | | | | | | | | | | | | | | |
| Residential Housing Units | 16,071 | 6,744 | 134 | 86 | 96 | 28 | 8,089 | 28 | 532 | 1 | 1 | 3 | - | 329 |
| Households | 15,088 | 6,220 | 130 | 80 | 91 | 26 | 7,694 | 24 | 514 | 1 | 1 | 3 | - | 304 |
| Population | 72,910 | 29,860 | 614 | 389 | 327 | 119 | 37,570 | 140 | 2,464 | 10 | 3 | 15 | - | 1,399 |
| **Compressed Cement/Bricks** | | | | | | | | | | | | | | |
| Residential Housing Units | 59,821 | 44 | 12 | 6 | 37 | 5 | 28,985 | 12 | 29,520 | 555 | 258 | 80 | 37 | 270 |
| Households | 57,958 | 44 | 12 | 6 | 37 | 5 | 27,950 | 12 | 28,843 | 473 | 248 | 72 | 33 | 223 |
| Population | 305,364 | 223 | 61 | 26 | 217 | 28 | 141,404 | 71 | 158,634 | 2,252 | 1,097 | 311 | 146 | 894 |
| **Concrete blocks/slab** | | | | | | | | | | | | | | |
| Residential Housing Units | 127,412 | 39 | 5 | 3 | 10 | 8 | 51,631 | 11 | 73,192 | 1,200 | 1,030 | 165 | 16 | 102 |
| Households | 124,629 | 37 | 5 | 4 | 8 | 7 | 50,036 | 11 | 72,280 | 997 | 990 | 146 | 14 | 94 |
| Population | 654,004 | 172 | 20 | 17 | 32 | 40 | 250,964 | 52 | 392,372 | 4,601 | 4,457 | 727 | 85 | 465 |
| **Cement blocks** | | | | | | | | | | | | | | |
| Residential Housing Units | 438,178 | 116 | 18 | 5 | 62 | 26 | 200,273 | 40 | 231,505 | 2,676 | 2,448 | 481 | 50 | 478 |
| Households | 425,208 | 87 | 18 | 4 | 61 | 25 | 192,964 | 40 | 226,642 | 2,135 | 2,311 | 439 | 43 | 439 |
| Population | 2,168,578 | 382 | 73 | 17 | 298 | 107 | 942,041 | 186 | 1,199,502 | 10,397 | 11,142 | 2,200 | 197 | 2,036 |
| **Stone** | | | | | | | | | | | | | | |
| Residential Housing Units | 498 | 6 | - | - | - | - | 263 | - | 208 | 7 | 7 | 1 | 1 | 5 |
| Households | 465 | 6 | - | - | - | - | 244 | - | 196 | 7 | 7 | 1 | 1 | 3 |
| Population | 2,314 | 35 | - | - | - | - | 1,147 | - | 1,031 | 29 | 40 | 6 | 2 | 24 |
| **Iron sheets** | | | | | | | | | | | | | | |
| Residential Housing Units | 950 | 5 | 4 | 1 | 3 | 2 | 790 | 3 | 89 | - | 1 | 1 | - | 51 |
| Households | 837 | 5 | 4 | 1 | 3 | 2 | 692 | 2 | 89 | - | 1 | 1 | - | 37 |
| Population | 3,546 | 25 | 14 | 5 | 17 | 8 | 2,861 | 6 | 455 | - | 6 | 6 | - | 143 |
| **Asbestos/hardboard/wood** | | | | | | | | | | | | | | |
| Residential Housing Units | 759 | 96 | 8 | 3 | 135 | 23 | 310 | 27 | 119 | 1 | - | - | - | 37 |
| Households | 651 | 89 | 9 | 3 | 130 | 23 | 221 | 26 | 114 | - | - | - | - | 36 |
| Population | 2,609 | 359 | 23 | 16 | 446 | 82 | 897 | 89 | 563 | - | - | - | - | 134 |
| **Pole and dagga/mud** | | | | | | | | | | | | | | |
| Residential Housing Units | 9,680 | 4,877 | 52 | 84 | 88 | 3 | 4,076 | 21 | 132 | - | - | 1 | 1 | 345 |
| Households | 9,288 | 4,623 | 51 | 76 | 82 | 3 | 3,971 | 20 | 130 | - | - | 1 | 1 | 330 |
| Population | 42,784 | 20,709 | 214 | 294 | 316 | 9 | 19,146 | 80 | 507 | - | - | 6 | 5 | 1,498 |
| **Grass** | | | | | | | | | | | | | | |
| Residential Housing Units | 1,855 | 1,285 | 113 | 13 | 11 | 9 | 25 | 17 | 1 | - | - | - | - | 381 |
| Households | 1,756 | 1,200 | 111 | 11 | 11 | 9 | 25 | 17 | 1 | - | - | - | - | 371 |
| Population | 7,630 | 5,196 | 472 | 33 | 50 | 38 | 118 | 110 | 2 | - | - | - | - | 1,611 |
| **Other** | | | | | | | | | | | | | | |
| Residential Housing Units | 3,532 | 225 | 89 | 9 | 54 | 16 | 2,105 | 13 | 345 | 20 | 1 | 1 | 2 | 652 |
| Households | 3,389 | 208 | 86 | 8 | 52 | 16 | 2,040 | 11 | 329 | 18 | 1 | 1 | 2 | 617 |
| Population | 16,474 | 983 | 345 | 37 | 227 | 58 | 10,235 | 44 | 1,667 | 101 | 4 | 2 | 9 | 2,762 |

## Table H5: Number of Residential Housing Units, Households and Population (De Jure) by Construction Material of Walls and Floors, Rural/Urban, Zambia 2010

| Construction Material of Walls, Residential Housing Units and Population | Total | Construction Material of the Floor | | | | | | | | |
|---|---|---|---|---|---|---|---|---|---|---|
| | | Concrete | Cement | Brick | Tiles | Mud | Wood (not wooden tiles) | Marble | Terrazzo | Other |
| **Zambia Total** | | | | | | | | | | |
| Residential Housing Units | 2,660,989 | 196,580 | 858,891 | 7,825 | 50,884 | 1,511,414 | 3,000 | 430 | 885 | 31,080 |
| Households | 2,513,768 | 189,433 | 825,455 | 7,317 | 47,211 | 1,410,862 | 2,824 | 380 | 824 | 29,462 |
| Population | 13,092,666 | 1,006,534 | 4,339,971 | 38,864 | 248,514 | 7,299,262 | 14,065 | 1,878 | 4,276 | 139,302 |
| **Burnt bricks** | | | | | | | | | | |
| Residential Housing Units | 646,788 | 51,604 | 255,211 | 3,250 | 9,456 | 324,333 | 328 | 98 | 117 | 2,391 |
| Households | 609,545 | 49,076 | 241,455 | 3,059 | 8,737 | 304,566 | 305 | 77 | 109 | 2,161 |
| Population | 3,389,110 | 275,184 | 1,342,939 | 16,896 | 45,010 | 1,694,769 | 1,673 | 345 | 588 | 11,706 |
| **Mud bricks** | | | | | | | | | | |
| Residential Housing Units | 822,564 | 9,309 | 84,808 | 3,830 | 91 | 720,085 | 1,027 | 2 | - | 3,412 |
| Households | 761,659 | 8,928 | 80,917 | 3,552 | 87 | 664,046 | 950 | 1 | - | 3,178 |
| Population | 3,903,713 | 44,796 | 413,813 | 18,418 | 462 | 3,405,111 | 4,818 | 3 | - | 16,292 |
| **Compressed Mud** | | | | | | | | | | |
| Residential Housing Units | 260,163 | 900 | 8,375 | 263 | 24 | 248,085 | 319 | - | - | 2,197 |
| Households | 245,975 | 870 | 8,012 | 243 | 21 | 234,438 | 300 | - | - | 2,091 |
| Population | 1,248,607 | 4,382 | 41,710 | 1,201 | 109 | 1,189,584 | 1,479 | - | - | 10,142 |
| **Compressed Cement/Bricks** | | | | | | | | | | |
| Residential Housing Units | 68,346 | 14,464 | 48,160 | 68 | 4,737 | 465 | 3 | 33 | 63 | 353 |
| Households | 65,777 | 13,884 | 46,588 | 64 | 4,401 | 437 | 2 | 28 | 59 | 314 |
| Population | 347,935 | 73,675 | 246,596 | 312 | 23,124 | 2,264 | 9 | 139 | 314 | 1,502 |
| **Concrete blocks/slab** | | | | | | | | | | |
| Residential Housing Units | 141,508 | 45,310 | 83,570 | 48 | 11,284 | 477 | 9 | 94 | 237 | 479 |
| Households | 137,650 | 44,142 | 81,765 | 44 | 10,512 | 437 | 8 | 84 | 226 | 432 |
| Population | 723,205 | 233,897 | 428,252 | 270 | 55,038 | 2,185 | 32 | 425 | 1,136 | 1,970 |
| **Cement blocks** | | | | | | | | | | |
| Residential Housing Units | 475,881 | 73,966 | 372,732 | 200 | 25,127 | 1,742 | 10 | 199 | 450 | 1,455 |
| Households | 459,661 | 71,644 | 360,968 | 193 | 23,299 | 1,605 | 9 | 186 | 415 | 1,342 |
| Population | 2,350,263 | 370,669 | 1,837,011 | 957 | 124,021 | 7,978 | 48 | 958 | 2,149 | 6,472 |
| **Stone** | | | | | | | | | | |
| Residential Housing Units | 703 | 122 | 452 | 1 | 55 | 45 | - | 3 | 10 | 15 |
| Households | 657 | 112 | 428 | 1 | 50 | 44 | - | 3 | 7 | 12 |
| Population | 3,296 | 500 | 2,199 | 6 | 253 | 244 | - | 7 | 31 | 56 |
| **Iron sheets** | | | | | | | | | | |
| Residential Housing Units | 1,961 | 220 | 682 | 9 | - | 706 | 36 | - | - | 308 |
| Households | 1,769 | 204 | 626 | 7 | - | 630 | 23 | - | - | 279 |
| Population | 7,817 | 721 | 2,923 | 25 | - | 2,838 | 67 | - | - | 1,243 |
| **Asbestos/hardboard/wood** | | | | | | | | | | |
| Residential Housing Units | 5,375 | 177 | 323 | 6 | 7 | 4,438 | 221 | - | - | 203 |
| Households | 4,993 | 87 | 316 | 6 | 6 | 4,174 | 212 | - | - | 192 |
| Population | 23,859 | 366 | 1,387 | 29 | 31 | 20,142 | 1,071 | - | - | 833 |
| **Pole and dagga/mud** | | | | | | | | | | |
| Residential Housing Units | 174,275 | 185 | 2,847 | 93 | 15 | 165,315 | 743 | - | - | 5,077 |
| Households | 166,245 | 181 | 2,752 | 93 | 15 | 157,607 | 724 | - | - | 4,873 |
| Population | 818,466 | 846 | 14,534 | 466 | 69 | 775,842 | 3,514 | - | - | 23,195 |
| **Grass** | | | | | | | | | | |
| Residential Housing Units | 49,084 | 9 | 144 | 38 | 1 | 38,135 | 209 | - | - | 10,548 |
| Households | 46,154 | 9 | 141 | 37 | 1 | 35,636 | 202 | - | - | 10,128 |
| Population | 210,805 | 37 | 704 | 196 | 3 | 163,904 | 927 | - | - | 45,034 |
| **Other** | | | | | | | | | | |
| Residential Housing Units | 14,341 | 314 | 1,587 | 19 | 87 | 7,588 | 95 | 1 | 8 | 4,642 |
| Households | 13,683 | 296 | 1,487 | 18 | 82 | 7,242 | 89 | 1 | 8 | 4,460 |
| Population | 65,590 | 1,461 | 7,903 | 88 | 394 | 34,401 | 427 | 1 | 58 | 20,857 |

## Table H5: Number of Residential Housing Units, Households and Population (De Jure) by Construction Material of Walls and Floors, Rural, Zambia 2010

| Construction Material of Walls, Residential Housing Units and Population | Total | Construction Material of the Floor | | | | | | | | |
|---|---|---|---|---|---|---|---|---|---|---|
| | | Concrete | Cement | Brick | Tiles | Mud | Wood (not wooden tiles) | Marble | Terrazzo | Other |
| **Zambia Rural** | | | | | | | | | | |
| Residential Housing Units | 1,598,186 | 40,289 | 194,205 | 6,257 | 2,684 | 1,328,350 | 2,739 | 43 | 146 | 23,473 |
| Households | 1,495,861 | 37,681 | 181,740 | 5,870 | 2,348 | 1,243,129 | 2,589 | 32 | 129 | 22,343 |
| Population | 7,919,216 | 217,646 | 1,037,563 | 31,823 | 11,737 | 6,500,778 | 13,033 | 162 | 671 | 105,803 |
| **Burnt bricks** | | | | | | | | | | |
| Residential Housing Units | 446,822 | 23,784 | 118,580 | 2,767 | 411 | 299,409 | 309 | 16 | 33 | 1,513 |
| Households | 420,110 | 22,531 | 111,243 | 2,605 | 373 | 281,646 | 287 | 6 | 29 | 1,390 |
| Population | 2,396,017 | 137,126 | 654,592 | 14,603 | 1,934 | 1,578,004 | 1,595 | 30 | 164 | 7,969 |
| **Mud bricks** | | | | | | | | | | |
| Residential Housing Units | 618,483 | 2,237 | 25,754 | 3,065 | 36 | 584,161 | 896 | 1 | - | 2,333 |
| Households | 572,456 | 2,106 | 24,379 | 2,859 | 36 | 540,076 | 828 | - | - | 2,172 |
| Population | 2,999,569 | 12,097 | 136,391 | 15,066 | 183 | 2,820,093 | 4,228 | - | - | 11,511 |
| **Compressed Mud** | | | | | | | | | | |
| Residential Housing Units | 244,092 | 195 | 3,751 | 189 | 11 | 237,856 | 309 | - | - | 1,781 |
| Households | 230,887 | 185 | 3,594 | 174 | 11 | 224,943 | 290 | - | - | 1,690 |
| Population | 1,175,697 | 1,075 | 19,733 | 889 | 56 | 1,144,255 | 1,433 | - | - | 8,256 |
| **Compressed Cement/Bricks** | | | | | | | | | | |
| Residential Housing Units | 8,525 | 1,837 | 6,102 | 30 | 248 | 260 | - | 2 | 14 | 32 |
| Households | 7,819 | 1,694 | 5,595 | 28 | 216 | 244 | - | 2 | 11 | 29 |
| Population | 42,571 | 9,081 | 30,798 | 163 | 1,044 | 1,291 | - | 5 | 46 | 143 |
| **Concrete blocks/slab** | | | | | | | | | | |
| Residential Housing Units | 14,096 | 5,223 | 7,859 | 22 | 636 | 227 | - | 10 | 36 | 83 |
| Households | 13,021 | 4,804 | 7,316 | 21 | 561 | 200 | - | 10 | 33 | 76 |
| Population | 69,201 | 25,028 | 39,635 | 143 | 2,819 | 1,043 | - | 62 | 128 | 343 |
| **Cement blocks** | | | | | | | | | | |
| Residential Housing Units | 37,703 | 6,677 | 28,958 | 41 | 1,298 | 536 | 1 | 10 | 55 | 127 |
| Households | 34,453 | 6,045 | 26,578 | 41 | 1,108 | 499 | 1 | 10 | 50 | 121 |
| Population | 181,685 | 31,884 | 140,479 | 223 | 5,519 | 2,665 | 8 | 57 | 293 | 557 |
| **Stone** | | | | | | | | | | |
| Residential Housing Units | 205 | 35 | 116 | - | 7 | 29 | - | 3 | 2 | 13 |
| Households | 192 | 31 | 111 | - | 7 | 29 | - | 3 | - | 11 |
| Population | 982 | 134 | 611 | - | 26 | 153 | - | 7 | - | 51 |
| **Iron sheets** | | | | | | | | | | |
| Residential Housing Units | 1,011 | 104 | 417 | 1 | - | 378 | 5 | - | - | 106 |
| Households | 932 | 102 | 394 | 1 | - | 341 | 3 | - | - | 91 |
| Population | 4,271 | 339 | 1,917 | 1 | - | 1,615 | 12 | - | - | 387 |
| **Asbestos/hardboard/wood** | | | | | | | | | | |
| Residential Housing Units | 4,616 | 28 | 211 | 5 | 3 | 4,008 | 212 | - | - | 149 |
| Households | 4,342 | 20 | 205 | 5 | 3 | 3,761 | 204 | - | - | 144 |
| Population | 21,250 | 88 | 951 | 25 | 14 | 18,509 | 1,027 | - | - | 636 |
| **Pole and dagga/mud** | | | | | | | | | | |
| Residential Housing Units | 164,595 | 75 | 1,667 | 84 | 11 | 157,675 | 721 | - | - | 4,362 |
| Households | 156,957 | 73 | 1,604 | 84 | 11 | 150,302 | 702 | - | - | 4,181 |
| Population | 775,682 | 356 | 8,743 | 438 | 51 | 742,643 | 3,437 | - | - | 20,014 |
| **Grass** | | | | | | | | | | |
| Residential Housing Units | 47,229 | 3 | 101 | 35 | 1 | 37,058 | 203 | - | - | 9,828 |
| Households | 44,398 | 3 | 98 | 35 | 1 | 34,641 | 197 | - | - | 9,423 |
| Population | 203,175 | 10 | 492 | 191 | 3 | 159,608 | 911 | - | - | 41,960 |
| **Other** | | | | | | | | | | |
| Residential Housing Units | 10,809 | 91 | 689 | 18 | 22 | 6,753 | 83 | 1 | 6 | 3,146 |
| Households | 10,294 | 87 | 623 | 17 | 21 | 6,447 | 77 | 1 | 6 | 3,015 |
| Population | 49,116 | 428 | 3,221 | 81 | 88 | 30,899 | 382 | 1 | 40 | 13,976 |

## Table H5: Number of Residential Housing Units, Households and Population (De Jure) by Construction Material of Walls and Floors, Urban, Zambia 2010

| Construction Material of Walls, Residential Housing Units and Population | Total | Construction Material of the Floor | | | | | | | | |
|---|---|---|---|---|---|---|---|---|---|---|
| | | Concrete | Cement | Brick | Tiles | Mud | Wood (not wooden tiles) | Marble | Terrazzo | Other |
| **Zambia Urban** | | | | | | | | | | |
| Residential Housing Units | 1,062,803 | 156,291 | 664,686 | 1,568 | 48,200 | 183,064 | 261 | 387 | 739 | 7,607 |
| Households | 1,017,907 | 151,752 | 643,715 | 1,447 | 44,863 | 167,733 | 235 | 348 | 695 | 7,119 |
| Population | 5,173,450 | 788,888 | 3,302,408 | 7,041 | 236,777 | 798,484 | 1,032 | 1,716 | 3,605 | 33,499 |
| **Burnt bricks** | | | | | | | | | | |
| Residential Housing Units | 199,966 | 27,820 | 136,631 | 483 | 9,045 | 24,924 | 19 | 82 | 84 | 878 |
| Households | 189,435 | 26,545 | 130,212 | 454 | 8,364 | 22,920 | 18 | 71 | 80 | 771 |
| Population | 993,093 | 138,058 | 688,347 | 2,293 | 43,076 | 116,765 | 78 | 315 | 424 | 3,737 |
| **Mud bricks** | | | | | | | | | | |
| Residential Housing Units | 204,081 | 7,072 | 59,054 | 765 | 55 | 135,924 | 131 | 1 | - | 1,079 |
| Households | 189,203 | 6,822 | 56,538 | 693 | 51 | 123,970 | 122 | 1 | - | 1,006 |
| Population | 904,144 | 32,699 | 277,422 | 3,352 | 279 | 585,018 | 590 | 3 | - | 4,781 |
| **Compressed Mud** | | | | | | | | | | |
| Residential Housing Units | 16,071 | 705 | 4,624 | 74 | 13 | 10,229 | 10 | - | - | 416 |
| Households | 15,088 | 685 | 4,418 | 69 | 10 | 9,495 | 10 | - | - | 401 |
| Population | 72,910 | 3,307 | 21,977 | 312 | 53 | 45,329 | 46 | - | - | 1,886 |
| **Compressed Cement/Bricks** | | | | | | | | | | |
| Residential Housing Units | 59,821 | 12,627 | 42,058 | 38 | 4,489 | 205 | 3 | 31 | 49 | 321 |
| Households | 57,958 | 12,190 | 40,993 | 36 | 4,185 | 193 | 2 | 26 | 48 | 285 |
| Population | 305,364 | 64,594 | 215,798 | 149 | 22,080 | 973 | 9 | 134 | 268 | 1,359 |
| **Concrete blocks/slab** | | | | | | | | | | |
| Residential Housing Units | 127,412 | 40,087 | 75,711 | 26 | 10,648 | 250 | 9 | 84 | 201 | 396 |
| Households | 124,629 | 39,338 | 74,449 | 23 | 9,951 | 237 | 8 | 74 | 193 | 356 |
| Population | 654,004 | 208,869 | 388,617 | 127 | 52,219 | 1,142 | 32 | 363 | 1,008 | 1,627 |
| **Cement blocks** | | | | | | | | | | |
| Residential Housing Units | 438,178 | 67,289 | 343,774 | 159 | 23,829 | 1,206 | 9 | 189 | 395 | 1,328 |
| Households | 425,208 | 65,599 | 334,390 | 152 | 22,191 | 1,106 | 8 | 176 | 365 | 1,221 |
| Population | 2,168,578 | 338,785 | 1,696,532 | 734 | 118,502 | 5,313 | 40 | 901 | 1,856 | 5,915 |
| **Stone** | | | | | | | | | | |
| Residential Housing Units | 498 | 87 | 336 | 1 | 48 | 16 | - | - | 8 | 2 |
| Households | 465 | 81 | 317 | 1 | 43 | 15 | - | - | 7 | 1 |
| Population | 2,314 | 366 | 1,588 | 6 | 227 | 91 | - | - | 31 | 5 |
| **Iron sheets** | | | | | | | | | | |
| Residential Housing Units | 950 | 116 | 265 | 8 | - | 328 | 31 | - | - | 202 |
| Households | 837 | 102 | 232 | 6 | - | 289 | 20 | - | - | 188 |
| Population | 3,546 | 382 | 1,006 | 24 | - | 1,223 | 55 | - | - | 856 |
| **Asbestos/hardboard/wood** | | | | | | | | | | |
| Residential Housing Units | 759 | 149 | 112 | 1 | 4 | 430 | 9 | - | - | 54 |
| Households | 651 | 67 | 111 | 1 | 3 | 413 | 8 | - | - | 48 |
| Population | 2,609 | 278 | 436 | 4 | 17 | 1,633 | 44 | - | - | 197 |
| **Pole and dagga/mud** | | | | | | | | | | |
| Residential Housing Units | 9,680 | 110 | 1,180 | 9 | 4 | 7,640 | 22 | - | - | 715 |
| Households | 9,288 | 108 | 1,148 | 9 | 4 | 7,305 | 22 | - | - | 692 |
| Population | 42,784 | 490 | 5,791 | 28 | 18 | 33,199 | 77 | - | - | 3,181 |
| **Grass** | | | | | | | | | | |
| Residential Housing Units | 1,855 | 6 | 43 | 3 | - | 1,077 | 6 | - | - | 720 |
| Households | 1,756 | 6 | 43 | 2 | - | 995 | 5 | - | - | 705 |
| Population | 7,630 | 27 | 212 | 5 | - | 4,296 | 16 | - | - | 3,074 |
| **Other** | | | | | | | | | | |
| Residential Housing Units | 3,532 | 223 | 898 | 1 | 65 | 835 | 12 | - | 2 | 1,496 |
| Households | 3,389 | 209 | 864 | 1 | 61 | 795 | 12 | - | 2 | 1,445 |
| Population | 16,474 | 1,033 | 4,682 | 7 | 306 | 3,502 | 45 | - | 18 | 6,881 |

## Table H6i: Number of Households by Number of Living Rooms, Type of Occupancy and Rural/Urban, Zambia 2010

| Type of Occupancy and Rural/Urban | Total Households | Number of Living Rooms | | | | | | | | |
|---|---|---|---|---|---|---|---|---|---|---|
| | | 0 | 1 | 2 | 3 | 4 | 5 | 6 | 7 | 8+ |
| **Zambia Total** | | | | | | | | | | |
| **Total** | **2,513,768** | **331,560** | **2,015,337** | **124,741** | **26,900** | **8,573** | **2,949** | **1,158** | **777** | **1,773** |
| Rural | 1,495,861 | 222,575 | 1,177,319 | 70,004 | 16,874 | 5,411 | 1,617 | 601 | 421 | 1,039 |
| Urban | 1,017,907 | 108,985 | 838,018 | 54,737 | 10,026 | 3,162 | 1,332 | 557 | 356 | 734 |
| **Single Household** | | | | | | | | | | |
| **Total** | **2,272,694** | **294,898** | **1,830,591** | **110,362** | **23,583** | **7,499** | **2,558** | **975** | **673** | **1,555** |
| Rural | 1,342,706 | 198,469 | 1,061,579 | 60,522 | 14,480 | 4,627 | 1,337 | 465 | 346 | 881 |
| Urban | 929,988 | 96,429 | 769,012 | 49,840 | 9,103 | 2,872 | 1,221 | 510 | 327 | 674 |
| **One Household in Several Housing Units** | | | | | | | | | | |
| **Total** | **213,699** | **31,349** | **164,891** | **12,660** | **3,029** | **975** | **345** | **170** | **84** | **196** |
| Rural | 146,591 | 23,115 | 110,771 | 9,079 | 2,285 | 746 | 260 | 130 | 62 | 143 |
| Urban | 67,108 | 8,234 | 54,120 | 3,581 | 744 | 229 | 85 | 40 | 22 | 53 |
| **Shared** | | | | | | | | | | |
| **Total** | **27,375** | **5,313** | **19,855** | **1,719** | **288** | **99** | **46** | **13** | **20** | **22** |
| Rural | 6,564 | 991 | 4,969 | 403 | 109 | 38 | 20 | 6 | 13 | 15 |
| Urban | 20,811 | 4,322 | 14,886 | 1,316 | 179 | 61 | 26 | 7 | 7 | 7 |

## Table H6ii: Number of Household by Number of Bedrooms Rooms, Type of Occupancy and Rural/Urban, Zambia 2010

| Type of Occupancy and Rural/Urban | Total Households | Number of Bedrooms | | | | | | | |
|---|---|---|---|---|---|---|---|---|---|
| | | 1 | 2 | 3 | 4 | 5 | 6 | 7 | 8+ |
| **Zambia Total** | | | | | | | | | |
| **Total** | **2,513,768** | **1,228,590** | **758,684** | **391,424** | **99,265** | **22,271** | **7,027** | **2,535** | **3,972** |
| Rural | 1,495,861 | 713,239 | 492,009 | 212,013 | 56,044 | 13,642 | 4,478 | 1,685 | 2,751 |
| Urban | 1,017,907 | 515,351 | 266,675 | 179,411 | 43,221 | 8,629 | 2,549 | 850 | 1,221 |
| **Single Household** | | | | | | | | | |
| **Total** | **2,272,694** | **1,136,199** | **683,557** | **343,432** | **82,280** | **17,177** | **5,058** | **1,875** | **3,116** |
| Rural | 1,342,706 | 668,201 | 437,631 | 177,102 | 43,553 | 9,932 | 3,018 | 1,172 | 2,097 |
| Urban | 929,988 | 467,998 | 245,926 | 166,330 | 38,727 | 7,245 | 2,040 | 703 | 1,019 |
| **One Household in Several Housing Units** | | | | | | | | | |
| **Total** | **213,699** | **79,698** | **69,066** | **42,926** | **14,659** | **4,319** | **1,722** | **587** | **722** |
| Rural | 146,591 | 42,643 | 52,469 | 33,567 | 11,932 | 3,518 | 1,391 | 481 | 590 |
| Urban | 67,108 | 37,055 | 16,597 | 9,359 | 2,727 | 801 | 331 | 106 | 132 |
| **Shared** | | | | | | | | | |
| **Total** | **27,375** | **12,693** | **6,061** | **5,066** | **2,326** | **775** | **247** | **73** | **134** |
| Rural | 6,564 | 2,395 | 1,909 | 1,344 | 559 | 192 | 69 | 32 | 64 |
| Urban | 20,811 | 10,298 | 4,152 | 3,722 | 1,767 | 583 | 178 | 41 | 70 |

## Table H7: Number of Households by Household Size, Number of Living Rooms and Rural/Urban, Zambia 2010

| Household Size and Rural/Urban | Number of Households | Number of Living Rooms | | | | | | | | |
|---|---|---|---|---|---|---|---|---|---|---|
| | | 0 | 1 | 2 | 3 | 4 | 5 | 6 | 7 | 8+ |
| **Zambia Total** | **2,513,768** | **331,560** | **2,015,337** | **124,741** | **26,900** | **8,573** | **2,949** | **1,158** | **777** | **1773** |
| 1 | 163,630 | 49,055 | 108,829 | 4,348 | 920 | 254 | 77 | 37 | 31 | 79 |
| 2 | 225,661 | 47,633 | 167,834 | 7,821 | 1,567 | 436 | 152 | 61 | 46 | 111 |
| 3 | 320,669 | 55,882 | 249,732 | 11,510 | 2,354 | 676 | 183 | 81 | 67 | 184 |
| 4 | 371,193 | 51,566 | 300,078 | 14,850 | 3,122 | 928 | 269 | 81 | 88 | 211 |
| 5 | 368,344 | 41,547 | 304,523 | 16,826 | 3,555 | 1,096 | 344 | 130 | 87 | 236 |
| 6 | 324,706 | 30,471 | 271,531 | 17,081 | 3,697 | 1,109 | 351 | 159 | 86 | 221 |
| 7 | 260,447 | 21,552 | 218,036 | 15,623 | 3,330 | 1,094 | 394 | 118 | 89 | 211 |
| 8 | 280,640 | 20,006 | 233,150 | 20,017 | 4,594 | 1,633 | 614 | 235 | 129 | 262 |
| 9 | 62,281 | 4,663 | 51,646 | 4,512 | 915 | 304 | 109 | 40 | 33 | 59 |
| 10+ | 136,197 | 9,185 | 109,978 | 12,153 | 2,846 | 1,043 | 456 | 216 | 121 | 199 |
| | | | | | | | | | | |
| **Zambia Rural** | **1,495,861** | **222,575** | **1,177,319** | **70,004** | **16,874** | **5,411** | **1,617** | **601** | **421** | **1,039** |
| 1 | 89,281 | 24,584 | 61,234 | 2,584 | 584 | 158 | 54 | 16 | 17 | 50 |
| 2 | 127,634 | 25,849 | 95,522 | 4,771 | 998 | 288 | 84 | 31 | 23 | 68 |
| 3 | 185,627 | 33,946 | 142,539 | 6,884 | 1,566 | 418 | 94 | 45 | 37 | 98 |
| 4 | 217,257 | 35,310 | 170,738 | 8,344 | 1,935 | 565 | 156 | 37 | 48 | 124 |
| 5 | 219,119 | 31,137 | 175,364 | 9,315 | 2,215 | 667 | 174 | 73 | 38 | 136 |
| 6 | 196,938 | 24,441 | 159,857 | 9,301 | 2,237 | 673 | 171 | 79 | 44 | 135 |
| 7 | 161,273 | 18,106 | 131,445 | 8,539 | 2,044 | 709 | 220 | 53 | 42 | 115 |
| 8 | 181,672 | 17,465 | 148,119 | 11,390 | 2,950 | 1,058 | 331 | 130 | 75 | 154 |
| 9 | 36,231 | 3,947 | 29,156 | 2,305 | 533 | 172 | 52 | 15 | 20 | 31 |
| 10+ | 80,829 | 7,790 | 63,345 | 6,571 | 1,812 | 703 | 281 | 122 | 77 | 128 |
| | | | | | | | | | | |
| **Zambia Urban** | **1,017,907** | **108,985** | **838,018** | **54,737** | **10,026** | **3,162** | **1,332** | **557** | **356** | **734** |
| 1 | 74,349 | 24,471 | 47,595 | 1,764 | 336 | 96 | 23 | 21 | 14 | 29 |
| 2 | 98,027 | 21,784 | 72,312 | 3,050 | 569 | 148 | 68 | 30 | 23 | 43 |
| 3 | 135,042 | 21,936 | 107,193 | 4,626 | 788 | 258 | 89 | 36 | 30 | 86 |
| 4 | 153,936 | 16,256 | 129,340 | 6,506 | 1,187 | 363 | 113 | 44 | 40 | 87 |
| 5 | 149,225 | 10,410 | 129,159 | 7,511 | 1,340 | 429 | 170 | 57 | 49 | 100 |
| 6 | 127,768 | 6,030 | 111,674 | 7,780 | 1,460 | 436 | 180 | 80 | 42 | 86 |
| 7 | 99,174 | 3,446 | 86,591 | 7,084 | 1,286 | 385 | 174 | 65 | 47 | 96 |
| 8 | 98,968 | 2,541 | 85,031 | 8,627 | 1,644 | 575 | 283 | 105 | 54 | 108 |
| 9 | 26,050 | 716 | 22,490 | 2,207 | 382 | 132 | 57 | 25 | 13 | 28 |
| 10+ | 55,368 | 1,395 | 46,633 | 5,582 | 1,034 | 340 | 175 | 94 | 44 | 71 |

## Table H8: Number of Households by Household Size, Number of Bedrooms and Rural/Urban, Zambia 2010

| Household Size and Rural/Urban | Number of Households | Number of Bedrooms | | | | | | | |
|---|---|---|---|---|---|---|---|---|---|
| | | 1 | 2 | 3 | 4 | 5 | 6 | 7 | 8+ |
| **Zambia Total** | **2,513,768** | **1,228,590** | **758,684** | **391,424** | **99,265** | **22,271** | **7,027** | **2,535** | **3,972** |
| 1 | 163,630 | 130,029 | 22,167 | 8,405 | 2,043 | 511 | 178 | 94 | 203 |
| 2 | 225,661 | 158,944 | 47,082 | 14,970 | 3,277 | 765 | 243 | 108 | 272 |
| 3 | 320,669 | 212,471 | 77,840 | 23,742 | 4,757 | 1,020 | 331 | 155 | 353 |
| 4 | 371,193 | 216,555 | 108,806 | 36,221 | 7,303 | 1,325 | 408 | 149 | 426 |
| 5 | 368,344 | 182,323 | 125,039 | 48,699 | 9,306 | 1,872 | 501 | 189 | 415 |
| 6 | 324,706 | 132,054 | 120,372 | 57,378 | 11,711 | 2,073 | 575 | 193 | 350 |
| 7 | 260,447 | 85,314 | 98,544 | 59,568 | 13,289 | 2,478 | 664 | 238 | 352 |
| 8 | 280,640 | 70,387 | 100,468 | 79,975 | 22,650 | 4,800 | 1,370 | 423 | 567 |
| 9 | 62,281 | 14,330 | 20,901 | 19,409 | 5,811 | 1,260 | 357 | 100 | 113 |
| 10+ | 136,197 | 26,183 | 37,465 | 43,057 | 19,118 | 6,167 | 2,400 | 886 | 921 |
| | | | | | | | | | |
| **Zambia Rural** | **1,495,861** | **713,239** | **492,009** | **212,013** | **56,044** | **13,642** | **4,478** | **1,685** | **2,751** |
| 1 | 89,281 | 70,338 | 13,449 | 4,085 | 937 | 230 | 78 | 49 | 115 |
| 2 | 127,634 | 88,203 | 29,942 | 7,282 | 1,524 | 341 | 109 | 57 | 176 |
| 3 | 185,627 | 120,410 | 50,615 | 11,502 | 2,186 | 463 | 160 | 72 | 219 |
| 4 | 217,257 | 123,621 | 71,296 | 17,797 | 3,388 | 600 | 203 | 80 | 272 |
| 5 | 219,119 | 105,884 | 81,998 | 25,139 | 4,570 | 919 | 243 | 104 | 262 |
| 6 | 196,938 | 79,328 | 78,654 | 31,195 | 5,969 | 1,124 | 331 | 107 | 230 |
| 7 | 161,273 | 53,116 | 64,697 | 33,657 | 7,539 | 1,483 | 369 | 158 | 254 |
| 8 | 181,672 | 47,182 | 67,222 | 47,980 | 14,450 | 3,202 | 936 | 295 | 405 |
| 9 | 36,231 | 8,841 | 12,423 | 10,369 | 3,389 | 831 | 238 | 70 | 70 |
| 10+ | 80,829 | 16,316 | 21,713 | 23,007 | 12,092 | 4,449 | 1,811 | 693 | 748 |
| | | | | | | | | | |
| **Zambia Urban** | **1,017,907** | **515,351** | **266,675** | **179,411** | **43,221** | **8,629** | **2,549** | **850** | **1,221** |
| 1 | 74,349 | 59,691 | 8,718 | 4,320 | 1,106 | 281 | 100 | 45 | 88 |
| 2 | 98,027 | 70,741 | 17,140 | 7,688 | 1,753 | 424 | 134 | 51 | 96 |
| 3 | 135,042 | 92,061 | 27,225 | 12,240 | 2,571 | 557 | 171 | 83 | 134 |
| 4 | 153,936 | 92,934 | 37,510 | 18,424 | 3,915 | 725 | 205 | 69 | 154 |
| 5 | 149,225 | 76,439 | 43,041 | 23,560 | 4,736 | 953 | 258 | 85 | 153 |
| 6 | 127,768 | 52,726 | 41,718 | 26,183 | 5,742 | 949 | 244 | 86 | 120 |
| 7 | 99,174 | 32,198 | 33,847 | 25,911 | 5,750 | 995 | 295 | 80 | 98 |
| 8 | 98,968 | 23,205 | 33,246 | 31,995 | 8,200 | 1,598 | 434 | 128 | 162 |
| 9 | 26,050 | 5,489 | 8,478 | 9,040 | 2,422 | 429 | 119 | 30 | 43 |
| 10+ | 55,368 | 9,867 | 15,752 | 20,050 | 7,026 | 1,718 | 589 | 193 | 173 |

## Table H9 : Number of Housing Units, Households and Population (DeJure) by Type of Housing Unit and Source of Energy for Lighting, Rural/Urban, Zambia 2010

| Source of Energy for Lighting, Housing Units, Households and Population | Occupied Housing Units | Type of Housing Unit | | | | | | | | | | |
|---|---|---|---|---|---|---|---|---|---|---|---|---|
| | | Traditional | Improved Traditional | Mixed | Conventional Flat | Conventional House | Mobile | Part of commercial building | Improvised/ Makeshift | Collective/ Institutional quarters | Unintended | Other |
| **Zambia Total** | | | | | | | | | | | | |
| Housing Units | 2,494,869 | 1,096,115 | 538,669 | 78,743 | 232,473 | 510,076 | 1,523 | 9,426 | 12,070 | 3,606 | 4,753 | 7,415 |
| Households | 2,513,768 | 1,098,990 | 541,234 | 79,462 | 235,161 | 519,586 | 1,539 | 9,552 | 12,204 | 3,670 | 4,850 | 7,520 |
| Population | 13,092,666 | 5,612,328 | 2,939,264 | 425,807 | 1,084,776 | 2,854,130 | 6,729 | 44,387 | 54,404 | 15,413 | 19,606 | 35,822 |
| **Electricity** | | | | | | | | | | | | |
| Housing Units | 545,309 | 4,073 | 33,663 | 26,507 | 146,870 | 321,546 | 383 | 4,070 | 1,478 | 1,931 | 1,690 | 3,098 |
| Households | 553,438 | 4,110 | 33,958 | 26,861 | 148,548 | 327,082 | 389 | 4,139 | 1,516 | 1,959 | 1,742 | 3,134 |
| Population | 2,932,625 | 19,996 | 173,601 | 147,440 | 703,975 | 1,830,273 | 1,750 | 18,596 | 7,011 | 7,941 | 6,701 | 15,341 |
| **Gas** | | | | | | | | | | | | |
| Housing Units | 3,289 | 2,022 | 756 | 100 | 92 | 283 | 2 | 10 | 11 | 4 | 5 | 4 |
| Households | 3,308 | 2,026 | 763 | 101 | 97 | 283 | 2 | 11 | 12 | 4 | 5 | 4 |
| Population | 17,629 | 10,474 | 4,286 | 661 | 460 | 1,587 | 6 | 44 | 63 | 7 | 18 | 23 |
| **Wood** | | | | | | | | | | | | |
| Housing Units | 40,706 | 30,786 | 8,061 | 298 | 166 | 432 | 19 | 29 | 706 | 16 | 54 | 139 |
| Households | 40,796 | 30,838 | 8,083 | 300 | 170 | 441 | 19 | 29 | 706 | 17 | 54 | 139 |
| Population | 182,488 | 136,222 | 37,708 | 1,412 | 766 | 2,321 | 67 | 121 | 3,041 | 64 | 191 | 575 |
| **Candle** | | | | | | | | | | | | |
| Housing Units | 691,064 | 237,470 | 190,506 | 31,658 | 75,771 | 143,548 | 478 | 2,534 | 4,252 | 889 | 1,810 | 2,148 |
| Households | 697,501 | 238,069 | 191,587 | 31,913 | 76,630 | 147,000 | 482 | 2,565 | 4,325 | 905 | 1,838 | 2,187 |
| Population | 3,474,786 | 1,190,581 | 982,864 | 160,595 | 332,179 | 755,583 | 2,047 | 11,435 | 18,447 | 3,647 | 7,342 | 10,066 |
| **Paraffin** | | | | | | | | | | | | |
| Housing Units | 507,565 | 350,220 | 132,913 | 7,354 | 2,631 | 11,356 | 96 | 565 | 1,542 | 147 | 274 | 467 |
| Households | 509,144 | 351,103 | 133,380 | 7,391 | 2,682 | 11,469 | 99 | 570 | 1,550 | 152 | 275 | 473 |
| Population | 2,622,345 | 1,769,402 | 721,048 | 40,243 | 12,952 | 64,518 | 398 | 2,747 | 6,986 | 664 | 1,232 | 2,155 |
| **Solar** | | | | | | | | | | | | |
| Housing Units | 72,320 | 24,575 | 28,854 | 2,638 | 1,666 | 12,995 | 62 | 643 | 221 | 305 | 166 | 195 |
| Households | 72,650 | 24,645 | 28,976 | 2,652 | 1,683 | 13,087 | 62 | 651 | 222 | 305 | 170 | 197 |
| Population | 445,132 | 141,941 | 184,843 | 16,770 | 9,494 | 82,939 | 315 | 3,837 | 1,338 | 1,719 | 840 | 1,096 |
| **Bio Fuel** | | | | | | | | | | | | |
| Housing Units | 2,797 | 1,464 | 775 | 61 | 96 | 369 | 2 | 9 | 9 | 5 | 2 | 5 |
| Households | 2,810 | 1,470 | 777 | 61 | 96 | 373 | 2 | 10 | 9 | 5 | 2 | 5 |
| Population | 15,566 | 7,837 | 4,539 | 362 | 451 | 2,208 | 13 | 43 | 51 | 37 | 8 | 17 |
| **Diesel** | | | | | | | | | | | | |
| Housing Units | 54,284 | 38,014 | 13,748 | 780 | 240 | 1,104 | 29 | 45 | 220 | 14 | 39 | 51 |
| Households | 54,508 | 38,135 | 13,827 | 786 | 243 | 1,115 | 29 | 46 | 221 | 16 | 39 | 51 |
| Population | 299,430 | 204,489 | 80,190 | 4,674 | 1,255 | 6,844 | 150 | 212 | 1,109 | 55 | 203 | 249 |
| **None** | | | | | | | | | | | | |
| Housing Units | 30,432 | 24,689 | 4,139 | 450 | 122 | 487 | 54 | 33 | 283 | 7 | 60 | 108 |
| Households | 30,519 | 24,749 | 4,157 | 451 | 122 | 493 | 54 | 33 | 283 | 8 | 60 | 109 |
| Population | 136,081 | 108,965 | 20,062 | 2,052 | 498 | 2,398 | 209 | 118 | 1,053 | 34 | 245 | 447 |
| **Other** | | | | | | | | | | | | |
| Housing Units | 547,103 | 382,802 | 125,254 | 8,897 | 4,819 | 17,956 | 398 | 1,488 | 3,348 | 288 | 653 | 1,200 |
| Households | 549,094 | 383,845 | 125,726 | 8,946 | 4,890 | 18,243 | 401 | 1,498 | 3,360 | 299 | 665 | 1,221 |
| Population | 2,966,584 | 2,022,421 | 730,123 | 51,598 | 22,746 | 105,459 | 1,774 | 7,234 | 15,305 | 1,245 | 2,826 | 5,853 |

## Table H9 : Number of Housing Units, Households and Population (DeJure) by Type of Housing Unit and Source of Energy for Lighting, Rural/Urban, Zambia 2010

| Source of Energy for Lighting, Housing Units, Households and Population | Occupied Housing Units | Type of Housing Unit | | | | | | | | | | |
|---|---|---|---|---|---|---|---|---|---|---|---|---|
| | | Traditional | Improved Traditional | Mixed | Conventional Flat | Conventional House | Mobile | Part of commercial building | Improvised/ Makeshift | Collective/ Institutional quarters | Unintended | Other |
| **Zambia Rural** | | | | | | | | | | | | |
| Housing Units | 1,490,802 | 1,003,283 | 362,906 | 24,630 | 15,474 | 64,855 | 1,023 | 4,496 | 7,654 | 1,359 | 2,005 | 3,117 |
| Households | 1,495,861 | 1,005,889 | 364,257 | 24,744 | 15,731 | 65,436 | 1,028 | 4,529 | 7,680 | 1,391 | 2,032 | 3,144 |
| Population | 7,919,216 | 5,172,528 | 2,061,570 | 140,370 | 77,297 | 377,287 | 4,288 | 22,010 | 34,472 | 6,150 | 8,408 | 14,836 |
| **Electricity** | | | | | | | | | | | | |
| Housing Units | 45,680 | 2,962 | 10,118 | 2,189 | 7,656 | 20,542 | 96 | 679 | 229 | 310 | 294 | 605 |
| Households | 46,098 | 2,978 | 10,189 | 2,200 | 7,779 | 20,714 | 96 | 690 | 229 | 317 | 300 | 606 |
| Population | 241,967 | 14,724 | 54,931 | 11,947 | 37,286 | 113,190 | 263 | 3,142 | 1,106 | 1,292 | 1,125 | 2,961 |
| **Gas** | | | | | | | | | | | | |
| Housing Units | 2,573 | 1,805 | 609 | 65 | 14 | 61 | 2 | 7 | 5 | 1 | 4 | 0 |
| Households | 2,588 | 1,809 | 614 | 65 | 19 | 61 | 2 | 7 | 6 | 1 | 4 | 0 |
| Population | 13,850 | 9,406 | 3,471 | 448 | 90 | 351 | 6 | 27 | 33 | 2 | 16 | 0 |
| **Wood** | | | | | | | | | | | | |
| Housing Units | 38,963 | 30,090 | 7,496 | 234 | 55 | 178 | 18 | 21 | 683 | 11 | 48 | 129 |
| Households | 39,037 | 30,139 | 7,517 | 236 | 55 | 179 | 18 | 21 | 683 | 12 | 48 | 129 |
| Population | 174,424 | 133,230 | 34,943 | 1,094 | 255 | 1,033 | 66 | 92 | 2,943 | 47 | 178 | 543 |
| **Candle** | | | | | | | | | | | | |
| Housing Units | 315,465 | 197,624 | 84,677 | 7,135 | 3,873 | 17,136 | 313 | 1,376 | 1,663 | 387 | 664 | 617 |
| Households | 316,620 | 198,122 | 85,005 | 7,163 | 3,946 | 17,325 | 314 | 1,383 | 1,673 | 394 | 671 | 624 |
| Population | 1,644,818 | 1,007,577 | 465,001 | 38,728 | 18,054 | 93,887 | 1,340 | 6,282 | 7,003 | 1,609 | 2,572 | 2,765 |
| **Paraffin** | | | | | | | | | | | | |
| Housing Units | 434,196 | 314,247 | 105,941 | 4,849 | 935 | 5,746 | 85 | 453 | 1,251 | 95 | 212 | 382 |
| Households | 435,400 | 315,034 | 106,264 | 4,872 | 950 | 5,783 | 87 | 456 | 1,256 | 100 | 212 | 386 |
| Population | 2,259,713 | 1,597,589 | 583,904 | 27,153 | 5,249 | 34,424 | 353 | 2,225 | 5,675 | 415 | 952 | 1,774 |
| **Solar** | | | | | | | | | | | | |
| Housing Units | 64,797 | 23,373 | 26,219 | 2,205 | 1,083 | 10,476 | 56 | 585 | 175 | 291 | 156 | 178 |
| Households | 65,077 | 23,441 | 26,327 | 2,216 | 1,093 | 10,546 | 56 | 592 | 175 | 291 | 160 | 180 |
| Population | 401,683 | 135,361 | 169,527 | 14,241 | 6,485 | 67,733 | 278 | 3,538 | 1,083 | 1,647 | 792 | 998 |
| **Bio Fuel** | | | | | | | | | | | | |
| Housing Units | 2,174 | 1,367 | 578 | 37 | 25 | 143 | 2 | 8 | 7 | 4 | 1 | 2 |
| Households | 2,179 | 1,372 | 578 | 37 | 25 | 143 | 2 | 8 | 7 | 4 | 1 | 2 |
| Population | 12,176 | 7,341 | 3,439 | 227 | 137 | 896 | 13 | 41 | 41 | 34 | 2 | 5 |
| **Diesel** | | | | | | | | | | | | |
| Housing Units | 52,441 | 37,169 | 13,099 | 724 | 181 | 897 | 27 | 42 | 208 | 14 | 34 | 46 |
| Households | 52,646 | 37,284 | 13,168 | 730 | 183 | 906 | 27 | 43 | 209 | 16 | 34 | 46 |
| Population | 290,097 | 200,413 | 76,895 | 4,373 | 958 | 5,610 | 137 | 195 | 1,045 | 55 | 187 | 229 |
| **None** | | | | | | | | | | | | |
| Housing Units | 28,812 | 24,042 | 3,689 | 394 | 39 | 172 | 51 | 25 | 246 | 6 | 52 | 96 |
| Households | 28,893 | 24,100 | 3,706 | 395 | 39 | 176 | 51 | 25 | 246 | 7 | 52 | 96 |
| Population | 129,473 | 106,507 | 18,182 | 1,805 | 144 | 933 | 196 | 98 | 949 | 32 | 220 | 407 |
| **Other** | | | | | | | | | | | | |
| Housing Units | 505,701 | 370,604 | 110,480 | 6,798 | 1,613 | 9,504 | 373 | 1,300 | 3,187 | 240 | 540 | 1,062 |
| Households | 507,323 | 371,610 | 110,889 | 6,830 | 1,642 | 9,603 | 375 | 1,304 | 3,196 | 249 | 550 | 1,075 |
| Population | 2,751,015 | 1,960,380 | 651,277 | 40,354 | 8,639 | 59,230 | 1,636 | 6,370 | 14,594 | 1,017 | 2,364 | 5,154 |

## Table H9 : Number of Housing Units, Households and Population (DeJure) by Type of Housing Unit and Source of Energy for Lighting, Rural/Urban, Zambia 2010

| Source of Energy for Lighting, Housing Units, Households and Population | Occupied Housing Units | Type of Housing Unit | | | | | | | | | | |
|---|---|---|---|---|---|---|---|---|---|---|---|---|
| | | Traditional | Improved Traditional | Mixed | Conventional Flat | Conventional House | Mobile | Part of commercial building | Improvised/ Makeshift | Collective/ Institutional quarters | Unintended | Other |
| **Zambia Urban** | | | | | | | | | | | | |
| Housing Units | 1,004,067 | 92,832 | 175,763 | 54,113 | 216,999 | 445,221 | 500 | 4,930 | 4,416 | 2,247 | 2,748 | 4,298 |
| Households | 1,017,907 | 93,101 | 176,977 | 54,718 | 219,430 | 454,150 | 511 | 5,023 | 4,524 | 2,279 | 2,818 | 4,376 |
| Population | 5,173,450 | 439,800 | 877,694 | 285,437 | 1,007,479 | 2,476,843 | 2,441 | 22,377 | 19,932 | 9,263 | 11,198 | 20,986 |
| **Electricity** | | | | | | | | | | | | |
| Housing Units | 499,629 | 1,111 | 23,545 | 24,318 | 139,214 | 301,004 | 287 | 3,391 | 1,249 | 1,621 | 1,396 | 2,493 |
| Households | 507,340 | 1,132 | 23,769 | 24,661 | 140,769 | 306,368 | 293 | 3,449 | 1,287 | 1,642 | 1,442 | 2,528 |
| Population | 2,690,658 | 5,272 | 118,670 | 135,493 | 666,689 | 1,717,083 | 1,487 | 15,454 | 5,905 | 6,649 | 5,576 | 12,380 |
| **Gas** | | | | | | | | | | | | |
| Housing Units | 716 | 217 | 147 | 35 | 78 | 222 | 0 | 3 | 6 | 3 | 1 | 4 |
| Households | 720 | 217 | 149 | 36 | 78 | 222 | 0 | 4 | 6 | 3 | 1 | 4 |
| Population | 3,779 | 1,068 | 815 | 213 | 370 | 1,236 | 0 | 17 | 30 | 5 | 2 | 23 |
| **Wood** | | | | | | | | | | | | |
| Housing Units | 1,743 | 696 | 565 | 64 | 111 | 254 | 1 | 8 | 23 | 5 | 6 | 10 |
| Households | 1,759 | 699 | 566 | 64 | 115 | 262 | 1 | 8 | 23 | 5 | 6 | 10 |
| Population | 8,064 | 2,992 | 2,765 | 318 | 511 | 1,288 | 1 | 29 | 98 | 17 | 13 | 32 |
| **Candle** | | | | | | | | | | | | |
| Housing Units | 375,599 | 39,846 | 105,829 | 24,523 | 71,898 | 126,412 | 165 | 1,158 | 2,589 | 502 | 1,146 | 1,531 |
| Households | 380,881 | 39,947 | 106,582 | 24,750 | 72,684 | 129,675 | 168 | 1,182 | 2,652 | 511 | 1,167 | 1,563 |
| Population | 1,829,968 | 183,004 | 517,863 | 121,867 | 314,125 | 661,696 | 707 | 5,153 | 11,444 | 2,038 | 4,770 | 7,301 |
| **Paraffin** | | | | | | | | | | | | |
| Housing Units | 73,369 | 35,973 | 26,972 | 2,505 | 1,696 | 5,610 | 11 | 112 | 291 | 52 | 62 | 85 |
| Households | 73,744 | 36,069 | 27,116 | 2,519 | 1,732 | 5,686 | 12 | 114 | 294 | 52 | 63 | 87 |
| Population | 362,632 | 171,813 | 137,144 | 13,090 | 7,703 | 30,094 | 45 | 522 | 1,311 | 249 | 280 | 381 |
| **Solar** | | | | | | | | | | | | |
| Housing Units | 7,523 | 1,202 | 2,635 | 433 | 583 | 2,519 | 6 | 58 | 46 | 14 | 10 | 17 |
| Households | 7,573 | 1,204 | 2,649 | 436 | 590 | 2,541 | 6 | 59 | 47 | 14 | 10 | 17 |
| Population | 43,449 | 6,580 | 15,316 | 2,529 | 3,009 | 15,206 | 37 | 299 | 255 | 72 | 48 | 98 |
| **Bio Fuel** | | | | | | | | | | | | |
| Housing Units | 623 | 97 | 197 | 24 | 71 | 226 | 0 | 1 | 2 | 1 | 1 | 3 |
| Households | 631 | 98 | 199 | 24 | 71 | 230 | 0 | 2 | 2 | 1 | 1 | 3 |
| Population | 3,390 | 496 | 1,100 | 135 | 314 | 1,312 | 0 | 2 | 10 | 3 | 6 | 12 |
| **Diesel** | | | | | | | | | | | | |
| Housing Units | 1,843 | 845 | 649 | 56 | 59 | 207 | 2 | 3 | 12 | 0 | 5 | 5 |
| Households | 1,862 | 851 | 659 | 56 | 60 | 209 | 2 | 3 | 12 | 0 | 5 | 5 |
| Population | 9,333 | 4,076 | 3,295 | 301 | 297 | 1,234 | 13 | 17 | 64 | 0 | 16 | 20 |
| **None** | | | | | | | | | | | | |
| Housing Units | 1,620 | 647 | 450 | 56 | 83 | 315 | 3 | 8 | 37 | 1 | 8 | 12 |
| Households | 1,626 | 649 | 451 | 56 | 83 | 317 | 3 | 8 | 37 | 1 | 8 | 13 |
| Population | 6,608 | 2,458 | 1,880 | 247 | 354 | 1,465 | 13 | 20 | 104 | 2 | 25 | 40 |
| **Other** | | | | | | | | | | | | |
| Housing Units | 41,402 | 12,198 | 14,774 | 2,099 | 3,206 | 8,452 | 25 | 188 | 161 | 48 | 113 | 138 |
| Households | 41,771 | 12,235 | 14,837 | 2,116 | 3,248 | 8,640 | 26 | 194 | 164 | 50 | 115 | 146 |
| Population | 215,569 | 62,041 | 78,846 | 11,244 | 14,107 | 46,229 | 138 | 864 | 711 | 228 | 462 | 699 |

## Table H10: Number of Housing Units, Households and Population (DeJure) by Type of Housing Unit and Source of Energy for Heating, Rural/Urban, Zambia 2010

| Source of Energy for Heating, Housing Units, Households and Population | Occupied Housing Units | Type of Housing Unit | | | | | | | | | | |
|---|---|---|---|---|---|---|---|---|---|---|---|---|
| | | Traditional | Improved Traditional | Mixed | Conventional Flat | Conventional House | Mobile | Part of commercial building | Improvised/ Makeshift | Collective/ Institutional quarters | Unintended | Other |
| **Zambia Total** | | | | | | | | | | | | |
| Housing Units | 2,494,869 | 1,096,115 | 538,669 | 78,743 | 232,473 | 510,076 | 1,523 | 9,426 | 12,070 | 3,606 | 4,753 | 7,415 |
| Households | 2,513,768 | 1,098,990 | 541,234 | 79,462 | 235,161 | 519,586 | 1,539 | 9,552 | 12,204 | 3,670 | 4,850 | 7,520 |
| Population | 13,092,666 | 5,612,328 | 2,939,264 | 425,807 | 1,084,776 | 2,854,130 | 6,729 | 44,387 | 54,404 | 15,413 | 19,606 | 35,822 |
| **Electricity** | | | | | | | | | | | | |
| Housing Units | 292,562 | 832 | 12,003 | 12,159 | 79,465 | 181,747 | 207 | 1,830 | 743 | 1,241 | 735 | 1,600 |
| Households | 296,806 | 844 | 12,113 | 12,339 | 80,453 | 184,591 | 211 | 1,859 | 763 | 1,253 | 764 | 1,616 |
| Population | 1,552,246 | 3,870 | 60,481 | 67,120 | 372,745 | 1,019,446 | 1,045 | 8,250 | 3,559 | 4,872 | 3,041 | 7,817 |
| **Gas** | | | | | | | | | | | | |
| Housing Units | 1,974 | 1,142 | 429 | 41 | 57 | 249 | 14 | 7 | 15 | 6 | 8 | 6 |
| Households | 1,986 | 1,145 | 431 | 41 | 62 | 251 | 14 | 7 | 15 | 6 | 8 | 6 |
| Population | 10,354 | 5,816 | 2,458 | 257 | 253 | 1,302 | 67 | 34 | 74 | 25 | 39 | 29 |
| **Wood** | | | | | | | | | | | | |
| Housing Units | 844,519 | 592,972 | 205,440 | 12,108 | 4,115 | 20,869 | 497 | 1,485 | 4,403 | 388 | 766 | 1,476 |
| Households | 847,212 | 594,506 | 206,242 | 12,158 | 4,178 | 21,053 | 500 | 1,493 | 4,423 | 403 | 774 | 1,482 |
| Population | 4,525,008 | 3,075,337 | 1,182,801 | 70,880 | 22,165 | 131,424 | 2,184 | 7,393 | 20,462 | 1,880 | 3,417 | 7,065 |
| **Paraffin** | | | | | | | | | | | | |
| Housing Units | 3,355 | 1,902 | 915 | 77 | 127 | 301 | 1 | 5 | 20 | 1 | 2 | 4 |
| Households | 3,375 | 1,910 | 923 | 78 | 129 | 301 | 1 | 5 | 21 | 1 | 2 | 4 |
| Population | 17,305 | 9,788 | 4,882 | 363 | 592 | 1,543 | 1 | 19 | 84 | 5 | 14 | 14 |
| **Cow dung** | | | | | | | | | | | | |
| Housing Units | 2,324 | 1,199 | 469 | 71 | 186 | 340 | 2 | 6 | 29 | 1 | 2 | 19 |
| Households | 2,342 | 1,203 | 472 | 71 | 188 | 348 | 2 | 6 | 29 | 1 | 2 | 20 |
| Population | 11,399 | 5,739 | 2,398 | 351 | 790 | 1,884 | 3 | 29 | 97 | 3 | 9 | 96 |
| **Charcoal** | | | | | | | | | | | | |
| Housing Units | 814,596 | 247,626 | 210,989 | 35,653 | 106,108 | 200,111 | 400 | 3,963 | 4,143 | 1,309 | 1,885 | 2,409 |
| Households | 821,889 | 248,319 | 212,062 | 35,967 | 107,207 | 204,031 | 404 | 4,017 | 4,179 | 1,327 | 1,923 | 2,453 |
| Population | 4,191,516 | 1,239,703 | 1,102,594 | 187,270 | 492,638 | 1,104,930 | 1,796 | 19,137 | 18,153 | 5,778 | 7,742 | 11,775 |
| **Coal** | | | | | | | | | | | | |
| Housing Units | 2,543 | 1,045 | 580 | 82 | 220 | 533 | 3 | 26 | 17 | 8 | 10 | 19 |
| Households | 2,566 | 1,047 | 583 | 83 | 226 | 544 | 3 | 26 | 17 | 8 | 10 | 19 |
| Population | 13,426 | 5,299 | 3,186 | 442 | 1,081 | 3,016 | 17 | 123 | 79 | 28 | 29 | 126 |
| **Solar** | | | | | | | | | | | | |
| Housing Units | 5,618 | 2,268 | 1,466 | 402 | 282 | 1,086 | 2 | 20 | 8 | 14 | 22 | 48 |
| Households | 5,651 | 2,269 | 1,473 | 410 | 286 | 1,097 | 2 | 20 | 8 | 14 | 22 | 50 |
| Population | 30,476 | 11,733 | 8,387 | 2,165 | 1,537 | 6,064 | 9 | 125 | 28 | 48 | 96 | 284 |
| **Bio Fuel** | | | | | | | | | | | | |
| Housing Units | 602 | 286 | 119 | 19 | 41 | 119 | 0 | 4 | 8 | 1 | 3 | 2 |
| Households | 605 | 286 | 120 | 19 | 41 | 121 | 0 | 4 | 8 | 1 | 3 | 2 |
| Population | 3,019 | 1,381 | 630 | 93 | 186 | 661 | 0 | 17 | 28 | 2 | 14 | 7 |
| **Diesel** | | | | | | | | | | | | |
| Housing Units | 1,223 | 451 | 233 | 48 | 137 | 336 | 1 | 4 | 4 | 3 | 2 | 4 |
| Households | 1,235 | 453 | 235 | 48 | 140 | 341 | 1 | 4 | 4 | 3 | 2 | 4 |
| Population | 6,389 | 2,284 | 1,254 | 251 | 637 | 1,869 | 8 | 18 | 24 | 23 | 10 | 11 |
| **None** | | | | | | | | | | | | |
| Housing Units | 448,994 | 206,648 | 90,044 | 15,222 | 36,963 | 92,500 | 333 | 1,765 | 2,362 | 503 | 1,171 | 1,483 |
| Households | 452,996 | 207,156 | 90,507 | 15,348 | 37,418 | 94,796 | 336 | 1,796 | 2,417 | 520 | 1,192 | 1,510 |
| Population | 2,335,566 | 1,050,234 | 484,968 | 81,282 | 169,991 | 515,804 | 1,325 | 7,712 | 10,406 | 2,221 | 4,621 | 7,002 |
| **Other** | | | | | | | | | | | | |
| Housing Units | 76,559 | 39,744 | 15,982 | 2,861 | 4,772 | 11,885 | 63 | 311 | 318 | 131 | 147 | 345 |
| Households | 77,105 | 39,852 | 16,073 | 2,900 | 4,833 | 12,112 | 65 | 315 | 320 | 133 | 148 | 354 |
| Population | 395,962 | 201,144 | 85,225 | 15,333 | 22,161 | 66,187 | 274 | 1,530 | 1,410 | 528 | 574 | 1,596 |

## Table H10: Number of Housing Units, Households and Population (DeJure) by Type of Housing Unit and Source of Energy for Heating, Rural, Zambia 2010

| Source of Energy for Heating, Housing Units, Households and Population | Occupied Housing Units | Type of Housing Unit | | | | | | | | | | |
|---|---|---|---|---|---|---|---|---|---|---|---|---|
| | | Traditional | Improved Traditional | Mixed | Conventional Flat | Conventional House | Mobile | Part of commercial building | Improvised/ Makeshift | Collective/ Institutional quarters | Unintended | Other |
| **Zambia Rural** | | | | | | | | | | | | |
| Housing Units | 1,490,802 | 1,003,283 | 362,906 | 24,630 | 15,474 | 64,855 | 1,023 | 4,496 | 7,654 | 1,359 | 2,005 | 3,117 |
| Households | 1,495,861 | 1,005,889 | 364,257 | 24,744 | 15,731 | 65,436 | 1,028 | 4,529 | 7,680 | 1,391 | 2,032 | 3,144 |
| Population | 7,919,216 | 5,172,528 | 2,061,570 | 140,370 | 77,297 | 377,287 | 4,288 | 22,010 | 34,472 | 6,150 | 8,408 | 14,836 |
| **Electricity** | | | | | | | | | | | | |
| Housing Units | 19,457 | 406 | 2,912 | 802 | 3,825 | 10,606 | 25 | 200 | 92 | 178 | 110 | 301 |
| Households | 19,665 | 407 | 2,927 | 807 | 3,909 | 10,695 | 25 | 204 | 92 | 182 | 115 | 302 |
| Population | 101,202 | 1,922 | 15,106 | 4,109 | 19,187 | 56,704 | 85 | 910 | 490 | 781 | 455 | 1,453 |
| **Gas** | | | | | | | | | | | | |
| Housing Units | 1,663 | 1,085 | 395 | 30 | 16 | 89 | 13 | 4 | 13 | 6 | 8 | 4 |
| Households | 1,672 | 1,088 | 396 | 30 | 21 | 89 | 13 | 4 | 13 | 6 | 8 | 4 |
| Population | 8,810 | 5,558 | 2,272 | 195 | 66 | 486 | 63 | 21 | 68 | 25 | 39 | 17 |
| **Wood** | | | | | | | | | | | | |
| Housing Units | 805,064 | 572,643 | 193,091 | 10,788 | 3,177 | 16,983 | 461 | 1,414 | 4,102 | 351 | 696 | 1,358 |
| Households | 807,627 | 574,140 | 193,859 | 10,835 | 3,229 | 17,126 | 464 | 1,422 | 4,121 | 364 | 704 | 1,363 |
| Population | 4,320,551 | 2,974,503 | 1,116,716 | 63,549 | 17,440 | 108,578 | 2,057 | 7,086 | 19,254 | 1,715 | 3,157 | 6,496 |
| **Paraffin** | | | | | | | | | | | | |
| Housing Units | 2,320 | 1,652 | 579 | 23 | 12 | 40 | 0 | 2 | 8 | 0 | 2 | 2 |
| Households | 2,330 | 1,659 | 582 | 23 | 12 | 40 | 0 | 2 | 8 | 0 | 2 | 2 |
| Population | 12,111 | 8,530 | 3,133 | 114 | 52 | 214 | 0 | 13 | 36 | 0 | 14 | 5 |
| **Cow dung** | | | | | | | | | | | | |
| Housing Units | 1,465 | 1,100 | 242 | 26 | 7 | 40 | 2 | 4 | 27 | 0 | 1 | 16 |
| Households | 1,470 | 1,103 | 242 | 26 | 7 | 41 | 2 | 4 | 27 | 0 | 1 | 17 |
| Population | 7,243 | 5,314 | 1,317 | 156 | 43 | 221 | 3 | 15 | 82 | 0 | 6 | 86 |
| **Charcoal** | | | | | | | | | | | | |
| Housing Units | 335,118 | 196,654 | 96,621 | 7,152 | 5,368 | 23,470 | 210 | 1,863 | 2,009 | 530 | 636 | 605 |
| Households | 336,373 | 197,196 | 96,958 | 7,194 | 5,442 | 23,676 | 210 | 1,878 | 2,015 | 539 | 647 | 618 |
| Population | 1,759,280 | 1,001,560 | 533,038 | 40,068 | 26,216 | 132,404 | 838 | 9,201 | 8,319 | 2,302 | 2,487 | 2,847 |
| **Coal** | | | | | | | | | | | | |
| Housing Units | 1,583 | 954 | 427 | 19 | 17 | 126 | 3 | 17 | 7 | 6 | 4 | 3 |
| Households | 1,586 | 955 | 429 | 19 | 17 | 126 | 3 | 17 | 7 | 6 | 4 | 3 |
| Population | 8,422 | 4,896 | 2,435 | 119 | 68 | 725 | 17 | 83 | 33 | 23 | 9 | 14 |
| **Solar** | | | | | | | | | | | | |
| Housing Units | 3,146 | 1,830 | 839 | 62 | 59 | 297 | 2 | 16 | 5 | 13 | 17 | 6 |
| Households | 3,150 | 1,831 | 841 | 62 | 59 | 298 | 2 | 16 | 5 | 13 | 17 | 6 |
| Population | 17,211 | 9,622 | 4,963 | 360 | 320 | 1,680 | 9 | 91 | 19 | 40 | 80 | 27 |
| **Bio Fuel** | | | | | | | | | | | | |
| Housing Units | 346 | 231 | 73 | 11 | 4 | 22 | 0 | 1 | 3 | 0 | 1 | 0 |
| Households | 346 | 231 | 73 | 11 | 4 | 22 | 0 | 1 | 3 | 0 | 1 | 0 |
| Population | 1,692 | 1,105 | 364 | 63 | 23 | 113 | 0 | 6 | 10 | 0 | 8 | 0 |
| **Diesel** | | | | | | | | | | | | |
| Housing Units | 612 | 401 | 140 | 10 | 7 | 45 | 1 | 3 | 1 | 2 | 2 | 0 |
| Households | 615 | 403 | 140 | 10 | 7 | 46 | 1 | 3 | 1 | 2 | 2 | 0 |
| Population | 3,220 | 2,068 | 778 | 63 | 39 | 221 | 8 | 17 | 8 | 8 | 10 | 0 |
| **None** | | | | | | | | | | | | |
| Housing Units | 269,491 | 189,883 | 57,423 | 4,779 | 2,455 | 11,329 | 258 | 778 | 1,216 | 226 | 447 | 697 |
| Households | 270,302 | 190,340 | 57,595 | 4,796 | 2,488 | 11,443 | 260 | 781 | 1,216 | 230 | 450 | 703 |
| Population | 1,416,160 | 972,123 | 324,492 | 26,589 | 11,384 | 65,379 | 988 | 3,631 | 5,404 | 1,032 | 1,839 | 3,299 |
| **Other** | | | | | | | | | | | | |
| Housing Units | 50,537 | 36,444 | 10,164 | 928 | 527 | 1,808 | 48 | 194 | 171 | 47 | 81 | 125 |
| Households | 50,725 | 36,536 | 10,215 | 931 | 536 | 1,834 | 48 | 197 | 172 | 49 | 81 | 126 |
| Population | 263,314 | 185,327 | 56,956 | 4,985 | 2,459 | 10,562 | 220 | 936 | 749 | 224 | 304 | 592 |

# Table H10: Number of Housing Units, Households and Population (DeJure) by Type of Housing Unit and Source of Energy for Heating, Urban, Zambia 2010

| Source of Energy for Heating, Housing Units, Households and Population | Occupied Housing Units | Type of Housing Unit | | | | | | | | | | |
|---|---|---|---|---|---|---|---|---|---|---|---|---|
| | | Traditional | Improved Traditional | Mixed | Conventional Flat | Conventional House | Mobile | Part of commercial building | Improvised/ Makeshift | Collective/ Institutional quarters | Unintended | Other |
| **Zambia Urban** | | | | | | | | | | | | |
| Housing Units | 1,004,067 | 92,832 | 175,763 | 54,113 | 216,999 | 445,221 | 500 | 4,930 | 4,416 | 2,247 | 2,748 | 4,298 |
| Households | 1,017,907 | 93,101 | 176,977 | 54,718 | 219,430 | 454,150 | 511 | 5,023 | 4,524 | 2,279 | 2,818 | 4,376 |
| Population | 5,173,450 | 439,800 | 877,694 | 285,437 | 1,007,479 | 2,476,843 | 2,441 | 22,377 | 19,932 | 9,263 | 11,198 | 20,986 |
| **Electricity** | | | | | | | | | | | | |
| Housing Units | 273,105 | 426 | 9,091 | 11,357 | 75,640 | 171,141 | 182 | 1,630 | 651 | 1,063 | 625 | 1,299 |
| Households | 277,141 | 437 | 9,186 | 11,532 | 76,544 | 173,896 | 186 | 1,655 | 671 | 1,071 | 649 | 1,314 |
| Population | 1,451,044 | 1,948 | 45,375 | 63,011 | 353,558 | 962,742 | 960 | 7,340 | 3,069 | 4,091 | 2,586 | 6,364 |
| **Gas** | | | | | | | | | | | | |
| Housing Units | 311 | 57 | 34 | 11 | 41 | 160 | 1 | 3 | 2 | 0 | 0 | 2 |
| Households | 314 | 57 | 35 | 11 | 41 | 162 | 1 | 3 | 2 | 0 | 0 | 2 |
| Population | 1,544 | 258 | 186 | 62 | 187 | 816 | 4 | 13 | 6 | 0 | 0 | 12 |
| **Wood** | | | | | | | | | | | | |
| Housing Units | 39,455 | 20,329 | 12,349 | 1,320 | 938 | 3,886 | 36 | 71 | 301 | 37 | 70 | 118 |
| Households | 39,585 | 20,366 | 12,383 | 1,323 | 949 | 3,927 | 36 | 71 | 302 | 39 | 70 | 119 |
| Population | 204,457 | 100,834 | 66,085 | 7,331 | 4,725 | 22,846 | 127 | 307 | 1,208 | 165 | 260 | 569 |
| **Paraffin** | | | | | | | | | | | | |
| Housing Units | 1,035 | 250 | 336 | 54 | 115 | 261 | 1 | 3 | 12 | 1 | 0 | 2 |
| Households | 1,045 | 251 | 341 | 55 | 117 | 261 | 1 | 3 | 13 | 1 | 0 | 2 |
| Population | 5,194 | 1,258 | 1,749 | 249 | 540 | 1,329 | 1 | 6 | 48 | 5 | 0 | 9 |
| **Cow dung** | | | | | | | | | | | | |
| Housing Units | 859 | 99 | 227 | 45 | 179 | 300 | 0 | 2 | 2 | 1 | 1 | 3 |
| Households | 872 | 100 | 230 | 45 | 181 | 307 | 0 | 2 | 2 | 1 | 1 | 3 |
| Population | 4,156 | 425 | 1,081 | 195 | 747 | 1,663 | 0 | 14 | 15 | 3 | 3 | 10 |
| **Charcoal** | | | | | | | | | | | | |
| Housing Units | 479,478 | 50,972 | 114,368 | 28,501 | 100,740 | 176,641 | 190 | 2,100 | 2,134 | 779 | 1,249 | 1,804 |
| Households | 485,516 | 51,123 | 115,104 | 28,773 | 101,765 | 180,355 | 194 | 2,139 | 2,164 | 788 | 1,276 | 1,835 |
| Population | 2,432,236 | 238,143 | 569,556 | 147,202 | 466,422 | 972,526 | 958 | 9,936 | 9,834 | 3,476 | 5,255 | 8,928 |
| **Coal** | | | | | | | | | | | | |
| Housing Units | 960 | 91 | 153 | 63 | 203 | 407 | 0 | 9 | 10 | 2 | 6 | 16 |
| Households | 980 | 92 | 154 | 64 | 209 | 418 | 0 | 9 | 10 | 2 | 6 | 16 |
| Population | 5,004 | 403 | 751 | 323 | 1,013 | 2,291 | 0 | 40 | 46 | 5 | 20 | 112 |
| **Solar** | | | | | | | | | | | | |
| Housing Units | 2,472 | 438 | 627 | 340 | 223 | 789 | 0 | 4 | 3 | 1 | 5 | 42 |
| Households | 2,501 | 438 | 632 | 348 | 227 | 799 | 0 | 4 | 3 | 1 | 5 | 44 |
| Population | 13,265 | 2,111 | 3,424 | 1,805 | 1,217 | 4,384 | 0 | 34 | 9 | 8 | 16 | 257 |
| **Bio Fuel** | | | | | | | | | | | | |
| Housing Units | 256 | 55 | 46 | 8 | 37 | 97 | 0 | 3 | 5 | 1 | 2 | 2 |
| Households | 259 | 55 | 47 | 8 | 37 | 99 | 0 | 3 | 5 | 1 | 2 | 2 |
| Population | 1,327 | 276 | 266 | 30 | 163 | 548 | 0 | 11 | 18 | 2 | 6 | 7 |
| **Diesel** | | | | | | | | | | | | |
| Housing Units | 611 | 50 | 93 | 38 | 130 | 291 | 0 | 1 | 3 | 1 | 0 | 4 |
| Households | 620 | 50 | 95 | 38 | 133 | 295 | 0 | 1 | 3 | 1 | 0 | 4 |
| Population | 3,169 | 216 | 476 | 188 | 598 | 1,648 | 0 | 1 | 16 | 15 | 0 | 11 |
| **None** | | | | | | | | | | | | |
| Housing Units | 179,503 | 16,765 | 32,621 | 10,443 | 34,508 | 81,171 | 75 | 987 | 1,146 | 277 | 724 | 786 |
| Households | 182,694 | 16,816 | 32,912 | 10,552 | 34,930 | 83,353 | 76 | 1,015 | 1,201 | 290 | 742 | 807 |
| Population | 919,406 | 78,111 | 160,476 | 54,693 | 158,607 | 450,425 | 337 | 4,081 | 5,002 | 1,189 | 2,782 | 3,703 |
| **Other** | | | | | | | | | | | | |
| Housing Units | 26,022 | 3,300 | 5,818 | 1,933 | 4,245 | 10,077 | 15 | 117 | 147 | 84 | 66 | 220 |
| Households | 26,380 | 3,316 | 5,858 | 1,969 | 4,297 | 10,278 | 17 | 118 | 148 | 84 | 67 | 228 |
| Population | 132,648 | 15,817 | 28,269 | 10,348 | 19,702 | 55,625 | 54 | 594 | 661 | 304 | 270 | 1,004 |

## Table H11 : Number of Housing Units with/without Kitchen by Type of Housing Unit and Source of Energy for Cooking, Rural/Urban, Zambia 2010

| Source of Energy for Cooking | Occupied Housing Units | Type of Housing Unit | | | | | | | | | | |
|---|---|---|---|---|---|---|---|---|---|---|---|---|
| | | Traditional | Improved Traditional | Mixed | Conventional Flat | Conventional House | Mobile | Part of commercial building | Improvised/ Makeshift | Collective/ Institutional quarters | Unintended | Other |
| **Zambia Total** | | | | | | | | | | | | |
| **Total** | **2,494,869** | **1,096,115** | **538,669** | **78,743** | **232,473** | **510,076** | **1,523** | **9,426** | **12,070** | **3,606** | **4,753** | **7,415** |
| Electricity | 417,945 | 1,237 | 18,779 | 18,013 | 116,377 | 254,490 | 297 | 2,683 | 950 | 1,634 | 1,147 | 2,338 |
| Gas | 3,517 | 1,822 | 724 | 99 | 119 | 646 | 27 | 15 | 20 | 19 | 15 | 11 |
| Wood | 1,338,292 | 936,972 | 323,330 | 21,014 | 6,567 | 36,481 | 769 | 2,893 | 6,122 | 577 | 1,280 | 2,287 |
| Candle | 0 | 0 | 0 | 0 | 0 | 0 | 0 | 0 | 0 | 0 | 0 | 0 |
| Paraffin | 1,894 | 1,194 | 463 | 24 | 51 | 136 | 1 | 6 | 13 | 3 | 1 | 2 |
| Cow dung | 2,684 | 1,499 | 485 | 84 | 182 | 352 | 4 | 4 | 37 | 1 | 3 | 33 |
| Charcoal | 722,116 | 149,466 | 193,076 | 39,242 | 108,557 | 216,518 | 408 | 3,750 | 4,840 | 1,325 | 2,263 | 2,671 |
| Coal | 1,744 | 529 | 409 | 77 | 195 | 463 | 1 | 17 | 18 | 7 | 8 | 20 |
| Solar | 397 | 77 | 108 | 17 | 25 | 157 | 2 | 3 | 3 | 4 | 0 | 1 |
| Bio Fuel | 926 | 538 | 238 | 17 | 37 | 84 | 1 | 3 | 4 | 2 | 1 | 1 |
| Diesel | 147 | 57 | 37 | 5 | 13 | 25 | 0 | 0 | 1 | 2 | 6 | 1 |
| None | 832 | 336 | 154 | 39 | 119 | 128 | 2 | 20 | 8 | 12 | 9 | 5 |
| Other | 4,375 | 2,388 | 866 | 112 | 231 | 596 | 11 | 32 | 54 | 20 | 20 | 45 |
| **Housing Units with Kitchen** | | | | | | | | | | | | |
| **Total** | **1,321,556** | **557,909** | **295,954** | **37,627** | **81,384** | **335,019** | **439** | **4,083** | **2,975** | **1,772** | **1,159** | **3,235** |
| Electricity | 282,727 | 570 | 10,364 | 11,351 | 55,642 | 200,084 | 170 | 1,402 | 430 | 848 | 359 | 1,507 |
| Gas | 2,162 | 929 | 470 | 56 | 78 | 568 | 12 | 10 | 9 | 18 | 6 | 6 |
| Wood | 713,999 | 473,989 | 196,850 | 11,410 | 3,024 | 24,185 | 154 | 1,453 | 1,510 | 333 | 383 | 708 |
| Candle | 0 | 0 | 0 | 0 | 0 | 0 | 0 | 0 | 0 | 0 | 0 | 0 |
| Paraffin | 1,025 | 652 | 260 | 15 | 11 | 80 | 0 | 3 | 1 | 3 | 0 | 0 |
| Cow dung | 753 | 326 | 179 | 27 | 37 | 173 | 0 | 3 | 4 | 0 | 1 | 3 |
| Charcoal | 316,834 | 79,619 | 86,885 | 14,657 | 22,412 | 109,047 | 95 | 1,187 | 998 | 550 | 398 | 986 |
| Coal | 924 | 305 | 219 | 24 | 53 | 291 | 1 | 3 | 7 | 7 | 2 | 12 |
| Solar | 286 | 45 | 75 | 14 | 12 | 130 | 2 | 2 | 3 | 2 | 0 | 1 |
| Bio Fuel | 418 | 235 | 114 | 11 | 9 | 42 | 0 | 3 | 1 | 2 | 0 | 1 |
| Diesel | 96 | 34 | 21 | 3 | 11 | 21 | 0 | 0 | 0 | 1 | 5 | 0 |
| None | 173 | 86 | 34 | 7 | 10 | 35 | 0 | 0 | 0 | 1 | 0 | 0 |
| Other | 2,159 | 1,119 | 483 | 52 | 85 | 363 | 5 | 17 | 12 | 7 | 5 | 11 |
| **Housing Units without a Kitchen** | | | | | | | | | | | | |
| **Total** | **1,173,313** | **538,206** | **242,715** | **41,116** | **151,089** | **175,057** | **1,084** | **5,343** | **9,095** | **1,834** | **3,594** | **4,180** |
| Electricity | 135,218 | 667 | 8,415 | 6,662 | 60,735 | 54,406 | 127 | 1,281 | 520 | 786 | 788 | 831 |
| Gas | 1,355 | 893 | 254 | 43 | 41 | 78 | 15 | 5 | 11 | 1 | 9 | 5 |
| Wood | 624,293 | 462,983 | 126,480 | 9,604 | 3,543 | 12,296 | 615 | 1,440 | 4,612 | 244 | 897 | 1,579 |
| Candle | 0 | 0 | 0 | 0 | 0 | 0 | 0 | 0 | 0 | 0 | 0 | 0 |
| Paraffin | 869 | 542 | 203 | 9 | 40 | 56 | 1 | 3 | 12 | 0 | 1 | 2 |
| Cow dung | 1,931 | 1,173 | 306 | 57 | 145 | 179 | 4 | 1 | 33 | 1 | 2 | 30 |
| Charcoal | 405,282 | 69,847 | 106,191 | 24,585 | 86,145 | 107,471 | 313 | 2,563 | 3,842 | 775 | 1,865 | 1,685 |
| Coal | 820 | 224 | 190 | 53 | 142 | 172 | 0 | 14 | 11 | 0 | 6 | 8 |
| Solar | 111 | 32 | 33 | 3 | 13 | 27 | 0 | 1 | 0 | 2 | 0 | 0 |
| Bio Fuel | 508 | 303 | 124 | 6 | 28 | 42 | 1 | 0 | 3 | 0 | 1 | 0 |
| Diesel | 51 | 23 | 16 | 2 | 2 | 4 | 0 | 0 | 1 | 1 | 1 | 1 |
| None | 659 | 250 | 120 | 32 | 109 | 93 | 2 | 20 | 8 | 11 | 9 | 5 |
| Other | 2,216 | 1,269 | 383 | 60 | 146 | 233 | 6 | 15 | 42 | 13 | 15 | 34 |

## Table H11 : Number of Housing Units with/without Kitchen by Type of Housing Unit and Source of Energy for Cooking, Rural, Zambia 2010

| Source of Energy for Cooking | Occupied Housing Units | Type of Housing Unit | | | | | | | | | | |
|---|---|---|---|---|---|---|---|---|---|---|---|---|
| | | Traditional | Improved Traditional | Mixed | Conventional Flat | Conventional House | Mobile | Part of commercial building | Improvised/ Makeshift | Collective/ Institutional quarters | Unintended | Other |
| **Zambia Rural** | | | | | | | | | | | | |
| **Total** | **1,490,802** | **1,003,283** | **362,906** | **24,630** | **15,474** | **64,855** | **1,023** | **4,496** | **7,654** | **1,359** | **2,005** | **3,117** |
| Electricity | 30,438 | 702 | 5,072 | 1,493 | 6,128 | 15,628 | 69 | 305 | 144 | 227 | 181 | 489 |
| Gas | 2,876 | 1,733 | 655 | 70 | 37 | 294 | 23 | 7 | 17 | 19 | 12 | 9 |
| Wood | 1,267,488 | 901,135 | 300,537 | 18,499 | 5,076 | 29,446 | 712 | 2,730 | 5,626 | 521 | 1,139 | 2,067 |
| Candle | 0 | 0 | 0 | 0 | 0 | 0 | 0 | 0 | 0 | 0 | 0 | 0 |
| Paraffin | 1,532 | 1,090 | 363 | 14 | 8 | 37 | 0 | 5 | 10 | 2 | 1 | 2 |
| Cow dung | 1,735 | 1,393 | 219 | 23 | 5 | 28 | 4 | 2 | 32 | 0 | 0 | 29 |
| Charcoal | 181,419 | 93,749 | 54,886 | 4,452 | 4,157 | 19,074 | 203 | 1,420 | 1,771 | 567 | 656 | 484 |
| Coal | 819 | 449 | 240 | 9 | 12 | 86 | 1 | 9 | 3 | 3 | 5 | 2 |
| Solar | 255 | 73 | 77 | 13 | 6 | 77 | 2 | 2 | 1 | 4 | 0 | 0 |
| Bio Fuel | 588 | 402 | 140 | 9 | 5 | 25 | 1 | 1 | 2 | 1 | 1 | 1 |
| Diesel | 101 | 55 | 25 | 3 | 3 | 12 | 0 | 0 | 1 | 2 | 0 | 0 |
| None | 446 | 301 | 99 | 9 | 5 | 20 | 2 | 2 | 3 | 1 | 2 | 2 |
| Other | 3,105 | 2,201 | 593 | 36 | 32 | 128 | 6 | 13 | 44 | 12 | 8 | 32 |
| **Housing Units with Kitchen** | | | | | | | | | | | | |
| **Total** | **821,156** | **518,065** | **226,457** | **14,018** | **8,022** | **47,884** | **211** | **2,137** | **1,807** | **783** | **547** | **1,225** |
| Electricity | 21,883 | 315 | 3,386 | 1,013 | 3,554 | 12,790 | 16 | 166 | 75 | 140 | 51 | 377 |
| Gas | 1,706 | 890 | 437 | 41 | 30 | 261 | 9 | 3 | 7 | 18 | 5 | 5 |
| Wood | 684,957 | 459,668 | 187,578 | 10,474 | 2,549 | 20,403 | 148 | 1,404 | 1,422 | 307 | 359 | 645 |
| Candle | 0 | 0 | 0 | 0 | 0 | 0 | 0 | 0 | 0 | 0 | 0 | 0 |
| Paraffin | 873 | 605 | 220 | 10 | 4 | 29 | 0 | 2 | 1 | 2 | 0 | 0 |
| Cow dung | 398 | 281 | 82 | 6 | 2 | 18 | 0 | 2 | 4 | 0 | 0 | 3 |
| Charcoal | 108,621 | 54,658 | 34,068 | 2,428 | 1,846 | 14,132 | 32 | 548 | 289 | 307 | 127 | 186 |
| Coal | 504 | 267 | 153 | 6 | 6 | 64 | 1 | 1 | 1 | 3 | 1 | 1 |
| Solar | 193 | 42 | 58 | 11 | 4 | 71 | 2 | 2 | 1 | 2 | 0 | 0 |
| Bio Fuel | 319 | 212 | 78 | 8 | 3 | 14 | 0 | 1 | 1 | 1 | 0 | 1 |
| Diesel | 64 | 32 | 15 | 2 | 3 | 11 | 0 | 0 | 0 | 1 | 0 | 0 |
| None | 122 | 81 | 28 | 3 | 1 | 8 | 0 | 0 | 0 | 1 | 0 | 0 |
| Other | 1,516 | 1,014 | 354 | 16 | 20 | 83 | 3 | 8 | 6 | 1 | 4 | 7 |
| **Housing Units without a Kitchen** | | | | | | | | | | | | |
| **Total** | **669,646** | **485,218** | **136,449** | **10,612** | **7,452** | **16,971** | **812** | **2,359** | **5,847** | **576** | **1,458** | **1,892** |
| Electricity | 8,555 | 387 | 1,686 | 480 | 2,574 | 2,838 | 53 | 139 | 69 | 87 | 130 | 112 |
| Gas | 1,170 | 843 | 218 | 29 | 7 | 33 | 14 | 4 | 10 | 1 | 7 | 4 |
| Wood | 582,531 | 441,467 | 112,959 | 8,025 | 2,527 | 9,043 | 564 | 1,326 | 4,204 | 214 | 780 | 1,422 |
| Candle | 0 | 0 | 0 | 0 | 0 | 0 | 0 | 0 | 0 | 0 | 0 | 0 |
| Paraffin | 659 | 485 | 143 | 4 | 4 | 8 | 0 | 3 | 9 | 0 | 1 | 2 |
| Cow dung | 1,337 | 1,112 | 137 | 17 | 3 | 10 | 4 | 0 | 28 | 0 | 0 | 26 |
| Charcoal | 72,798 | 39,091 | 20,818 | 2,024 | 2,311 | 4,942 | 171 | 872 | 1,482 | 260 | 529 | 298 |
| Coal | 315 | 182 | 87 | 3 | 6 | 22 | 0 | 8 | 2 | 0 | 4 | 1 |
| Solar | 62 | 31 | 19 | 2 | 2 | 6 | 0 | 0 | 0 | 2 | 0 | 0 |
| Bio Fuel | 269 | 190 | 62 | 1 | 2 | 11 | 1 | 0 | 1 | 0 | 1 | 0 |
| Diesel | 37 | 23 | 10 | 1 | 0 | 1 | 0 | 0 | 1 | 1 | 0 | 0 |
| None | 324 | 220 | 71 | 6 | 4 | 12 | 2 | 2 | 3 | 0 | 2 | 2 |
| Other | 1,589 | 1,187 | 239 | 20 | 12 | 45 | 3 | 5 | 38 | 11 | 4 | 25 |

## Table H11 : Number of Housing Units with/without Kitchen by Type of Housing Unit and Source of Energy for Cooking, Urban, Zambia 2010

| Source of Energy for Cooking | Occupied Housing Units | Type of Housing Unit | | | | | | | | | | |
|---|---|---|---|---|---|---|---|---|---|---|---|---|
| | | Traditional | Improved Traditional | Mixed | Conventional Flat | Conventional House | Mobile | Part of commercial building | Improvised/ Makeshift | Collective/ Institutional quarters | Unintended | Other |
| **Zambia Urban** | | | | | | | | | | | | |
| **Total** | **1,004,067** | **92,832** | **175,763** | **54,113** | **216,999** | **445,221** | **500** | **4,930** | **4,416** | **2,247** | **2,748** | **4,298** |
| Electricity | 387,507 | 535 | 13,707 | 16,520 | 110,249 | 238,862 | 228 | 2,378 | 806 | 1,407 | 966 | 1,849 |
| Gas | 641 | 89 | 69 | 29 | 82 | 352 | 4 | 8 | 3 | 0 | 3 | 2 |
| Wood | 70,804 | 35,837 | 22,793 | 2,515 | 1,491 | 7,035 | 57 | 163 | 496 | 56 | 141 | 220 |
| Candle | 0 | 0 | 0 | 0 | 0 | 0 | 0 | 0 | 0 | 0 | 0 | 0 |
| Paraffin | 362 | 104 | 100 | 10 | 43 | 99 | 1 | 1 | 3 | 1 | 0 | 0 |
| Cow dung | 949 | 106 | 266 | 61 | 177 | 324 | 0 | 2 | 5 | 1 | 3 | 4 |
| Charcoal | 540,697 | 55,717 | 138,190 | 34,790 | 104,400 | 197,444 | 205 | 2,330 | 3,069 | 758 | 1,607 | 2,187 |
| Coal | 925 | 80 | 169 | 68 | 183 | 377 | 0 | 8 | 15 | 4 | 3 | 18 |
| Solar | 142 | 4 | 31 | 4 | 19 | 80 | 0 | 1 | 2 | 0 | 0 | 1 |
| Bio Fuel | 338 | 136 | 98 | 8 | 32 | 59 | 0 | 2 | 2 | 1 | 0 | 0 |
| Diesel | 46 | 2 | 12 | 2 | 10 | 13 | 0 | 0 | 0 | 0 | 6 | 1 |
| None | 386 | 35 | 55 | 30 | 114 | 108 | 0 | 18 | 5 | 11 | 7 | 3 |
| Other | 1,270 | 187 | 273 | 76 | 199 | 468 | 5 | 19 | 10 | 8 | 12 | 13 |
| **Housing Units with Kitchen** | | | | | | | | | | | | |
| **Total** | **500,400** | **39,844** | **69,497** | **23,609** | **73,362** | **287,135** | **228** | **1,946** | **1,168** | **989** | **612** | **2,010** |
| Electricity | 260,844 | 255 | 6,978 | 10,338 | 52,088 | 187,294 | 154 | 1,236 | 355 | 708 | 308 | 1,130 |
| Gas | 456 | 39 | 33 | 15 | 48 | 307 | 3 | 7 | 2 | 0 | 1 | 1 |
| Wood | 29,042 | 14,321 | 9,272 | 936 | 475 | 3,782 | 6 | 49 | 88 | 26 | 24 | 63 |
| Candle | 0 | 0 | 0 | 0 | 0 | 0 | 0 | 0 | 0 | 0 | 0 | 0 |
| Paraffin | 152 | 47 | 40 | 5 | 7 | 51 | 0 | 1 | 0 | 1 | 0 | 0 |
| Cow dung | 355 | 45 | 97 | 21 | 35 | 155 | 0 | 1 | 0 | 0 | 1 | 0 |
| Charcoal | 208,213 | 24,961 | 52,817 | 12,229 | 20,566 | 94,915 | 63 | 639 | 709 | 243 | 271 | 800 |
| Coal | 420 | 38 | 66 | 18 | 47 | 227 | 0 | 2 | 6 | 4 | 1 | 11 |
| Solar | 93 | 3 | 17 | 3 | 8 | 59 | 0 | 0 | 2 | 0 | 0 | 1 |
| Bio Fuel | 99 | 23 | 36 | 3 | 6 | 28 | 0 | 2 | 0 | 1 | 0 | 0 |
| Diesel | 32 | 2 | 6 | 1 | 8 | 10 | 0 | 0 | 0 | 0 | 5 | 0 |
| None | 51 | 5 | 6 | 4 | 9 | 27 | 0 | 0 | 0 | 0 | 0 | 0 |
| Other | 643 | 105 | 129 | 36 | 65 | 280 | 2 | 9 | 6 | 6 | 1 | 4 |
| **Housing Units without a Kitchen** | | | | | | | | | | | | |
| **Total** | **503,667** | **52,988** | **106,266** | **30,504** | **143,637** | **158,086** | **272** | **2,984** | **3,248** | **1,258** | **2,136** | **2,288** |
| Electricity | 126,663 | 280 | 6,729 | 6,182 | 58,161 | 51,568 | 74 | 1,142 | 451 | 699 | 658 | 719 |
| Gas | 185 | 50 | 36 | 14 | 34 | 45 | 1 | 1 | 1 | 0 | 2 | 1 |
| Wood | 41,762 | 21,516 | 13,521 | 1,579 | 1,016 | 3,253 | 51 | 114 | 408 | 30 | 117 | 157 |
| Candle | 0 | 0 | 0 | 0 | 0 | 0 | 0 | 0 | 0 | 0 | 0 | 0 |
| Paraffin | 210 | 57 | 60 | 5 | 36 | 48 | 1 | 0 | 3 | 0 | 0 | 0 |
| Cow dung | 594 | 61 | 169 | 40 | 142 | 169 | 0 | 1 | 5 | 1 | 2 | 4 |
| Charcoal | 332,484 | 30,756 | 85,373 | 22,561 | 83,834 | 102,529 | 142 | 1,691 | 2,360 | 515 | 1,336 | 1,387 |
| Coal | 505 | 42 | 103 | 50 | 136 | 150 | 0 | 6 | 9 | 0 | 2 | 7 |
| Solar | 49 | 1 | 14 | 1 | 11 | 21 | 0 | 1 | 0 | 0 | 0 | 0 |
| Bio Fuel | 239 | 113 | 62 | 5 | 26 | 31 | 0 | 0 | 2 | 0 | 0 | 0 |
| Diesel | 14 | 0 | 6 | 1 | 2 | 3 | 0 | 0 | 0 | 0 | 1 | 1 |
| None | 335 | 30 | 49 | 26 | 105 | 81 | 0 | 18 | 5 | 11 | 7 | 3 |
| Other | 627 | 82 | 144 | 40 | 134 | 188 | 3 | 10 | 4 | 2 | 11 | 9 |

## Table H12: Number of Households and Population (Dejure) by Type of Housing Unit and Source of Water Supply, Rural/Urban, Zambia 2010

| Source of Household Water Supply, Households and Population | Total | Type of Housing Unit | | | | | | | | | | |
|---|---|---|---|---|---|---|---|---|---|---|---|---|
| | | Traditional | Improved Traditional | Mixed | Conventional Flat | Conventional House | Mobile | Part of commercial building | Improvised/ Makeshift | Collective/ Institutional quarters | Unintended | Other |
| **Zambia Total** | | | | | | | | | | | | |
| Households | 2,513,768 | 1,098,990 | 541,234 | 79,462 | 235,161 | 519,586 | 1,539 | 9,552 | 12,204 | 3,670 | 4,850 | 7,520 |
| Population | 13,092,666 | 5,612,328 | 2,939,264 | 425,807 | 1,084,776 | 2,854,130 | 6,729 | 44,387 | 54,404 | 15,413 | 19,606 | 35,822 |
| **Piped water inside the housing unit** | | | | | | | | | | | | |
| Households | 153,485 | 0 | 0 | 3,881 | 30,677 | 116,103 | 97 | 819 | 0 | 596 | 324 | 988 |
| Population | 844,343 | 0 | 0 | 22,948 | 145,848 | 662,731 | 489 | 3,863 | 0 | 2,308 | 1,295 | 4,861 |
| **Piped water outside housing unit within stand/plot** | | | | | | | | | | | | |
| Households | 250,371 | 4,029 | 23,206 | 16,798 | 55,529 | 144,872 | 231 | 1,401 | 1,121 | 797 | 921 | 1,466 |
| Population | 1,302,008 | 19,028 | 112,907 | 89,739 | 259,961 | 794,341 | 1,073 | 6,224 | 5,018 | 3,371 | 3,336 | 7,010 |
| **Communal tap** | | | | | | | | | | | | |
| Households | 227,064 | 24,549 | 36,383 | 11,854 | 69,663 | 79,220 | 160 | 1,243 | 1,576 | 671 | 697 | 1,048 |
| Population | 1,095,280 | 114,243 | 178,373 | 58,551 | 313,001 | 407,678 | 645 | 5,432 | 6,851 | 2,572 | 2,869 | 5,065 |
| **Protected well** | | | | | | | | | | | | |
| Households | 280,125 | 122,082 | 89,622 | 10,679 | 14,213 | 40,424 | 84 | 926 | 974 | 270 | 385 | 466 |
| Population | 1,471,594 | 623,711 | 481,122 | 57,455 | 67,511 | 227,380 | 388 | 4,310 | 4,605 | 1,232 | 1,640 | 2,240 |
| **Protected borehole** | | | | | | | | | | | | |
| Households | 428,999 | 238,717 | 110,420 | 10,607 | 13,637 | 48,514 | 420 | 2,701 | 1,441 | 751 | 933 | 858 |
| Population | 2,284,755 | 1,233,168 | 626,614 | 58,890 | 63,628 | 269,959 | 1,845 | 12,887 | 6,692 | 3,373 | 3,740 | 3,959 |
| **Unprotected well** | | | | | | | | | | | | |
| Households | 537,400 | 336,961 | 136,190 | 11,049 | 16,114 | 32,128 | 225 | 937 | 1,967 | 169 | 666 | 994 |
| Population | 2,834,151 | 1,735,839 | 756,140 | 61,576 | 76,301 | 181,193 | 916 | 4,585 | 9,273 | 736 | 2,848 | 4,744 |
| **Unprotected borehole** | | | | | | | | | | | | |
| Households | 57,201 | 34,791 | 14,981 | 1,224 | 1,481 | 4,116 | 34 | 195 | 141 | 57 | 83 | 98 |
| Population | 304,026 | 179,048 | 84,756 | 7,053 | 7,081 | 23,214 | 141 | 957 | 669 | 275 | 368 | 464 |
| **River/Dam/Stream** | | | | | | | | | | | | |
| Households | 427,119 | 312,581 | 95,017 | 4,971 | 1,796 | 6,902 | 192 | 451 | 3,831 | 167 | 356 | 855 |
| Population | 2,211,420 | 1,584,412 | 524,138 | 27,485 | 8,746 | 41,086 | 847 | 2,198 | 16,295 | 713 | 1,558 | 3,942 |
| **Rain water tank** | | | | | | | | | | | | |
| Households | 3,548 | 2,277 | 831 | 44 | 116 | 216 | 4 | 7 | 36 | 5 | 7 | 5 |
| Population | 18,421 | 11,733 | 4,402 | 245 | 578 | 1,181 | 8 | 45 | 158 | 22 | 22 | 27 |
| **Other tap** | | | | | | | | | | | | |
| Households | 60,245 | 5,035 | 10,098 | 3,252 | 14,325 | 25,851 | 52 | 334 | 520 | 165 | 295 | 318 |
| Population | 296,361 | 23,857 | 48,978 | 16,586 | 64,624 | 134,958 | 219 | 1,437 | 2,228 | 719 | 1,230 | 1,525 |
| **Water kiosk** | | | | | | | | | | | | |
| Households | 63,184 | 6,605 | 19,994 | 4,372 | 15,075 | 15,991 | 21 | 465 | 256 | 12 | 110 | 283 |
| Population | 304,962 | 30,971 | 97,865 | 21,481 | 66,368 | 82,978 | 89 | 2,139 | 1,177 | 59 | 460 | 1,375 |
| **Water vendor** | | | | | | | | | | | | |
| Households | 5,648 | 391 | 707 | 306 | 1,748 | 2,373 | 5 | 29 | 24 | 1 | 32 | 32 |
| Population | 26,850 | 1,797 | 3,436 | 1,491 | 7,571 | 12,078 | 14 | 115 | 115 | 5 | 105 | 123 |
| **Mineral/bottled water** | | | | | | | | | | | | |
| Households | 93 | 0 | 0 | 2 | 28 | 56 | 3 | 1 | 0 | 0 | 1 | 2 |
| Population | 414 | 0 | 0 | 10 | 103 | 278 | 10 | 3 | 0 | 0 | 4 | 6 |
| **Other** | | | | | | | | | | | | |
| Households | 19,286 | 10,972 | 3,785 | 423 | 759 | 2,820 | 11 | 43 | 317 | 9 | 40 | 107 |
| Population | 98,081 | 54,521 | 20,533 | 2,297 | 3,455 | 15,075 | 45 | 192 | 1,323 | 28 | 131 | 481 |

## Table H12: Number of Households and Population (Dejure) by Type of Housing Unit and Source of Water Supply, Rural, Zambia 2010

| Source of Household Water Supply, Households and Population | Total | Type of Housing Unit | | | | | | | | | | |
|---|---|---|---|---|---|---|---|---|---|---|---|---|
| | | Traditional | Improved Traditional | Mixed | Conventional Flat | Conventional House | Mobile | Part of commercial building | Improvised/ Makeshift | Collective/ Institutional quarters | Unintended | Other |
| **Zambia Rural** | | | | | | | | | | | | |
| Households | 1,495,861 | 1,005,889 | 364,257 | 24,744 | 15,731 | 65,436 | 1,028 | 4,529 | 7,680 | 1,391 | 2,032 | 3,144 |
| Population | 7,919,216 | 5,172,528 | 2,061,570 | 140,370 | 77,297 | 377,287 | 4,288 | 22,010 | 34,472 | 6,150 | 8,408 | 14,836 |
| **Piped water inside the housing unit** | | | | | | | | | | | | |
| Households | 7,742 | 0 | 0 | 160 | 1,644 | 5,381 | 4 | 32 | 0 | 115 | 25 | 381 |
| Population | 41,137 | 0 | 0 | 829 | 8,517 | 29,304 | 14 | 159 | 0 | 490 | 80 | 1,744 |
| **Piped water outside housing unit within stand/plot** | | | | | | | | | | | | |
| Households | 14,316 | 1,385 | 3,379 | 696 | 2,420 | 5,875 | 64 | 140 | 65 | 74 | 103 | 115 |
| Population | 73,538 | 6,407 | 18,039 | 3,700 | 11,213 | 31,701 | 208 | 640 | 304 | 282 | 401 | 643 |
| **Communal tap** | | | | | | | | | | | | |
| Households | 33,301 | 12,137 | 10,834 | 1,382 | 2,981 | 5,144 | 86 | 210 | 208 | 105 | 108 | 106 |
| Population | 164,077 | 56,854 | 55,692 | 7,143 | 13,689 | 27,029 | 320 | 910 | 981 | 433 | 437 | 589 |
| **Protected well** | | | | | | | | | | | | |
| Households | 165,039 | 102,353 | 48,923 | 3,269 | 1,312 | 7,618 | 56 | 519 | 486 | 118 | 214 | 171 |
| Population | 883,943 | 530,343 | 276,152 | 18,793 | 6,498 | 44,865 | 251 | 2,454 | 2,380 | 495 | 902 | 810 |
| **Protected borehole** | | | | | | | | | | | | |
| Households | 362,968 | 225,248 | 95,083 | 8,051 | 4,157 | 24,694 | 379 | 2,317 | 1,027 | 599 | 711 | 702 |
| Population | 1,953,826 | 1,168,651 | 548,591 | 45,769 | 20,729 | 143,392 | 1,666 | 11,270 | 4,892 | 2,798 | 2,852 | 3,216 |
| **Unprotected well** | | | | | | | | | | | | |
| Households | 432,209 | 314,893 | 98,988 | 5,396 | 1,299 | 7,890 | 195 | 653 | 1,692 | 133 | 404 | 666 |
| Population | 2,304,680 | 1,631,466 | 567,782 | 31,726 | 7,109 | 48,872 | 772 | 3,349 | 8,079 | 564 | 1,782 | 3,179 |
| **Unprotected borehole** | | | | | | | | | | | | |
| Households | 48,041 | 32,496 | 12,292 | 736 | 342 | 1,677 | 33 | 157 | 117 | 49 | 66 | 76 |
| Population | 257,723 | 167,981 | 70,926 | 4,486 | 1,885 | 10,075 | 140 | 791 | 545 | 235 | 295 | 364 |
| **River/Dam/Stream** | | | | | | | | | | | | |
| Households | 406,849 | 301,734 | 88,812 | 4,466 | 971 | 5,236 | 188 | 410 | 3,719 | 148 | 331 | 834 |
| Population | 2,110,261 | 1,532,376 | 491,926 | 24,785 | 4,975 | 31,677 | 834 | 1,993 | 15,775 | 642 | 1,422 | 3,856 |
| **Rain water tank** | | | | | | | | | | | | |
| Households | 3,033 | 2,152 | 703 | 30 | 16 | 75 | 4 | 6 | 35 | 4 | 4 | 4 |
| Population | 15,868 | 11,088 | 3,823 | 173 | 96 | 438 | 8 | 43 | 152 | 18 | 9 | 20 |
| **Other tap** | | | | | | | | | | | | |
| Households | 6,453 | 2,232 | 1,830 | 308 | 467 | 1,379 | 5 | 52 | 53 | 39 | 48 | 40 |
| Population | 32,288 | 11,248 | 9,355 | 1,552 | 2,026 | 7,147 | 20 | 237 | 180 | 168 | 159 | 196 |
| **Water kiosk** | | | | | | | | | | | | |
| Households | 2,187 | 1,117 | 674 | 106 | 64 | 200 | 2 | 11 | 7 | 0 | 4 | 2 |
| Population | 11,434 | 5,680 | 3,608 | 580 | 287 | 1,165 | 7 | 54 | 26 | 0 | 19 | 8 |
| **Water vendor** | | | | | | | | | | | | |
| Households | 275 | 144 | 101 | 0 | 3 | 14 | 1 | 1 | 5 | 1 | 3 | 2 |
| Population | 1,606 | 795 | 662 | 0 | 23 | 74 | 3 | 6 | 18 | 5 | 6 | 14 |
| **Mineral/bottled water** | | | | | | | | | | | | |
| Households | 4 | 0 | 0 | 0 | 0 | 2 | 2 | 0 | 0 | 0 | 0 | 0 |
| Population | 13 | 0 | 0 | 0 | 0 | 4 | 9 | 0 | 0 | 0 | 0 | 0 |
| **Other** | | | | | | | | | | | | |
| Households | 13,444 | 9,998 | 2,638 | 144 | 55 | 251 | 9 | 21 | 266 | 6 | 11 | 45 |
| Population | 68,822 | 49,639 | 15,014 | 834 | 250 | 1,544 | 36 | 104 | 1,140 | 20 | 44 | 197 |

## Table H12: Number of Households and Population (Dejure) by Type of Housing Unit and Source of Water Supply, Urban, Zambia 2010

| Source of Household Water Supply, Households and Population | Total | Type of Housing Unit | | | | | | | | | | |
|---|---|---|---|---|---|---|---|---|---|---|---|---|
| | | Traditional | Improved Traditional | Mixed | Conventional Flat | Conventional House | Mobile | Part of commercial building | Improvised/ Makeshift | Collective/ Institutional quarters | Unintended | Other |
| **Zambia Urban** | | | | | | | | | | | | |
| Households | 1,017,907 | 93,101 | 176,977 | 54,718 | 219,430 | 454,150 | 511 | 5,023 | 4,524 | 2,279 | 2,818 | 4,376 |
| Population | 5,173,450 | 439,800 | 877,694 | 285,437 | 1,007,479 | 2,476,843 | 2,441 | 22,377 | 19,932 | 9,263 | 11,198 | 20,986 |
| **Piped water inside the housing unit** | | | | | | | | | | | | |
| Households | 145,743 | 0 | 0 | 3,721 | 29,033 | 110,722 | 93 | 787 | 0 | 481 | 299 | 607 |
| Population | 803,206 | 0 | 0 | 22,119 | 137,331 | 633,427 | 475 | 3,704 | 0 | 1,818 | 1,215 | 3,117 |
| **Piped water outside housing unit within stand/plot** | | | | | | | | | | | | |
| Households | 236,055 | 2,644 | 19,827 | 16,102 | 53,109 | 138,997 | 167 | 1,261 | 1,056 | 723 | 818 | 1,351 |
| Population | 1,228,470 | 12,621 | 94,868 | 86,039 | 248,748 | 762,640 | 865 | 5,584 | 4,714 | 3,089 | 2,935 | 6,367 |
| **Communal tap** | | | | | | | | | | | | |
| Households | 193,763 | 12,412 | 25,549 | 10,472 | 66,682 | 74,076 | 74 | 1,033 | 1,368 | 566 | 589 | 942 |
| Population | 931,203 | 57,389 | 122,681 | 51,408 | 299,312 | 380,649 | 325 | 4,522 | 5,870 | 2,139 | 2,432 | 4,476 |
| **Protected well** | | | | | | | | | | | | |
| Households | 115,086 | 19,729 | 40,699 | 7,410 | 12,901 | 32,806 | 28 | 407 | 488 | 152 | 171 | 295 |
| Population | 587,651 | 93,368 | 204,970 | 38,662 | 61,013 | 182,515 | 137 | 1,856 | 2,225 | 737 | 738 | 1,430 |
| **Protected borehole** | | | | | | | | | | | | |
| Households | 66,031 | 13,469 | 15,337 | 2,556 | 9,480 | 23,820 | 41 | 384 | 414 | 152 | 222 | 156 |
| Population | 330,929 | 64,517 | 78,023 | 13,121 | 42,899 | 126,567 | 179 | 1,617 | 1,800 | 575 | 888 | 743 |
| **Unprotected well** | | | | | | | | | | | | |
| Households | 105,191 | 22,068 | 37,202 | 5,653 | 14,815 | 24,238 | 30 | 284 | 275 | 36 | 262 | 328 |
| Population | 529,471 | 104,373 | 188,358 | 29,850 | 69,192 | 132,321 | 144 | 1,236 | 1,194 | 172 | 1,066 | 1,565 |
| **Unprotected borehole** | | | | | | | | | | | | |
| Households | 9,160 | 2,295 | 2,689 | 488 | 1,139 | 2,439 | 1 | 38 | 24 | 8 | 17 | 22 |
| Population | 46,303 | 11,067 | 13,830 | 2,567 | 5,196 | 13,139 | 1 | 166 | 124 | 40 | 73 | 100 |
| **River/Dam/Stream** | | | | | | | | | | | | |
| Households | 20,270 | 10,847 | 6,205 | 505 | 825 | 1,666 | 4 | 41 | 112 | 19 | 25 | 21 |
| Population | 101,159 | 52,036 | 32,212 | 2,700 | 3,771 | 9,409 | 13 | 205 | 520 | 71 | 136 | 86 |
| **Rain water tank** | | | | | | | | | | | | |
| Households | 515 | 125 | 128 | 14 | 100 | 141 | 0 | 1 | 1 | 1 | 3 | 1 |
| Population | 2,553 | 645 | 579 | 72 | 482 | 743 | 0 | 2 | 6 | 4 | 13 | 7 |
| **Other tap** | | | | | | | | | | | | |
| Households | 53,792 | 2,803 | 8,268 | 2,944 | 13,858 | 24,472 | 47 | 282 | 467 | 126 | 247 | 278 |
| Population | 264,073 | 12,609 | 39,623 | 15,034 | 62,598 | 127,811 | 199 | 1,200 | 2,048 | 551 | 1,071 | 1,329 |
| **Water kiosk** | | | | | | | | | | | | |
| Households | 60,997 | 5,488 | 19,320 | 4,266 | 15,011 | 15,791 | 19 | 454 | 249 | 12 | 106 | 281 |
| Population | 293,528 | 25,291 | 94,257 | 20,901 | 66,081 | 81,813 | 82 | 2,085 | 1,151 | 59 | 441 | 1,367 |
| **Water vendor** | | | | | | | | | | | | |
| Households | 5,373 | 247 | 606 | 306 | 1,745 | 2,359 | 4 | 28 | 19 | 0 | 29 | 30 |
| Population | 25,244 | 1,002 | 2,774 | 1,491 | 7,548 | 12,004 | 11 | 109 | 97 | 0 | 99 | 109 |
| **Mineral/bottled water** | | | | | | | | | | | | |
| Households | 89 | 0 | 0 | 2 | 28 | 54 | 1 | 1 | 0 | 0 | 1 | 2 |
| Population | 401 | 0 | 0 | 10 | 103 | 274 | 1 | 3 | 0 | 0 | 4 | 6 |
| Other | | | | | | | | | | | | |
| Households | 5,842 | 974 | 1,147 | 279 | 704 | 2,569 | 2 | 22 | 51 | 3 | 29 | 62 |
| Population | 29,259 | 4,882 | 5,519 | 1,463 | 3,205 | 13,531 | 9 | 88 | 183 | 8 | 87 | 284 |

## Table H13: Number of Households and Population by Type of Housing Unit and Source of Drinking Water, Rural/Urban, Zambia 2010

| Source of Household Drinking Water, Households and Population | Total | Type of Housing Unit | | | | | | | | | | |
|---|---|---|---|---|---|---|---|---|---|---|---|---|
| | | Traditional | Improved Traditional | Mixed | Conventional Flat | Conventional House | Mobile | Part of commercial building | Improvised/ Makeshift | Collective/ Institutional quarters | Unintended | Other |
| **Zambia Total** | | | | | | | | | | | | |
| Households | 2,513,768 | 1,098,990 | 541,234 | 79,462 | 235,161 | 519,586 | 1,539 | 9,552 | 12,204 | 3,670 | 4,850 | 7,520 |
| Population | 13,092,666 | 5,612,328 | 2,939,264 | 425,807 | 1,084,776 | 2,854,130 | 6,729 | 44,387 | 54,404 | 15,413 | 19,606 | 35,822 |
| **Piped water inside the housing unit** | | | | | | | | | | | | |
| Households | 154,800 | 0 | 0 | 3,897 | 30,442 | 117,677 | 95 | 811 | 0 | 591 | 318 | 969 |
| Population | 855,143 | 0 | 0 | 23,203 | 145,008 | 674,216 | 487 | 3,838 | 0 | 2,295 | 1,270 | 4,826 |
| **Piped water outside housing unit within stand/plot** | | | | | | | | | | | | |
| Households | 248,500 | 4,293 | 23,471 | 16,774 | 55,286 | 142,778 | 231 | 1,397 | 1,110 | 790 | 922 | 1,448 |
| Population | 1,288,625 | 20,350 | 114,105 | 89,427 | 258,117 | 780,791 | 1,080 | 6,187 | 4,972 | 3,341 | 3,361 | 6,894 |
| Communal tap | | | | | | | | | | | | |
| Households | 254,781 | 26,173 | 40,023 | 12,681 | 80,440 | 89,652 | 167 | 1,327 | 1,729 | 684 | 781 | 1,124 |
| Population | 1,233,939 | 122,173 | 197,329 | 62,796 | 363,037 | 463,264 | 689 | 5,807 | 7,535 | 2,652 | 3,232 | 5,425 |
| **Protected well** | | | | | | | | | | | | |
| Households | 257,573 | 122,392 | 83,798 | 9,380 | 8,082 | 31,156 | 77 | 815 | 818 | 265 | 352 | 438 |
| Population | 1,355,961 | 625,430 | 451,397 | 50,528 | 38,977 | 176,873 | 341 | 3,750 | 3,863 | 1,218 | 1,478 | 2,106 |
| **Protected borehole** | | | | | | | | | | | | |
| Households | 453,387 | 248,825 | 115,910 | 11,186 | 16,387 | 53,644 | 443 | 2,830 | 1,520 | 772 | 974 | 896 |
| Population | 2,419,075 | 1,287,810 | 658,109 | 62,272 | 77,667 | 299,140 | 1,971 | 13,522 | 7,068 | 3,429 | 3,896 | 4,191 |
| **Unprotected well** | | | | | | | | | | | | |
| Households | 497,465 | 336,410 | 124,779 | 9,230 | 4,007 | 18,511 | 202 | 748 | 1,983 | 147 | 559 | 889 |
| Population | 2,629,209 | 1,731,891 | 696,536 | 51,818 | 19,920 | 107,787 | 782 | 3,761 | 9,404 | 642 | 2,433 | 4,235 |
| **Unprotected borehole** | | | | | | | | | | | | |
| Households | 55,677 | 35,001 | 14,406 | 1,134 | 1,046 | 3,493 | 34 | 195 | 150 | 51 | 75 | 92 |
| Population | 296,177 | 180,176 | 81,888 | 6,517 | 5,027 | 19,742 | 136 | 968 | 702 | 259 | 330 | 432 |
| **River/Dam/Stream** | | | | | | | | | | | | |
| Households | 409,597 | 299,003 | 92,743 | 4,696 | 1,365 | 6,217 | 182 | 407 | 3,685 | 162 | 309 | 828 |
| Population | 2,116,712 | 1,513,229 | 509,847 | 25,857 | 6,668 | 36,891 | 806 | 1,974 | 15,611 | 676 | 1,331 | 3,822 |
| **Rain water tank** | | | | | | | | | | | | |
| Households | 3,459 | 2,160 | 822 | 43 | 145 | 228 | 5 | 8 | 32 | 4 | 7 | 5 |
| Population | 18,006 | 11,137 | 4,406 | 248 | 700 | 1,248 | 9 | 47 | 144 | 18 | 22 | 27 |
| **Other tap** | | | | | | | | | | | | |
| Households | 67,591 | 5,586 | 11,740 | 3,672 | 16,016 | 28,750 | 51 | 359 | 570 | 171 | 326 | 350 |
| Population | 334,667 | 26,675 | 57,310 | 19,022 | 72,798 | 150,870 | 222 | 1,537 | 2,421 | 753 | 1,362 | 1,697 |
| **Water kiosk** | | | | | | | | | | | | |
| Households | 84,942 | 9,274 | 28,756 | 5,949 | 18,775 | 20,846 | 25 | 557 | 288 | 18 | 147 | 307 |
| Population | 416,282 | 43,996 | 142,888 | 29,915 | 83,439 | 109,819 | 109 | 2,583 | 1,344 | 88 | 627 | 1,474 |
| **Water vendor** | | | | | | | | | | | | |
| Households | 6,498 | 455 | 879 | 354 | 2,013 | 2,659 | 5 | 35 | 26 | 1 | 33 | 38 |
| Population | 31,144 | 2,119 | 4,319 | 1,763 | 8,771 | 13,642 | 14 | 141 | 120 | 5 | 108 | 142 |
| **Mineral/bottled water** | | | | | | | | | | | | |
| Households | 1,717 | 34 | 79 | 33 | 400 | 1,098 | 11 | 19 | 3 | 7 | 5 | 28 |
| Population | 6,473 | 154 | 331 | 112 | 1,241 | 4,426 | 31 | 76 | 8 | 14 | 15 | 65 |
| **Other** | | | | | | | | | | | | |
| Households | 17,781 | 9,384 | 3,828 | 433 | 757 | 2,877 | 11 | 44 | 290 | 7 | 42 | 108 |
| Population | 91,253 | 47,188 | 20,799 | 2,329 | 3,406 | 15,421 | 52 | 196 | 1,212 | 23 | 141 | 486 |

## Table H13: Number of Households and Population by Type of Housing Unit and Source of Drinking Water, Rural, Zambia 2010

| Source of Household Drinking Water, Households and Population | Total | Type of Housing Unit | | | | | | | | | | |
|---|---|---|---|---|---|---|---|---|---|---|---|---|
| | | Traditional | Improved Traditional | Mixed | Conventional Flat | Conventional House | Mobile | Part of commercial building | Improvised/ Makeshift | Collective/ Institutional quarters | Unintended | Other |
| **Zambia Rural** | | | | | | | | | | | | |
| Households | 1,495,861 | 1,005,889 | 364,257 | 24,744 | 15,731 | 65,436 | 1,028 | 4,529 | 7,680 | 1,391 | 2,032 | 3,144 |
| Population | 7,919,216 | 5,172,528 | 2,061,570 | 140,370 | 77,297 | 377,287 | 4,288 | 22,010 | 34,472 | 6,150 | 8,408 | 14,836 |
| **Piped water inside the housing unit** | | | | | | | | | | | | |
| Households | 7,654 | 0 | 0 | 155 | 1,619 | 5,352 | 4 | 31 | 0 | 112 | 22 | 359 |
| Population | 40,673 | 0 | 0 | 806 | 8,390 | 29,079 | 14 | 143 | 0 | 480 | 69 | 1,692 |
| **Piped water outside housing unit within stand/plot** | | | | | | | | | | | | |
| Households | 13,985 | 1,508 | 3,352 | 661 | 2,320 | 5,581 | 63 | 144 | 67 | 73 | 104 | 112 |
| Population | 71,472 | 7,026 | 17,799 | 3,487 | 10,709 | 29,973 | 207 | 649 | 312 | 275 | 413 | 622 |
| **Communal tap** | | | | | | | | | | | | |
| Households | 34,864 | 12,775 | 11,543 | 1,422 | 2,980 | 5,304 | 86 | 211 | 213 | 104 | 114 | 112 |
| Population | 172,636 | 60,166 | 59,728 | 7,360 | 13,690 | 27,946 | 320 | 910 | 1,002 | 430 | 460 | 624 |
| **Protected well** | | | | | | | | | | | | |
| Households | 166,428 | 104,064 | 48,865 | 3,269 | 1,281 | 7,387 | 54 | 497 | 501 | 121 | 212 | 177 |
| Population | 890,800 | 538,945 | 275,844 | 18,742 | 6,459 | 43,590 | 236 | 2,326 | 2,423 | 513 | 877 | 845 |
| **Protected borehole** | | | | | | | | | | | | |
| Households | 378,822 | 234,931 | 99,407 | 8,415 | 4,402 | 25,719 | 395 | 2,399 | 1,081 | 610 | 740 | 723 |
| Population | 2,042,624 | 1,221,098 | 574,287 | 48,036 | 21,927 | 149,472 | 1,764 | 11,710 | 5,159 | 2,824 | 2,992 | 3,355 |
| **Unprotected well** | | | | | | | | | | | | |
| Households | 430,113 | 316,635 | 96,118 | 5,216 | 1,173 | 7,224 | 186 | 610 | 1,752 | 128 | 415 | 656 |
| Population | 2,289,626 | 1,637,983 | 551,618 | 30,623 | 6,520 | 45,175 | 713 | 3,141 | 8,356 | 559 | 1,853 | 3,085 |
| **Unprotected borehole** | | | | | | | | | | | | |
| Households | 47,975 | 32,769 | 12,029 | 715 | 342 | 1,615 | 33 | 163 | 125 | 43 | 63 | 78 |
| Population | 257,266 | 169,345 | 69,609 | 4,375 | 1,842 | 9,696 | 135 | 828 | 581 | 216 | 276 | 363 |
| **River/Dam/Stream** | | | | | | | | | | | | |
| Households | 390,347 | 288,320 | 86,399 | 4,260 | 961 | 5,006 | 180 | 381 | 3,593 | 146 | 288 | 813 |
| Population | 2,021,390 | 1,462,446 | 476,955 | 23,491 | 4,912 | 30,136 | 801 | 1,851 | 15,197 | 625 | 1,209 | 3,767 |
| **Rain water tank** | | | | | | | | | | | | |
| Households | 2,893 | 2,038 | 691 | 26 | 17 | 67 | 5 | 6 | 31 | 4 | 4 | 4 |
| Population | 15,166 | 10,502 | 3,798 | 154 | 88 | 387 | 9 | 43 | 138 | 18 | 9 | 20 |
| Other tap | | | | | | | | | | | | |
| Households | 7,104 | 2,415 | 2,038 | 326 | 488 | 1,585 | 5 | 52 | 62 | 41 | 50 | 42 |
| Population | 35,851 | 12,214 | 10,424 | 1,662 | 2,112 | 8,407 | 20 | 237 | 210 | 182 | 171 | 212 |
| **Water kiosk** | | | | | | | | | | | | |
| Households | 2,776 | 1,350 | 924 | 141 | 72 | 261 | 2 | 13 | 7 | 0 | 4 | 2 |
| Population | 14,637 | 6,948 | 4,977 | 807 | 328 | 1,458 | 7 | 59 | 26 | 0 | 19 | 8 |
| **Water vendor** | | | | | | | | | | | | |
| Households | 309 | 161 | 116 | 1 | 3 | 15 | 1 | 1 | 5 | 1 | 3 | 2 |
| Population | 1,763 | 868 | 732 | 4 | 23 | 84 | 3 | 6 | 18 | 5 | 6 | 14 |
| **Mineral/bottled water** | | | | | | | | | | | | |
| Households | 130 | 23 | 17 | 4 | 12 | 42 | 5 | 1 | 1 | 4 | 0 | 21 |
| Population | 440 | 100 | 75 | 13 | 30 | 155 | 16 | 4 | 5 | 8 | 0 | 34 |
| **Other** | | | | | | | | | | | | |
| Households | 12,461 | 8,900 | 2,758 | 133 | 61 | 278 | 9 | 20 | 242 | 4 | 13 | 43 |
| Population | 64,872 | 44,887 | 15,724 | 810 | 267 | 1,729 | 43 | 103 | 1,045 | 15 | 54 | 195 |

## Table H13: Total Number of Households and Population by Type of Housing Unit and Source of Drinking Water, Urban, Zambia 2010

| Source of Household Drinking Water, Households and Population | Total | Type of Housing Unit | | | | | | | | | | |
|---|---|---|---|---|---|---|---|---|---|---|---|---|
| | | Traditional | Improved Traditional | Mixed | Conventional Flat | Conventional House | Mobile | Part of commercial building | Improvised/ Makeshift | Collective/ Institutional quarters | Unintended | Other |
| **Zambia Urban** | | | | | | | | | | | | |
| Households | 1,017,907 | 93,101 | 176,977 | 54,718 | 219,430 | 454,150 | 511 | 5,023 | 4,524 | 2,279 | 2,818 | 4,376 |
| Population | 5,173,450 | 439,800 | 877,694 | 285,437 | 1,007,479 | 2,476,843 | 2,441 | 22,377 | 19,932 | 9,263 | 11,198 | 20,986 |
| **Piped water inside the housing unit** | | | | | | | | | | | | |
| Households | 147,146 | 0 | 0 | 3,742 | 28,823 | 112,325 | 91 | 780 | 0 | 479 | 296 | 610 |
| Population | 814,470 | 0 | 0 | 22,397 | 136,618 | 645,137 | 473 | 3,695 | 0 | 1,815 | 1,201 | 3,134 |
| **Piped water outside housing unit within stand/plot** | | | | | | | | | | | | |
| Households | 234,515 | 2,785 | 20,119 | 16,113 | 52,966 | 137,197 | 168 | 1,253 | 1,043 | 717 | 818 | 1,336 |
| Population | 1,217,153 | 13,324 | 96,306 | 85,940 | 247,408 | 750,818 | 873 | 5,538 | 4,660 | 3,066 | 2,948 | 6,272 |
| **Communal tap** | | | | | | | | | | | | |
| Households | 219,917 | 13,398 | 28,480 | 11,259 | 77,460 | 84,348 | 81 | 1,116 | 1,516 | 580 | 667 | 1,012 |
| Population | 1,061,303 | 62,007 | 137,601 | 55,436 | 349,347 | 435,318 | 369 | 4,897 | 6,533 | 2,222 | 2,772 | 4,801 |
| **Protected well** | | | | | | | | | | | | |
| Households | 91,145 | 18,328 | 34,933 | 6,111 | 6,801 | 23,769 | 23 | 318 | 317 | 144 | 140 | 261 |
| Population | 465,161 | 86,485 | 175,553 | 31,786 | 32,518 | 133,283 | 105 | 1,424 | 1,440 | 705 | 601 | 1,261 |
| **Protected borehole** | | | | | | | | | | | | |
| Households | 74,565 | 13,894 | 16,503 | 2,771 | 11,985 | 27,925 | 48 | 431 | 439 | 162 | 234 | 173 |
| Population | 376,451 | 66,712 | 83,822 | 14,236 | 55,740 | 149,668 | 207 | 1,812 | 1,909 | 605 | 904 | 836 |
| **Unprotected well** | | | | | | | | | | | | |
| Households | 67,352 | 19,775 | 28,661 | 4,014 | 2,834 | 11,287 | 16 | 138 | 231 | 19 | 144 | 233 |
| Population | 339,583 | 93,908 | 144,918 | 21,195 | 13,400 | 62,612 | 69 | 620 | 1,048 | 83 | 580 | 1,150 |
| **Unprotected borehole** | | | | | | | | | | | | |
| Households | 7,702 | 2,232 | 2,377 | 419 | 704 | 1,878 | 1 | 32 | 25 | 8 | 12 | 14 |
| Population | 38,911 | 10,831 | 12,279 | 2,142 | 3,185 | 10,046 | 1 | 140 | 121 | 43 | 54 | 69 |
| **River/Dam/Stream** | | | | | | | | | | | | |
| Households | 19,250 | 10,683 | 6,344 | 436 | 404 | 1,211 | 2 | 26 | 92 | 16 | 21 | 15 |
| Population | 95,322 | 50,783 | 32,892 | 2,366 | 1,756 | 6,755 | 5 | 123 | 414 | 51 | 122 | 55 |
| **Rain water tank** | | | | | | | | | | | | |
| Households | 566 | 122 | 131 | 17 | 128 | 161 | 0 | 2 | 1 | 0 | 3 | 1 |
| Population | 2,840 | 635 | 608 | 94 | 612 | 861 | 0 | 4 | 6 | 0 | 13 | 7 |
| **Other tap** | | | | | | | | | | | | |
| Households | 60,487 | 3,171 | 9,702 | 3,346 | 15,528 | 27,165 | 46 | 307 | 508 | 130 | 276 | 308 |
| Population | 298,816 | 14,461 | 46,886 | 17,360 | 70,686 | 142,463 | 202 | 1,300 | 2,211 | 571 | 1,191 | 1,485 |
| **Water kiosk** | | | | | | | | | | | | |
| Households | 82,166 | 7,924 | 27,832 | 5,808 | 18,703 | 20,585 | 23 | 544 | 281 | 18 | 143 | 305 |
| Population | 401,645 | 37,048 | 137,911 | 29,108 | 83,111 | 108,361 | 102 | 2,524 | 1,318 | 88 | 608 | 1,466 |
| **Water vendor** | | | | | | | | | | | | |
| Households | 6,189 | 294 | 763 | 353 | 2,010 | 2,644 | 4 | 34 | 21 | 0 | 30 | 36 |
| Population | 29,381 | 1,251 | 3,587 | 1,759 | 8,748 | 13,558 | 11 | 135 | 102 | 0 | 102 | 128 |
| **Mineral/bottled water** | | | | | | | | | | | | |
| Households | 1,587 | 11 | 62 | 29 | 388 | 1,056 | 6 | 18 | 2 | 3 | 5 | 7 |
| Population | 6,033 | 54 | 256 | 99 | 1,211 | 4,271 | 15 | 72 | 3 | 6 | 15 | 31 |
| **Other** | | | | | | | | | | | | |
| Households | 5,320 | 484 | 1,070 | 300 | 696 | 2,599 | 2 | 24 | 48 | 3 | 29 | 65 |
| Population | 26,381 | 2,301 | 5,075 | 1,519 | 3,139 | 13,692 | 9 | 93 | 167 | 8 | 87 | 291 |

## Table H14: Number of Households by Ownership of Household Assets and Type of Housing Unit, Rural/Urban, Zambia 2010

| Household Assets and Rural/Urban | Total Number of Households | Type of Housing Unit | | | | | | | | | | |
|---|---|---|---|---|---|---|---|---|---|---|---|---|
| | | Traditional | Improved Traditional | Mixed | Conventional Flat | Conventional House | Mobile | Part of Commercial Building | Improvised/ Makeshift | Collective/ Institutional Quarters | Unintended | Other |
| **Zambia** | | | | | | | | | | | | |
| **Total** | **2,513,768** | **1,098,990** | **541,234** | **79,462** | **235,161** | **519,586** | **1,539** | **9,552** | **12,204** | **3,670** | **4,850** | **7,520** |
| Rural | 1,495,861 | 1,005,889 | 364,257 | 24,744 | 15,731 | 65,436 | 1,028 | 4,529 | 7,680 | 1,391 | 2,032 | 3,144 |
| Urban | 1,017,907 | 93,101 | 176,977 | 54,718 | 219,430 | 454,150 | 511 | 5,023 | 4,524 | 2,279 | 2,818 | 4,376 |
| **Radio** | | | | | | | | | | | | |
| **Total** | **1,465,729** | **499,103** | **320,929** | **52,610** | **168,264** | **400,930** | **932** | **6,961** | **5,784** | **2,673** | **2,730** | **4,813** |
| Rural | 760,047 | 454,133 | 215,391 | 16,287 | 11,474 | 51,848 | 565 | 3,346 | 3,297 | 973 | 1,033 | 1,700 |
| Urban | 705,682 | 44,970 | 105,538 | 36,323 | 156,790 | 349,082 | 367 | 3,615 | 2,487 | 1,700 | 1,697 | 3,113 |
| **Television** | | | | | | | | | | | | |
| **Total** | **770,672** | **55,343** | **121,077** | **37,097** | **161,031** | **380,413** | **510** | **4,879** | **2,358** | **2,370** | **1,817** | **3,777** |
| Rural | 169,975 | 45,939 | 64,531 | 7,050 | 9,037 | 38,884 | 185 | 1,575 | 650 | 698 | 494 | 932 |
| Urban | 600,697 | 9,404 | 56,546 | 30,047 | 151,994 | 341,529 | 325 | 3,304 | 1,708 | 1,672 | 1,323 | 2,845 |
| **Fridge** | | | | | | | | | | | | |
| **Total** | **400,384** | **3,766** | **19,672** | **16,296** | **94,097** | **258,349** | **276** | **2,469** | **840** | **1,528** | **833** | **2,258** |
| Rural | 32,182 | 2,700 | 6,270 | 1,299 | 4,458 | 15,967 | 48 | 397 | 130 | 276 | 136 | 501 |
| Urban | 368,202 | 1,066 | 13,402 | 14,997 | 89,639 | 242,382 | 228 | 2,072 | 710 | 1,252 | 697 | 1,757 |
| **Telephone** | | | | | | | | | | | | |
| **Total** | **39,075** | **4,253** | **3,353** | **1,277** | **5,768** | **23,768** | **24** | **260** | **72** | **100** | **39** | **161** |
| Rural | 8,897 | 3,916 | 2,376 | 226 | 369 | 1,862 | 5 | 45 | 29 | 21 | 14 | 34 |
| Urban | 30,178 | 337 | 977 | 1,051 | 5,399 | 21,906 | 19 | 215 | 43 | 79 | 25 | 127 |
| **Bicycle** | | | | | | | | | | | | |
| **Total** | **958,543** | **470,619** | **264,903** | **26,496** | **38,986** | **145,781** | **448** | **4,130** | **3,138** | **1,011** | **1,193** | **1,838** |
| Rural | 713,955 | 438,641 | 211,209 | 13,890 | 6,080 | 36,354 | 336 | 2,809 | 2,367 | 571 | 741 | 957 |
| Urban | 244,588 | 31,978 | 53,694 | 12,606 | 32,906 | 109,427 | 112 | 1,321 | 771 | 440 | 452 | 881 |
| **Motor Vehicle** | | | | | | | | | | | | |
| **Total** | **120,742** | **2,665** | **9,176** | **3,660** | **21,770** | **81,228** | **118** | **897** | **177** | **336** | **171** | **544** |
| Rural | 18,688 | 2,397 | 6,158 | 724 | 1,250 | 7,498 | 45 | 269 | 65 | 84 | 48 | 150 |
| Urban | 102,054 | 268 | 3,018 | 2,936 | 20,520 | 73,730 | 73 | 628 | 112 | 252 | 123 | 394 |
| **Internet** | | | | | | | | | | | | |
| **Total** | **33,787** | **808** | **1,736** | **860** | **6,735** | **22,835** | **55** | **255** | **36** | **139** | **78** | **250** |
| Rural | 5,057 | 725 | 1,055 | 181 | 445 | 2,404 | 27 | 48 | 10 | 39 | 29 | 94 |
| Urban | 28,730 | 83 | 681 | 679 | 6,290 | 20,431 | 28 | 207 | 26 | 100 | 49 | 156 |
| **Computer/ Laptop** | | | | | | | | | | | | |
| **Total** | **92,980** | **369** | **2,658** | **2,233** | **19,654** | **66,212** | **102** | **519** | **113** | **438** | **167** | **515** |
| Rural | 7,971 | 296 | 1,148 | 244 | 1,008 | 4,868 | 36 | 51 | 23 | 102 | 35 | 160 |
| Urban | 85,009 | 73 | 1,510 | 1,989 | 18,646 | 61,344 | 66 | 468 | 90 | 336 | 132 | 355 |
| **Motorcycle** | | | | | | | | | | | | |
| **Total** | **16,448** | **2,427** | **4,170** | **646** | **1,195** | **7,586** | **13** | **157** | **44** | **90** | **28** | **92** |
| Rural | 9,761 | 2,239 | 3,504 | 396 | 314 | 3,042 | 12 | 85 | 31 | 69 | 21 | 48 |
| Urban | 6,687 | 188 | 666 | 250 | 881 | 4,544 | 1 | 72 | 13 | 21 | 7 | 44 |
| **Plough** | | | | | | | | | | | | |
| **Total** | **233,434** | **132,609** | **74,284** | **6,037** | **2,092** | **16,131** | **56** | **906** | **690** | **82** | **182** | **365** |
| Rural | 224,102 | 131,014 | 72,275 | 5,547 | 1,308 | 11,864 | 51 | 843 | 663 | 72 | 165 | 300 |
| Urban | 9,332 | 1,595 | 2,009 | 490 | 784 | 4,267 | 5 | 63 | 27 | 10 | 17 | 65 |
| **Boat/Canoe** | | | | | | | | | | | | |
| **Total** | **95,227** | **63,822** | **25,537** | **1,100** | **365** | **2,027** | **53** | **95** | **1,700** | **26** | **77** | **425** |
| Rural | 90,217 | 61,803 | 23,770 | 972 | 203 | 1,149 | 51 | 82 | 1,684 | 22 | 72 | 409 |
| Urban | 5,010 | 2,019 | 1,767 | 128 | 162 | 878 | 2 | 13 | 16 | 4 | 5 | 16 |

## Table H14: Number of Households by Ownership of Household Assets and Type of Housing Unit, Rural/Urban, Zambia 2010

| Household Assets and Rural/Urban | Total Number of Households | Type of Housing Unit | | | | | | | | | | |
|---|---|---|---|---|---|---|---|---|---|---|---|---|
| | | Traditional | Improved Traditional | Mixed | Conventional Flat | Conventional House | Mobile | Part of Commercial Building | Improvised/ Makeshift | Collective/ Institutional Quarters | Unintended | Other |
| **Scotch Cart** | | | | | | | | | | | | |
| **Total** | **82,563** | **38,774** | **31,501** | **2,649** | **961** | **7,742** | **30** | **464** | **217** | **38** | **66** | **121** |
| Rural | 79,378 | 38,218 | 30,677 | 2,470 | 694 | 6,456 | 26 | 438 | 201 | 31 | 61 | 106 |
| Urban | 3,185 | 556 | 824 | 179 | 267 | 1,286 | 4 | 26 | 16 | 7 | 5 | 15 |
| **Donkey** | | | | | | | | | | | | |
| **Total** | **7,273** | **3,990** | **1,936** | **221** | **226** | **812** | **5** | **34** | **23** | **8** | **7** | **11** |
| Rural | 6,307 | 3,893 | 1,748 | 162 | 40 | 402 | 4 | 22 | 20 | 6 | 6 | 4 |
| Urban | 966 | 97 | 188 | 59 | 186 | 410 | 1 | 12 | 3 | 2 | 1 | 7 |
| **Mobile Phone** | | | | | | | | | | | | |
| **Total** | **1,311,089** | **286,408** | **284,032** | **57,380** | **202,208** | **456,272** | **910** | **7,428** | **5,446** | **2,887** | **3,329** | **4,789** |
| Rural | 491,575 | 242,556 | 161,854 | 13,730 | 12,111 | 51,929 | 503 | 3,227 | 2,249 | 954 | 1,121 | 1,341 |
| Urban | 819,514 | 43,852 | 122,178 | 43,650 | 190,097 | 404,343 | 407 | 4,201 | 3,197 | 1,933 | 2,208 | 3,448 |
| **Oxen** | | | | | | | | | | | | |
| **Total** | **185,798** | **105,978** | **56,984** | **4,827** | **2,399** | **13,672** | **52** | **724** | **605** | **89** | **143** | **325** |
| Rural | 175,162 | 104,313 | 54,630 | 4,094 | 1,093 | 9,275 | 48 | 667 | 576 | 67 | 119 | 280 |
| Urban | 10,636 | 1,665 | 2,354 | 733 | 1,306 | 4,397 | 4 | 57 | 29 | 22 | 24 | 45 |
| **Wheelbarrow** | | | | | | | | | | | | |
| **Total** | **228,839** | **27,669** | **46,266** | **10,664** | **26,267** | **113,697** | **148** | **1,532** | **819** | **360** | **474** | **943** |
| Rural | 67,902 | 23,269 | 26,307 | 2,616 | 1,602 | 12,818 | 54 | 518 | 264 | 87 | 153 | 214 |
| Urban | 160,937 | 4,400 | 19,959 | 8,048 | 24,665 | 100,879 | 94 | 1,014 | 555 | 273 | 321 | 729 |

## Table H15: Occupied Housing Units by Type of Housing Unit and How the Housing Units were Acquired, Rural/Urban, Zambia 2010

| Type of Housing Unit and Rural/ Urban | Occupied Housing Units | Total Owned | Housing Units by How they were Acquired | | | | | |
|---|---|---|---|---|---|---|---|---|
| | | | Purchased | Mortgage | Freely | Inherited | Self built | Other |
| **Zambia Total** | **2,494,869** | **1,765,675** | **164,634** | **4,397** | **71,712** | **76,915** | **1,431,124** | **16,893** |
| Traditional | 1,096,115 | 1,002,317 | 29,712 | 0 | 44,385 | 30,676 | 889,336 | 8,208 |
| Improved Traditional | 538,669 | 417,653 | 22,497 | 0 | 14,914 | 16,699 | 359,803 | 3,740 |
| Mixed | 78,743 | 43,780 | 8,479 | 320 | 1,726 | 3,227 | 29,493 | 535 |
| Conventional Flat | 232,473 | 52,337 | 18,806 | 544 | 2,461 | 7,141 | 22,345 | 1,040 |
| Conventional House | 510,076 | 229,759 | 83,320 | 3,338 | 7,023 | 18,139 | 115,167 | 2,772 |
| Mobile | 1,523 | 778 | 74 | 1 | 287 | 14 | 334 | 68 |
| Part of commercial building | 9,426 | 4,943 | 485 | 23 | 269 | 361 | 3,717 | 88 |
| Improvised/Makeshift | 12,070 | 8,523 | 475 | 0 | 304 | 332 | 7,246 | 166 |
| Collective/Institutional quarters | 3,606 | 480 | 76 | 13 | 56 | 33 | 272 | 30 |
| Unintended | 4,753 | 1,434 | 135 | 2 | 143 | 115 | 979 | 60 |
| Other | 7,415 | 3,671 | 575 | 156 | 144 | 178 | 2,432 | 186 |
| | | | | | | | | |
| **Zambia Rural** | **1,490,802** | **1,315,458** | **34,214** | **736** | **56,485** | **39,311** | **1,173,291** | **11,421** |
| Traditional | 1,003,283 | 933,553 | 22,951 | 0 | 41,432 | 27,096 | 834,690 | 7,384 |
| Improved Traditional | 362,906 | 316,724 | 8,522 | 0 | 11,146 | 9,304 | 285,119 | 2,633 |
| Mixed | 24,630 | 18,717 | 426 | 37 | 793 | 673 | 16,557 | 231 |
| Conventional Flat | 15,474 | 3,799 | 352 | 81 | 389 | 241 | 2,610 | 126 |
| Conventional House | 64,855 | 29,371 | 1,603 | 452 | 1,894 | 1,666 | 23,106 | 650 |
| Mobile | 1,023 | 607 | 23 | 1 | 263 | 1 | 260 | 59 |
| Part of commercial building | 4,496 | 3,072 | 159 | 11 | 145 | 145 | 2,573 | 39 |
| Improvised/Makeshift | 7,654 | 6,623 | 99 | 0 | 204 | 91 | 6,100 | 129 |
| Collective/Institutional quarters | 1,359 | 182 | 7 | 7 | 40 | 8 | 100 | 20 |
| Unintended | 2,005 | 868 | 24 | 2 | 83 | 42 | 668 | 49 |
| Other | 3,117 | 1,942 | 48 | 145 | 96 | 44 | 1,508 | 101 |
| | | | | | | | | |
| **Zambia Urban** | **1,004,067** | **450,217** | **130,420** | **3,661** | **15,227** | **37,604** | **257,833** | **5,472** |
| Traditional | 92,832 | 68,764 | 6,761 | 0 | 2,953 | 3,580 | 54,646 | 824 |
| Improved Traditional | 175,763 | 100,929 | 13,975 | 0 | 3,768 | 7,395 | 74,684 | 1,107 |
| Mixed | 54,113 | 25,063 | 8,053 | 283 | 933 | 2,554 | 12,936 | 304 |
| Conventional Flat | 216,999 | 48,538 | 18,454 | 463 | 2,072 | 6,900 | 19,735 | 914 |
| Conventional House | 445,221 | 200,388 | 81,717 | 2,886 | 5,129 | 16,473 | 92,061 | 2,122 |
| Mobile | 500 | 171 | 51 | 0 | 24 | 13 | 74 | 9 |
| Part of commercial building | 4,930 | 1,871 | 326 | 12 | 124 | 216 | 1,144 | 49 |
| Improvised/Makeshift | 4,416 | 1,900 | 376 | 0 | 100 | 241 | 1,146 | 37 |
| Collective/Institutional quarters | 2,247 | 298 | 69 | 6 | 16 | 25 | 172 | 10 |
| Unintended | 2,748 | 566 | 111 | 0 | 60 | 73 | 311 | 11 |
| Other | 4,298 | 1,729 | 527 | 11 | 48 | 134 | 924 | 85 |

## Table H16: Occupied Housing Units and Housing Units rented from Employer by Type of Housing Unit and Type of Employer, Rural/Urban, Zambia 2010

| Type of Housing Unit and Rural/Urban | Occupied Housing Units | Housing Units Rented from Employer | Employer from which Housing Unit is Rented | | | | |
|---|---|---|---|---|---|---|---|
| | | | The Central Government | Local Government | Parastatal | A Private Organisation | An Individual |
| **Zambia Total** | **2,494,869** | **27,885** | **4,719** | **1,134** | **1,173** | **4,591** | **16,268** |
| Traditional | 1,096,115 | 2,741 | 72 | 30 | 34 | 151 | 2,454 |
| Improved Traditional | 538,669 | 4,804 | 640 | 236 | 52 | 367 | 3,509 |
| Mixed | 78,743 | 1,114 | 123 | 43 | 23 | 93 | 832 |
| Conventional Flat | 232,473 | 4,778 | 508 | 168 | 290 | 991 | 2,821 |
| Conventional House | 510,076 | 13,726 | 3,253 | 623 | 736 | 2,846 | 6,268 |
| Mobile | 1,523 | 13 | 1 | 0 | 2 | 5 | 5 |
| Part of commercial building | 9,426 | 232 | 7 | 12 | 9 | 31 | 173 |
| Improvised/Makeshift | 12,070 | 92 | 8 | 4 | 5 | 6 | 69 |
| Collective/Institutional quarters | 3,606 | 110 | 63 | 2 | 0 | 24 | 21 |
| Unintended | 4,753 | 102 | 12 | 9 | 1 | 19 | 61 |
| Other | 7,415 | 173 | 32 | 7 | 21 | 58 | 55 |
| | | | | | | | |
| **Zambia Rural** | **1,490,802** | **7,196** | **2,228** | **503** | **462** | **1,293** | **2,710** |
| Traditional | 1,003,283 | 1,304 | 61 | 20 | 19 | 118 | 1,086 |
| Improved Traditional | 362,906 | 1,793 | 537 | 201 | 28 | 250 | 777 |
| Mixed | 24,630 | 215 | 79 | 7 | 8 | 17 | 104 |
| Conventional Flat | 15,474 | 698 | 137 | 44 | 97 | 215 | 205 |
| Conventional House | 64,855 | 2,888 | 1,342 | 220 | 304 | 618 | 404 |
| Mobile | 1,023 | 2 | 0 | 0 | 1 | 0 | 1 |
| Part of commercial building | 4,496 | 104 | 5 | 3 | 1 | 9 | 86 |
| Improvised/Makeshift | 7,654 | 19 | 2 | 0 | 4 | 2 | 11 |
| Collective/Institutional quarters | 1,359 | 62 | 50 | 0 | 0 | 8 | 4 |
| Unintended | 2,005 | 38 | 4 | 3 | 0 | 10 | 21 |
| Other | 3,117 | 73 | 11 | 5 | 0 | 46 | 11 |
| | | | | | | | |
| **Zambia Urban** | **1,004,067** | **20,689** | **2,491** | **631** | **711** | **3,298** | **13,558** |
| Traditional | 92,832 | 1,437 | 11 | 10 | 15 | 33 | 1,368 |
| Improved Traditional | 175,763 | 3,011 | 103 | 35 | 24 | 117 | 2,732 |
| Mixed | 54,113 | 899 | 44 | 36 | 15 | 76 | 728 |
| Conventional Flat | 216,999 | 4,080 | 371 | 124 | 193 | 776 | 2,616 |
| Conventional House | 445,221 | 10,838 | 1,911 | 403 | 432 | 2,228 | 5,864 |
| Mobile | 500 | 11 | 1 | 0 | 1 | 5 | 4 |
| Part of commercial building | 4,930 | 128 | 2 | 9 | 8 | 22 | 87 |
| Improvised/Makeshift | 4,416 | 73 | 6 | 4 | 1 | 4 | 58 |
| Collective/Institutional quarters | 2,247 | 48 | 13 | 2 | 0 | 16 | 17 |
| Unintended | 2,748 | 64 | 8 | 6 | 1 | 9 | 40 |
| Other | 4,298 | 100 | 21 | 2 | 21 | 12 | 44 |

## Table H17: Occupied Housing Units and Total Rented Housing Units by Type of Housing Unit and Source of Housing Unit Rented by Households, Rural/Urban, Zambia 2010

| Type of Housing Unit and Rural/Urban | Occupied Housing Units | Total Rented Units | Source of Housing Units Rented by Households | | | | |
|---|---|---|---|---|---|---|---|
| | | | The Central Government | Local Government | Parastatal | A Private Organisation | An Individual |
| **Zambia Total** | **2,494,869** | **487,147** | **3,924** | **2,688** | **2,710** | **5,033** | **472,792** |
| Traditional | 1,096,115 | 39,417 | 117 | 102 | 298 | 248 | 38,652 |
| Improved Traditional | 538,669 | 72,599 | 530 | 549 | 188 | 717 | 70,615 |
| Mixed | 78,743 | 25,075 | 178 | 119 | 24 | 128 | 24,626 |
| Conventional Flat | 232,473 | 148,520 | 647 | 460 | 1,040 | 1,435 | 144,938 |
| Conventional House | 510,076 | 192,792 | 2,083 | 1,347 | 1,098 | 2,255 | 186,009 |
| Mobile | 1,523 | 227 | 13 | 8 | 5 | 23 | 178 |
| Part of commercial building | 9,426 | 2,402 | 21 | 18 | 15 | 63 | 2,285 |
| Improvised/Makeshift | 12,070 | 1,772 | 22 | 9 | 12 | 21 | 1,708 |
| Collective/Institutional quarters | 3,606 | 1,065 | 247 | 26 | 5 | 59 | 728 |
| Unintended | 4,753 | 1,419 | 34 | 34 | 8 | 55 | 1,288 |
| Other | 7,415 | 1,859 | 32 | 16 | 17 | 29 | 1,765 |
| | | | | | | | |
| **Zambia Rural** | **1,490,802** | **46,669** | **1,653** | **1,086** | **1,267** | **2,068** | **40,595** |
| Traditional | 1,003,283 | 22,385 | 108 | 95 | 282 | 206 | 21,694 |
| Improved Traditional | 362,906 | 12,599 | 435 | 453 | 108 | 555 | 11,048 |
| Mixed | 24,630 | 1,434 | 73 | 40 | 12 | 74 | 1,235 |
| Conventional Flat | 15,474 | 2,900 | 184 | 89 | 237 | 305 | 2,085 |
| Conventional House | 64,855 | 6,141 | 751 | 371 | 600 | 818 | 3,601 |
| Mobile | 1,023 | 65 | 10 | 5 | 3 | 19 | 28 |
| Part of commercial building | 4,496 | 451 | 15 | 7 | 2 | 23 | 404 |
| Improvised/Makeshift | 7,654 | 223 | 9 | 2 | 10 | 5 | 197 |
| Collective/Institutional quarters | 1,359 | 106 | 30 | 6 | 5 | 18 | 47 |
| Unintended | 2,005 | 197 | 23 | 9 | 4 | 24 | 137 |
| Other | 3,117 | 168 | 15 | 9 | 4 | 21 | 119 |
| | | | | | | | |
| **Zambia Urban** | **1,004,067** | **440,478** | **2,271** | **1,602** | **1,443** | **2,965** | **432,197** |
| Traditional | 92,832 | 17,032 | 9 | 7 | 16 | 42 | 16,958 |
| Improved Traditional | 175,763 | 60,000 | 95 | 96 | 80 | 162 | 59,567 |
| Mixed | 54,113 | 23,641 | 105 | 79 | 12 | 54 | 23,391 |
| Conventional Flat | 216,999 | 145,620 | 463 | 371 | 803 | 1,130 | 142,853 |
| Conventional House | 445,221 | 186,651 | 1,332 | 976 | 498 | 1,437 | 182,408 |
| Mobile | 500 | 162 | 3 | 3 | 2 | 4 | 150 |
| Part of commercial building | 4,930 | 1,951 | 6 | 11 | 13 | 40 | 1,881 |
| Improvised/Makeshift | 4,416 | 1,549 | 13 | 7 | 2 | 16 | 1,511 |
| Collective/Institutional quarters | 2,247 | 959 | 217 | 20 | - | 41 | 681 |
| Unintended | 2,748 | 1,222 | 11 | 25 | 4 | 31 | 1,151 |
| Other | 4,298 | 1,691 | 17 | 7 | 13 | 8 | 1,646 |

## Table H18: Number of Households with Access to a Toilet Facility by Type of Toilet Facility and Location of Toilet, Rural/Urban, Zambia 2010

| Type of Toilet Facility and Rural/ Urban | Households with Access to Toilet | Toilet Inside Housing Unit | | Toilet Outside Housing Unit | |
|---|---|---|---|---|---|
| | | Used Exclusively By Household Members | Shared with Other Non Household Members | Used Exclusively By Household Members | Shared with Other Non Household Members |
| **Zambia** | | | | | |
| **Total** | **1,994,519** | **190,838** | **14,951** | **1,206,251** | **582,479** |
| Rural | 1,021,916 | 12,266 | 848 | 806,525 | 202,277 |
| Urban | 972,603 | 178,572 | 14,103 | 399,726 | 380,202 |
| **Flush private connected to water sewer system** | | | | | |
| **Total** | **254,127** | **155,001** | **12,264** | **51,619** | **35,243** |
| Rural | 7,743 | 6,447 | 405 | 718 | 173 |
| Urban | 246,384 | 148,554 | 11,859 | 50,901 | 35,070 |
| **Flush private connected to stand-alone soak away** | | | | | |
| **Total** | **57,442** | **35,635** | **2,611** | **11,445** | **7,751** |
| Rural | 7,723 | 5,740 | 425 | 1,096 | 462 |
| Urban | 49,719 | 29,895 | 2,186 | 10,349 | 7,289 |
| **Flush communal** | | | | | |
| **Total** | **16,884** | **0** | **0** | **4,992** | **11,892** |
| Rural | 2,355 | 0 | 0 | 1,022 | 1,333 |
| Urban | 14,529 | 0 | 0 | 3,970 | 10,559 |
| **Pit Latrine** | | | | | |
| **Total** | **1,605,217** | **0** | **0** | **1,098,932** | **506,285** |
| Rural | 971,400 | 0 | 0 | 779,867 | 191,533 |
| Urban | 633,817 | 0 | 0 | 319,065 | 314,752 |
| **Ventilated Improved Pit (VIP)** | | | | | |
| **Total** | **45,907** | **0** | **0** | **30,004** | **15,903** |
| Rural | 20,284 | 0 | 0 | 15,830 | 4,454 |
| Urban | 25,623 | 0 | 0 | 14,174 | 11,449 |
| **Bucket** | | | | | |
| **Total** | **706** | **0** | **0** | **435** | **271** |
| Rural | 289 | 0 | 0 | 226 | 63 |
| Urban | 417 | 0 | 0 | 209 | 208 |
| **Other** | | | | | |
| **Total** | **14,236** | **202** | **76** | **8,824** | **5,134** |
| Rural | 12,122 | 79 | 18 | 7,766 | 4,259 |
| Urban | 2,114 | 123 | 58 | 1,058 | 875 |

## Table H19: Number of Households by Type of Housing Unit and Refuse Disposal, Rural/Urban, Zambia 2010

| Type of Refuse Disposal and Rural/Urban | Number of Households | Type of Housing Unit | | | | | | | | | | |
|---|---|---|---|---|---|---|---|---|---|---|---|---|
| | | Traditional | Improved Traditional | Mixed | Conventional Flat | Conventional House | Mobile | Part of commercial building | Improvised/ Makeshift | Collective/ Institutional quarters | Unintended | Other |
| **Zambia** | | | | | | | | | | | | |
| **Total** | **2,513,768** | **1,098,990** | **541,234** | **79,462** | **235,161** | **519,586** | **1,539** | **9,552** | **12,204** | **3,670** | **4,850** | **7,520** |
| Rural | 1,495,861 | 1,005,889 | 364,257 | 24,744 | 15,731 | 65,436 | 1,028 | 4,529 | 7,680 | 1,391 | 2,032 | 3,144 |
| Urban | 1,017,907 | 93,101 | 176,977 | 54,718 | 219,430 | 454,150 | 511 | 5,023 | 4,524 | 2,279 | 2,818 | 4,376 |
| **Regular collected** | | | | | | | | | | | | |
| **Total** | **182,980** | **16,668** | **10,890** | **6,376** | **56,154** | **88,687** | **78** | **1,097** | **516** | **841** | **389** | **1,284** |
| Rural | 23,893 | 15,031 | 5,494 | 423 | 553 | 1,722 | 12 | 52 | 175 | 47 | 20 | 364 |
| Urban | 159,087 | 1,637 | 5,396 | 5,953 | 55,601 | 86,965 | 66 | 1,045 | 341 | 794 | 369 | 920 |
| **Irregularly collected** | | | | | | | | | | | | |
| **Total** | **60,428** | **14,802** | **5,577** | **2,503** | **15,948** | **20,544** | **24** | **316** | **390** | **66** | **123** | **135** |
| Rural | 18,451 | 13,902 | 3,419 | 239 | 107 | 440 | 12 | 62 | 201 | 13 | 18 | 38 |
| Urban | 41,977 | 900 | 2,158 | 2,264 | 15,841 | 20,104 | 12 | 254 | 189 | 53 | 105 | 97 |
| **Burnt** | | | | | | | | | | | | |
| **Total** | **201,018** | **93,606** | **50,330** | **7,398** | **11,716** | **35,097** | **120** | **696** | **953** | **200** | **403** | **499** |
| Rural | 124,685 | 85,081 | 29,042 | 2,412 | 1,244 | 5,384 | 77 | 351 | 571 | 86 | 165 | 272 |
| Urban | 76,333 | 8,525 | 21,288 | 4,986 | 10,472 | 29,713 | 43 | 345 | 382 | 114 | 238 | 227 |
| **Roadside dumping** | | | | | | | | | | | | |
| **Total** | **162,025** | **64,460** | **29,790** | **6,501** | **25,146** | **33,514** | **122** | **694** | **992** | **116** | **363** | **327** |
| Rural | 80,401 | 59,352 | 15,926 | 1,331 | 547 | 2,246 | 82 | 276 | 376 | 35 | 125 | 105 |
| Urban | 81,624 | 5,108 | 13,864 | 5,170 | 24,599 | 31,268 | 40 | 418 | 616 | 81 | 238 | 222 |
| **Other dumping** | | | | | | | | | | | | |
| **Total** | **405,862** | **243,135** | **84,738** | **10,709** | **23,978** | **35,970** | **381** | **1,366** | **3,326** | **337** | **833** | **1,089** |
| Rural | 313,773 | 230,431 | 63,490 | 5,131 | 1,867 | 7,689 | 338 | 823 | 2,685 | 153 | 444 | 722 |
| Urban | 92,089 | 12,704 | 21,248 | 5,578 | 22,111 | 28,281 | 43 | 543 | 641 | 184 | 389 | 367 |
| **Burying/pit** | | | | | | | | | | | | |
| **Total** | **1,403,875** | **596,798** | **342,014** | **44,252** | **100,213** | **301,732** | **681** | **5,200** | **4,807** | **2,050** | **2,540** | **3,588** |
| Rural | 847,933 | 535,510 | 231,673 | 14,095 | 11,100 | 46,526 | 380 | 2,833 | 2,528 | 1,014 | 1,118 | 1,156 |
| Urban | 555,942 | 61,288 | 110,341 | 30,157 | 89,113 | 255,206 | 301 | 2,367 | 2,279 | 1,036 | 1,422 | 2,432 |
| **Other** | | | | | | | | | | | | |
| **Total** | **97,580** | **69,521** | **17,895** | **1,723** | **2,006** | **4,042** | **133** | **183** | **1,220** | **60** | **199** | **598** |
| Rural | 86,725 | 66,582 | 15,213 | 1,113 | 313 | 1,429 | 127 | 132 | 1,144 | 43 | 142 | 487 |
| Urban | 10,855 | 2,939 | 2,682 | 610 | 1,693 | 2,613 | 6 | 51 | 76 | 17 | 57 | 111 |

# Agriculture Tables

**Table I1: Total Number of Households and Number of Households engaged in Agricultural Activity 12 Months prior to the Census by Sex of Household Head, Province and Rural/Urban, Zambia 2010**

| Province | Number of Households | Agricultural Households | | | | | | | | |
|---|---|---|---|---|---|---|---|---|---|---|
| | | Total Households | | | Rural Households | | | Urban Households | | |
| | | All Households | Male Headed | Female Headed | All Households | Male Headed | Female Headed | All Households | Male Headed | Female Headed |
| **Zambia Total** | **2,513,768** | **1,540,390** | **1,197,945** | **342,445** | **1,305,053** | **1,012,964** | **292,089** | **235,337** | **184,981** | **50,356** |
| Central | 235,560 | 170,374 | 136,083 | 34,291 | 145,914 | 117,071 | 28,843 | 24,460 | 19,012 | 5,448 |
| Copperbelt | 371,125 | 146,369 | 117,541 | 28,828 | 66,111 | 53,358 | 12,753 | 80,258 | 64,183 | 16,075 |
| Eastern | 305,198 | 268,614 | 209,601 | 59,013 | 253,041 | 197,206 | 55,835 | 15,573 | 12,395 | 3,178 |
| Luapula | 194,962 | 155,966 | 124,148 | 31,818 | 132,920 | 106,282 | 26,638 | 23,046 | 17,866 | 5,180 |
| Lusaka | 444,418 | 65,213 | 51,994 | 13,219 | 39,629 | 31,687 | 7,942 | 25,584 | 20,307 | 5,277 |
| Muchinga | 138,783 | 119,443 | 94,677 | 24,766 | 107,127 | 84,771 | 22,356 | 12,316 | 9,906 | 2,410 |
| Northern | 220,561 | 176,571 | 142,815 | 33,756 | 159,554 | 129,422 | 30,132 | 17,017 | 13,393 | 3,624 |
| North Western | 130,803 | 104,106 | 80,314 | 23,792 | 89,336 | 69,040 | 20,296 | 14,770 | 11,274 | 3,496 |
| Southern | 292,179 | 186,173 | 141,065 | 45,108 | 172,374 | 130,293 | 42,081 | 13,799 | 10,772 | 3,027 |
| Western | 180,179 | 147,561 | 99,707 | 47,854 | 139,047 | 93,834 | 45,213 | 8,514 | 5,873 | 2,641 |

**Table I2: Total Number of Households and Number of Households engaged in raising of Poultry/Livestock 12 Months prior to the Census by Sex of Household Head, Province and Rural/Urban, Zambia 2010**

| Province | Number of House-holds | Poultry/Livestock Raising Households | | | | | | | | | | | | | | |
|---|---|---|---|---|---|---|---|---|---|---|---|---|---|---|---|---|
| | | Total Households | | | | | Rural Households | | | | | Urban Households | | | | |
| | | All House-holds | Male Headed | Percent Male Headed | Female Headed | Percent Female Headed | All House-holds | Male Headed | Percent Male Headed | Female Headed | Percent Female Headed | All House-holds | Male Headed | Percent Male Headed | Female Headed | Percent Female Headed |
| **Zambia Total** | **2,513,768** | **1,153,605** | **916,143** | **79.4** | **237,462** | **20.6** | **1045867** | **829616** | **79.3** | **216251** | **20.7** | **107738** | **86527** | **80.3** | **21211** | **19.7** |
| Central | 235,560 | 140,875 | 113,604 | 80.6 | 27,271 | 19.4 | 128667 | 103884 | 80.7 | 24783 | 19.3 | 12208 | 9720 | 79.6 | 2488 | 20.4 |
| Copperbelt | 371,125 | 80,742 | 65,852 | 81.6 | 14,890 | 18.4 | 49646 | 40450 | 81.5 | 9196 | 18.5 | 31096 | 25402 | 81.7 | 5694 | 18.3 |
| Eastern | 305,198 | 222,314 | 176,386 | 79.3 | 45,928 | 20.7 | 215652 | 171063 | 79.3 | 44589 | 20.7 | 6662 | 5323 | 79.9 | 1339 | 20.1 |
| Luapula | 194,962 | 105,094 | 85,397 | 81.3 | 19,697 | 18.7 | 91464 | 74645 | 81.6 | 16819 | 18.4 | 13630 | 10752 | 78.9 | 2878 | 21.1 |
| Lusaka | 444,418 | 40,211 | 32,258 | 80.2 | 7,953 | 19.8 | 29537 | 23499 | 79.6 | 6038 | 20.4 | 10674 | 8759 | 82.1 | 1915 | 17.9 |
| Muchinga | 138,783 | 95,017 | 76,369 | 80.4 | 18,648 | 19.6 | 88907 | 71374 | 80.3 | 17533 | 19.7 | 6110 | 4995 | 81.8 | 1115 | 18.2 |
| Northern | 220,561 | 134,743 | 111,228 | 82.5 | 23,515 | 17.5 | 126208 | 104388 | 82.7 | 21820 | 17.3 | 8535 | 6840 | 80.1 | 1695 | 19.9 |
| North Western | 130,803 | 70,128 | 56,424 | 80.5 | 13,704 | 19.5 | 63017 | 50795 | 80.6 | 12222 | 19.4 | 7111 | 5629 | 79.2 | 1482 | 20.8 |
| Southern | 292,179 | 165,325 | 126,947 | 76.8 | 38,378 | 23.2 | 157273 | 120549 | 76.6 | 36724 | 23.4 | 8052 | 6398 | 79.6 | 1654 | 20.4 |
| Western | 180,179 | 99,156 | 71,678 | 72.3 | 27,478 | 27.7 | 95496 | 68969 | 72.2 | 26527 | 27.8 | 3660 | 2709 | 74.0 | 951 | 26.0 |

## Table I3: Total Number of Households and Number of Households engaged in Fish Farming 12 Months prior to the Census by Sex of Household Head and, Province and Rural/Urban, Zambia 2010

| Province | Number of House-holds | Households Engaged in Fish Farming | | | | | | | | | | | | | | |
|---|---|---|---|---|---|---|---|---|---|---|---|---|---|---|---|---|
| | | Total Households | | | | | Rural Households | | | | | Urban Households | | | | |
| | | All House-holds | Male Headed | Percent Male Headed | Female Headed | Percent Female Headed | All House-holds | Male Headed | Percent Male Headed | Female Headed | Percent Female Headed | All House-holds | Male Headed | Percent Male Headed | Female Headed | Percent Female Headed |
| **Zambia Total** | **2,513,768** | **34,812** | **29,965** | **86.1** | **4,847** | **13.9** | **31,006** | **26,763** | **86.3** | **4,243** | **13.7** | **3,806** | **3,202** | **84.1** | **604** | **15.9** |
| Central | 235,560 | 3,402 | 2,936 | 86.3 | 466 | 13.7 | 3,063 | 2,652 | 86.6 | 411 | 13.4 | 339 | 284 | 83.8 | 55 | 16.2 |
| Copperbelt | 371,125 | 2,594 | 2,242 | 86.4 | 352 | 13.6 | 1,391 | 1,222 | 87.9 | 169 | 12.1 | 1,203 | 1,020 | 84.8 | 183 | 15.2 |
| Eastern | 305,198 | 3,270 | 2,731 | 83.5 | 539 | 16.5 | 3,128 | 2,614 | 83.6 | 514 | 16.4 | 142 | 117 | 82.4 | 25 | 17.6 |
| Luapula | 194,962 | 3,877 | 3,445 | 88.9 | 432 | 11.1 | 3,502 | 3,113 | 88.9 | 389 | 11.1 | 375 | 332 | 88.5 | 43 | 11.5 |
| Lusaka | 444,418 | 976 | 831 | 85.1 | 145 | 14.9 | 547 | 473 | 86.5 | 74 | 13.5 | 429 | 358 | 83.4 | 71 | 16.6 |
| Muchinga | 138,783 | 3,412 | 3,001 | 88 | 411 | 12 | 3,226 | 2,832 | 87.8 | 394 | 12.2 | 186 | 169 | 90.9 | 17 | 9.1 |
| Northern | 220,561 | 8,169 | 7,358 | 90.1 | 811 | 9.9 | 7,814 | 7,048 | 90.2 | 766 | 9.8 | 355 | 310 | 87.3 | 45 | 12.7 |
| North Western | 130,803 | 3,265 | 2,757 | 84.4 | 508 | 15.6 | 2,934 | 2,500 | 85.2 | 434 | 14.8 | 331 | 257 | 77.6 | 74 | 22.4 |
| Southern | 292,179 | 2,620 | 2,119 | 80.9 | 501 | 19.1 | 2,396 | 1,935 | 80.8 | 461 | 19.2 | 224 | 184 | 82.1 | 40 | 17.9 |
| Western | 180,179 | 3,227 | 2,545 | 78.9 | 682 | 21.1 | 3,005 | 2374 | 79.0 | 631 | 21.0 | 222 | 171 | 77.0 | 51 | 23.0 |

## Table I4: Total Number of Households and Number of Households engaged in Game Ranching 12 Months prior to the Census by Sex of Household Head, Province and Rural/Urban, Zambia 2010

| Province | Number of House-holds | Households Engaged in Game Ranching | | | | | | | | | | | | | | |
|---|---|---|---|---|---|---|---|---|---|---|---|---|---|---|---|---|
| | | Total Households | | | | | Rural Households | | | | | Urban Households | | | | |
| | | All House-holds | Male Headed | Percent Male Headed | Female Headed | Percent Female Headed | All House-holds | Male Headed | Percent Male Headed | Female Headed | Percent Female Headed | All House-holds | Male Headed | Percent Male Headed | Female Headed | Percent Female Headed |
| **Zambia Total** | **2,513,768** | **11,755** | **9,646** | **82.1** | **2,109** | **17.9** | **10,296** | **8,478** | **82.3** | **1,818** | **17.7** | **1459** | **1,168** | **80.1** | **291** | **19.9** |
| Central | 235,560 | 1,478 | 1,220 | 82.5 | 258 | 17.5 | 1,313 | 1,091 | 83.1 | 222 | 16.9 | 165 | 129 | 78.2 | 36 | 21.8 |
| Copperbelt | 371,125 | 1,039 | 876 | 84.3 | 163 | 15.7 | 593 | 510 | 86.0 | 83 | 14.0 | 446 | 366 | 82.1 | 80 | 17.9 |
| Eastern | 305,198 | 1,903 | 1,560 | 82.0 | 343 | 18.0 | 1,816 | 1,491 | 82.1 | 325 | 17.9 | 87 | 69 | 79.3 | 18 | 20.7 |
| Luapula | 194,962 | 1,164 | 968 | 83.2 | 196 | 16.8 | 1,000 | 831 | 83.1 | 169 | 16.9 | 164 | 137 | 83.5 | 27 | 16.5 |
| Lusaka | 444,418 | 418 | 338 | 80.9 | 80 | 19.1 | 223 | 184 | 82.5 | 39 | 17.5 | 195 | 154 | 79.0 | 41 | 21.0 |
| Muchinga | 138,783 | 1,281 | 1,050 | 82.0 | 231 | 18.0 | 1,218 | 995 | 81.7 | 223 | 18.3 | 63 | 55 | 87.3 | 8 | 12.7 |
| Northern | 220,561 | 1,670 | 1,434 | 85.9 | 236 | 14.1 | 1,563 | 1,345 | 86.1 | 218 | 13.9 | 107 | 89 | 83.2 | 18 | 16.8 |
| North Western | 130,803 | 865 | 701 | 81.0 | 164 | 19.0 | 747 | 620 | 83.0 | 127 | 17.0 | 118 | 81 | 68.6 | 37 | 31.4 |
| Southern | 292,179 | 1,240 | 985 | 79.4 | 255 | 20.6 | 1,160 | 917 | 79.1 | 243 | 20.9 | 80 | 68 | 85.0 | 12 | 15.0 |
| Western | 180,179 | 697 | 514 | 73.7 | 183 | 26.3 | 663 | 494 | 74.5 | 169 | 25.5 | 34 | 20 | 58.8 | 14 | 41.2 |

## Table I5: Total Number of Households and Number of Households Enganged in Raising of Poultry/Livestock 12 Months prior to the Census by Type of Poultry/Livestock and Province, Zambia 2010

| Province | Number of Households | Livestock/Poultry Raising Households | Cattle | Goats | Pigs | Sheep | Donkeys | Chickens | Other Poultry | Other Livestock |
|---|---|---|---|---|---|---|---|---|---|---|
| **Zambia Total** | **2,513,768** | **1,153,605** | **320,170** | **382,649** | **201,026** | **21,340** | **9,461** | **1,045,732** | **112,044** | **72,537** |
| Central | 235,560 | 140,875 | 45,790 | 56,288 | 11,979 | 1,453 | 1,247 | 133,295 | 16,707 | 12,537 |
| Copperbelt | 371,125 | 80,742 | 7,158 | 18,479 | 7,061 | 1,043 | 455 | 74,580 | 6,829 | 4,578 |
| Eastern | 305,198 | 222,314 | 87,590 | 68,953 | 89,467 | 6,676 | 1,138 | 191,516 | 19,521 | 10,405 |
| Luapula | 194,962 | 105,094 | 2,546 | 33,841 | 9,592 | 1,612 | 437 | 95,516 | 7,601 | 3,355 |
| Lusaka | 444,418 | 40,211 | 8,195 | 13,832 | 4,245 | 810 | 298 | 36,516 | 4,676 | 2,760 |
| Muchinga | 138,783 | 95,017 | 13,747 | 26,156 | 12,992 | 1,292 | 240 | 89,097 | 7,787 | 5,028 |
| Northern | 220,561 | 134,743 | 11,699 | 41,088 | 17,811 | 2,302 | 631 | 125,384 | 8,440 | 4,995 |
| North Western | 130,803 | 70,128 | 8,446 | 26,207 | 5,496 | 1,114 | 192 | 59,910 | 3,773 | 4,343 |
| Southern | 292,179 | 165,325 | 93,392 | 88,022 | 34,011 | 4,907 | 3,330 | 153,822 | 31,522 | 16,910 |
| Western | 180,179 | 99,156 | 41,607 | 9,783 | 8,372 | 131 | 1,493 | 86,096 | 5,188 | 7,626 |

## Table I6: Total Number of Households and Number of Households Engaged in Crop Growing 12 Months Prior to the Census by Type of Crop and Province, Zambia 2010

| Type of Crop | Province | | | | | | | | | | |
|---|---|---|---|---|---|---|---|---|---|---|---|
| | Zambia | Central | Copperbelt | Eastern | Luapula | Lusaka | Muchinga | Northern | North Western | Southern | Western |
| Maize | 1,340,038 | 162,620 | 133,239 | 264,714 | 100,606 | 57,231 | 99,158 | 121,567 | 91,950 | 173,639 | 135,314 |
| Sorghum | 140,639 | 14,461 | 6,575 | 5,949 | 4,608 | 2,153 | 25,908 | 12,907 | 11,089 | 24,549 | 32,440 |
| Millet | 250,524 | 21,880 | 5,409 | 6,895 | 15,758 | 914 | 64,231 | 77,415 | 8,010 | 14,418 | 35,594 |
| Rice | 114,448 | 1,940 | 949 | 14,153 | 8,300 | 660 | 21,716 | 22,181 | 4,424 | 253 | 39,872 |
| Cassava | 707,556 | 48,560 | 43,976 | 47,949 | 142,377 | 9,373 | 84,659 | 153,362 | 73,593 | 15,390 | 88,317 |
| Sweet Potatoes | 827,603 | 117,234 | 80,939 | 108,119 | 85,121 | 30,618 | 72,892 | 114,767 | 66,150 | 105,921 | 45,842 |
| Irish Potatoes | 81,396 | 7,879 | 6,800 | 18,007 | 2,443 | 2,531 | 6,721 | 12,941 | 16,084 | 6,375 | 1,615 |
| Groundnuts | 984,567 | 117,098 | 94,743 | 209,172 | 99,767 | 31,706 | 82,608 | 123,088 | 45,702 | 126,496 | 54,187 |
| Mixed Beans | 464,832 | 46,346 | 46,570 | 53,440 | 45,038 | 12,154 | 55,508 | 91,631 | 47,900 | 35,729 | 30,516 |
| Cow Peas | 124,002 | 17,588 | 4,211 | 37,679 | 5,485 | 3,855 | 11,568 | 12,463 | 3,718 | 21,619 | 5,816 |
| Wheat | 4,420 | 711 | 459 | 484 | 344 | 230 | 601 | 627 | 308 | 375 | 281 |
| Cotton | 145,598 | 28,038 | 563 | 88,003 | 137 | 1,616 | 9,300 | 270 | 93 | 17,387 | 191 |
| Burly Tobacco | 26,426 | 1,809 | 235 | 14,895 | 1,074 | 232 | 2,678 | 1,363 | 848 | 844 | 2,448 |
| Virginia Tobacco | 12,240 | 2,123 | 181 | 3,243 | 357 | 133 | 1,139 | 808 | 488 | 1,274 | 2,494 |
| Sunflower | 191,677 | 13,070 | 1,088 | 131,381 | 677 | 1,964 | 8,962 | 10,358 | 877 | 23,036 | 264 |
| Soya Beans | 110,319 | 20,047 | 7,668 | 36,680 | 5,107 | 2,352 | 12,553 | 15,936 | 3,967 | 4,912 | 1,097 |
| Paprika | 13,256 | 2,420 | 1,836 | 1,325 | 1,521 | 538 | 1,047 | 1,568 | 1,030 | 1,328 | 643 |
| Sugar Cane | 115,989 | 12,319 | 9,739 | 38,492 | 4,166 | 4,454 | 8,030 | 11,694 | 5,919 | 13,602 | 7,574 |
| Cashew Nuts | 18,112 | 2,708 | 1,698 | 2,830 | 1,951 | 254 | 2,030 | 2,458 | 1,039 | 2,060 | 1,084 |
| Vegetables | 508,846 | 74,750 | 65,964 | 74,290 | 33,872 | 28,176 | 33,151 | 45,951 | 30,329 | 91,271 | 31,092 |
| Orchards | 40,218 | 7,056 | 5,331 | 6,314 | 1,444 | 4,285 | 3,502 | 3,999 | 1,976 | 4,893 | 1,418 |
| Coffee | 2,008 | 513 | 237 | 230 | 115 | 160 | 206 | 172 | 86 | 230 | 59 |
| Velvet Beans | 21,868 | 2,194 | 1,326 | 5,500 | 1,836 | 728 | 1,812 | 4,090 | 1,426 | 2,454 | 502 |
| Bambara Nuts | 65,605 | 4,324 | 2,256 | 9,557 | 13,234 | 873 | 3,332 | 8,805 | 2,042 | 17,896 | 3,286 |
| Pineapple | 4,887 | 416 | 524 | 260 | 960 | 199 | 235 | 429 | 1,523 | 158 | 183 |
| Other Crops | 101,310 | 15,068 | 10,124 | 9,190 | 10,016 | 5,236 | 5,685 | 10,582 | 10,888 | 16,825 | 7,696 |

# General and Maternal Mortality Tables

## Table J1: Reported Number of Deaths 12 Months Prior to the Census by Province, District, Sex and Rural/Urban, Zambia 2010

| Province and District | Reported Household Deaths 12 Months Prior to the Census | | | | | | | | |
|---|---|---|---|---|---|---|---|---|---|
| | Total | | | Rural | | | Urban | | |
| | Total | Males | Females | Total | Males | Females | Total | Males | Females |
| **Zambia Total** | **164,385** | **87,693** | **76,692** | **101,720** | **54,166** | **47,554** | **62,665** | **33,527** | **29,138** |
| **Central Province** | **16,314** | **8,717** | **7,597** | **11,661** | **6,238** | **5,423** | **4,653** | **2,479** | **2,174** |
| Chibombo District | 3,394 | 1,795 | 1,599 | 3,193 | 1,683 | 1,510 | 201 | 112 | 89 |
| Kabwe District | 2,836 | 1,514 | 1,322 | - | - | - | 2,836 | 1,514 | 1,322 |
| Kapiri Mposhi District | 3,554 | 1,923 | 1,631 | 2,868 | 1,571 | 1,297 | 686 | 352 | 334 |
| Mkushi District | 1,628 | 851 | 777 | 1,382 | 718 | 664 | 246 | 133 | 113 |
| Mumbwa District | 2,734 | 1,443 | 1,291 | 2,284 | 1,203 | 1,081 | 450 | 240 | 210 |
| Serenje District | 2,168 | 1,191 | 977 | 1,934 | 1,063 | 871 | 234 | 128 | 106 |
| **Copperbelt Province** | **25,784** | **13,791** | **11,993** | **5,456** | **2,917** | **2,539** | **20,328** | **10,874** | **9,454** |
| Chililabombwe District | 1,012 | 530 | 482 | 238 | 127 | 111 | 774 | 403 | 371 |
| Chingola District | 2,730 | 1,458 | 1,272 | 526 | 284 | 242 | 2,204 | 1,174 | 1,030 |
| Kalulushi District | 1,299 | 670 | 629 | 321 | 167 | 154 | 978 | 503 | 475 |
| Kitwe District | 6,462 | 3,465 | 2,997 | 205 | 116 | 89 | 6,257 | 3,349 | 2,908 |
| Luanshya District | 2,034 | 1,079 | 955 | 431 | 227 | 204 | 1,603 | 852 | 751 |
| Lufwanyama District | 953 | 486 | 467 | 904 | 466 | 438 | 49 | 20 | 29 |
| Masaiti District | 1,567 | 859 | 708 | 1,529 | 838 | 691 | 38 | 21 | 17 |
| Mpongwe District | 1,353 | 725 | 628 | 1,142 | 603 | 539 | 211 | 122 | 89 |
| Mufulira District | 2,015 | 1,090 | 925 | 160 | 89 | 71 | 1,855 | 1,001 | 854 |
| Ndola District | 6,359 | 3,429 | 2,930 | - | - | - | 6,359 | 3,429 | 2,930 |
| **Eastern Province** | **23,746** | **12,441** | **11,305** | **21,314** | **11,185** | **10,129** | **2,432** | **1,256** | **1,176** |
| Chadiza District | 1,205 | 633 | 572 | 1,174 | 622 | 552 | 31 | 11 | 20 |
| Chipata District | 6,772 | 3,489 | 3,283 | 5,397 | 2,776 | 2,621 | 1,375 | 713 | 662 |
| Katete District | 3,255 | 1,740 | 1,515 | 3,071 | 1,632 | 1,439 | 184 | 108 | 76 |
| Lundazi District | 4,774 | 2,591 | 2,183 | 4,572 | 2,477 | 2,095 | 202 | 114 | 88 |
| Mambwe District | 985 | 533 | 452 | 908 | 489 | 419 | 77 | 44 | 33 |
| Nyimba District | 1,732 | 891 | 841 | 1,601 | 829 | 772 | 131 | 62 | 69 |
| Petauke District | 5,023 | 2,564 | 2,459 | 4,591 | 2,360 | 2,231 | 432 | 204 | 228 |
| **Luapula Province** | **16,216** | **8,659** | **7,557** | **13,186** | **7,088** | **6,098** | **3,030** | **1,571** | **1,459** |
| Chienge District | 2,648 | 1,380 | 1,268 | 2,571 | 1,351 | 1,220 | 77 | 29 | 48 |
| Kawambwa District | 1,919 | 994 | 925 | 1,424 | 746 | 678 | 495 | 248 | 247 |
| Mansa District | 3,369 | 1,829 | 1,540 | 2,126 | 1,174 | 952 | 1,243 | 655 | 588 |
| Milenge District | 485 | 273 | 212 | 464 | 264 | 200 | 21 | 9 | 12 |
| Mwense District | 1,687 | 873 | 814 | 1,439 | 746 | 693 | 248 | 127 | 121 |
| Nchelenge District | 3,110 | 1,646 | 1,464 | 2,557 | 1,361 | 1,196 | 553 | 285 | 268 |
| Samfya District | 2,998 | 1,664 | 1,334 | 2,605 | 1,446 | 1,159 | 393 | 218 | 175 |
| **Lusaka Province** | **24,813** | **13,415** | **11,398** | **3,760** | **1,994** | **1,766** | **21,053** | **11,421** | **9,632** |
| Chongwe District | 2,185 | 1,164 | 1,021 | 2,078 | 1,109 | 969 | 107 | 55 | 52 |
| Kafue District | 2,287 | 1,208 | 1,079 | 1,292 | 675 | 617 | 995 | 533 | 462 |
| Luangwa District | 444 | 240 | 204 | 390 | 210 | 180 | 54 | 30 | 24 |
| Lusaka District | 19,897 | 10,803 | 9,094 | - | - | - | 19,897 | 10,803 | 9,094 |

## Table J1: Reported Number of Household Deaths 12 Months Prior to the Census by Region, Sex, District Province, Zambia 2010

| Province and District | Reported Household Deaths 12 Months Prior to the Census | | | | | | | | |
|---|---|---|---|---|---|---|---|---|---|
| | Total | | | Rural | | | Urban | | |
| | Total | Males | Females | Total | Males | Females | Total | Males | Females |
| **Muchinga Province** | **8,389** | **4,621** | **3,768** | **6,976** | **3,804** | **3,172** | **1,413** | **817** | **596** |
| Chama District | 1,401 | 771 | 630 | 1,328 | 726 | 602 | 73 | 45 | 28 |
| Chinsali District | 1,429 | 808 | 621 | 1,282 | 725 | 557 | 147 | 83 | 64 |
| Isoka District | 748 | 430 | 318 | 634 | 364 | 270 | 114 | 66 | 48 |
| Mafinga District | 701 | 370 | 331 | 701 | 370 | 331 | - | - | - |
| Mpika District | 2,715 | 1,459 | 1,256 | 2,260 | 1,192 | 1,068 | 455 | 267 | 188 |
| Nakonde District | 1,395 | 783 | 612 | 771 | 427 | 344 | 624 | 356 | 268 |
| **Northern Province** | **13,062** | **7,079** | **5,983** | **10,829** | **5,870** | **4,959** | **2,233** | **1,209** | **1,024** |
| Chilubi District | 1,205 | 644 | 561 | 1,109 | 585 | 524 | 96 | 59 | 37 |
| Kaputa District | 1,922 | 1,020 | 902 | 1,789 | 963 | 826 | 133 | 57 | 76 |
| Kasama District | 2,337 | 1,285 | 1,052 | 1,377 | 754 | 623 | 960 | 531 | 429 |
| Luwingu District | 1,712 | 925 | 787 | 1,569 | 852 | 717 | 143 | 73 | 70 |
| Mbala District | 1,892 | 1,051 | 841 | 1,626 | 900 | 726 | 266 | 151 | 115 |
| Mporokoso District | 1,248 | 664 | 584 | 1,067 | 558 | 509 | 181 | 106 | 75 |
| Mpulungu District | 1,145 | 608 | 537 | 767 | 415 | 352 | 378 | 193 | 185 |
| Mungwi District | 1,601 | 882 | 719 | 1,525 | 843 | 682 | 76 | 39 | 37 |
| **North-Western Province** | **7,444** | **3,843** | **3,601** | **5,479** | **2,879** | **2,600** | **1,965** | **964** | **1,001** |
| Chavuma District | 292 | 160 | 132 | 244 | 134 | 110 | 48 | 26 | 22 |
| Ikelenge District | 236 | 122 | 114 | 236 | 122 | 114 | - | - | - |
| Kabompo District | 776 | 418 | 358 | 516 | 294 | 222 | 260 | 124 | 136 |
| Kasempa District | 574 | 309 | 265 | 560 | 300 | 260 | 14 | 9 | 5 |
| Mufumbwe District | 489 | 222 | 267 | 423 | 191 | 232 | 66 | 31 | 35 |
| Mwinilunga District | 940 | 495 | 445 | 767 | 398 | 369 | 173 | 97 | 76 |
| Solwezi District | 3,329 | 1,692 | 1,637 | 2,067 | 1,077 | 990 | 1,262 | 615 | 647 |
| Zambezi District | 808 | 425 | 383 | 666 | 363 | 303 | 142 | 62 | 80 |
| **Southern Province** | **15,222** | **8,150** | **7,072** | **11,459** | **6,153** | **5,306** | **3,763** | **1,997** | **1,766** |
| Choma District | 2,503 | 1,328 | 1,175 | 1,851 | 989 | 862 | 652 | 339 | 313 |
| Gwembe District | 552 | 302 | 250 | 530 | 292 | 238 | 22 | 10 | 12 |
| Itezhi Tezhi District | 733 | 408 | 325 | 609 | 338 | 271 | 124 | 70 | 54 |
| Kalomo District | 2,329 | 1,231 | 1,098 | 2,125 | 1,124 | 1,001 | 204 | 107 | 97 |
| Kazungula District | 989 | 540 | 449 | 981 | 535 | 446 | 8 | 5 | 3 |
| Livingstone District | 1,466 | 764 | 702 | 35 | 13 | 22 | 1,431 | 751 | 680 |
| Mazabuka District | 2,153 | 1,177 | 976 | 1,475 | 813 | 662 | 678 | 364 | 314 |
| Monze District | 1,615 | 829 | 786 | 1,312 | 662 | 650 | 303 | 167 | 136 |
| Namwala District | 1,098 | 593 | 505 | 1,046 | 572 | 474 | 52 | 21 | 31 |
| Siavonga District | 851 | 473 | 378 | 639 | 357 | 282 | 212 | 116 | 96 |
| Sinazongwe District | 933 | 505 | 428 | 856 | 458 | 398 | 77 | 47 | 30 |
| **Western Province** | **13,395** | **6,977** | **6,418** | **11,600** | **6,038** | **5,562** | **1,795** | **939** | **856** |
| Kalabo District | 1,907 | 1,000 | 907 | 1,860 | 967 | 893 | 47 | 33 | 14 |
| Kaoma District | 2,755 | 1,423 | 1,332 | 2,511 | 1,296 | 1,215 | 244 | 127 | 117 |
| Lukulu District | 954 | 491 | 463 | 814 | 413 | 401 | 140 | 78 | 62 |
| Mongu District | 3,373 | 1,759 | 1,614 | 2,502 | 1,314 | 1,188 | 871 | 445 | 426 |
| Senanga District | 2,150 | 1,105 | 1,045 | 1,908 | 981 | 927 | 242 | 124 | 118 |
| Sesheke District | 1,164 | 617 | 547 | 948 | 502 | 446 | 216 | 115 | 101 |
| Shang'ombo District | 1,092 | 582 | 510 | 1,057 | 565 | 492 | 35 | 17 | 18 |

## Table J2: Population (De facto) of Women 12-49 Years and Total Live Births 12 Months Prior to the Census by 5 Year Age Group, Maternal Mortality and Province, Rural/Urban, Zambia 2010

| Age Group, Province and Rural/Urban | Women Defacto Population | Number of Births 12 months Prior to the Census | Total Dead Women | Maternal Deaths | | | |
|---|---|---|---|---|---|---|---|
| | | | | Death Occuring while Pregnant | Death Occuring during Child Birth | Death Occuring 6 weeks after Pregnancy | Total Maternal Death |
| **Zambia Total** | | | | | | | |
| **Total** | **3,488,895** | **442,998** | **29,914** | **1,893** | **1,029** | **861** | **3,783** |
| 12-14 | 486,104 | 921 | 2,286 | 42 | 29 | 15 | 86 |
| 15 - 19 | 735,025 | 58,999 | 3,035 | 265 | 169 | 126 | 560 |
| 20 - 24 | 612,600 | 128,270 | 4,221 | 375 | 189 | 151 | 715 |
| 25 - 29 | 541,751 | 114,701 | 5,359 | 425 | 218 | 222 | 865 |
| 30 - 34 | 403,076 | 74,066 | 5,319 | 370 | 202 | 181 | 753 |
| 35 - 39 | 314,852 | 45,452 | 4,274 | 228 | 123 | 115 | 466 |
| 40 - 44 | 215,331 | 16,150 | 3,002 | 123 | 67 | 37 | 227 |
| 45 - 49 | 180,156 | 4,439 | 2,418 | 65 | 32 | 14 | 111 |
| **Zambia Rural** | | | | | | | |
| **Total** | **1,936,788** | **294,803** | **16,000** | **1,248** | **729** | **530** | **2,507** |
| 12-14 | 282,652 | 672 | 1,489 | 28 | 24 | 10 | 62 |
| 15 - 19 | 405,259 | 41,899 | 1,798 | 186 | 128 | 90 | 404 |
| 20 - 24 | 328,902 | 84,736 | 2,240 | 259 | 135 | 84 | 478 |
| 25 - 29 | 288,345 | 72,639 | 2,744 | 272 | 143 | 132 | 547 |
| 30 - 34 | 216,425 | 47,489 | 2,684 | 228 | 135 | 111 | 474 |
| 35 - 39 | 178,320 | 31,641 | 2,179 | 155 | 89 | 63 | 307 |
| 40 - 44 | 127,920 | 12,286 | 1,607 | 81 | 49 | 30 | 160 |
| 45 - 49 | 108,965 | 3,441 | 1,259 | 39 | 26 | 10 | 75 |
| **Zambia Urban** | | | | | | | |
| **Total** | **1,552,107** | **148,195** | **13,914** | **645** | **300** | **331** | **1,276** |
| 12-14 | 203,452 | 249 | 797 | 14 | 5 | 5 | 24 |
| 15 - 19 | 329,766 | 17,100 | 1,237 | 79 | 41 | 36 | 156 |
| 20 - 24 | 283,698 | 43,534 | 1,981 | 116 | 54 | 67 | 237 |
| 25 - 29 | 253,406 | 42,062 | 2,615 | 153 | 75 | 90 | 318 |
| 30 - 34 | 186,651 | 26,577 | 2,635 | 142 | 67 | 70 | 279 |
| 35 - 39 | 136,532 | 13,811 | 2,095 | 73 | 34 | 52 | 159 |
| 40 - 44 | 87,411 | 3,864 | 1,395 | 42 | 18 | 7 | 67 |
| 45 - 49 | 71,191 | 998 | 1,159 | 26 | 6 | 4 | 36 |

## Table J2: Population (De facto) of Women Aged 12-49 Years, Maternal Deaths 12 Months Prior to the Census and Maternal Mortality Ratios by 5 Year Age Group, Rural/Urban, Zambia 2010

| Age Group, Province and Rural/Urban | Women Defacto Population | Number of Births 12 months Prior to the Census | Total Dead Women | Maternal Deaths | | | |
|---|---|---|---|---|---|---|---|
| | | | | Death Occuring while Pregnant | Death Occuring during Child Birth | Death Occuring 6 weeks after Pregnancy | Total Maternal Death |
| **Central Province** | | | | | | | |
| **Total** | **336,301** | **44,660** | **3,132** | **190** | **132** | **87** | **409** |
| 12-14 | 50,404 | 101 | 224 | 2 | 2 | 1 | 5 |
| 15 - 19 | 73,509 | 6,330 | 319 | 23 | 26 | 14 | 63 |
| 20 - 24 | 57,899 | 12,981 | 447 | 45 | 21 | 13 | 79 |
| 25 - 29 | 49,022 | 10,994 | 513 | 36 | 23 | 28 | 87 |
| 30 - 34 | 37,606 | 7,269 | 568 | 34 | 25 | 20 | 79 |
| 35 - 39 | 29,774 | 4,778 | 449 | 21 | 20 | 10 | 51 |
| 40 - 44 | 20,611 | 1,741 | 342 | 18 | 10 | 1 | 29 |
| 45 - 49 | 17,476 | 466 | 270 | 11 | 5 | - | 16 |
| **Rural** | | | | | | | |
| **Total** | **238,990** | **35,474** | **2,081** | **138** | **101** | **61** | **300** |
| 12-14 | 36,855 | 76 | 162 | 1 | 1 | 1 | 3 |
| 15 - 19 | 50,910 | 5,133 | 222 | 20 | 20 | 9 | 49 |
| 20 - 24 | 40,843 | 10,358 | 285 | 32 | 13 | 11 | 56 |
| 25 - 29 | 34,326 | 8,490 | 347 | 30 | 16 | 19 | 65 |
| 30 - 34 | 26,631 | 5,669 | 364 | 21 | 21 | 13 | 55 |
| 35 - 39 | 21,558 | 3,905 | 296 | 12 | 18 | 8 | 38 |
| 40 - 44 | 15,076 | 1,467 | 228 | 14 | 8 | - | 22 |
| 45 - 49 | 12,791 | 376 | 177 | 8 | 4 | - | 12 |
| **Urban** | | | | | | | |
| **Total** | **97,311** | **9,186** | **1,051** | **52** | **31** | **26** | **109** |
| 12-14 | 13,549 | 25 | 62 | 1 | 1 | - | 2 |
| 15 - 19 | 22,599 | 1,197 | 97 | 3 | 6 | 5 | 14 |
| 20 - 24 | 17,056 | 2,623 | 162 | 13 | 8 | 2 | 23 |
| 25 - 29 | 14,696 | 2,504 | 166 | 6 | 7 | 9 | 22 |
| 30 - 34 | 10,975 | 1,600 | 204 | 13 | 4 | 7 | 24 |
| 35 - 39 | 8,216 | 873 | 153 | 9 | 2 | 2 | 13 |
| 40 - 44 | 5,535 | 274 | 114 | 4 | 2 | 1 | 7 |
| 45 - 49 | 4,685 | 90 | 93 | 3 | 1 | - | 4 |

## Table J2: Population (De facto) of Women Aged 12-49 Years, Maternal Deaths 12 Months Prior to the Census and Maternal Mortality Ratios by 5 Year Age Group, Rural/Urban, Zambia 2010

| Age Group, Province and Rural/Urban | Women Defacto Population | Number of Births 12 months Prior to the Census | Total Dead Women | Maternal Deaths | | | |
|---|---|---|---|---|---|---|---|
| | | | | Death Occuring while Pregnant | Death Occuring during Child Birth | Death Occuring 6 weeks after Pregnancy | Total Maternal Death |
| **Copperbelt Province** | | | | | | | |
| **Total** | **570,336** | **55,254** | **5,782** | **256** | **144** | **131** | **531** |
| 12-14 | 78,987 | 93 | 292 | 5 | 1 | - | 6 |
| 15 - 19 | 126,613 | 6,710 | 505 | 29 | 18 | 12 | 59 |
| 20 - 24 | 97,747 | 15,737 | 725 | 38 | 21 | 29 | 88 |
| 25 - 29 | 87,140 | 15,146 | 1,105 | 57 | 30 | 33 | 120 |
| 30 - 34 | 64,442 | 9,868 | 1,136 | 62 | 41 | 28 | 131 |
| 35 - 39 | 50,349 | 5,565 | 911 | 36 | 19 | 23 | 78 |
| 40 - 44 | 34,841 | 1,698 | 589 | 19 | 10 | 2 | 31 |
| 45 - 49 | 30,217 | 437 | 519 | 10 | 4 | 4 | 18 |
| **Rural** | | | | | | | |
| **Total** | **92,016** | **12,471** | **1,028** | **61** | **39** | **34** | **134** |
| 12-14 | 14,051 | 32 | 81 | 2 | 1 | - | 3 |
| 15 - 19 | 19,807 | 1,822 | 95 | 7 | 6 | 5 | 18 |
| 20 - 24 | 14,679 | 3,523 | 135 | 10 | 6 | 8 | 24 |
| 25 - 29 | 13,173 | 2,988 | 185 | 10 | 6 | 7 | 23 |
| 30 - 34 | 10,293 | 2,073 | 190 | 14 | 11 | 5 | 30 |
| 35 - 39 | 8,512 | 1,389 | 141 | 13 | 5 | 6 | 24 |
| 40 - 44 | 6,012 | 512 | 112 | 4 | 2 | 1 | 7 |
| 45 - 49 | 5,489 | 132 | 89 | 1 | 2 | 2 | 5 |
| **Urban** | | | | | | | |
| **Total** | **478,320** | **42,783** | **4,754** | **195** | **105** | **97** | **397** |
| 12-14 | 64,936 | 61 | 211 | 3 | - | - | 3 |
| 15 - 19 | 106,806 | 4,888 | 410 | 22 | 12 | 7 | 41 |
| 20 - 24 | 83,068 | 12,214 | 590 | 28 | 15 | 21 | 64 |
| 25 - 29 | 73,967 | 12,158 | 920 | 47 | 24 | 26 | 97 |
| 30 - 34 | 54,149 | 7,795 | 946 | 48 | 30 | 23 | 101 |
| 35 - 39 | 41,837 | 4,176 | 770 | 23 | 14 | 17 | 54 |
| 40 - 44 | 28,829 | 1,186 | 477 | 15 | 8 | 1 | 24 |
| 45 - 49 | 24,728 | 305 | 430 | 9 | 2 | 2 | 13 |

## Table J2: Population (De facto) of Women Aged 12-49 Years, Maternal Deaths 12 Months Prior to the Census and Maternal Mortality Ratios by 5 Year Age Group, Rural/Urban, Zambia 2010

| Age Group, Province and Rural/Urban | Women Defacto Population | Number of Births 12 months Prior to the Census | Total Dead Women | Maternal Deaths | | | |
|---|---|---|---|---|---|---|---|
| | | | | Death Occuring while Pregnant | Death Occuring during Child Birth | Death Occuring 6 weeks after Pregnancy | Total Maternal Death |
| **Eastern Province** | | | | | | | |
| **Total** | **400,556** | **57,880** | **3,155** | **254** | **122** | **93** | **469** |
| 12-14 | 57,904 | 138 | 300 | 5 | 5 | - | 10 |
| 15 - 19 | 83,226 | 8,852 | 356 | 41 | 26 | 17 | 84 |
| 20 - 24 | 69,000 | 17,274 | 438 | 48 | 23 | 17 | 88 |
| 25 - 29 | 60,690 | 14,151 | 477 | 47 | 25 | 21 | 93 |
| 30 - 34 | 44,858 | 8,885 | 527 | 49 | 19 | 20 | 88 |
| 35 - 39 | 37,164 | 5,759 | 471 | 35 | 15 | 10 | 60 |
| 40 - 44 | 25,745 | 2,169 | 321 | 23 | 4 | 8 | 35 |
| 45 - 49 | 21,969 | 652 | 265 | 6 | 5 | - | 11 |
| **Rural** | | | | | | | |
| **Total** | **342,257** | **52,344** | **2,650** | **229** | **117** | **76** | **422** |
| 12-14 | 50,003 | 124 | 272 | 5 | 5 | - | 10 |
| 15 - 19 | 71,083 | 8,106 | 312 | 38 | 26 | 14 | 78 |
| 20 - 24 | 58,136 | 15,570 | 352 | 42 | 22 | 11 | 75 |
| 25 - 29 | 50,886 | 12,612 | 390 | 40 | 23 | 19 | 82 |
| 30 - 34 | 37,849 | 7,953 | 434 | 44 | 18 | 15 | 77 |
| 35 - 39 | 32,227 | 5,308 | 394 | 31 | 14 | 9 | 54 |
| 40 - 44 | 22,669 | 2,043 | 276 | 23 | 4 | 8 | 35 |
| 45 - 49 | 19,404 | 628 | 220 | 6 | 5 | - | 11 |
| **Urban** | | | | | | | |
| **Total** | **58,299** | **5,536** | **505** | **25** | **5** | **17** | **47** |
| 12-14 | 7,901 | 14 | 28 | - | - | - | - |
| 15 - 19 | 12,143 | 746 | 44 | 3 | - | 3 | 6 |
| 20 - 24 | 10,864 | 1,704 | 86 | 6 | 1 | 6 | 13 |
| 25 - 29 | 9,804 | 1,539 | 87 | 7 | 2 | 2 | 11 |
| 30 - 34 | 7,009 | 932 | 93 | 5 | 1 | 5 | 11 |
| 35 - 39 | 4,937 | 451 | 77 | 4 | 1 | 1 | 6 |
| 40 - 44 | 3,076 | 126 | 45 | - | - | - | - |
| 45 - 49 | 2,565 | 24 | 45 | - | - | - | - |

## Table J2: Population (De facto) of Women Aged 12-49 Years, Maternal Deaths 12 Months Prior to the Census and Maternal Mortality Ratios by 5 Year Age Group, Rural/Urban, Zambia 2010

| Age Group, Province and Rural/Urban | Women Defacto Population | Number of Births 12 months Prior to the Census | Total Dead Women | Maternal Deaths | | | |
|---|---|---|---|---|---|---|---|
| | | | | Death Occuring while Pregnant | Death Occuring during Child Birth | Death Occuring 6 weeks after Pregnancy | Total Maternal Death |
| **Luapula Province** | | | | | | | |
| **Total** | **249,079** | **36,932** | **2,606** | **227** | **115** | **75** | **417** |
| 12-14 | 34,813 | 74 | 264 | 11 | 7 | 3 | 21 |
| 15 - 19 | 51,760 | 4,255 | 294 | 34 | 25 | 11 | 70 |
| 20 - 24 | 42,004 | 10,243 | 442 | 51 | 27 | 14 | 92 |
| 25 - 29 | 38,315 | 9,740 | 479 | 55 | 19 | 16 | 90 |
| 30 - 34 | 28,441 | 6,552 | 414 | 29 | 14 | 15 | 58 |
| 35 - 39 | 23,095 | 3,996 | 334 | 23 | 12 | 10 | 45 |
| 40 - 44 | 16,735 | 1,624 | 211 | 17 | 7 | 4 | 28 |
| 45 - 49 | 13,916 | 448 | 168 | 7 | 4 | 2 | 13 |
| **Rural** | | | | | | | |
| **Total** | **196,955** | **31,240** | **2,007** | **187** | **94** | **67** | **348** |
| 12-14 | 27,418 | 59 | 208 | 6 | 6 | 1 | 13 |
| 15 - 19 | 40,263 | 3,580 | 230 | 26 | 17 | 10 | 53 |
| 20 - 24 | 33,012 | 8,679 | 331 | 38 | 20 | 11 | 69 |
| 25 - 29 | 30,363 | 8,156 | 358 | 50 | 17 | 14 | 81 |
| 30 - 34 | 22,487 | 5,535 | 320 | 23 | 12 | 15 | 50 |
| 35 - 39 | 18,563 | 3,412 | 269 | 22 | 12 | 10 | 44 |
| 40 - 44 | 13,676 | 1,431 | 164 | 16 | 6 | 4 | 26 |
| 45 - 49 | 11,173 | 388 | 127 | 6 | 4 | 2 | 12 |
| **Urban** | | | | | | | |
| **Total** | **52,124** | **5,692** | **599** | **40** | **21** | **8** | **69** |
| 12-14 | 7,395 | 15 | 56 | 5 | 1 | 2 | 8 |
| 15 - 19 | 11,497 | 675 | 64 | 8 | 8 | 1 | 17 |
| 20 - 24 | 8,992 | 1,564 | 111 | 13 | 7 | 3 | 23 |
| 25 - 29 | 7,952 | 1,584 | 121 | 5 | 2 | 2 | 9 |
| 30 - 34 | 5,954 | 1,017 | 94 | 6 | 2 | - | 8 |
| 35 - 39 | 4,532 | 584 | 65 | 1 | - | - | 1 |
| 40 - 44 | 3,059 | 193 | 47 | 1 | 1 | - | 2 |
| 45 - 49 | 2,743 | 60 | 41 | 1 | - | - | 1 |

## Table J2: Population (De facto) of Women Aged 12-49 Years, Maternal Deaths 12 Months Prior to the Census and Maternal Mortality Ratios by 5 Year Age Group, Rural/Urban, Zambia 2010

| Age Group, Province and Rural/Urban | Women Defacto Population | Number of Births 12 months Prior to the Census | Total Dead Women | Maternal Deaths | | | |
|---|---|---|---|---|---|---|---|
| | | | | Death Occuring while Pregnant | Death Occuring during Child Birth | Death Occuring 6 weeks after Pregnancy | Total Maternal Death |
| **Lusaka Province** | | | | | | | |
| **Total** | **662,333** | **65,922** | **5,297** | **227** | **86** | **145** | **458** |
| 12-14 | 83,894 | 87 | 317 | 1 | 1 | 5 | 7 |
| 15 - 19 | 131,397 | 7,022 | 485 | 33 | 10 | 15 | 58 |
| 20 - 24 | 124,116 | 19,520 | 756 | 37 | 16 | 25 | 78 |
| 25 - 29 | 112,492 | 18,756 | 954 | 61 | 17 | 40 | 118 |
| 30 - 34 | 85,030 | 12,146 | 1,009 | 49 | 22 | 32 | 103 |
| 35 - 39 | 60,613 | 6,272 | 779 | 18 | 14 | 23 | 55 |
| 40 - 44 | 36,550 | 1,711 | 567 | 20 | 5 | 5 | 30 |
| 45 - 49 | 28,241 | 408 | 430 | 8 | 1 | - | 9 |
| **Rural** | | | | | | | |
| **Total** | **85,373** | **10,997** | **725** | **28** | **14** | **18** | **60** |
| 12-14 | 12,643 | 18 | 45 | - | - | 2 | 2 |
| 15 - 19 | 17,987 | 1,514 | 86 | 8 | 2 | 2 | 12 |
| 20 - 24 | 14,723 | 3,312 | 91 | 5 | 4 | 2 | 11 |
| 25 - 29 | 12,823 | 2,704 | 113 | 5 | 2 | 4 | 11 |
| 30 - 34 | 9,943 | 1,812 | 145 | 6 | 3 | 4 | 13 |
| 35 - 39 | 7,716 | 1,151 | 92 | 1 | 2 | 2 | 5 |
| 40 - 44 | 5,154 | 398 | 86 | 3 | 1 | 2 | 6 |
| 45 - 49 | 4,384 | 88 | 67 | - | - | - | - |
| **Urban** | | | | | | | |
| **Total** | **576,960** | **54,925** | **4,572** | **199** | **72** | **127** | **398** |
| 12-14 | 71,251 | 69 | 272 | 1 | 1 | 3 | 5 |
| 15 - 19 | 113,410 | 5,508 | 399 | 25 | 8 | 13 | 46 |
| 20 - 24 | 109,393 | 16,208 | 665 | 32 | 12 | 23 | 67 |
| 25 - 29 | 99,669 | 16,052 | 841 | 56 | 15 | 36 | 107 |
| 30 - 34 | 75,087 | 10,334 | 864 | 43 | 19 | 28 | 90 |
| 35 - 39 | 52,897 | 5,121 | 687 | 17 | 12 | 21 | 50 |
| 40 - 44 | 31,396 | 1,313 | 481 | 17 | 4 | 3 | 24 |
| 45 - 49 | 23,857 | 320 | 363 | 8 | 1 | - | 9 |

## Table J2: Population (De facto) of Women Aged 12-49 Years, Maternal Deaths 12 Months Prior to the Census and Maternal Mortality Ratios by 5 Year Age Group, Rural/Urban, Zambia 2010

| Age Group, Province and Rural/Urban | Women Defacto Population | Number of Births 12 months Prior to the Census | Total Dead Women | Maternal Deaths | | | |
|---|---|---|---|---|---|---|---|
| | | | | Death Occuring while Pregnant | Death Occuring during Child Birth | Death Occuring 6 weeks after Pregnancy | Total Maternal Death |
| **Muchinga Province** | | | | | | | |
| **Total** | **177,918** | **26,781** | **1,167** | **74** | **57** | **30** | **161** |
| 12-14 | 25,620 | 52 | 149 | 1 | 1 | 1 | 3 |
| 15 - 19 | 37,480 | 3,596 | 151 | 11 | 11 | 6 | 28 |
| 20 - 24 | 31,336 | 7,867 | 163 | 17 | 14 | 6 | 37 |
| 25 - 29 | 27,392 | 6,892 | 196 | 15 | 12 | 10 | 37 |
| 30 - 34 | 19,141 | 4,167 | 183 | 13 | 4 | 3 | 20 |
| 35 - 39 | 16,054 | 2,803 | 138 | 11 | 7 | 3 | 21 |
| 40 - 44 | 11,244 | 1,094 | 107 | 5 | 6 | 1 | 12 |
| 45 - 49 | 9,651 | 310 | 80 | 1 | 2 | - | 3 |
| **Rural** | | | | | | | |
| **Total** | **144,194** | **22,710** | **892** | **63** | **49** | **26** | **138** |
| 12-14 | 21,067 | 42 | 121 | 1 | 1 | 1 | 3 |
| 15 - 19 | 30,270 | 3,100 | 131 | 10 | 9 | 6 | 25 |
| 20 - 24 | 24,885 | 6,581 | 113 | 17 | 12 | 5 | 34 |
| 25 - 29 | 21,816 | 5,763 | 147 | 10 | 8 | 9 | 27 |
| 30 - 34 | 15,283 | 3,507 | 135 | 12 | 4 | 2 | 18 |
| 35 - 39 | 13,149 | 2,448 | 102 | 8 | 7 | 3 | 18 |
| 40 - 44 | 9,500 | 983 | 80 | 4 | 6 | - | 10 |
| 45 - 49 | 8,224 | 286 | 63 | 1 | 2 | - | 3 |
| **Urban** | | | | | | | |
| **Total** | **33,724** | **4,071** | **275** | **11** | **8** | **4** | **23** |
| 12-14 | 4,553 | 10 | 28 | - | - | - | - |
| 15 - 19 | 7,210 | 496 | 20 | 1 | 2 | - | 3 |
| 20 - 24 | 6,451 | 1,286 | 50 | - | 2 | 1 | 3 |
| 25 - 29 | 5,576 | 1,129 | 49 | 5 | 4 | 1 | 10 |
| 30 - 34 | 3,858 | 660 | 48 | 1 | - | 1 | 2 |
| 35 - 39 | 2,905 | 355 | 36 | 3 | - | - | 3 |
| 40 - 44 | 1,744 | 111 | 27 | 1 | - | 1 | 2 |
| 45 - 49 | 1,427 | 24 | 17 | - | - | - | - |

## Table J2: Population (De facto) of Women Aged 12-49 Years, Maternal Deaths 12 Months Prior to the Census and Maternal Mortality Ratios by 5 Year Age Group, Rural/Urban, Zambia 2010

| Age Group, Province and Rural/Urban | Women Defacto Population | Number of Births 12 months Prior to the Census | Total Dead Women | Maternal Deaths | | | |
|---|---|---|---|---|---|---|---|
| | | | | Death Occuring while Pregnant | Death Occuring during Child Birth | Death Occuring 6 weeks after Pregnancy | Total Maternal Death |
| **Northern Province** | | | | | | | |
| **Total** | **272,524** | **42,826** | **1,905** | **175** | **118** | **73** | **366** |
| 12-14 | 38,349 | 81 | 241 | 8 | 4 | 1 | 13 |
| 15 - 19 | 57,448 | 5,636 | 261 | 24 | 21 | 20 | 65 |
| 20 - 24 | 47,718 | 12,744 | 263 | 39 | 20 | 11 | 70 |
| 25 - 29 | 42,294 | 11,174 | 345 | 38 | 25 | 14 | 77 |
| 30 - 34 | 29,201 | 6,497 | 278 | 26 | 22 | 12 | 60 |
| 35 - 39 | 24,898 | 4,479 | 240 | 24 | 14 | 9 | 47 |
| 40 - 44 | 17,327 | 1,677 | 144 | 7 | 9 | 4 | 20 |
| 45 - 49 | 15,289 | 538 | 133 | 9 | 3 | 2 | 14 |
| **Rural** | | | | | | | |
| **Total** | **216,836** | **36,850** | **1,473** | **138** | **102** | **62** | **302** |
| 12-14 | 30,461 | 67 | 195 | 7 | 4 | 1 | 12 |
| 15 - 19 | 45,011 | 4,789 | 214 | 17 | 20 | 18 | 55 |
| 20 - 24 | 37,743 | 10,953 | 208 | 33 | 18 | 10 | 61 |
| 25 - 29 | 33,491 | 9,537 | 255 | 30 | 17 | 9 | 56 |
| 30 - 34 | 23,035 | 5,551 | 207 | 20 | 19 | 12 | 51 |
| 35 - 39 | 20,137 | 3,944 | 184 | 18 | 13 | 6 | 37 |
| 40 - 44 | 14,337 | 1,529 | 101 | 6 | 8 | 4 | 18 |
| 45 - 49 | 12,621 | 480 | 109 | 7 | 3 | 2 | 12 |
| **Urban** | | | | | | | |
| **Total** | **55,688** | **5,976** | **432** | **37** | **16** | **11** | **64** |
| 12-14 | 7,888 | 14 | 46 | 1 | - | - | 1 |
| 15 - 19 | 12,437 | 847 | 47 | 7 | 1 | 2 | 10 |
| 20 - 24 | 9,975 | 1,791 | 55 | 6 | 2 | 1 | 9 |
| 25 - 29 | 8,803 | 1,637 | 90 | 8 | 8 | 5 | 21 |
| 30 - 34 | 6,166 | 946 | 71 | 6 | 3 | - | 9 |
| 35 - 39 | 4,761 | 535 | 56 | 6 | 1 | 3 | 10 |
| 40 - 44 | 2,990 | 148 | 43 | 1 | 1 | - | 2 |
| 45 - 49 | 2,668 | 58 | 24 | 2 | - | - | 2 |

## Table J2: Population (De facto) of Women Aged 12-49 Years, Maternal Deaths 12 Months Prior to the Census and Maternal Mortality Ratios by 5 Year Age Group, Rural/Urban, Zambia 2010

| Age Group, Province and Rural/Urban | Women Defacto Population | Number of Births 12 months Prior to the Census | Total Dead Women | Maternal Deaths | | | |
|---|---|---|---|---|---|---|---|
| | | | | Death Occuring while Pregnant | Death Occuring during Child Birth | Death Occuring 6 weeks after Pregnancy | Total Maternal Death |
| **North-Western Province** | | | | | | | |
| **Total** | **179,051** | **25,948** | **1,399** | **108** | **52** | **44** | **204** |
| 12-14 | 26,187 | 70 | 140 | 2 | 3 | 2 | 7 |
| 15 - 19 | 38,708 | 3,656 | 142 | 18 | 5 | 4 | 27 |
| 20 - 24 | 30,186 | 7,009 | 184 | 19 | 11 | 5 | 35 |
| 25 - 29 | 27,316 | 6,784 | 262 | 31 | 13 | 16 | 60 |
| 30 - 34 | 19,887 | 4,304 | 219 | 22 | 13 | 8 | 43 |
| 35 - 39 | 15,873 | 2,804 | 196 | 13 | 4 | 5 | 22 |
| 40 - 44 | 11,528 | 1,042 | 132 | 2 | 1 | 3 | 6 |
| 45 - 49 | 9,366 | 279 | 124 | 1 | 2 | 1 | 4 |
| **Rural** | | | | | | | |
| **Total** | **133,417** | **20,726** | **945** | **88** | **38** | **36** | **162** |
| 12-14 | 19,815 | 55 | 109 | 1 | 1 | 2 | 4 |
| 15 - 19 | 28,459 | 2,875 | 94 | 13 | 4 | 3 | 20 |
| 20 - 24 | 21,643 | 5,439 | 122 | 16 | 9 | 2 | 27 |
| 25 - 29 | 19,891 | 5,382 | 165 | 27 | 9 | 13 | 49 |
| 30 - 34 | 14,821 | 3,471 | 149 | 18 | 10 | 8 | 36 |
| 35 - 39 | 12,305 | 2,352 | 128 | 10 | 4 | 5 | 19 |
| 40 - 44 | 9,039 | 905 | 92 | 2 | - | 2 | 4 |
| 45 - 49 | 7,444 | 247 | 86 | 1 | 1 | 1 | 3 |
| **Urban** | | | | | | | |
| **Total** | **45,634** | **5,222** | **454** | **20** | **14** | **8** | **42** |
| 12-14 | 6,372 | 15 | 31 | 1 | 2 | - | 3 |
| 15 - 19 | 10,249 | 781 | 48 | 5 | 1 | 1 | 7 |
| 20 - 24 | 8,543 | 1,570 | 62 | 3 | 2 | 3 | 8 |
| 25 - 29 | 7,425 | 1,402 | 97 | 4 | 4 | 3 | 11 |
| 30 - 34 | 5,066 | 833 | 70 | 4 | 3 | - | 7 |
| 35 - 39 | 3,568 | 452 | 68 | 3 | - | - | 3 |
| 40 - 44 | 2,489 | 137 | 40 | - | 1 | 1 | 2 |
| 45 - 49 | 1,922 | 32 | 38 | - | 1 | - | 1 |

## Table J2: Population (De facto) of Women Aged 12-49 Years, Maternal Deaths 12 Months Prior to the Census and Maternal Mortality Ratios by 5 Year Age Group, Rural/Urban, Zambia 2010

| Age Group, Province and Rural/Urban | Women Defacto Population | Number of Births 12 months Prior to the Census | Total Dead Women | Maternal Deaths | | | |
|---|---|---|---|---|---|---|---|
| | | | | Death Occuring while Pregnant | Death Occuring during Child Birth | Death Occuring 6 weeks after Pregnancy | Total Maternal Death |
| **Southern Province** | | | | | | | |
| **Total** | **413,325** | **56,626** | **2,649** | **170** | **93** | **80** | **343** |
| 12-14 | 59,424 | 117 | 203 | 4 | 1 | 1 | 6 |
| 15 - 19 | 88,705 | 8,502 | 254 | 23 | 11 | 11 | 45 |
| 20 - 24 | 72,608 | 16,565 | 382 | 33 | 22 | 14 | 69 |
| 25 - 29 | 62,186 | 13,703 | 483 | 46 | 26 | 18 | 90 |
| 30 - 34 | 47,991 | 9,357 | 450 | 29 | 19 | 22 | 70 |
| 35 - 39 | 36,314 | 5,768 | 388 | 25 | 7 | 6 | 38 |
| 40 - 44 | 25,485 | 2,116 | 272 | 5 | 4 | 4 | 13 |
| 45 - 49 | 20,612 | 498 | 217 | 5 | 3 | 4 | 12 |
| **Rural** | | | | | | | |
| **Total** | **294,736** | **45,147** | **1,847** | **132** | **75** | **58** | **265** |
| 12-14 | 44,429 | 101 | 157 | 3 | 1 | 1 | 5 |
| 15 - 19 | 63,278 | 7,045 | 188 | 21 | 10 | 9 | 40 |
| 20 - 24 | 49,921 | 13,033 | 250 | 24 | 18 | 9 | 51 |
| 25 - 29 | 42,347 | 10,449 | 331 | 35 | 21 | 15 | 71 |
| 30 - 34 | 33,678 | 7,443 | 299 | 22 | 15 | 17 | 54 |
| 35 - 39 | 26,322 | 4,800 | 276 | 20 | 4 | 1 | 25 |
| 40 - 44 | 19,137 | 1,831 | 204 | 3 | 4 | 4 | 11 |
| 45 - 49 | 15,624 | 445 | 142 | 4 | 2 | 2 | 8 |
| **Urban** | | | | | | | |
| **Total** | **118,589** | **11,479** | **802** | **38** | **18** | **22** | **78** |
| 12-14 | 14,995 | 16 | 46 | 1 | - | - | 1 |
| 15 - 19 | 25,427 | 1,457 | 66 | 2 | 1 | 2 | 5 |
| 20 - 24 | 22,687 | 3,532 | 132 | 9 | 4 | 5 | 18 |
| 25 - 29 | 19,839 | 3,254 | 152 | 11 | 5 | 3 | 19 |
| 30 - 34 | 14,313 | 1,914 | 151 | 7 | 4 | 5 | 16 |
| 35 - 39 | 9,992 | 968 | 112 | 5 | 3 | 5 | 13 |
| 40 - 44 | 6,348 | 285 | 68 | 2 | - | - | 2 |
| 45 - 49 | 4,988 | 53 | 75 | 1 | 1 | 2 | 4 |

## Table J2: Population (De facto) of Women Aged 12-49 Years, Maternal Deaths 12 Months Prior to the Census and Maternal Mortality Ratios by 5 Year Age Group, Rural/Urban, Zambia 2010

| Age Group, Province and Rural/Urban | Women Defacto Population | Number of Births 12 months Prior to the Census | Total Dead Women | Maternal Deaths | | | |
|---|---|---|---|---|---|---|---|
| | | | | Death Occuring while Pregnant | Death Occuring during Child Birth | Death Occuring 6 weeks after Pregnancy | Total Maternal Death |
| **Western Province** | | | | | | | |
| **Total** | **227,472** | **30,169** | **2,822** | **212** | **110** | **103** | **425** |
| 12-14 | 30,522 | 108 | 156 | 3 | 4 | 1 | 8 |
| 15 - 19 | 46,179 | 4,440 | 268 | 29 | 16 | 16 | 61 |
| 20 - 24 | 39,986 | 8,330 | 421 | 48 | 14 | 17 | 79 |
| 25 - 29 | 34,904 | 7,361 | 545 | 39 | 28 | 26 | 93 |
| 30 - 34 | 26,479 | 5,021 | 535 | 57 | 23 | 21 | 101 |
| 35 - 39 | 20,718 | 3,228 | 368 | 22 | 11 | 16 | 49 |
| 40 - 44 | 15,265 | 1,278 | 317 | 7 | 11 | 5 | 23 |
| 45 - 49 | 13,419 | 403 | 212 | 7 | 3 | 1 | 11 |
| **Rural** | | | | | | | |
| **Total** | **192,014** | **26,844** | **2,352** | **184** | **100** | **92** | **376** |
| 12-14 | 25,910 | 98 | 139 | 2 | 4 | 1 | 7 |
| 15 - 19 | 38,191 | 3,935 | 226 | 26 | 14 | 14 | 54 |
| 20 - 24 | 33,317 | 7,288 | 353 | 42 | 13 | 15 | 70 |
| 25 - 29 | 29,229 | 6,558 | 453 | 35 | 24 | 23 | 82 |
| 30 - 34 | 22,405 | 4,475 | 441 | 48 | 22 | 20 | 90 |
| 35 - 39 | 17,831 | 2,932 | 297 | 20 | 10 | 13 | 43 |
| 40 - 44 | 13,320 | 1,187 | 264 | 6 | 10 | 5 | 21 |
| 45 - 49 | 11,811 | 371 | 179 | 5 | 3 | 1 | 9 |
| **Urban** | | | | | | | |
| **Total** | **35,458** | **3,325** | **470** | **28** | **10** | **11** | **49** |
| 12-14 | 4,612 | 10 | 17 | 1 | - | - | 1 |
| 15 - 19 | 7,988 | 505 | 42 | 3 | 2 | 2 | 7 |
| 20 - 24 | 6,669 | 1,042 | 68 | 6 | 1 | 2 | 9 |
| 25 - 29 | 5,675 | 803 | 92 | 4 | 4 | 3 | 11 |
| 30 - 34 | 4,074 | 546 | 94 | 9 | 1 | 1 | 11 |
| 35 - 39 | 2,887 | 296 | 71 | 2 | 1 | 3 | 6 |
| 40 - 44 | 1,945 | 91 | 53 | 1 | 1 | - | 2 |
| 45 - 49 | 1,608 | 32 | 33 | 2 | - | - | 2 |

## Table J3: Population (De Facto), Deaths 12 Months Prior to the Census and Age Specific Death Rates by Age (Single and Grouped) and Sex, Rural/Urban, Zambia 2010

| Age and Rural/ Urban | Population | | | Reported Household Deaths 12 Months Prior to the Census | | | Unadjusted Age Specific Mortality Rates Per 1000 Population | | |
|---|---|---|---|---|---|---|---|---|---|
| | Total | Male | Female | Total | Male | Female | Total | Male | Female |
| **Zambia Total** | **12,526,314** | **6,117,253** | **6,409,061** | **164,385** | **87,693** | **76,692** | **13.1** | **14.3** | **12.0** |
| 0 | 436,304 | 217,890 | 218,414 | 35,103 | 18,953 | 16,150 | 80.5 | 87.0 | 73.9 |
| 1 | 437,960 | 218,257 | 219,703 | 12,005 | 6,280 | 5,725 | 27.4 | 28.8 | 26.1 |
| 2 | 452,387 | 225,299 | 227,088 | 8,667 | 4,685 | 3,982 | 19.2 | 20.8 | 17.5 |
| 3 | 442,255 | 218,642 | 223,613 | 5,066 | 2,644 | 2,422 | 11.5 | 12.1 | 10.8 |
| 4 | 445,981 | 222,184 | 223,797 | 3,205 | 1,688 | 1,517 | 7.2 | 7.6 | 6.8 |
| 0 - 4 | 2,214,887 | 1,102,272 | 1,112,615 | 64,046 | 34,250 | 29,796 | 28.9 | 31.1 | 26.8 |
| 5-9 | 1,856,336 | 924,395 | 931,941 | 8,405 | 4,519 | 3,886 | 4.5 | 4.9 | 4.2 |
| 10-14 | 1,699,042 | 840,834 | 858,208 | 4,836 | 2,550 | 2,286 | 2.8 | 3.0 | 2.7 |
| 15 - 19 | 1,427,884 | 692,859 | 735,025 | 5,856 | 2,821 | 3,035 | 4.1 | 4.1 | 4.1 |
| 20 - 24 | 1,117,476 | 504,876 | 612,600 | 7,967 | 3,746 | 4,221 | 7.1 | 7.4 | 6.9 |
| 25 - 29 | 1,006,244 | 464,493 | 541,751 | 10,471 | 5,112 | 5,359 | 10.4 | 11.0 | 9.9 |
| 30 - 34 | 800,770 | 397,694 | 403,076 | 11,305 | 5,986 | 5,319 | 14.1 | 15.1 | 13.2 |
| 35 - 39 | 647,813 | 332,961 | 314,852 | 9,972 | 5,698 | 4,274 | 15.4 | 17.1 | 13.6 |
| 40 - 44 | 447,584 | 232,253 | 215,331 | 7,336 | 4,334 | 3,002 | 16.4 | 18.7 | 13.9 |
| 45 - 49 | 354,989 | 174,833 | 180,156 | 6,021 | 3,603 | 2,418 | 17.0 | 20.6 | 13.4 |
| 50 - 54 | 270,254 | 128,696 | 141,558 | 4,770 | 2,687 | 2,083 | 17.7 | 20.9 | 14.7 |
| 55 - 59 | 184,828 | 90,037 | 94,791 | 3,546 | 2,056 | 1,490 | 19.2 | 22.8 | 15.7 |
| 60 - 64 | 161,875 | 73,419 | 88,456 | 3,929 | 2,146 | 1,783 | 24.3 | 29.2 | 20.2 |
| 65 - 69 | 118,582 | 53,695 | 64,887 | 3,363 | 1,732 | 1,631 | 28.4 | 32.3 | 25.1 |
| 70 - 74 | 90,802 | 41,929 | 48,873 | 4,084 | 2,138 | 1,946 | 45.0 | 51.0 | 39.8 |
| 75+ | 126,948 | 62,007 | 64,941 | 8,478 | 4,315 | 4,163 | 66.8 | 69.6 | 64.1 |
| | | | | | | | | | |
| **Zambia Rural** | **7,505,292** | **3,664,349** | **3,840,943** | **101,720** | **54,166** | **47,554** | **13.6** | **14.8** | **12.4** |
| 0 | 288,243 | 143,874 | 144,369 | 24,609 | 13,448 | 11,161 | 85.4 | 93.5 | 77.3 |
| 1 | 280,428 | 139,921 | 140,507 | 8,188 | 4,305 | 3,883 | 29.2 | 30.8 | 27.6 |
| 2 | 300,708 | 149,275 | 151,433 | 6,446 | 3,474 | 2,972 | 21.4 | 23.3 | 19.6 |
| 3 | 290,561 | 144,190 | 146,371 | 3,855 | 1,995 | 1,860 | 13.3 | 13.8 | 12.7 |
| 4 | 294,445 | 146,898 | 147,547 | 2,404 | 1,268 | 1,136 | 8.2 | 8.6 | 7.7 |
| 0 - 4 | 1,454,385 | 724,158 | 730,227 | 45,502 | 24,490 | 21,012 | 31.3 | 33.8 | 28.8 |
| 5-9 | 1,212,247 | 608,241 | 604,006 | 6,162 | 3,303 | 2,859 | 5.1 | 5.4 | 4.7 |
| 10-14 | 1,045,977 | 529,978 | 515,999 | 3,203 | 1,714 | 1,489 | 3.1 | 3.2 | 2.9 |
| 15 - 19 | 802,997 | 397,738 | 405,259 | 3,517 | 1,719 | 1,798 | 4.4 | 4.3 | 4.4 |
| 20 - 24 | 598,270 | 269,368 | 328,902 | 4,255 | 2,015 | 2,240 | 7.1 | 7.5 | 6.8 |
| 25 - 29 | 529,481 | 241,136 | 288,345 | 5,211 | 2,467 | 2,744 | 9.8 | 10.2 | 9.5 |
| 30 - 34 | 419,932 | 203,507 | 216,425 | 5,469 | 2,785 | 2,684 | 13.0 | 13.7 | 12.4 |
| 35 - 39 | 351,970 | 173,650 | 178,320 | 4,888 | 2,709 | 2,179 | 13.9 | 15.6 | 12.2 |
| 40 - 44 | 254,964 | 127,044 | 127,920 | 3,785 | 2,178 | 1,607 | 14.8 | 17.1 | 12.6 |
| 45 - 49 | 209,868 | 100,903 | 108,965 | 3,065 | 1,806 | 1,259 | 14.6 | 17.9 | 11.6 |
| 50 - 54 | 162,395 | 75,868 | 86,527 | 2,548 | 1,445 | 1,103 | 15.7 | 19.0 | 12.7 |
| 55 - 59 | 111,511 | 52,225 | 59,286 | 1,874 | 1,071 | 803 | 16.8 | 20.5 | 13.5 |
| 60 - 64 | 107,038 | 45,761 | 61,277 | 2,218 | 1,190 | 1,028 | 20.7 | 26.0 | 16.8 |
| 65 - 69 | 83,157 | 36,611 | 46,546 | 1,959 | 1,010 | 949 | 23.6 | 27.6 | 20.4 |
| 70 - 74 | 66,388 | 30,553 | 35,835 | 2,638 | 1,407 | 1,231 | 39.7 | 46.1 | 34.4 |
| 75+ | 94,712 | 47,608 | 47,104 | 5,426 | 2,857 | 2,569 | 57.3 | 60.0 | 54.5 |

## Table J3: Population (De Facto), Deaths 12 Months Prior to the Census and Age Specific Death Rates by Age (Single and Grouped) and Sex, Rural/Urban, Zambia 2010

| Age and Rural/ Urban | Population | | | Reported Household Deaths 12 Months Prior to the Census | | | Unadjusted Age Specific Mortality Rates Per 1000 Population | | |
|---|---|---|---|---|---|---|---|---|---|
| | Total | Male | Female | Total | Male | Female | Total | Male | Female |
| **Zambia Urban** | **5,021,022** | **2,452,904** | **2,568,118** | **62,665** | **33,527** | **29,138** | **12.5** | **13.7** | **11.3** |
| 0 | 148,061 | 74,016 | 74,045 | 10,494 | 5,505 | 4,989 | 70.9 | 74.4 | 67.4 |
| 1 | 157,532 | 78,336 | 79,196 | 3,817 | 1,975 | 1,842 | 24.2 | 25.2 | 23.3 |
| 2 | 151,679 | 76,024 | 75,655 | 2,221 | 1,211 | 1,010 | 14.6 | 15.9 | 13.4 |
| 3 | 151,694 | 74,452 | 77,242 | 1,211 | 649 | 562 | 8.0 | 8.7 | 7.3 |
| 4 | 151,536 | 75,286 | 76,250 | 801 | 420 | 381 | 5.3 | 5.6 | 5.0 |
| 0 - 4 | 760,502 | 378,114 | 382,388 | 18,544 | 9,760 | 8,784 | 24.4 | 25.8 | 23.0 |
| 5-9 | 644,089 | 316,154 | 327,935 | 2,243 | 1,216 | 1,027 | 3.5 | 3.8 | 3.1 |
| 10-14 | 653,065 | 310,856 | 342,209 | 1,633 | 836 | 797 | 2.5 | 2.7 | 2.3 |
| 15 - 19 | 624,887 | 295,121 | 329,766 | 2,339 | 1,102 | 1,237 | 3.7 | 3.7 | 3.8 |
| 20 - 24 | 519,206 | 235,508 | 283,698 | 3,712 | 1,731 | 1,981 | 7.1 | 7.4 | 7.0 |
| 25 - 29 | 476,763 | 223,357 | 253,406 | 5,260 | 2,645 | 2,615 | 11.0 | 11.8 | 10.3 |
| 30 - 34 | 380,838 | 194,187 | 186,651 | 5,836 | 3,201 | 2,635 | 15.3 | 16.5 | 14.1 |
| 35 - 39 | 295,843 | 159,311 | 136,532 | 5,084 | 2,989 | 2,095 | 17.2 | 18.8 | 15.3 |
| 40 - 44 | 192,620 | 105,209 | 87,411 | 3,551 | 2,156 | 1,395 | 18.4 | 20.5 | 16.0 |
| 45 - 49 | 145,121 | 73,930 | 71,191 | 2,956 | 1,797 | 1,159 | 20.4 | 24.3 | 16.3 |
| 50 - 54 | 107,859 | 52,828 | 55,031 | 2,222 | 1,242 | 980 | 20.6 | 23.5 | 17.8 |
| 55 - 59 | 73,317 | 37,812 | 35,505 | 1,672 | 985 | 687 | 22.8 | 26.0 | 19.3 |
| 60 - 64 | 54,837 | 27,658 | 27,179 | 1,711 | 956 | 755 | 31.2 | 34.6 | 27.8 |
| 65 - 69 | 35,425 | 17,084 | 18,341 | 1,404 | 722 | 682 | 39.6 | 42.3 | 37.2 |
| 70 - 74 | 24,414 | 11,376 | 13,038 | 1,446 | 731 | 715 | 59.2 | 64.3 | 54.8 |
| 75+ | 32,236 | 14,399 | 17,837 | 3,052 | 1,458 | 1,594 | 94.7 | 101.3 | 89.4 |

## Table J4: Population (De Facto) and Number of Household Deaths 12 months prior to the Census by Cause of Death, 5 Year Age Group, Sex and Province, Zambia 2010

| Age Group, Sex and Province | Population | Household Deaths 12 Months Prior to the Census | Cause of Death | | | | | | | | |
|---|---|---|---|---|---|---|---|---|---|---|---|
| | | | Total | Accident | Injury | Suicide | Spousal Violence | Other Violence | Sickness/ Disease | Witchcraft | Other |
| **Zambia Total** | **12,526,314** | **164,385** | **164,385** | **5,568** | **818** | **1,015** | **301** | **1,526** | **124,521** | **10,775** | **19,861** |
| 0 - 4 | 2,214,887 | 64,046 | 64,046 | 1,183 | 223 | - | - | 382 | 45,415 | 4,079 | 12,764 |
| 5-9 | 1,856,336 | 8,405 | 8,405 | 440 | 42 | - | - | 51 | 6,404 | 822 | 646 |
| 10-14 | 1,699,042 | 4,836 | 4,836 | 362 | 49 | 25 | 6 | 63 | 3,414 | 513 | 404 |
| 15 - 19 | 1,427,884 | 5,856 | 5,856 | 450 | 59 | 127 | 23 | 79 | 4,045 | 546 | 527 |
| 20 - 24 | 1,117,476 | 7,967 | 7,967 | 569 | 46 | 198 | 47 | 134 | 5,774 | 585 | 614 |
| 25 - 29 | 1,006,244 | 10,471 | 10,471 | 590 | 70 | 187 | 52 | 168 | 8,006 | 641 | 757 |
| 30 - 34 | 800,770 | 11,305 | 11,305 | 478 | 54 | 131 | 49 | 150 | 9,000 | 643 | 800 |
| 35 - 39 | 647,813 | 9,972 | 9,972 | 433 | 63 | 107 | 33 | 129 | 8,001 | 600 | 606 |
| 40 - 44 | 447,584 | 7,336 | 7,336 | 241 | 34 | 74 | 26 | 88 | 5,977 | 461 | 435 |
| 45 - 49 | 354,989 | 6,021 | 6,021 | 209 | 35 | 55 | 22 | 54 | 4,867 | 400 | 379 |
| 50 - 54 | 270,254 | 4,770 | 4,770 | 150 | 23 | 23 | 12 | 55 | 3,879 | 323 | 305 |
| 55 - 59 | 184,828 | 3,546 | 3,546 | 100 | 13 | 20 | 4 | 32 | 2,930 | 222 | 225 |
| 60 - 64 | 161,875 | 3,929 | 3,929 | 84 | 25 | 11 | 7 | 36 | 3,274 | 252 | 240 |
| 65 - 69 | 118,582 | 3,363 | 3,363 | 69 | 16 | 13 | 6 | 26 | 2,862 | 205 | 166 |
| 70 - 74 | 90,802 | 4,084 | 4,084 | 83 | 14 | 19 | 1 | 31 | 3,459 | 210 | 267 |
| 75 - 79 | 61,420 | 3,163 | 3,163 | 57 | 15 | 8 | 4 | 17 | 2,719 | 122 | 221 |
| 80 - 84 | 32,821 | 2,755 | 2,755 | 46 | 24 | 10 | 5 | 16 | 2,349 | 75 | 230 |
| 85 - 89 | 18,655 | 2,091 | 2,091 | 18 | 11 | 5 | 3 | 14 | 1,754 | 66 | 220 |
| 90 - 94 | 6,308 | 469 | 469 | 6 | 2 | 2 | 1 | 1 | 392 | 10 | 55 |
| 95+ | 7,744 | - | - | - | - | - | - | - | - | - | - |
| | | | | | | | | | | | |
| **Male** | **6,117,253** | **87,693** | **87,693** | **3,870** | **523** | **775** | **117** | **1,085** | **64,642** | **5,969** | **10,712** |
| 0 - 4 | 1,102,272 | 34,250 | 34,250 | 665 | 125 | - | - | 213 | 24,058 | 2,156 | 7,033 |
| 5-9 | 924,395 | 4,519 | 4,519 | 287 | 23 | - | - | 32 | 3,375 | 440 | 362 |
| 10-14 | 840,834 | 2,550 | 2,550 | 233 | 30 | 15 | 5 | 39 | 1,740 | 264 | 224 |
| 15 - 19 | 692,859 | 2,821 | 2,821 | 298 | 32 | 76 | 6 | 52 | 1,854 | 286 | 217 |
| 20 - 24 | 504,876 | 3,746 | 3,746 | 422 | 34 | 141 | 16 | 105 | 2,466 | 300 | 262 |
| 25 - 29 | 464,493 | 5,112 | 5,112 | 449 | 51 | 150 | 11 | 133 | 3,638 | 336 | 344 |
| 30 - 34 | 397,694 | 5,986 | 5,986 | 377 | 36 | 110 | 21 | 124 | 4,581 | 360 | 377 |
| 35 - 39 | 332,961 | 5,698 | 5,698 | 354 | 46 | 86 | 12 | 106 | 4,389 | 360 | 345 |
| 40 - 44 | 232,253 | 4,334 | 4,334 | 194 | 23 | 64 | 9 | 70 | 3,460 | 262 | 252 |
| 45 - 49 | 174,833 | 3,603 | 3,603 | 154 | 30 | 44 | 14 | 46 | 2,808 | 278 | 229 |
| 50 - 54 | 128,696 | 2,687 | 2,687 | 105 | 16 | 19 | 6 | 45 | 2,087 | 214 | 195 |
| 55 - 59 | 90,037 | 2,056 | 2,056 | 78 | 7 | 17 | 2 | 24 | 1,664 | 132 | 132 |
| 60 - 64 | 73,419 | 2,146 | 2,146 | 64 | 16 | 10 | 6 | 27 | 1,733 | 148 | 142 |
| 65 - 69 | 53,695 | 1,732 | 1,732 | 53 | 11 | 10 | 3 | 19 | 1,438 | 111 | 87 |
| 70 - 74 | 41,929 | 2,138 | 2,138 | 54 | 10 | 17 | 1 | 20 | 1,747 | 136 | 153 |
| 75 - 79 | 30,662 | 1,652 | 1,652 | 36 | 13 | 6 | 1 | 9 | 1,400 | 85 | 102 |
| 80 - 84 | 16,045 | 1,352 | 1,352 | 30 | 13 | 7 | 2 | 12 | 1,124 | 49 | 115 |
| 85 - 89 | 9,347 | 1,064 | 1,064 | 12 | 6 | 2 | 2 | 8 | 877 | 47 | 110 |
| 90 - 94 | 3,152 | 247 | 247 | 5 | 1 | 1 | - | 1 | 203 | 5 | 31 |
| 95+ | 2,801 | - | - | - | - | - | - | - | - | - | - |

## Table J4: Population (De Facto) and Number of Household Deaths 12 months prior to the Census by Cause of Death, 5 Year Age Group, Sex and Province, Zambia 2010

| Age Group, Sex and Province | Population | Household Deaths 12 Months Prior to the Census | Cause of Death | | | | | | | | |
|---|---|---|---|---|---|---|---|---|---|---|---|
| | | | Total | Accident | Injury | Suicide | Spousal Violence | Other Violence | Sickness/ Disease | Witchcraft | Other |
| **Female** | **6,409,061** | **76,692** | **76,692** | **1,698** | **295** | **240** | **184** | **441** | **59,879** | **4,806** | **9,149** |
| 0 - 4 | 1,112,615 | 29,796 | 29,796 | 518 | 98 | - | - | 169 | 21,357 | 1,923 | 5,731 |
| 5-9 | 931,941 | 3,886 | 3,886 | 153 | 19 | - | - | 19 | 3,029 | 382 | 284 |
| 10-14 | 858,208 | 2,286 | 2,286 | 129 | 19 | 10 | 1 | 24 | 1,674 | 249 | 180 |
| 15 - 19 | 735,025 | 3,035 | 3,035 | 152 | 27 | 51 | 17 | 27 | 2,191 | 260 | 310 |
| 20 - 24 | 612,600 | 4,221 | 4,221 | 147 | 12 | 57 | 31 | 29 | 3,308 | 285 | 352 |
| 25 - 29 | 541,751 | 5,359 | 5,359 | 141 | 19 | 37 | 41 | 35 | 4,368 | 305 | 413 |
| 30 - 34 | 403,076 | 5,319 | 5,319 | 101 | 18 | 21 | 28 | 26 | 4,419 | 283 | 423 |
| 35 - 39 | 314,852 | 4,274 | 4,274 | 79 | 17 | 21 | 21 | 23 | 3,612 | 240 | 261 |
| 40 - 44 | 215,331 | 3,002 | 3,002 | 47 | 11 | 10 | 17 | 18 | 2,517 | 199 | 183 |
| 45 - 49 | 180,156 | 2,418 | 2,418 | 55 | 5 | 11 | 8 | 8 | 2,059 | 122 | 150 |
| 50 - 54 | 141,558 | 2,083 | 2,083 | 45 | 7 | 4 | 6 | 10 | 1,792 | 109 | 110 |
| 55 - 59 | 94,791 | 1,490 | 1,490 | 22 | 6 | 3 | 2 | 8 | 1,266 | 90 | 93 |
| 60 - 64 | 88,456 | 1,783 | 1,783 | 20 | 9 | 1 | 1 | 9 | 1,541 | 104 | 98 |
| 65 - 69 | 64,887 | 1,631 | 1,631 | 16 | 5 | 3 | 3 | 7 | 1,424 | 94 | 79 |
| 70 - 74 | 48,873 | 1,946 | 1,946 | 29 | 4 | 2 | - | 11 | 1,712 | 74 | 114 |
| 75 - 79 | 30,758 | 1,511 | 1,511 | 21 | 2 | 2 | 3 | 8 | 1,319 | 37 | 119 |
| 80 - 84 | 16,776 | 1,403 | 1,403 | 16 | 11 | 3 | 3 | 4 | 1,225 | 26 | 115 |
| 85 - 89 | 9,308 | 1,027 | 1,027 | 6 | 5 | 3 | 1 | 6 | 877 | 19 | 110 |
| 90 - 94 | 3,156 | 222 | 222 | 1 | 1 | 1 | 1 | - | 189 | 5 | 24 |
| 95+ | 4,943 | - | - | - | - | - | - | - | - | - | - |
| **Central Province** | | | | | | | | | | | |
| **Total** | **1,245,089** | **16,314** | **16,314** | **667** | **84** | **148** | **33** | **149** | **12,276** | **950** | **2,007** |
| 0 - 4 | 224,289 | 5,996 | 5,996 | 128 | 15 | - | - | 33 | 4,213 | 333 | 1,274 |
| 5-9 | 190,254 | 773 | 773 | 50 | 7 | - | - | 6 | 578 | 66 | 66 |
| 10-14 | 175,827 | 500 | 500 | 49 | 9 | 4 | 1 | 6 | 347 | 38 | 46 |
| 15 - 19 | 144,976 | 602 | 602 | 67 | 6 | 18 | 2 | 7 | 409 | 43 | 50 |
| 20 - 24 | 108,067 | 811 | 811 | 67 | 3 | 24 | 5 | 6 | 582 | 51 | 73 |
| 25 - 29 | 91,398 | 1,004 | 1,004 | 69 | 7 | 20 | 9 | 16 | 746 | 52 | 85 |
| 30 - 34 | 74,143 | 1,179 | 1,179 | 60 | 8 | 18 | 6 | 12 | 936 | 60 | 79 |
| 35 - 39 | 61,248 | 1,056 | 1,056 | 52 | 6 | 23 | 6 | 20 | 833 | 64 | 52 |
| 40 - 44 | 43,287 | 770 | 770 | 26 | 2 | 12 | 2 | 12 | 637 | 42 | 37 |
| 45 - 49 | 34,498 | 676 | 676 | 29 | 7 | 4 | 1 | 8 | 538 | 44 | 45 |
| 50 - 54 | 27,078 | 482 | 482 | 15 | 1 | 3 | - | 8 | 387 | 42 | 26 |
| 55 - 59 | 18,546 | 394 | 394 | 9 | 2 | 5 | 1 | 3 | 331 | 22 | 21 |
| 60 - 64 | 16,675 | 385 | 385 | 7 | 3 | - | - | 3 | 324 | 22 | 26 |
| 65 - 69 | 12,234 | 358 | 358 | 10 | 2 | 3 | - | 2 | 298 | 22 | 21 |
| 70 - 74 | 9,334 | 433 | 433 | 11 | 2 | 6 | - | 1 | 361 | 24 | 28 |
| 75 - 79 | 6,651 | 321 | 321 | 9 | - | 3 | - | 3 | 269 | 8 | 29 |
| 80 - 84 | 3,274 | 288 | 288 | 7 | 3 | 2 | - | 3 | 241 | 6 | 26 |
| 85 - 89 | 1,977 | 229 | 229 | 1 | 1 | 3 | - | - | 197 | 6 | 21 |
| 90 - 94 | 607 | 57 | 57 | 1 | - | - | - | - | 49 | 5 | 2 |
| 95+ | 726 | - | - | - | - | - | - | - | - | - | - |

## Table J4: Population (De Facto) and Number of Household Deaths 12 months prior to the Census by Cause of Death, 5 Year Age Group, Sex and Province, Zambia 2010

| Age Group, Sex and Province | Population | Household Deaths 12 Months Prior to the Census | Cause of Death | | | | | | | | |
|---|---|---|---|---|---|---|---|---|---|---|---|
| | | | Total | Accident | Injury | Suicide | Spousal Violence | Other Violence | Sickness/ Disease | Witchcraft | Other |
| **Male** | **613,372** | **8,717** | **8,717** | **437** | **52** | **119** | **13** | **105** | **6,363** | **541** | **1,087** |
| 0 - 4 | 111,530 | 3,254 | 3,254 | 67 | 7 | - | - | 20 | 2,256 | 183 | 721 |
| 5-9 | 95,203 | 427 | 427 | 33 | 4 | - | - | 5 | 312 | 37 | 36 |
| 10-14 | 87,537 | 276 | 276 | 32 | 6 | 3 | 1 | 3 | 186 | 19 | 26 |
| 15 - 19 | 71,467 | 283 | 283 | 40 | 3 | 12 | 1 | 3 | 179 | 22 | 23 |
| 20 - 24 | 50,168 | 364 | 364 | 42 | 1 | 17 | 2 | 4 | 242 | 23 | 33 |
| 25 - 29 | 42,376 | 491 | 491 | 50 | 4 | 18 | 3 | 14 | 335 | 30 | 37 |
| 30 - 34 | 36,537 | 611 | 611 | 44 | 7 | 17 | 3 | 11 | 459 | 35 | 35 |
| 35 - 39 | 31,474 | 607 | 607 | 41 | 6 | 19 | 1 | 15 | 459 | 38 | 28 |
| 40 - 44 | 22,676 | 428 | 428 | 20 | 1 | 10 | - | 7 | 351 | 25 | 14 |
| 45 - 49 | 17,022 | 406 | 406 | 19 | 7 | 4 | 1 | 8 | 311 | 31 | 25 |
| 50 - 54 | 12,960 | 258 | 258 | 9 | 1 | 2 | - | 6 | 207 | 24 | 9 |
| 55 - 59 | 9,219 | 235 | 235 | 6 | - | 4 | 1 | 2 | 196 | 12 | 14 |
| 60 - 64 | 7,891 | 206 | 206 | 6 | 2 | - | - | 1 | 163 | 15 | 19 |
| 65 - 69 | 5,815 | 201 | 201 | 9 | 2 | 3 | - | 2 | 159 | 16 | 10 |
| 70 - 74 | 4,630 | 198 | 198 | 7 | 1 | 5 | - | 1 | 156 | 14 | 14 |
| 75 - 79 | 3,415 | 179 | 179 | 6 | - | 2 | - | 1 | 150 | 7 | 13 |
| 80 - 84 | 1,757 | 142 | 142 | 5 | - | 1 | - | 2 | 115 | 4 | 15 |
| 85 - 89 | 1,088 | 118 | 118 | - | - | 2 | - | - | 99 | 4 | 13 |
| 90 - 94 | 349 | 33 | 33 | 1 | - | - | - | - | 28 | 2 | 2 |
| 95+ | 258 | - | - | - | - | - | - | - | - | - | - |
| | | | | | | | | | | | |
| **Female** | **631,717** | **7,597** | **7,597** | **230** | **32** | **29** | **20** | **44** | **5,913** | **409** | **920** |
| 0 - 4 | 112,759 | 2,742 | 2,742 | 61 | 8 | - | - | 13 | 1,957 | 150 | 553 |
| 5-9 | 95,051 | 346 | 346 | 17 | 3 | - | - | 1 | 266 | 29 | 30 |
| 10-14 | 88,290 | 224 | 224 | 17 | 3 | 1 | - | 3 | 161 | 19 | 20 |
| 15 - 19 | 73,509 | 319 | 319 | 27 | 3 | 6 | 1 | 4 | 230 | 21 | 27 |
| 20 - 24 | 57,899 | 447 | 447 | 25 | 2 | 7 | 3 | 2 | 340 | 28 | 40 |
| 25 - 29 | 49,022 | 513 | 513 | 19 | 3 | 2 | 6 | 2 | 411 | 22 | 48 |
| 30 - 34 | 37,606 | 568 | 568 | 16 | 1 | 1 | 3 | 1 | 477 | 25 | 44 |
| 35 - 39 | 29,774 | 449 | 449 | 11 | - | 4 | 5 | 5 | 374 | 26 | 24 |
| 40 - 44 | 20,611 | 342 | 342 | 6 | 1 | 2 | 2 | 5 | 286 | 17 | 23 |
| 45 - 49 | 17,476 | 270 | 270 | 10 | - | - | - | - | 227 | 13 | 20 |
| 50 - 54 | 14,118 | 224 | 224 | 6 | - | 1 | - | 2 | 180 | 18 | 17 |
| 55 - 59 | 9,327 | 159 | 159 | 3 | 2 | 1 | - | 1 | 135 | 10 | 7 |
| 60 - 64 | 8,784 | 179 | 179 | 1 | 1 | - | - | 2 | 161 | 7 | 7 |
| 65 - 69 | 6,419 | 157 | 157 | 1 | - | - | - | - | 139 | 6 | 11 |
| 70 - 74 | 4,704 | 235 | 235 | 4 | 1 | 1 | - | - | 205 | 10 | 14 |
| 75 - 79 | 3,236 | 142 | 142 | 3 | - | 1 | - | 2 | 119 | 1 | 16 |
| 80 - 84 | 1,517 | 146 | 146 | 2 | 3 | 1 | - | 1 | 126 | 2 | 11 |
| 85 - 89 | 889 | 111 | 111 | 1 | 1 | 1 | - | - | 98 | 2 | 8 |
| 90 - 94 | 258 | 24 | 24 | - | - | - | - | - | 21 | 3 | - |
| 95+ | 468 | - | - | - | - | - | - | - | - | - | - |

## Table J4: Population (De Facto) and Number of Household Deaths 12 months prior to the Census by Cause of Death, 5 Year Age Group, Sex and Province, Zambia 2010

| Age Group, Sex and Province | Population | Household Deaths 12 Months Prior to the Census | Cause of Death | | | | | | | | |
|---|---|---|---|---|---|---|---|---|---|---|---|
| | | | Total | Accident | Injury | Suicide | Spousal Violence | Other Violence | Sickness/ Disease | Witchcraft | Other |
| **Copperbelt Province** | | | | | | | | | | | |
| **Total** | **1,920,611** | **25,784** | **25,784** | **825** | **143** | **180** | **65** | **261** | **20,815** | **859** | **2,636** |
| 0 - 4 | 288,184 | 6,983 | 6,983 | 122 | 30 | - | - | 25 | 5,167 | 261 | 1,378 |
| 5-9 | 248,404 | 938 | 938 | 49 | 7 | - | - | 2 | 764 | 47 | 69 |
| 10-14 | 258,545 | 663 | 663 | 47 | 11 | 6 | 1 | 10 | 491 | 46 | 51 |
| 15 - 19 | 244,789 | 968 | 968 | 52 | 13 | 19 | 6 | 14 | 747 | 40 | 77 |
| 20 - 24 | 183,304 | 1,443 | 1,443 | 107 | 10 | 32 | 5 | 40 | 1,102 | 49 | 98 |
| 25 - 29 | 167,226 | 2,172 | 2,172 | 102 | 16 | 32 | 13 | 31 | 1,775 | 63 | 140 |
| 30 - 34 | 133,289 | 2,479 | 2,479 | 85 | 7 | 25 | 16 | 39 | 2,074 | 75 | 158 |
| 35 - 39 | 106,800 | 2,111 | 2,111 | 63 | 15 | 21 | 5 | 26 | 1,799 | 66 | 116 |
| 40 - 44 | 75,181 | 1,510 | 1,510 | 44 | 6 | 14 | 5 | 19 | 1,302 | 52 | 68 |
| 45 - 49 | 60,717 | 1,229 | 1,229 | 40 | 6 | 14 | 3 | 9 | 1,035 | 40 | 82 |
| 50 - 54 | 47,907 | 908 | 908 | 27 | 2 | 4 | 3 | 14 | 771 | 29 | 58 |
| 55 - 59 | 34,061 | 704 | 704 | 27 | 3 | 1 | 1 | 7 | 587 | 23 | 55 |
| 60 - 64 | 26,641 | 776 | 776 | 20 | 4 | 6 | - | 6 | 665 | 17 | 58 |
| 65 - 69 | 17,738 | 668 | 668 | 10 | 4 | 2 | 2 | 4 | 597 | 17 | 32 |
| 70 - 74 | 12,520 | 714 | 714 | 15 | 1 | 2 | 1 | 2 | 637 | 11 | 45 |
| 75 - 79 | 7,946 | 579 | 579 | 7 | 1 | 1 | 1 | 6 | 499 | 15 | 49 |
| 80 - 84 | 3,967 | 490 | 490 | 7 | 7 | - | 1 | 2 | 429 | 3 | 41 |
| 85 - 89 | 2,098 | 381 | 381 | - | - | - | 2 | 5 | 317 | 5 | 52 |
| 90 - 94 | 654 | 68 | 68 | 1 | - | 1 | - | - | 57 | - | 9 |
| 95+ | 640 | - | - | - | - | - | - | - | - | - | - |
| | | | | | | | | | | | |
| **Male** | **950,775** | **13,791** | **13,791** | **586** | **92** | **142** | **25** | **208** | **10,846** | **491** | **1,401** |
| 0 - 4 | 143,515 | 3,638 | 3,638 | 63 | 14 | - | - | 16 | 2,682 | 136 | 727 |
| 5-9 | 122,799 | 490 | 490 | 36 | 3 | - | - | 2 | 379 | 26 | 44 |
| 10-14 | 125,884 | 371 | 371 | 27 | 5 | 4 | 1 | 7 | 269 | 30 | 28 |
| 15 - 19 | 118,176 | 463 | 463 | 38 | 10 | 8 | 1 | 8 | 340 | 24 | 34 |
| 20 - 24 | 85,557 | 718 | 718 | 76 | 7 | 26 | 2 | 31 | 496 | 30 | 50 |
| 25 - 29 | 80,086 | 1,067 | 1,067 | 73 | 12 | 26 | 2 | 27 | 825 | 34 | 68 |
| 30 - 34 | 68,847 | 1,343 | 1,343 | 69 | 5 | 23 | 6 | 33 | 1,101 | 33 | 73 |
| 35 - 39 | 56,451 | 1,200 | 1,200 | 55 | 11 | 18 | 2 | 22 | 981 | 48 | 63 |
| 40 - 44 | 40,340 | 921 | 921 | 36 | 4 | 13 | 1 | 17 | 788 | 24 | 38 |
| 45 - 49 | 30,500 | 710 | 710 | 32 | 4 | 9 | 3 | 7 | 584 | 28 | 43 |
| 50 - 54 | 23,568 | 515 | 515 | 17 | 1 | 4 | 2 | 12 | 421 | 22 | 36 |
| 55 - 59 | 17,929 | 421 | 421 | 21 | 3 | - | 1 | 5 | 338 | 17 | 36 |
| 60 - 64 | 13,898 | 444 | 444 | 16 | 2 | 6 | - | 5 | 369 | 10 | 36 |
| 65 - 69 | 9,062 | 356 | 356 | 7 | 4 | 2 | 2 | 4 | 311 | 9 | 17 |
| 70 - 74 | 6,317 | 368 | 368 | 10 | 1 | 2 | 1 | 1 | 313 | 8 | 32 |
| 75 - 79 | 4,224 | 305 | 305 | 5 | 1 | 1 | - | 5 | 258 | 8 | 27 |
| 80 - 84 | 1,990 | 227 | 227 | 4 | 5 | - | - | 2 | 195 | 2 | 19 |
| 85 - 89 | 1,051 | 197 | 197 | - | - | - | 1 | 4 | 168 | 2 | 22 |
| 90 - 94 | 343 | 37 | 37 | 1 | - | - | - | - | 28 | - | 8 |
| 95+ | 238 | - | - | - | - | - | - | - | - | - | - |

## Table J4: Population (De Facto) and Number of Household Deaths 12 months prior to the Census by Cause of Death, 5 Year Age Group, Sex and Province, Zambia 2010

| Age Group, Sex and Province | Population | Household Deaths 12 Months Prior to the Census | Cause of Death | | | | | | | | |
|---|---|---|---|---|---|---|---|---|---|---|---|
| | | | Total | Accident | Injury | Suicide | Spousal Violence | Other Violence | Sickness/ Disease | Witchcraft | Other |
| **Female** | **969,836** | **11,993** | **11,993** | **239** | **51** | **38** | **40** | **53** | **9,969** | **368** | **1,235** |
| 0 - 4 | 144,669 | 3,345 | 3,345 | 59 | 16 | - | - | 9 | 2,485 | 125 | 651 |
| 5-9 | 125,605 | 448 | 448 | 13 | 4 | - | - | - | 385 | 21 | 25 |
| 10-14 | 132,661 | 292 | 292 | 20 | 6 | 2 | - | 3 | 222 | 16 | 23 |
| 15 - 19 | 126,613 | 505 | 505 | 14 | 3 | 11 | 5 | 6 | 407 | 16 | 43 |
| 20 - 24 | 97,747 | 725 | 725 | 31 | 3 | 6 | 3 | 9 | 606 | 19 | 48 |
| 25 - 29 | 87,140 | 1,105 | 1,105 | 29 | 4 | 6 | 11 | 4 | 950 | 29 | 72 |
| 30 - 34 | 64,442 | 1,136 | 1,136 | 16 | 2 | 2 | 10 | 6 | 973 | 42 | 85 |
| 35 - 39 | 50,349 | 911 | 911 | 8 | 4 | 3 | 3 | 4 | 818 | 18 | 53 |
| 40 - 44 | 34,841 | 589 | 589 | 8 | 2 | 1 | 4 | 2 | 514 | 28 | 30 |
| 45 - 49 | 30,217 | 519 | 519 | 8 | 2 | 5 | - | 2 | 451 | 12 | 39 |
| 50 - 54 | 24,339 | 393 | 393 | 10 | 1 | - | 1 | 2 | 350 | 7 | 22 |
| 55 - 59 | 16,132 | 283 | 283 | 6 | - | 1 | - | 2 | 249 | 6 | 19 |
| 60 - 64 | 12,743 | 332 | 332 | 4 | 2 | - | - | 1 | 296 | 7 | 22 |
| 65 - 69 | 8,676 | 312 | 312 | 3 | - | - | - | - | 286 | 8 | 15 |
| 70 - 74 | 6,203 | 346 | 346 | 5 | - | - | - | 1 | 324 | 3 | 13 |
| 75 - 79 | 3,722 | 274 | 274 | 2 | - | - | 1 | 1 | 241 | 7 | 22 |
| 80 - 84 | 1,977 | 263 | 263 | 3 | 2 | - | 1 | - | 234 | 1 | 22 |
| 85 - 89 | 1,047 | 184 | 184 | - | - | - | 1 | 1 | 149 | 3 | 30 |
| 90 - 94 | 311 | 31 | 31 | - | - | 1 | - | - | 29 | - | 1 |
| 95+ | 402 | - | - | - | - | - | - | - | - | - | - |
| **Eastern Province** | | | | | | | | | | | |
| **Total** | **1,525,123** | **23,746** | **23,746** | **599** | **110** | **88** | **38** | **188** | **18,115** | **1,579** | **3,029** |
| 0 - 4 | 280,797 | 11,905 | 11,905 | 170 | 38 | - | - | 58 | 8,618 | 769 | 2,252 |
| 5-9 | 239,952 | 1,711 | 1,711 | 73 | 8 | - | - | 9 | 1,338 | 170 | 113 |
| 10-14 | 212,911 | 657 | 657 | 35 | 5 | 1 | - | 11 | 485 | 75 | 45 |
| 15 - 19 | 166,676 | 650 | 650 | 38 | 6 | 9 | 4 | 13 | 446 | 69 | 65 |
| 20 - 24 | 127,428 | 781 | 781 | 40 | 5 | 17 | 5 | 11 | 574 | 61 | 68 |
| 25 - 29 | 112,688 | 934 | 934 | 43 | 4 | 17 | 6 | 17 | 734 | 61 | 52 |
| 30 - 34 | 88,441 | 1,058 | 1,058 | 46 | 3 | 16 | 3 | 16 | 842 | 60 | 72 |
| 35 - 39 | 74,483 | 1,028 | 1,028 | 38 | 8 | 10 | 2 | 12 | 830 | 72 | 56 |
| 40 - 44 | 51,632 | 728 | 728 | 17 | 4 | 8 | 3 | 6 | 608 | 38 | 44 |
| 45 - 49 | 42,696 | 653 | 653 | 21 | 6 | 4 | 6 | 6 | 528 | 36 | 46 |
| 50 - 54 | 32,008 | 532 | 532 | 12 | 4 | 4 | 2 | 4 | 447 | 29 | 30 |
| 55 - 59 | 22,571 | 399 | 399 | 12 | 3 | 2 | - | 3 | 329 | 24 | 26 |
| 60 - 64 | 22,135 | 492 | 492 | 9 | 5 | - | 5 | 7 | 410 | 27 | 29 |
| 65 - 69 | 16,845 | 422 | 422 | 9 | 1 | - | - | 3 | 360 | 23 | 26 |
| 70 - 74 | 13,758 | 555 | 555 | 12 | 2 | - | - | 5 | 476 | 29 | 31 |
| 75 - 79 | 9,301 | 444 | 444 | 8 | 1 | - | - | 1 | 412 | 3 | 19 |
| 80 - 84 | 5,339 | 398 | 398 | 9 | 5 | - | 1 | 1 | 336 | 18 | 28 |
| 85 - 89 | 3,079 | 326 | 326 | 7 | 1 | - | - | 4 | 281 | 12 | 21 |
| 90 - 94 | 1,048 | 73 | 73 | - | 1 | - | 1 | 1 | 61 | 3 | 6 |
| 95+ | 1,335 | - | - | - | - | - | - | - | - | - | - |

## Table J4: Population (De Facto) and Number of Household Deaths 12 months prior to the Census by Cause of Death, 5 Year Age Group, Sex and Province, Zambia 2010

| Age Group, Sex and Province | Population | Household Deaths 12 Months Prior to the Census | Cause of Death | | | | | | | |
|---|---|---|---|---|---|---|---|---|---|---|
| | | | Total | Accident | Injury | Suicide | Spousal Violence | Other Violence | Sickness/ Disease | Witchcraft | Other |
| **Male** | **744,354** | **12,441** | **12,441** | **380** | **69** | **66** | **16** | **123** | **9,303** | **842** | **1,642** |
| 0 - 4 | 139,282 | 6,311 | 6,311 | 87 | 18 | - | - | 27 | 4,527 | 385 | 1,267 |
| 5-9 | 119,932 | 872 | 872 | 40 | 3 | - | - | 7 | 688 | 72 | 62 |
| 10-14 | 107,614 | 357 | 357 | 24 | 2 | 1 | - | 8 | 256 | 44 | 22 |
| 15 - 19 | 83,450 | 294 | 294 | 19 | 3 | 8 | 1 | 9 | 200 | 30 | 24 |
| 20 - 24 | 58,428 | 343 | 343 | 28 | 4 | 9 | 1 | 9 | 232 | 40 | 20 |
| 25 - 29 | 51,998 | 457 | 457 | 35 | 3 | 12 | 2 | 13 | 336 | 38 | 18 |
| 30 - 34 | 43,583 | 531 | 531 | 31 | 2 | 13 | 1 | 14 | 408 | 28 | 34 |
| 35 - 39 | 37,319 | 557 | 557 | 27 | 8 | 8 | 1 | 12 | 424 | 47 | 30 |
| 40 - 44 | 25,887 | 407 | 407 | 15 | 3 | 6 | - | 2 | 333 | 24 | 24 |
| 45 - 49 | 20,727 | 388 | 388 | 14 | 6 | 4 | 5 | 5 | 295 | 28 | 31 |
| 50 - 54 | 14,882 | 293 | 293 | 12 | 4 | 4 | 1 | 4 | 233 | 19 | 16 |
| 55 - 59 | 10,614 | 227 | 227 | 9 | 2 | 1 | - | 1 | 183 | 18 | 13 |
| 60 - 64 | 9,023 | 272 | 272 | 6 | 4 | - | 4 | 5 | 226 | 13 | 14 |
| 65 - 69 | 6,949 | 214 | 214 | 8 | 1 | - | - | 1 | 177 | 10 | 17 |
| 70 - 74 | 5,671 | 313 | 313 | 8 | 2 | - | - | 3 | 259 | 23 | 18 |
| 75 - 79 | 4,320 | 229 | 229 | 6 | 1 | - | - | - | 213 | 2 | 7 |
| 80 - 84 | 2,318 | 194 | 194 | 5 | 1 | - | - | - | 164 | 12 | 12 |
| 85 - 89 | 1,421 | 146 | 146 | 6 | 1 | - | - | 2 | 120 | 7 | 10 |
| 90 - 94 | 459 | 36 | 36 | - | 1 | - | - | 1 | 29 | 2 | 3 |
| 95+ | 477 | - | - | - | - | - | - | - | - | - | - |
| | | | | | | | | | | | |
| **Female** | **780,769** | **11,305** | **11,305** | **219** | **41** | **22** | **22** | **65** | **8,812** | **737** | **1,387** |
| 0 - 4 | 141,515 | 5,594 | 5,594 | 83 | 20 | - | - | 31 | 4,091 | 384 | 985 |
| 5-9 | 120,020 | 839 | 839 | 33 | 5 | - | - | 2 | 650 | 98 | 51 |
| 10-14 | 105,297 | 300 | 300 | 11 | 3 | - | - | 3 | 229 | 31 | 23 |
| 15 - 19 | 83,226 | 356 | 356 | 19 | 3 | 1 | 3 | 4 | 246 | 39 | 41 |
| 20 - 24 | 69,000 | 438 | 438 | 12 | 1 | 8 | 4 | 2 | 342 | 21 | 48 |
| 25 - 29 | 60,690 | 477 | 477 | 8 | 1 | 5 | 4 | 4 | 398 | 23 | 34 |
| 30 - 34 | 44,858 | 527 | 527 | 15 | 1 | 3 | 2 | 2 | 434 | 32 | 38 |
| 35 - 39 | 37,164 | 471 | 471 | 11 | - | 2 | 1 | - | 406 | 25 | 26 |
| 40 - 44 | 25,745 | 321 | 321 | 2 | 1 | 2 | 3 | 4 | 275 | 14 | 20 |
| 45 - 49 | 21,969 | 265 | 265 | 7 | - | - | 1 | 1 | 233 | 8 | 15 |
| 50 - 54 | 17,126 | 239 | 239 | - | - | - | 1 | - | 214 | 10 | 14 |
| 55 - 59 | 11,957 | 172 | 172 | 3 | 1 | 1 | - | 2 | 146 | 6 | 13 |
| 60 - 64 | 13,112 | 220 | 220 | 3 | 1 | - | 1 | 2 | 184 | 14 | 15 |
| 65 - 69 | 9,896 | 208 | 208 | 1 | - | - | - | 2 | 183 | 13 | 9 |
| 70 - 74 | 8,087 | 242 | 242 | 4 | - | - | - | 2 | 217 | 6 | 13 |
| 75 - 79 | 4,981 | 215 | 215 | 2 | - | - | - | 1 | 199 | 1 | 12 |
| 80 - 84 | 3,021 | 204 | 204 | 4 | 4 | - | 1 | 1 | 172 | 6 | 16 |
| 85 - 89 | 1,658 | 180 | 180 | 1 | - | - | - | 2 | 161 | 5 | 11 |
| 90 - 94 | 589 | 37 | 37 | - | - | - | 1 | - | 32 | 1 | 3 |
| 95+ | 858 | - | - | - | - | - | - | - | - | - | - |

## Table J4: Population (De Facto) and Number of Household Deaths 12 months prior to the Census by Cause of Death, 5 Year Age Group, Sex and Province, Zambia 2010

| Age Group, Sex and Province | Population | Household Deaths 12 Months Prior to the Census | Cause of Death | | | | | | | |
|---|---|---|---|---|---|---|---|---|---|---|
| | | | Total | Accident | Injury | Suicide | Spousal Violence | Other Violence | Sickness/ Disease | Witchcraft | Other |
| **Luapula Province** | | | | | | | | | | | |
| **Total** | **938,391** | **16,216** | **16,216** | **525** | **63** | **78** | **31** | **133** | **12,247** | **1,497** | **1,642** |
| 0 - 4 | 178,815 | 7,708 | 7,708 | 97 | 27 | - | - | 53 | 5,905 | 573 | 1,053 |
| 5-9 | 149,455 | 1,064 | 1,064 | 54 | - | - | - | 3 | 812 | 126 | 69 |
| 10-14 | 130,510 | 580 | 580 | 42 | 5 | 2 | - | 7 | 396 | 82 | 46 |
| 15 - 19 | 100,669 | 582 | 582 | 54 | 3 | 10 | 2 | 7 | 365 | 87 | 54 |
| 20 - 24 | 73,509 | 790 | 790 | 61 | 2 | 21 | 6 | 11 | 532 | 102 | 55 |
| 25 - 29 | 67,803 | 871 | 871 | 60 | 5 | 15 | 5 | 11 | 628 | 82 | 65 |
| 30 - 34 | 54,625 | 859 | 859 | 42 | 2 | 14 | 3 | 9 | 638 | 89 | 62 |
| 35 - 39 | 45,626 | 715 | 715 | 36 | 2 | 5 | 4 | 7 | 543 | 77 | 41 |
| 40 - 44 | 34,119 | 541 | 541 | 18 | 2 | 2 | 4 | 4 | 415 | 58 | 38 |
| 45 - 49 | 27,403 | 421 | 421 | 15 | 5 | 2 | 2 | 4 | 317 | 54 | 22 |
| 50 - 54 | 21,820 | 338 | 338 | 11 | 2 | 1 | 2 | 5 | 265 | 37 | 15 |
| 55 - 59 | 14,911 | 251 | 251 | 7 | - | 2 | 1 | 1 | 209 | 26 | 5 |
| 60 - 64 | 13,597 | 316 | 316 | 9 | - | - | - | 2 | 259 | 28 | 18 |
| 65 - 69 | 9,773 | 270 | 270 | 3 | 2 | - | 2 | 1 | 229 | 23 | 10 |
| 70 - 74 | 7,261 | 337 | 337 | 6 | 2 | 2 | - | 3 | 270 | 30 | 24 |
| 75 - 79 | 4,535 | 247 | 247 | 2 | 2 | 1 | - | 3 | 203 | 15 | 21 |
| 80 - 84 | 2,166 | 182 | 182 | 4 | 1 | 1 | - | 2 | 148 | 4 | 22 |
| 85 - 89 | 1,152 | 123 | 123 | 2 | 1 | - | - | - | 97 | 4 | 19 |
| 90 - 94 | 322 | 21 | 21 | 2 | - | - | - | - | 16 | - | 3 |
| 95+ | 320 | - | - | - | - | - | - | - | - | - | - |
| | | | | | | | | | | | |
| **Male** | **455,046** | **8,659** | **8,659** | **394** | **35** | **50** | **8** | **83** | **6,338** | **839** | **912** |
| 0 - 4 | 88,914 | 4,200 | 4,200 | 63 | 13 | - | - | 28 | 3,155 | 331 | 610 |
| 5-9 | 74,858 | 588 | 588 | 39 | - | - | - | 2 | 438 | 72 | 37 |
| 10-14 | 65,735 | 316 | 316 | 30 | 3 | 1 | - | 5 | 205 | 40 | 32 |
| 15 - 19 | 48,909 | 288 | 288 | 37 | 2 | 5 | 1 | 3 | 168 | 50 | 22 |
| 20 - 24 | 31,505 | 348 | 348 | 46 | 1 | 11 | 3 | 7 | 210 | 49 | 21 |
| 25 - 29 | 29,488 | 392 | 392 | 49 | 3 | 11 | 1 | 10 | 255 | 37 | 26 |
| 30 - 34 | 26,184 | 445 | 445 | 36 | 1 | 10 | - | 6 | 312 | 52 | 28 |
| 35 - 39 | 22,531 | 381 | 381 | 33 | - | 3 | 1 | 5 | 277 | 39 | 23 |
| 40 - 44 | 17,384 | 330 | 330 | 14 | 1 | 2 | 1 | 3 | 249 | 34 | 26 |
| 45 - 49 | 13,487 | 253 | 253 | 11 | 5 | 1 | - | 3 | 183 | 36 | 14 |
| 50 - 54 | 10,527 | 192 | 192 | 9 | 1 | 1 | - | 4 | 138 | 26 | 13 |
| 55 - 59 | 6,953 | 144 | 144 | 5 | - | 2 | - | 1 | 117 | 16 | 3 |
| 60 - 64 | 6,054 | 176 | 176 | 8 | - | - | - | 2 | 146 | 12 | 8 |
| 65 - 69 | 4,508 | 134 | 134 | 2 | 1 | - | 1 | 1 | 111 | 12 | 6 |
| 70 - 74 | 3,483 | 172 | 172 | 4 | 1 | 2 | - | - | 139 | 17 | 9 |
| 75 - 79 | 2,474 | 126 | 126 | 1 | 2 | 1 | - | 1 | 101 | 10 | 10 |
| 80 - 84 | 1,164 | 97 | 97 | 3 | 1 | - | - | 2 | 77 | 2 | 12 |
| 85 - 89 | 612 | 64 | 64 | 2 | - | - | - | - | 47 | 4 | 11 |
| 90 - 94 | 168 | 13 | 13 | 2 | - | - | - | - | 10 | - | 1 |
| 95+ | 108 | - | - | - | - | - | - | - | - | - | - |

## Table J4: Population (De Facto) and Number of Household Deaths 12 months prior to the Census by Cause of Death, 5 Year Age Group, Sex and Province, Zambia 2010

| Age Group, Sex and Province | Population | Household Deaths 12 Months Prior to the Census | Cause of Death | | | | | | | | |
|---|---|---|---|---|---|---|---|---|---|---|---|
| | | | Total | Accident | Injury | Suicide | Spousal Violence | Other Violence | Sickness/ Disease | Witchcraft | Other |
| **Female** | **483,345** | **7,557** | **7,557** | **131** | **28** | **28** | **23** | **50** | **5,909** | **658** | **730** |
| 0 - 4 | 89,901 | 3,508 | 3,508 | 34 | 14 | - | - | 25 | 2,750 | 242 | 443 |
| 5-9 | 74,597 | 476 | 476 | 15 | - | - | - | 1 | 374 | 54 | 32 |
| 10-14 | 64,775 | 264 | 264 | 12 | 2 | 1 | - | 2 | 191 | 42 | 14 |
| 15 - 19 | 51,760 | 294 | 294 | 17 | 1 | 5 | 1 | 4 | 197 | 37 | 32 |
| 20 - 24 | 42,004 | 442 | 442 | 15 | 1 | 10 | 3 | 4 | 322 | 53 | 34 |
| 25 - 29 | 38,315 | 479 | 479 | 11 | 2 | 4 | 4 | 1 | 373 | 45 | 39 |
| 30 - 34 | 28,441 | 414 | 414 | 6 | 1 | 4 | 3 | 3 | 326 | 37 | 34 |
| 35 - 39 | 23,095 | 334 | 334 | 3 | 2 | 2 | 3 | 2 | 266 | 38 | 18 |
| 40 - 44 | 16,735 | 211 | 211 | 4 | 1 | - | 3 | 1 | 166 | 24 | 12 |
| 45 - 49 | 13,916 | 168 | 168 | 4 | - | 1 | 2 | 1 | 134 | 18 | 8 |
| 50 - 54 | 11,293 | 146 | 146 | 2 | 1 | - | 2 | 1 | 127 | 11 | 2 |
| 55 - 59 | 7,958 | 107 | 107 | 2 | - | - | 1 | - | 92 | 10 | 2 |
| 60 - 64 | 7,543 | 140 | 140 | 1 | - | - | - | - | 113 | 16 | 10 |
| 65 - 69 | 5,265 | 136 | 136 | 1 | 1 | - | 1 | - | 118 | 11 | 4 |
| 70 - 74 | 3,778 | 165 | 165 | 2 | 1 | - | - | 3 | 131 | 13 | 15 |
| 75 - 79 | 2,061 | 121 | 121 | 1 | - | - | - | 2 | 102 | 5 | 11 |
| 80 - 84 | 1,002 | 85 | 85 | 1 | - | 1 | - | - | 71 | 2 | 10 |
| 85 - 89 | 540 | 59 | 59 | - | 1 | - | - | - | 50 | - | 8 |
| 90 - 94 | 154 | 8 | 8 | - | - | - | - | - | 6 | - | 2 |
| 95+ | 212 | - | - | - | - | - | - | - | - | - | - |
| **Lusaka Province** | | | | | | | | | | | |
| **Total** | **2,138,907** | **24,813** | **24,813** | **1,002** | **153** | **175** | **32** | **237** | **19,283** | **693** | **3,238** |
| 0 - 4 | 330,249 | 7,911 | 7,911 | 197 | 39 | - | - | 39 | 5,462 | 242 | 1,932 |
| 5-9 | 274,057 | 816 | 816 | 52 | 10 | - | - | 9 | 636 | 37 | 72 |
| 10-14 | 267,942 | 615 | 615 | 65 | 8 | 3 | - | 8 | 456 | 24 | 51 |
| 15 - 19 | 246,301 | 931 | 931 | 69 | 7 | 19 | 4 | 12 | 690 | 46 | 84 |
| 20 - 24 | 224,293 | 1,474 | 1,474 | 95 | 12 | 42 | 6 | 31 | 1,121 | 49 | 118 |
| 25 - 29 | 214,183 | 2,035 | 2,035 | 102 | 18 | 41 | 2 | 39 | 1,624 | 60 | 149 |
| 30 - 34 | 174,999 | 2,259 | 2,259 | 109 | 14 | 23 | 7 | 30 | 1,845 | 52 | 179 |
| 35 - 39 | 135,925 | 2,014 | 2,014 | 101 | 12 | 16 | 4 | 21 | 1,673 | 38 | 149 |
| 40 - 44 | 84,105 | 1,466 | 1,466 | 52 | 6 | 12 | 3 | 18 | 1,236 | 31 | 108 |
| 45 - 49 | 59,683 | 1,139 | 1,139 | 48 | 5 | 7 | 3 | 5 | 971 | 29 | 71 |
| 50 - 54 | 42,330 | 838 | 838 | 25 | 3 | 3 | - | 8 | 710 | 26 | 63 |
| 55 - 59 | 28,016 | 623 | 623 | 20 | 1 | 2 | - | 5 | 540 | 10 | 45 |
| 60 - 64 | 21,101 | 618 | 618 | 18 | 5 | 1 | 1 | 3 | 528 | 17 | 45 |
| 65 - 69 | 13,619 | 489 | 489 | 18 | 3 | 1 | - | 3 | 422 | 13 | 29 |
| 70 - 74 | 9,344 | 524 | 524 | 12 | - | 2 | - | 2 | 468 | 7 | 33 |
| 75 - 79 | 6,055 | 410 | 410 | 10 | 3 | - | - | 1 | 353 | 8 | 35 |
| 80 - 84 | 3,281 | 356 | 356 | 7 | 3 | 3 | 2 | 3 | 297 | 2 | 39 |
| 85 - 89 | 1,999 | 242 | 242 | 2 | 4 | - | - | - | 205 | 2 | 29 |
| 90 - 94 | 641 | 53 | 53 | - | - | - | - | - | 46 | - | 7 |
| 95+ | 784 | - | - | - | - | - | - | - | - | - | - |

## Table J4: Population (De Facto) and Number of Household Deaths 12 months prior to the Census by Cause of Death, 5 Year Age Group, Sex and Province, Zambia 2010

| Age Group, Sex and Province | Population | Household Deaths 12 Months Prior to the Census | Cause of Death | | | | | | | | |
|---|---|---|---|---|---|---|---|---|---|---|---|
| | | | Total | Accident | Injury | Suicide | Spousal Violence | Other Violence | Sickness/ Disease | Witchcraft | Other |
| Male | 1,049,215 | 13,415 | 13,415 | 687 | 104 | 140 | 14 | 179 | 10,140 | 360 | 1,791 |
| 0 - 4 | 164,089 | 4,152 | 4,152 | 105 | 26 | - | - | 24 | 2,840 | 108 | 1,049 |
| 5-9 | 134,904 | 445 | 445 | 34 | 6 | - | - | 2 | 337 | 24 | 42 |
| 10-14 | 126,766 | 298 | 298 | 48 | 7 | 1 | - | 5 | 198 | 10 | 29 |
| 15 - 19 | 114,904 | 446 | 446 | 47 | 3 | 10 | 1 | 10 | 317 | 20 | 38 |
| 20 - 24 | 100,177 | 718 | 718 | 73 | 8 | 32 | 2 | 27 | 490 | 24 | 62 |
| 25 - 29 | 101,691 | 1,081 | 1,081 | 71 | 15 | 35 | 1 | 30 | 807 | 38 | 84 |
| 30 - 34 | 89,969 | 1,250 | 1,250 | 84 | 10 | 17 | 4 | 26 | 976 | 29 | 104 |
| 35 - 39 | 75,312 | 1,235 | 1,235 | 77 | 11 | 15 | 2 | 17 | 997 | 23 | 93 |
| 40 - 44 | 47,555 | 899 | 899 | 39 | 4 | 12 | 1 | 14 | 745 | 13 | 71 |
| 45 - 49 | 31,442 | 709 | 709 | 30 | 5 | 7 | 1 | 5 | 588 | 25 | 48 |
| 50 - 54 | 21,064 | 478 | 478 | 17 | 1 | 2 | - | 6 | 391 | 16 | 45 |
| 55 - 59 | 14,318 | 349 | 349 | 15 | 1 | 2 | - | 5 | 298 | 3 | 25 |
| 60 - 64 | 10,517 | 329 | 329 | 14 | 1 | 1 | 1 | 2 | 276 | 10 | 24 |
| 65 - 69 | 6,640 | 241 | 241 | 12 | 1 | 1 | - | 2 | 211 | 2 | 12 |
| 70 - 74 | 4,331 | 286 | 286 | 10 | - | 2 | - | 2 | 249 | 7 | 16 |
| 75 - 79 | 2,731 | 196 | 196 | 5 | 2 | - | - | 1 | 168 | 8 | 12 |
| 80 - 84 | 1,438 | 169 | 169 | 5 | 2 | 3 | 1 | 1 | 138 | - | 19 |
| 85 - 89 | 861 | 110 | 110 | 1 | 1 | - | - | - | 92 | - | 16 |
| 90 - 94 | 286 | 24 | 24 | - | - | - | - | - | 22 | - | 2 |
| 95+ | 220 | - | - | - | - | - | - | - | - | - | - |
| | | | | | | | | | | | |
| **Female** | **1,089,692** | **11,398** | **11,398** | **315** | **49** | **35** | **18** | **58** | **9,143** | **333** | **1,447** |
| 0 - 4 | 166,160 | 3,759 | 3,759 | 92 | 13 | - | - | 15 | 2,622 | 134 | 883 |
| 5-9 | 139,153 | 371 | 371 | 18 | 4 | - | - | 7 | 299 | 13 | 30 |
| 10-14 | 141,176 | 317 | 317 | 17 | 1 | 2 | - | 3 | 258 | 14 | 22 |
| 15 - 19 | 131,397 | 485 | 485 | 22 | 4 | 9 | 3 | 2 | 373 | 26 | 46 |
| 20 - 24 | 124,116 | 756 | 756 | 22 | 4 | 10 | 4 | 4 | 631 | 25 | 56 |
| 25 - 29 | 112,492 | 954 | 954 | 31 | 3 | 6 | 1 | 9 | 817 | 22 | 65 |
| 30 - 34 | 85,030 | 1,009 | 1,009 | 25 | 4 | 6 | 3 | 4 | 869 | 23 | 75 |
| 35 - 39 | 60,613 | 779 | 779 | 24 | 1 | 1 | 2 | 4 | 676 | 15 | 56 |
| 40 - 44 | 36,550 | 567 | 567 | 13 | 2 | - | 2 | 4 | 491 | 18 | 37 |
| 45 - 49 | 28,241 | 430 | 430 | 18 | - | - | 2 | - | 383 | 4 | 23 |
| 50 - 54 | 21,266 | 360 | 360 | 8 | 2 | 1 | - | 2 | 319 | 10 | 18 |
| 55 - 59 | 13,698 | 274 | 274 | 5 | - | - | - | - | 242 | 7 | 20 |
| 60 - 64 | 10,584 | 289 | 289 | 4 | 4 | - | - | 1 | 252 | 7 | 21 |
| 65 - 69 | 6,979 | 248 | 248 | 6 | 2 | - | - | 1 | 211 | 11 | 17 |
| 70 - 74 | 5,013 | 238 | 238 | 2 | - | - | - | - | 219 | - | 17 |
| 75 - 79 | 3,324 | 214 | 214 | 5 | 1 | - | - | - | 185 | - | 23 |
| 80 - 84 | 1,843 | 187 | 187 | 2 | 1 | - | 1 | 2 | 159 | 2 | 20 |
| 85 - 89 | 1,138 | 132 | 132 | 1 | 3 | - | - | - | 113 | 2 | 13 |
| 90 - 94 | 355 | 29 | 29 | - | - | - | - | - | 24 | - | 5 |
| 95+ | 564 | - | - | - | - | - | - | - | - | - | - |

## Table J4: Population (De Facto) and Number of Household Deaths 12 months prior to the Census by Cause of Death, 5 Year Age Group, Sex and Province, Zambia 2010

| Age Group, Sex and Province | Population | Household Deaths 12 Months Prior to the Census | Cause of Death | | | | | | | | |
|---|---|---|---|---|---|---|---|---|---|---|---|
| | | | Total | Accident | Injury | Suicide | Spousal Violence | Other Violence | Sickness/ Disease | Witchcraft | Other |
| **Muchinga Province** | | | | | | | | | | | |
| **Total** | **677,507** | **8,389** | **8,389** | **291** | **40** | **45** | **14** | **110** | **5,826** | **892** | **1,171** |
| 0 - 4 | 130,097 | 4,157 | 4,157 | 91 | 10 | - | - | 59 | 2,745 | 401 | 851 |
| 5-9 | 111,376 | 515 | 515 | 17 | 4 | - | - | 7 | 377 | 69 | 41 |
| 10-14 | 94,047 | 293 | 293 | 22 | 1 | 3 | - | 4 | 193 | 48 | 22 |
| 15 - 19 | 72,759 | 292 | 292 | 27 | 2 | 4 | - | 4 | 182 | 47 | 26 |
| 20 - 24 | 55,816 | 339 | 339 | 24 | 4 | 8 | 3 | 8 | 216 | 47 | 29 |
| 25 - 29 | 49,634 | 392 | 392 | 31 | 4 | 9 | 1 | 9 | 272 | 39 | 27 |
| 30 - 34 | 37,396 | 392 | 392 | 16 | 3 | 3 | 1 | 4 | 289 | 40 | 36 |
| 35 - 39 | 31,699 | 329 | 329 | 25 | 2 | 5 | 2 | 4 | 236 | 32 | 23 |
| 40 - 44 | 22,491 | 274 | 274 | 13 | - | 3 | 2 | - | 209 | 29 | 18 |
| 45 - 49 | 18,634 | 223 | 223 | 4 | 2 | 4 | 3 | 5 | 161 | 26 | 18 |
| 50 - 54 | 13,900 | 194 | 194 | 7 | 2 | - | 1 | - | 147 | 21 | 16 |
| 55 - 59 | 9,424 | 139 | 139 | 2 | - | 2 | - | - | 101 | 26 | 8 |
| 60 - 64 | 9,342 | 149 | 149 | 2 | 2 | - | - | 1 | 120 | 18 | 6 |
| 65 - 69 | 7,276 | 149 | 149 | 4 | - | 2 | - | - | 120 | 15 | 8 |
| 70 - 74 | 5,810 | 186 | 186 | 1 | 2 | 1 | - | 3 | 151 | 14 | 14 |
| 75 - 79 | 3,986 | 133 | 133 | 3 | - | - | 1 | 2 | 109 | 11 | 7 |
| 80 - 84 | 2,054 | 133 | 133 | 1 | 1 | 1 | - | - | 115 | 7 | 8 |
| 85 - 89 | 1,068 | 82 | 82 | 1 | 1 | - | - | - | 71 | 2 | 7 |
| 90 - 94 | 322 | 18 | 18 | - | - | - | - | - | 12 | - | 6 |
| 95+ | 376 | - | - | - | - | - | - | - | - | - | - |
| | | | | | | | | | | | |
| **Male** | **328,980** | **4,621** | **4,621** | **204** | **31** | **33** | **5** | **67** | **3,130** | **490** | **661** |
| 0 - 4 | 64,625 | 2,287 | 2,287 | 52 | 9 | - | - | 33 | 1,493 | 209 | 491 |
| 5-9 | 55,735 | 293 | 293 | 10 | 4 | - | - | 5 | 212 | 41 | 21 |
| 10-14 | 47,482 | 144 | 144 | 16 | - | 2 | - | 2 | 89 | 26 | 9 |
| 15 - 19 | 35,279 | 141 | 141 | 19 | 1 | 3 | - | 2 | 83 | 22 | 11 |
| 20 - 24 | 24,480 | 176 | 176 | 22 | 4 | 6 | 1 | 6 | 105 | 20 | 12 |
| 25 - 29 | 22,242 | 196 | 196 | 24 | 3 | 6 | - | 6 | 125 | 20 | 12 |
| 30 - 34 | 18,255 | 209 | 209 | 12 | 2 | 2 | - | 3 | 148 | 24 | 18 |
| 35 - 39 | 15,645 | 191 | 191 | 21 | 1 | 3 | - | 3 | 133 | 19 | 11 |
| 40 - 44 | 11,247 | 167 | 167 | 11 | - | 3 | 1 | - | 124 | 17 | 11 |
| 45 - 49 | 8,983 | 143 | 143 | 4 | 1 | 4 | 1 | 4 | 96 | 18 | 15 |
| 50 - 54 | 6,589 | 119 | 119 | 5 | 1 | - | 1 | - | 81 | 17 | 14 |
| 55 - 59 | 4,469 | 83 | 83 | 2 | - | 2 | - | - | 58 | 15 | 6 |
| 60 - 64 | 3,975 | 97 | 97 | 1 | 2 | - | - | 1 | 76 | 12 | 5 |
| 65 - 69 | 3,182 | 73 | 73 | 3 | - | 1 | - | - | 59 | 7 | 3 |
| 70 - 74 | 2,747 | 102 | 102 | - | 1 | 1 | - | 2 | 80 | 10 | 8 |
| 75 - 79 | 2,066 | 77 | 77 | 1 | - | - | 1 | - | 64 | 7 | 4 |
| 80 - 84 | 1,103 | 69 | 69 | 1 | 1 | - | - | - | 60 | 5 | 2 |
| 85 - 89 | 565 | 45 | 45 | - | 1 | - | - | - | 38 | 1 | 5 |
| 90 - 94 | 162 | 9 | 9 | - | - | - | - | - | 6 | - | 3 |
| 95+ | 149 | - | - | - | - | - | - | - | - | - | - |

## Table J4: Population (De Facto) and Number of Household Deaths 12 months prior to the Census by Cause of Death, 5 Year Age Group, Sex and Province, Zambia 2010

| Age Group, Sex and Province | Population | Household Deaths 12 Months Prior to the Census | Cause of Death | | | | | | | | |
|---|---|---|---|---|---|---|---|---|---|---|---|
| | | | Total | Accident | Injury | Suicide | Spousal Violence | Other Violence | Sickness/ Disease | Witchcraft | Other |
| **Female** | **348,527** | **3,768** | **3,768** | **87** | **9** | **12** | **9** | **43** | **2,696** | **402** | **510** |
| 0 - 4 | 65,472 | 1,870 | 1,870 | 39 | 1 | - | - | 26 | 1,252 | 192 | 360 |
| 5-9 | 55,641 | 222 | 222 | 7 | - | - | - | 2 | 165 | 28 | 20 |
| 10-14 | 46,565 | 149 | 149 | 6 | 1 | 1 | - | 2 | 104 | 22 | 13 |
| 15 - 19 | 37,480 | 151 | 151 | 8 | 1 | 1 | - | 2 | 99 | 25 | 15 |
| 20 - 24 | 31,336 | 163 | 163 | 2 | - | 2 | 2 | 2 | 111 | 27 | 17 |
| 25 - 29 | 27,392 | 196 | 196 | 7 | 1 | 3 | 1 | 3 | 147 | 19 | 15 |
| 30 - 34 | 19,141 | 183 | 183 | 4 | 1 | 1 | 1 | 1 | 141 | 16 | 18 |
| 35 - 39 | 16,054 | 138 | 138 | 4 | 1 | 2 | 2 | 1 | 103 | 13 | 12 |
| 40 - 44 | 11,244 | 107 | 107 | 2 | - | - | 1 | - | 85 | 12 | 7 |
| 45 - 49 | 9,651 | 80 | 80 | - | 1 | - | 2 | 1 | 65 | 8 | 3 |
| 50 - 54 | 7,311 | 75 | 75 | 2 | 1 | - | - | - | 66 | 4 | 2 |
| 55 - 59 | 4,955 | 56 | 56 | - | - | - | - | - | 43 | 11 | 2 |
| 60 - 64 | 5,367 | 52 | 52 | 1 | - | - | - | - | 44 | 6 | 1 |
| 65 - 69 | 4,094 | 76 | 76 | 1 | - | 1 | - | - | 61 | 8 | 5 |
| 70 - 74 | 3,063 | 84 | 84 | 1 | 1 | - | - | 1 | 71 | 4 | 6 |
| 75 - 79 | 1,920 | 56 | 56 | 2 | - | - | - | 2 | 45 | 4 | 3 |
| 80 - 84 | 951 | 64 | 64 | - | - | 1 | - | - | 55 | 2 | 6 |
| 85 - 89 | 503 | 37 | 37 | 1 | - | - | - | - | 33 | 1 | 2 |
| 90 - 94 | 160 | 9 | 9 | - | - | - | - | - | 6 | - | 3 |
| 95+ | 227 | - | - | - | - | - | - | - | - | - | - |
| **Northern Province** | | | | | | | | | | | |
| **Total** | **1,044,955** | **13,062** | **13,062** | **413** | **60** | **58** | **17** | **80** | **9,492** | **1,263** | **1,679** |
| 0 - 4 | 205,958 | 6,386 | 6,386 | 113 | 17 | - | - | 31 | 4,496 | 513 | 1,216 |
| 5-9 | 169,675 | 789 | 789 | 38 | 2 | - | - | 3 | 594 | 101 | 51 |
| 10-14 | 144,083 | 454 | 454 | 30 | 3 | 2 | 2 | 3 | 302 | 78 | 34 |
| 15 - 19 | 111,259 | 485 | 485 | 29 | 10 | 9 | 1 | 3 | 320 | 60 | 53 |
| 20 - 24 | 84,699 | 530 | 530 | 41 | 3 | 17 | 3 | 4 | 378 | 50 | 34 |
| 25 - 29 | 76,556 | 669 | 669 | 41 | 5 | 11 | 6 | 5 | 478 | 74 | 49 |
| 30 - 34 | 56,707 | 592 | 592 | 28 | 3 | 6 | - | 4 | 441 | 68 | 42 |
| 35 - 39 | 49,211 | 538 | 538 | 22 | 3 | 2 | 1 | 9 | 402 | 63 | 36 |
| 40 - 44 | 34,969 | 423 | 423 | 18 | 4 | 3 | 2 | 4 | 298 | 58 | 36 |
| 45 - 49 | 29,770 | 336 | 336 | 20 | 1 | 3 | 1 | 4 | 245 | 40 | 22 |
| 50 - 54 | 22,143 | 292 | 292 | 11 | 3 | 1 | - | 3 | 218 | 40 | 16 |
| 55 - 59 | 15,358 | 238 | 238 | 3 | 2 | - | - | 2 | 204 | 17 | 10 |
| 60 - 64 | 13,948 | 238 | 238 | 3 | - | 1 | - | 1 | 201 | 22 | 10 |
| 65 - 69 | 11,106 | 235 | 235 | 7 | - | 1 | - | 2 | 197 | 24 | 4 |
| 70 - 74 | 8,306 | 279 | 279 | 2 | - | - | - | - | 238 | 23 | 16 |
| 75 - 79 | 5,681 | 200 | 200 | 3 | 1 | - | - | - | 171 | 13 | 12 |
| 80 - 84 | 2,926 | 194 | 194 | 3 | 1 | 1 | - | 1 | 166 | 7 | 15 |
| 85 - 89 | 1,576 | 151 | 151 | 1 | 2 | 1 | 1 | 1 | 114 | 11 | 20 |
| 90 - 94 | 458 | 33 | 33 | - | - | - | - | - | 29 | 1 | 3 |
| 95+ | 566 | - | - | - | - | - | - | - | - | - | - |

## Table J4: Population (De Facto) and Number of Household Deaths 12 months prior to the Census by Cause of Death, 5 Year Age Group, Sex and Province, Zambia 2010

| Age Group, Sex and Province | Population | Household Deaths 12 Months Prior to the Census | Cause of Death | | | | | | | | |
|---|---|---|---|---|---|---|---|---|---|---|---|
| | | | Total | Accident | Injury | Suicide | Spousal Violence | Other Violence | Sickness/ Disease | Witchcraft | Other |
| **Male** | **508,226** | **7,079** | **7,079** | **283** | **37** | **42** | **7** | **52** | **5,067** | **690** | **901** |
| 0 - 4 | 102,856 | 3,506 | 3,506 | 61 | 12 | - | - | 15 | 2,465 | 264 | 689 |
| 5-9 | 84,431 | 430 | 430 | 24 | 1 | - | - | - | 319 | 56 | 30 |
| 10-14 | 72,726 | 213 | 213 | 15 | 1 | 1 | 2 | 2 | 141 | 34 | 17 |
| 15 - 19 | 53,811 | 224 | 224 | 21 | 4 | 4 | - | 3 | 149 | 25 | 18 |
| 20 - 24 | 36,981 | 267 | 267 | 32 | 2 | 12 | 2 | 3 | 177 | 29 | 10 |
| 25 - 29 | 34,262 | 324 | 324 | 30 | 2 | 9 | 1 | 3 | 224 | 34 | 21 |
| 30 - 34 | 27,506 | 314 | 314 | 23 | - | 5 | - | 3 | 223 | 42 | 18 |
| 35 - 39 | 24,313 | 298 | 298 | 20 | 2 | 2 | - | 7 | 219 | 37 | 11 |
| 40 - 44 | 17,642 | 279 | 279 | 15 | 4 | 3 | 1 | 3 | 194 | 38 | 21 |
| 45 - 49 | 14,481 | 203 | 203 | 17 | 1 | 3 | - | 4 | 140 | 28 | 10 |
| 50 - 54 | 10,721 | 177 | 177 | 9 | 3 | 1 | - | 3 | 125 | 27 | 9 |
| 55 - 59 | 7,352 | 142 | 142 | 2 | 1 | - | - | 2 | 123 | 10 | 4 |
| 60 - 64 | 6,055 | 129 | 129 | 3 | - | 1 | - | 1 | 104 | 13 | 7 |
| 65 - 69 | 4,961 | 115 | 115 | 5 | - | - | - | 1 | 94 | 13 | 2 |
| 70 - 74 | 4,067 | 137 | 137 | 1 | - | - | - | - | 112 | 15 | 9 |
| 75 - 79 | 3,137 | 113 | 113 | 2 | 1 | - | - | - | 94 | 11 | 5 |
| 80 - 84 | 1,621 | 105 | 105 | 2 | 1 | 1 | - | 1 | 86 | 5 | 9 |
| 85 - 89 | 861 | 83 | 83 | 1 | 2 | - | 1 | 1 | 60 | 9 | 9 |
| 90 - 94 | 245 | 20 | 20 | - | - | - | - | - | 18 | - | 2 |
| 95+ | 197 | - | - | - | - | - | - | - | - | - | - |
| | | | | | | | | | | | |
| **Female** | **536,729** | **5,983** | **5,983** | **130** | **23** | **16** | **10** | **28** | **4,425** | **573** | **778** |
| 0 - 4 | 103,102 | 2,880 | 2,880 | 52 | 5 | - | - | 16 | 2,031 | 249 | 527 |
| 5-9 | 85,244 | 359 | 359 | 14 | 1 | - | - | 3 | 275 | 45 | 21 |
| 10-14 | 71,357 | 241 | 241 | 15 | 2 | 1 | - | 1 | 161 | 44 | 17 |
| 15 - 19 | 57,448 | 261 | 261 | 8 | 6 | 5 | 1 | - | 171 | 35 | 35 |
| 20 - 24 | 47,718 | 263 | 263 | 9 | 1 | 5 | 1 | 1 | 201 | 21 | 24 |
| 25 - 29 | 42,294 | 345 | 345 | 11 | 3 | 2 | 5 | 2 | 254 | 40 | 28 |
| 30 - 34 | 29,201 | 278 | 278 | 5 | 3 | 1 | - | 1 | 218 | 26 | 24 |
| 35 - 39 | 24,898 | 240 | 240 | 2 | 1 | - | 1 | 2 | 183 | 26 | 25 |
| 40 - 44 | 17,327 | 144 | 144 | 3 | - | - | 1 | 1 | 104 | 20 | 15 |
| 45 - 49 | 15,289 | 133 | 133 | 3 | - | - | 1 | - | 105 | 12 | 12 |
| 50 - 54 | 11,422 | 115 | 115 | 2 | - | - | - | - | 93 | 13 | 7 |
| 55 - 59 | 8,006 | 96 | 96 | 1 | 1 | - | - | - | 81 | 7 | 6 |
| 60 - 64 | 7,893 | 109 | 109 | - | - | - | - | - | 97 | 9 | 3 |
| 65 - 69 | 6,145 | 120 | 120 | 2 | - | 1 | - | 1 | 103 | 11 | 2 |
| 70 - 74 | 4,239 | 142 | 142 | 1 | - | - | - | - | 126 | 8 | 7 |
| 75 - 79 | 2,544 | 87 | 87 | 1 | - | - | - | - | 77 | 2 | 7 |
| 80 - 84 | 1,305 | 89 | 89 | 1 | - | - | - | - | 80 | 2 | 6 |
| 85 - 89 | 715 | 68 | 68 | - | - | 1 | - | - | 54 | 2 | 11 |
| 90 - 94 | 213 | 13 | 13 | - | - | - | - | - | 11 | 1 | 1 |
| 95+ | 369 | - | - | - | - | - | - | - | - | - | - |

## Table J4: Population (De Facto) and Number of Household Deaths 12 months prior to the Census by Cause of Death, 5 Year Age Group, Sex and Province, Zambia 2010

| Age Group, Sex and Province | Population | Household Deaths 12 Months Prior to the Census | Cause of Death | | | | | | | | |
|---|---|---|---|---|---|---|---|---|---|---|---|
| | | | Total | Accident | Injury | Suicide | Spousal Violence | Other Violence | Sickness/ Disease | Witchcraft | Other |
| **North-Western Province** | | | | | | | | | | | |
| **Total** | **681,698** | **7,444** | **7,444** | **244** | **30** | **50** | **20** | **81** | **5,187** | **823** | **1,009** |
| 0 - 4 | 132,975 | 2,699 | 2,699 | 50 | 10 | - | - | 16 | 1,761 | 244 | 618 |
| 5-9 | 109,165 | 388 | 388 | 26 | 2 | - | - | 3 | 258 | 67 | 32 |
| 10-14 | 95,597 | 283 | 283 | 17 | 1 | 1 | - | 3 | 180 | 51 | 30 |
| 15 - 19 | 76,074 | 281 | 281 | 26 | 5 | 10 | 3 | 2 | 179 | 33 | 23 |
| 20 - 24 | 55,377 | 336 | 336 | 21 | 1 | 7 | 2 | 5 | 222 | 46 | 32 |
| 25 - 29 | 49,985 | 471 | 471 | 22 | 2 | 12 | 4 | 7 | 319 | 61 | 44 |
| 30 - 34 | 38,742 | 421 | 421 | 11 | 2 | 4 | 1 | 7 | 314 | 47 | 35 |
| 35 - 39 | 31,051 | 400 | 400 | 23 | 1 | 5 | 2 | 6 | 289 | 51 | 23 |
| 40 - 44 | 22,881 | 303 | 303 | 8 | - | 3 | 2 | 3 | 223 | 41 | 23 |
| 45 - 49 | 18,163 | 262 | 262 | 5 | 1 | 3 | - | 4 | 207 | 25 | 17 |
| 50 - 54 | 13,384 | 275 | 275 | 10 | 2 | 2 | 2 | 3 | 198 | 37 | 21 |
| 55 - 59 | 9,389 | 162 | 162 | 2 | - | 2 | 1 | 4 | 117 | 19 | 17 |
| 60 - 64 | 8,423 | 223 | 223 | 8 | - | - | - | 4 | 176 | 27 | 8 |
| 65 - 69 | 6,657 | 192 | 192 | 2 | 1 | - | 2 | 5 | 146 | 20 | 16 |
| 70 - 74 | 5,398 | 251 | 251 | 6 | - | 1 | - | 7 | 198 | 24 | 15 |
| 75 - 79 | 3,968 | 183 | 183 | 5 | 1 | - | 1 | - | 148 | 14 | 14 |
| 80 - 84 | 2,184 | 155 | 155 | - | - | - | - | 2 | 128 | 8 | 17 |
| 85 - 89 | 1,148 | 121 | 121 | 2 | - | - | - | - | 96 | 8 | 15 |
| 90 - 94 | 483 | 38 | 38 | - | 1 | - | - | - | 28 | - | 9 |
| 95+ | 654 | - | - | - | - | - | - | - | - | - | - |
| | | | | | | | | | | | |
| **Male** | **332,376** | **3,843** | **3,843** | **164** | **19** | **38** | **7** | **61** | **2,579** | **464** | **511** |
| 0 - 4 | 66,280 | 1,468 | 1,468 | 30 | 8 | - | - | 11 | 964 | 139 | 316 |
| 5-9 | 54,525 | 219 | 219 | 14 | 1 | - | - | 2 | 141 | 43 | 18 |
| 10-14 | 47,857 | 143 | 143 | 7 | 1 | 1 | - | 1 | 95 | 21 | 17 |
| 15 - 19 | 37,366 | 139 | 139 | 20 | 2 | 8 | 1 | 1 | 81 | 19 | 7 |
| 20 - 24 | 25,191 | 152 | 152 | 16 | 1 | 5 | - | 4 | 91 | 20 | 15 |
| 25 - 29 | 22,669 | 209 | 209 | 18 | 2 | 10 | - | 6 | 125 | 30 | 18 |
| 30 - 34 | 18,855 | 202 | 202 | 9 | 2 | 4 | 1 | 5 | 141 | 28 | 12 |
| 35 - 39 | 15,178 | 204 | 204 | 16 | - | 2 | 2 | 5 | 140 | 26 | 13 |
| 40 - 44 | 11,353 | 171 | 171 | 8 | - | 3 | 2 | 2 | 119 | 25 | 12 |
| 45 - 49 | 8,797 | 138 | 138 | 4 | - | 2 | - | 4 | 102 | 16 | 10 |
| 50 - 54 | 6,284 | 138 | 138 | 8 | 1 | - | 1 | 3 | 83 | 26 | 16 |
| 55 - 59 | 4,520 | 80 | 80 | 1 | - | 2 | - | 2 | 57 | 10 | 8 |
| 60 - 64 | 3,698 | 105 | 105 | 4 | - | - | - | 3 | 79 | 15 | 4 |
| 65 - 69 | 2,898 | 97 | 97 | 2 | - | - | - | 3 | 72 | 11 | 9 |
| 70 - 74 | 2,549 | 122 | 122 | 2 | - | 1 | - | 7 | 92 | 14 | 6 |
| 75 - 79 | 2,038 | 98 | 98 | 4 | 1 | - | - | - | 76 | 10 | 7 |
| 80 - 84 | 1,102 | 70 | 70 | - | - | - | - | 2 | 54 | 4 | 10 |
| 85 - 89 | 651 | 67 | 67 | 1 | - | - | - | - | 51 | 7 | 8 |
| 90 - 94 | 278 | 21 | 21 | - | - | - | - | - | 16 | - | 5 |
| 95+ | 287 | - | - | - | - | - | - | - | - | - | - |

## Table J4: Population (De Facto) and Number of Household Deaths 12 months prior to the Census by Cause of Death, 5 Year Age Group, Sex and Province, Zambia 2010

| Age Group, Sex and Province | Population | Household Deaths 12 Months Prior to the Census | Cause of Death | | | | | | | | |
|---|---|---|---|---|---|---|---|---|---|---|---|
| | | | Total | Accident | Injury | Suicide | Spousal Violence | Other Violence | Sickness/ Disease | Witchcraft | Other |
| **Female** | **349,322** | **3,601** | **3,601** | **80** | **11** | **12** | **13** | **20** | **2,608** | **359** | **498** |
| 0 - 4 | 66,695 | 1,231 | 1,231 | 20 | 2 | - | - | 5 | 797 | 105 | 302 |
| 5-9 | 54,640 | 169 | 169 | 12 | 1 | - | - | 1 | 117 | 24 | 14 |
| 10-14 | 47,740 | 140 | 140 | 10 | - | - | - | 2 | 85 | 30 | 13 |
| 15 - 19 | 38,708 | 142 | 142 | 6 | 3 | 2 | 2 | 1 | 98 | 14 | 16 |
| 20 - 24 | 30,186 | 184 | 184 | 5 | - | 2 | 2 | 1 | 131 | 26 | 17 |
| 25 - 29 | 27,316 | 262 | 262 | 4 | - | 2 | 4 | 1 | 194 | 31 | 26 |
| 30 - 34 | 19,887 | 219 | 219 | 2 | - | - | - | 2 | 173 | 19 | 23 |
| 35 - 39 | 15,873 | 196 | 196 | 7 | 1 | 3 | - | 1 | 149 | 25 | 10 |
| 40 - 44 | 11,528 | 132 | 132 | - | - | - | - | 1 | 104 | 16 | 11 |
| 45 - 49 | 9,366 | 124 | 124 | 1 | 1 | 1 | - | - | 105 | 9 | 7 |
| 50 - 54 | 7,100 | 137 | 137 | 2 | 1 | 2 | 1 | - | 115 | 11 | 5 |
| 55 - 59 | 4,869 | 82 | 82 | 1 | - | - | 1 | 2 | 60 | 9 | 9 |
| 60 - 64 | 4,725 | 118 | 118 | 4 | - | - | - | 1 | 97 | 12 | 4 |
| 65 - 69 | 3,759 | 95 | 95 | - | 1 | - | 2 | 2 | 74 | 9 | 7 |
| 70 - 74 | 2,849 | 129 | 129 | 4 | - | - | - | - | 106 | 10 | 9 |
| 75 - 79 | 1,930 | 85 | 85 | 1 | - | - | 1 | - | 72 | 4 | 7 |
| 80 - 84 | 1,082 | 85 | 85 | - | - | - | - | - | 74 | 4 | 7 |
| 85 - 89 | 497 | 54 | 54 | 1 | - | - | - | - | 45 | 1 | 7 |
| 90 - 94 | 205 | 17 | 17 | - | 1 | - | - | - | 12 | - | 4 |
| 95+ | 367 | - | - | - | - | - | - | - | - | - | - |
| **Southern Province** | | | | | | | | | | | |
| **Total** | **1,517,088** | **15,222** | **15,222** | **601** | **80** | **115** | **28** | **159** | **10,941** | **1,205** | **2,093** |
| 0 - 4 | 285,543 | 6,037 | 6,037 | 124 | 21 | - | - | 40 | 3,993 | 506 | 1,353 |
| 5-9 | 236,602 | 818 | 818 | 49 | 2 | - | - | 4 | 590 | 85 | 88 |
| 10-14 | 209,728 | 411 | 411 | 41 | 3 | 2 | 2 | 6 | 269 | 38 | 50 |
| 15 - 19 | 175,472 | 536 | 536 | 58 | 5 | 13 | 1 | 10 | 339 | 49 | 61 |
| 20 - 24 | 134,896 | 687 | 687 | 64 | 2 | 20 | 4 | 9 | 471 | 52 | 65 |
| 25 - 29 | 115,241 | 921 | 921 | 70 | 3 | 21 | 4 | 16 | 661 | 73 | 73 |
| 30 - 34 | 93,577 | 1,005 | 1,005 | 45 | 5 | 10 | 6 | 14 | 782 | 63 | 80 |
| 35 - 39 | 73,027 | 918 | 918 | 45 | 11 | 11 | 2 | 9 | 711 | 62 | 67 |
| 40 - 44 | 50,447 | 649 | 649 | 25 | 6 | 10 | 2 | 13 | 504 | 54 | 35 |
| 45 - 49 | 39,587 | 539 | 539 | 14 | 2 | 7 | 2 | 6 | 419 | 53 | 36 |
| 50 - 54 | 29,666 | 474 | 474 | 23 | 2 | 5 | 2 | 7 | 372 | 30 | 33 |
| 55 - 59 | 18,724 | 323 | 323 | 14 | 1 | 3 | - | 4 | 253 | 26 | 22 |
| 60 - 64 | 16,467 | 359 | 359 | 6 | 3 | 2 | 1 | 7 | 295 | 26 | 19 |
| 65 - 69 | 13,009 | 293 | 293 | 5 | 3 | 3 | - | 5 | 242 | 26 | 9 |
| 70 - 74 | 10,251 | 381 | 381 | 9 | 4 | 3 | - | 4 | 308 | 22 | 31 |
| 75 - 79 | 7,064 | 339 | 339 | 4 | 4 | 3 | 1 | 1 | 292 | 16 | 18 |
| 80 - 84 | 3,797 | 282 | 282 | 4 | 2 | 2 | 1 | 1 | 237 | 14 | 21 |
| 85 - 89 | 2,198 | 192 | 192 | - | 1 | - | - | 3 | 156 | 10 | 22 |
| 90 - 94 | 801 | 58 | 58 | 1 | - | - | - | - | 47 | - | 10 |
| 95+ | 991 | - | - | - | - | - | - | - | - | - | - |

## Table J4: Population (De Facto) and Number of Household Deaths 12 months prior to the Census by Cause of Death, 5 Year Age Group, Sex and Province, Zambia 2010

| Age Group, Sex and Province | Population | Household Deaths 12 Months Prior to the Census | Cause of Death | | | | | | | | |
|---|---|---|---|---|---|---|---|---|---|---|---|
| | | | Total | Accident | Injury | Suicide | Spousal Violence | Other Violence | Sickness/ Disease | Witchcraft | Other |
| **Male** | **738,996** | **8,150** | **8,150** | **435** | **51** | **90** | **14** | **116** | **5,697** | **655** | **1,092** |
| 0 - 4 | 142,222 | 3,154 | 3,154 | 71 | 10 | - | - | 25 | 2,089 | 259 | 700 |
| 5-9 | 118,028 | 423 | 423 | 32 | 1 | - | - | 3 | 300 | 38 | 49 |
| 10-14 | 104,407 | 208 | 208 | 25 | 3 | 1 | 1 | 4 | 129 | 17 | 28 |
| 15 - 19 | 86,767 | 282 | 282 | 38 | 2 | 6 | - | 7 | 168 | 32 | 29 |
| 20 - 24 | 62,288 | 305 | 305 | 47 | 2 | 15 | 1 | 8 | 183 | 24 | 25 |
| 25 - 29 | 53,055 | 438 | 438 | 56 | 3 | 15 | - | 14 | 287 | 30 | 33 |
| 30 - 34 | 45,586 | 555 | 555 | 41 | 4 | 9 | 4 | 11 | 417 | 37 | 32 |
| 35 - 39 | 36,713 | 530 | 530 | 40 | 6 | 10 | 2 | 6 | 391 | 34 | 41 |
| 40 - 44 | 24,962 | 377 | 377 | 23 | 5 | 7 | 1 | 13 | 278 | 31 | 19 |
| 45 - 49 | 18,975 | 322 | 322 | 12 | 1 | 7 | 2 | 4 | 236 | 37 | 23 |
| 50 - 54 | 13,756 | 294 | 294 | 14 | 1 | 5 | 1 | 4 | 230 | 18 | 21 |
| 55 - 59 | 8,846 | 209 | 209 | 13 | - | 3 | - | 4 | 155 | 15 | 19 |
| 60 - 64 | 7,051 | 204 | 204 | 5 | 3 | 2 | 1 | 6 | 153 | 19 | 15 |
| 65 - 69 | 5,412 | 165 | 165 | 4 | 2 | 3 | - | 4 | 132 | 15 | 5 |
| 70 - 74 | 4,224 | 210 | 210 | 7 | 3 | 3 | - | 1 | 159 | 19 | 18 |
| 75 - 79 | 3,238 | 185 | 185 | 4 | 3 | 2 | - | 1 | 156 | 11 | 8 |
| 80 - 84 | 1,705 | 149 | 149 | 3 | 1 | 2 | 1 | 1 | 120 | 10 | 11 |
| 85 - 89 | 1,051 | 111 | 111 | - | 1 | - | - | - | 90 | 9 | 11 |
| 90 - 94 | 366 | 29 | 29 | - | - | - | - | - | 24 | - | 5 |
| 95+ | 344 | - | - | - | - | - | - | - | - | - | - |
| | | | | | | | | | | | |
| **Female** | **778,092** | **7,072** | **7,072** | **166** | **29** | **25** | **14** | **43** | **5,244** | **550** | **1,001** |
| 0 - 4 | 143,321 | 2,883 | 2,883 | 53 | 11 | - | - | 15 | 1,904 | 247 | 653 |
| 5-9 | 118,574 | 395 | 395 | 17 | 1 | - | - | 1 | 290 | 47 | 39 |
| 10-14 | 105,321 | 203 | 203 | 16 | - | 1 | 1 | 2 | 140 | 21 | 22 |
| 15 - 19 | 88,705 | 254 | 254 | 20 | 3 | 7 | 1 | 3 | 171 | 17 | 32 |
| 20 - 24 | 72,608 | 382 | 382 | 17 | - | 5 | 3 | 1 | 288 | 28 | 40 |
| 25 - 29 | 62,186 | 483 | 483 | 14 | - | 6 | 4 | 2 | 374 | 43 | 40 |
| 30 - 34 | 47,991 | 450 | 450 | 4 | 1 | 1 | 2 | 3 | 365 | 26 | 48 |
| 35 - 39 | 36,314 | 388 | 388 | 5 | 5 | 1 | - | 3 | 320 | 28 | 26 |
| 40 - 44 | 25,485 | 272 | 272 | 2 | 1 | 3 | 1 | - | 226 | 23 | 16 |
| 45 - 49 | 20,612 | 217 | 217 | 2 | 1 | - | - | 2 | 183 | 16 | 13 |
| 50 - 54 | 15,910 | 180 | 180 | 9 | 1 | - | 1 | 3 | 142 | 12 | 12 |
| 55 - 59 | 9,878 | 114 | 114 | 1 | 1 | - | - | - | 98 | 11 | 3 |
| 60 - 64 | 9,416 | 155 | 155 | 1 | - | - | - | 1 | 142 | 7 | 4 |
| 65 - 69 | 7,597 | 128 | 128 | 1 | 1 | - | - | 1 | 110 | 11 | 4 |
| 70 - 74 | 6,027 | 171 | 171 | 2 | 1 | - | - | 3 | 149 | 3 | 13 |
| 75 - 79 | 3,826 | 154 | 154 | - | 1 | 1 | 1 | - | 136 | 5 | 10 |
| 80 - 84 | 2,092 | 133 | 133 | 1 | 1 | - | - | - | 117 | 4 | 10 |
| 85 - 89 | 1,147 | 81 | 81 | - | - | - | - | 3 | 66 | 1 | 11 |
| 90 - 94 | 435 | 29 | 29 | 1 | - | - | - | - | 23 | - | 5 |
| 95+ | 647 | - | - | - | - | - | - | - | - | - | - |

## Table J4: Population (De Facto) and Number of Household Deaths 12 months prior to the Census by Cause of Death, 5 Year Age Group, Sex and Province, Zambia 2010

| Age Group, Sex and Province | Population | Household Deaths 12 Months Prior to the Census | Cause of Death | | | | | | | | |
|---|---|---|---|---|---|---|---|---|---|---|---|
| | | | Total | Accident | Injury | Suicide | Spousal Violence | Other Violence | Sickness/ Disease | Witchcraft | Other |
| **Western Province** | | | | | | | | | | | |
| **Total** | **836,945** | **13,395** | **13,395** | **401** | **55** | **78** | **23** | **128** | **10,339** | **1,014** | **1,357** |
| 0 - 4 | 157,980 | 4,264 | 4,264 | 91 | 16 | - | - | 28 | 3,055 | 237 | 837 |
| 5-9 | 127,396 | 593 | 593 | 32 | - | - | - | 5 | 457 | 54 | 45 |
| 10-14 | 109,852 | 380 | 380 | 14 | 3 | 1 | - | 5 | 295 | 33 | 29 |
| 15 - 19 | 88,909 | 529 | 529 | 30 | 2 | 16 | - | 7 | 368 | 72 | 34 |
| 20 - 24 | 70,087 | 776 | 776 | 49 | 4 | 10 | 8 | 9 | 576 | 78 | 42 |
| 25 - 29 | 61,530 | 1,002 | 1,002 | 50 | 6 | 9 | 2 | 17 | 769 | 76 | 73 |
| 30 - 34 | 48,851 | 1,061 | 1,061 | 36 | 7 | 12 | 6 | 15 | 839 | 89 | 57 |
| 35 - 39 | 38,743 | 863 | 863 | 28 | 3 | 9 | 5 | 15 | 685 | 75 | 43 |
| 40 - 44 | 28,472 | 672 | 672 | 20 | 4 | 7 | 1 | 9 | 545 | 58 | 28 |
| 45 - 49 | 23,838 | 543 | 543 | 13 | - | 7 | 1 | 3 | 446 | 53 | 20 |
| 50 - 54 | 20,018 | 437 | 437 | 9 | 2 | - | - | 3 | 364 | 32 | 27 |
| 55 - 59 | 13,828 | 313 | 313 | 4 | 1 | 1 | - | 3 | 259 | 29 | 16 |
| 60 - 64 | 13,546 | 373 | 373 | 2 | 3 | 1 | - | 2 | 296 | 48 | 21 |
| 65 - 69 | 10,325 | 287 | 287 | 1 | - | 1 | - | 1 | 251 | 22 | 11 |
| 70 - 74 | 8,820 | 424 | 424 | 9 | 1 | 2 | - | 4 | 352 | 26 | 30 |
| 75 - 79 | 6,233 | 307 | 307 | 6 | 2 | - | - | - | 263 | 19 | 17 |
| 80 - 84 | 3,833 | 277 | 277 | 4 | 1 | - | - | 1 | 252 | 6 | 13 |
| 85 - 89 | 2,360 | 244 | 244 | 2 | - | 1 | - | 1 | 220 | 6 | 14 |
| 90 - 94 | 972 | 50 | 50 | 1 | - | 1 | - | - | 47 | 1 | - |
| 95+ | 1,352 | - | - | - | - | - | - | - | - | - | - |
| | | | | | | | | | | | |
| **Male** | **395,913** | **6,977** | **6,977** | **300** | **33** | **55** | **8** | **91** | **5,179** | **597** | **714** |
| 0 - 4 | 78,959 | 2,280 | 2,280 | 66 | 8 | - | - | 14 | 1,587 | 142 | 463 |
| 5-9 | 63,980 | 332 | 332 | 25 | - | - | - | 4 | 249 | 31 | 23 |
| 10-14 | 54,826 | 224 | 224 | 9 | 2 | - | - | 2 | 172 | 23 | 16 |
| 15 - 19 | 42,730 | 261 | 261 | 19 | 2 | 12 | - | 6 | 169 | 42 | 11 |
| 20 - 24 | 30,101 | 355 | 355 | 40 | 4 | 8 | 2 | 6 | 240 | 41 | 14 |
| 25 - 29 | 26,626 | 457 | 457 | 43 | 4 | 8 | 1 | 10 | 319 | 45 | 27 |
| 30 - 34 | 22,372 | 526 | 526 | 28 | 3 | 10 | 2 | 12 | 396 | 52 | 23 |
| 35 - 39 | 18,025 | 495 | 495 | 24 | 1 | 6 | 1 | 14 | 368 | 49 | 32 |
| 40 - 44 | 13,207 | 355 | 355 | 13 | 1 | 5 | 1 | 9 | 279 | 31 | 16 |
| 45 - 49 | 10,419 | 331 | 331 | 11 | - | 3 | 1 | 2 | 273 | 31 | 10 |
| 50 - 54 | 8,345 | 223 | 223 | 5 | 2 | - | - | 3 | 178 | 19 | 16 |
| 55 - 59 | 5,817 | 166 | 166 | 4 | - | 1 | - | 2 | 139 | 16 | 4 |
| 60 - 64 | 5,257 | 184 | 184 | 1 | 2 | - | - | 1 | 141 | 29 | 10 |
| 65 - 69 | 4,268 | 136 | 136 | 1 | - | - | - | 1 | 112 | 16 | 6 |
| 70 - 74 | 3,910 | 230 | 230 | 5 | 1 | 1 | - | 3 | 188 | 9 | 23 |
| 75 - 79 | 3,019 | 144 | 144 | 2 | 2 | - | - | - | 120 | 11 | 9 |
| 80 - 84 | 1,847 | 130 | 130 | 2 | 1 | - | - | 1 | 115 | 5 | 6 |
| 85 - 89 | 1,186 | 123 | 123 | 1 | - | - | - | 1 | 112 | 4 | 5 |
| 90 - 94 | 496 | 25 | 25 | 1 | - | 1 | - | - | 22 | 1 | - |
| 95+ | 523 | - | - | - | - | - | - | - | - | - | - |

## Table J4: Population (De Facto) and Number of Household Deaths 12 months prior to the Census by Cause of Death, 5 Year Age Group, Sex and Province, Zambia 2010

| Age Group, Sex and Province | Population | Household Deaths 12 Months Prior to the Census | Cause of Death | | | | | | | |
|---|---|---|---|---|---|---|---|---|---|---|
| | | | Total | Accident | Injury | Suicide | Spousal Violence | Other Violence | Sickness/ Disease | Witchcraft | Other |
| **Female** | **441,032** | **6,418** | **6,418** | **101** | **22** | **23** | **15** | **37** | **5,160** | **417** | **643** |
| 0 - 4 | 79,021 | 1,984 | 1,984 | 25 | 8 | - | - | 14 | 1,468 | 95 | 374 |
| 5-9 | 63,416 | 261 | 261 | 7 | - | - | - | 1 | 208 | 23 | 22 |
| 10-14 | 55,026 | 156 | 156 | 5 | 1 | 1 | - | 3 | 123 | 10 | 13 |
| 15 - 19 | 46,179 | 268 | 268 | 11 | - | 4 | - | 1 | 199 | 30 | 23 |
| 20 - 24 | 39,986 | 421 | 421 | 9 | - | 2 | 6 | 3 | 336 | 37 | 28 |
| 25 - 29 | 34,904 | 545 | 545 | 7 | 2 | 1 | 1 | 7 | 450 | 31 | 46 |
| 30 - 34 | 26,479 | 535 | 535 | 8 | 4 | 2 | 4 | 3 | 443 | 37 | 34 |
| 35 - 39 | 20,718 | 368 | 368 | 4 | 2 | 3 | 4 | 1 | 317 | 26 | 11 |
| 40 - 44 | 15,265 | 317 | 317 | 7 | 3 | 2 | - | - | 266 | 27 | 12 |
| 45 - 49 | 13,419 | 212 | 212 | 2 | - | 4 | - | 1 | 173 | 22 | 10 |
| 50 - 54 | 11,673 | 214 | 214 | 4 | - | - | - | - | 186 | 13 | 11 |
| 55 - 59 | 8,011 | 147 | 147 | - | 1 | - | - | 1 | 120 | 13 | 12 |
| 60 - 64 | 8,289 | 189 | 189 | 1 | 1 | 1 | - | 1 | 155 | 19 | 11 |
| 65 - 69 | 6,057 | 151 | 151 | - | - | 1 | - | - | 139 | 6 | 5 |
| 70 - 74 | 4,910 | 194 | 194 | 4 | - | 1 | - | 1 | 164 | 17 | 7 |
| 75 - 79 | 3,214 | 163 | 163 | 4 | - | - | - | - | 143 | 8 | 8 |
| 80 - 84 | 1,986 | 147 | 147 | 2 | - | - | - | - | 137 | 1 | 7 |
| 85 - 89 | 1,174 | 121 | 121 | 1 | - | 1 | - | - | 108 | 2 | 9 |
| 90 - 94 | 476 | 25 | 25 | - | - | - | - | - | 25 | - | - |
| 95+ | 829 | - | - | - | - | - | - | - | - | - | - |

# 2010 Census of Population and Housing Questionnaire

# REPUBLIC OF ZAMBIA

CENTRAL STATISTICAL OFFICE

## 2010 CENSUS OF POPULATION AND HOUSING

IDENTIFICATION

Province

District

Constituency

Ward

Region Rural Urban

CSA No.

SEA No.

Census Building No. (CBN)

Housing Unit No. (HUN)

Household No. (HHN)

Village/ Locality Name

Residential Address

Chief's Area

INTERVIEW STATUS

Completed (occupied)

Non-contact (occupied)

Not interviewed (vacant)

Non-residential

Refused

Other

MARK HERE IF MORE THAN ONE QUESTIONNAIRE

Questionnaire

of

SECTION P - QUESTIONS ON POPULATION FOR ALL MEMBERS OF THE HOUSEHOLD

ENUMERATOR

Name Date

SUPERVISOR

Name Date

CONFIDENTIAL: The Census is being conducted under the Census & Statistics Act, CAP 127 of the laws of Zambia. The information will be strictly confidential and used for statistical purposes only.

## HOUSEHOLD SUMMARY COUNT

| | Usual members present | Usual members absent | Visitors |
|---|---|---|---|
| Male | | | |
| Female | | | |
| Total | | | |

## INSTITUTIONAL

Type of Collective Quarters

Hotel/Motel/Lodge
Hostel/Guest House/Inn
Hospital
Learning Institution
Prison
Police Cell
Refugee Camp
Other (specify below)

Summary Count

| | |
|---|---|
| Male | |
| Female | |
| Total | |

99000046 (82)

## FOR ALL MEMBERS PRESENT

| Person Number | P12 What is [NAME]'s ethnicity? | P13 What is [NAME]'s predominant language of communication? | P14 Where was [NAME] residing in October 2009? | P15 How long has [NAME] been living continuously in [NAME OF CURRENT PLACE OF RESIDENCE]? | P16 Is [NAME] disabled in any way? | P17 What is [NAME]'s disability? Mark all those that apply | P18 What is the cause of [NAME]'s disability? Mark all those that apply | P19 Is [NAME] [illegible] alive? | P20 Is [NAME]'s biological mother alive? | P21 Does [NAME]'s biological mother live in this household? | P22 Is [NAME]'s biological father alive? | P23 Does [NAME]'s biological father live in this household? | P24 Does [NAME] have a birth certificate? | P25 Can [NAME] read and write in any language? | P26 Has [NAME] ever attended school? | P27 Is [NAME] currently attending school? | P28 What highest level of education has [NAME] completed? |
|---|---|---|---|---|---|---|---|---|---|---|---|---|---|---|---|---|---|
| | Write and insert code for ethnicity | Write name of predominant language then code | Record code for district or code for foreign country | YEARS / MONTHS | | | | | ONLY FOR PERSONS LESS THAN 18 YEARS OLD | | | | | | | | Enter code from manual |
| 1 | | | | Same as Head of Household | | | | | | | | | | | | | |
| 2 | | | | Same as Head of Household | | | | | | | | | | | | | |
| 3 | | | | Same as Head of Household | | | | | | | | | | | | | |
| 4 | | | | Same as Head of Household | | | | | | | | | | | | | |
| 5 | | | | Same as Head of Household | | | | | | | | | | | | | |
| 6 | | | | Same as Head of Household | | | | | | | | | | | | | |
| 7 | | | | Same as Head of Household | | | | | | | | | | | | | |
| 8 | | | | Same as Head of Household | | | | | | | | | | | | | |

## ONLY FOR PERSONS AGED 5 YEARS AND OLDER

- Person Number
- [illegible] What is the highest professional or vocational qualification (NAME) has completed?
- [illegible] What is the field of study for the highest professional or vocational qualification completed? Write field of study and enter code.

## ONLY FOR PERSONS AGED 12 YEARS OR OLDER

- [illegible] What did (NAME) do in the last 7 days?
- [illegible] What did (NAME) do in the last 12 months?
- [illegible] What was (NAME)'s employment status in the last 12 months?
- [illegible] What kind of work did (NAME) do in his/her main job or business during the last 12 months? Write main occupation and enter code.

## ONLY FOR PERSONS AGED 12 YEARS AND OLDER

- Person Number
- [illegible] What kind of business/service was mainly carried out by (NAME)'s employer/establishment/business in the last 12 months? Write name of industry and enter code.
- [illegible] What is (NAME)'s marital status?
- [illegible]

## FEMALES 12 YEARS AND OLDER

- [illegible] Have you ever had a live birth, including babies who died after birth?
- Of the children born to you alive how many are...?
  - Living with you now?
  - Living elsewhere?
  - Dead?

## FEMALES 12 - 49 YEARS OLD

- [illegible] Did you have any live births in the last 12 months?
- Of the children born to you alive in the last 12 months how many are...?
  - Living with you now?
  - Living elsewhere?
  - Dead?

## PERSONS 18 YEARS+

- [illegible]
- [illegible] Are you a registered voter?

ENUMERATOR: GO TO THE NEXT PAGE TO CONTINUE WITH THE LIST OF THE HOUSEHOLD'S MEMBERS. IF THE PERSON IS THE LAST MEMBER OF THE HOUSEHOLD, PROCEED TO SECTION D

## SECTION H - HOUSING CHARACTERISTICS

[illegible]

## SECTION A - AGRICULTURE

[illegible]

## SECTION M: GENERAL AND MATERNAL DEATHS

Please record information on the deaths that occurred in the household during the last 12 months.
Do not forget the children.

[illegible]

# Persons involved in the Production of the Descriptive Tabulation Reports

| No. | Name | Surname | Responsibility | Tables for Review/Production/Formatting |
|---|---|---|---|---|
| 1 | Richard | Banda | Analyst | Series A: General Population Tables;<br>Series J: General & Maternal Mortality Tables and;<br>Series H: Housing and Household Tables |
| 2 | Nchimunya | Nkombo | Analyst | Series C: Fertility Tables |
| 3 | Iven | Sikanyiti | Analyst | Series G: Migration |
| 4 | Chibesa | Musamba | Analyst | Series F: Disability Tables |
| 5 | Linda | Chonya | Analyst | Series B: Social Tables |
| 6 | Gerson | Banda | Analyst | Series E: Economic Tables |
| 7 | Joseph | Mutemwa | Analyst | Series D: Education Tables |
| 8 | Catherine | Mwape | Analyst | Series B: Social Tables |
| 9 | Masiliso | Sooka | Analyst | Series I: Agriculture Tables |
| 10 | Chola N. | Daka | Analyst | Editing all Tables |
| 11 | Etambuyu | Lukonga | Analyst | Editing all Tables |
| 12 | Frank | Kakungu | Analyst/Programmer | Series H: Housing and Household Tables |
| 13 | Anthony | Nkole | Desktop Publishing Officer | Layout and Formatting All Tables |
| 14 | Perry | Musenge | Assistant Desktop Publishing Officer | Formatting All Tables |
| 15 | Victor | Bwalya | Programmer | Series F: Disability Tables, Series J: General & Maternal Mortality Tables |
| 16 | Tabo | Simutanyi | Programmer | Series B: Social Tables |
| 17 | Chibesa | Ndawa | Programmer | Series I: Agriculture Tables, Series H: Housing and Household Tables |
| 18 | Makoselo | Bowa | Programmer/Desktop Publishing | Series E: Economic Tables |
| 19 | Betha | Nachinga | Programmer | Series D: Education Tables |
| 20 | Hilda | Chileshe | Programmer | Series A: General Population Tables |
| 21 | Costain | Munsaka | Programmer | Series G: Migration |
| 22 | Chonde | Namutowe | Programmer | Series C: Fertility Tables |
| 23 | Morris | Munkondya | Driver | |
| 24 | Britias | Chipoba | Driver | |
