# Supplementary material for: Impact of Pregnancy-Related Deaths on Female Life Expectancy in Zambia: Application of Life Table Techniques to Census Data
Source: PLoS One. 2015 Oct 29;10(10):e0141689. doi: 10.1371/journal.pone.0141689 (PMC4626102; doi:10.1371/journal.pone.0141689)
Supplement: S4 Text — (PDF) [file pone.0141689.s007.pdf]

Table 1: Results of the estimation of ${}_na_x$ values using the graduation method with two iterations, Zambia Total, 2010 Census

${}_na_x=(-n/24*{}_nd_{x-n} + n/2*{}_nd_{x+n} + n/24*{}_nd_{x+n})/{}_nd_x$

Zambia Total

Age	Direct using recorded deaths	Iteration 1	Iteration 2
5	2.39	1.89	1.95
10	1.90	2.42	2.49
15	2.63	2.63	2.69
20	2.64	2.61	2.67
25	2.52	2.54	2.61
30	2.48	2.46	2.53
35	2.40	2.39	2.47
40	2.36	2.37	2.47
45	2.40	2.42	2.50
50	2.42	2.41	2.47
55	2.48	2.46	2.52
60	2.50	2.52	2.56
65	2.50	2.52	2.59
70	2.50	2.49	2.55
75	2.61	2.60	2.92

Table 1: Results of the estimation of ${}_{n}a_{x}$ values using the graduation method with two iterations, Zambia Rural, 2010 Census

Zambia Rural

Age	Direct using recorded deaths	Iteration 1	Iteration 2
5	2.38	1.89	1.99
10	1.84	2.35	2.46
15	2.52	2.59	2.66
20	2.64	2.59	2.66
25	2.51	2.53	2.60
30	2.48	2.46	2.52
35	2.41	2.40	2.47
40	2.37	2.38	2.46
45	2.40	2.42	2.50
50	2.43	2.41	2.47
55	2.50	2.48	2.52
60	2.52	2.53	2.56
65	2.52	2.54	2.61
70	2.52	2.50	2.57
75	2.60	2.58	3.03

Table 1: Results of the estimation of ${}_na_x$ values using the graduation method with two iterations, Zambia Urban, 2010 Census

Zambia Urban

Age	Direct using recorded deaths	Iteration 1	Iteration 2
5	2.40	1.89	1.87
10	2.02	2.55	2.58
15	2.74	2.70	2.73
20	2.64	2.64	2.69
25	2.53	2.55	2.62
30	2.48	2.46	2.54
35	2.40	2.38	2.48
40	2.34	2.36	2.49
45	2.40	2.43	2.50
50	2.42	2.40	2.46
55	2.46	2.43	2.55
60	2.48	2.50	2.57
65	2.47	2.49	2.55
70	2.48	2.46	2.50
75	2.63	2.62	2.71
